# The Catalytic Asymmetric Alkylation of Olefins with Alcohol Derivatives

**DOI:** 10.1021/jacs.6c12914

**Published:** 2026-08-17

**Authors:** Luc M. Debie, Thibault Gotta, Mathias Turberg, Nils Nöthling, Benjamin List

**Affiliations:** Max-Planck-Institut für Kohlenforschung, Mülheim an der Ruhr D-45470, Germany

## Abstract

Both olefins and arenes are abundant and versatile feedstock chemicals. Although the electrophilic alkylation of arenes constitutes an advanced technology, the corresponding alkylation of olefins is rather undeveloped. Here, we report an asymmetric catalytic benzylation and allylation of unactivated olefins with acetates as electrophiles. The reaction is catalyzed by a single imidodiphosphorimidate organocatalyst operating as a silylium Lewis acid. Mechanistic experiments support an enantioconvergent S_N_1 mechanism featuring stereoablative ion pair formation, resulting in a dynamic kinetic resolution. The enantioenriched hydrocarbon products are readily derivatized to odorants and nonsteroidal anti-inflammatory drugs in one or two steps. The developed method provides a metal-free alternative to cornerstone *Tsuji*–*Trost*-type allylations proceeding though π-allyl metal complexes.

## Graphical abstract

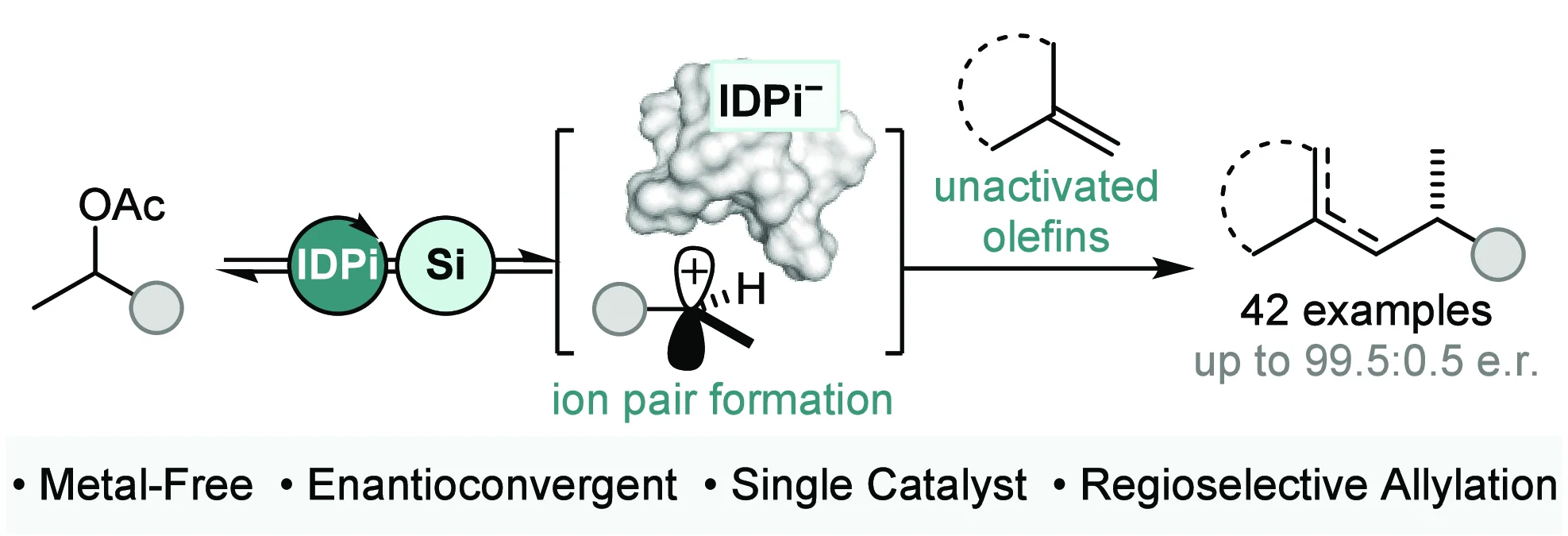

Olefin alkylation reactions are of high relevance in terpene biosynthesis. The archetypical example is the evolutionarily conserved isoprenyl pyrophosphate synthase (IPPS)-catalyzed synthesis of geranyl pyrophosphate (Figure 1A).^1^ This reaction provides a building block for chain elongation toward polyprenyl pyrophosphates as well as cyclase-catalyzed reactions toward terpene natural products.^2^

**Figure 1:**
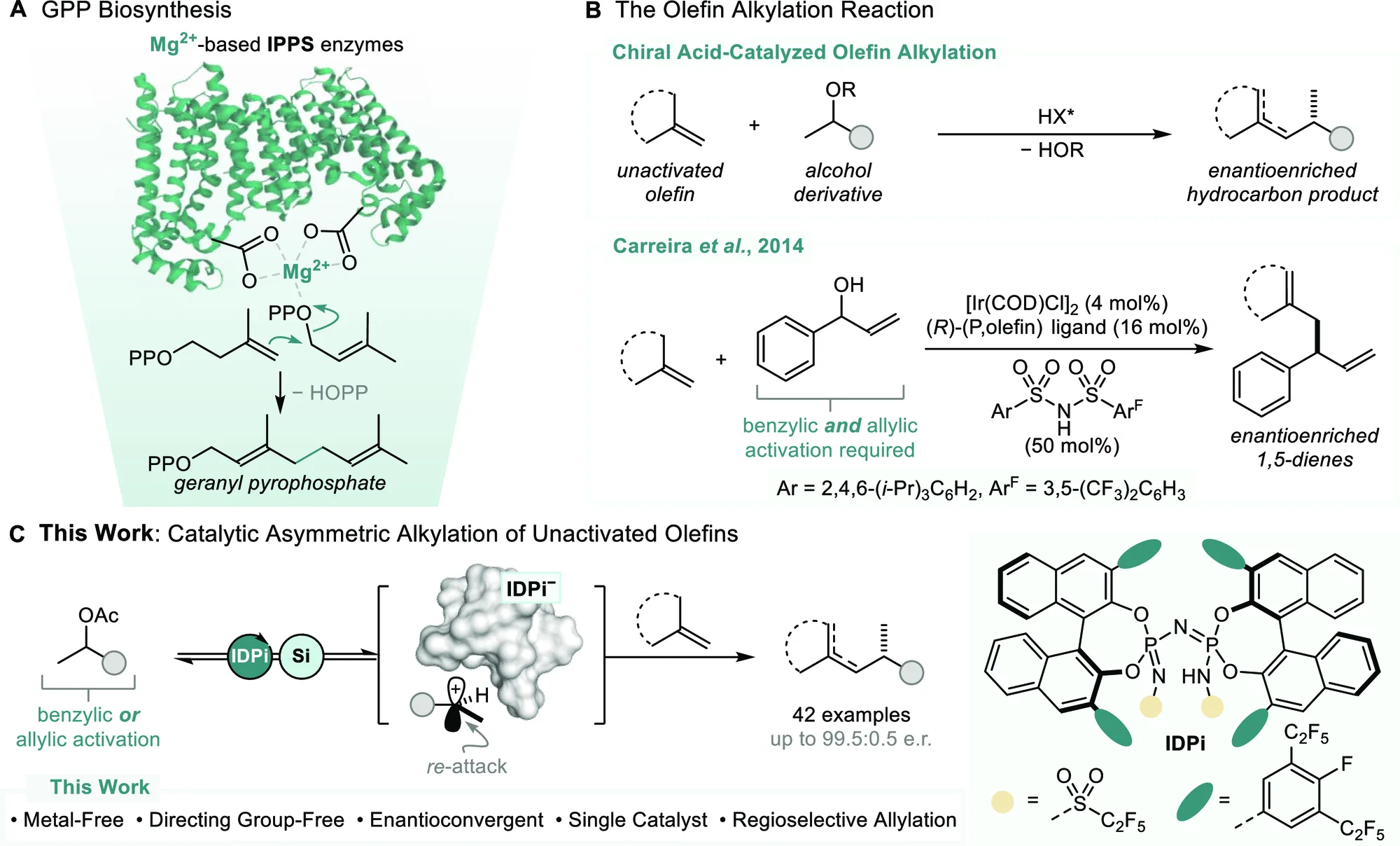
Biocatalysis and small-molecule catalysis in olefin alkylation with alcohol derivatives. (A) Geranyl pyrophosphate biosynthesis. IPPS structure adapted from ref 15. Available under a CC-BY 4.0 license. Copyright 2011 Hsieh et al. (PDB ID: 3APZ, 2.60 Å resolution).^15,16^ (B) Asymmetric olefin alkylation with small-molecule acid catalysts. (C) This work: realization of an asymmetric organocatalytic intermolecular olefin alkylation of unactivated olefins with acetates.

Small-molecule catalysis in olefin alkylation reactions remains underdeveloped (Figure 1B). Furthermore, metal-free catalytic asymmetric olefin alkylation reactions with alcohol derivatives are unprecedented. Unactivated olefins are challenging substrates in asymmetric transformations due to their weak nucleophilicity and lack of directing groups. Activated π-nucleophiles such as allylsilanes,^3–5^ allylboranes,^6^ and silyl enol ethers^7^ have been used to overcome these hurdles, besides asymmetric *Tsuji*–*Trost*-type allylations.^8,9^

In 2014, Carreira reported the first asymmetric allylation of olefins with alcohols in which iridium π-allyl complexes were leveraged to furnish 1,5-dienes with high regio- and enantiocontrol.^10^ Crucially, a strongly acidic sulfonimide cocatalyst was required for the activation of alcohol substrates bearing both an aryl- and vinyl substituent.

We envisioned our imidodiphosphorimidates (IDPis) as bifunctional catalysts to enable both substrate activation and enantioinduction.^11^ Confinement of the IDPi active site provides a well-defined stereochemical environment for enantioselective C–C bond formation through asymmetric counteranion-directed catalysis (ACDC).^12,13^ Furthermore, our group recently reported on IDPi-catalyzed transformations of benzylic acetates.^14^ These developments motivated us to tackle the metal-free asymmetric alkylation of olefins with alcohol derivatives (Figure 1C).

By screening substrates and conditions (see Supporting Information), we identified a model reaction between **1a** and **2a** using IDPi **3a** and *N*,*O*-bis­(trimethylsilyl)­trifluoroacetamide (BSTFA). Importantly, BSTFA acted as a silylium reservoir to deprotosilylate the IDPi, accessing a silylium Lewis acid regime.^11^ This suppressed olefin isomerization and provided a driving force for acetate activation by forming a strong Si–O bond.

Screening IDPi catalysts revealed the indispensability of electron-withdrawing 3,3′-“wings” (Figure 2A), as IDPi **3b** favored elimination (see Supporting Information for further discussion of catalyst optimization). Tuning the wings and sulfonyl “core” (**3c**–**3e**) gave **3e** as the optimal catalyst. Finally, decreasing the reaction temperature and concentration afforded **4a** with 79% yield (75% isolated) and an e.r. of 96:4.

**Figure 2:**
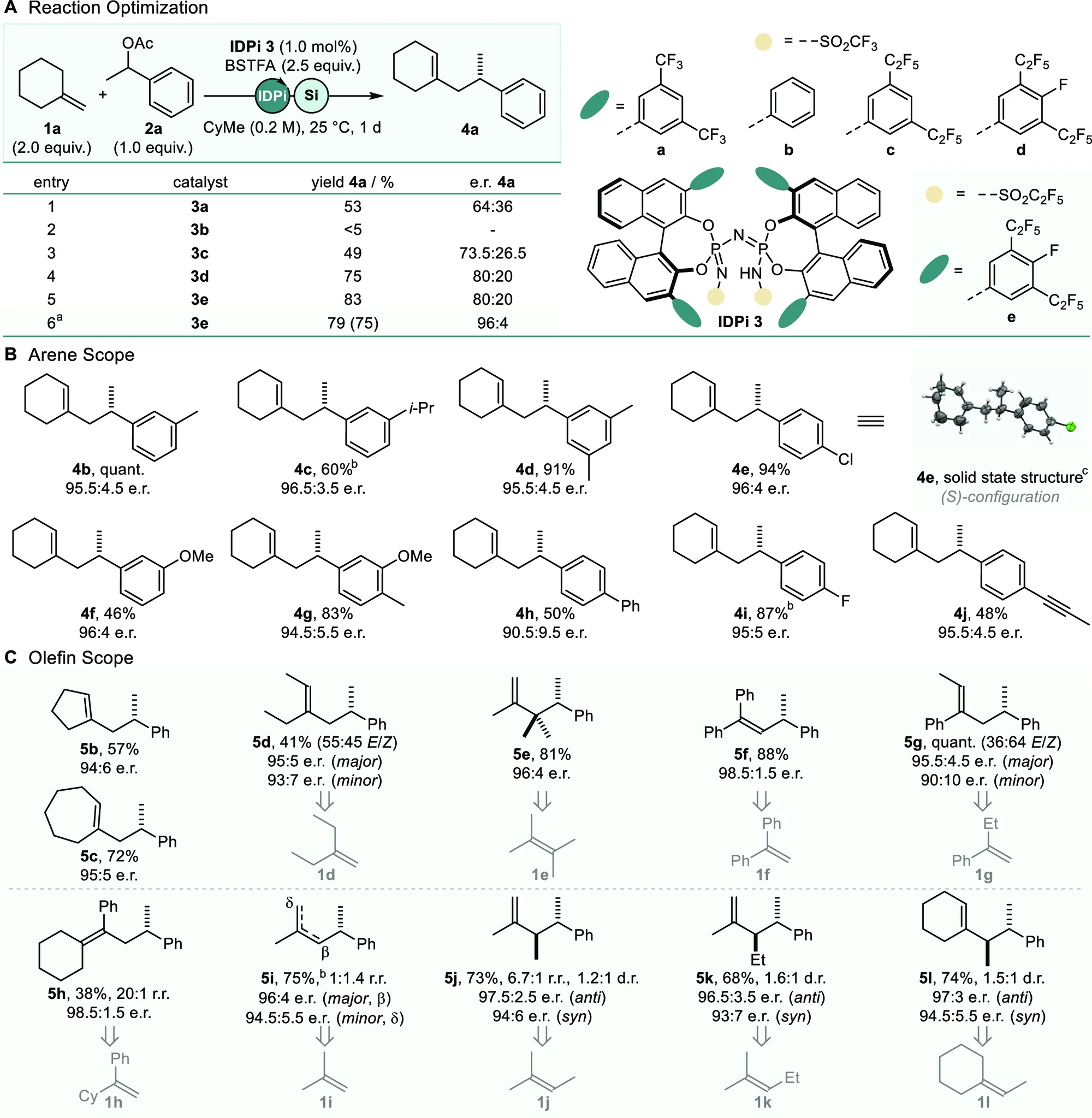
Development of an asymmetric olefin alkylation. (A) Discovery and optimization. Reactions performed at 0.02 mmol scale. Yields determined by ^1^H NMR with mesitylene internal standard. Yield in brackets: isolated yield at 0.2 mmol scale. (B) Arene scope and (C) olefin scope. Scope conditions: acetate **2** (0.2 mmol, 1.0 equiv), olefin **1** (8.0 equiv), IDPi **3e** (2.5 mol %), BSTFA (2.5 equiv), CyMe (0.05 M), −60 °C, 3 d. Yields are isolated yields after column chromatography. Reactions performed at 0.2 mmol scale. ^a^ **3e** (2.5 mol %), **1a** (8.0 equiv), −60 °C, in CyMe (0.05 M), ^b^–70 °C, and ^c^thermal ellipsoids drawn at 50% probability level; two TFM molecules omitted for clarity. Color code: white, H; gray, C; green, Cl.

Arene substitution gave *m*-alkyl-substituted **4b**–**4d** with good yields and excellent enantioselectivities (Figure 2B). *p*-Chloro-substitution similarly yielded **4e**, which formed a multicomponent crystal with 1,3,5,7-tetrakis­(2-fluoro-4-methoxyphenyl)-adamantane (TFM).^17^ Using anomalous dispersion in crystal structure determination, we found that (*S*,*S*)-**3e** afforded (*S*)-product. **4f** and **4g** bearing *m*-methoxy substituents were obtained with comparable enantioselectivities, alongside minor separable spirocyclic byproducts. Lastly, **4h**–**4j** were obtained with very good to excellent enantioselectivities.

Investigation of olefins demonstrated that nucleophilic olefins expectedly formed **5b**–**5c** with comparable enantioselectivities (Figure 2C).^18,19^ *E*/*Z*-mixture **5d** demonstrated near-identical enantioselectivities between diastereomers, indicating stepwise C–C bond formation and deprotonation.

Gratifyingly, tetramethylethylene underwent alkylation despite its low nucleophilicity,^19^ yielding product **5e** with excellent enantioselectivity. Furthermore, formal vinylation product **5f** was accessed from **1f**.

Reactions with **1g**–**1i** produced products with excellent enantioselectivities. The formation of *E*/*Z* mixture **5g** indicated preferential deprotonation toward the least sterically hindered position, whereas **5h**–**5i** favored the thermodynamic products. Although no byproducts were observed, incomplete conversion led to a moderate yield of **5h**, possibly due to increased steric hindrance on **1h**. Separation of the regioisomers of **5i** was achieved on silica gel impregnated with AgNO_3_ by virtue of their distinct coordination to silver­(I).^20^

Finally, trisubstituted olefins were investigated. **1j** preferentially yielded 1,1-disubstitued *anti*-**5j**.^21^ Interestingly, insertion of an additional methylene unit in **1k** resulted in regioselective deprotonation toward the 1,1-disubstituted alkene. We hypothesize that a gradual increase in steric demand from the methyl- and ethyl substituents in **5j** and **5k** compared to **5i** prevents the cationic intermediates from entering the thermodynamic “Zaitsev” deprotonation pathway. Slight diastereomeric enrichment was observed for **5k** compared to **5j**, a trend which carried over to **5l**.

A number of mixed scope entries were explored, followed by transformations toward biologically active compounds (Figure 3A). First, olefins **1b** and **1e** were benzylated to give **6a**–**6d**, demonstrating compatibility with *p*-bromo- and terminal alkyne groups and chemoselectivity toward terminal olefins and allyl benzyl ethers.

**Figure 3:**
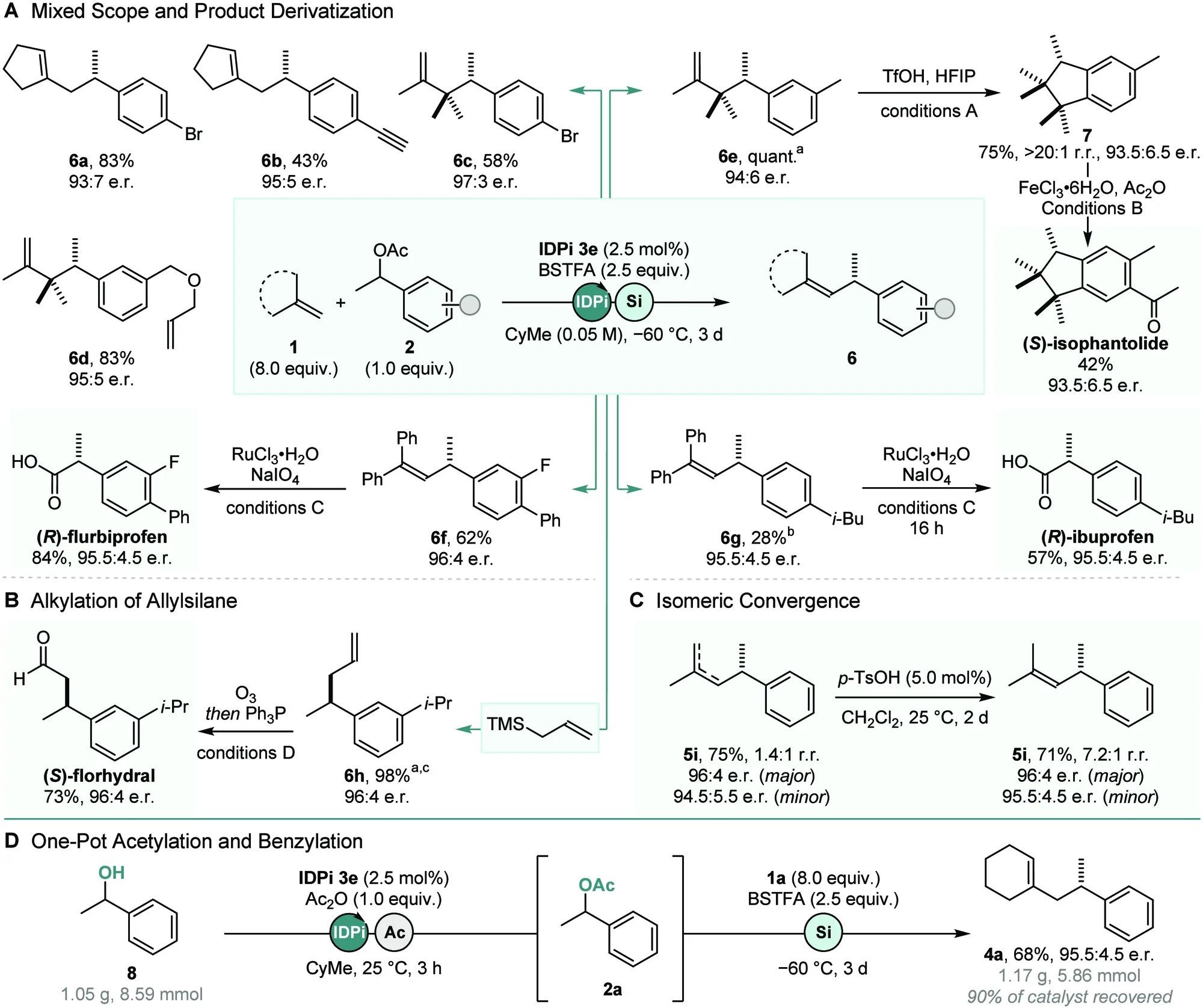
Application of the olefin benzylation reaction. (A) Mixed scope and product derivatization. Yields are isolated yields after column chromatography. Reactions performed at 0.2 mmol scale. (B) Alkylation of allylsilane in (*S*)-florhydral synthesis. (C) Isomeric convergence of **5i**. (D) One-pot acetylation and benzylation. Conditions A: TfOH (2.0 equiv), HFIP, 25 °C, 10 min. Conditions B: FeCl_3_·6H_2_O (5.0 mol %), Ac_2_O (3.0 equiv), propylene carbonate, 80 °C, 2 d. Conditions C: RuCl_3_·H_2_O (2.0 mol %), NaIO_4_ (4.1 equiv), MeCN/CCl_4_/H_2_O, 25 °C, 1 h. Conditions D: O_3_, CH_2_Cl_2_/MeOH, Ph_3_P (1.1 equiv), −78→25 °C, 1 h. ^a^–70 °C; ^b^–85 °C; ^c^allyltrimethylsilane (8.0 equiv) instead of BSTFA and olefin.

Preparation of *m*-methylated **6e** from **1e** enabled the two-step synthesis of a novel isomer of known musk odorant phantolide: (*S*)-isophantolide. The preparation of phantolide analogues is highly relevant because aromatic musk derivatives often possess desirable odorant properties and lower bioaccumulation propensity.^22–24^

Cyclizing **6e** according to a modified procedure by Fu yielded **7** with excellent regioselectivity.^25^ The TfOH-mediated reaction occurred with near-complete stereoretention, contrary to an earlier report of a similar AlCl_3_-catalyzed cyclization.^26^ Subsequent *Friedel*–*Crafts* acylation using conditions from Cherif delivered (*S*)-isophantolide in 32% overall yield.^27^

**1f** was applied to the synthesis of nonsteroidal anti-inflammatory drugs (NSAIDs). Benzylation products **6f**–**6g** were transformed into (*R*)-flurbiprofen and (*R*)-ibuprofen with near-complete stereoretention according to a *Djerassi*–*Rylander* oxidation under *Sharpless* conditions reported by Bäckvall and others.^28,29^

In lieu of α-olefins undergoing alkylation (see Supporting Information), allyltrimethylsilane was explored as a C_3_-nucleophile to obtain **6h** with excellent enantioselectivity (Figure 3B). Ozonolysis then gave (*S*)-florhydral, the more potent enantiomer of this synthetic muguet odorant.^30^

We were eager to isomerically enrich olefinic mixture **5i**. Screening a variety of catalysts (see Supporting Information), we found that subjecting **5i** to catalytic *p*-TsOH significantly enriched the thermodynamic product without racemization or cyclization side-reactivity (Figure 3C).

During reaction optimization, the acetate provided a reactivity handle at low temperatures. We envisioned that telescoping acid-catalyzed acetylation^31^ and olefin alkylation in one pot would improve step economy. Consequently, we sought to employ the IDPi in both the Brønsted- and Lewis acidic regimes (Figure 3D). Screening of reagents and conditions (see Supporting Information) revealed efficient acetylation of 1-phenylethanol (**8**) using Ac_2_O and **3e**. At gram scale, acetylation was followed by olefin alkylation according to the optimized conditions, affording **4a** with comparable yield and enantioselectivity.

Revisiting our inspiration of geranyl pyrophosphate biosynthesis, we explored olefin allylation. By regulating reaction temperature, we were able to achieve allylation of **1a** with (*E*)-pent-3-en-2-yl acetate, affording **10a** with moderate yield and an e.r. of 94.5:5.5 (Figure 4). Encouraged by this result, we explored 3-substitution, affording products **10b**–**10d** with superior yields and enantioselectivities. The reaction toward **10d** produced a number of byproducts related to cationic cyclization, which were removed using AgNO_3_-silica. Surprisingly, halogenated substrates were smoothly converted to **10e**–**10f**. Multicomponent crystallization of **10f** with TFM allowed us to unambiguously assign the (*S*)-configuration using single-crystal X-ray diffraction (SC-XRD).

**Figure 4:**
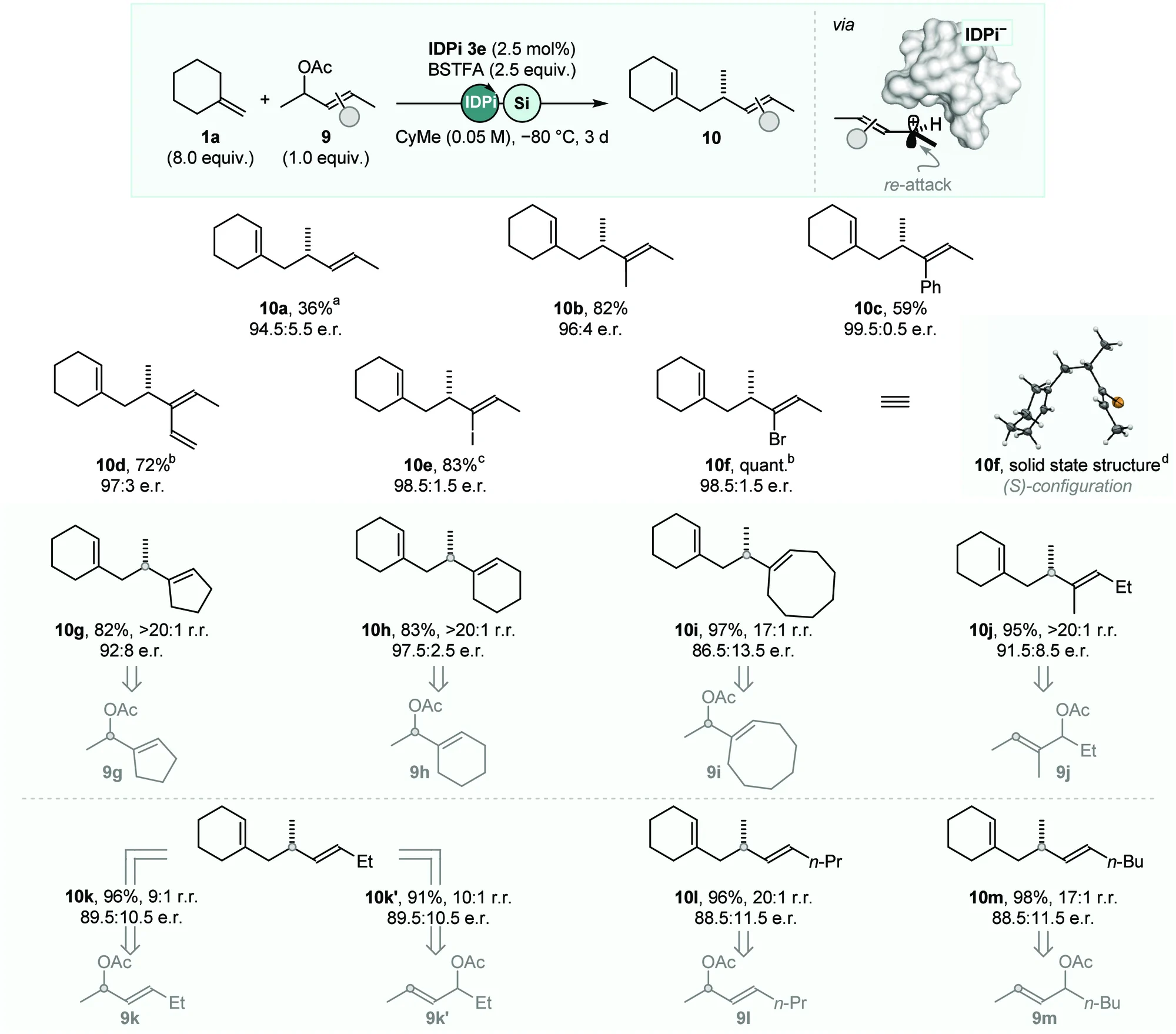
Olefin alkylation with allylic acetates. Yields are isolated yields after column chromatography. Reactions performed at 0.2 mmol scale. All products obtained with shown double bond geometry in ratios >20:1. ^a^–90 °C, ^b^–50 °C, and ^c^–60 °C. ^d^Thermal ellipsoids drawn at 50% probability level; two TFM molecules and one **10f** molecule omitted for clarity. Color code: white, H; gray, C; brown, Br.

Next, allylic acetates possessing nonequivalent electrophilic sites were explored for the regioselective allylation of **1a**. Substrates **9g**–**9i** were cleanly converted to **10g**–**10i** with very good yields and enantioselectivities. Nucleophilic attack occurred almost exclusively at the least sterically hindered site, providing complementary selectivity to Carreira’s synthesis of branched 1,5-dienes.^10^

Allylation with linear **9j** yielded **10j** with excellent regioselectivity, highlighting the confined catalyst’s ability to distinguish between methyl- and ethyl substituents. Moreover, reactions with allylic transposition isomers **9k**–**9k′** yielded near-identical results for **10k**–**10k′**, suggesting an allylic cation intermediate. Methylene-extended acetates **9l**–**9m** demonstrated high regioselectivities. However, additional conformational freedom in **10k**–**10m** may cause steric clash with the catalyst, decreasing enantioselectivity.

Reaction monitoring was performed to probe the reaction mechanism (Figure 5A). At high conversion, a starkly decreased reaction rate was observed. Furthermore, **2a** became significantly enantioenriched early on, plateauing at higher conversions. Variable time normalization analysis (VTNA) of reactions including TMSOAc (0.0–1.0 equiv) revealed that this byproduct acts as a competitive inhibitor with an estimated partial order of −0.5.^32^

**Figure 5:**
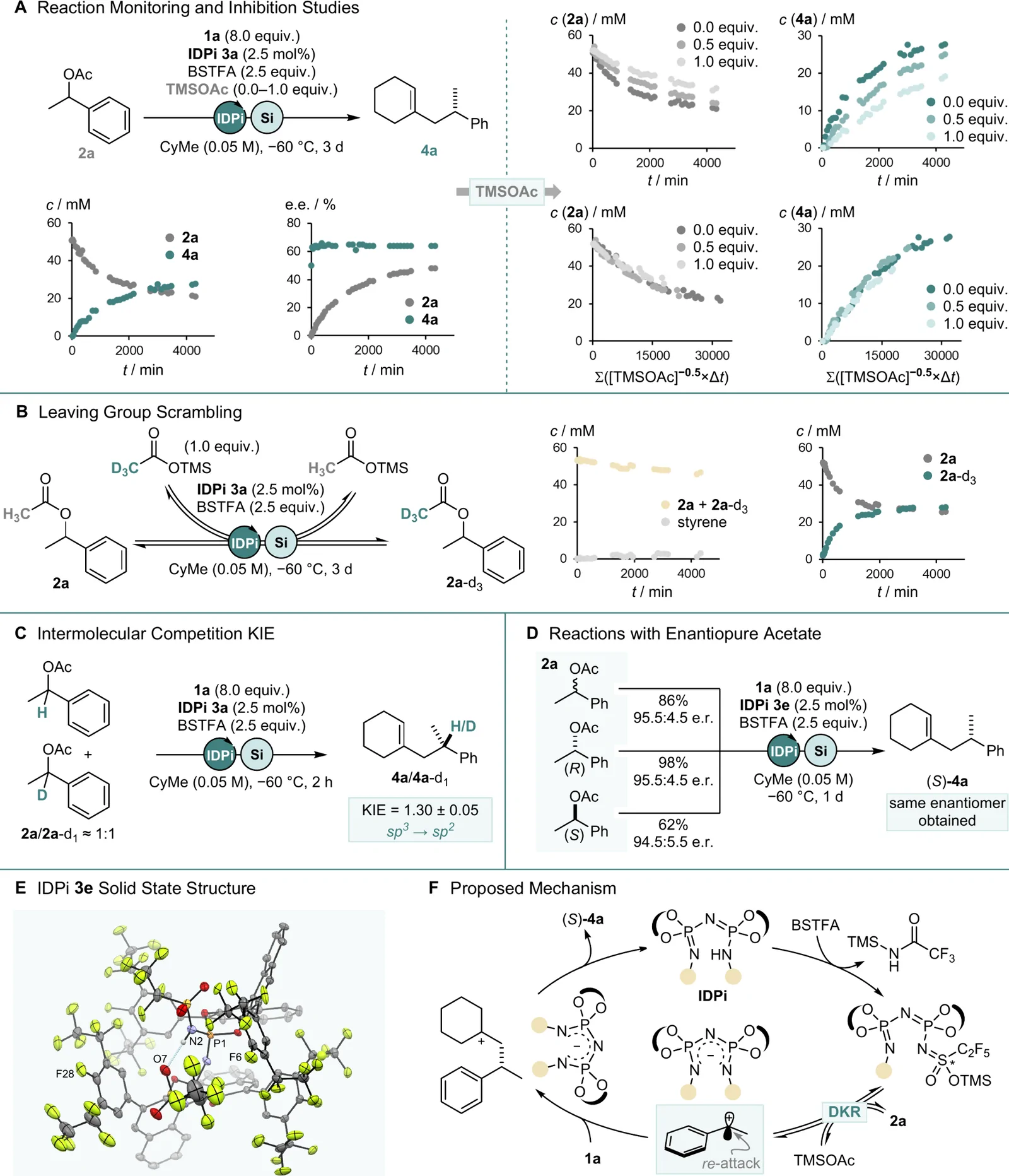
Mechanistic investigations. Yields determined by ^1^H NMR using mesitylene or 1,2-dichloroethane internal standard. (A) Reaction monitoring and TMSOAc inhibition. (B) Leaving group scrambling. (C) Intermolecular competition KIE. (D) Reactions with enantiopure starting materials. (E) Solid-state structure of **3e**. Thermal ellipsoids drawn at 50% probability level. Disordered parts (CF_3_-fluorine atoms) and apolar hydrogens omitted for clarity. Intramolecular H-bond highlighted in light blue. Color code: white, H; gray, C; light blue, N; red, O; light green, F; orange, P; yellow, S. (F) Proposed mechanism.

Competitive nucleophilicity toward the cationic intermediate could underlie inhibition by TMSOAc, causing reversible ion-pair formation. Leaving-group scrambling studies were performed to investigate this hypothesis (Figure 5B). **2a** was subjected to reaction conditions lacking an olefin and equimolar TMSOAc-*d*_3_. Monitoring **2a**/**2a**-*d*_3_ revealed convergence toward a 1:1 mixture, while decomposition remained minimal. These findings confirmed the reversibility of leaving group activation, clarifying byproduct inhibition. Additionally, leaving-group scrambling confirms that **2a** undergoes in situ racemization, which becomes dominant at higher concentrations of TMSOAc, causing a dynamic kinetic resolution (DKR).

A large normal secondary kinetic isotope effect (KIE) of 1.30 corroborated significant sp^3^→sp^2^ rehybridization (Figure 5C)^33^ and suggested that ionization could be rate-limiting in the early phases since there is no upstream sp^3^→sp^2^ rehybridization and catalyst silylation is fast.^34^

An S_N_2 pathway was ruled out using enantiopure starting materials (Figure 5D), which afforded the same enantiomer of **4a** with comparable enantioselectivities. Moreover, higher conversion for (*R*)-**2a** is incompatible with an S_N_2 pathway, leading to (*S*)-**4a** considering Walden inversion. Recovered starting material was enriched for (*S*)-**2a** in all cases (see Supporting Information), substantiating in situ racemization.

We obtained the solid-state structure of IDPi **3e** using SC-XRD (Figure 5E), which revealed that fluorine atoms F6 and F28 flank the acidic core of the catalyst. Nonclassical C–H···F interactions between these fluorine atoms and the substrate could explain the high enantioinduction observed during reaction optimization. Furthermore, a N2–O7 distance of 2.710 Å and a slightly elongated P1–N2 bond (1.653(3) Å) indicate an intramolecular hydrogen bond.

These mechanistic experiments allowed us to propose a mechanistic hypothesis (Figure 5F). Deprotosilylation generates fast-equilibrating silylium-IDPi diastereomers as the resting state and facilitates a self-drying cycle.^11,34^ Acetate activation then furnishes an ion pair that can undergo forward reaction with the olefin in the enantiodetermining C–C bond-forming step, giving the product after deprotonation. The competing backward reaction with TMSOAc causes racemization of the acetate, prompting a DKR.

This study presents the first organocatalytic intermolecular asymmetric olefin alkylation reaction with alcohol derivatives. Key to the discovery of the method was the development of a highly acidic IDPi catalyst that allowed for the activation of unbiased acetate electrophiles. The use of the IDPi as a silylium Lewis acid proved crucial to suppress olefin isomerization, introduce a self-drying and repair cycle, and provide a driving force for electrophile activation by forming a strong Si–O bond. Using the developed method, a number of enantioenriched odorants and pharmaceuticals were accessed. Experimental mechanistic studies evidenced reversible ion pair formation and an enantioconvergent cationic DKR. Notably, this method encompasses the first organocatalytic regio- and enantioselective olefin allylation, providing a complementary approach to *Tsuji*–*Trost*-type allylations proceeding through π-allyl metal complexes.

## Supplementary Material



## Notes

Experimental procedures, characterization data for new compounds, detailed discussion of reaction optimization, limitations of the method, and mechanistic investigations (PDF)

## Abbreviations

ACDC: asymmetric counteranion-directed catalysis
BSTFA: *N*,*O*-bis­(trimethylsilyl)­trifluoroacetamide
CyMe: methylcyclohexane
DKR: dynamic kinetic resolution
HFIP: 1,1,1,3,3,3-hexafluoropropan-2-ol
HOPP: pyrophosphoric acid
IDPi: imidodiphosphorimidate
IPPS: isoprenyl pyrophosphate synthase
KIE: kinetic isotope effect
NSAID: nonsteroidal anti-inflammatory drugs
*p*-TsOH: *p*-toluenesulfonic acid
SC-XRD: single-crystal X-ray diffraction
TMS: trimethylsilyl
TMSOAc: trimethylsilyl acetate
TFM: 1,3,5,7-tetrakis­(2-fluoro-4-methoxyphenyl)-adamantane
TfOH: trifluoromethanesulfonic acid or triflic acid
VTNA: variable time normalization analysis
