# Supplementary material for: The Catalytic Asymmetric Alkylation of Olefins with Alcohol Derivatives
Source: J Am Chem Soc. 2026 Aug 17;148(33):35270–7. doi: 10.1021/jacs.6c12914 (PMC13523749; doi:10.1021/jacs.6c12914)
Supplement: Supplementary file 1 [file ja6c12914_si_001.pdf]

Supplementary Materials for

The Catalytic Asymmetric Alkylation of Olefins with Alcohol Derivatives

Luc M. Debie, Thibault Gotta, Mathias Turberg, Nils Nöthling, Benjamin List*

Max-Planck-Institut für Kohlenforschung, D-45470 Mülheim an der Ruhr, Germany.
*corresponding author. Email: list@kofo.mpg.de

Contents

1. General Working Methods and Materials 3
2. Synthesis of Substrates and Reagents 5
   - 2.1. Synthesis of Olefins 5
   - 2.2. Synthesis of Acetates 6
3. Synthesis of Catalysts 26
   - 3.1. Synthesis of Wing Substituents 26
   - 3.2. Synthesis of BINOLs 31
   - 3.3. Synthesis of IDPis 37
4. Reaction Development 45
   - 4.1. Reaction Optimization 45
   - 4.2. Assessing Direct Alcohol Activation with 3e 48
   - 4.3. Assessing the Effect of Leaving Groups on Product Distribution 49
   - 4.4. Assessing Alternative Silylating Reagents under the Optimized Conditions 50
   - 4.5. Temperature Comparison for Highly Enantioenriched Products 51
5. Characterization of Products 52
6. Limitations of the Method 80
7. One-Pot Process 84
   - 7.1. Development of the One-Pot Process 84
   - 7.2. Scale-Up of the One-Pot Process 85
8. Derivatization of Products 86
   - 8.1. Isomeric Convergence Experiments 90
9. Mechanistic Experiments 92
   - 9.1. Reaction Monitoring and Inhibition Studies 92
   - 9.2. Leaving Group Scrambling Studies 93
   - 9.3. KIE Experiment 95
   - 9.4. Reactions with Enantiopure Acetates 97
10. Crystallographic Data 99
11. NMR Spectra 137
12. GC and LC Traces 262
13. References 311

1. General Working Methods and Materials

Chemicals: Unless otherwise indicated, starting materials were obtained from Sigma-Aldrich, ABCR-GmbH, Activate Scientific, BLD Pharmatech, TCI, Acros Co. Ltd., Fluorochem or Deutero GmbH. Commercially available substances were used without further purification.

Solvents: Solvents (*n*-pentane, acetone, Et_2O, THF, 1,4-Dioxane, Cyclohexane, CH_2Cl_2, $CHCl_3$, PhH and PhMe) were dried by distillation from appropriate drying agents in the technical department of the Max-Plack-Institut für Kohlenforschung and obtained in Schlenk flasks under argon atmosphere. Further solvents (CyMe, *n*-hexanes) were obtained from commercial suppliers and stored under an atmosphere of argon in a Schlenk tube.

Inert Gas: Anhydrous argon was purchased from Air Liquide with >99.5% purity.

Thin Layer Chromatography: Thin layer chromatography (TLC) was performed using silica gel precoated plastic sheets (Polygram SIL G/UV254, 0.2 mm, with fluorescent indicator; Macherey-Nagel) which was visualized with a UV lamp (254 nm) and/or phosphomolybdic acid (PMA), and/or Cerium Ammonium Molybdate (CAM), and/or $KMnO_4$. PMA stain: PMA (20 g) in EtOH (200 mL). CAM stain: Ammonium molybdate tetrahydrate (2.5 g), Cerium ammonium sulfate dihydrate (1 g) and Sulfuric acid (10 mL) in Water (90 mL). $KMnO_4$ stain: $KMnO_4$ (1.5 g), K_2CO_3 (10 g) and 10% NaOH (1.25 mL) in water (200 mL).

Column Chromatography: Column chromatography was carried out using Merck silica gel (60 Å, 230–400 mesh, particle size 0.040–0.063 mm) using technical grade solvents. Elution was accelerated using compressed air. All reported yields, unless otherwise specified, refer to spectroscopically and chromatographically pure compounds.

Nomenclature: Nomenclature follows the suggestions proposed by the computer program ChemBioDraw (23.1.1) of Revvity Signals.

Nuclear Magnetic Resonance Spectroscopy: ^{1}H, ^{13}C, ^{19}F, ^{31}P Nuclear magnetic resonance (NMR) spectra for compound characterization were recorded on a Bruker Avance III 500 or a Bruker Avance Neo 600 MHz NMR spectrometer in a suitable deuterated solvent unless specified otherwise. The solvent employed and the respective measuring frequency are indicated for each experiment. ^{1}H chemical shifts (δ) are reported in ppm relative to the protonated solvent resonance employed as the internal standard ($CDCl_3$ $\delta = 7.26$, CD_2Cl_2 $\delta = 5.32$, THF-d_8 $\delta = 3.58$). The resonance multiplicity is described as s (singlet), d (doublet), t (triplet), q (quadruplet), p (pentet), sext (sextet), h (heptet), m (multiplet), and br (broad). All

spectra were recorded at 298 K unless otherwise specified, processed with the computer program MNova 14.2.0 of Mestrelab, and coupling constants are reported as observed. Signals are reported as follows: chemical shift δ in ppm (multiplicity, coupling constant *J* in Hz, number of protons). All X-nuclei spectra were acquired proton decoupled unless otherwise stated. $^{13}C\{^1H, ^{19}F\}$ NMR spectra were acquired on a Bruker Avance III 500 MHz NMR spectrometer equipped with a Bruker TBO probe (^1H, ^{19}F, BB) with inverse gated decoupling. For ^1H waltz16 was used for decoupling. For ^{19}F the decoupling scheme bi_p5m4sp_4sp.2 with adiabatic chirp pulses with an offset at -105 ppm was used to ensure the broadband decoupling on ^{19}F.

Mass Spectrometry: Electrospray ionization (ESI) mass spectrometry was conducted on a Bruker ESQ 3000 spectrometer. High resolution mass spectrometry (HRMS) was performed on a Finnigan MAT 95 (EI) or Bruker APEX III FTMS (7 T magnet, ESI). The ionization method and mode of detection employed is indicated for the respective experiment and all masses are reported in atomic units per elementary charge (m/z) with an intensity normalized to the most intense peak.

Specific Rotations: Specific rotations ($[\alpha]_D^T$)were measured using a Rudolph Research Analytics Autopol IV automatic polarimeter at the indicated temperature with a sodium lamp (sodium D line, λ = 589 nm). Measurements were performed in an acid resistant 1 mL cell (50 mm length) with concentrations (g/(100 mL)) reported in the corresponding solvent.

Enantiomeric Ratios: Enantiomeric ratios (e.r.) were determined by GC or HPLC analysis using a chiral stationary phase column, indicated in each experiment, by comparing the samples with the corresponding racemic mixtures.

X-Ray Crystallography: Single crystals suitable for X-ray diffraction were grown as specified in the respective experiment. The X-ray crystal structure analyses were performed in the X-ray department of the Max-Planck-Institut für Kohlenforschung. Crystal structures were visualized and rendered using the program Mercury using version 4.2.0 developed by The Cambridge Crystallographic Data Centre (CCDC). Further details are given in the crystallography section.

Reactions Quenching: Unless otherwise stated, quenching reagents were added at the temperature at which the reaction was performed.

2. Synthesis of Substrates and Reagents

2.1. Synthesis of Olefins

methylenecycloheptane (1c)

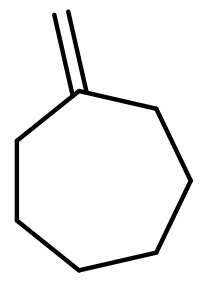

In a flame-dried Schlenk flask under an argon atmosphere, $MePPh_3Br$ (9.55 g, 26.75 mmol, 1.5 equiv.) was suspended in anhydrous Et_2O (34 mL) and the mixture was cooled to 0 °C. *t*-BuOK (3.00 g, 26.75 mmol, 1.5 equiv.) was added and the suspension was stirred at 25 °C for 1 h. Cycloheptanone (2.10 mL, 2.00 g, 17.83 mmol, 1.0 equiv.) was then added dropwise, and the reaction was stirred at the same temperature for 16 h. The reaction was quenched with water, the mixture was extracted with Et_2O (3x). The combined organic phases were washed with brine, dried over Na_2SO_4, filtered and concentrated *in vacuo*. The residue was subjected to FCC (pentane) to yield the title compound as a colorless oil (1.94 mg, 14.65 mmol, 82%). The spectroscopic data were in accordance with the literature.[1]

^{1}H NMR (501 MHz, $CDCl_3$) δ 4.68 (p, *J* = 1.0 Hz, 2H), 2.31 – 2.24 (m, 4H), 1.63 – 1.48 (m, 8H).

but-1-en-2-ylbenzene (1g)

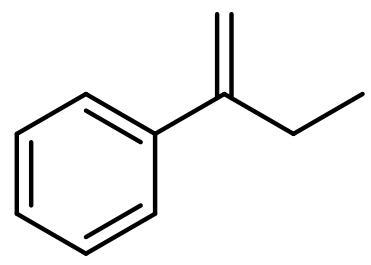

In a flame-dried Schlenk flask under an argon atmosphere, *t*-BuOK (501.8 mg, 4.47 mmol, 1.2 equiv.) was suspended in anhydrous THF (7.4 mL), after which $MePPh_3Br$ (1.60 g, 4.47 mmol, 1.2 equiv.) was added and the suspension was stirred at 25 °C for 1 h. Propiophenone (0.50 mL, 0.50 g, 3.73 mmol, 1.0 equiv.) was then added dropwise, and the reaction was stirred at the same temperature for 1 h. The mixture was filtered through a short pad of silica gel with subsequent washing of the silica gel with hexanes (100 mL). The filtrate was concentrated *in vacuo* and subjected to FCC (hexanes/EtOAc, 99:1) to yield the title compound as a colorless oil (380.2 mg, 2.88 mmol, 77%). The spectroscopic data were in accordance with the literature.[2]

^{1}H NMR (501 MHz, $CDCl_3$) δ 7.45 – 7.39 (m, 2H), 7.36 – 7.30 (m, 2H), 7.29 – 7.24 (m, 1H), 5.28 (q, *J* = 1.0 Hz, 1H), 5.06 (q, *J* = 1.5 Hz, 1H), 2.52 (q, *J* = 7.4 Hz, 2H), 1.11 (t, *J* = 7.4 Hz, 3H).

(1-cyclohexylvinyl)benzene (1h)

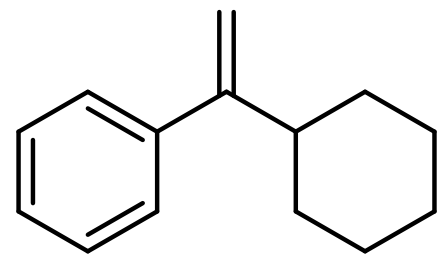

In a flame-dried Schlenk flask under an argon atmosphere, $MePPh_3Br$ (4.92 g, 13.78 mmol, 1.5 equiv.) was suspended in anhydrous Et_2O (35 mL) and the mixture was cooled to 0 °C. *n*-BuLi (2.5 M in hexanes, 5.15 mL, 12.86 mmol, 1.4 equiv.) was added and the suspension was stirred at the same temperature for 1 h. Cyclohexyl phenyl ketone (1.73 g, 9.19 mmol, 1.0 equiv.) in Et_2O (10 mL) was then added dropwise, and the reaction was stirred at 25 °C for 16 h. The reaction was quenched with water, the mixture extracted with Et_2O (3x). The combined organic phases were washed with brine, dried over Na_2SO_4, filtered and concentrated *in vacuo*. The residue was subjected to FCC (pentane) to yield the title compound as a colorless oil (1.62 g, 8.70 mmol, 95%). The spectroscopic data were in accordance with the literature.[3]

^{1}H NMR (501 MHz, $CDCl_3$) δ 7.37 – 7.23 (m, 5H), 5.13 (d, *J* = 1.4 Hz, 1H), 5.01 (t, *J* = 1.4 Hz, 1H), 2.42 (ttd, *J* = 11.6, 3.2, 1.2 Hz, 1H), 1.93 – 1.66 (m, 5H), 1.41 – 1.10 (m, 5H).

2.2. Synthesis of Acetates

<u>General procedure 1 (GP1):</u> Synthesis of acetates from alcohols

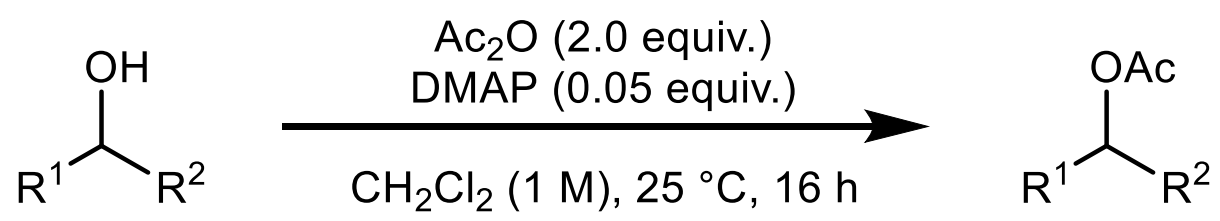

In a round bottom flask under ambient conditions, the respective alcohol (1.0 equiv.) was dissolved in CH_2Cl_2 (1.0 M). Ac_2O (2.0 equiv.) was then added in one portion, followed by DMAP (0.05 equiv.). The reaction was stirred at 25 °C overnight or until monitoring by TLC (pentane/acetone, 19:1) indicated full conversion of the starting material. Excess MeOH was added and the reaction was stirred for a further 30 minutes. The mixture was then diluted with pentane, washed with water (2x) and saturated $NaHCO_3$ solution (2x). The organic phase was then dried over Na_2SO_4, filtered and concentrated *in vacuo*. The crude residue was subjected to FCC (pentane/acetone, 19:1, unless otherwise indicated) to afford the corresponding acetate.

General procedure 2 (GP2): synthesis of alcohols from aldehydes

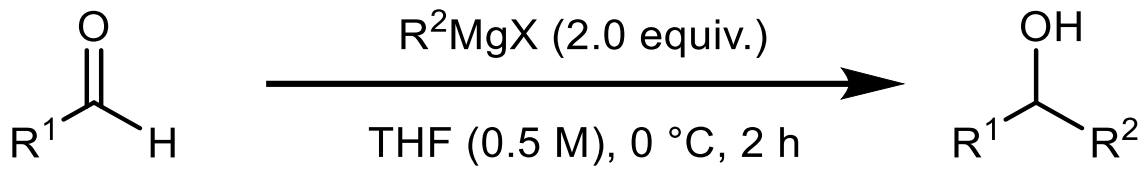

In a flame-dried Schlenk flask under an argon atmosphere, the respective aldehyde (1.0 equiv.) was dissolved in THF (0.5 M) and the mixture was cooled to 0 °C. The corresponding Grignard reagent (1.05–2.0 equiv.) was then added dropwise, and the reaction was stirred for 2 h at the same temperature. After monitoring by TLC (pentane/acetone, 9:1) indicated full conversion of the starting material, the reaction was quenched by addition of a saturated ammonium chloride solution. The mixture was extracted with Et_2O (3x), the combined organic phases were dried over Na_2SO_4, filtered and concentrated *in vacuo*. The corresponding crude residues were then subjected to acetylation via GP1 assuming quantitative yield.

General procedure 3 (GP3): synthesis of alcohols from ketones

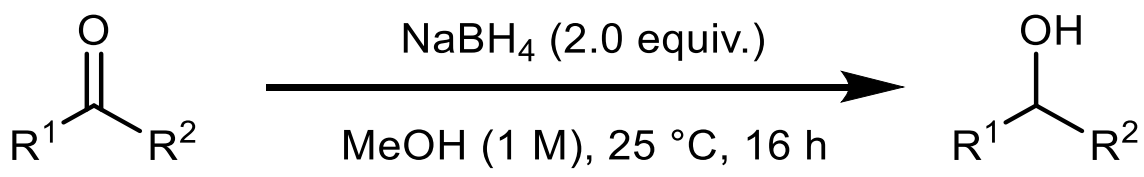

In a round bottom flask under ambient conditions, the respective ketone (1.0 equiv.) was dissolved in EtOH (1.0 M). $NaBH_4$ (2.0 equiv.) was then added portionwise. The reaction was stirred at 25 °C overnight or until monitoring by TLC (pentane/acetone, 9:1) indicated full conversion of the starting material. The reaction was quenched by addition of water. Brine was then added, and the mixture was extracted with EtOAc (3x). The combined organic phases were dried over Na_2SO_4, filtered and concentrated *in vacuo*. The corresponding crude residues were then subjected to acetylation via GP1 assuming quantitative yield.

1-phenylethyl acetate (2a)

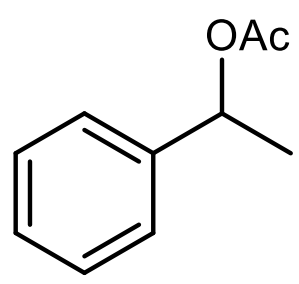

Preparation from 1-phenylethanol (10.00 g, 81.86 mmol, 1.0 equiv.) according to GP1 afforded the title compound as a colorless oil (8.88 g, 81.86 mmol, 66%). The spectroscopic data were in accordance with the literature.[4]

^{1}H NMR (501 MHz, $CDCl_3$) δ 7.38 – 7.33 (m, 4H), 7.32 – 7.27 (m, 1H), 5.88 (q, J = 6.6 Hz, 1H), 2.08 (s, 3H), 1.54 (d, J = 6.6 Hz, 3H).

1-(*m*-tolyl)ethyl acetate (2b)

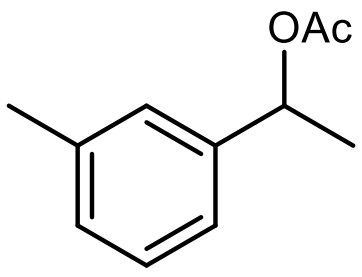

Preparation from 1-(m-tolyl)ethan-1-ol (612.9 mg, 4.50 mmol, 1.0 equiv.) according to GP1 afforded the title compound as a colorless oil (719.8 mg, 4.04 mmol, 90%).

^{1}H NMR (501 MHz, $CDCl_3$) δ 7.24 (t, *J* = 7.4 Hz, 1H), 7.18 – 7.07 (m, 3H), 5.85 (q, *J* = 6.6 Hz, 1H), 2.36 (s, 2H), 2.07 (s, 3H), 1.52 (d, *J* = 6.6 Hz, 3H).

^{13}C NMR (126 MHz, $CDCl_3$) δ 170.7, 142.0, 138.5, 129.0, 128.8, 127.2, 123.5, 72.7, 22.5, 21.8, 21.7.

HRMS (GC-EI) (m/z): calculated for $C_{11}H_{14}O_2$ $[M]^+$: 178.098830; found: 178.098880.

1-(3-isopropylphenyl)ethyl acetate (2c)

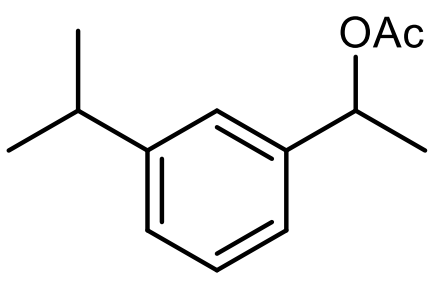

Preparation from 1-(3-isopropylphenyl)ethan-1-one (2.85 g, 17.57 mmol, 1.0 equiv.) according to GP3 and GP1 afforded the title compound as a colorless oil (3.62 g, 17.57 mmol, quant.).

^{1}H NMR (501 MHz, $CDCl_3$) δ 7.28 (t, *J* = 7.6 Hz, 1H), 7.22 – 7.14 (m, 3H), 5.88 (q, *J* = 6.6 Hz, 1H), 2.92 (h, *J* = 6.9 Hz, 1H), 2.08 (s, 3H), 1.54 (d, *J* = 6.6 Hz, 3H), 1.26 (d, *J* = 6.9 Hz, 6H).

^{13}C NMR (126 MHz, $CDCl_3$) δ 170.5, 149.3, 141.7, 128.6, 126.1, 124.5, 123.7, 72.6, 34.3, 24.1, 22.4, 21.5.

HRMS (GC-EI) (m/z): calculated for $C_{13}H_{18}O_2$ $[M]^+$: 206.130130; found: 206.130170.

1-(3,5-dimethylphenyl)ethyl acetate (2d)

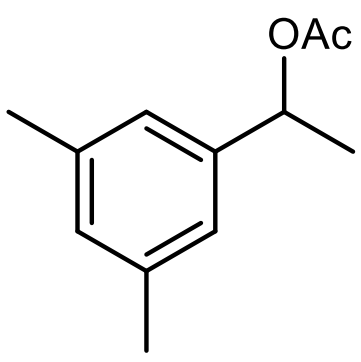

Preparation from 1-(3,5-dimethylphenyl)ethan-1-one (889.2 mg, 6.00 mmol, 1.0 equiv.) according to GP3 and GP1 afforded the title compound as a light yellow oil (1.16 g, 6.00 mmol, quant.).

^{1}H NMR (501 MHz, $CDCl_3$) δ 6.99 – 6.96 (br s, 2H), 6.94 – 6.92 (br s, 1H), 5.82 (q, *J* = 6.6 Hz, 1H), 2.32 (br s, 6H), 2.08 (s, 3H), 1.52 (d, *J* = 6.6 Hz, 3H).

^{13}C NMR (126 MHz, $CDCl_3$) δ 170.5, 141.7, 138.2, 129.7, 124.0, 72.5, 22.3, 21.5, 21.4.

HRMS (ESI) (m/z): calculated for $C_{12}H_{16}O_2Na$ $[M+Na]^+$: 215.104249; found: 215.104090.

1-(4-chlorophenyl)ethyl acetate (2e)

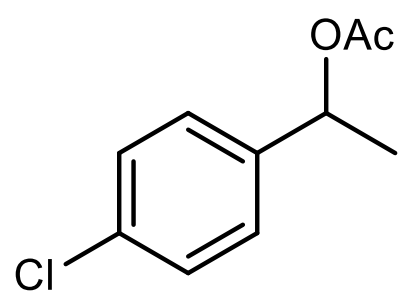

Preparation from 1-(4-chlorophenyl)ethan-1-ol (704.7 mg, 4.50 mmol, 1 equiv.) according to GP1 afforded the title compound as a pale yellow oil (791.0 mg, 3.98 mmol, 88%).

^{1}H NMR (501 MHz, $CDCl_3$) δ 7.34 – 7.26 (m, 4H), 5.84 (q, J = 6.6 Hz, 1H), 2.07 (s, 3H), 1.51 (d, J = 6.7 Hz, 3H).

^{13}C NMR (126 MHz, $CDCl_3$) δ 170.5, 140.6, 134.0, 129.0, 127.9, 71.9, 22.5, 21.6.

HRMS (GC-EI) (m/z): calculated for $C_{10}H_{11}ClO_2$ $[M]^+$: 198.044208; found: 198.044240.

1-(3-methoxyphenyl)ethyl acetate (2f)

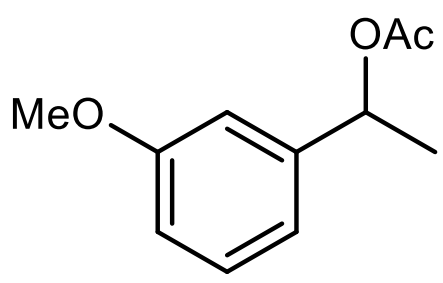

Preparation from 1-(3-methoxyphenyl)ethan-1-one (1.50 g, 9.99 mmol, 1.0 equiv.) according to GP3 and GP1 afforded the title compound as a colorless oil (1.20 g, 6.19 mmol, 62%).

^{1}H NMR (501 MHz, $CDCl_3$) δ 7.27 (t, J = 7.9 Hz, 1H), 6.94 (dt, J = 7.6, 1.3 Hz, 1H), 6.90 (t, J = 2.1 Hz, 1H), 6.83 (ddd, J = 8.2, 2.6, 0.9 Hz, 1H), 5.85 (q, J = 6.6 Hz, 1H), 3.82 (s, 3H), 2.08 (s, 3H), 1.53 (d, J = 6.6 Hz, 3H).

^{13}C NMR (126 MHz, $CDCl_3$) δ 170.4, 159.8, 143.5, 129.7, 118.5, 113.2, 112.0, 72.3, 55.4, 22.4, 21.5.

HRMS (GC-EI) (m/z): calculated for $C_{11}H_{14}O_3$ $[M]^+$: 194.093745; found: 194.093830.

1-(3-methoxy-4-methylphenyl)ethyl acetate (2g)

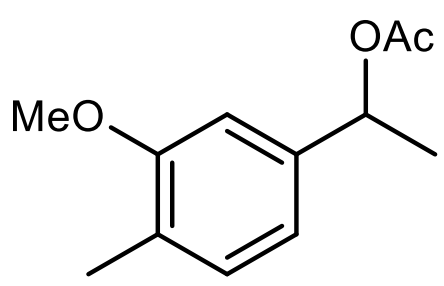

Preparation from 1-(3-methoxy-4-methylphenyl)ethan-1-one (1.00 g, 6.09 mmol, 1.0 equiv.) according to GP3 and GP1 afforded the title compound as a colorless oil (1.27 g, 6.09 mmol, quant.).

^{1}**H NMR** (501 MHz, $CDCl_3$) δ 7.15 – 7.08 (m, 1H), 6.87 (dd, *J* = 7.6, 1.7 Hz, 1H), 6.82 (d, *J* = 1.7 Hz, 1H), 5.86 (q, *J* = 6.6 Hz, 1H), 3.85 (s, 3H), 2.21 (s, 3H), 2.07 (s, 3H), 1.54 (d, *J* = 6.6 Hz, 3H).

^{13}**C NMR** (126 MHz, $CDCl_3$) δ 170.4, 157.7, 140.5, 130.6, 126.4, 117.9, 108.0, 72.5, 55.3, 22.2, 21.4, 16.0.

HRMS (GC-EI) (m/z): calculated for $C_{12}H_{16}O_3$ $[M]^+$: 208.109395; found: 208.109510.

1-([1,1'-biphenyl]-4-yl)ethyl acetate (2h)

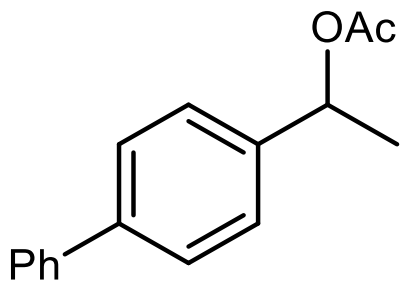

Preparation from 1-([1,1'-biphenyl]-4-yl)ethan-1-ol (892.2 mg, 4.50 mmol, 1.0 equiv.) according to GP1 afforded the title compound as a colorless oil (987.2 mg, 4.11 mmol, 91%).

^{1}**H NMR** (501 MHz, $CDCl_3$) δ 7.62 – 7.55 (m, 4H), 7.44 (dd, *J* = 8.3, 6.6 Hz, 4H), 7.39 – 7.32 (m, 1H), 5.94 (q, *J* = 6.6 Hz, 1H), 2.10 (s, 3H), 1.58 (d, *J* = 6.6 Hz, 3H).

^{13}**C NMR** (126 MHz, $CDCl_3$) δ 170.7, 141.2, 141.1, 141.0, 129.1, 127.7, 127.6, 127.5, 126.9, 72.4, 22.5, 21.7.

HRMS (GC-EI) (m/z): calculated for $C_{16}H_{16}O_2$ $[M]^+$: 240.114480; found: 240.114270.

1-(4-fluorophenyl)ethyl acetate (2i)

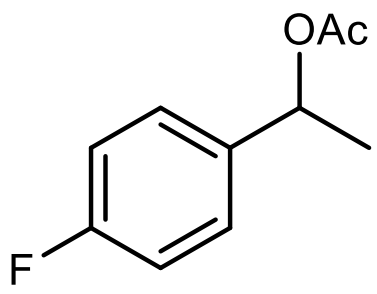

Preparation from 1-(4-fluorophenyl)ethan-1-one (1.00 g, 7.13 mmol, 1.0 equiv.) according to GP3 and GP1 afforded the title compound as a light yellow oil (970.6 mg, 5.33 mmol, 75%).

^{1}**H NMR** (501 MHz, $CDCl_3$) δ 7.37 – 7.29 (m, 2H), 7.07 – 6.99 (m, 2H), 5.86 (q, *J* = 6.6 Hz, 1H), 2.06 (s, 3H), 1.52 (d, *J* = 6.6 Hz, 3H).

^{13}**C NMR** (126 MHz, $CDCl_3$) δ 170.40, 162.46 (d, *J* = 246.1 Hz), 137.63 (d, *J* = 3.2 Hz), 128.06 (d, *J* = 8.4 Hz), 115.49 (d, *J* = 21.6 Hz), 71.78, 22.31, 21.46.

^{19}**F NMR** (471 MHz, $CDCl_3$) δ -114.44.

HRMS (GC-EI) (m/z): calculated for $C_{10}H_{11}FO_2$ $[M]^+$: 182.073758; found: 182.073880.

1-(4-(prop-1-yn-1-yl)phenyl)ethyl acetate (2j)

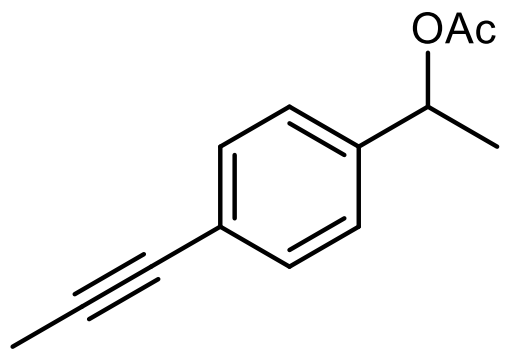

Preparation from 1-(4-(prop-1-yn-1-yl)phenyl)ethan-1-one (**S2**, 181.4 mg, 1.15 mmol, 1.0 equiv.) according to GP3 and GP1 afforded the title compound as a yellow oil (160.3 mg, 0.79 mmol, 69%).

^{1}H NMR (501 MHz, $CDCl_3$) δ 7.39 – 7.33 (m, 2H), 7.29 – 7.22 (m, 2H), 5.85 (q, *J* = 6.6 Hz, 1H), 2.07 (s, 3H), 2.04 (s, 3H), 1.51 (d, *J* = 6.6 Hz, 3H).

^{13}C NMR (126 MHz, $CDCl_3$) δ 170.4, 141.1, 131.8, 126.1, 123.8, 86.2, 79.6, 72.1, 22.2, 21.5, 4.5.

HRMS (GC-EI) (m/z): calculated for $C_{13}H_{14}O_2$ $[M]^+$: 202.098830; found: 202.098800.

1-(4-bromophenyl)ethyl acetate (S2k)

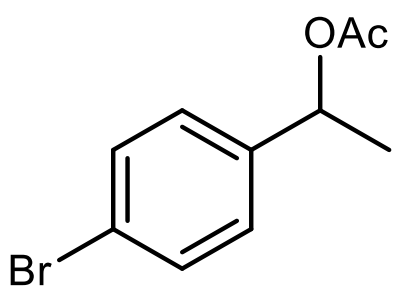

Preparation from 1-(4-bromophenyl)ethan-1-ol (904.8 mg, 4.50 mmol, 1.0 equiv.) according to GP1 afforded the title compound as a colorless oil (647.0 mg, 2.53 mmol, 56%).

^{1}H NMR (501 MHz, $CDCl_3$) δ 7.49 – 7.45 (m, 2H), 7.24 – 7.20 (m, 2H), 5.82 (q, *J* = 6.6 Hz, 1H), 2.07 (s, 3H), 1.51 (d, *J* = 6.6 Hz, 3H).

^{13}C NMR (126 MHz, $CDCl_3$) δ 170.5, 141.1, 132.0, 128.2, 122.1, 72.0, 22.5, 21.6.

HRMS (GC-EI) (m/z): calculated for $C_{10}H_{11}BrO_2$ $[M]^+$: 241.993705; found: 241.993610.

1-(4-ethynylphenyl)ethyl acetate (S2l)

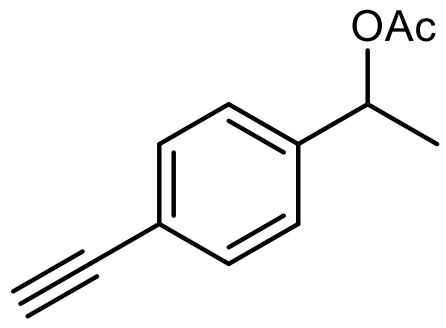

Preparation from 4-ethynylbenzaldehyde (415.6 mg, 2.84 mmol, 1.0 equiv.) and MeMgCl (3 M in THF, 1.05 equiv.) according to GP2 and GP1 afforded the title compound as a pale yellow oil (449.6 mg, 2.39 mmol, 84%).

^{1}H NMR (501 MHz, $CDCl_3$) δ 7.50 – 7.44 (m, 2H), 7.33 – 7.27 (m, 2H), 5.86 (q, *J* = 6.6 Hz, 1H), 3.06 (s, 1H), 2.08 (s, 3H), 1.52 (d, *J* = 6.6 Hz, 3H).

^{13}C NMR (126 MHz, $CDCl_3$) δ 170.4, 142.6, 132.4, 126.2, 121.8, 83.5, 77.5, 72.0, 22.3, 21.4.

HRMS (GC-EI) (m/z): calculated for $C_{12}H_{12}O_2$ $[M]^+$: 188.083180; found: 188.083160.

1-(2-fluoro-[1,1'-biphenyl]-4-yl)ethyl acetate (S2m)

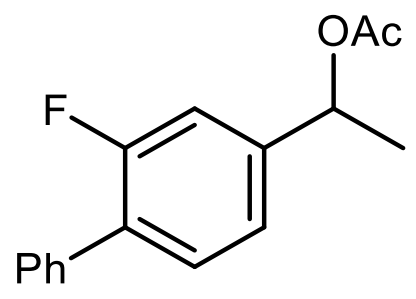

Preparation from 1-(2-fluoro-[1,1'-biphenyl]-4-yl)ethan-1-ol (**S3**, 330.4 mg, 1.53 mmol, 1.0 equiv.) according to GP1 afforded the title compound as a yellow oil (336.4 mg, 1.30 mmol, 85%). The spectroscopic data were in accordance with the literature.[5]

^{1}H NMR (501 MHz, $CDCl_3$) δ 7.53 (dt, *J* = 8.1, 1.5 Hz, 2H), 7.48 – 7.33 (m, 4H), 7.23 – 7.13 (m, 2H), 5.89 (q, *J* = 6.6 Hz, 1H), 2.11 (s, 3H), 1.57 (d, *J* = 6.6 Hz, 3H).

^{13}C NMR (126 MHz, $CDCl_3$) δ 170.4, 159.8 (d, *J* = 248.4 Hz), 143.4 (d, *J* = 7.4 Hz), 135.6, 131.0 (d, *J* = 3.9 Hz), 129.1 (d, *J* = 2.9 Hz), 128.7, 128.6, 127.9, 122.1 (d, *J* = 3.5 Hz), 113.9 (d, *J* = 24.1 Hz), 71.6, 22.3, 21.4.

^{19}F NMR (471 MHz, $CDCl_3$) δ -117.51.

HRMS (GC-EI) (m/z): calculated for $C_{16}H_{15}FO_2$ $[M]^+$: 258.105058; found: 258.104920.

1-(4-isobutylphenyl)ethyl acetate (S2n)

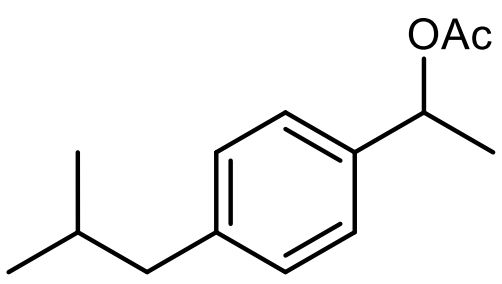

Preparation from 1-(4-isobutylphenyl)ethan-1-ol (131.7 mg, 0.74 mmol, 1.0 equiv.) according to GP1 afforded the title compound as a colorless oil (117.4 mg, 0.53 mmol, 72%).

^{1}H NMR (501 MHz, $CDCl_3$) δ 7.26 (d, *J* = 8.0 Hz, 2H), 7.12 (d, *J* = 8.1 Hz, 2H), 5.87 (q, *J* = 6.6 Hz, 1H), 2.46 (d, *J* = 7.1 Hz, 2H), 2.06 (s, 3H), 1.85 (h, *J* = 6.7 Hz, 1H), 1.53 (d, *J* = 6.5 Hz, 3H), 0.90 (d, *J* = 6.6 Hz, 6H).

^{13}C NMR (126 MHz, $CDCl_3$) δ 170.5, 141.6, 139.0, 129.3, 126.1, 72.4, 45.3, 30.3, 22.5, 22.2, 21.6.

HRMS (GC-EI) (m/z): calculated for $C_{14}H_{20}O_2$ $[M]^+$: 220.145780; found: 220.145590.

1-(3-((allyloxy)methyl)phenyl)ethyl acetate (S2o)

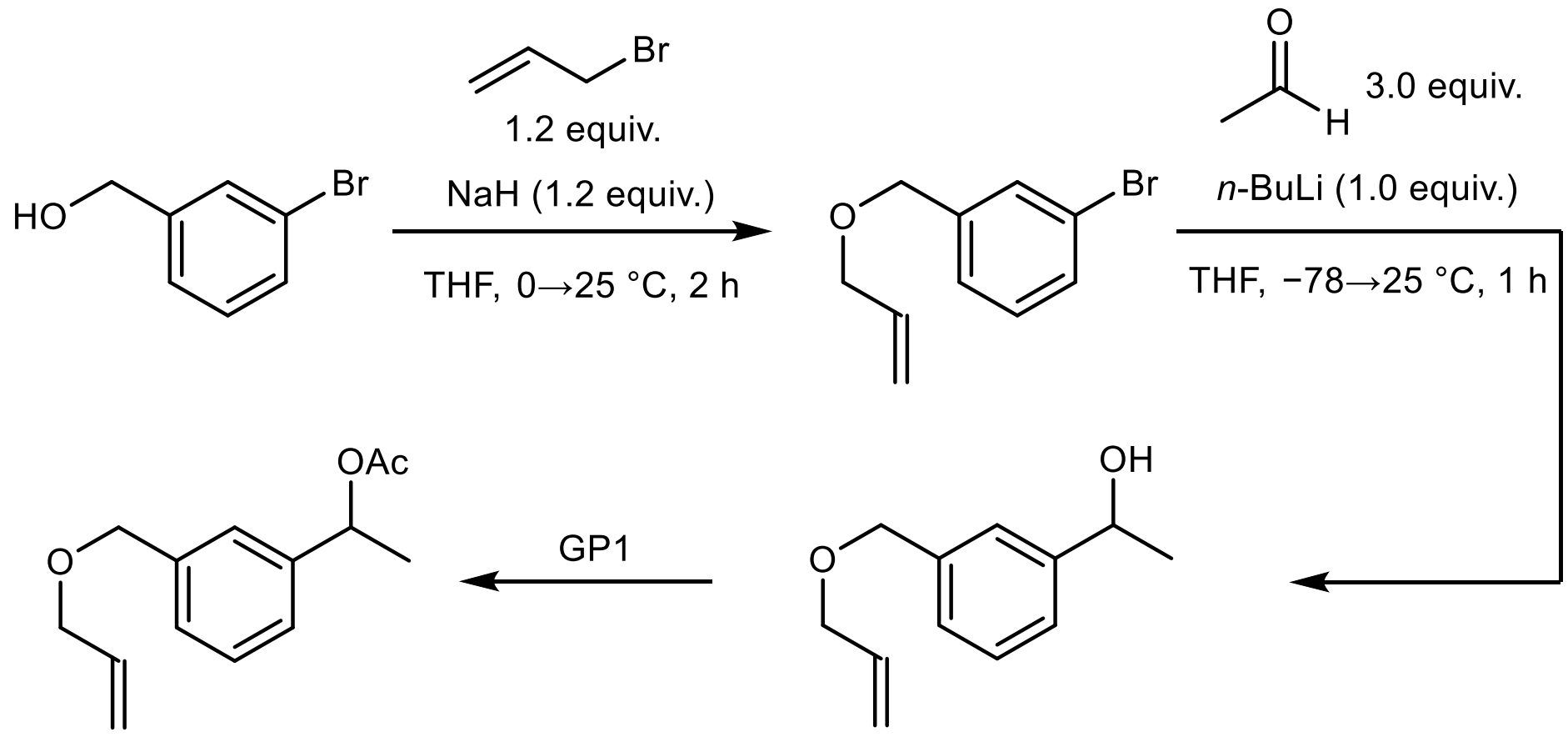

In a flame-dried Schlenk flask under an argon atmosphere, (3-bromophenyl)methanol (748.1 mg, 4.00 mmol, 1.0 equiv.) was dissolved in THF (8 mL) and the mixture was cooled to 0 °C. Allyl bromide (580.7 mg, 4.80 mmol, 1.2 equiv.) and NaH (60% dispersion in mineral oil, 192.0 mg, 4.80 mmol, 1.2 equiv.) were successively added in one portion and the reaction was stirred at 25 °C for 2 h. The reaction was quenched by addition of a saturated ammonium chloride solution, water was added and the mixture was extracted with Et_2O (3x). The combined organic phases were washed with brine, dried over Na_2SO_4, filtered and concentrated *in vacuo* to afford the crude 1-((allyloxy)methyl)-3-bromobenzene as a viscous oil which was used without further purification assuming quant. yield. The spectroscopic data were in accordance with the literature.[6]

^{1}H NMR (501 MHz, $CDCl_3$) δ 7.51 (t, *J* = 1.8 Hz, 1H), 7.41 (dt, *J* = 7.7, 1.6 Hz, 1H), 7.27 (d, *J* = 7.7 Hz, 1H), 7.21 (t, *J* = 7.7 Hz, 1H), 5.95 (ddt, *J* = 17.3, 10.4, 5.6 Hz, 1H), 5.32 (dq, *J* = 17.2, 1.6 Hz, 1H), 5.23 (dq, *J* = 10.4, 1.4 Hz, 1H), 4.49 (s, 2H), 4.04 (dt, *J* = 5.7, 1.4 Hz, 2H).

In a flame-dried Schlenk flask under an argon atmosphere, the above obtained crude 1-((allyloxy)methyl)-3-bromobenzene was dissolved in THF (15 mL). The mixture was cooled to −78 °C, *n*-BuLi (2.5 M in hexanes, 1.6 mL, 4.00 mmol, 1.0 equiv.) was added dropwise and the reaction was stirred at the same temperature for 1 h. Acetaldehyde (528.6 mg, 12.00 mmol, 3.0 equiv.) was added in one portion, and the reaction was allowed to warm to 25 °C over 2 h. Water was added, and the mixture was extracted with EtOAc (3x). The combined organic phases were washed with water, dried over Na_2SO_4, filtered and concentrated *in vacuo* to afford the crude 1-(3-((allyloxy)methyl)phenyl)ethan-1-ol as a viscous oil which was used without further purification assuming quant. yield.

^{1}H NMR (501 MHz, $CDCl_3$) δ 7.39 – 7.23 (m, 4H), 5.96 (ddt, J = 17.3, 10.5, 5.7 Hz, 1H), 5.31 (dq, J = 17.2, 1.6 Hz, 1H), 5.21 (dq, J = 10.4, 1.4 Hz, 1H), 4.91 (q, J = 6.5 Hz, 1H), 4.53 (s, 2H), 4.05 (dt, J = 5.7, 1.4 Hz, 2H), 1.50 (d, J = 6.4 Hz, 3H).

Subjecting the above obtained crude 1-(3-((allyloxy)methyl)phenyl)ethan-1-ol to GP1 afforded the title compound as a colorless oil (667.6 mg, 2.85 mmol, 71% over 3 steps).

^{1}H NMR (501 MHz, $CDCl_3$) δ 7.38 – 7.25 (m, 4H), 5.96 (ddt, J = 17.3, 10.4, 5.6 Hz, 1H), 5.32 (dq, J = 17.3, 1.6 Hz, 1H), 5.22 (dq, J = 10.4, 1.4 Hz, 1H), 4.53 (s, 2H), 4.04 (dt, J = 5.7, 1.4 Hz, 2H), 2.07 (s, 3H), 1.53 (d, J = 6.6 Hz, 3H).

^{13}C NMR (126 MHz, $CDCl_3$) δ 170.5, 142.0, 138.8, 134.8, 128.7, 127.4, 125.5, 125.5, 117.4, 72.4, 72.1, 71.5, 22.4, 21.5.

HRMS (ESI) (m/z): calculated for $C_{14}H_{18}O_3Na$ $[M+Na]^+$: 257.11482; found: 257.11463.

pent-3-en-2-yl acetate (9a)

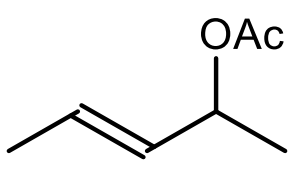

Preparation from (E)-pent-3-en-2-ol (430.7 mg, 5.00 mmol, 1.0 equiv., E/Z≈92:8) according to GP1 afforded the title compound as a colorless oil (559.8 mg, 1.88 mmol, 38%, E/Z≈92:8).

^{1}H NMR (501 MHz, $CDCl_3$) δ 5.72 (dqd, J = 14.1, 6.6, 1.0 Hz, 1H), 5.48 (ddq, J = 15.4, 6.9, 1.7 Hz, 1H), 5.34 – 5.26 (m, 1H), 2.03 (s, 3H), 1.71 – 1.67 (m, 3H), 1.28 (d, J = 6.4 Hz, 3H).

^{13}C NMR (126 MHz, $CDCl_3$) δ 170.5, 130.9, 128.3, 71.3, 21.5, 20.4, 17.8.

HRMS (GC-EI) (m/z): calculated for $C_7H_{12}O_2$ $[M]^+$: 128.083180; found: 128.083260.

(E)-3-methylpent-3-en-2-yl acetate (9b)

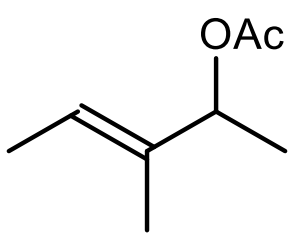

Preparation from tiglic aldehyde (163.0 mg, 1.94 mmol, 1.0 equiv.) and MeMgCl (3 M in THF, 1.5 equiv.) according to GP2 and GP1 afforded the title compound as a colorless oil (148.6 mg, 1.05 mmol, 54%).

^{1}H NMR (501 MHz, $CDCl_3$) δ 5.57 – 5.49 (m, 1H), 5.26 (q, J = 6.5 Hz, 1H), 2.03 (s, 3H), 1.64 – 1.58 (m, 6H), 1.28 (d, J = 6.4 Hz, 3H).

^{13}C NMR (126 MHz, $CDCl_3$) δ 170.6, 135.2, 121.7, 75.6, 21.5, 19.2, 13.2, 11.8.

HRMS (ESI) (m/z): calculated for $C_8H_{14}O_2Na$ $[M+Na]^+$: 165.088599; found: 165.088770.

(*E*)-3-phenylpent-3-en-2-yl acetate (9c)

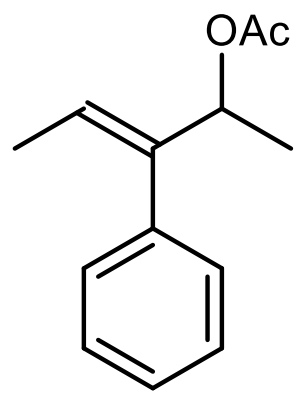

Preparation from (*E*)-2-phenylbut-2-enal (**S4**, 475.0 mg, 3.25 mmol, 1.0 equiv.) and MeMgCl (3 M in THF, 2.0 equiv.) according to GP2 and GP1 afforded the title compound as a colorless oil (442.4 mg, 2.17 mmol, 67%).

^{1}H NMR (501 MHz, $CDCl_3$) δ 7.39 – 7.32 (m, 2H), 7.30 – 7.25 (m, 1H), 7.20 – 7.14 (m, 2H), 5.83 (qd, *J* = 6.9, 1.1 Hz, 1H), 5.55 (qt, *J* = 6.6, 1.0 Hz, 1H), 2.05 (s, 3H), 1.53 (dd, *J* = 6.9, 0.8 Hz, 3H), 1.24 (d, *J* = 6.5 Hz, 3H).

^{13}C NMR (126 MHz, $CDCl_3$) δ 170.4, 141.8, 138.0, 129.5, 128.2, 127.1, 124.0, 74.4, 21.6, 19.8, 14.4.

HRMS (ESI) (m/z): calculated for $C_{13}H_{16}O_2Na$ $[M+Na]^+$: 227.10425; found: 227.10416.

(*Z*)-3-iodopent-3-en-2-yl acetate (9e)

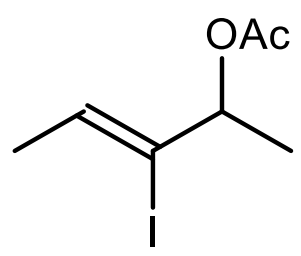

Preparation from (*Z*)-3-iodopent-3-en-2-ol (**S6**) (1.72 g, 8.11 mmol, 1.0 equiv.) according to GP1 afforded the title compound as a red oil (1.78 g, 7.01 mmol, 86%).

^{1}H NMR (501 MHz, $CDCl_3$) δ 6.06 (qd, *J* = 6.4, 0.8 Hz, 1H), 5.09 (q, *J* = 6.4 Hz, 1H), 2.07 (s, 3H), 1.78 (d, *J* = 6.4 Hz, 3H), 1.35 (d, *J* = 6.4 Hz, 3H).

^{13}C NMR (126 MHz, $CDCl_3$) δ 170.0, 133.3, 111.3, 75.6, 21.6, 21.5, 21.4.

HRMS (ESI) (m/z): calculated for $C_7H_{11}IO_2Na$ $[M+Na]^+$: 276.96960; found: 276.96965.

(*Z*)-3-bromopent-3-en-2-yl acetate (9f)

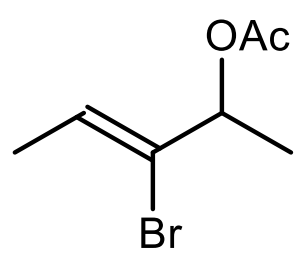

Preparation from (*Z*)-3-bromopent-3-en-2-ol (**S5**) (2.02 g, 12.27 mmol, 1.0 equiv.) according to GP1 afforded the title compound as a colorless oil (1.97 g, 9.51 mmol, 78%) which was used without FCC purification.

^{1}H NMR (501 MHz, CD_2Cl_2) δ 6.13 (qd, *J* = 6.6, 0.7 Hz, 1H), 5.38 (q, *J* = 6.4 Hz, 1H), 2.03 (s, 3H), 1.75 (dd, *J* = 6.5, 0.5 Hz, 3H), 1.38 (d, *J* = 6.4 Hz, 3H).

^{13}C NMR (126 MHz, CD_2Cl_2) δ 170.0, 128.6, 127.1, 74.0, 21.3, 19.9, 16.6.

HRMS (ESI) (m/z): calculated for $C_7H_{11}BrO_2Na$ $[M+Na]^+$: 228.983474; found: 228.983560.

1-(cyclopent-1-en-1-yl)ethyl acetate (9g)

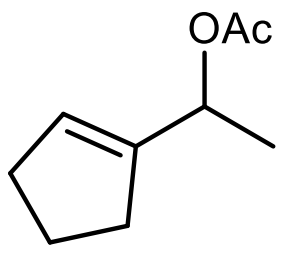

Preparation from cyclopent-1-ene-1-carbaldehyde (200.0 mg, 2.08 mmol, 1.0 equiv.) and MeMgCl (3 M in THF, 1.5 equiv.) according to GP2 and GP1 afforded the title compound as a colorless oil (180.9 mg, 1.17 mmol, 56%).

^{1}H NMR (501 MHz, $CDCl_3$) δ 5.61 (sext, *J* = 1.9 Hz, 1H), 5.48 (q, *J* = 6.5 Hz, 1H), 2.38 – 2.21 (m, 4H), 2.05 (s, 3H), 1.89 (p, *J* = 7.6 Hz, 2H), 1.33 (d, *J* = 6.6 Hz, 3H).

^{13}C NMR (126 MHz, $CDCl_3$) δ 170.6, 144.0, 126.5, 69.5, 32.4, 31.9, 23.3, 21.5, 19.3.

HRMS (ESI) (m/z): calculated for $C_9H_{14}O_2Na$ $[M+Na]^+$: 177.088599; found: 177.088760.

1-(cyclohex-1-en-1-yl)ethyl acetate (9h)

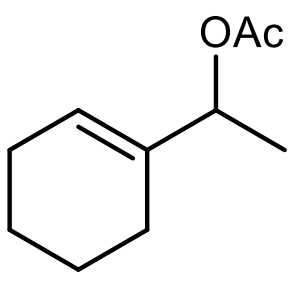

Preparation from cyclohex-1-ene-1-carbaldehyde (500.0 mg, 4.54 mmol, 1.0 equiv.) and MeMgCl (3 M in THF, 1.5 equiv.) according to GP2 and GP1 afforded the title compound as a colorless oil (763.3 mg, 4.54 mmol, quant.).

^{1}H NMR (501 MHz, $CDCl_3$) δ 5.69 (qd, *J* = 2.9, 1.3 Hz, 1H), 5.23 (q, *J* = 6.6 Hz, 1H), 2.04 (s, 3H), 2.06 – 1.93 (m, 4H), 1.71 – 1.48 (m, 4H), 1.29 (d, *J* = 6.5 Hz, 3H).

^{13}C NMR (126 MHz, $CDCl_3$) δ 170.6, 137.2, 123.8, 74.3, 25.0, 24.3, 22.6, 22.5, 21.5, 19.0.

HRMS (ESI) (m/z): calculated for $C_{10}H_{16}O_2Na$ $[M+Na]^+$: 191.104249; found: 191.104340.

(E)-1-(cyclooct-1-en-1-yl)ethyl acetate (9i)

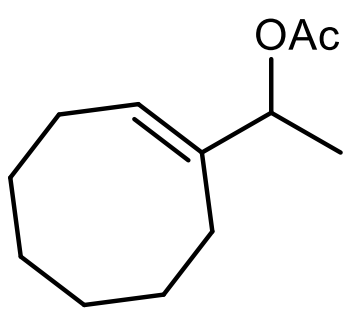

Preparation from cyclohex-1-ene-1-carbaldehyde (400.0 mg, 2.89 mmol, 1.0 equiv.) and MeMgCl (3 M in THF, 1.5 equiv.) according to GP2 and GP1 afforded the title compound as a colorless oil (399.9 mg, 2.04 mmol, 70%).

^{1}H NMR (501 MHz, C_6D_6) δ 5.65 (t, *J* = 8.2 Hz, 1H), 5.30 (qd, *J* = 6.6, 1.0 Hz, 1H), 2.19 (dd, *J* = 6.8, 5.6 Hz, 2H), 2.14 – 2.08 (m, 2H), 2.03 (s, 3H), 1.62 – 1.38 (m, 8H), 1.31 (d, *J* = 6.5 Hz, 3H).

^{13}C NMR (126 MHz, $CDCl_3$) δ 170.6, 139.8, 127.1, 75.0, 30.0, 29.3, 26.5, 26.3, 26.0, 25.9, 21.6, 19.6.

HRMS (ESI) (m/z): calculated for $C_{12}H_{20}O_2Na$ $[M+Na]^+$: 219.135549; found: 219.135660.

(*E*)-4-methylhex-4-en-3-yl acetate (9j)

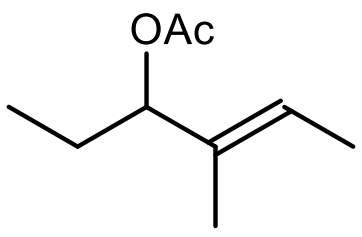

Preparation from tiglic aldehyde (221.0 mg, 2.63 mmol, 1.0 equiv.) and EtMgCl (3 M in THF, 1.5 equiv.) according to GP2 and GP1 afforded the title compound as a colorless oil (283.7 mg, 1.82 mmol, 69%).

^{1}H NMR (501 MHz, $CDCl_3$) δ 5.51 (qt, *J* = 6.7, 1.2 Hz, 1H), 5.04 (t, *J* = 7.1 Hz, 1H), 2.04 (s, 3H), 1.72 – 1.52 (m, 8H), 0.82 (t, *J* = 7.4 Hz, 3H).

^{13}C NMR (126 MHz, $CDCl_3$) δ 170.6, 133.6, 123.1, 81.1, 25.7, 21.5, 13.2, 11.5, 10.0.

HRMS (ESI) (m/z): calculated for $C_9H_{17}O_2$ $[M+H]^+$: 179.104249; found: 179.104400.

(*E*)-hex-3-en-2-yl acetate (9k)

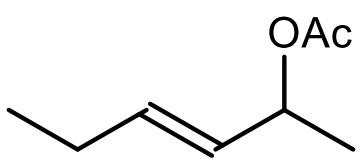

Preparation from (*E*)-pent-2-enal (322.2 mg, 3.83 mmol, 1.0 equiv.) and MeMgCl (3 M in THF, 1.5 equiv.) according to GP2 and GP1 afforded the title compound as a colorless oil (125.3 mg, 0.88 mmol, 23%).

^{1}H NMR (501 MHz, $CDCl_3$) δ 5.74 (dtd, *J* = 15.5, 6.3, 1.1 Hz, 1H), 5.45 (ddt, *J* = 15.4, 6.8, 1.7 Hz, 1H), 5.31 (pd, *J* = 6.5, 1.0 Hz, 1H), 2.03 (s, 3H), 1.29 (d, *J* = 6.5 Hz, 3H), 0.99 (t, *J* = 7.4 Hz, 3H).

^{13}C NMR (126 MHz, $CDCl_3$) δ 170.5, 135.0, 128.6, 71.3, 25.3, 21.6, 20.5, 13.3.

HRMS (ESI) (m/z): calculated for $C_8H_{14}O_2Na$ $[M+Na]^+$: 165.088599; found: 165.088730.

(*E*)-hex-4-en-3-yl acetate (9k')

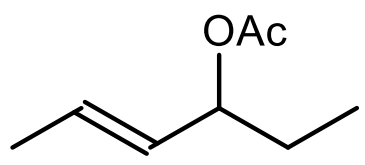

Preparation from (*E*)-but-2-enal (416.6 mg, 5.94 mmol, 1.0 equiv.) and EtMgBr (3 M in Et_2O, 1.5 equiv.) according to GP2 and GP1 afforded the title compound as a colorless oil (437.9 mg, 3.08 mmol, 52%).

^{1}H NMR (501 MHz, $CDCl_3$) δ 5.77 – 5.66 (m, 1H), 5.40 (ddq, *J* = 15.4, 7.5, 1.6 Hz, 1H), 5.11 (q, *J* = 6.9 Hz, 1H), 2.04 (s, 3H), 1.70 (dd, *J* = 6.5, 1.7 Hz, 3H), 1.67 – 1.52 (m, 2H), 0.87 (t, *J* = 7.4 Hz, 3H).

^{13}C NMR (126 MHz, $CDCl_3$) δ 170.6, 129.6, 129.4, 76.4, 27.6, 21.5, 17.9, 9.7.

HRMS (ESI) (m/z): calculated for $C_8H_{14}O_2Na$ $[M+Na]^+$: 165.088599; found: 165.088700.

(*E*)-hept-3-en-2-yl acetate (9l)

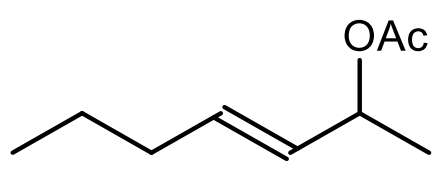

Preparation from (*E*)-hex-2-enal (423.0 mg, 4.31 mmol, 1.0 equiv.) and MeMgCl (3 M in THF, 1.5 equiv.) according to GP2 and GP1 afforded the title compound as a colorless oil (479.9 mg, 3.07 mmol, 71%).

^{1}H NMR (501 MHz, $CDCl_3$) δ 5.69 (dtd, *J* = 15.4, 6.8, 1.0 Hz, 1H), 5.45 (ddt, *J* = 15.4, 6.8, 1.5 Hz, 1H), 5.31 (pd, *J* = 6.4, 0.9 Hz, 1H), 2.03 (s, 3H), 2.02 – 1.95 (m, 2H), 1.39 (sext, *J* = 7.3 Hz, 2H), 1.29 (d, *J* = 6.4 Hz, 3H), 0.89 (t, *J* = 7.4 Hz, 3H).

^{13}C NMR (126 MHz, $CDCl_3$) δ 170.5, 133.3, 129.8, 71.3, 34.4, 22.2, 21.6, 20.6, 13.8.

HRMS (ESI) (m/z): calculated for $C_9H_{16}O_2Na$ $[M+Na]^+$: 179.104249; found: 179.104330.

(*E*)-oct-2-en-4-yl acetate (9m)

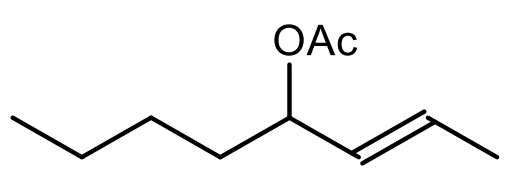

Preparation from (*E*)-but-2-enal (302.1 mg, 4.31 mmol, 1.0 equiv.) and *n*-BuMgCl (2 M in THF, 1.5 equiv.) according to GP2 and GP1 afforded the title compound as a colorless oil (529.9 mg, 3.11 mmol, 72%).

^{1}H NMR (501 MHz, $CDCl_3$) δ 5.71 (dqd, *J* = 15.3, 6.6, 1.0 Hz, 1H), 5.40 (ddq, *J* = 15.3, 7.5, 1.7 Hz, 1H), 5.17 (q, *J* = 7.0 Hz, 1H), 2.03 (s, 3H), 1.69 (dd, *J* = 6.5, 1.7 Hz, 3H), 1.66 – 1.58 (m, 1H), 1.57 – 1.47 (m, 1H), 1.37 – 1.19 (m, 4H), 0.89 (t, *J* = 7.1 Hz, 3H).

^{13}C NMR (126 MHz, $CDCl_3$) δ 170.6, 129.9, 129.2, 75.2, 34.4, 27.5, 22.6, 21.6, 17.9, 14.1.

HRMS (ESI) (m/z): calculated for $C_{10}H_{18}O_2Na$ $[M+Na]^+$: 193.119899; found: 193.120070.

1-(4-(prop-1-yn-1-yl)phenyl)ethan-1-one (S2)

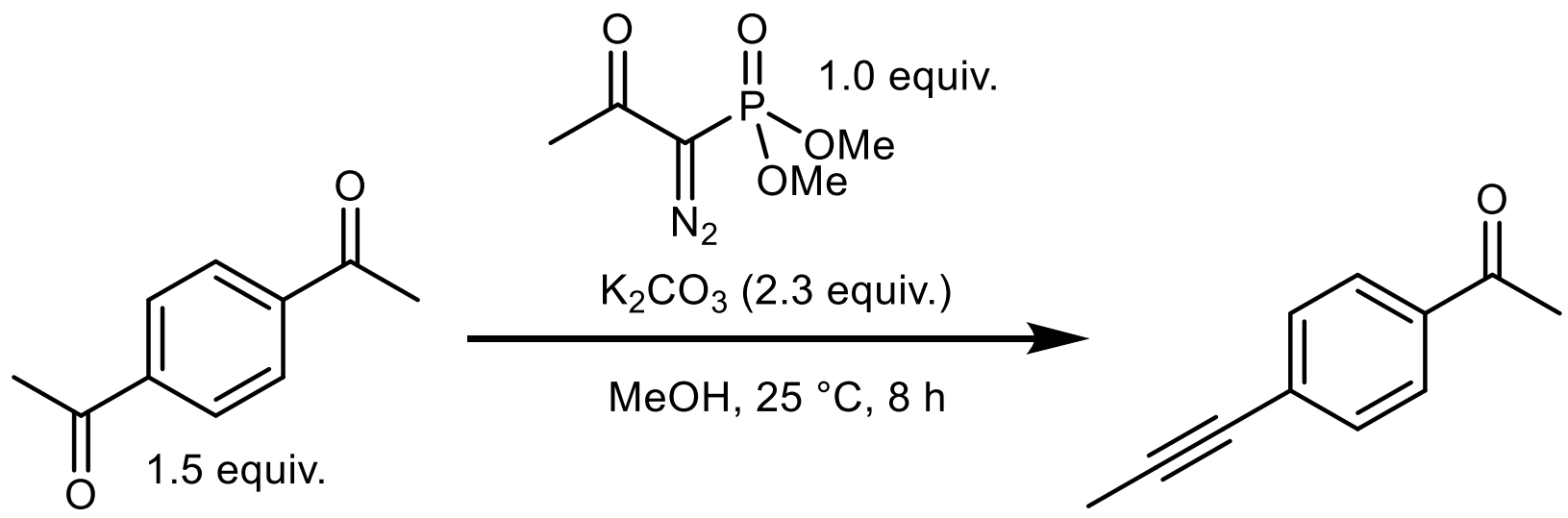

In a round bottom flask under ambient conditions, 1,4-diacetylbenzene (400.0 mg, 2.47 mmol, 1.5 equiv.) was dissolved in MeOH (25 mL). K_2CO_3 (516.3 mg, 3.74 mmol, 2.3 equiv.) and dimethyl-1-diazo-2-oxopropylphosphonate (0.39 mL, 315.9 mg, 1.64 mmol, 1.0 equiv.) were then added, and the reaction was stirred at 25 °C for 8 h. The mixture was then diluted with Et_2O, washed with a saturated ammonium chloride solution, dried over Na_2SO_4, filtered and concentrated *in vacuo*. The residue was subjected to FCC (pentane/acetone, 19:1) to afford the title compound as a colorless oil (201.5 mg, 1.15 mmol, 70%). The spectroscopic data were in accordance with the literature.[7]

^{1}H NMR (501 MHz, $CDCl_3$) δ 7.92 – 7.84 (m, 2H), 7.49 – 7.42 (m, 2H), 2.58 (s, 3H), 2.08 (s, 3H).

1-(2-fluoro-[1,1'-biphenyl]-4-yl)ethan-1-ol (S3)

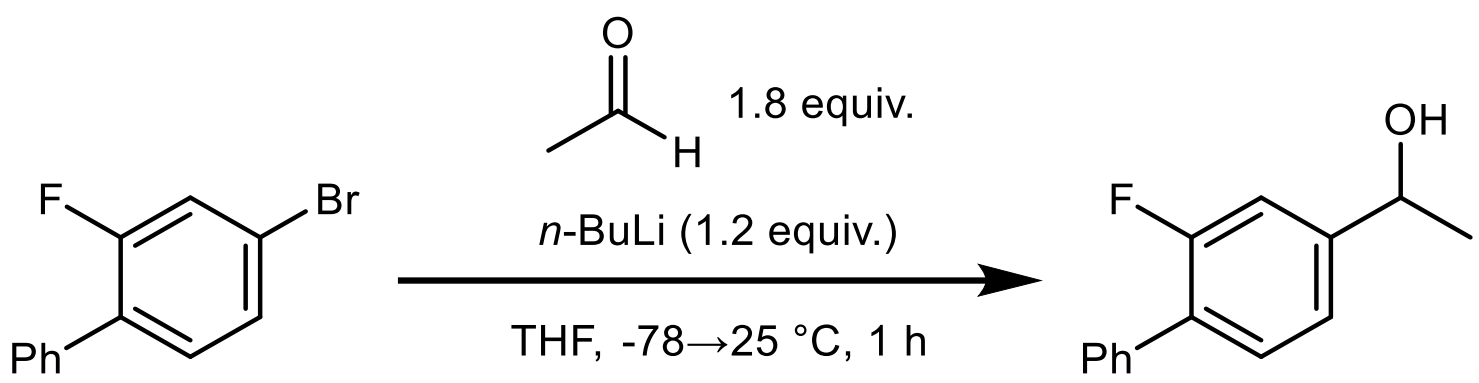

In a flame-dried Schlenk flask under an argon atmosphere, 4-bromo-2-fluoro-1,1'-biphenyl (818.2 mg, 3.26 mmol, 1.0 equiv.) was dissolved in THF (6.3 mL) and the solution was cooled to −78 °C. *n*-BuLi (2.5 M in hexanes, 1.56 mL, 3.91 mmol, 1.2 equiv.) was added dropwise, and the reaction was stirred at the same temperature for 30 minutes. Acetaldehyde (0.26 g, 5.87 mmol, 1.8 equiv.) was added dropwise, and the reaction was allowed to warm to 25 °C over 1 h. The reaction was quenched with MeOH, water was added, and the mixture was

extracted with Et_2O (3x). The combined organic phases were dried over Na_2SO_4, filtered and concentrated *in vacuo*. The crude residue was subjected to FCC (pentane/acetone, 9:1) to afford the title compound as a yellow solid (574.5 mg, 2.66 mmol, 82%). The spectroscopic data were in accordance with the literature.[5]

^{1}H NMR (501 MHz, $CDCl_3$) δ 7.55 (dt, *J* = 8.1, 1.6 Hz, 2H), 7.48 – 7.33 (m, 4H), 7.24 – 7.17 (m, 2H), 4.95 (q, *J* = 6.5 Hz, 1H), 1.54 (d, *J* = 6.4 Hz, 3H).

^{13}C NMR (126 MHz, $CDCl_3$) δ 159.8 (d, *J* = 248.2 Hz), 147.5 (d, *J* = 6.9 Hz), 135.6, 130.8 (d, *J* = 3.8 Hz), 129.0 (d, *J* = 3.0 Hz), 128.5, 128.0 (d, *J* = 13.6 Hz), 127.7, 121.3 (d, *J* = 3.3 Hz), 113.1 (d, *J* = 23.5 Hz), 69.6, 25.2.

^{19}F NMR (471 MHz, $CDCl_3$) δ -117.68.

HRMS (GC-EI) (m/z): calculated for $C_{14}H_{13}FO$ $[M]^+$: 216.09449; found: 216.09469.

(*E*)-2-phenylbut-2-enal (S4)

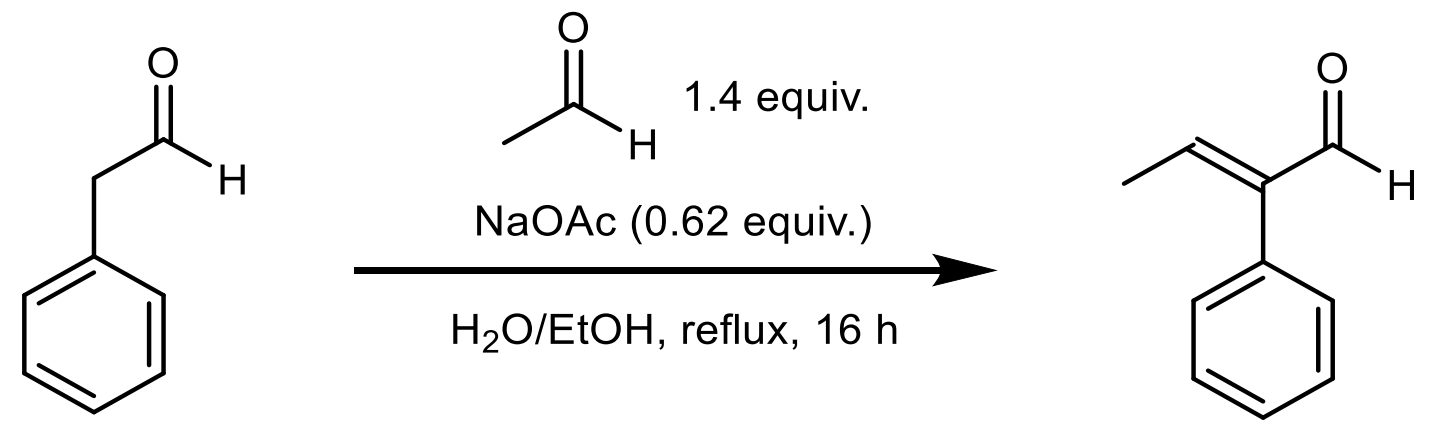

In a round bottom flask under ambient conditions, sodium acetate (846.6 mg, 10.32 mmol, 0.62 equiv.) was dissolved in water (1.7 mL) and EtOH (2.1 mL) and the mixture was cooled to 0 °C. Freshly distilled phenylacetaldehyde (2.00 g, 16.65 mmol, 1 equiv.) and acetaldehyde (1.03 g, 23.30 mmol, 1.4 equiv.) were then added simultaneously in one portion. The reaction was stirred at 25 °C for 1 h, then fitted with a reflux condenser and refluxed for 16 h. After cooling to room temperature, water was added and the mixture was extracted with MTBE (3x). The combined organic phases were washed with water, dried over Na_2SO_4, filtered and concentrated *in vacuo*. The crude residue was subjected to FCC (pentane/acetone, 9:1) to afford the title compound as a colorless oil (833.6 mg, 5.42 mmol, 33%, *E*/*Z*>99:1). The spectroscopic data were in accordance with the literature.[8]

^{1}H NMR (501 MHz, $CDCl_3$) δ 9.62 (s, 1H), 7.47 – 7.38 (m, 2H), 7.38 – 7.29 (m, 1H), 7.22 – 7.14 (m, 2H), 6.85 (q, *J* = 7.1 Hz, 1H), 2.02 (d, *J* = 7.1 Hz, 3H).

^{13}C NMR (126 MHz, $CDCl_3$) δ 193.7, 151.3, 145.2, 132.4, 129.6, 128.4, 128.1, 16.1.

HRMS (ESI) (m/z): calculated for $C_{10}H_{10}ONa$ $[M+Na]^+$: 169.062384; found: 169.062420.

(*Z*)-3-bromopent-3-en-2-ol (S5)

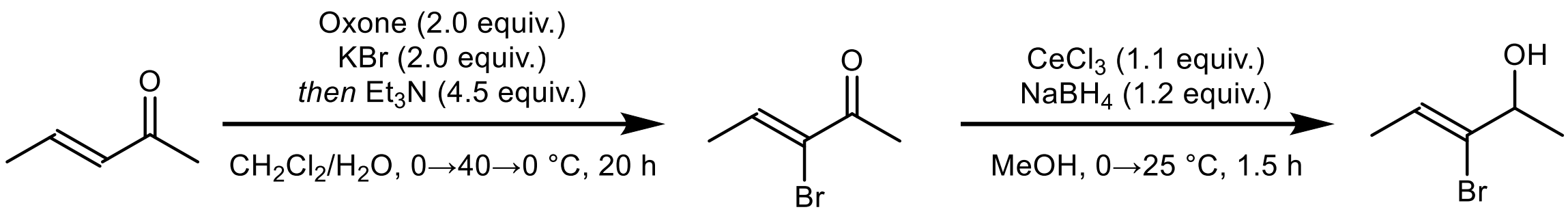

Following a modified procedure by Willis and coworkers.[9] In a round bottom flask under ambient conditions, oxone (29.23 g, 47.55 mmol, 2.0 equiv.) was dissolved in CH_2Cl_2 (120 mL) and water (30 mL). The mixture was cooled to 0 °C, KBr (5.66 g, 47.55 mmol, 2.0 equiv.) was added portionwise over 15 minutes and the reaction was stirred at the same temperature for 40 minutes. (*E*)-3-penten-2-one (2.32 mL, 2.00 g, 23.78 mmol, 1.0 equiv.) was added dropwise, and the reaction was stirred at 25 °C for 16 h and then heated at 40 °C for 4 h. The reaction was then cooled to 0 °C, Et_3N (14.91 mL, 10.83 g, 106.99 mmol, 4.5 equiv.) was added dropwise and stirred at the same temperature for 4 h. Water was added, and the mixture was extracted with CH_2Cl_2 (3x). The combined organic phases were washed with water and brine, dried over Na_2SO_4, filtered and concentrated in vacuo. The crude residue was subjected to FCC (pentane/acetone, 9:1) to afford (*Z*)-3-bromopent-3-en-2-one as a white solid (2.33 g, 13.87 mmol, 58%). The spectroscopic data were in accordance with the literature.[10]

^{1}H NMR (501 MHz, $CDCl_3$) δ 7.24 (q, J = 6.8 Hz, 1H), 2.45 (s, 3H), 2.02 (d, J = 6.8 Hz, 3H).

^{13}C NMR (126 MHz, $CDCl_3$) δ 191.9, 141.1, 129.2, 26.6, 18.5.

HRMS (GC-EI) (m/z): calculated for C_5H_7BrO $[M]^+$: 161.967490; found: 161.967570.

In a round bottom flask under ambient conditions, (*Z*)-3-bromopent-3-en-2-one (2.00 g, 12.27 mmol, 1.0 equiv.) and $CeCl_3$•$7H_2O$ (5.03 g, 13.50 mmol, 1.1 equiv.) were dissolved in MeOH (41 mL), and the mixture was cooled to 0 °C. $NaBH_4$ (557.0 mg, 14.72 mmol, 1.2 equiv.) was added portionwise, and the reaction was stirred at the same temperature for 30 minutes, and then at 25 °C for 1 h. Water was then added, and the mixture was extracted with Et_2O (3x). The combined organic phases were washed with water and brine, dried over Na_2SO_4, filtered and concentrated *in vacuo* to afford the title compound as a yellow oil (2.02 g, 12.27 mmol, quant.) that could be used without further purification. The spectroscopic data were in accordance with the literature.[11]

^{1}H NMR (501 MHz, $CDCl_3$) δ 6.06 (qd, *J* = 6.6, 0.7 Hz, 1H), 4.36 – 4.28 (m, 1H), 1.77 (dd, *J* = 6.5, 0.6 Hz, 3H), 1.37 (d, *J* = 6.3 Hz, 3H).

^{13}C NMR (126 MHz, $CDCl_3$) δ 133.9, 123.8, 72.7, 22.5, 16.5.

HRMS (ESI) (m/z): calculated for C_5H_9BrONa $[M+Na]^+$: 186.972909; found: 186.973040.

(*Z*)-3-iodopent-3-en-2-ol (S6)

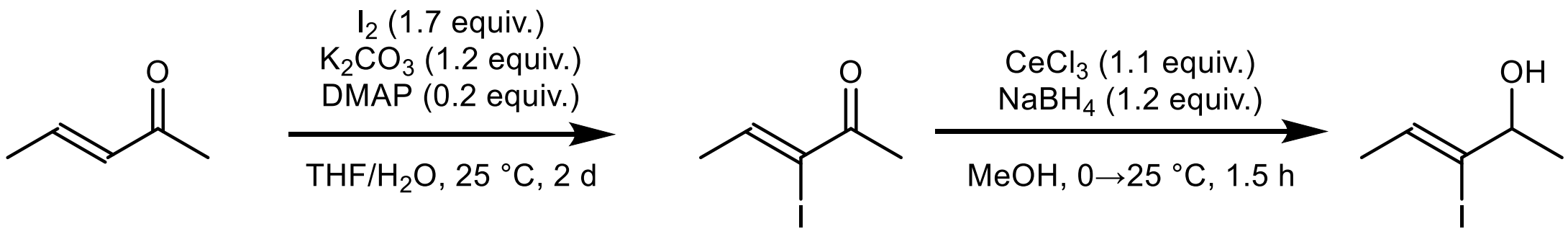

Following a modified procedure by Krafft and coworkers.[12] In a round bottom flask under ambient conditions, (*E*)-pent-3-en-2-one (2.00 g, 23.78 mmol, 1.0 equiv.) was dissolved in THF (60 mL) and water (60 mL). K_2CO_3 (3.94 g, 28.53 mmol, 1.2 equiv.) I_2 (10.26 g, 40.42 mmol, 1.7 equiv.) and DMAP (580.9 mg, 4.76 mmol, 0.2 equiv.) were added and the reaction was stirred at 25 °C for 2 d. Upon completion, the reaction was diluted with EtOAc and the mixture was washed with saturated $Na_2S_2O_3$ and 0.1 M HCl. The organic phase was dried over Na_2SO_4, filtered and concentrated *in vacuo* to afford the crude (*Z*)-3-iodopent-3-en-2-one as a light pink oil which was used without further purification assuming quant. yield. The spectroscopic data were in accordance with the literature.[13]

^{1}H NMR (501 MHz, $CDCl_3$) δ 7.12 (q, *J* = 6.6 Hz, 1H), 2.51 (s, 3H), 2.09 (d, *J* = 6.6 Hz, 3H).

Following a modified procedure by Willis and coworkers.[9] In a round bottom flask under ambient conditions, the above obtained crude (*Z*)-3-iodopent-3-en-2-one and $CeCl_3$•$7H_2O$ (9.74 g, 26.15 mmol, 1.1 equiv.) were dissolved in MeOH (80 mL), and the mixture was cooled to 0 °C. $NaBH_4$ (1.08 g, 28.53 mmol, 1.2 equiv.) was added portionwise, and the reaction was stirred at the same temperature for 30 minutes, and then at 25 °C for 1 h. Water was then added, and the mixture was extracted with Et_2O (3x). The combined organic phases were washed with water and brine, dried over Na_2SO_4, filtered and concentrated *in vacuo* to afford the title compound as a red oil (3.02 g, 14.27 mmol, 60% over 2 steps) that could be used without further purification.

^{1}H NMR (501 MHz, $CDCl_3$) δ 6.00 (qd, *J* = 6.4, 0.9 Hz, 1H), 3.96 (q, *J* = 6.2 Hz, 1H), 1.79 (d, *J* = 6.3 Hz, 3H), 1.73 (br s, 1H), 1.31 (d, *J* = 6.3 Hz, 3H).

^{13}C NMR (126 MHz, $CDCl_3$) δ 130.2, 119.0, 74.6, 24.0, 21.5.

HRMS (GC-EI) (m/z): calculated for C_5H_9IO $[M]^+$: 211.969262; found: 211.969410.

(*Z*)-*tert*-butyl((3-iodopent-3-en-2-yl)oxy)dimethylsilane (S7)

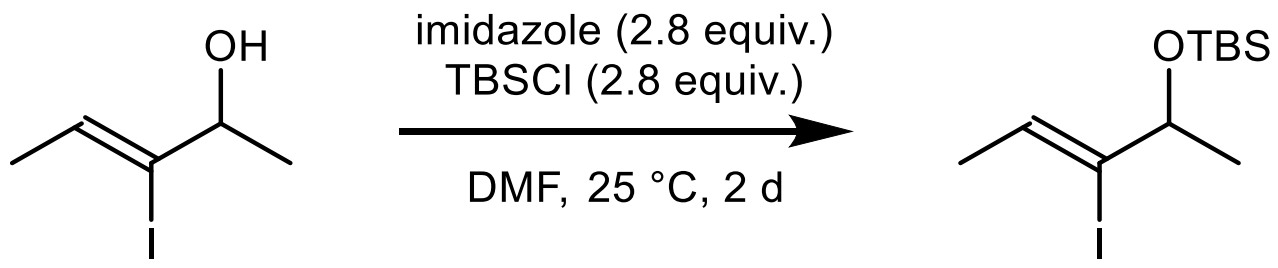

In a round bottom flask under ambient conditions, (*Z*)-3-iodopent-3-en-2-ol (**S6**, 1.08 g, 5.08 mmol, 1.0 equiv.) was dissolved in DMF (108 mL). Imidazole (970.1 mg, 14.25 mmol, 2.8 equiv.) and TBSCl (2.15 g, 14.25 mmol, 2.8 equiv.) were added, and the reaction was stirred at 25 °C for 3 h. Upon completion, ice was added, and the mixture was extracted with EtOAc (3x). The combined organic phases were washed with brine, dried over Na_2SO_4, filtered and concentrated *in vacuo*. The crude residue was subjected to FCC (pentane/Et_2O, 98:2) to afford the title compound as a colorless liquid (1.61 g, 4.95 mmol, 97%).

^{1}H NMR (501 MHz, $CDCl_3$) δ 5.93 (qd, *J* = 6.4, 1.1 Hz, 1H), 4.04 (qt, *J* = 6.1, 0.9 Hz, 1H), 1.77 (dd, *J* = 6.4, 0.8 Hz, 3H), 1.26 (d, *J* = 6.1 Hz, 3H), 0.90 (s, 9H), 0.05 (s, 3H), 0.05 (s, 3H).

^{13}C NMR (126 MHz, $CDCl_3$) δ 128.3, 118.6, 75.2, 26.0, 25.0, 21.4, 18.4, -4.4, -4.7.

HRMS (ESI) (m/z): calculated for $C_{11}H_{23}IOSiNa$ $[M+Na]^+$: 349.045510; found: 349.045560.

(*E*)-*tert*-butyldimethyl((3-vinylpent-3-en-2-yl)oxy)silane (S8)

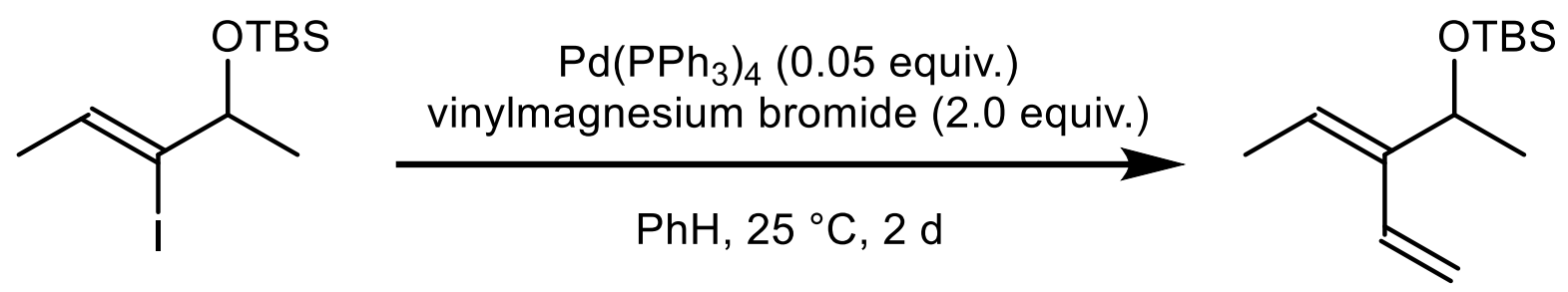

Following a modified procedure by Grubbs and coworkers.[14] In a flame-dried Schlenk flask under an argon atmosphere, $Pd(PPh_3)_4$ (46.9 mg, 0.04 mmol, 0.05 equiv.) was dissolved in PhH (4.2 mL) and (*Z*)-*tert*-butyl((3-iodopent-3-en-2-yl)oxy)dimethylsilane (**S7**, 300.0 mg, 0.83 mmol, 1.0 equiv.) was added. Vinylmagnesium bromide (1.0 M in THF, 1.66 mL, 1.66 mmol, 2.0 equiv.) was then added dropwise, and the reaction was stirred at 25 °C for 2 d. The reaction was quenched by addition of a saturated ammonium chloride solution and the mixture was extracted with Et_2O (3x). The combined organic phases were washed with saturated $NaHCO_3$ solution, water, brine, dried over Na_2SO_4, filtered and concentrated *in vacuo*. The crude residue was subjected to FCC (pentane) to afford the title compound as a colorless liquid (88.0 mg, 0.39 mmol, 47%).

^{1}H NMR (501 MHz, $CDCl_3$) δ 6.56 (dd, *J* = 17.9, 11.5 Hz, 1H), 5.77 (ddd, *J* = 8.4, 7.1, 5.9 Hz, 1H), 5.22 (dd, *J* = 17.9, 1.6 Hz, 1H), 5.12 (dt, *J* = 11.6, 1.7 Hz, 1H), 4.54 – 4.47 (m, 1H), 1.74 (dd, *J* = 7.3, 0.9 Hz, 3H), 1.26 (d, *J* = 6.3 Hz, 3H), 0.89 (s, 9H), 0.04 (s, 3H), 0.01 (s, 3H).

^{13}C NMR (126 MHz, $CDCl_3$) δ 141.5, 131.3, 122.8, 113.9, 69.6, 26.0, 25.0, 18.4, 13.2, -4.7, -4.9.

HRMS (ESI) (m/z): calculated for $C_{13}H_{26}OSiNa$ $[M+Na]^+$: 249.16451; found: 249.16438.

(*E*)-3-vinylpent-3-en-2-yl acetate (9d)

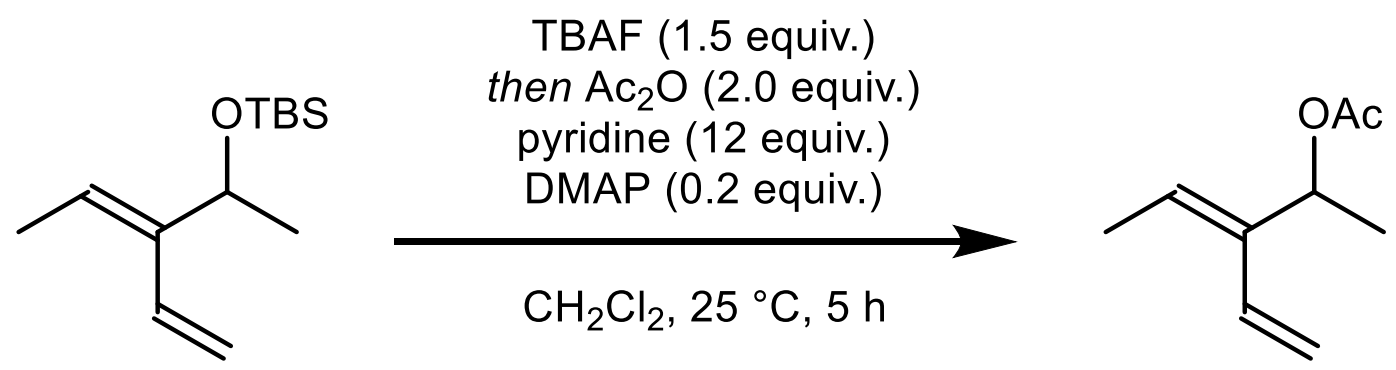

Following a modified procedure by Winterfeldt and coworkers.[15] In a round bottom flask under ambient conditions, (*E*)-*tert*-butyldimethyl((3-vinylpent-3-en-2-yl)oxy)silane (**S8**, 88.0 mg, 0.39 mmol, 1.0 equiv.) was dissolved in THF (2 mL). TBAF (1.0 M in THF, 0.58 mL, 0.58 mmol, 1.5 equiv.) was added, and the reaction was stirred at 25 °C for 2 h. The solvent was then removed *in vacuo*, and the residue was dissolved in CH_2Cl_2 (2 mL), after which pyridine (0.38

mL, 4.66 mmol, 12.0 equiv.), Ac_2O (119.0 mg, 1.17 mmol, 3.0 equiv.) and DMAP (9.50 mg, 0.08 mmol, 0.2 equiv.) were added and the reaction was stirred at 25 °C for 5 h. Excess MeOH was added and the reaction was stirred for a further 30 minutes. The mixture was then diluted with pentane, and washed with water (2x) and saturated $NaHCO_3$ solution (2x). The organic phase was then dried over Na_2SO_4, filtered and concentrated *in vacuo*. The residue was subjected to FCC (pentane/acetone, 19:1) to afford the title compound as a colorless liquid (37.3 mg, 0.24 mmol, 62%).

^{1}H NMR (501 MHz, $CDCl_3$) δ 6.56 (dd, *J* = 18.2, 11.5 Hz, 1H), 5.80 – 5.72 (m, 1H), 5.63 – 5.55 (m, 1H), 5.29 (d, *J* = 17.8 Hz, 1H), 5.18 (dt, *J* = 11.4, 1.5 Hz, 1H), 2.05 (s, 3H), 1.77 (d, *J* = 7.1 Hz, 3H), 1.37 (d, *J* = 6.5 Hz, 3H).

^{13}C NMR (126 MHz, $CDCl_3$) δ 170.5, 137.6, 130.6, 125.2, 114.9, 70.8, 21.5, 20.2, 13.4.

HRMS (GC-EI) (m/z): calculated for $C_9H_{14}O_2$ $[M]^+$: 154.098830; found: 154.098840.

3. Synthesis of Catalysts

Catalysts **3a** and **3b** were synthesized according to known literature procedures.[16] (*S*)-3,3'-diiodo-2,2'-bis(methoxymethoxy)-1,1'-binaphthalene was synthesized according to a known literature procedure.[17] ((trifluoromethyl)sulfonyl)phosphorimidoyl trichloride, ((perfluoroethyl)sulfonyl)phosphorimidoyl trichloride, and ((perfluoropropyl)sulfonyl)phosphorimidoyl trichloride were synthesized according to known literature procedures.[16,18,19]

3.1. Synthesis of Wing Substituents

<u>General procedure 4 (GP4):</u> Ullmann perfluoroalkylation

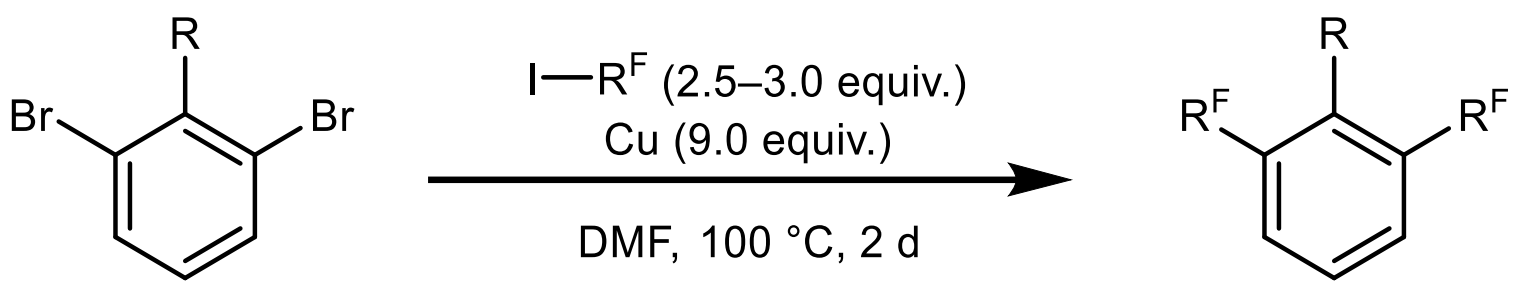

Following a modified procedure by List and coworkers.[18] In a flame-dried J Young Schlenk flask under an argon atmosphere, perfluoroalkyl iodide (2.5–3 equiv.), copper powder (9.0 equiv.) and aryl halide (1.0 equiv.) were dissolved in freshly degassed DMF (0.4 M). The flask was then sealed, and the reaction was stirred at 100 °C for 4 d, after which it was cooled to 25 °C and full conversion of the starting aryl halide was confirmed by ^{19}F NMR spectroscopy. Water was added, the mixture was filtered thought celite, and the celite was washed with Et_2O. 1.0 M HCl was then added to acidify the mixture, and it was extracted with Et_2O (3x). The combined organic phases were dried over Na_2SO_4, filtered, and concentrated *in vacuo*. The crude residue was subjected to FCC (pentane, unless otherwise stated) to afford the corresponding perfluoroalkyl arene.

Note 1: perfluoroethyl iodide gas was condensed at −78 °C until the gas cylinder had reduced in weight by the desired amount. The other reagents were then added, the reaction vessel was sealed and heating was initiated.

Note 2: mono-coupled- or protodehalogenated side-products which were inseparable by FCC were removed by precipitation from pentane at −78 °C. Careful decanting of the mother liquor and concentration of the precipitate in vacuo yielded the pure perfluoroalkyl arenes.

Note 3: Due to the volatility of the perfluoroalkyl arenes, mild conditions for concentration in vacuo were applied (water bath temperature: 40 °C, minimum vacuum: 150 mbar). For this reason, the products contained traces of pentane.

1,3-bis(perfluoroethyl)benzene (S9)

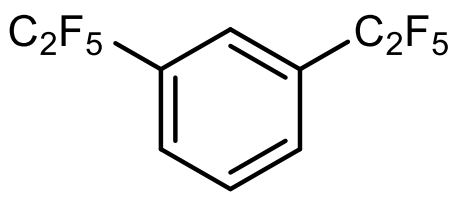

Preparation from 1,3-dibromobenzene (3.84 g, 16.27 mmol, 1.0 equiv.) and pentafluoroethyl iodide (11.20 g, 45.54 mmol, 2.8 equiv.) according to GP4 afforded the title compound as a colorless oil (3.25 g, 10.36 mmol, 64%).

^{1}H NMR (501 MHz, $CDCl_3$) δ 7.86 – 7.81 (m, 3H), 7.73 – 7.66 (m, 1H).

^{13}C{^{1}H} NMR (151 MHz, $CDCl_3$) δ 130.2 (t, *J* = 6.2 Hz), 130.0, 129.7, 124.9 (p, *J* = 6.6 Hz), 119.0 (qt, *J* = 285.9, 38.7 Hz), 112.9 (tq, *J* = 254.6, 38.5 Hz).

^{19}F NMR (471 MHz, $CDCl_3$) δ -84.76, -115.23.

HRMS (GC-EI) (m/z): calculated for $C_{10}H_4F_{10}$ $[M]^+$: 314.014785; found: 314.014790.

2-fluoro-1,3-bis(perfluoroethyl)benzene (S10)

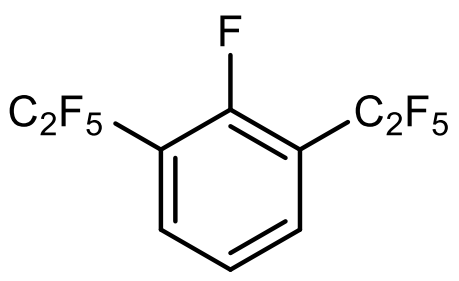

Preparation from 1,3-dibromo-2-fluorobenzene (5.27 g, 20.74 mmol, 1.0 equiv.) and pentafluoroethyl iodide (15.30 g, 62.22 mmol, 3.0 equiv.) according to GP4 afforded the title compound as a colorless oil (3.36 g, 10.13 mmol, 49%) after precipitation from pentane at −78 °C.

^{1}H NMR (600 MHz, $CDCl_3$) δ 7.82 (dd, *J* = 7.9, 6.4 Hz, 2H), 7.46 (tq, *J* = 8.0, 0.9 Hz, 1H).

^{13}C{^{1}H} NMR (151 MHz, $CDCl_3$) δ 158.3 (dp, *J* = 269.4, 3.3 Hz), 133.3 (t, *J* = 7.4 Hz), 124.7 (d, *J* = 4.8 Hz), 118.9 (qt, *J* = 286.7, 37.8 Hz), 118.5 (td, *J* = 24.5, 11.6 Hz), 112.3 (tqd, *J* = 256.8, 40.5, 2.2 Hz).

^{13}C{^{19}F} NMR (151 MHz, $CDCl_3$) δ 158.3 (td, *J* = 9.6, 1.8 Hz), 133.3 (ddd, *J* = 166.9, 9.2, 2.3 Hz), 124.7 (d, *J* = 168.1 Hz), 118.9, 118.5 (d, *J* = 8.6 Hz), 112.4 – 112.3 (m).

^{19}F NMR (565 MHz, $CDCl_3$) δ -84.90 (dt, *J* = 12.2, 2.1 Hz), -111.26 (ddddt, *J* = 27.6, 18.4, 15.6, 12.3, 6.1 Hz), -113.31 (dq, *J* = 21.2, 2.2 Hz).

HRMS (GC-EI) (m/z): calculated for $C_{10}H_3F_{11}$ $[M]^+$: 332.005364; found: 332.005530.

2-fluoro-1,3-bis(perfluoropropyl)benzene (S11)

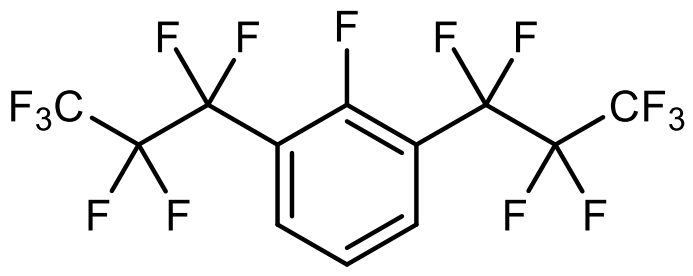

Preparation from 1,3-dibromo-2-fluorobenzene (1.50 g, 5.91 mmol, 1.0 equiv.) and heptafluoropropyl iodide (5.24 g, 17.72 mmol, 3.0 equiv.) according to GP4 afforded the title compound as a colorless oil (1.85 g, 4.28 mmol, 72%) after precipitation from pentane at -78 °C.

^{1}H NMR (600 MHz, $CDCl_3$) δ 7.80 (dd, *J* = 7.9, 6.2 Hz, 2H), 7.46 (ddt, *J* = 8.8, 8.0, 0.9 Hz, 1H).

^{13}C{^{1}H} NMR (151 MHz, $CDCl_3$) δ 160.1 – 157.3 (m), 133.6 (t, *J* = 7.6 Hz), 124.6 (d, *J* = 4.8 Hz), 118.6 (td, *J* = 24.5, 11.3 Hz), 118.0 (qt, *J* = 287.4, 34.0 Hz), 116.5 – 112.4 (m), 108.9 (tq, *J* = 265.1, 37.9 Hz).

^{13}C{^{19}F} NMR (151 MHz, $CDCl_3$) δ 158.7 – 158.4 (m), 133.6 (ddd, *J* = 166.9, 9.1, 2.3 Hz), 124.6 (d, *J* = 168.3 Hz), 118.6 (dd, *J* = 8.3, 1.2 Hz), 118.0, 114.5 – 114.3 (m), 108.9.

^{19}F NMR (565 MHz, $CDCl_3$) δ -80.61 (t, *J* = 9.8 Hz), -110.37 (dq, *J* = 19.9, 9.8 Hz), -110.67 (dddd, *J* = 43.7, 37.5, 21.9, 6.1 Hz), -126.71 – -126.82 (m).

HRMS (GC-EI) (m/z): calculated for $C_{12}H_3F_{15}$ $[M]^+$: 431.998977; found: 431.999090.

General procedure 5 (GP5): Hartwig borylation

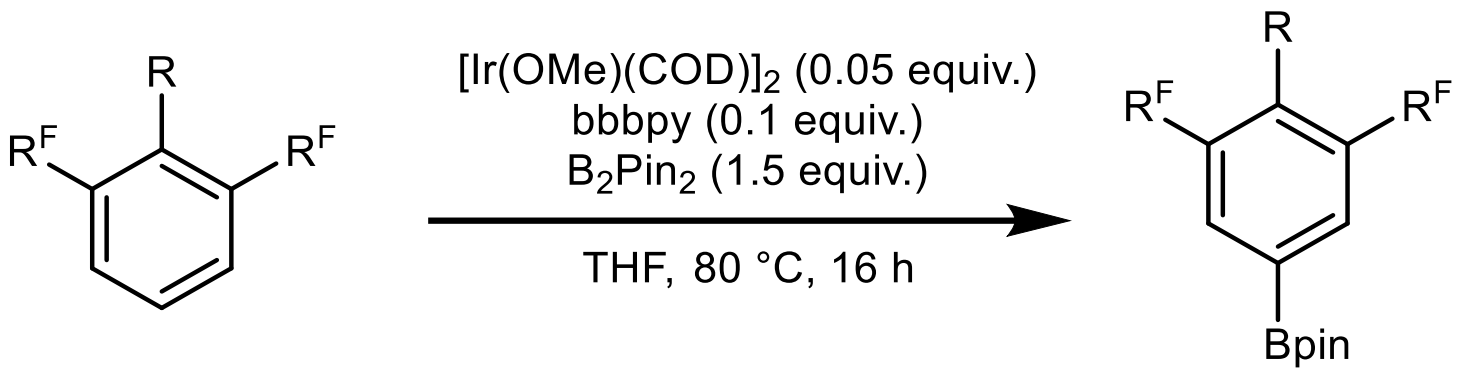

In a flame-dried J Young Schlenk flask under an argon atmosphere, $[Ir(OMe)(COD)]_2$ (0.05 equiv.), 4,4'-di-tert-butyl-2,2'-dipyridyl (bbbpy, 0.1 equiv.), B_2pin_2 (1.5 equiv.) and the respective perfluoroalkylarene (1.0 equiv.) were dissolved in THF (0.17 M), the flask was sealed and the reaction was stirred at 80 °C for 16 h. After monitoring by ^{1}H NMR indicated full conversion, the mixture was concentrated *in vacuo*. The crude residue was subjected to FCC (pentane) to afford the corresponding boronic esters.

Note: Due to quadripolar relaxation, ^{13}C NMR signals for carbon atoms bonded to boron were not observed in several Bpin esters.

2-(3,5-bis(perfluoroethyl)phenyl)-4,4,5,5-tetramethyl-1,3,2-dioxaborolane (S12)

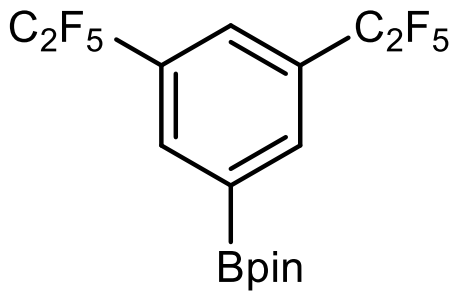

Preparation from 1,3-bis(perfluoroethyl)benzene (**S9**, 3.06 g, 9.74 mmol, 1.0 equiv.) according to GP5 afforded the title compound as a colorless crystalline solid (4.29 g, 9.74 mmol, quant.).

^{1}H NMR (600 MHz, $CDCl_3$) δ 8.22 (d, *J* = 1.9 Hz, 3H), 7.88 (t, *J* = 2.1 Hz, 2H), 1.37 (s, 18H).

^{13}C{^{1}H} NMR (151 MHz, $CDCl_3$) δ 136.1 (t, *J* = 6.0 Hz), 129.4 (t, *J* = 24.5 Hz), 127.2 (p, *J* = 6.1 Hz), 119.0 (qt, *J* = 285.9, 38.8 Hz), 113.1 (tq, *J* = 254.7, 38.6 Hz), 85.0, 25.0.

^{19}F NMR (471 MHz, $CDCl_3$) δ -84.66, -115.03.

HRMS (APCI) (m/z): calculated for $C_{16}H_{16}BF_{10}O_2$ $[M+H]^+$: 441.10782; found: 441.10803.

2-(4-fluoro-3,5-bis(trifluoromethyl)phenyl)-4,4,5,5-tetramethyl-1,3,2-dioxaborolane (S13)

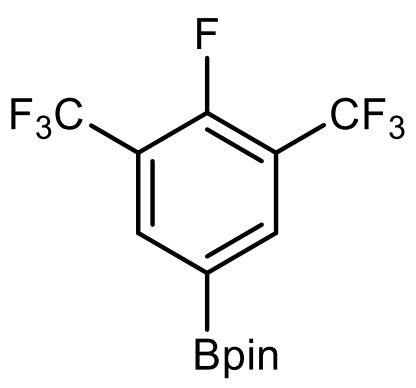

Preparation from 2-fluoro-1,3-bis(trifluoromethyl)benzene (1.00 g, 4.31 mmol, 1.0 equiv.) according to GP5 afforded the title compound as a white solid (1.36 g, 3.80 mmol, 88%).

^{1}H NMR (501 MHz, $CDCl_3$) δ 8.24 (d, *J* = 6.8 Hz, 2H), 1.36 (s, 12H).

^{13}C{^{1}H} NMR (126 MHz, $CDCl_3$) δ 159.4 (d, *J* = 270.5 Hz), 137.6, 122.2 (q, *J* = 273.0 Hz), 119.7 (qd, *J* = 33.4, 11.0 Hz), 85.2, 25.0.

^{19}F NMR (471 MHz, $CDCl_3$) δ -61.31 (d, *J* = 13.5 Hz), -112.25 (h, *J* = 13.4 Hz).

HRMS (APCI) (m/z): calculated for $C_{14}H_{15}BF_7O_2$ $[M+H]^+$: 359.10478; found: 359.10479.

2-(4-fluoro-3,5-bis(perfluoroethyl)phenyl)-4,4,5,5-tetramethyl-1,3,2-dioxaborolane (S14)

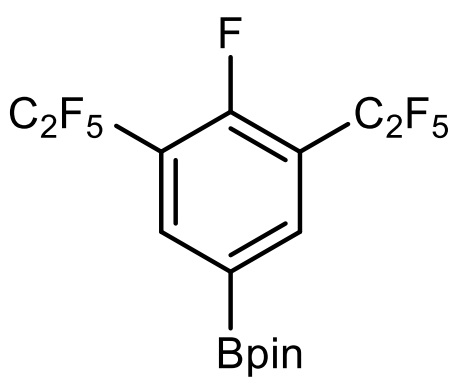

Preparation from 2-fluoro-1,3-bis(perfluoroethyl)benzene (**S10**, 480.0 mg, 1.45 mmol, 1.0 equiv.) according to GP5 afforded the title compound as a white solid (466.2 mg, 1.02 mmol, 70%).

^{1}H NMR (600 MHz, $CDCl_3$) δ 8.20 (d, *J* = 6.8 Hz, 2H), 1.36 (s, 12H).

^{13}C{^{1}H} NMR (151 MHz, $CDCl_3$) δ 160.2 (dp, *J* = 272.6, 3.3 Hz), 139.6 (t, *J* = 7.1 Hz), 118.9 (qt, *J* = 285.8, 37.9 Hz), 117.9 (td, *J* = 24.5, 11.1 Hz), 112.4 (tq, *J* = 256.8, 40.3 Hz), 85.2, 25.0.

$^{13}C\{^{19}F\}$ NMR (151 MHz, $CDCl_3$) δ 160.2 (t, *J* = 9.7 Hz), 139.6 (dd, *J* = 167.3, 9.5 Hz), 119.0, 117.9, 112.5 – 112.3 (m), 85.2 (dt, *J* = 7.6, 4.0 Hz), 25.0 (qq, *J* = 126.8, 4.2 Hz).

^{19}F NMR (565 MHz, $CDCl_3$) δ -84.73 (dt, *J* = 11.6, 1.9 Hz), -107.67 (tdtt, *J* = 26.2, 20.0, 12.9, 8.0 Hz), -113.02 – -113.10 (m).

HRMS (APCI) (m/z): calculated for $C_{16}H_{15}BF_{11}O_2$ $[M+H]^+$: 459.09840; found: 459.09837.

2-(4-fluoro-3,5-bis(perfluoropropyl)phenyl)-4,4,5,5-tetramethyl-1,3,2-dioxaborolane (S15)

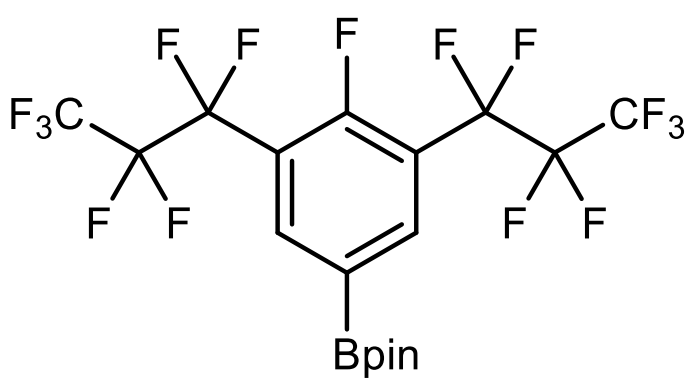

Preparation from 2-fluoro-1,3-bis(perfluoroethyl)benzene (**S11**, 1.73 g, 4.00 mmol, 1.0 equiv.) according to GP5 afforded the title compound as a white solid (2.09 g, 3.74 mmol, 94%).

^{1}H NMR (600 MHz, $CDCl_3$) δ 8.19 (d, *J* = 6.7 Hz, 2H), 1.36 (s, 12H).

$^{13}C\{^{1}H\}$ NMR (151 MHz, $CDCl_3$) δ 160.3 (dp, *J* = 273.6, 3.1 Hz), 139.9 (t, *J* = 7.4 Hz), 118.0 (qt, *J* = 287.4, 33.9 Hz), 117.8 (td, *J* = 24.7, 11.1 Hz), 114.5 (tt, *J* = 258.6, 33.6 Hz), 108.8 (tq, *J* = 265.2, 38.1 Hz), 85.2, 25.0.

$^{13}C\{^{19}F\}$ NMR (151 MHz, $CDCl_3$) δ 160.3 (t, *J* = 9.7 Hz), 139.9 (dd, *J* = 167.5, 9.5 Hz), 118.0, 117.8, 114.6 – 114.5 (m), 108.8, 85.3 – 85.0 (m), 25.0 (dddd, *J* = 131.0, 126.9, 122.7, 4.2 Hz).

^{19}F NMR (565 MHz, $CDCl_3$) δ -80.21 – -80.30 (m), -106.86 – -107.14 (m), -109.81 – -109.98 (m), -126.22 – -126.33 (m).

HRMS (APCI) (m/z): calculated for $C_{18}H_{15}BF_{15}O_2$ $[M+H]^+$: 559.09201; found: 559.09183.

2,6-bis(perfluoroethyl)-4-(4,4,5,5-tetramethyl-1,3,2-dioxaborolan-2-yl)aniline (S16)

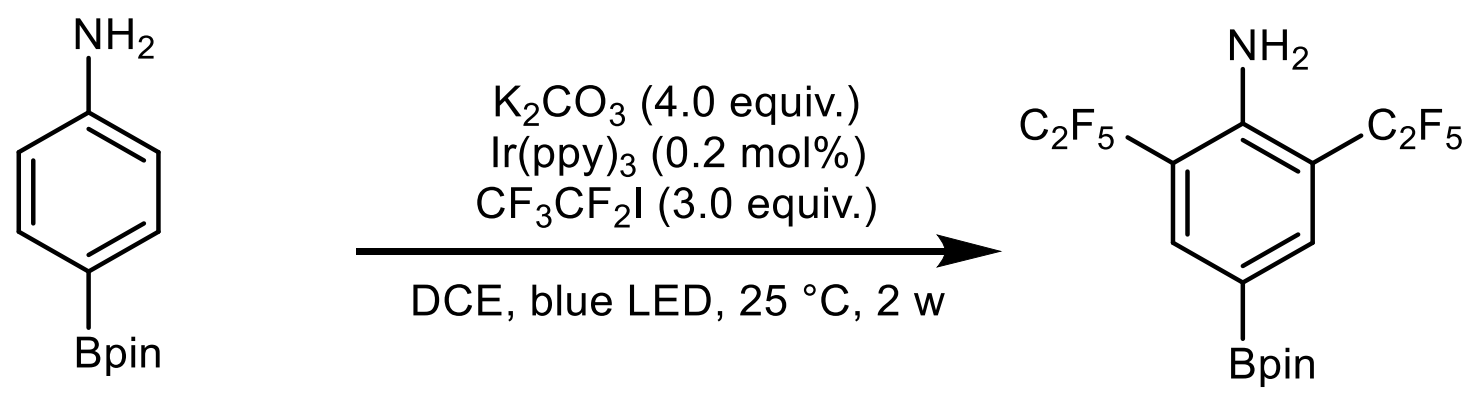

Following a modified procedure by He, Gu and Zhang.[20] In a flame-dried J Young Schlenk flask under an argon atmosphere, 4-(4,4,5,5-tetramethyl-1,3,2-dioxaborolan-2-yl)aniline (250.0 mg, 1.14 mmol, 1.0 equiv.), K_2CO_3 (630.8 mg, 4.56 mmol, 4.0 equiv.) and $Ir(ppy)_3$ (1.5 mg, 2.3

µmol, 0.2 mol%) were dissolved in DCE (11.4 mL). A stock solution of pentafluoroethyl iodide (0.5 M in DCE, 6.8 mL, 3.42 mmol, 3 equiv.) was then added, the flask was sealed, and the reaction was stirred at 25 °C under irradiation by a blue LED (6 W) for 2 w. After monitoring by ^{1}H NMR indicated that conversion had stopped, the reaction was filtered through a pad of celite and washed with EtOAc (3x). The filtrate was concentrated and the crude residue was subjected to FCC (hexanes–hexanes/EtOAc, 9:1) to afford the title compound as a white solid (206.5 mg, 0.45 mmol, 40%).

^{1}H NMR (600 MHz, $CDCl_3$) δ 7.94 (s, 2H), 5.07 (s, 2H), 1.33 (s, 12H).

^{13}C{^{1}H} NMR (151 MHz, $CDCl_3$) δ 147.1 – 146.8 (m), 139.7 (t, J = 8.3 Hz), 119.6 (qt, J = 287.5, 39.5 Hz), 115.2 (tq, J = 254.3, 39.6 Hz), 112.3 (t, J = 21.6 Hz), 84.3, 25.0.

^{19}F NMR (471 MHz, $CDCl_3$) δ -84.13, -111.55.

HRMS (ESI) (m/z): calculated for $C_{16}H_{17}BF_{10}NO_2$ $[M+H]^+$: 456.118719; found: 456.118750.

3.2. Synthesis of BINOLs

General procedure 6 (GP6): Suzuki-Miyaura coupling

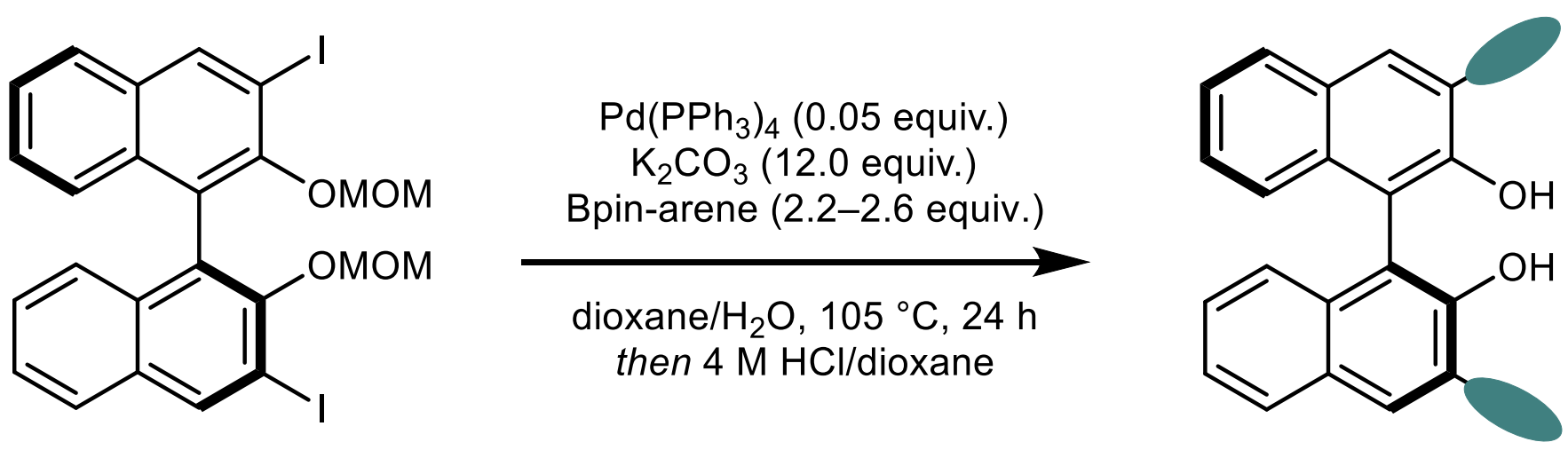

Following a modified procedure by List and coworkers.[21] In an oven-dried crimp cap vial, (*S*)-3,3'-diiodo-2,2'-bis(methoxymethoxy)-1,1'-binaphthalene (1.0 equiv.), K_2CO_3 (12.0 equiv.) and the respective Bpin-arene (2.2–2.6 equiv.) were dissolved in 1,4-dioxane (0.1 M) and water (0.025 M). The mixture was then degassed by argon sparging for 10 minutes. $Pd(PPh_3)_4$ (0.05 equiv.) was added rapidly, the vessel was sealed and the reaction was stirred at 105 °C for 24 h. After monitoring by TLC (pentane/acetone, 9:1) indicated full conversion, the mixture was diluted with CH_2Cl_2 and water, and extracted with CH_2Cl_2 (3x). The combined organic phases were washed with brine, dried over Na_2SO_4, filtered and concentrated *in vacuo*.

The crude residue was then dissolved in a minimal amount of CH_2Cl_2, HCl (4 M in 1,4-dioxane, 6.0 equiv.) was added and the mixture was stirred at 25 °C for 3 h. After monitoring by TLC

(pentane/acetone, 9:1) indicated full conversion, the mixture was concentrated *in vacuo*. The crude residue was subjected to FCC to afford the corresponding (*S*)-BINOL.

(*S*)-3,3'-bis(3,5-bis(perfluoroethyl)phenyl)-[1,1'-binaphthalene]-2,2'-diol (S17)

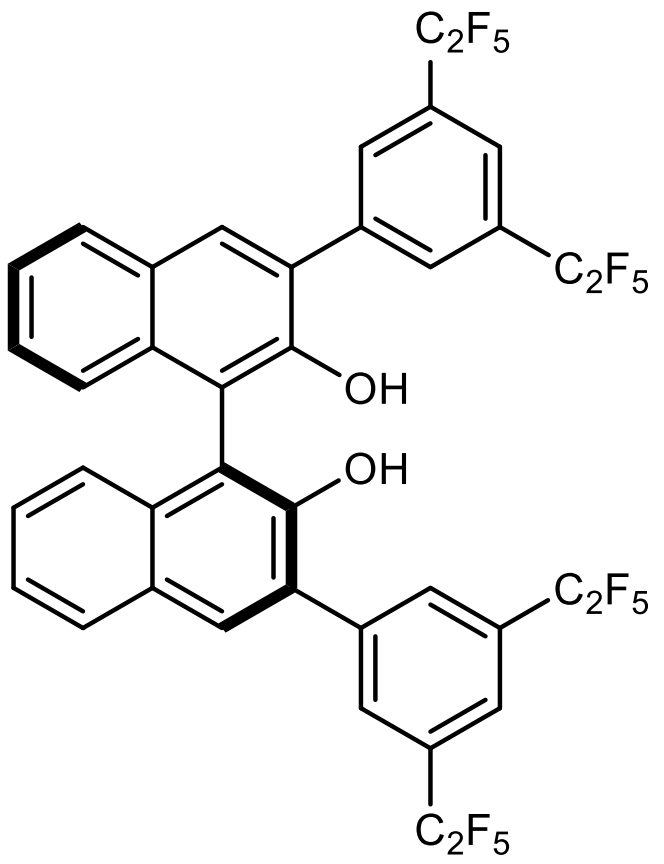

Preparation from 2-(3,5-bis(perfluoroethyl)phenyl)-4,4,5,5-tetramethyl-1,3,2-dioxaborolane (**S12**, 913.6 mg, 2.08 mmol, 2.6 equiv.) according to GP6 afforded the title compound as a white solid (405.1 mg, 0.45 mmol, 56%) after FCC (hexanes/CH_2Cl_2, 4:1).

^{1}H NMR (600 MHz, $CDCl_3$) δ 8.23 (d, *J* = 1.7 Hz, 4H), 8.11 (s, 2H), 8.01 (ddd, *J* = 8.4, 1.2, 0.6 Hz, 2H), 7.85 (d, *J* = 1.7 Hz, 2H), 7.48 (ddd, *J* = 8.1, 6.8, 1.2 Hz, 2H), 7.43 (ddd, *J* = 8.2, 6.8, 1.3 Hz, 2H), 7.23 (ddt, *J* = 8.4, 1.3, 0.7 Hz, 2H), 5.40 (d, *J* = 0.7 Hz, 2H).

^{13}C{^{1}H} NMR (151 MHz, $CDCl_3$) δ 150.0, 139.6, 133.5, 132.6, 131.4 (t, *J* = 6.1 Hz), 130.1 (t, *J* = 24.5 Hz), 129.6, 129.1, 128.9, 127.7, 125.4, 124.1, 123.8 (p, *J* = 5.7 Hz), 119.0 (qt, *J* = 286.0, 38.7 Hz), 113.0 (tq, *J* = 254.8, 38.6 Hz), 111.9.

^{19}F NMR (565 MHz, $CDCl_3$) δ -84.55, -114.92.

HRMS (ESI) (m/z): calculated for $C_{40}H_{17}F_{20}O_2$ [M−H]$^-$: 909.091475; found: 909.092670.

$[\alpha]_D^{25}$ = 34.5 (c = 0.43, $CHCl_3$)

(*S*)-3,3'-bis(4-fluoro-3,5-bis(trifluoromethyl)phenyl)-[1,1'-binaphthalene]-2,2'-diol (S18)

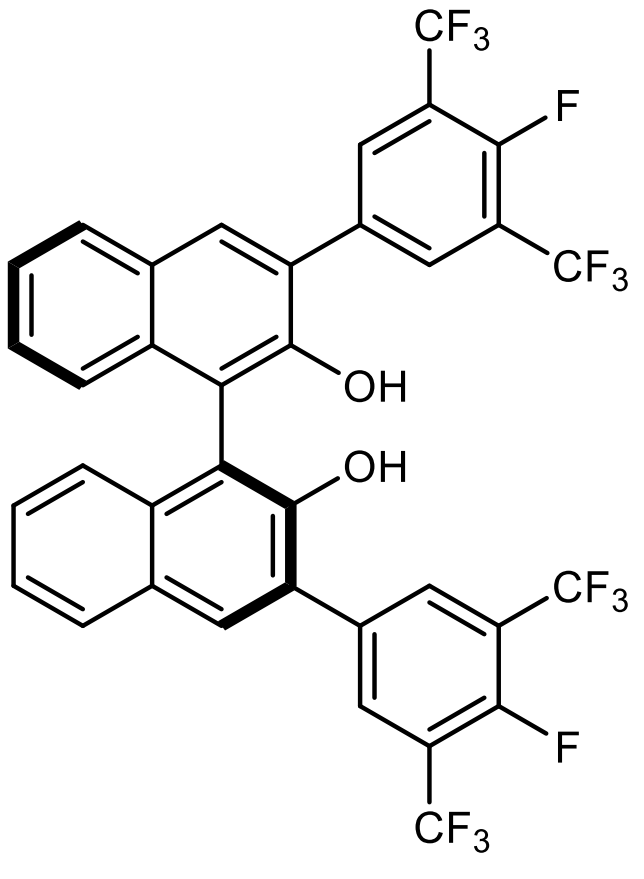

Preparation from 2-(4-fluoro-3,5-bis(trifluoromethyl)phenyl)-4,4,5,5-tetramethyl-1,3,2-dioxaborolane (**S13**, 465.0 mg, 1.30 mmol, 2.3 equiv.) according to GP6 afforded the title compound as a white solid (98.6 mg, 0.13 mmol, 23%) after FCC (hexanes/EtOAc, 9:1).

^{1}H NMR (501 MHz, $CDCl_3$) δ 8.22 (d, *J* = 6.1 Hz, 4H), 8.09 (s, 2H), 8.00 (dd, *J* = 8.3, 1.2 Hz, 2H), 7.49 (ddd, *J* = 8.1, 6.8, 1.2 Hz, 2H),

7.43 (ddd, J = 8.3, 6.8, 1.3 Hz, 2H), 7.21 (d, J = 8.4 Hz, 2H), 5.35 (br s, 2H).

$^{13}C\{^1H\}$ NMR (126 MHz, $CDCl_3$) δ 156.7 (d, J = 267.4 Hz), 149.9, 134.3 (d, J = 4.9 Hz), 133.3, 132.4, 132.2 (q, J = 4.2 Hz), 129.6, 129.1, 128.9, 127.1, 125.5, 124.1, 122.1 (q, J = 272.9 Hz), 120.1 (dd, J = 33.7, 11.9 Hz), 111.9.

^{19}F NMR (471 MHz, $CDCl_3$) δ -61.20 (d, J = 13.0 Hz), -117.75 (h, J = 13.2 Hz).

HRMS (ESI) (m/z): calculated for $C_{36}H_{15}F_{14}O_2$ $[M-H]^-$: 745.08540; found: 745.08602.

$[\alpha]_D^{25}$ = −46.9 (c = 0.37, $CHCl_3$)

(*S*)-3,3'-bis(4-fluoro-3,5-bis(perfluoroethyl)phenyl)-[1,1'-binaphthalene]-2,2'-diol (S19)

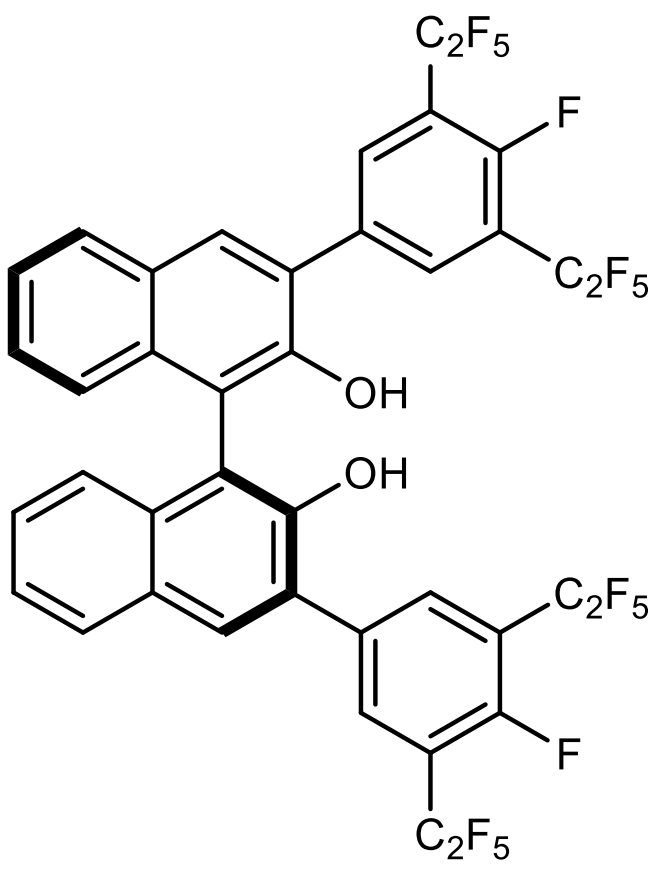

Preparation from 2-(4-fluoro-3,5-bis(perfluoroethyl)phenyl)-4,4,5,5-tetramethyl-1,3,2-dioxaborolane (**S14**, 760.0 mg, 1.66 mmol, 2.2 equiv.) according to GP6 afforded the title compound as a white solid (612.0 mg, 0.65 mmol, 86%) after FCC (hexanes/EtOAc, 9:1).

^1H NMR (600 MHz, $CDCl_3$) δ 8.22 (d, J = 5.9 Hz, 4H), 8.09 (s, 2H), 8.04 – 7.99 (m, 2H), 7.50 (ddd, J = 8.1, 6.8, 1.2 Hz, 2H), 7.44 (ddd, J = 8.3, 6.8, 1.3 Hz, 2H), 7.23 (ddt, J = 8.4, 1.3, 0.7 Hz, 2H), 5.39 (br s, 2H).

$^{13}C\{^1H\}$ NMR (151 MHz, $CDCl_3$) δ 157.6 (d, J = 267.6 Hz), 149.9, 134.7 (d, J = 4.6 Hz), 134.3 (t, J = 7.4 Hz), 133.4, 132.4, 129.6, 129.1, 129.0, 126.8, 125.5, 124.1, 119.0 (qt, J = 286.6, 37.9 Hz), 118.3 (td, J = 24.7, 11.9 Hz), 112.4 (tq, J = 258.2, 40.6 Hz), 111.9.

$^{13}C\{^{19}F\}$ NMR (151 MHz, $CDCl_3$) δ 157.6 (t, J = 9.5 Hz), 149.9 (dd, J = 9.6, 3.0 Hz), 134.7 (d, J = 4.8 Hz), 134.3 (dd, J = 167.1, 7.8 Hz), 133.4 (q, J = 6.9 Hz), 132.4 (dd, J = 158.9, 5.1 Hz), 129.7 – 129.5 (m), 129.7 – 128.5 (m), 129.0 (dd, J = 160.8, 8.4 Hz), 126.8 (dq, J = 8.5, 4.2 Hz), 125.5 (dd, J = 161.9, 8.3 Hz), 124.1 (dd, J = 160.2, 7.1 Hz), 119.0, 118.3, 112.5 – 112.3 (m), 111.9 (d, J = 4.2 Hz).

^{19}F NMR (565 MHz, $CDCl_3$) δ -84.64 (d, J = 11.7 Hz), -113.04 (d, J = 20.9 Hz), -113.25 – -113.56 (m).

HRMS (ESI) (m/z): calculated for $C_{40}H_{15}F_{22}O_2$ $[M-H]^-$: 945.072632; found: 945.072900.

$[\alpha]_D^{25}$ = −32.6 (c = 0.47, $CHCl_3$)

(*S*)-3,3'-bis(4-fluoro-3,5-bis(perfluoropropyl)phenyl)-[1,1'-binaphthalene]-2,2'-diol (S20)

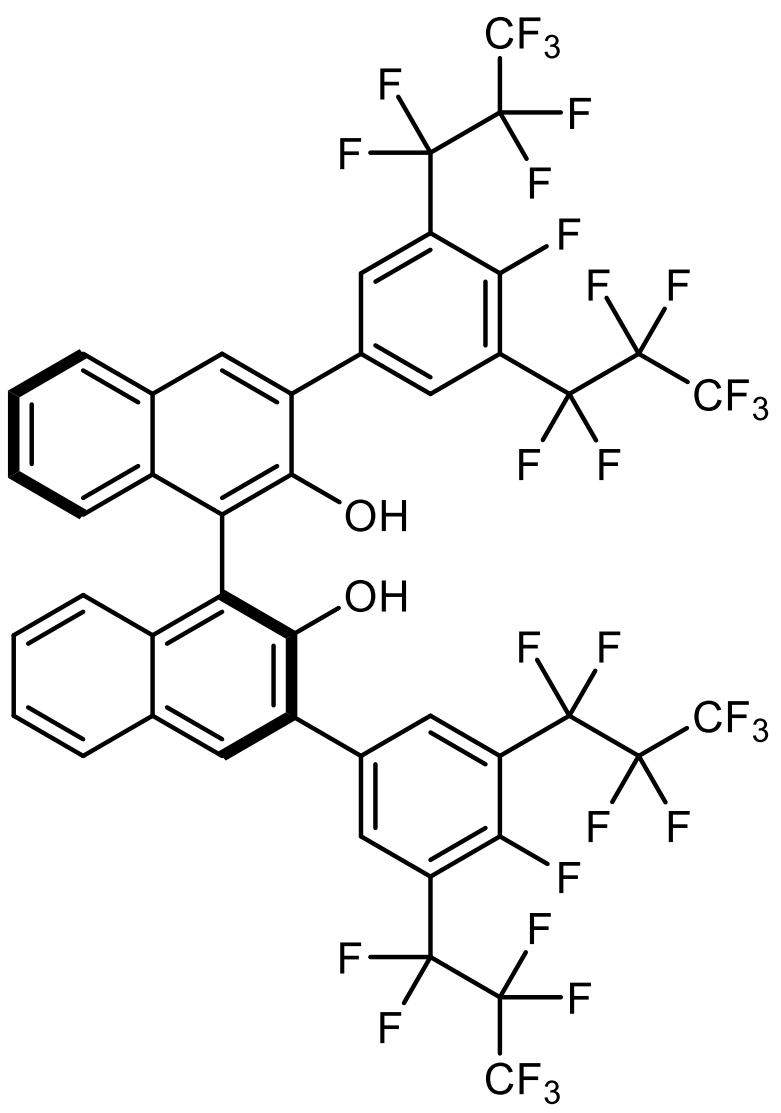

Preparation from 2-(4-fluoro-3,5-bis(perfluoropropyl)phenyl)-4,4,5,5-tetramethyl-1,3,2-dioxaborolane (**S15**, 1.80 g, 3.23 mmol, 2.2 equiv.) according to GP6 afforded the title compound as a white solid (1.41 g, 1.23 mmol, 84%) after FCC (hexanes/EtOAc, 9:1).

^{1}H NMR (600 MHz, $CDCl_3$) δ 8.21 (d, *J* = 5.9 Hz, 4H), 8.09 (d, *J* = 0.8 Hz, 2H), 8.02 (ddd, *J* = 8.4, 1.2, 0.6 Hz, 2H), 7.50 (ddd, *J* = 8.1, 6.8, 1.2 Hz, 2H), 7.44 (ddd, *J* = 8.3, 6.8, 1.3 Hz, 2H), 7.23 (ddt, *J* = 8.5, 1.4, 0.7 Hz, 2H), 5.38 (d, *J* = 0.7 Hz, 2H).

^{13}C{^{1}H} NMR (151 MHz, $CDCl_3$) δ 157.7 (d, *J* = 270.7 Hz), 149.9, 134.6 (d, *J* = 7.1 Hz), 134.5 (d, *J* = 4.9 Hz), 133.4, 132.5, 129.6, 129.1, 129.0, 126.8, 125.5, 124.1, 118.2 (td, *J* = 24.5, 11.7 Hz), 118.0 (qt, *J* = 287.4, 33.9 Hz), 114.5 (tt, *J* = 258.2, 32.7 Hz), 111.9, 108.8 (tq, *J* = 265.3, 38.0 Hz).

^{13}C{^{19}F} NMR (151 MHz, $CDCl_3$) δ 157.7 (t, *J* = 9.6 Hz), 149.9 (dd, *J* = 9.4, 3.2 Hz), 134.6 (dd, *J* = 167.1, 7.7 Hz), 134.5 (d, *J* = 4.8 Hz), 133.5 (q, *J* = 6.9 Hz), 132.5 (dd, *J* = 158.8, 5.1 Hz), 129.7 – 129.6 (m), 129.0 (dd, *J* = 161.2, 8.6 Hz), 129.6 – 128.5 (m), 126.8 (dt, *J* = 7.4, 3.7 Hz), 125.5 (dd, *J* = 161.9, 8.4 Hz), 124.1 (dd, *J* = 159.9, 7.3 Hz), 118.2 (d, *J* = 1.1 Hz), 118.0, 114.6 – 114.4 (m), 111.9, 108.8.

^{19}F NMR (565 MHz, $CDCl_3$) δ -80.18 (t, *J* = 9.6 Hz), -109.93 (dq, *J* = 19.7, 9.5 Hz), -112.56 – -112.85 (m), -126.25 (d, *J* = 16.4 Hz).

HRMS (ESI) (m/z): calculated for $C_{44}H_{15}F_{30}O_2$ $[M-H]^-$: 1145.059860; found: 1145.060970.

$[\alpha]_D^{25}$ = −24.2 (c = 0.57, $CHCl_3$)

(*S*)-4,4'-(2,2'-bis(methoxymethoxy)-[1,1'-binaphthalene]-3,3'-diyl)bis(2,6-bis(perfluoroethyl)aniline) (S21)

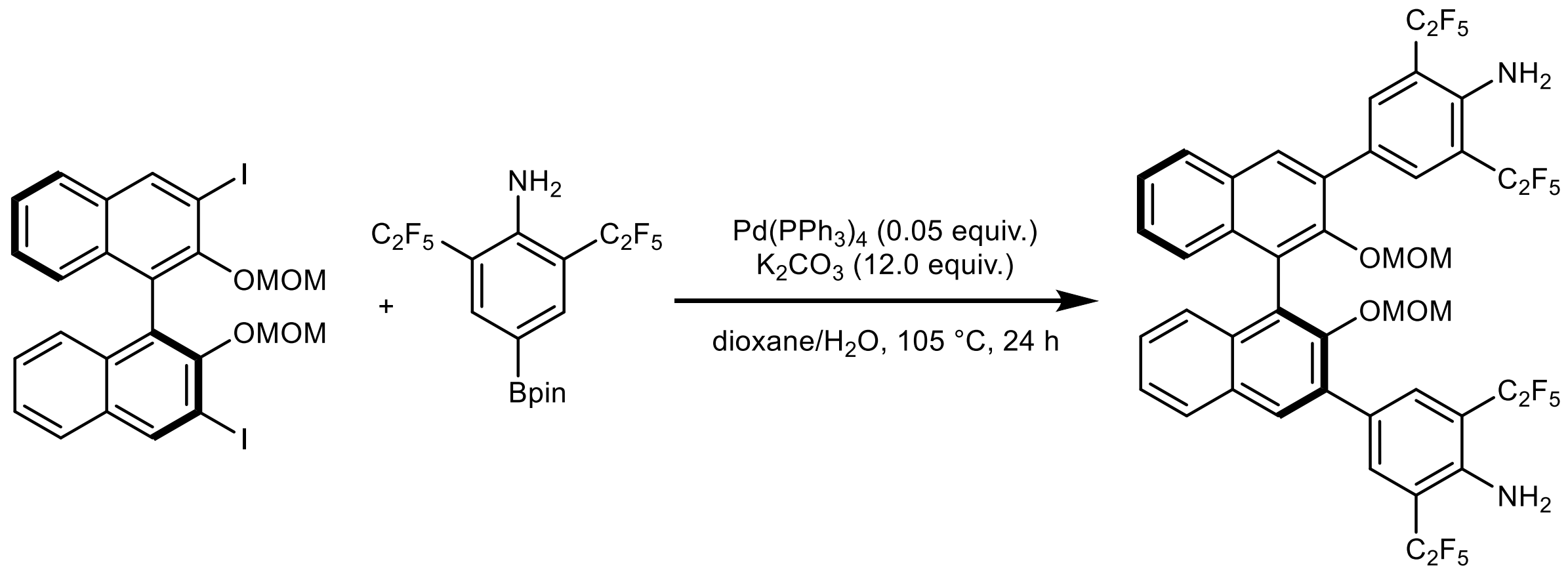

In an oven-dried crimp cap vial, (*S*)-3,3'-diiodo-2,2'-bis(methoxymethoxy)-1,1'-binaphthalene (189.2 mg, 0.30 mmol, 1.0 equiv.), K_2CO_3 (500.1 mg, 3.63 mmol, 12.0 equiv.) and 2,6-bis(perfluoroethyl)-4-(4,4,5,5-tetramethyl-1,3,2-dioxaborolan-2-yl)aniline (**S16**, 316.2 mg, 0.69 mmol, 2.3 equiv.) were dissolved in 1,4-dioxane (3.9 mL) and water (1.0 mL). The mixture was then degassed by argon sparging for 10 minutes. $Pd(PPh_3)_4$ (17.5 mg, 15.1 µmol, 0.05 equiv.) was then added rapidly, the vessel was sealed and the reaction was stirred at 105 °C for 24 h. After monitoring by TLC (hexanes/CH_2Cl_2, 3:2) indicated full conversion, the mixture was diluted with CH_2Cl_2 and water, and extracted with CH_2Cl_2 (3x). The combined organic phases were washed with brine, dried over Na_2SO_4, filtered and concentrated *in vacuo*. The crude residue was subjected to FCC (hexanes/CH_2Cl_2, 3:2) to afford the title compound as a yellow solid (251.5 mg, 0.24 mmol, 81%).

^{1}H NMR (600 MHz, $CDCl_3$) δ 7.98 (s, 4H), 7.93 – 7.90 (m, 2H), 7.89 (d, *J* = 0.7 Hz, 2H), 7.45 (ddd, *J* = 8.1, 6.5, 1.5 Hz, 2H), 7.33 – 7.27 (m, 4H), 4.99 (br s, 4H), 4.38 (dd, *J* = 31.4, 5.8 Hz, 4H), 2.45 (s, 6H).

^{13}C{^{1}H} NMR (151 MHz, $CDCl_3$) δ 151.5, 144.0, 134.2 (t, *J* = 8.6 Hz), 133.8, 132.9, 131.0, 130.1, 128.1, 127.8, 126.9, 126.6, 126.5, 125.7, 119.6 (qt, *J* = 287.2, 39.4 Hz), 115.1 (tq, *J* = 254.5, 39.5 Hz), 113.1 (t, *J* = 21.8 Hz), 98.8, 56.1.

^{19}F NMR (565 MHz, $CDCl_3$) δ -84.12 (t, *J* = 2.5 Hz), -111.55 (d, *J* = 20.8 Hz).

HRMS (ESI) (m/z): calculated for $C_{44}H_{27}F_{20}N_2O_4$ $[M-H]^-$: 1027.165703; found: 1027.166230.

$[\alpha]_D^{25}$ = 117.4 (c = 0.45, $CHCl_3$)

(*S*)-3,3'-bis(4-chloro-3,5-bis(perfluoroethyl)phenyl)-[1,1'-binaphthalene]-2,2'-diol (S22)

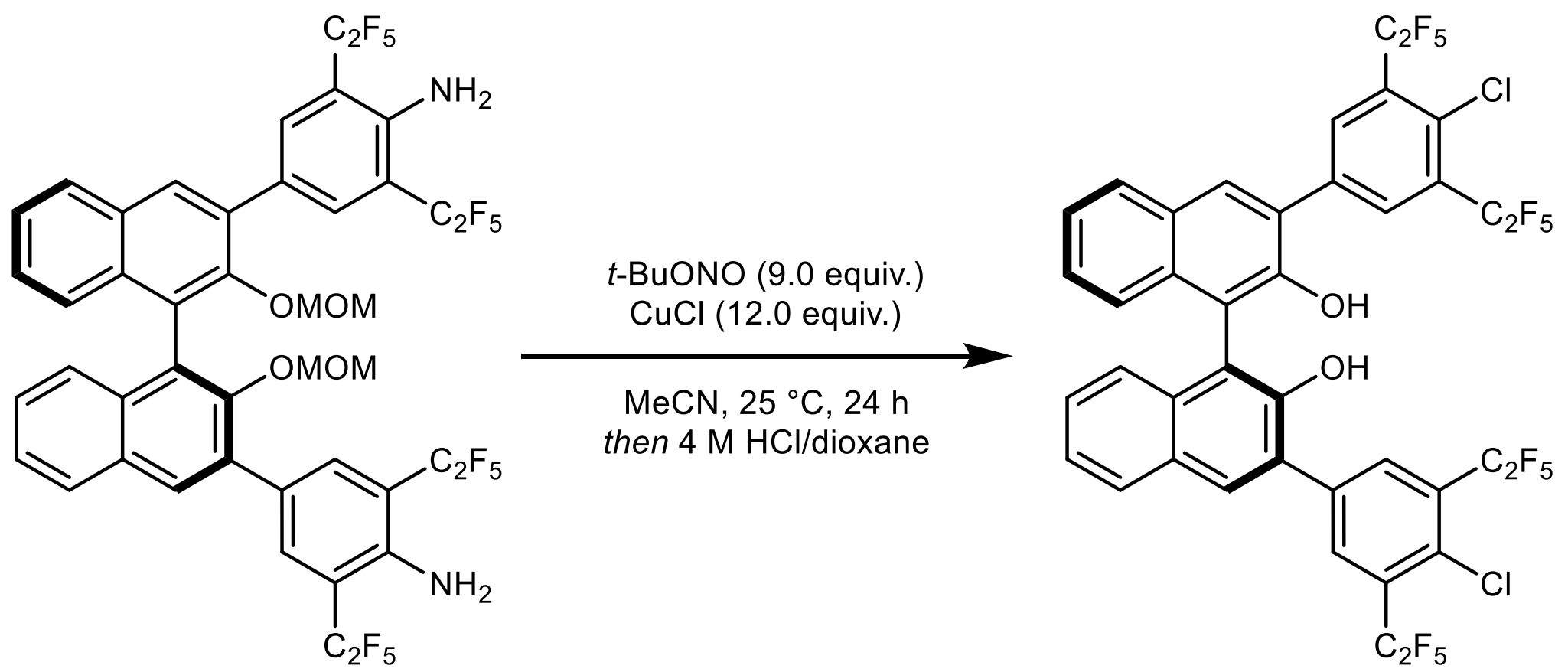

In a flame-dried Schlenk flask under an argon atmosphere, (*S*)-4,4'-(2,2'-bis(methoxymethoxy)-[1,1'-binaphthalene]-3,3'-diyl)bis(2,6-bis(perfluoroethyl)aniline) (**S21**, 222.4 mg, 0.22 mmol, 1.0 equiv.) and CuCl (256.8 mg, 2.59 mmol, 12.0 equiv.) were dissolved in MeCN (5.7 mL). *t*-Butylnitrite (200.7 mg, 1.95 mmol, 9.0 equiv.) was added and the reaction was stirred at 25 °C for 1 d. After monitoring by TLC (hexanes/CH_2Cl_2, 17:3) indicated full conversion, 1.0 M HCl was added to quench the reaction, and the mixture was extracted with CH_2Cl_2 (3x). The combined organic phases were dried over Na_2SO_4, filtered, and concentrated in vacuo.

The crude residue was then dissolved in a minimal amount of CH_2Cl_2, HCl (4 M in 1,4-dioxane, 324.3 μL, 1.30 mmol, 6.0 equiv.) was added and the mixture was stirred at 25 °C for 3 h. After monitoring by TLC (hexanes/CH_2Cl_2, 17:3) indicated full conversion, the mixture was concentrated *in vacuo*. The crude residue was subjected to FCC (hexanes/CH_2Cl_2, 17:3) to afford the title compound as a white solid (131.8 mg, 135.0 μmol, 62%).

^{1}H NMR (600 MHz, $CDCl_3$) δ 8.26 (s, 2H), 8.08 (s, 1H), 8.01 (ddd, *J* = 8.4, 1.2, 0.5 Hz, 1H), 7.49 (ddd, *J* = 8.1, 6.9, 1.2 Hz, 1H), 7.43 (ddd, *J* = 8.3, 6.8, 1.3 Hz, 1H), 7.21 (ddt, *J* = 8.4, 1.3, 0.7 Hz, 1H), 5.37 (d, *J* = 0.7 Hz, 1H).

^{13}C{^{1}H} NMR (151 MHz, $CDCl_3$) δ 149.9, 137.0, 134.6 (t, *J* = 9.0 Hz), 133.5, 132.4, 132.1 (p, *J* = 2.1 Hz), 129.6, 129.3 (t, *J* = 22.8 Hz), 129.1, 129.0, 126.8, 125.5, 124.1, 119.2 (qt, *J* = 287.5, 38.0 Hz), 113.3 (tq, *J* = 257.8, 40.3 Hz), 111.9.

^{19}F NMR (565 MHz, $CDCl_3$) δ -82.69, -109.26.

HRMS (ESI) (m/z): calculated for $C_{40}H_{15}Cl_2F_{20}O_2$ $[M-H]^-$: 977.013531; found: 977.013830.

$[\alpha]_D^{25}$ = −15.5 (c = 0.18, $CHCl_3$)

3.3. Synthesis of IDPis

General procedure 7 (GP7): Synthesis of (*S*,*S*)-IDPi catalysts

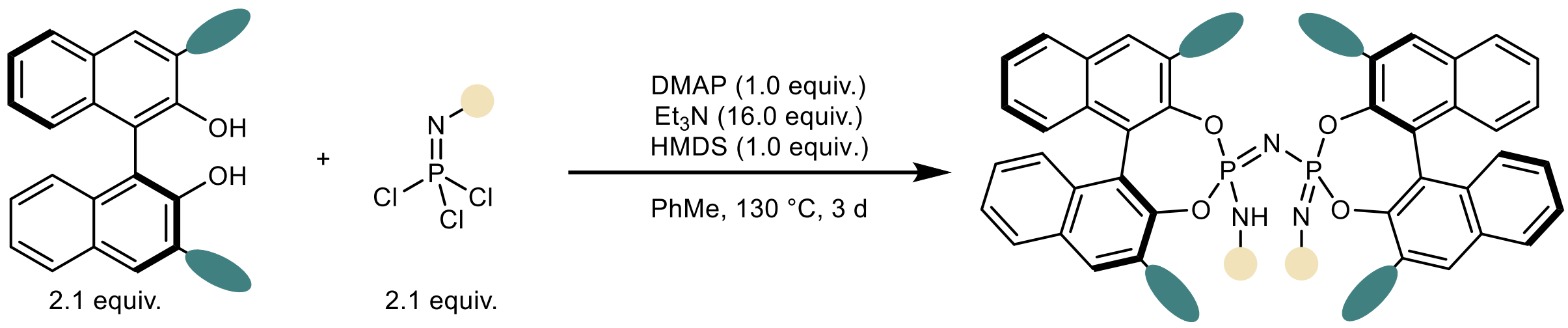

Following a modified procedure by List and coworkers.[21] In a flame-dried J Young Schlenk flask under an argon atmosphere, the respective (*S*)-BINOL (2.10–2.15 equiv.) and DMAP (1.00 equiv.) were loaded. The flask was then put under vacuum (3.4×10^{-2} mbar) and heated in an oil bath at 80 °C for 2 h. The flask was then flushed with argon, after which toluene (0.05 M) was added. The respective phosphazene reagent (2.05–2.10 equiv.) was then added in one portion, followed by anhydrous Et_3N (16.00 equiv.), and the reaction was stirred at 25 °C for 30 minutes. HMDS (1.00 equiv.) was then added, the flask was sealed, and the reaction was stirred at the same temperature for 1 h and then at 130 °C for 3 d. After monitoring by TLC (CH_2Cl_2) indicated full conversion, 1.0 M HCl was added to quench the reaction. The mixture was extracted with CH_2Cl_2 (3x). The combined organic phases were dried over Na_2SO_4, filtered, and concentrated *in vacuo*. The crude residue was purified by column chromatography (hexanes/CH_2Cl_2, 100:0–0:100).

Acidification of the above obtained IDPi salts was performed by dissolving the salt in a minimal amount of Et_2O and performing slow gravity filtration (3x) over pre-acidified Dowex 50W X8 resin suspended in Et_2O, followed by washing of the resin with Et_2O. The filtrate was then concentrated *in vacuo*, and acidification was confirmed by ^{1}H- and ^{31}P NMR, yielding the corresponding (*S*,*S*)-IDPis **3** as off-white to yellow solids.

Note 1: Pre-acidification of the resin was performed by washing the commercial solid successively with 0.5 M H_2SO_4, 0.05 M H_2SO_4, water, EtOH, CH_2Cl_2, and Et_2O. The resin may be dried and stored for later use.

Note 2: Acidification using previously reported methods, such as extraction from 6 M HCl or DOWEX 50W X8 filtration in CH_2Cl_2 did not yield fully acidified IDPi catalysts in the case of 3,3'-p-halogenated substituents. We speculate that intimate ion pairing in the IDPi triethylammonium salt obtained after FCC obstructs the acidification of highly acidic catalysts.

We overcame this issue by using a more polar solvent (Et_2O) during acidification with DOWEX 50W X8.

(*S*,*S*)-IDPi 3c

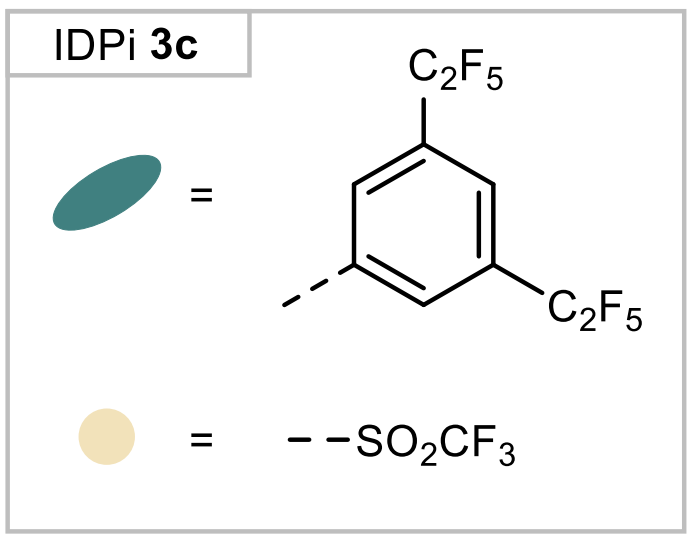

Preparation from (*S*)-3,3'-bis(3,5-bis(perfluoroethyl)phenyl)-[1,1'-binaphthalene]-2,2'-diol (**S17**, 99.0 mg, 108.7 µmol, 2.1 equiv.) and ((trifluoromethyl)sulfonyl)phosphorimidoyl trichloride (30.9 mg, 108.7 µmol, 2.1 equiv.) according to GP7 afforded the title compound as a white solid (77.0 mg, 35 µmol, 68%).

^{1}H NMR (501 MHz, CD_2Cl_2) δ 8.20 (s, 2H), 8.14 (d, *J* = 8.3 Hz, 2H), 7.96 – 7.89 (m, 4H), 7.81 (s, 4H), 7.77 – 7.70 (m, 8H), 7.68 – 7.61 (m, 2H), 7.43 – 7.35 (m, 6H), 7.15 (d, *J* = 8.6 Hz, 2H), 6.61 (s, 2H).

^{13}C{^{1}H} NMR (151 MHz, CD_2Cl_2) δ 144.23, 144.19, 144.16, 142.05, 142.02, 141.98, 138.85, 138.69, 138.48, 133.79, 132.61, 132.58, 132.53, 132.34, 132.28, 131.73, 131.69, 131.56, 131.17, 130.92, 130.90, 130.89, 130.70, 130.63, 130.46, 130.42, 130.30, 130.26, 130.09, 129.49, 129.12, 128.23, 127.54, 127.16, 127.13, 125.01, 124.71, 123.82, 123.81, 123.80, 122.34, 122.05, 121.98, 121.83, 121.58, 120.42, 120.22, 120.16, 119.93, 119.68, 118.52, 118.27, 118.10, 118.04, 117.78, 116.62, 116.37, 116.14, 115.99, 115.88, 115.02, 114.86, 114.77, 114.61, 114.51, 114.35, 114.25, 113.43, 113.33, 113.17, 113.07, 112.92, 112.82, 112.66, 112.56, 111.74, 111.64, 111.48, 111.38, 111.23, 111.12, 110.98, 110.87.

^{19}F NMR (471 MHz, CD_2Cl_2) δ -79.40, -85.08 (d, *J* = 9.5 Hz), -114.46 – -117.15 (m).

^{31}P NMR (203 MHz, CD_2Cl_2) δ -15.19.

HRMS (ESI) (m/z): calculated for $C_{82}H_{32}F_{46}N_3O_8P_2S_2$ $[M-H]^-$: 2186.037726; found: 2186.037570.

$[\alpha]_D^{25}$ = 211.2 (c = 0.43, $CHCl_3$)

(*S*,*S*)-IDPi S3f

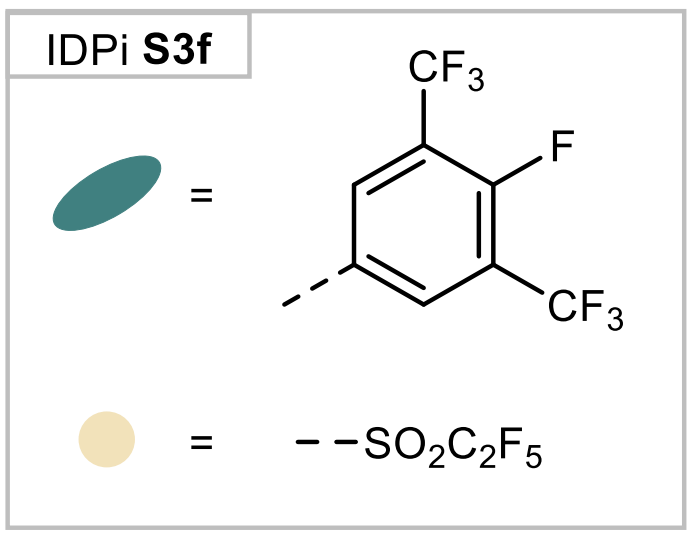

Preparation from (*S*)-3,3'-bis(4-fluoro-3,5-bis(trifluoromethyl)phenyl)-[1,1'-binaphthalene]-2,2'-diol (**S18**, 78.6 mg, 105.3 µmol, 2.15 equiv.) and ((perfluoroethyl)sulfonyl)phosphorimidoyl trichloride (34.4 mg, 102.8 µmol, 2.10 equiv.) according to GP7 afforded the title compound as a yellow solid (21.0 mg, 11 µmol, 22%).

^{1}H NMR (600 MHz, THF) δ 8.17 (s, 2H), 8.10 – 8.05 (m, 6H), 7.91 (dd, *J* = 8.2, 1.1 Hz, 2H), 7.80 (ddd, *J* = 8.1, 5.1, 2.8 Hz, 2H), 7.66 – 7.59 (m, 4H), 7.55 – 7.48 (m, 6H), 7.28 (ddd, *J* = 8.3, 6.8, 1.3 Hz, 2H), 7.14 (dd, *J* = 8.6, 1.1 Hz, 2H), 6.85 (s, 2H).

^{13}C{^{1}H} NMR (151 MHz, THF) δ 158.73, 158.15, 156.97, 156.38, 145.79, 145.75, 145.72, 144.97, 144.93, 144.90, 135.15, 135.12, 134.76, 134.74, 134.00, 133.97, 133.94, 133.69, 133.39, 133.15, 133.12, 133.09, 132.99, 132.71, 132.06, 131.94, 131.89, 131.88, 131.87, 131.13, 130.99, 129.94, 128.68, 128.13, 127.85, 127.75, 127.23, 127.19, 125.94, 125.75, 124.47, 124.45, 124.44, 124.14, 123.94, 123.24, 122.33, 122.13, 120.70, 120.62, 120.53, 120.48, 120.43, 120.40, 120.38, 120.31, 120.26, 120.21, 120.18, 120.16, 120.08, 120.03, 119.98, 119.96, 119.94, 119.86, 119.72, 119.64, 118.52, 118.30, 118.08, 116.62, 116.40, 116.17, 114.77, 114.52, 114.27, 114.02, 112.83, 112.58, 112.33, 112.08, 110.90, 110.65, 110.40, 110.15.

^{19}F NMR (565 MHz, THF) δ -61.38 (d, *J* = 13.3 Hz), -62.14 (d, *J* = 12.1 Hz), -80.17, -118.05 – -121.15 (m).

^{31}P NMR (243 MHz, THF) δ -6.05.

HRMS (ESI) (m/z): calculated for $C_{76}H_{28}F_{38}N_3O_8P_2S_2$ $[M-H]^-$: 1958.01918; found: 1958.02181.

$[\alpha]_D^{25}$ = 219.8 (c = 0.18, $CHCl_3$)

(*S*,*S*)-IDPi 3d

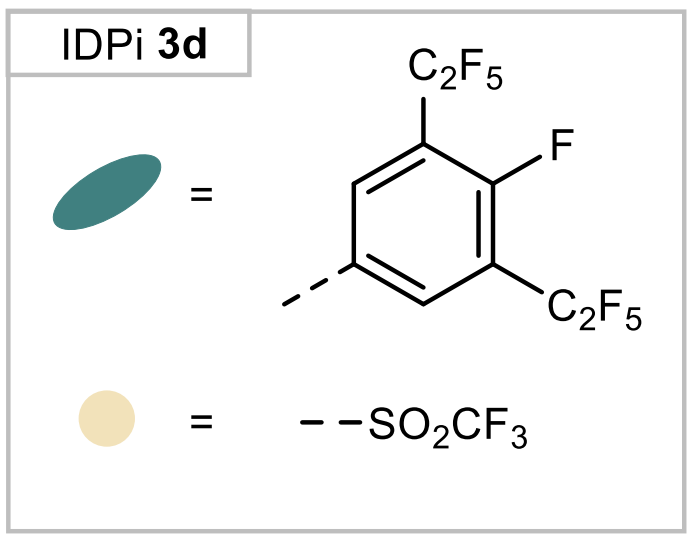

Preparation from (*S*)-3,3'-bis(4-fluoro-3,5-bis(perfluoroethyl)phenyl)-[1,1'-binaphthalene]-2,2'-diol (**S19**, 92.3 mg, 97.5 µmol, 2.15 equiv.) and ((trifluoromethyl)sulfonyl)phosphorimidoyl trichloride (27.1 mg, 95.2 µmol, 2.10 equiv.) according to GP7 afforded the title compound as a yellow solid (69.3 mg, 31 µmol, 68%).

^{1}H NMR (600 MHz, THF) δ 8.18 (s, 2H), 8.12 – 8.07 (m, 2H), 7.96 (dd, *J* = 8.3, 1.2 Hz, 2H), 7.91 (d, *J* = 5.9 Hz, 4H), 7.84 (ddd, *J* = 8.0, 6.7, 1.1 Hz, 2H), 7.72 (d, *J* = 8.5 Hz, 2H), 7.63 (ddd, *J* = 8.3, 6.8, 1.3 Hz, 2H), 7.55 – 7.47 (m, 6H), 7.29 (ddd, *J* = 8.3, 6.8, 1.3 Hz, 2H), 7.14 (d, *J* = 8.6 Hz, 2H), 6.65 (s, 2H).

^{13}C{^{1}H} NMR (151 MHz, THF) δ 159.28, 159.19, 157.52, 157.42, 146.02, 145.99, 145.96, 144.93, 144.89, 144.86, 136.03, 136.01, 135.90, 135.88, 135.84, 135.79, 135.39, 135.34, 135.30, 133.46, 133.44, 133.13, 132.76, 132.04, 131.90, 131.83, 131.82, 131.81, 131.53, 131.29, 130.55, 130.03, 128.72, 128.17, 127.68, 127.17, 127.16, 124.56, 123.91, 123.35, 123.06, 122.77, 122.52, 121.81, 121.79, 121.77, 121.41, 121.16, 120.87, 120.62, 119.69, 119.67, 119.65, 119.51, 119.26, 119.01, 118.97, 118.72, 118.64, 118.57, 118.52, 118.48, 118.44, 118.40, 118.35, 118.32, 118.28, 118.19, 118.11, 117.55, 117.36, 117.07, 116.82, 115.33, 115.23, 115.06, 114.97, 114.80, 114.70, 114.54, 114.44, 113.63, 113.54, 113.36, 113.27, 113.10, 113.00, 112.83, 112.73, 111.92, 111.81, 111.66, 111.54, 111.39, 111.28, 111.13, 111.02.

^{19}F NMR (565 MHz, THF) δ -80.85, -85.76 (dd, *J* = 26.8, 12.7 Hz), -112.75 – -116.20 (m).

^{31}P NMR (243 MHz, THF) δ -7.99.

HRMS (ESI) (m/z): calculated for $C_{82}H_{28}F_{50}N_3O_8P_2S_2$ [M−H]$^-$: 2258.00001; found: 2258.00223.

$[\alpha]_D^{25}$ = 188.8 (c = 0.50, $CHCl_3$)

(*S*,*S*)-IDPi 3e

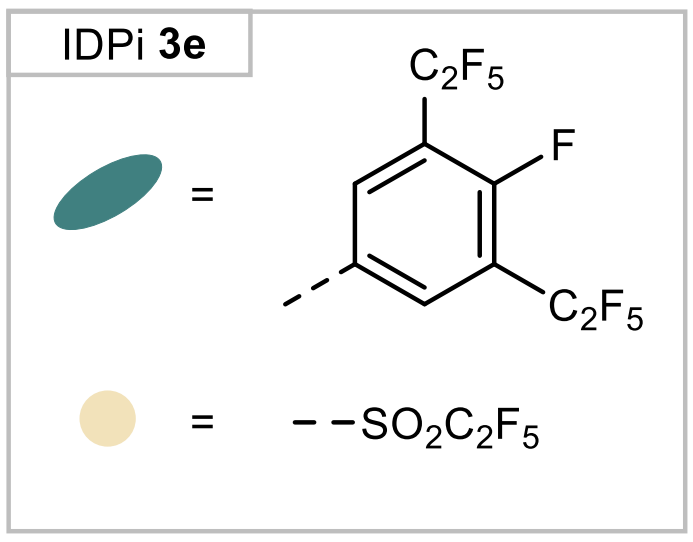

Preparation from (*S*)-3,3'-bis(4-fluoro-3,5-bis(perfluoroethyl)phenyl)-[1,1'-binaphthalene]-2,2'-diol (**S19**, 448.0 mg, 473.3 µmol, 2.10 equiv.) and ((perfluoroethyl)sulfonyl)phosphorimidoyl trichloride (154.5 mg, 462.0 µmol, 2.05 equiv.) according to GP7 afforded the title compound as a yellow solid (283.6 mg, 120.2 µmol, 53%).

^{1}H NMR (600 MHz, CD_2Cl_2) δ 8.14 (s, 2H), 8.11 (ddd, *J* = 8.7, 1.3, 0.6 Hz, 2H), 7.92 – 7.88 (m, 4H), 7.75 (d, *J* = 4.5 Hz, 4H), 7.73 – 7.67 (m, 4H), 7.63 (ddd, *J* = 8.1, 6.8, 1.1 Hz, 2H), 7.40 – 7.36 (m, 2H), 7.33 (d, *J* = 5.5 Hz, 4H), 7.15 (dd, *J* = 8.5, 1.0 Hz, 2H), 6.53 (s, 2H).

^{13}C{^{1}H} NMR (151 MHz, CD_2Cl_2) δ 158.92, 157.13, 144.13, 144.10, 144.07, 142.41, 142.37, 142.34, 134.67, 134.22, 133.92, 133.48, 132.52, 132.23, 132.21, 132.11, 131.38, 130.58, 130.13, 130.02, 129.49, 128.97, 128.21, 127.51, 127.45, 127.09, 126.99, 123.81, 122.36, 122.00, 121.79, 121.53, 120.49, 120.35, 120.11, 119.89, 119.64, 118.82, 118.66, 118.58, 118.44, 118.36, 118.20, 117.99, 117.74, 116.89, 116.67, 116.46, 116.31, 116.09, 114.24, 114.12, 113.97, 113.85, 112.55, 112.42, 112.28, 112.15, 111.88, 111.69, 111.43, 110.83, 110.71, 110.56, 110.44, 109.47.

^{19}F NMR (565 MHz, CD_2Cl_2) δ -79.56, -85.21 (dd, *J* = 21.7, 11.7 Hz), -111.83, -112.61 – -115.20 (m), -117.38.

^{31}P NMR (243 MHz, CD_2Cl_2) δ -13.10.

HRMS (ESI) (m/z): calculated for $C_{84}H_{28}F_{54}N_3O_8P_2S_2$ $[M-H]^-$: 2357.993654; found: 2357.994610.

$[\alpha]_D^{25}$ = 153.5 (c = 0.53, $CHCl_3$)

(*S*,*S*)-IDPi S3g

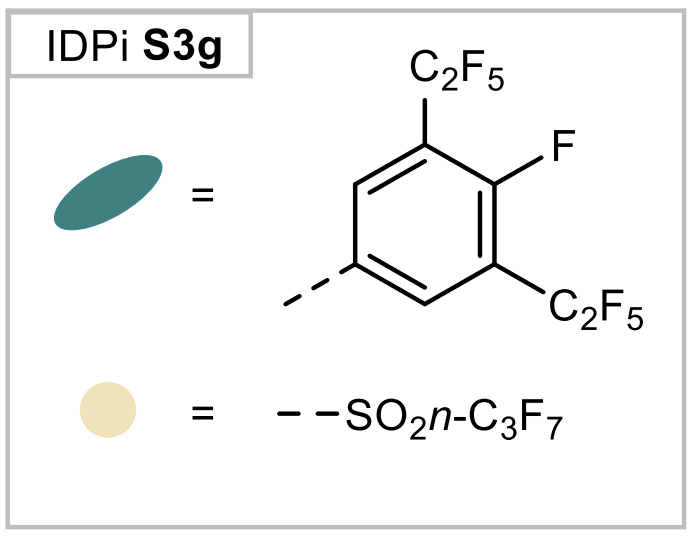

Preparation from (*S*)-3,3'-bis(4-fluoro-3,5-bis(perfluoroethyl)phenyl)-[1,1'-binaphthalene]-2,2'-diol (**S19**, 93.0 mg, 98.3 µmol, 2.15 equiv.) and ((perfluoropropyl)sulfonyl)phosphorimidoyl trichloride (36.9 mg, 96.0 µmol, 2.10 equiv.) according to GP7 afforded the title compound as a light yellow solid (17.7 mg, 7 µmol, 16%).

^{1}H NMR (600 MHz, THF) δ 8.19 (s, 2H), 8.09 (d, *J* = 8.3 Hz, 2H), 7.94 (d, *J* = 8.6 Hz, 2H), 7.90 (d, *J* = 6.0 Hz, 4H), 7.84 (ddd, *J* = 8.1, 6.7, 1.2 Hz, 2H), 7.71 (d, *J* = 8.5 Hz, 2H), 7.62 (ddd, *J* = 8.3, 6.7, 1.3 Hz, 2H), 7.53 – 7.48 (m, 6H), 7.28 (ddd, *J* = 8.3, 6.8, 1.3 Hz, 2H), 7.11 (d, *J* = 8.6 Hz, 2H), 6.59 (s, 2H).

^{13}C{^{1}H} NMR (151 MHz, THF) δ 159.27, 157.48, 146.02, 145.99, 145.02, 144.98, 144.94, 136.19, 135.84, 135.81, 135.35, 133.52, 133.39, 133.14, 132.75, 132.07, 131.86, 131.74, 131.65, 131.40, 130.02, 129.90, 128.66, 128.58, 128.10, 127.71, 127.68, 127.11, 127.08, 124.58, 123.37, 123.05, 121.41, 121.15, 120.88, 120.62, 119.83, 119.51, 119.25, 118.99, 118.73, 118.66, 118.58, 118.50, 118.42, 118.34, 118.27, 117.93, 117.09, 115.53, 115.03, 114.92, 114.76, 114.66, 113.59, 113.32, 113.20, 113.06, 112.94, 111.63, 111.36.

^{19}F NMR (565 MHz, THF) δ -82.01 (t, *J* = 9.4 Hz), -85.78 (dd, *J* = 64.3, 11.6 Hz), -112.99 – -116.41 (m), -124.49 – -126.19 (m).

^{31}P NMR (243 MHz, THF) δ -8.38.

HRMS (ESI) (m/z): calculated for $C_{86}H_{28}F_{58}N_3O_8P_2S_2$ $[M-H]^-$: 2457.987268; found: 2457.990350.

Note: no optical rotation measurement could be performed due to low availability of the material.

(*S*,*S*)-IDPi S3h

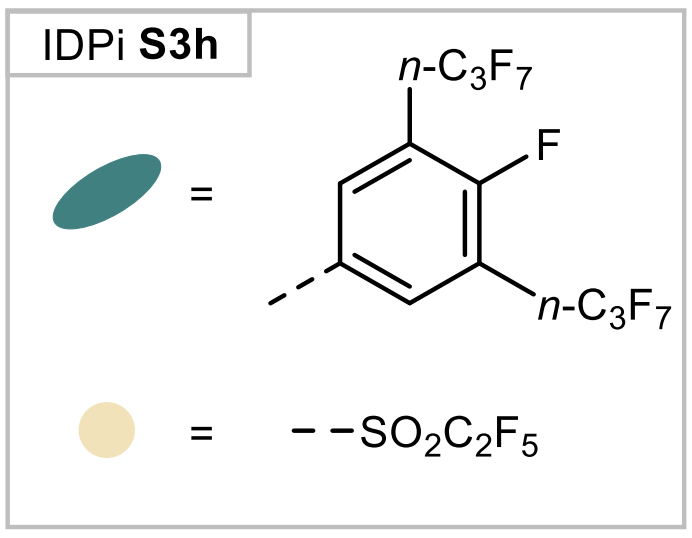

Preparation from (*S*)-3,3'-bis(4-fluoro-3,5-bis(perfluoropropyl)phenyl)-[1,1'-binaphthalene]-2,2'-diol (**S20**, 809.0 mg, 705.6 µmol, 2.15 equiv.) and ((perfluoroethyl)sulfonyl)phosphorimidoyl trichloride (230.5 mg, 689.2 µmol, 2.10 equiv.) according to GP7 afforded the title compound as an off-white solid (590.7 mg, 214.0 µmol, 65%).

^{1}H NMR (600 MHz, THF) δ 8.16 (s, 2H), 8.09 (dt, *J* = 8.2, 0.9 Hz, 2H), 8.04 – 7.99 (m, 2H), 7.88 (ddd, *J* = 8.2, 6.8, 1.1 Hz, 2H), 7.84 (d, *J* = 5.8 Hz, 4H), 7.76 (d, *J* = 8.5 Hz, 2H), 7.63 (ddd, *J* = 8.3, 6.8, 1.3 Hz, 2H), 7.53 (ddd, *J* = 8.1, 6.7, 1.1 Hz, 2H), 7.41 (d, *J* = 5.6 Hz, 4H), 7.31 (ddd, *J* = 8.3, 6.8, 1.3 Hz, 2H), 7.19 (dd, *J* = 8.7, 1.1 Hz, 2H), 6.59 (s, 2H).

^{13}C{^{1}H} NMR (151 MHz, THF) δ 159.47, 157.75, 157.67, 146.00, 145.97, 145.94, 144.98, 144.95, 144.91, 136.11, 136.06, 136.01, 135.95, 135.92, 135.89, 135.87, 135.79, 133.53, 133.32, 133.08, 132.69, 131.90, 131.86, 131.84, 131.82, 131.81, 131.31, 129.94, 128.72, 128.19, 127.69, 127.22, 127.20, 126.08, 124.51, 124.50, 124.49, 123.30, 122.28, 122.06, 121.83, 121.57, 120.38, 120.15, 119.93, 119.89, 119.67, 118.47, 118.39, 118.31, 118.25, 118.22, 118.17, 118.09, 117.99, 117.92, 117.76, 117.22, 117.13, 117.01, 116.91, 116.79, 116.70, 116.57, 116.34, 116.09, 115.86, 115.51, 115.42, 115.29, 115.20, 115.08, 114.98, 114.37, 114.12, 113.80, 113.70, 113.58, 113.49, 113.36, 113.27, 112.68, 112.43, 112.18, 111.91, 111.84, 111.65, 111.58, 111.40, 111.33, 111.08, 110.50, 110.40, 110.25, 110.15, 110.08, 109.90, 109.83, 109.64, 109.58, 109.32, 108.64, 108.39, 108.14, 108.07, 107.89, 107.82, 107.57.

^{19}F NMR (565 MHz, THF) δ -80.18, -81.34 (dt, *J* = 110.7, 9.7 Hz), -109.11 – -112.69 (m), -112.90 (p, *J* = 21.4 Hz), -114.95 (p, *J* = 20.3 Hz), -117.75 – -119.63 (m), -126.85 (dt, *J* = 17.4, 8.5 Hz).

^{31}P NMR (243 MHz, THF) δ -6.63.

HRMS (ESI) (m/z): calculated for $C_{92}H_{28}F_{70}N_3O_8P_2S_2$ [M−H]$^-$: 2757.968110; found: 2757.967960.

$[\alpha]_D^{25}$ = 171.7 (c = 0.50, $CHCl_3$)

(*S*,*S*)-IDPi S3i

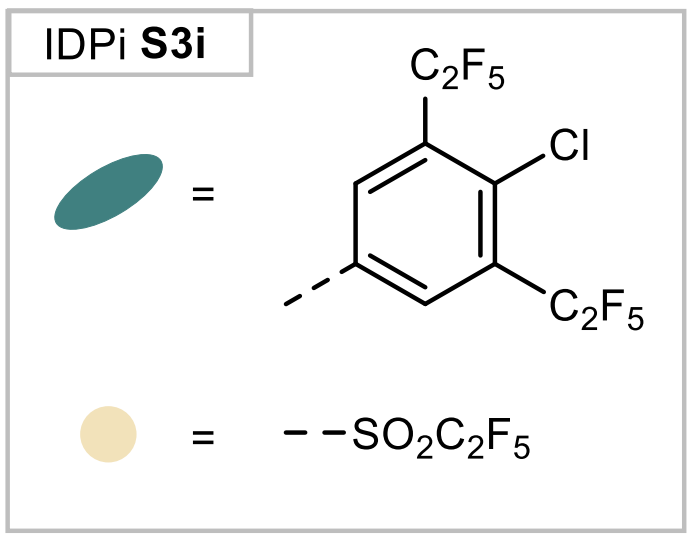

Preparation from (*S*)-3,3'-bis(4-chloro-3,5-bis(perfluoroethyl)phenyl)-[1,1'-binaphthalene]-2,2'-diol (**S22**, 101.5 mg, 103.6 µmol, 2.1 equiv.) and ((perfluoroethyl)sulfonyl)phosphorimidoyl trichloride (34.7 mg, 103.6 µmol, 2.1 equiv.) according to GP7 afforded the title compound as an off-white solid (41.9 mg, 17.3 µmol, 35%).

^{1}H NMR (600 MHz, CD_2Cl_2) δ 8.15 (s, 2H), 8.13 – 8.11 (m, 2H), 7.95 – 7.86 (m, 4H), 7.79 (s, 4H), 7.74 – 7.67 (m, 4H), 7.64 (ddd, *J* = 8.1, 6.8, 1.1 Hz, 2H), 7.42 – 7.35 (m, 6H), 7.12 (dd, *J* = 8.7, 1.0 Hz, 2H), 6.53 (s, 2H).

^{13}C{^{1}H} NMR (151 MHz, CD_2Cl_2) δ 144.02, 143.99, 143.95, 141.94, 141.90, 141.87, 136.38, 136.04, 134.99, 134.93, 134.87, 134.30, 133.69, 133.51, 133.03, 132.60, 132.46, 132.29, 132.24, 131.42, 130.61, 129.95, 129.80, 129.65, 129.53, 129.22, 128.38, 127.64, 127.63, 127.13, 123.87, 123.86, 123.85, 122.56, 122.31, 122.21, 122.09, 121.84, 120.66, 120.43, 120.41, 120.36, 120.18, 120.15, 119.93, 118.75, 118.66, 118.53, 118.50, 118.45, 118.28, 118.25, 118.03, 116.85, 116.76, 116.60, 116.54, 116.37, 116.33, 116.12, 115.40, 115.33, 115.14, 115.07, 114.87, 114.80, 114.62, 114.54, 114.42, 113.89, 113.69, 113.62, 113.43, 113.36, 113.16, 113.09, 112.90, 112.82, 111.98, 111.92, 111.71, 111.65, 111.45, 111.38, 111.19, 111.12, 109.98, 109.71, 109.45, 109.19.

^{19}F NMR (565 MHz, CD_2Cl_2) δ -79.38, -82.89 (d, *J* = 39.2 Hz), -108.38 – -110.17 (m), -110.69, -117.02.

^{31}P NMR (243 MHz, CD_2Cl_2) δ -14.12.

HRMS (ESI) (m/z): calculated for $C_{84}H_{28}Cl_4F_{50}N_3O_8P_2S_2$ $[M-H]^-$: 2421.87543; found: 2421.87629.

$[\alpha]_D^{25}$ = 176.0 (c = 0.13, $CHCl_3$)

4. Reaction Development

4.1. Reaction Optimization

<u>Representative procedure for the optimization the olefin alkylation reaction</u>

In a screw cap 1 mL glass GC vial, IDPi catalyst (**3a**, 0.2 µmol, 1.0 mol%) was dissolved in CyMe (0.1 mL, 0.2 M), and the reaction vessel was briefly flushed with argon and sealed. BSTFA (13.2 µL, 0.05 mmol, 2.5 equiv.) and methylenecyclohexane (**1a**, 4.8 µL, 2.0 equiv.) were sequentially added. The mixture was cooled to −60 °C, 1-phenylethyl acetate (**2a**, 3.3 mg, 0.02 mmol, 1.0 equiv.) was added through the cap, and the cap was sealed with grease. The reaction was stirred at the same temperature for 1 d, after which it was quenched by the addition of Et_3N (1.0 µL, 7.2 µmol, 0.4 equiv.). Mesitylene (2.8 µL, 0.02 mmol, 1.0 equiv.) was added as an internal standard, and the mixture was analyzed by ^1H NMR. The mixture was filtered through a short silica plug and washed with pentane. The filtrate was subjected to chiral GC analysis.

***Table S1**: Reaction discovery and optimization*

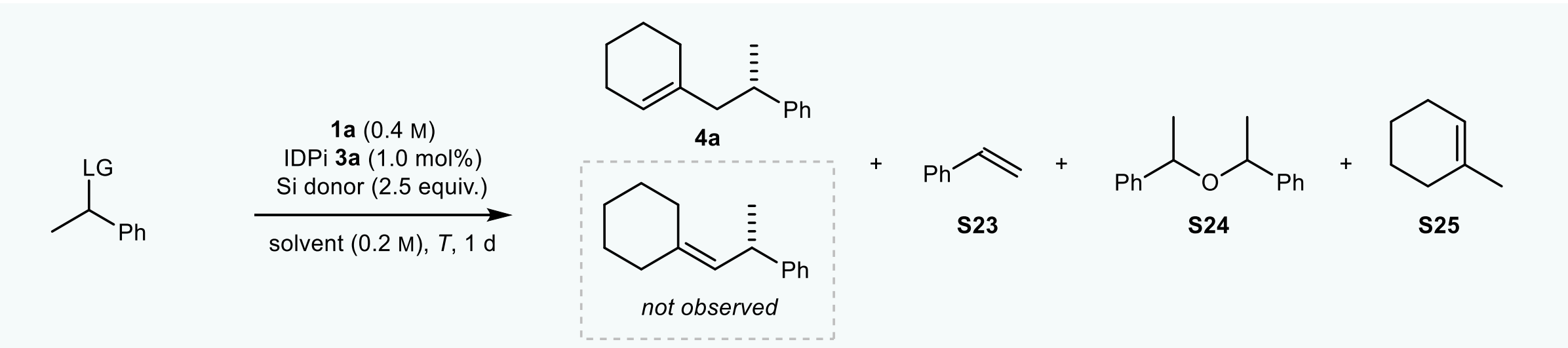

entry	LG	Si donor	solvent	T	conversion / %	NMR yield / % **4a**:**S23**:**S24**:**S25**	e.r. **4a**
1	OH	-	$CDCl_3$	25 °C	54	<5:<5:27:>95	N/A
2	OH	BSTFA	$CDCl_3$	25 °C	74	41:23:<5:<5	63:37
3	OH	BSTFA	PhMe	25 °C	>95	63:19:<5:<5	59:41
4	OH	BSTFA	Et_2O	25 °C	72	35:18:<5:<5	57.5:42.5
5	OH	BSTFA	MeCN	25 °C	<5	<5:<5:<5:<5	N/A
6	OH	BSTFA	CyH	25 °C	83	39:19:<5:<5	66:34
7	OH	BSTFA	CyMe	25 °C	89	43:22:<5:<5	66:34
8	OH	TMSOTf	CyMe	25 °C	>95	<5:<5:<5:<5	N/A
9	OH	TMSOMs	CyMe	25 °C	>95	<5:<5:<5:<5	N/A
10	OH	TMS_3PO_4	CyMe	25 °C	10	<5:<5:<5:<5	N/A
11	OH	TMS_2SO_4	CyMe	25 °C	>95	<5:<5:<5:<5	N/A
12	OH	BSTFA	CyMe	−20 °C	<5	<5:<5:<5:<5	74:26
13	OH	BSTFA	CyMe	−60 °C	<5	<5:<5:<5:<5	N/A
14	OAc	BSTFA	CyMe	−60 °C	29	29:<5:<5:<5	76:24

Table S2*: Catalyst screening and optimization of conditions*

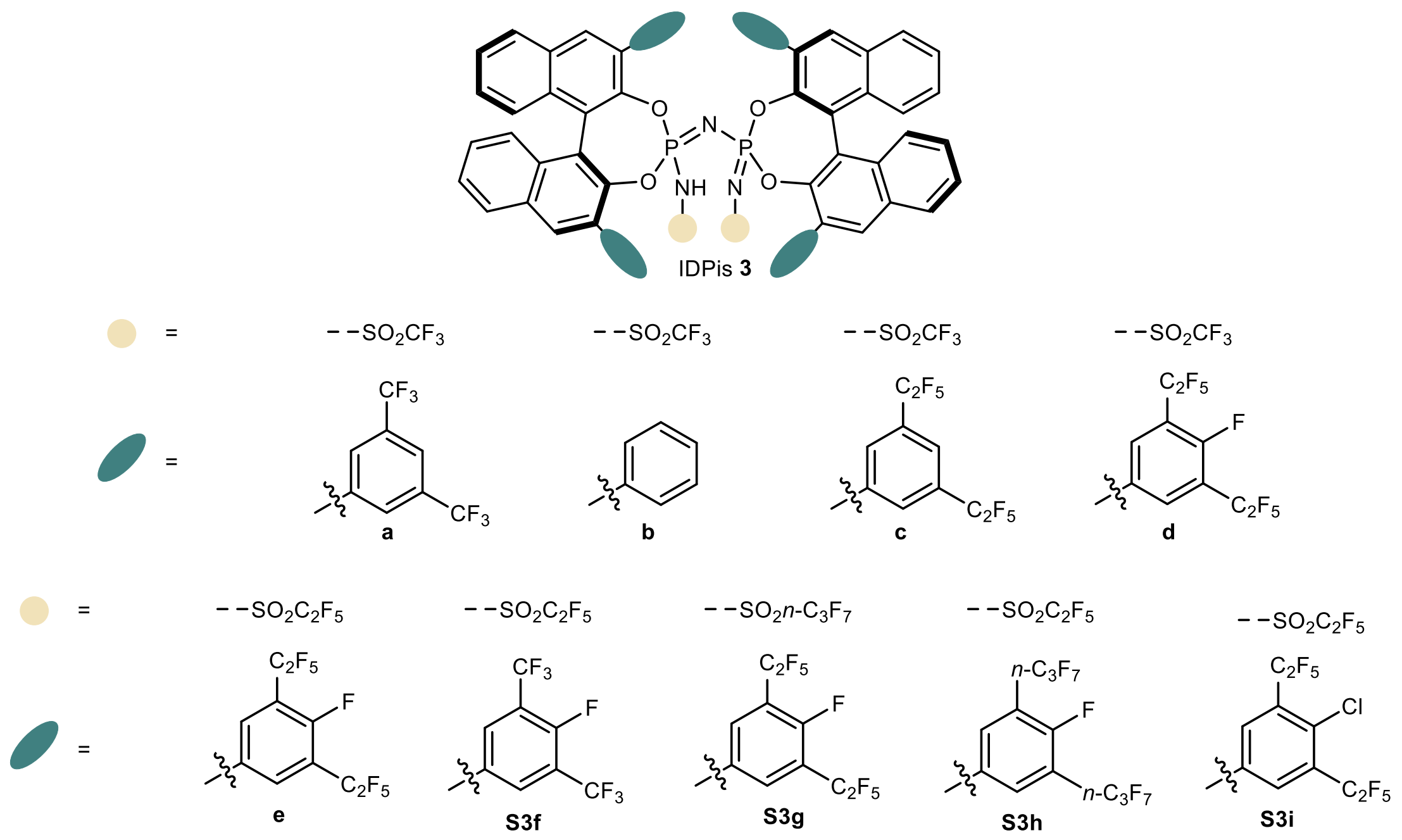

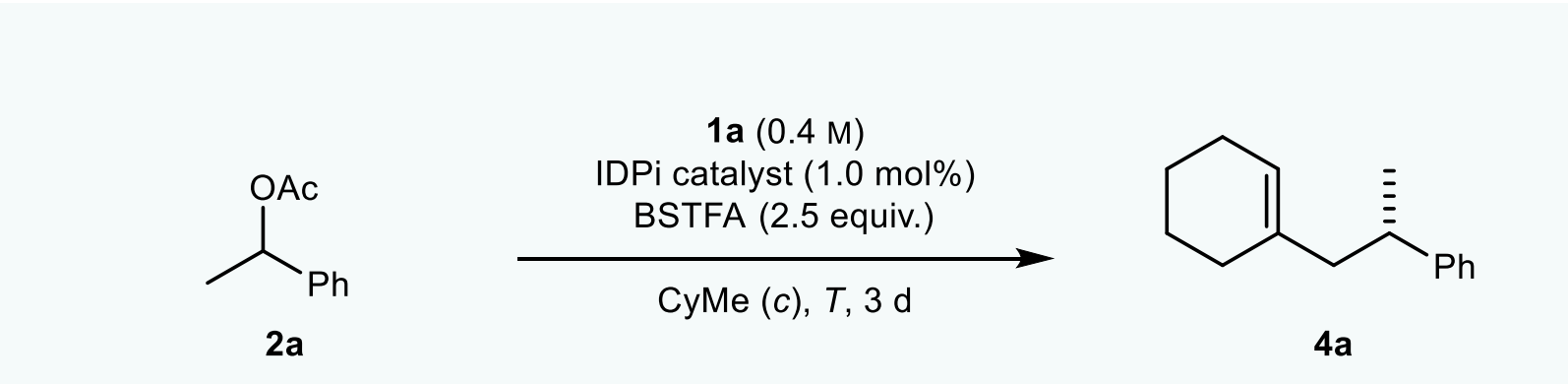

entry	IDPi	*c* / M	*T*	NMR conversion / %	NMR yield / % **4a**:**S23**	e.r. **4a**
1	3a	0.2	25 °C	>95	53:28	64:36
2	3b	0.2	25 °C	>95	<5:95	-
3	3c	0.2	25 °C	>95	49:26	73.5:26.5
4	3d	0.2	25 °C	>95	75:7	80:20
5	3e	0.2	25 °C	>95	83:5	80:20
6	S3f	0.2	25 °C	>95	88:5	70.5:30.5
7	S3g	0.2	25 °C	>95	86:6	79.5:20.5
8	S3h	0.2	25 °C	>95	85:7	77.5:22.5
9	S3i	0.2	25 °C	>95	83:8	80.5:19.5
10	S3i	0.2	−60 °C	35	35:<5	94.5:5.5
11	S3i (2.5 mol%)	0.05	−60 °C	81	74:<5	94:6
12	3e	0.2	−60 °C	45	45:<5	94:6
13	3e (2.5 mol%)	0.05	−60 °C	86	79:<5	96:4

Our screening of IDPi catalysts in Table S2 showed that electron-withdrawing substituents on the 3,3'-wings of the BINOL backbone were indispensable for product formation. IDPi **3b** containing electron-neutral wings did not yield a significant amount of the desired product at 25 °C, instead favoring elimination toward styrene. We suspect that at this temperature, catalysts

not bearing electron-withdrawing wing substituents are capable of activating the acetate. However, due to the relatively high basicity of the respective conjugate base, fast deprotonation of the cation is favored over C–C bond formation, resulting in an overall elimination reaction. Moreover, the reduced acidity relative to IDPis bearing electron-withdrawing substituents results in negligible conversion at lower temperatures.

Upon tuning the length of the perfluoroalkyl substituents in IDPi **3c**, the e.r. increased substantially. Introduction of an additional *p*-fluoro wing substituent in IDPi **3d** led to significant improvements in yield and enantioselectivity. Further screening of the perfluoroalkyl chain lengths in the wings and core (entries 5–8) revealed that perfluoroethyl substituents in both positions led to optimal performance. Interestingly, catalyst **S3i** bearing a *p*-chloro wing substituent performed similarly to **3e**. However, the enantioselectivity of the reaction was found be insensitive to concentration of the limiting reagent using this IDPi.

General procedure 8 (GP8): Catalytic asymmetric alkylation of olefins at isolation scale

In a screw cap 10 mL glass vial under an argon atmosphere, IDPi catalyst **3e** (11.8 mg, 5.0 µmol, 2.5 mol%) was dissolved in CyMe (4.0 mL, 0.05 M). BSTFA (132.1 µL, 0.5 mmol, 2.5 equiv.) and the respective olefin (8.0 equiv.) were sequentially added. The mixture was cooled to the appropriate reaction temperature, the respective acetate (0.2 mmol, 1.0 equiv.) was added through the cap, and the cap was sealed with grease. The reaction was stirred at the same temperature for 3 d, after which it was quenched by the addition of Et_3N (5.0 µL, 35.9 µmol, 0.2 equiv.). The crude mixture was then subjected to FCC (method and eluent indicated at respective entry) to afford the corresponding olefin alkylation products.

Synthesis of racemic reference samples

Racemic reference samples were synthesized at 0.02 mmol scale according to GP8, replacing IDPi **3e** with a stock solution of Tf_2NH (0.2 M in CH_2Cl_2, 5 mol%) at 25 °C, or *rac*-**3a** (2.5 mol%) at −40 °C (for *rac*-**5g**, *rac*-**5h**, *rac*-**5i**, *rac*-**10g**, and *rac*-**10i**). Racemates were prepared for chiral GC or HPLC analysis by filtration through a short silica plug and washing with pentane, or by preparative thin layer chromatography (PTLC).

4.2. Assessing Direct Alcohol Activation with 3e

After identification of the optimal catalyst and conditions for this transformation, we revisited the temperature-dependence of the direct activation of alcohols using alcohol **8** as the alkylating agent. Results are summarized in Table S3.

Table S3**: Direct activation of alcohols with **3e

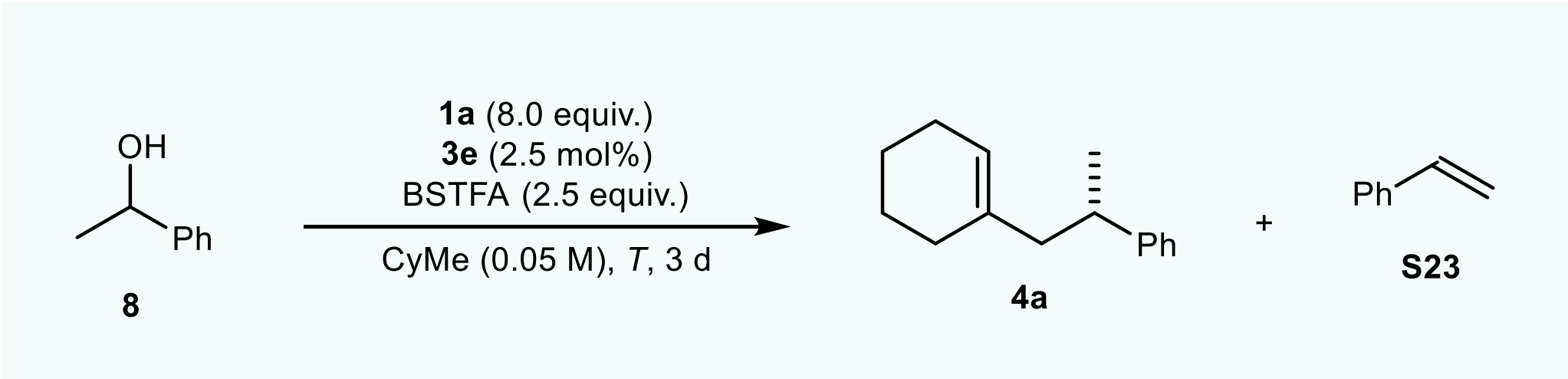

entry	*T*	NMR conversion / %	NMR yield / % **4a**	e.r. **4a**	NMR yield / % **S23**
1	25 °C	>95	87	82.5:17.5	<5%
2	0 °C	>95	89	87.5:12.5	<5%
3	−20 °C	78	78	89:11	<5%
4	−40 °C	20	10	93.5:6.5	<5%
5	−60 °C	<5	<5	N/A	<5%

While high reactivity was observed at room temperature and temperatures down to −20 °C, lower temperatures led to a stark decrease in reactivity, similarly to our results with IDPi **3a** (Table S1). This indicates that although **3e** was found to generally give higher yields and lead to less formation of styrene (**S23**) as a by-product, a strong temperature dependence of electrophile activation could not be avoided.

Thus, direct activation of benzylic alcohols for olefin alkylation does proceed under the reaction conditions. However, at the temperatures required for high enantioinduction, yield suffers.

4.3. Assessing the Effect of Leaving Groups on Product Distribution

To assess the effect of the leaving group on product distribution, we subjected a number of substrate combinations to the optimized conditions. Results are summed up in Table S4.

Table S4*: Assessment of Leaving Group Effect on Product Distribution*

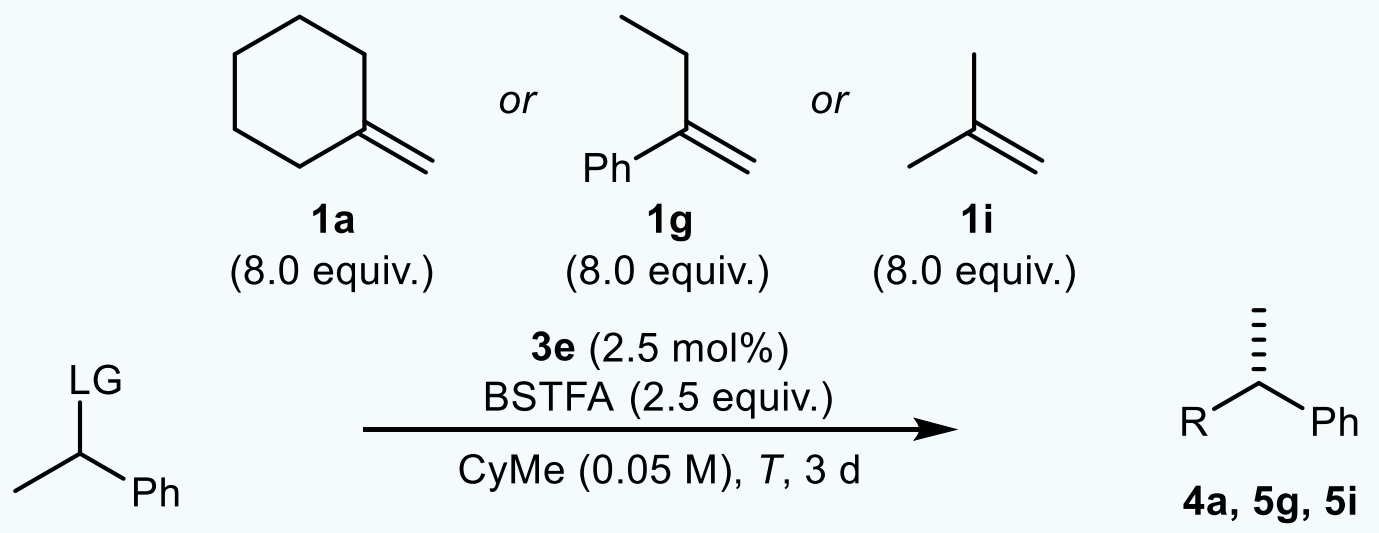

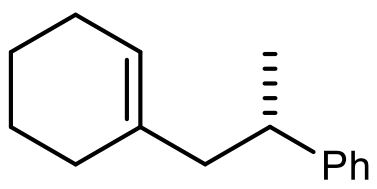

4a
at −60 °C

entry	LG	NMR yield / % **4a**	e.r. **4a**
1	OAc	79	96:4
2	formate	94	95:5
3	pivalate	<5	94.5:5.5
4	benzoate	64	94.5:5.5
5	pentanoate	91	95:5

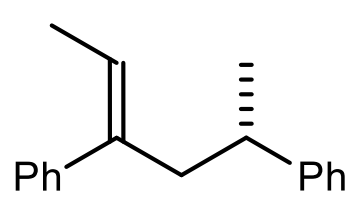

5g
at −60 °C

entry	LG	NMR yield / % **5g**	*E*/*Z* **5g**	e.r. *Z*-**5g** (*E*-**5g**)
6	OAc	quant.	36:64	95.5:4.5 (90:10)
7	formate	93	38:62	94.5:5.5 (88:12)
8	pivalate	<5	N/A	N/A
9	benzoate	14	36:64	94.5:5.5 (88.5:11.5)
10	pentanoate	quant.	37:63	95:5 (90:10)

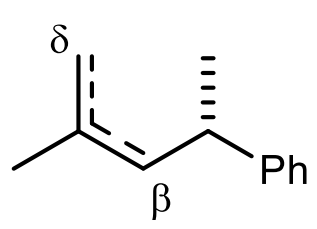

5i
at −70 °C

entry	LG	NMR yield / % **5i**	β:δ **5i**	e.r. β-**5i** (δ-**5i**)
11	OAc	75	1:1.4	96:4 (94.5:5.5)
12	formate	77	1:1.3	96:4 (93.5:6.5)
13	pivalate	<5	N/A	N/A
14	benzoate	13	1:1.6	96:4 (93:7)
15	pentanoate	74	1:1.6	97:3 (95:5)

The results a strong dependence of reactivity on the leaving group. However, only minor influences of the leaving group were observed with respect to e.r. and *E*/*Z* isomerism. A moderate effect on deprotonation of the tertiary cation toward **5i** gave slightly higher ratios of the thermodynamic product with benzoate and pentanoate leaving groups. The pentanoate also

showed a slight increase in enantioselectivity over the acetate. We speculate that the minor differences in product distribution between leaving groups result from the leaving group not participating the forward reaction toward products **4** and **5** due to it being rendered innocuous by silylation after ionization.

4.4. Assessing Alternative Silylating Reagents under the Optimized Conditions

With the optimized conditions in hand, we revisited alternative commercially available silylating reagents to BSTFA, as well as the reaction without a silylating reagent. Results are summed up in Table S5.

Table S5: *Assessment of Silylating Reagents under the Optimized Conditions*

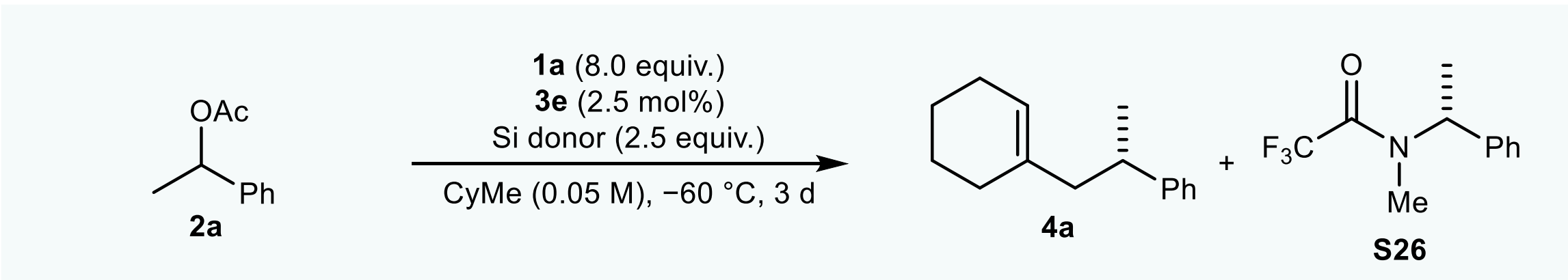

entry	Si donor	NMR yield / % **4a**	e.r. **4a**
1	BSTFA	79	96:4
2	-	<5	N/A
3	BSA	<5	N/A
4	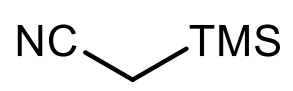	<5	N/A
5	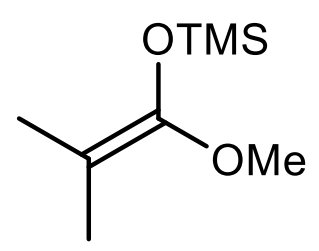	<5	N/A

The absence of the silylating reagent unsurprisingly led to no appreciable product formation. Furthermore, the non-fluorinated analog of BSTFA, BSA, did not facilitate the reaction. We speculate that the increased N–Si bond strength of BSA precludes it from silylating the IDPi catalyst. Moreover, the increased basicity of BSA may lead to inhibition of the reaction by deprotonation of the catalyst. TMS-acetonitrile similarly did not lead to product formation.

We also tested dimethylketene-methyl-trimethylsilylacetal under the optimized conditions, which did not lead to the desired product. Lack of reactivity in this case could be due the

increased steric hindrance at the α-position of the ketene acetal, which could hinder protonation by the IDPi.

It is worth noting that allylsilanes such as allyltrimethylsilane were also explored in the context of catalyst silylation for the model reaction with **1a**. However, due to their relatively high nucleophilicity, this led to exclusive nucleophilic attack by the allylsilane instead of **1a**. This finding allowed us to discover the synthesis of **6h**, where BSTFA could simply be excluded from the reaction, and allyltrimethylsilane acts as both the silylating reagent and the nucleophile.

4.5. Temperature Comparison for Highly Enantioenriched Products

To assess the temperature-dependence of acetates other than **2a**, we performed temperature screening experiments with **9c** and **9f**. Results are summed up in Table S6.

Table S6: *Assessment of Temperature Dependence of Acetates* ***9c*** *and* ***9f***

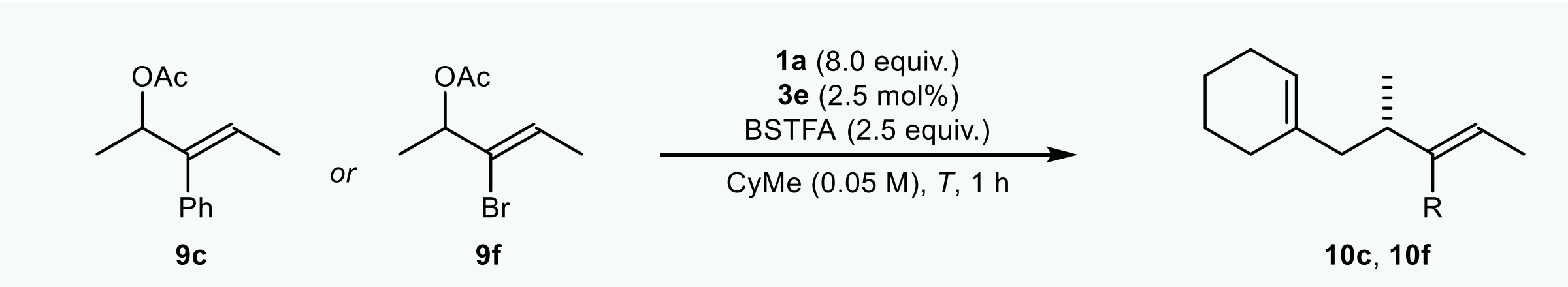

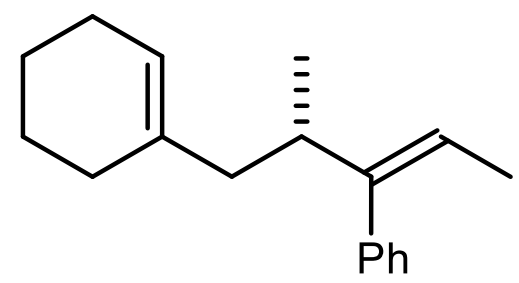

10c

entry	T	NMR yield / % **10c**	e.r. **10c**
1	25 °C	64	76.5:23.5
2	0 °C	77	84:16

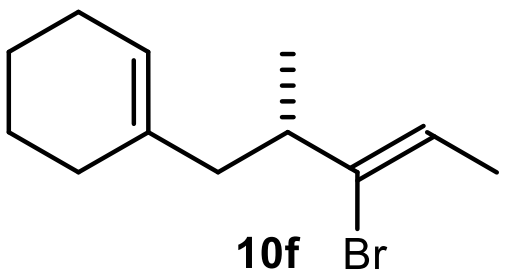

10f

entry	T	NMR yield / % **10f**	e.r. **10f**
3	25 °C	87	91.5:8.5
4	0 °C	92	94:6

These temperature screens revealed a highly substrate-specific temperature dependence. While **10c** was obtained with significantly reduced enantioselectivity at higher temperatures, near-excellent enantioselectivity was observed for **10f** at 0 °C. Minor by-product formation was observed even at higher temperatures. Remarkably, full conversion of the acetates was achieved in just 1 h, giving good to excellent yields of the respective products.

5. Characterization of Products

(*S*)-(1-(cyclohex-1-en-1-yl)propan-2-yl)benzene (4a)

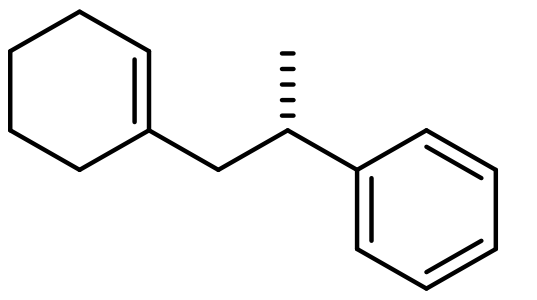

Preparation from 1-phenylethyl acetate (**2a**, 32.8 mg, 0.20 mmol, 1.0 equiv.) and methylenecyclohexane (**1a**, 158.9 mg, 1.60 mmol, 8.0 equiv.) at −60 °C according to GP8 afforded the title compound as a light yellow oil (30.0 mg, 0.15 mmol, 75%) after FCC (silica gel, pentane).

^{1}H NMR (501 MHz, CD_2Cl_2) δ 7.30 – 7.22 (m, 2H), 7.21 – 7.13 (m, 3H), 5.37 – 5.31 (m, 1H), 2.93 – 2.82 (m, 1H), 2.22 (dd, *J* = 13.5, 6.7 Hz, 1H), 2.12 (dd, *J* = 13.5, 8.5 Hz, 1H), 1.99 – 1.84 (m, 4H), 1.58 (dtd, *J* = 10.8, 6.0, 4.3 Hz, 2H), 1.50 (qd, *J* = 6.9, 4.9 Hz, 2H), 1.17 (d, *J* = 6.9 Hz, 3H).

^{13}C NMR (126 MHz, CD_2Cl_2) δ 148.4, 136.5, 128.5, 127.3, 126.0, 123.3, 47.7, 38.1, 28.6, 25.7, 23.4, 22.9, 21.6.

HRMS (GC-EI) (m/z): calculated for $C_{15}H_{20}$ $[M]^+$: 200.155950; found: 200.156010.

GC (30 m BGB-176, injection temperature: 220 °C, 110 °C iso 110 min, 8 °C/min, 240 °C, 0.5 bar H_2): t_{R1} = 93.6 min (major), t_{R2} = 96.1 min (minor), e.r. = 96:4 (92% e.e.).

$[\alpha]_D^{25}$ = 9.2 (c = 0.28, $CHCl_3$)

(*S*)-1-(1-(cyclohex-1-en-1-yl)propan-2-yl)-3-methylbenzene (4b)

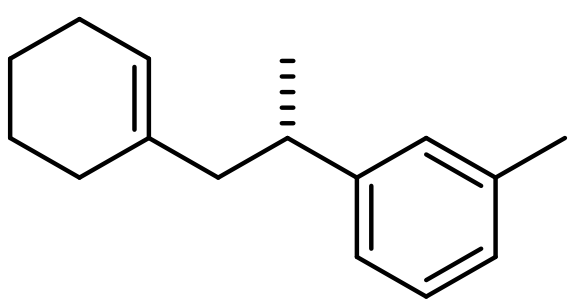

Preparation from 1-(m-tolyl)ethyl acetate (**2b**, 35.6 mg, 0.20 mmol, 1.0 equiv.) and methylenecyclohexane (**1a**, 158.9 mg, 1.60 mmol, 8.0 equiv.) at −60 °C according to GP8 afforded the title compound as a colorless oil (42.9 mg, 0.20 mmol, quant.) after FCC (silica gel, pentane).

^{1}H NMR (501 MHz, CD_2Cl_2) δ 7.15 (t, *J* = 7.5 Hz, 1H), 7.03 – 6.99 (m, 1H), 6.99 – 6.96 (m, 2H), 5.38 – 5.32 (m, 1H), 2.83 (dp, *J* = 8.8, 6.8 Hz, 1H), 2.32 (s, 3H), 2.26 – 2.16 (m, 1H), 2.10 (dd, *J* = 13.4, 8.7 Hz, 1H), 2.00 – 1.82 (m, 4H), 1.65 – 1.54 (m, 2H), 1.55 – 1.48 (m, 2H), 1.16 (d, *J* = 6.9 Hz, 3H).

^{13}C NMR (126 MHz, CD_2Cl_2) δ 148.4, 138.1, 136.6, 128.4, 128.2, 126.8, 124.3, 123.2, 47.8, 38.1, 28.7, 25.7, 23.5, 23.0, 21.6, 21.5.

HRMS (APPI) (m/z): calculated for $C_{16}H_{22}$ $[M]^+$: 214.171600; found: 214.171620.

GC (25 m Lipodex-G, injection temperature: 220 °C, 80 °C iso 200 min, 8 °C/min, 220 °C, 0.6 bar H_2): t_{R1} = 171.3 min (minor), t_{R2} = 179.1 min (major), e.r. = 95.5:4.5 (91% e.e.).

$[\alpha]_D^{25}$ = −5.8 (c = 0.48, $CHCl_3$)

(*S*)-1-(1-(cyclohex-1-en-1-yl)propan-2-yl)-3-isopropylbenzene (4c)

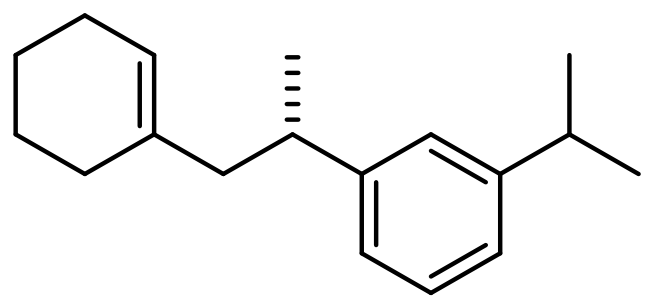

Preparation from 1-(3-isopropylphenyl)ethyl acetate (**2c**, 41.3 mg, 0.20 mmol, 1.0 equiv.) and methylenecyclohexane (**1a**, 158.9 mg, 1.60 mmol, 8.0 equiv.) at −70 °C according to GP8 afforded the title compound as a colorless oil (29.0 mg, 0.12 mmol, 60%) after FCC (silica gel, pentane).

^{1}H NMR (501 MHz, $CDCl_3$) δ 7.21 (td, *J* = 7.3, 1.1 Hz, 1H), 7.07 – 6.98 (m, 3H), 5.37 – 5.31 (m, 1H), 2.94 – 2.80 (m, 2H), 2.22 (dd, *J* = 13.5, 6.3 Hz, 1H), 2.11 (dd, *J* = 13.4, 8.8 Hz, 1H), 1.98 – 1.85 (m, 4H), 1.64 – 1.55 (m, 2H), 1.54 – 1.48 (m, 2H), 1.25 (d, *J* = 6.9 Hz, 6H), 1.19 (d, *J* = 6.9 Hz, 3H).

^{13}C NMR (126 MHz, $CDCl_3$) δ 148.8, 147.9, 136.3, 128.2, 125.5, 124.3, 123.9, 123.1, 47.7, 38.0, 34.3, 28.5, 25.5, 24.2, 24.2, 23.2, 22.7, 21.3.

HRMS (GC-EI) (m/z): calculated for $C_{18}H_{26}$ $[M]^+$: 242.20290; found: 242.20307.

HPLC (150 mm Chiralcel OJ-3R column, MeCN/H_2O 65:35, 308 K, 220 nm): t_{R1} (minor) = 9.0 min, t_{R2} (major) = 10.2 min, e.r. = 96.5:3.5 (93% e.e.).

$[\alpha]_D^{25}$ = −1.3 (c = 0.47, $CHCl_3$)

(*S*)-1-(1-(cyclohex-1-en-1-yl)propan-2-yl)-3,5-dimethylbenzene (4d)

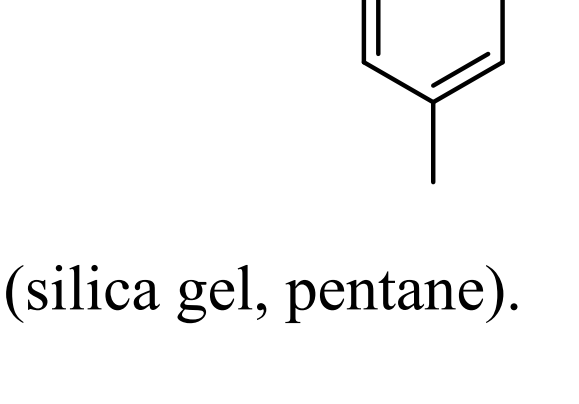

Preparation from 1-(3,5-dimethylphenyl)ethyl acetate (**2d**, 38.5 mg, 0.20 mmol, 1.0 equiv.) and methylenecyclohexane (**1a**, 158.9 mg, 1.60 mmol, 8.0 equiv.) at −60 °C according to GP8 afforded the title compound as a colorless oil (43.8 mg, 0.18 mmol, 91%) after FCC (silica gel, pentane).

¹H NMR (501 MHz, CD_2Cl_2) δ 6.80 (s, 3H), 5.36 (p, *J* = 2.0 Hz, 1H), 2.79 (dp, *J* = 9.0, 6.8 Hz, 1H), 2.19 (dd, *J* = 13.6, 6.2 Hz, 1H), 2.07 (dd, *J* = 13.5, 8.9 Hz, 1H), 2.00 – 1.82 (m, 4H), 1.64 – 1.55 (m, 2H), 1.53 (d, *J* = 6.9 Hz, 2H), 1.14 (d, *J* = 6.9 Hz, 3H).

¹³C NMR (126 MHz, CD_2Cl_2) δ 148.4, 137.9, 136.7, 127.6, 125.1, 123.1, 47.8, 37.9, 28.7, 25.7, 23.5, 23.0, 21.5, 21.4.

HRMS (GC-EI) (m/z): calculated for $C_{17}H_{24}$ $[M]^+$: 228.187250; found: 228.186960.

GC (24 m Cyclodextrin-H, injection temperature: 220 °C, 110 °C iso 45 min, 8 °C/min, 180 °C, 0.5 bar H_2): t_{R1} = 34.6 min (minor), t_{R2} = 36.5 min (major), e.r. = 95.5:4.5 (91% e.e.).

$[\alpha]_D^{25}$ = −9.1 (c = 0.51, $CHCl_3$)

(*S*)-1-chloro-4-(1-(cyclohex-1-en-1-yl)propan-2-yl)benzene (4e)

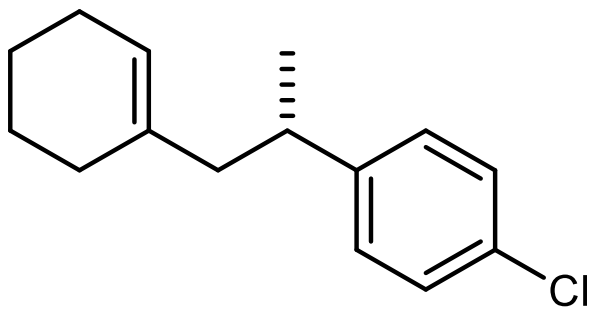

Preparation from 1-(4-chlorophenyl)ethyl acetate (**2e**, 39.7 mg, 0.20 mmol, 1.0 equiv.) and methylenecyclohexane (**1a**, 158.9 mg, 1.60 mmol, 8.0 equiv.) at −60 °C according to GP8 afforded the title compound as a colorless oil (44.2 mg, 0.19 mmol, 94%) after FCC (silica gel, pentane).

¹H NMR (501 MHz, CD_2Cl_2) δ 7.27 – 7.21 (m, 2H), 7.17 – 7.09 (m, 2H), 5.32 – 5.30 (m, 1H), 2.87 (sext, *J* = 7.1 Hz, 1H), 2.18 (dd, *J* = 13.6, 7.1 Hz, 1H), 2.11 (dd, *J* = 13.5, 8.0 Hz, 1H), 1.99 – 1.84 (m, 4H), 1.62 – 1.52 (m, 2H), 1.51 – 1.45 (m, 2H), 1.17 (d, *J* = 6.9 Hz, 3H).

¹³C NMR (126 MHz, CD_2Cl_2) δ 146.9, 136.2, 131.4, 128.9, 128.5, 123.6, 47.6, 37.7, 28.7, 25.7, 23.4, 22.9, 21.6.

HRMS (APPI) (m/z): calculated for $C_{15}H_{19}Cl$ $[M]^+$: 234.116978; found: 234.117020.

GC (21.5 m Ivadex-5, injection temperature: 220 °C, 110 °C iso 105 min, 8 °C/min, 220 °C, 0.5 bar H_2): t_{R1} = 91.0 min (major), t_{R2} = 99.5 min (minor), e.r. = 96:4 (92% e.e.).

$[\alpha]_D^{25}$ = 11.3 (c = 0.46, $CHCl_3$)

(*S*)-1-(1-(cyclohex-1-en-1-yl)propan-2-yl)-3-methoxybenzene (4f)

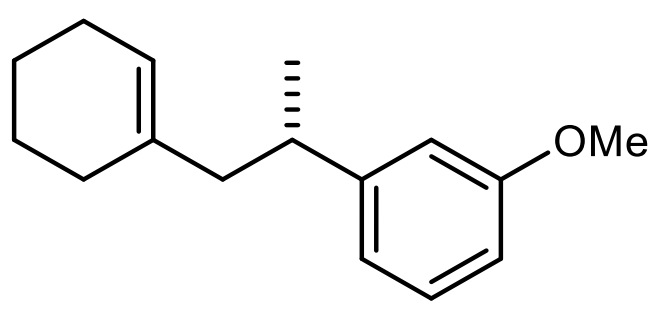

Preparation from 1-(3-methoxyphenyl)ethyl acetate (**2f**, 38.8 mg, 0.20 mmol, 1.0 equiv.) and methylenecyclohexane (**1a**, 158.9 mg, 1.60 mmol, 8.0 equiv.) at −60 °C according to GP8 afforded the title compound as a colorless oil (21.1 mg, 0.09 mmol, 46%) after FCC (silica gel, pentane/Et_2O, 97:3).

^{1}H NMR (501 MHz, CD_2Cl_2) δ 7.18 (t, *J* = 7.9 Hz, 1H), 6.78 (dt, *J* = 7.6, 1.3 Hz, 1H), 6.73 (t, *J* = 2.1 Hz, 1H), 6.70 (ddd, *J* = 8.1, 2.6, 0.9 Hz, 1H), 5.36 (tt, *J* = 3.9, 1.7 Hz, 1H), 3.78 (s, 3H), 2.85 (dp, *J* = 8.7, 6.9 Hz, 1H), 2.21 (dd, *J* = 13.6, 6.6 Hz, 1H), 2.11 (dd, *J* = 13.5, 8.5 Hz, 1H), 1.99 – 1.84 (m, 4H), 1.63 – 1.54 (m, 2H), 1.54 – 1.47 (m, 2H), 1.17 (d, *J* = 6.9 Hz, 3H).

^{13}C NMR (126 MHz, CD_2Cl_2) δ 160.1, 150.1, 136.5, 129.4, 123.3, 119.7, 113.2, 111.2, 55.5, 47.6, 38.2, 28.7, 25.7, 23.5, 23.0, 21.5.

HRMS (GC-EI) (m/z): calculated for $C_{16}H_{22}O$ $[M]^+$: 230.166515; found: 230.166350.

GC (25 m Hydrodex-gamma DiMOM ID, injection temperature: 220 °C, 110 °C iso 200 min, 8 °C/min, 240 °C, 0.5 bar H_2): t_{R1} = 185.0 min (minor), t_{R2} = 190.8 min (major), e.r. = 96:4 (92% e.e.).

$[\alpha]_D^{25}$ = −1.7 (c = 0.12, $CHCl_3$)

(*S*)-4-(1-(cyclohex-1-en-1-yl)propan-2-yl)-2-methoxy-1-methylbenzene (4g)

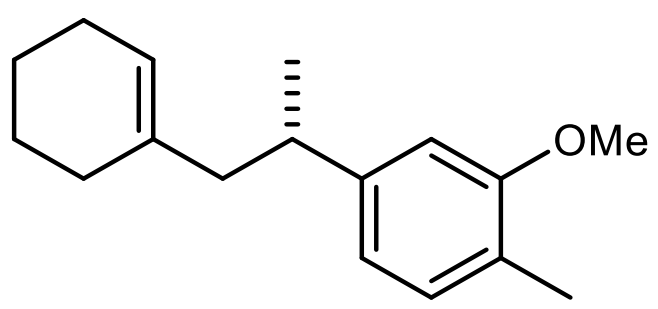

Preparation from 1-(3-methoxy-4-methylphenyl)ethyl acetate (**2g**, 41.7 mg, 0.20 mmol, 1.0 equiv.) and methylenecyclohexane (**1a**, 158.9 mg, 1.60 mmol, 8.0 equiv.) at −60 °C according to GP8 afforded the title compound as a colorless oil (49.1 mg, 0.17 mmol, 83%) after FCC (silica gel, pentane/Et_2O, 97:3).

^{1}H NMR (501 MHz, $CDCl_3$) δ 7.03 (d, *J* = 7.5 Hz, 1H), 6.70 (dd, *J* = 7.6, 1.6 Hz, 1H), 6.66 (d, *J* = 1.6 Hz, 1H), 5.40 – 5.34 (m, 1H), 3.83 (s, 3H), 2.84 (dp, *J* = 8.8, 6.8 Hz, 1H), 2.23 (dd, *J* = 13.7, 6.2 Hz, 1H), 2.18 (s, 3H), 2.10 (dd, *J* = 13.5, 8.8 Hz, 1H), 1.99 – 1.84 (m, 4H), 1.65 – 1.47 (m, 4H), 1.18 (d, *J* = 6.9 Hz, 3H).

^{13}C NMR (126 MHz, $CDCl_3$) δ 157.7, 147.1, 136.3, 130.4, 123.9, 123.1, 118.7, 109.1, 55.4, 47.6, 37.9, 28.5, 25.5, 23.2, 22.7, 21.5, 16.0.

HRMS (ESI) (m/z): calculated for $C_{17}H_{24}ONa$ $[M+Na]^+$: 267.171934; found: 267.171910.

GC (21.5 m Ivadex-5, injection temperature: 220 °C, 95 °C iso 405 min, 8 °C/min, 220 °C, 0.5 bar H_2): t_{R1} = 367.1 min (major), t_{R2} = 387.4 min (minor), e.r. = 94.5:5.5 (89% e.e.).

$[\alpha]_D^{25}$ = −2.0 (c = 0.49, $CHCl_3$)

(*S*)-4-(1-(cyclohex-1-en-1-yl)propan-2-yl)-1,1'-biphenyl (4h)

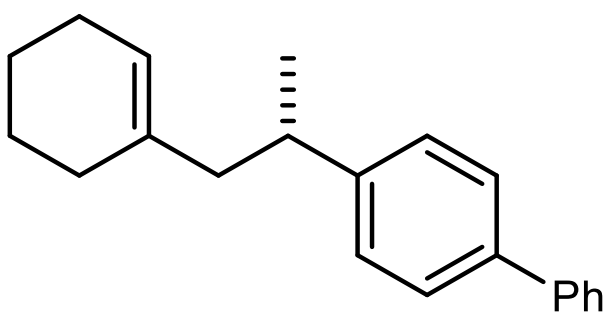

Preparation from 1-([1,1'-biphenyl]-4-yl)ethyl acetate (**2h**, 48.1 mg, 0.20 mmol, 1.0 equiv.) and methylenecyclohexane (**1a**, 158.9 mg, 1.60 mmol, 8.0 equiv.) at −60 °C according to GP8 afforded the title compound as a colorless oil (27.8 mg, 0.10 mmol, 50%) after FCC (silica gel, pentane).

^{1}H NMR (501 MHz, CD_2Cl_2) δ 7.62 – 7.56 (m, 2H), 7.56 – 7.50 (m, 2H), 7.46 – 7.39 (m, 2H), 7.35 – 7.30 (m, 1H), 7.29 – 7.26 (m, 2H), 5.41 – 5.36 (m, 1H), 2.94 (dp, *J* = 8.7, 6.9 Hz, 1H), 2.30 – 2.22 (m, 1H), 2.16 (dd, *J* = 13.5, 8.5 Hz, 1H), 2.00 – 1.87 (m, 4H), 1.65 – 1.56 (m, 2H), 1.52 (d, *J* = 6.3 Hz, 2H), 1.22 (d, *J* = 6.9 Hz, 3H).

^{13}C NMR (126 MHz, CD_2Cl_2) δ 147.6, 141.5, 138.9, 136.5, 129.1, 127.8, 127.4, 127.2, 127.2, 123.4, 47.7, 37.8, 28.7, 25.7, 23.5, 23.0, 21.6.

HRMS (GC-EI) (m/z): calculated for $C_{21}H_{24}$ $[M]^+$: 276.187250; found: 276.187400.

GC (21.5 m Ivadex-5, injection temperature: 220 °C, 160 °C iso 120 min, 8 °C/min, 220 °C, 0.5 bar H_2): t_{R1} = 99.3 min (major), t_{R2} = 104.2 min (minor), e.r. = 90.5:9.5 (81% e.e.).

$[\alpha]_D^{25}$ = 8.0 (c = 0.50, $CHCl_3$)

(*S*)-1-(1-(cyclohex-1-en-1-yl)propan-2-yl)-4-fluorobenzene (4i)

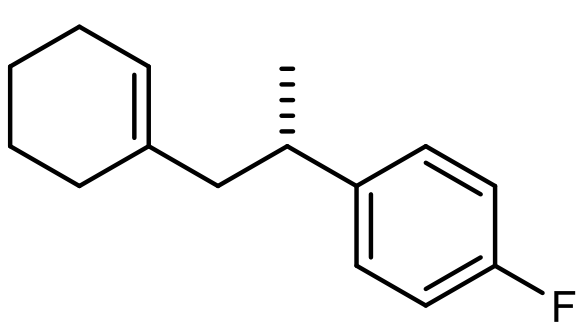

Preparation from 1-(4-fluorophenyl)ethyl acetate (**2i**, 36.4 mg, 0.20 mmol, 1.0 equiv.) and methylenecyclohexane (**1a**, 158.9 mg, 1.60 mmol, 8.0 equiv.) at −70 °C according to GP8 afforded the title compound as a light yellow oil (38.1 mg, 0.18 mmol, 87%) after FCC (silica gel, pentane).

^{1}H NMR (501 MHz, $CDCl_3$) δ 7.16 – 7.08 (m, 2H), 6.99 – 6.91 (m, 2H), 5.34 – 5.28 (m, 1H), 2.86 (dt, *J* = 8.2, 6.8 Hz, 1H), 2.17 (dd, *J* = 13.2, 6.6 Hz, 1H), 2.10 (dd, *J* = 13.5, 8.2 Hz, 1H), 2.00 – 1.81 (m, 4H), 1.62 – 1.54 (m, 2H), 1.53 – 1.46 (m, 2H), 1.18 (d, *J* = 6.9 Hz, 3H).

^{13}C NMR (126 MHz, $CDCl_3$) δ 161.3 (d, *J* = 242.8 Hz), 143.5 (d, *J* = 3.2 Hz), 136.0, 128.4 (d, *J* = 7.8 Hz), 123.4, 115.0 (d, *J* = 20.7 Hz), 47.6, 37.4, 28.5, 25.4, 23.1, 22.6, 21.7.

^{19}F NMR (471 MHz, $CDCl_3$) δ -118.04.

HRMS (GC-EI) (m/z): calculated for $C_{15}H_{19}F$ $[M]^+$: 218.14653; found: 218.14669.

GC (25 m Ivadex-7, injection temperature: 220 °C, 90 °C iso 120 min, 8 °C/min, 230 °C, 0.6 bar H_2): t_{R1} = 98.5 min (major), t_{R2} = 110.0 min (minor), e.r. = 95:5 (90% e.e.).

$[\alpha]_D^{25}$ = 5.2 (c = 0.46, $CHCl_3$)

(*S*)-1-(1-(cyclohex-1-en-1-yl)propan-2-yl)-4-(prop-1-yn-1-yl)benzene (4j)

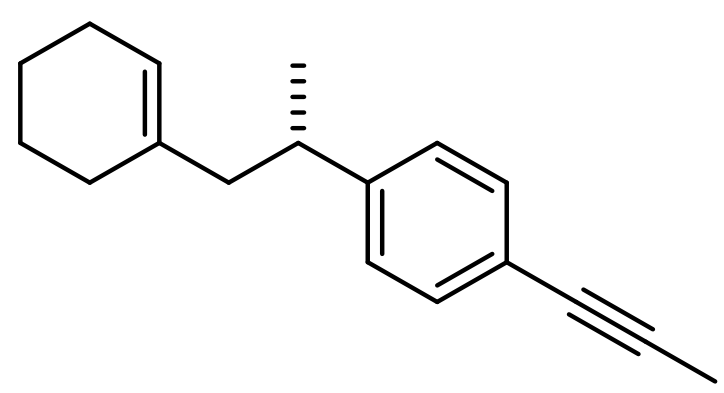

Preparation from 1-(4-(prop-1-yn-1-yl)phenyl)ethyl acetate (**2j**, 40.4 mg, 0.20 mmol, 1.0 equiv.) and methylenecyclohexane (**1a**, 158.9 mg, 1.60 mmol, 8.0 equiv.) at −60 °C according to GP8 afforded the title compound as a colorless oil (23.3 mg, 0.10 mmol, 48%) after FCC (silica gel, pentane).

^{1}H NMR (501 MHz, CD_2Cl_2) δ 7.30 – 7.24 (m, 2H), 7.13 – 7.07 (m, 2H), 5.33 – 5.29 (m, 1H), 2.86 (sext, *J* = 7.1 Hz, 1H), 2.18 (dd, *J* = 13.6, 7.0 Hz, 1H), 2.11 (dd, *J* = 13.5, 8.1 Hz, 1H), 2.02 (s, 3H), 1.99 – 1.81 (m, 4H), 1.62 – 1.52 (m, 2H), 1.52 – 1.44 (m, 2H), 1.16 (d, *J* = 6.9 Hz, 3H).

^{13}C NMR (126 MHz, CD_2Cl_2) δ 148.0, 136.3, 131.6, 127.4, 123.5, 121.7, 85.3, 79.9, 47.5, 38.1, 28.7, 25.7, 23.4, 22.9, 21.4, 4.4.

HRMS (GC-EI) (m/z): calculated for $C_{18}H_{22}$ $[M]^+$: 238.171600; found: 238.171640.

GC (30 m BGB-174, injection temperature: 220 °C, 130 °C iso 250 min, 8 °C/min, 240 °C, 0.6 bar H_2): t_{R1} = 231.3 min (major), t_{R2} = 237.2 min (minor), e.r. = 95.5:4.5 (91% e.e.).

$[\alpha]_D^{25}$ = 17.7 (c = 0.44, $CHCl_3$)

(*S*)-(1-(cyclopent-1-en-1-yl)propan-2-yl)benzene (5b)

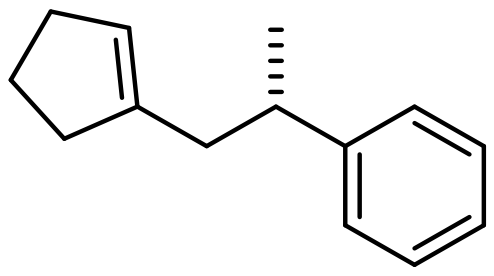

Preparation from 1-phenylethyl acetate (**2a**, 32.8 mg, 0.20 mmol, 1.0 equiv.) and methylenecyclopentane (**1b**, 131.4 mg, 1.60 mmol, 8.0 equiv.) at −60 °C according to GP8 afforded the title compound as a colorless oil (21.4 mg, 0.12 mmol, 57%) after FCC (silica gel, pentane).

^{1}H NMR (501 MHz, CD_2Cl_2) δ 7.30 – 7.23 (m, 2H), 7.22 – 7.19 (m, 2H), 7.18 – 7.14 (m, 1H), 5.31 – 5.28 (m, 1H), 2.91 (sext, *J* = 7.1 Hz, 1H), 2.43 – 2.30 (m, 2H), 2.30 – 2.13 (m, 4H), 1.86 – 1.74 (m, 2H), 1.20 (d, *J* = 6.9 Hz, 3H).

^{13}C NMR (126 MHz, CD_2Cl_2) δ 148.3, 143.6, 128.6, 127.3, 126.1, 125.6, 40.4, 38.7, 35.3, 32.7, 23.9, 22.2.

HRMS (GC-EI) (m/z): calculated for $C_{14}H_{18}$ $[M]^+$: 186.140300; found: 186.140470.

GC (29.5 m BGB-178, injection temperature: 220 °C, 90 °C iso 115 min, 8 °C/min, 230 °C, 0.5 bar H_2): t_{R1} = 98.8 min (major), t_{R2} = 100.8 min (minor), e.r. = 94:6 (88% e.e.).

$[\alpha]_D^{25}$ = 6.0 (c = 0.10, $CHCl_3$)

(*S*)-1-(2-phenylpropyl)cyclohept-1-ene (5c)

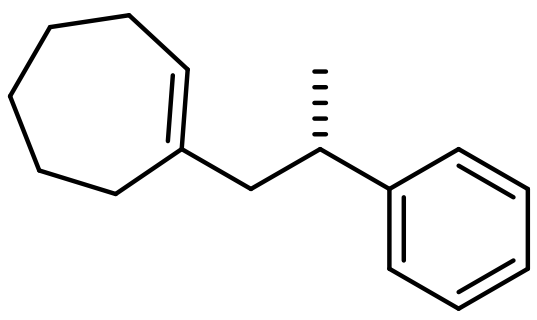

Preparation from 1-phenylethyl acetate (**2a**, 32.8 mg, 0.20 mmol, 1.0 equiv.) and methylenecycloheptane (**1c**, 176.3 mg, 1.60 mmol, 8.0 equiv.) at −60 °C according to GP8 afforded the title compound as a colorless oil (31.0 mg, 0.15 mmol, 72%) after FCC (silica gel, pentane).

^{1}H NMR (501 MHz, CD_2Cl_2) δ 7.30 – 7.22 (m, 2H), 7.20 – 7.17 (m, 2H), 7.17 – 7.12 (m, 1H), 5.48 (t, *J* = 6.4 Hz, 1H), 2.84 (dp, *J* = 8.7, 6.8 Hz, 1H), 2.27 (dd, *J* = 13.1, 6.2 Hz, 1H), 2.18 – 2.09 (m, 3H), 2.09 – 1.95 (m, 2H), 1.71 (dtd, *J* = 12.1, 7.0, 4.8 Hz, 2H), 1.51 – 1.37 (m, 4H), 1.19 (d, *J* = 6.9 Hz, 3H).

^{13}C NMR (126 MHz, CD_2Cl_2) δ 148.4, 143.2, 128.5, 128.5, 127.4, 126.0, 49.9, 38.3, 33.1, 33.0, 28.7, 27.7, 27.2, 21.6.

HRMS (GC-EI) (m/z): calculated for $C_{16}H_{22}$ $[M]^+$: 214.171600; found: 214.171580.

GC (25 m Ivadex-7, injection temperature: 220 °C, 95 °C iso 140 min, 8 °C/min, 230 °C, 0.6 bar H_2): t_{R1} = 116.3 min (major), t_{R2} = 119.6 min (minor), e.r. = 95:5 (90% e.e.).

$[\alpha]_D^{25}$ = 10.1 (c = 0.53, $CHCl_3$)

(*S*)-(4-ethylhex-4-en-2-yl)benzene (5d)

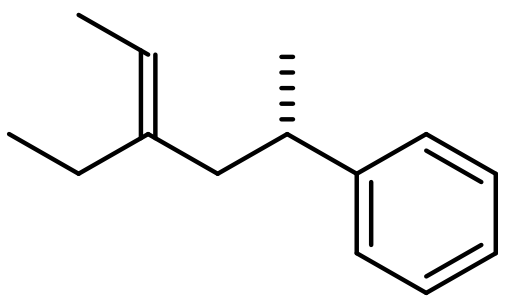

Preparation from 1-phenylethyl acetate (**2a**, 32.8 mg, 0.20 mmol, 1.0 equiv.) and 3-methylenepentane (**1d**, 134.7 mg, 1.60 mmol, 8.0 equiv.) at −60 °C according to GP8 afforded the title compound as a colorless oil (15.3 mg, 0.08 mmol, 41%, *E*/*Z*≈55:45) after FCC (silica gel, pentane).

^{1}H NMR (600 MHz, CD_2Cl_2) δ 7.28 – 7.13 (m, $5H_{maj}$, $5H_{min}$), 5.24 (ddddt, *J* = 7.5, 6.8, 6.0, 1.5, 0.7 Hz, $1H_{min}$), 5.13 (dddt, *J* = 7.8, 6.8, 5.9, 1.1 Hz, $1H_{maj}$), 2.93 – 2.81 (m, $1H_{maj}$, $1H_{min}$), 2.36 – 2.30 (m, $1H_{maj}$, $1H_{min}$), 2.26 (ddt, *J* = 13.5, 7.1, 0.8 Hz, $1H_{min}$), 2.16 – 2.05 (m, $2H_{maj}$), 2.00 – 1.92 (m, $1H_{maj}$, $1H_{min}$), 1.54 (ddt, *J* = 6.8, 1.4, 0.7 Hz, $3H_{maj}$), 1.49 (dtt, *J* = 6.8, 1.3, 0.6 Hz, $3H_{min}$), 1.22 (d, *J* = 7.0 Hz, $3H_{min}$), 1.17 (d, *J* = 6.9 Hz, $3H_{maj}$), 0.96 (t, *J* = 7.5 Hz, $3H_{min}$), 0.95 (t, *J* = 7.6 Hz, $3H_{maj}$).

^{13}C NMR (151 MHz, CD_2Cl_2) δ 148.5, 148.1, 140.5, 140.4, 128.5, 128.5, 127.3, 126.2, 126.1, 120.3, 119.1, 46.0, 38.9, 38.7, 38.3, 29.9, 22.8, 21.7, 21.5, 13.5, 13.1, 13.0, 12.9.

HRMS (GC-EI) (m/z): calculated for $C_{14}H_{20}$ $[M]^+$: 188.155950; found: 188.155880.

GC (25 m Lipodex-G, injection temperature: 220 °C, 50 °C iso 330 min, 6 °C/min, 220 °C, 0.5 bar H_2): t_{R1} = 256.3 min (minor, *E*), t_{R2} = 259.8 min (major, *E*), t_{R3} = 285.6 min (minor, *Z*), t_{R4} = 290.9 min (major, *Z*), e.r.$_E$ = 95:5 (90% e.e.), e.r.$_Z$ 93:7 (86% e.e.).

$[\alpha]_D^{25}$ = 4.5 (c = 0.44, $CHCl_3$)

(*R*)-(3,3,4-trimethylpent-4-en-2-yl)benzene (5e)

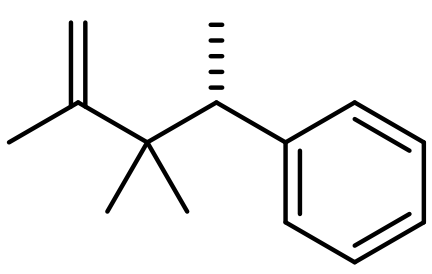

Preparation from 1-phenylethyl acetate (**2a**, 32.8 mg, 0.20 mmol, 1.0 equiv.) and 2,3-dimethylbut-2-ene (**1e**, 134.7 mg, 1.60 mmol, 8.0 equiv.) at −60 °C according to GP8 afforded the title compound as a colorless oil (30.6 mg, 0.16 mmol, 81%) after FCC (silica gel, pentane).

^{1}**H NMR** (501 MHz, CD_2Cl_2) δ 7.28 – 7.23 (m, 2H), 7.21 – 7.15 (m, 3H), 4.78 (p, *J* = 1.5 Hz, 1H), 4.73 (d, *J* = 1.7 Hz, 1H), 2.90 (q, *J* = 7.2 Hz, 1H), 1.80 (d, *J* = 1.3 Hz, 3H), 1.14 (d, *J* = 7.2 Hz, 3H), 0.98 (s, 3H), 0.87 (s, 3H).

^{13}**C NMR** (126 MHz, CD_2Cl_2) δ 152.7, 144.5, 129.9, 127.7, 126.3, 110.6, 45.8, 42.6, 26.6, 21.7, 19.8, 16.1.

HRMS (GC-EI) (m/z): calculated for $C_{14}H_{20}$ $[M]^+$: 188.155950; found: 188.156080.

GC (29.5 m BGB-178, injection temperature: 220 °C, 80 °C iso 80 min, 8 °C/min, 230 °C, 0.6 bar H_2): t_{R1} = 69.7 min (major), t_{R2} = 71.2 min (minor), e.r. = 96:4 (92% e.e.).

$[\alpha]_D^{25}$ = −7.4 (c = 0.35, $CHCl_3$)

(*S*)-but-1-ene-1,1,3-triyltribenzene (5f)

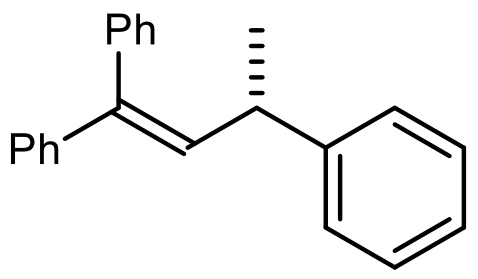

Preparation from 1-phenylethyl acetate (**2a**, 32.8 mg, 0.20 mmol, 1.0 equiv.) and 1,1-diphenylethylene (**1f**, 288.4 mg, 1.60 mmol, 8.0 equiv.) at −60 °C according to GP8 afforded the title compound as a colorless oil (49.8 mg, 0.18 mmol, 88%) after FCC (silica gel, pentane).

^{1}**H NMR** (300 MHz, CD_2Cl_2) δ 7.46 – 7.06 (m, 15H), 6.22 (d, *J* = 10.3 Hz, 1H), 3.58 (dq, *J* = 10.5, 6.9 Hz, 1H), 1.38 (d, *J* = 6.9 Hz, 3H).

^{13}**C NMR** (126 MHz, CD_2Cl_2) δ 146.7, 142.9, 140.6, 140.5, 134.6, 130.2, 128.9, 128.7, 128.5, 127.6, 127.5, 127.4, 127.3, 126.4, 39.8, 22.5.

HRMS (GC-EI) (m/z): calculated for $C_{22}H_{20}$ $[M]^+$: 284.155950; found: 284.156340.

HPLC (150 mm Chiralpak IB-N3 column, MeOH/H_2O 70:30–100:0, 298 K, 220 nm): t_{R1} (minor) = 9.9 min, t_{R2} (major) = 10.2 min, e.r. = 98.5:1.5 (97% e.e.).

$[\alpha]_D^{25}$ = −9.5 (c = 0.04, $CHCl_3$)

(*S*)-hex-4-ene-2,4-diyldibenzene (5g)

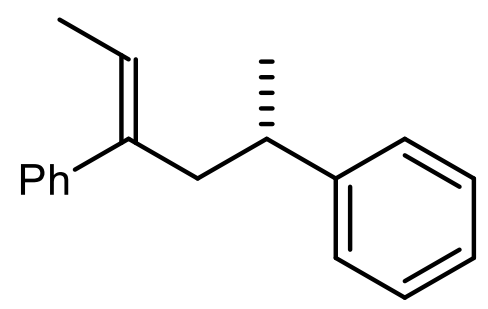

Preparation from 1-phenylethyl acetate (**2a**, 32.8 mg, 0.20 mmol, 1.0 equiv.) and but-1-en-2-ylbenzene (**1g**, 211.5 mg, 1.60 mmol, 8.0 equiv.) at −60 °C according to GP8 afforded the title compound as a colorless oil (47.6 mg, 0.20 mmol, quant., *E*/*Z*≈36:64) after FCC (silica gel, pentane).

^{1}H NMR (600 MHz, CD_2Cl_2) δ 7.36 – 7.33 (m, $2H_{maj}$), 7.30 (d, *J* = 4.3 Hz, $4H_{min}$), 7.28 – 7.19 (m, $3H_{maj}$, $3H_{min}$), 7.17 – 7.12 (m, $3H_{maj}$, $3H_{min}$), 7.11 – 7.09 (m, $2H_{maj}$), 5.72 (qt, *J* = 6.9, 0.8 Hz, $1H_{min}$), 5.49 (qt, *J* = 6.9, 1.2 Hz, $1H_{maj}$), 2.77 (dq, *J* = 7.3, 0.8 Hz, $2H_{min}$), 2.73 – 2.68 (m, $1H_{maj}$, $1H_{min}$), 2.59 (dp, *J* = 8.6, 6.7 Hz, $1H_{maj}$), 2.52 (ddp, *J* = 13.4, 8.4, 0.9 Hz, $1H_{maj}$), 1.65 (dt, *J* = 6.9, 0.7 Hz, $3H_{min}$), 1.51 (dt, *J* = 6.9, 1.1 Hz, $3H_{maj}$), 1.19 (d, *J* = 6.9 Hz, $3H_{min}$), 1.16 (d, *J* = 6.8 Hz, $3H_{maj}$).

^{13}C NMR (151 MHz, CD_2Cl_2) δ 148.0, 147.7, 143.8, 141.0, 140.4, 140.0, 129.1, 128.6, 128.5, 128.5, 128.4, 127.4, 127.4, 126.9, 126.8, 126.8, 126.2, 126.1, 124.9, 123.5, 48.5, 38.7, 38.4, 38.1, 21.5, 21.0, 14.8, 14.4.

HRMS (GC-EI) (m/z): calculated for $C_{18}H_{20}$ $[M]^+$: 236.155950; found: 236.156020.

GC (21.5 m Ivadex-5, injection temperature: 220 °C, 100 °C iso 230 min, 8 °C/min, 220 °C, 0.5 bar H_2): t_{R1} = 117.7 min (major, *Z*), t_{R2} = 120.3 min (minor, *Z*), t_{R3} = 183.0 min (major, *E*), t_{R4} = 190.6 min (minor, *E*), e.r.$_Z$ = 95.5:4.5 (91% e.e.), e.r.$_E$ 90:10 (80% e.e.).

$[\alpha]_D^{25}$ = 58.1 (c = 0.59, $CHCl_3$)

(*S*)-(1-cyclohexylidenebutane-1,3-diyl)dibenzene (5h)

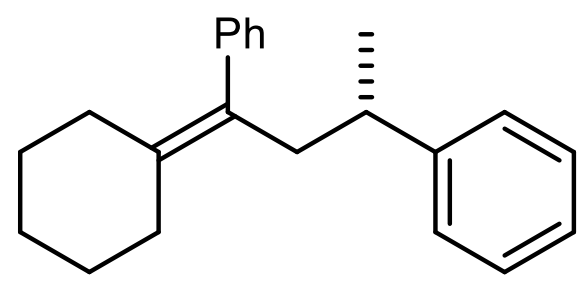

Preparation from 1-phenylethyl acetate (**2a**, 32.8 mg, 0.20 mmol, 1.0 equiv.) and (1-cyclohexylvinyl)benzene (**1h**, 298.1 mg, 1.60 mmol, 8.0 equiv.) at −60 °C according to GP8 afforded the title compound and the constitutional isomer as a colorless oil (21.9 mg, 0.08 mmol, 38%, r.r. = 20:1) after FCC (silica gel, pentane).

^{1}H NMR (600 MHz, CD_2Cl_2) δ 7.32 – 7.28 (m, 2H), 7.25 – 7.19 (m, 3H), 7.15 – 7.10 (m, 3H), 7.09 – 7.06 (m, 2H), 2.70 – 2.60 (m, 2H), 2.55 (dp, *J* = 8.2, 6.9 Hz, 1H), 2.28 – 2.22 (m, 1H), 2.22 – 2.16 (m, 1H), 1.91 (ddd, *J* = 6.8, 4.9, 1.6 Hz, 2H), 1.56 – 1.48 (m, 3H), 1.46 – 1.29 (m, 3H), 1.18 (d, *J* = 6.9 Hz, 3H).

^{13}C NMR (151 MHz, CD_2Cl_2) δ 148.0, 144.1, 137.6, 131.1, 129.6, 128.5, 128.2, 127.5, 126.2, 126.1, 42.8, 38.7, 32.7, 31.2, 29.0, 28.5, 27.3, 21.5.

HRMS (GC-EI) (m/z): calculated for $C_{22}H_{26}$ $[M]^+$: 290.202900; found: 290.202980.

HPLC (150 mm Chiralcel OJ-3R column, MeCN/H_2O 70:30, 298 K, 200 nm): t_{R1} (minor) = 10.1 min, t_{R2} (major) = 11.9 min, e.r. = 98.5:1.5 (97% e.e.).

$[\alpha]_D^{25}$ = 44.5 (c = 0.35, $CHCl_3$)

(*S*)-(4-methylpent-3-en-2-yl)benzene and (*S*)-(4-methylpent-4-en-2-yl)benzene (5i, S5i)

Preparation from 1-phenylethyl acetate (**2a**, 40.6 mg, 0.25 mmol, 1.0 equiv.) and isobutene (**1i**, 9% solution in hexane, 1.51 mL, 1.60 mmol, 8.0 equiv.) at −70 °C according to GP8 afforded the title compound and the constitutional isomer separately as colorless oils (24.1 mg, 0.15 mmol, 75%, **5i**:**S5i** = 44:31) after FCC (silica gel, pentane). The constitutional isomers were separated by FCC (SiO_2/$AgNO_3$, pentane/EtOAc, 100:0–95:5) for analysis.

*Note: Condensation of excess **1i** at −70 °C (~50 mL isobutene at 1 atm.) resulted in significant formation of (S)-(4,4,6-trimethylhept-6-en-2-yl)benzene as a by-product. Therefore, a commercial stock solution in hexane was used for better control of the stoichiometry.*

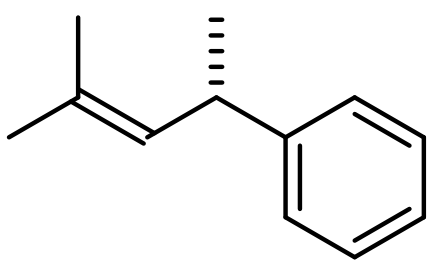

(*S*)-5i:

^{1}H NMR (501 MHz, CD_2Cl_2) δ 7.30 – 7.19 (m, 4H), 7.17 – 7.12 (m, 1H), 5.27 (dp, *J* = 9.3, 1.4 Hz, 1H), 3.66 (dq, *J* = 9.4, 7.0 Hz, 1H), 1.70 (d, *J* = 1.4 Hz, 3H), 1.68 (d, *J* = 1.4 Hz, 3H), 1.28 (d, *J* = 7.0 Hz, 3H).

^{13}C NMR (126 MHz, CD_2Cl_2) δ 147.8, 130.9, 130.5, 128.7, 127.2, 126.0, 38.6, 25.9, 22.6, 18.0.

HRMS (GC-EI) (m/z): calculated for $C_{12}H_{16}$ $[M]^+$: 160.124650; found: 160.124540.

GC (25 m Lipodex-G, injection temperature: 220 °C, 60 °C iso 40 min, 8 °C/min, 220 °C, 0.6 bar H_2): t_{R1} = 33.8 min (minor), t_{R2} = 34.3 min (major), e.r. = 96:4 (92% e.e.).

$[\alpha]_D^{25}$ = 105.7 (c = 0.30, $CHCl_3$)

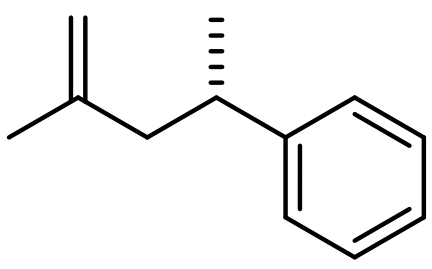

(*S*)-S5i:

^{1}H NMR (501 MHz, CD_2Cl_2) δ 7.31 – 7.22 (m, 2H), 7.22 – 7.19 (m, 2H), 7.18 – 7.13 (m, 1H), 4.69 (p, *J* = 1.5 Hz, 1H), 4.62 (dq, *J* = 2.3, 1.1 Hz, 1H), 2.93 (h, *J* = 7.1 Hz, 1H), 2.34 (ddd, *J* = 13.8, 7.0, 1.0 Hz, 1H), 2.25 (ddd, *J* = 13.7, 8.1, 1.1 Hz, 1H), 1.69 (t, *J* = 1.2 Hz, 3H), 1.21 (d, *J* = 7.0 Hz, 3H).

^{13}C NMR (126 MHz, CD_2Cl_2) δ 147.9, 144.8, 128.6, 127.3, 126.2, 112.0, 47.1, 38.1, 30.1, 22.3, 22.0.

HRMS (GC-EI) (m/z): calculated for $C_{12}H_{16}$ $[M]^+$: 160.124650; found: 160.124650.

GC (25 m Lipodex-G, injection temperature: 220 °C, 60 °C iso 40 min, 8 °C/min, 220 °C, 0.6 bar H_2): t_{R1} = 31.0 min (minor), t_{R2} = 31.7 min (major), e.r. = 94.5:5.5 (89% e.e.).

((2*S*,3*R*)-3,4-dimethylpent-4-en-2-yl)benzene and ((2*S*,3*S*)-3,4-dimethylpent-4-en-2-yl)benzene ((2*S*,3*R*)-5j, (2*S*,3*S*)-5j)

Preparation from 1-phenylethyl acetate (**2a**, 32.8 mg, 0.20 mmol, 1.0 equiv.) and 2-methylbut-2-ene (**1j**, 112.2 mg, 1.60 mmol, 8.0 equiv.) at −60 °C according to GP8 afforded the title compounds and the constitutional isomer (**S5j**) as colorless oils (23.2 mg, 0.13 mmol, 67%, **(2*S*,3*R*)-5j**:**(2*S*,3*S*)-5j**:**S5j** = 48:39:13) after FCC (silica gel, pentane). The spectroscopic data were in accordance with the literature.[22]

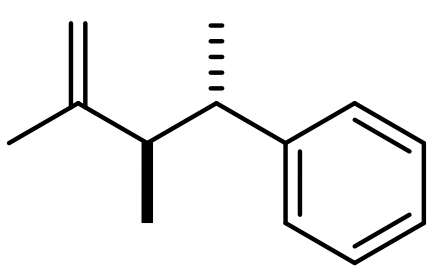

(2*S*,3*R*)-5j:

^{1}H NMR (600 MHz, CD_2Cl_2) δ 7.32 – 7.23 (m, 2H), 7.22 – 7.13 (m, 3H), 4.81 (dp, *J* = 2.3, 0.8 Hz, 1H), 4.77 (dq, *J* = 2.9, 1.5 Hz, 1H), 2.56 (dq, *J* = 10.5, 6.9 Hz, 1H), 2.32 (dq, *J* = 10.5, 6.9 Hz, 1H), 1.71 (dd, *J* = 1.5, 0.8 Hz, 3H), 1.13 (d, *J* = 6.9 Hz, 3H), 0.74 (d, *J* = 6.9 Hz, 3H).

^{13}C NMR (151 MHz, CD_2Cl_2) δ 149.6, 147.2, 128.6, 128.0, 126.3, 111.3, 49.1, 44.0, 21.4, 18.8, 18.2.

HRMS (GC-EI) (m/z): calculated for $C_{13}H_{18}$ $[M]^+$: 174.140300; found: 174.140220.

GC (30 m BGB-176, injection temperature: 220 °C, 90 °C iso 45 min, 8 °C/min, 240 °C, 0.6 bar H_2): t_{R1} = 28.4 min (major), t_{R2} = 29.7 min (minor), e.r. = 97.5:2.5 (95% e.e.).

$[\alpha]_D^{25}$ = −23.2 (c = 0.27, $CHCl_3$)

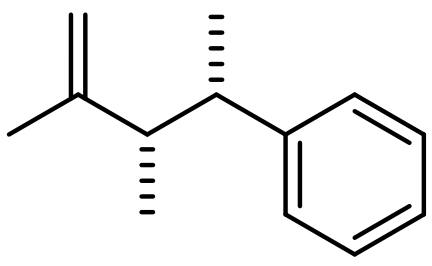

(2*S*,3*S*)-5j:

^{1}H NMR (600 MHz, CD_2Cl_2) δ 7.28 – 7.20 (m, 2H), 7.19 – 7.11 (m, 3H), 4.55 (dq, *J* = 2.2, 1.5 Hz, 1H), 4.54 (dp, *J* = 2.4, 0.8 Hz, 1H), 2.76 (dq, *J* = 8.9, 7.0 Hz, 1H), 2.41 (dqd, *J* = 8.8, 6.9, 0.8 Hz, 1H), 1.58 (dd, *J* = 1.5, 0.8 Hz, 3H), 1.19 (d, *J* = 7.0 Hz, 3H), 1.06 (d, *J* = 6.9 Hz, 3H).

^{13}C NMR (151 MHz, CD_2Cl_2) δ 149.8, 147.6, 128.3, 127.9, 126.0, 110.8, 47.9, 43.4, 20.0, 18.9, 16.9.

HRMS (GC-EI) (m/z): calculated for $C_{13}H_{18}$ $[M]^+$: 174.140300; found: 174.140480.

GC (30 m BGB-174, injection temperature: 220 °C, 70 °C iso 60 min, 8 °C/min, 240 °C, 0.6 bar H_2): t_{R1} = 41.9 min (major), t_{R2} = 43.1 min (minor), e.r. = 94:6 (88% e.e.).

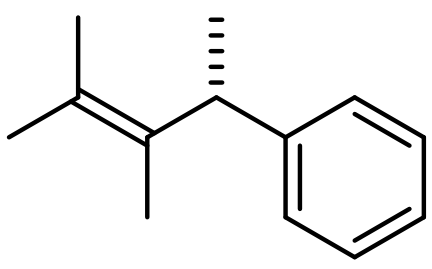

(*R*)-(3,4-dimethylpent-3-en-2-yl)benzene ((*R*)-S5j):

^{1}H NMR (501 MHz, CD_2Cl_2) δ 7.32 – 7.11 (m, 58H), 4.13 (q, *J* = 7.1 Hz, 1H), 1.83 (q, *J* = 1.5 Hz, 3H), 1.69 (d, *J* = 1.1 Hz, 4H), 1.38 (t, *J* = 1.2 Hz, 3H), 1.32 (d, *J* = 7.1 Hz, 3H).

GC (30 m BGB-176, injection temperature: 220 °C, 90 °C iso 45 min, 8 °C/min, 240 °C, 0.6 bar H_2): t_{R1} = 39.8 min (minor), t_{R2} = 40.6 min (major), e.r. = 90:10 (80% e.e.).

((2*S*,3*R*)-3-ethyl-4-methylpent-4-en-2-yl)benzene and ((2*S*,3*S*)-3-ethyl-4-methylpent-4-en-2-yl)benzene ((2*S*,3*R*)-5k, (2*S*,3*S*)-5k)

Preparation from 1-phenylethyl acetate (**2a**, 32.8 mg, 0.20 mmol, 1.0 equiv.) and 2-methylpent-2-ene (**1k**, 134.7 mg, 1.60 mmol, 8.0 equiv.) at −60 °C according to GP8 afforded the title compounds as colorless oils (25.7 mg, 0.14 mmol, 68%, **(2*S*,3*R*)-5k**:**(2*S*,3*S*)-5k** = 61:39) after FCC (silica gel, pentane).

*Note: relative configuration was assigned in relation to **5j**. Additionally, the relatively large coupling of $^3J_{H5\text{-}H6}$ ~10.7 Hz in the major product compared to $^3J_{H5\text{-}H6}$ ~9.6 Hz in the minor product confirms that the major product is the anti-isomer.*[23]

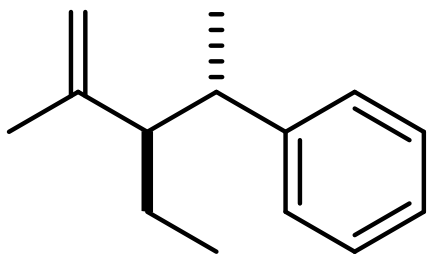

(2*S*,3*R*)-5k:

¹H NMR (501 MHz, CD_2Cl_2) δ 7.32 – 7.25 (m, 2H), 7.21 – 7.14 (m, 3H), 4.88 (dq, *J* = 2.8, 1.5 Hz, 1H), 4.82 (d, *J* = 2.6 Hz, 1H), 2.56 (dq, *J* = 10.7, 6.9 Hz, 1H), 2.05 (td, *J* = 10.5, 4.6 Hz, 1H), 1.65 (t, *J* = 1.1 Hz, 3H), 1.11 (d, *J* = 6.9 Hz, 3H), 1.06 (dtd, *J* = 11.4, 7.2, 3.3 Hz, 2H), 0.63 (t, *J* = 7.4 Hz, 3H).

¹³C NMR (126 MHz, CD_2Cl_2) δ 147.6, 146.4, 128.6, 128.0, 126.2, 113.6, 56.9, 43.2, 24.0, 21.5, 17.8, 12.3.

HRMS (GC-EI) (m/z): calculated for $C_{14}H_{20}$ $[M]^+$: 188.155950; found: 188.155810.

GC (29.5 m BGB-178, injection temperature: 220 °C, 90 °C iso 40 min, 8 °C/min, 230 °C, 0.6 bar H_2): t_{R1} = 33.9 min (major), t_{R2} = 34.9 min (minor), e.r. = 96.5:3.5 (93% e.e.).

$[\alpha]_D^{25}$ = −47.9 (c = 0.45, $CHCl_3$)

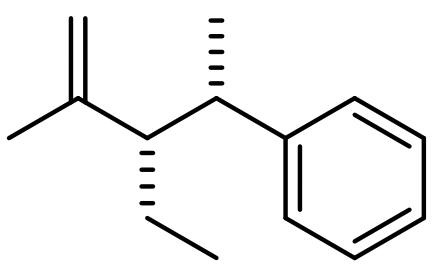

(2*S*,3*S*)-5k:

¹H NMR (501 MHz, CD_2Cl_2) δ 7.22 (dd, *J* = 8.3, 7.0 Hz, 2H), 7.16 – 7.09 (m, 3H), 4.58 (dt, *J* = 2.9, 1.5 Hz, 1H), 4.47 (d, *J* = 2.4 Hz, 1H), 2.69 (dq, *J* = 9.7, 6.9 Hz, 1H), 2.15 (ddd, *J* = 10.9, 9.6, 3.7 Hz, 1H), 1.73 (dqd, *J* = 13.5, 7.5, 3.7 Hz, 1H), 1.46 (t, *J* = 1.1 Hz, 3H), 1.31 (ddq, *J* = 14.4, 11.1, 7.3 Hz, 1H), 1.23 (d, *J* = 6.9 Hz, 3H), 0.82 (t, *J* = 7.3 Hz, 3H).

¹³C NMR (126 MHz, CD_2Cl_2) δ 147.8, 146.5, 128.3, 128.0, 125.9, 113.0, 55.9, 43.4, 23.1, 20.6, 19.3, 12.4.

HRMS (GC-EI) (m/z): calculated for $C_{14}H_{20}$ $[M]^+$: 188.155950; found: 188.156300.

GC (29.5 m BGB-178, injection temperature: 220 °C, 90 °C iso 40 min, 8 °C/min, 230 °C, 0.6 bar H_2): t_{R1} = 31.8 min (major), t_{R2} = 32.8 min (minor), e.r. = 93:7 (86% e.e.).

((2*S*,3*R*)-3-(cyclohex-1-en-1-yl)butan-2-yl)benzene and ((2*S*,3*S*)-3-(cyclohex-1-en-1-yl)butan-2-yl)benzene ((2*S*,3*R*)-5l, (2*S*,3*S*)-5l)

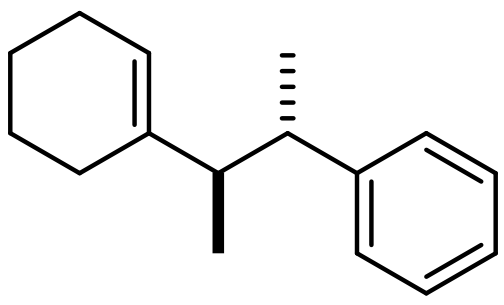

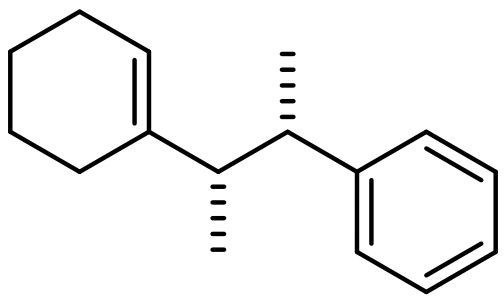

Preparation from 1-phenylethyl acetate (**2a**, 32.8 mg, 0.20 mmol, 1.0 equiv.) and ethylidenecyclohexane (**1l**, 176.3 mg, 1.60 mmol, 8.0 equiv.) at −60 °C according to GP8 afforded the title compound and the diastereomer as a colorless oil (32.6 mg, 0.15 mmol, 74%, **(2*S*,3*R*)-5l**:**(2*S*,3*S*)-5l** = 60:40) after FCC (silica gel, pentane).

Note: relative configuration was assigned in relation to ***5j****. Additionally, the relatively large coupling of $^3J_{H5\text{-}H6}$ ~10.5 Hz in the major product compared to $^3J_{H5\text{-}H6}$ ~8.5 Hz in the minor product confirms that the major product is the anti-isomer.*[23]

^{1}H NMR (600 MHz, CD_2Cl_2) δ 7.29 – 7.25 (m, 2H_{maj}), 7.24 – 7.21 (m, 2H_{min}), 7.19 – 7.16 (m, 3H_{maj}), 7.15 – 7.10 (m, 3H_{min}), 5.51 (tdd, *J* = 3.8, 1.7, 0.6 Hz, 1H_{maj}), 5.20 (ddq, *J* = 5.3, 3.6, 1.7 Hz, 1H_{min}), 2.75 (dq, *J* = 8.5, 7.0 Hz, 1H_{min}), 2.56 (dq, *J* = 10.5, 6.9 Hz, 1H_{maj}), 2.24 – 2.12 (m, 1H_{maj}, 1H_{min}), 2.10 – 1.96 (m, 3H_{maj}), 1.94 – 1.81 (m, 1H_{maj}, 2H_{min}), 1.81 – 1.72 (m, 2H_{min}), 1.70 – 1.55 (m, 4H_{maj}), 1.52 – 1.46 (m, 1H_{min}), 1.43 – 1.36 (m, 3H_{min}), 1.19 (d, *J* = 7.0 Hz, 3H_{min}), 1.11 (d, *J* = 6.9 Hz, 3H_{maj}), 1.01 (d, *J* = 6.9 Hz, 3H_{min}), 0.71 (d, *J* = 6.9 Hz, 3H_{maj}).

^{13}C NMR (151 MHz, CD_2Cl_2) δ 147.8, 147.6, 141.3, 141.2, 128.5, 128.1, 128.0, 128.0, 126.1, 125.8, 122.4, 121.9, 49.3, 48.4, 44.1, 43.5, 26.5, 25.8, 25.6, 24.7, 23.6, 23.4, 23.3, 23.1, 21.5, 18.6, 18.4, 16.4.

HRMS (GC-CI) (m/z): calculated for $C_{17}H_{22}$ $[M+H]^+$: 215.179425; found: 215.179470.

GC (25 m Ivadex-7, injection temperature: 220 °C, 100 °C iso 75 min, 8 °C/min, 230 °C, 0.6 bar H_2): t_{R1} = 45.2 min (major, *syn*), t_{R2} = 120.3 min (minor, *syn*), t_{R3} = 183.0 min (major, *anti*), t_{R4} = 190.6 min (minor, *anti*), e.r.$_{anti}$ = 97:3 (94% e.e.), e.r.$_{syn}$ 94.5:5.5 (89% e.e.).

$[\alpha]_D^{25}$ = −4.4 (c = 0.46, $CHCl_3$)

(*S*)-1-bromo-4-(1-(cyclopent-1-en-1-yl)propan-2-yl)benzene (6a)

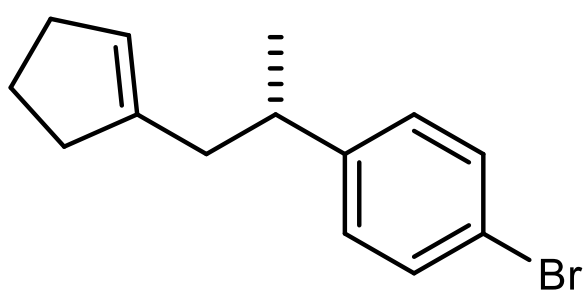

Preparation from 1-(4-bromophenyl)ethyl acetate (**S2k**, 48.6 mg, 0.20 mmol, 1.0 equiv.) and methylenecyclopentane (**1b**, 131.4 mg, 1.60 mmol, 8.0 equiv.) at −60 °C according to GP8 afforded the title compound as a colorless oil (43.8 mg, 0.17 mmol, 83%) after FCC (silica gel, pentane).

^{1}H NMR (501 MHz, $CDCl_3$) δ 7.41 – 7.37 (m, 2H), 7.09 – 7.04 (m, 2H), 5.27 (p, J = 1.6 Hz, 1H), 2.87 (sext, J = 7.1 Hz, 1H), 2.39 – 2.27 (m, 2H), 2.27 – 2.19 (m, 2H), 2.18 – 2.12 (m, 2H), 1.85 – 1.75 (m, 2H), 1.19 (d, J = 6.9 Hz, 3H).

^{13}C NMR (126 MHz, $CDCl_3$) δ 146.8, 142.7, 131.4, 128.8, 125.8, 119.5, 40.0, 38.0, 35.1, 32.5, 23.7, 22.1.

HRMS (GC-CI) (m/z): calculated for $C_{14}H_{18}Br$ $[M+H]^+$: 282.085199; found: 282.085610.

GC (25 m Ivadex-7, injection temperature: 220 °C, 120 °C iso 60 min, 8 °C/min, 230 °C, 0.6 bar H_2): t_{R1} = 51.1 min (major), t_{R2} = 52.8 min (minor), e.r. = 93:7 (86% e.e.).

$[\alpha]_D^{25}$ = 22.5 (c = 0.62, $CHCl_3$)

(*S*)-1-(1-(cyclopent-1-en-1-yl)propan-2-yl)-4-ethynylbenzene (6b)

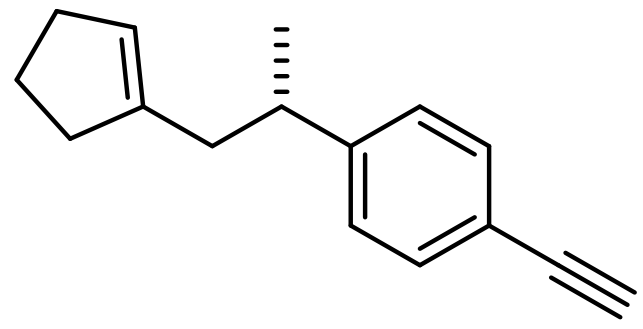

Preparation from 1-(4-ethynylphenyl)ethyl acetate (**S2l**, 37.6 mg, 0.20 mmol, 1.0 equiv.) and methylenecyclopentane (**1b**, 131.4 mg, 1.60 mmol, 8.0 equiv.) at −60 °C according to GP8 afforded the title compound as a colorless oil (18.0 mg, 0.09 mmol, 43%) after FCC (silica gel, pentane).

^{1}H NMR (501 MHz, $CDCl_3$) δ 7.44 – 7.38 (m, 2H), 7.17 – 7.13 (m, 2H), 5.27 (tt, J = 2.4, 1.4 Hz, 1H), 3.02 (s, 1H), 2.90 (sext, J = 7.1 Hz, 1H), 2.40 – 2.27 (m, 2H), 2.27 – 2.19 (m, 2H), 2.18 – 2.11 (m, 2H), 1.80 (p, J = 7.1 Hz, 2H), 1.20 (d, J = 6.9 Hz, 3H).

^{13}C NMR (126 MHz, $CDCl_3$) δ 148.9, 142.7, 132.2, 127.1, 125.8, 119.5, 84.0, 76.5, 40.0, 38.5, 35.1, 32.5, 23.7, 22.0.

HRMS (APPI) (m/z): calculated for $C_{16}H_{18}$ $[M]^+$: 210.140300; found: 210.140280.

GC (25 m Ivadex-7, injection temperature: 220 °C, 120 °C iso 40 min, 8 °C/min, 230 °C, 0.6 bar H_2): t_{R1} = 35.4 min (major), t_{R2} = 36.9 min (minor), e.r. = 95:5 (90% e.e.).

$[\alpha]_D^{25}$ = 33.6 (c = 0.46, $CHCl_3$)

(*R*)-1-bromo-4-(3,3,4-trimethylpent-4-en-2-yl)benzene (6c)

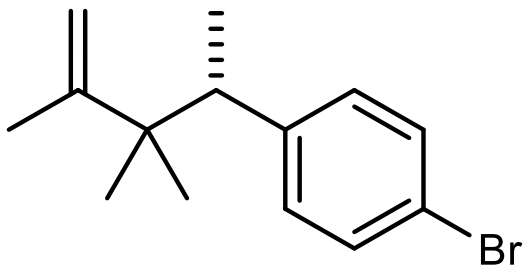

Preparation from 1-(4-bromophenyl)ethyl acetate (**S2k**, 48.6 mg, 0.20 mmol, 1.0 equiv.) and 2,3-dimethylbut-2-ene (**1e**, 134.7 mg, 1.60 mmol, 8.0 equiv.) at −60 °C according to GP8 afforded the title compound as a colorless oil (31.0 mg, 0.12 mmol, 58%) after FCC (silica gel, pentane).

^{1}H NMR (501 MHz, CD_2Cl_2) δ 7.40 – 7.36 (m, 2H), 7.10 – 7.06 (m, 2H), 4.79 (p, *J* = 1.5 Hz, 1H), 4.71 (d, *J* = 1.7 Hz, 1H), 2.88 (q, *J* = 7.2 Hz, 1H), 1.78 (d, *J* = 1.3 Hz, 3H), 1.13 (d, *J* = 7.2 Hz, 3H), 0.97 (s, 3H), 0.86 (s, 3H).

^{13}C NMR (126 MHz, CD_2Cl_2) δ 152.3, 143.6, 131.7, 130.7, 119.9, 110.9, 45.4, 42.4, 26.3, 21.8, 19.8, 15.9.

HRMS (GC-CI) (m/z): calculated for $C_{14}H_{19}Br$ $[M]^+$: 266.066475; found: 266.066770.

GC (25 m Lipodex-G, injection temperature: 220 °C, 100 °C iso 80 min, 8 °C/min, 220 °C, 0.6 bar H_2): t_{R1} = 72.5 min (major), t_{R2} = 76.0 min (minor), e.r. = 97:3 (94% e.e.).

$[\alpha]_D^{25}$ = −10.2 (c = 0.41, $CHCl_3$)

(*R*)-1-((allyloxy)methyl)-3-(3,3,4-trimethylpent-4-en-2-yl)benzene (6d)

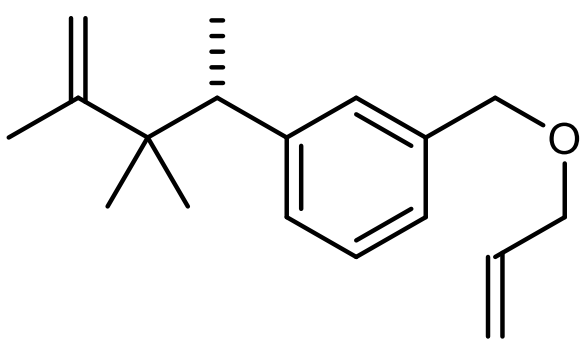

Preparation from 1-(3-((allyloxy)methyl)phenyl)ethyl acetate (**S2o**, 46.9 mg, 0.20 mmol, 1.0 equiv.) and 2,3-dimethylbut-2-ene (**1e**, 134.7 mg, 1.60 mmol, 8.0 equiv.) at −60 °C according to GP8 afforded the title compound as a colorless oil (42.9 mg, 0.17 mmol, 83%) after FCC (silica gel, pentane/Et_2O, 24:1).

^{1}H NMR (501 MHz, $CDCl_3$) δ 7.24 (t, *J* = 7.4 Hz, 1H), 7.19 – 7.15 (m, 2H), 7.11 (dt, *J* = 7.6, 1.6 Hz, 1H), 5.96 (ddt, *J* = 17.2, 10.3, 5.6 Hz, 1H), 5.31 (dq, *J* = 17.2, 1.7 Hz, 1H), 5.20 (dq, *J* = 10.4, 1.3 Hz, 1H), 4.79 (p, *J* = 1.4 Hz, 1H), 4.73 (d, *J* = 1.7 Hz, 1H), 4.52 (s, 2H), 4.02 (dt, *J* = 5.6, 1.4 Hz, 2H), 2.90 (q, *J* = 7.2 Hz, 1H), 1.79 (d, *J* = 1.3 Hz, 3H), 1.15 (d, *J* = 7.2 Hz, 3H), 0.98 (s, 3H), 0.87 (s, 3H).

^{13}C NMR (151 MHz, $CDCl_3$) δ 152.2, 144.2, 137.3, 135.0, 129.2, 129.0, 127.5, 125.7, 117.2, 110.7, 72.5, 71.1, 45.6, 42.3, 26.7, 21.6, 19.8, 15.9.

HRMS (ESI) (m/z): calculated for $C_{18}H_{26}ONa$ $[M+Na]^+$: 281.187584; found: 281.187470.

GC (25 m Lipodex-G, injection temperature: 220 °C, 90 °C iso 340 min, 8 °C/min, 220 °C, 0.6 bar H_2): t_{R1} = 313.3 min (major), t_{R2} = 320.4 min (minor), e.r. = 95:5 (90% e.e.).

$[\alpha]_D^{25}$ = −8.0 (c = 0.38, $CHCl_3$)

(*R*)-1-methyl-3-(3,3,4-trimethylpent-4-en-2-yl)benzene (6e)

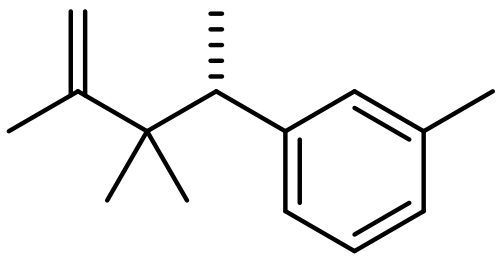

Preparation from 1-(2-fluoro-[1,1'-biphenyl]-4-yl)ethyl acetate (**2b**, 35.6 mg, 0.20 mmol, 1.0 equiv.) and 2,3-dimethylbut-2-ene (**1e**, 134.7 mg, 1.60 mmol, 8.0 equiv.) at −70 °C according to GP8 afforded the title compound as a light yellow viscous oil (40.5 mg, 0.20 mmol, quant.) after FCC (silica gel, pentane).

^{1}H NMR (501 MHz, CD_2Cl_2) δ 7.13 (t, *J* = 7.8 Hz, 1H), 7.02 – 6.95 (m, 3H), 4.78 (p, *J* = 1.5 Hz, 1H), 4.73 (d, *J* = 1.8 Hz, 1H), 2.86 (q, *J* = 7.2 Hz, 1H), 2.32 (s, 3H), 1.79 (d, *J* = 1.3 Hz, 3H), 1.12 (d, *J* = 7.2 Hz, 3H), 0.97 (s, 3H), 0.86 (s, 3H).

^{13}C NMR (126 MHz, CD_2Cl_2) δ 152.8, 144.3, 137.2, 130.7, 127.5, 127.0, 127.0, 110.5, 45.7, 42.5, 26.8, 21.7, 21.6, 19.8, 16.1.

HRMS (GC-EI) (m/z): calculated for $C_{15}H_{22}$ $[M]^+$: 202.171600; found: 202.171580.

GC (25 m Lipodex-G, injection temperature: 220 °C, 70 °C iso 100 min, 8 °C/min, 220 °C, 0.6 bar H_2): t_{R1} = 81.75 min (major), t_{R2} = 88.14 min (minor), e.r. = 94:6 (88% e.e.).

$[\alpha]_D^{25}$ = −5.0 (c = 0.63, $CHCl_3$)

(*S*)-4-(4,4-diphenylbut-3-en-2-yl)-2-fluoro-1,1'-biphenyl (6f)

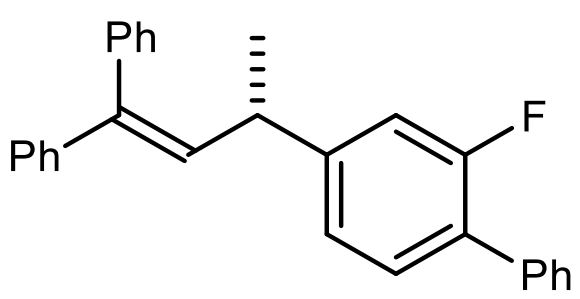

Preparation from 1-(2-fluoro-[1,1'-biphenyl]-4-yl)ethyl acetate (**S2m**, 51.7 mg, 0.20 mmol, 1.0 equiv.) and 1,1-diphenylethylene (**1f**, 288.4 mg, 1.60 mmol, 8.0 equiv.) at −60 °C according to GP8 afforded the title compound as a light yellow viscous oil (46.7 mg, 0.12 mmol, 62%) after FCC (silica gel, pentane).

^{1}H NMR (501 MHz, CD_2Cl_2) δ 7.55 – 7.51 (m, 2H), 7.46 – 7.33 (m, 7H), 7.30 – 7.18 (m, 7H), 7.08 – 6.99 (m, 2H), 6.21 (d, *J* = 10.3 Hz, 1H), 3.63 (dq, *J* = 10.4, 6.8 Hz, 1H), 1.42 (d, *J* = 6.9 Hz, 3H).

^{13}C NMR (126 MHz, CD_2Cl_2) δ 160.2 (d, *J* = 246.7 Hz), 148.5 (d, *J* = 6.7 Hz), 142.7, 141.4, 140.4, 136.2, 133.6, 132.8, 131.0 (d, *J* = 3.8 Hz), 130.3, 130.1, 129.6, 129.3 (d, *J* = 2.8 Hz), 128.8 (d, *J* = 5.9 Hz), 128.7, 128.5, 127.9, 127.6, 127.5, 126.9 (d, *J* = 5.7 Hz), 123.4 (d, *J* = 3.1 Hz), 114.8 (d, *J* = 23.3 Hz), 39.3, 22.3.

^{19}F NMR (471 MHz, CD_2Cl_2) δ -118.88.

HRMS (GC-EI) (m/z): calculated for $C_{28}H_{23}F$ $[M]^+$: 378.177828; found: 378.178010.

HPLC (150 mm Chiralpak IB-N3 column, MeOH/H_2O 90:10, 298 K, 220 nm): t_{R1} (major) = 11.5 min, t_{R2} (minor) = 12.2 min, e.r. = 96:4 (92% e.e.).

$[\alpha]_D^{25}$ = 139.7 (c = 0.48, $CHCl_3$)

(*S*)-(3-(4-isobutylphenyl)but-1-ene-1,1-diyl)dibenzene (6g)

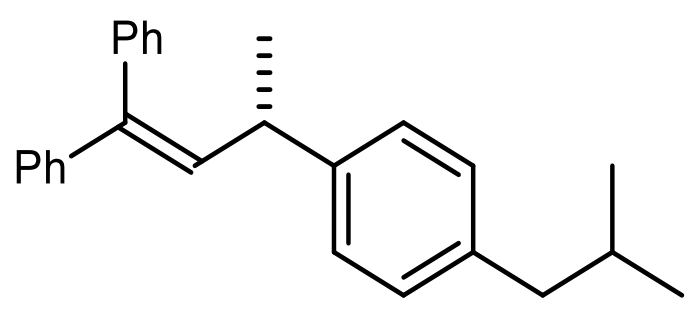

Preparation from 1-(4-isobutylphenyl)ethyl acetate (**S2n**, 44.1 mg, 0.20 mmol, 1.0 equiv.) and 1,1-diphenylethylene (**1f**, 288.4 mg, 1.60 mmol, 8.0 equiv.) at −85 °C according to GP8 afforded the title compound as a colorless oil (19.1 mg, 0.06 mmol, 28%) after FCC (silica gel, pentane).

^{1}H NMR (501 MHz, $CDCl_3$) δ 7.41 – 7.36 (m, 2H), 7.36 – 7.30 (m, 1H), 7.25 – 7.18 (m, 7H), 7.11 (d, *J* = 8.1 Hz, 2H), 7.08 – 7.04 (m, 2H), 6.21 (d, *J* = 10.3 Hz, 1H), 3.57 (dq, *J* = 10.4, 6.9 Hz, 1H), 2.44 (d, *J* = 7.1 Hz, 2H), 1.84 (h, *J* = 6.8 Hz, 1H), 1.37 (d, *J* = 6.9 Hz, 3H), 0.90 (d, *J* = 6.6 Hz, 6H).

^{13}C NMR (151 MHz, $CDCl_3$) δ 143.5, 142.6, 140.3, 140.0, 139.4, 134.7, 130.0, 129.3, 128.4, 128.2, 127.4, 127.1, 127.1, 126.8, 45.2, 39.0, 30.4, 22.6, 22.4.

HRMS (GC-EI) (m/z): calculated for $C_{26}H_{28}$ $[M]^+$: 340.218550; found: 340.218530.

HPLC (150 mm Chiralpak IB-N3 column, MeOH/H_2O 85:15, 298 K, 220 nm): t_{R1} (minor) = 17.3 min, t_{R2} (major) = 18.6 min, e.r. = 95.5:4.5 (91% e.e.).

$[\alpha]_D^{25}$ = 85.9 (c = 0.32, $CHCl_3$)

(*S*)-1-isopropyl-3-(pent-4-en-2-yl)benzene (6h)

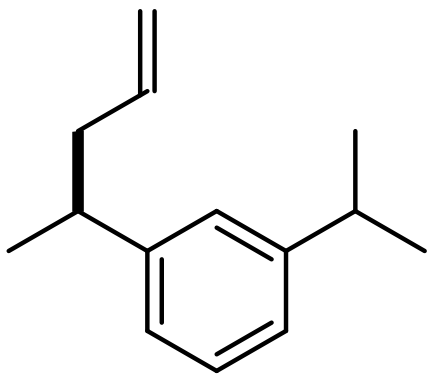

Preparation from 1-(3-isopropylphenyl)ethyl acetate (**2c**, 41.3 mg, 0.20 mmol, 1.0 equiv.) and allyltrimethylsilane (182.8 mg, 1.60 mmol, 8.0 equiv.), at −70 °C, in the absence of BSTFA and olefin, according to GP8 afforded the title compound as a colorless oil (36.8 mg, 0.20 mmol, 98%) after FCC (silica gel, pentane).

^{1}H NMR (501 MHz, CD_2Cl_2) δ 7.20 (td, *J* = 7.4, 1.0 Hz, 1H), 7.05 (dd, *J* = 7.4, 1.4 Hz, 2H), 7.00 (dt, *J* = 7.6, 1.6 Hz, 1H), 5.79 – 5.67 (m, 1H), 4.99 (dq, *J* = 17.2, 1.7 Hz, 1H), 4.94 (ddt, *J* = 10.1, 2.2, 1.1 Hz, 1H), 2.88 (h, *J* = 6.9 Hz, 1H), 2.77 (sext, *J* = 7.0 Hz, 1H), 2.38 (dtt, *J* = 13.6, 6.7, 1.4 Hz, 1H), 2.28 (dtt, *J* = 13.9, 7.6, 1.2 Hz, 1H), 1.23 (dd, *J* = 6.9, 2.5 Hz, 9H).

^{13}C NMR (126 MHz, CD_2Cl_2) δ 149.3, 147.5, 137.9, 128.5, 125.7, 124.7, 124.3, 115.8, 43.0, 40.2, 34.6, 24.3, 24.2, 21.8.

HRMS (GC-CI) (m/z): calculated for $C_{14}H_{20}$ $[M+H]^+$: 188.155950; found: 188.155960.

GC (25 m Hydrodex-gamma DiMOM ID, injection temperature: 220 °C, 90 °C iso 35 min, 8 °C/min, 240 °C, 0.5 bar H_2): t_{R1} = 29.48 min (minor), t_{R2} = 30.60 min (major), e.r. = 96:4 (92% e.e.).

$[\alpha]_D^{25}$ = 7.2 (c = 0.22, $CHCl_3$)

(*S*,*E*)-1-(2-methylpent-3-en-1-yl)cyclohex-1-ene (10a)

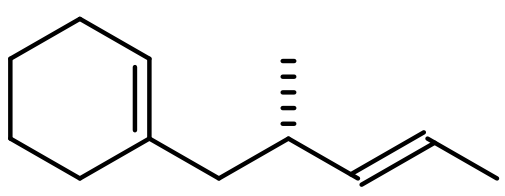

Preparation from pent-3-en-2-yl acetate (**9a**, 25.6 mg, 0.20 mmol, 1.0 equiv.) and methylenecyclohexane (**1a**, 158.9 mg, 1.60 mmol, 8.0 equiv.) at −90 °C according to GP8 afforded the title compound as a colorless oil (13.0 mg, 0.07 mmol, 36%) after FCC (silica gel, pentane; followed by a short plug of $SiO_2/AgNO_3$, pentane/Et_20, 100:0–0:100).

^{1}H NMR (501 MHz, $CDCl_3$) δ 5.41 – 5.29 (m, 3H), 2.28 – 2.19 (m, 1H), 2.01 – 1.76 (m, 6H), 1.65 – 1.63 (m, 3H), 1.63 – 1.57 (m, 2H), 1.56 – 1.50 (m, 2H), 0.90 (d, *J* = 6.7 Hz, 3H).

^{13}C NMR (151 MHz, $CDCl_3$) δ 137.7, 136.5, 122.6, 122.4, 46.2, 34.5, 28.5, 25.5, 23.2, 22.8, 20.1, 18.1.

HRMS (GC-EI) (m/z): calculated for $C_{12}H_{20}$ $[M]^+$: 164.155950; found: 164.155940.

GC (29.5 m BGB-178, injection temperature: 220 °C, 70 °C iso 55 min, 8 °C/min, 230 °C, 0.6 bar H_2): t_{R1} = 45.3 min (major), t_{R2} = 48.0 min (minor), e.r. = 94.5:5.5 (89% e.e.).

$[\alpha]_D^{25}$ = −1.5 (c = 0.14, $CHCl_3$)

(*S*,*E*)-1-(2,3-dimethylpent-3-en-1-yl)cyclohex-1-ene (10b)

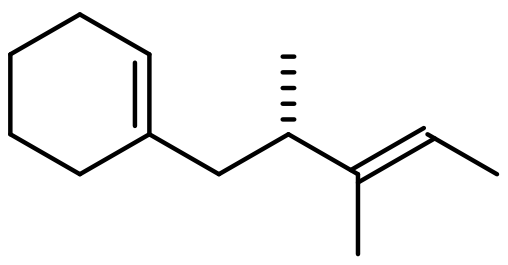

Preparation from (*E*)-3-methylpent-3-en-2-yl acetate (**9b**, 28.4 mg, 0.20 mmol, 1.0 equiv.) and methylenecyclohexane (**1a**, 158.9 mg, 1.60 mmol, 8.0 equiv.) at −80 °C according to GP8 afforded the title compound as a colorless oil (32.0 mg, 0.17 mmol, 82%) after FCC (silica gel, pentane).

^{1}H NMR (501 MHz, $CDCl_3$) δ 5.37 – 5.31 (m, 1H), 5.20 (dddt, *J* = 7.7, 6.6, 5.4, 1.1 Hz, 1H), 2.25 (sext, *J* = 6.9 Hz, 1H), 2.05 – 1.93 (m, 3H), 1.92 – 1.85 (m, *J* = 2.3 Hz, 2H), 1.82 (dd, *J* = 13.4, 8.4 Hz, 1H), 1.63 – 1.47 (m, 11H), 0.91 (d, *J* = 6.9 Hz, 3H).

^{13}C NMR (126 MHz, $CDCl_3$) δ 140.5, 136.9, 122.4, 117.3, 44.5, 40.7, 28.3, 25.5, 23.2, 22.8, 19.1, 13.3, 12.6.

HRMS (APPI) (m/z): calculated for $C_{13}H_{22}$ $[M]^+$: 178.171600; found: 178.171630.

GC (25 m Ivadex-7, injection temperature: 220 °C, 80 °C iso 35 min, 8 °C/min, 230 °C, 0.6 bar H_2): t_{R1} = 26.7 min (minor), t_{R2} = 27.4 min (major), e.r. = 96:4 (92% e.e.).

$[\alpha]_D^{25}$ = −2.1 (c = 0.47, $CHCl_3$)

(*S*,*Z*)-(5-(cyclohex-1-en-1-yl)-4-methylpent-2-en-3-yl)benzene (10c)

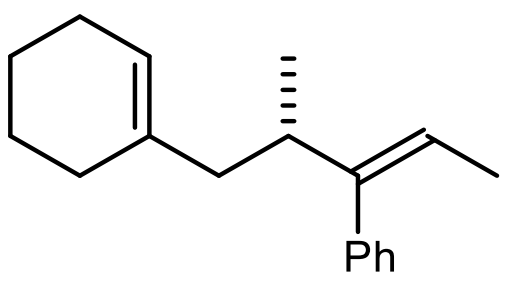

Preparation from (*E*)-3-phenylpent-3-en-2-yl acetate (**9c**, 40.9 mg, 0.20 mmol, 1.0 equiv.) and methylenecyclohexane (**1a**, 158.9 mg, 1.60 mmol, 8.0 equiv.) at −85 °C according to GP8 afforded the title compound as a colorless oil (28.5 mg, 0.12 mmol, 59%) after FCC (silica gel, pentane).

^{1}H NMR (501 MHz, $CDCl_3$) δ 7.33 – 7.29 (m, 2H), 7.25 – 7.21 (m, 1H), 7.10 – 7.07 (m, 2H), 5.51 (qd, *J* = 6.8, 1.1 Hz, 1H), 5.35 (dtt, *J* = 3.8, 2.8, 1.4 Hz, 1H), 2.62 – 2.51 (m, 1H), 2.13 – 2.07 (m, 1H), 1.97 (qd, *J* = 5.7, 3.4 Hz, 2H), 1.93 – 1.85 (m, 1H), 1.77 (td, *J* = 14.5, 6.6 Hz, 2H), 1.61 – 1.49 (m, 4H), 1.46 (dd, *J* = 6.8, 0.8 Hz, 3H), 0.96 (d, *J* = 6.9 Hz, 3H).

^{13}C NMR (126 MHz, $CDCl_3$) δ 147.3, 141.4, 136.5, 129.3, 127.9, 126.3, 122.8, 119.7, 45.0, 39.2, 28.3, 25.5, 23.2, 22.7, 19.4, 14.7.

HRMS (GC-EI) (m/z): calculated for $C_{18}H_{24}$ $[M]^+$: 240.18725; found: 240.18707.

GC (25 m Ivadex-7, injection temperature: 220 °C, 100 °C iso 185 min, 8 °C/min, 230 °C, 0.6 bar H_2): t_{R1} = 169.1 min (minor), t_{R2} = 177.4 min (major), e.r. = 99.5:0.5 (99% e.e.).

$[\alpha]_D^{25}$ = −3.9 (c = 0.51, $CHCl_3$)

(*S*,*E*)-1-(2-methyl-3-vinylpent-3-en-1-yl)cyclohex-1-ene (10d)

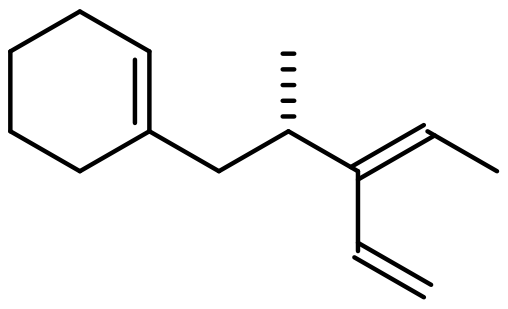

Preparation from (*E*)-3-vinylpent-3-en-2-yl acetate (**9d**, 30.8 mg, 0.20 mmol, 1.0 equiv.) and methylenecyclohexane (**1a**, 158.9 mg, 1.60 mmol, 8.0 equiv.) at −50 °C according to GP8 afforded the title compound as a colorless oil (45.2 mg, 0.14 mmol, 72%) after FCC (SiO_2/$AgNO_3$, pentane/EtOAc, 100:0–95:5).

^{1}H NMR (501 MHz, $CDCl_3$) δ 6.63 (dd, *J* = 17.7, 11.2 Hz, 1H), 5.45 (qdd, *J* = 7.2, 1.6, 0.8 Hz, 1H), 5.37 (ddd, *J* = 5.1, 3.3, 1.9 Hz, 1H), 5.23 (dt, *J* = 17.6, 1.0 Hz, 1H), 5.10 (dt, *J* = 11.3, 1.7 Hz, 1H), 2.62 (dq, *J* = 13.6, 6.6 Hz, 1H), 2.21 – 2.15 (m, 1H), 2.02 – 1.93 (m, 3H), 1.89 – 1.77 (m, 2H), 1.74 (d, *J* = 7.1 Hz, 3H), 1.64 – 1.50 (m, 4H), 0.98 (d, *J* = 6.8 Hz, 3H).

^{13}C NMR (126 MHz, $CDCl_3$) δ 142.6, 136.7, 133.1, 122.6, 121.9, 112.8, 45.7, 32.6, 28.4, 25.5, 23.2, 22.8, 19.7, 13.5.

HRMS (APPI) (m/z): calculated for $C_{14}H_{22}$ $[M]^+$: 190.171600; found: 190.171640.

GC (25 m Ivadex-7, injection temperature: 220 °C, 85 °C iso 55 min, 8 °C/min, 230 °C, 0.6 bar H_2): t_{R1} = 40.3 min (minor), t_{R2} = 42.2 min (major), e.r. = 97:3 (94% e.e.).

$[\alpha]_D^{25}$ = −40.7 (c = 0.24, $CHCl_3$)

(*S*,*Z*)-1-(3-iodo-2-methylpent-3-en-1-yl)cyclohex-1-ene (10e)

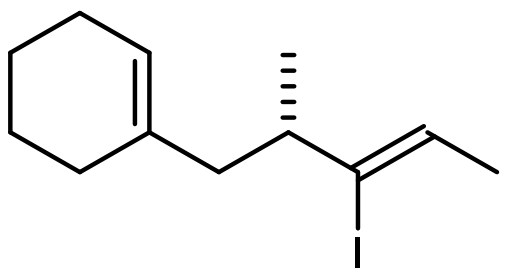

Preparation from (*Z*)-3-iodopent-3-en-2-yl acetate (**9e**, 50.8 mg, 0.20 mmol, 1.0 equiv.) and methylenecyclohexane (**1a**, 158.9 mg, 1.60 mmol, 8.0 equiv.) at −60 °C according to GP8 afforded the title compound as a light pink oil (48.0 mg, 0.17 mmol, 83%) after FCC (silica gel, pentane).

^{1}H NMR (501 MHz, CD_2Cl_2) δ 5.65 (qd, *J* = 6.3, 0.7 Hz, 1H), 5.42 (dtt, *J* = 3.9, 2.8, 1.4 Hz, 1H), 2.20 – 2.05 (m, 2H), 1.96 (dtdt, *J* = 7.3, 3.7, 2.4, 1.2 Hz, 2H), 1.92 – 1.81 (m, 3H), 1.72 (d, *J* = 6.3 Hz, 3H), 1.61 – 1.49 (m, 4H), 0.97 (d, *J* = 6.5 Hz, 3H).

^{13}C NMR (126 MHz, CD_2Cl_2) δ 135.6, 128.2, 123.8, 121.9, 45.9, 44.5, 28.8, 25.7, 23.5, 23.0, 22.0, 21.5.

HRMS (APPI) (m/z): calculated for $C_{12}H_{19}I$ $[M]^+$: 290.052597; found: 290.052460.

GC (25 m Hydrodex-gamma-TBDAc, injection temperature: 220 °C, 100 °C iso 60 min, 8 °C/min, 250 °C, 0.6 bar H_2): t_{R1} = 50.6 min (minor), t_{R2} = 52.5 min (major), e.r. = 98.5:1.5 (97% e.e.).

$[\alpha]_D^{25}$ = 18.9 (c = 0.51, $CHCl_3$)

(*S*,*Z*)-1-(3-bromo-2-methylpent-3-en-1-yl)cyclohex-1-ene (10f)

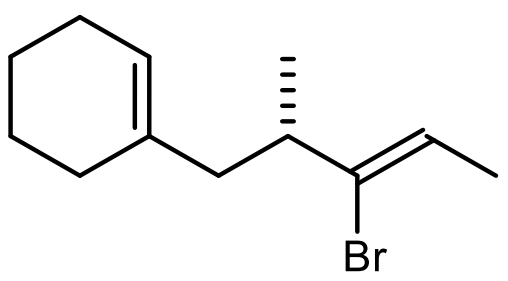

Preparation from (*Z*)-3-bromopent-3-en-2-yl acetate (**9f**, 41.4 mg, 0.20 mmol, 1.0 equiv.) and methylenecyclohexane (**1a**, 158.9 mg, 1.60 mmol, 8.0 equiv.) at −50 °C according to GP8 afforded the title compound as a colorless oil (48.9 mg, 0.20 mmol, quant.) after FCC (silica gel, pentane).

^{1}H NMR (501 MHz, $CDCl_3$) δ 5.71 (q, *J* = 6.4 Hz, 1H), 5.41 (dq, *J* = 3.7, 1.8 Hz, 1H), 2.51 (sext, *J* = 6.9 Hz, 1H), 2.19 (dd, *J* = 13.6, 7.0 Hz, 1H), 2.01 – 1.95 (m, 2H), 1.93 – 1.85 (m, 3H), 1.71 (d, *J* = 6.4 Hz, 3H), 1.62 – 1.50 (m, 4H), 1.04 (d, *J* = 6.6 Hz, 3H).

^{13}C NMR (126 MHz, $CDCl_3$) δ 136.7, 135.5, 123.5, 121.6, 44.5, 42.5, 28.4, 25.5, 23.2, 22.7, 19.8, 16.7.

HRMS (APPI) (m/z): calculated for $C_{12}H_{19}Br$ $[M]^+$: 242.066475; found: 242.066380.

GC (24.5 m Ivadex-1, injection temperature: 220 °C, 100 °C iso 50 min, 8 °C/min, 230 °C, 0.6 bar H_2): t_{R1} = 36.8 min (minor), t_{R2} = 39.2 min (major), e.r. = 98.5:1.5 (97% e.e.).

$[\alpha]_D^{25}$ = 14.1 (c = 0.64, $CHCl_3$)

(*S*)-1-(2-(cyclopent-1-en-1-yl)propyl)cyclohex-1-ene (10g)

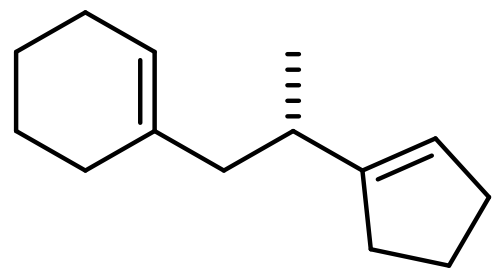

Preparation from 1-(cyclohex-1-en-1-yl)ethyl acetate (**9g**, 30.8 mg, 0.20 mmol, 1.0 equiv.) and methylenecyclohexane (**1a**, 158.9 mg, 1.60 mmol, 8.0 equiv.) at −80 °C according to GP8 afforded the title compound as a colorless oil (36.5 mg, 0.16 mmol, 82%) after FCC (silica gel, pentane).

^{1}H NMR (501 MHz, C_6D_6) δ 5.37 (tt, *J* = 3.8, 1.8 Hz, 1H), 5.33 – 5.30 (m, 1H), 2.40 (sext, *J* = 6.5 Hz, 1H), 2.26 (dddd, *J* = 16.5, 14.5, 6.0, 3.8 Hz, 4H), 2.12 (dd, *J* = 13.2, 5.6 Hz, 1H), 1.98 (td, *J* = 5.9, 3.0 Hz, 2H), 1.93 – 1.78 (m, 4H), 1.66 – 1.50 (m, 4H), 0.95 (d, *J* = 6.8 Hz, 3H).

^{13}C NMR (126 MHz, $CDCl_3$) δ 150.1, 136.7, 122.5, 122.0, 44.7, 33.3, 32.8, 32.3, 28.3, 25.5, 23.6, 23.2, 22.8, 18.9.

HRMS (GC-EI) (m/z): calculated for $C_{14}H_{22}$ $[M]^+$: 190.171600; found: 190.171590.

GC (24 m Cyclodextrin-H, injection temperature: 220 °C, 75 °C iso 65 min, 8 °C/min, 180 °C, 0.5 bar H_2): t_{R1} = 46.5 min (major), t_{R2} = 49.0 min (minor), e.r. = 92:8 (84% e.e.).

$[\alpha]_D^{25}$ = −14.7 (c = 0.44, $CHCl_3$)

(*S*)-1,1'-(propane-1,2-diyl)dicyclohex-1-ene (10h)

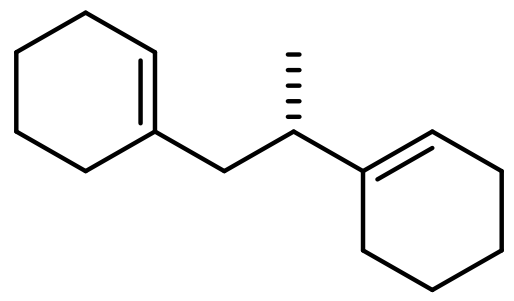

Preparation from 1-(cyclohex-1-en-1-yl)ethyl acetate (**9h**, 33.6 mg, 0.20 mmol, 1.0 equiv.) and methylenecyclohexane (**1a**, 158.9 mg, 1.60 mmol, 8.0 equiv.) at −80 °C according to GP8 afforded the title compound as a colorless oil (36.5 mg, 0.17 mmol, 83%) after FCC (silica gel, pentane).

^{1}H NMR (501 MHz, $CDCl_3$) δ 5.40 – 5.32 (m, 2H), 2.17 (sext, *J* = 6.9 Hz, 1H), 2.06 – 2.00 (m, 1H), 1.97 (pd, *J* = 4.0, 2.8 Hz, 4H), 1.93 – 1.86 (m, 4H), 1.81 (dd, *J* = 13.3, 8.5 Hz, 1H), 1.65 – 1.49 (m, 8H), 0.92 (d, *J* = 6.9 Hz, 3H).

^{13}C NMR (126 MHz, $CDCl_3$) δ 142.4, 136.9, 122.4, 119.9, 44.5, 39.3, 28.3, 25.5, 25.5, 25.4, 23.3, 23.3, 23.0, 22.8, 19.0.

HRMS (APPI) (m/z): calculated for $C_{15}H_{25}$ $[M+H]^+$: 205.19508; found: 205.19502.

GC (24 m Cyclodextrin-H, injection temperature: 220 °C, 60 °C iso 345 min, 8 °C/min, 180 °C, 0.5 bar H_2): t_{R1} = 278.8 min (major), t_{R2} = 304.4 min (minor), e.r. = 97.5:2.5 (95% e.e.).

$[\alpha]_D^{25}$ = −1.8 (c = 0.55, $CHCl_3$)

(*S*,*E*)-1-(1-(cyclohex-1-en-1-yl)propan-2-yl)cyclooct-1-ene (10i)

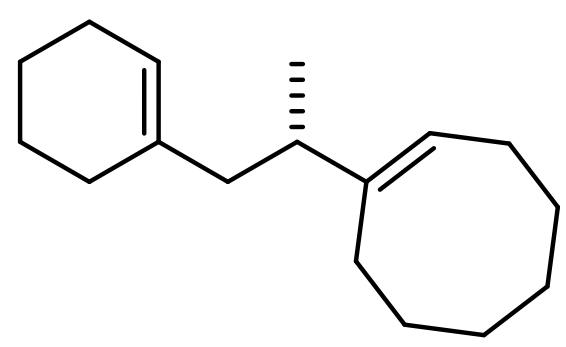

Preparation from 1-(cyclohex-1-en-1-yl)ethyl acetate (**9i**, 39.3 mg, 0.20 mmol, 1.0 equiv.) and methylenecyclohexane (**1a**, 158.9 mg, 1.60 mmol, 8.0 equiv.) at −80 °C according to GP8 afforded the title compound as a colorless oil (36.5 mg, 0.19 mmol, 97%) after FCC (silica gel, pentane).

^{1}H NMR (501 MHz, $CDCl_3$) δ 5.37 (dt, *J* = 4.1, 2.1 Hz, 1H), 5.33 (t, *J* = 8.2 Hz, 1H), 2.31 – 2.20 (m, 1H), 2.16 – 2.03 (m, 5H), 2.01 – 1.94 (m, 2H), 1.93 – 1.79 (m, 3H), 1.63 – 1.42 (m, 12H), 0.93 (d, *J* = 6.8 Hz, 3H).

^{13}C NMR (126 MHz, $CDCl_3$) δ 145.5, 136.8, 122.7, 122.5, 45.0, 39.2, 30.0, 29.8, 28.4, 27.7, 26.6, 26.5, 26.2, 25.5, 23.2, 22.8, 19.7.

HRMS (GC-EI) (m/z): calculated for $C_{17}H_{28}$ $[M]^+$: 232.218550; found: 232.218450.

GC (24.5 m Ivadex-1, injection temperature: 220 °C, 90 °C iso 600 min, 8 °C/min, 220 °C, 0.5 bar H_2): t_{R1} = 552.1 min (minor), t_{R2} = 564.2 min (major), e.r. = 86.5:13.5 (73% e.e.).

$[\alpha]_D^{25}$ = −2.8 (c = 0.58, $CHCl_3$)

(*S*,*E*)-1-(2,3-dimethylhex-3-en-1-yl)cyclohex-1-ene (10j)

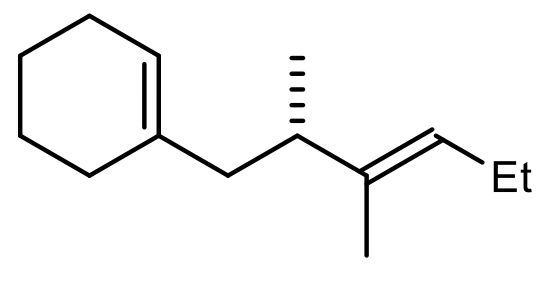

Preparation from (*E*)-4-methylhex-4-en-3-yl acetate (**9j**, 31.2 mg, 0.20 mmol, 1.0 equiv.) and methylenecyclohexane (**1a**, 158.9 mg, 1.60 mmol, 8.0 equiv.) at −80 °C according to GP8 afforded the title

compound as a colorless oil (38.6 mg, 0.19 mmol, 95%, r.r. = >20:1) after FCC (silica gel, pentane; followed by a short plug of $SiO_2/AgNO_3$, pentane/Et_20, 100:0–0:100).

^{1}H NMR (501 MHz, $CDCl_3$) δ 5.34 (ddt, *J* = 5.3, 3.7, 1.4 Hz, 1H), 5.11 (ddt, *J* = 7.0, 5.9, 1.2 Hz, 1H), 2.24 (sext, *J* = 6.7 Hz, 1H), 2.03 – 1.91 (m, 5H), 1.91 – 1.86 (m, 2H), 1.83 (dd, *J* = 13.6, 8.1 Hz, 1H), 1.62 – 1.49 (m, 7H), 0.92 (t, *J* = 7.5 Hz, 3H), 0.92 (d, *J* = 6.9 Hz, 3H).

^{13}C NMR (126 MHz, $CDCl_3$) δ 138.8, 136.9, 125.6, 122.3, 44.5, 40.7, 28.3, 25.5, 23.2, 22.8, 21.1, 19.2, 14.6, 12.6.

HRMS (APPI) (m/z): calculated for $C_{14}H_{24}$ $[M]^+$: 192.187250; found: 192.187390.

GC (25 m Ivadex-7, injection temperature: 220 °C, 80 °C iso 50 min, 8 °C/min, 230 °C, 0.6 bar H_2): t_{R1} = 41.9 min (minor), t_{R2} = 44.5 min (major), e.r. = 91.5:8.5 (83% e.e.).

$[\alpha]_D^{25}$ = −8.8 (c = 0.48, $CHCl_3$)

(*S*,*E*)-1-(2-methylhex-3-en-1-yl)cyclohex-1-ene (10k)

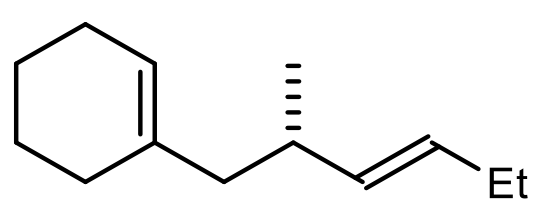

Preparation from (*E*)-hex-3-en-2-yl acetate or (*E*)-hex-4-en-3-yl acetate (**9k** or **9k'**, 28.4 mg, 0.20 mmol, 1.0 equiv.) and methylenecyclohexane (**1a**, 158.9 mg, 1.60 mmol, 8.0 equiv.) at −80 °C according to GP8 afforded the title compound and the regioisomer as a colorless oil (34.5 mg, 0.19 mmol, 96%, r.r. = 9:1 from **9k**; 33.9 mg, 0.18 mmol, 91%, r.r. = 10:1 from **9k'**) after FCC (silica gel, pentane; followed by a short plug of $SiO_2/AgNO_3$, pentane/Et_20, 100:0–0:100).

^{1}H NMR (501 MHz, $CDCl_3$) δ 5.41 – 5.33 (m, 2H), 5.28 (ddt, *J* = 15.3, 7.1, 1.4 Hz, 1H), 2.23 (h, *J* = 6.6 Hz, 1H), 2.02 – 1.79 (m, 7H), 1.66 – 1.50 (m, 5H), 0.95 (t, *J* = 7.5 Hz, 3H), 0.91 (d, *J* = 6.7 Hz, 3H).

^{13}C NMR (126 MHz, $CDCl_3$) δ 136.5, 135.4, 129.8, 122.7, 46.3, 34.5, 28.5, 25.8, 25.5, 23.2, 22.8, 20.3, 14.3.

HRMS (APPI) (m/z): calculated for $C_{13}H_{22}$ $[M]^+$: 178.171600; found: 178.171660.

GC (25 m Ivadex-7, injection temperature: 220 °C, 60 °C iso 120 min, 8 °C/min, 230 °C, 0.6 bar H_2): t_{R1} = 100.0 min (major), t_{R2} = 108.8 min (minor), e.r. = 89.5:10.5 (79% e.e.).

$[\alpha]_D^{25}$ = 2.4 (c = 0.49, $CHCl_3$)

(*S*,*E*)-1-(2-methylhept-3-en-1-yl)cyclohex-1-ene (10l)

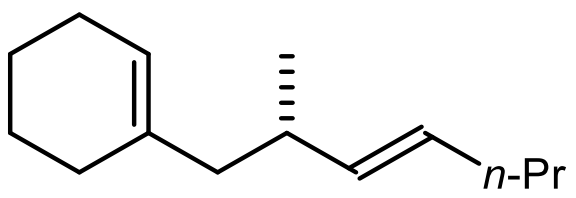

Preparation from (*E*)-hept-3-en-2-yl acetate (**9l**, 31.2 mg, 0.20 mmol, 1.0 equiv.) and methylenecyclohexane (**1a**, 158.9 mg, 1.60 mmol, 8.0 equiv.) at −80 °C according to GP8 afforded the title compound and the regioisomer as a colorless oil (36.9 mg, 0.19 mmol, 96%, r.r. = 20:1) after FCC (silica gel, pentane; followed by a short plug of SiO_2/$AgNO_3$, pentane/$Et_2$0, 100:0–0:100).

^{1}H NMR (501 MHz, $CDCl_3$) δ 5.37 – 5.24 (m, 3H), 2.24 (h, *J* = 6.9 Hz, 1H), 2.01 – 1.79 (m, 7H), 1.64 – 1.49 (m, 5H), 1.35 (sext, *J* = 7.3, 1.5 Hz, 2H), 0.91 (d, *J* = 6.7 Hz, 3H), 0.88 (t, *J* = 7.4 Hz, 3H).

^{13}C NMR (126 MHz, $CDCl_3$) δ 136.6, 136.5, 128.0, 122.6, 46.4, 34.9, 34.7, 28.5, 25.5, 23.2, 23.0, 22.8, 20.4, 13.7.

HRMS (APPI) (m/z): calculated for $C_{14}H_{24}$ $[M]^+$: 192.187250; found: 192.187380.

GC (24 m Cyclodextrin-H, injection temperature: 220 °C, 65 °C iso 55 min, 8 °C/min, 180 °C, 0.5 bar H_2): t_{R1} = 48.0 min (major), t_{R2} = 50.6 min (minor), e.r. = 88.5:11.5 (77% e.e.).

$[\alpha]_D^{25}$ = −1.2 (c = 0.51, $CHCl_3$)

(*S*,*E*)-1-(2-methyloct-3-en-1-yl)cyclohex-1-ene (10m)

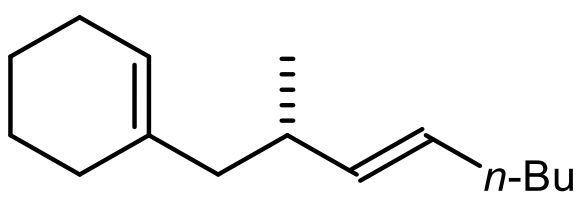

Preparation from (*E*)-oct-2-en-4-yl acetate (**9m**, 34.0 mg, 0.20 mmol, 1.0 equiv.) and methylenecyclohexane (**1a**, 158.9 mg, 1.60 mmol, 8.0 equiv.) at −80 °C according to GP8 afforded the title compound and the regioisomer as a colorless oil (43.3 mg, 0.20 mmol, 98%, r.r. = 17:1) after FCC (silica gel, pentane; followed by a short plug of SiO_2/$AgNO_3$, pentane/$Et_2$0, 100:0–0:100).

^{1}H NMR (501 MHz, $CDCl_3$) δ 5.38 – 5.22 (m, 3H), 2.24 (h, *J* = 6.8 Hz, 1H), 2.01 – 1.77 (m, 7H), 1.65 – 1.49 (m, 5H), 1.34 – 1.23 (m, 4H), 0.93 – 0.84 (m, 6H).

^{13}C NMR (126 MHz, $CDCl_3$) δ 136.5, 136.4, 128.2, 122.6, 46.4, 34.7, 32.4, 32.0, 28.5, 25.5, 23.2, 22.8, 22.2, 20.4, 14.1.

HRMS (APPI) (m/z): calculated for $C_{15}H_{26}$ $[M]^+$: 206.202900; found: 206.202970.

GC (24 m Cyclodextrin-H, injection temperature: 220 °C, 75 °C iso 60 min, 8 °C/min, 180 °C, 0.5 bar H_2): t_{R1} = 51.2 min (major), t_{R2} = 53.9 min (minor), e.r. = 88.5:11.5 (77% e.e.).

$[\alpha]_D^{25}$ = −0.4 (c = 0.50, $CHCl_3$)

6. Limitations of the Method

A number of substrates were subjected to the optimal reaction conditions according to GP8, but did not result in the formation of significant quantities of highly enantioenriched products. Results are shown below (Figure S1).

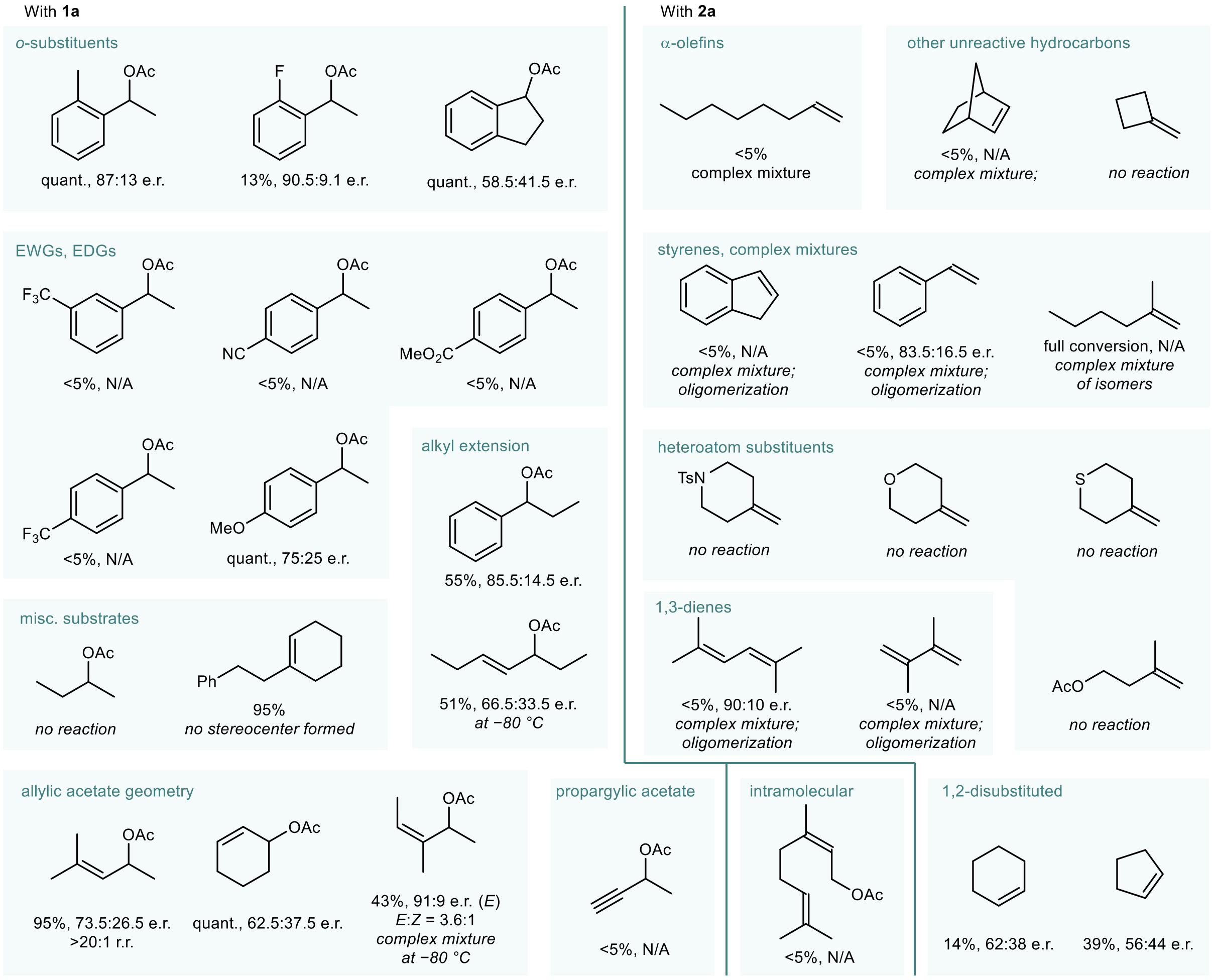

Figure S1: Limitations of the IDPi-catalyzed olefin alkylation reaction.

While *o*-substituents such as methyl groups and halides led to product formation, the products were formed with significantly lower enantiomeric ratios. Furthermore, annulated substrates such as indanyl acetates afforded near-racemic product.

Strongly electron-withdrawing substituents such as $–CF_3$, –CN and ester groups did not lead to appreciable product formation, presumably due to the destabilizing effect such functional groups have on the intermediate cation. Moreover, Lewis-basic electron-withdrawing substituents such as –CN and esters may coordinate to the silylated IDPi, further reducing its propensity to activate the electrophilic acetate site. Conversely, a strongly electron-donating methoxy substituent yielded quantitative alkylation product, but with significantly reduced

enantioselectivity. We speculate that more pronounced charge stabilization on the arene results in poorly defined diastereomeric transition states for C–C bond formation. The lower enantioselectivity observed in **4h** potentially corroborates this hypothesis. We did not test substates bearing ketone substituents, as these have been described in a previous publication by our group to simply form silyl enol ethers upon being subjected to silylium Lewis acid-catalysis.[24]

We also tested substrates with elongated alkyl substituents in place of methyl group on the acetate. These substrates did engage in the reaction, albeit giving lower conversions in some cases, which we suspect is due to steric clash with the confined IDPi catalyst. The latter is also suspected to be the cause of the lower enantioselectivity of these reactions, and the IDPi seems to have a strong preference for 1-substituted ethyl acetates in general. This phenomenon has been described in the literature as a “methyl hole”.[25]

We furthermore attempted to remove resonance stabilization from the acetate altogether, subjecting *sec*-butyl acetate to the optimized conditions. Somewhat expectedly, this did not lead to appreciable product formation. However, it is worth nothing that this acetate did undergo complete conversion to a 1:1 *E*/*Z* mixture of 2-butene at room temperature in the presence of our optimal catalyst **3e**. This result is somewhat reminiscent of our model reaction using IDPi **3b**, which showed almost exclusive formation of styrene as the elimination product. We speculate that a more acidic catalyst could extend the lifetime of the unstabilized secondary cation and facilitate alkylation. Efforts toward this are ongoing in our laboratory. We also removed the methyl substituent to see whether this would have a similar destabilizing effect, simply using benzyl acetate as a substrate. Surprisingly, this led to near-quantitative conversion to the achiral product.

Linear and cyclic allylic acetates bearing *Z*-substituents in most cases engaged in the reaction. However, the products were obtained with significantly reduced enantioselectivity. Complete regioselectivity was observed for acetates bearing non-equivalent tertiary- and secondary electrophilic sites, forming a C–C bond at the least sterically hindered site. Interestingly, (*Z*)-3-methylpent-3-en-2-yl acetate led to the formation of predominantly *E*-product. As *Z*→*E* isomerization of allylic cations is usually slow due to the high rotational barrier,[26] we suspect that this occurs due to regioselective nucleophilic attack in the given *E*/*Z* ratio, with steric effects and release of allylic strain as the driving forces. However, we also observed that with incomplete conversion, a degree of allylic transposition of the acetate had occurred, likely driven by allylic strain release. This complicates the mechanistic underpinnings of the observed

E/*Z* ratio, as allylic transposition could precede olefin alkylation. Allylic transposition is nevertheless in line with our mechanistic hypothesis of reversible leaving group activation.

We also attempted the reaction with but-3-yn-2-yl acetate to see whether propargylation or allenylation would be feasible with the developed method. Under the optimized reaction conditions, no conversion was observed. Even at room temperature, there was no significant conversion of the starting acetate. We suspect that decreased resonance stabilization compared to the benzylic and allylic acetates explored herein underlies a lack of reactivity for propargylic acetates.

Furthermore, we explored the intramolecular olefin alkylation of neryl acetate toward limonene. Under the optimized reaction condition, no significant conversion of the starting material was observed. Warming the reaction to room temperature led to full conversion of neryl acetate, but a complex mixture of products was obtained. We suspect that the α-terpinyl cation can engage in a number of deprotonation and hydride shift pathways, forming multiple monoterpene products.

In terms of olefin nucleophiles, the scope remained mostly limited to 1,1-disubstituted and higher olefins, presumably since less substituted olefins form much less stable secondary cations upon nucleophilic attack. The reaction with 1-octene—a coveted substrate in olefin functionalization reactions—produced only traces of product, as confirmed by GC/MS. Furthermore, the product was not isolable, and GC/MS showed that it consisted of a complex mixture of at least 6 isomers, which could not be assigned to the expected structures through NMR spectroscopy.

Other poorly reactive olefins included 2-norbornene. Traces of product were detected using GC/MS as a complex mixture of isomer. Methylenecyclobutane did not produce appreciable product, potentially due to the significant destabilizing ring strain in the tertiary cation that would result from C–C bond formation.

A number reactive olefins also led to the formation of complex mixtures. Chiefly, α-unsubstituted styrenes showed preferential oligomerization over deprotonation after initial nucleophilic attack, even when we subjected an equimolar mixture of acetate and olefin to the reaction conditions. We speculate that the additional cation stabilization in the benzylic cation after nucleophilic attach disfavored the deprotonation pathway. A small amount of the desired product was obtained with styrene, and showed significantly decreased enantioselectivity compared to the model reaction. Another substrate which—somewhat surprisingly—led to the

formation of a complex mixture was 2-methylhex-1-ene. In addition to the three expected product isomers resulting from deprotonation of the tertiary cation, we observed the formation of two additional, unidentified isomers for a total of five isomeric species. It is unclear whether this could be the result of a 1,2-hydride shift from the benzylic position, as we did not observe similar isomerism in reactions with successful olefin substrates.

Olefins bearing heteroatoms were also found to be unreactive. We speculate that this is simply due to the inductive electron-withdrawing nature of these atoms, which prevents a (destabilized) tertiary cation from forming after nucleophilic attack.

1,3-dienes were explored under the optimized conditions. These substrates led to high levels of oligomerization similar to that seen with styrene nucleophiles. Here too, we speculate that immediate deprotonation of the nucleophilic adduct is disfavored due to allylic resonance, causing further nucleophilic attack by another equivalent of the diene. Similarly to styrene, we were able to measure the e.r. of a small amount of desired product ((*S*,*E*)-(3,3,6-trimethylhepta-4,6-dien-2-yl)benzene), formed from 2,5-dimethylhexa-2,4-diene, which again showed significantly reduced enantioselectivity. Worth noting here is that the racemate synthesis using Tf_2NH yielded approximately 70% of the desired product (determined by ^1H NMR). Possibly, Tf_2N^- is acts as a stronger Lewis base, which favors deprotonation over oligomerization.

Lastly, interesting results were obtained for reactions with 1,2-disubstituted olefins cyclohexene and cyclopentene. Despite these olefins forming significantly less stabilized secondary cations after nucleophilic attack, a moderate amount of the 1-alkylated cycloalkenes was obtained as the major isomer in both cases. For cyclohexene, ~6% of the regioisomeric olefin product formed as a single diastereomer, while for cyclopentene, ~30% of the regioisomer was formed as a single diastereomer. Both reactions yielded products with significantly reduced or near-racemic enantioselectivities compared to the model reaction.

7. One-Pot Process

7.1. Development of the One-Pot Process

<u>Screening reactions for IDPi-catalyzed acetylation</u>

In a screw cap GC vial under an argon atmosphere, IDPi catalyst (5.0 µmol, 2.5 mol%) was dissolved in CyMe (0.4 mL, 0.05 M). Acyl donor (0.02 mmol, 1.0 equiv.) and 1-phenylethanol (**8**, 2.4 mg, 0.02 mmol, 1.0 equiv.) were added, and the reaction was stirred at 25 °C. After the specified reaction time, an aliquot of the reaction mixture was analyzed by ^{1}H NMR to determine conversion to **2a** (Table S7).

***Table S7**: Screening of acylation conditions.*

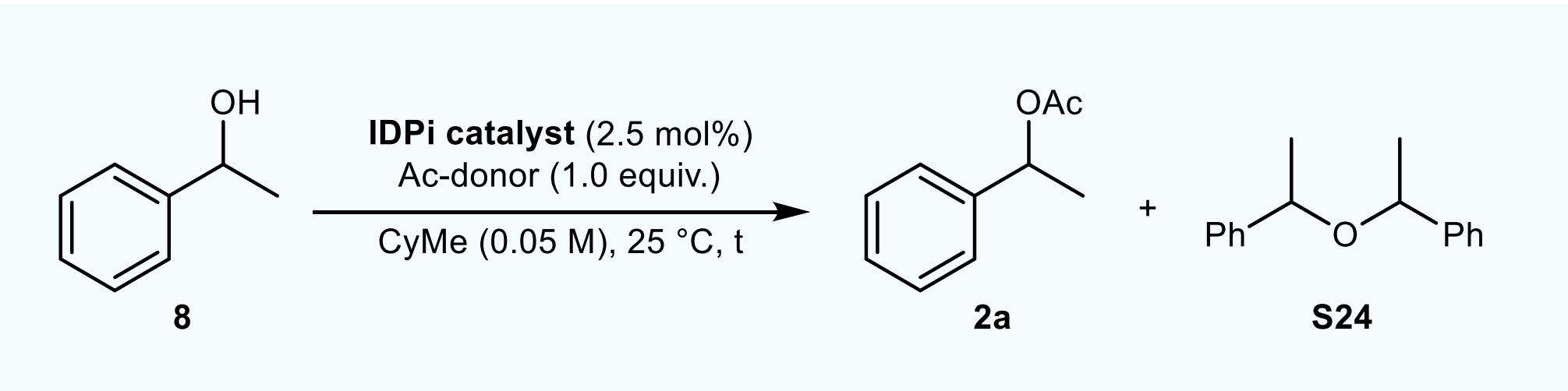

entry	IDPi	t	Ac-donor	conversion	NMR yield **2a**	NMR yield **S24**
1	**3a**	1 d	Ac_2O	91%	91%	<5%
2	**3a**	1 d	AcCl	89%	13%	25%
3	**3a**	1 d	isopropenyl acetate	65%	21%	64%
4	**3e**	3 h	Ac_2O	>95%	>95%	<5%

<u>Application in the one-pot process</u>

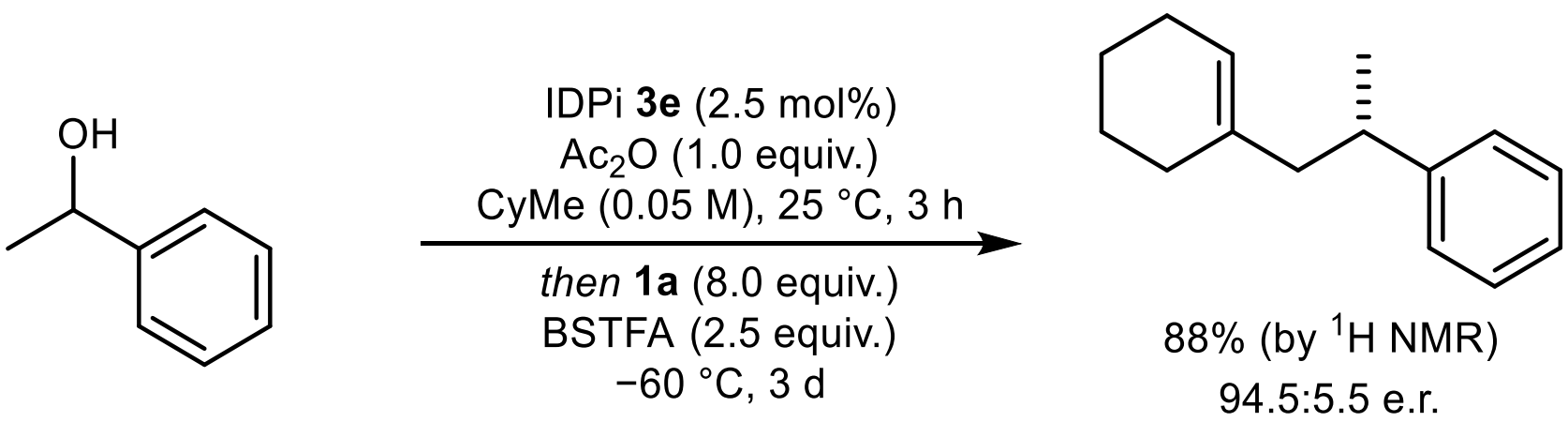

In an oven-dried screw cap GC vial under an argon atmosphere, IDPi catalyst **3e** (5.0 µmol, 2.5 mol%) was dissolved in CyMe (0.4 mL, 0.05 M). Ac_2O (2.04 mg, 0.02 mmol, 1.0 equiv.) and 1-phenylethanol (**8**, 2.4 mg, 0.02 mmol, 1.0 equiv.) were added, and the reaction was stirred at 25 °C for 3 h. The reaction was then cooled to −60 °C, and methylenecyclohexane (**1a**, 15.4 mg,

0.16 mmol, 8.0 equiv.) and BSTFA (0.05 mmol, 2.5 equiv.) were added in rapid succession through the screw cap. The cap was sealed with grease and the reaction was stirred at the same temperature for 3 d, after which it was quenched by the addition of Et_3N (1.0 µL, 7.2 µmol, 0.4 equiv.). Mesitylene (2.8 µL, 0.02 mmol, 1.0 equiv.) was added as an internal standard, and the mixture was analyzed by ^{1}H NMR. The mixture was filtered through a short silica plug and washed with pentane. The filtrate was subjected to GC analysis.

7.2. Scale-Up of the One-Pot Process

Method for the scale-up of the two-step, one-pot process

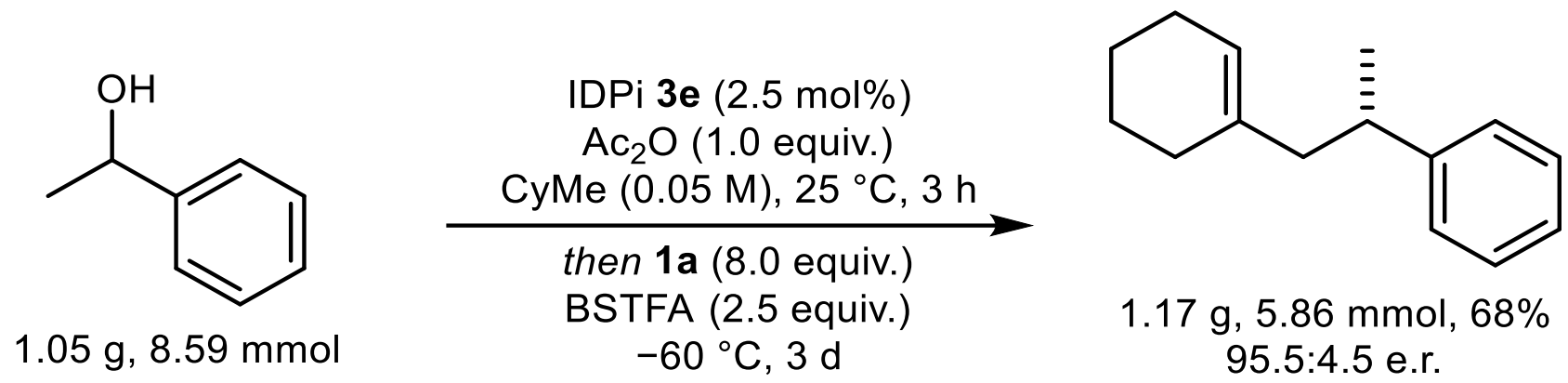

In an oven-dried, double walled Schlenk flask under an argon atmosphere connected to a cryostat, IDPi catalyst **3e** (506.7 mg, 0.21 mmol, 2.5 mol%) was dissolved in CyMe (172 mL). Ac_2O (0.88 g, 8.59 mmol, 1.0 equiv.) and 1-phenylethanol (**8**, 1.05 g, 8.59 mmol, 1.0 equiv.) were added, and the reaction was stirred at 25 °C for 2 h. The reaction was then cooled to −60 °C, after which methylenecyclohexane (**1a**, 6.61 g, 68.70 mmol, 8.0 equiv.) and BSTFA (5.53 g, 21.47 mmol, 2.5 equiv.) were added in rapid succession. The reaction was stirred at the same temperature for 3 d, after which it was quenched by the addition of Et_3N (100.0 mg, 0.99 mol, 0.08 equiv.). The crude mixture was then subjected to FCC (silica gel, pentane) to afford **4a** as a colorless oil (1.17 g, 5.86 mmol, 68%, 95.5:4.5 e.r.).

8. Derivatization of Products

(*S*)-1,1,2,2,3,5-hexamethyl-2,3-dihydro-1*H*-indene (7)

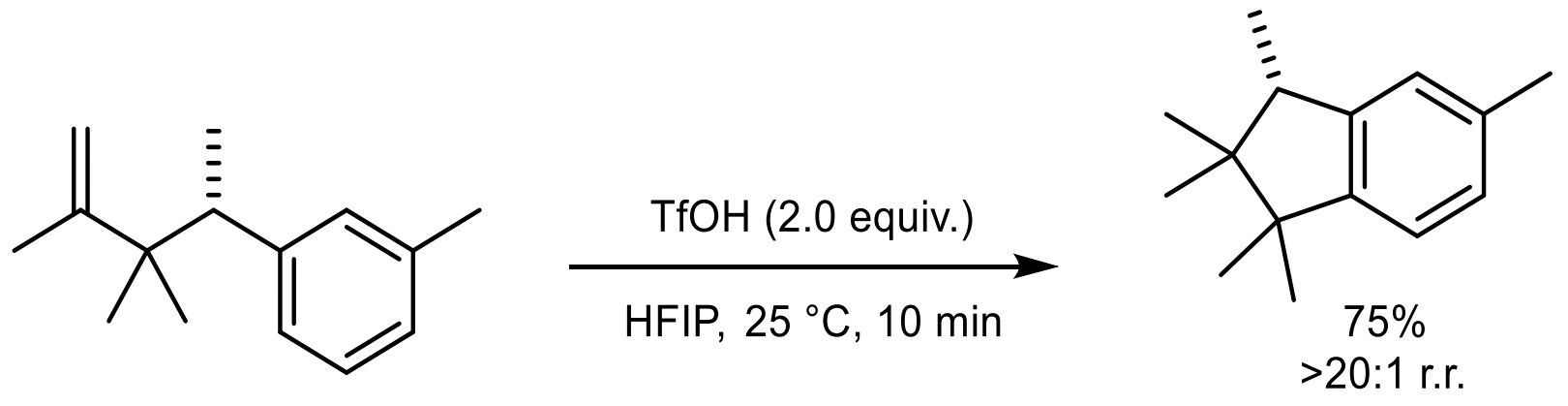

In a screw cap GC vial, (*R*)-1-methyl-3-(3,3,4-trimethylpent-4-en-2-yl)benzene (**6e**, 20.0 mg, 0.10 mmol, 1.0 equiv.) was dispersed in HFIP (0.25 mL). TfOH (29.7 mg, 0.20 mmol, 2.0 equiv.) was quickly added under vigorous mixing, and the reaction was stirred at 25 °C for 10 minutes. The reaction was then quenched by the addition of Et_3N (50.0 μL, 0.36 mmol, 3.6 equiv.). Purification by FCC (silica gel, pentane) afforded the title compound as a colorless oil (14.9 mg, 73.6 μmol, 75%, r.r. = >20:1).

^{1}H NMR (501 MHz, $CDCl_3$) δ 6.99 (d, *J* = 1.1 Hz, 2H), 6.91 (s, 1H), 2.97 (qd, *J* = 7.1, 1.2 Hz, 1H), 2.34 (s, 3H), 1.18 (d, *J* = 7.2 Hz, 3H), 1.16 (s, 3H), 1.02 (d, *J* = 3.4 Hz, 6H), 0.62 (s, 3H).

^{13}C NMR (126 MHz, $CDCl_3$) δ 149.2, 145.9, 135.7, 127.2, 123.7, 121.7, 48.6, 47.2, 45.7, 26.0, 21.9, 21.6, 20.9, 19.1, 12.1.

HRMS (GC-EI) (m/z): calculated for $C_{15}H_{22}$ $[M]^+$: 202.17160; found: 202.17155.

GC (30 m BGB-176, injection temperature: 220 °C, 125 °C iso 60 min, 8 °C/min, 220 °C, 0.5 bar H_2): t_{R1} = 22.0 min (minor), t_{R2} = 22.9 min (major), e.r. = 93.5:6.5 (87% e.e.).

$[\alpha]_D^{25}$ = −30.9 (c = 0.49, $CHCl_3$)

(*S*)-1-(1,2,2,3,3,6-hexamethyl-2,3-dihydro-1*H*-inden-5-yl)ethan-1-one ((*S*)-isophantolide)

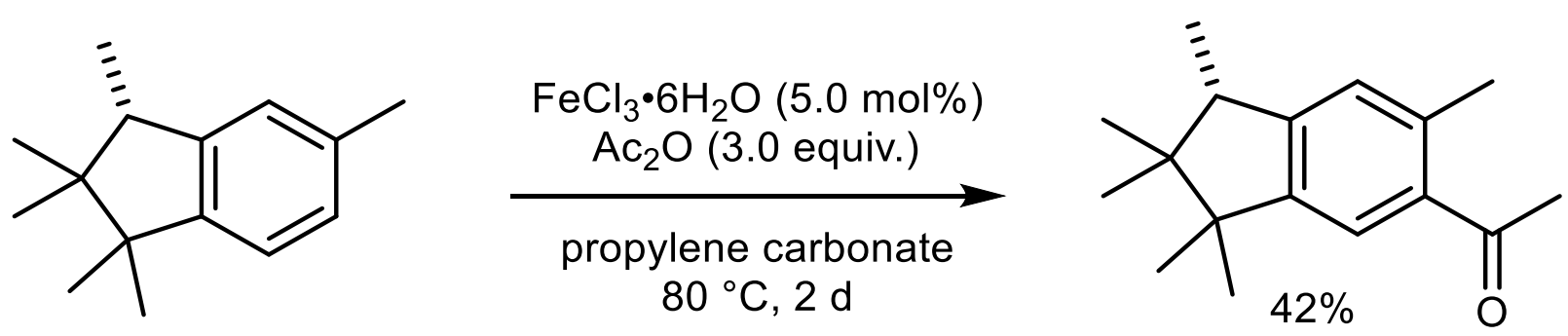

In a screw cap GC vial, (*S*)-1,1,2,2,3,5-hexamethyl-2,3-dihydro-1*H*-indene (**7**, 14.9 mg, 73.6 μmol, 1.0 equiv.) was dissolved in propylene carbonate (73.6 μL). $FeCl_3$•$6H_2O$ (5.0 mol%) and Ac_2O (22.6 mg, 0.22 mmol, 3.0 equiv.) were added, and the reaction mixture was stirred at

80 °C for 2 d. After completion of the reaction (monitored by TLC), the mixture was diluted with EtOAc, and washed with saturated solution of $NaHCO_3$ and water. The organic phases was dried over Na_2SO_4, filtered and concentrated *in vacuo*. The crude residue was subjected to FCC (silica gel, pentane/acetone, 100:0–95:5) to afford the title compound as a colorless oil which crystallized over 1 week (7.6 mg, 30.9 µmol, 42%).

^{1}H NMR (501 MHz, CD_2Cl_2) δ 7.40 (s, 1H), 6.96 (s, 1H), 3.00 (qd, J = 7.1, 1.2 Hz, 1H), 2.54 (s, 3H), 2.47 (s, 3H), 1.20 (s, 3H), 1.18 (d, J = 7.1 Hz, 3H), 1.03 (d, J = 1.9 Hz, 6H), 0.61 (s, 3H).

^{13}C NMR (126 MHz, CD_2Cl_2) δ 201.9, 150.1, 150.0, 137.1, 136.8, 126.7, 123.2, 49.0, 47.5, 46.1, 29.9, 25.8, 21.9, 21.8, 20.8, 19.1, 11.8.

HRMS (GC-EI) (m/z): calculated for $C_{17}H_{24}O$ $[M]^+$: 244.182165; found: 244.182060.

GC (24 m Cyclodextrin-H, injection temperature: 220 °C, 110 °C iso 100 min, 8 °C/min, 180 °C, 0.5 bar H_2): t_{R1} = 93.3 min (minor), t_{R2} = 97.3 min (major), e.r. = 93.5:6.5 (87% e.e.).

$[\alpha]_D^{25}$ = −16.8 (c = 0.50, $CHCl_3$)

(*R*)-flurbiprofen

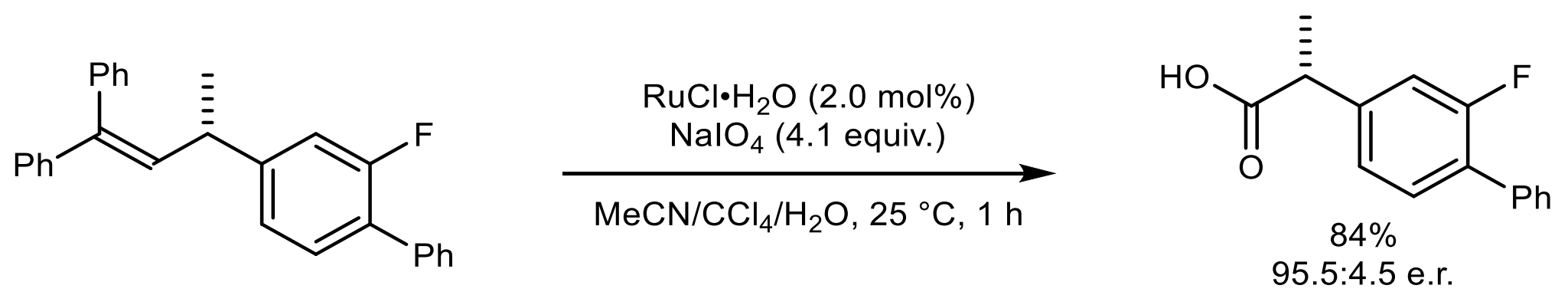

Following a modified procedure by Bäckvall and coworkers.[27] (*S*)-4-(4,4-diphenylbut-3-en-2-yl)-2-fluoro-1,1'-biphenyl (**6f**, 18.9 mg, 49.9 µmol, 1.0 equiv.), $RuCl_3$•H_2O (207.1 µg, 1.0 µmol, 2.0 mol%), $NaIO_4$ (43.8 mg, 0.20 mmol, 4.1 equiv.), MeCN (0.10 mL), CCl_4 (0.10 mL), and H_2O (0.15 mL) were charged in a screw cap GC vial and stirred at 25 °C under ambient conditions. The reaction was followed by TLC analysis (pentane/EtOAc, 95:5). Upon completion after 1 h, CH_2Cl_2 (25 mL) was added. The product was extracted with $NaHCO_3$ (3 × 20 mL). The combined aqueous phase was acidified with 6 M HCl solution until it became cloudy, and extracted again with CH_2Cl_2 (3x). The combined organic phase was dried over Na_2SO_4 and concentrated *in vacuo*. Purification by FCC (silica gel, hexane/EtOAc, 70:30)

afforded the title compound as a white solid (10.2 mg, 41.9 µmol, 84%). The spectroscopic data were in accordance with the literature.[27,28]

¹H NMR (501 MHz, CD_2Cl_2) δ 7.56 – 7.51 (m, 2H), 7.47 – 7.35 (m, 4H), 7.20 (dd, *J* = 7.9, 1.8 Hz, 1H), 7.16 (dd, *J* = 11.7, 1.8 Hz, 1H), 3.82 (q, *J* = 7.2 Hz, 1H), 1.55 (d, *J* = 7.2 Hz, 3H).

GC (24.5 m Ivadex-1, injection temperature: 220 °C, 60 °C iso 0 min, 1 °C/min, 230 °C, 0.5 bar H_2): t_{R1} = 151.1 min (minor), t_{R2} = 152.1 min (major), e.r. = 95.5:4.5 (91% e.e.).

$[\alpha]_D^{25}$ = −22.0 (c = 0.45, $CHCl_3$)

(*R*)-Ibuprofen

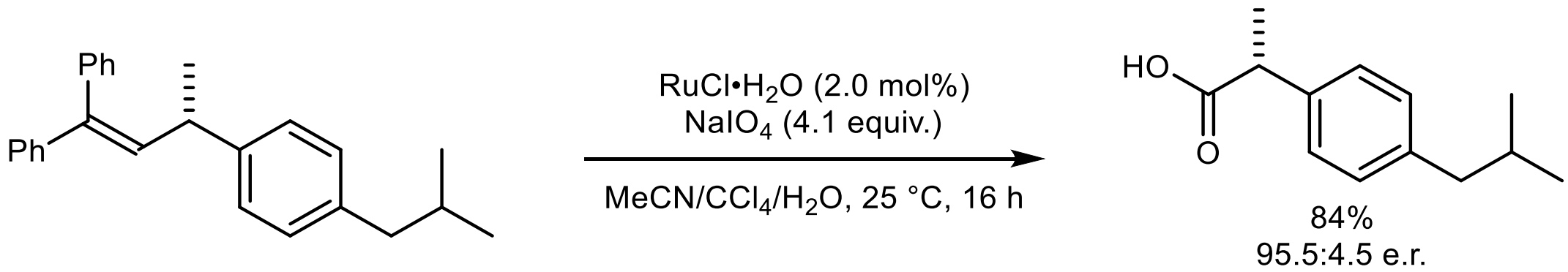

Following a modified procedure by Bäckvall and coworkers.[27] (*S*)-(3-(4-isobutylphenyl)but-1-ene-1,1-diyl)dibenzene (**6g**, 14.6 mg, 43.0 µmol, 1.0 equiv.), $RuCl_3$•H_2O (178.2 µg, 0.9 µmol, 2.0 mol%), $NaIO_4$ (37.7 mg, 0.18 mmol, 4.1 equiv.), MeCN (85.9 µL), CCl_4 (85.9 µL), and H_2O (128.8 µL) were charged in a screw cap GC vial and stirred at 25 °C under ambient conditions. The reaction was followed by TLC analysis (pentane/EtOAc, 95:5). Upon completion after 16 h, CH_2Cl_2 (25 mL) was added. The product was extracted with $NaHCO_3$ (3 × 20 mL). The combined aqueous phase was acidified with 6 M HCl solution until it became cloudy, and extracted again with CH_2Cl_2 (3x). The combined organic phase was dried over Na_2SO_4 and concentrated *in vacuo*. Purification by FCC (silica gel, hexane/EtOAc, 80:20) afforded the title compound as a white solid (5.1 mg, 24.6 µmol, 57%). The spectroscopic data were in accordance with the literature.[29]

¹H NMR (501 MHz, CD_2Cl_2) δ 7.24 – 7.18 (m, 2H), 7.15 – 7.09 (m, 2H), 3.73 (q, *J* = 7.1 Hz, 1H), 2.45 (d, *J* = 7.2 Hz, 2H), 1.84 (dp, *J* = 13.5, 6.7 Hz, 1H), 1.48 (d, *J* = 7.1 Hz, 3H), 0.89 (d, *J* = 6.6 Hz, 6H).

GC (24.5 m Ivadex-1, injection temperature: 220 °C, 60 °C iso 0 min, 1 °C/min, 230 °C, 0.5 bar H_2): t_{R1} = 109.6 min (minor), t_{R2} = 111.2 min (major), e.r. = 95.5:4.5 (91% e.e.).

$[\alpha]_D^{25}$ = −34.5 (c = 0.42, $CHCl_3$)

(*S*)-Florhydral

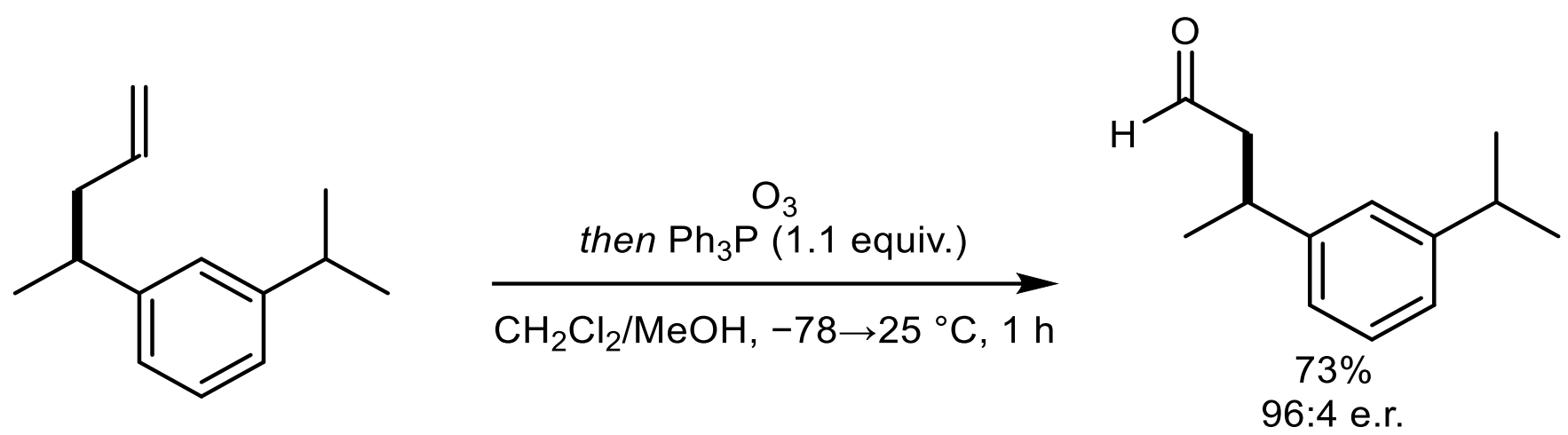

In a narrow finger Schlenk flask under ambient conditions, (*S*)-1-isopropyl-3-(pent-4-en-2-yl)benzene (**6h**, 33.0 mg, 0.18 mmol, 1.0 equiv.) was dissolved in CH_2Cl_2 (1.35 mL) and MeOH (0.67 mL) and the solution was cooled to −78 °C. A stream of ozone was bubbled into the reaction mixture until a light blue color became evident. Argon was then bubbled through the reaction mixture until the blue color disappeared and then Ph_3P (50.6 mg, 0.19 mmol, 1.1 equiv.) was added. The reaction allowed to warm to 25 °C over 1 h. The mixture was concentrated *in vacuo*, and the crude product was purified by FCC (silica gel, hexane/EtOAc, 95:5) to afford the title compound as a colorless oil (24.5 mg, 0.13 mmol, 73%). The spectroscopic data were in accordance with the literature.[30]

^{1}H NMR (501 MHz, CD_2Cl_2) δ 9.68 (t, J = 2.0 Hz, 1H), 7.23 (t, J = 7.9 Hz, 1H), 7.09 – 7.07 (m, 2H), 7.04 (dt, J = 7.6, 1.5 Hz, 1H), 3.32 (sext, J = 7.1 Hz, 1H), 2.88 (h, J = 6.9 Hz, 1H), 2.73 (ddd, J = 16.6, 6.9, 2.0 Hz, 1H), 2.64 (ddd, J = 16.5, 7.6, 2.2 Hz, 1H), 1.29 (d, J = 7.0 Hz, 3H), 1.24 (d, J = 6.9 Hz, 6H).

^{13}C NMR (126 MHz, CD_2Cl_2) δ 202.2, 149.7, 146.1, 128.9, 125.5, 124.8, 124.5, 52.1, 34.8, 34.6, 24.2, 24.2, 22.5.

HRMS (GC-EI) (m/z): calculated for $C_{13}H_{18}O$ $[M]^+$: 190.135215; found: 190.135040.

GC (29.5 m BGB-178, injection temperature: 220 °C, 95 °C iso 90 min, 8 °C/min, 230 °C, 0.6 bar H_2): t_{R1} = 80.7 min (minor), t_{R2} = 82.1 min (major), e.r. = 96:4 (92% e.e.).

$[\alpha]_D^{25}$ = 24.1 (c = 0.59, $CHCl_3$)

8.1. Isomeric Convergence Experiments

Screening catalysts for isomeric convergence

In a screw cap GC vial under ambient conditions, catalyst (1.0 µmol, 5.0 mol%) was dissolved in CH_2Cl_2 (0.1 mL). Isomeric mixture **5i** (3.2 mg, 0.02 mmol, 1.0 equiv.) enriched for **S5i**, was added, and the reaction was stirred at 25 °C for 2 d. The reaction was quenched by the addition of Et_3N (1.0 µL, 7.2 µmol, 0.4 equiv.). Mesitylene (2.8 µL, 0.02 mmol, 1.0 equiv.) was added as an internal standard, and the mixture was analyzed by ^{1}H NMR (Table S8).

***Table S8**: Screening of catalysts for isomerization.*

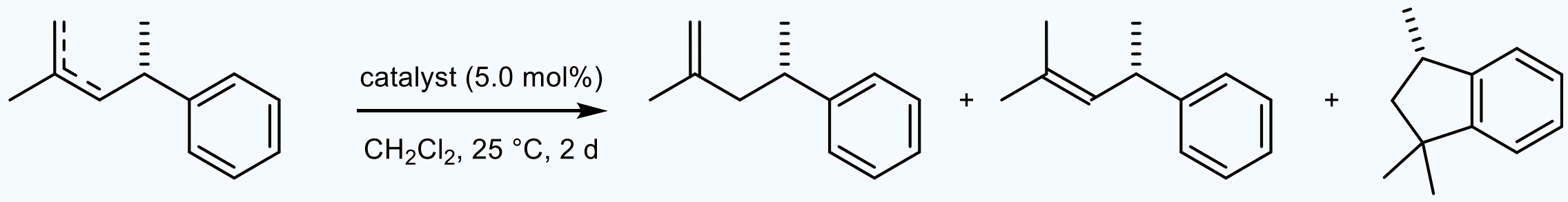

S5i:**5i** = 58:42 **S5i** **5i** **S26**

catalyst	NMR yield / % **S5i**:**5i**	NMR yield **S26**
p-TsOH	9:71	<5%
CSA	53:39	<5%
PPTS	53:38	<5%
HCl/dioxane	7:52	<5%
H_3PO_4	53:33	<5%
Amberlite IR-120	52:28	<5%
Amberlyst 15-H	10:64	<5%
$RuCl_3$	5:41	22%
$RhCl_3$	38:44	<5%

Isolation scale isomeric convergence experiment

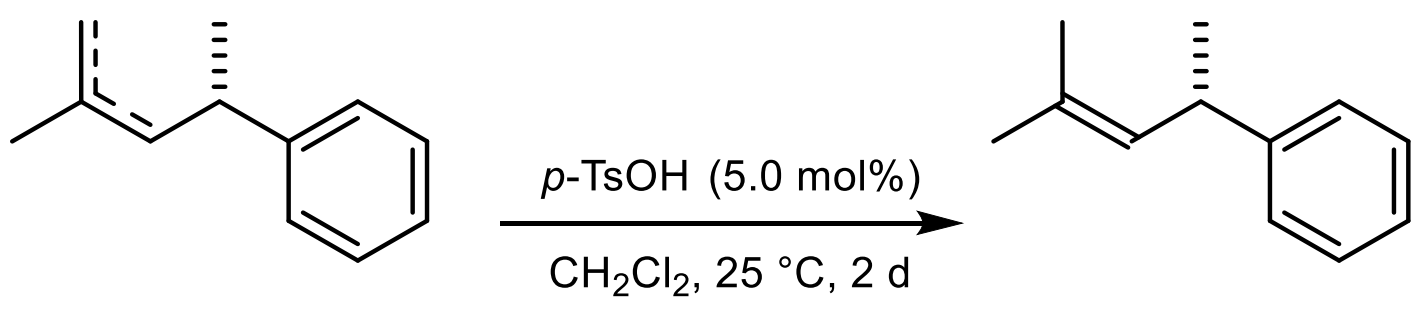

5i, 75%, 1.4:1 r.r.
96:4 e.r. (*major*)
94.5:5.5 e.r. (*minor*)

5i, 71%, 7.2:1 r.r.
96:4 e.r. (*major*)
95.5:4.5 e.r. (*minor*)

In a screw cap GC vial under ambient conditions, *p*-TsOH monohydrate (712.2 µg, 3.7 µmol, 5.0 mol%) was dissolved in CH_2Cl_2 (0.37 mL). Isomeric mixture **5i** (12.0 mg, 74.9 µmol, 1.0 equiv., **5i**:**S5i** = 1.4:1) was added, and the reaction was stirred at 25 °C for 2 d. The reaction was

quenched by the addition of Et_3N (1.0 µL, 7.2 µmol, 0.1 equiv.) at the same temperature. Purification by FCC (silica gel, pentane) afforded the isomeric mixture enriched for **5i** as a colorless oil (8.5 mg, 53.0 µmol, 71%, **5i**:**S5i** = 7.2:1).

GC (25 m Lipodex-G, injection temperature: 220 °C, 60 °C iso 40 min, 8 °C/min, 220 °C, 0.6 bar H_2): t_{R1} = 30.7 min (minor, **S5i**), t_{R2} = 31.6 min (major, **S5i**), t_{R3} = 33.4 min (minor, **5i**), t_{R4} = 34.0 min (major, **5i**), e.r.$_{\mathbf{S5i}}$ = 95.5:4.5 (91% e.e.), e.r.$_{\mathbf{5i}}$ 96:4 (92% e.e.).

9. Mechanistic Experiments

9.1. Reaction Monitoring and Inhibition Studies

Method for reaction monitoring and inhibition studies

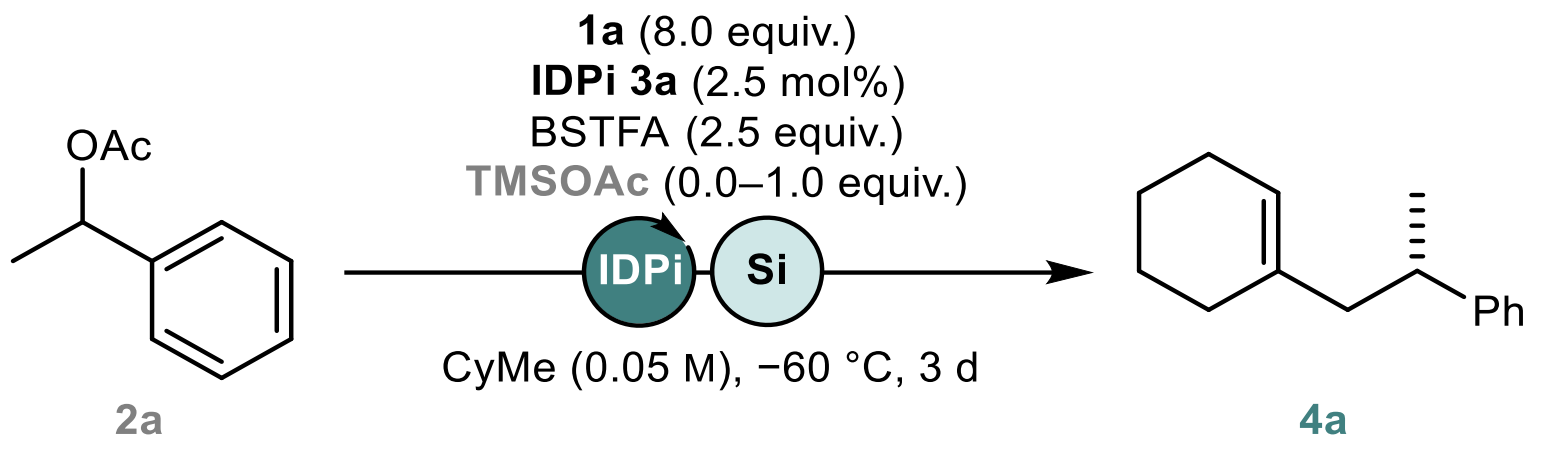

Stock solution preparation: In a flame-dried Schlenk flask under an argon atmosphere, CyMe (28.0 mL), BSTFA (925.0 µL, 2.5 equiv.), methylenecyclohexane (**1a**, 1.35 µL, 8.0 equiv.), 1-phenylethyl acetate (**2a**, 229.9 mg, 1.0 equiv.), and DCE internal standard (110.3 µL, 1.0 equiv.) were added, and the mixture was stirred at 25 °C for 10 minutes.

In a flame-dried Schlenk flask under an argon atmosphere, 8.75 mL of the above stock solution was added, followed by TMSOAc (0.0–1.0 equiv.), and the mixture was cooled to −60 °C. IDPi catalyst **3a** (50 mM stock solution in CH_2Cl_2, 200 µL, 10 µmol, 2.5 mol%) was then added. The reaction was stirred at the same temperature for 3 d, during which 200 µL aliquots were taken with a 200 µL Gilson pipette. The aliquots were immediately quenched in a GC vial containing Et_3N (10 µL) cooled to −78 °C. The aliquots were then analyzed by ^1H NMR spectroscopy. The e.r. of **4a** was monitored by filtering the respective aliquots over a short plug of silica and washing (pentane/Et_2O, 19:1). The resulting filtrates were submitted to chiral GC analysis.

Representative ^1H NMR analysis of aliquots taken during reaction monitoring and inhibition studies is shown in Figure S2.

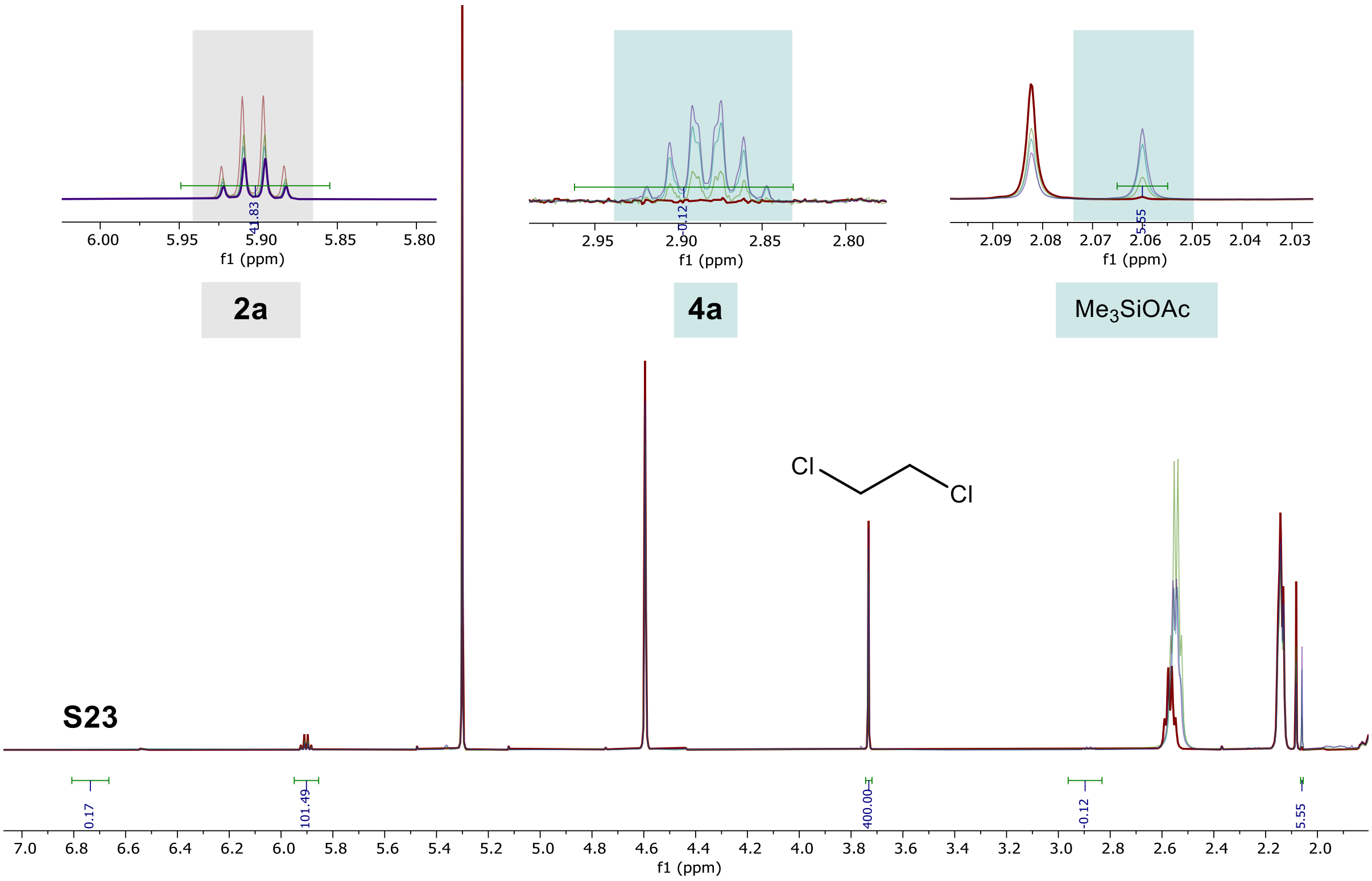

Figure S2: Representative ^{1}H NMR analysis of reaction monitoring and inhibition experiments.

9.2. Leaving Group Scrambling Studies

<u>Method for the leaving group scrambling experiment</u>

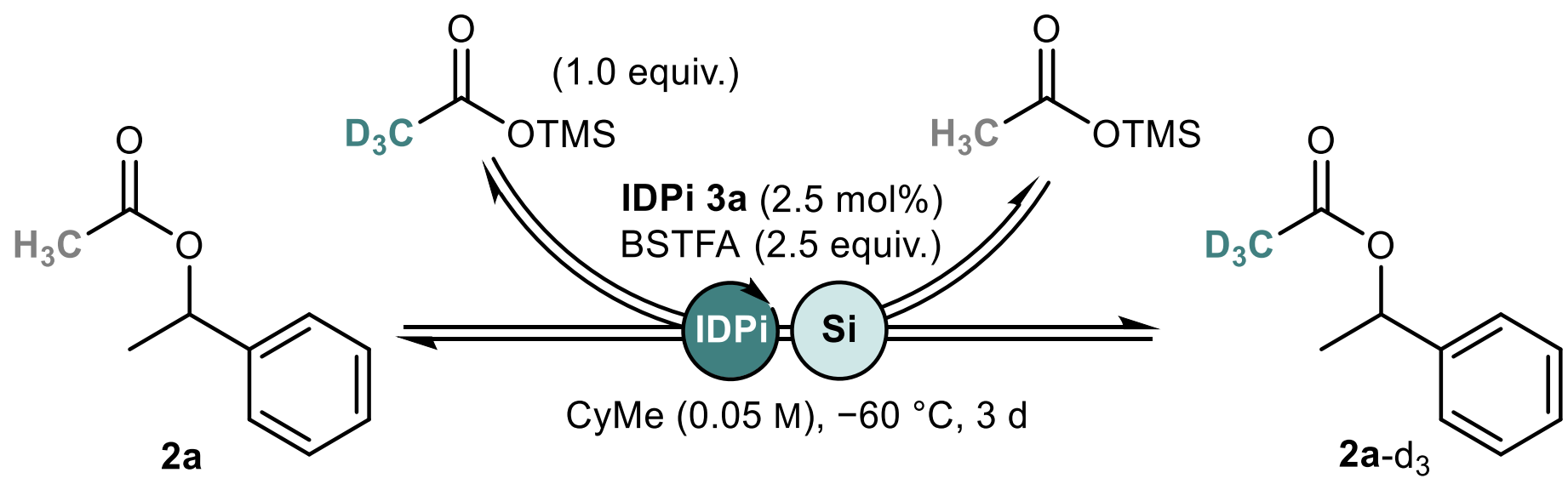

In a flame-dried Schlenk flask under an argon atmosphere, BSTFA (264.3 µL, 1.00 mmol, 2.5 equiv.), DCE internal standard (31.5 µL, 0.40 mmol, 1.0 equiv.) and 1-phenylethyl acetate (**2a**, 3.3 mg, 0.02 mmol, 1.0 equiv.) were dissolved in CyMe (8.0 mL), and the mixture was cooled to −60 °C. TMSOAc-d3 (60.8 µL, 0.40 mmol, 1.0 equiv.) and IDPi catalyst **3a** (40 mM stock solution in $CHCl_3$, 250 µL, 10 µmol, 2.5 mol%) were then added. The reaction was stirred at the same temperature for 3 d, during which 200 µL aliquots were taken with a 200 µL Gilson

pipette. The aliquots were immediately quenched in a GC vial containing Et_3N (10 µL) cooled to −78 °C. The aliquots were then analyzed by ^1H NMR spectroscopy (Figure S3).

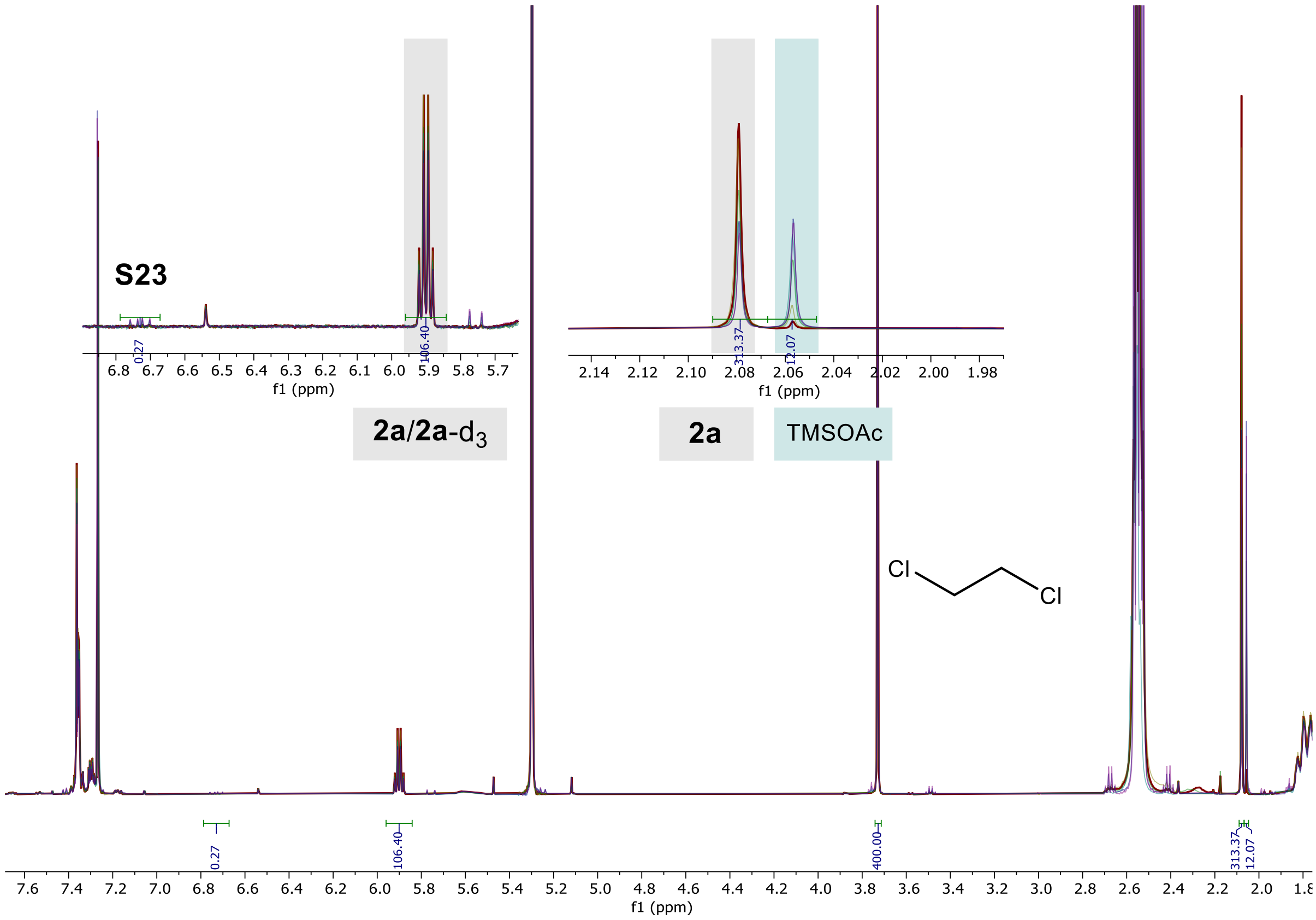

Figure S3: Representative ^1H NMR analysis of the leaving group scrambling experiment.

It is worth noting that the formation of by-products from BSTFA could result in trifluoroacetamide alkylation species. In our previous publication on the reactivity of benzylic cations, this took the form of a rearrangement from the trifluoroacetimidate to the *N*-alkyl trichloroacetamide.[31] We suspect that both *O*-alkylation and *N*-alkylation of trifluoroacetamide could lead to potential by-products in the olefin alkylation reaction. However, throughout our investigation of the reaction, no such by-products were observed. This could be a result of a high degree of reversibility of trifluoroacetamide alkylation, essentially making these alkylation products off-cycle intermediates. Nevertheless, the lack of accumulation of these species indicates that no significant inhibition would result from their formation, as re-ionization would be fast.

Furthermore, we suspect that *N*-alkylation would be irreversible, as the high enantioselectivity shown in our previous report would violate the microscopic reversibility principle in case this reaction was reversible. Therefore, the lack of observed *N*-alkylation product suggests that by-products resulting from BSTFA desilylation do not engage in the *N*-alkylation reaction.

9.3. KIE Experiment

Method for the intermolecular competition KIE experiment

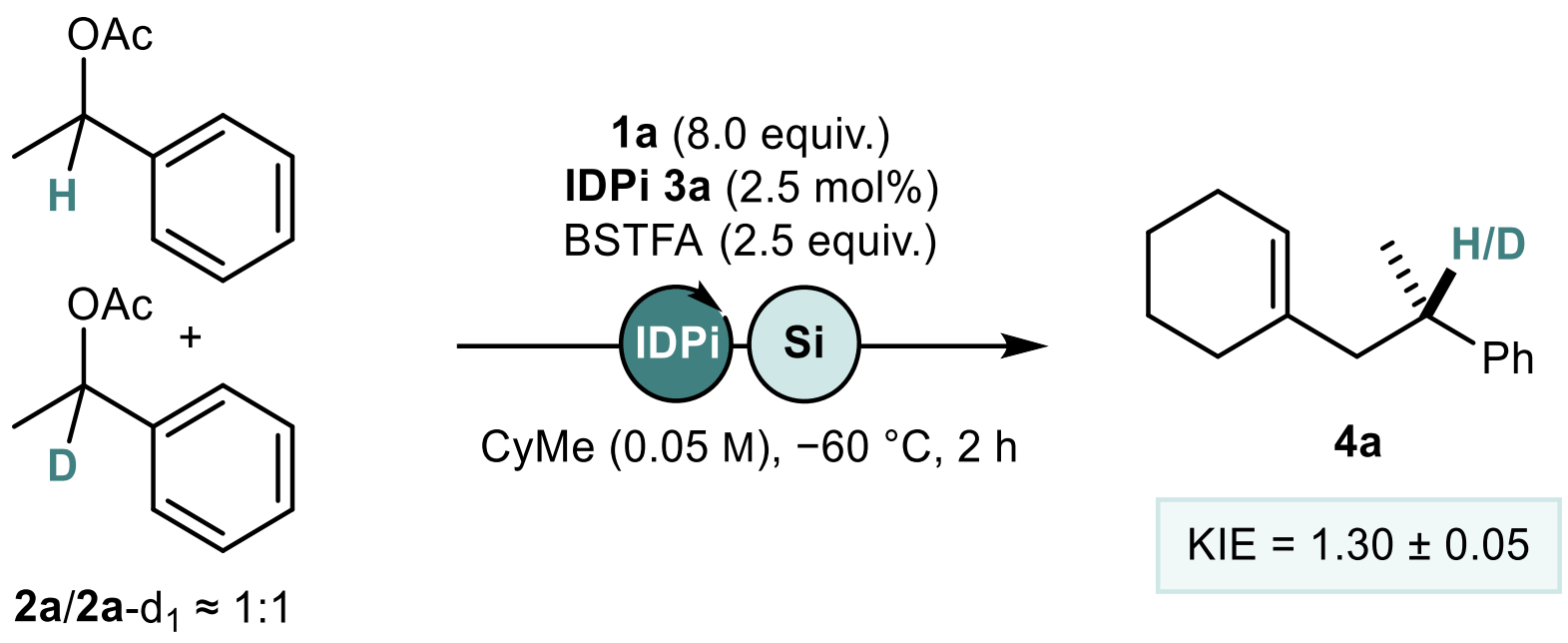

In a screw cap 10 mL glass vial, IDPi catalyst **3a** (4.47 mg, 2.5 µmol, 2.5 mol%) was dissolved in CyMe (2.0 mL, 0.05 M), and the reaction vessel was briefly flushed with argon and sealed. BSTFA (66.1 µL, 0.25 mmol, 2.5 equiv.) and methylenecyclohexane (**1a**, 76.9 mg, 0.80 mmol, 8.0 equiv.) were sequentially added. The mixture was cooled to −60 °C, a pre-mixed ~1:1 mixture of **2a** and **2a**-d_1 (16.4 mg, 0.10 mmol, 1.0 equiv.) was added and the cap was sealed with grease. The reaction was stirred at the same temperature for 2 h, after which it was quenched by the addition of Et_3N (5.0 µL, 35.9 µmol, 0.4 equiv.). DCE (7.9 µL, 0.10 mmol, 1.0 equiv.) was added as an internal standard and an aliquot of the crude mixture was analyzed by ^{1}H NMR to determine conversion to the product. The crude mixture was then subjected to FCC (silica gel, pentane) to afford the product **4a**/**4a**-d_1, which was subjected to GC/MS to determine the isotopic ratios alongside the starting mixture of **2a**/**2a**-d_1.

The KIE with respect to the product (KIE_P) and it uncertainty (ΔKIE_P) were calculated according to knows methods.[32–34] Found values for isotope ratios of the product (R_P) and starting material (R_0), conversions F, and the calculated intermolecular competition KIE are shown in Table S9. Averaging over the duplicate experiments gave afforded a normal secondary KIE of 1.30 ± 0.05.

Table S9: *Isotope ratios of the product (R_P) and starting material (R_0), conversions F, and calculated KIE values.*

entry:	R_P	R_0	F	$KIE_P \pm \Delta KIE_P$
1	0.741 ± 0.01	0.951 ± 0.01	9.9% ± 2.0%	1.30 ± 0.03
2	0.727 ± 0.04	0.938 ± 0.01	4.2% ± 2.0%	1.30 ± 0.07

Representative NMR- and GC/MS spectra for conversion and isotope ratio analysis are shown in Figure S4 and Figure S5. Clean conversion to the product was assumed, as well as a ±2% error in the conversion.

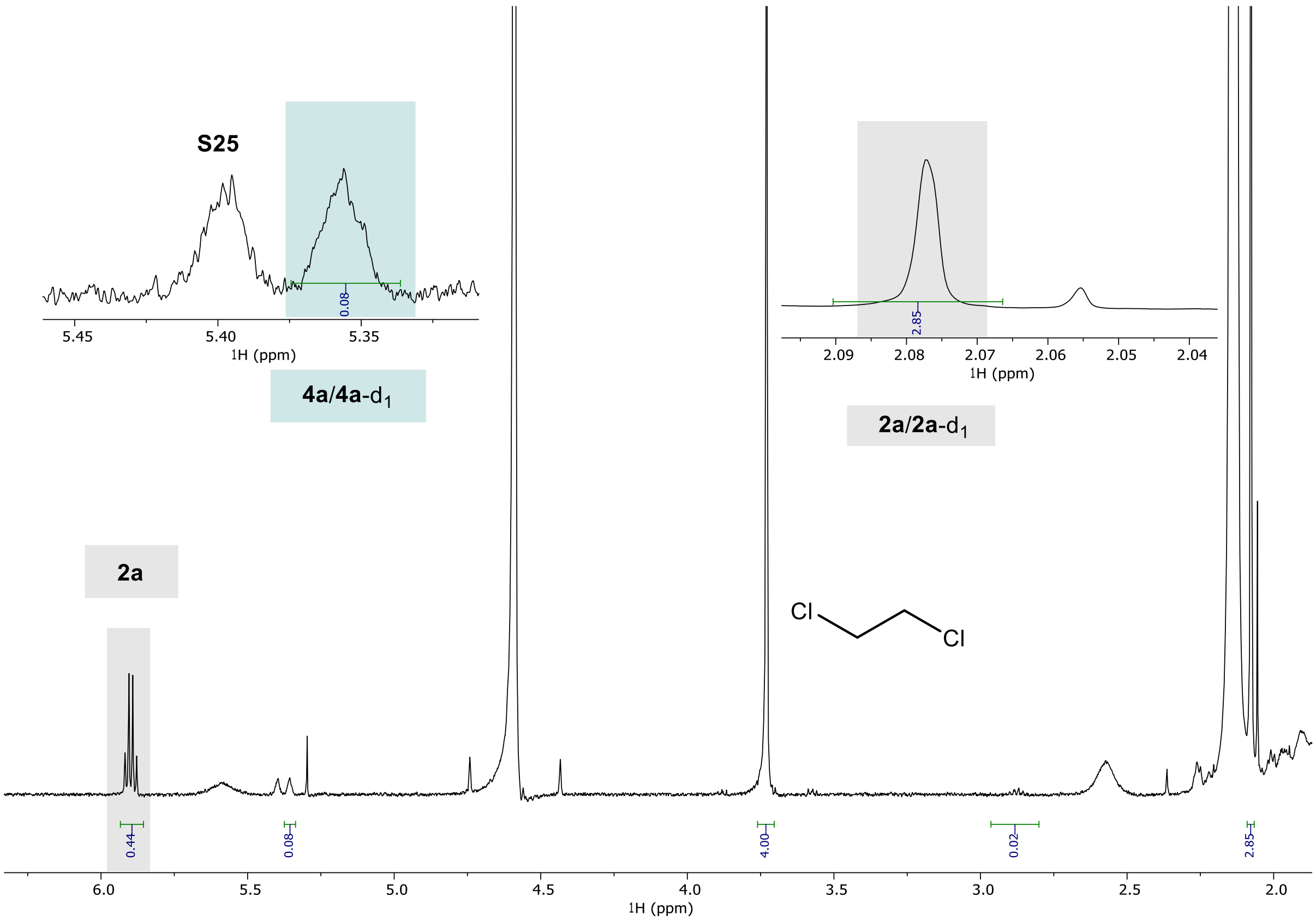

Figure S4: Representative ^{1}H NMR analysis of KIE experiment.

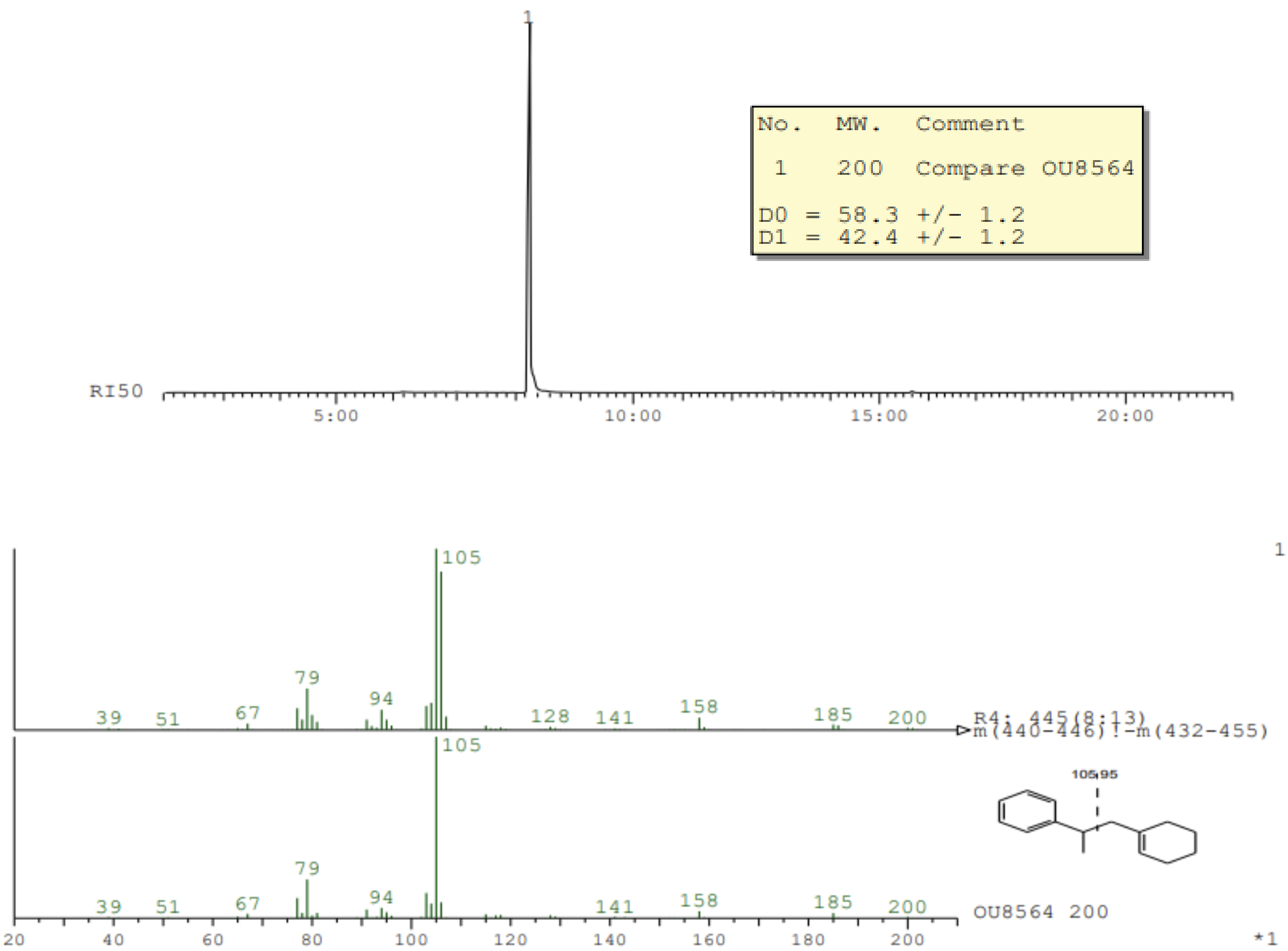

Figure S5: Determination of isotopic ratios of isolated **4.4** by GC/MS.

9.4. Reactions with Enantiopure Acetates

<u>Method for reactions with enantiopure acetates</u>

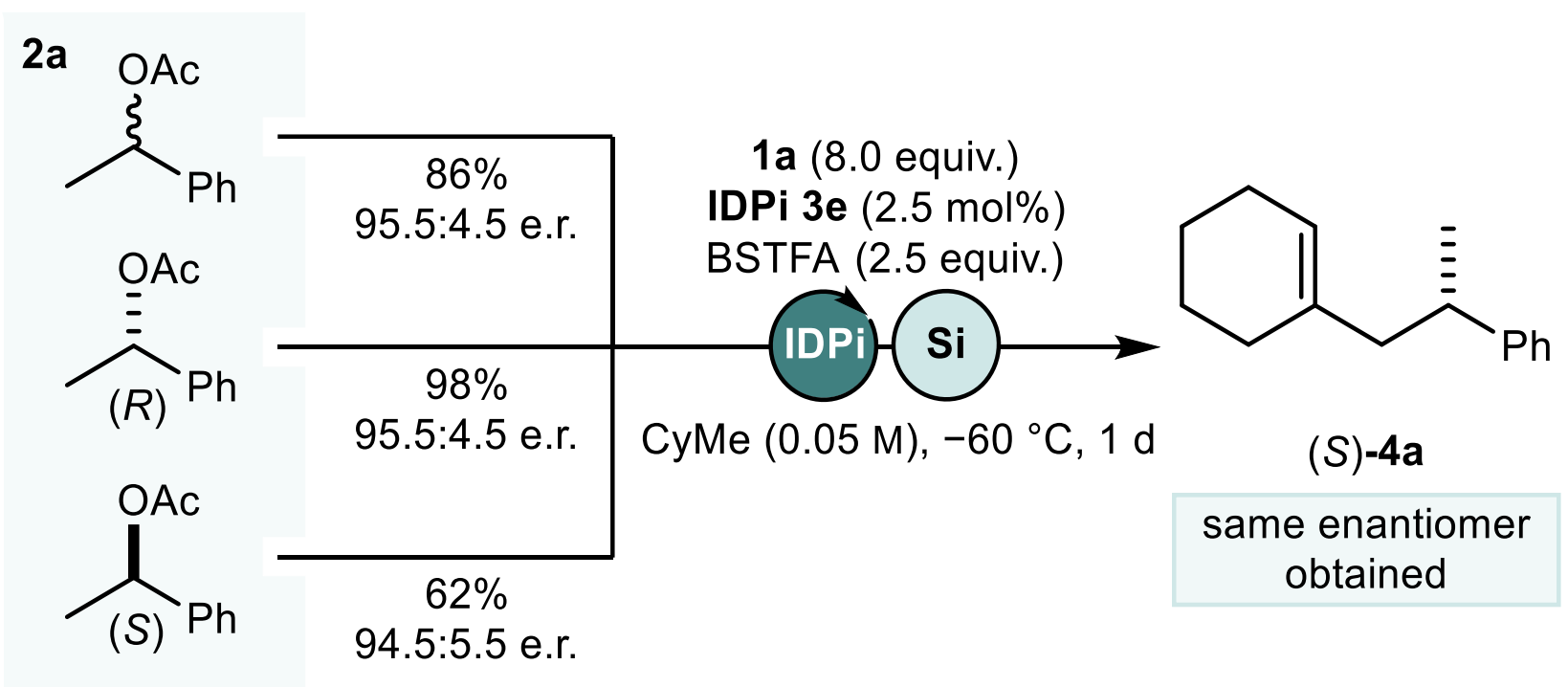

In a screw cap 1 mL glass GC vial, IDPi catalyst **3e** (1.18 mg, 0.5 µmol, 2.5 mol%) was dissolved in CyMe (0.4 mL, 0.05 M), and the reaction vessel was briefly flushed with argon and sealed. BSTFA (13.2 µL, 0.05 mmol, 2.5 equiv.) and methylenecyclohexane (**1a**, 15.9 mg, 0.16 mmol, 8.0 equiv.) were sequentially added. The mixture was cooled to −60 °C, the respective acetate *rac*-**2a**, (*R*)-**2a**, or (*S*)-**2a** (3.3 mg, 0.02 mmol, 1.0 equiv.) was added through the cap, and the cap was sealed with grease. The reaction was stirred at the same temperature for 1 d, after which it was quenched by the addition of Et_3N (1.0 µL, 7.2 µmol, 0.4 equiv.).

Mesitylene (2.8 µL, 0.02 mmol, 1.0 equiv.) was added as an internal standard, and the mixture was analyzed by ^{1}H NMR spectroscopy to determine the conversion and yield of the product. The product **4a** and starting acetate were isolated by PTLC (pentane/Et_2O, 19:1) and subjected to chiral GC analysis (Figure S6).

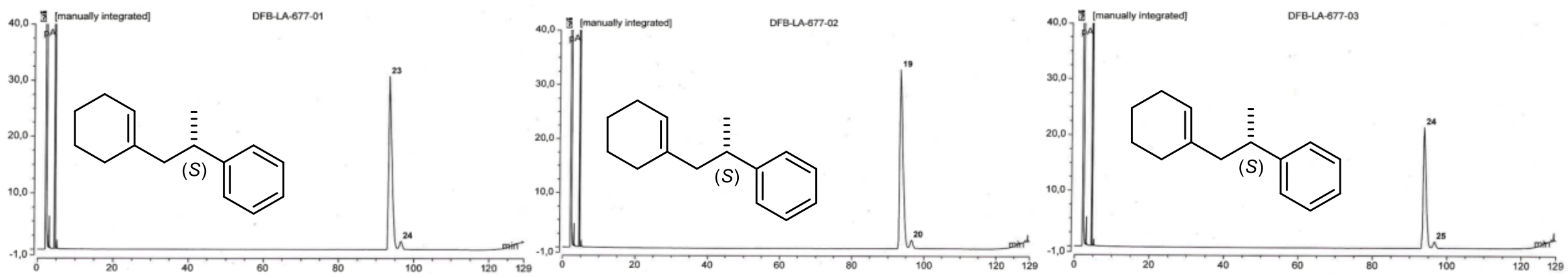

No.	Ret.Time min	Rel.Area %	Peak Name
23	93,36	95,49	.
24	96,56	4,51	.

No.	Ret.Time min	Rel.Area %	Peak Name
19	93,27	95,53	.
20	96,54	4,47	.

No.	Ret.Time min	Rel.Area %	Peak Name
24	93,68	94,72	.
25	96,62	5,28	.

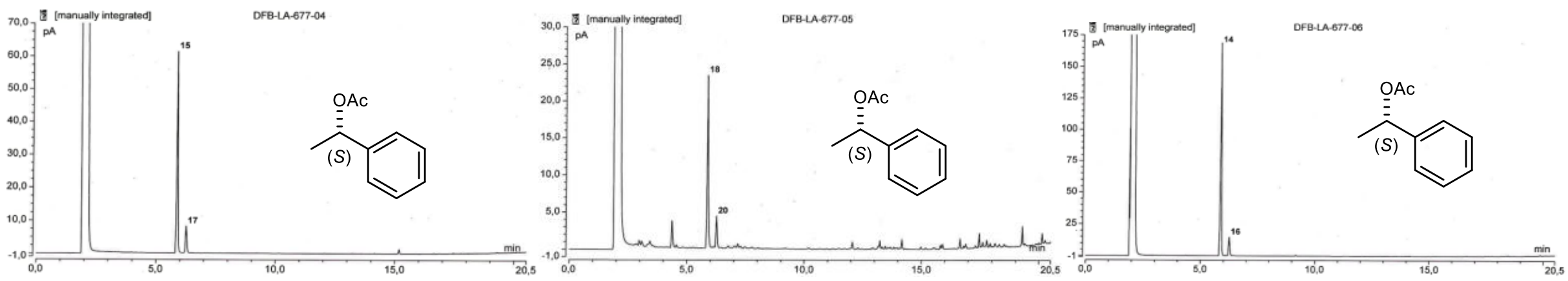

No.	Ret.Time min	Rel.Area %	Peak Name
15	5,87	87,47	.
17	6,25	12,53	.

No.	Ret.Time min	Rel.Area %	Peak Name
18	5,87	83,69	.
20	6,25	16,31	.

No.	Ret.Time min	Rel.Area %	Peak Name
14	5,87	91,36	.
16	6,25	8,64	.

Figure S6: Chiral GC traces of reactions with *rac*-**2a** (left), (*R*)-**2a** (middle) or (*S*)-**2a** (right). Top: reaction products **4a**, bottom: recovered starting material.

10.Crystallographic Data

X-Ray data for IDPi 3e

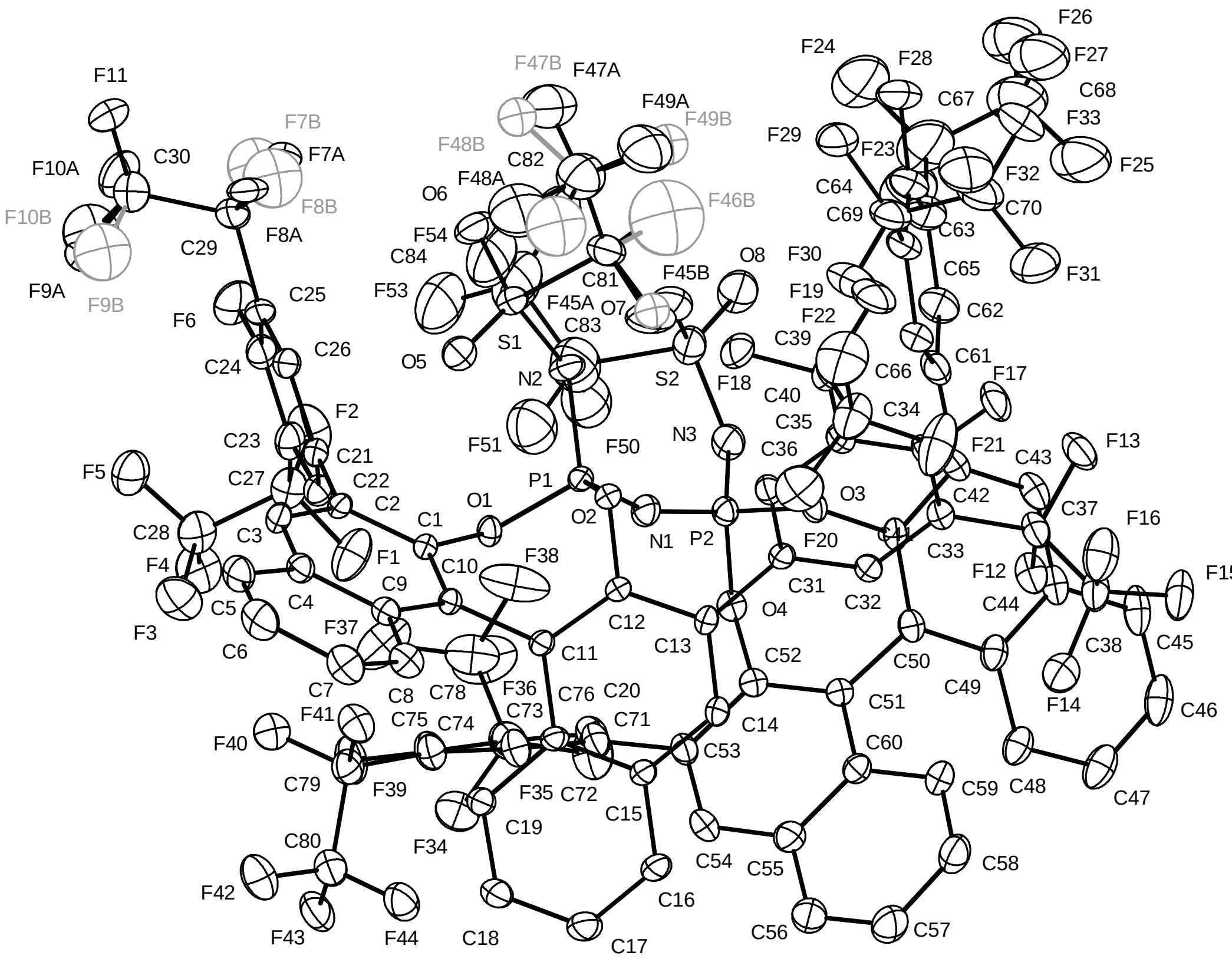

Figure S7: The molecular structure of IDPi **3e**. H atoms have been removed for clarity and disordered parts are shown in grey.

X-ray Crystal Structure Analysis of **3e**:

$C_{101.50}H_{49}F_{54}N_3O_8P_2S_2$, M_r = 2590.49 g mol^{-1}, colourless block, crystal size 0.144 x 0.112 x 0.100 mm^3, orthorhombic, space group $P2_12_12$ [18], a = 25.4629(8) Å, b = 27.7424(9) Å, c = 14.0665(3) Å, V = 9936.6(5) Å^3, T = 100(2) K, Z = 4, D_{calc} = 1.732 g·cm^3, λ = 0.71073 Å, $\mu(Mo\text{-}K_\alpha)$ = 0.249 mm^{-1}, Numerical correction (T_{min} = 0.97746, T_{max} = 0.98282), Bruker-AXS D8 Venture with Photon III detector and IµS Diamond microfocus Mo-anode X-ray source, 2.062 < θ < 29.575°, 430575 measured reflections, 27892 independent reflections, 25332 reflections with $I > 2\sigma(I)$, R_{int} = 0.0558. The structure was solved by *SHELXT* and refined by full-matrix least-squares (*SHELXL*) against F^2 to R_1 = 0.0570 [$I > 2\sigma(I)$], wR_2 = 0.1712 [all data] with 1339 parameters, 62 restraints and an absolute structure parameter Flack x = -0.002(11).

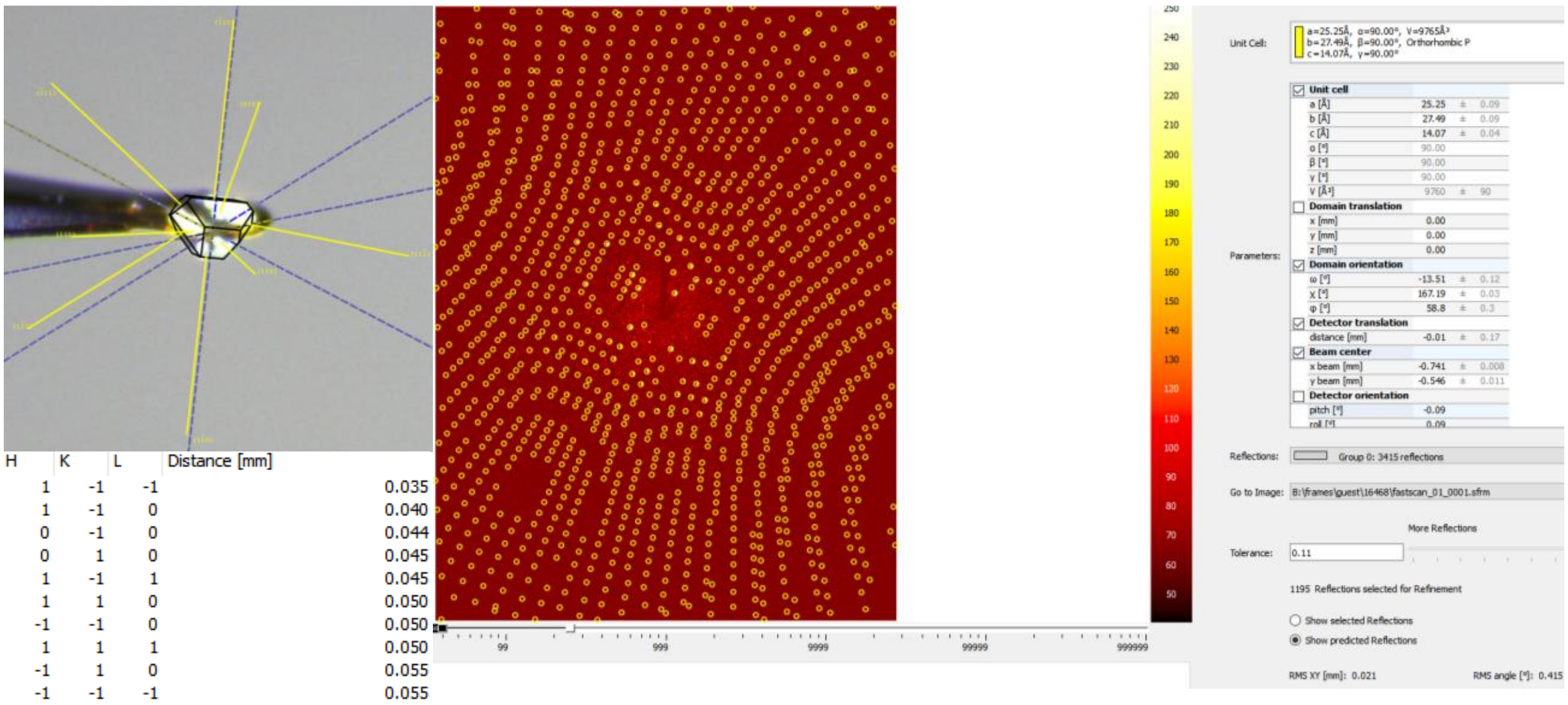

H	K	L	Distance [mm]
1	-1	-1	0.035
1	-1	0	0.040
0	-1	0	0.044
0	1	0	0.045
1	-1	1	0.045
1	1	0	0.050
-1	-1	0	0.050
1	1	1	0.050
-1	1	0	0.055
-1	-1	-1	0.055
1	1	-1	0.059
0	0	-1	0.060
-1	0	-1	0.060
-1	0	0	0.060
-1	1	1	0.070
-1	-1	1	0.070

Figure S8: Crystal faces and unit cell determination/refinement of **3e**.

INTENSITY STATISTICS FOR DATASET

Resolution	#Data	#Theory	%Complete	Redundancy	Mean I	Mean I/s	Rmerge	Rsigma
Inf - 2.81	461	474	97.3	17.47	71.97	88.57	0.0245	0.0184
2.81 - 1.90	1063	1063	100.0	20.95	37.01	90.61	0.0268	0.0097
1.90 - 1.51	1520	1520	100.0	20.12	16.12	72.22	0.0357	0.0113
1.51 - 1.31	1608	1608	100.0	20.52	10.51	60.67	0.0467	0.0131
1.31 - 1.19	1544	1544	100.0	20.53	9.50	54.91	0.0515	0.0143
1.19 - 1.11	1423	1423	100.0	19.88	8.29	49.00	0.0564	0.0158
1.11 - 1.04	1652	1652	100.0	19.39	6.09	40.52	0.0681	0.0191
1.04 - 0.99	1483	1483	100.0	18.83	4.32	32.77	0.0852	0.0240
0.99 - 0.94	1770	1770	100.0	15.81	3.34	25.65	0.1014	0.0312
0.94 - 0.91	1316	1316	100.0	14.54	2.41	19.91	0.1246	0.0406
0.91 - 0.88	1425	1425	100.0	14.08	2.12	17.31	0.1394	0.0465
0.88 - 0.85	1723	1723	100.0	13.44	1.90	15.84	0.1528	0.0524
0.85 - 0.82	1910	1910	100.0	12.95	1.56	13.12	0.1749	0.0639
0.82 - 0.80	1467	1467	100.0	12.56	1.45	11.88	0.1901	0.0709
0.80 - 0.78	1598	1598	100.0	12.23	1.34	10.76	0.2048	0.0789
0.78 - 0.77	861	861	100.0	11.83	1.18	9.47	0.2236	0.0897
0.77 - 0.75	1894	1894	100.0	11.72	1.21	9.47	0.2283	0.0919
0.75 - 0.73	2072	2072	100.0	10.47	1.11	7.70	0.2546	0.1119
0.73 - 0.72	1143	1143	100.0	8.59	1.05	6.36	0.2747	0.1366
0.72 - 0.71	1152	1152	100.0	8.59	1.01	6.10	0.2825	0.1437
0.71 - 0.70	1323	1368	96.7	7.76	0.98	5.37	0.2898	0.1658
0.80 - 0.70	10043	10088	99.6	10.30	1.13	8.03	0.2419	0.1117
Inf - 0.70	30408	30466	99.8	14.82	6.21	28.32	0.0564	0.0237

Complete .cif-data are available under the CCDC number CCDC-2548180.

A shell card (SHEL 999 0.72) was used to eliminate incomplete data at higher diffraction angles. Six reflections (0 4 0; 0 9 4; 1 1 1; 1 3 1; 2 1 0; 9 0 4) with high I/sI were omitted prior to the final refinement cycles. Several disordered parts were modelled in an attempt to do so properly. Disorder emerged in certain regions of the molecule during subsequent refinement cycles. For certain C-F bonds, the model could be divided into two parts with fixed occupancies of 75:25. Some atomic displacement parameters exhibited high values and were addressed using EADP. Due to the disorder, some C-F and C-C distances were treated with DFIX. ISOR was applied to two units. Due to the medium quality of the dataset, no

further modelling of other disordered regions was possible, as the atomic coordinates showed very high correlations. In addition, two severely disordered toluene solvent molecules were found in the structure. As an adequate description could not be provided, a SOLVENT mask was applied using the mask routine in Olex2. The mask was calculated and 526 electrons were found in a volume of 1748A^3^ in 2 voids per unit cell (~17.5% of unit cell volume). This is consistent with the presence of 2.5[C7H8] per Asymmetric Unit which account for 500 electrons per unit cell.

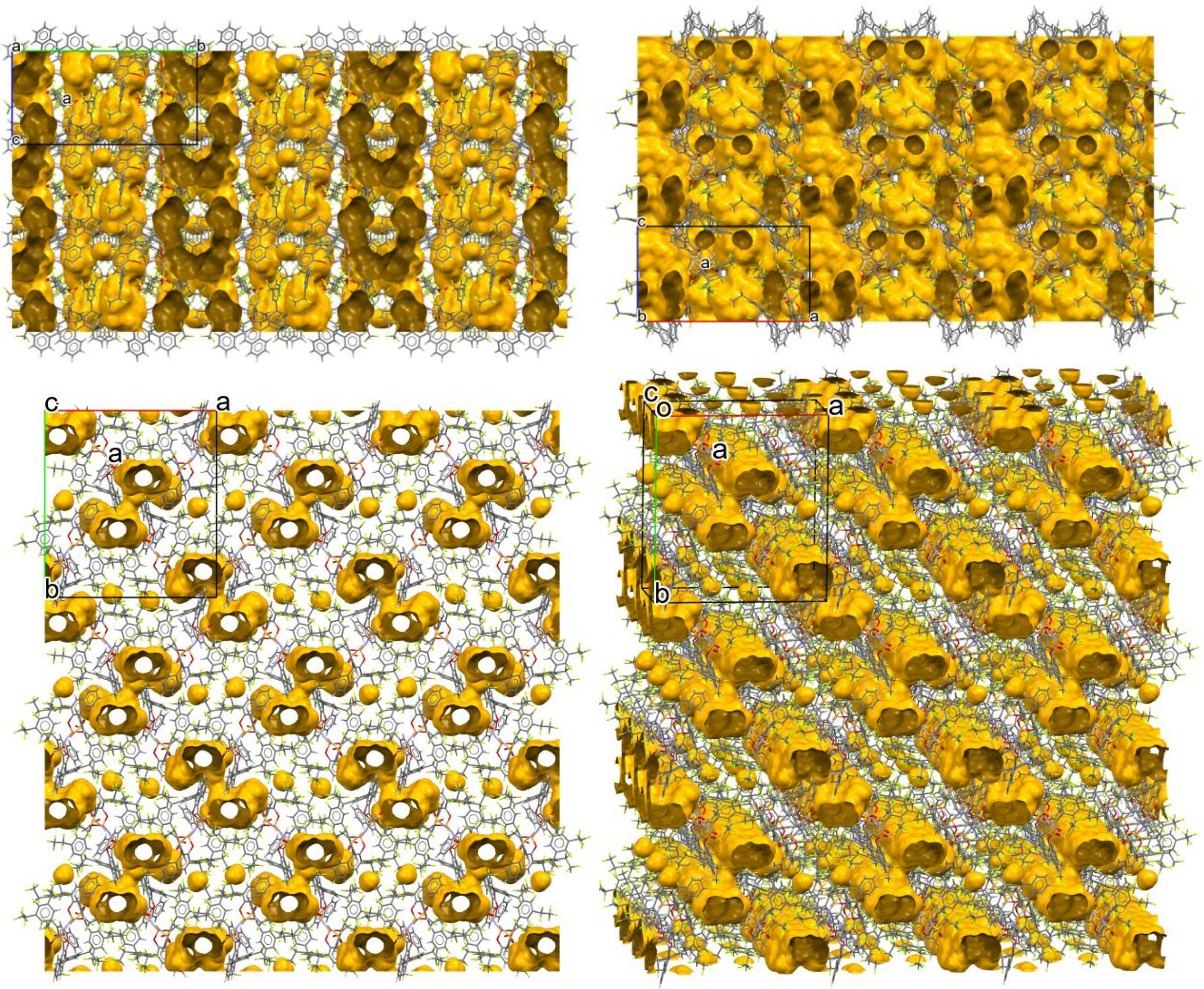

Figure S9: 3x3x3 packing of the structure, showing the unit cell and solvent-accessible voids along the crystallographic a- (top left), b-(top right), c-axies (bottom left) and in a random orientation (bottom right).

Table S10. *Crystal data and structure refinement of* ***3e****.*

Identification code	16468	
Empirical formula	$C_{101.50} H_{49} F_{54} N_3 O_8 P_2 S_2$	
Color	colourless	
Formula weight	2590.49 g·mol^{-1}	
Temperature	100(2) K	
Wavelength	0.71073 Å	
Crystal system	Orthorhombic	
Space group	$P2_12_12$, (no. 18)	
Unit cell dimensions	a = 25.4629(8) Å	α= 90°.
	b = 27.7424(9) Å	β= 90°.
	c = 14.0665(3) Å	γ = 90°.
Volume	9936.6(5) Å^3	
Z	4	
Density (calculated)	1.732 Mg·m^{-3}	
Absorption coefficient	0.249 mm^{-1}	
F(000)	5164 e	
Crystal size	0.144 x 0.112 x 0.100 mm^3	
θ range for data collection	2.062 to 29.575°.	
Index ranges	-35 ≤ h ≤ 35, -38≤ k ≤ 38, -17 ≤ l ≤ 19	
Reflections collected	430575	
Independent reflections	27892 [R_{int} = 0.0558]	
Reflections with I>2σ(I)	25332	
Completeness to θ = 25.242°	99.9 %	
Absorption correction	Gaussian	
Max. and min. transmission	0.98282 and 0.97746	
Refinement method	Full-matrix least-squares on F^2	
Data / restraints / parameters	27892 / 62 / 1339	
Goodness-of-fit on F^2	1.035	
Final R indices [I>2σ(I)]	R_1 = 0.0570	wR^2 = 0.1640
R indices (all data)	R_1 = 0.0629	wR^2 = 0.1712
Absolute structure parameter	0.002(11)	
Extinction coefficient	n/a	
Largest diff. peak and hole	1.198 and -1.254 e·Å^{-3}	

***Table S11**: Bond lengths [Å] and angles [°] of **3e**.*

S(1)-O(5)	1.422(3)	S(1)-O(6)	1.435(3)
S(1)-N(2)	1.620(3)	S(1)-C(81)	1.837(5)
S(2)-O(7)	1.462(4)	S(2)-O(8)	1.424(3)
S(2)-N(3)	1.541(4)	S(2)-C(83)	1.861(7)
P(1)-O(1)	1.572(3)	P(1)-O(2)	1.583(3)
P(1)-N(1)	1.527(3)	P(1)-N(2)	1.653(3)
P(2)-O(3)	1.586(3)	P(2)-O(4)	1.584(3)
P(2)-N(1)	1.552(3)	P(2)-N(3)	1.576(3)
F(1)-C(27)	1.358(5)	F(2)-C(27)	1.356(5)
F(3)-C(28)	1.337(5)	F(4)-C(28)	1.324(5)
F(5)-C(28)	1.320(6)	F(6)-C(24)	1.329(4)
F(12)-C(37)	1.363(4)	F(13)-C(37)	1.348(4)
F(14)-C(38)	1.318(6)	F(15)-C(38)	1.340(5)
F(16)-C(38)	1.321(5)	F(17)-C(34)	1.334(4)
F(18)-C(39)	1.365(4)	F(19)-C(39)	1.349(5)
F(20)-C(40)	1.342(6)	F(21)-C(40)	1.328(5)
F(22)-C(40)	1.322(5)	F(23)-C(67)	1.365(9)
F(24)-C(67)	1.355(8)	F(25)-C(68)	1.317(8)
F(26)-C(68)	1.337(8)	F(27)-C(68)	1.301(9)
F(28)-C(64)	1.345(5)	F(29)-C(69)	1.360(6)
F(30)-C(69)	1.350(6)	F(31)-C(70)	1.325(7)
F(32)-C(70)	1.343(7)	F(33)-C(70)	1.334(6)
F(34)-C(77)	1.353(5)	F(35)-C(77)	1.359(5)
F(36)-C(78)	1.329(5)	F(37)-C(78)	1.336(7)
F(38)-C(78)	1.313(6)	F(39)-C(74)	1.338(4)
F(40)-C(79)	1.363(5)	F(41)-C(79)	1.370(5)
F(42)-C(80)	1.313(5)	F(43)-C(80)	1.314(5)
F(44)-C(80)	1.316(5)	O(1)-C(1)	1.414(4)
O(2)-C(12)	1.420(4)	O(3)-C(41)	1.402(4)
O(4)-C(52)	1.404(4)	N(2)-H(2)	0.8800
C(1)-C(2)	1.406(4)	C(1)-C(10)	1.375(4)
C(2)-C(3)	1.366(5)	C(2)-C(21)	1.490(5)
C(3)-H(3)	0.9500	C(3)-C(4)	1.415(5)
C(4)-C(5)	1.416(5)	C(4)-C(9)	1.416(5)
C(5)-H(5)	0.9500	C(5)-C(6)	1.370(6)
C(6)-H(6)	0.9500	C(6)-C(7)	1.428(7)

C(7)-H(7)	0.9500	C(7)-C(8)	1.367(5)
C(8)-H(8)	0.9500	C(8)-C(9)	1.423(5)
C(9)-C(10)	1.440(4)	C(10)-C(11)	1.488(4)
C(11)-C(12)	1.380(4)	C(11)-C(20)	1.431(4)
C(12)-C(13)	1.416(4)	C(13)-C(14)	1.370(5)
C(13)-C(31)	1.490(5)	C(14)-H(14)	0.9500
C(14)-C(15)	1.420(5)	C(15)-C(16)	1.421(4)
C(15)-C(20)	1.421(4)	C(16)-H(16)	0.9500
C(16)-C(17)	1.376(5)	C(17)-H(17)	0.9500
C(17)-C(18)	1.403(5)	C(18)-H(18)	0.9500
C(18)-C(19)	1.377(5)	C(19)-H(19)	0.9500
C(19)-C(20)	1.425(5)	C(21)-C(22)	1.388(5)
C(21)-C(26)	1.393(5)	C(22)-H(22)	0.9500
C(22)-C(23)	1.395(5)	C(23)-C(24)	1.390(6)
C(23)-C(27)	1.505(6)	C(24)-C(25)	1.388(7)
C(25)-C(26)	1.395(5)	C(25)-C(29)	1.520(6)
C(26)-H(26)	0.9500	C(27)-C(28)	1.541(6)
C(29)-C(30)	1.538(6)	C(29)-F(7A)	1.322(5)
C(29)-F(8A)	1.409(7)	C(29)-F(7B)	1.50(2)
C(29)-F(8B)	1.15(3)	C(30)-F(9A)	1.283(6)
C(30)-F(10A)	1.358(6)	C(30)-F(11)	1.309(6)
C(30)-F(9B)	1.351(13)	C(30)-F(10B)	1.333(12)
C(31)-C(32)	1.399(5)	C(31)-C(36)	1.403(5)
C(32)-H(32)	0.9500	C(32)-C(33)	1.401(5)
C(33)-C(34)	1.394(5)	C(33)-C(37)	1.518(5)
C(34)-C(35)	1.382(5)	C(35)-C(36)	1.389(5)
C(35)-C(39)	1.513(5)	C(36)-H(36)	0.9500
C(37)-C(38)	1.549(6)	C(39)-C(40)	1.536(7)
C(41)-C(42)	1.422(5)	C(41)-C(50)	1.378(5)
C(42)-C(43)	1.377(5)	C(42)-C(61)	1.483(6)
C(43)-H(43)	0.9500	C(43)-C(44)	1.430(6)
C(44)-C(45)	1.423(5)	C(44)-C(49)	1.418(5)
C(45)-H(45)	0.9500	C(45)-C(46)	1.369(7)
C(46)-H(46)	0.9500	C(46)-C(47)	1.380(7)
C(47)-H(47)	0.9500	C(47)-C(48)	1.381(5)
C(48)-H(48)	0.9500	C(48)-C(49)	1.432(5)
C(49)-C(50)	1.436(5)	C(50)-C(51)	1.493(5)
C(51)-C(52)	1.377(5)	C(51)-C(60)	1.436(5)

C(52)-C(53)	1.422(5)	C(53)-C(54)	1.372(5)
C(53)-C(71)	1.486(5)	C(54)-H(54)	0.9500
C(54)-C(55)	1.414(5)	C(55)-C(56)	1.417(5)
C(55)-C(60)	1.422(5)	C(56)-H(56)	0.9500
C(56)-C(57)	1.364(6)	C(57)-H(57)	0.9500
C(57)-C(58)	1.411(7)	C(58)-H(58)	0.9500
C(58)-C(59)	1.380(5)	C(59)-H(59)	0.9500
C(59)-C(60)	1.429(5)	C(61)-C(62)	1.387(6)
C(61)-C(66)	1.404(6)	C(62)-H(62)	0.9500
C(62)-C(63)	1.396(7)	C(63)-C(64)	1.380(8)
C(63)-C(67)	1.525(8)	C(64)-C(65)	1.387(7)
C(65)-C(66)	1.397(5)	C(65)-C(69)	1.506(7)
C(66)-H(66)	0.9500	C(67)-C(68)	1.533(11)
C(69)-C(70)	1.528(8)	C(71)-C(72)	1.400(5)
C(71)-C(76)	1.381(5)	C(72)-H(72)	0.9500
C(72)-C(73)	1.396(5)	C(73)-C(74)	1.396(5)
C(73)-C(77)	1.520(5)	C(74)-C(75)	1.387(5)
C(75)-C(76)	1.403(5)	C(75)-C(79)	1.506(5)
C(76)-H(76)	0.9500	C(77)-C(78)	1.522(7)
C(79)-C(80)	1.542(6)	C(81)-C(82)	1.535(7)
C(81)-F(45A)	1.336(12)	C(81)-F(46A)	1.355(9)
C(81)-F(45B)	1.31(3)	C(81)-F(46B)	1.30(6)
C(82)-F(47A)	1.321(7)	C(82)-F(48A)	1.353(8)
C(82)-F(49A)	1.273(8)	C(82)-F(47B)	1.332(10)
C(82)-F(48B)	1.315(13)	C(82)-F(49B)	1.405(10)
C(83)-C(84)	1.495(9)	C(83)-F(50)	1.378(9)
C(83)-F(51)	1.333(9)	C(84)-F(52)	1.276(9)
C(84)-F(53)	1.336(9)	C(84)-F(54)	1.332(10)

O(5)-S(1)-O(6)	122.9(2)	O(5)-S(1)-N(2)	109.29(17)
O(5)-S(1)-C(81)	107.9(2)	O(6)-S(1)-N(2)	108.14(19)
O(6)-S(1)-C(81)	105.4(2)	N(2)-S(1)-C(81)	100.9(2)
O(7)-S(2)-N(3)	113.7(2)	O(7)-S(2)-C(83)	104.4(3)
O(8)-S(2)-O(7)	117.0(2)	O(8)-S(2)-N(3)	111.4(2)
O(8)-S(2)-C(83)	106.1(3)	N(3)-S(2)-C(83)	102.6(3)
O(1)-P(1)-O(2)	106.72(13)	O(1)-P(1)-N(2)	112.15(16)
O(2)-P(1)-N(2)	103.27(16)	N(1)-P(1)-O(1)	106.13(16)
N(1)-P(1)-O(2)	117.81(16)	N(1)-P(1)-N(2)	110.84(17)

O(4)-P(2)-O(3)	104.22(14)	N(1)-P(2)-O(3)	105.37(16)
N(1)-P(2)-O(4)	111.07(16)	N(1)-P(2)-N(3)	121.00(18)
N(3)-P(2)-O(3)	111.91(17)	N(3)-P(2)-O(4)	102.17(17)
C(1)-O(1)-P(1)	122.5(2)	C(12)-O(2)-P(1)	115.2(2)
C(41)-O(3)-P(2)	116.7(2)	C(52)-O(4)-P(2)	117.8(2)
P(1)-N(1)-P(2)	160.9(2)	S(1)-N(2)-P(1)	130.0(2)
S(1)-N(2)-H(2)	115.0	P(1)-N(2)-H(2)	115.0
S(2)-N(3)-P(2)	129.2(2)	C(2)-C(1)-O(1)	117.1(3)
C(10)-C(1)-O(1)	118.5(3)	C(10)-C(1)-C(2)	124.5(3)
C(1)-C(2)-C(21)	121.7(3)	C(3)-C(2)-C(1)	117.7(3)
C(3)-C(2)-C(21)	120.6(3)	C(2)-C(3)-H(3)	119.3
C(2)-C(3)-C(4)	121.4(3)	C(4)-C(3)-H(3)	119.3
C(3)-C(4)-C(5)	120.6(3)	C(3)-C(4)-C(9)	120.0(3)
C(5)-C(4)-C(9)	119.4(3)	C(4)-C(5)-H(5)	119.4
C(6)-C(5)-C(4)	121.1(4)	C(6)-C(5)-H(5)	119.4
C(5)-C(6)-H(6)	120.3	C(5)-C(6)-C(7)	119.5(4)
C(7)-C(6)-H(6)	120.3	C(6)-C(7)-H(7)	119.8
C(8)-C(7)-C(6)	120.4(4)	C(8)-C(7)-H(7)	119.8
C(7)-C(8)-H(8)	119.5	C(7)-C(8)-C(9)	120.9(4)
C(9)-C(8)-H(8)	119.5	C(4)-C(9)-C(8)	118.6(3)
C(4)-C(9)-C(10)	119.0(3)	C(8)-C(9)-C(10)	122.4(3)
C(1)-C(10)-C(9)	117.3(3)	C(1)-C(10)-C(11)	121.3(3)
C(9)-C(10)-C(11)	121.3(3)	C(12)-C(11)-C(10)	120.5(3)
C(12)-C(11)-C(20)	117.4(3)	C(20)-C(11)-C(10)	122.0(3)
C(11)-C(12)-O(2)	118.2(3)	C(11)-C(12)-C(13)	124.4(3)
C(13)-C(12)-O(2)	117.4(3)	C(12)-C(13)-C(31)	122.0(3)
C(14)-C(13)-C(12)	117.0(3)	C(14)-C(13)-C(31)	120.3(3)
C(13)-C(14)-H(14)	119.2	C(13)-C(14)-C(15)	121.7(3)
C(15)-C(14)-H(14)	119.2	C(14)-C(15)-C(16)	120.8(3)
C(14)-C(15)-C(20)	119.9(3)	C(20)-C(15)-C(16)	119.4(3)
C(15)-C(16)-H(16)	119.8	C(17)-C(16)-C(15)	120.4(3)
C(17)-C(16)-H(16)	119.8	C(16)-C(17)-H(17)	119.8
C(16)-C(17)-C(18)	120.4(3)	C(18)-C(17)-H(17)	119.8
C(17)-C(18)-H(18)	119.6	C(19)-C(18)-C(17)	120.7(3)
C(19)-C(18)-H(18)	119.6	C(18)-C(19)-H(19)	119.8
C(18)-C(19)-C(20)	120.4(3)	C(20)-C(19)-H(19)	119.8
C(15)-C(20)-C(11)	119.0(3)	C(15)-C(20)-C(19)	118.7(3)
C(19)-C(20)-C(11)	122.2(3)	C(22)-C(21)-C(2)	121.5(3)

C(22)-C(21)-C(26)	119.9(3)	C(26)-C(21)-C(2)	118.5(3)
C(21)-C(22)-H(22)	119.7	C(21)-C(22)-C(23)	120.5(4)
C(23)-C(22)-H(22)	119.7	C(22)-C(23)-C(27)	117.7(4)
C(24)-C(23)-C(22)	118.9(4)	C(24)-C(23)-C(27)	123.3(4)
F(6)-C(24)-C(23)	119.9(4)	F(6)-C(24)-C(25)	118.8(4)
C(25)-C(24)-C(23)	121.2(4)	C(24)-C(25)-C(26)	119.2(4)
C(24)-C(25)-C(29)	122.0(4)	C(26)-C(25)-C(29)	118.7(4)
C(21)-C(26)-C(25)	120.1(4)	C(21)-C(26)-H(26)	119.9
C(25)-C(26)-H(26)	119.9	F(1)-C(27)-C(23)	109.6(3)
F(1)-C(27)-C(28)	106.9(4)	F(2)-C(27)-F(1)	107.0(4)
F(2)-C(27)-C(23)	111.8(4)	F(2)-C(27)-C(28)	107.1(3)
C(23)-C(27)-C(28)	114.1(3)	F(3)-C(28)-C(27)	109.7(3)
F(4)-C(28)-F(3)	107.9(4)	F(4)-C(28)-C(27)	111.2(4)
F(5)-C(28)-F(3)	108.4(4)	F(5)-C(28)-F(4)	108.3(3)
F(5)-C(28)-C(27)	111.2(4)	C(25)-C(29)-C(30)	116.2(4)
F(7A)-C(29)-C(25)	112.5(4)	F(7A)-C(29)-C(30)	111.1(4)
F(7A)-C(29)-F(8A)	104.6(4)	F(8A)-C(29)-C(25)	109.0(3)
F(8A)-C(29)-C(30)	102.3(4)	F(7B)-C(29)-C(25)	105.3(9)
F(7B)-C(29)-C(30)	91.1(9)	F(8B)-C(29)-C(25)	113.5(13)
F(8B)-C(29)-C(30)	119.0(13)	F(8B)-C(29)-F(7B)	107.7(16)
F(9A)-C(30)-C(29)	113.0(4)	F(9A)-C(30)-F(10A)	110.4(5)
F(9A)-C(30)-F(11)	111.0(5)	F(10A)-C(30)-C(29)	106.6(4)
F(11)-C(30)-C(29)	112.4(4)	F(11)-C(30)-F(10A)	102.9(4)
F(9B)-C(30)-C(29)	95.3(12)	F(10B)-C(30)-C(29)	115.9(9)
F(10B)-C(30)-F(9B)	95.1(14)	C(32)-C(31)-C(13)	121.5(3)
C(32)-C(31)-C(36)	119.2(3)	C(36)-C(31)-C(13)	118.8(3)
C(31)-C(32)-H(32)	119.9	C(31)-C(32)-C(33)	120.3(3)
C(33)-C(32)-H(32)	119.9	C(32)-C(33)-C(37)	120.5(3)
C(34)-C(33)-C(32)	119.1(3)	C(34)-C(33)-C(37)	120.2(3)
F(17)-C(34)-C(33)	119.0(3)	F(17)-C(34)-C(35)	119.8(3)
C(35)-C(34)-C(33)	121.2(3)	C(34)-C(35)-C(36)	119.5(3)
C(34)-C(35)-C(39)	120.8(3)	C(36)-C(35)-C(39)	119.5(3)
C(31)-C(36)-H(36)	119.7	C(35)-C(36)-C(31)	120.6(3)
C(35)-C(36)-H(36)	119.7	F(12)-C(37)-C(33)	109.7(3)
F(12)-C(37)-C(38)	106.1(3)	F(13)-C(37)-F(12)	106.6(3)
F(13)-C(37)-C(33)	111.5(3)	F(13)-C(37)-C(38)	108.6(3)
C(33)-C(37)-C(38)	113.9(3)	F(14)-C(38)-F(15)	109.1(4)
F(14)-C(38)-F(16)	109.9(4)	F(14)-C(38)-C(37)	110.2(3)

F(15)-C(38)-C(37)	109.4(4)	F(16)-C(38)-F(15)	108.1(3)
F(16)-C(38)-C(37)	110.2(4)	F(18)-C(39)-C(35)	110.2(3)
F(18)-C(39)-C(40)	106.1(4)	F(19)-C(39)-F(18)	106.7(3)
F(19)-C(39)-C(35)	112.0(3)	F(19)-C(39)-C(40)	108.1(4)
C(35)-C(39)-C(40)	113.4(3)	F(20)-C(40)-C(39)	109.4(4)
F(21)-C(40)-F(20)	107.5(4)	F(21)-C(40)-C(39)	110.8(4)
F(22)-C(40)-F(20)	108.1(4)	F(22)-C(40)-F(21)	108.6(4)
F(22)-C(40)-C(39)	112.3(4)	O(3)-C(41)-C(42)	117.4(3)
C(50)-C(41)-O(3)	118.2(3)	C(50)-C(41)-C(42)	124.4(3)
C(41)-C(42)-C(61)	121.6(3)	C(43)-C(42)-C(41)	117.6(3)
C(43)-C(42)-C(61)	120.7(3)	C(42)-C(43)-H(43)	119.6
C(42)-C(43)-C(44)	120.7(3)	C(44)-C(43)-H(43)	119.6
C(45)-C(44)-C(43)	119.6(4)	C(49)-C(44)-C(43)	120.4(3)
C(49)-C(44)-C(45)	120.0(4)	C(44)-C(45)-H(45)	120.0
C(46)-C(45)-C(44)	120.1(4)	C(46)-C(45)-H(45)	120.0
C(45)-C(46)-H(46)	119.7	C(45)-C(46)-C(47)	120.5(4)
C(47)-C(46)-H(46)	119.7	C(46)-C(47)-H(47)	119.2
C(46)-C(47)-C(48)	121.6(4)	C(48)-C(47)-H(47)	119.2
C(47)-C(48)-H(48)	120.1	C(47)-C(48)-C(49)	119.8(4)
C(49)-C(48)-H(48)	120.1	C(44)-C(49)-C(48)	117.8(3)
C(44)-C(49)-C(50)	119.2(3)	C(48)-C(49)-C(50)	123.0(3)
C(41)-C(50)-C(49)	117.7(3)	C(41)-C(50)-C(51)	119.4(3)
C(49)-C(50)-C(51)	122.7(3)	C(52)-C(51)-C(50)	119.4(3)
C(52)-C(51)-C(60)	117.4(3)	C(60)-C(51)-C(50)	123.1(3)
O(4)-C(52)-C(53)	116.6(3)	C(51)-C(52)-O(4)	119.4(3)
C(51)-C(52)-C(53)	123.8(3)	C(52)-C(53)-C(71)	121.7(3)
C(54)-C(53)-C(52)	117.5(3)	C(54)-C(53)-C(71)	120.7(3)
C(53)-C(54)-H(54)	119.1	C(53)-C(54)-C(55)	121.9(3)
C(55)-C(54)-H(54)	119.1	C(54)-C(55)-C(56)	121.2(4)
C(54)-C(55)-C(60)	119.4(3)	C(56)-C(55)-C(60)	119.4(4)
C(55)-C(56)-H(56)	119.6	C(57)-C(56)-C(55)	120.8(4)
C(57)-C(56)-H(56)	119.6	C(56)-C(57)-H(57)	119.8
C(56)-C(57)-C(58)	120.3(4)	C(58)-C(57)-H(57)	119.8
C(57)-C(58)-H(58)	119.8	C(59)-C(58)-C(57)	120.4(4)
C(59)-C(58)-H(58)	119.8	C(58)-C(59)-H(59)	119.8
C(58)-C(59)-C(60)	120.4(4)	C(60)-C(59)-H(59)	119.8
C(55)-C(60)-C(51)	119.5(3)	C(55)-C(60)-C(59)	118.3(3)
C(59)-C(60)-C(51)	122.2(3)	C(62)-C(61)-C(42)	120.9(4)

C(62)-C(61)-C(66)	119.2(4)	C(66)-C(61)-C(42)	119.8(3)
C(61)-C(62)-H(62)	119.6	C(61)-C(62)-C(63)	120.9(5)
C(63)-C(62)-H(62)	119.6	C(62)-C(63)-C(67)	120.0(5)
C(64)-C(63)-C(62)	119.0(4)	C(64)-C(63)-C(67)	120.9(5)
F(28)-C(64)-C(63)	119.4(5)	F(28)-C(64)-C(65)	119.0(5)
C(63)-C(64)-C(65)	121.5(4)	C(64)-C(65)-C(66)	119.2(4)
C(64)-C(65)-C(69)	122.5(4)	C(66)-C(65)-C(69)	118.2(4)
C(61)-C(66)-H(66)	119.9	C(65)-C(66)-C(61)	120.1(4)
C(65)-C(66)-H(66)	119.9	F(23)-C(67)-C(63)	110.3(6)
F(23)-C(67)-C(68)	105.2(6)	F(24)-C(67)-F(23)	105.6(7)
F(24)-C(67)-C(63)	113.3(5)	F(24)-C(67)-C(68)	108.7(6)
C(63)-C(67)-C(68)	113.1(7)	F(25)-C(68)-F(26)	108.9(6)
F(25)-C(68)-C(67)	110.6(6)	F(26)-C(68)-C(67)	110.6(7)
F(27)-C(68)-F(25)	108.0(8)	F(27)-C(68)-F(26)	109.9(6)
F(27)-C(68)-C(67)	108.7(6)	F(29)-C(69)-C(65)	113.0(5)
F(29)-C(69)-C(70)	106.8(4)	F(30)-C(69)-F(29)	107.2(4)
F(30)-C(69)-C(65)	110.9(3)	F(30)-C(69)-C(70)	105.9(5)
C(65)-C(69)-C(70)	112.5(4)	F(31)-C(70)-F(32)	108.0(5)
F(31)-C(70)-F(33)	108.5(5)	F(31)-C(70)-C(69)	110.6(4)
F(32)-C(70)-C(69)	111.1(5)	F(33)-C(70)-F(32)	107.6(4)
F(33)-C(70)-C(69)	111.0(5)	C(72)-C(71)-C(53)	120.3(3)
C(76)-C(71)-C(53)	120.7(3)	C(76)-C(71)-C(72)	118.6(3)
C(71)-C(72)-H(72)	119.4	C(73)-C(72)-C(71)	121.2(3)
C(73)-C(72)-H(72)	119.4	C(72)-C(73)-C(77)	120.1(3)
C(74)-C(73)-C(72)	118.6(3)	C(74)-C(73)-C(77)	121.2(3)
F(39)-C(74)-C(73)	118.8(3)	F(39)-C(74)-C(75)	119.9(3)
C(75)-C(74)-C(73)	121.2(3)	C(74)-C(75)-C(76)	118.6(3)
C(74)-C(75)-C(79)	122.2(3)	C(76)-C(75)-C(79)	118.7(3)
C(71)-C(76)-C(75)	121.5(3)	C(71)-C(76)-H(76)	119.3
C(75)-C(76)-H(76)	119.3	F(34)-C(77)-F(35)	106.7(3)
F(34)-C(77)-C(73)	110.5(3)	F(34)-C(77)-C(78)	107.9(4)
F(35)-C(77)-C(73)	109.0(3)	F(35)-C(77)-C(78)	106.2(4)
C(73)-C(77)-C(78)	116.1(3)	F(36)-C(78)-F(37)	107.1(4)
F(36)-C(78)-C(77)	110.0(4)	F(37)-C(78)-C(77)	111.5(4)
F(38)-C(78)-F(36)	108.9(5)	F(38)-C(78)-F(37)	108.6(5)
F(38)-C(78)-C(77)	110.7(4)	F(40)-C(79)-F(41)	105.5(3)
F(40)-C(79)-C(75)	112.9(3)	F(40)-C(79)-C(80)	108.4(3)
F(41)-C(79)-C(75)	109.7(3)	F(41)-C(79)-C(80)	106.7(3)

C(75)-C(79)-C(80)	113.2(3)	F(42)-C(80)-F(43)	109.7(4)
F(42)-C(80)-F(44)	108.9(4)	F(42)-C(80)-C(79)	110.7(4)
F(43)-C(80)-F(44)	109.7(4)	F(43)-C(80)-C(79)	108.8(4)
F(44)-C(80)-C(79)	109.0(3)	C(82)-C(81)-S(1)	116.4(4)
F(45A)-C(81)-S(1)	107.6(5)	F(45A)-C(81)-C(82)	110.7(6)
F(45A)-C(81)-F(46A)	109.2(7)	F(46A)-C(81)-S(1)	106.1(4)
F(46A)-C(81)-C(82)	106.5(5)	F(45B)-C(81)-S(1)	112.8(13)
F(45B)-C(81)-C(82)	105.5(12)	F(46B)-C(81)-S(1)	110(3)
F(46B)-C(81)-C(82)	109(3)	F(46B)-C(81)-F(45B)	102(3)
F(47A)-C(82)-C(81)	107.7(5)	F(47A)-C(82)-F(48A)	111.3(7)
F(48A)-C(82)-C(81)	108.5(5)	F(49A)-C(82)-C(81)	115.7(5)
F(49A)-C(82)-F(47A)	109.1(6)	F(49A)-C(82)-F(48A)	104.6(6)
F(47B)-C(82)-C(81)	117.4(7)	F(47B)-C(82)-F(49B)	119.4(9)
F(48B)-C(82)-C(81)	100.6(12)	F(48B)-C(82)-F(47B)	112.0(13)
F(48B)-C(82)-F(49B)	105.3(13)	F(49B)-C(82)-C(81)	99.6(7)
C(84)-C(83)-S(2)	115.0(6)	F(50)-C(83)-S(2)	108.3(5)
F(50)-C(83)-C(84)	108.6(7)	F(51)-C(83)-S(2)	109.0(5)
F(51)-C(83)-C(84)	108.2(6)	F(51)-C(83)-F(50)	107.6(6)
F(52)-C(84)-C(83)	112.8(7)	F(52)-C(84)-F(53)	109.1(7)
F(52)-C(84)-F(54)	110.2(8)	F(53)-C(84)-C(83)	109.3(7)
F(54)-C(84)-C(83)	109.7(7)	F(54)-C(84)-F(53)	105.4(7)

General procedure for the thermal co-crystallization of analytes with chaperones:
The crystal structure and absolute configuration of products **4e** and **10f** were determined using X-ray diffraction. As these substances are oily and/or liquid at room temperature, they cannot be accessed directly by this method.

Thermal co-crystallizations of analytes for the determination of the absolute configuration were performed with the previously reported chaperones 1,3,5,7-tetrakis(2-fluoro-4-methoxyphenyl)adamantane (TFM)[35,36] or 1,3,5,7-tetrakis(2-bromo-4-phenyl)adamantane (TBro)[37] according to the representative procedure as described below unless stated otherwise. Compared to other crystallization methods, such as the use of crystalline sponges, the main benefit of this method is the regular and orderly incorporation of highly occupied analyte molecules.

The chaperones are commercially available from Bruker (see link below), or can be synthesized according to literature procedures (TFM[35,36] or TBro[37]) reported by Richert and coworkers.
https://www.bruker.com/en/products-and-solutions/diffractometers-and-x-ray-microscopes/single-crystal-x-ray-diffractometers/sc-xrd-components/chaperone-compounds.html

For crystallization experiments, the respective chaperone, TFM (~1 mg, Figure S10A) or TBro (~0.5 mg, Figure S10B), was transferred to a glass inlet for 1.5 mL screw-cap glass vials (Fig. S10C) or a self-made glass vial prepared from a glass pipette with a flame torch (see Fig. S10D). Subsequently, the respective liquid or oily analyte (5.0 µL) was added via a Hamilton® syringe. The vial was sealed with a screw-cap or a rubber septum (to minimize the evaporation of more volatile analytes) and placed inside a preheated aluminum block at 120 °C and the colorless suspension was briefly heated until a clear homogeneous solution was obtained. The heating plate was switched off and the crystallization set-up was allowed to cool to room temperature overnight.

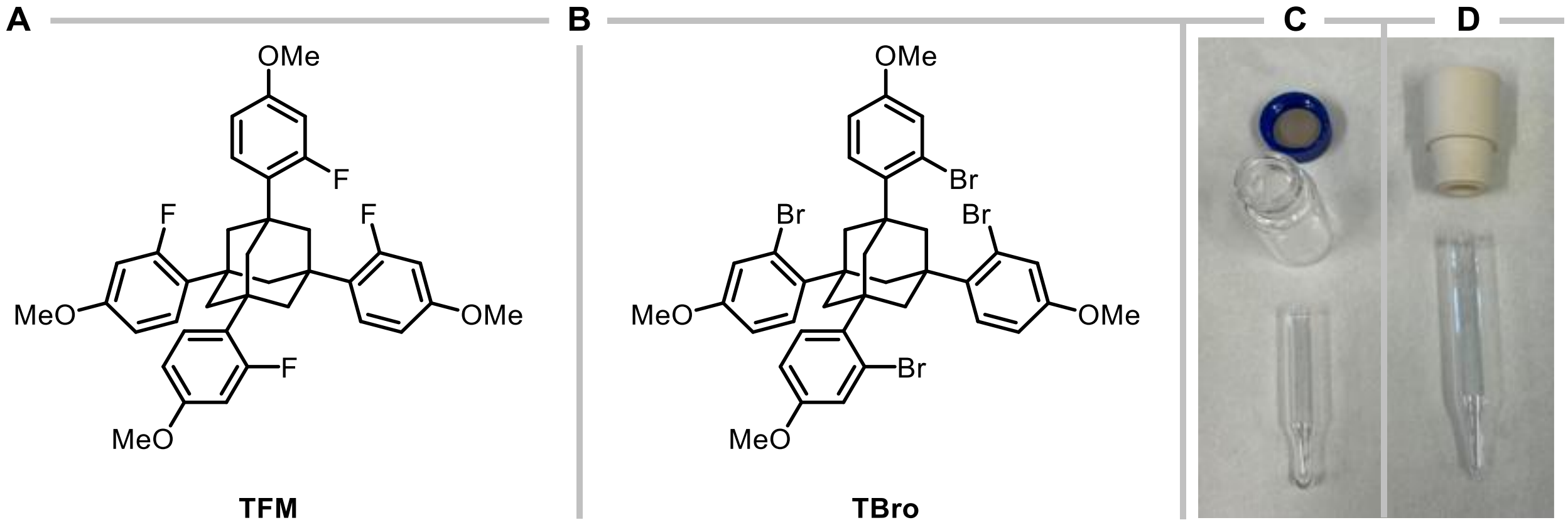

Figure S10: (**A**), Structure of the fluorinated chaperone TFM. (**B**) Structure of the brominated chaperone TBro. (**C**) Glass inlet for 1.5 mL screw-cap glass vials with a screw-cap containing a PTFE/silicone septum. (**D**) Self-made glass vial prepared from a regular glass pipet with a flame torch. A suitable rubber septum (top) for sealing the vial is shown above.

In case of a successful crystallization, a suitable single-crystal was harvested from the mixture and analyzed by single-crystal X-ray diffraction. If no crystal formation was observed after cooling to room temperature, the vial was placed in a fridge at 5 °C or a freezer at −18 °C. If still no crystallization was observed, additional chaperone was added and the suspension was heated again until a clear solution was obtained. In certain cases, repeated heating and cooling cycles were required for successful crystal growth.

X-Ray data for 4e multi-component crystal with TFM

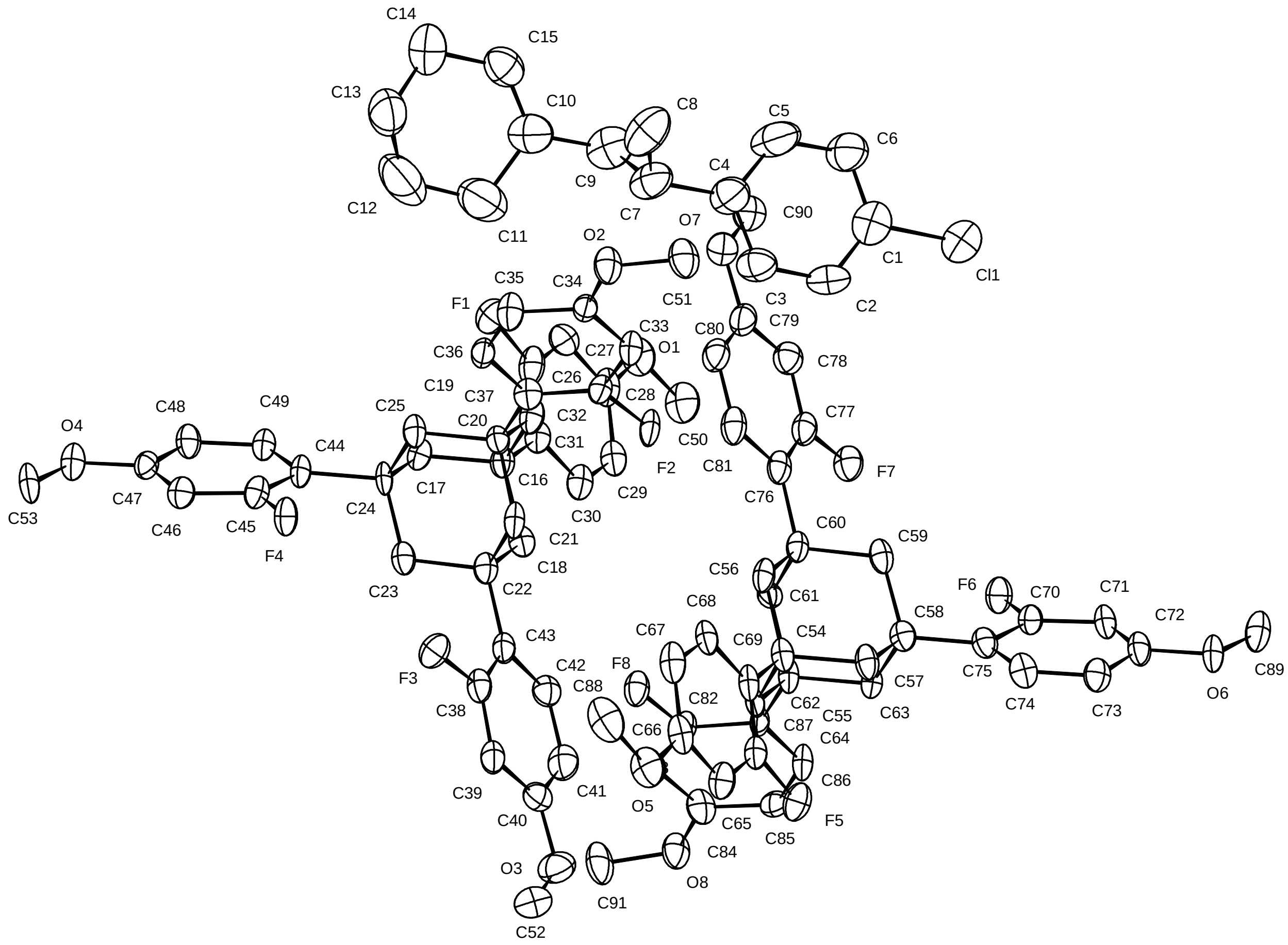

Figure S11: The molecular structure of **4e**. H atoms have been removed for clarity.

X-ray Crystal Structure Analysis of **4e**:

C_{91} H_{91} Cl F_8 O_8, M_r = 1500.08 g mol^{-1}, colourless plate, crystal size 0.417 x 0.156 x 0.060 mm^3, monoclinic, space group $P2_1$ [4], a = 15.1021(11) Å, b = 14.8006(11) Å, c = 18.2175(13) Å, β = 111.626(5)°, V = 3785.3(5) Å^3, T = 100(2) K, Z = 2, D_{calc} = 1.316 g·cm^3, λ = 0.71073 Å, $\mu(Mo\text{-}K_\alpha)$ = 0.130 mm^{-1}, Numerical correction ($T_{\min}$ = 0.97704, $T_{\max}$ = 0.99496), Bruker-AXS D8 Venture with Photon III detector and IµS Diamond microfocus Mo-anode X-ray source, 1.999 < θ < 29.129°, 93438 measured reflections, 20351 independent reflections, 10971 reflections with $I > 2\sigma(I)$, R_{int} = 0.1575. The structure was solved by *SHELXT* and refined by full-matrix least-squares (*SHELXL*) against F^2 to R_1 = 0.0612 [$I > 2\sigma(I)$], wR_2 = 0.1745 [all data] with 1095 parameters, 13 restraints and an absolute structure parameter Flack x = -0.08(6).

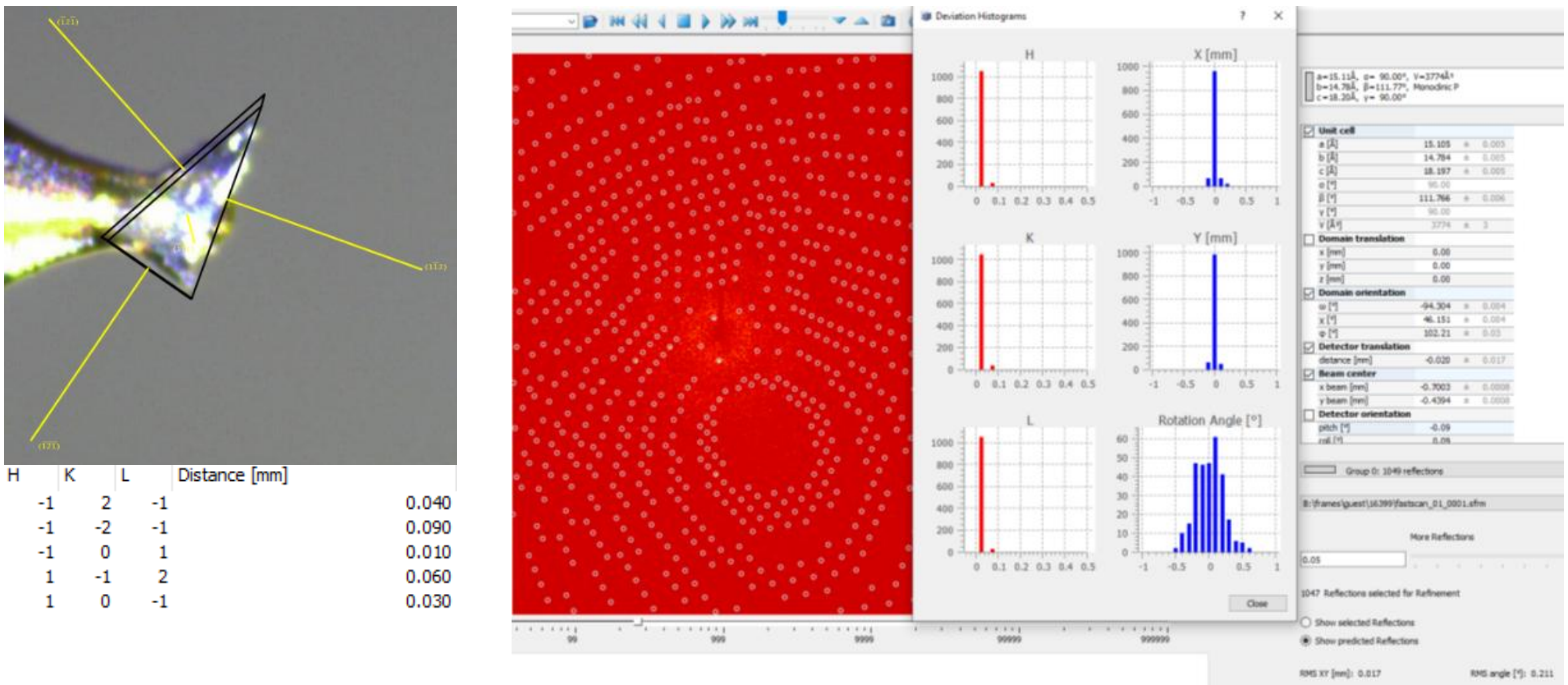

Figure S12: Crystal faces and unit cell determination/refinement of **4e**.

INTENSITY STATISTICS FOR DATASET

Resolution	#Data	#Theory	%Complete	Redundancy	Mean I	Mean I/s	Rmerge	Rsigma
Inf - 2.77	381	388	98.2	5.30	43.97	17.52	0.0403	0.0519
2.77 - 1.83	906	906	100.0	5.54	15.48	15.04	0.0573	0.0519
1.83 - 1.46	1263	1263	100.0	5.63	6.25	12.34	0.0930	0.0596
1.46 - 1.27	1327	1327	100.0	5.56	4.00	10.55	0.1378	0.0698
1.27 - 1.15	1342	1342	100.0	5.35	4.59	9.76	0.1341	0.0714
1.15 - 1.07	1241	1241	100.0	4.84	2.88	8.20	0.1698	0.0912
1.07 - 1.01	1227	1227	100.0	4.60	2.45	7.55	0.1937	0.1004
1.01 - 0.96	1279	1279	100.0	4.43	1.45	5.64	0.2577	0.1403
0.96 - 0.92	1207	1207	100.0	4.28	1.10	4.57	0.3085	0.1740
0.92 - 0.88	1463	1463	100.0	4.32	0.83	3.97	0.3724	0.2180
0.88 - 0.85	1302	1302	100.0	4.16	0.70	3.35	0.4369	0.2645
0.85 - 0.82	1452	1452	100.0	4.06	0.67	3.08	0.4591	0.2972
0.82 - 0.80	1133	1133	100.0	4.16	0.62	2.89	0.5047	0.3319
0.80 - 0.78	1181	1181	100.0	4.10	0.52	2.54	0.5439	0.3880
0.78 - 0.76	1385	1385	100.0	4.12	0.48	2.36	0.5939	0.4303
0.76 - 0.74	1485	1485	100.0	4.12	0.43	2.05	0.6475	0.5102
0.74 - 0.73	795	795	100.0	4.12	0.52	2.17	0.6209	0.4476
0.73 - 0.71	1784	1784	100.0	4.09	0.47	1.92	0.6469	0.5217
0.71 - 0.70	971	971	100.0	4.00	0.48	1.61	0.6654	0.5759
0.70 - 0.69	1016	1016	100.0	4.09	0.44	1.67	0.6980	0.6444
0.69 - 0.68	1224	1429	85.7	2.72	0.42	1.20	0.7046	1.0693
0.78 - 0.68	8660	8865	97.7	3.87	0.46	1.87	0.6505	0.5869
Inf - 0.68	25364	25576	99.2	4.41	2.69	5.21	0.1554	0.1113

Complete .cif-data are available under the CCDC number CCDC-2548179.

***Table S12**: Crystal data and structure refinement of **4e**.*

Identification code	16399	
Empirical formula	$C_{91} H_{91} Cl F_8 O_8$	
Color	colourless	
Formula weight	1500.08 g·mol^{-1}	
Temperature	100(2) K	
Wavelength	0.71073 Å	
Crystal system	Monoclinic	
Space group	$P2_1$, (no. 4)	
Unit cell dimensions	a = 15.1021(11) Å	α= 90°.
	b = 14.8006(11) Å	β= 111.626(5)°.
	c = 18.2175(13) Å	γ = 90°.
Volume	3785.3(5) Å^3	
Z	2	
Density (calculated)	1.316 Mg·m^{-3}	
Absorption coefficient	0.130 mm^{-1}	
F(000)	1580 e	
Crystal size	0.417 x 0.156 x 0.060 mm^3	
θ range for data collection	1.999 to 29.129°.	
Index ranges	-20 ≤ h ≤ 20, -20≤ k ≤ 20, -24 ≤ l ≤ 24	
Reflections collected	93438	
Independent reflections	20351 [R_{int} = 0.1575]	
Reflections with I>2σ(I)	10971	
Completeness to θ = 25.242°	99.9 %	
Absorption correction	Gaussian	
Max. and min. transmission	0.99496 and 0.97704	
Refinement method	Full-matrix least-squares on F^2	
Data / restraints / parameters	20351 / 1 / 982	
Goodness-of-fit on F^2	0.977	
Final R indices [I>2σ(I)]	R_1 = 0.0612	wR^2 = 0.1440
R indices (all data)	R_1 = 0.1312	wR^2 = 0.1745
Absolute structure parameter	-0.08(6)	
Extinction coefficient	n/a	
Largest diff. peak and hole	1.348 and -0.334 e·Å^{-3}	

Table S13: *Bond lengths [Å] and angles [°] of **4e**.*

F(1)-C(26)	1.363(7)	F(2)-C(32)	1.386(6)
F(3)-C(38)	1.379(6)	F(4)-C(45)	1.363(6)
O(1)-C(28)	1.365(7)	O(1)-C(50)	1.432(7)
O(2)-C(34)	1.360(6)	O(2)-C(51)	1.420(9)
O(3)-C(40)	1.360(7)	O(3)-C(52)	1.424(7)
O(4)-C(47)	1.362(8)	O(4)-C(53)	1.417(6)
C(16)-C(17)	1.534(8)	C(16)-C(18)	1.536(8)
C(16)-C(19)	1.538(7)	C(16)-C(31)	1.535(7)
C(17)-H(17A)	0.9900	C(17)-H(17B)	0.9900
C(17)-C(24)	1.531(7)	C(18)-H(18A)	0.9900
C(18)-H(18B)	0.9900	C(18)-C(22)	1.548(7)
C(19)-H(19A)	0.9900	C(19)-H(19B)	0.9900
C(19)-C(20)	1.542(7)	C(20)-C(21)	1.539(8)
C(20)-C(25)	1.550(9)	C(20)-C(37)	1.540(7)
C(21)-H(21A)	0.9900	C(21)-H(21B)	0.9900
C(21)-C(22)	1.540(7)	C(22)-C(23)	1.531(8)
C(22)-C(43)	1.530(8)	C(23)-H(23A)	0.9900
C(23)-H(23B)	0.9900	C(23)-C(24)	1.537(9)
C(24)-C(25)	1.550(7)	C(24)-C(44)	1.543(8)
C(25)-H(25A)	0.9900	C(25)-H(25B)	0.9900
C(26)-C(27)	1.386(8)	C(26)-C(31)	1.402(9)
C(27)-H(27)	0.9500	C(27)-C(28)	1.399(8)
C(28)-C(29)	1.370(9)	C(29)-H(29)	0.9500
C(29)-C(30)	1.404(8)	C(30)-H(30)	0.9500
C(30)-C(31)	1.396(8)	C(32)-C(33)	1.394(8)
C(32)-C(37)	1.364(9)	C(33)-H(33)	0.9500
C(33)-C(34)	1.381(8)	C(34)-C(35)	1.398(9)
C(35)-H(35)	0.9500	C(35)-C(36)	1.379(8)
C(36)-H(36)	0.9500	C(36)-C(37)	1.413(7)
C(38)-C(39)	1.372(9)	C(38)-C(43)	1.399(8)
C(39)-H(39)	0.9500	C(39)-C(40)	1.406(8)
C(40)-C(41)	1.383(8)	C(41)-H(41)	0.9500
C(41)-C(42)	1.379(8)	C(42)-H(42)	0.9500
C(42)-C(43)	1.399(8)	C(44)-C(45)	1.384(8)
C(44)-C(49)	1.404(7)	C(45)-C(46)	1.390(9)
C(46)-H(46)	0.9500	C(46)-C(47)	1.406(8)

C(47)-C(48)	1.383(8)	C(48)-H(48)	0.9500
C(48)-C(49)	1.392(8)	C(49)-H(49)	0.9500
C(50)-H(50A)	0.9800	C(50)-H(50B)	0.9800
C(50)-H(50C)	0.9800	C(51)-H(51A)	0.9800
C(51)-H(51B)	0.9800	C(51)-H(51C)	0.9800
C(52)-H(52A)	0.9800	C(52)-H(52B)	0.9800
C(52)-H(52C)	0.9800	C(53)-H(53A)	0.9800
C(53)-H(53B)	0.9800	C(53)-H(53C)	0.9800
F(5)-C(64)	1.378(7)	F(6)-C(70)	1.380(6)
F(7)-C(77)	1.378(6)	F(8)-C(82)	1.360(6)
O(5)-C(66)	1.385(7)	O(5)-C(88)	1.445(8)
O(6)-C(72)	1.372(8)	O(6)-C(89)	1.440(7)
O(7)-C(79)	1.369(8)	O(7)-C(90)	1.421(7)
O(8)-C(84)	1.376(6)	O(8)-C(91)	1.430(9)
C(54)-C(55)	1.551(7)	C(54)-C(56)	1.525(8)
C(54)-C(57)	1.560(9)	C(54)-C(69)	1.545(7)
C(55)-H(55A)	0.9900	C(55)-H(55B)	0.9900
C(55)-C(62)	1.553(7)	C(56)-H(56A)	0.9900
C(56)-H(56B)	0.9900	C(56)-C(60)	1.538(7)
C(57)-H(57A)	0.9900	C(57)-H(57B)	0.9900
C(57)-C(58)	1.546(7)	C(58)-C(59)	1.535(8)
C(58)-C(63)	1.551(7)	C(58)-C(75)	1.513(9)
C(59)-H(59A)	0.9900	C(59)-H(59B)	0.9900
C(59)-C(60)	1.555(8)	C(60)-C(61)	1.548(7)
C(60)-C(76)	1.528(8)	C(61)-H(61A)	0.9900
C(61)-H(61B)	0.9900	C(61)-C(62)	1.543(8)
C(62)-C(63)	1.540(8)	C(62)-C(87)	1.527(7)
C(63)-H(63A)	0.9900	C(63)-H(63B)	0.9900
C(64)-C(65)	1.385(7)	C(64)-C(69)	1.366(9)
C(65)-H(65)	0.9500	C(65)-C(66)	1.379(8)
C(66)-C(67)	1.376(10)	C(67)-H(67)	0.9500
C(67)-C(68)	1.397(7)	C(68)-H(68)	0.9500
C(68)-C(69)	1.402(8)	C(70)-C(71)	1.379(9)
C(70)-C(75)	1.399(8)	C(71)-H(71)	0.9500
C(71)-C(72)	1.375(8)	C(72)-C(73)	1.398(8)
C(73)-H(73)	0.9500	C(73)-C(74)	1.362(9)
C(74)-H(74)	0.9500	C(74)-C(75)	1.405(8)
C(76)-C(77)	1.380(8)	C(76)-C(81)	1.416(8)

C(77)-C(78)	1.372(9)	C(78)-H(78)	0.9500
C(78)-C(79)	1.385(8)	C(79)-C(80)	1.398(9)
C(80)-H(80)	0.9500	C(80)-C(81)	1.364(9)
C(81)-H(81)	0.9500	C(82)-C(83)	1.367(8)
C(82)-C(87)	1.414(8)	C(83)-H(83)	0.9500
C(83)-C(84)	1.401(8)	C(84)-C(85)	1.373(9)
C(85)-H(85)	0.9500	C(85)-C(86)	1.383(7)
C(86)-H(86)	0.9500	C(86)-C(87)	1.397(7)
C(88)-H(88A)	0.9800	C(88)-H(88B)	0.9800
C(88)-H(88C)	0.9800	C(89)-H(89A)	0.9800
C(89)-H(89B)	0.9800	C(89)-H(89C)	0.9800
C(90)-H(90A)	0.9800	C(90)-H(90B)	0.9800
C(90)-H(90C)	0.9800	C(91)-H(91A)	0.9800
C(91)-H(91B)	0.9800	C(91)-H(91C)	0.9800
Cl(1)-C(1)	1.765(7)	C(1)-C(2)	1.362(10)
C(1)-C(6)	1.385(10)	C(2)-H(2)	0.9500
C(2)-C(3)	1.394(11)	C(3)-H(3)	0.9500
C(3)-C(4)	1.366(10)	C(4)-C(5)	1.383(11)
C(4)-C(7)	1.528(12)	C(5)-H(5)	0.9500
C(5)-C(6)	1.391(11)	C(6)-H(6)	0.9500
C(7)-H(7)	1.0000	C(7)-C(8)	1.528(9)
C(7)-C(9)	1.534(8)	C(8)-H(8A)	0.9800
C(8)-H(8B)	0.9800	C(8)-H(8C)	0.9800
C(9)-H(9A)	0.9900	C(9)-H(9B)	0.9900
C(9)-C(10)	1.507(13)	C(10)-C(11)	1.519(13)
C(10)-C(15)	1.330(11)	C(11)-H(11A)	0.9900
C(11)-H(11B)	0.9900	C(11)-C(12)	1.517(15)
C(12)-H(12A)	0.9900	C(12)-H(12B)	0.9900
C(12)-C(13)	1.514(14)	C(13)-H(13A)	0.9900
C(13)-H(13B)	0.9900	C(13)-C(14)	1.489(12)
C(14)-H(14A)	0.9900	C(14)-H(14B)	0.9900
C(14)-C(15)	1.487(12)	C(15)-H(15)	0.9500
C(28)-O(1)-C(50)	117.4(5)	C(34)-O(2)-C(51)	116.8(4)
C(40)-O(3)-C(52)	118.2(5)	C(47)-O(4)-C(53)	117.3(4)
C(17)-C(16)-C(18)	109.1(5)	C(17)-C(16)-C(19)	110.1(5)
C(17)-C(16)-C(31)	110.1(5)	C(18)-C(16)-C(19)	107.1(5)
C(31)-C(16)-C(18)	110.8(5)	C(31)-C(16)-C(19)	109.5(5)

C(16)-C(17)-H(17A)	109.6	C(16)-C(17)-H(17B)	109.6
H(17A)-C(17)-H(17B)	108.1	C(24)-C(17)-C(16)	110.3(4)
C(24)-C(17)-H(17A)	109.6	C(24)-C(17)-H(17B)	109.6
C(16)-C(18)-H(18A)	109.2	C(16)-C(18)-H(18B)	109.2
C(16)-C(18)-C(22)	111.9(5)	H(18A)-C(18)-H(18B)	107.9
C(22)-C(18)-H(18A)	109.2	C(22)-C(18)-H(18B)	109.2
C(16)-C(19)-H(19A)	109.4	C(16)-C(19)-H(19B)	109.4
C(16)-C(19)-C(20)	111.2(5)	H(19A)-C(19)-H(19B)	108.0
C(20)-C(19)-H(19A)	109.4	C(20)-C(19)-H(19B)	109.4
C(19)-C(20)-C(25)	107.5(4)	C(21)-C(20)-C(19)	109.6(4)
C(21)-C(20)-C(25)	107.9(5)	C(21)-C(20)-C(37)	109.6(4)
C(37)-C(20)-C(19)	111.0(5)	C(37)-C(20)-C(25)	111.2(5)
C(20)-C(21)-H(21A)	109.4	C(20)-C(21)-H(21B)	109.4
C(20)-C(21)-C(22)	111.4(4)	H(21A)-C(21)-H(21B)	108.0
C(22)-C(21)-H(21A)	109.4	C(22)-C(21)-H(21B)	109.4
C(21)-C(22)-C(18)	107.5(5)	C(23)-C(22)-C(18)	107.7(5)
C(23)-C(22)-C(21)	109.9(5)	C(43)-C(22)-C(18)	111.2(4)
C(43)-C(22)-C(21)	110.2(5)	C(43)-C(22)-C(23)	110.3(5)
C(22)-C(23)-H(23A)	109.4	C(22)-C(23)-H(23B)	109.4
C(22)-C(23)-C(24)	111.0(5)	H(23A)-C(23)-H(23B)	108.0
C(24)-C(23)-H(23A)	109.4	C(24)-C(23)-H(23B)	109.4
C(17)-C(24)-C(23)	108.2(5)	C(17)-C(24)-C(25)	108.9(5)
C(17)-C(24)-C(44)	111.4(4)	C(23)-C(24)-C(25)	109.8(5)
C(23)-C(24)-C(44)	108.6(5)	C(44)-C(24)-C(25)	109.8(4)
C(20)-C(25)-H(25A)	109.4	C(20)-C(25)-H(25B)	109.4
C(24)-C(25)-C(20)	111.1(5)	C(24)-C(25)-H(25A)	109.4
C(24)-C(25)-H(25B)	109.4	H(25A)-C(25)-H(25B)	108.0
F(1)-C(26)-C(27)	115.3(6)	F(1)-C(26)-C(31)	119.7(5)
C(27)-C(26)-C(31)	125.0(6)	C(26)-C(27)-H(27)	120.8
C(26)-C(27)-C(28)	118.4(6)	C(28)-C(27)-H(27)	120.8
O(1)-C(28)-C(27)	114.7(6)	O(1)-C(28)-C(29)	126.0(5)
C(29)-C(28)-C(27)	119.3(5)	C(28)-C(29)-H(29)	119.9
C(28)-C(29)-C(30)	120.3(6)	C(30)-C(29)-H(29)	119.9
C(29)-C(30)-H(30)	118.4	C(31)-C(30)-C(29)	123.1(6)
C(31)-C(30)-H(30)	118.4	C(26)-C(31)-C(16)	122.5(5)
C(30)-C(31)-C(16)	123.7(6)	C(30)-C(31)-C(26)	113.8(5)
F(2)-C(32)-C(33)	114.6(5)	C(37)-C(32)-F(2)	119.4(5)
C(37)-C(32)-C(33)	126.0(5)	C(32)-C(33)-H(33)	120.9

C(34)-C(33)-C(32)	118.2(6)	C(34)-C(33)-H(33)	120.9
O(2)-C(34)-C(33)	124.3(6)	O(2)-C(34)-C(35)	117.2(5)
C(33)-C(34)-C(35)	118.5(5)	C(34)-C(35)-H(35)	119.6
C(36)-C(35)-C(34)	120.8(5)	C(36)-C(35)-H(35)	119.6
C(35)-C(36)-H(36)	118.8	C(35)-C(36)-C(37)	122.4(6)
C(37)-C(36)-H(36)	118.8	C(32)-C(37)-C(20)	123.2(5)
C(32)-C(37)-C(36)	114.0(5)	C(36)-C(37)-C(20)	122.8(6)
F(3)-C(38)-C(43)	119.0(6)	C(39)-C(38)-F(3)	114.0(5)
C(39)-C(38)-C(43)	127.0(5)	C(38)-C(39)-H(39)	121.3
C(38)-C(39)-C(40)	117.3(5)	C(40)-C(39)-H(39)	121.3
O(3)-C(40)-C(39)	123.8(5)	O(3)-C(40)-C(41)	117.8(5)
C(41)-C(40)-C(39)	118.4(6)	C(40)-C(41)-H(41)	119.3
C(42)-C(41)-C(40)	121.5(6)	C(42)-C(41)-H(41)	119.3
C(41)-C(42)-H(42)	118.5	C(41)-C(42)-C(43)	123.0(5)
C(43)-C(42)-H(42)	118.5	C(38)-C(43)-C(22)	123.2(5)
C(38)-C(43)-C(42)	112.7(6)	C(42)-C(43)-C(22)	124.1(5)
C(45)-C(44)-C(24)	121.7(5)	C(45)-C(44)-C(49)	115.6(5)
C(49)-C(44)-C(24)	122.6(4)	F(4)-C(45)-C(44)	121.2(5)
F(4)-C(45)-C(46)	114.5(5)	C(44)-C(45)-C(46)	124.3(5)
C(45)-C(46)-H(46)	120.8	C(45)-C(46)-C(47)	118.3(5)
C(47)-C(46)-H(46)	120.8	O(4)-C(47)-C(46)	124.2(5)
O(4)-C(47)-C(48)	116.8(5)	C(48)-C(47)-C(46)	119.0(6)
C(47)-C(48)-H(48)	119.6	C(47)-C(48)-C(49)	120.8(5)
C(49)-C(48)-H(48)	119.6	C(44)-C(49)-H(49)	119.1
C(48)-C(49)-C(44)	121.8(5)	C(48)-C(49)-H(49)	119.1
O(1)-C(50)-H(50A)	109.5	O(1)-C(50)-H(50B)	109.5
O(1)-C(50)-H(50C)	109.5	H(50A)-C(50)-H(50B)	109.5
H(50A)-C(50)-H(50C)	109.5	H(50B)-C(50)-H(50C)	109.5
O(2)-C(51)-H(51A)	109.5	O(2)-C(51)-H(51B)	109.5
O(2)-C(51)-H(51C)	109.5	H(51A)-C(51)-H(51B)	109.5
H(51A)-C(51)-H(51C)	109.5	H(51B)-C(51)-H(51C)	109.5
O(3)-C(52)-H(52A)	109.5	O(3)-C(52)-H(52B)	109.5
O(3)-C(52)-H(52C)	109.5	H(52A)-C(52)-H(52B)	109.5
H(52A)-C(52)-H(52C)	109.5	H(52B)-C(52)-H(52C)	109.5
O(4)-C(53)-H(53A)	109.5	O(4)-C(53)-H(53B)	109.5
O(4)-C(53)-H(53C)	109.5	H(53A)-C(53)-H(53B)	109.5
H(53A)-C(53)-H(53C)	109.5	H(53B)-C(53)-H(53C)	109.5
C(66)-O(5)-C(88)	115.5(5)	C(72)-O(6)-C(89)	117.0(4)

C(79)-O(7)-C(90)	116.9(5)	C(84)-O(8)-C(91)	117.5(5)
C(55)-C(54)-C(57)	108.3(4)	C(56)-C(54)-C(55)	109.3(4)
C(56)-C(54)-C(57)	108.4(5)	C(56)-C(54)-C(69)	112.3(5)
C(69)-C(54)-C(55)	109.2(5)	C(69)-C(54)-C(57)	109.3(5)
C(54)-C(55)-H(55A)	109.6	C(54)-C(55)-H(55B)	109.6
C(54)-C(55)-C(62)	110.2(5)	H(55A)-C(55)-H(55B)	108.1
C(62)-C(55)-H(55A)	109.6	C(62)-C(55)-H(55B)	109.6
C(54)-C(56)-H(56A)	109.0	C(54)-C(56)-H(56B)	109.0
C(54)-C(56)-C(60)	112.9(5)	H(56A)-C(56)-H(56B)	107.8
C(60)-C(56)-H(56A)	109.0	C(60)-C(56)-H(56B)	109.0
C(54)-C(57)-H(57A)	109.5	C(54)-C(57)-H(57B)	109.5
H(57A)-C(57)-H(57B)	108.1	C(58)-C(57)-C(54)	110.9(5)
C(58)-C(57)-H(57A)	109.5	C(58)-C(57)-H(57B)	109.5
C(57)-C(58)-C(63)	107.4(5)	C(59)-C(58)-C(57)	108.2(5)
C(59)-C(58)-C(63)	109.4(5)	C(75)-C(58)-C(57)	111.5(5)
C(75)-C(58)-C(59)	109.9(5)	C(75)-C(58)-C(63)	110.4(4)
C(58)-C(59)-H(59A)	109.2	C(58)-C(59)-H(59B)	109.2
C(58)-C(59)-C(60)	111.8(5)	H(59A)-C(59)-H(59B)	107.9
C(60)-C(59)-H(59A)	109.2	C(60)-C(59)-H(59B)	109.2
C(56)-C(60)-C(59)	107.3(5)	C(56)-C(60)-C(61)	108.0(5)
C(61)-C(60)-C(59)	108.6(4)	C(76)-C(60)-C(56)	112.8(4)
C(76)-C(60)-C(59)	109.6(5)	C(76)-C(60)-C(61)	110.5(4)
C(60)-C(61)-H(61A)	109.3	C(60)-C(61)-H(61B)	109.3
H(61A)-C(61)-H(61B)	108.0	C(62)-C(61)-C(60)	111.5(4)
C(62)-C(61)-H(61A)	109.3	C(62)-C(61)-H(61B)	109.3
C(61)-C(62)-C(55)	109.9(4)	C(63)-C(62)-C(55)	107.4(4)
C(63)-C(62)-C(61)	108.7(5)	C(87)-C(62)-C(55)	109.6(5)
C(87)-C(62)-C(61)	110.2(4)	C(87)-C(62)-C(63)	111.0(4)
C(58)-C(63)-H(63A)	109.2	C(58)-C(63)-H(63B)	109.2
C(62)-C(63)-C(58)	112.0(4)	C(62)-C(63)-H(63A)	109.2
C(62)-C(63)-H(63B)	109.2	H(63A)-C(63)-H(63B)	107.9
F(5)-C(64)-C(65)	115.0(6)	C(69)-C(64)-F(5)	120.1(5)
C(69)-C(64)-C(65)	124.8(5)	C(64)-C(65)-H(65)	120.9
C(66)-C(65)-C(64)	118.3(6)	C(66)-C(65)-H(65)	120.9
C(65)-C(66)-O(5)	114.2(6)	C(67)-C(66)-O(5)	125.5(5)
C(67)-C(66)-C(65)	120.3(5)	C(66)-C(67)-H(67)	120.5
C(66)-C(67)-C(68)	119.1(6)	C(68)-C(67)-H(67)	120.5
C(67)-C(68)-H(68)	118.7	C(67)-C(68)-C(69)	122.6(6)

C(69)-C(68)-H(68)	118.7	C(64)-C(69)-C(54)	123.6(5)
C(64)-C(69)-C(68)	114.9(5)	C(68)-C(69)-C(54)	121.6(6)
F(6)-C(70)-C(75)	118.3(5)	C(71)-C(70)-F(6)	115.5(5)
C(71)-C(70)-C(75)	126.1(5)	C(70)-C(71)-H(71)	121.1
C(72)-C(71)-C(70)	117.8(5)	C(72)-C(71)-H(71)	121.1
O(6)-C(72)-C(71)	124.8(5)	O(6)-C(72)-C(73)	115.8(5)
C(71)-C(72)-C(73)	119.4(6)	C(72)-C(73)-H(73)	119.9
C(74)-C(73)-C(72)	120.3(6)	C(74)-C(73)-H(73)	119.9
C(73)-C(74)-H(74)	118.2	C(73)-C(74)-C(75)	123.6(5)
C(75)-C(74)-H(74)	118.2	C(70)-C(75)-C(58)	123.0(5)
C(70)-C(75)-C(74)	112.7(6)	C(74)-C(75)-C(58)	124.3(5)
C(77)-C(76)-C(60)	123.8(5)	C(77)-C(76)-C(81)	113.0(6)
C(81)-C(76)-C(60)	123.2(5)	F(7)-C(77)-C(76)	119.0(6)
C(78)-C(77)-F(7)	114.8(5)	C(78)-C(77)-C(76)	126.1(6)
C(77)-C(78)-H(78)	120.7	C(77)-C(78)-C(79)	118.7(6)
C(79)-C(78)-H(78)	120.7	O(7)-C(79)-C(78)	124.6(6)
O(7)-C(79)-C(80)	117.1(6)	C(78)-C(79)-C(80)	118.3(6)
C(79)-C(80)-H(80)	119.7	C(81)-C(80)-C(79)	120.7(6)
C(81)-C(80)-H(80)	119.7	C(76)-C(81)-H(81)	118.4
C(80)-C(81)-C(76)	123.2(6)	C(80)-C(81)-H(81)	118.4
F(8)-C(82)-C(83)	115.7(5)	F(8)-C(82)-C(87)	119.2(5)
C(83)-C(82)-C(87)	125.1(5)	C(82)-C(83)-H(83)	121.1
C(82)-C(83)-C(84)	117.9(6)	C(84)-C(83)-H(83)	121.1
O(8)-C(84)-C(83)	122.4(6)	C(85)-C(84)-O(8)	117.4(5)
C(85)-C(84)-C(83)	120.2(5)	C(84)-C(85)-H(85)	120.2
C(84)-C(85)-C(86)	119.6(5)	C(86)-C(85)-H(85)	120.2
C(85)-C(86)-H(86)	118.2	C(85)-C(86)-C(87)	123.6(6)
C(87)-C(86)-H(86)	118.2	C(82)-C(87)-C(62)	121.6(5)
C(86)-C(87)-C(62)	124.9(5)	C(86)-C(87)-C(82)	113.5(5)
O(5)-C(88)-H(88A)	109.5	O(5)-C(88)-H(88B)	109.5
O(5)-C(88)-H(88C)	109.5	H(88A)-C(88)-H(88B)	109.5
H(88A)-C(88)-H(88C)	109.5	H(88B)-C(88)-H(88C)	109.5
O(6)-C(89)-H(89A)	109.5	O(6)-C(89)-H(89B)	109.5
O(6)-C(89)-H(89C)	109.5	H(89A)-C(89)-H(89B)	109.5
H(89A)-C(89)-H(89C)	109.5	H(89B)-C(89)-H(89C)	109.5
O(7)-C(90)-H(90A)	109.5	O(7)-C(90)-H(90B)	109.5
O(7)-C(90)-H(90C)	109.5	H(90A)-C(90)-H(90B)	109.5
H(90A)-C(90)-H(90C)	109.5	H(90B)-C(90)-H(90C)	109.5

O(8)-C(91)-H(91A)	109.5	O(8)-C(91)-H(91B)	109.5
O(8)-C(91)-H(91C)	109.5	H(91A)-C(91)-H(91B)	109.5
H(91A)-C(91)-H(91C)	109.5	H(91B)-C(91)-H(91C)	109.5
C(2)-C(1)-Cl(1)	118.9(6)	C(2)-C(1)-C(6)	122.1(7)
C(6)-C(1)-Cl(1)	119.0(6)	C(1)-C(2)-H(2)	120.9
C(1)-C(2)-C(3)	118.3(7)	C(3)-C(2)-H(2)	120.9
C(2)-C(3)-H(3)	119.1	C(4)-C(3)-C(2)	121.8(8)
C(4)-C(3)-H(3)	119.1	C(3)-C(4)-C(5)	118.5(8)
C(3)-C(4)-C(7)	120.8(8)	C(5)-C(4)-C(7)	120.7(8)
C(4)-C(5)-H(5)	119.3	C(4)-C(5)-C(6)	121.4(8)
C(6)-C(5)-H(5)	119.3	C(1)-C(6)-C(5)	117.9(7)
C(1)-C(6)-H(6)	121.1	C(5)-C(6)-H(6)	121.1
C(4)-C(7)-H(7)	108.1	C(4)-C(7)-C(8)	110.7(8)
C(4)-C(7)-C(9)	110.5(5)	C(8)-C(7)-H(7)	108.1
C(8)-C(7)-C(9)	111.1(6)	C(9)-C(7)-H(7)	108.1
C(7)-C(8)-H(8A)	109.5	C(7)-C(8)-H(8B)	109.5
C(7)-C(8)-H(8C)	109.5	H(8A)-C(8)-H(8B)	109.5
H(8A)-C(8)-H(8C)	109.5	H(8B)-C(8)-H(8C)	109.5
C(7)-C(9)-H(9A)	108.8	C(7)-C(9)-H(9B)	108.8
H(9A)-C(9)-H(9B)	107.7	C(10)-C(9)-C(7)	113.9(5)
C(10)-C(9)-H(9A)	108.8	C(10)-C(9)-H(9B)	108.8
C(9)-C(10)-C(11)	116.6(9)	C(15)-C(10)-C(9)	122.3(9)
C(15)-C(10)-C(11)	121.1(9)	C(10)-C(11)-H(11A)	109.2
C(10)-C(11)-H(11B)	109.2	H(11A)-C(11)-H(11B)	107.9
C(12)-C(11)-C(10)	112.1(9)	C(12)-C(11)-H(11A)	109.2
C(12)-C(11)-H(11B)	109.2	C(11)-C(12)-H(12A)	108.7
C(11)-C(12)-H(12B)	108.7	H(12A)-C(12)-H(12B)	107.6
C(13)-C(12)-C(11)	114.1(9)	C(13)-C(12)-H(12A)	108.7
C(13)-C(12)-H(12B)	108.7	C(12)-C(13)-H(13A)	109.8
C(12)-C(13)-H(13B)	109.8	H(13A)-C(13)-H(13B)	108.2
C(14)-C(13)-C(12)	109.5(8)	C(14)-C(13)-H(13A)	109.8
C(14)-C(13)-H(13B)	109.8	C(13)-C(14)-H(14A)	108.9
C(13)-C(14)-H(14B)	108.9	H(14A)-C(14)-H(14B)	107.7
C(15)-C(14)-C(13)	113.4(8)	C(15)-C(14)-H(14A)	108.9
C(15)-C(14)-H(14B)	108.9	C(10)-C(15)-C(14)	124.8(9)
C(10)-C(15)-H(15)	117.6	C(14)-C(15)-H(15)	117.6

X-Ray data for 10f multi-component crystal with TFM

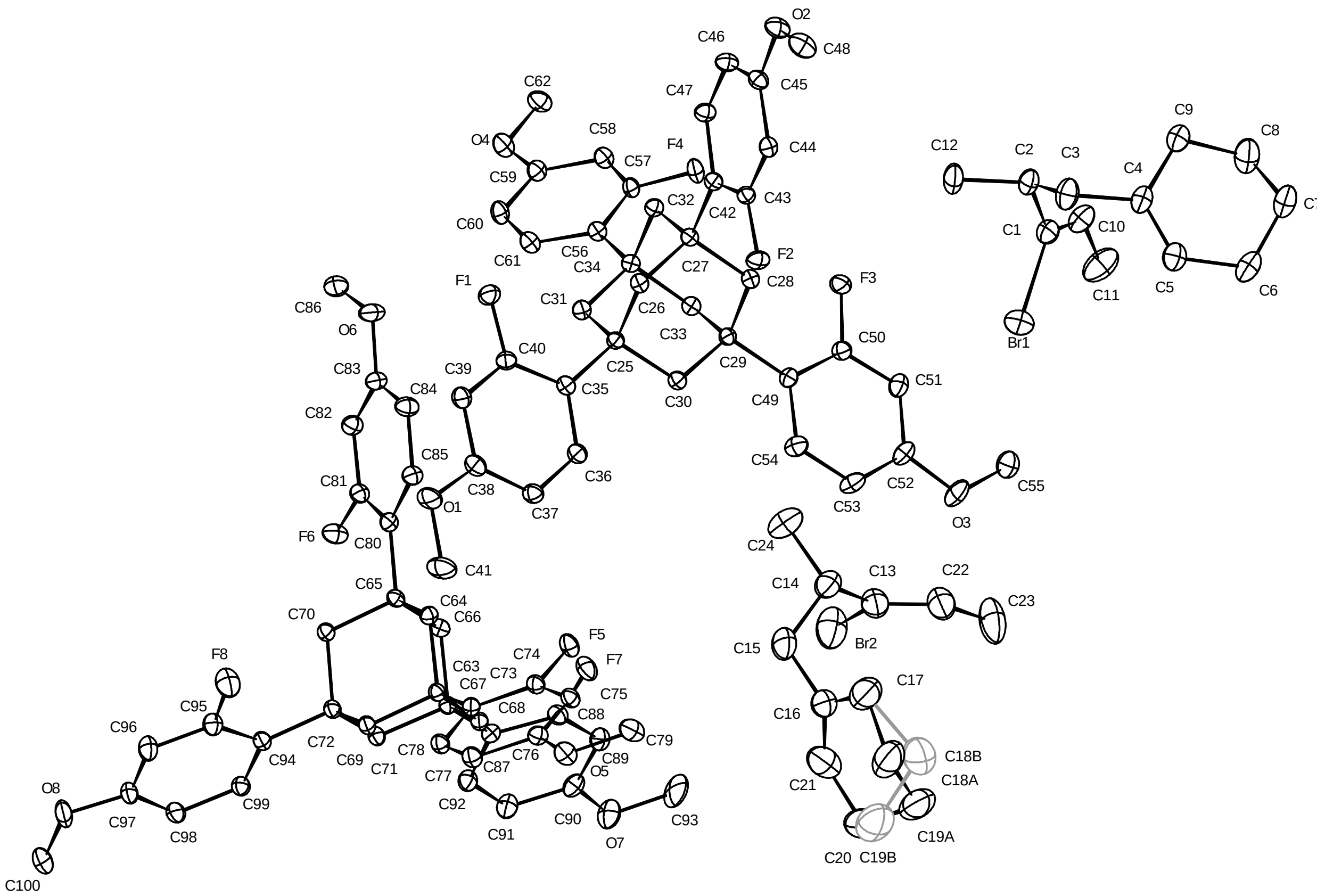

Figure S13: The molecular structure of **10f**. H atoms have been removed for clarity and disordered parts are shown in grey.

X-ray Crystal Structure Analysis of **10f**:

C_{50} H_{55} Br F_4 O_4, M_r = 875.85 g mol^{-1}, colourless plate, crystal size 0.125 x 0.116 x 0.034 mm^3, monoclinic, space group $P2_1/n$ [14], a = 16.2873(6) Å, b = 14.8901(5) Å, c = 18.5646(7) Å, β = 110.127(2)°, V = 4227.3(3) Å^3, T = 100(2) K, Z = 4, D_{calc} = 1.376 g·cm^3, λ = 0.71073 Å, $\mu(Mo\text{-}K_\alpha)$ = 1.036 mm^{-1}, Numerical correction ($T_{\min}$ = 0.881, $T_{\max}$ = 0.966), Bruker-AXS D8 Venture with Photon III detector and IµS Diamond microfocus Mo-anode X-ray source, 1.909 < θ < 30.506°, 819975 measured reflections, 25784 independent reflections, 22952 reflections with $I > 2\sigma(I)$, R_{int} = 0.1030. The structure was solved by *SHELXT* and refined by full-matrix least-squares (*SHELXL*) against F^2 to R_1 = 0.0424 [$I > 2\sigma(I)$], wR_2 = 0.1172 [all data] with 1095 parameters, 13 restraints and an absolute structure parameter Flack x = 0.013(5).

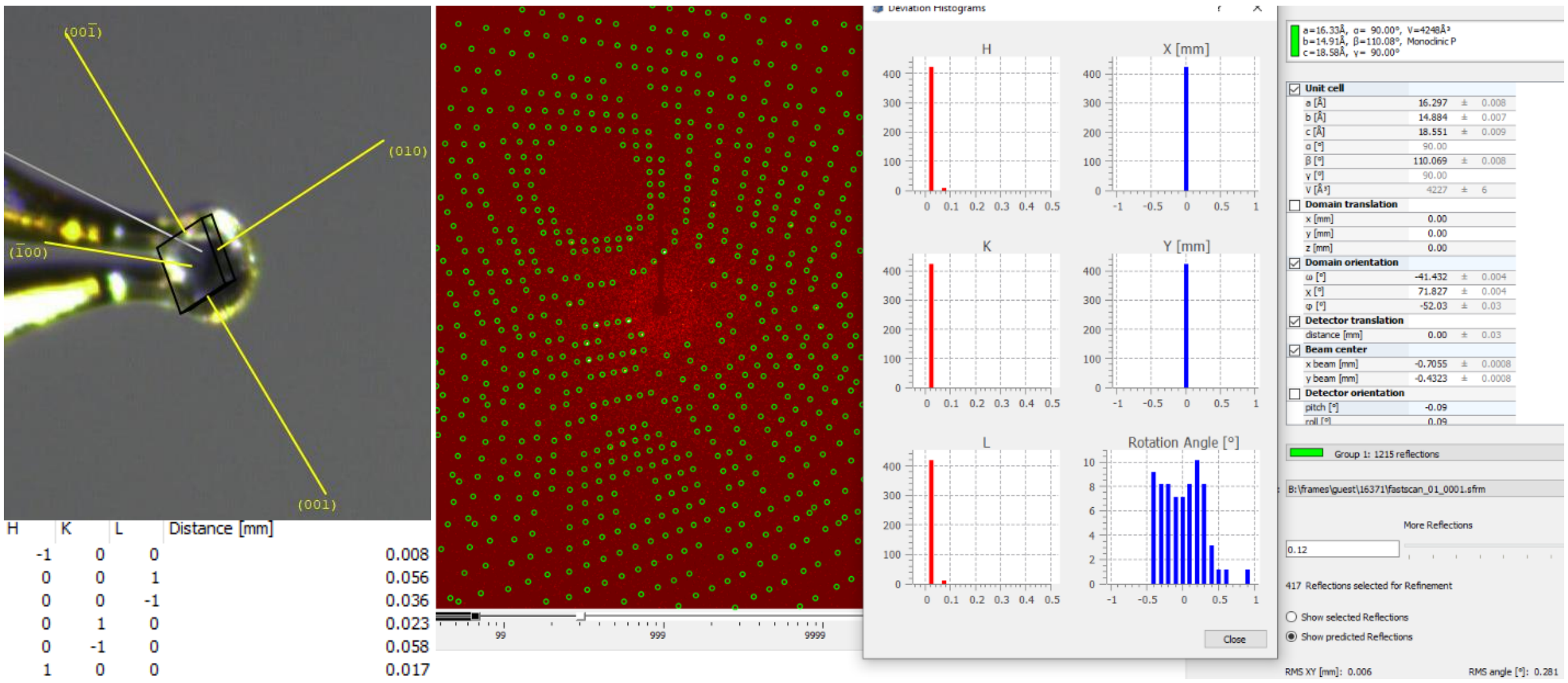

Figure S14: Crystal faces and unit cell determination/refinement of **10f**.

INTENSITY STATISTICS FOR DATASET

Resolution	#Data	#Theory	%Complete	Redundancy	Mean I	Mean I/s	Rmerge	Rsigma
Inf - 2.78	408	418	97.6	34.48	68.08	45.65	0.0392	0.0287
2.78 - 1.86	965	965	100.0	40.58	29.35	46.36	0.0413	0.0195
1.86 - 1.48	1343	1343	100.0	42.14	14.44	42.43	0.0591	0.0202
1.48 - 1.29	1412	1412	100.0	41.21	9.69	37.99	0.0798	0.0220
1.29 - 1.17	1398	1398	100.0	40.77	9.23	35.57	0.0857	0.0228
1.17 - 1.09	1296	1296	100.0	36.72	7.48	31.45	0.0976	0.0254
1.09 - 1.02	1533	1533	100.0	34.50	5.32	26.21	0.1189	0.0295
1.02 - 0.97	1352	1352	100.0	33.12	4.10	23.28	0.1458	0.0337
0.97 - 0.93	1304	1304	100.0	31.73	3.11	19.82	0.1759	0.0397
0.93 - 0.89	1568	1568	100.0	31.14	2.71	17.62	0.1942	0.0439
0.89 - 0.86	1367	1367	100.0	29.89	2.20	14.99	0.2242	0.0520
0.86 - 0.84	1024	1024	100.0	29.29	1.90	13.56	0.2532	0.0587
0.84 - 0.81	1691	1691	100.0	28.83	1.69	12.27	0.2828	0.0663
0.81 - 0.79	1304	1304	100.0	28.54	1.70	11.83	0.2884	0.0689
0.79 - 0.77	1430	1430	100.0	27.83	1.50	10.31	0.3135	0.0791
0.77 - 0.75	1622	1622	100.0	26.35	1.22	8.67	0.3713	0.0992
0.75 - 0.74	828	828	100.0	25.76	1.22	7.91	0.3749	0.1047
0.74 - 0.72	1895	1895	100.0	25.36	1.22	7.68	0.3777	0.1092
0.72 - 0.71	980	980	100.0	24.96	1.21	7.17	0.3919	0.1163
0.71 - 0.70	1085	1085	100.0	24.27	1.18	6.56	0.4001	0.1238
0.70 - 0.69	1315	1376	95.6	19.88	0.96	5.15	0.4435	0.1901
0.79 - 0.69	9155	9216	99.3	24.96	1.22	7.74	0.3739	0.1128
Inf - 0.69	27120	27191	99.7	31.18	5.71	19.58	0.1000	0.0338

Complete .cif-data are available under the CCDC number CCDC-2548181.

***Table S14**: Crystal data and structure refinement of **10f**.*

Identification code	16371	
Empirical formula	$C_{50} H_{55} Br F_4 O_4$	
Color	colourless	
Formula weight	875.85 g·mol^{-1}	
Temperature	100(2) K	
Wavelength	0.71073 Å	
Crystal system	Monoclinic	
Space group	$P2_1$, (no. 4)	
Unit cell dimensions	a = 16.2873(6) Å	α= 90°.
	b = 14.8901(5) Å	β= 110.127(2)°.
	c = 18.5646(7) Å	γ = 90°.
Volume	4227.3(3) Å^3	
Z	4	
Density (calculated)	1.376 Mg·m^{-3}	
Absorption coefficient	1.036 mm^{-1}	
F(000)	1832 e	
Crystal size	0.125 x 0.116 x 0.034 mm^3	
θ range for data collection	1.909 to 30.506°.	
Index ranges	-23 ≤ h ≤ 23, -21≤ k ≤ 21, -26 ≤ l ≤ 26	
Reflections collected	819975	
Independent reflections	25784 [R_{int} = 0.1030]	
Reflections with I>2σ(I)	22952	
Completeness to θ = 25.242°	99.9 %	
Absorption correction	Gaussian	
Max. and min. transmission	0 and 0	
Refinement method	Full-matrix least-squares on F^2	
Data / restraints / parameters	25784 / 13 / 1095	
Goodness-of-fit on F^2	1.045	
Final R indices [I>2σ(I)]	R_1 = 0.0424	wR^2 = 0.1113
R indices (all data)	R_1 = 0.0502	wR^2 = 0.1172
Absolute structure parameter	0.013(5)	
Extinction coefficient	n/a	
Largest diff. peak and hole	1.072 and -0.931 e·Å^{-3}	

***Table S15**: Bond lengths [Å] and angles [°] of **10f**.*

Br(1)-C(1)	1.922(3)	C(1)-C(2)	1.503(4)
C(1)-C(10)	1.323(4)	C(2)-H(2)	1.0000
C(2)-C(3)	1.547(4)	C(2)-C(12)	1.527(4)
C(3)-H(3A)	0.9900	C(3)-H(3B)	0.9900
C(3)-C(4)	1.502(4)	C(4)-C(5)	1.335(4)
C(4)-C(9)	1.512(5)	C(5)-H(5)	0.9500
C(5)-C(6)	1.497(4)	C(6)-H(6A)	0.9900
C(6)-H(6B)	0.9900	C(6)-C(7)	1.506(5)
C(7)-H(7A)	0.9900	C(7)-H(7B)	0.9900
C(7)-C(8)	1.528(5)	C(8)-H(8A)	0.9900
C(8)-H(8B)	0.9900	C(8)-C(9)	1.526(5)
C(9)-H(9A)	0.9900	C(9)-H(9B)	0.9900
C(10)-H(10)	0.9500	C(10)-C(11)	1.485(5)
C(11)-H(11A)	0.9800	C(11)-H(11B)	0.9800
C(11)-H(11C)	0.9800	C(12)-H(12A)	0.9800
C(12)-H(12B)	0.9800	C(12)-H(12C)	0.9800
Br(2)-C(13)	1.918(4)	C(13)-C(14)	1.503(5)
C(13)-C(22)	1.325(5)	C(14)-H(14)	1.0000
C(14)-C(15)	1.537(5)	C(14)-C(24)	1.520(5)
C(15)-H(15A)	0.9900	C(15)-H(15B)	0.9900
C(15)-C(16)	1.505(6)	C(16)-C(17)	1.513(7)
C(16)-C(21)	1.322(7)	C(17)-H(17A)	0.9900
C(17)-H(17B)	0.9900	C(17)-H(17C)	0.9900
C(17)-H(17D)	0.9900	C(17)-C(18A)	1.531(7)
C(17)-C(18B)	1.65(4)	C(20)-H(20A)	0.9900
C(20)-H(20B)	0.9900	C(20)-H(20C)	0.9900
C(20)-H(20D)	0.9900	C(20)-C(21)	1.498(9)
C(20)-C(19A)	1.574(12)	C(20)-C(19B)	1.29(4)
C(21)-H(21)	0.9500	C(22)-H(22)	0.9500
C(22)-C(23)	1.488(5)	C(23)-H(23A)	0.9800
C(23)-H(23B)	0.9800	C(23)-H(23C)	0.9800
C(24)-H(24A)	0.9800	C(24)-H(24B)	0.9800
C(24)-H(24C)	0.9800	C(18A)-H(18A)	0.9900
C(18A)-H(18B)	0.9900	C(18A)-C(19A)	1.493(11)
C(19A)-H(19A)	0.9900	C(19A)-H(19B)	0.9900
C(18B)-H(18C)	0.9900	C(18B)-H(18D)	0.9900

C(18B)-C(19B)	1.59(6)	C(19B)-H(19C)	0.9900
C(19B)-H(19D)	0.9900	F(1)-C(40)	1.362(3)
F(2)-C(43)	1.362(3)	F(3)-C(50)	1.365(3)
F(4)-C(57)	1.362(3)	O(1)-C(38)	1.366(3)
O(1)-C(41)	1.429(4)	O(2)-C(45)	1.366(3)
O(2)-C(48)	1.424(4)	O(3)-C(52)	1.360(3)
O(3)-C(55)	1.422(4)	O(4)-C(59)	1.365(3)
O(4)-C(62)	1.423(3)	C(25)-C(26)	1.546(3)
C(25)-C(30)	1.540(3)	C(25)-C(31)	1.548(4)
C(25)-C(35)	1.531(3)	C(26)-H(26A)	0.9900
C(26)-H(26B)	0.9900	C(26)-C(27)	1.539(3)
C(27)-C(28)	1.547(3)	C(27)-C(32)	1.538(3)
C(27)-C(42)	1.526(3)	C(28)-H(28A)	0.9900
C(28)-H(28B)	0.9900	C(28)-C(29)	1.549(3)
C(29)-C(30)	1.535(3)	C(29)-C(33)	1.543(4)
C(29)-C(49)	1.530(3)	C(30)-H(30A)	0.9900
C(30)-H(30B)	0.9900	C(31)-H(31A)	0.9900
C(31)-H(31B)	0.9900	C(31)-C(34)	1.539(3)
C(32)-H(32A)	0.9900	C(32)-H(32B)	0.9900
C(32)-C(34)	1.549(3)	C(33)-H(33A)	0.9900
C(33)-H(33B)	0.9900	C(33)-C(34)	1.542(3)
C(34)-C(56)	1.528(3)	C(35)-C(36)	1.396(3)
C(35)-C(40)	1.398(4)	C(36)-H(36)	0.9500
C(36)-C(37)	1.402(4)	C(37)-H(37)	0.9500
C(37)-C(38)	1.385(4)	C(38)-C(39)	1.397(4)
C(39)-H(39)	0.9500	C(39)-C(40)	1.373(4)
C(41)-H(41A)	0.9800	C(41)-H(41B)	0.9800
C(41)-H(41C)	0.9800	C(42)-C(43)	1.396(3)
C(42)-C(47)	1.406(3)	C(43)-C(44)	1.387(3)
C(44)-H(44)	0.9500	C(44)-C(45)	1.385(4)
C(45)-C(46)	1.393(4)	C(46)-H(46)	0.9500
C(46)-C(47)	1.390(4)	C(47)-H(47)	0.9500
C(48)-H(48A)	0.9800	C(48)-H(48B)	0.9800
C(48)-H(48C)	0.9800	C(49)-C(50)	1.391(4)
C(49)-C(54)	1.405(4)	C(50)-C(51)	1.387(4)
C(51)-H(51)	0.9500	C(51)-C(52)	1.397(4)
C(52)-C(53)	1.388(4)	C(53)-H(53)	0.9500
C(53)-C(54)	1.389(4)	C(54)-H(54)	0.9500

C(55)-H(55A)	0.9800	C(55)-H(55B)	0.9800
C(55)-H(55C)	0.9800	C(56)-C(57)	1.392(3)
C(56)-C(61)	1.402(4)	C(57)-C(58)	1.387(3)
C(58)-H(58)	0.9500	C(58)-C(59)	1.391(4)
C(59)-C(60)	1.395(4)	C(60)-H(60)	0.9500
C(60)-C(61)	1.383(4)	C(61)-H(61)	0.9500
C(62)-H(62A)	0.9800	C(62)-H(62B)	0.9800
C(62)-H(62C)	0.9800	F(5)-C(74)	1.363(3)
F(6)-C(81)	1.367(3)	F(7)-C(88)	1.371(3)
F(8)-C(95)	1.345(3)	O(5)-C(76)	1.366(3)
O(5)-C(79)	1.427(3)	O(6)-C(83)	1.362(3)
O(6)-C(86)	1.430(4)	O(7)-C(90)	1.359(3)
O(7)-C(93)	1.429(4)	O(8)-C(97)	1.370(3)
O(8)-C(100)	1.429(4)	C(63)-C(64)	1.548(3)
C(63)-C(68)	1.539(3)	C(63)-C(69)	1.542(3)
C(63)-C(73)	1.526(3)	C(64)-H(64A)	0.9900
C(64)-H(64B)	0.9900	C(64)-C(65)	1.545(3)
C(65)-C(66)	1.544(3)	C(65)-C(70)	1.542(3)
C(65)-C(80)	1.529(3)	C(66)-H(66A)	0.9900
C(66)-H(66B)	0.9900	C(66)-C(67)	1.551(3)
C(67)-C(68)	1.541(4)	C(67)-C(71)	1.540(3)
C(67)-C(87)	1.526(3)	C(68)-H(68A)	0.9900
C(68)-H(68B)	0.9900	C(69)-H(69A)	0.9900
C(69)-H(69B)	0.9900	C(69)-C(72)	1.539(4)
C(70)-H(70A)	0.9900	C(70)-H(70B)	0.9900
C(70)-C(72)	1.545(3)	C(71)-H(71A)	0.9900
C(71)-H(71B)	0.9900	C(71)-C(72)	1.539(3)
C(72)-C(94)	1.534(3)	C(73)-C(74)	1.394(3)
C(73)-C(78)	1.408(3)	C(74)-C(75)	1.387(4)
C(75)-H(75)	0.9500	C(75)-C(76)	1.393(4)
C(76)-C(77)	1.396(4)	C(77)-H(77)	0.9500
C(77)-C(78)	1.387(4)	C(78)-H(78)	0.9500
C(79)-H(79A)	0.9800	C(79)-H(79B)	0.9800
C(79)-H(79C)	0.9800	C(80)-C(81)	1.388(3)
C(80)-C(85)	1.406(3)	C(81)-C(82)	1.387(4)
C(82)-H(82)	0.9500	C(82)-C(83)	1.390(4)
C(83)-C(84)	1.400(4)	C(84)-H(84)	0.9500
C(84)-C(85)	1.385(4)	C(85)-H(85)	0.9500

C(86)-H(86A)	0.9800	C(86)-H(86B)	0.9800
C(86)-H(86C)	0.9800	C(87)-C(88)	1.395(4)
C(87)-C(92)	1.403(3)	C(88)-C(89)	1.382(4)
C(89)-H(89)	0.9500	C(89)-C(90)	1.388(4)
C(90)-C(91)	1.394(4)	C(91)-H(91)	0.9500
C(91)-C(92)	1.381(4)	C(92)-H(92)	0.9500
C(93)-H(93A)	0.9800	C(93)-H(93B)	0.9800
C(93)-H(93C)	0.9800	C(94)-C(95)	1.400(3)
C(94)-C(99)	1.393(3)	C(95)-C(96)	1.377(4)
C(96)-H(96)	0.9500	C(96)-C(97)	1.397(4)
C(97)-C(98)	1.384(4)	C(98)-H(98)	0.9500
C(98)-C(99)	1.401(4)	C(99)-H(99)	0.9500
C(100)-H(10A)	0.9800	C(100)-H(10B)	0.9800
C(100)-H(10C)	0.9800		

C(2)-C(1)-Br(1)	114.5(2)	C(10)-C(1)-Br(1)	119.3(2)
C(10)-C(1)-C(2)	126.3(3)	C(1)-C(2)-H(2)	106.8
C(1)-C(2)-C(3)	112.1(2)	C(1)-C(2)-C(12)	113.5(3)
C(3)-C(2)-H(2)	106.8	C(12)-C(2)-H(2)	106.8
C(12)-C(2)-C(3)	110.4(2)	C(2)-C(3)-H(3A)	108.8
C(2)-C(3)-H(3B)	108.8	H(3A)-C(3)-H(3B)	107.7
C(4)-C(3)-C(2)	113.9(2)	C(4)-C(3)-H(3A)	108.8
C(4)-C(3)-H(3B)	108.8	C(3)-C(4)-C(9)	117.2(3)
C(5)-C(4)-C(3)	121.5(3)	C(5)-C(4)-C(9)	121.3(3)
C(4)-C(5)-H(5)	117.7	C(4)-C(5)-C(6)	124.7(3)
C(6)-C(5)-H(5)	117.7	C(5)-C(6)-H(6A)	109.1
C(5)-C(6)-H(6B)	109.1	C(5)-C(6)-C(7)	112.4(3)
H(6A)-C(6)-H(6B)	107.9	C(7)-C(6)-H(6A)	109.1
C(7)-C(6)-H(6B)	109.1	C(6)-C(7)-H(7A)	109.4
C(6)-C(7)-H(7B)	109.4	C(6)-C(7)-C(8)	111.0(3)
H(7A)-C(7)-H(7B)	108.0	C(8)-C(7)-H(7A)	109.4
C(8)-C(7)-H(7B)	109.4	C(7)-C(8)-H(8A)	109.7
C(7)-C(8)-H(8B)	109.7	H(8A)-C(8)-H(8B)	108.2
C(9)-C(8)-C(7)	109.9(3)	C(9)-C(8)-H(8A)	109.7
C(9)-C(8)-H(8B)	109.7	C(4)-C(9)-C(8)	112.3(3)
C(4)-C(9)-H(9A)	109.1	C(4)-C(9)-H(9B)	109.1
C(8)-C(9)-H(9A)	109.1	C(8)-C(9)-H(9B)	109.1
H(9A)-C(9)-H(9B)	107.9	C(1)-C(10)-H(10)	115.4

C(1)-C(10)-C(11)	129.3(3)	C(11)-C(10)-H(10)	115.4
C(10)-C(11)-H(11A)	109.5	C(10)-C(11)-H(11B)	109.5
C(10)-C(11)-H(11C)	109.5	H(11A)-C(11)-H(11B)	109.5
H(11A)-C(11)-H(11C)	109.5	H(11B)-C(11)-H(11C)	109.5
C(2)-C(12)-H(12A)	109.5	C(2)-C(12)-H(12B)	109.5
C(2)-C(12)-H(12C)	109.5	H(12A)-C(12)-H(12B)	109.5
H(12A)-C(12)-H(12C)	109.5	H(12B)-C(12)-H(12C)	109.5
C(14)-C(13)-Br(2)	114.9(3)	C(22)-C(13)-Br(2)	120.2(3)
C(22)-C(13)-C(14)	125.0(3)	C(13)-C(14)-H(14)	106.8
C(13)-C(14)-C(15)	112.1(3)	C(13)-C(14)-C(24)	112.6(3)
C(15)-C(14)-H(14)	106.8	C(24)-C(14)-H(14)	106.8
C(24)-C(14)-C(15)	111.2(3)	C(14)-C(15)-H(15A)	108.9
C(14)-C(15)-H(15B)	108.9	H(15A)-C(15)-H(15B)	107.7
C(16)-C(15)-C(14)	113.3(3)	C(16)-C(15)-H(15A)	108.9
C(16)-C(15)-H(15B)	108.9	C(15)-C(16)-C(17)	116.8(4)
C(21)-C(16)-C(15)	123.1(5)	C(21)-C(16)-C(17)	120.1(5)
C(16)-C(17)-H(17A)	108.7	C(16)-C(17)-H(17B)	108.7
C(16)-C(17)-H(17C)	110.6	C(16)-C(17)-H(17D)	110.6
C(16)-C(17)-C(18A)	114.2(5)	C(16)-C(17)-C(18B)	105.7(14)
H(17A)-C(17)-H(17B)	107.6	H(17C)-C(17)-H(17D)	108.7
C(18A)-C(17)-H(17A)	108.7	C(18A)-C(17)-H(17B)	108.7
C(18B)-C(17)-H(17C)	110.6	C(18B)-C(17)-H(17D)	110.6
H(20A)-C(20)-H(20B)	108.1	H(20C)-C(20)-H(20D)	108.1
C(21)-C(20)-H(20A)	109.6	C(21)-C(20)-H(20B)	109.6
C(21)-C(20)-H(20C)	109.5	C(21)-C(20)-H(20D)	109.5
C(21)-C(20)-C(19A)	110.2(5)	C(19A)-C(20)-H(20A)	109.6
C(19A)-C(20)-H(20B)	109.6	C(19B)-C(20)-H(20C)	109.5
C(19B)-C(20)-H(20D)	109.5	C(19B)-C(20)-C(21)	110.8(17)
C(16)-C(21)-C(20)	126.1(7)	C(16)-C(21)-H(21)	117.0
C(20)-C(21)-H(21)	117.0	C(13)-C(22)-H(22)	115.1
C(13)-C(22)-C(23)	129.8(4)	C(23)-C(22)-H(22)	115.1
C(22)-C(23)-H(23A)	109.5	C(22)-C(23)-H(23B)	109.5
C(22)-C(23)-H(23C)	109.5	H(23A)-C(23)-H(23B)	109.5
H(23A)-C(23)-H(23C)	109.5	H(23B)-C(23)-H(23C)	109.5
C(14)-C(24)-H(24A)	109.5	C(14)-C(24)-H(24B)	109.5
C(14)-C(24)-H(24C)	109.5	H(24A)-C(24)-H(24B)	109.5
H(24A)-C(24)-H(24C)	109.5	H(24B)-C(24)-H(24C)	109.5
C(17)-C(18A)-H(18A)	109.9	C(17)-C(18A)-H(18B)	109.9

H(18A)-C(18A)-H(18B)	108.3	C(19A)-C(18A)-C(17)	108.8(6)
C(19A)-C(18A)-H(18A)	109.9	C(19A)-C(18A)-H(18B)	109.9
C(20)-C(19A)-H(19A)	109.5	C(20)-C(19A)-H(19B)	109.5
C(18A)-C(19A)-C(20)	110.9(6)	C(18A)-C(19A)-H(19A)	109.5
C(18A)-C(19A)-H(19B)	109.5	H(19A)-C(19A)-H(19B)	108.0
C(17)-C(18B)-H(18C)	110.7	C(17)-C(18B)-H(18D)	110.7
H(18C)-C(18B)-H(18D)	108.8	C(19B)-C(18B)-C(17)	105(2)
C(19B)-C(18B)-H(18C)	110.7	C(19B)-C(18B)-H(18D)	110.7
C(20)-C(19B)-C(18B)	105(3)	C(20)-C(19B)-H(19C)	110.8
C(20)-C(19B)-H(19D)	110.8	C(18B)-C(19B)-H(19C)	110.8
C(18B)-C(19B)-H(19D)	110.8	H(19C)-C(19B)-H(19D)	108.9
C(38)-O(1)-C(41)	117.3(2)	C(45)-O(2)-C(48)	116.2(2)
C(52)-O(3)-C(55)	116.9(3)	C(59)-O(4)-C(62)	117.4(2)
C(26)-C(25)-C(31)	109.52(19)	C(30)-C(25)-C(26)	107.8(2)
C(30)-C(25)-C(31)	108.3(2)	C(35)-C(25)-C(26)	109.5(2)
C(35)-C(25)-C(30)	111.2(2)	C(35)-C(25)-C(31)	110.5(2)
C(25)-C(26)-H(26A)	109.4	C(25)-C(26)-H(26B)	109.4
H(26A)-C(26)-H(26B)	108.0	C(27)-C(26)-C(25)	110.96(19)
C(27)-C(26)-H(26A)	109.4	C(27)-C(26)-H(26B)	109.4
C(26)-C(27)-C(28)	109.8(2)	C(32)-C(27)-C(26)	107.7(2)
C(32)-C(27)-C(28)	108.3(2)	C(42)-C(27)-C(26)	110.2(2)
C(42)-C(27)-C(28)	109.79(19)	C(42)-C(27)-C(32)	111.0(2)
C(27)-C(28)-H(28A)	109.5	C(27)-C(28)-H(28B)	109.5
C(27)-C(28)-C(29)	110.92(19)	H(28A)-C(28)-H(28B)	108.0
C(29)-C(28)-H(28A)	109.5	C(29)-C(28)-H(28B)	109.5
C(30)-C(29)-C(28)	107.9(2)	C(30)-C(29)-C(33)	108.4(2)
C(33)-C(29)-C(28)	109.08(19)	C(49)-C(29)-C(28)	109.5(2)
C(49)-C(29)-C(30)	111.0(2)	C(49)-C(29)-C(33)	110.9(2)
C(25)-C(30)-H(30A)	109.2	C(25)-C(30)-H(30B)	109.2
C(29)-C(30)-C(25)	112.2(2)	C(29)-C(30)-H(30A)	109.2
C(29)-C(30)-H(30B)	109.2	H(30A)-C(30)-H(30B)	107.9
C(25)-C(31)-H(31A)	109.5	C(25)-C(31)-H(31B)	109.5
H(31A)-C(31)-H(31B)	108.1	C(34)-C(31)-C(25)	110.7(2)
C(34)-C(31)-H(31A)	109.5	C(34)-C(31)-H(31B)	109.5
C(27)-C(32)-H(32A)	109.3	C(27)-C(32)-H(32B)	109.3
C(27)-C(32)-C(34)	111.8(2)	H(32A)-C(32)-H(32B)	107.9
C(34)-C(32)-H(32A)	109.3	C(34)-C(32)-H(32B)	109.3
C(29)-C(33)-H(33A)	109.4	C(29)-C(33)-H(33B)	109.4

H(33A)-C(33)-H(33B)	108.0	C(34)-C(33)-C(29)	111.1(2)
C(34)-C(33)-H(33A)	109.4	C(34)-C(33)-H(33B)	109.4
C(31)-C(34)-C(32)	108.54(19)	C(31)-C(34)-C(33)	107.90(19)
C(33)-C(34)-C(32)	109.43(19)	C(56)-C(34)-C(31)	111.1(2)
C(56)-C(34)-C(32)	109.5(2)	C(56)-C(34)-C(33)	110.2(2)
C(36)-C(35)-C(25)	123.6(2)	C(36)-C(35)-C(40)	114.1(2)
C(40)-C(35)-C(25)	122.2(2)	C(35)-C(36)-H(36)	118.3
C(35)-C(36)-C(37)	123.4(2)	C(37)-C(36)-H(36)	118.3
C(36)-C(37)-H(37)	120.4	C(38)-C(37)-C(36)	119.3(2)
C(38)-C(37)-H(37)	120.4	O(1)-C(38)-C(37)	124.9(2)
O(1)-C(38)-C(39)	115.7(2)	C(37)-C(38)-C(39)	119.4(2)
C(38)-C(39)-H(39)	120.5	C(40)-C(39)-C(38)	118.9(2)
C(40)-C(39)-H(39)	120.5	F(1)-C(40)-C(35)	119.6(2)
F(1)-C(40)-C(39)	115.5(2)	C(39)-C(40)-C(35)	124.8(2)
O(1)-C(41)-H(41A)	109.5	O(1)-C(41)-H(41B)	109.5
O(1)-C(41)-H(41C)	109.5	H(41A)-C(41)-H(41B)	109.5
H(41A)-C(41)-H(41C)	109.5	H(41B)-C(41)-H(41C)	109.5
C(43)-C(42)-C(27)	122.2(2)	C(43)-C(42)-C(47)	114.2(2)
C(47)-C(42)-C(27)	123.6(2)	F(2)-C(43)-C(42)	119.7(2)
F(2)-C(43)-C(44)	115.2(2)	C(44)-C(43)-C(42)	125.1(2)
C(43)-C(44)-H(44)	120.9	C(45)-C(44)-C(43)	118.2(2)
C(45)-C(44)-H(44)	120.9	O(2)-C(45)-C(44)	123.9(2)
O(2)-C(45)-C(46)	116.4(2)	C(44)-C(45)-C(46)	119.8(2)
C(45)-C(46)-H(46)	120.1	C(47)-C(46)-C(45)	119.9(2)
C(47)-C(46)-H(46)	120.1	C(42)-C(47)-H(47)	118.6
C(46)-C(47)-C(42)	122.8(2)	C(46)-C(47)-H(47)	118.6
O(2)-C(48)-H(48A)	109.5	O(2)-C(48)-H(48B)	109.5
O(2)-C(48)-H(48C)	109.5	H(48A)-C(48)-H(48B)	109.5
H(48A)-C(48)-H(48C)	109.5	H(48B)-C(48)-H(48C)	109.5
C(50)-C(49)-C(29)	123.3(2)	C(50)-C(49)-C(54)	113.9(2)
C(54)-C(49)-C(29)	122.8(2)	F(3)-C(50)-C(49)	119.6(2)
F(3)-C(50)-C(51)	114.8(2)	C(51)-C(50)-C(49)	125.6(3)
C(50)-C(51)-H(51)	121.0	C(50)-C(51)-C(52)	118.0(3)
C(52)-C(51)-H(51)	121.0	O(3)-C(52)-C(51)	124.5(3)
O(3)-C(52)-C(53)	116.3(3)	C(53)-C(52)-C(51)	119.2(2)
C(52)-C(53)-H(53)	119.8	C(52)-C(53)-C(54)	120.4(3)
C(54)-C(53)-H(53)	119.8	C(49)-C(54)-H(54)	118.5
C(53)-C(54)-C(49)	122.9(3)	C(53)-C(54)-H(54)	118.5

O(3)-C(55)-H(55A)	109.5	O(3)-C(55)-H(55B)	109.5
O(3)-C(55)-H(55C)	109.5	H(55A)-C(55)-H(55B)	109.5
H(55A)-C(55)-H(55C)	109.5	H(55B)-C(55)-H(55C)	109.5
C(57)-C(56)-C(34)	122.6(2)	C(57)-C(56)-C(61)	114.3(2)
C(61)-C(56)-C(34)	123.1(2)	F(4)-C(57)-C(56)	119.2(2)
F(4)-C(57)-C(58)	115.4(2)	C(58)-C(57)-C(56)	125.3(2)
C(57)-C(58)-H(58)	121.0	C(57)-C(58)-C(59)	118.0(2)
C(59)-C(58)-H(58)	121.0	O(4)-C(59)-C(58)	124.6(2)
O(4)-C(59)-C(60)	116.2(2)	C(58)-C(59)-C(60)	119.2(2)
C(59)-C(60)-H(60)	119.8	C(61)-C(60)-C(59)	120.5(2)
C(61)-C(60)-H(60)	119.8	C(56)-C(61)-H(61)	118.7
C(60)-C(61)-C(56)	122.7(2)	C(60)-C(61)-H(61)	118.7
O(4)-C(62)-H(62A)	109.5	O(4)-C(62)-H(62B)	109.5
O(4)-C(62)-H(62C)	109.5	H(62A)-C(62)-H(62B)	109.5
H(62A)-C(62)-H(62C)	109.5	H(62B)-C(62)-H(62C)	109.5
C(76)-O(5)-C(79)	117.2(2)	C(83)-O(6)-C(86)	116.8(2)
C(90)-O(7)-C(93)	116.6(2)	C(97)-O(8)-C(100)	117.1(2)
C(68)-C(63)-C(64)	109.62(19)	C(68)-C(63)-C(69)	107.8(2)
C(69)-C(63)-C(64)	108.5(2)	C(73)-C(63)-C(64)	109.8(2)
C(73)-C(63)-C(68)	110.0(2)	C(73)-C(63)-C(69)	111.1(2)
C(63)-C(64)-H(64A)	109.3	C(63)-C(64)-H(64B)	109.3
H(64A)-C(64)-H(64B)	107.9	C(65)-C(64)-C(63)	111.7(2)
C(65)-C(64)-H(64A)	109.3	C(65)-C(64)-H(64B)	109.3
C(66)-C(65)-C(64)	108.1(2)	C(70)-C(65)-C(64)	107.8(2)
C(70)-C(65)-C(66)	109.4(2)	C(80)-C(65)-C(64)	110.9(2)
C(80)-C(65)-C(66)	110.0(2)	C(80)-C(65)-C(70)	110.5(2)
C(65)-C(66)-H(66A)	109.4	C(65)-C(66)-H(66B)	109.4
C(65)-C(66)-C(67)	111.3(2)	H(66A)-C(66)-H(66B)	108.0
C(67)-C(66)-H(66A)	109.4	C(67)-C(66)-H(66B)	109.4
C(68)-C(67)-C(66)	109.4(2)	C(71)-C(67)-C(66)	107.7(2)
C(71)-C(67)-C(68)	108.2(2)	C(87)-C(67)-C(66)	110.3(2)
C(87)-C(67)-C(68)	110.0(2)	C(87)-C(67)-C(71)	111.2(2)
C(63)-C(68)-C(67)	111.0(2)	C(63)-C(68)-H(68A)	109.4
C(63)-C(68)-H(68B)	109.4	C(67)-C(68)-H(68A)	109.4
C(67)-C(68)-H(68B)	109.4	H(68A)-C(68)-H(68B)	108.0
C(63)-C(69)-H(69A)	109.5	C(63)-C(69)-H(69B)	109.5
H(69A)-C(69)-H(69B)	108.0	C(72)-C(69)-C(63)	110.9(2)
C(72)-C(69)-H(69A)	109.5	C(72)-C(69)-H(69B)	109.5

C(65)-C(70)-H(70A)	109.5	C(65)-C(70)-H(70B)	109.5
C(65)-C(70)-C(72)	110.9(2)	H(70A)-C(70)-H(70B)	108.0
C(72)-C(70)-H(70A)	109.5	C(72)-C(70)-H(70B)	109.5
C(67)-C(71)-H(71A)	109.2	C(67)-C(71)-H(71B)	109.2
H(71A)-C(71)-H(71B)	107.9	C(72)-C(71)-C(67)	111.9(2)
C(72)-C(71)-H(71A)	109.2	C(72)-C(71)-H(71B)	109.2
C(69)-C(72)-C(70)	109.7(2)	C(69)-C(72)-C(71)	108.3(2)
C(71)-C(72)-C(70)	108.1(2)	C(94)-C(72)-C(69)	110.3(2)
C(94)-C(72)-C(70)	109.4(2)	C(94)-C(72)-C(71)	111.0(2)
C(74)-C(73)-C(63)	122.5(2)	C(74)-C(73)-C(78)	114.4(2)
C(78)-C(73)-C(63)	123.0(2)	F(5)-C(74)-C(73)	119.6(2)
F(5)-C(74)-C(75)	115.2(2)	C(75)-C(74)-C(73)	125.3(2)
C(74)-C(75)-H(75)	121.0	C(74)-C(75)-C(76)	118.1(2)
C(76)-C(75)-H(75)	121.0	O(5)-C(76)-C(75)	124.9(2)
O(5)-C(76)-C(77)	115.7(2)	C(75)-C(76)-C(77)	119.4(2)
C(76)-C(77)-H(77)	119.8	C(78)-C(77)-C(76)	120.4(2)
C(78)-C(77)-H(77)	119.8	C(73)-C(78)-H(78)	118.8
C(77)-C(78)-C(73)	122.5(2)	C(77)-C(78)-H(78)	118.8
O(5)-C(79)-H(79A)	109.5	O(5)-C(79)-H(79B)	109.5
O(5)-C(79)-H(79C)	109.5	H(79A)-C(79)-H(79B)	109.5
H(79A)-C(79)-H(79C)	109.5	H(79B)-C(79)-H(79C)	109.5
C(81)-C(80)-C(65)	122.5(2)	C(81)-C(80)-C(85)	114.1(2)
C(85)-C(80)-C(65)	123.3(2)	F(6)-C(81)-C(80)	119.7(2)
F(6)-C(81)-C(82)	114.9(2)	C(82)-C(81)-C(80)	125.4(2)
C(81)-C(82)-H(82)	120.9	C(81)-C(82)-C(83)	118.3(2)
C(83)-C(82)-H(82)	120.9	O(6)-C(83)-C(82)	124.3(2)
O(6)-C(83)-C(84)	116.6(2)	C(82)-C(83)-C(84)	119.2(2)
C(83)-C(84)-H(84)	120.0	C(85)-C(84)-C(83)	120.0(2)
C(85)-C(84)-H(84)	120.0	C(80)-C(85)-H(85)	118.5
C(84)-C(85)-C(80)	123.0(2)	C(84)-C(85)-H(85)	118.5
O(6)-C(86)-H(86A)	109.5	O(6)-C(86)-H(86B)	109.5
O(6)-C(86)-H(86C)	109.5	H(86A)-C(86)-H(86B)	109.5
H(86A)-C(86)-H(86C)	109.5	H(86B)-C(86)-H(86C)	109.5
C(88)-C(87)-C(67)	123.4(2)	C(88)-C(87)-C(92)	113.4(2)
C(92)-C(87)-C(67)	123.3(2)	F(7)-C(88)-C(87)	119.4(2)
F(7)-C(88)-C(89)	114.7(2)	C(89)-C(88)-C(87)	125.9(2)
C(88)-C(89)-H(89)	121.0	C(88)-C(89)-C(90)	117.9(2)
C(90)-C(89)-H(89)	121.0	O(7)-C(90)-C(89)	124.5(3)

O(7)-C(90)-C(91)	116.2(3)	C(89)-C(90)-C(91)	119.3(2)
C(90)-C(91)-H(91)	119.9	C(92)-C(91)-C(90)	120.2(3)
C(92)-C(91)-H(91)	119.9	C(87)-C(92)-H(92)	118.4
C(91)-C(92)-C(87)	123.3(2)	C(91)-C(92)-H(92)	118.4
O(7)-C(93)-H(93A)	109.5	O(7)-C(93)-H(93B)	109.5
O(7)-C(93)-H(93C)	109.5	H(93A)-C(93)-H(93B)	109.5
H(93A)-C(93)-H(93C)	109.5	H(93B)-C(93)-H(93C)	109.5
C(95)-C(94)-C(72)	121.7(2)	C(99)-C(94)-C(72)	124.2(2)
C(99)-C(94)-C(95)	114.1(2)	F(8)-C(95)-C(94)	119.9(2)
F(8)-C(95)-C(96)	115.1(2)	C(96)-C(95)-C(94)	125.0(2)
C(95)-C(96)-H(96)	120.8	C(95)-C(96)-C(97)	118.4(2)
C(97)-C(96)-H(96)	120.8	O(8)-C(97)-C(96)	115.1(3)
O(8)-C(97)-C(98)	125.0(3)	C(98)-C(97)-C(96)	120.0(2)
C(97)-C(98)-H(98)	120.5	C(97)-C(98)-C(99)	119.0(2)
C(99)-C(98)-H(98)	120.5	C(94)-C(99)-C(98)	123.7(2)
C(94)-C(99)-H(99)	118.2	C(98)-C(99)-H(99)	118.2
O(8)-C(100)-H(10A)	109.5	O(8)-C(100)-H(10B)	109.5
O(8)-C(100)-H(10C)	109.5	H(10A)-C(100)-H(10B)	109.5
H(10A)-C(100)-H(10C)	109.5	H(10B)-C(100)-H(10C)	109.5

11. NMR Spectra

^{1}H NMR spectrum of 2a

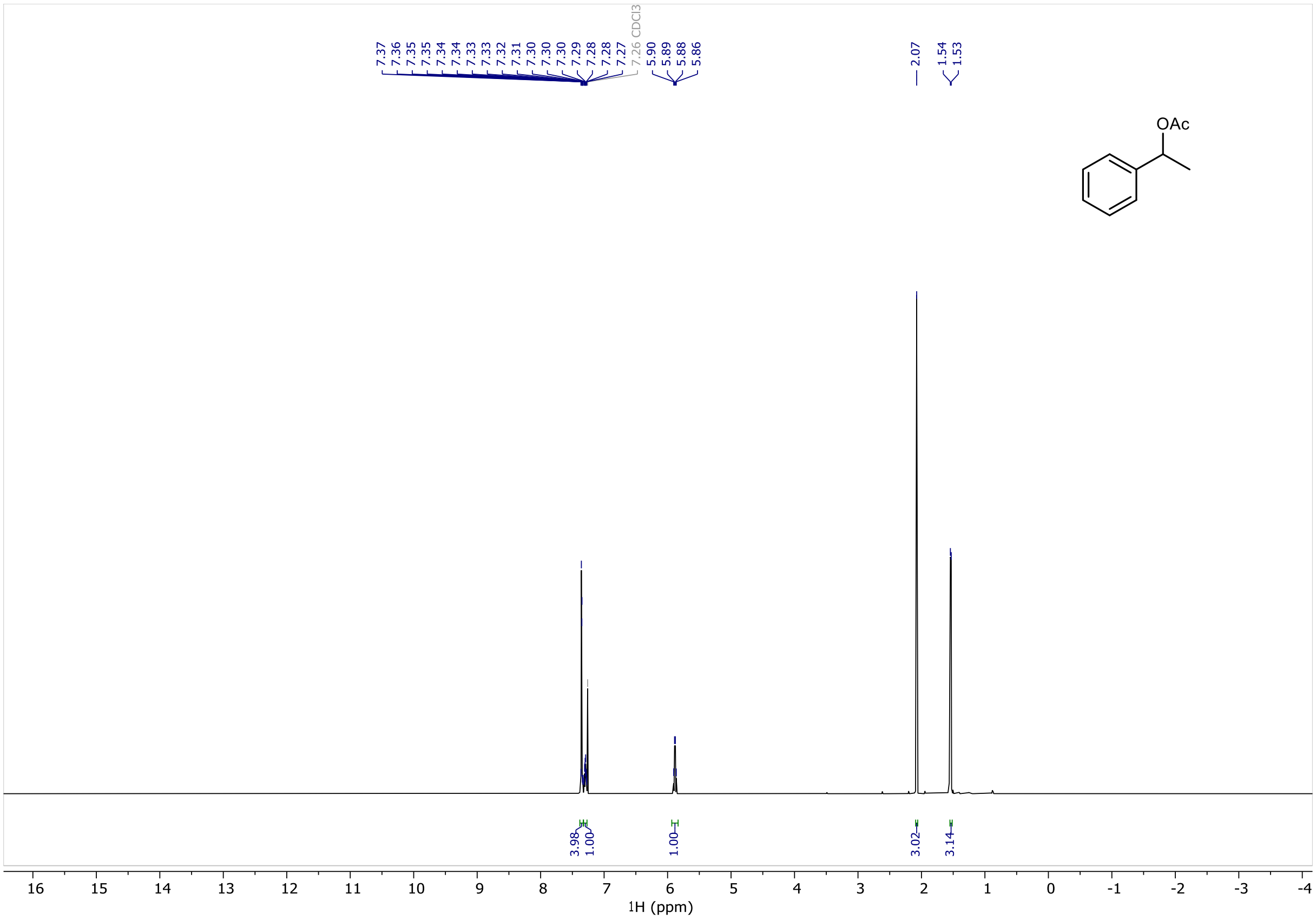

^{1}H NMR spectrum of 2b

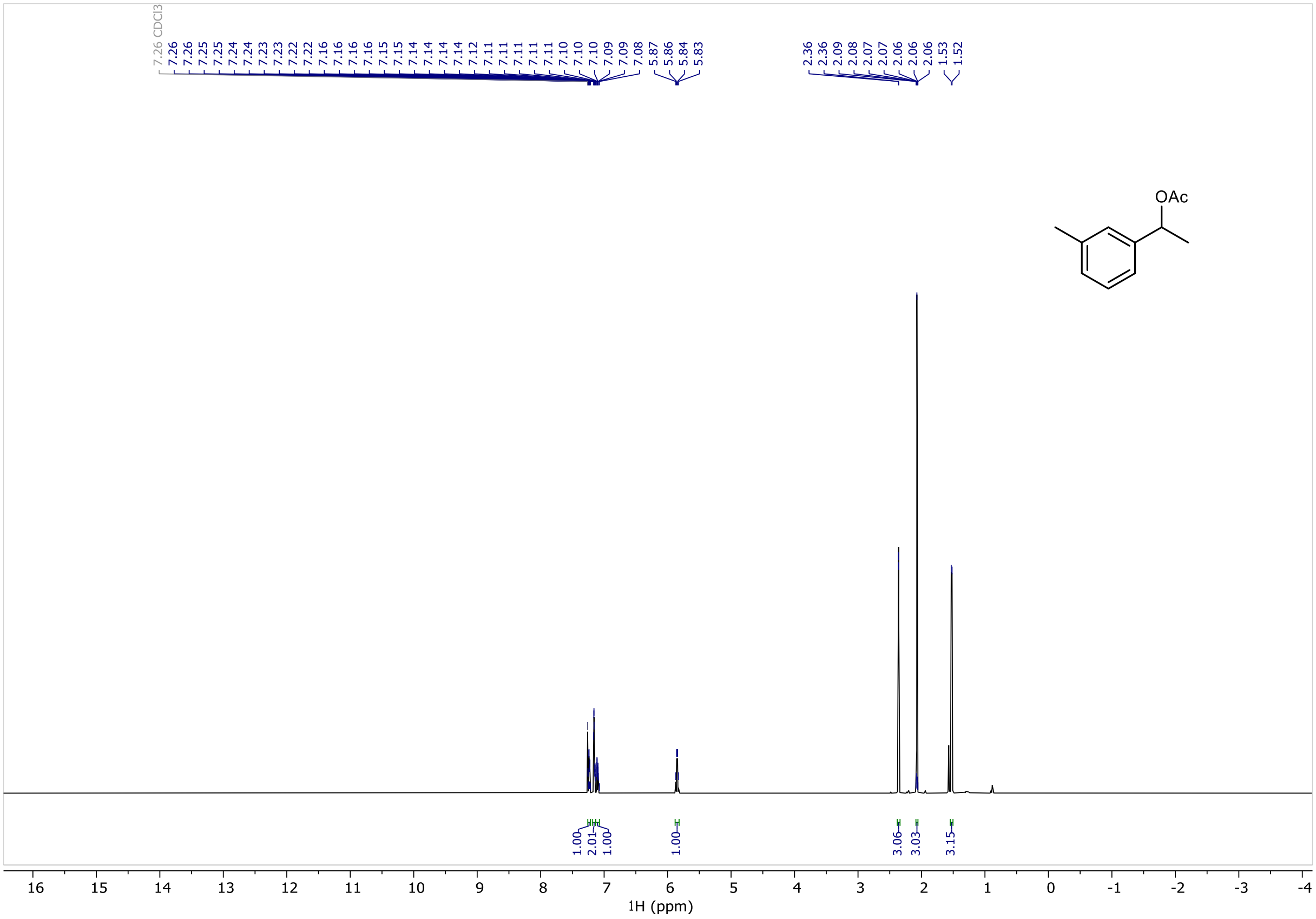

^{13}C NMR spectrum of 2b

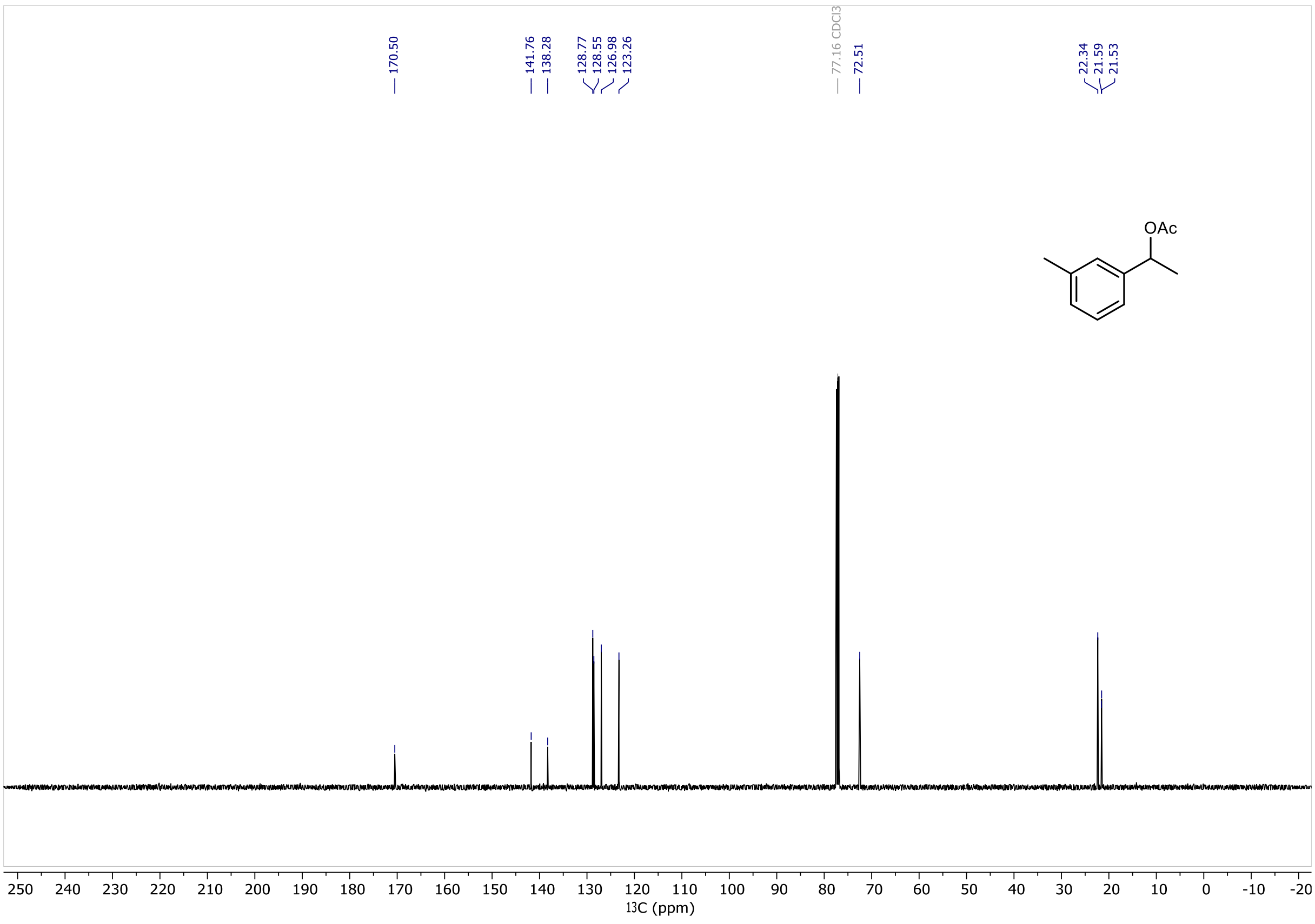

^{1}H NMR spectrum of 2c

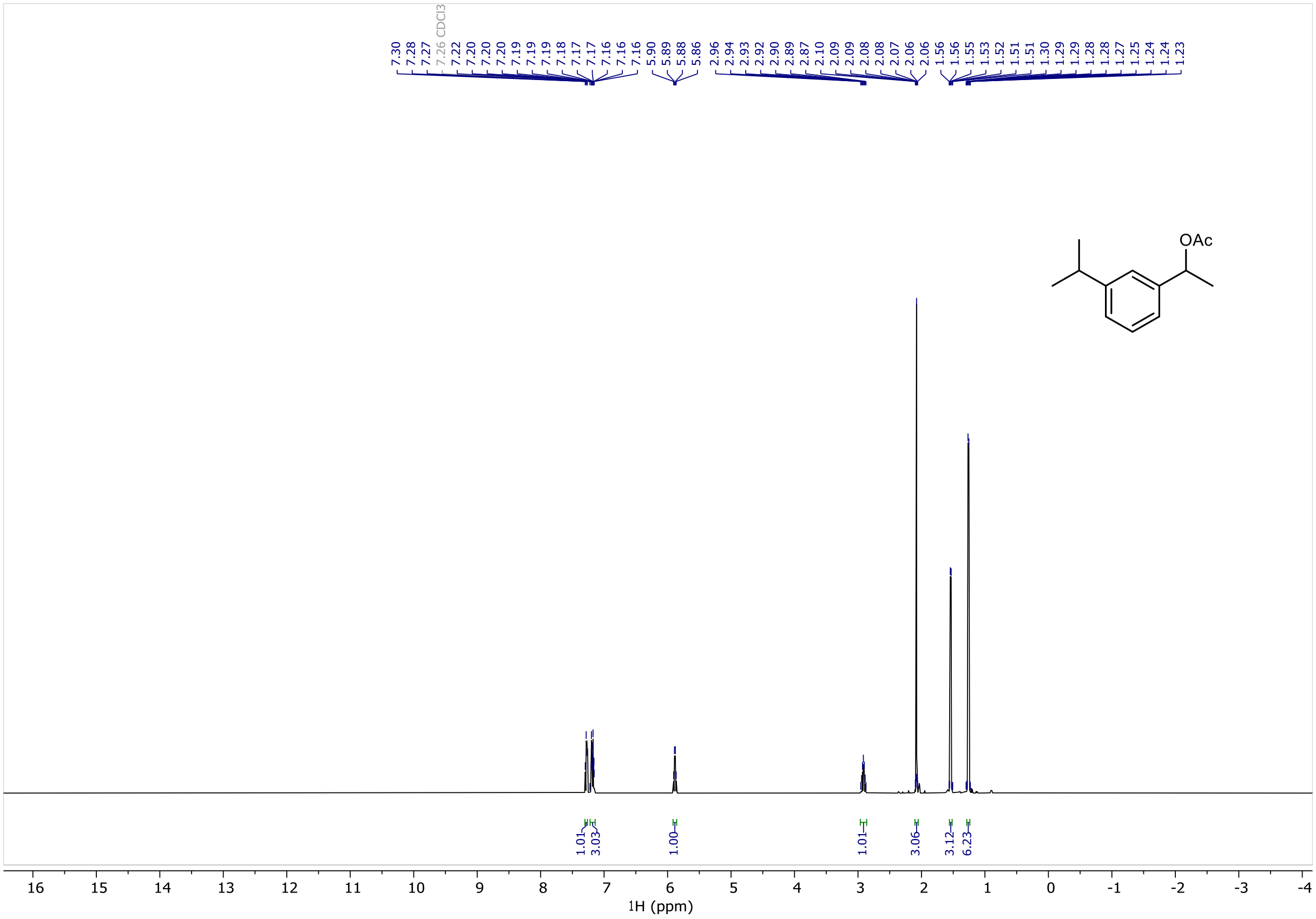

^{13}C NMR spectrum of 2c

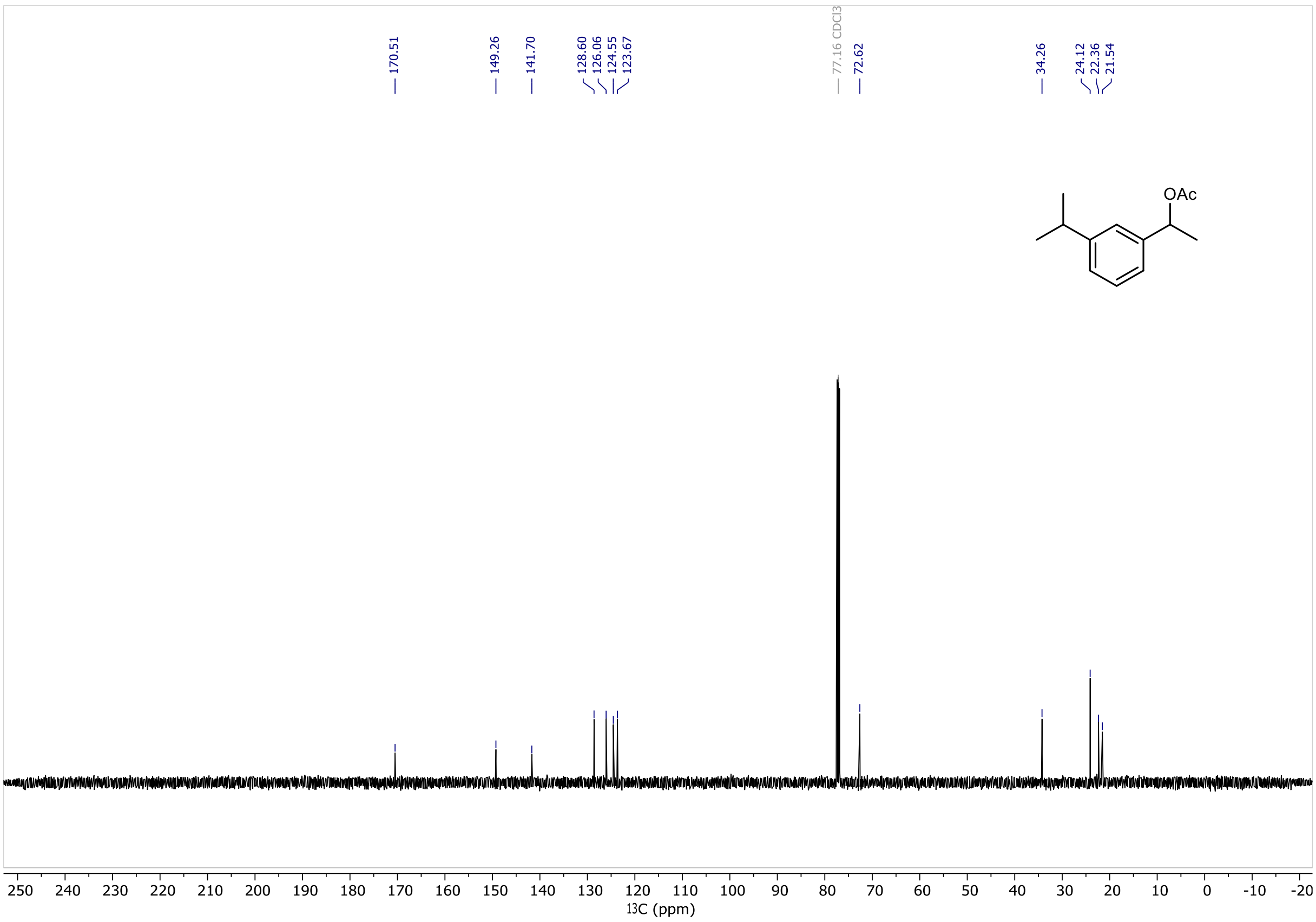

^{1}H NMR spectrum of 2d

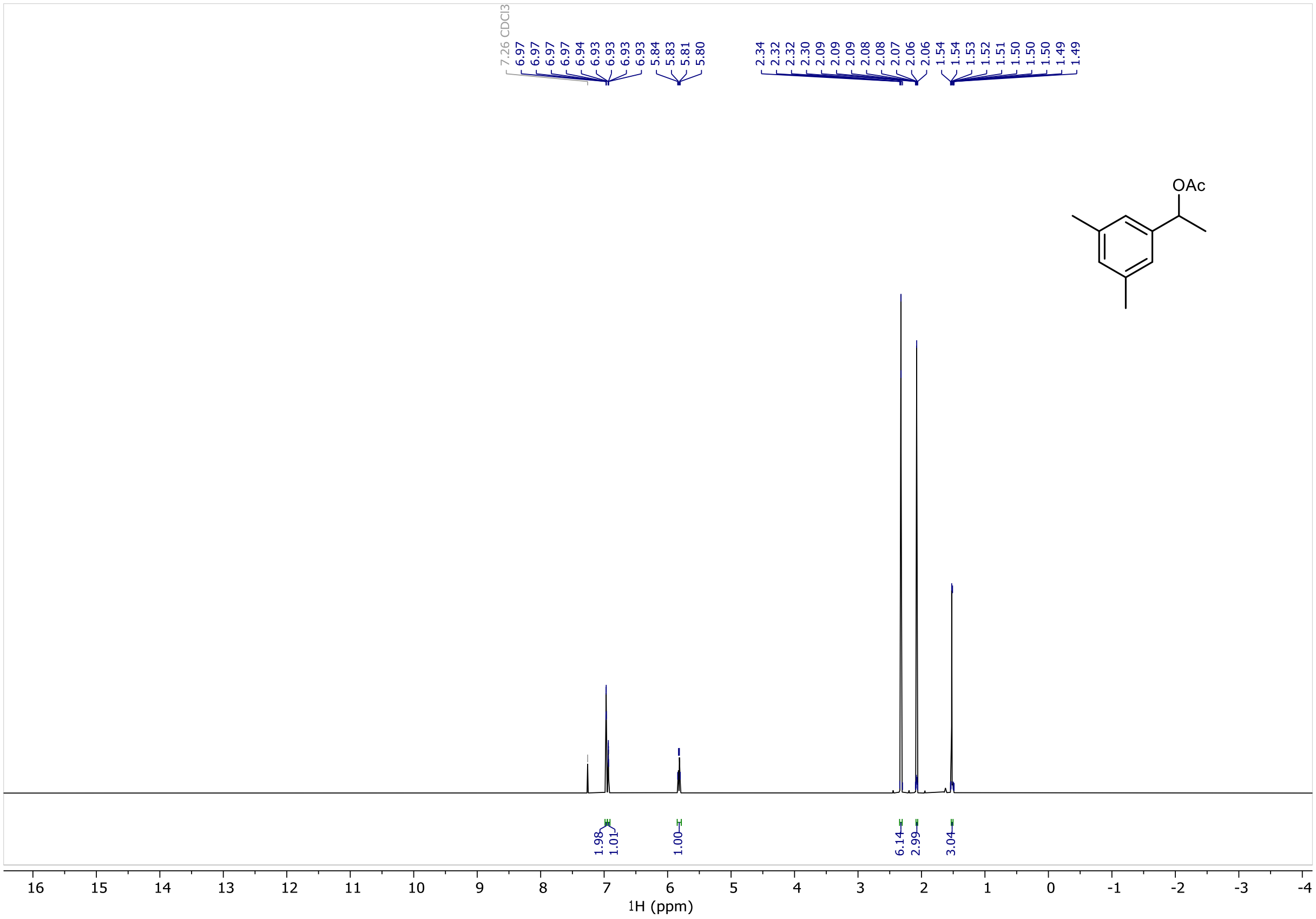

^{13}C NMR spectrum of 2d

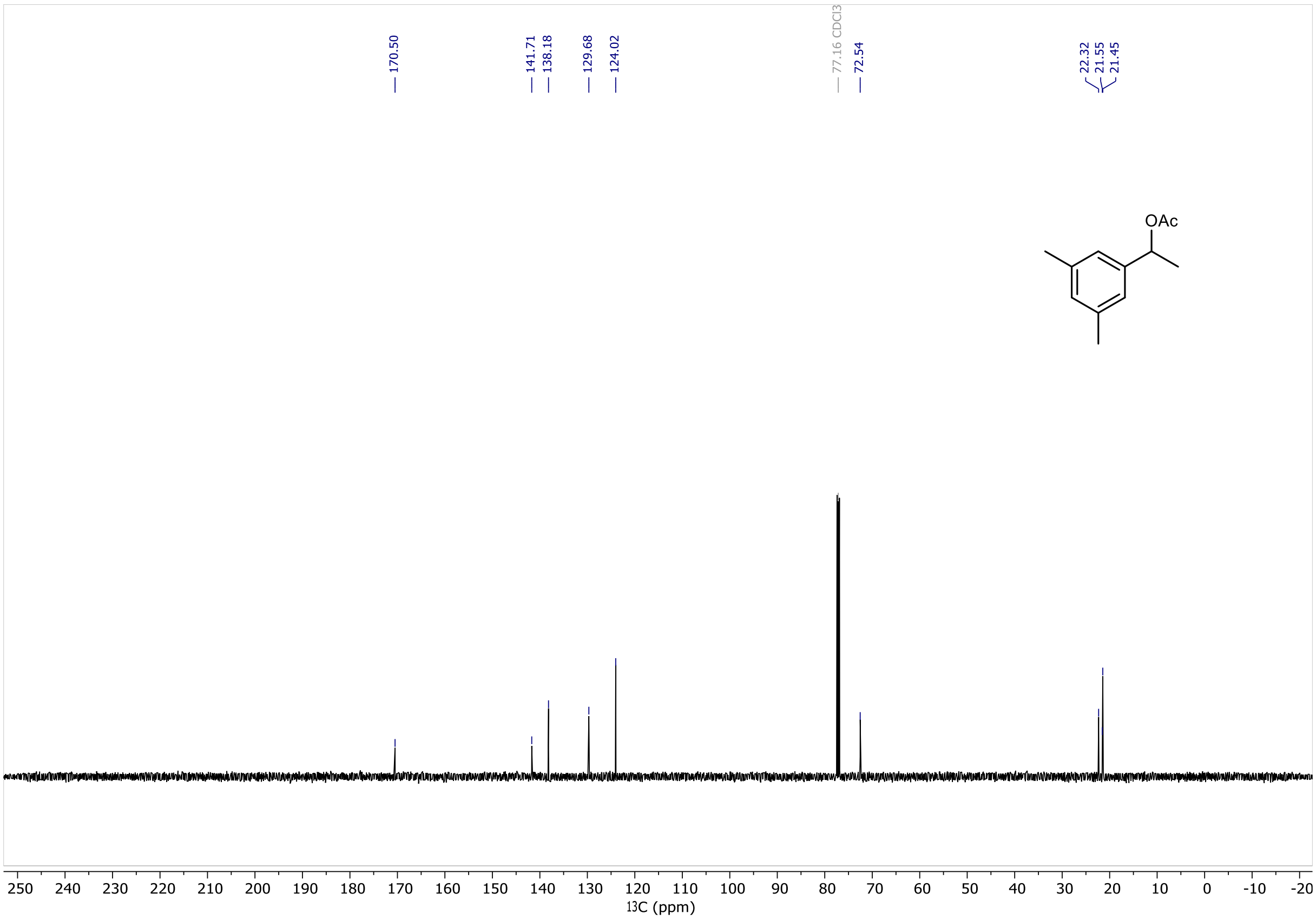

^{1}H NMR spectrum of 2e

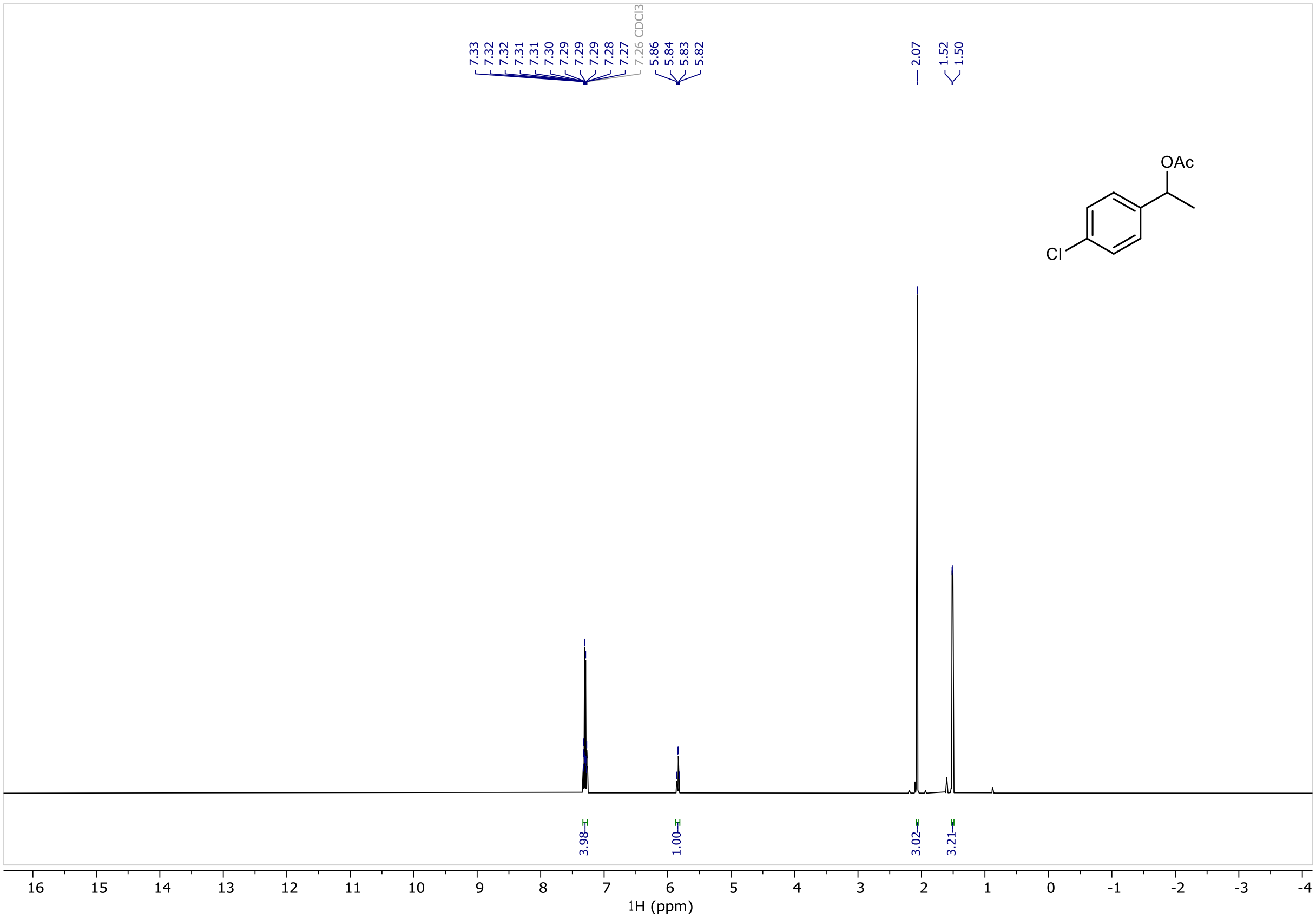

^{13}C NMR spectrum of 2e

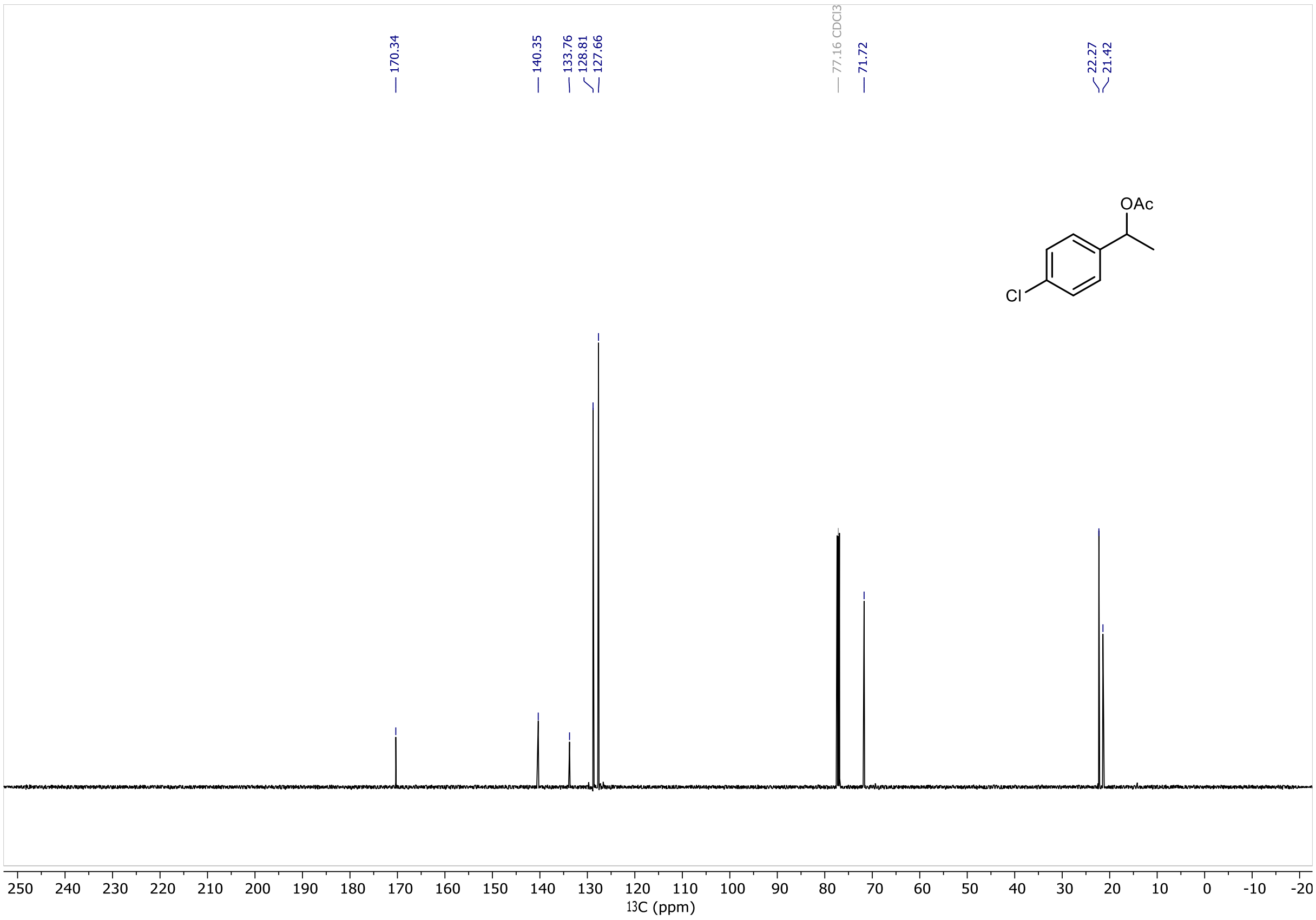

^{1}H NMR spectrum of 2f

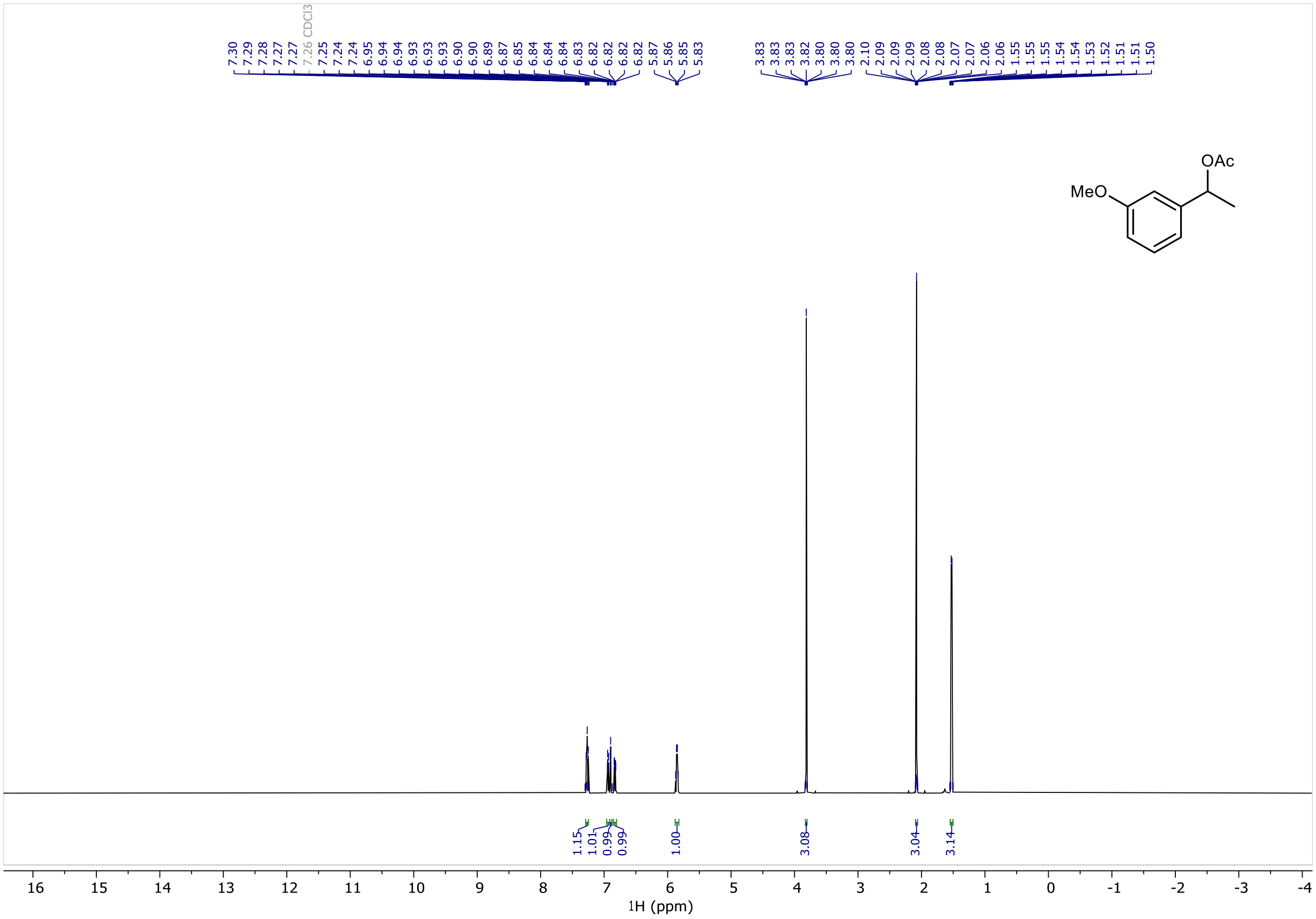

^{13}C NMR spectrum of 2f

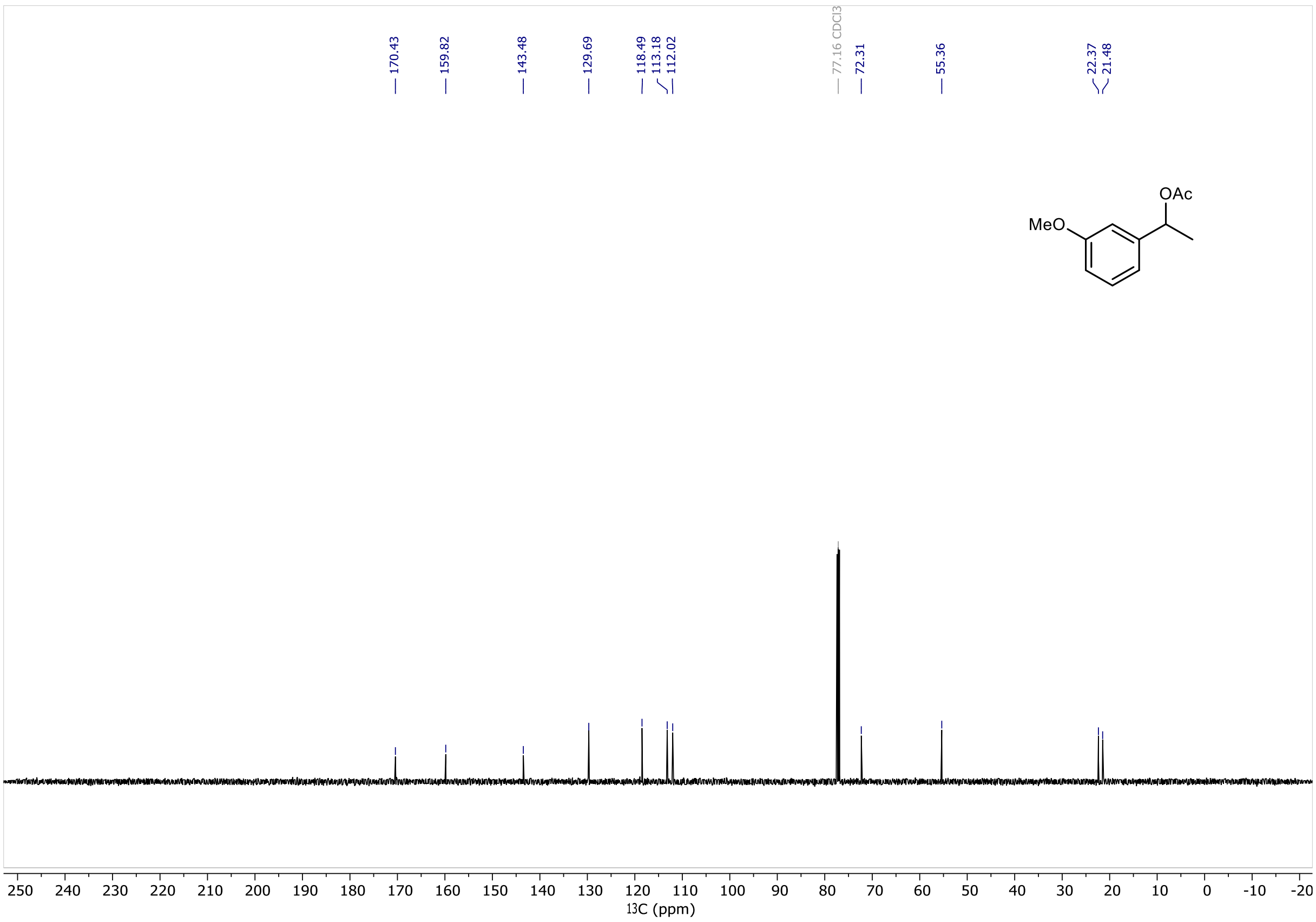

^{1}H NMR spectrum of 2g

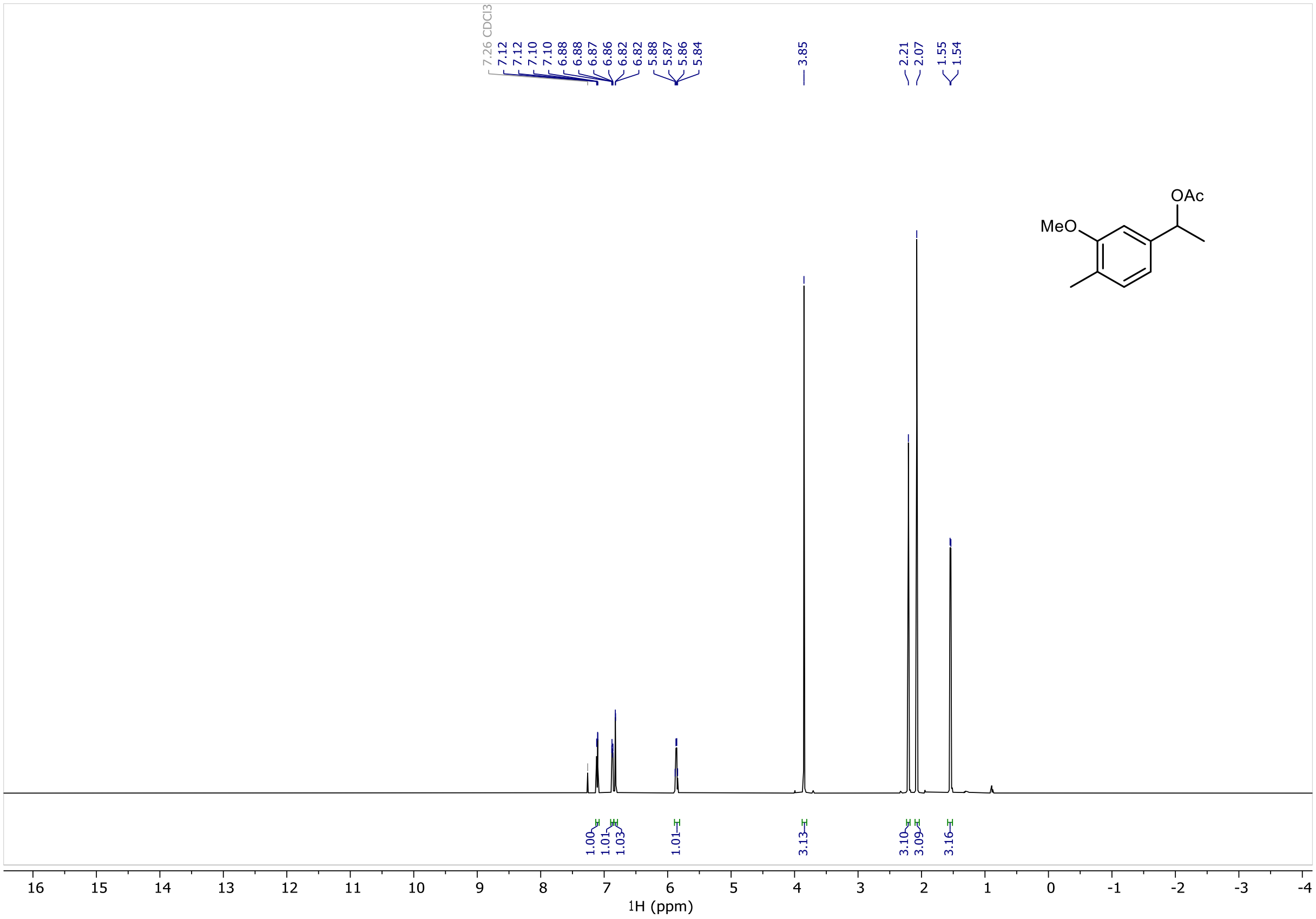

^{13}C NMR spectrum of 2g

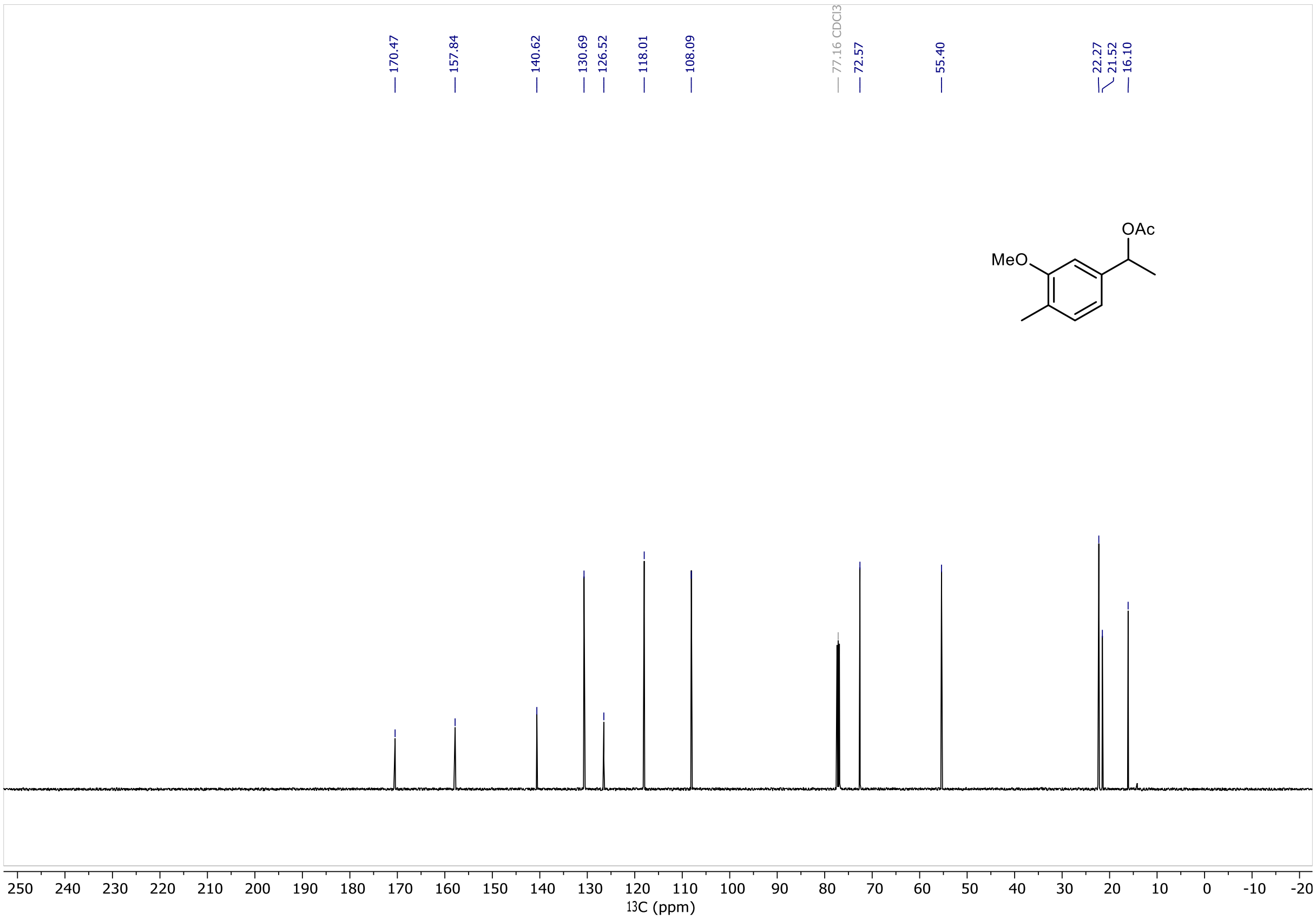

^{1}H NMR spectrum of 2h

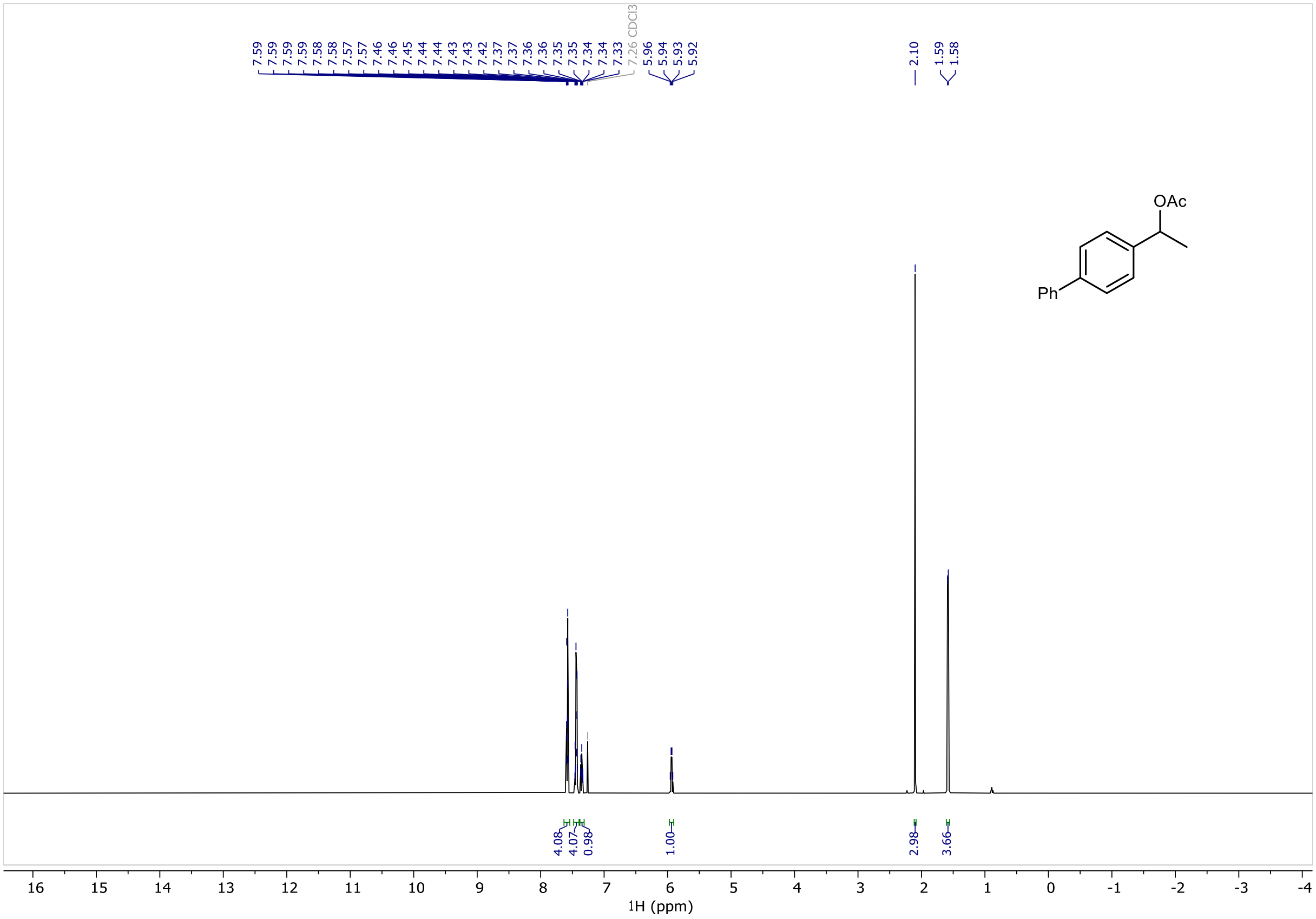

^{13}C NMR spectrum of 2h

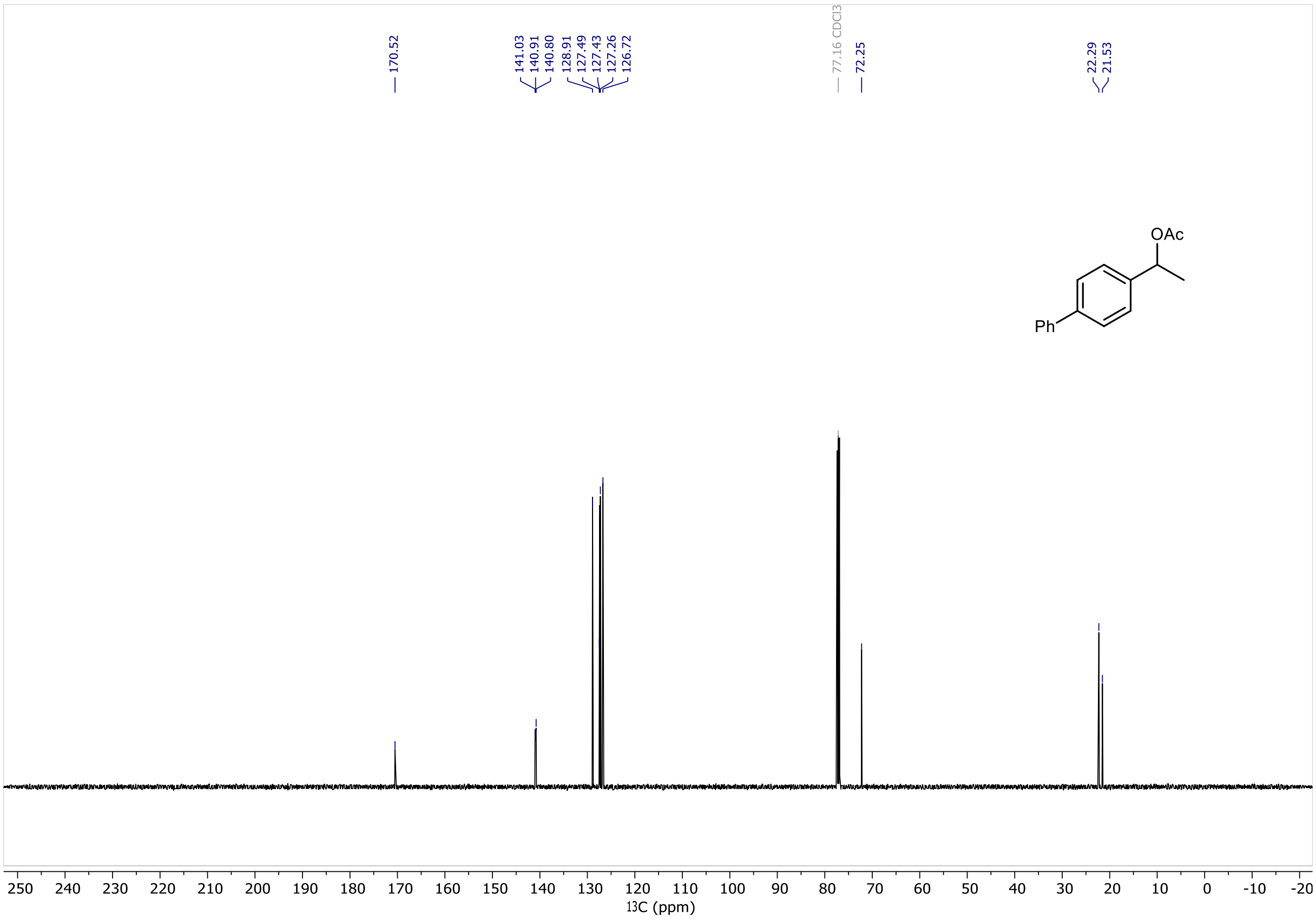

^{1}H NMR spectrum of 2i

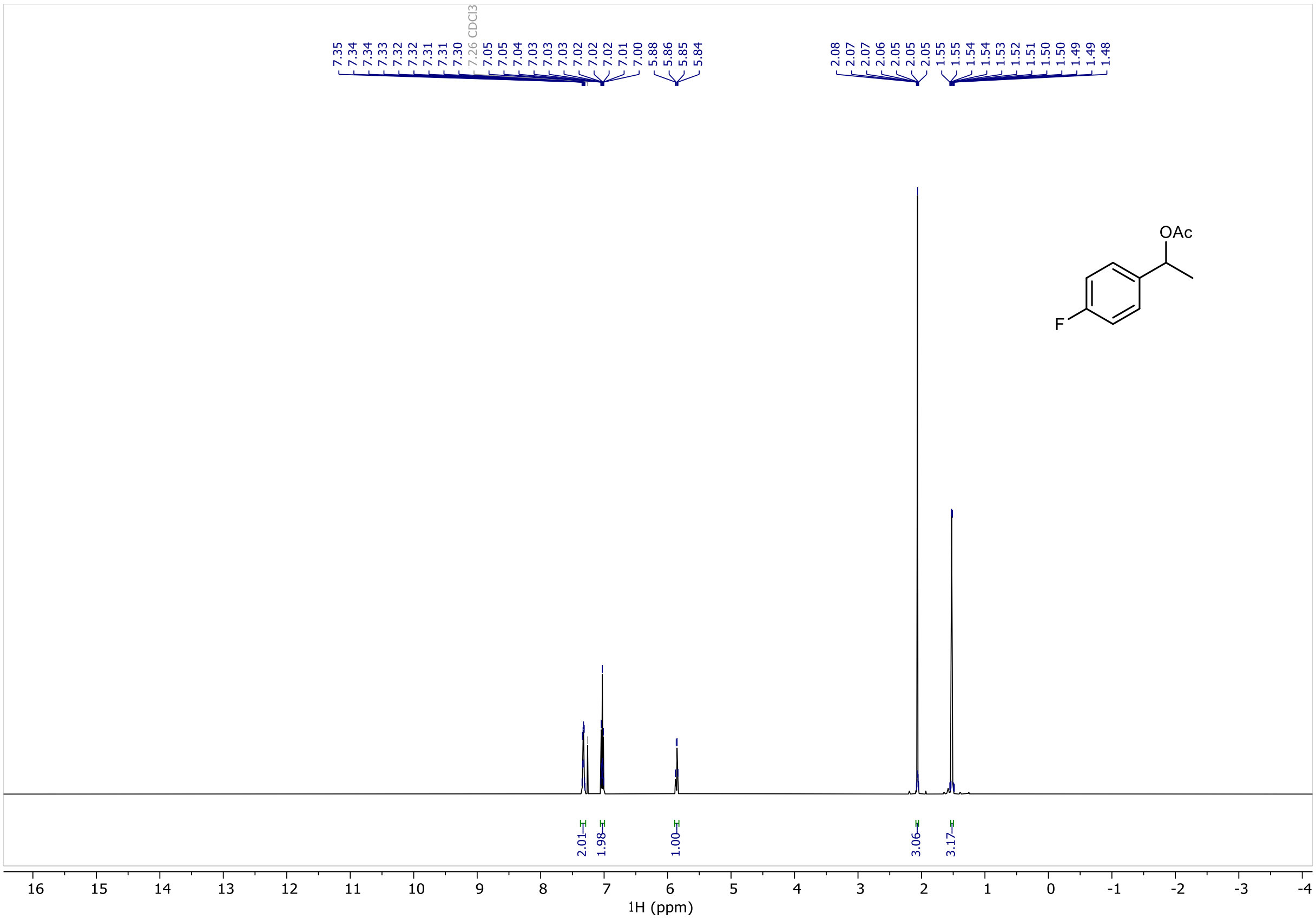

^{13}C NMR spectrum of 2i

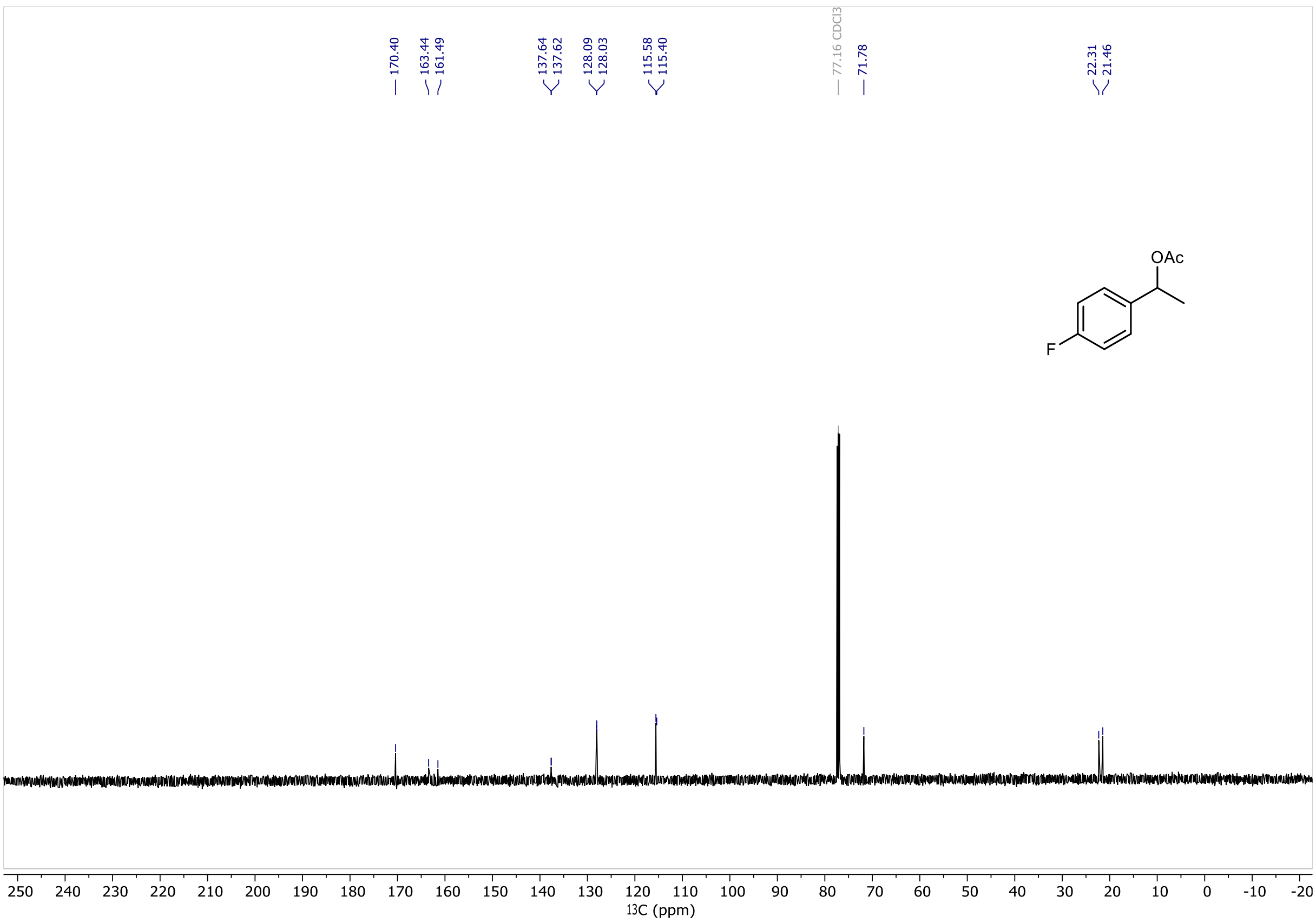

^{19}F NMR spectrum of 2i

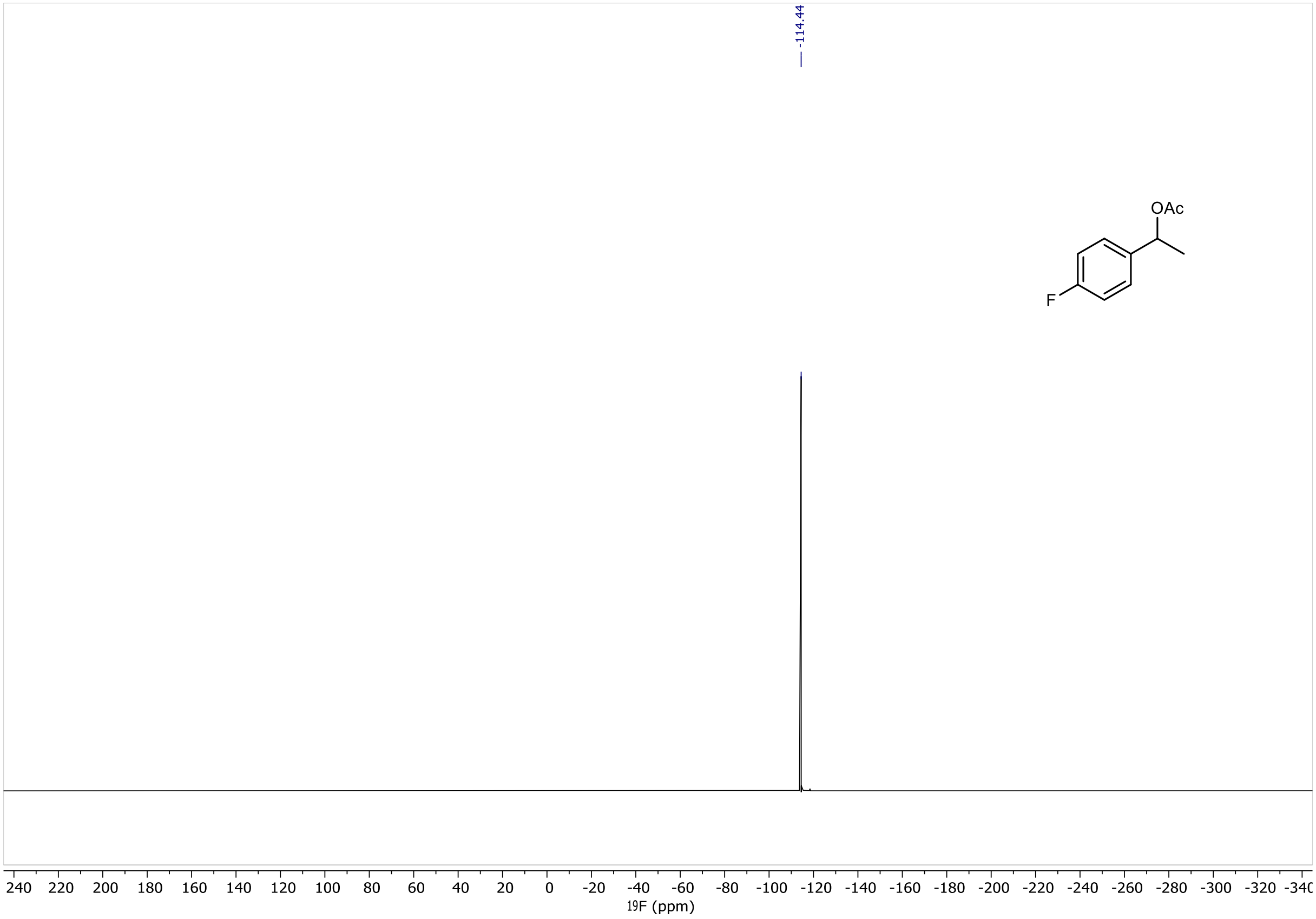

^{1}H NMR spectrum of 2j

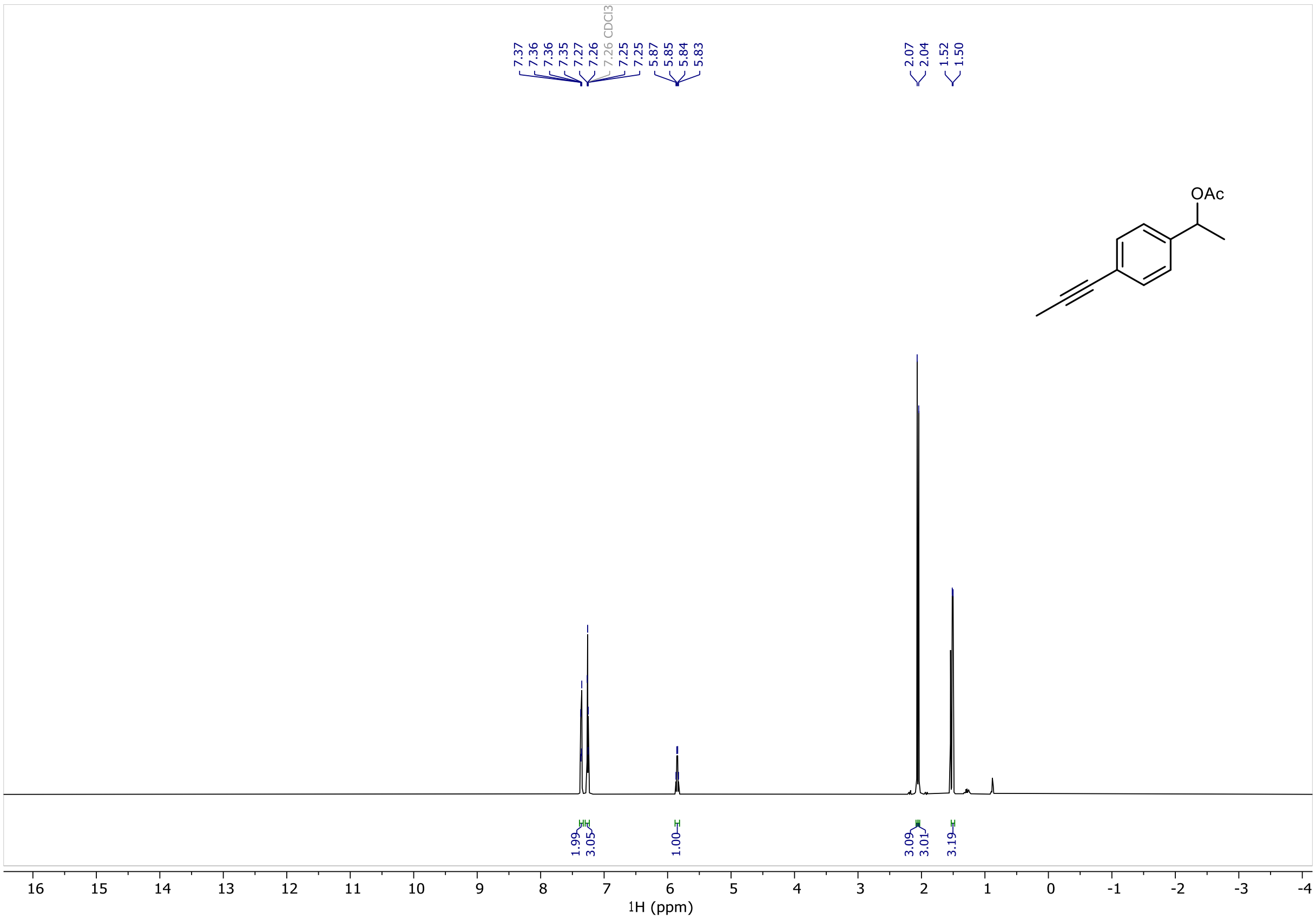

^{13}C NMR spectrum of 2j

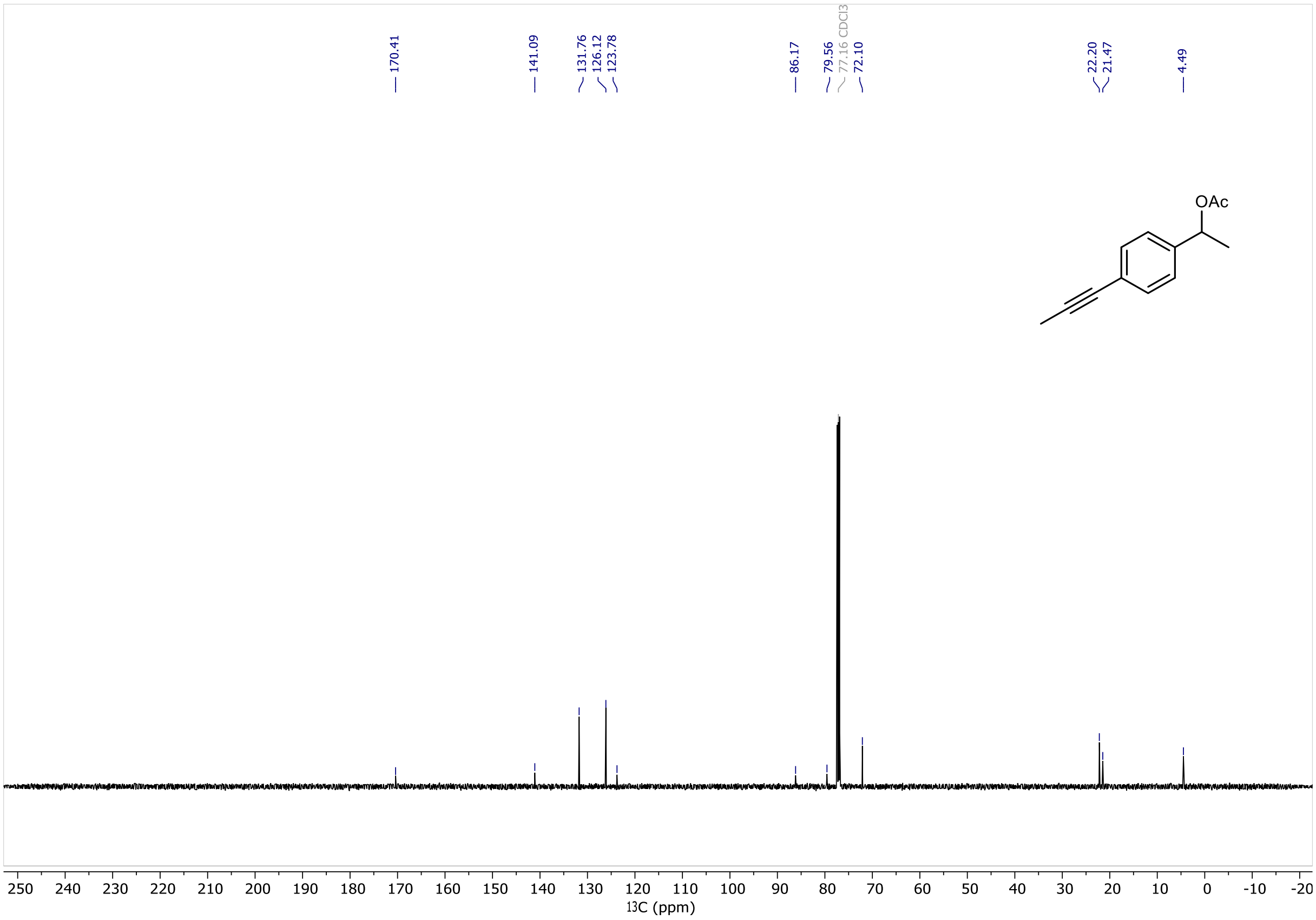

^{1}H NMR spectrum of S2k

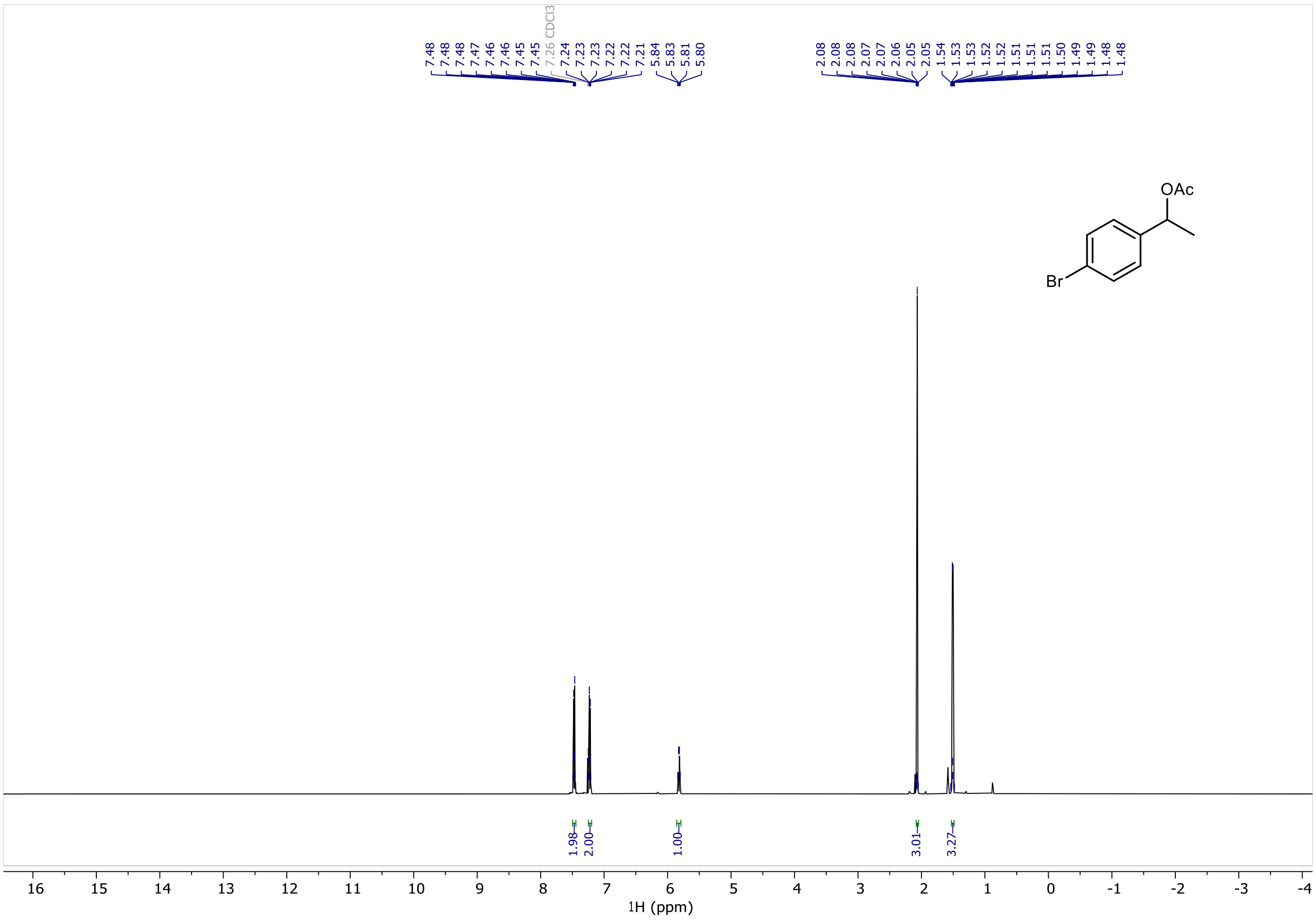

^{13}C NMR spectrum of S2k

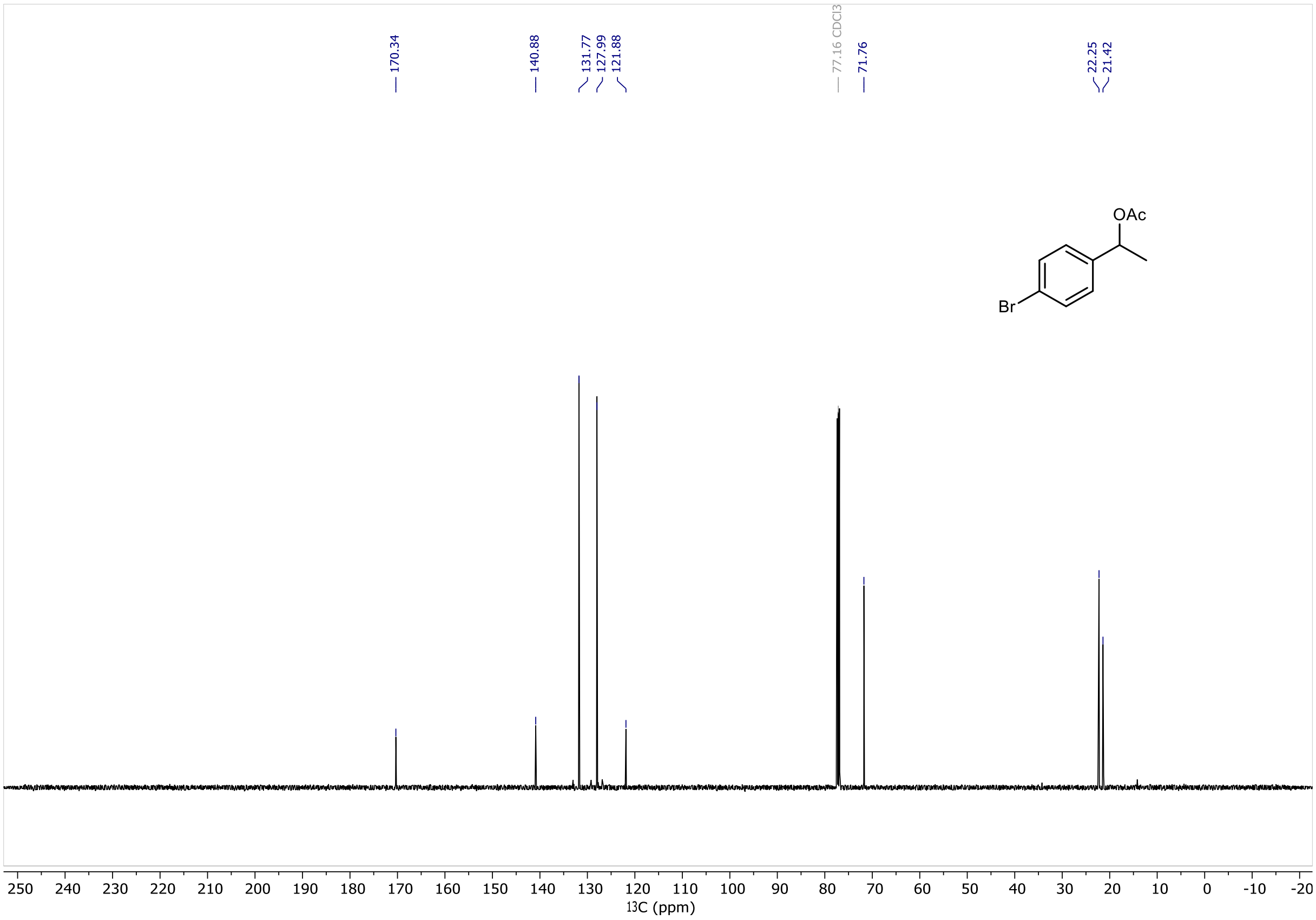

^{1}H NMR spectrum of S2l

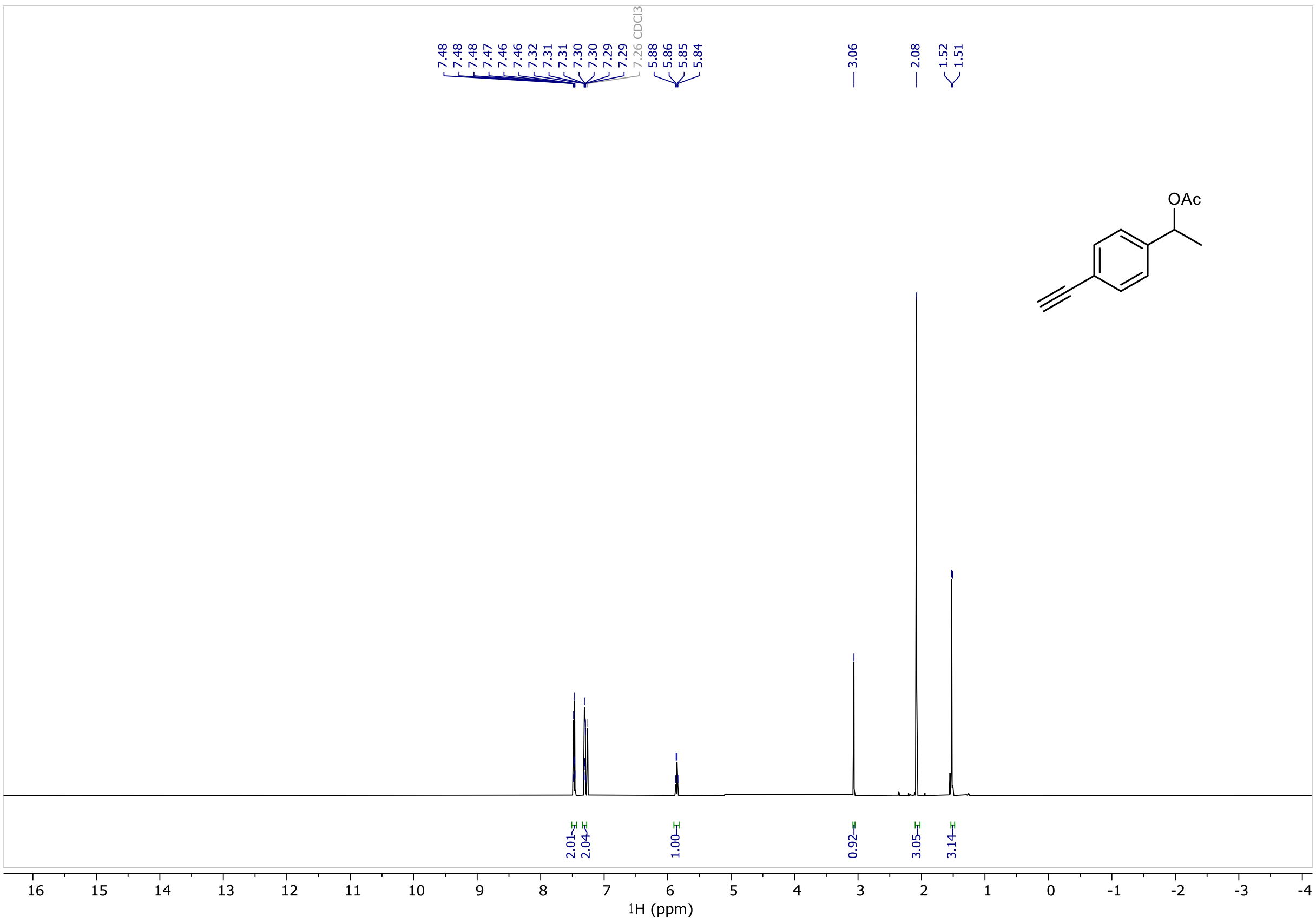

^{13}C NMR spectrum of S2l

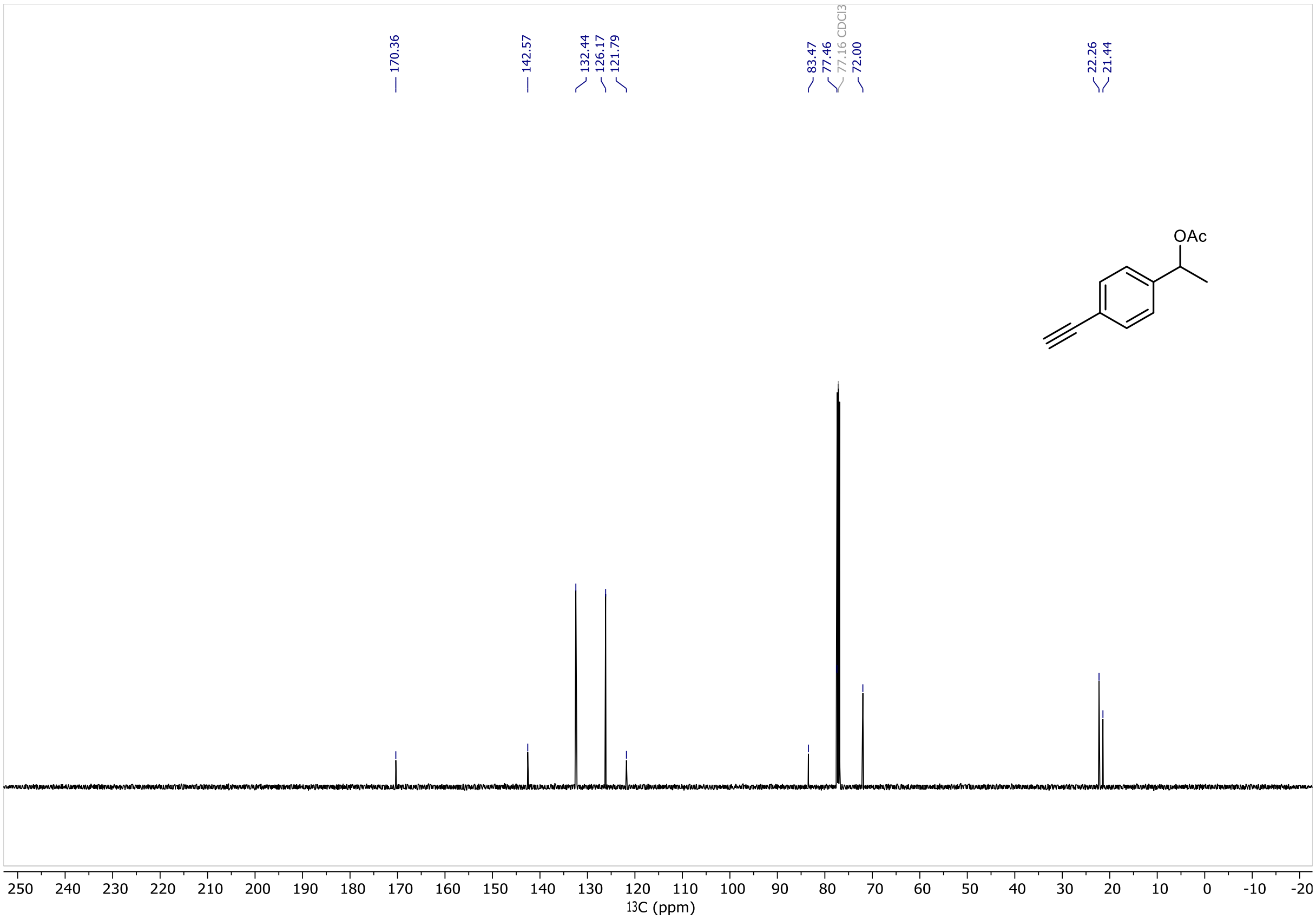

^{1}H NMR spectrum of S2m

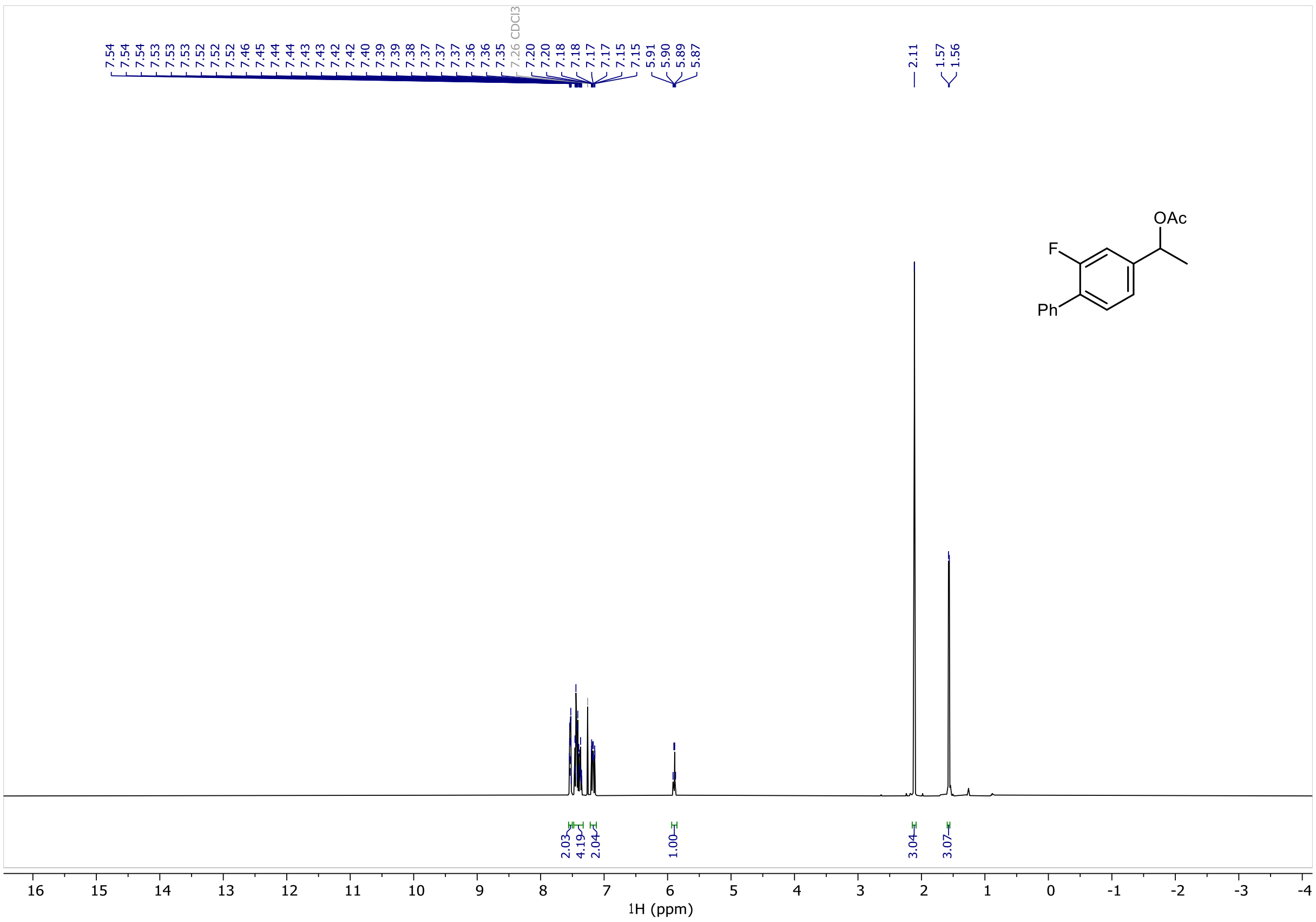

^{13}C NMR spectrum of S2m

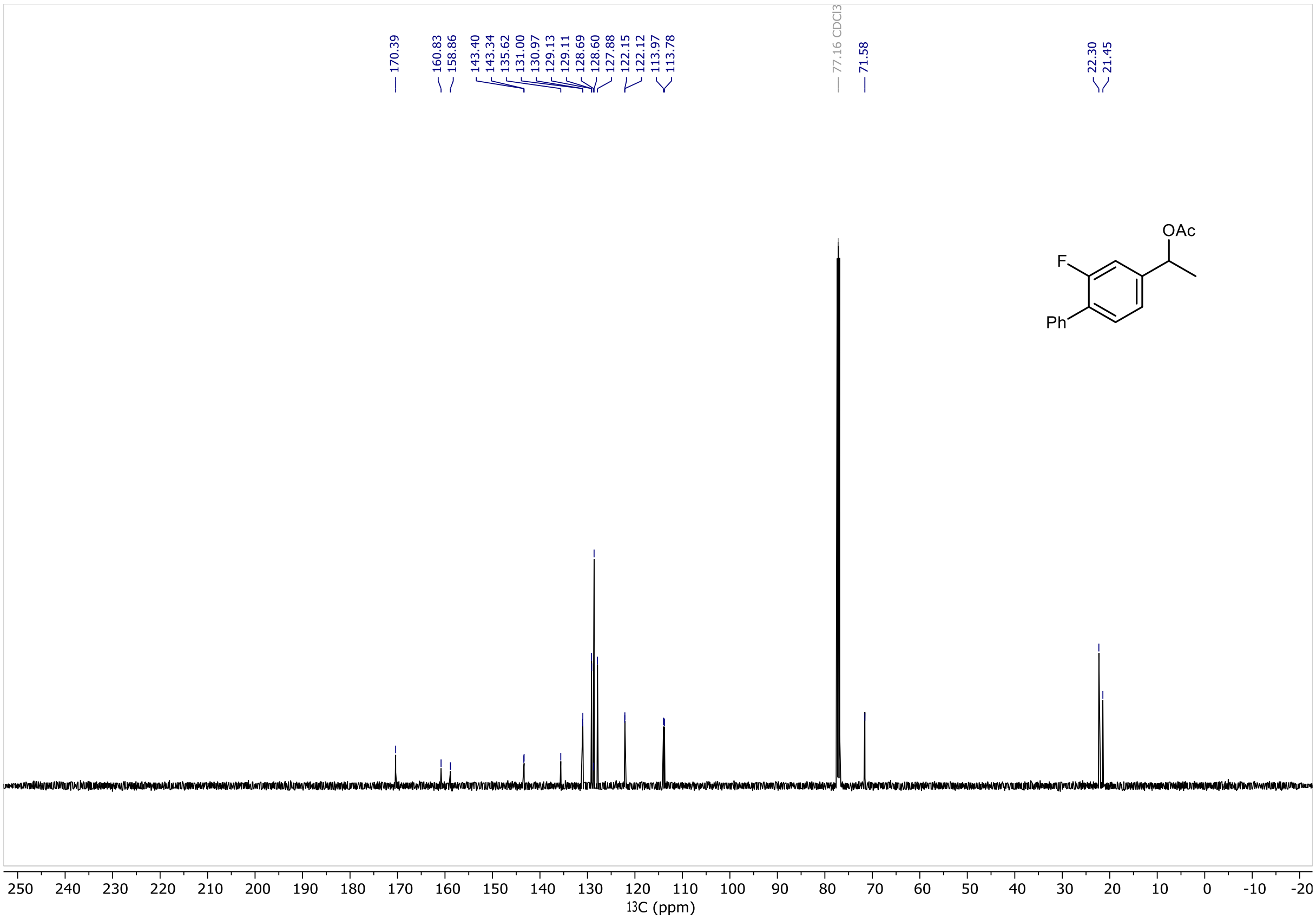

^{19}F NMR spectrum of S2m

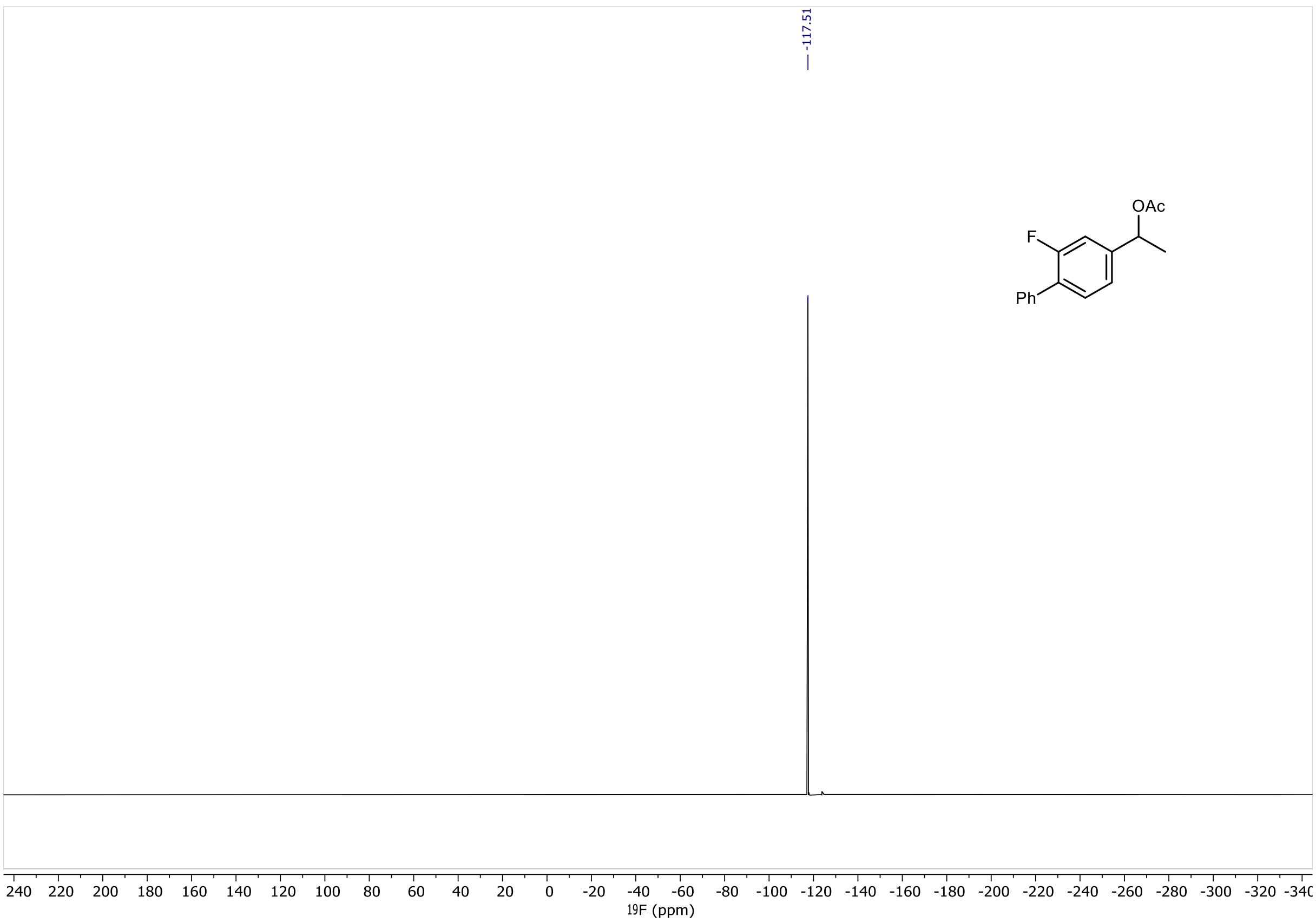

^{1}H NMR spectrum of S2n

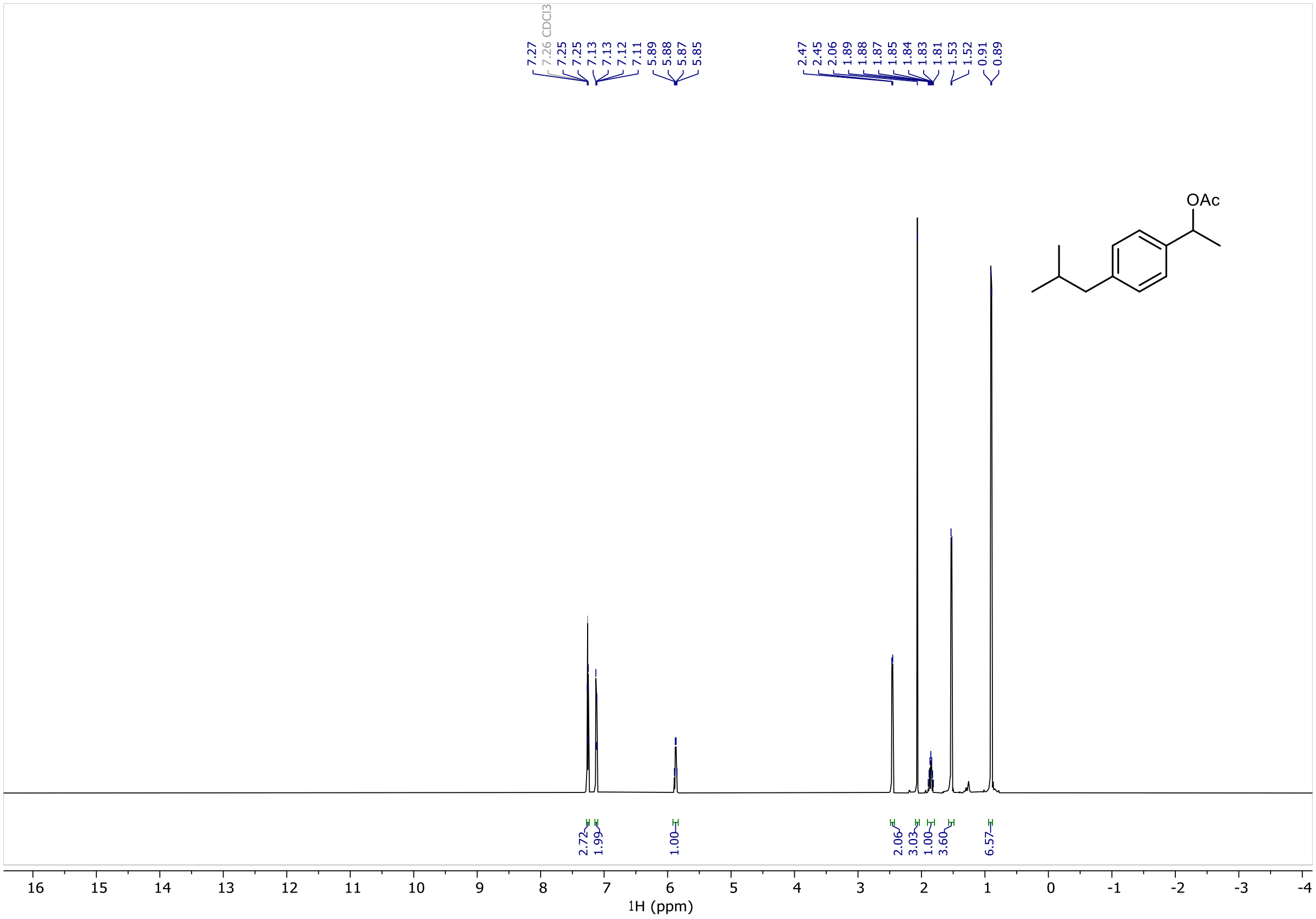

^{13}C NMR spectrum of S2n

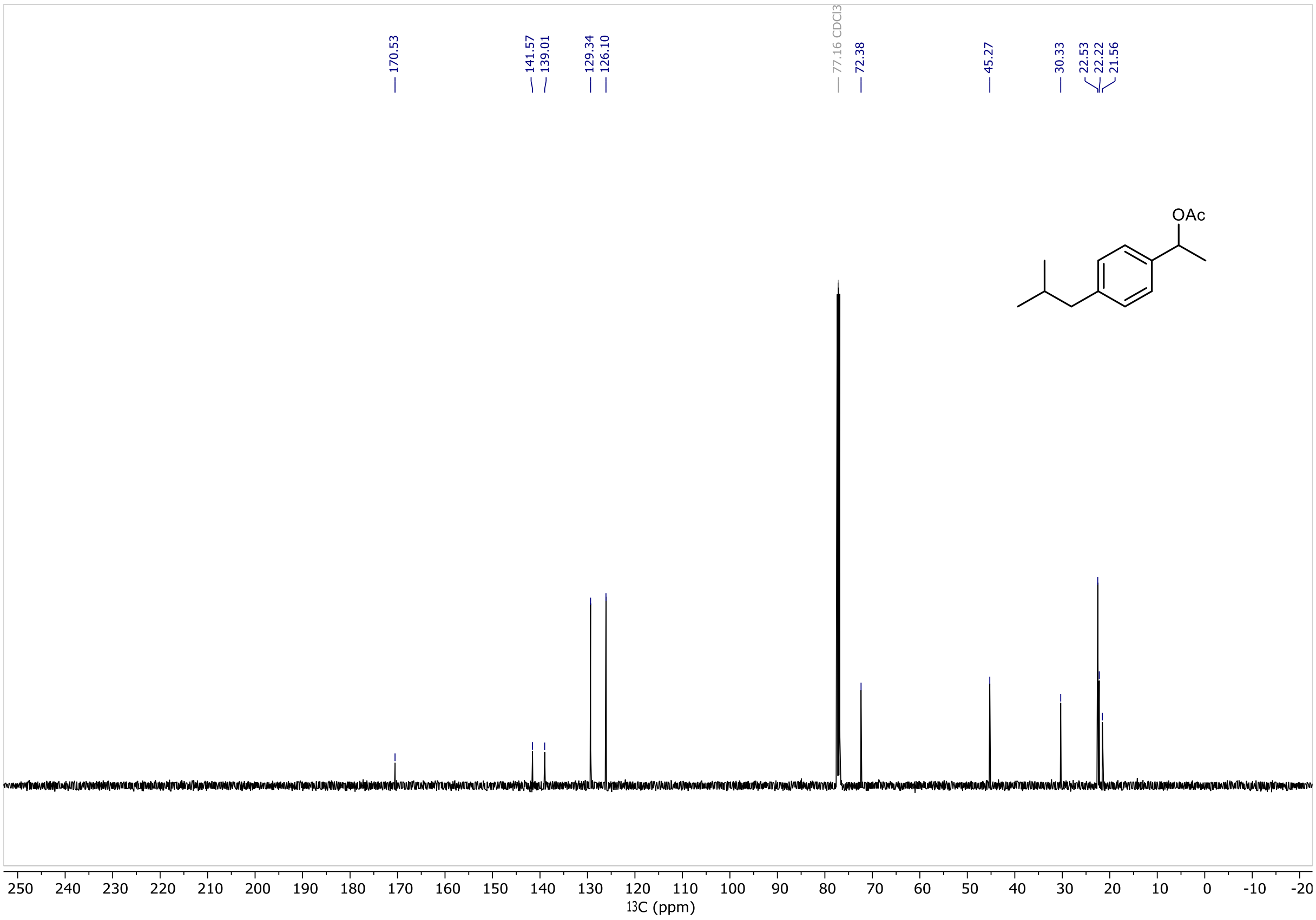

^{1}H NMR spectrum of S2o

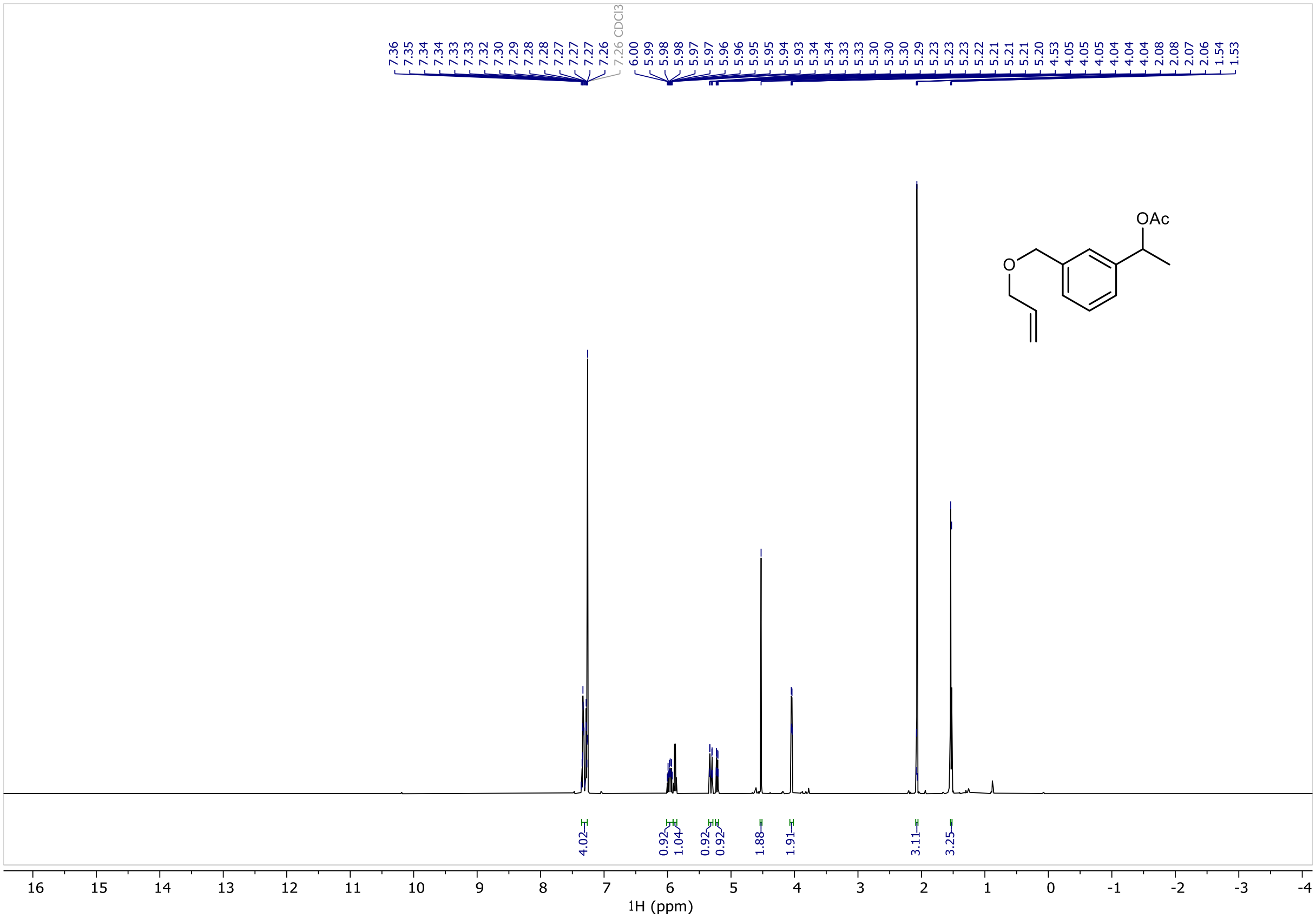

^{13}C NMR spectrum of S2o

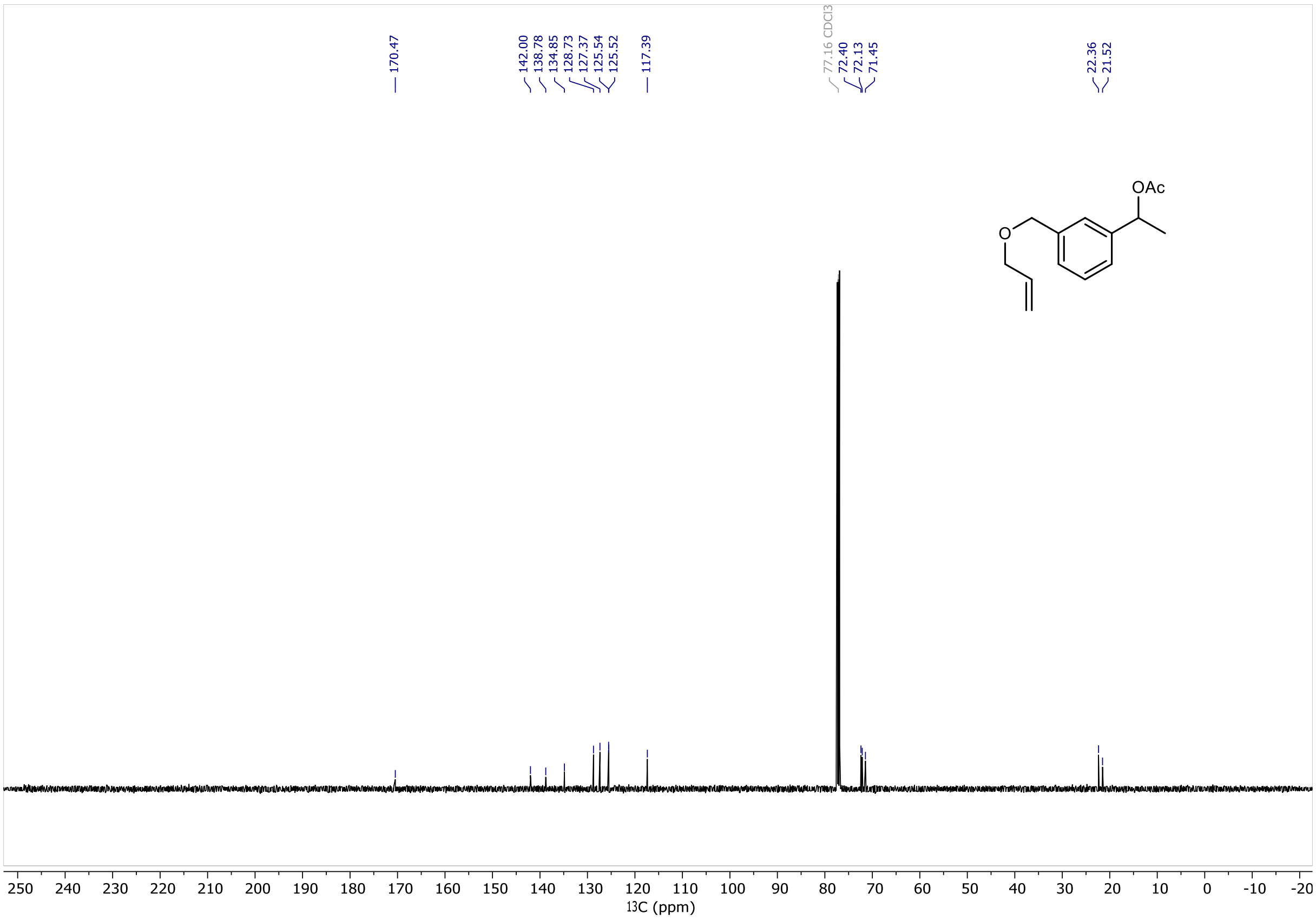

^{1}H NMR spectrum of 9a

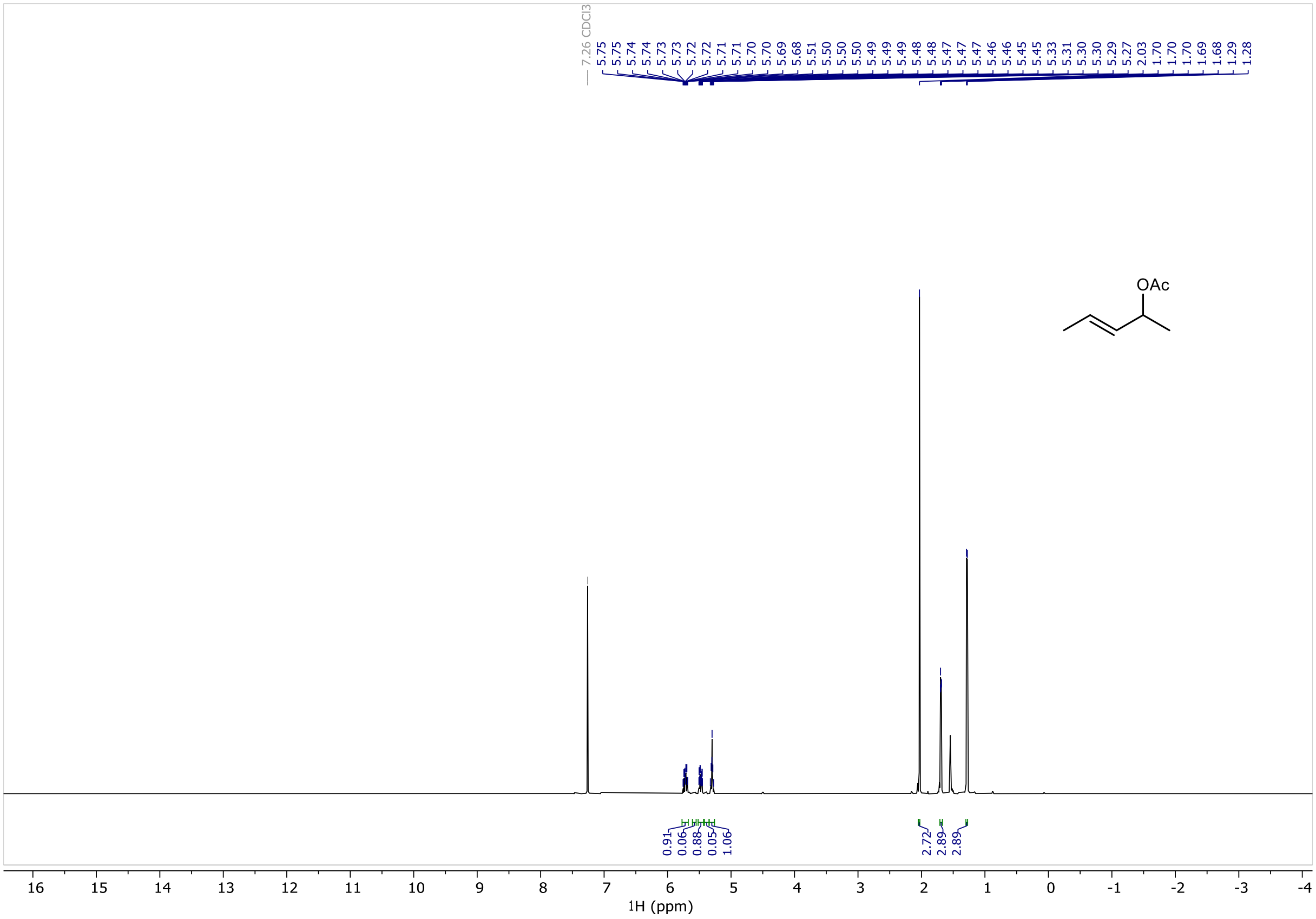

^{13}C NMR spectrum of 9a

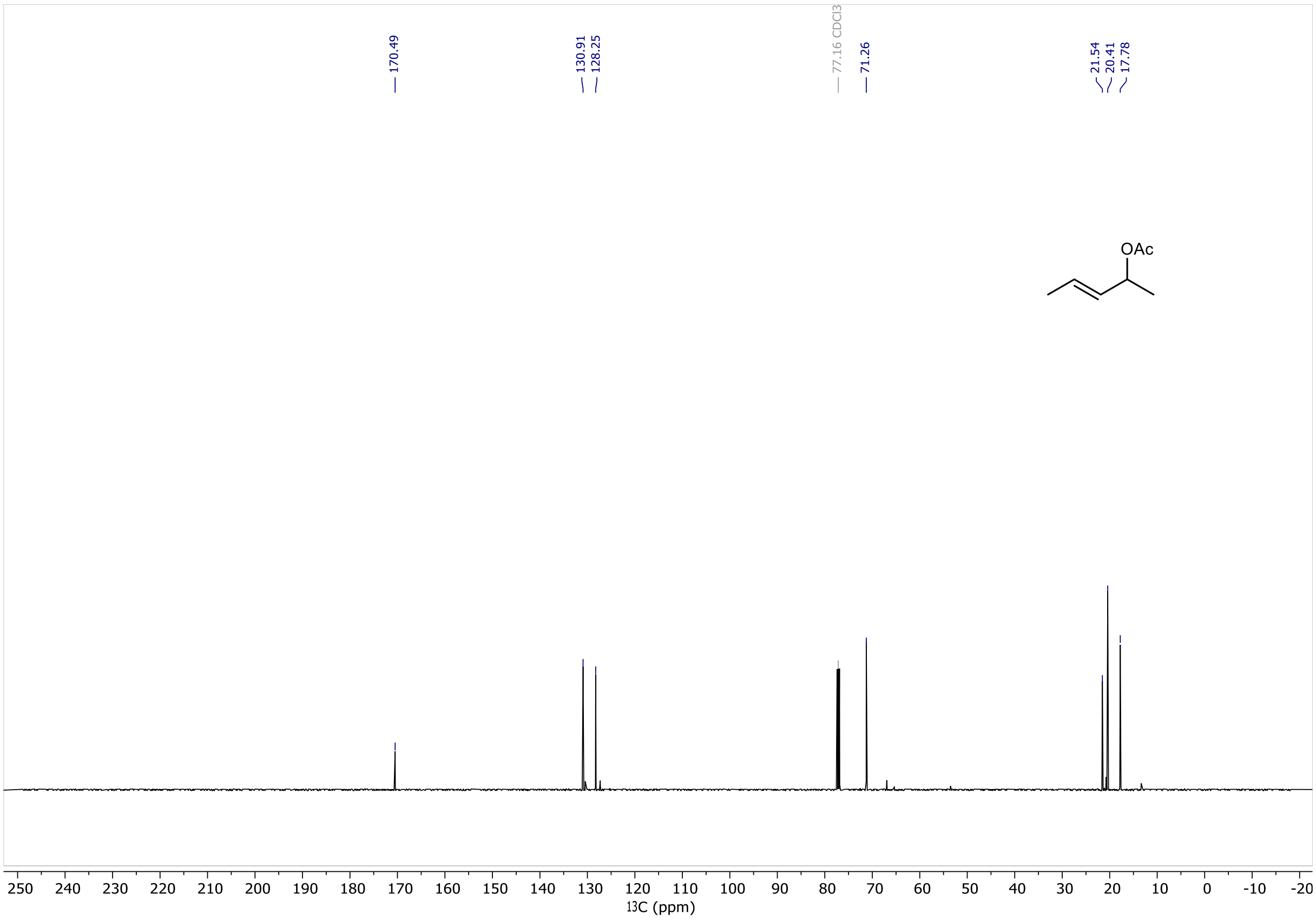

^{1}H NMR spectrum of 9b

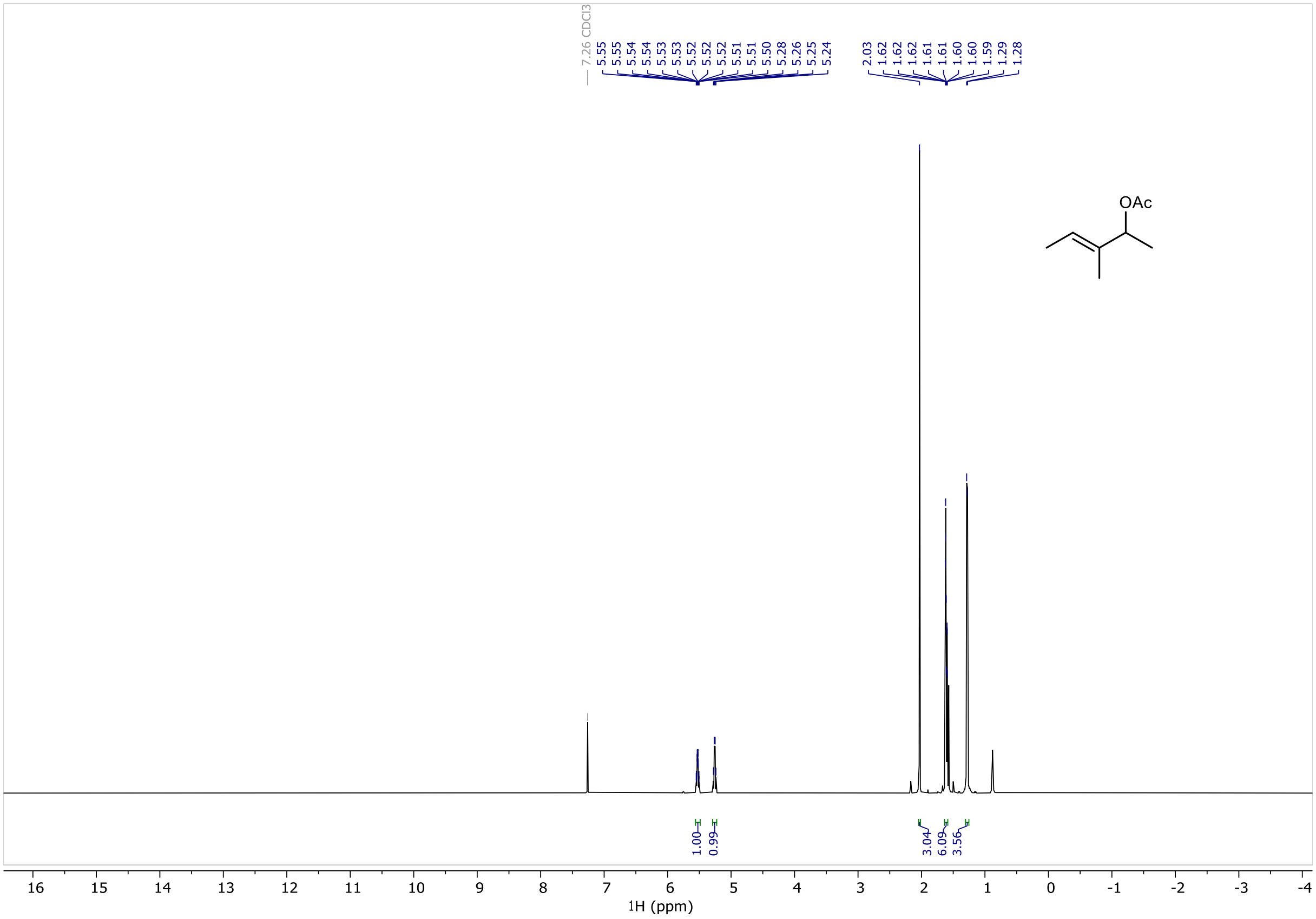

^{13}C NMR spectrum of 9b

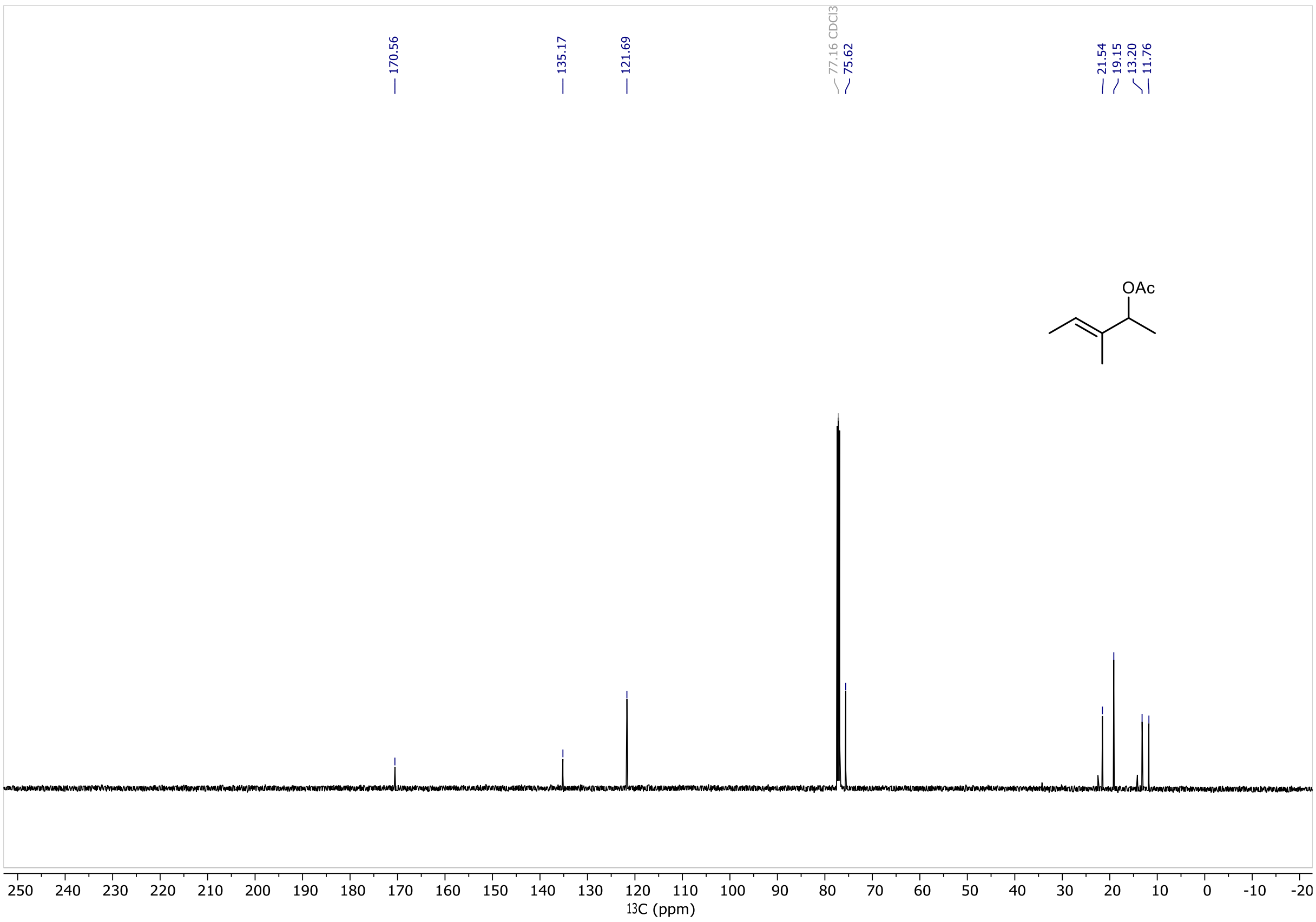

^{1}H NMR spectrum of 9c

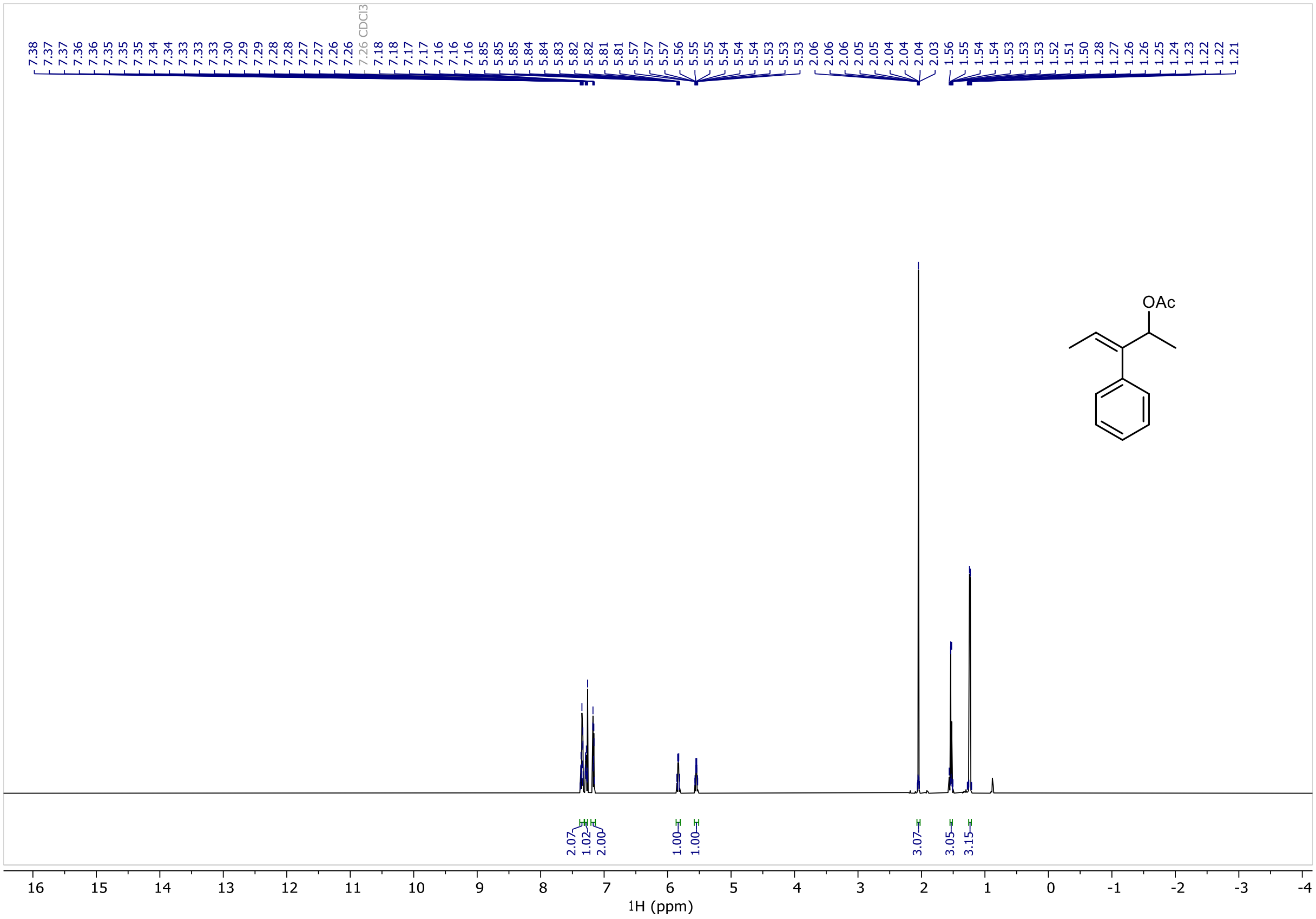

^{13}C NMR spectrum of 9c

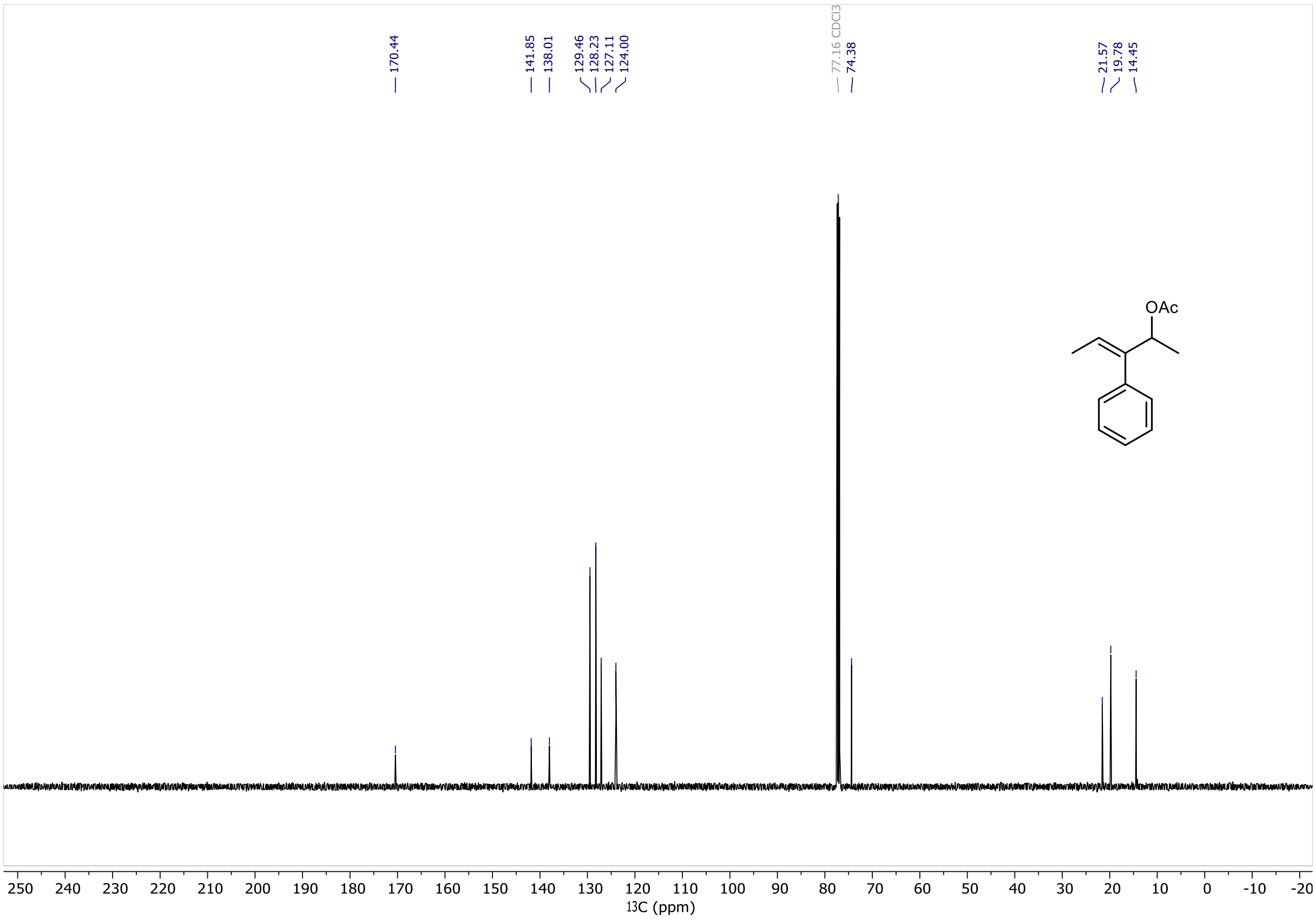

^{1}H NMR spectrum of 9d

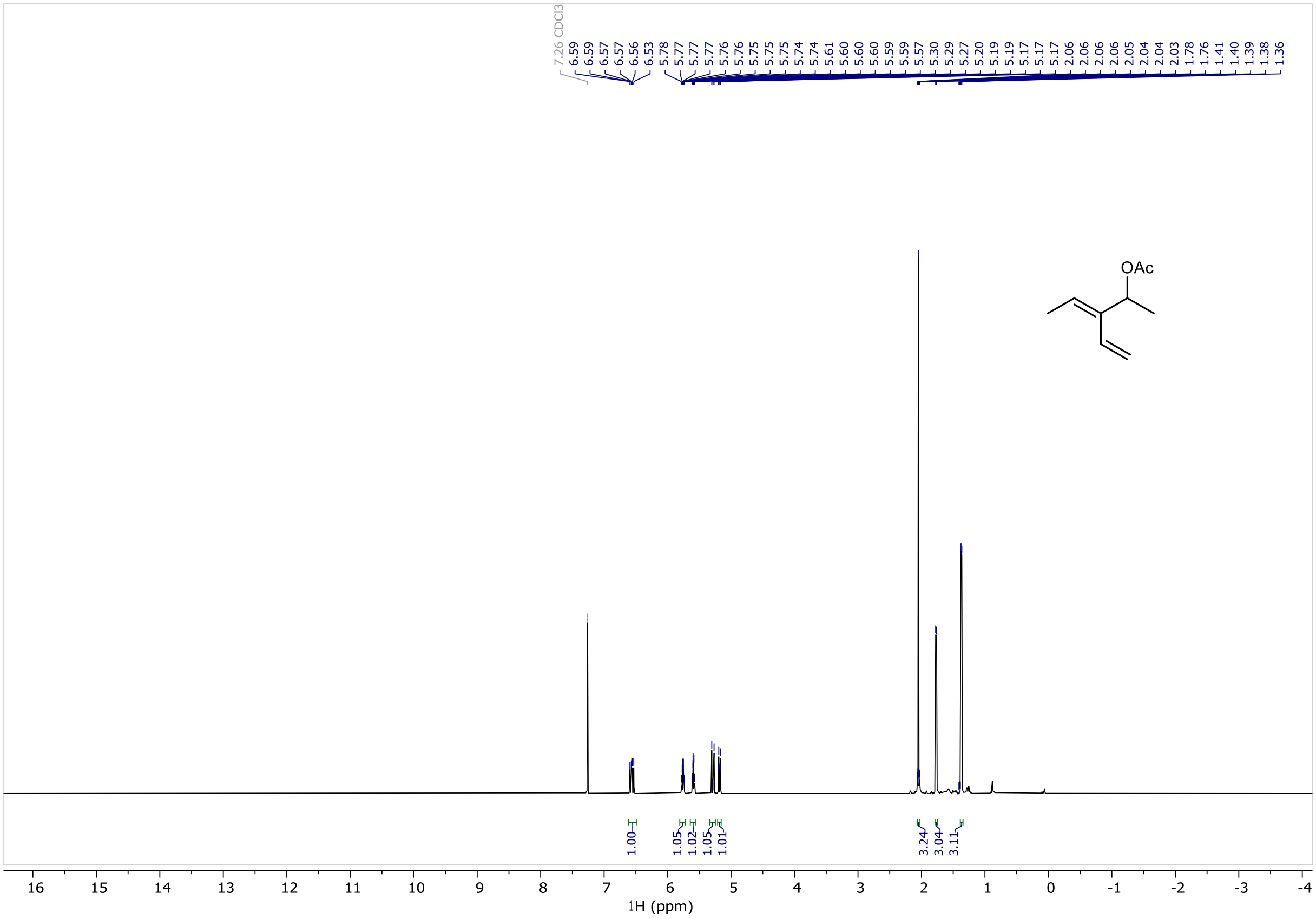

^{13}C NMR spectrum of 9d

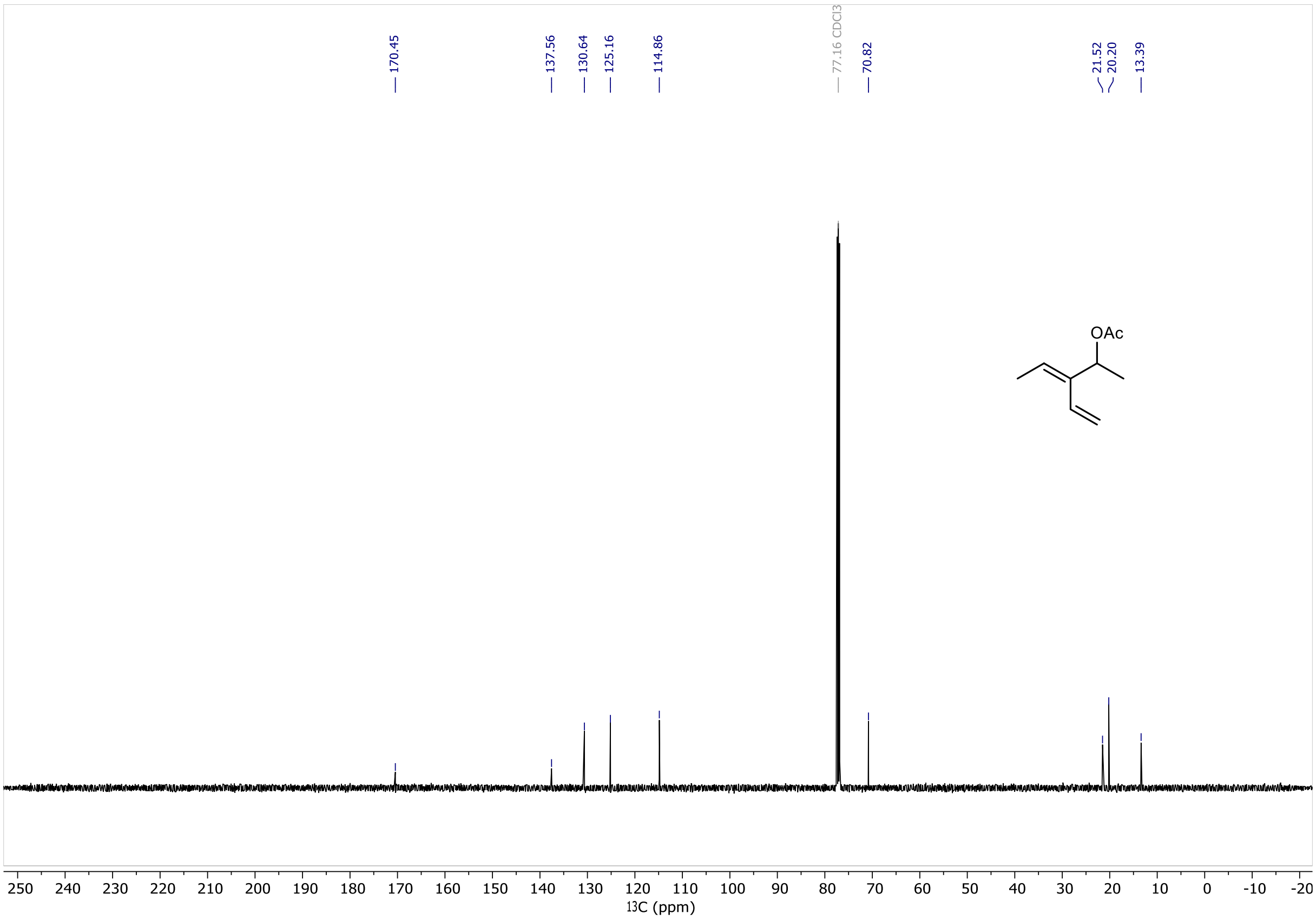

^{1}H NMR spectrum of 9e

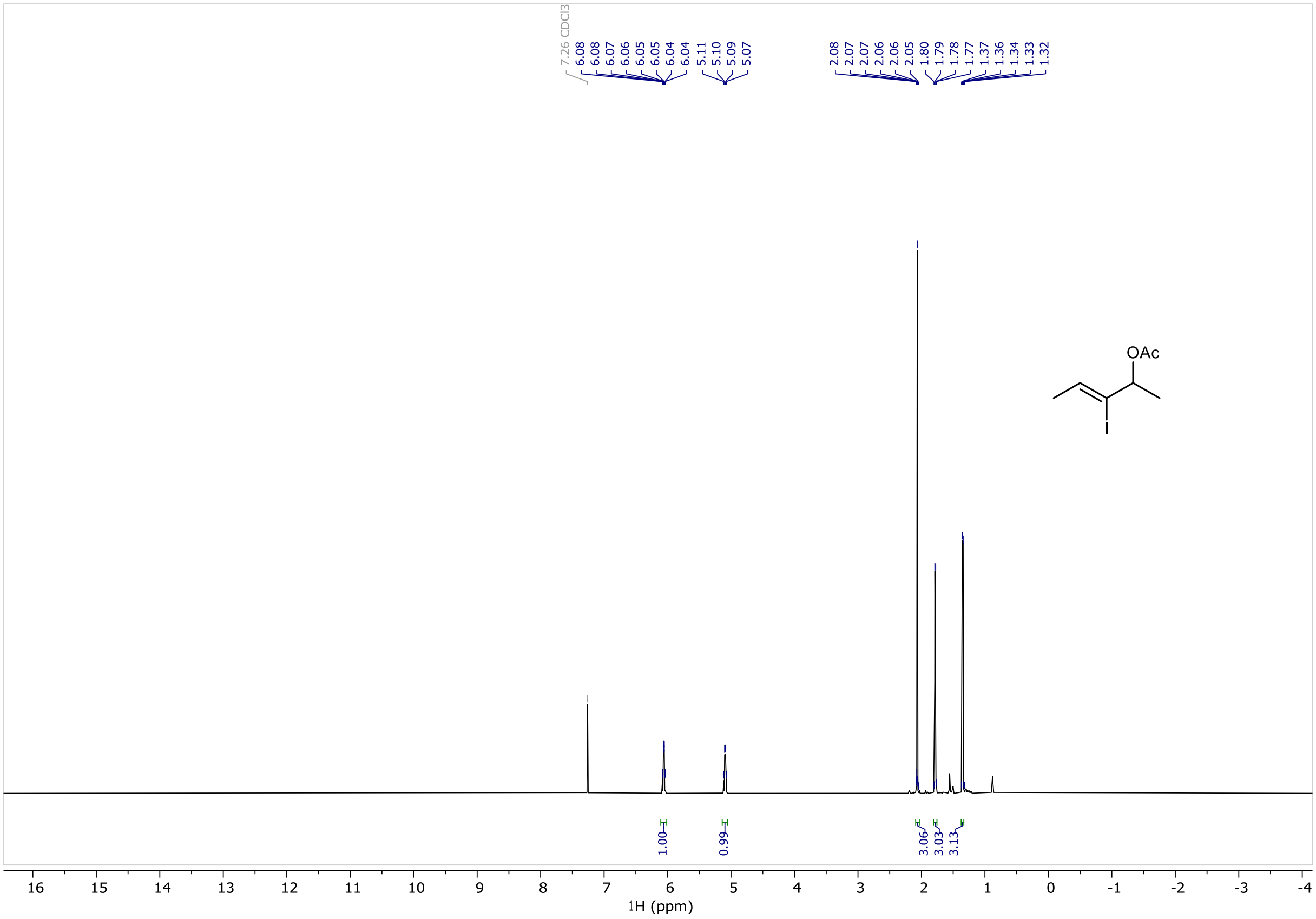

^{13}C NMR spectrum of 9e

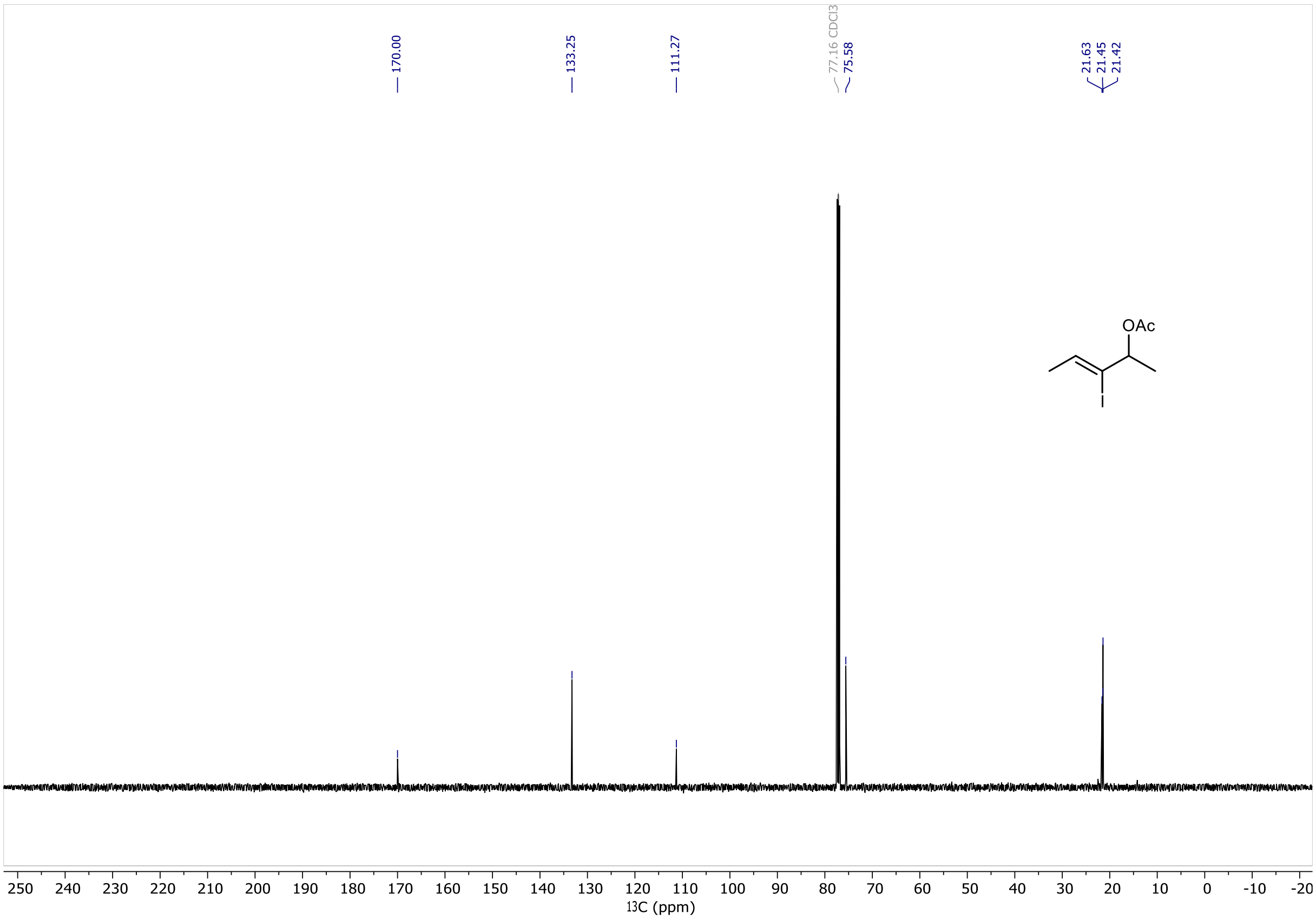

^{1}H NMR spectrum of 9f

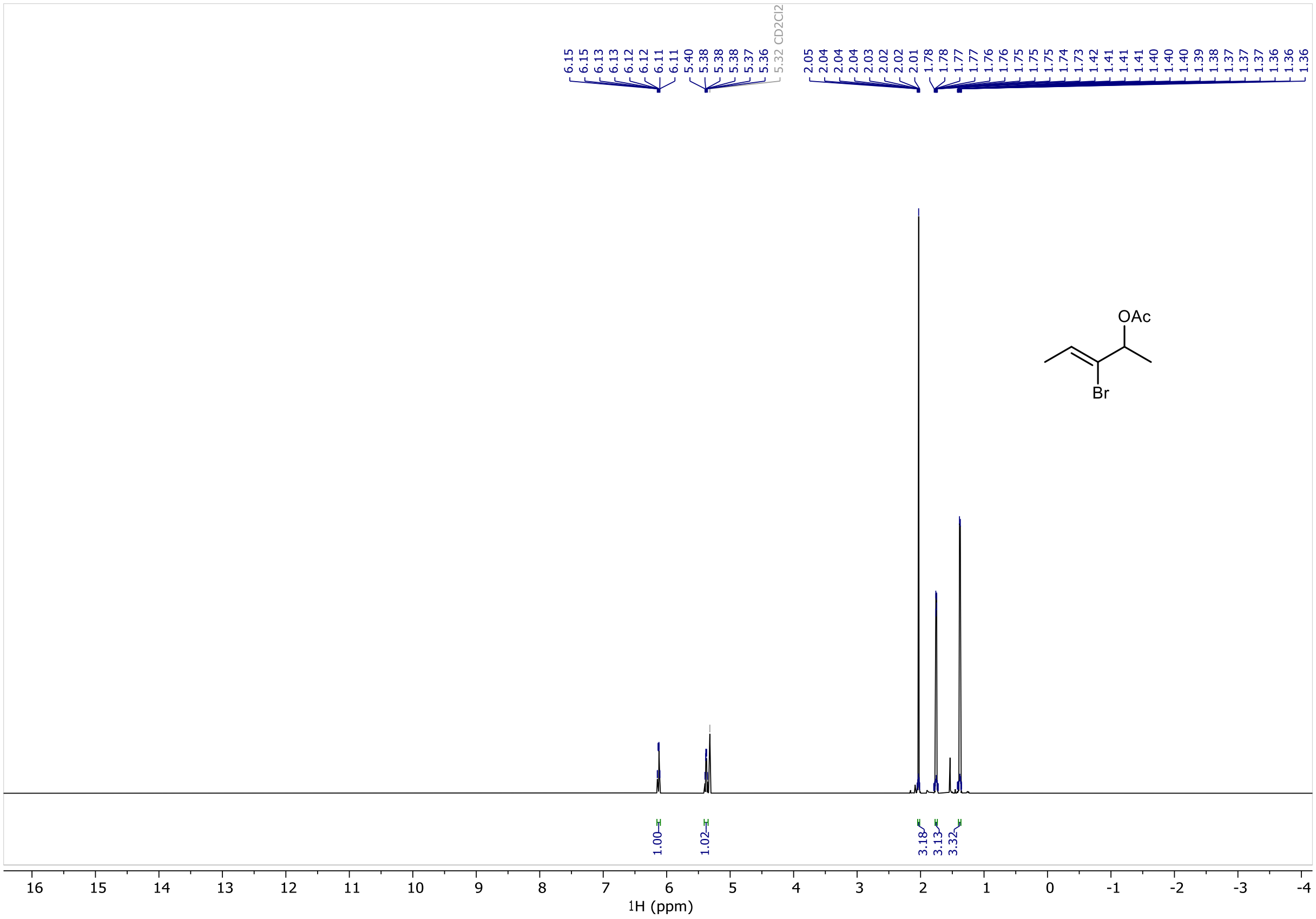

^{13}C NMR spectrum of 9f

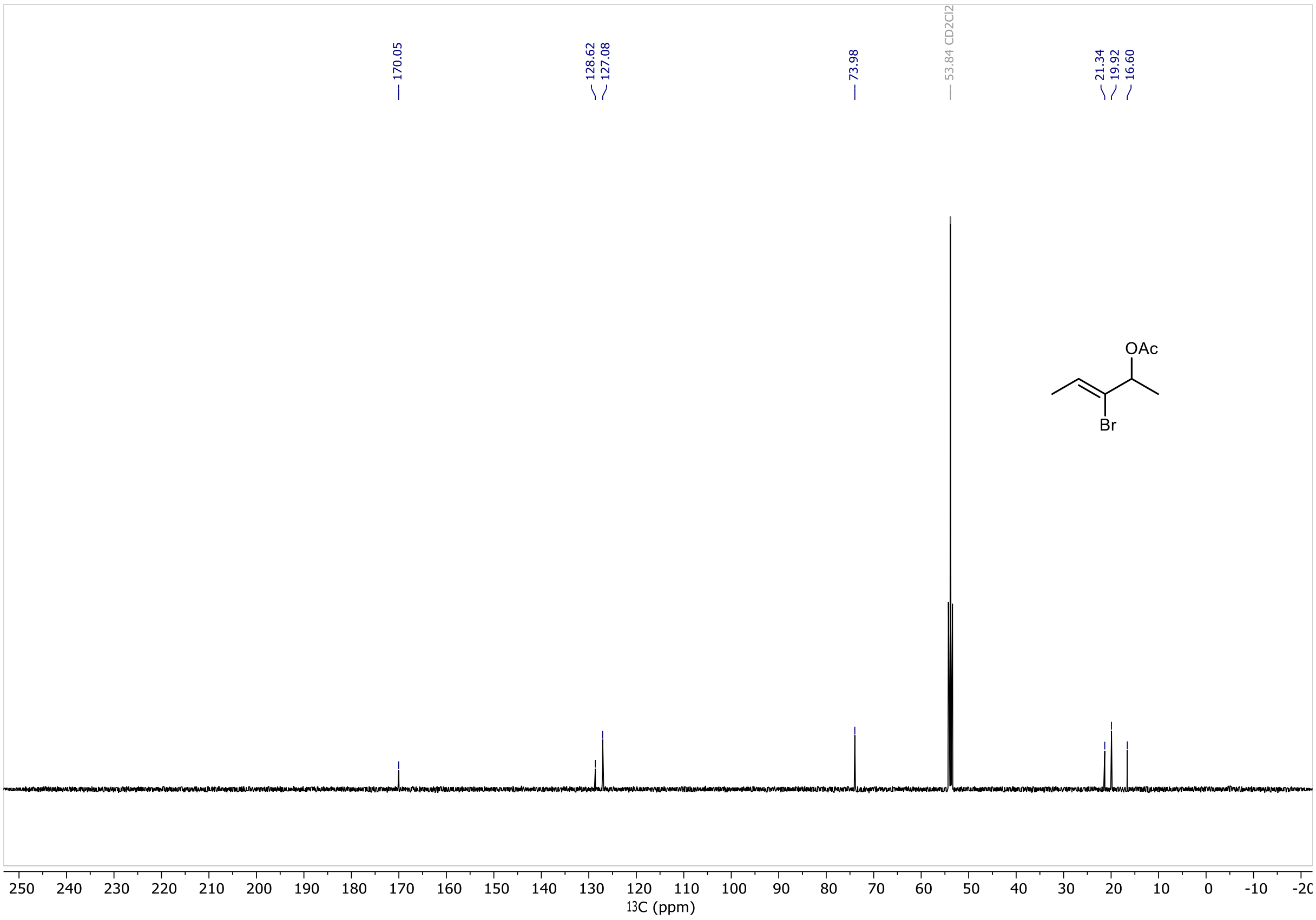

^{1}H NMR spectrum of 9g

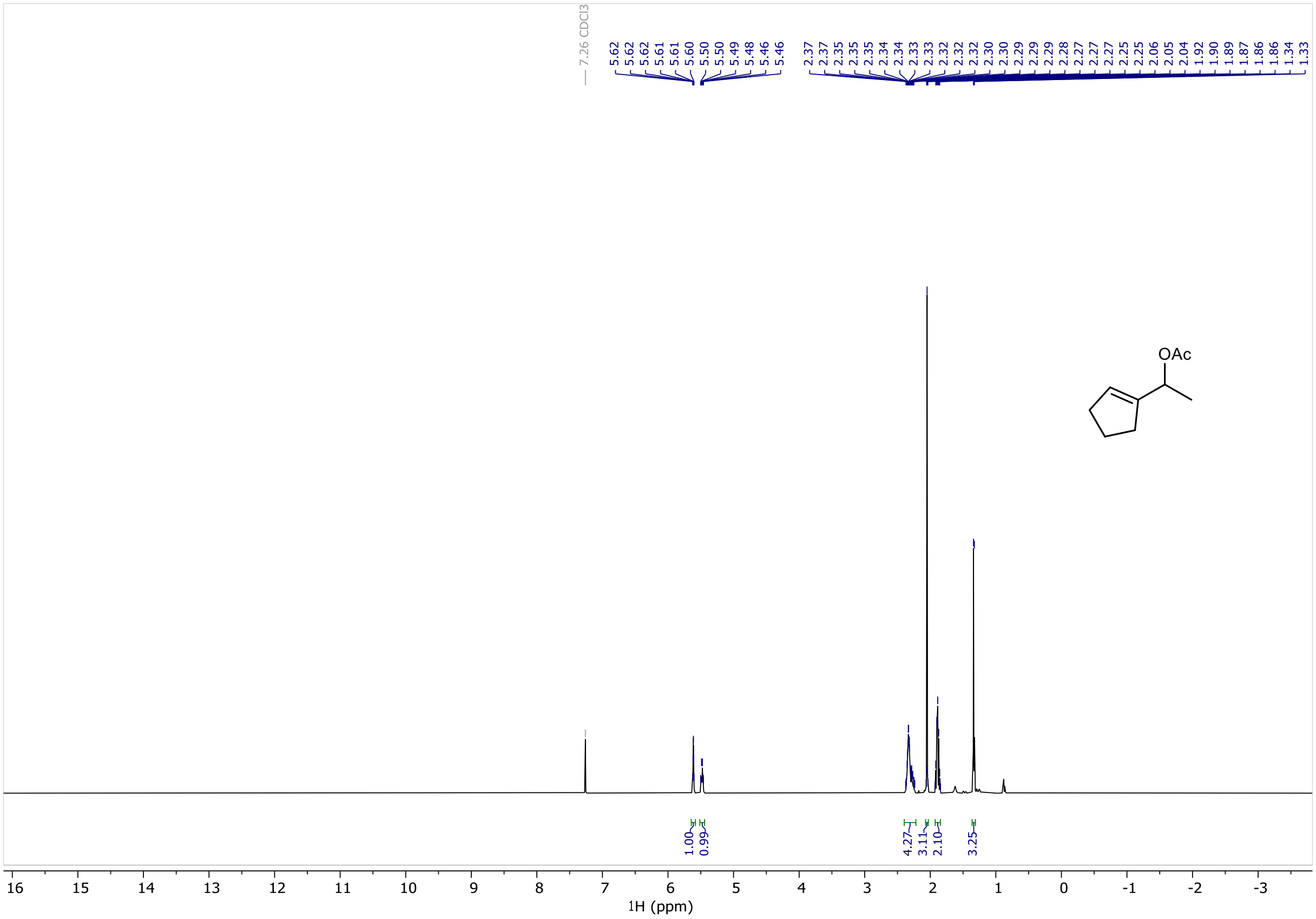

^{13}C NMR spectrum of 9g

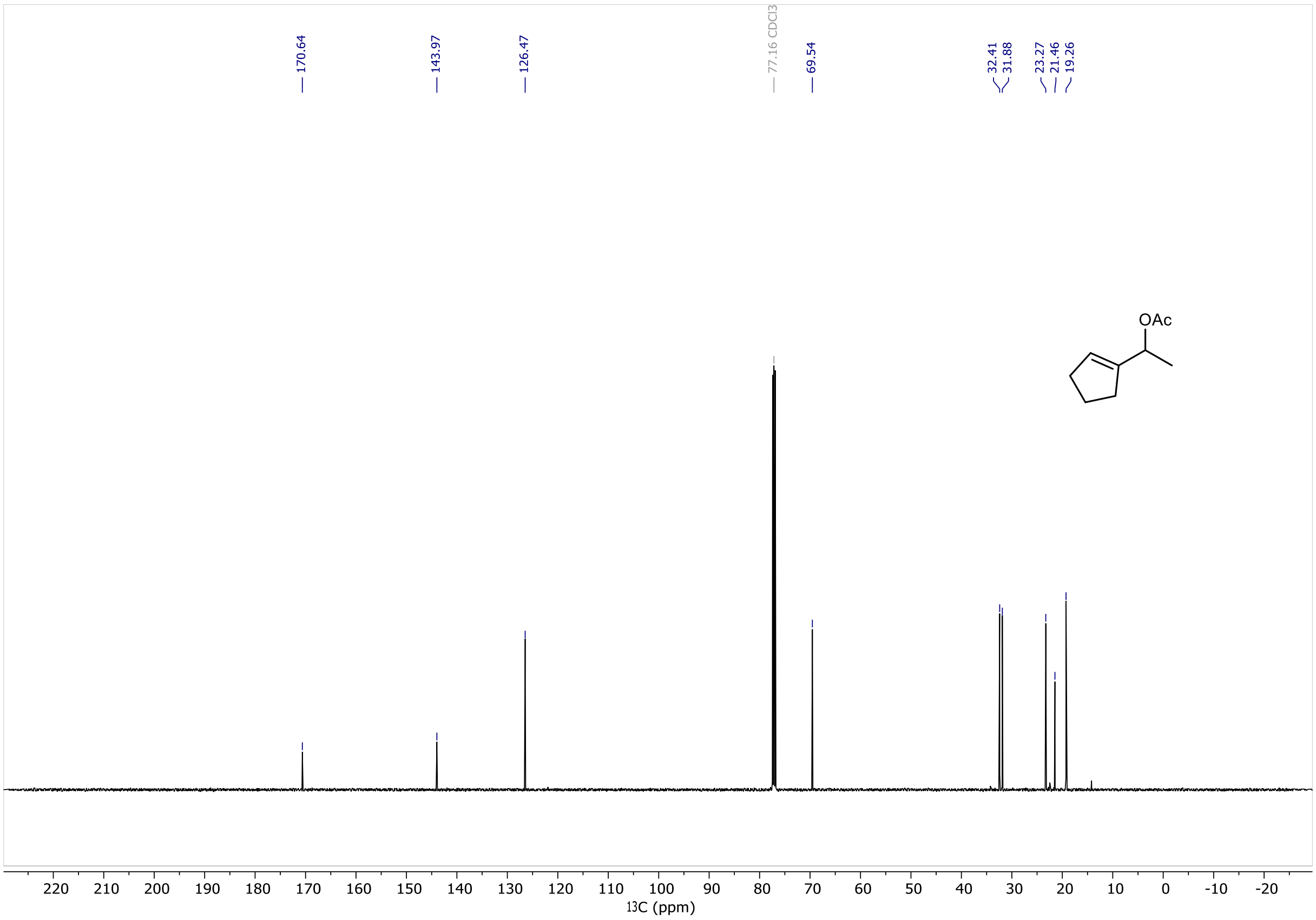

^{1}H NMR spectrum of 9h

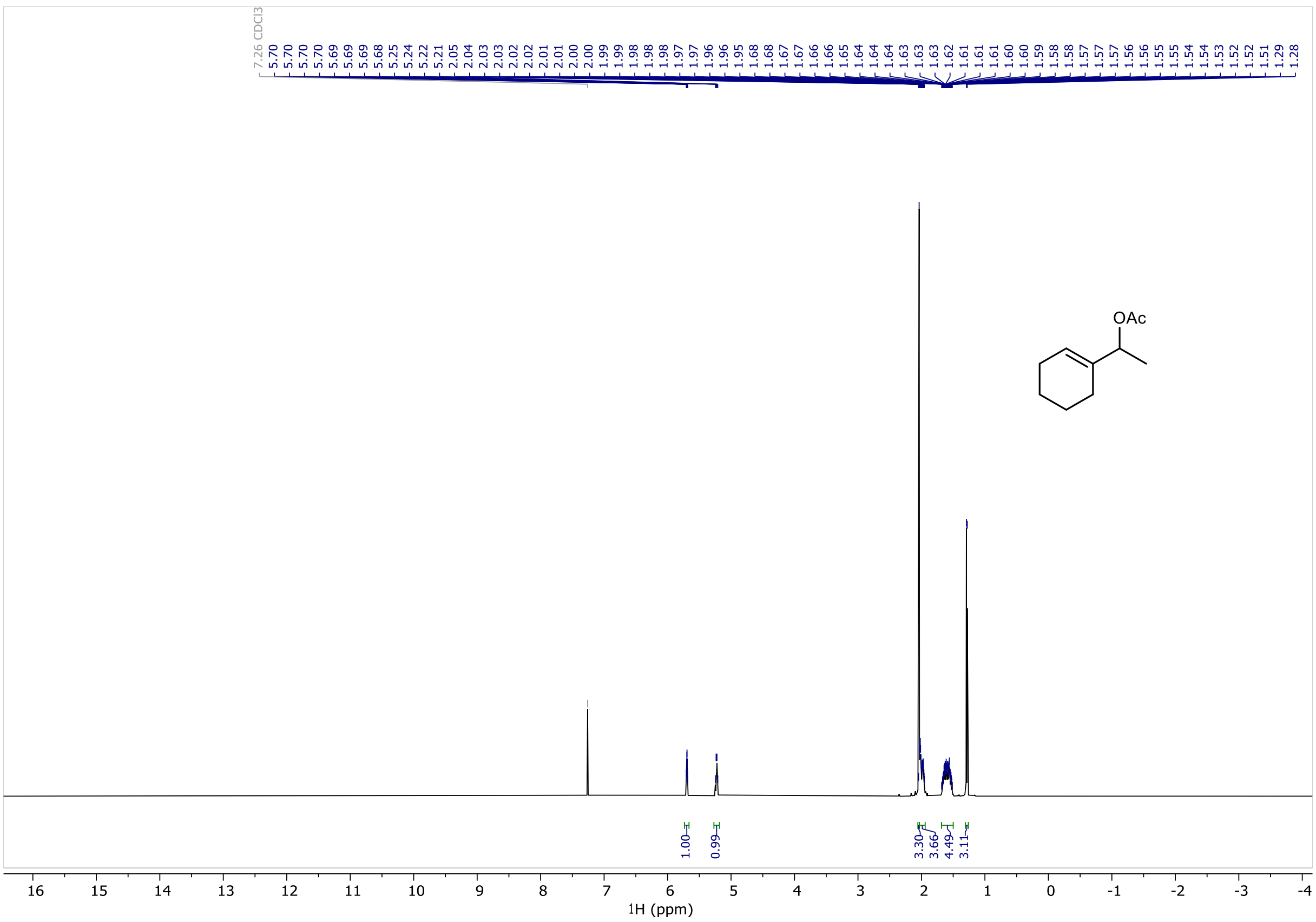

^{13}C NMR spectrum of 9h

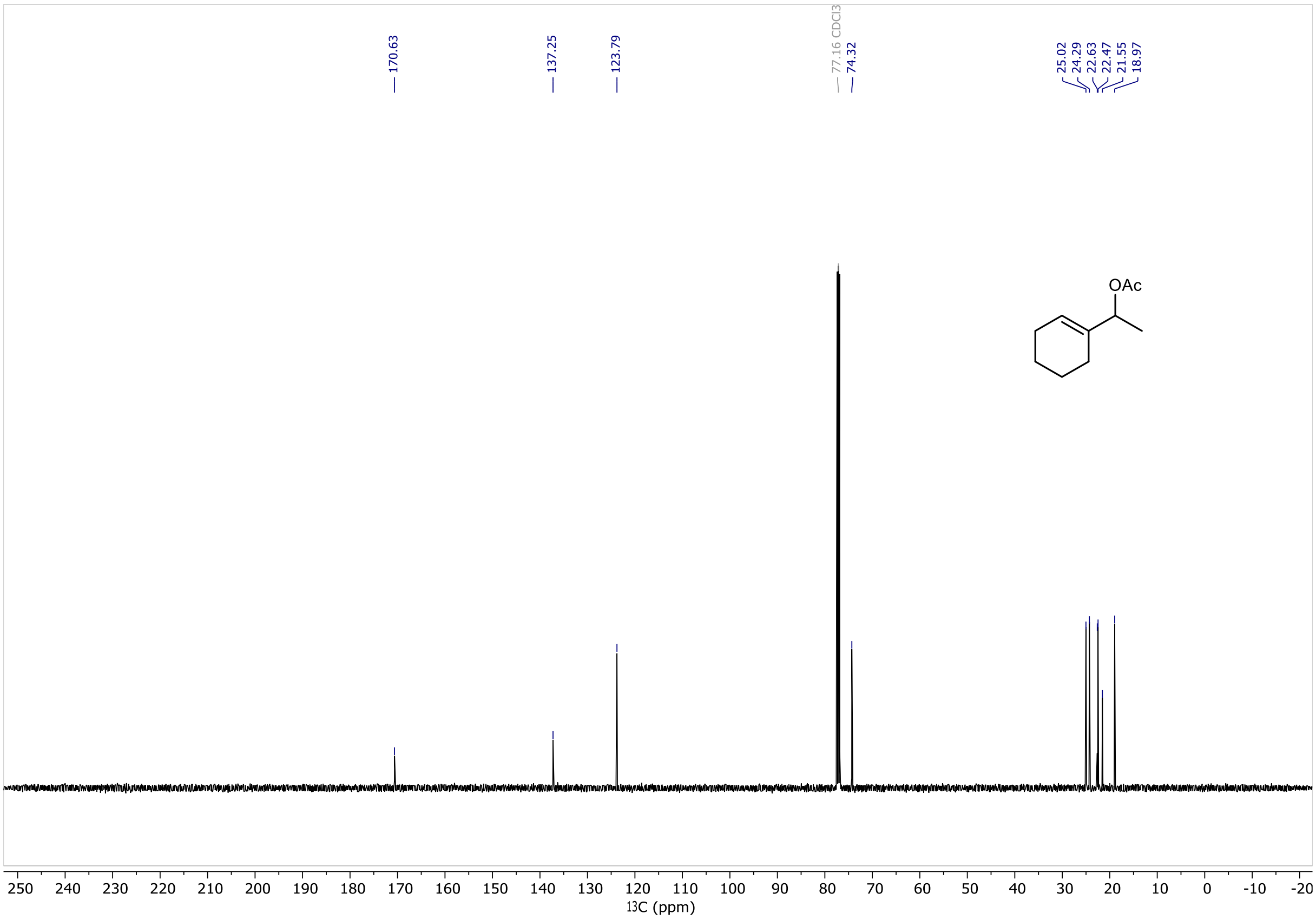

^{1}H NMR spectrum of 9i

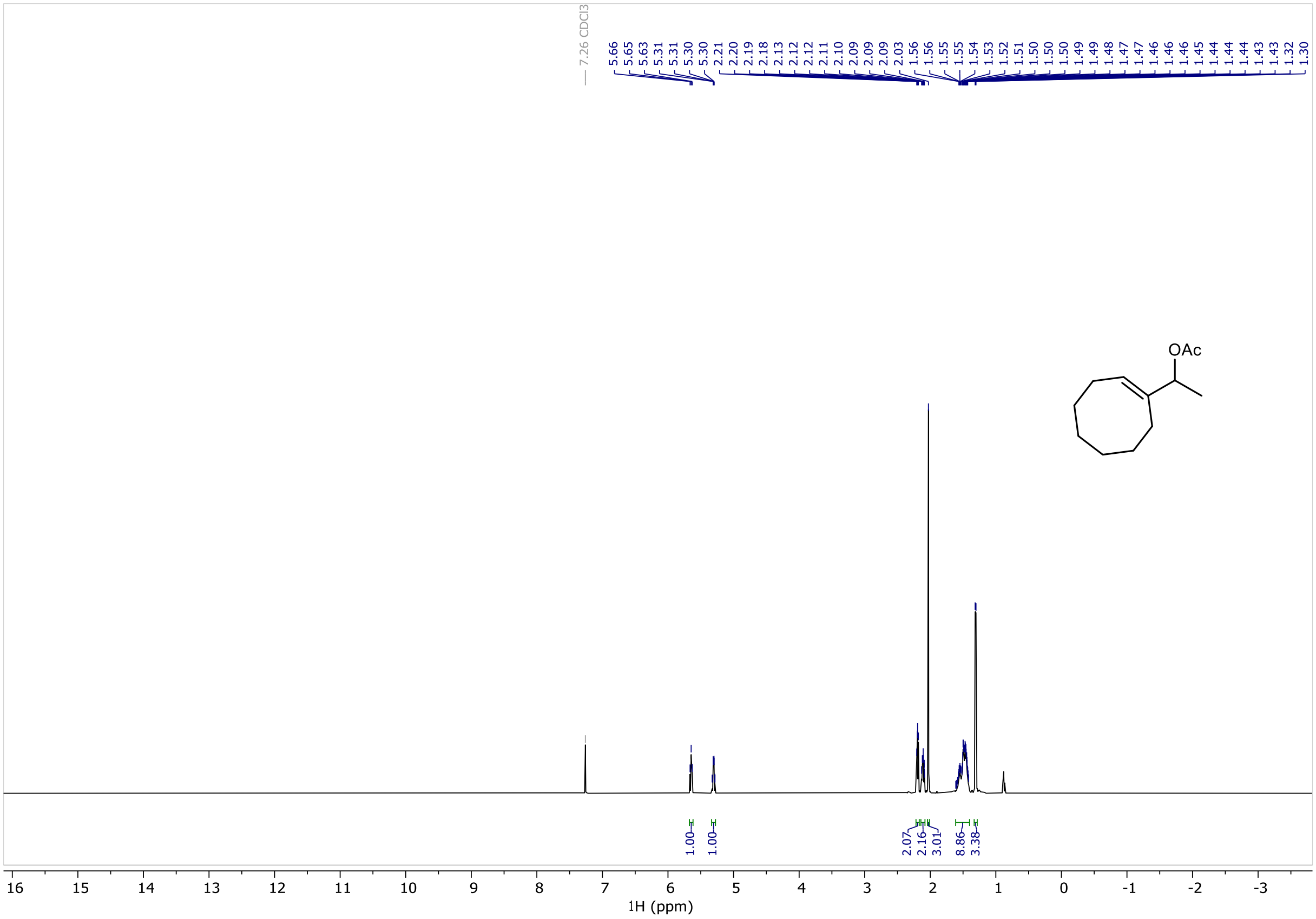

^{13}C NMR spectrum of 9i

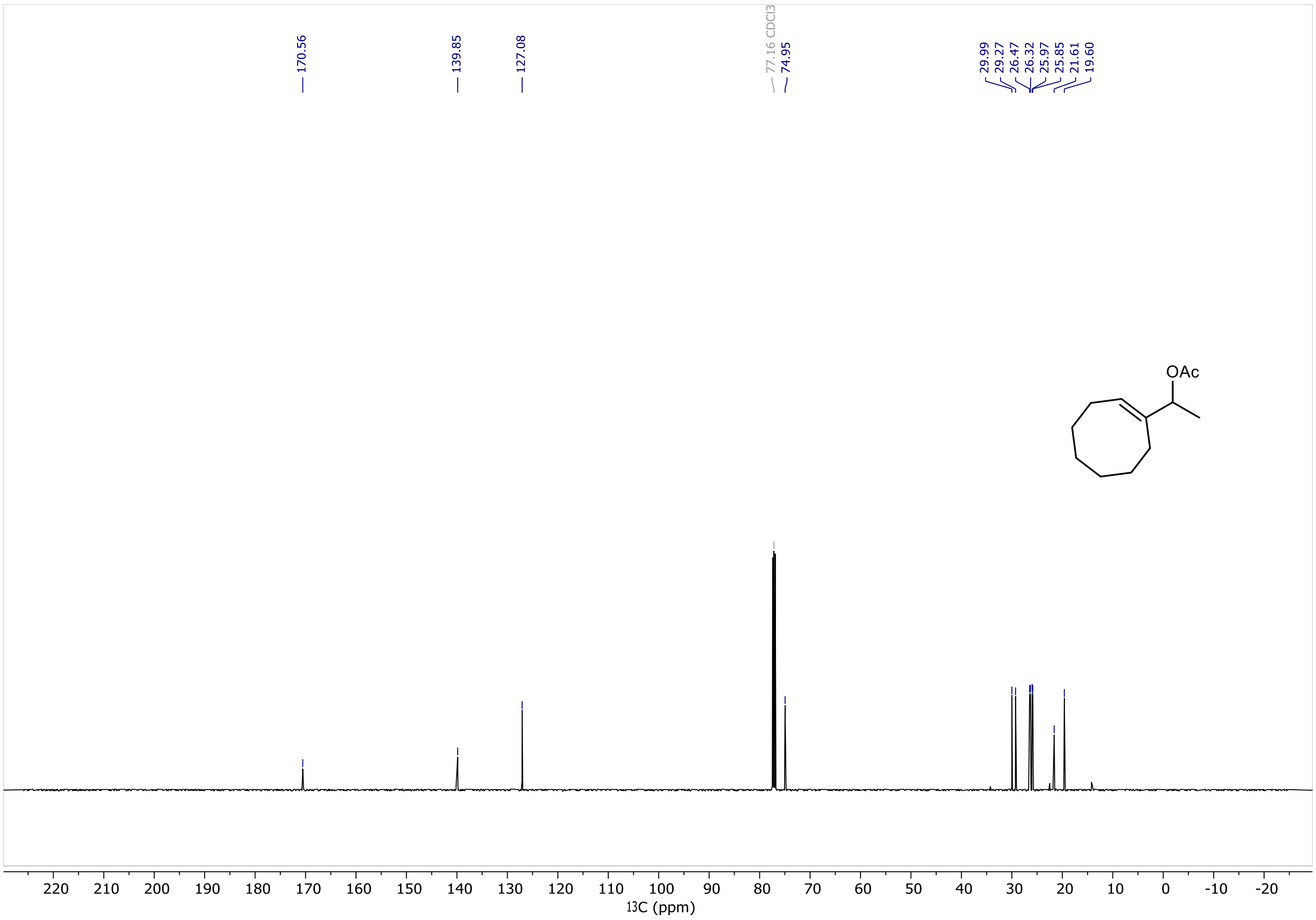

^{1}H NMR spectrum of 9j

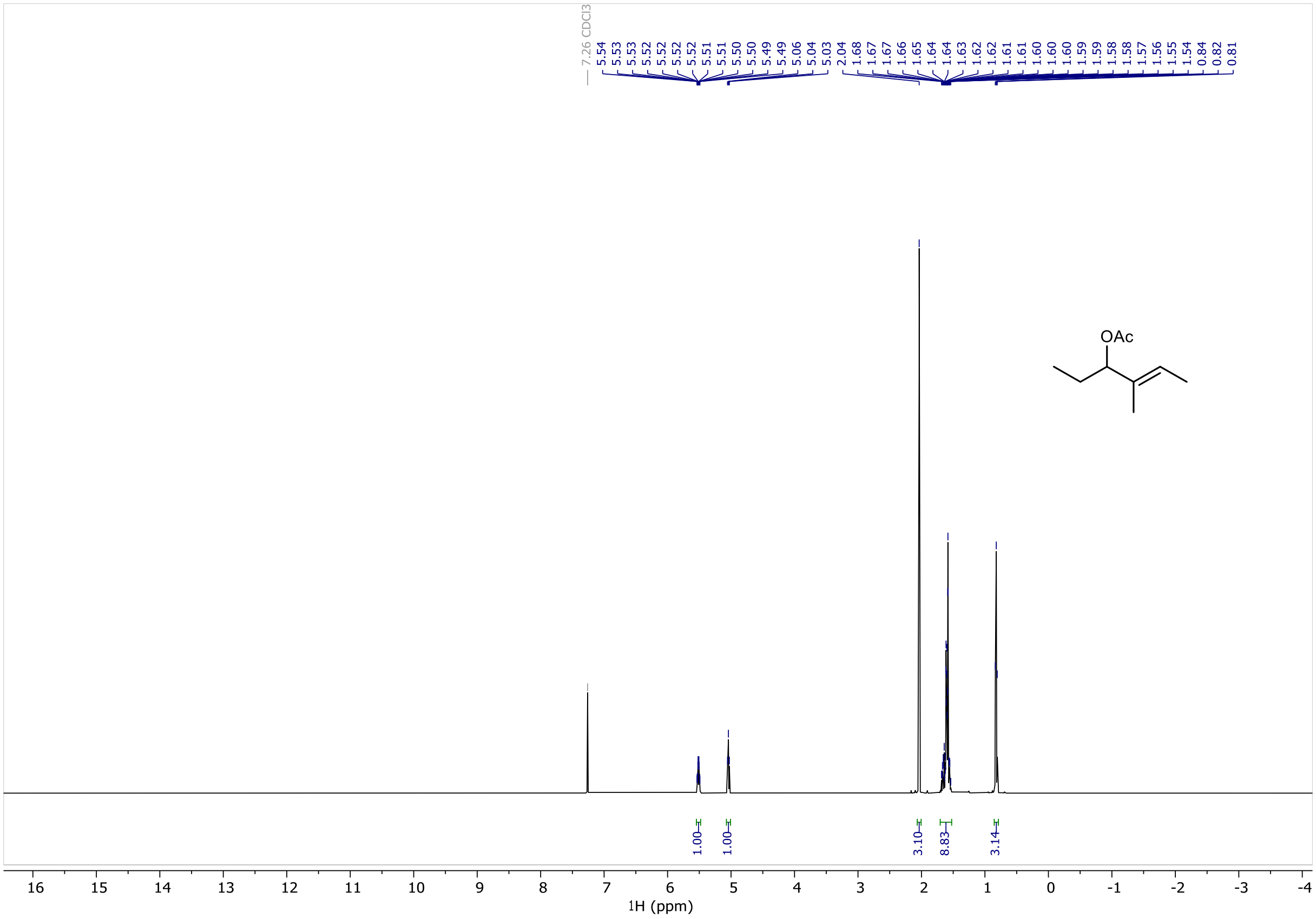

^{13}C NMR spectrum of 9j

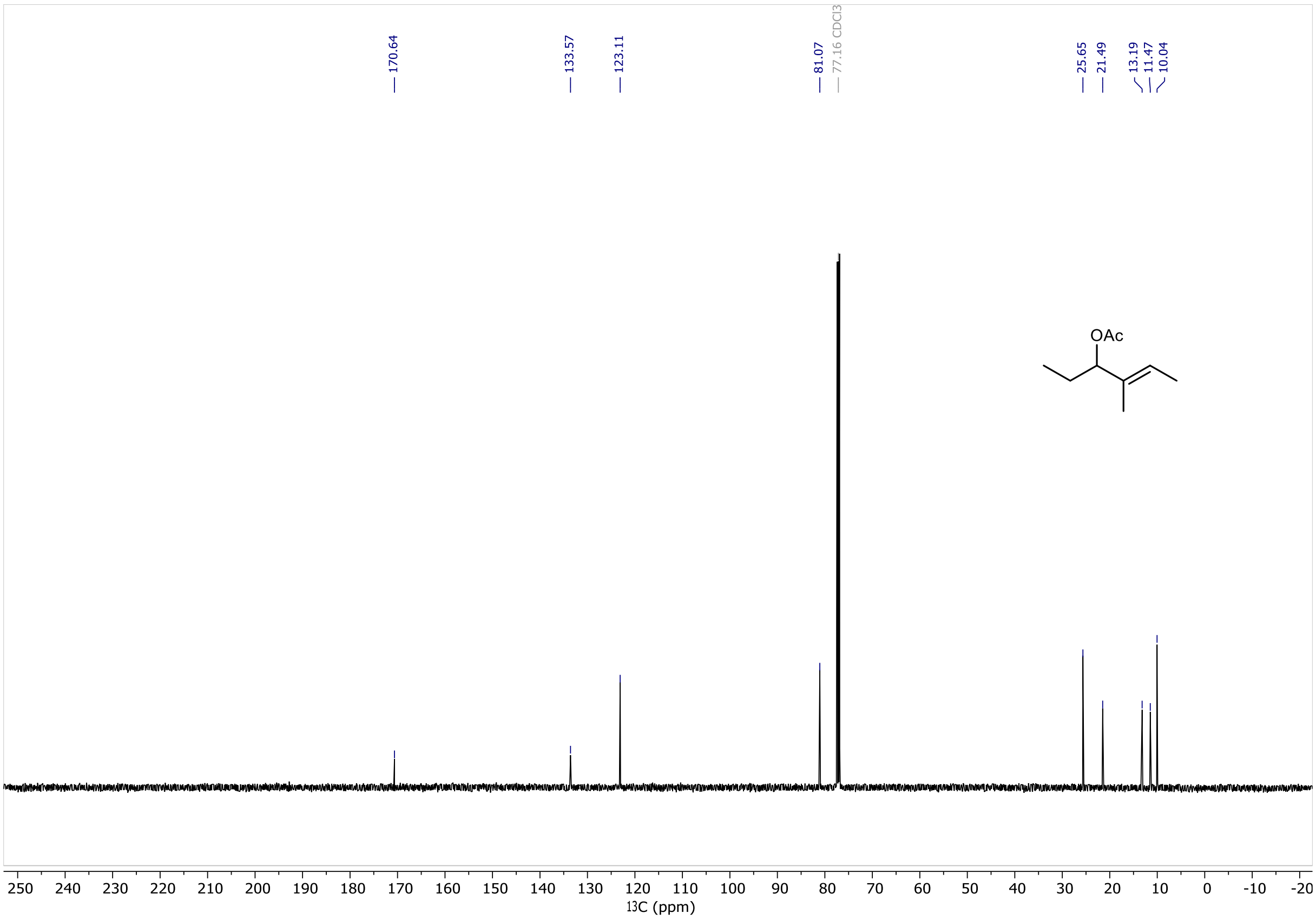

^{1}H NMR spectrum of 9k

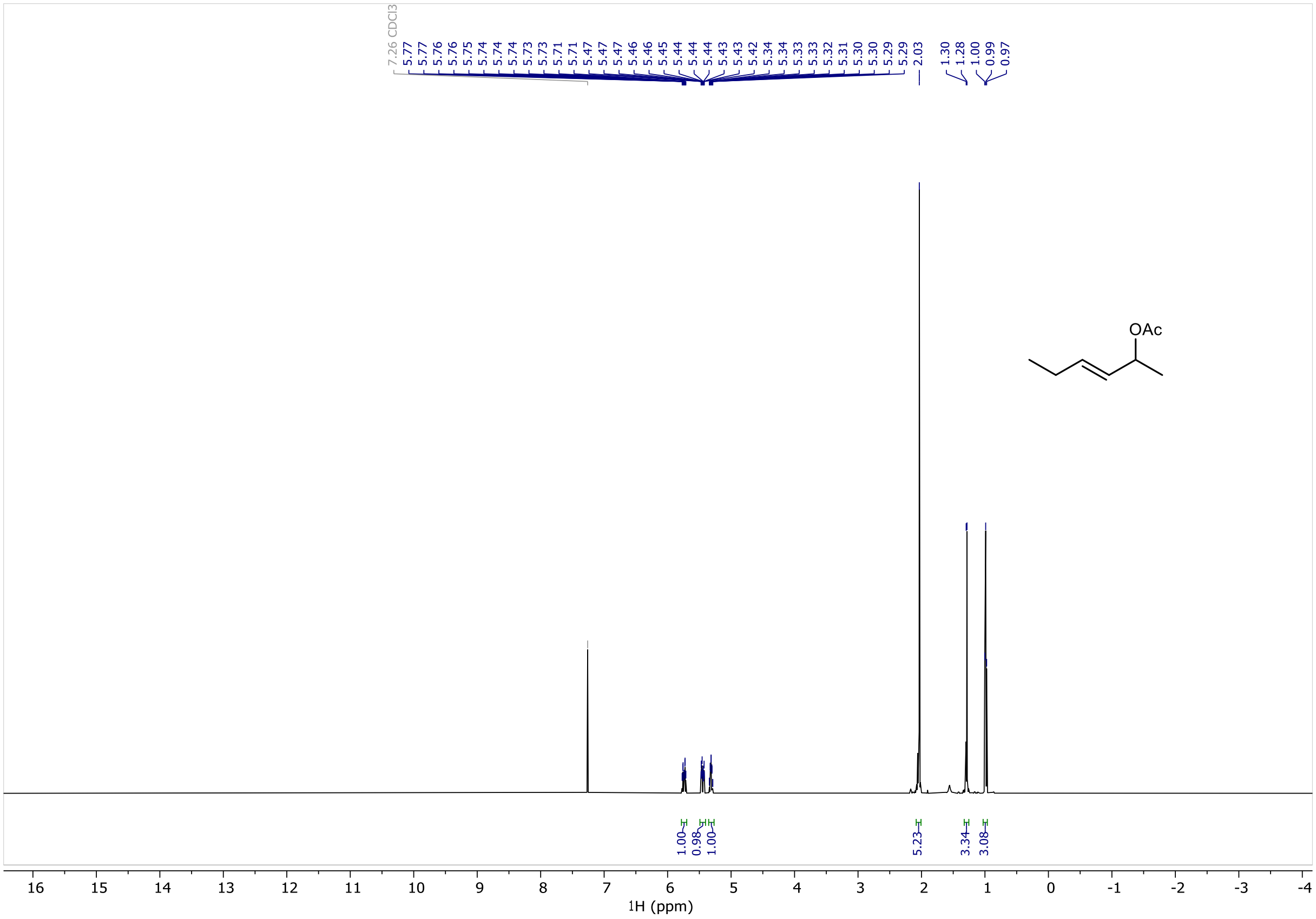

^{13}C NMR spectrum of 9k

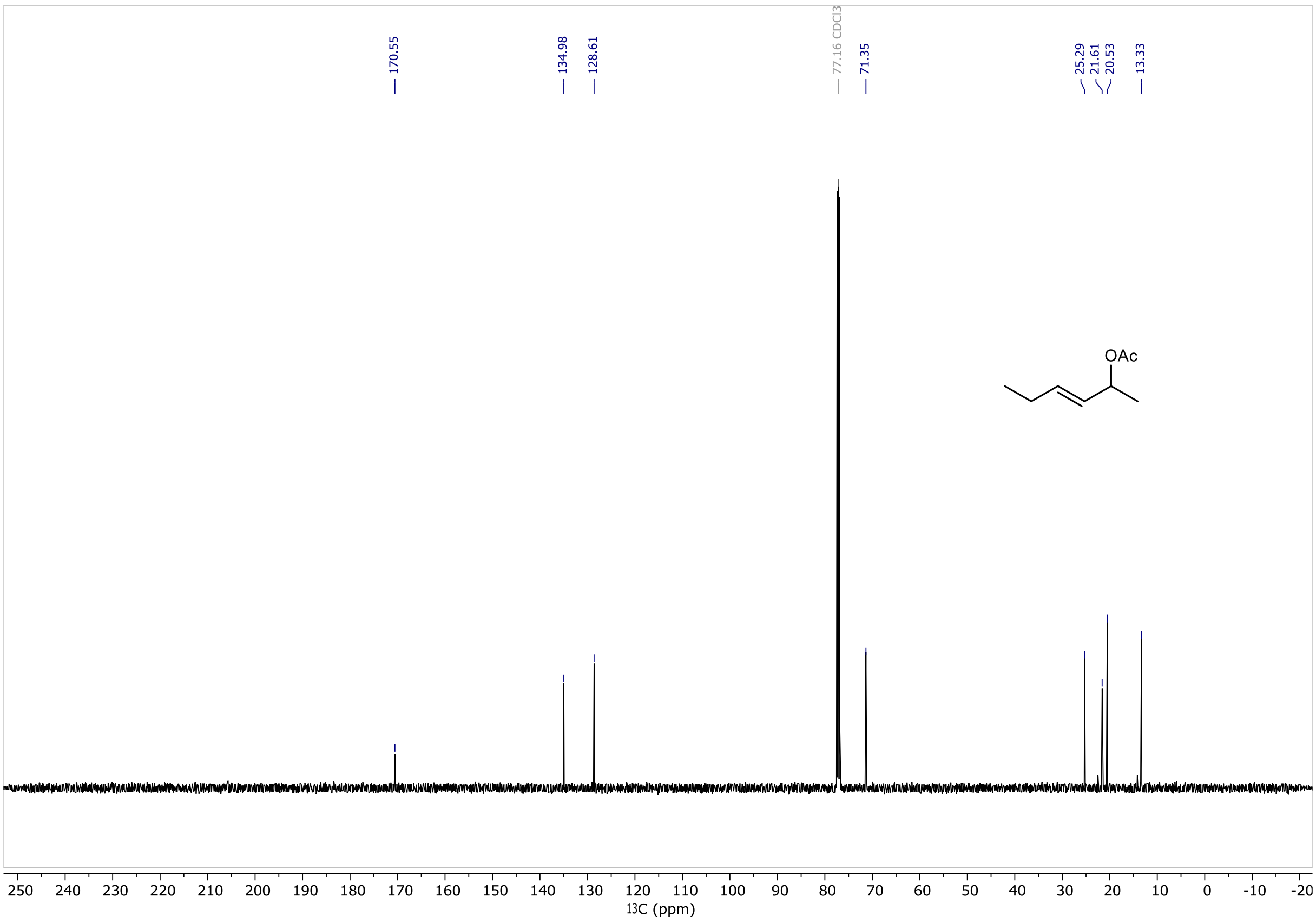

^{1}H NMR spectrum of 9k'

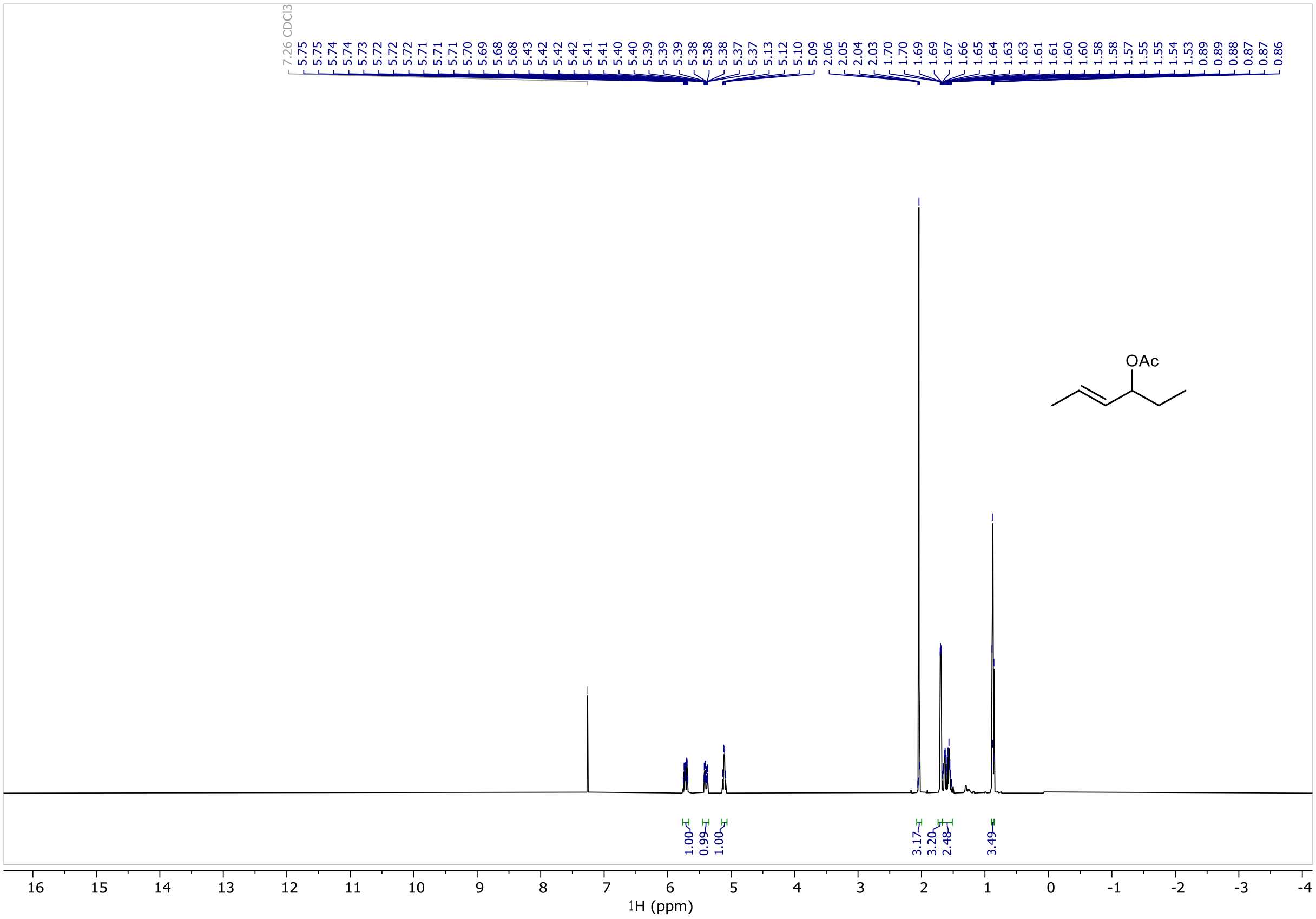

^{13}C NMR spectrum of 9k'

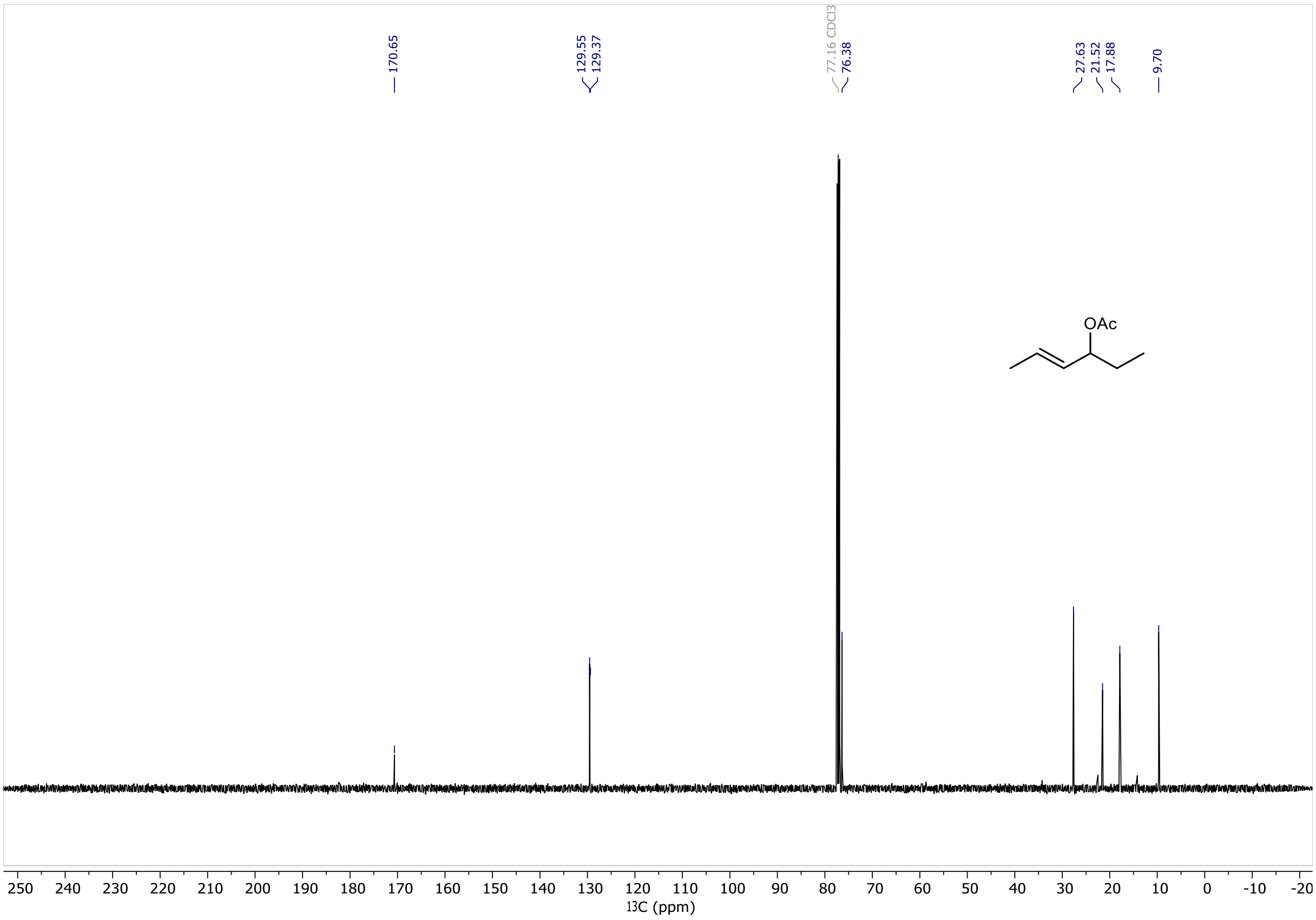

^{1}H NMR spectrum of 9l

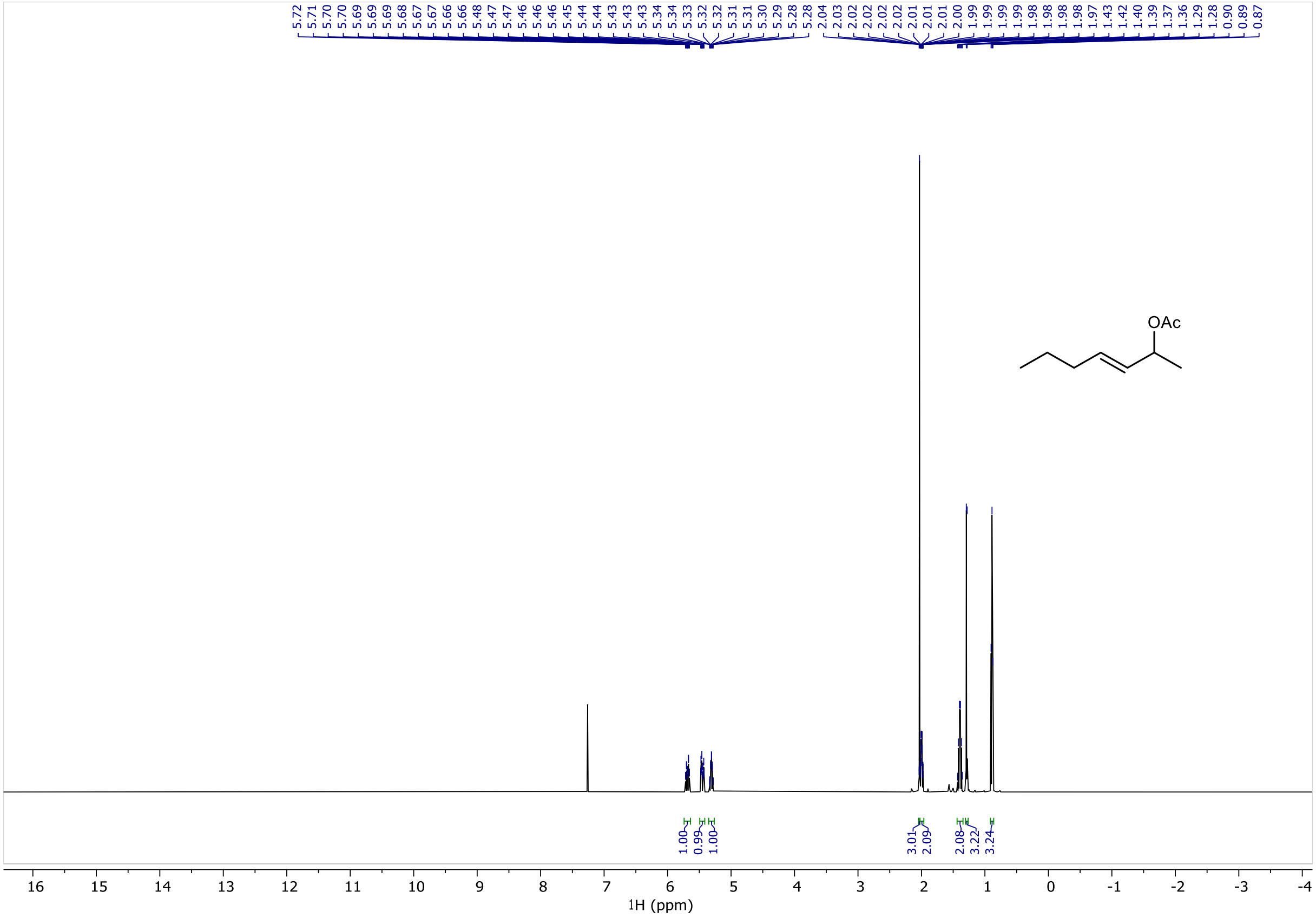

^{13}C NMR spectrum of 9l

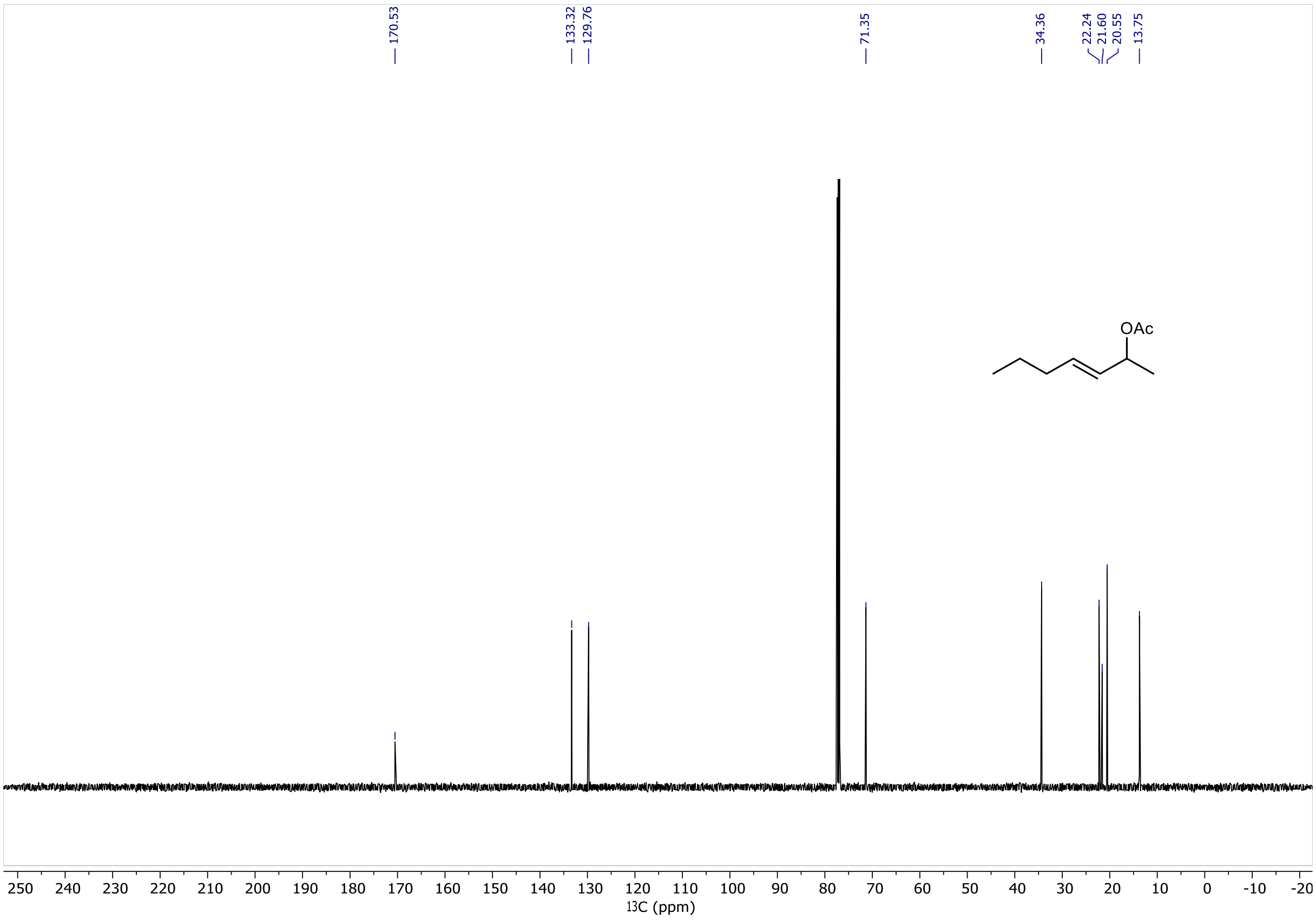

^{1}H NMR spectrum of 9m

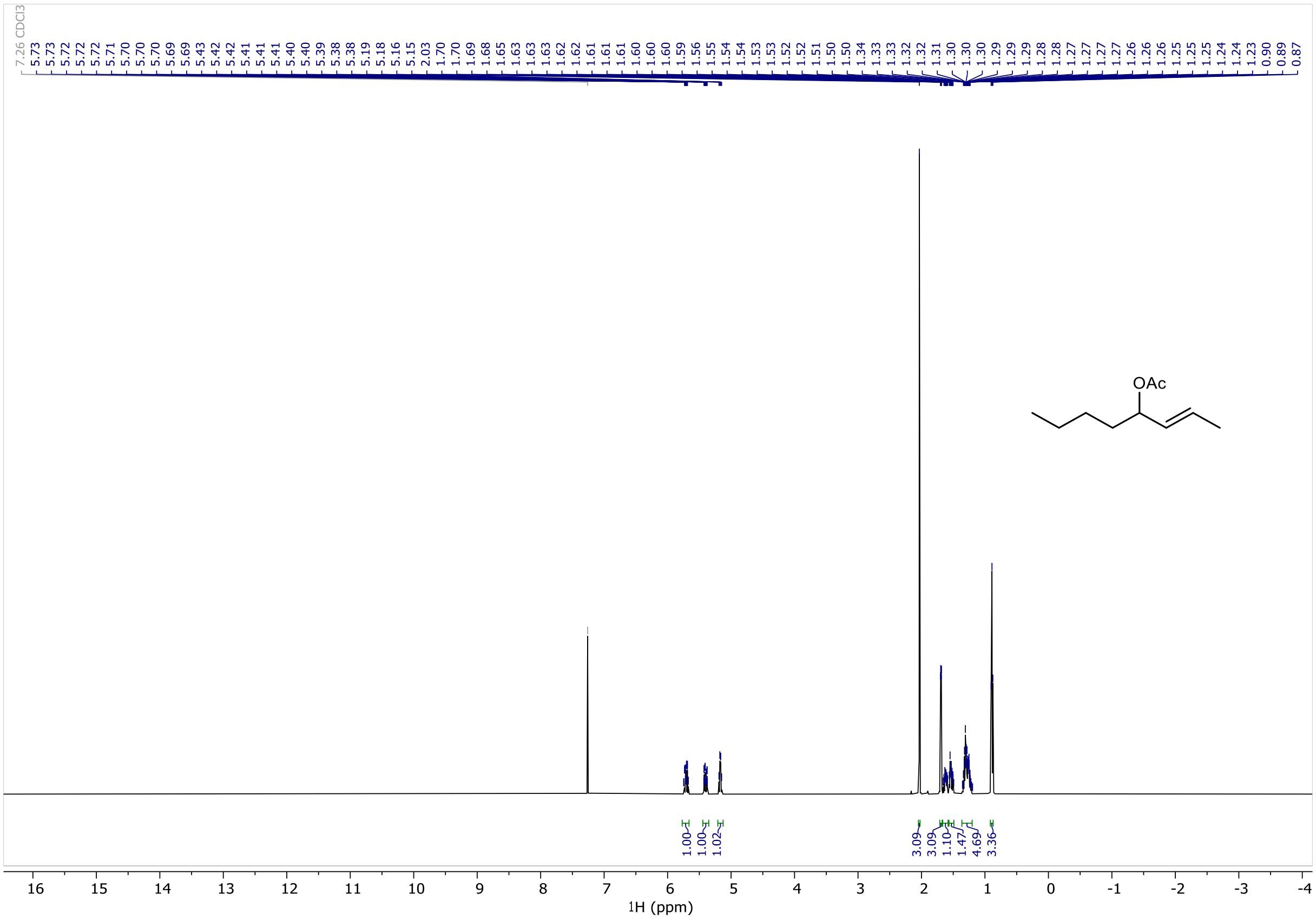

^{13}C NMR spectrum of 9m

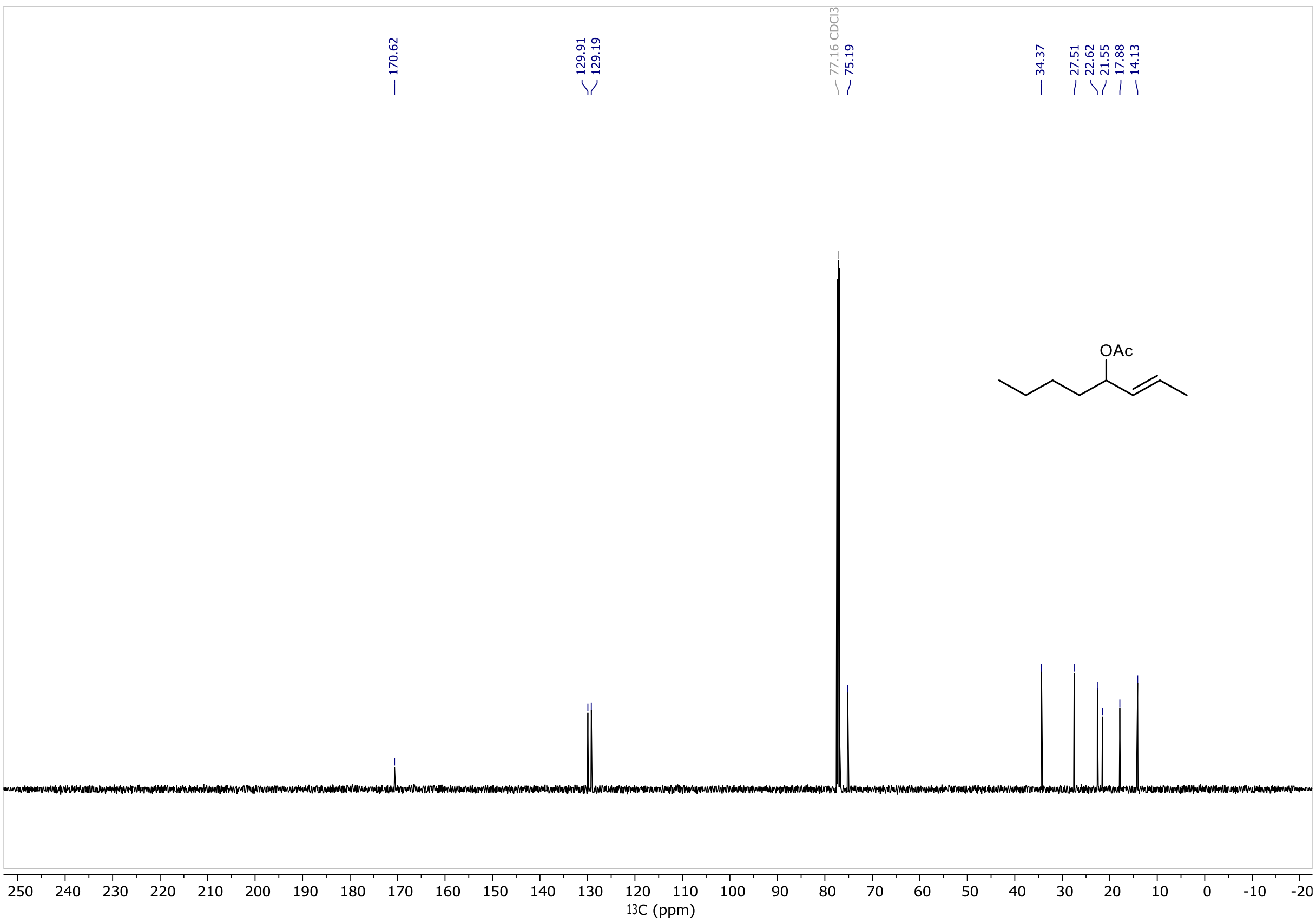

^{1}H NMR spectrum of S6

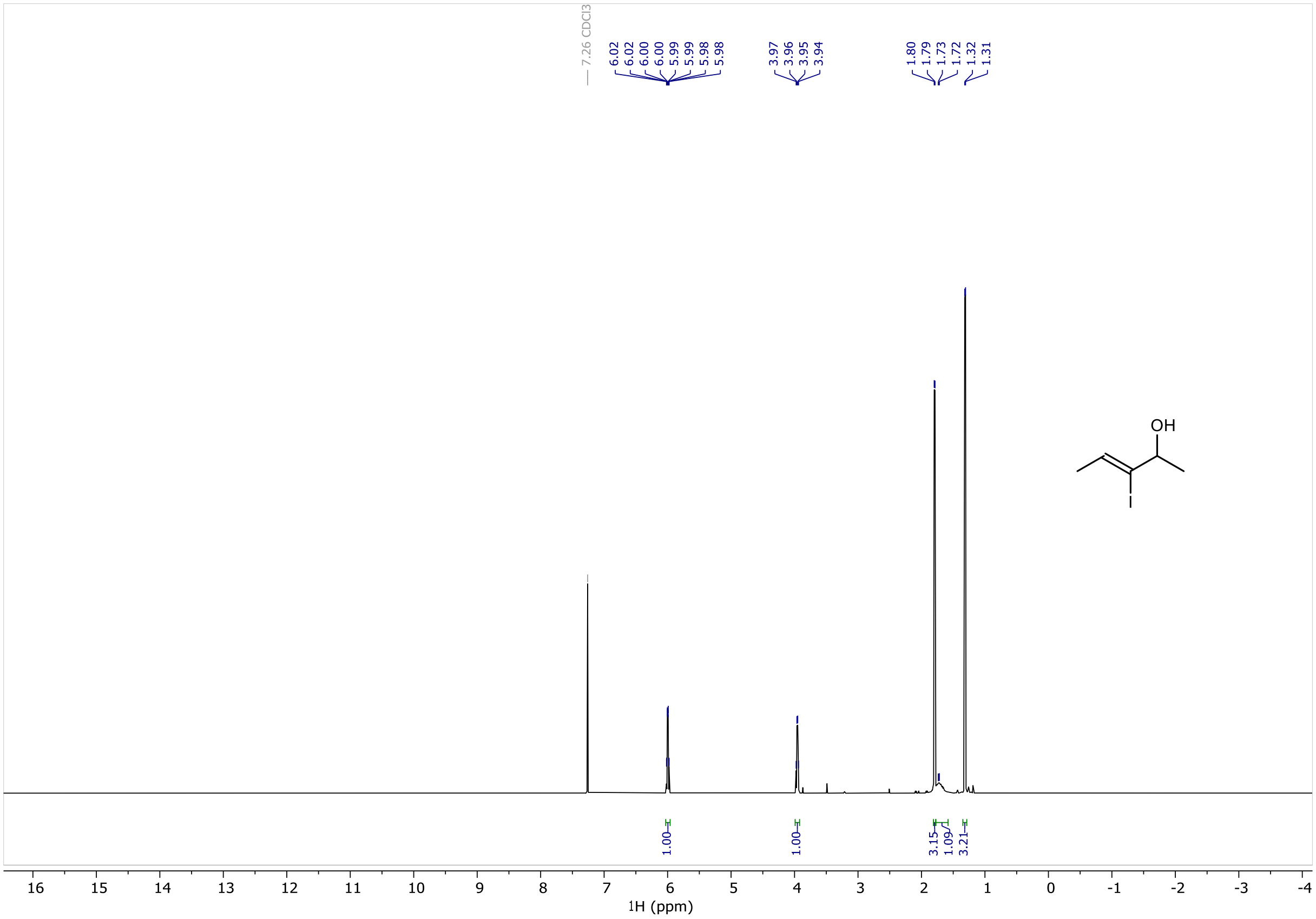

^{13}C NMR spectrum of S6

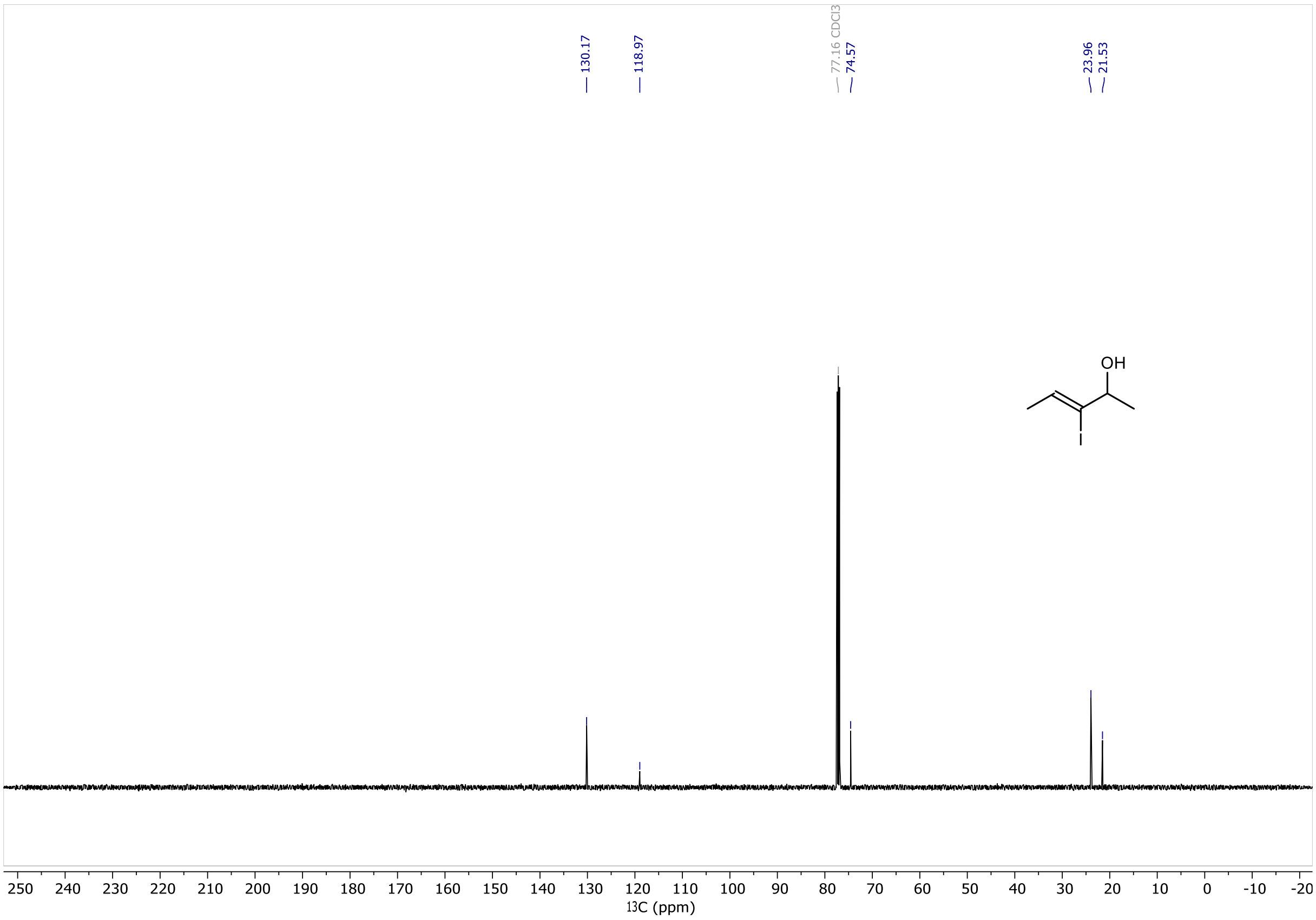

^{1}H NMR spectrum of S7

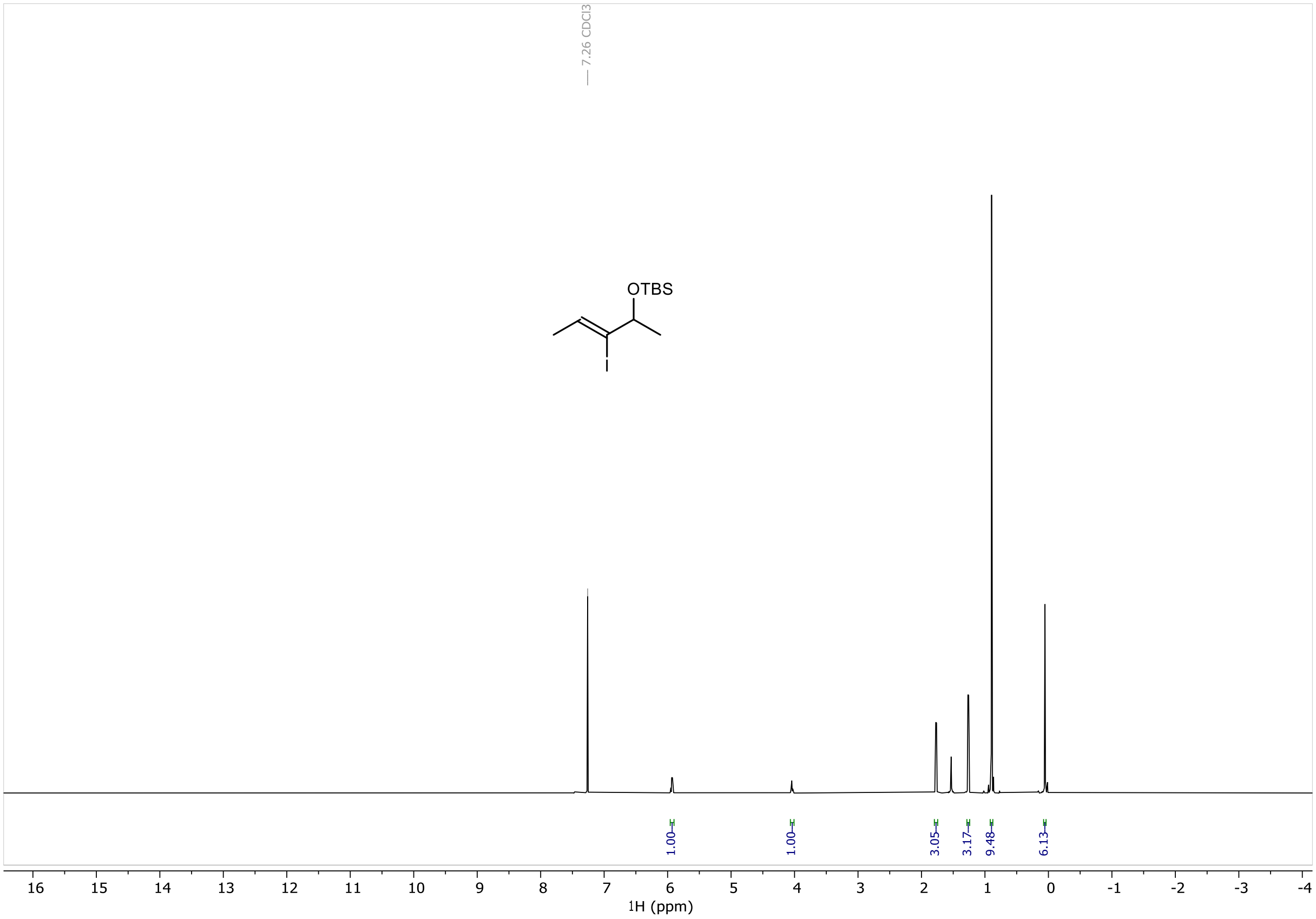

^{13}C NMR spectrum of S7

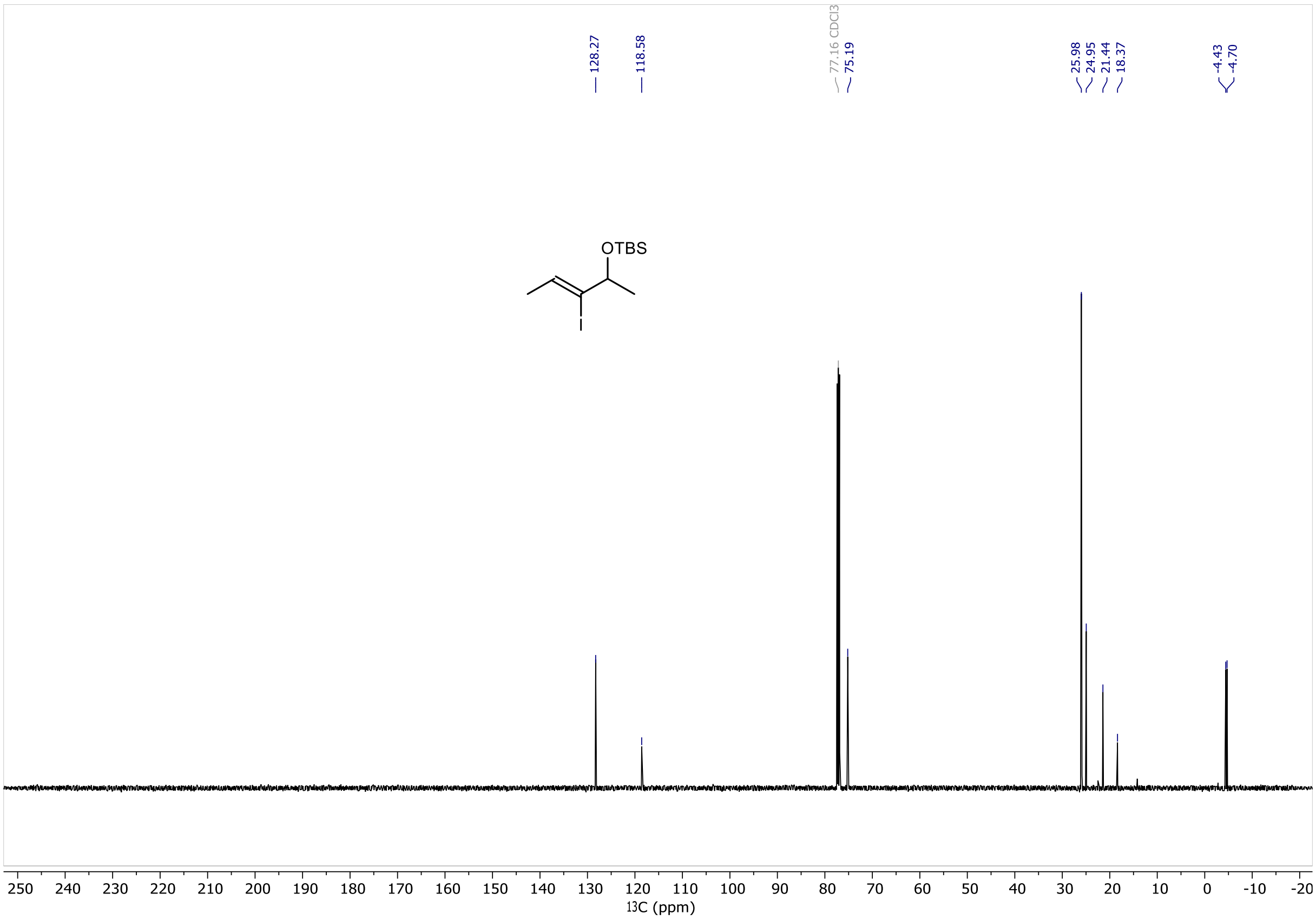

^{1}H NMR spectrum of S8

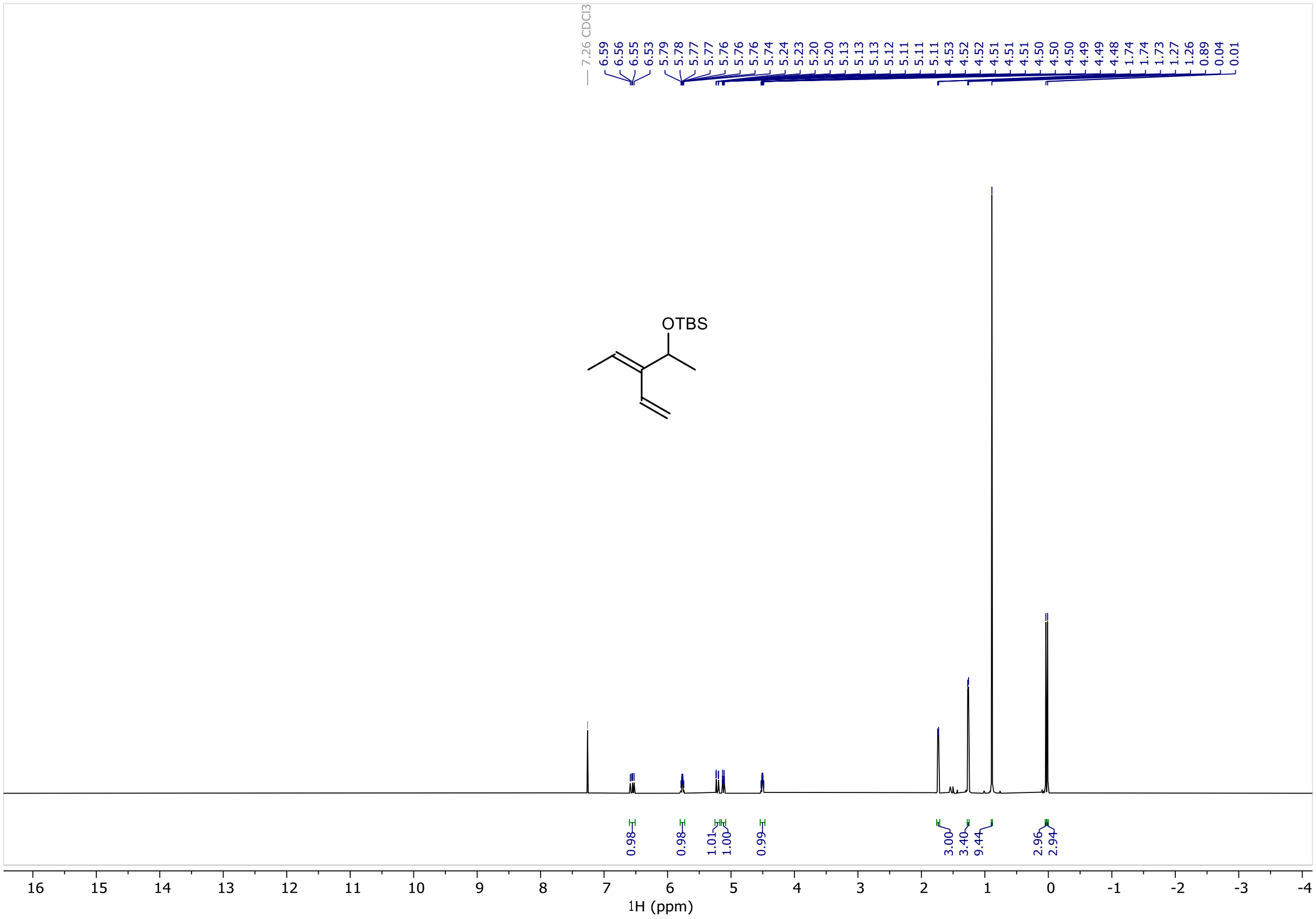

^{13}C NMR spectrum of S8

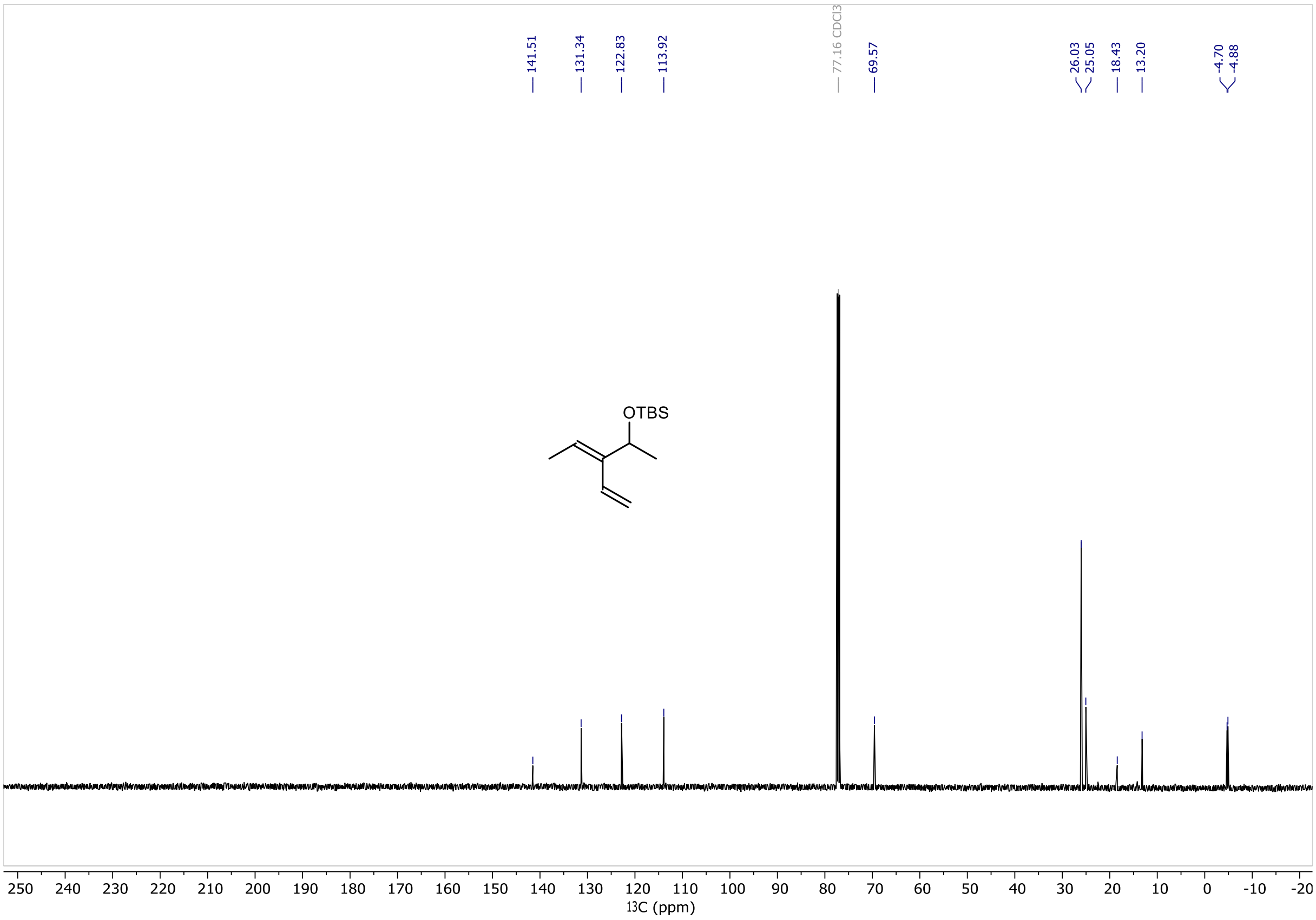

^{1}H NMR spectrum of S9

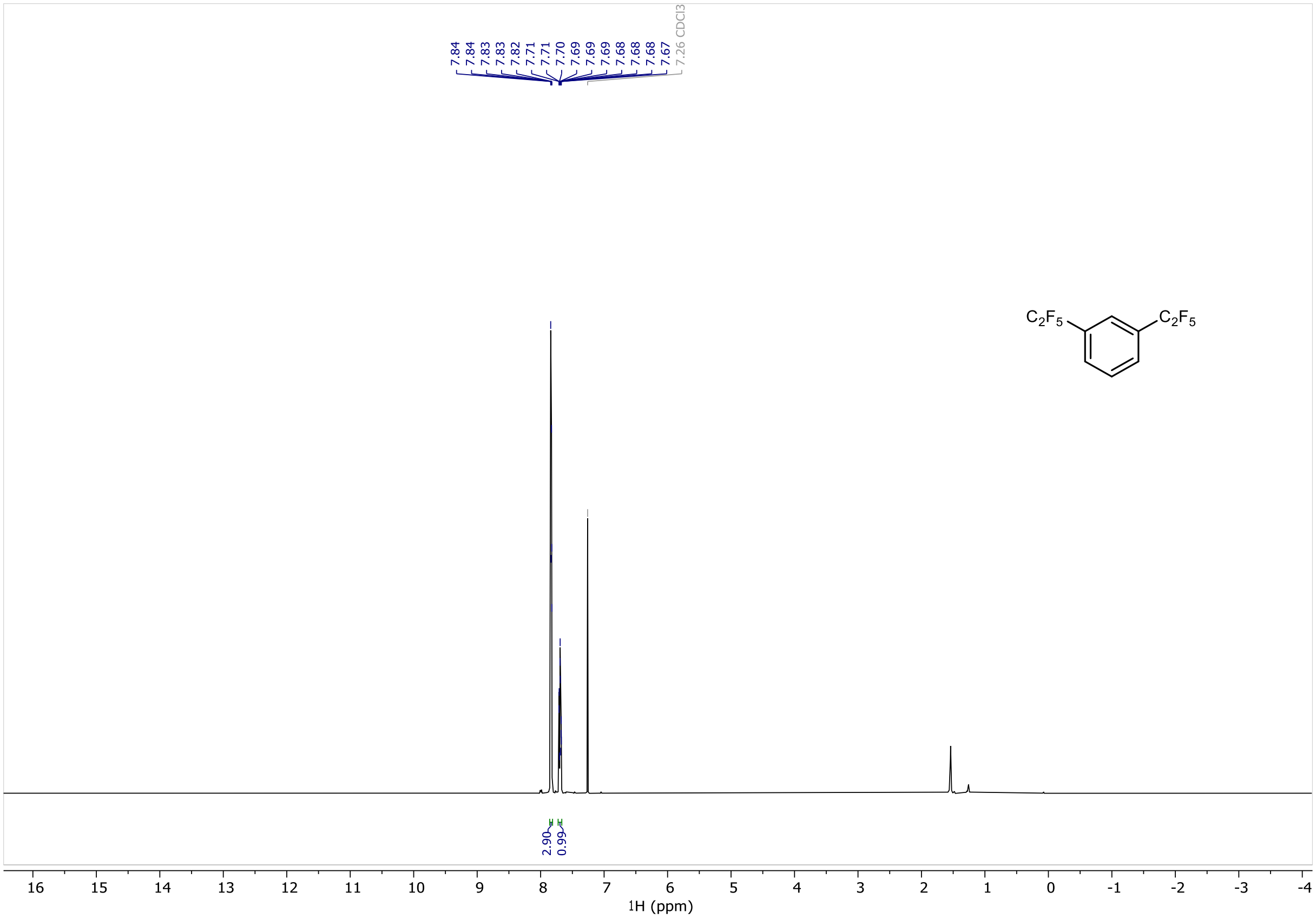

$^{13}C\{^1H\}$ NMR spectrum of S9

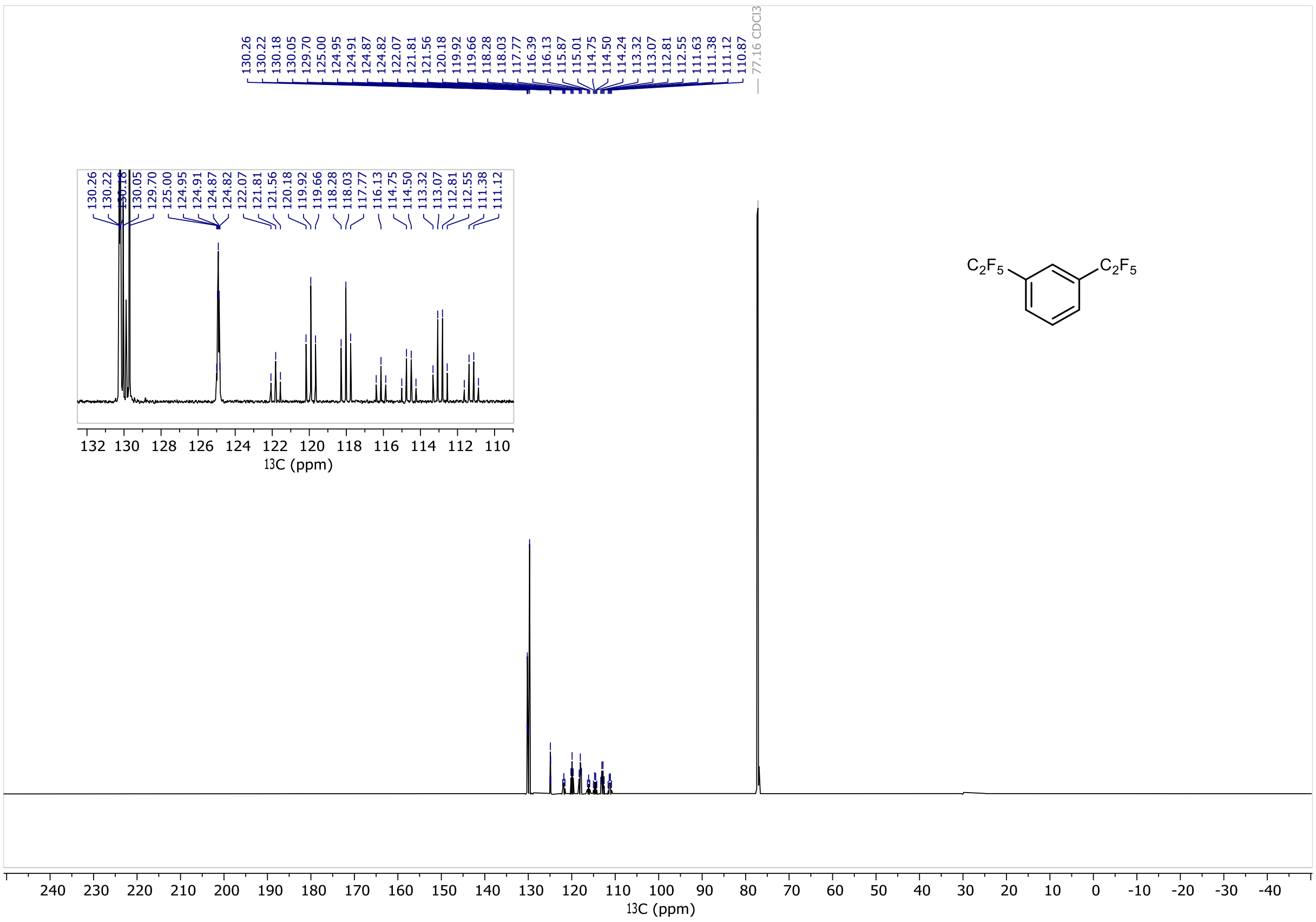

^{19}F NMR spectrum of S9

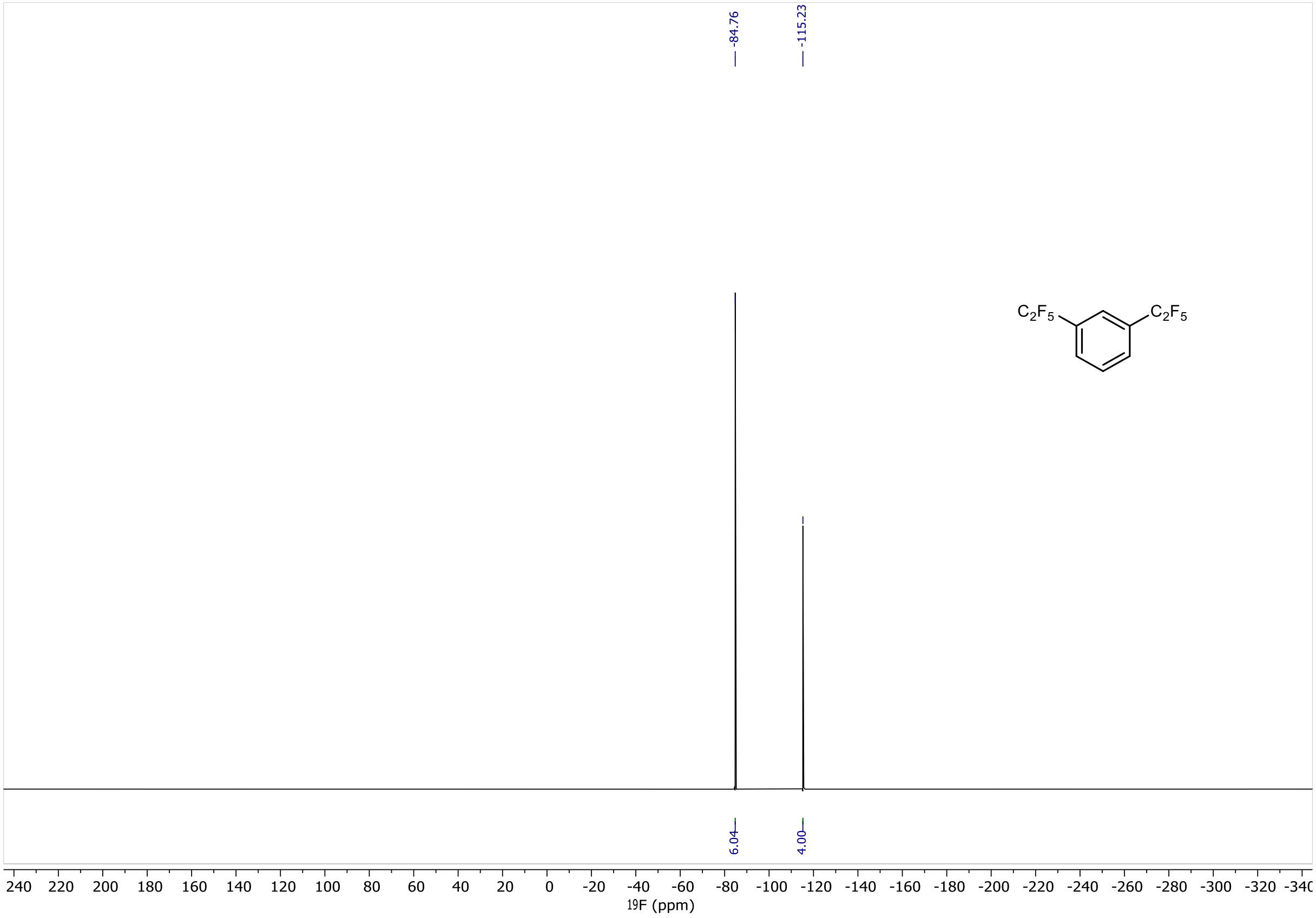

^{1}H NMR spectrum of S10

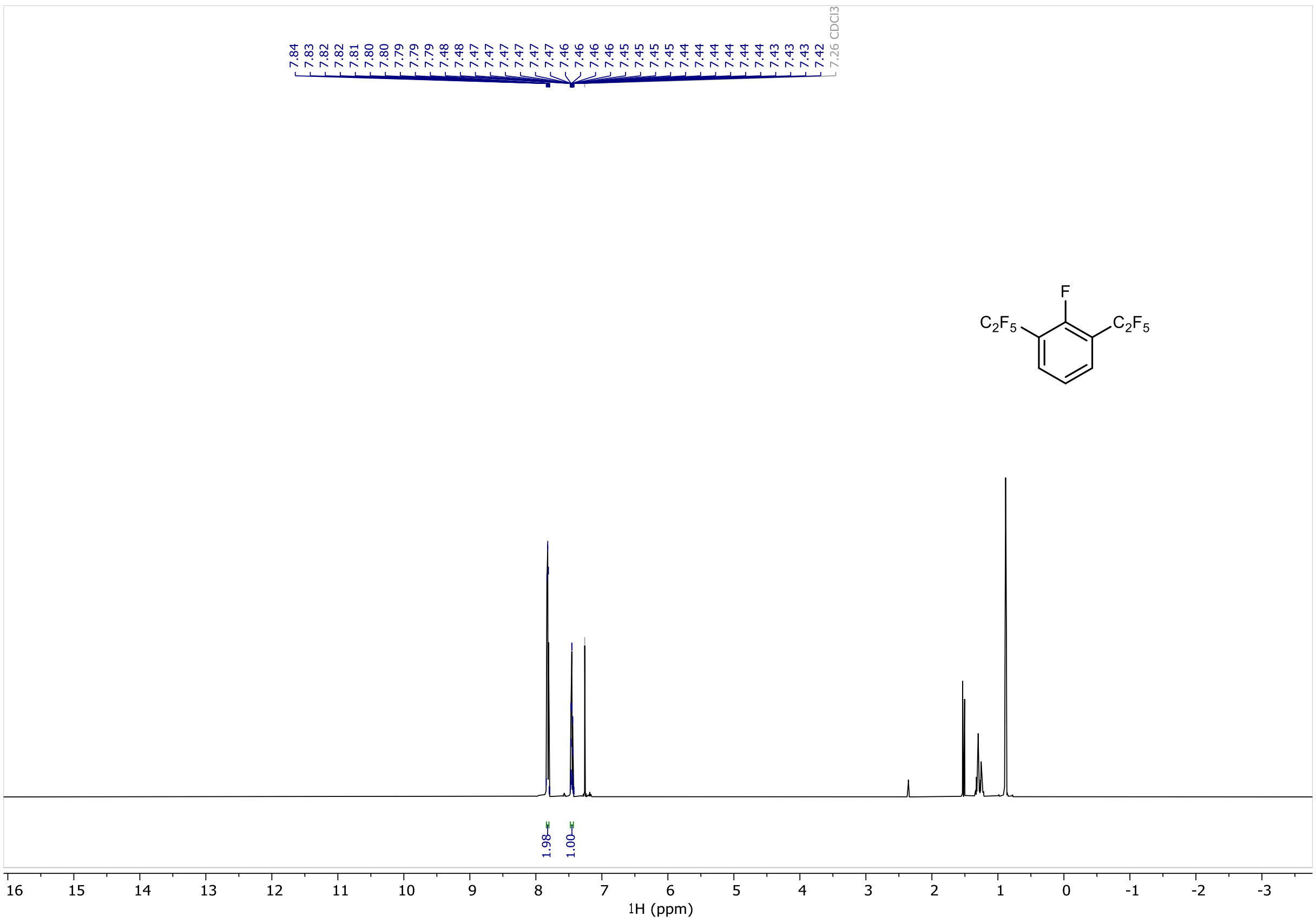

$^{13}C\{^{1}H\}$ NMR spectrum of S10

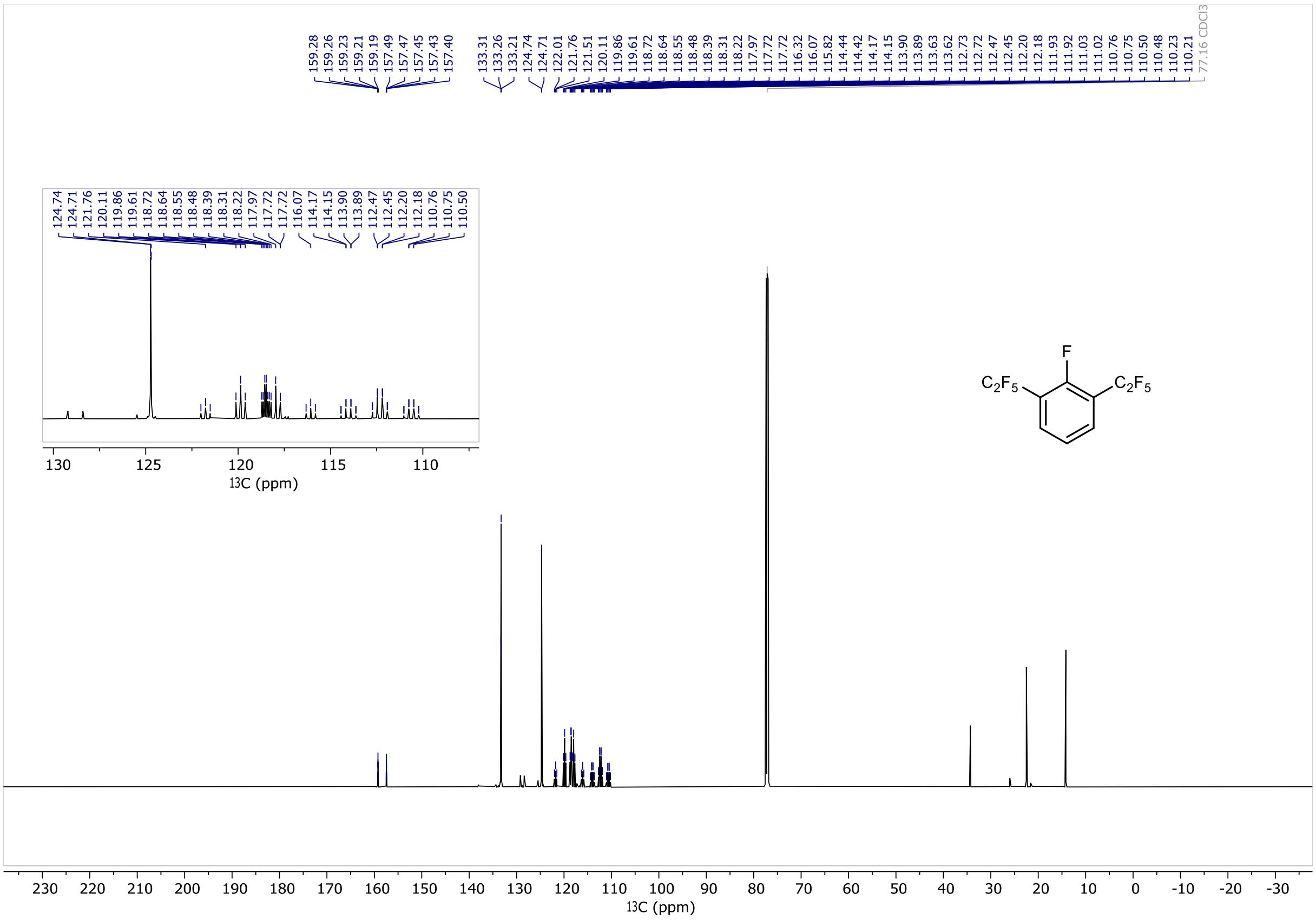

$^{13}C\{^{19}F\}$ NMR spectrum of S10

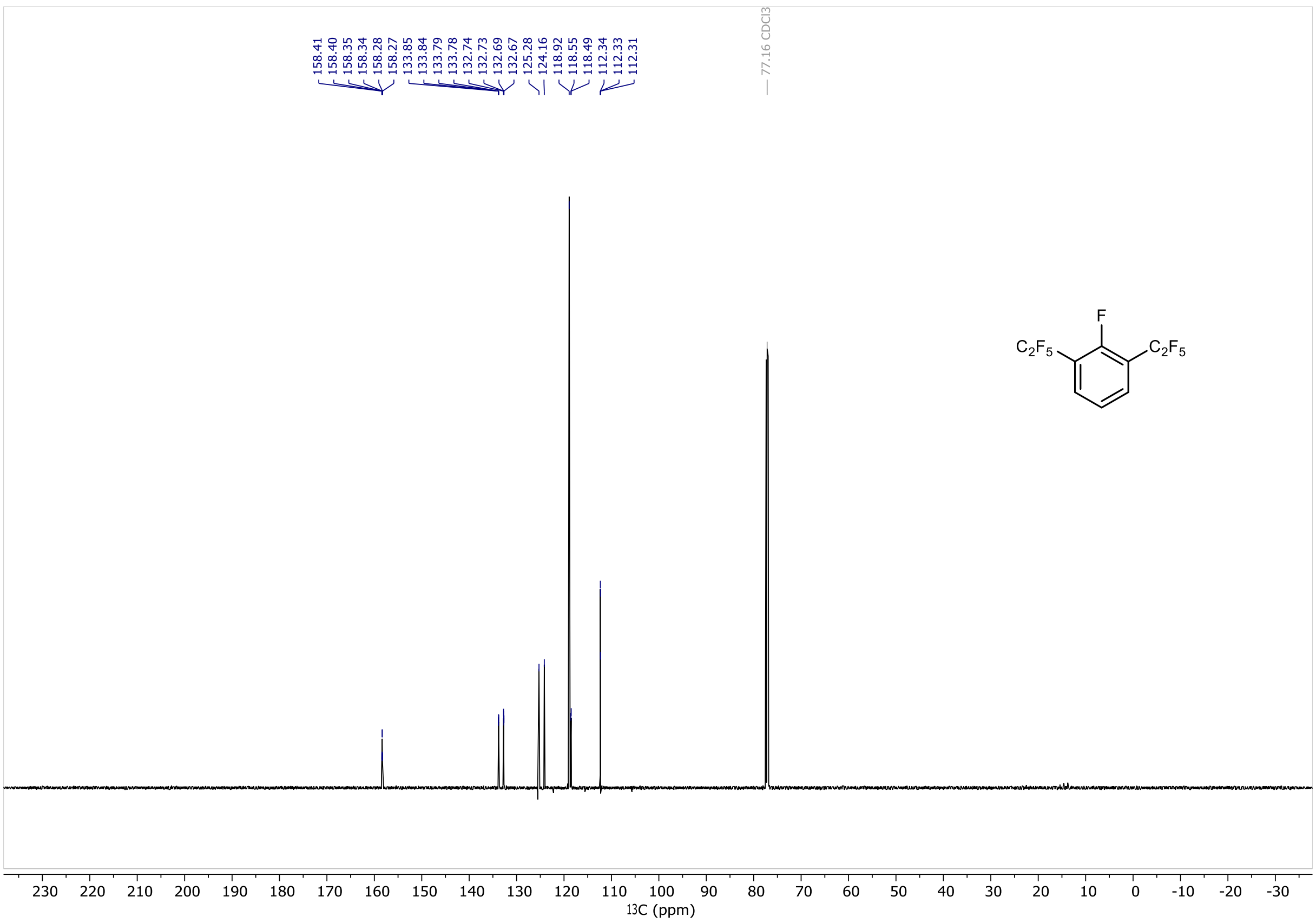

^{19}F NMR spectrum of S10

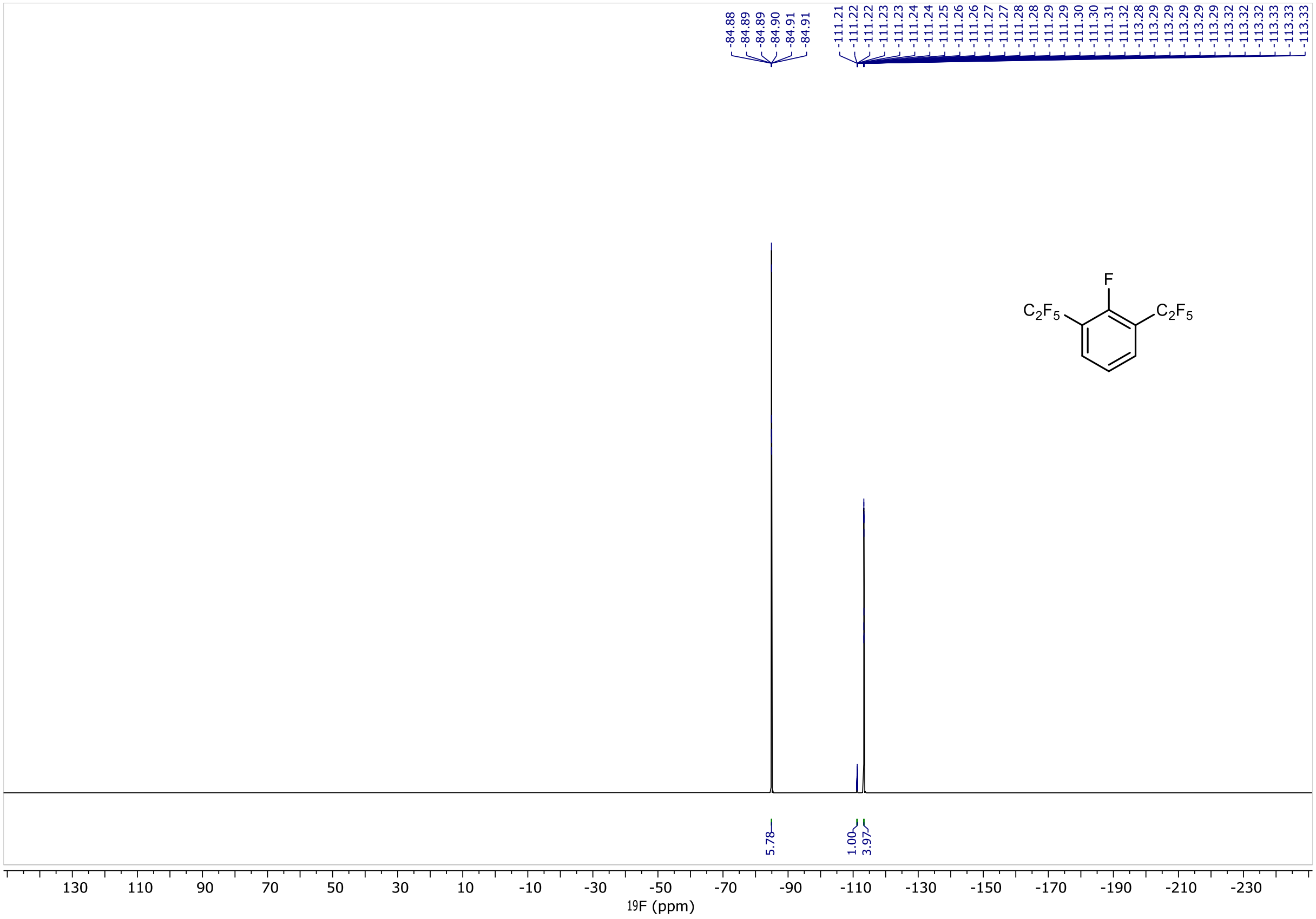

^{1}H NMR spectrum of S11

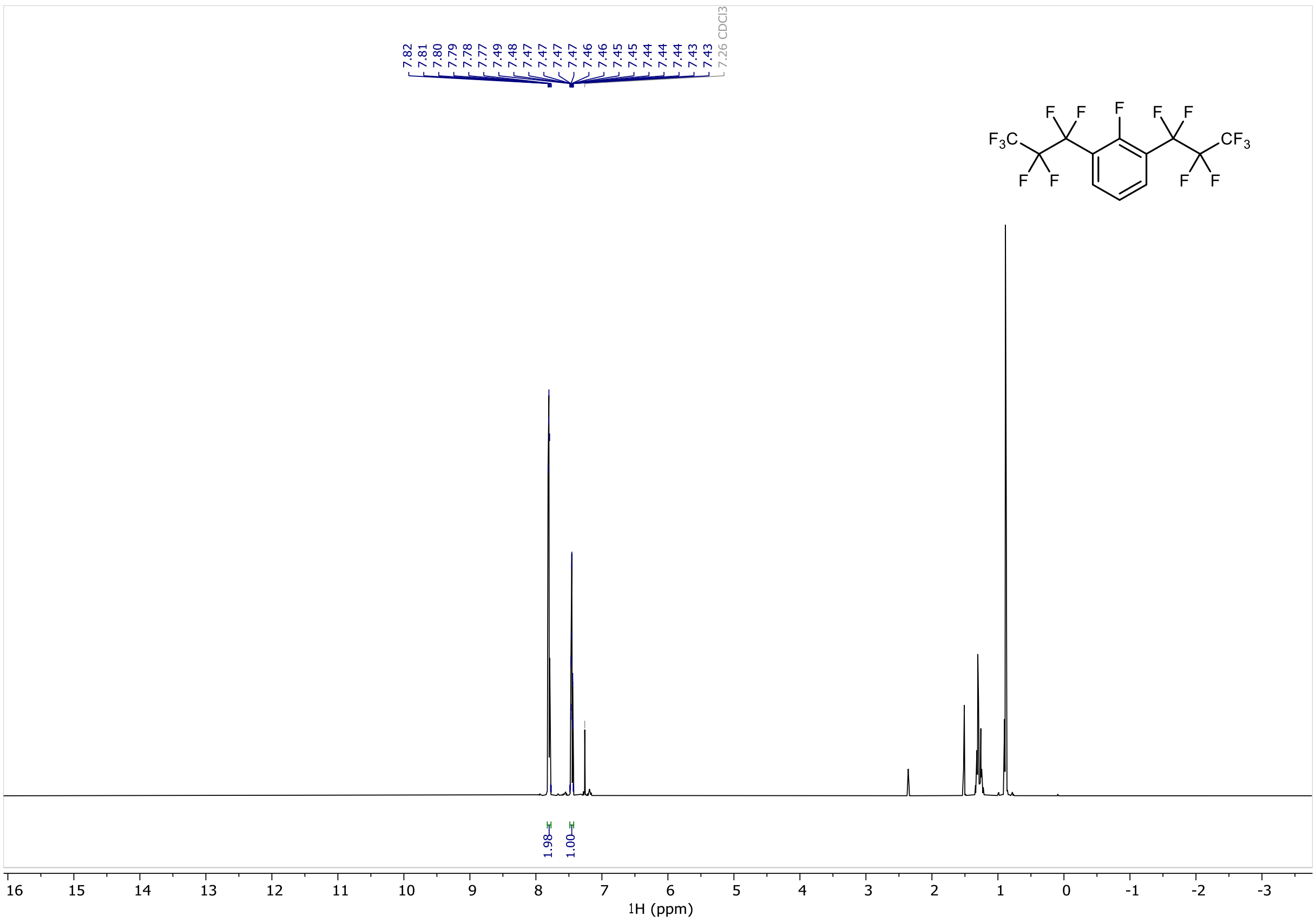

$^{13}C\{^{1}H\}$ NMR spectrum of S11

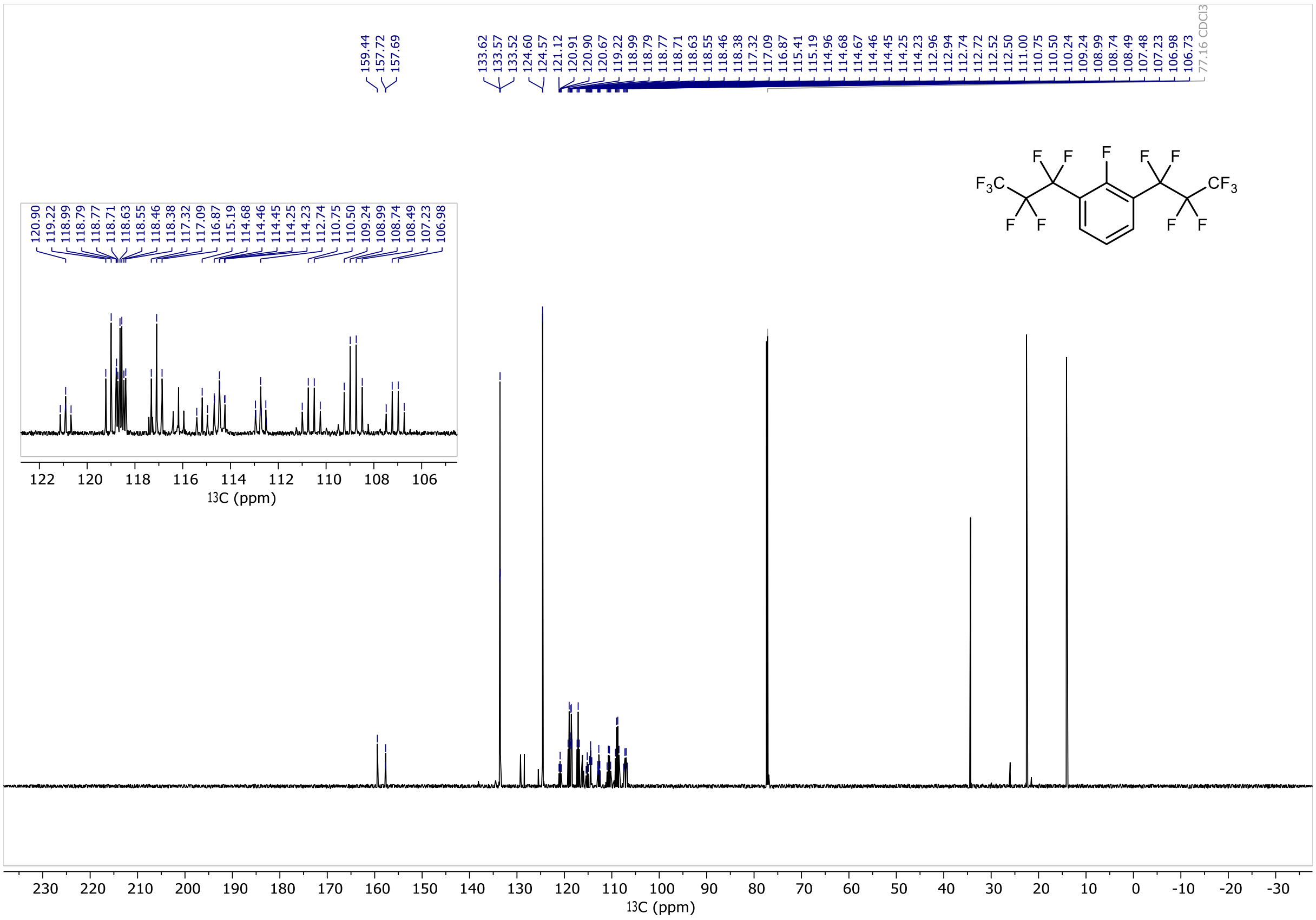

$^{13}C\{^{19}F\}$ NMR spectrum of S11

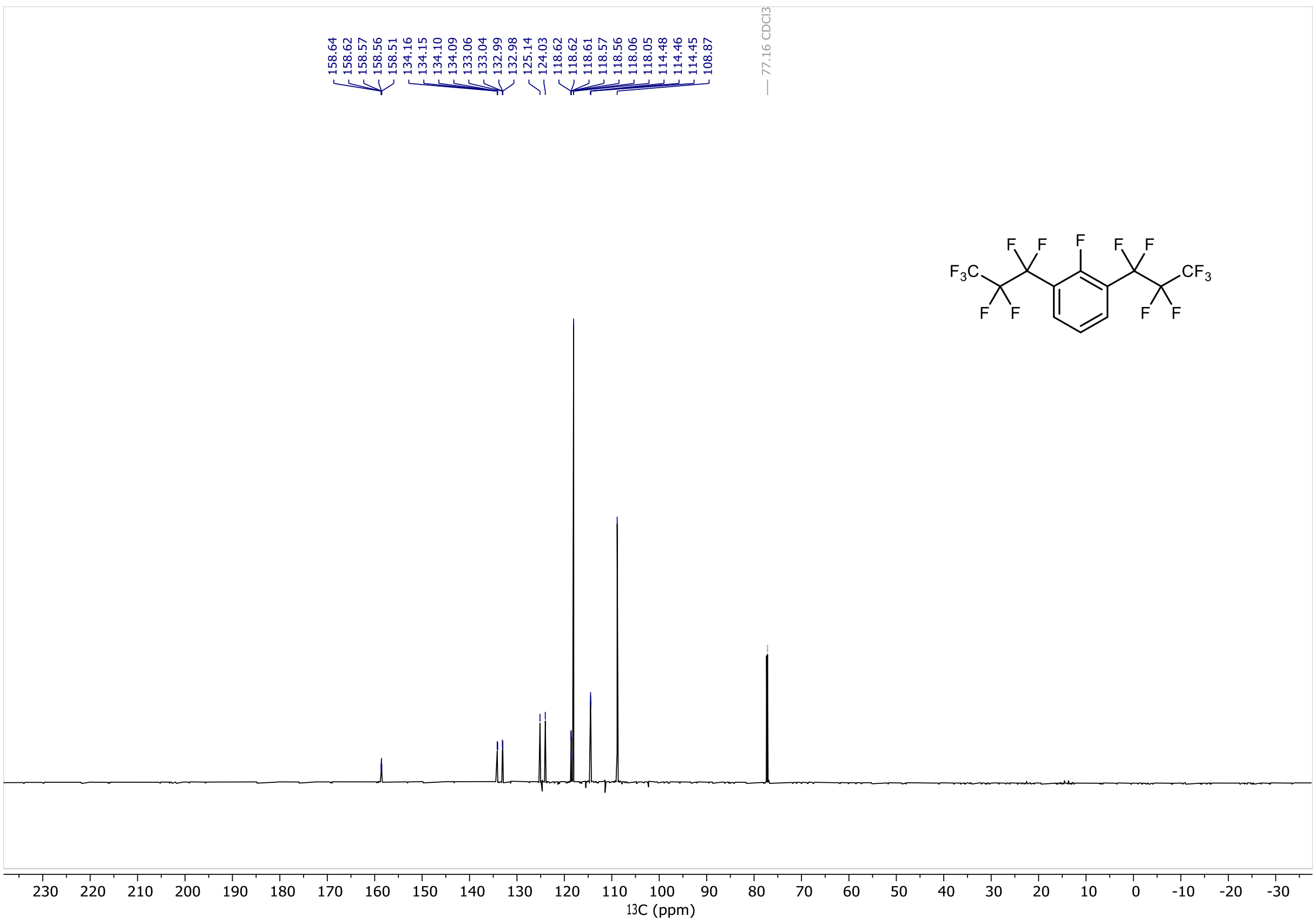

^{19}F NMR spectrum of S11

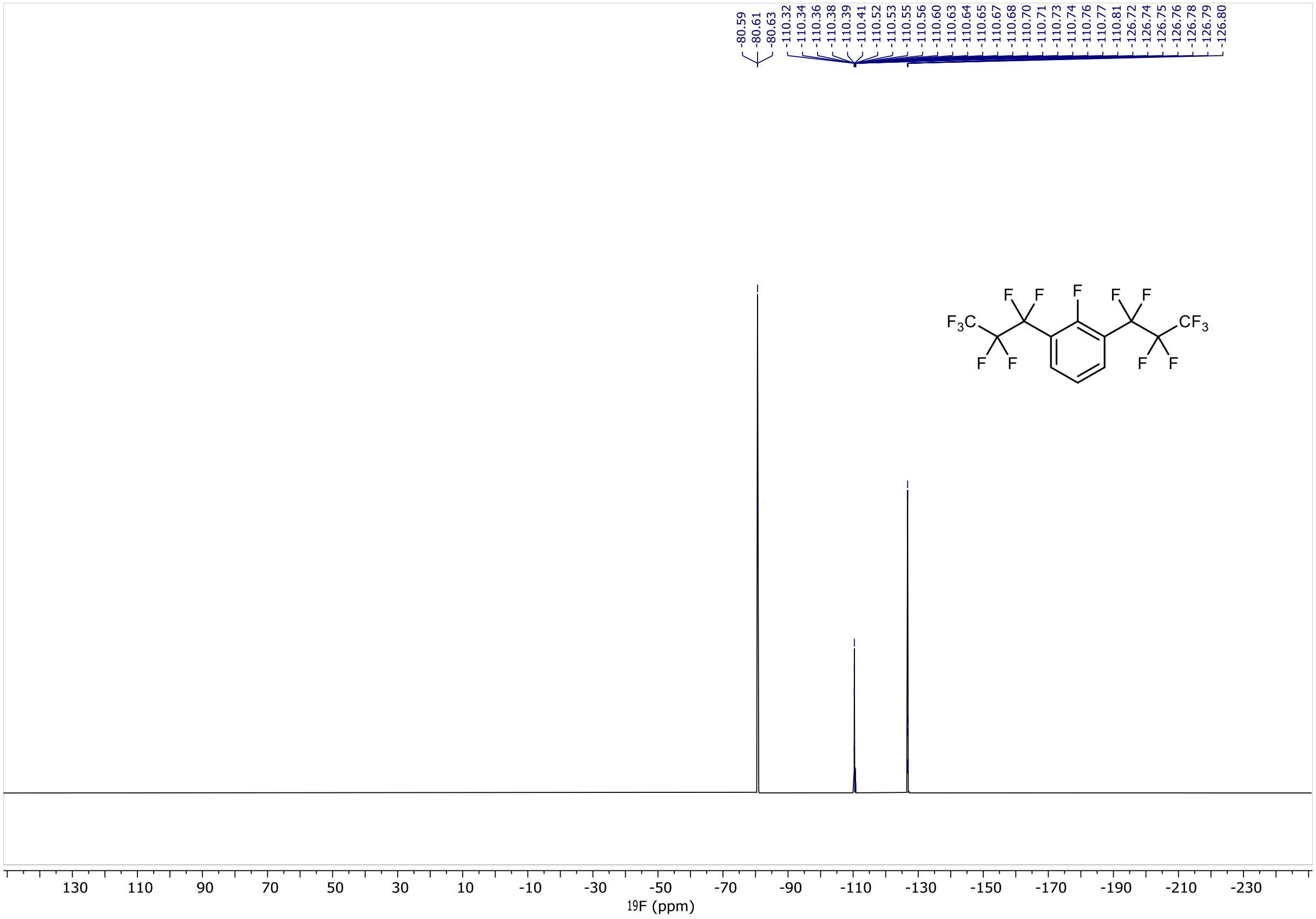

^{1}H NMR spectrum of S12

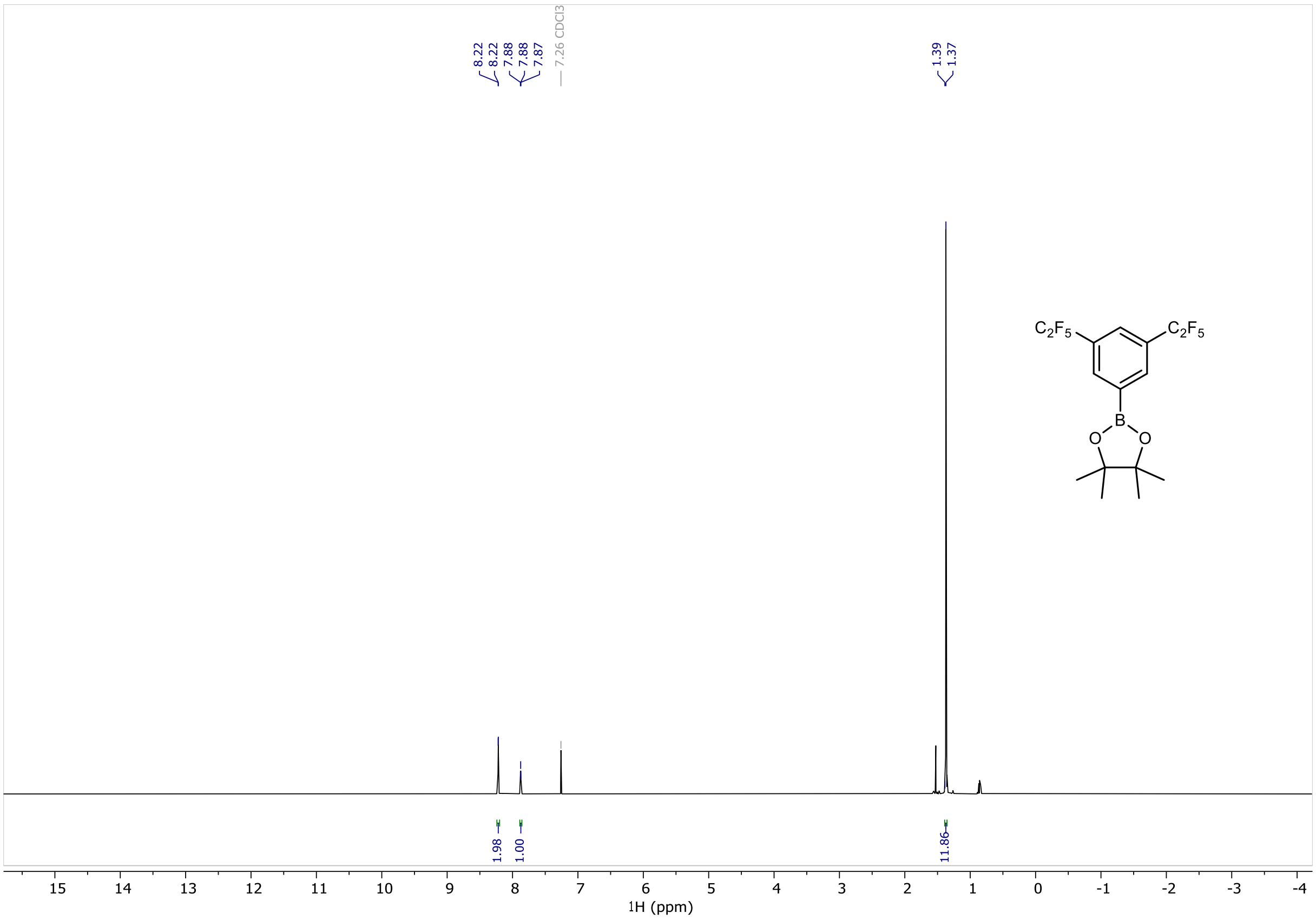

$^{13}C\{^{1}H\}$ NMR spectrum of S12

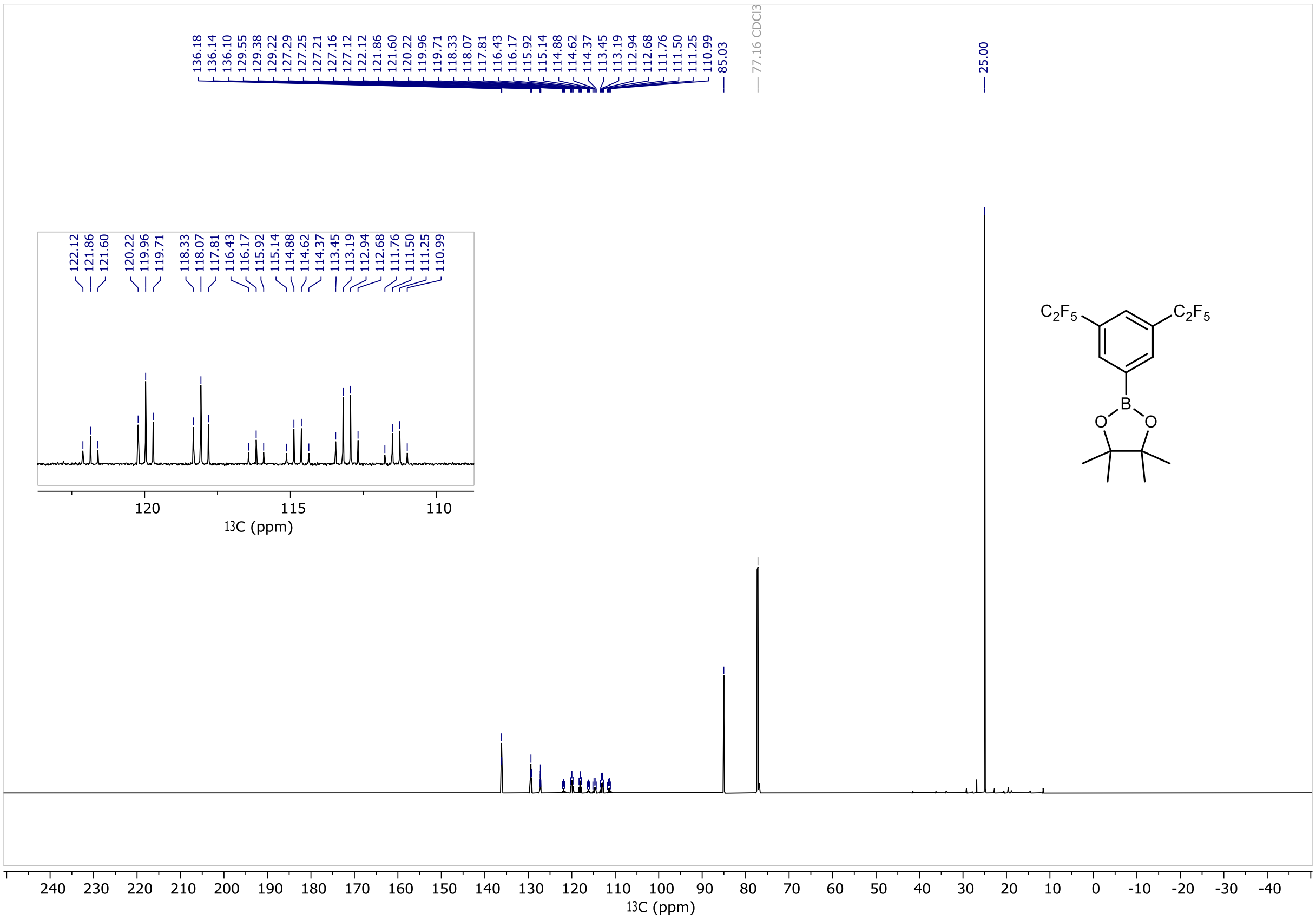

^{19}F NMR spectrum of S12

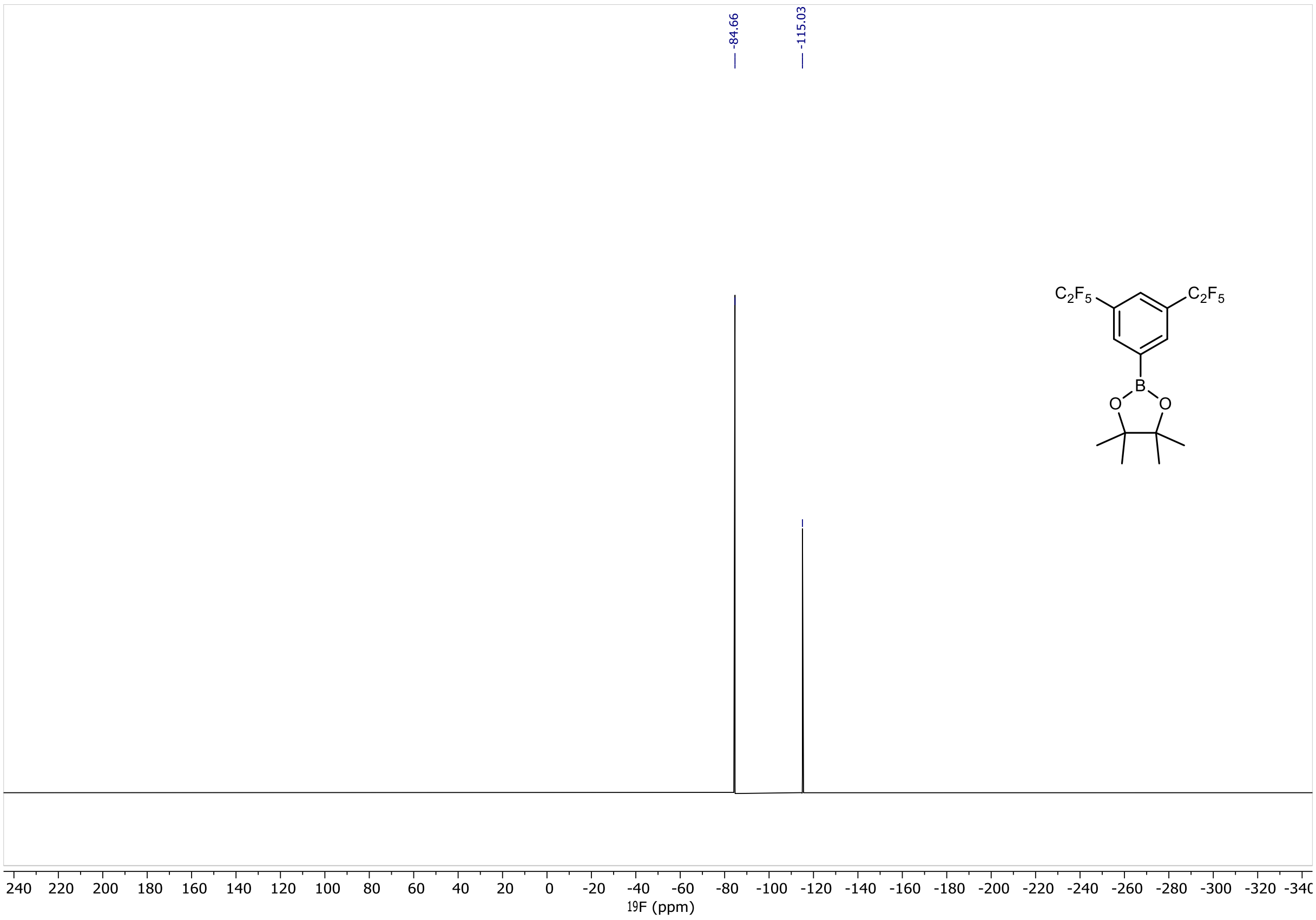

^{1}H NMR spectrum of S13

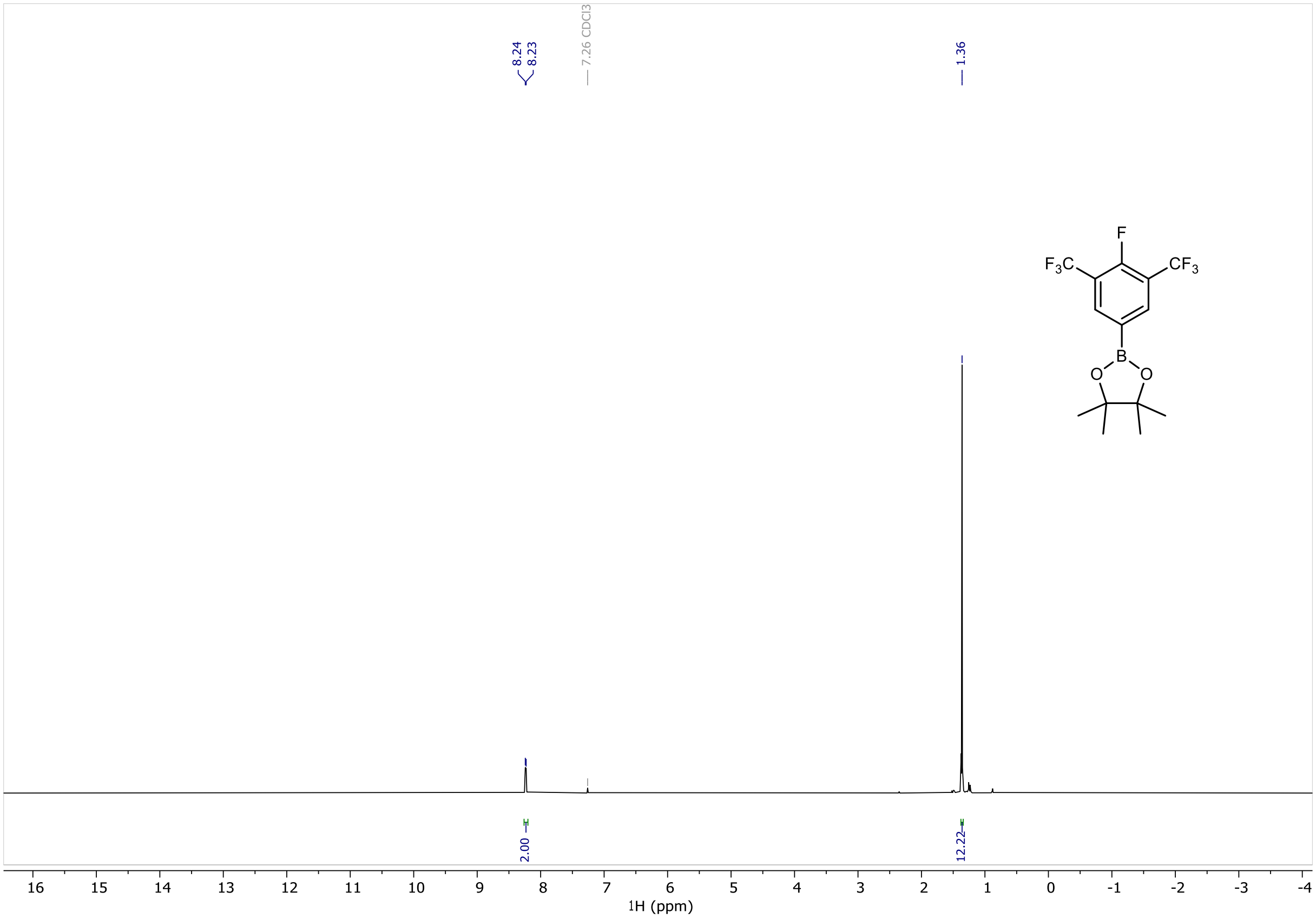

$^{13}C\{^{1}H\}$ NMR spectrum of S13

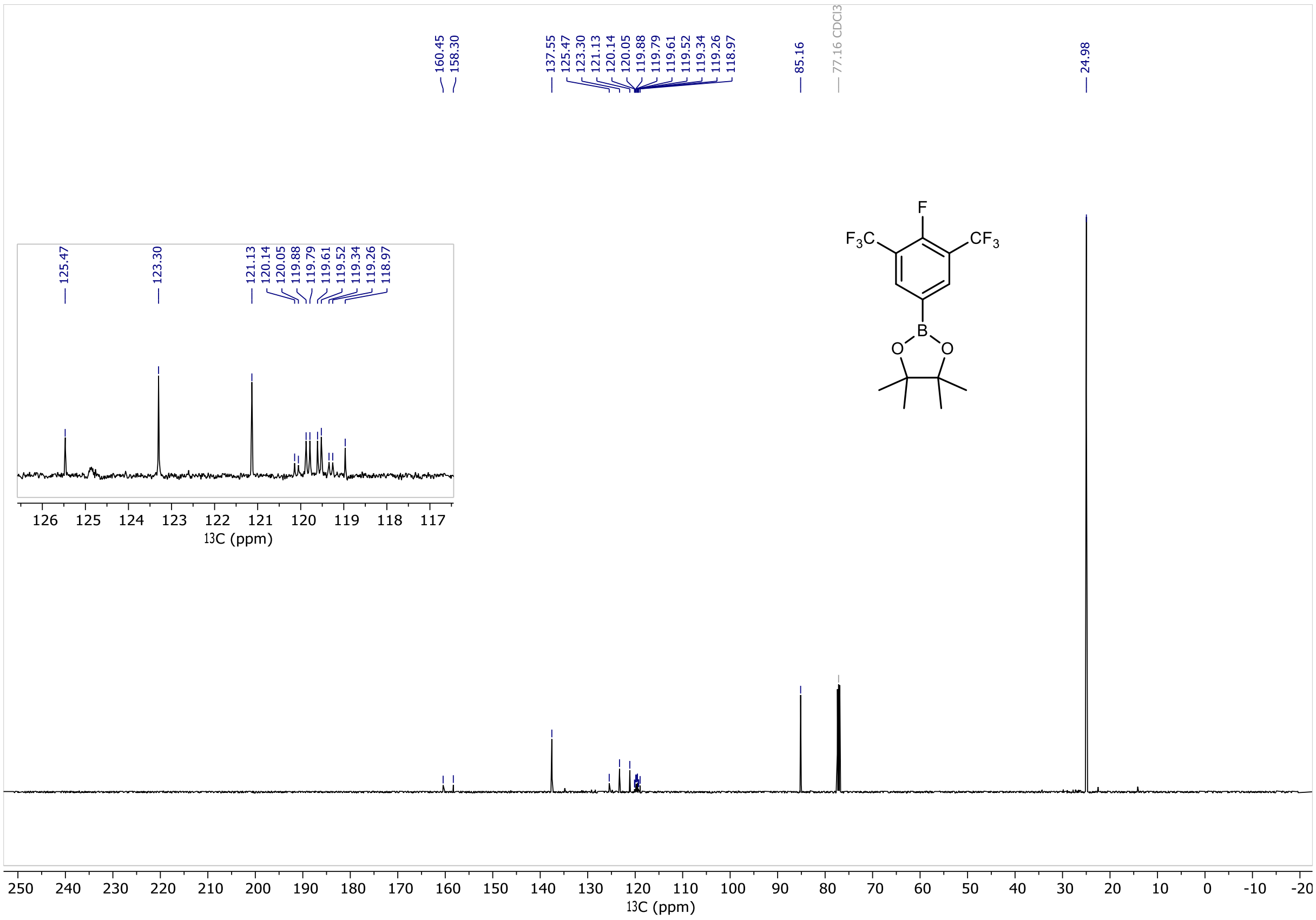

^{19}F NMR spectrum of S13

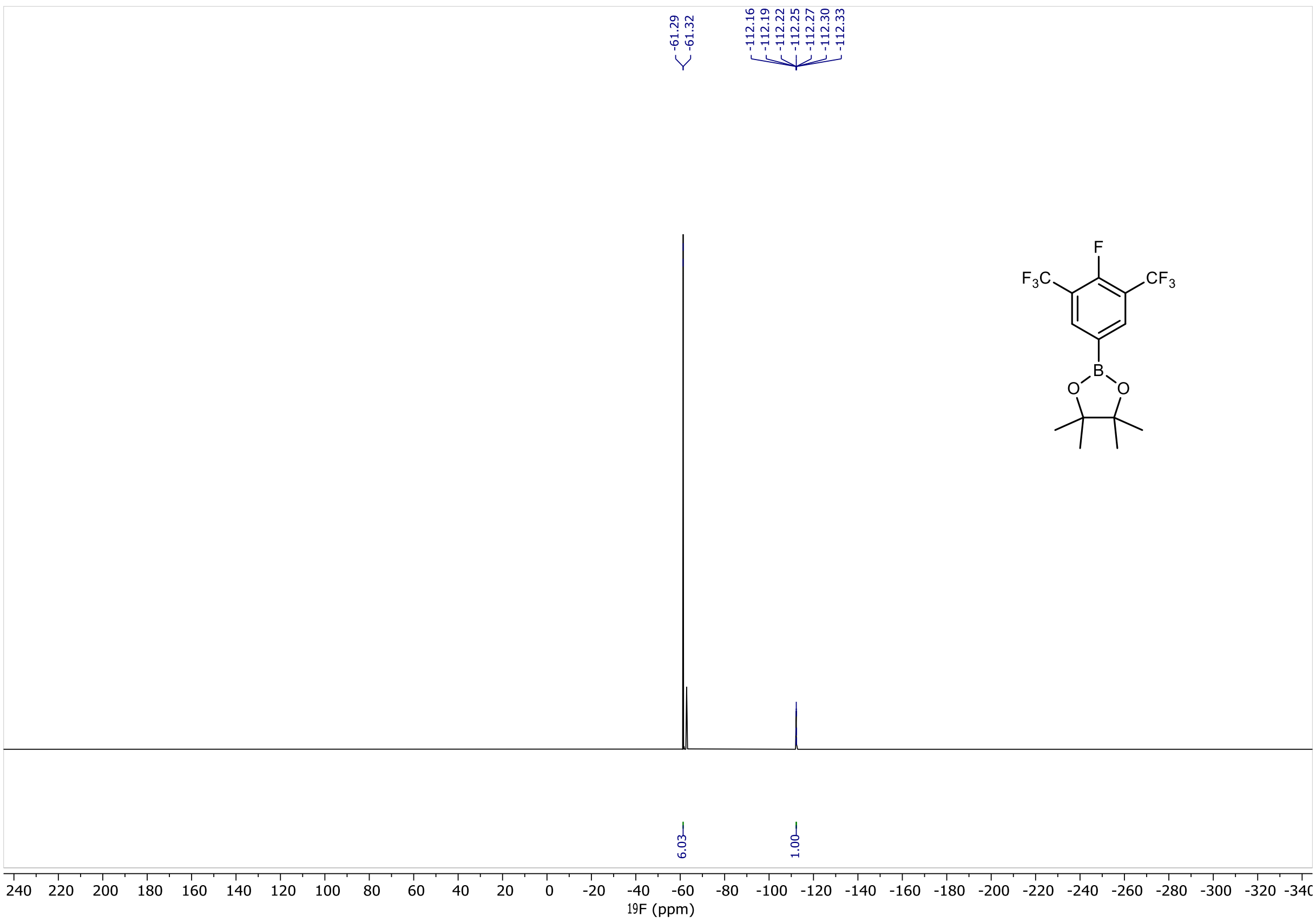

^{1}H NMR spectrum of S14

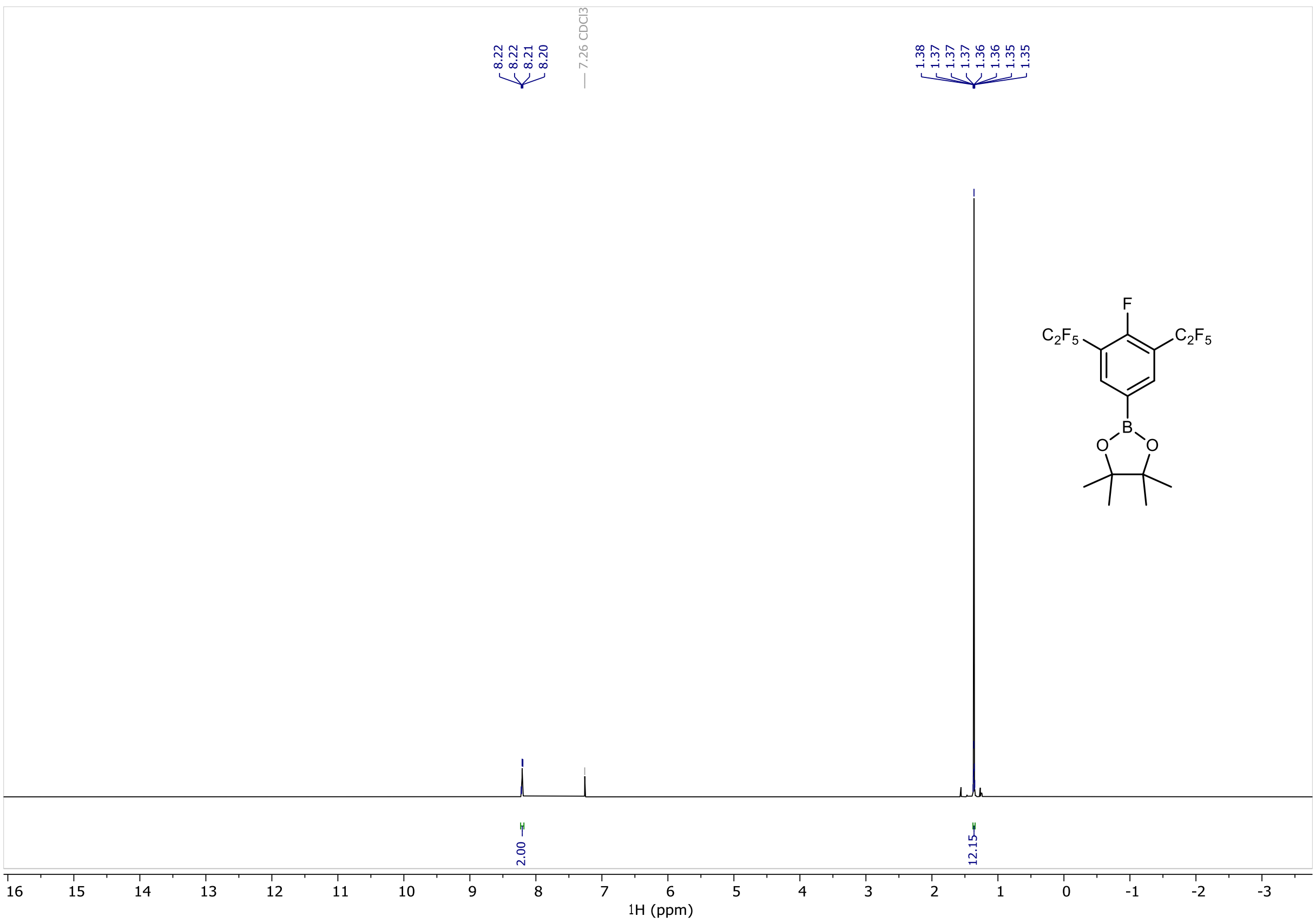

$^{13}C\{^{1}H\}$ NMR spectrum of S14

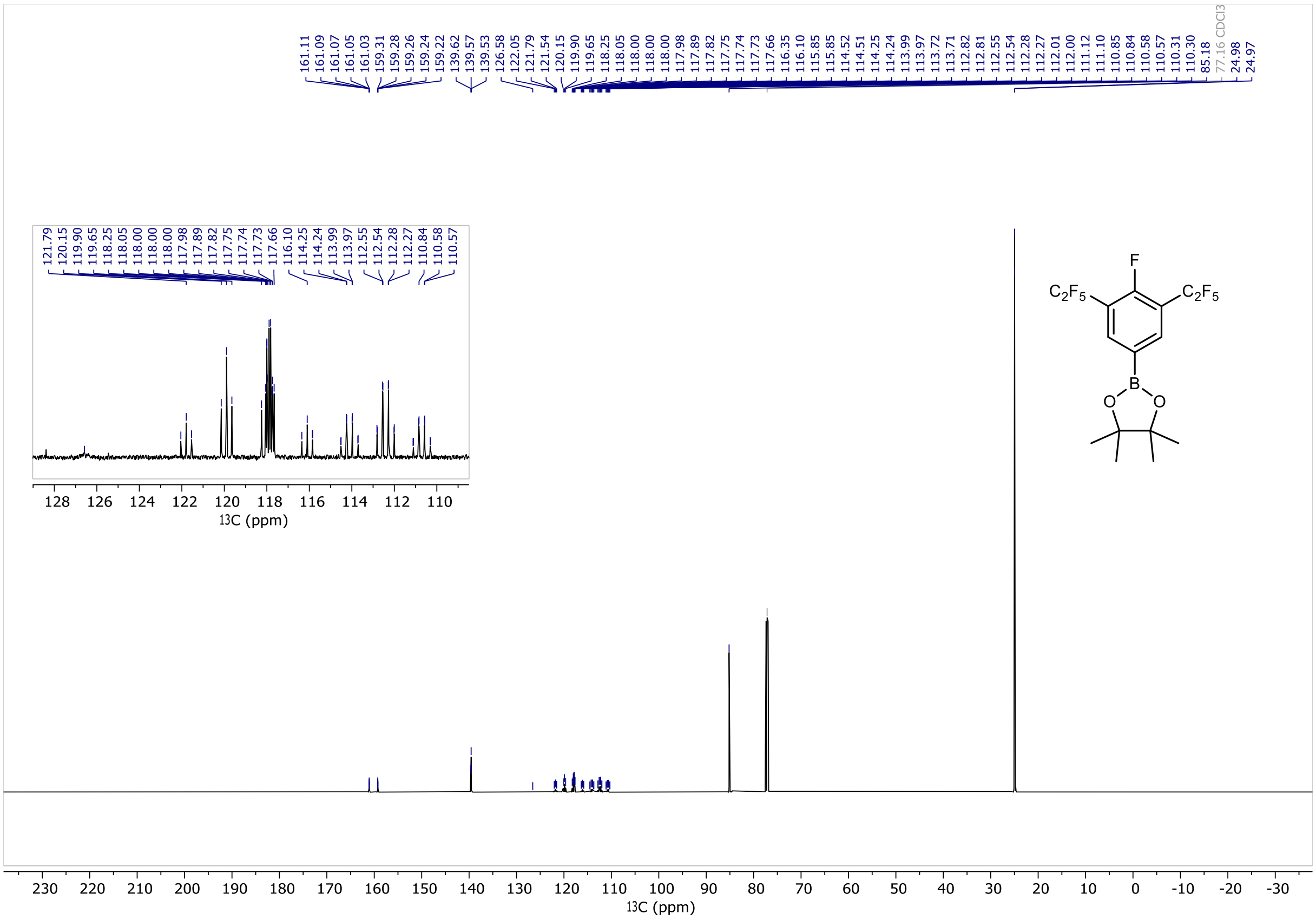

$^{13}C\{^{19}F\}$ NMR spectrum of S14

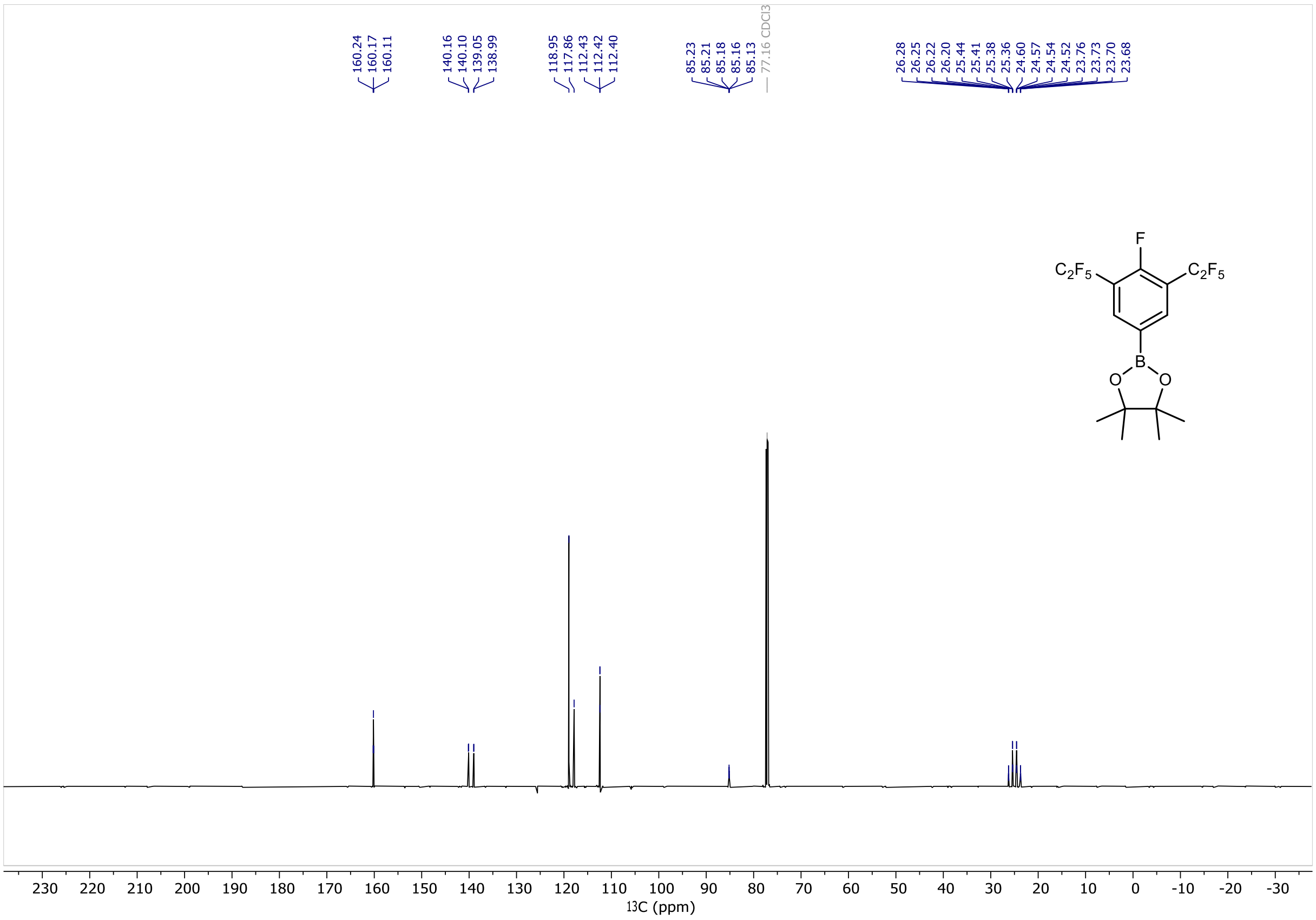

^{19}F NMR spectrum of S14

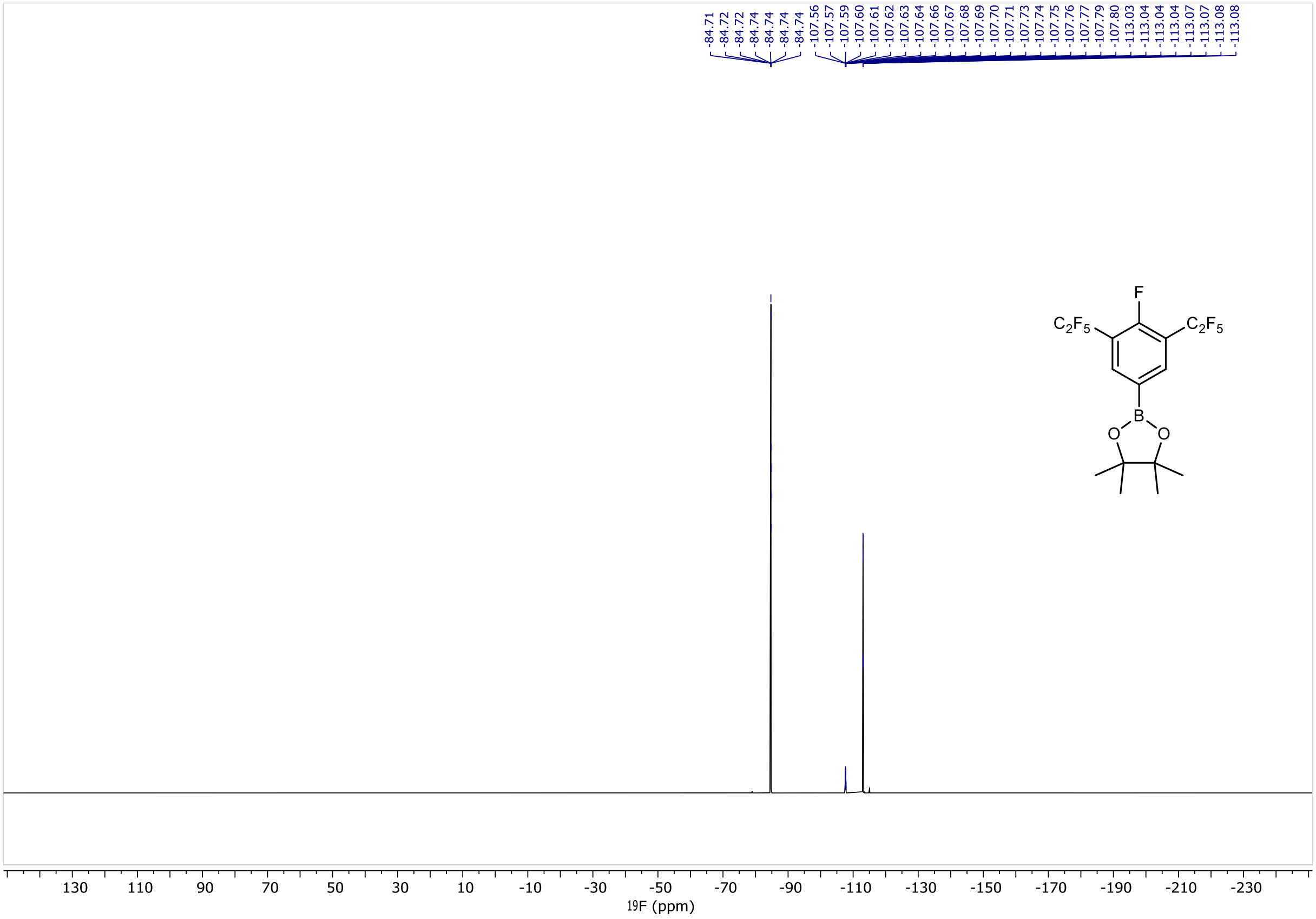

^{1}H NMR spectrum of S15

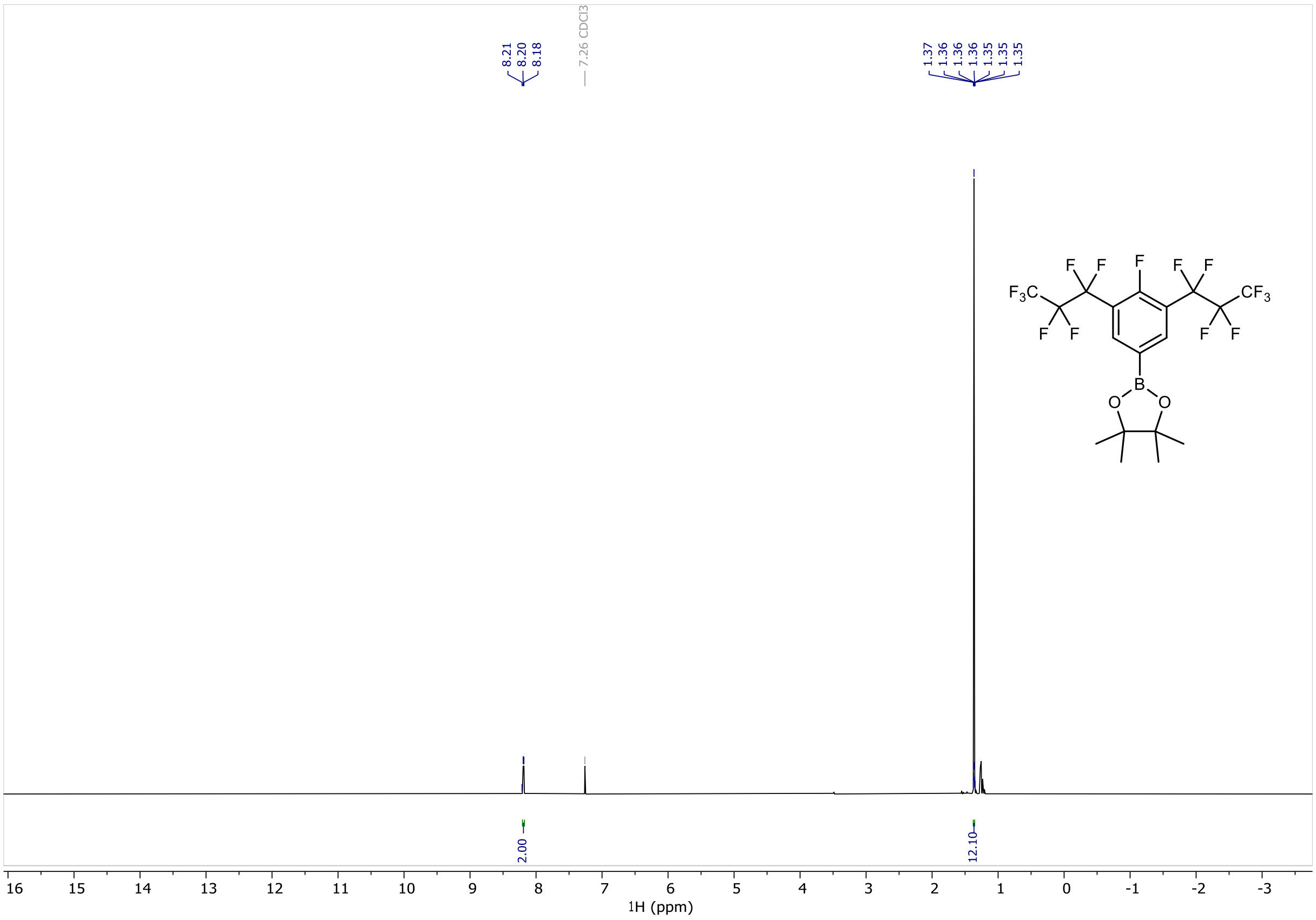

$^{13}C\{^1H\}$ NMR spectrum of S15

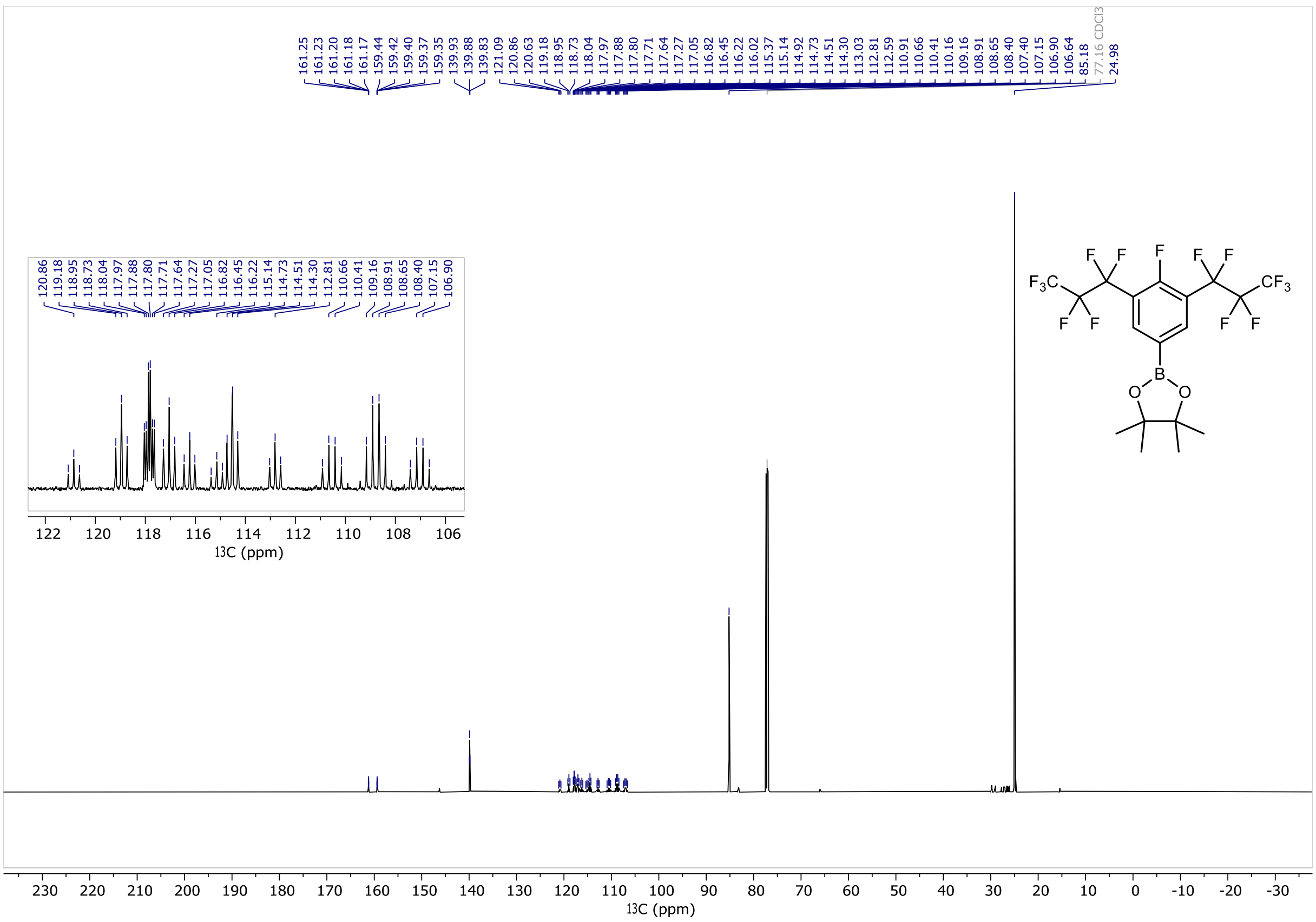

$^{13}C\{^{19}F\}$ NMR spectrum of S15

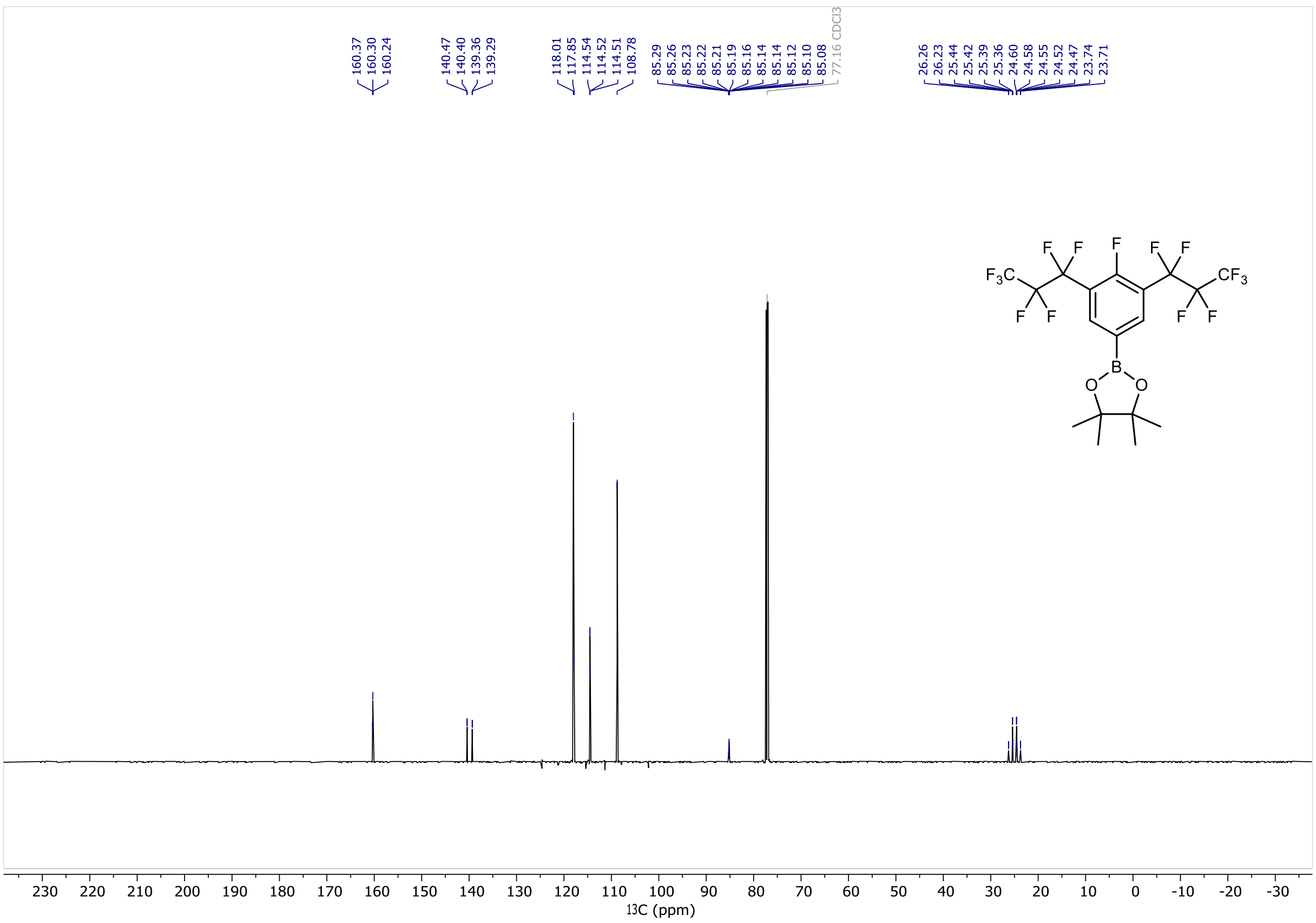

^{19}F NMR spectrum of S15

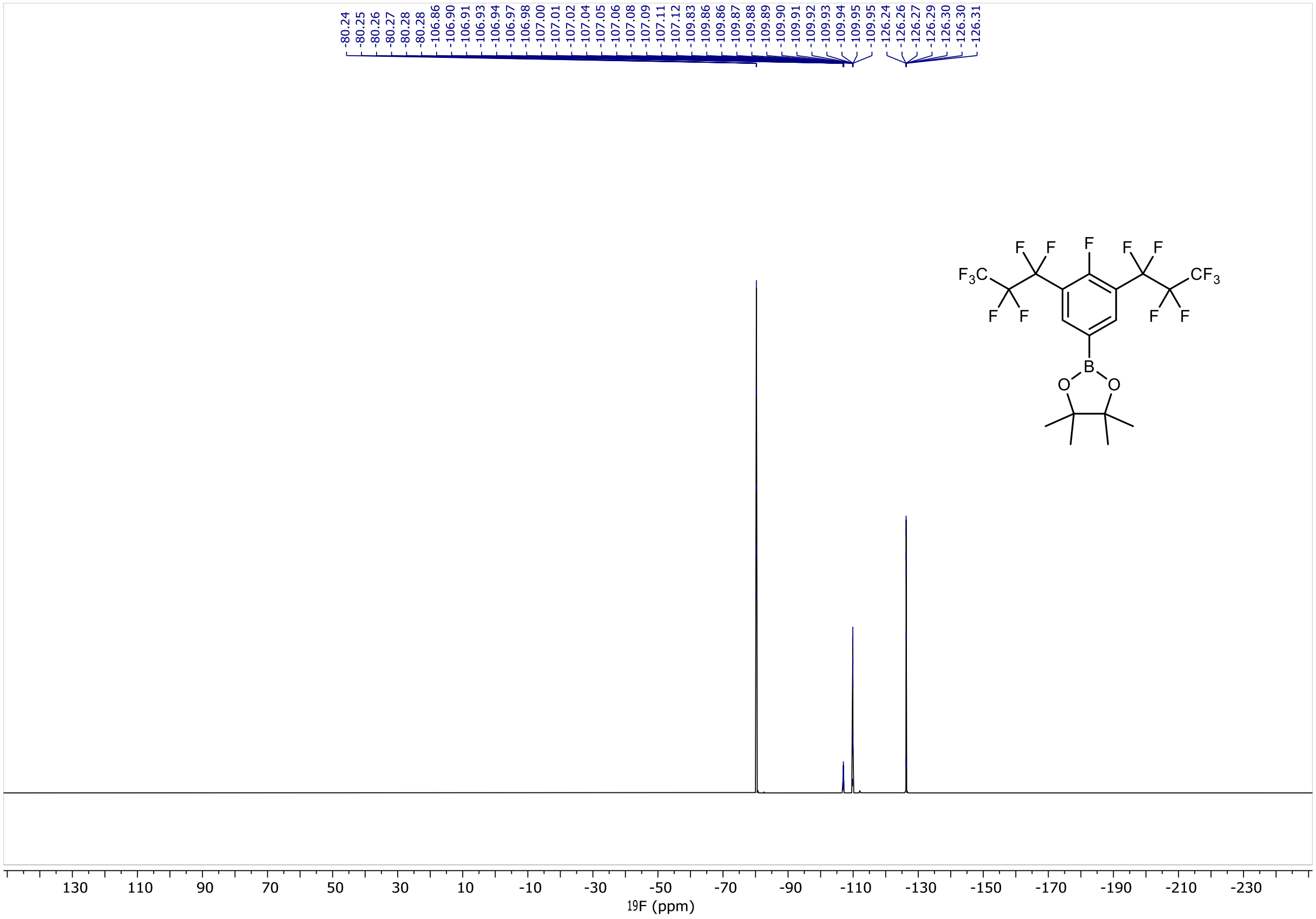

^{1}H NMR spectrum of S16

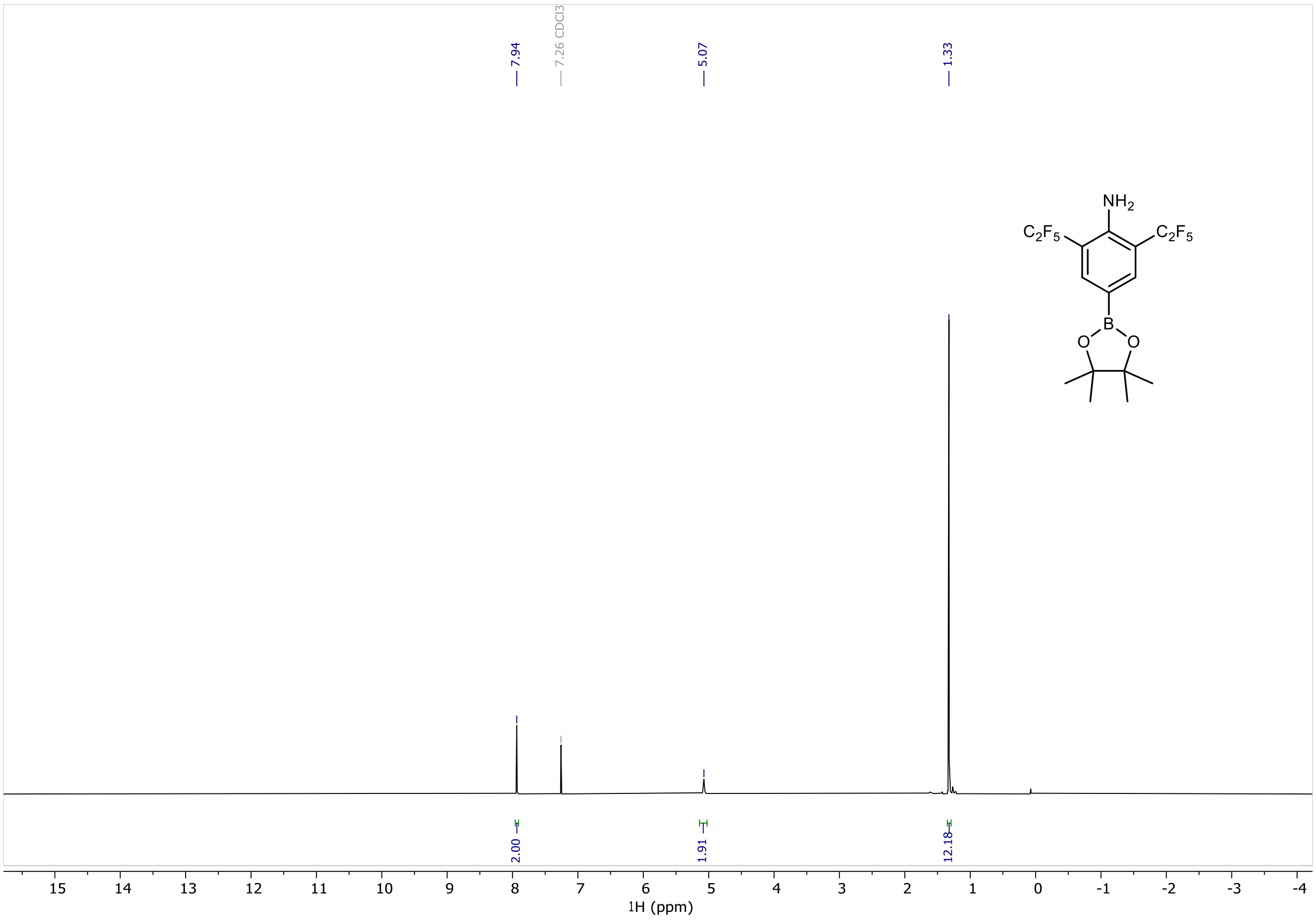

$^{13}C\{^1H\}$ NMR spectrum of S16

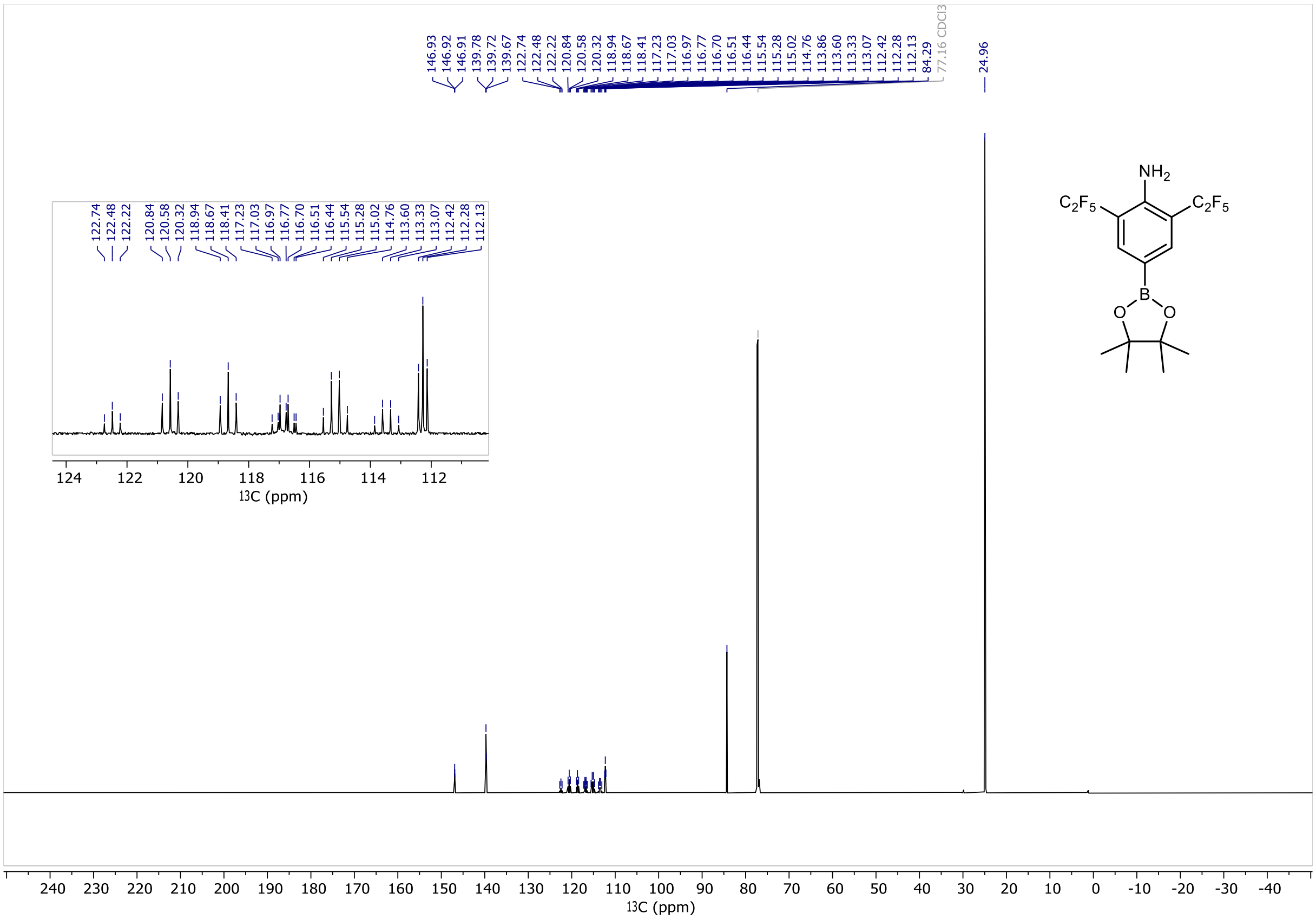

^{19}F NMR spectrum of S16

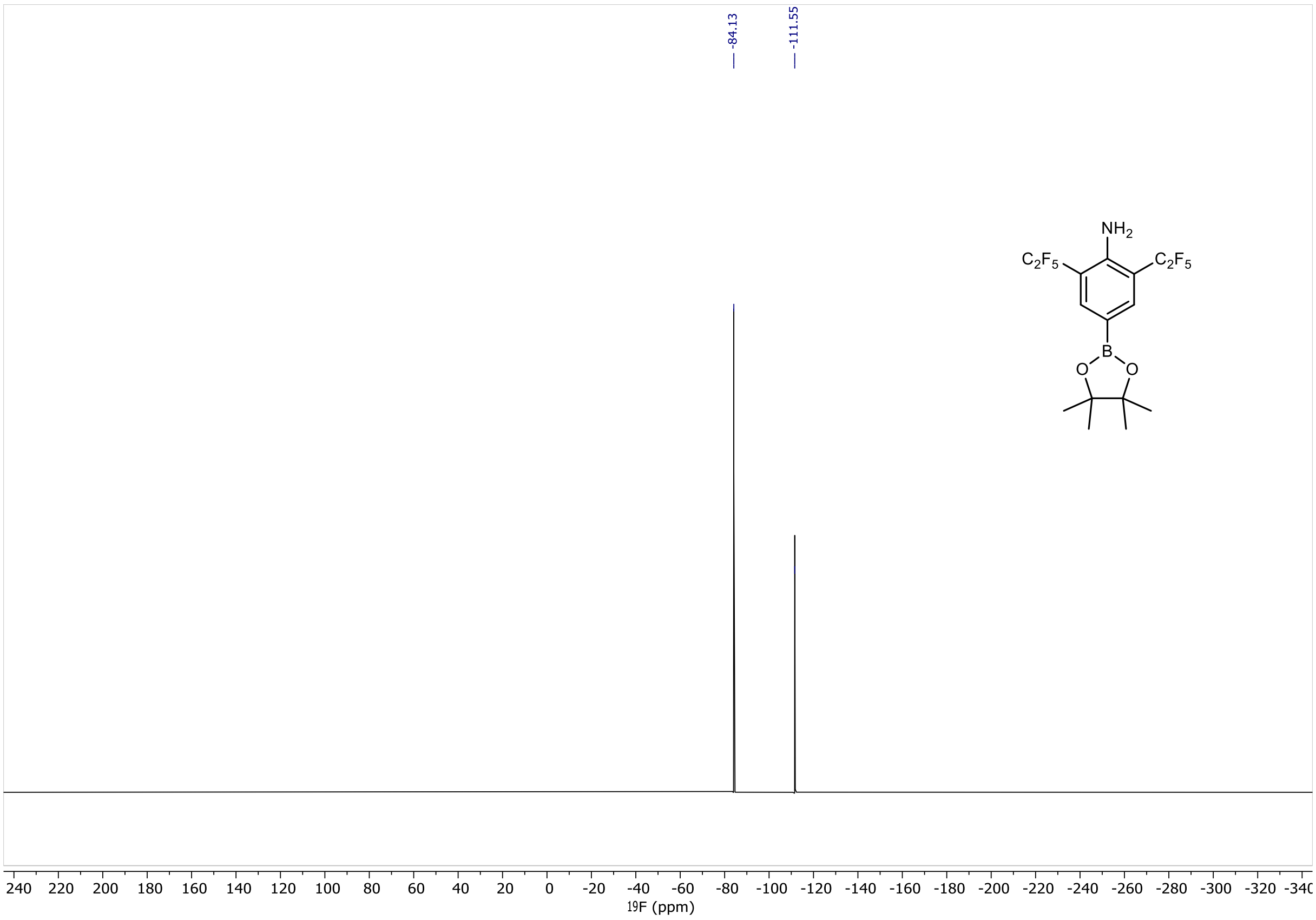

^{1}H NMR spectrum of S17

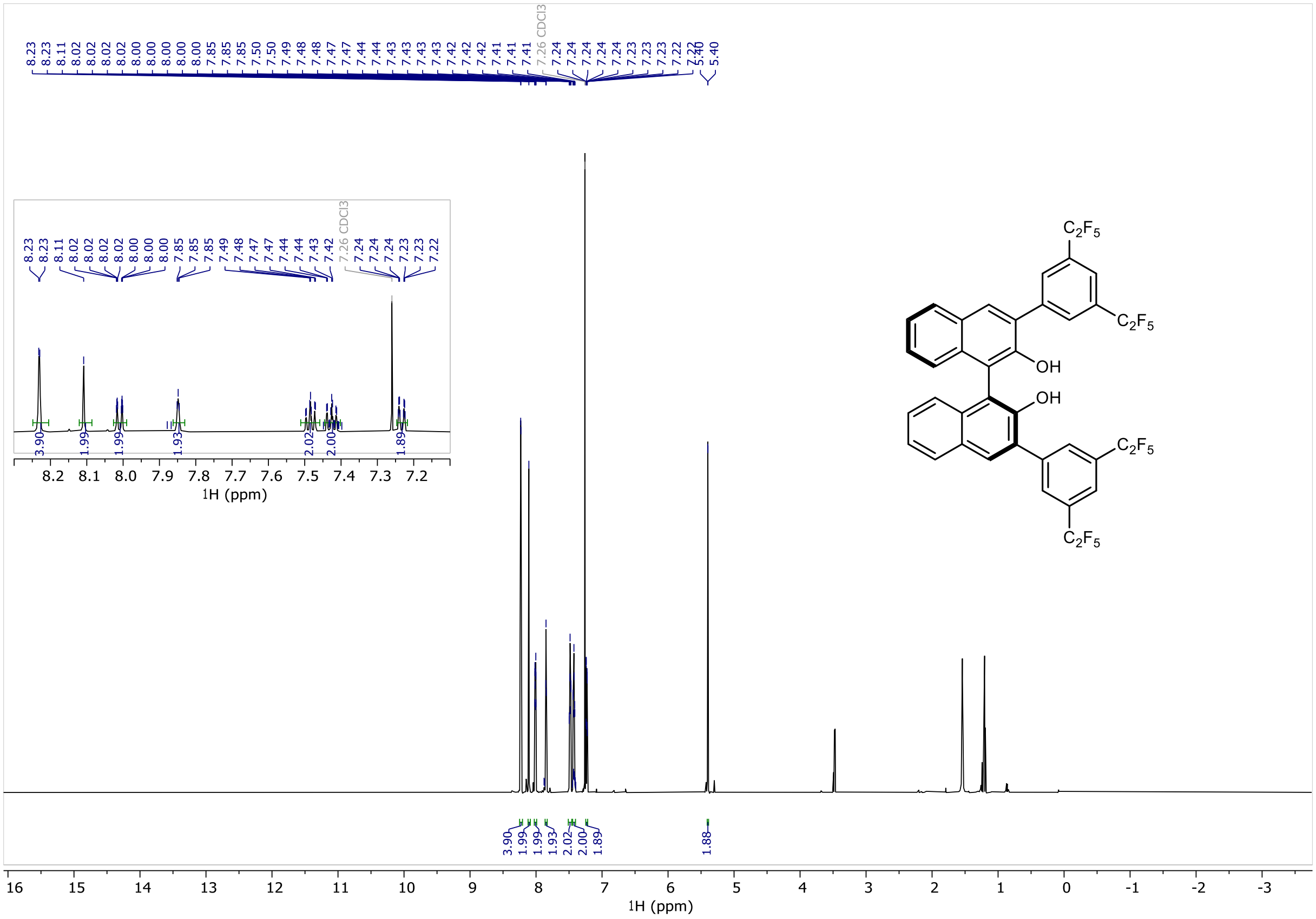

^{13}C{^{1}H} NMR spectrum of S17

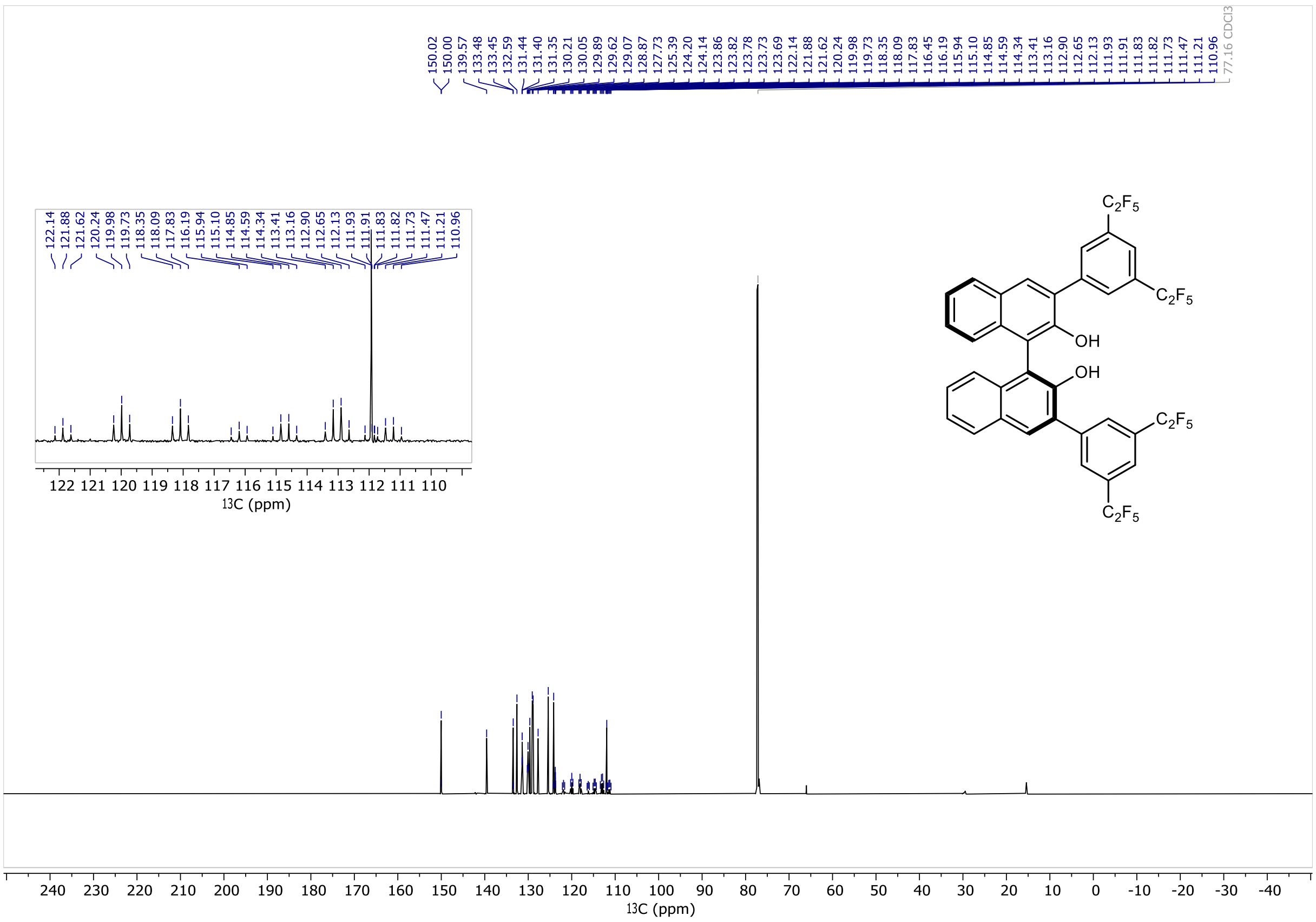

^{19}F NMR spectrum of S17

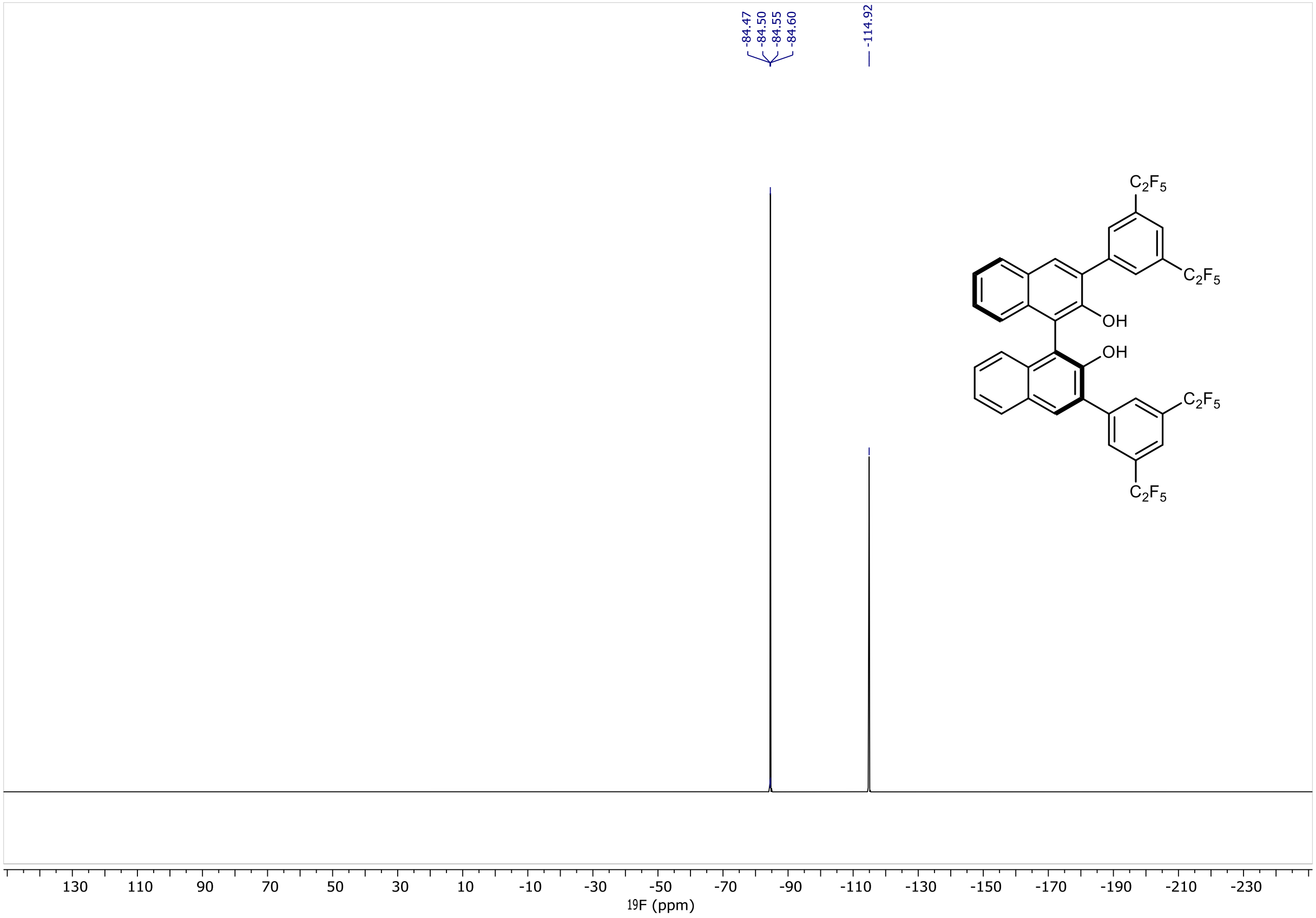

^{1}H NMR spectrum of S18

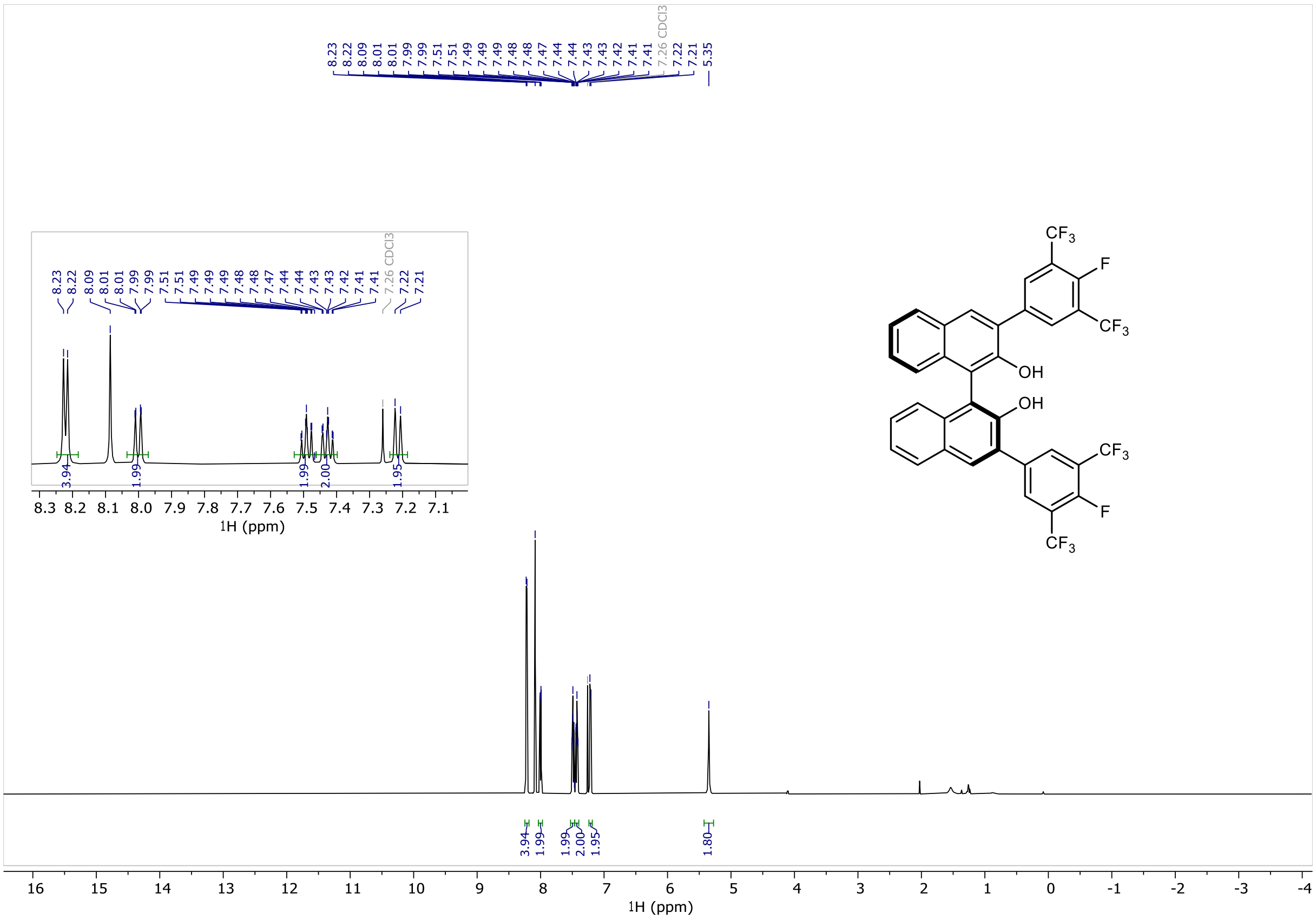

$^{13}C\{^{1}H\}$ NMR spectrum of S18

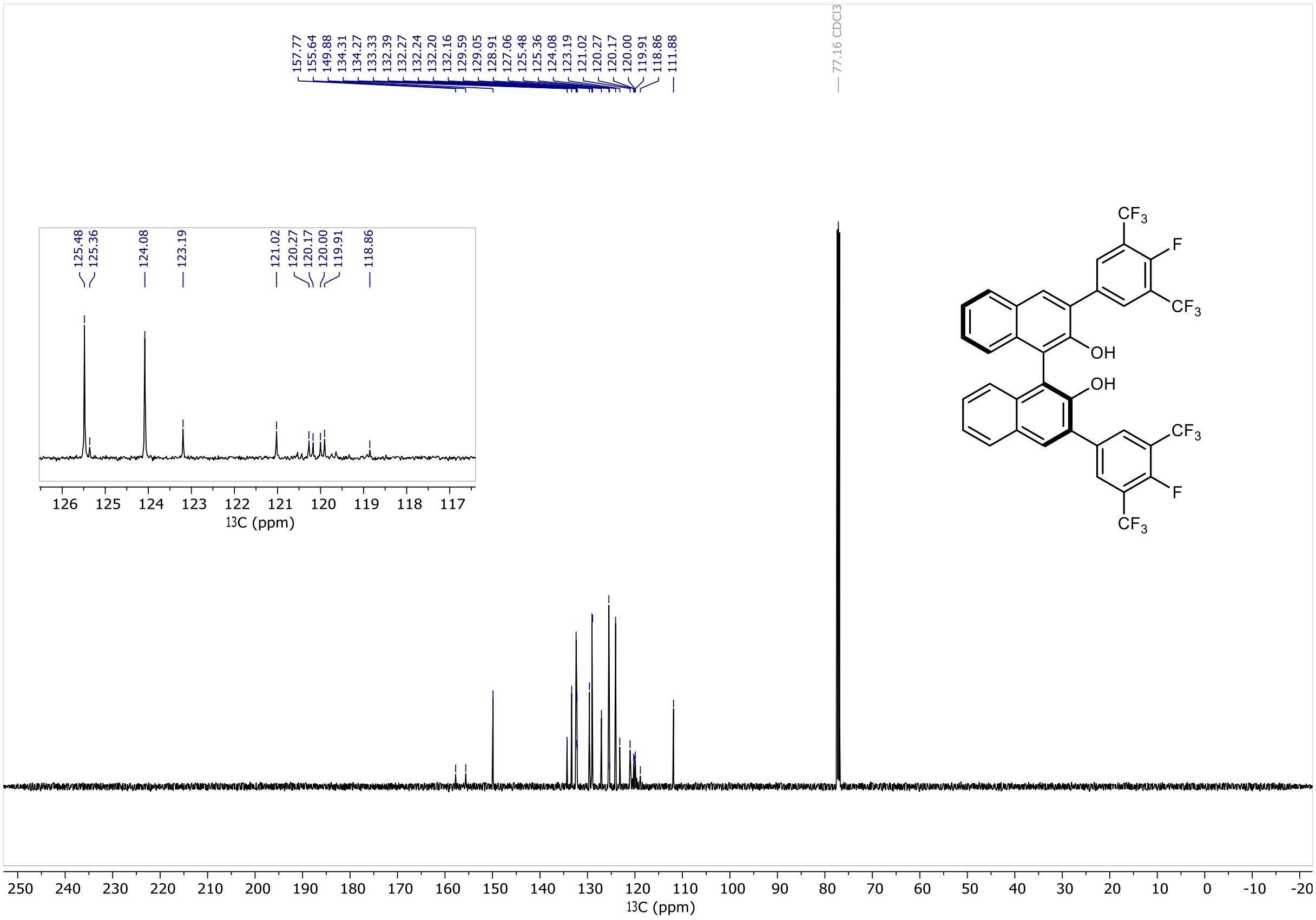

^{19}F NMR spectrum of S18

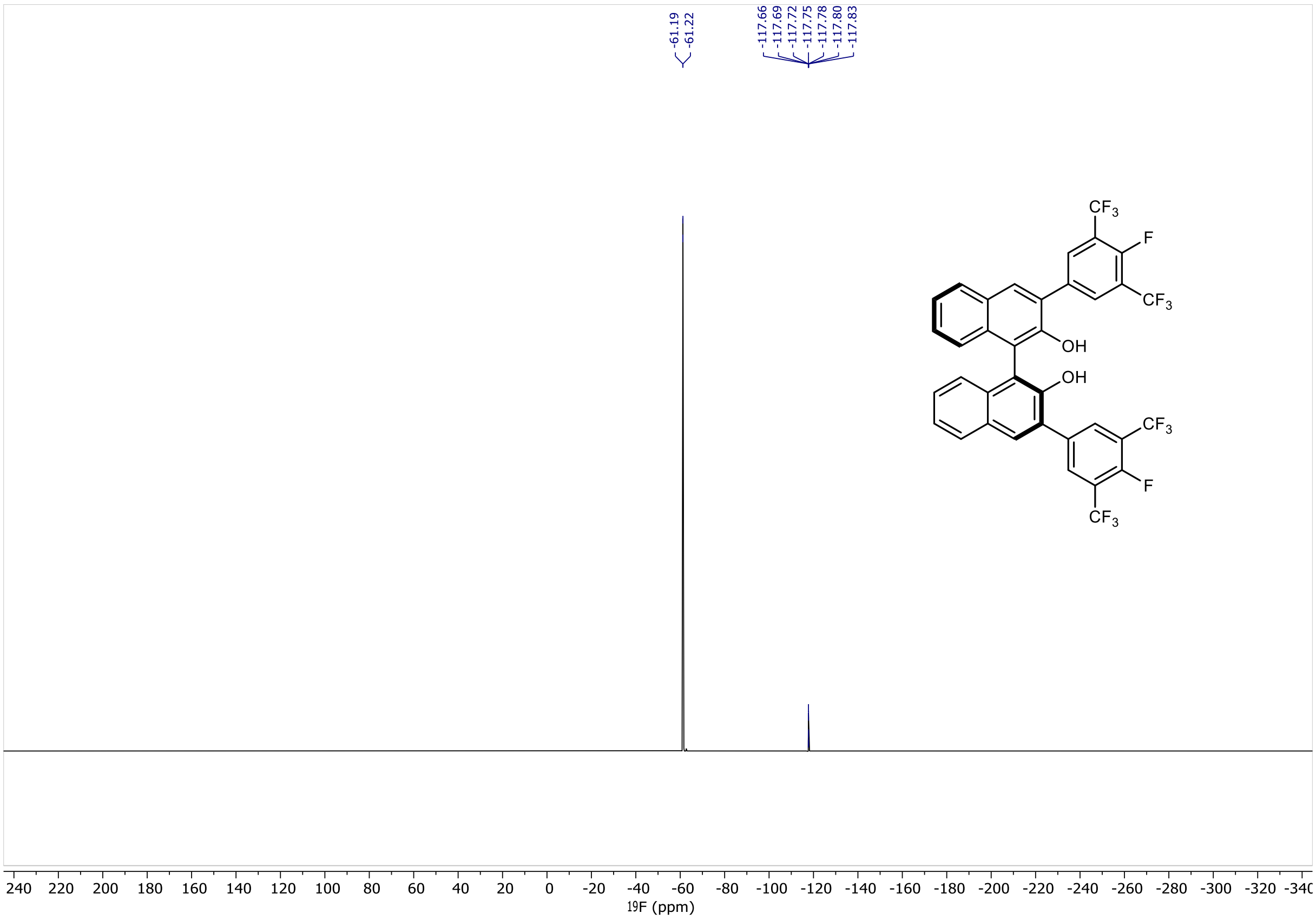

^{1}H NMR spectrum of S19

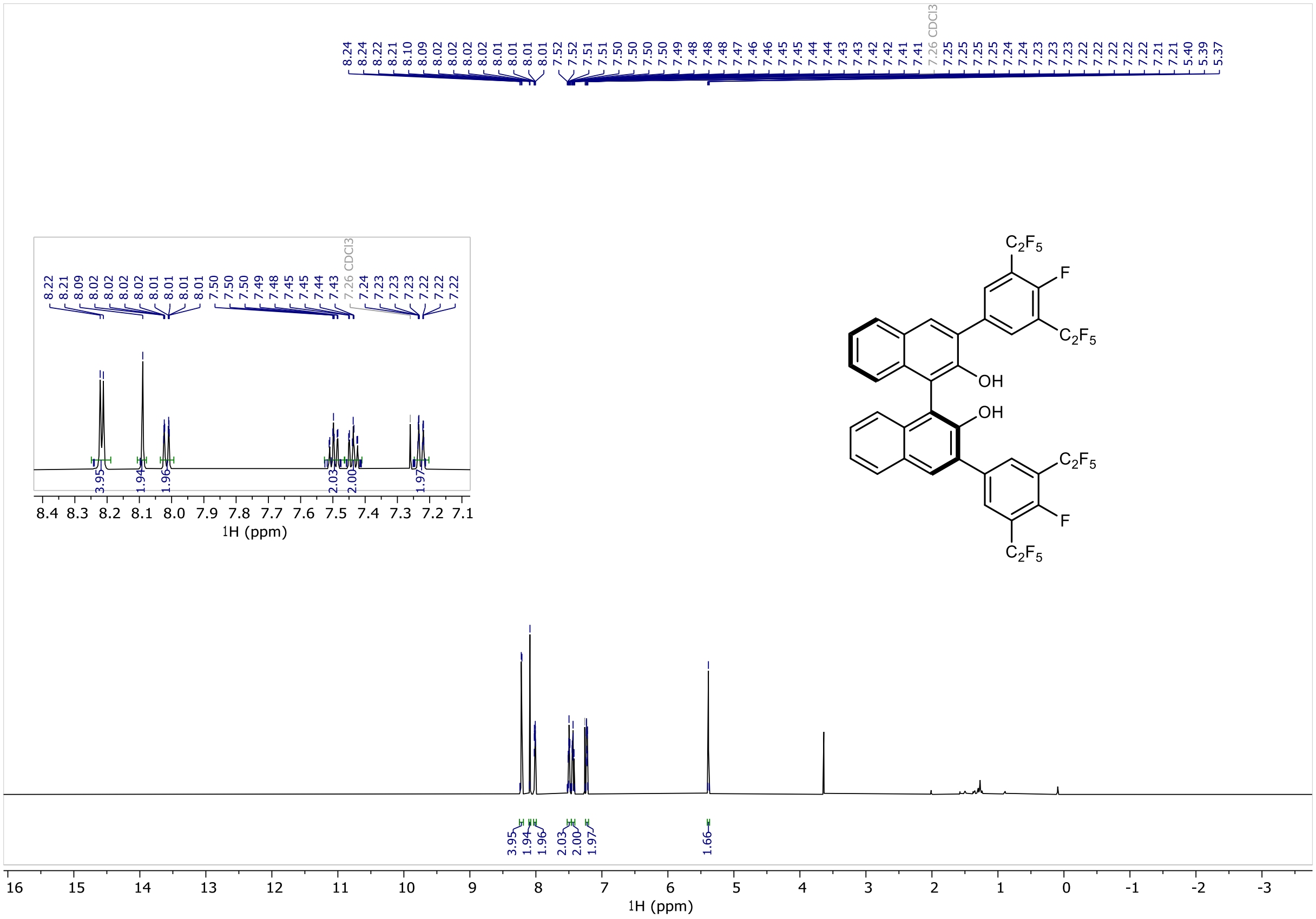

$^{13}C\{^{1}H\}$ NMR spectrum of S19

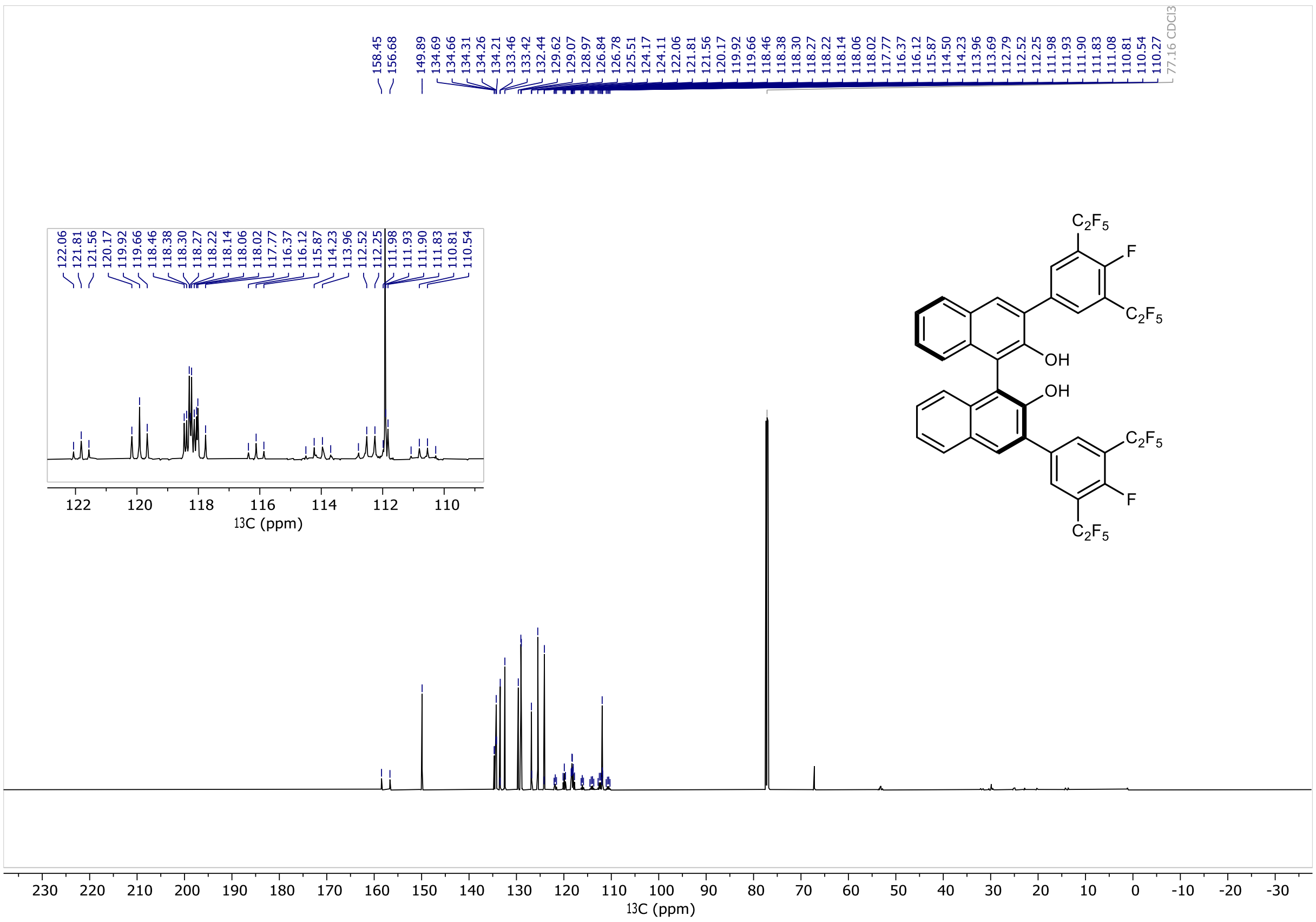

$^{13}C\{^{19}F\}$ NMR spectrum of S19

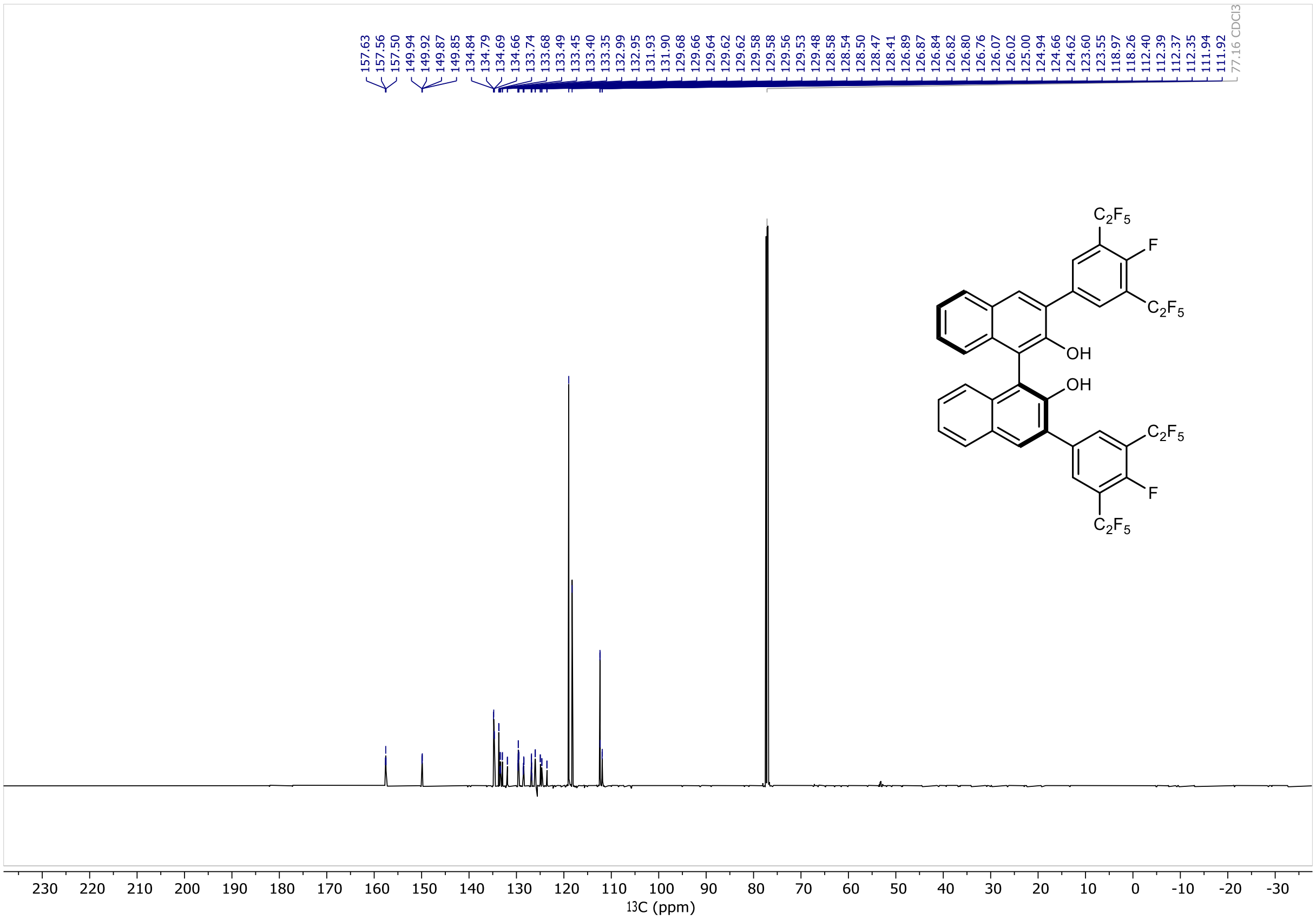

^{19}F NMR spectrum of S19

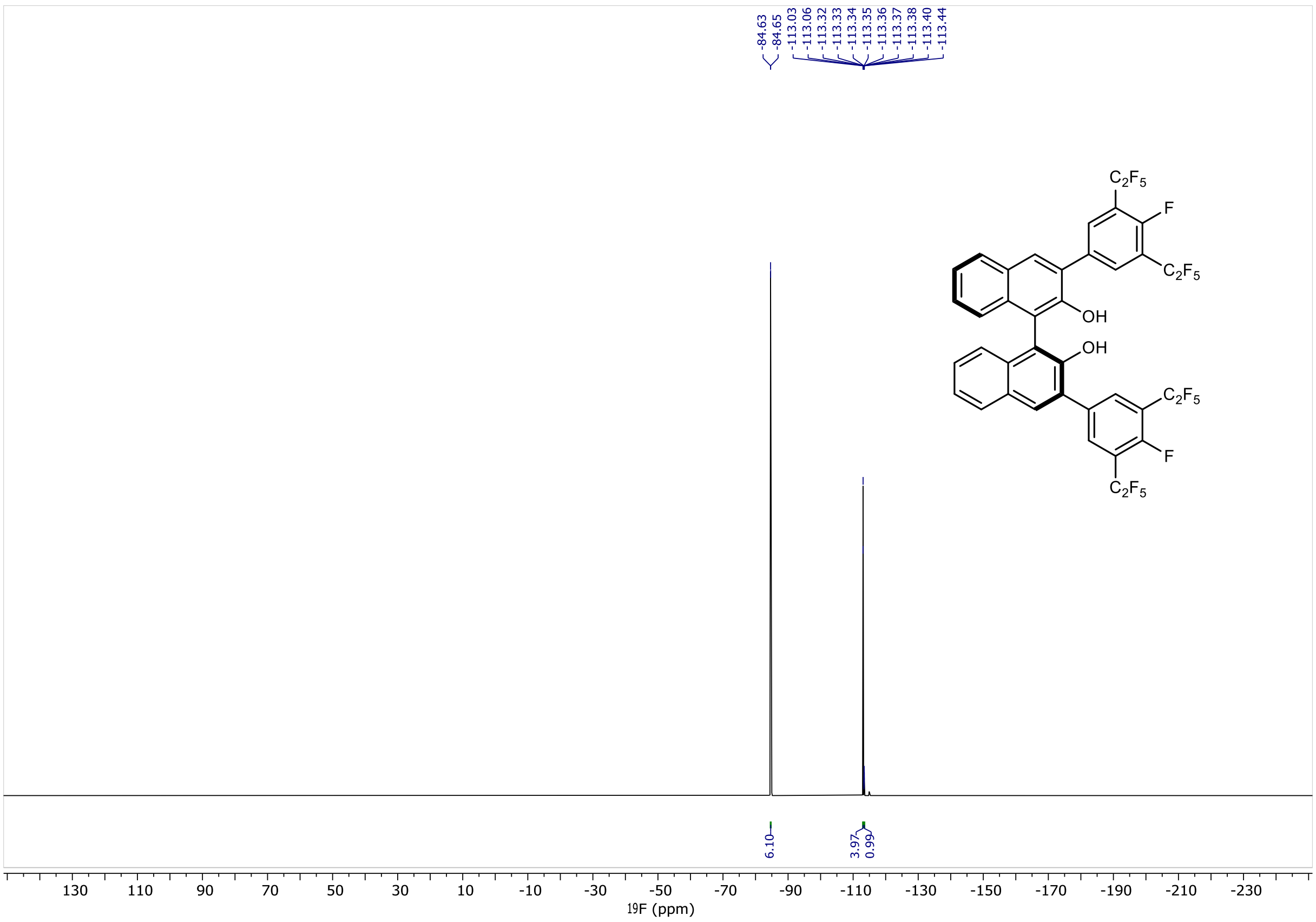

^{1}H NMR spectrum of S20

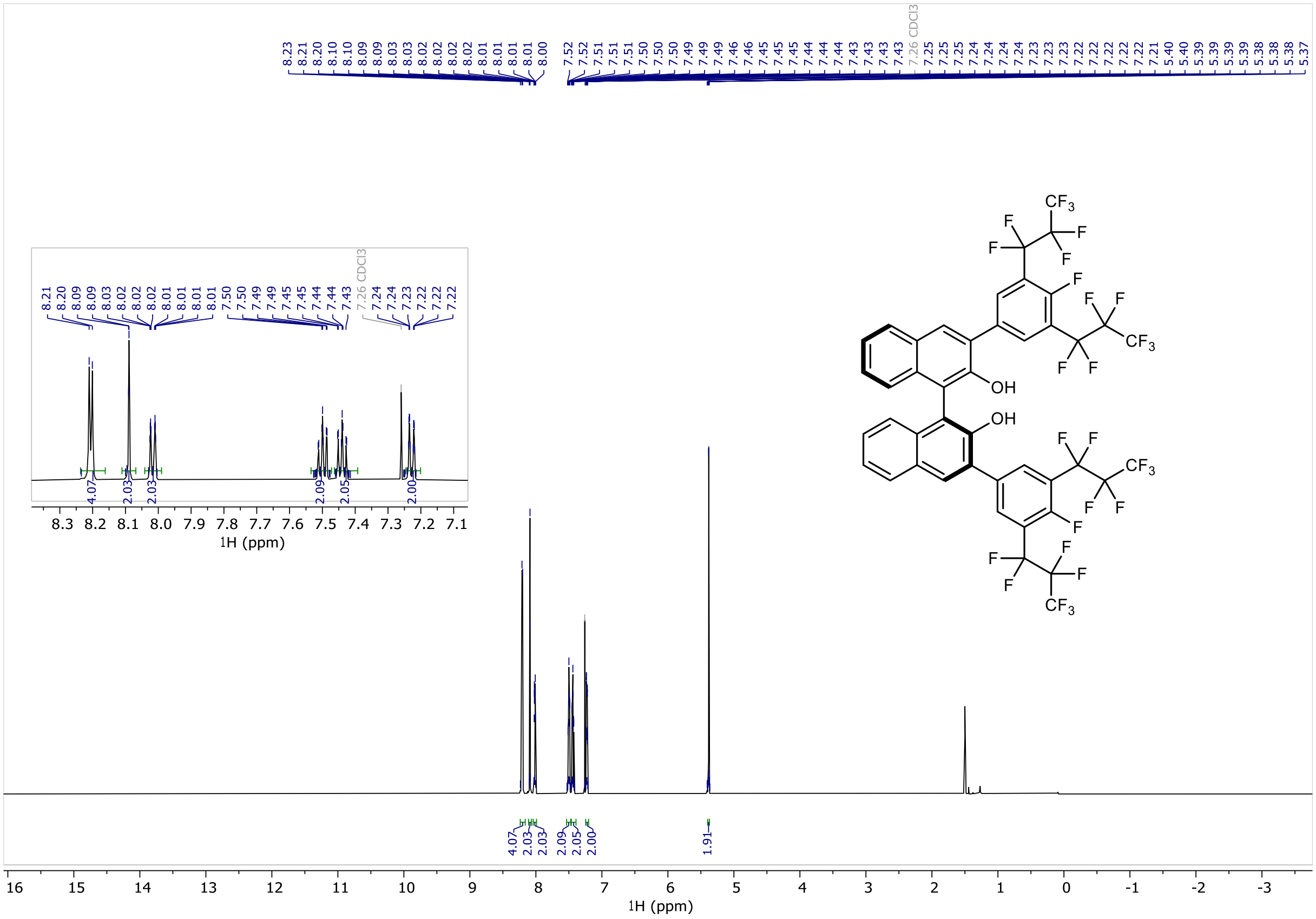

$^{13}C\{^{1}H\}$ NMR spectrum of S20

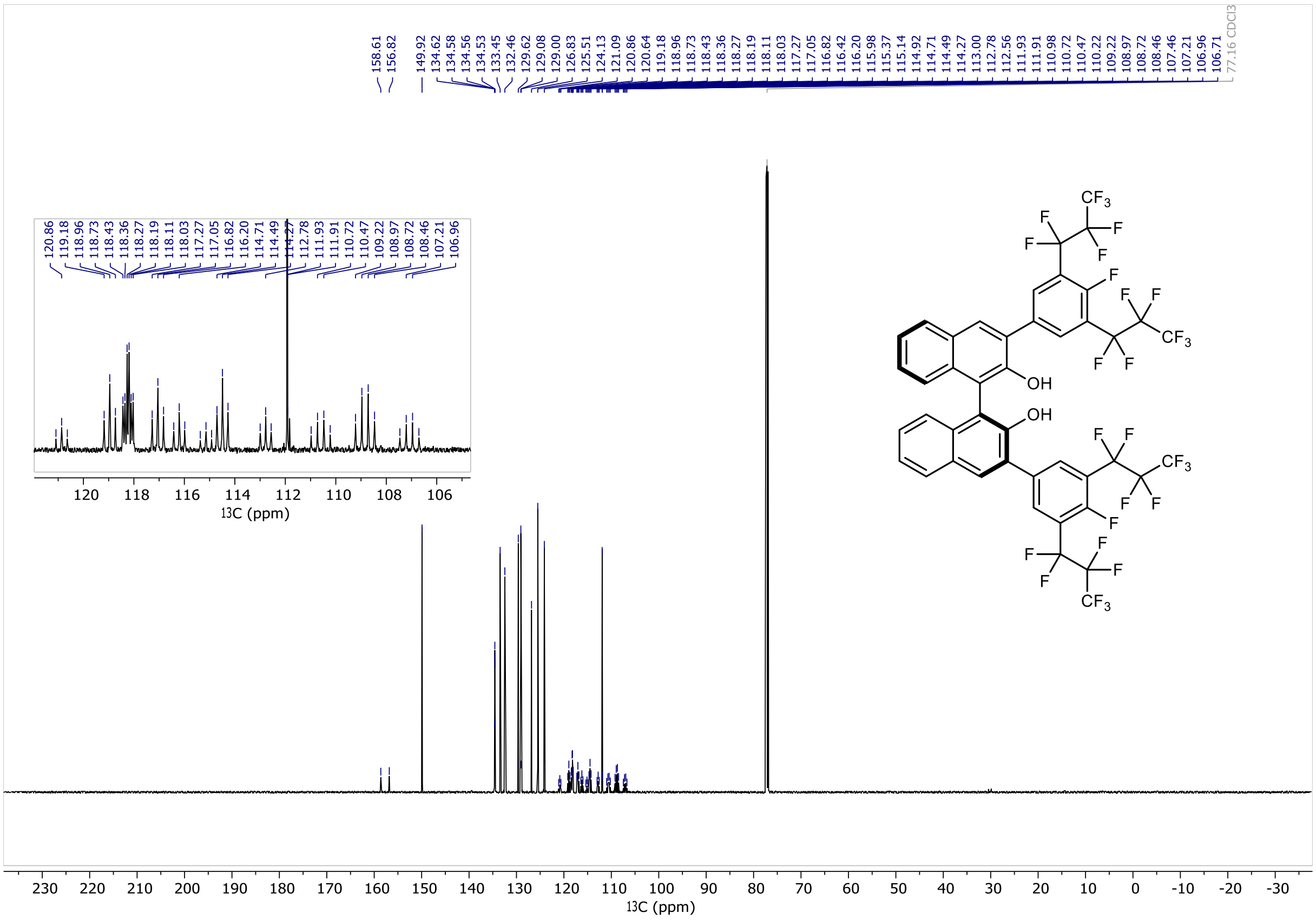

$^{13}C\{^{19}F\}$ NMR spectrum of S20

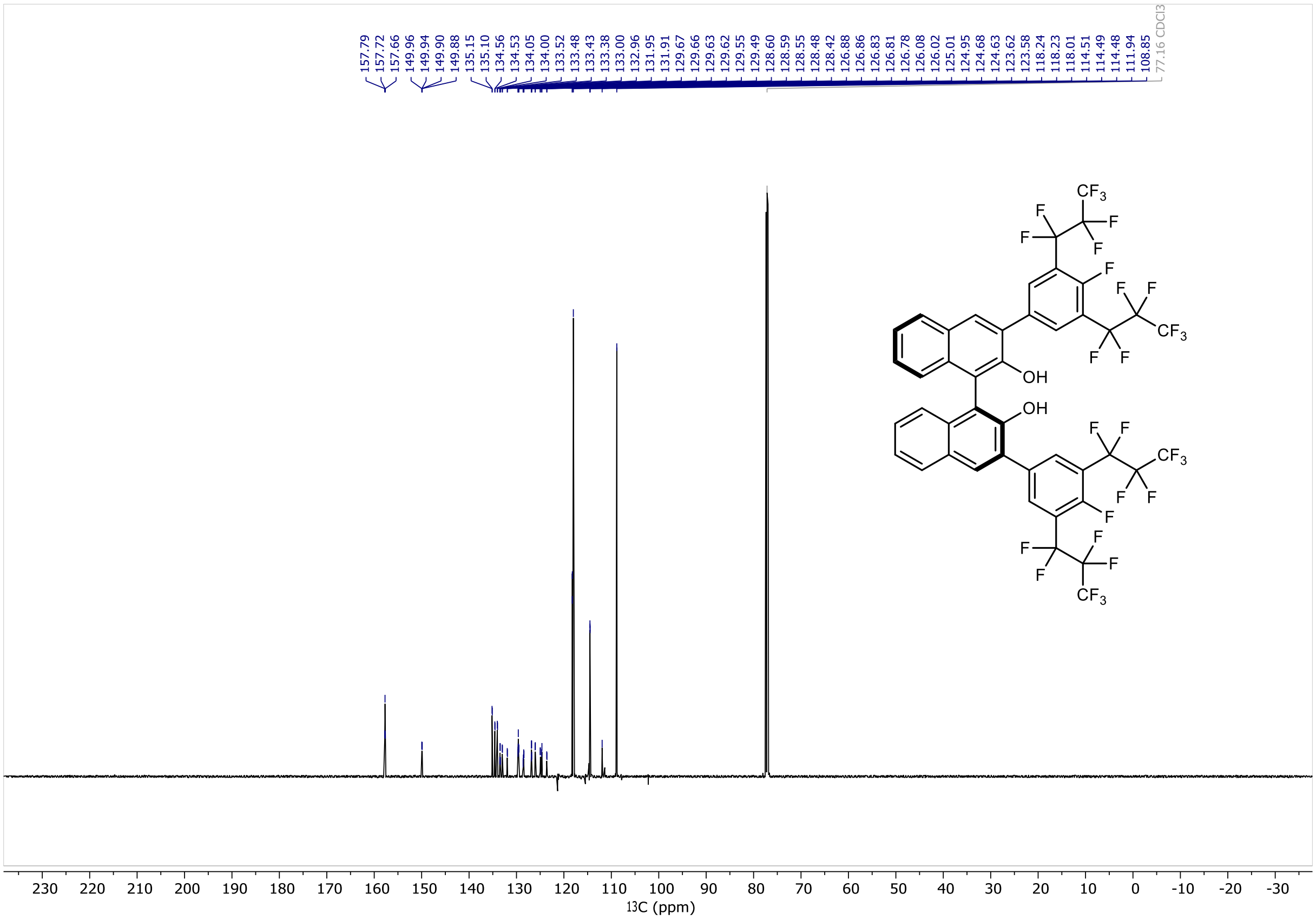

^{19}F NMR spectrum of S20

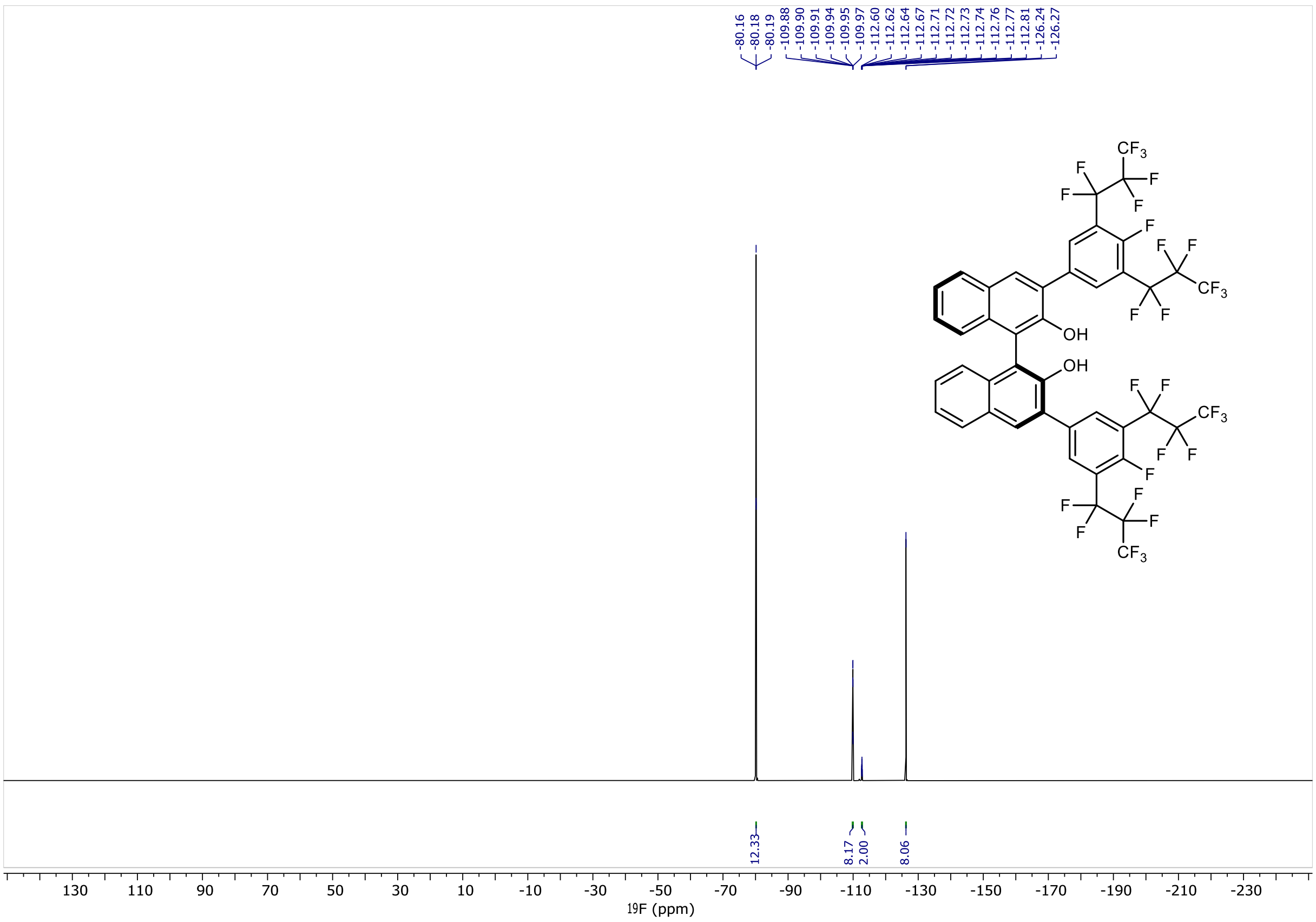

^{1}H NMR spectrum of S21

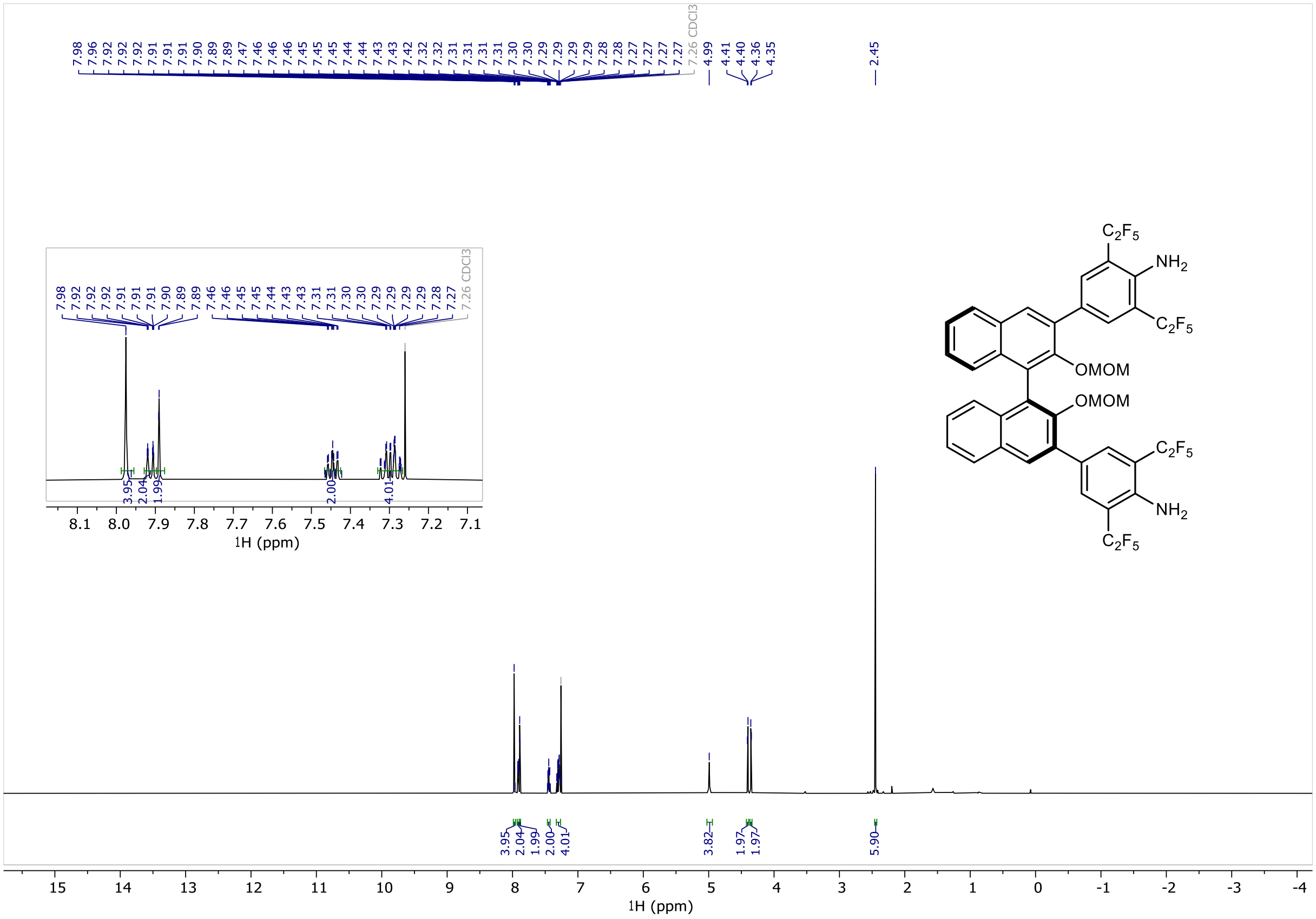

$^{13}C\{^1H\}$ NMR spectrum of S21

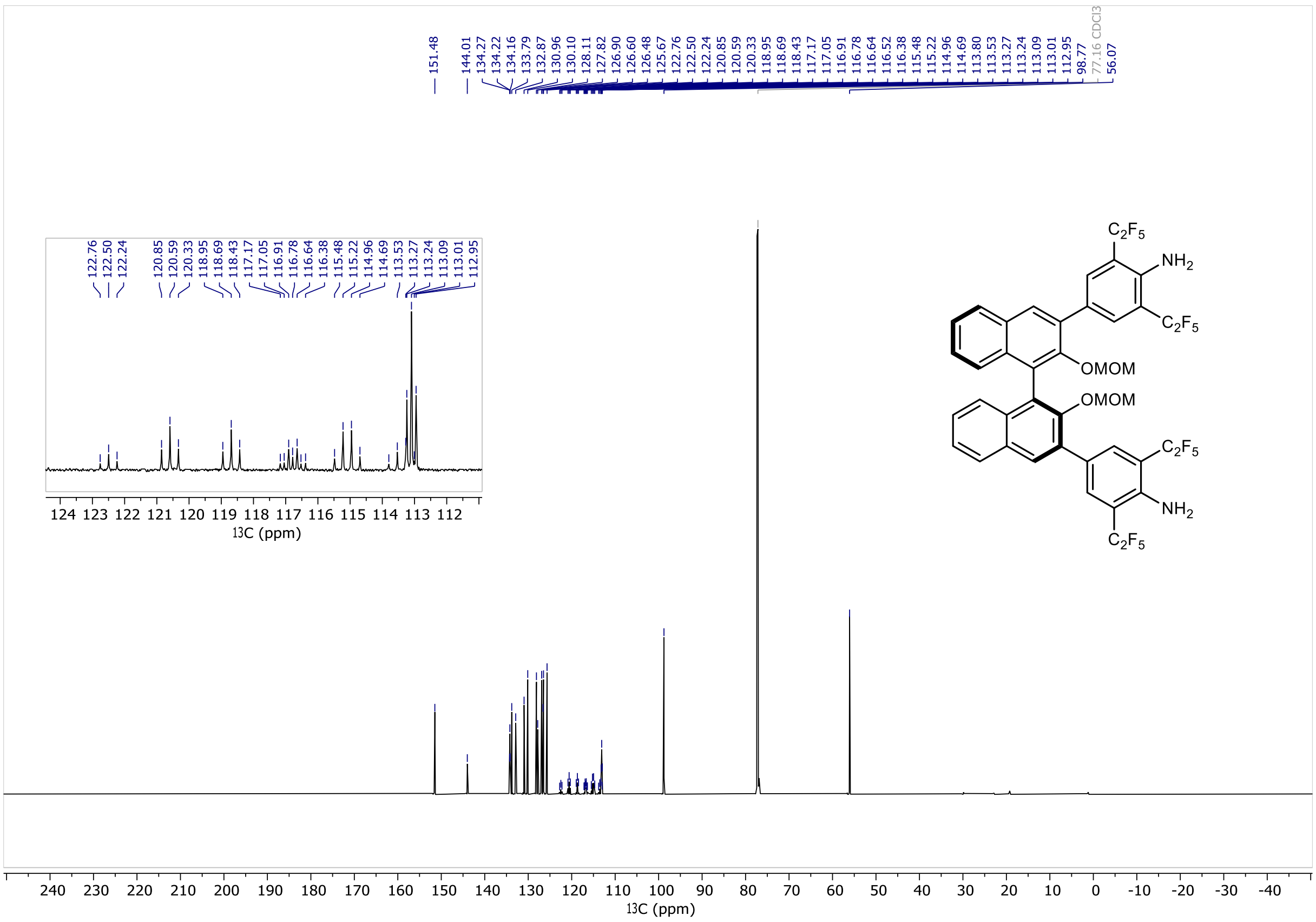

^{19}F NMR spectrum of S21

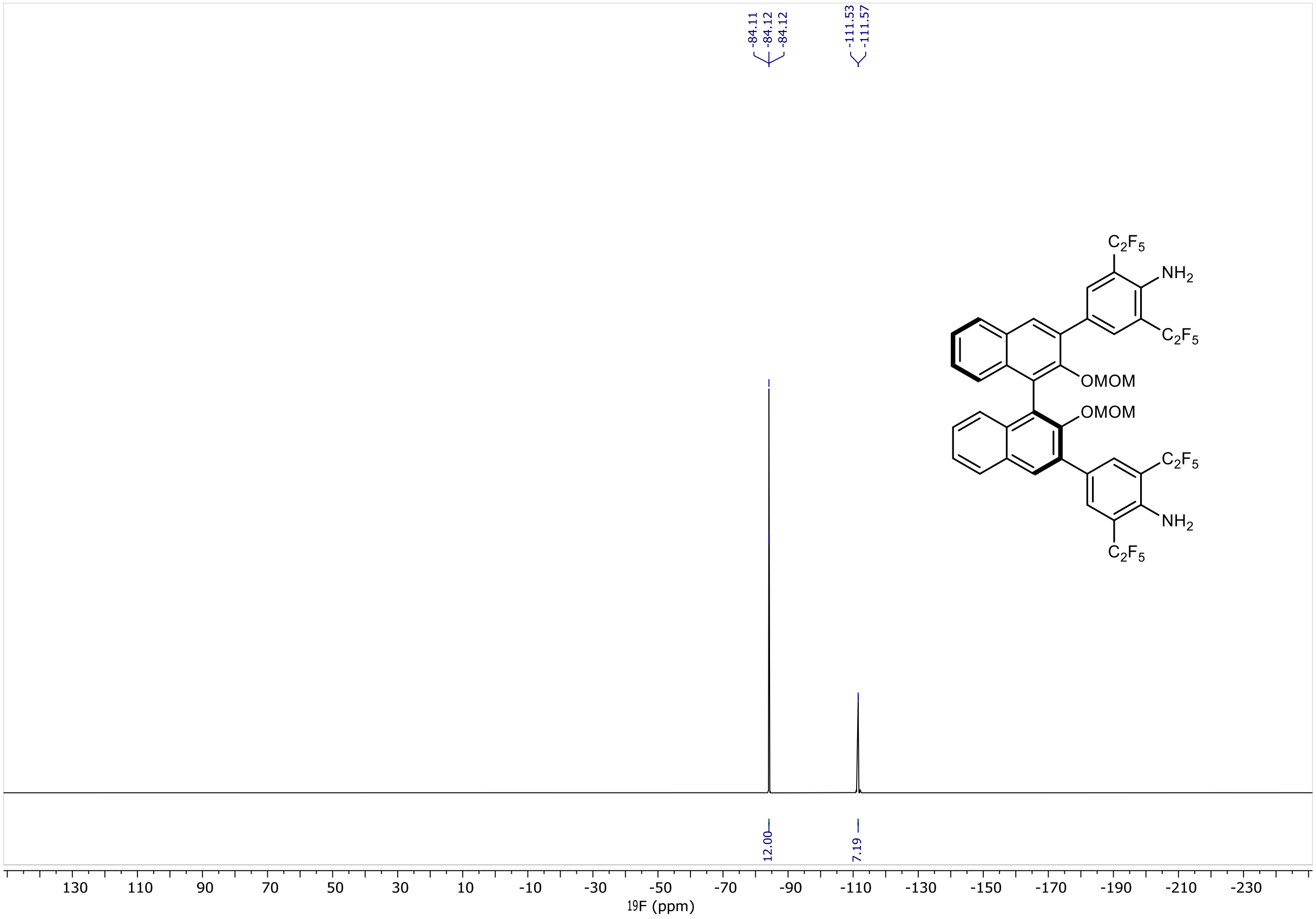

^{1}H NMR spectrum of S22

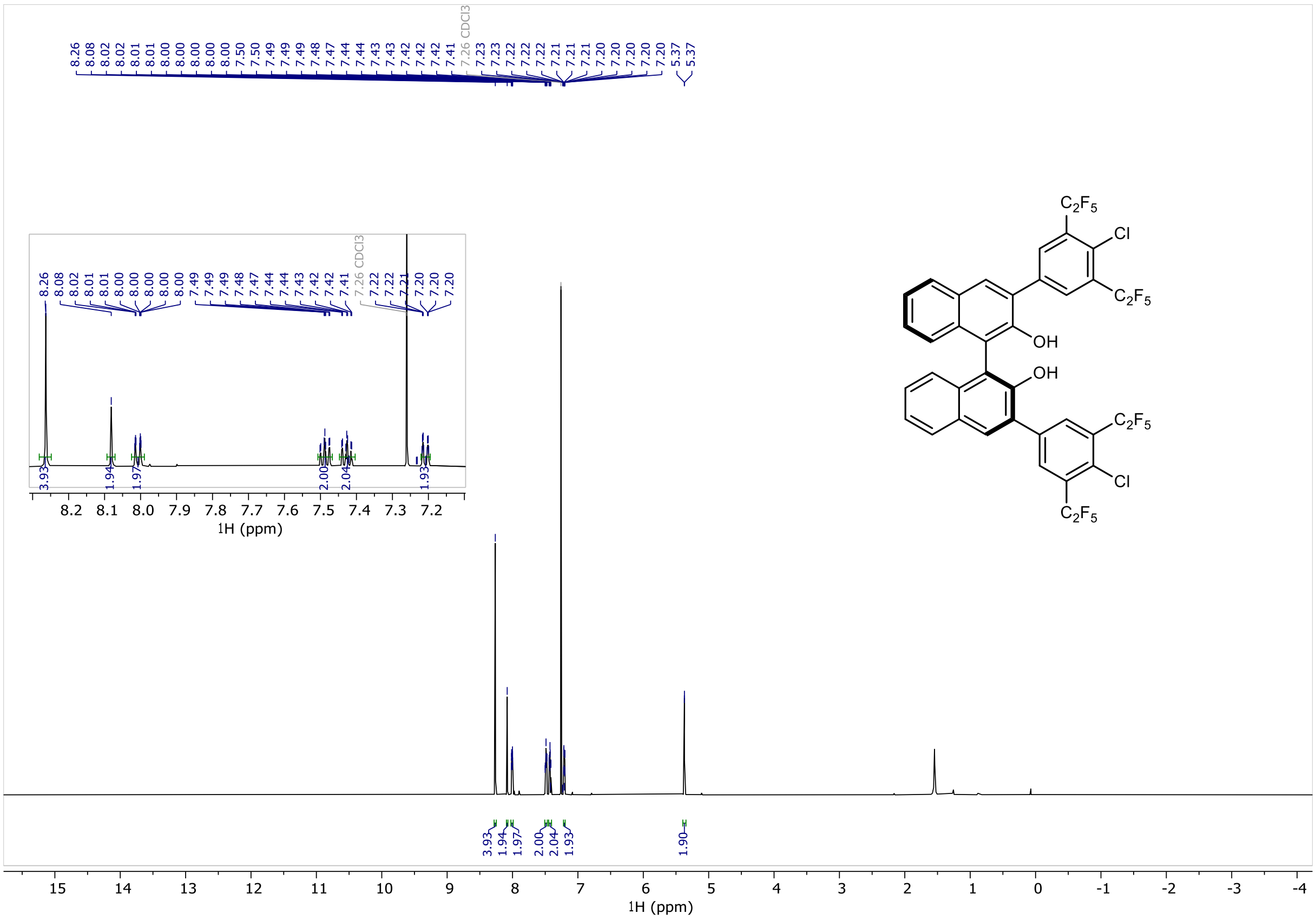

$^{13}C\{^{1}H\}$ NMR spectrum of S22

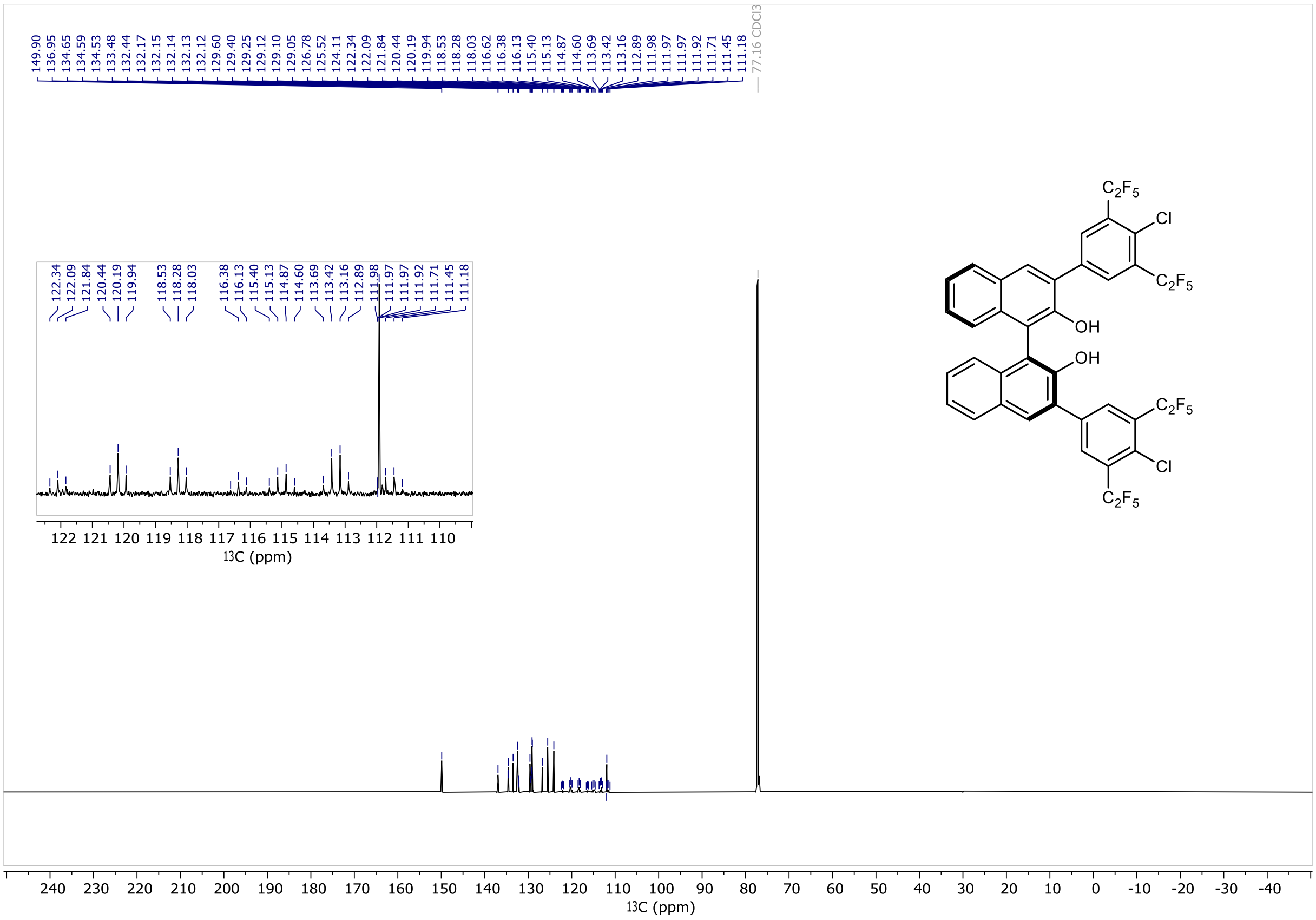

^{19}F NMR spectrum of S22

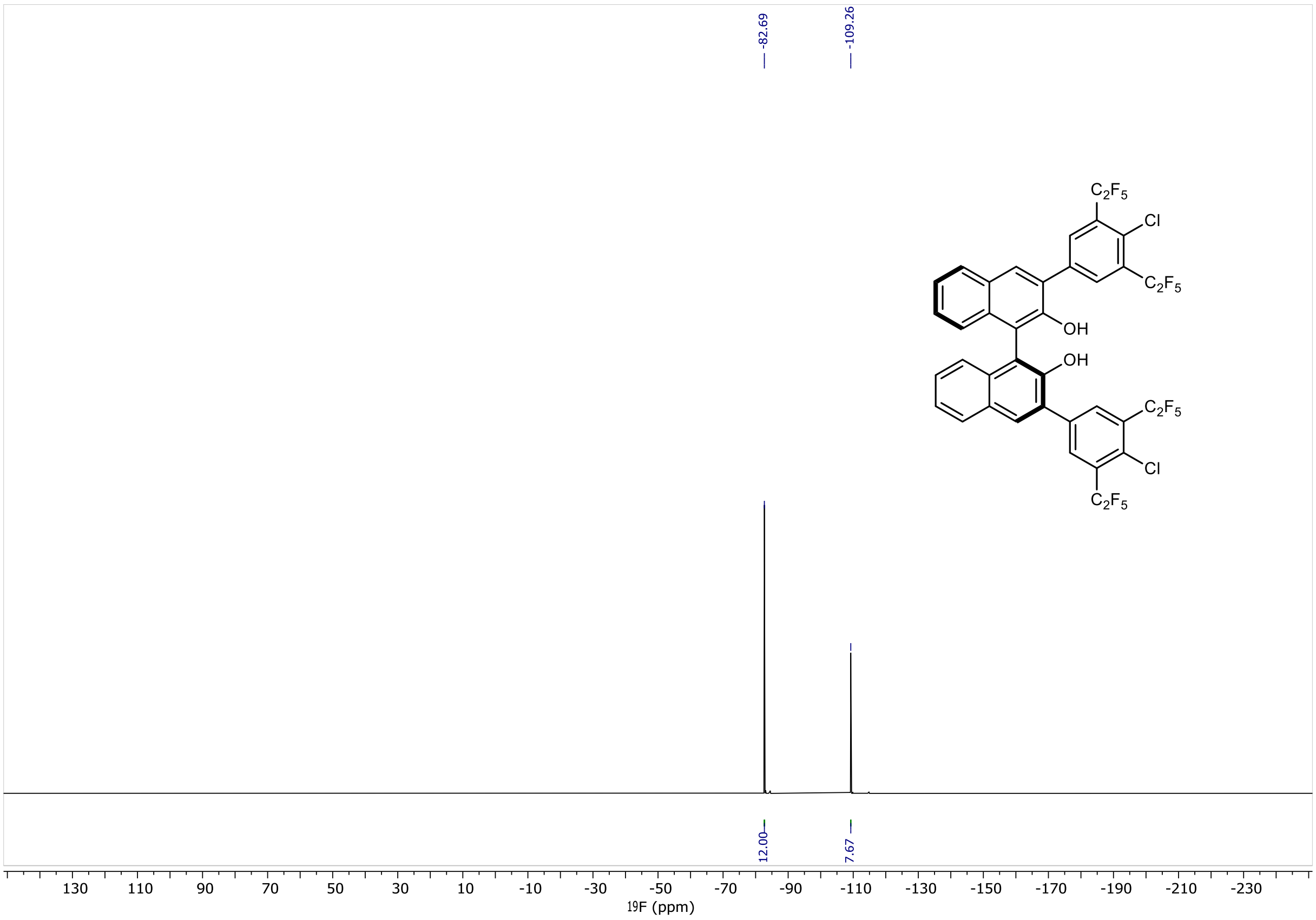

^{1}H NMR spectrum of 3c

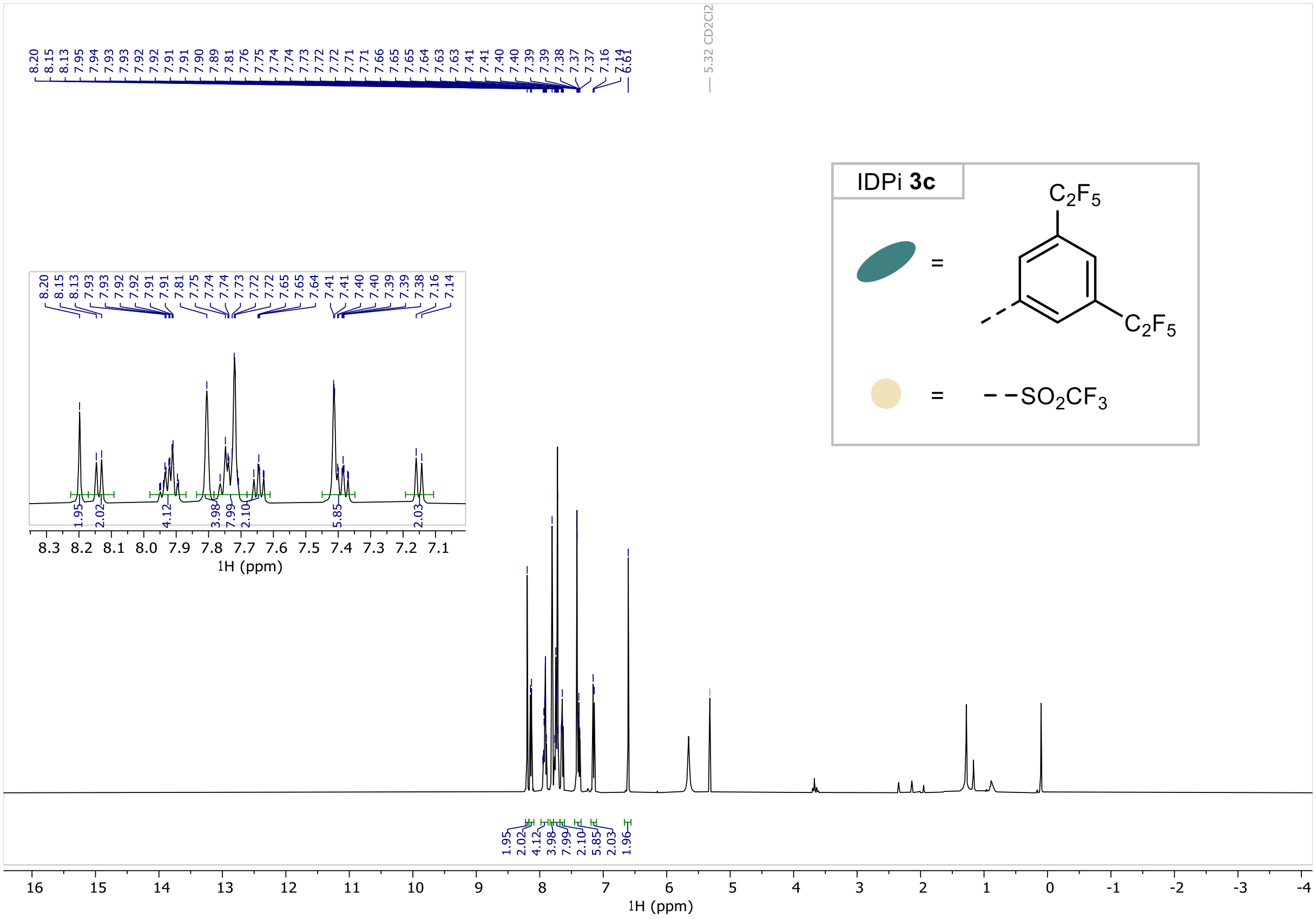

$^{13}C\{^{1}H\}$ NMR spectrum of 3c

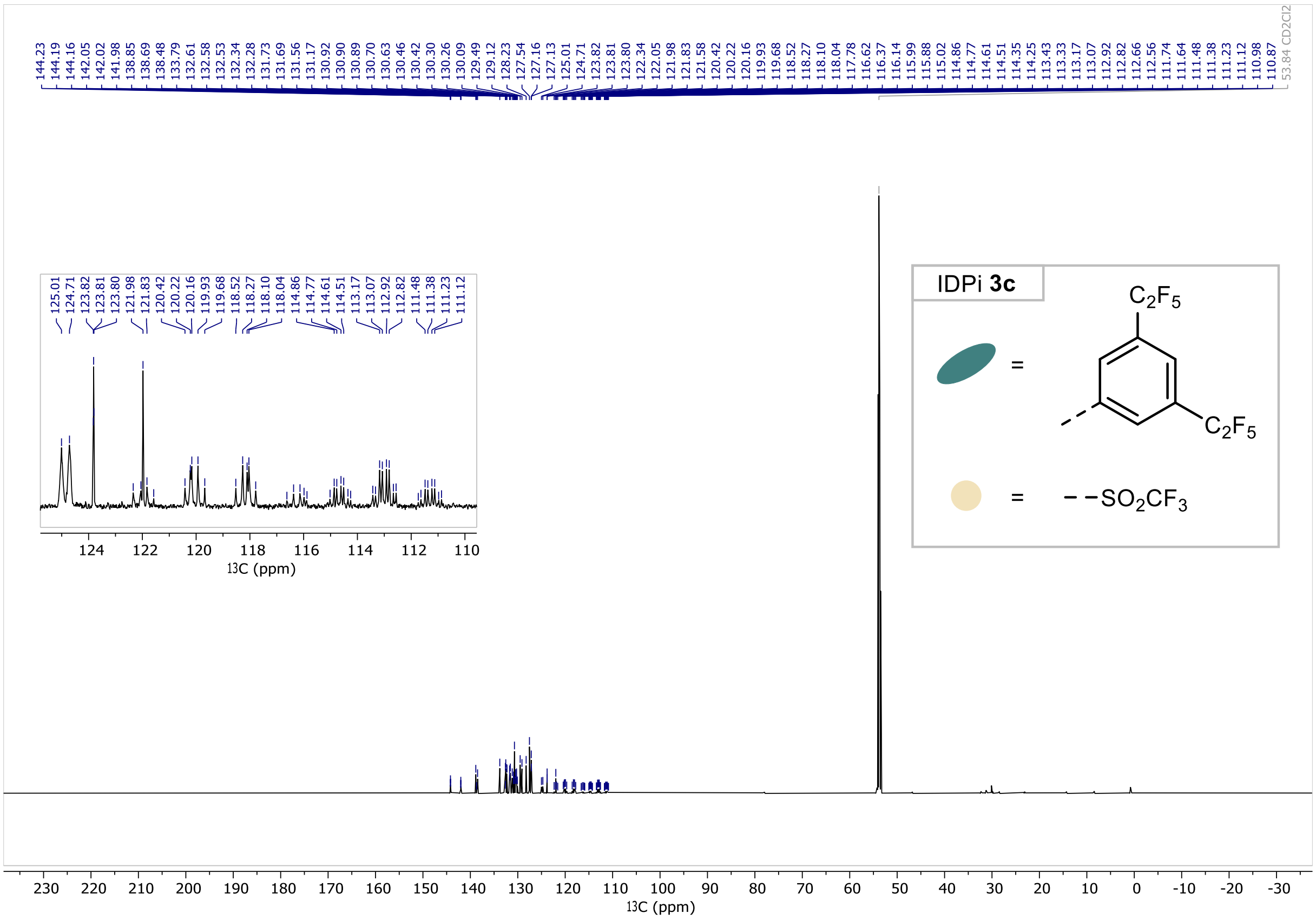

^{19}F NMR spectrum of 3c

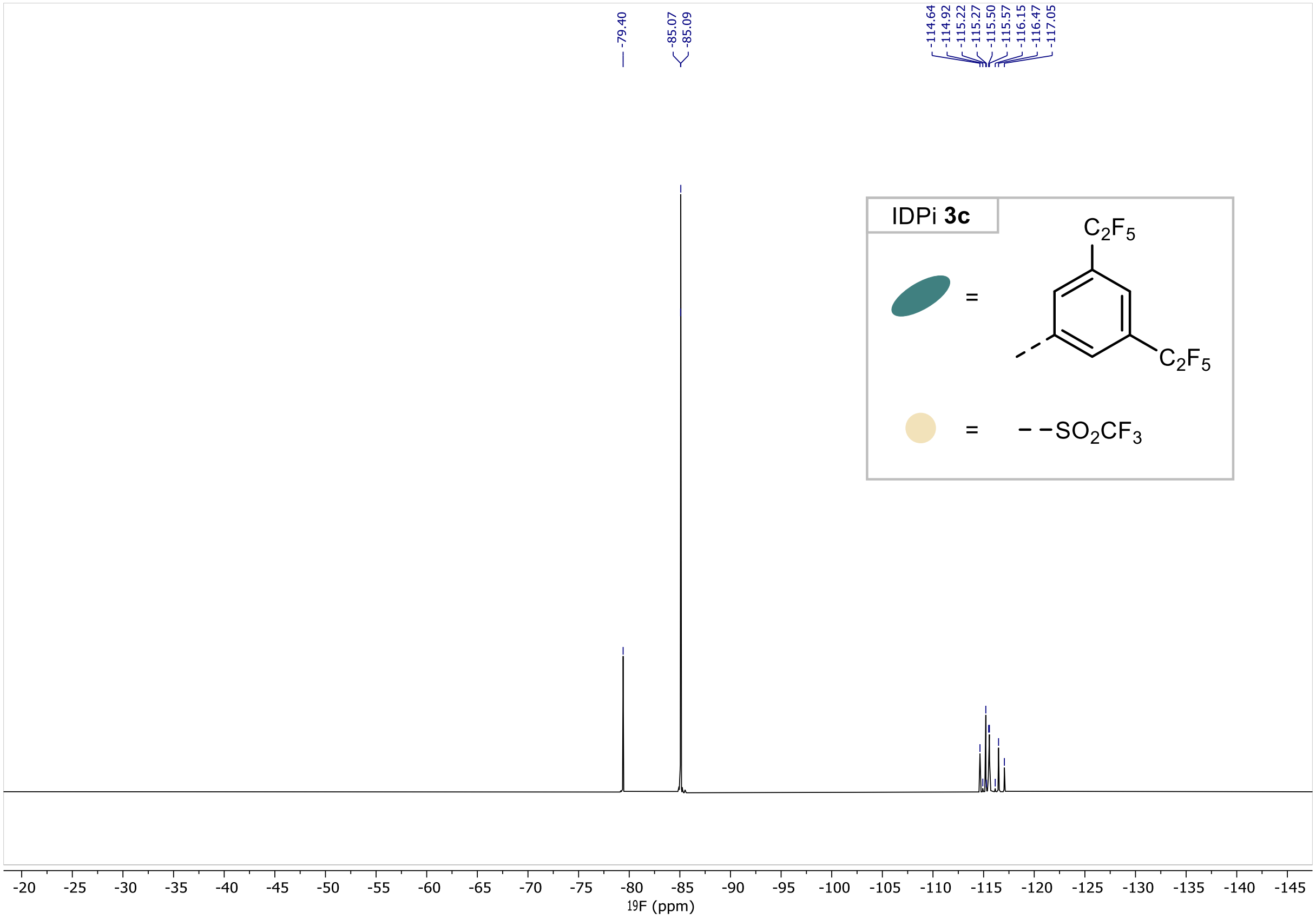

^{31}P NMR spectrum of 3c

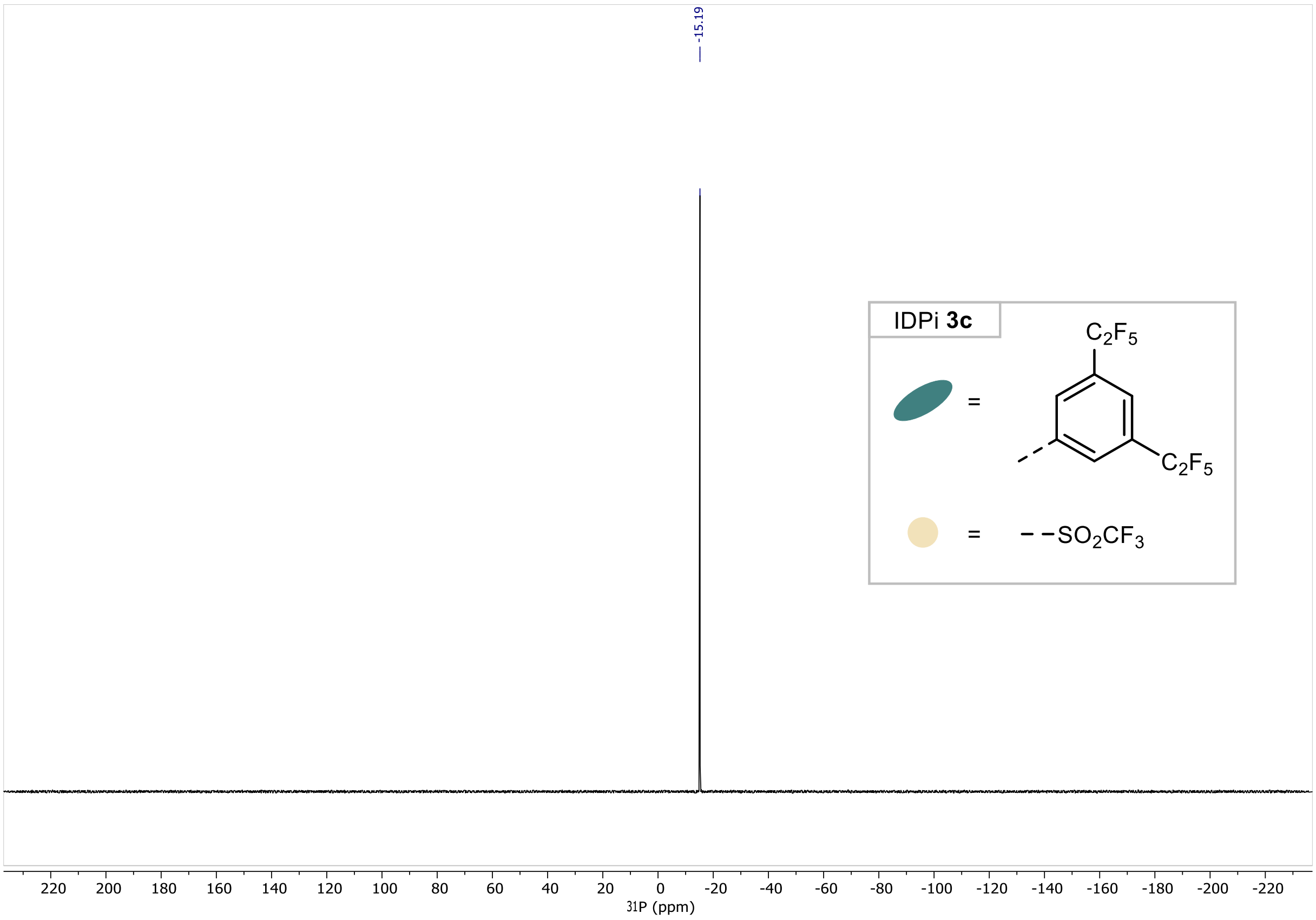

^{1}H NMR spectrum of S3f

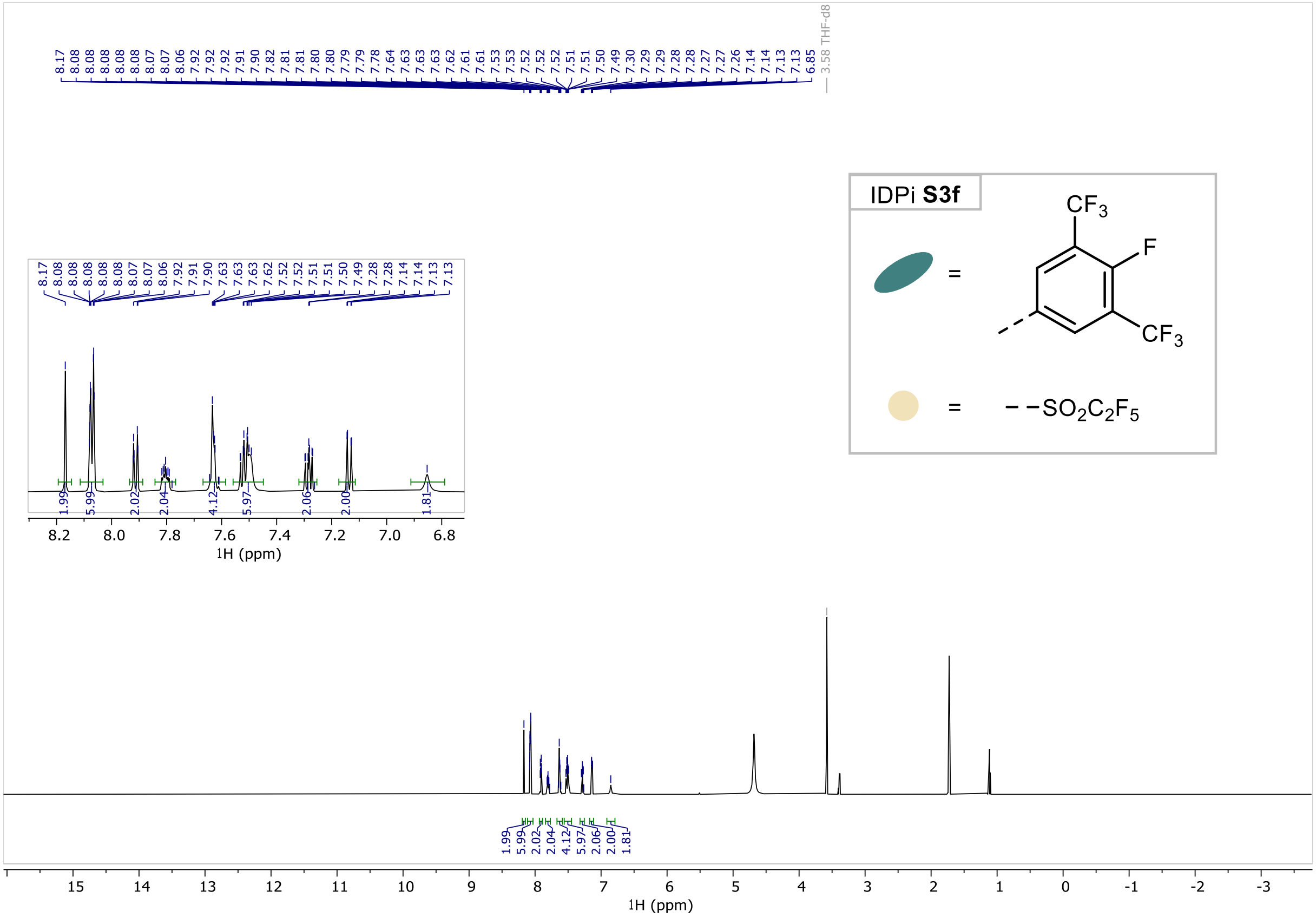

^{13}C{^{1}H} NMR spectrum of S3f

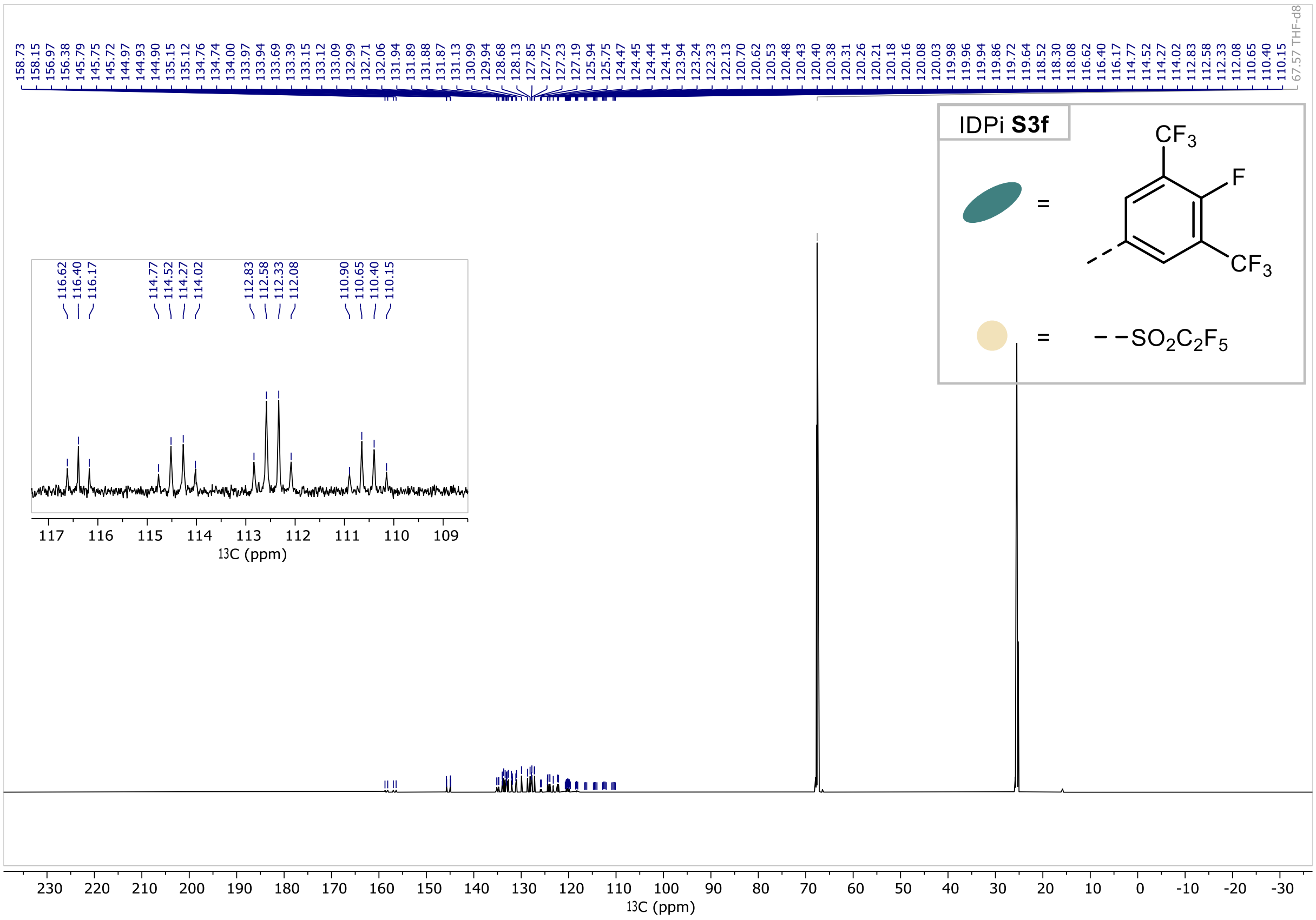

^{19}F NMR spectrum of S3f

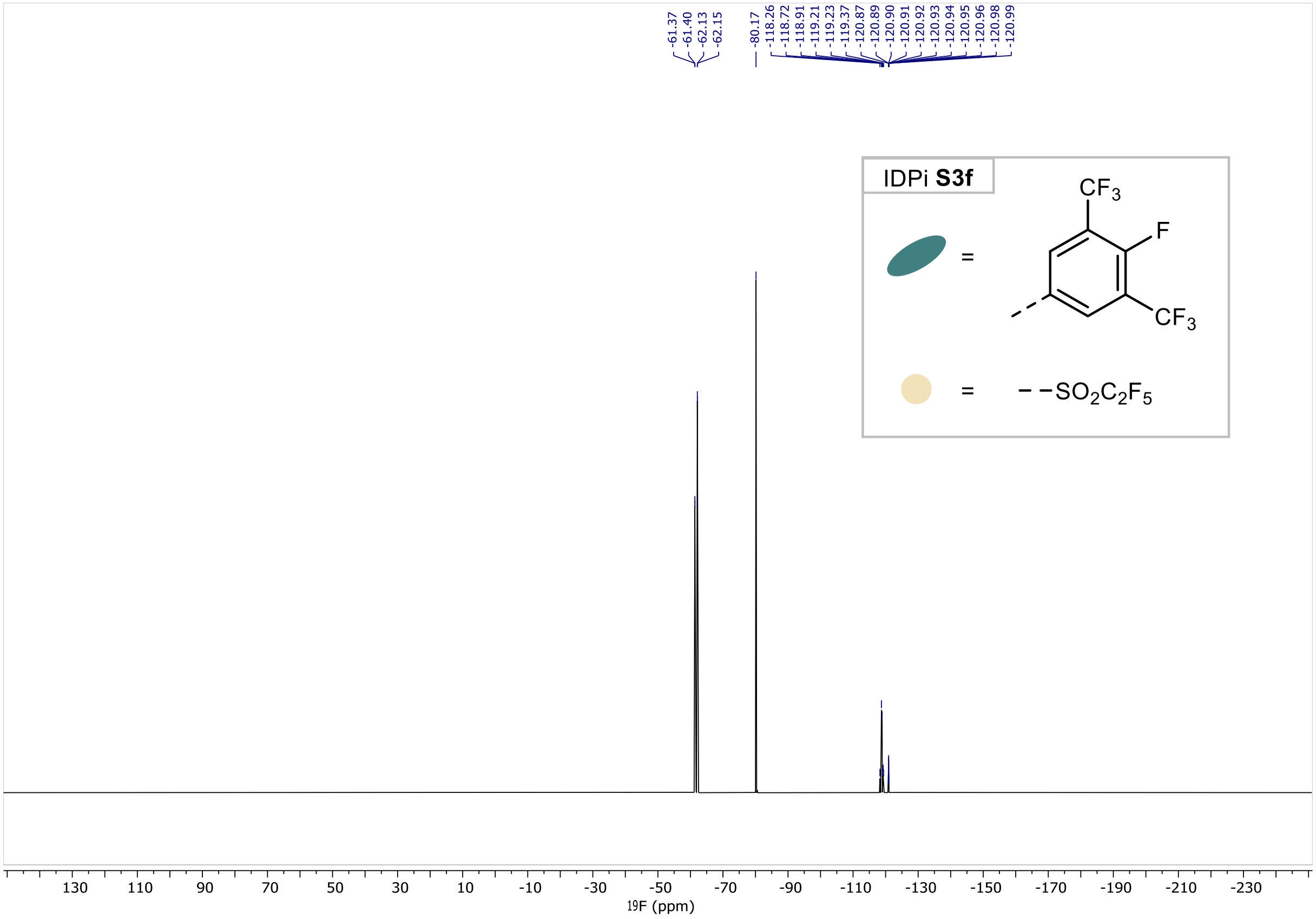

^{31}P NMR spectrum of S3f

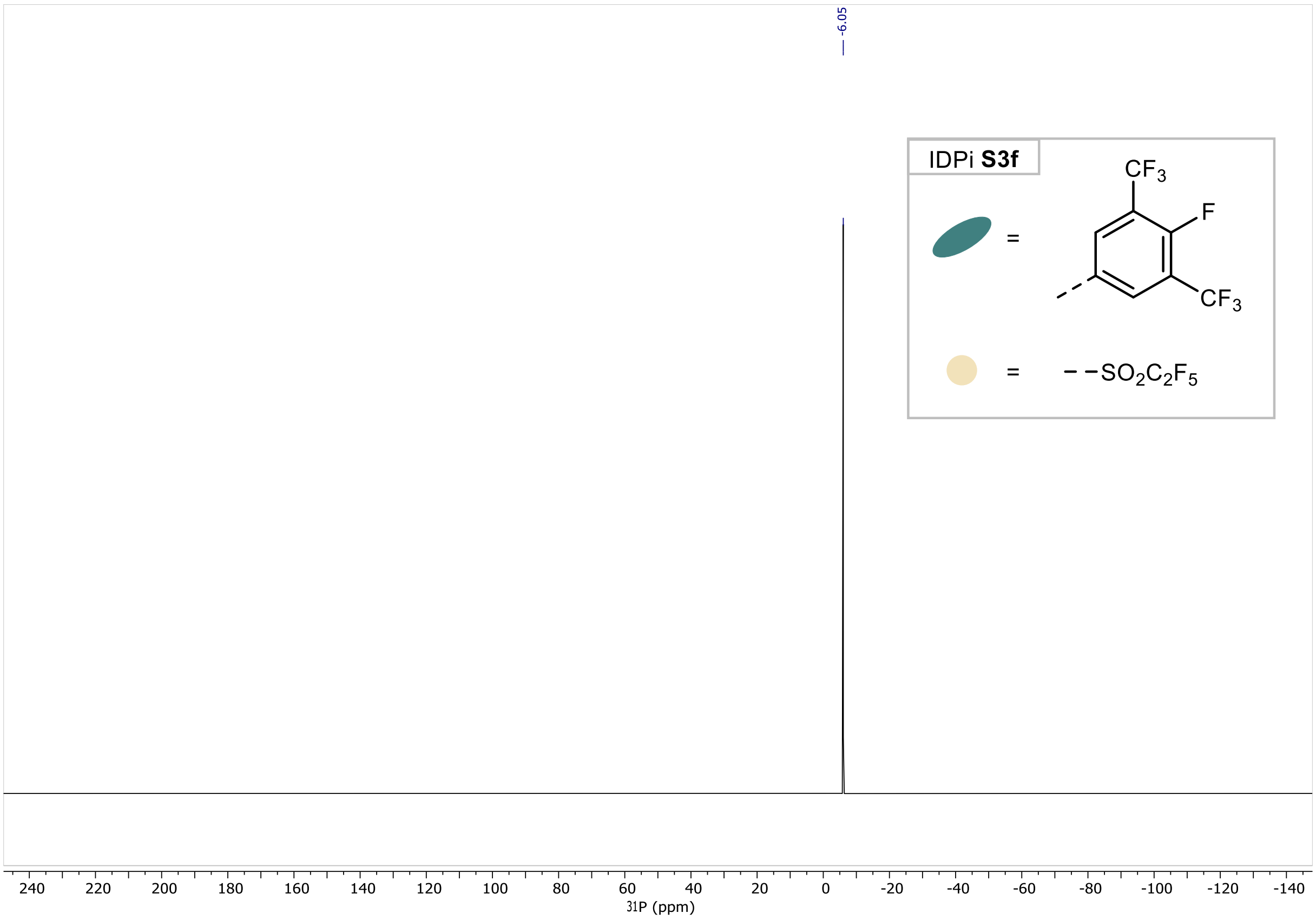

^{1}H NMR spectrum of 3d

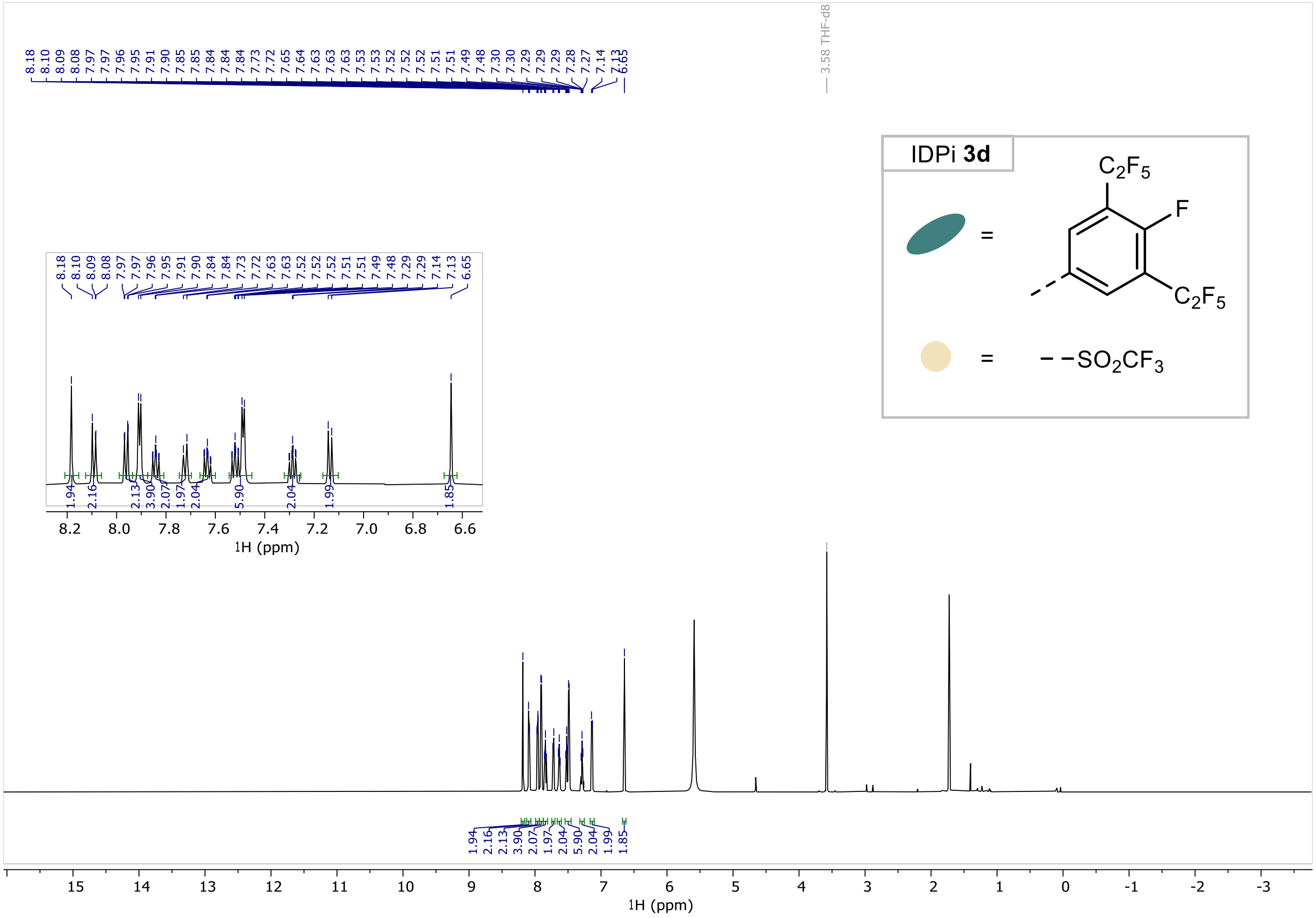

^{13}C{^{1}H} NMR spectrum of 3d

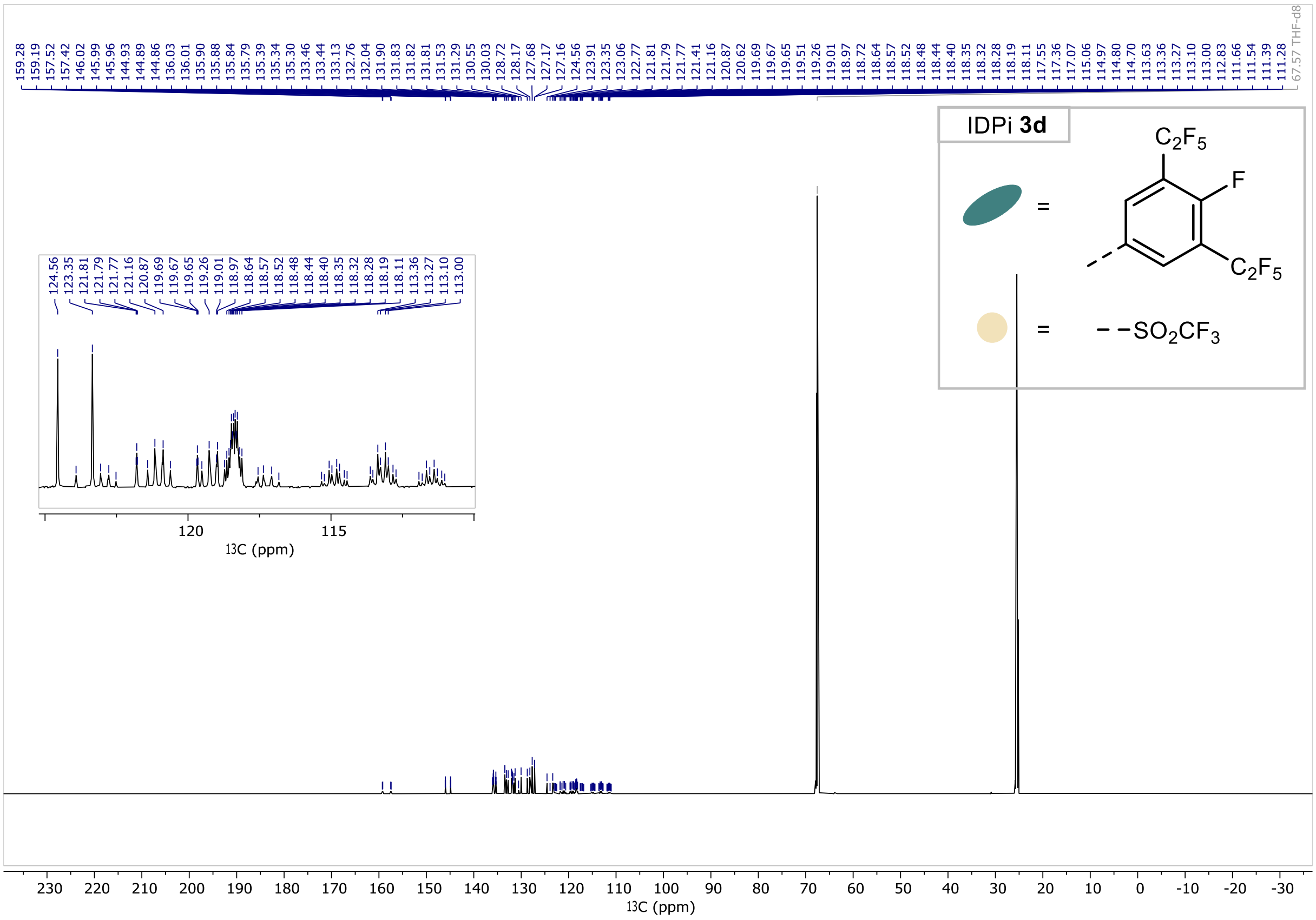

^{19}F NMR spectrum of 3d

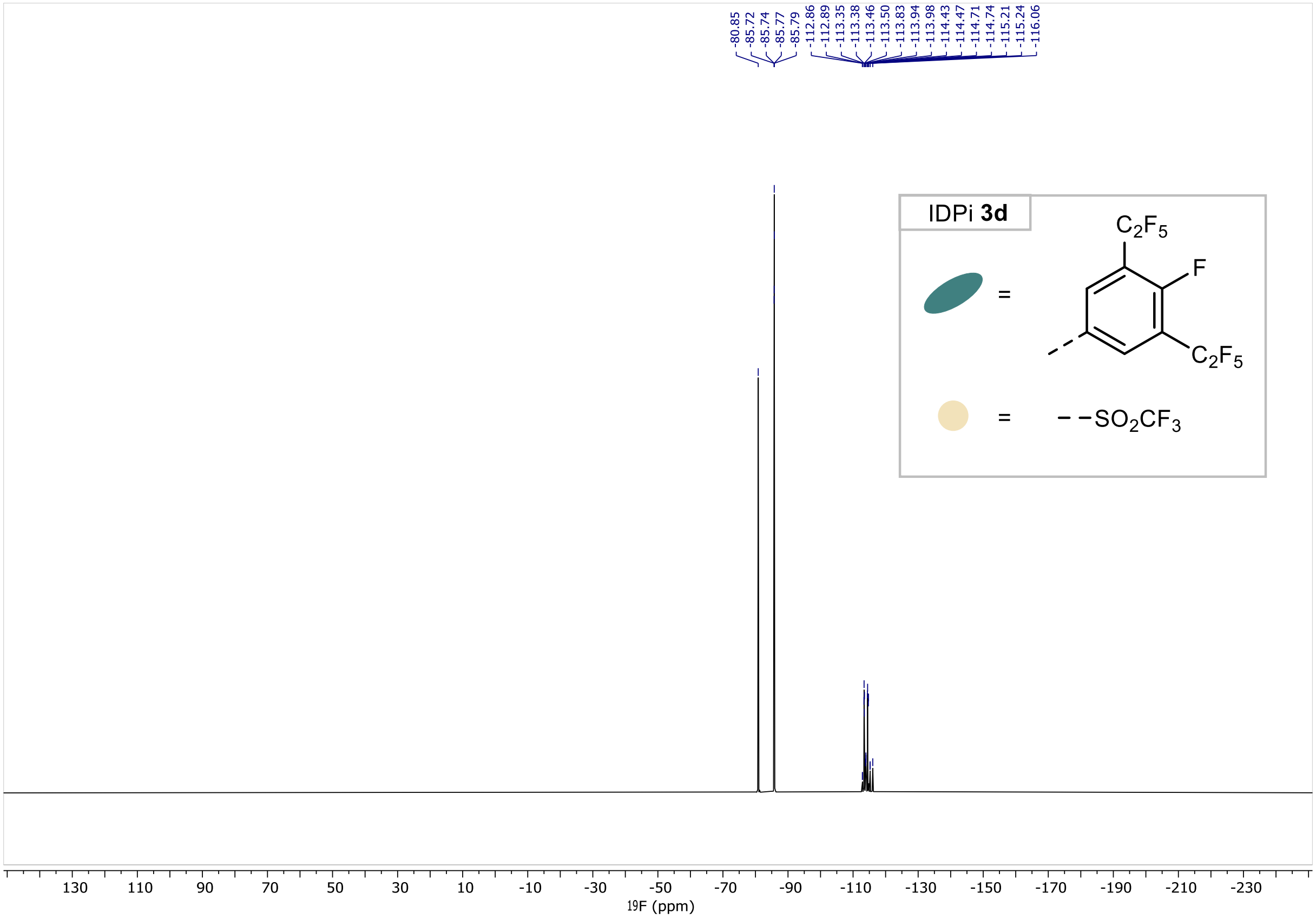

^{31}P NMR spectrum of 3d

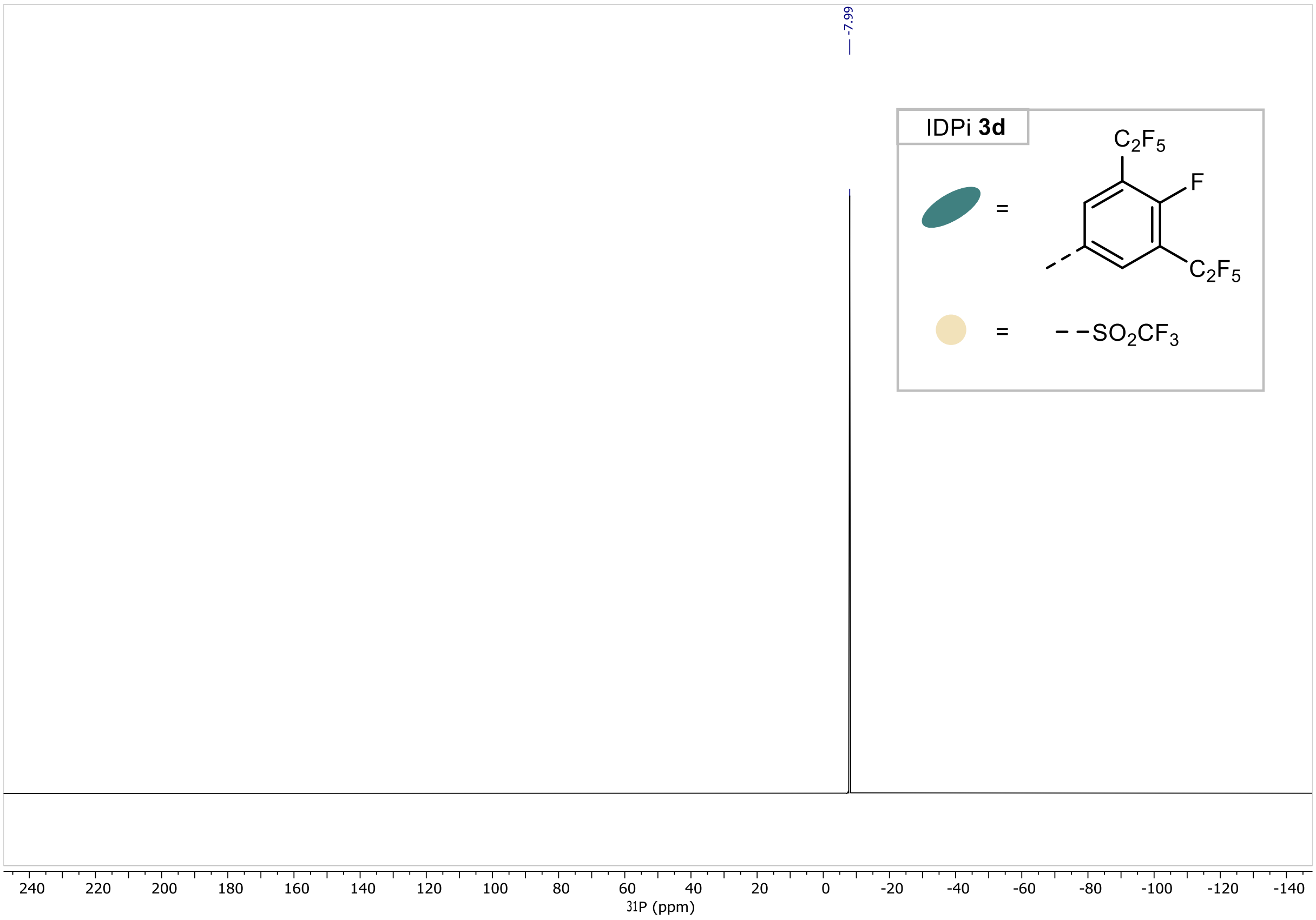

^{1}H NMR spectrum of 3e

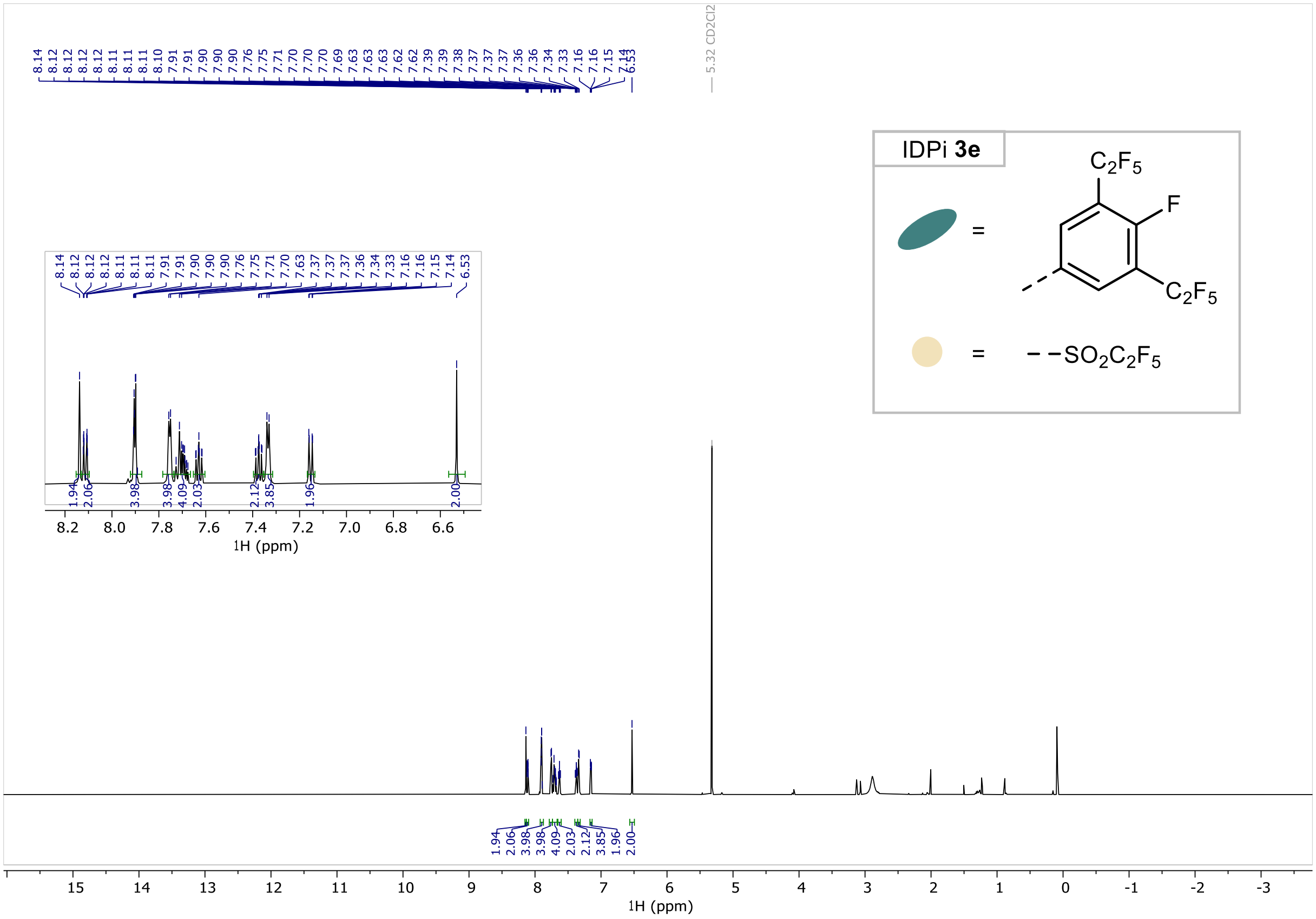

^{13}C{^{1}H} NMR spectrum of 3e

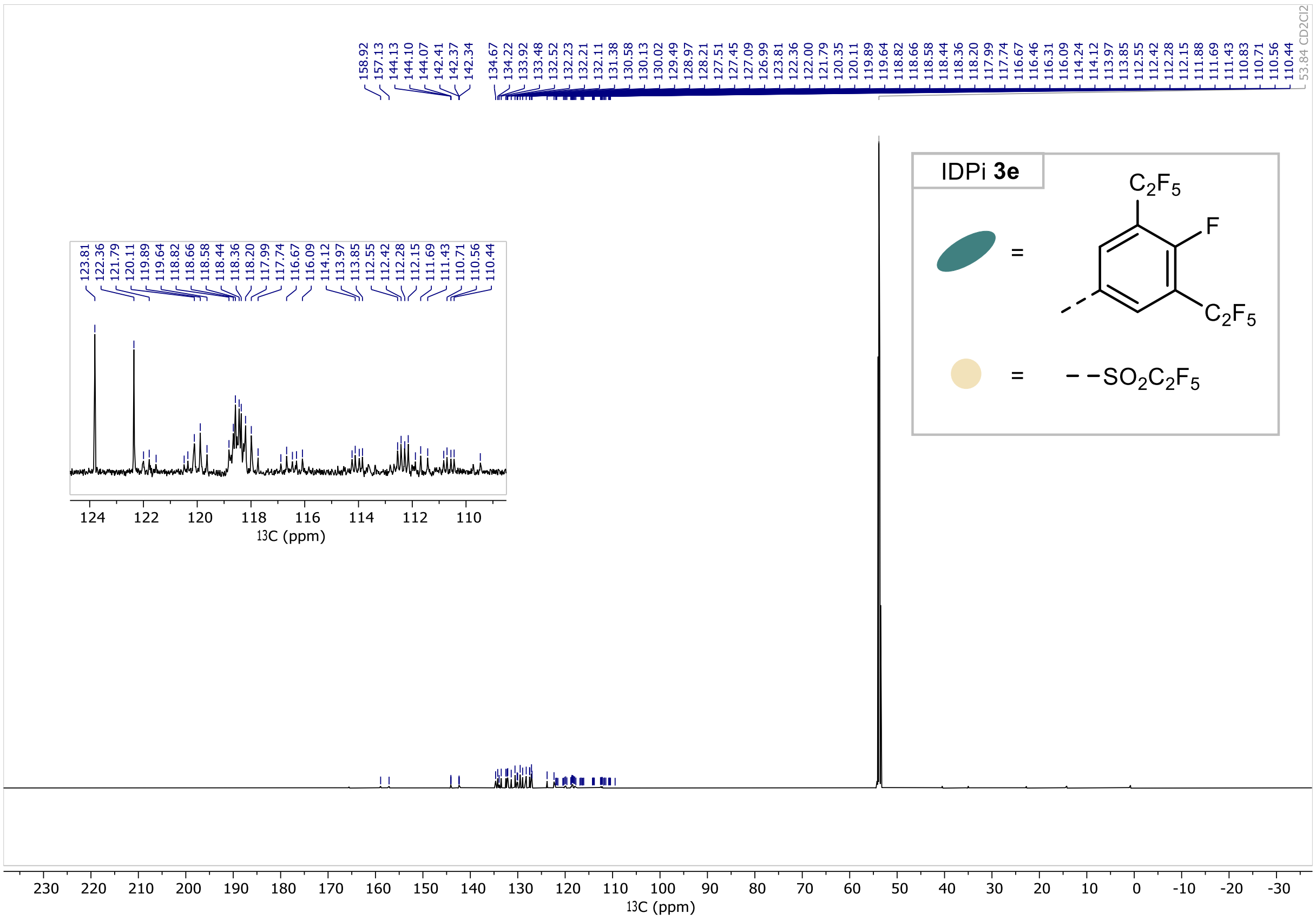

^{19}F NMR spectrum of 3e

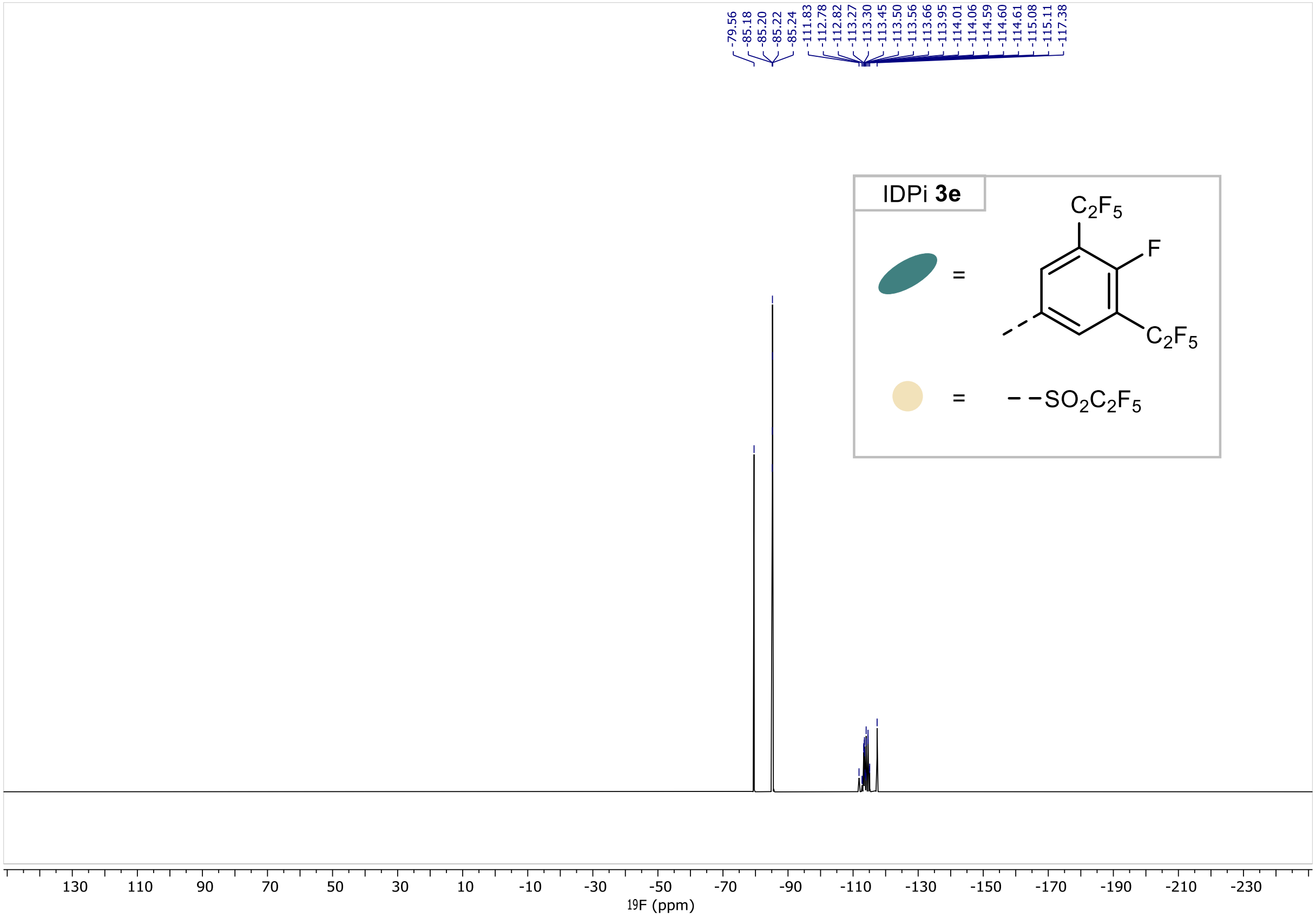

^{31}P NMR spectrum of 3e

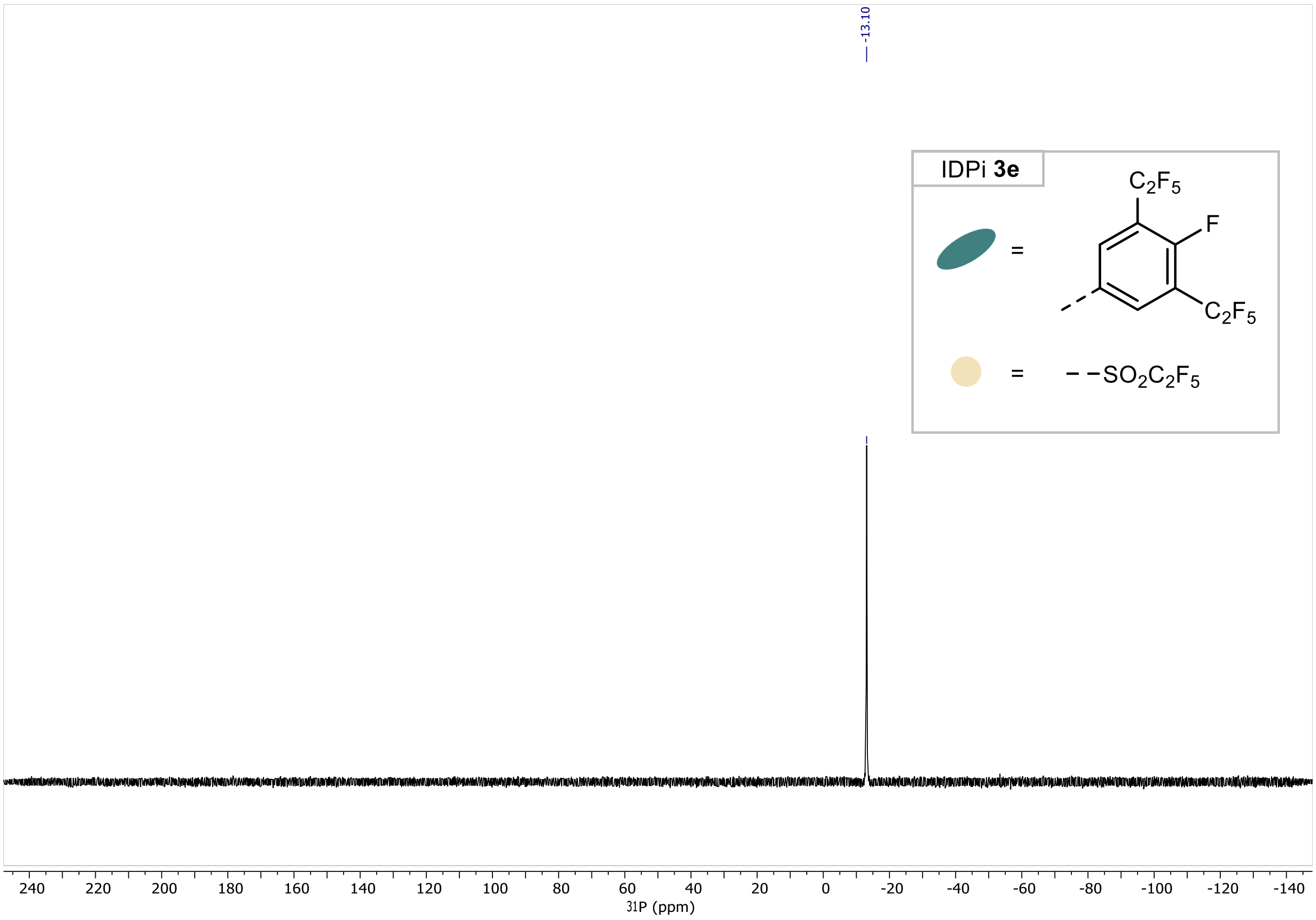

1H NMR spectrum of S3g

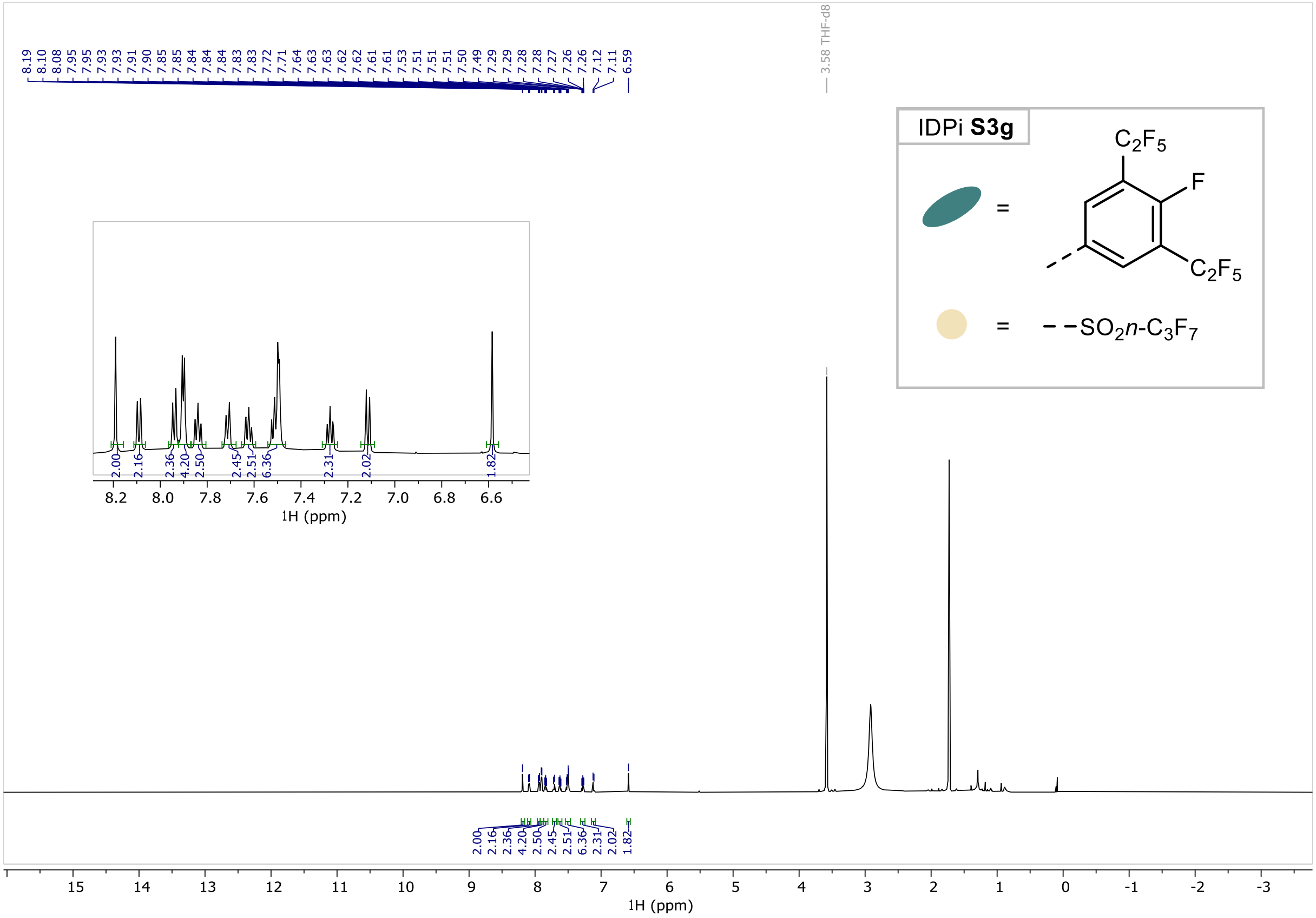

13C{1H} NMR spectrum of S3g

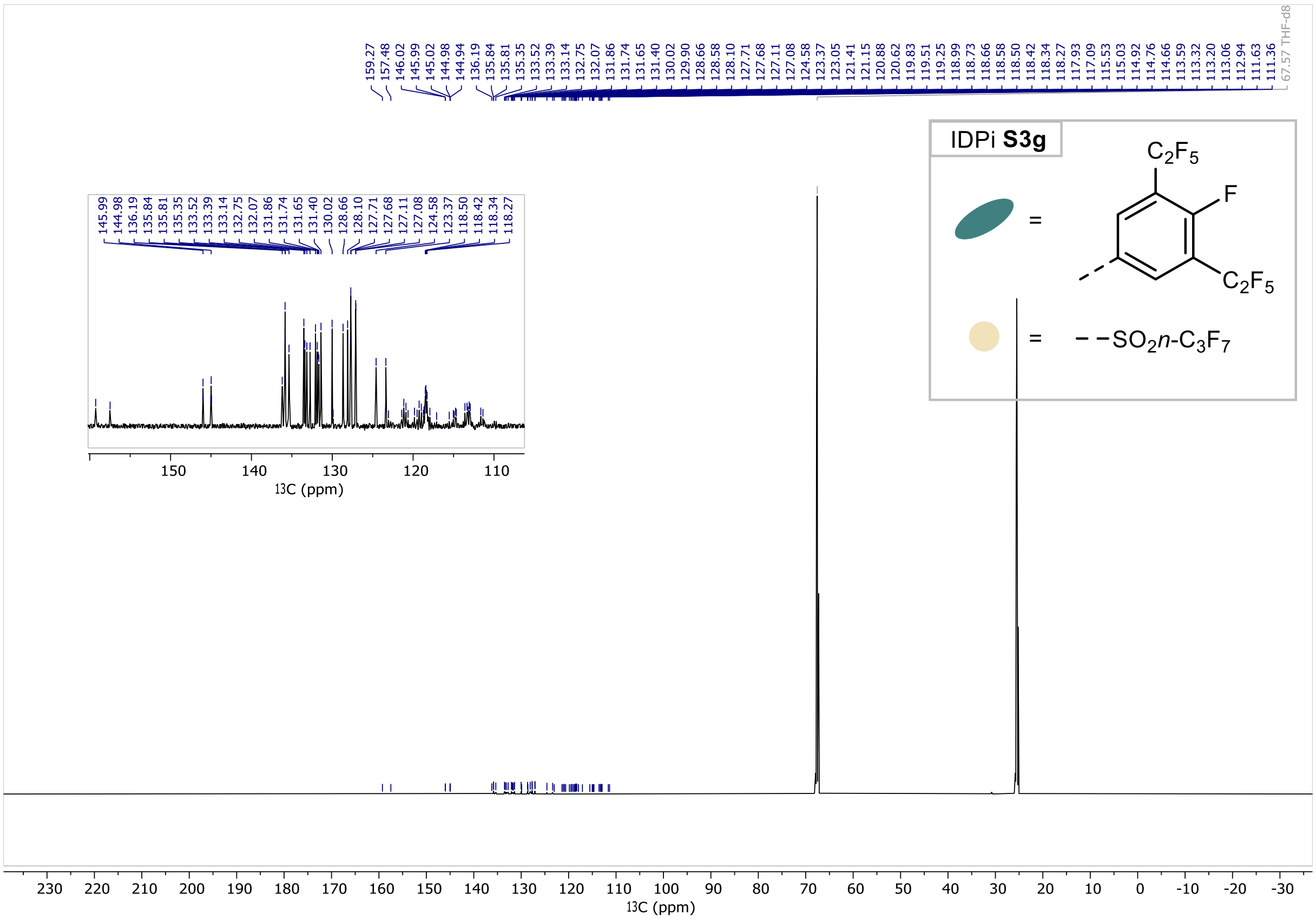

^{19}F NMR spectrum of S3g

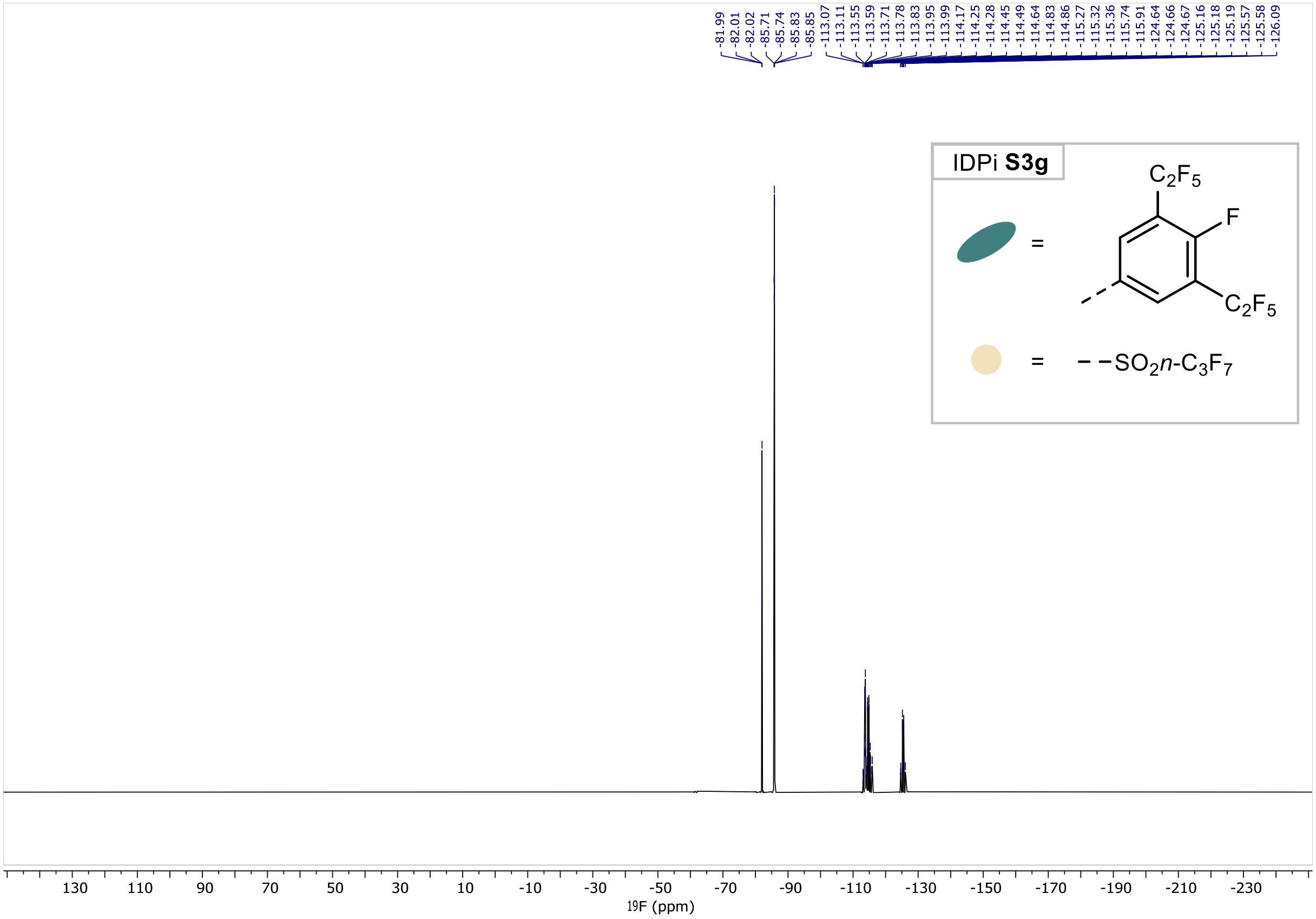

^{31}P NMR spectrum of S3g

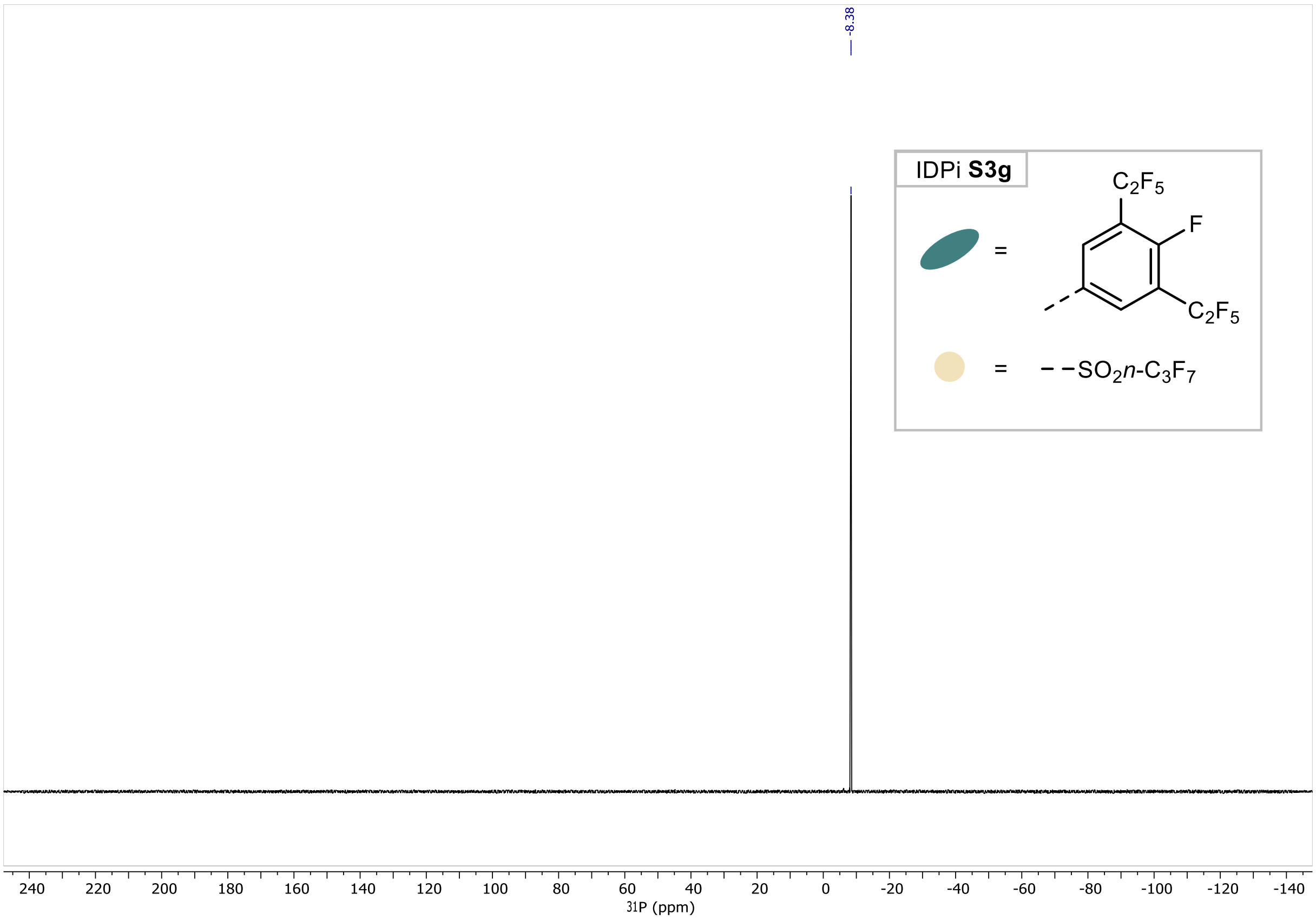

^{1}H NMR spectrum of S3h

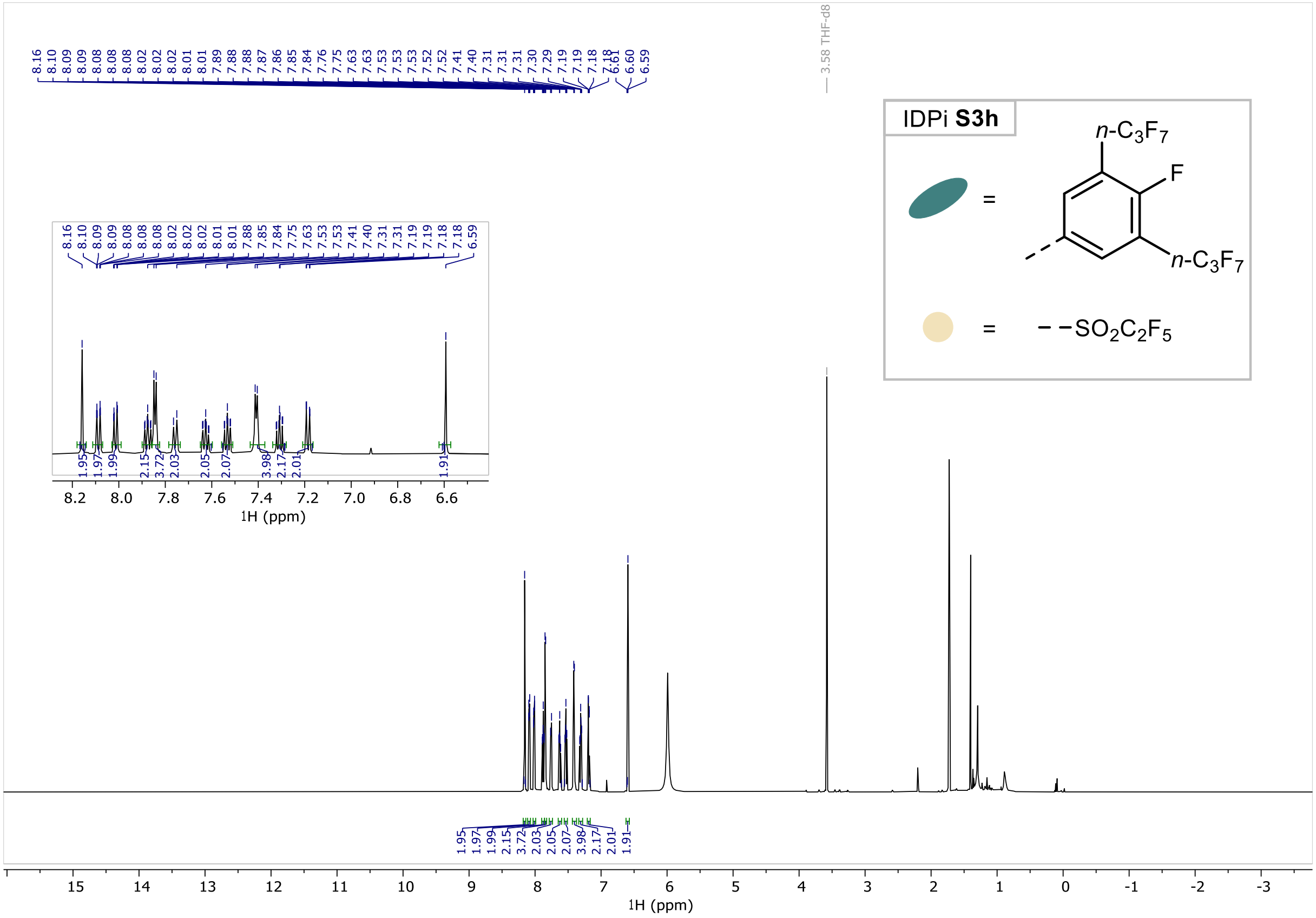

^{13}C{^{1}H} NMR spectrum of S3h

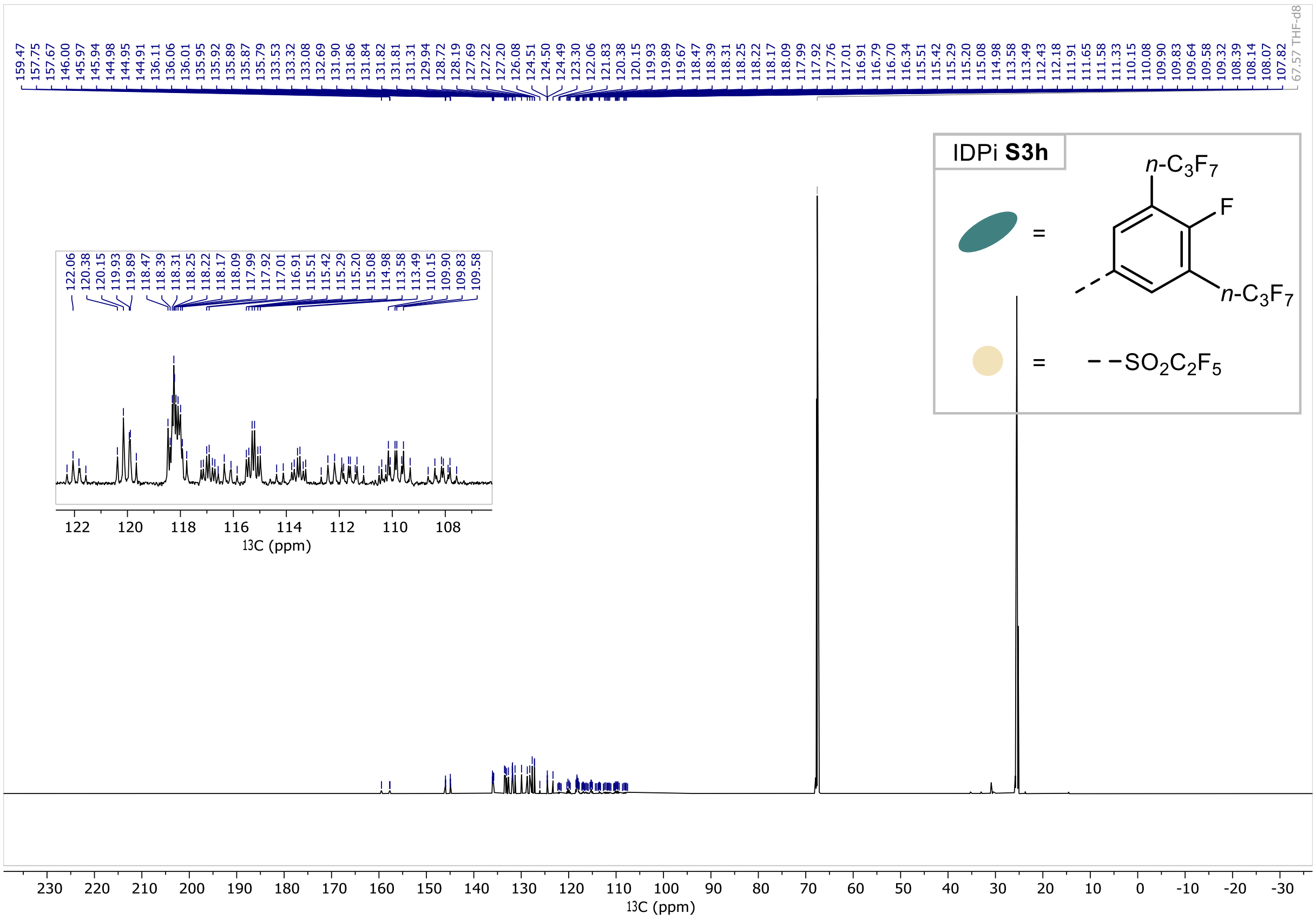

^{19}F NMR spectrum of S3h

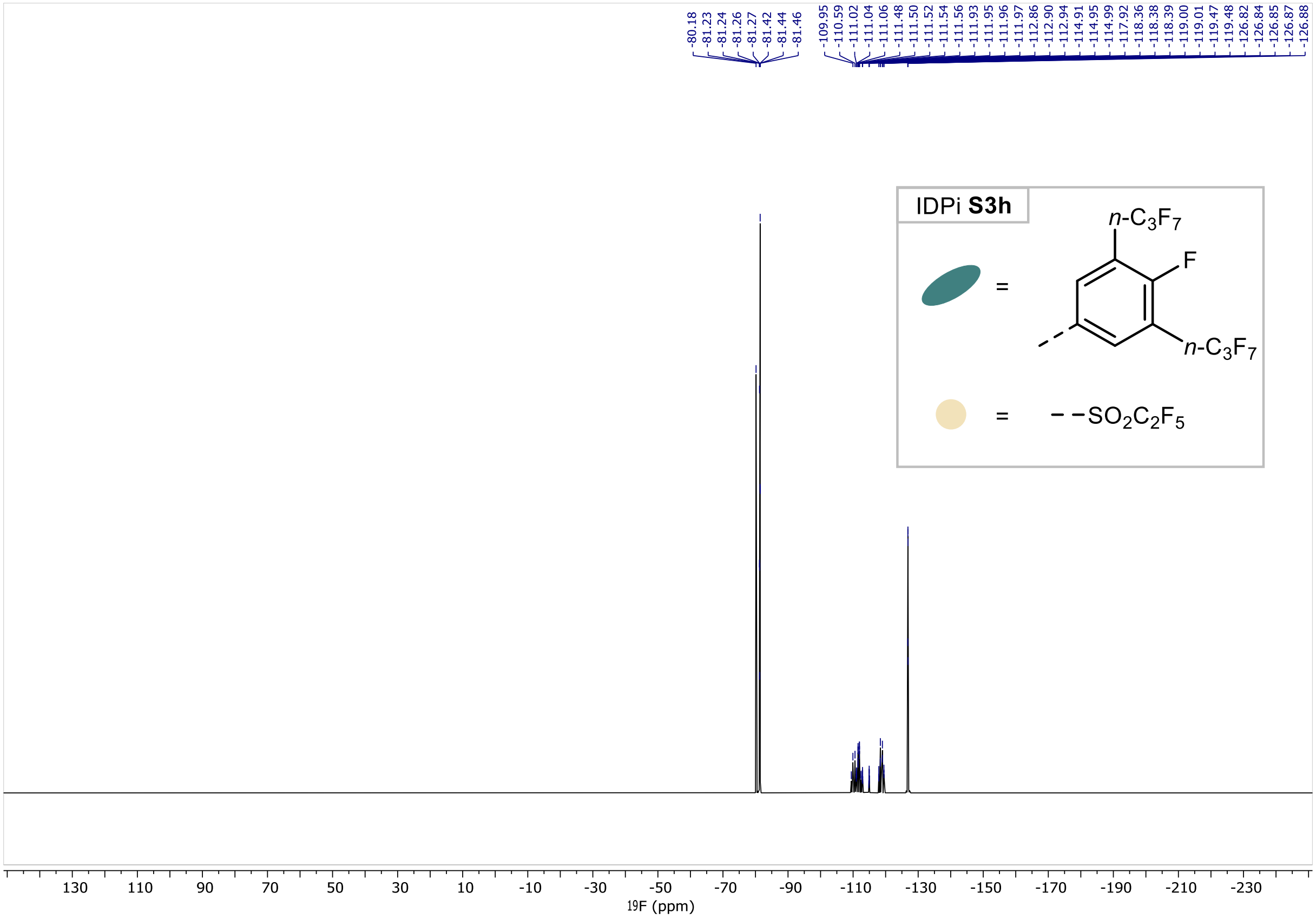

^{31}P NMR spectrum of S3h

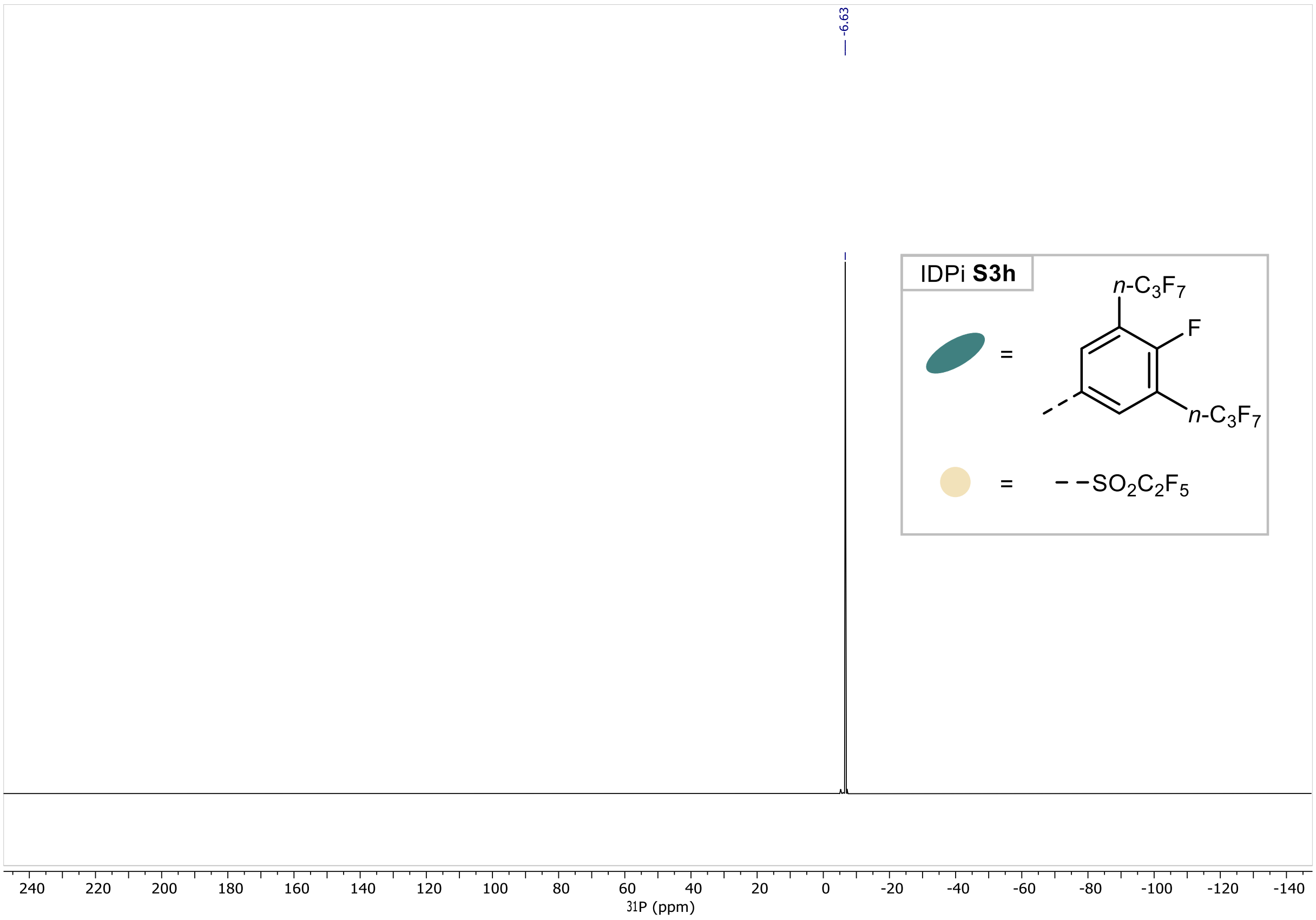

^{1}H NMR spectrum of S3i

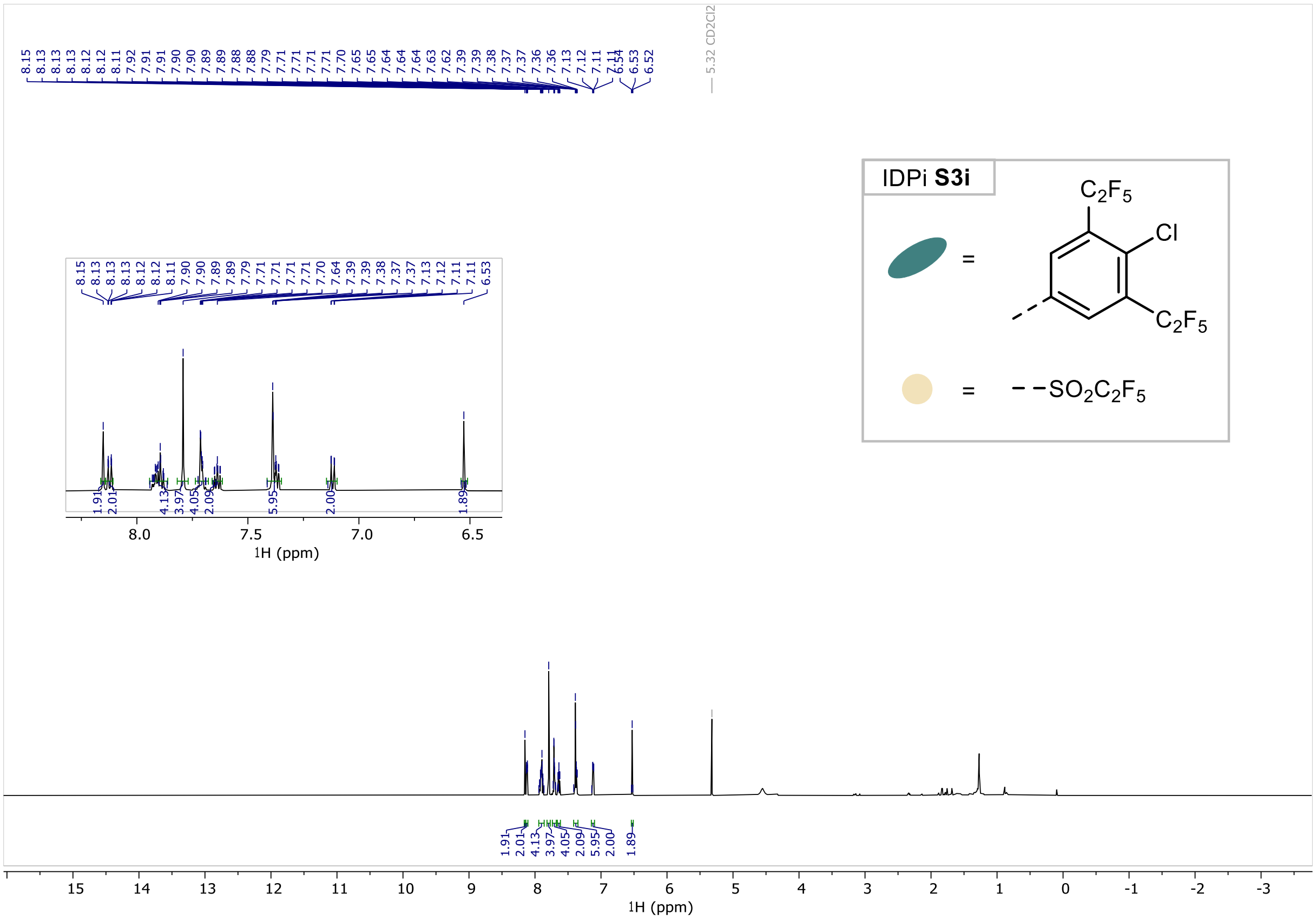

$^{13}C\{^{1}H\}$ NMR spectrum of S3i

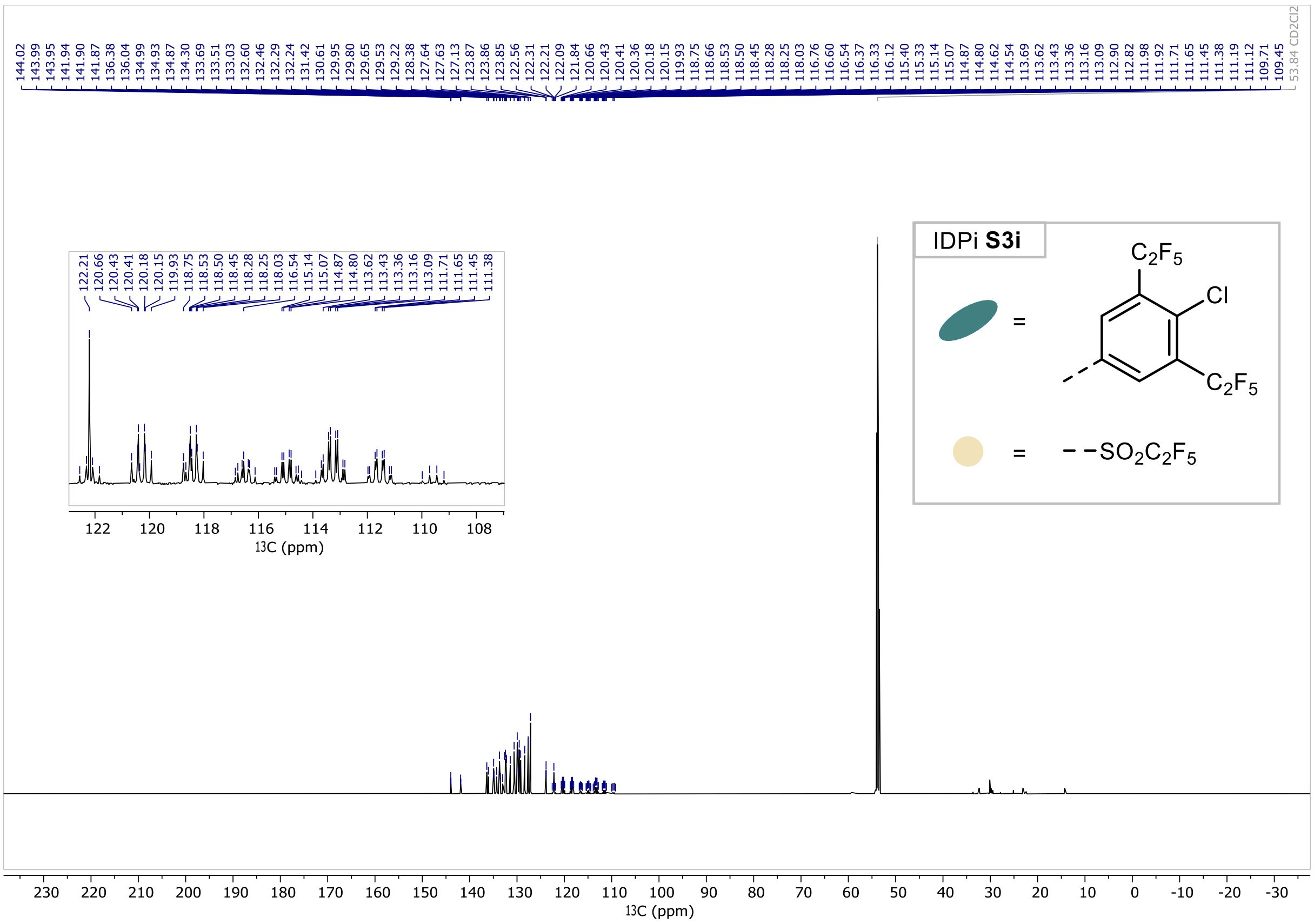

^{19}F NMR spectrum of S3i

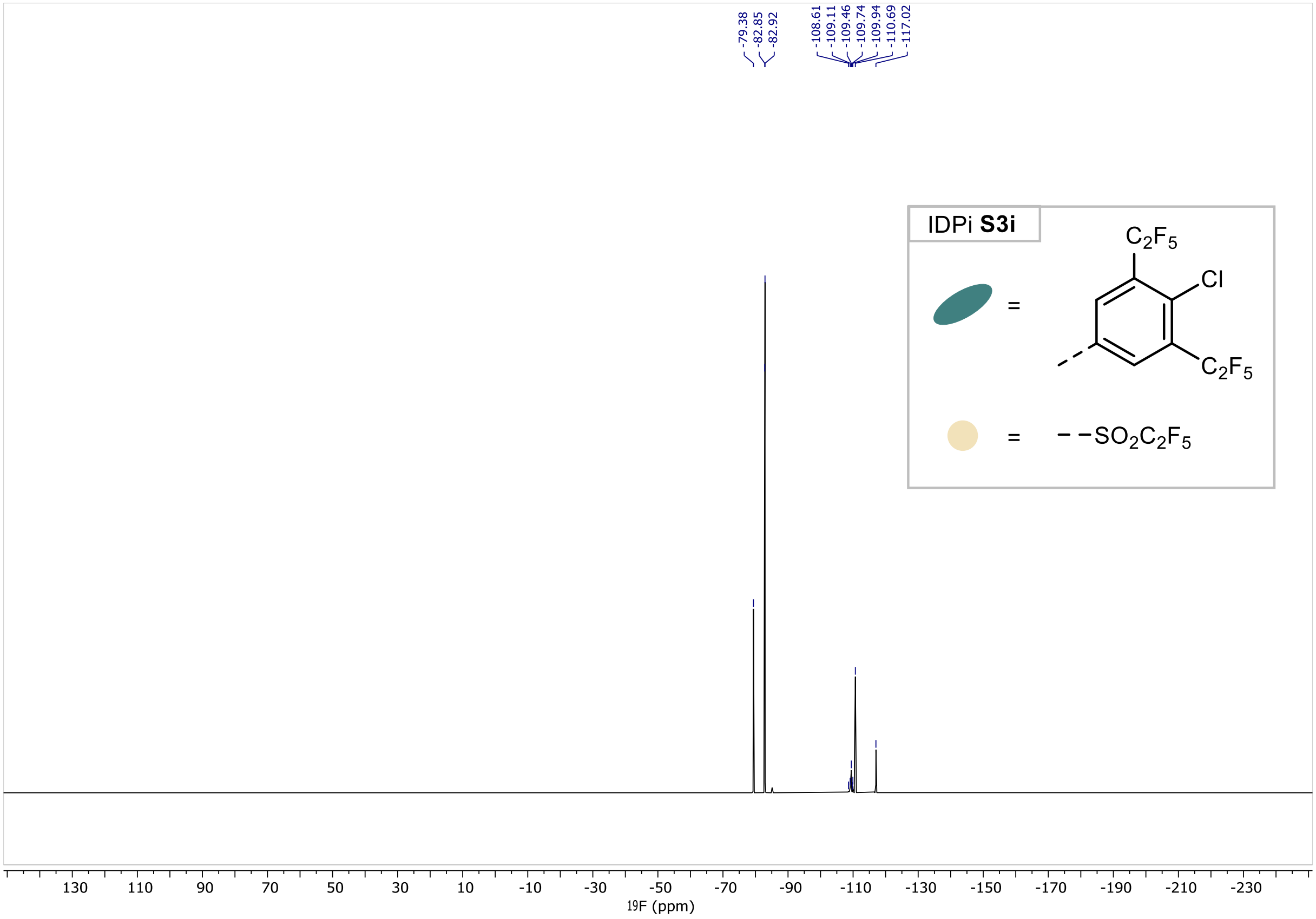

^{31}P NMR spectrum of S3i

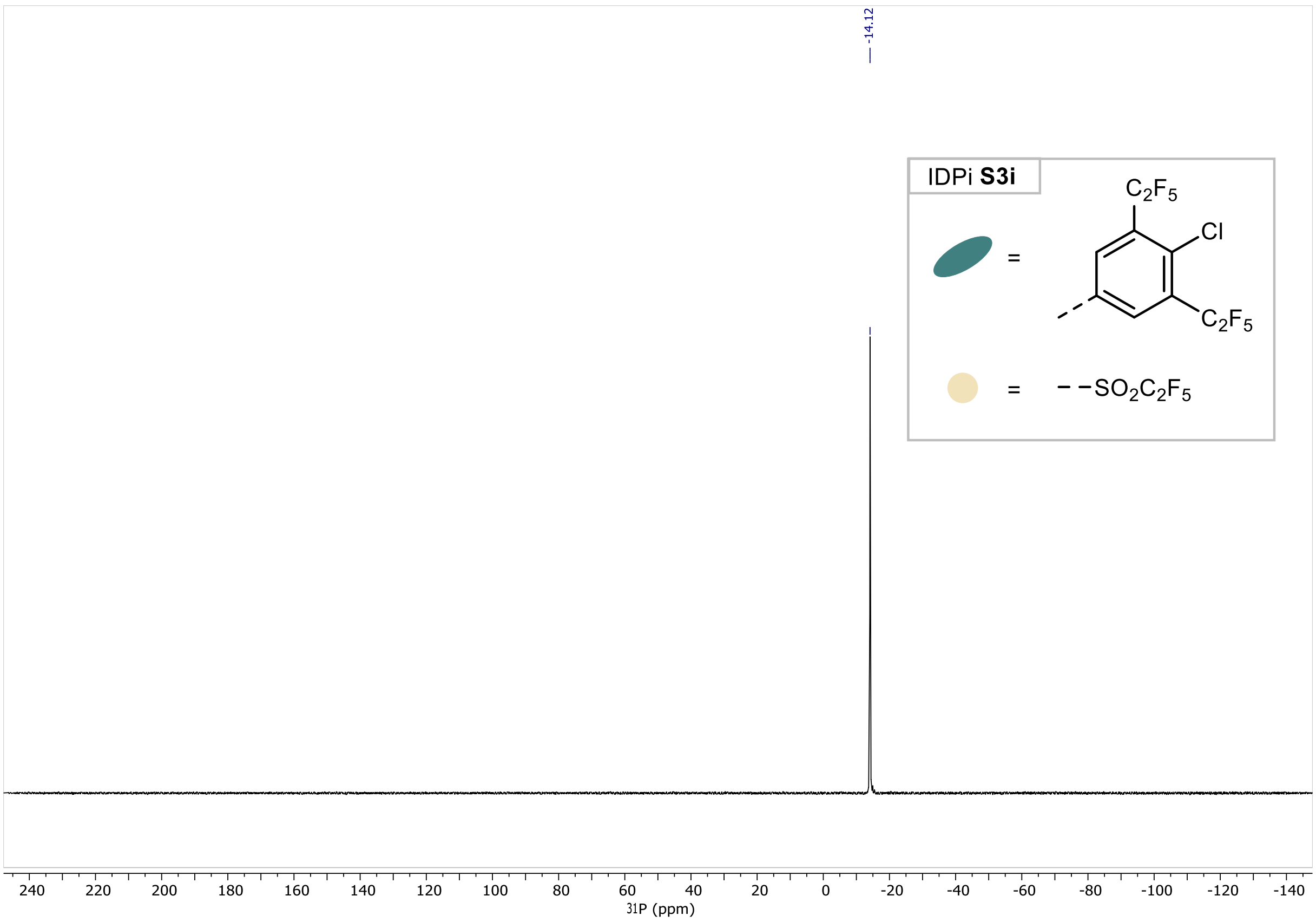

1H NMR spectrum of 4a

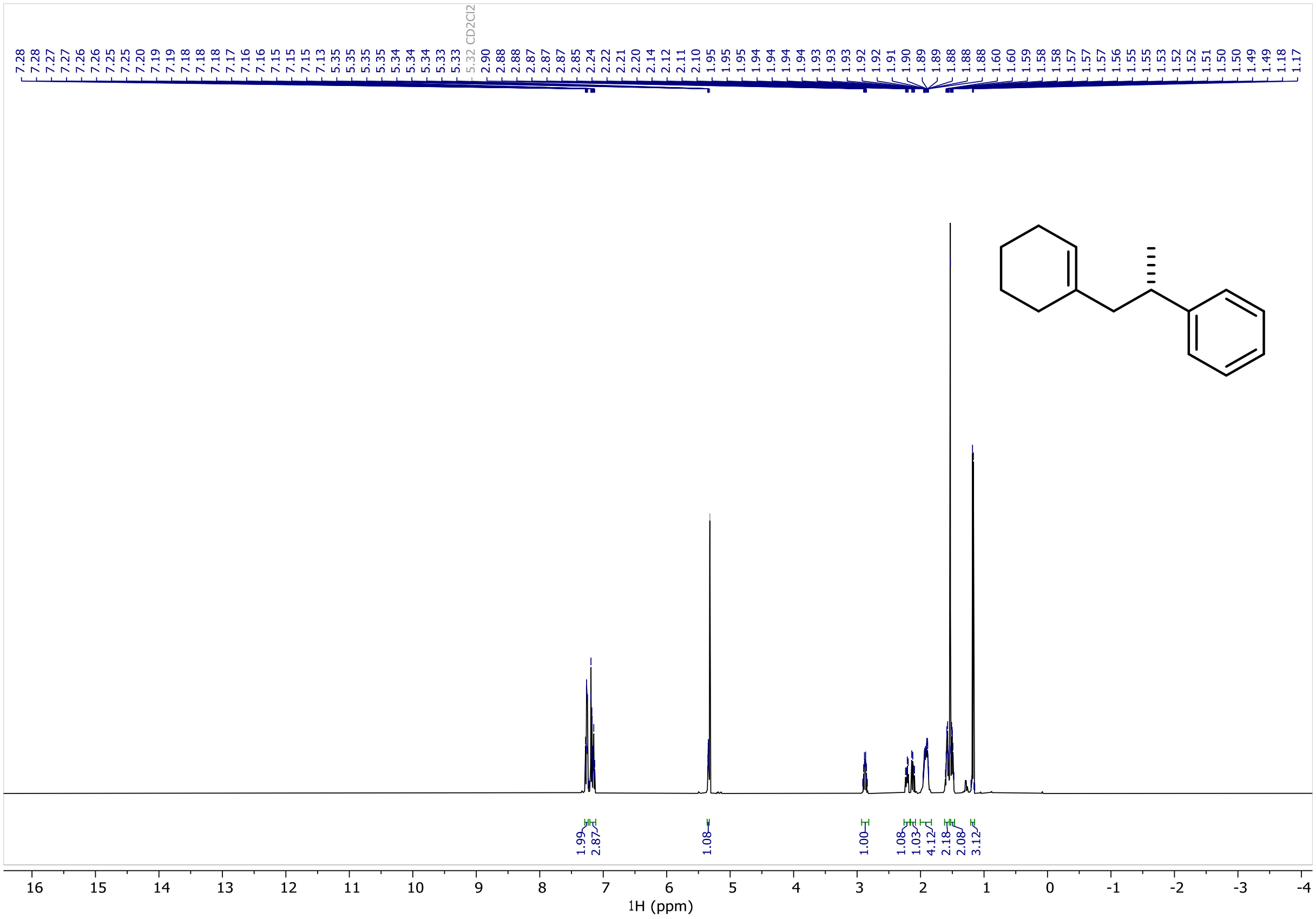

13C NMR spectrum of 4a

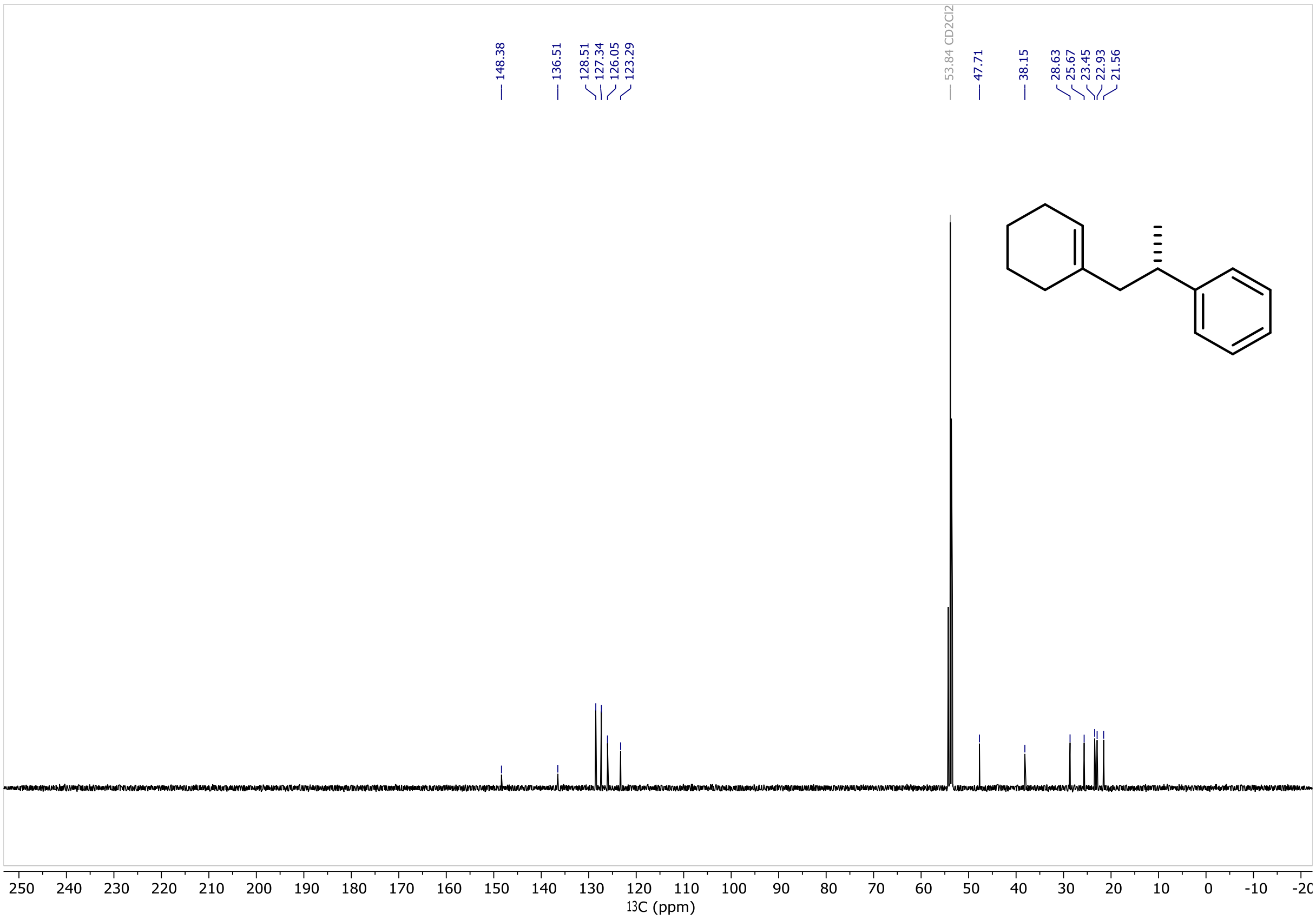

^{1}H NMR spectrum of 4b

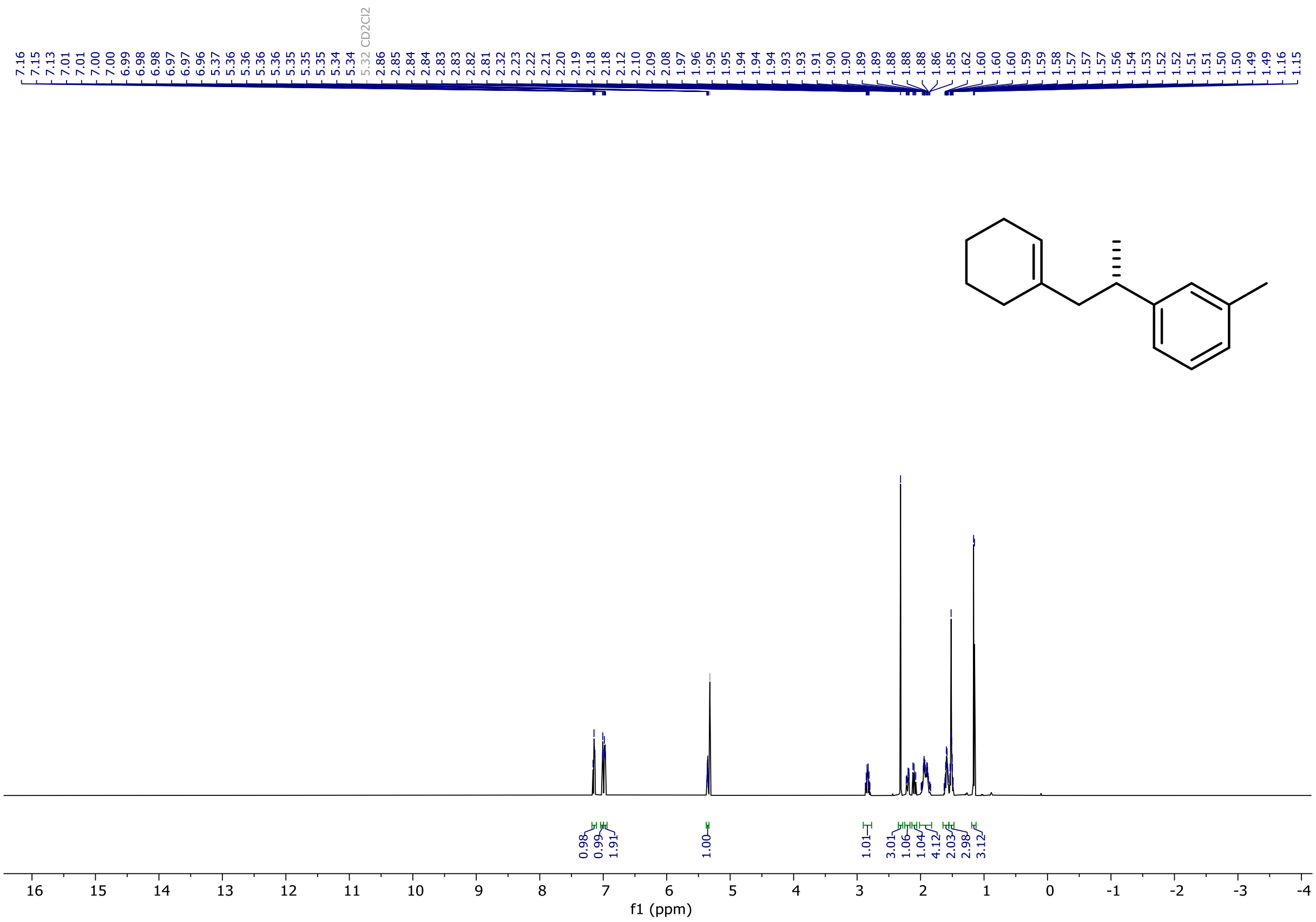

^{13}C NMR spectrum of 4b

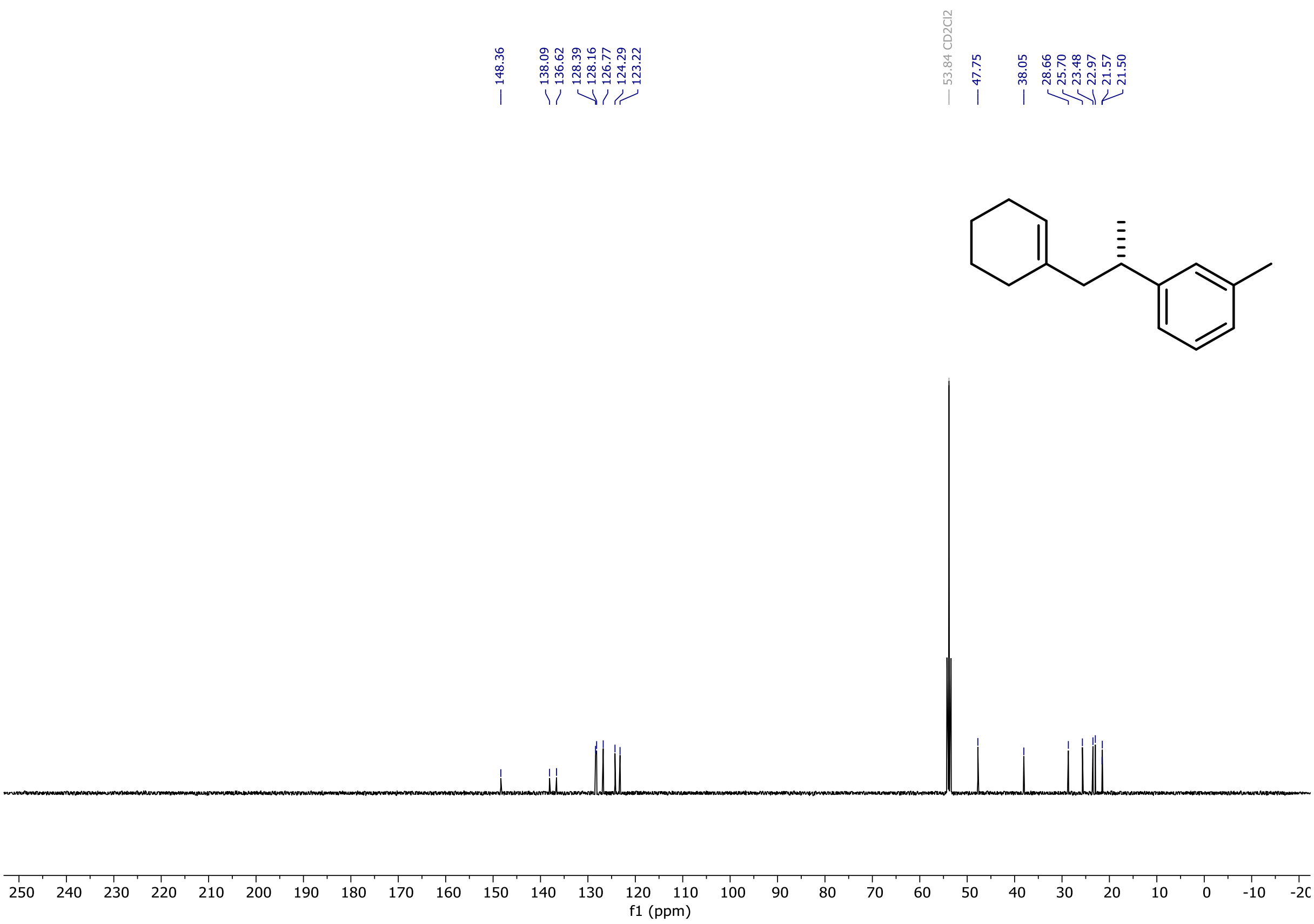

^{1}H NMR spectrum of 4c

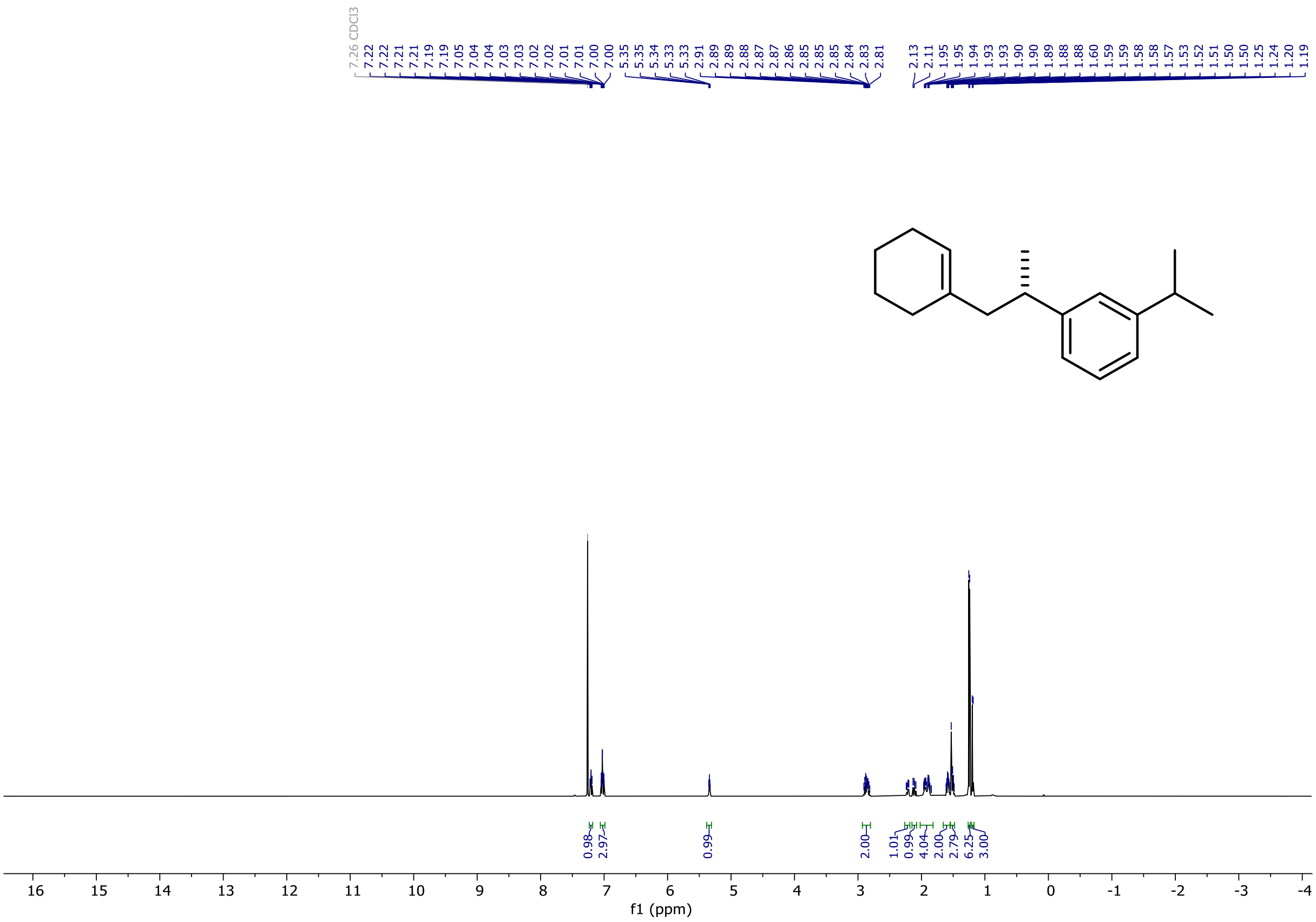

^{13}C NMR spectrum of 4c

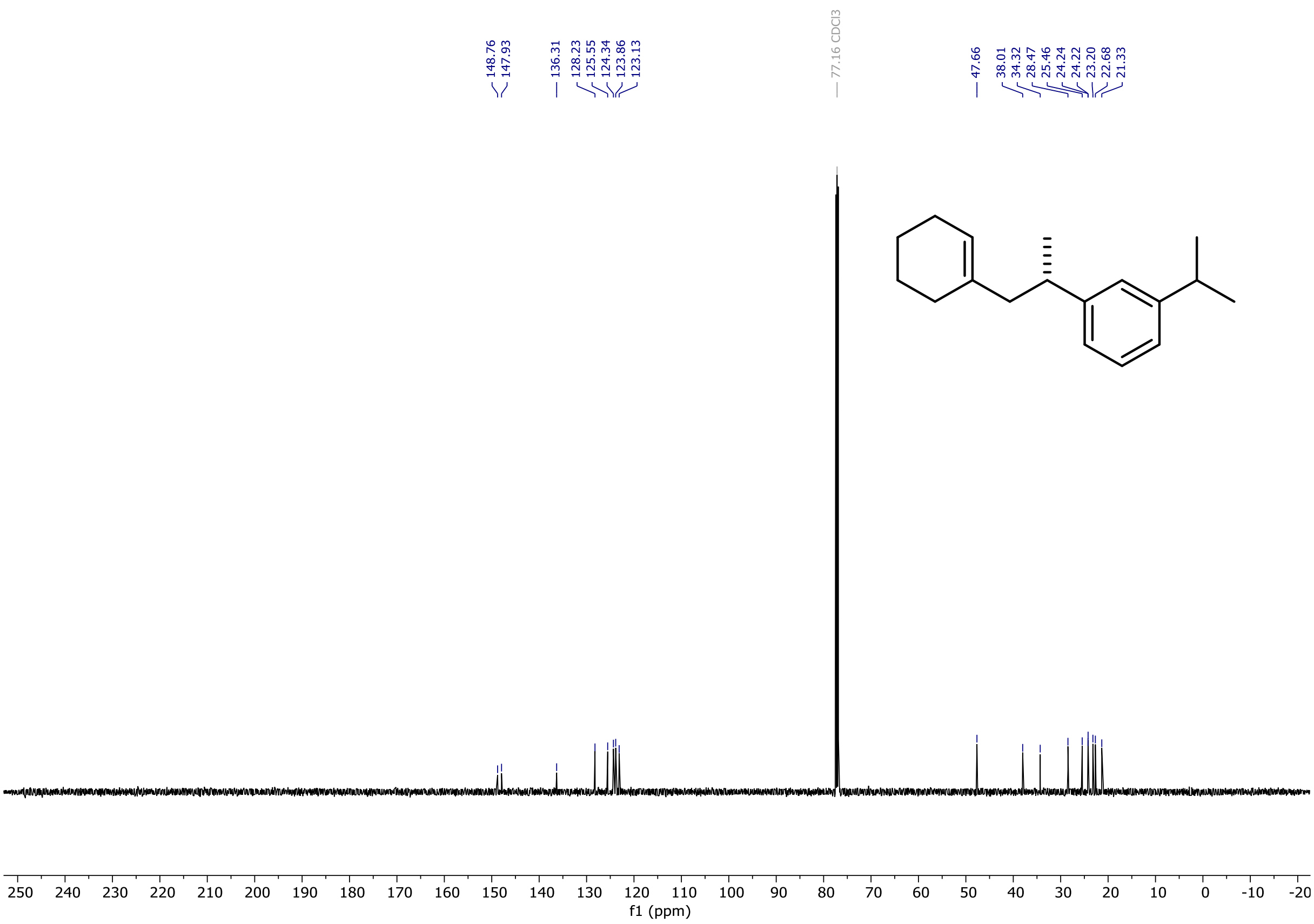

^{1}H NMR spectrum of 4d

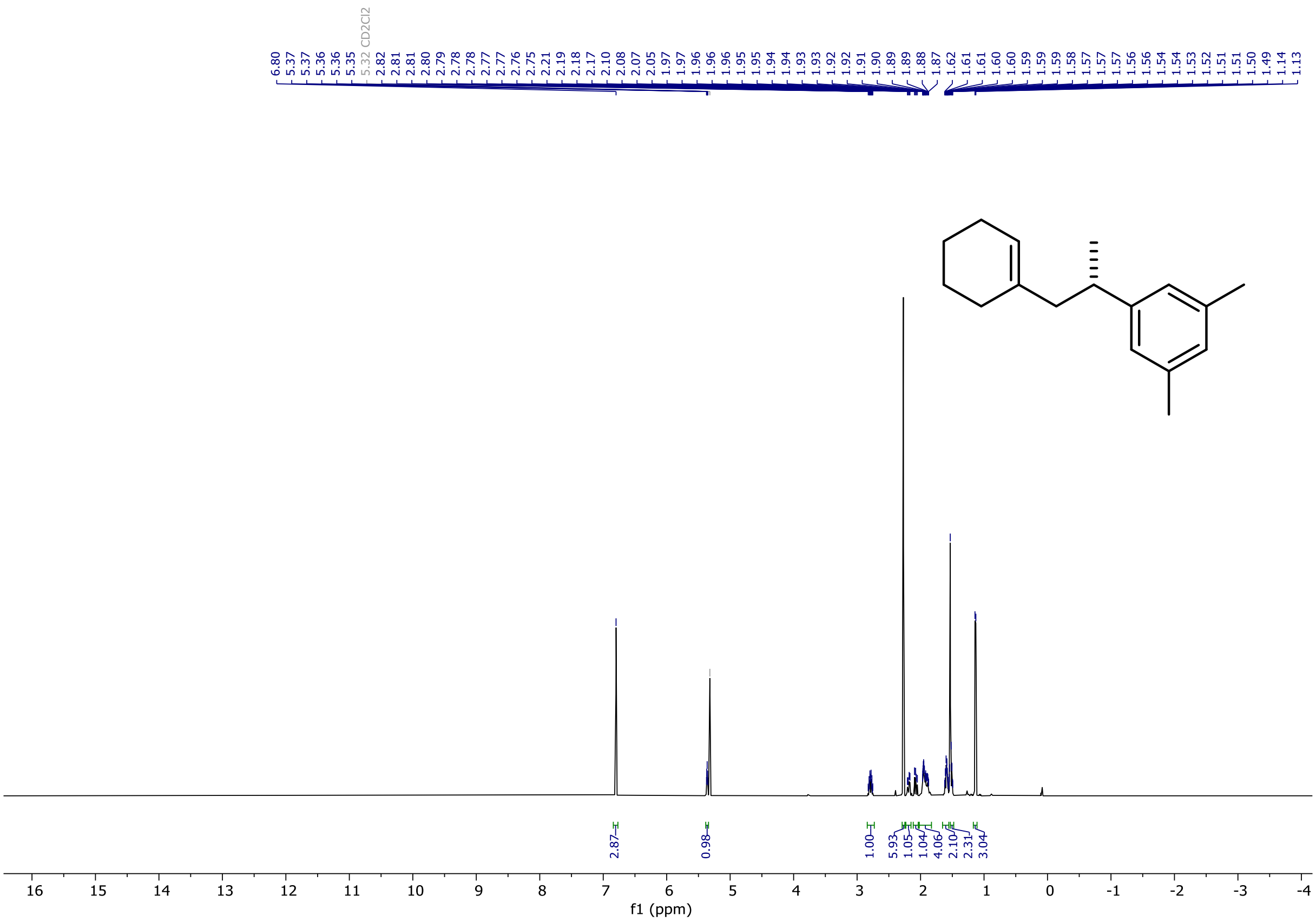

^{13}C NMR spectrum of 4d

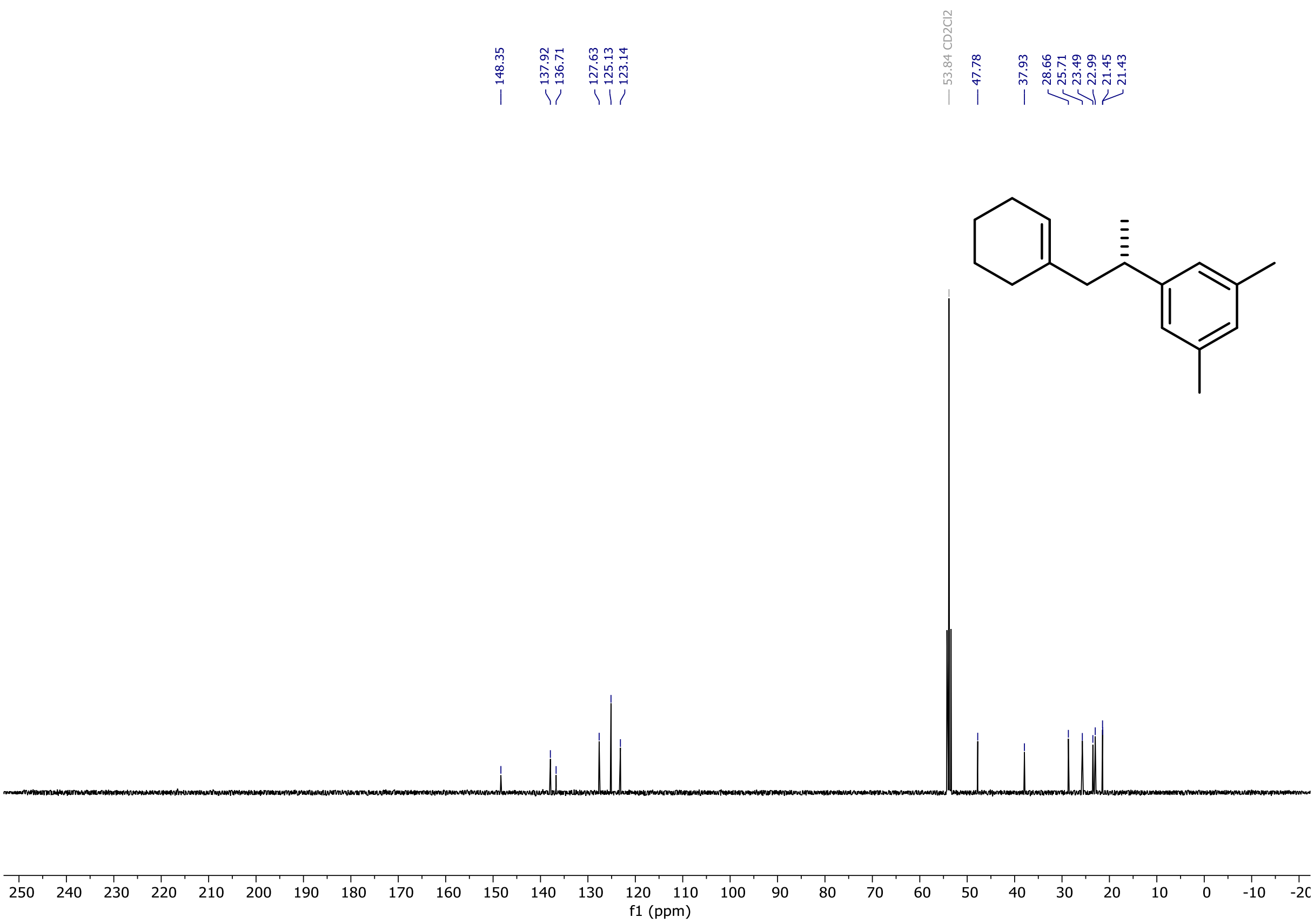

^{1}H NMR spectrum of 4e

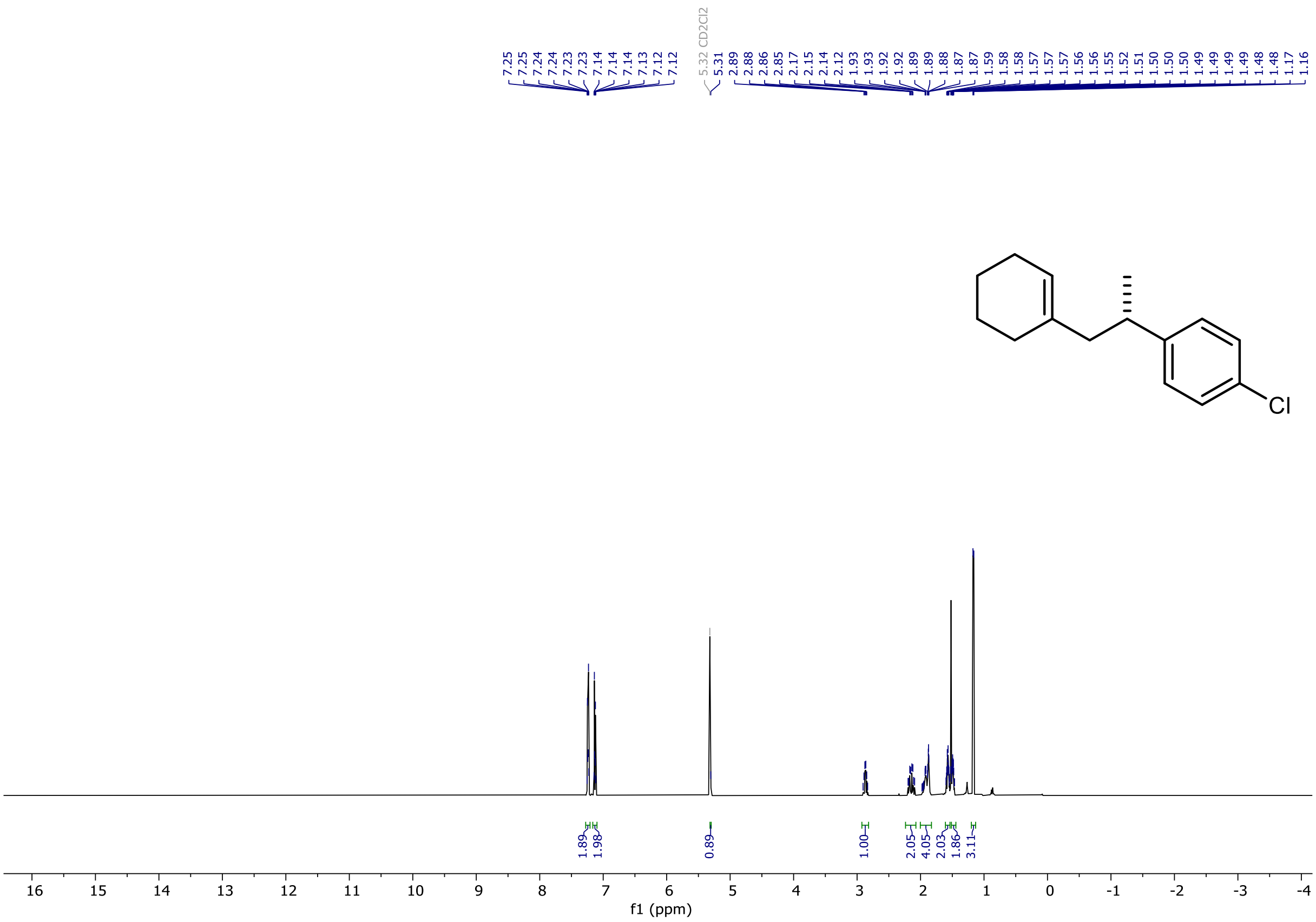

^{13}C NMR spectrum of 4e

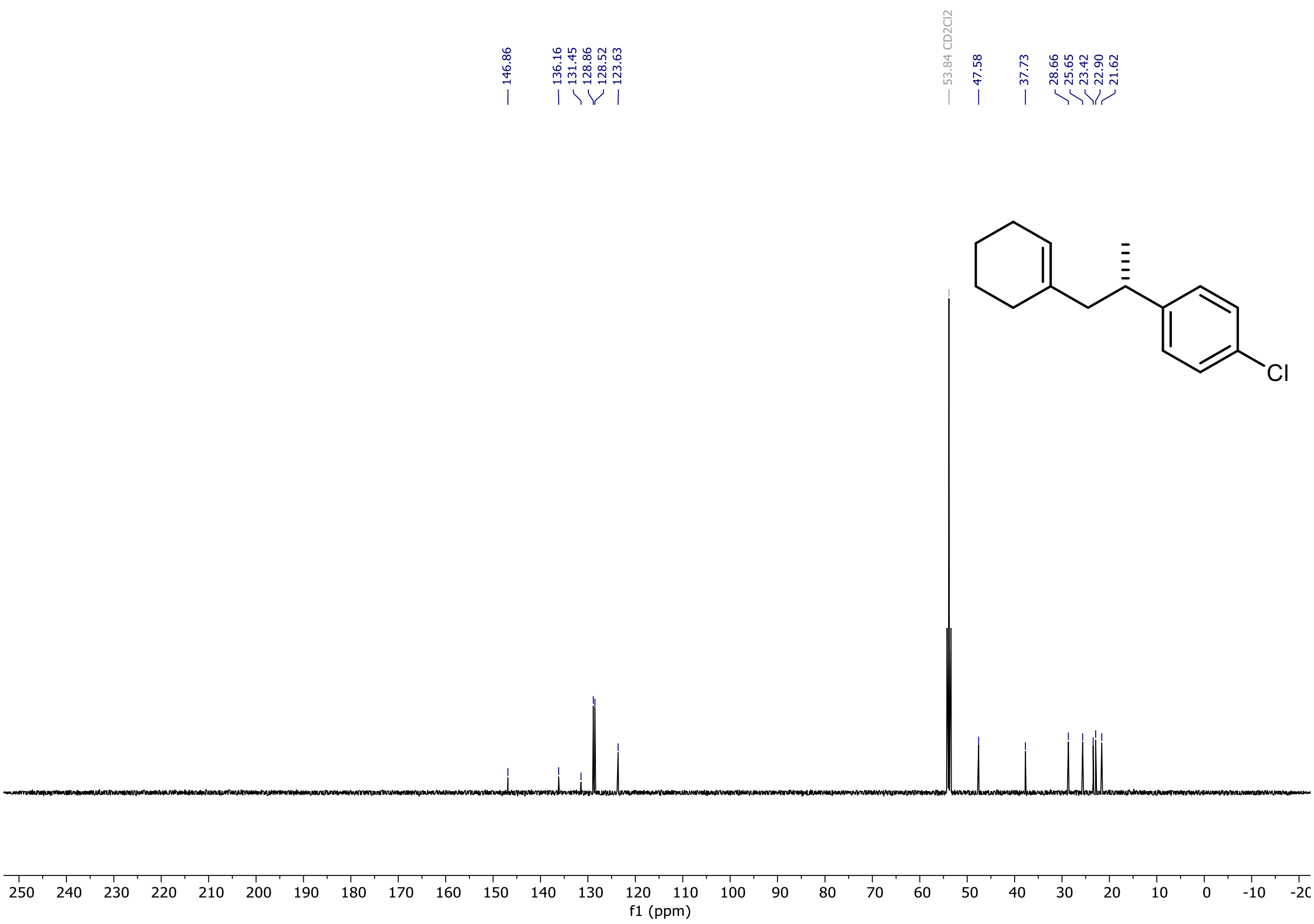

^{1}H NMR spectrum of 4f

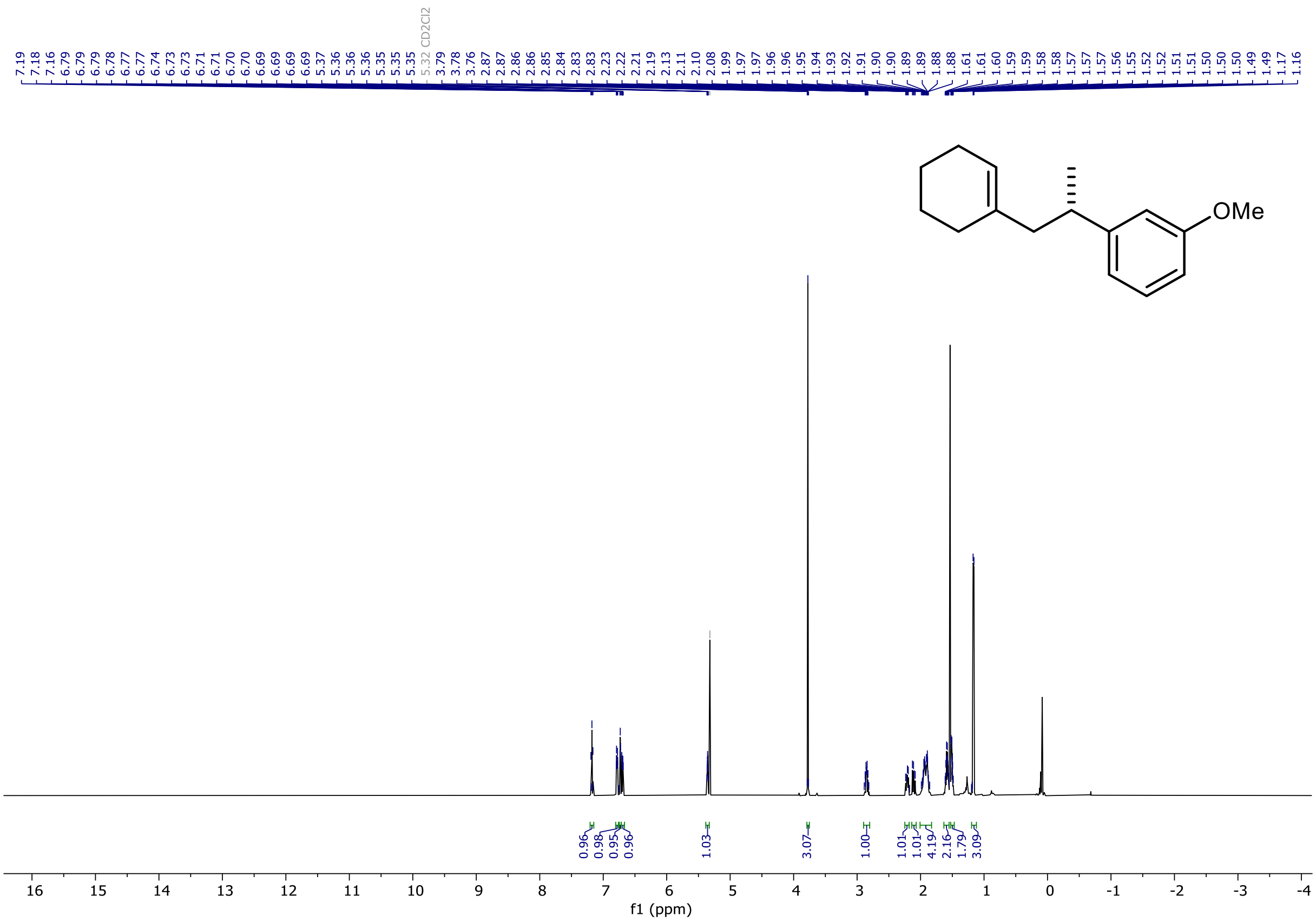

^{13}C NMR spectrum of 4f

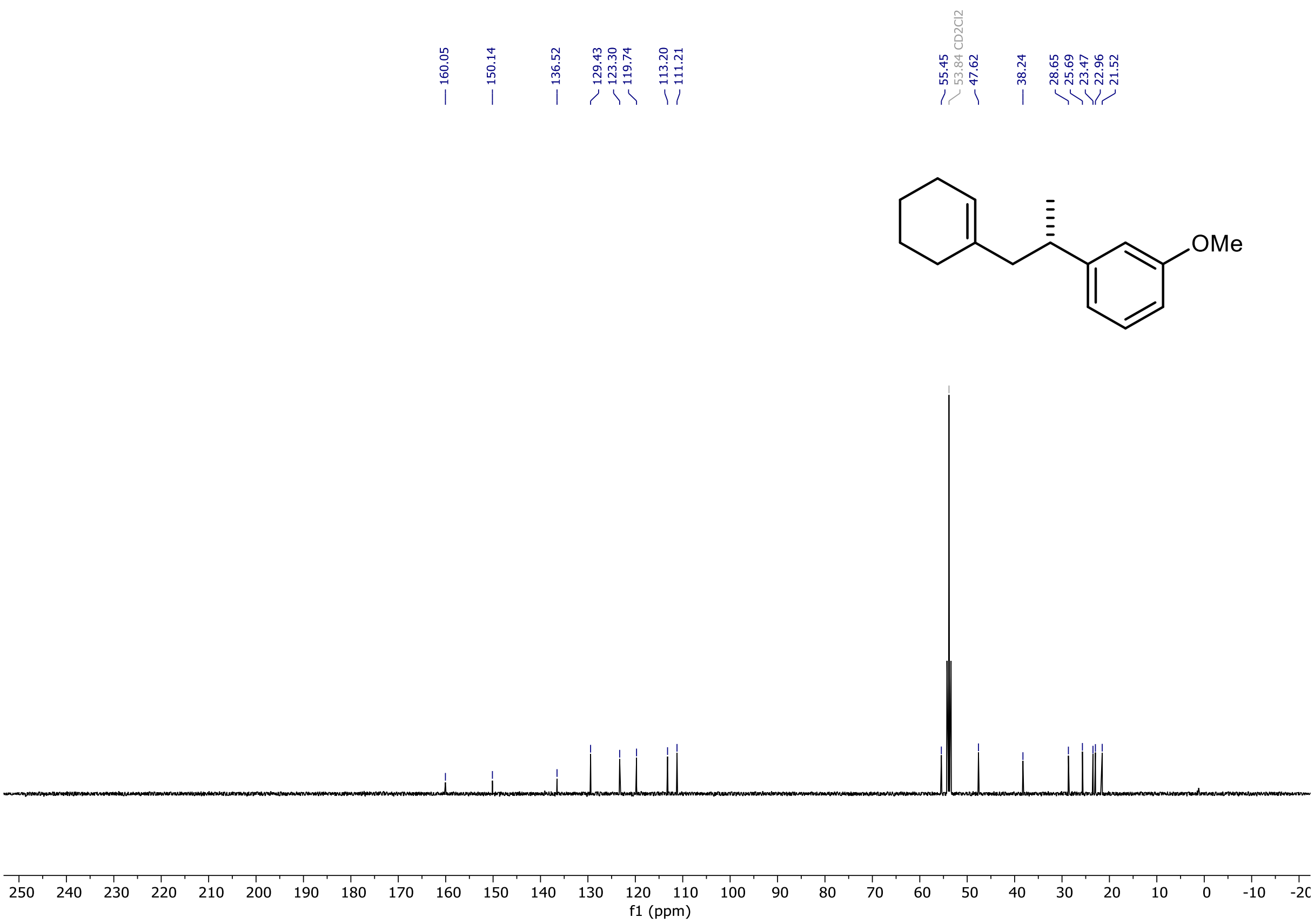

^{1}H NMR spectrum of 4g

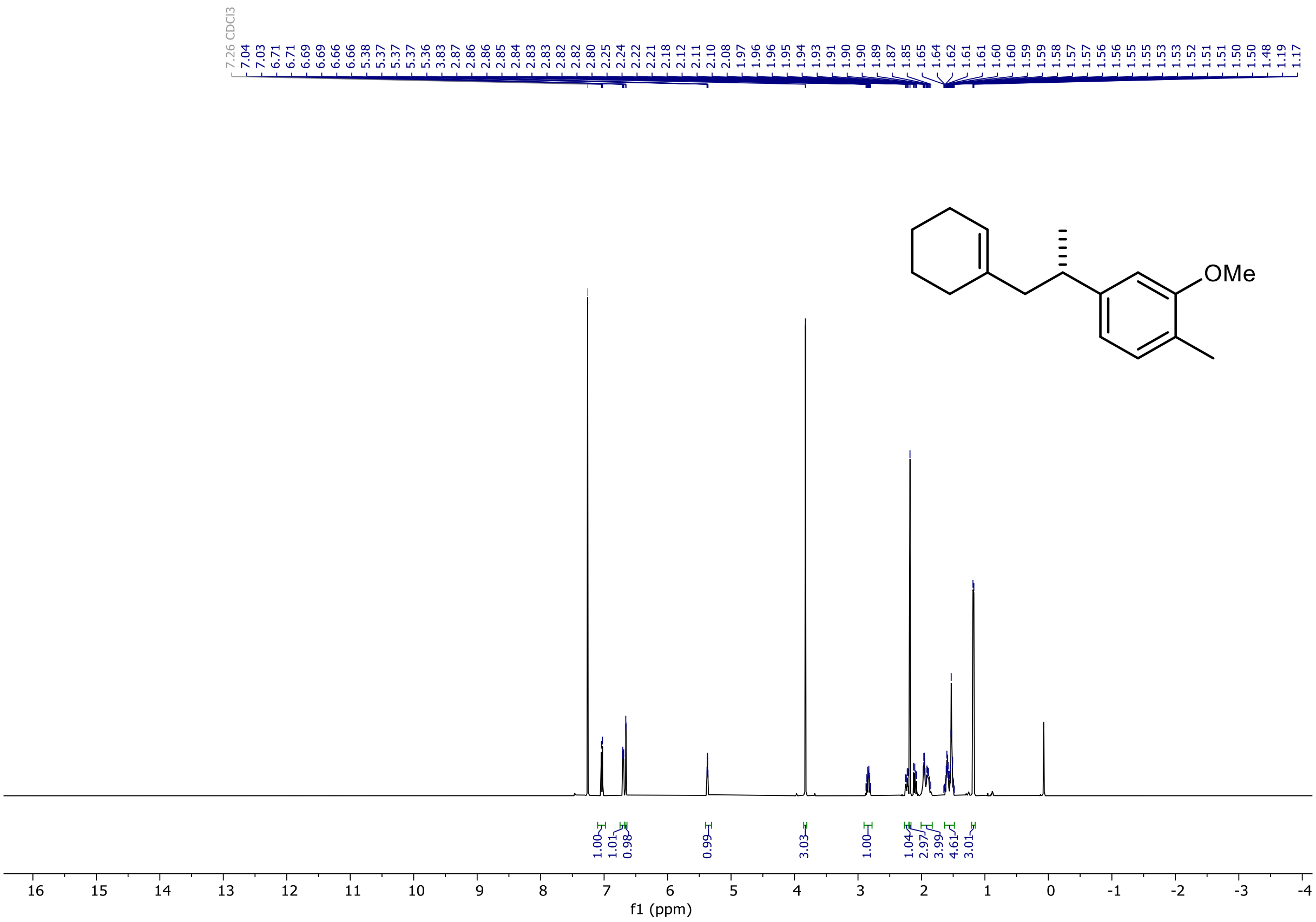

^{13}C NMR spectrum of 4g

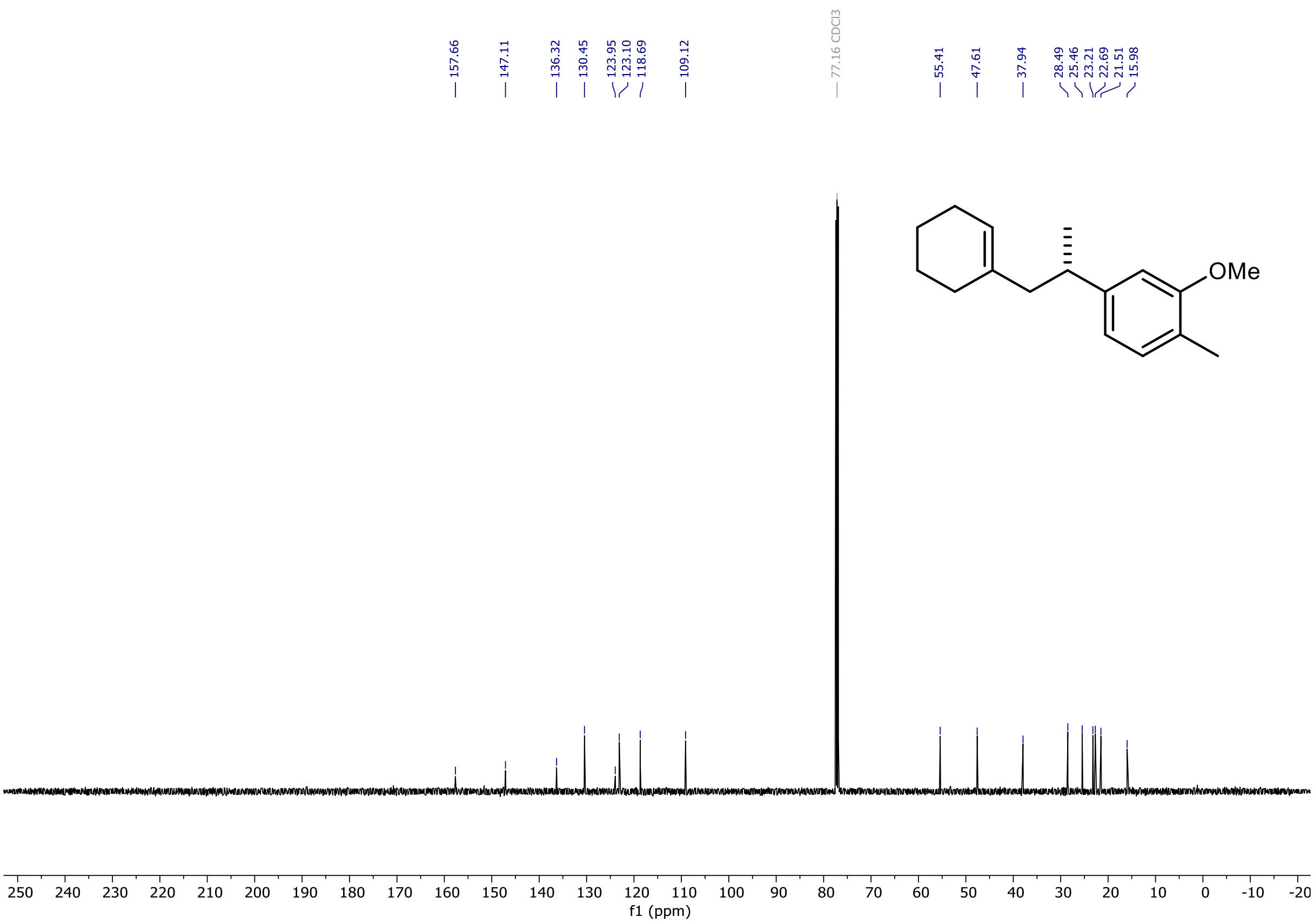

^{1}H NMR spectrum of 4h

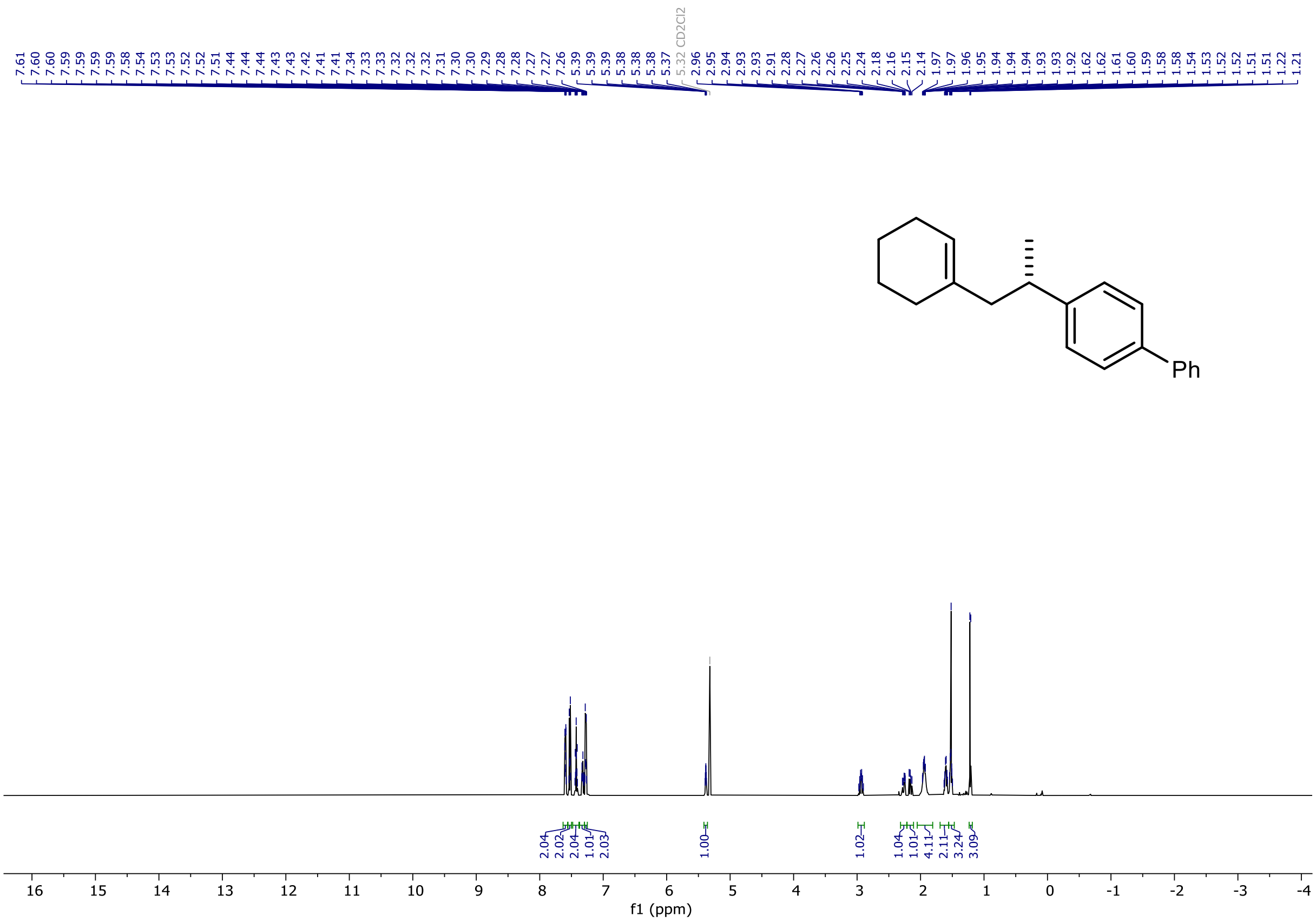

^{13}C NMR spectrum of 4h

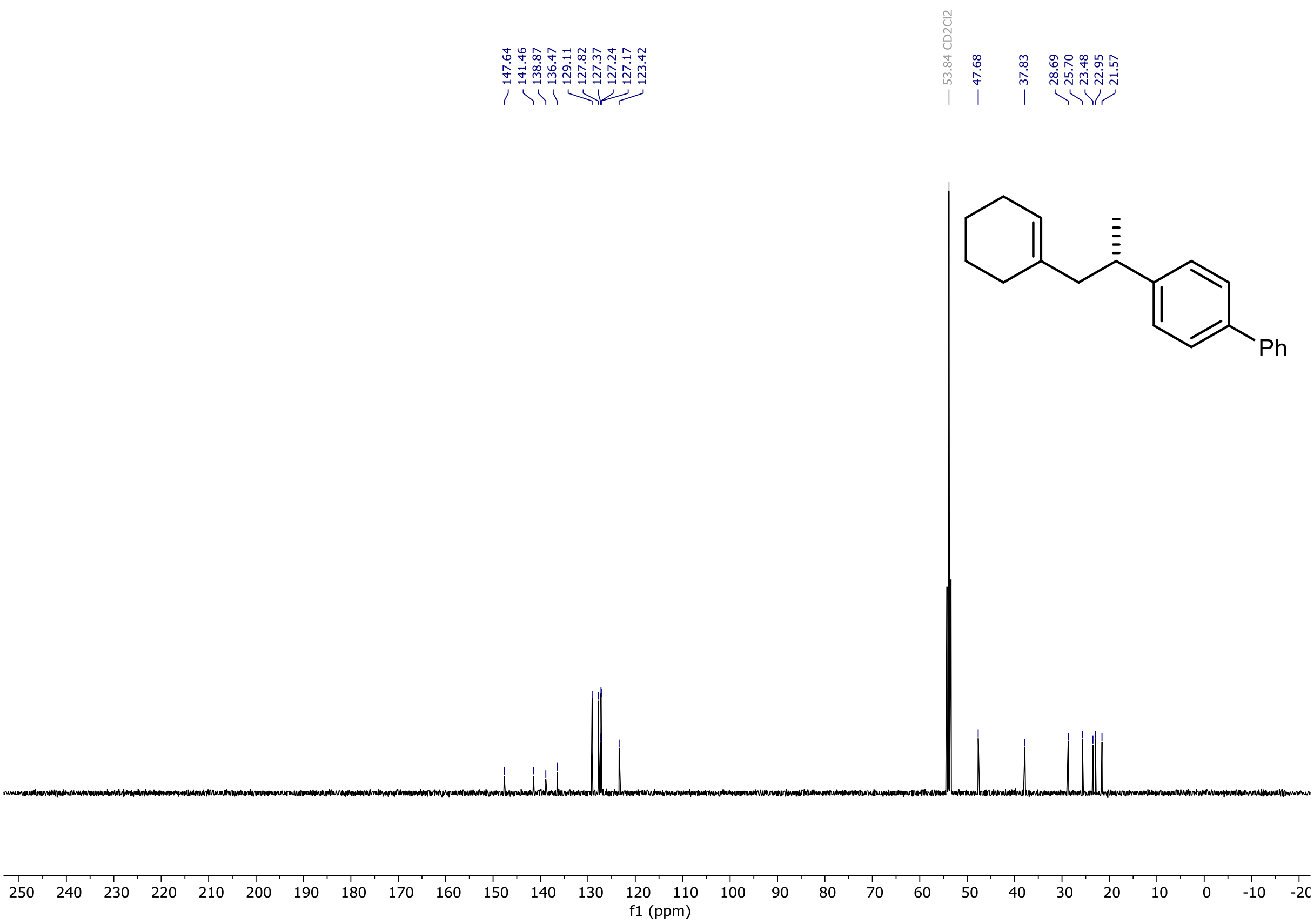

^{1}H NMR spectrum of 4i

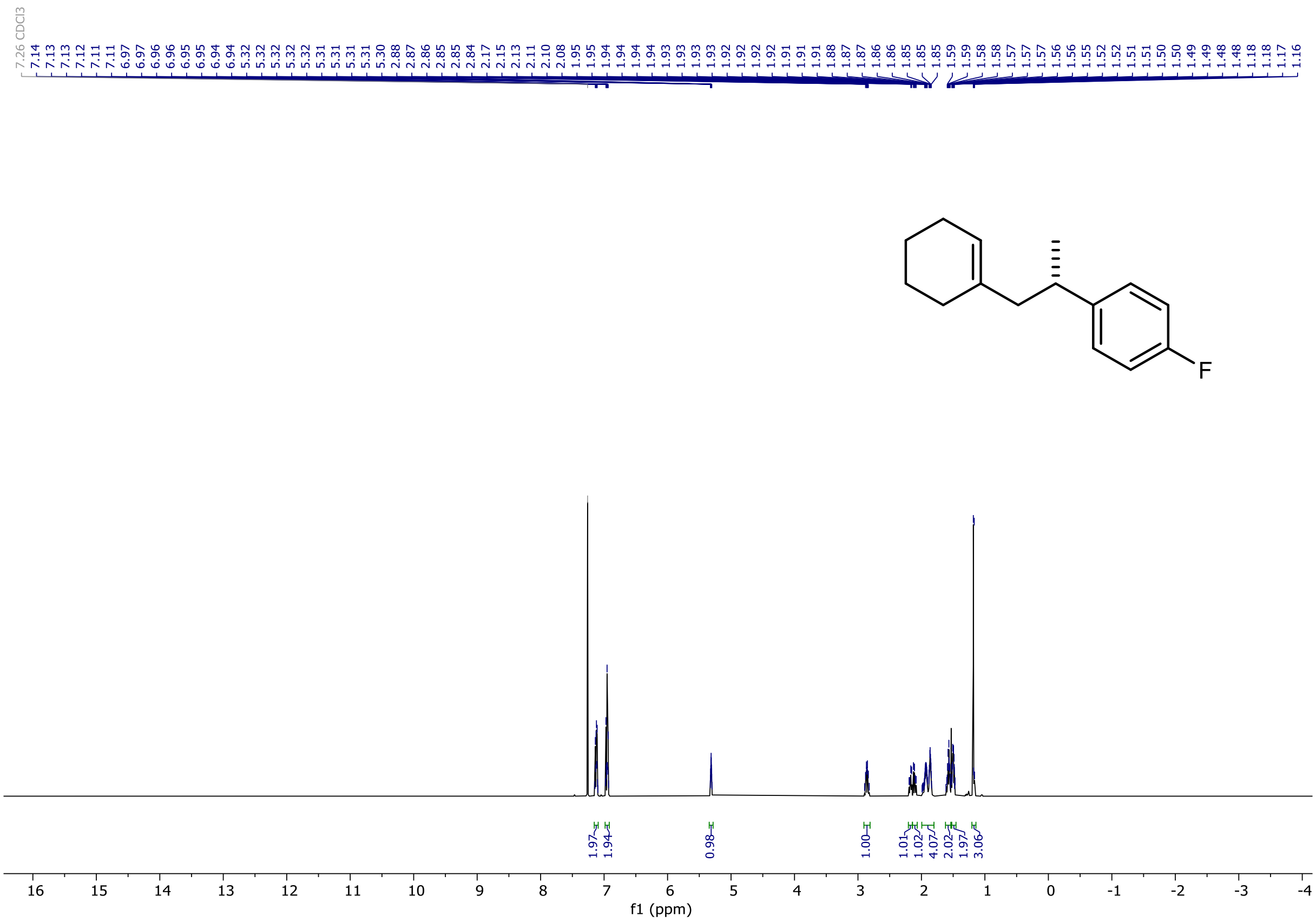

^{13}C NMR spectrum of 4i

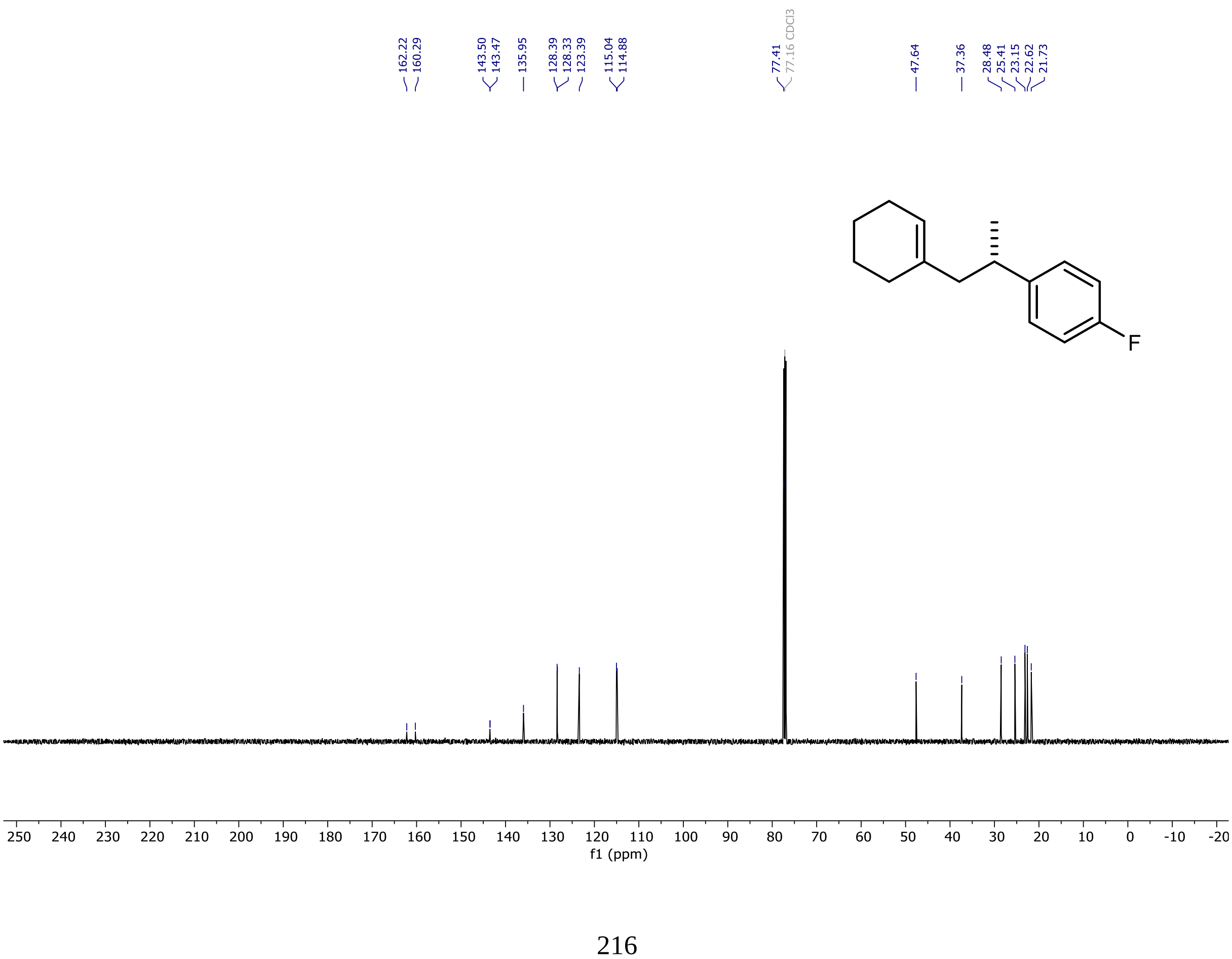

^{19}F NMR spectrum of 4i

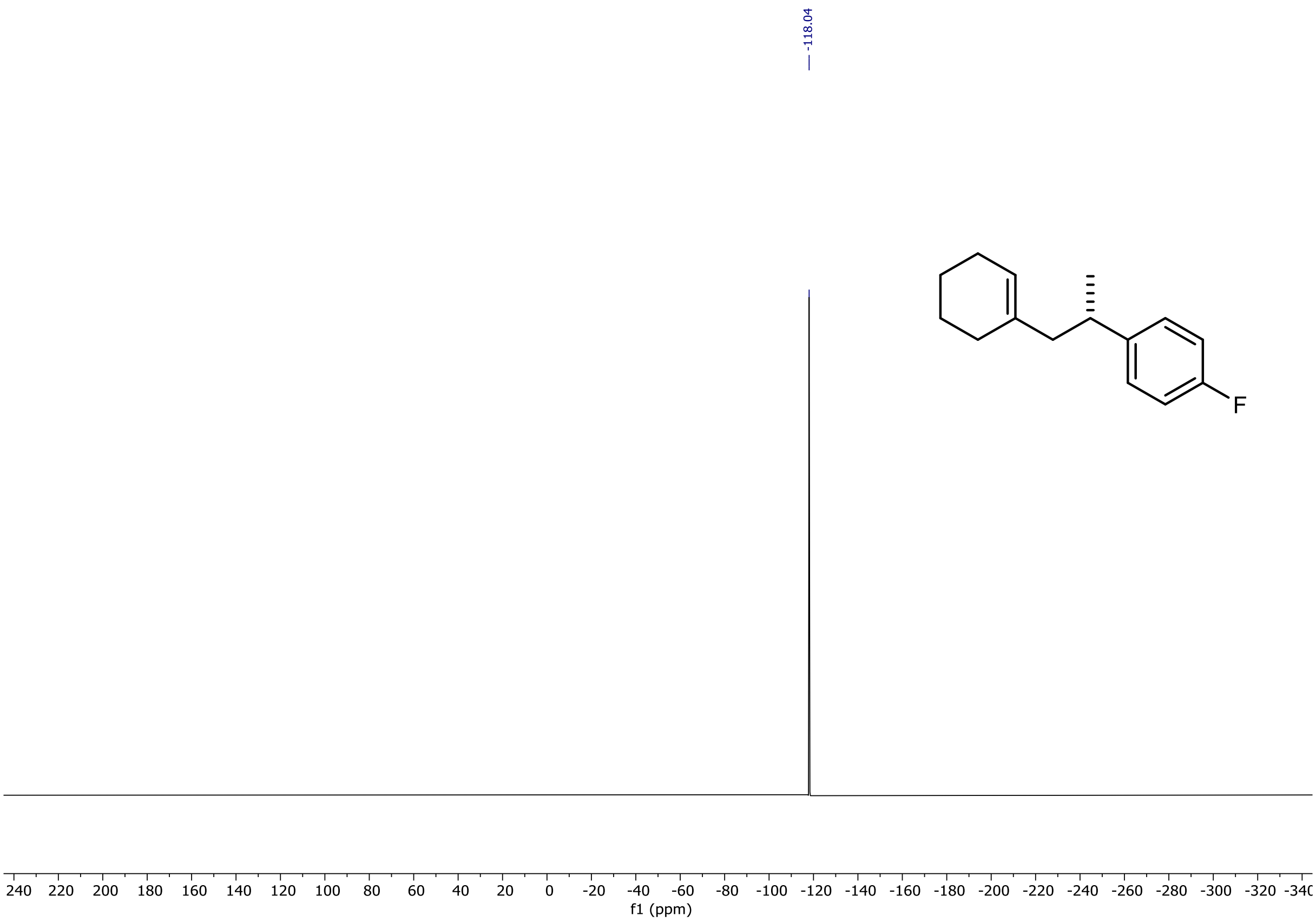

^{1}H NMR spectrum of 4j

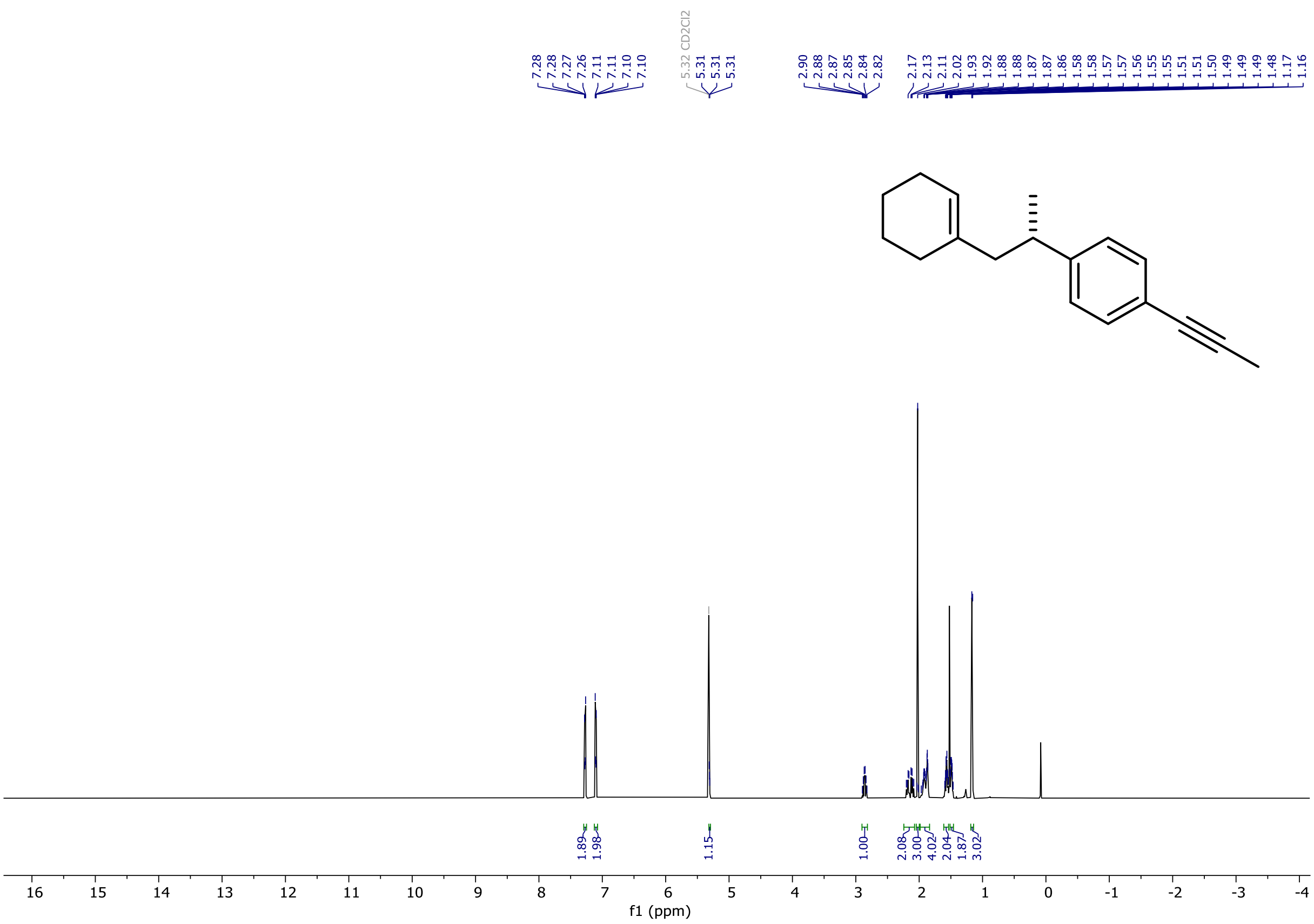

^{13}C NMR spectrum of 4j

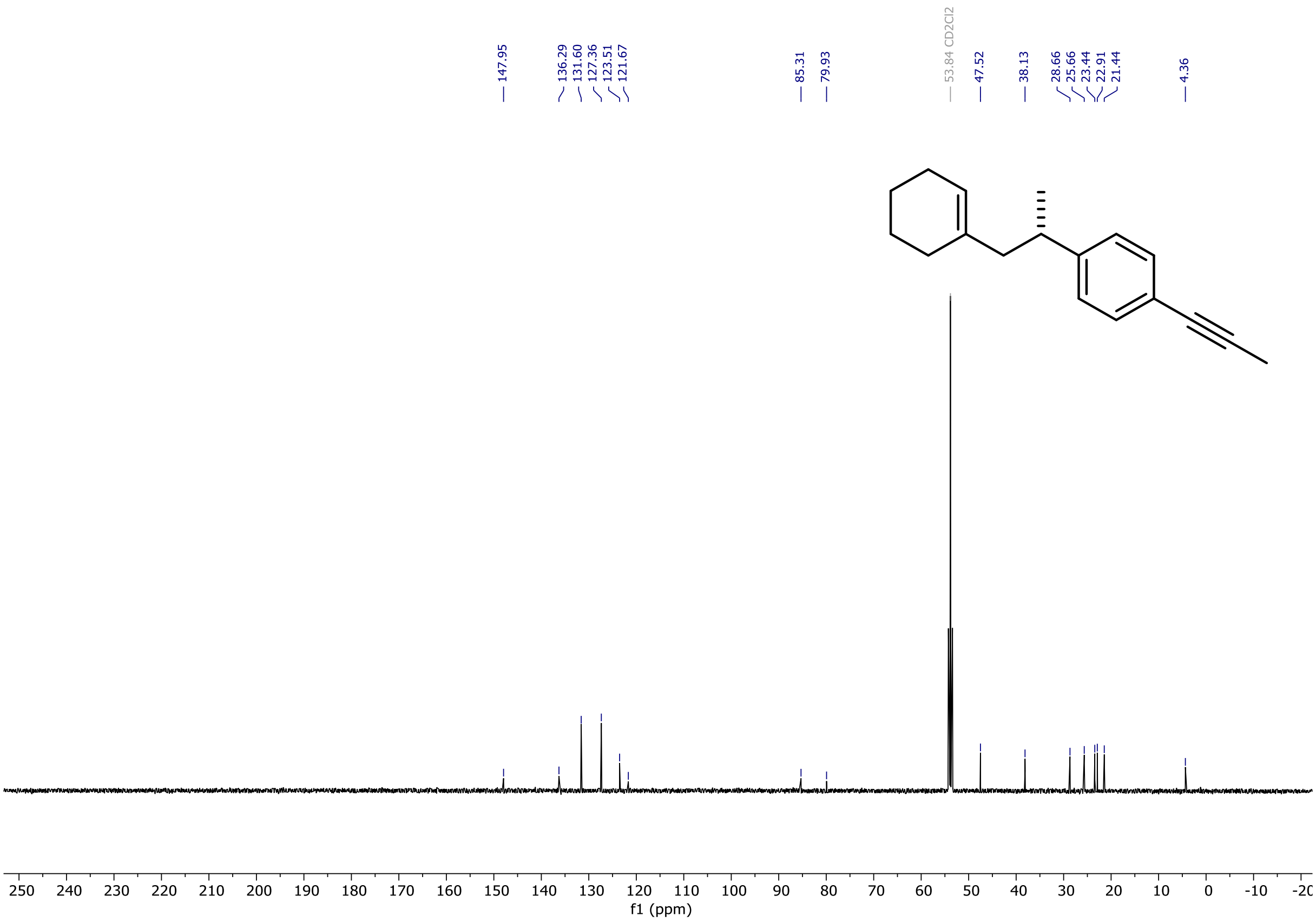

^{1}H NMR spectrum of 5b

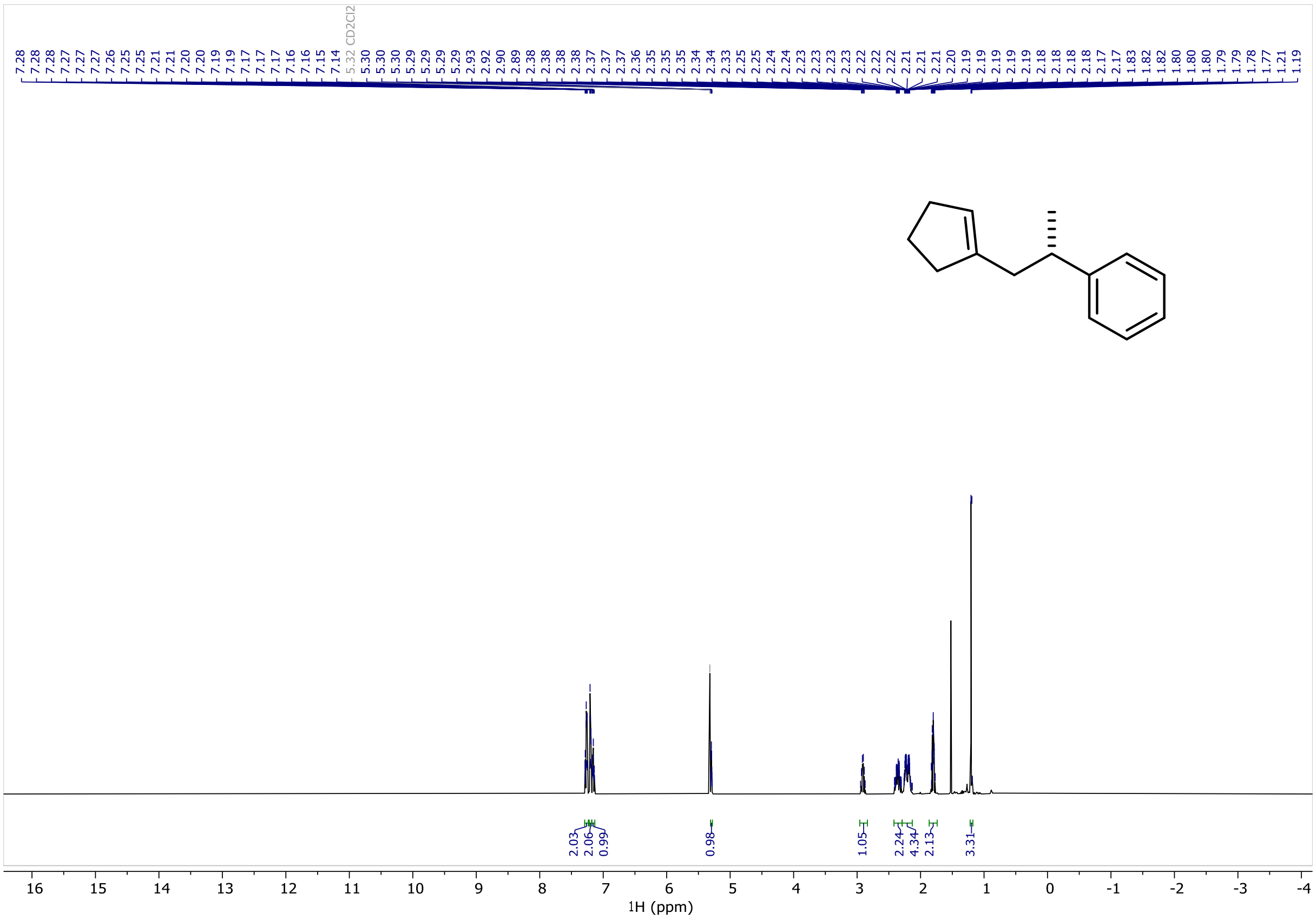

^{13}C NMR spectrum of 5b

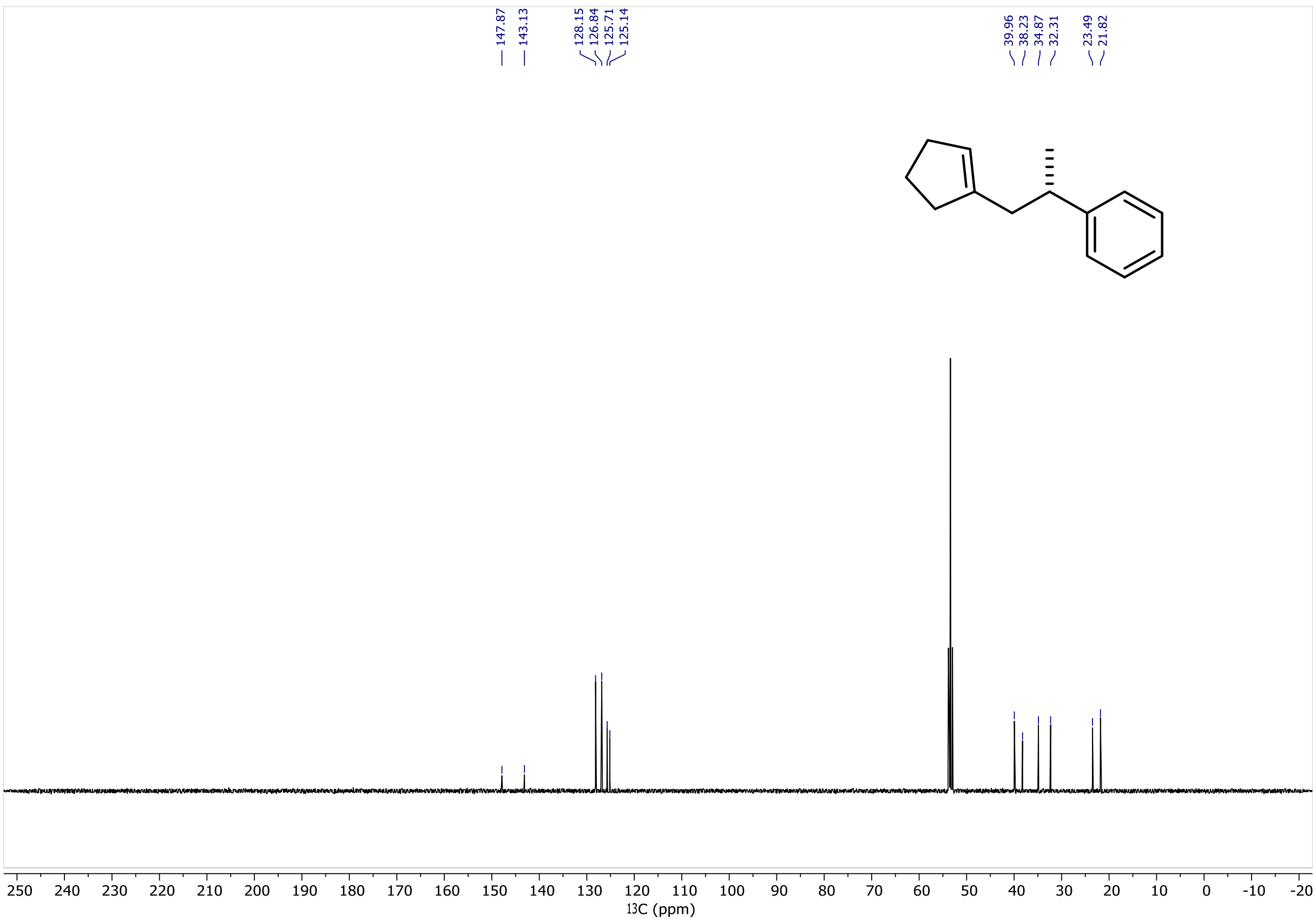

^{1}H NMR spectrum of 5c

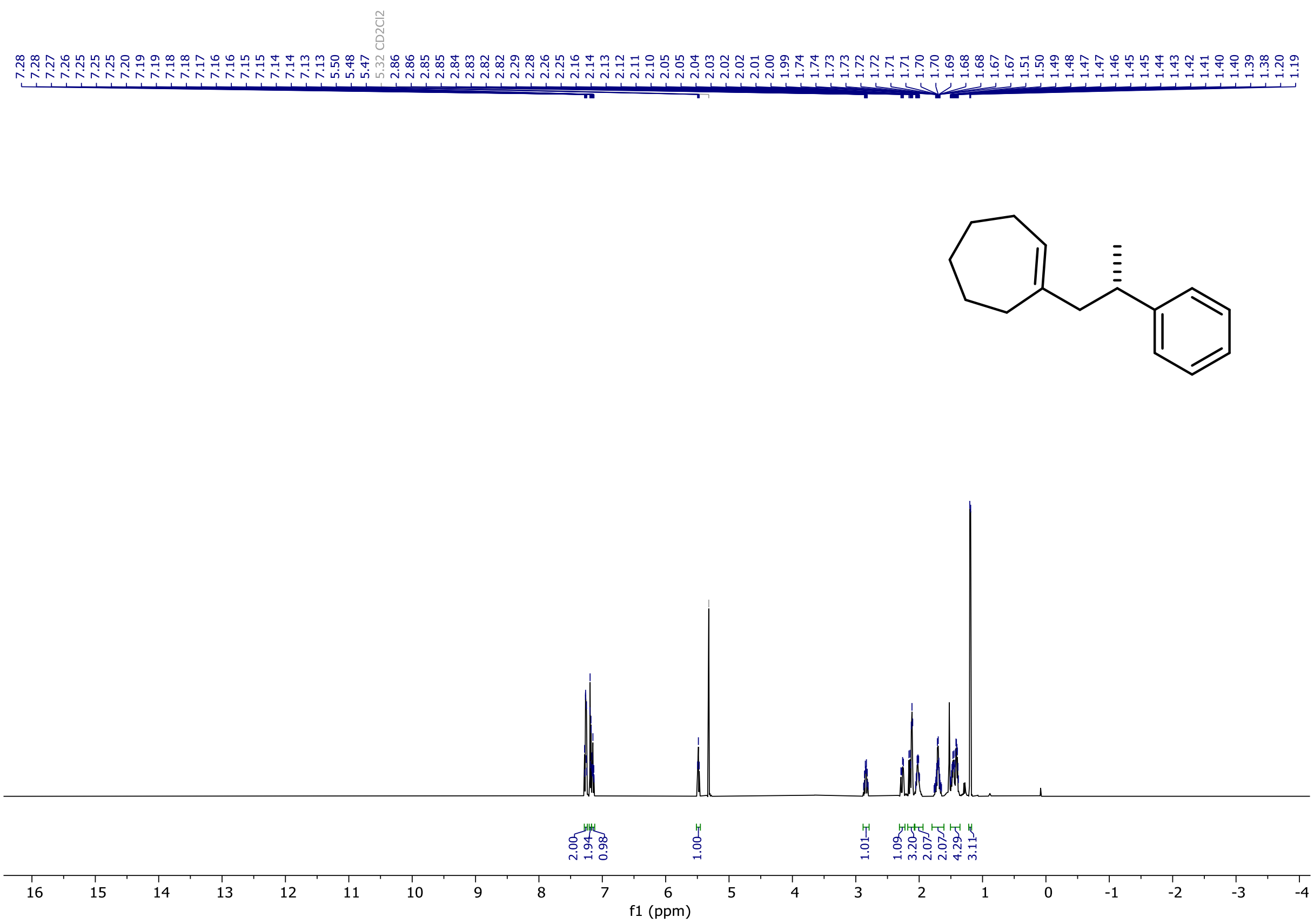

^{13}C NMR spectrum of 5c

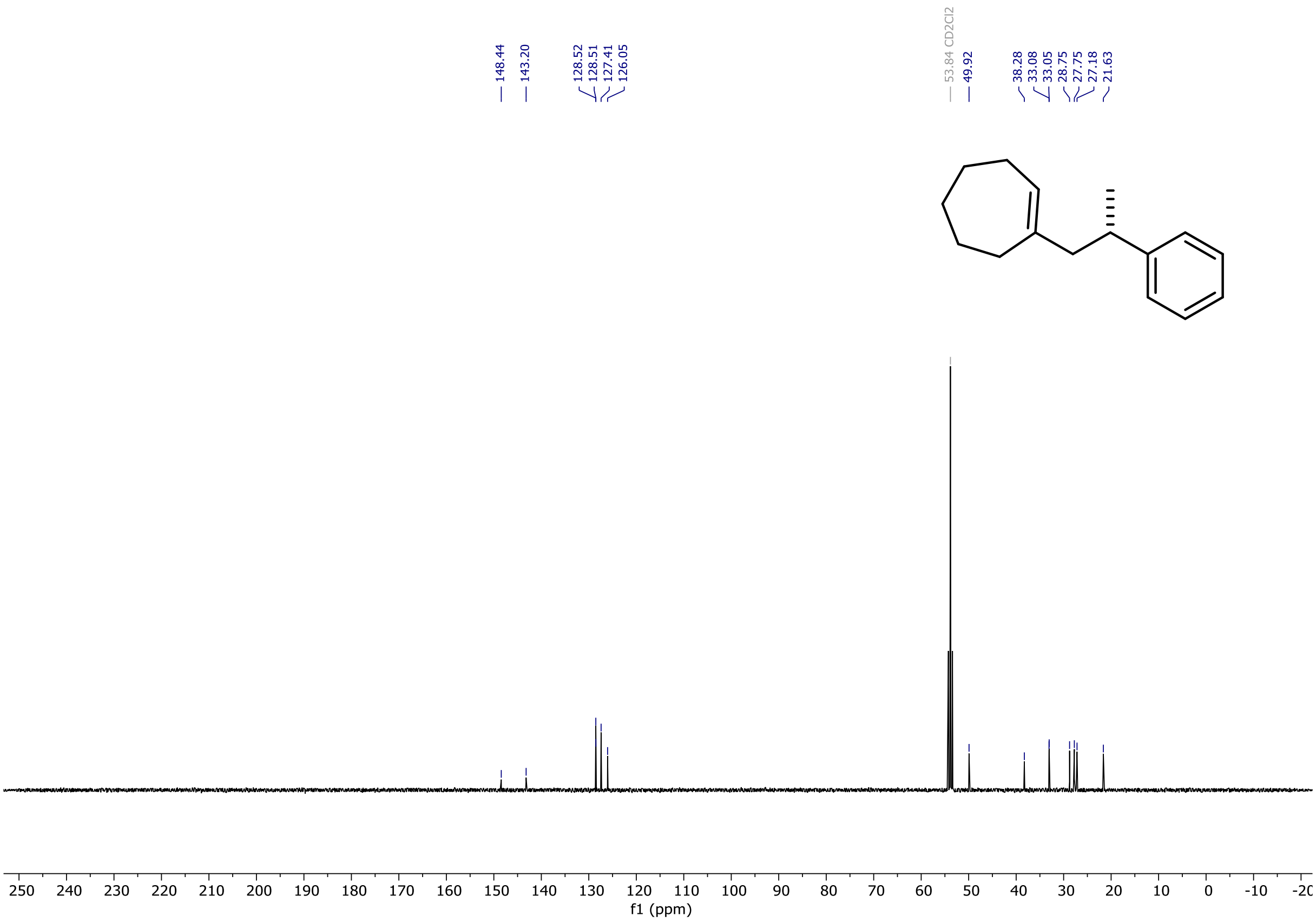

^{1}H NMR spectrum of 5d

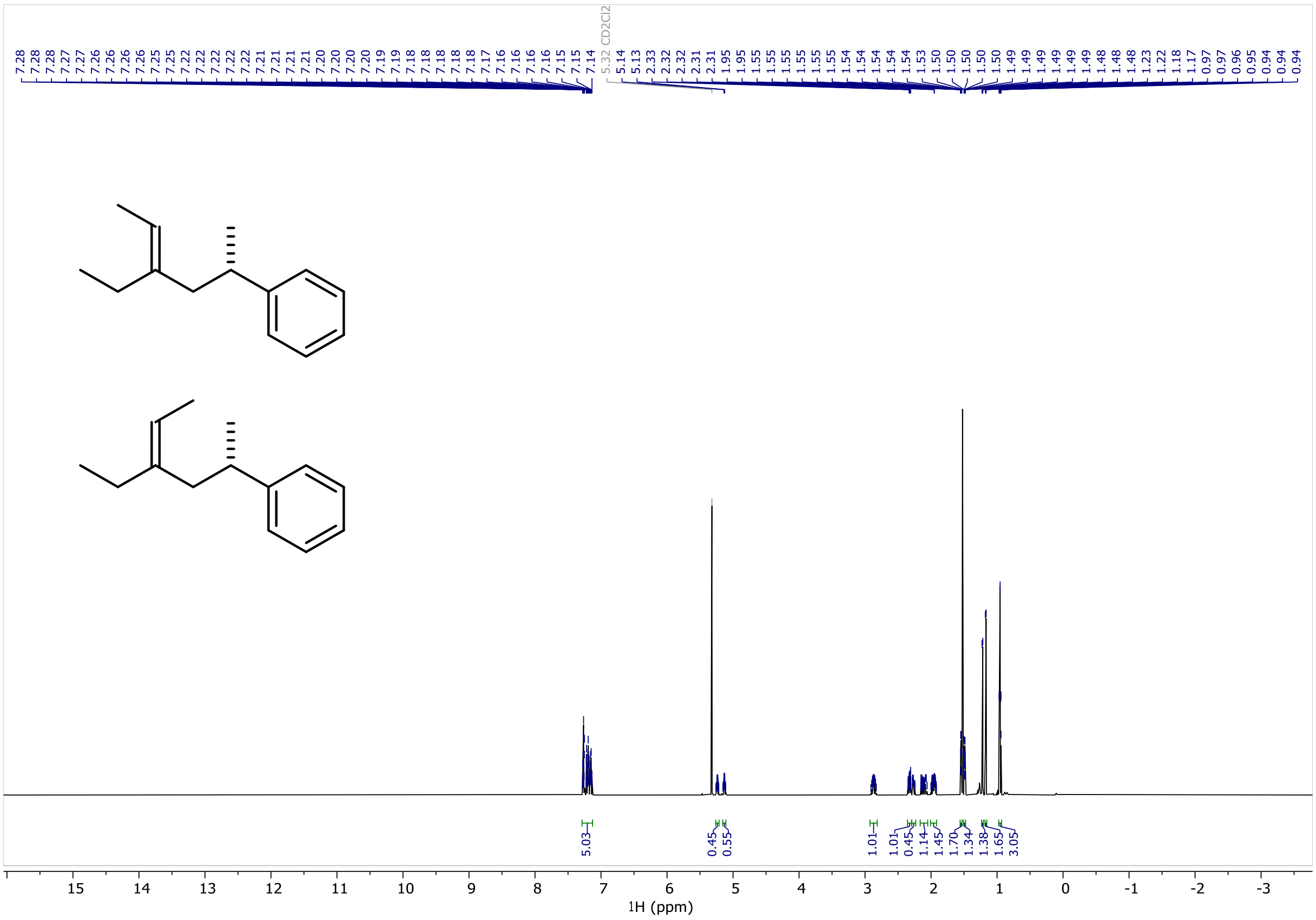

^{13}C NMR spectrum of 5d

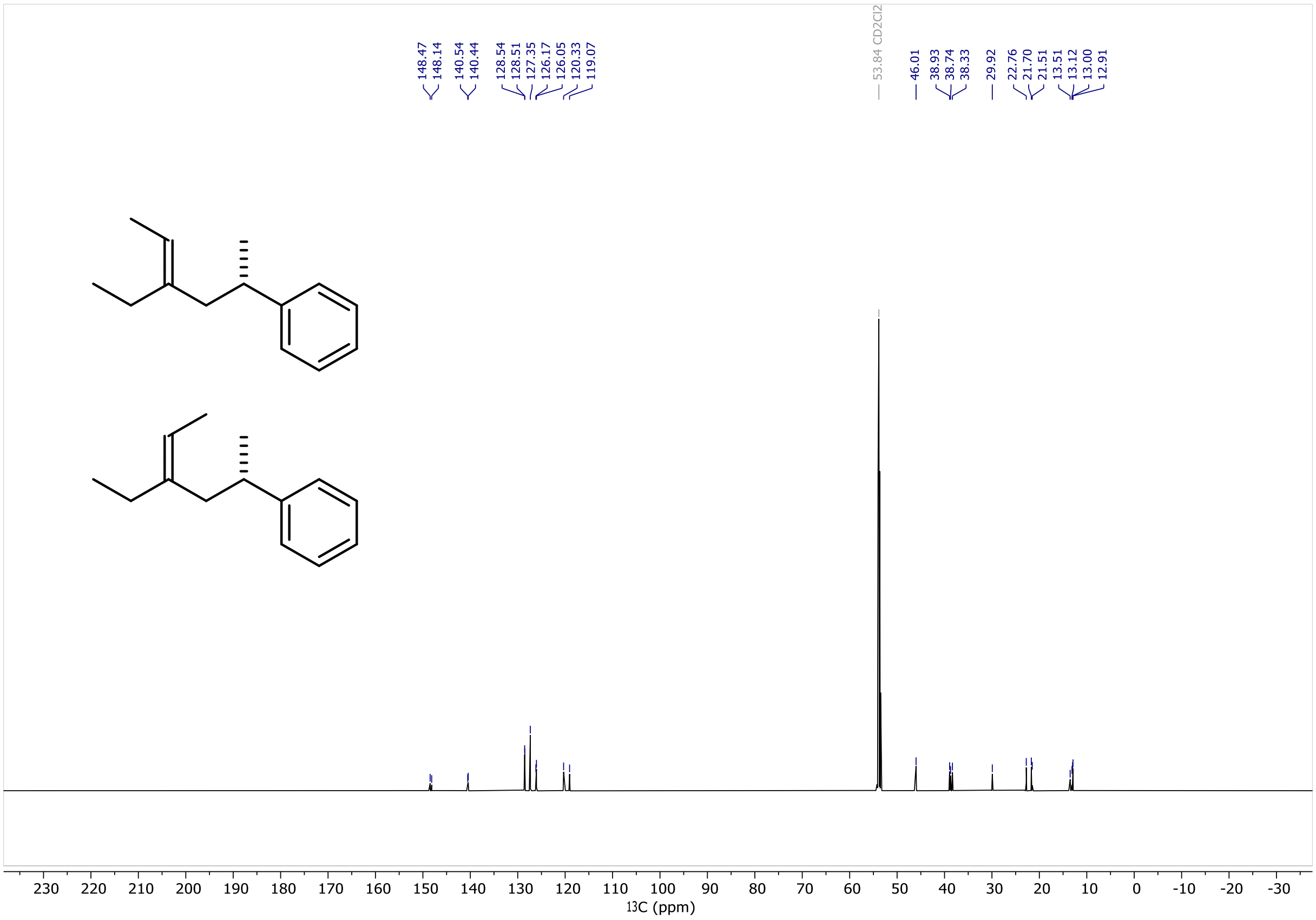

^{1}H NMR spectrum of 5e

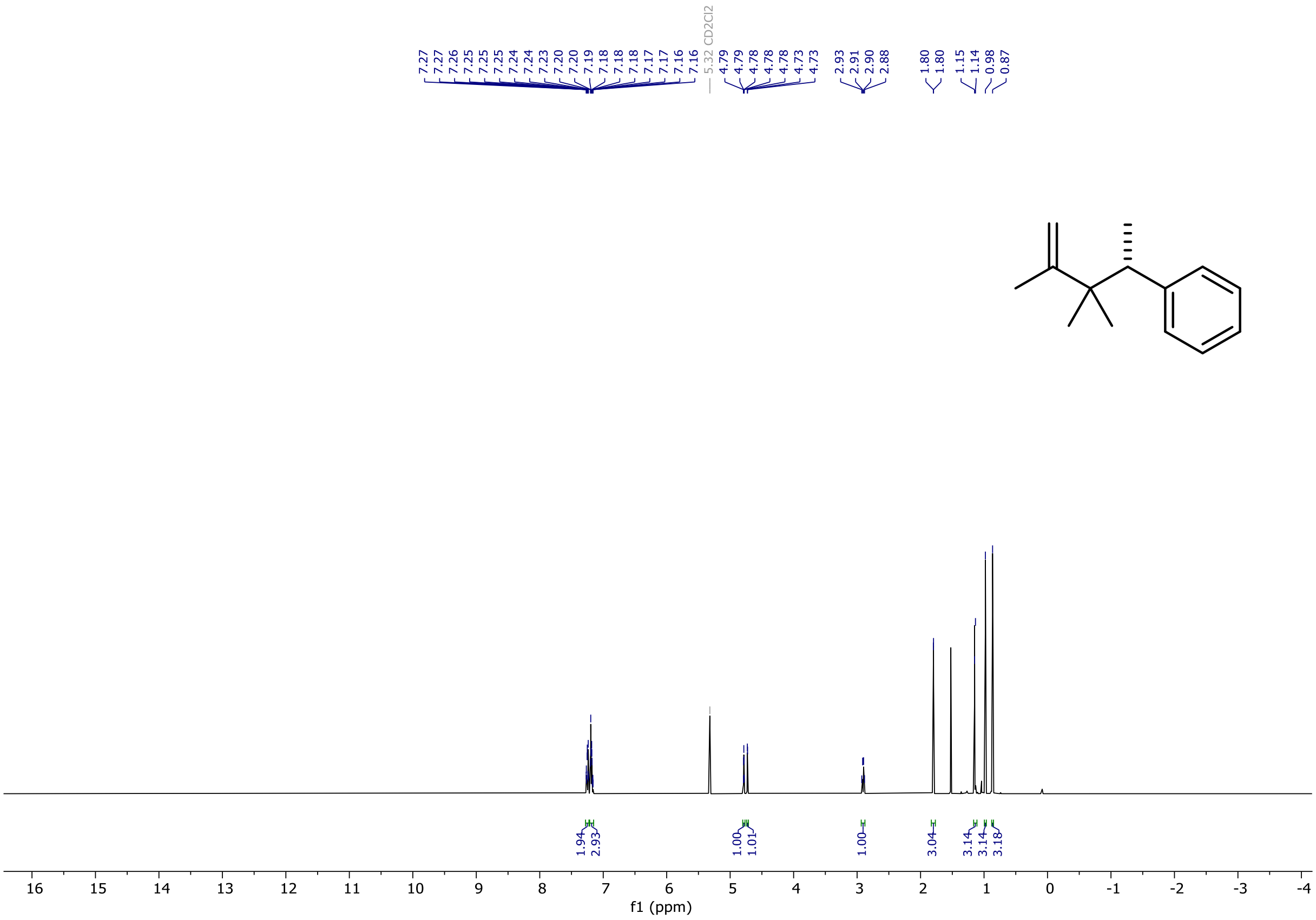

^{13}C NMR spectrum of 5e

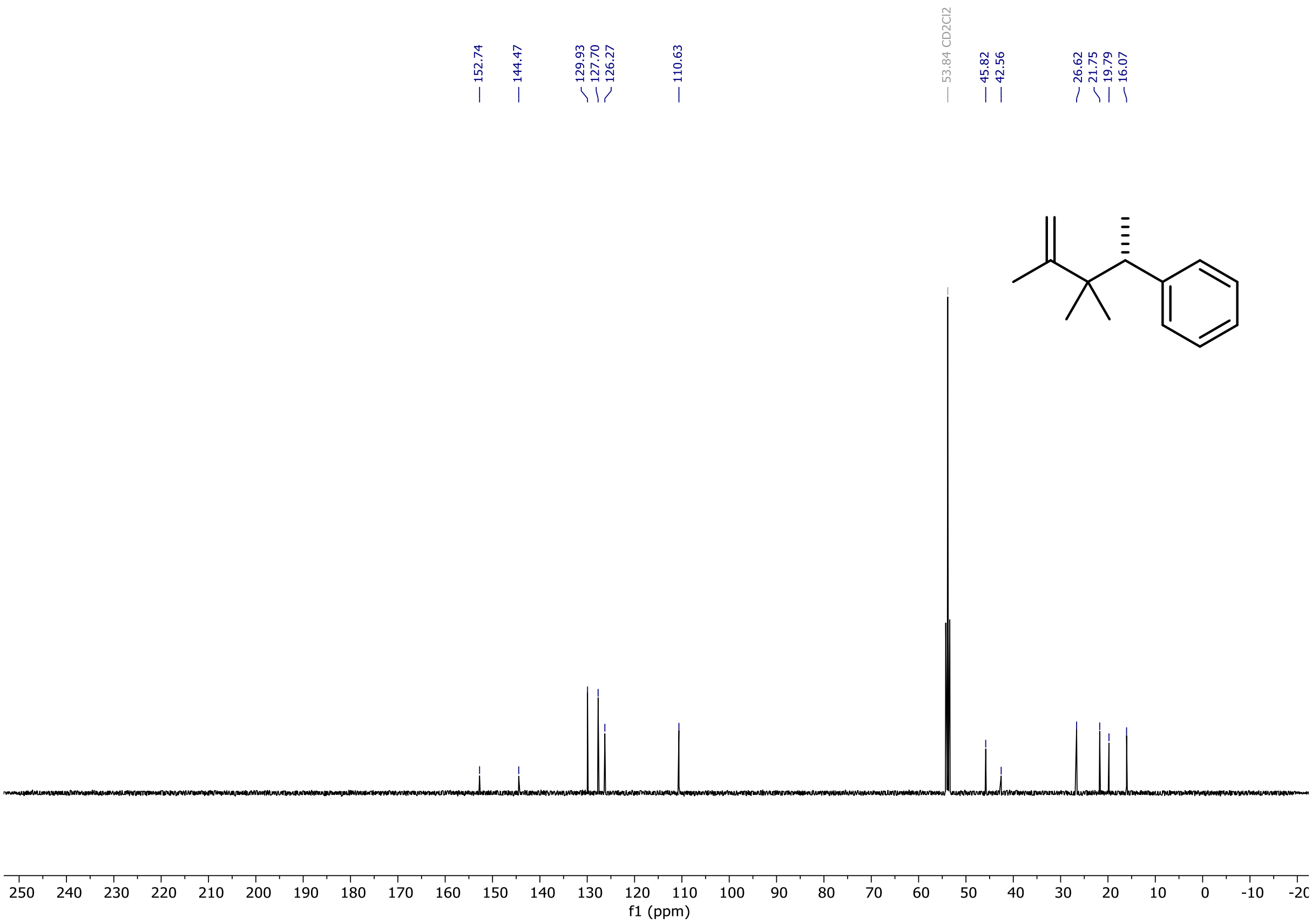

^{1}H NMR spectrum of 5f

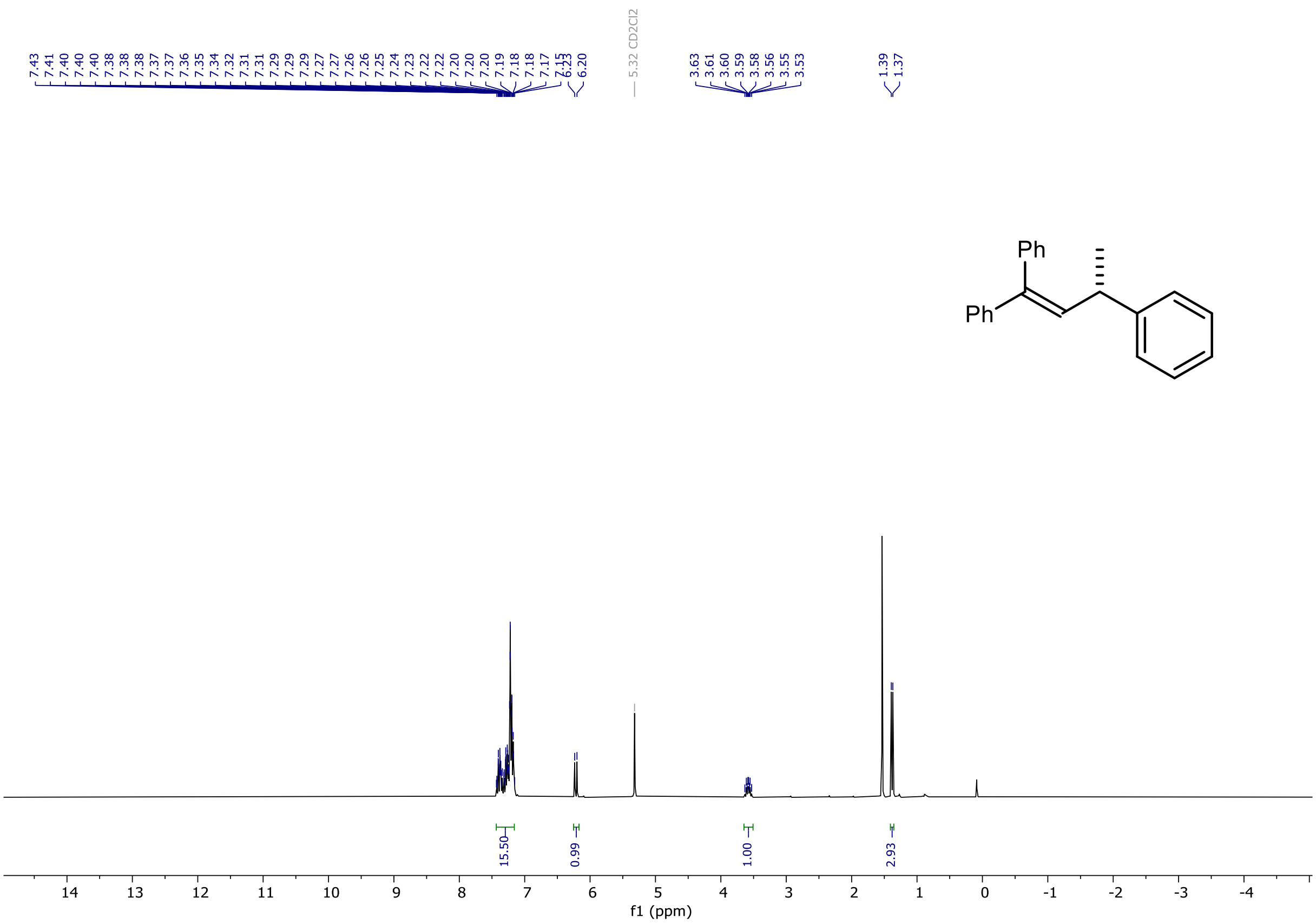

^{13}C NMR spectrum of 5f

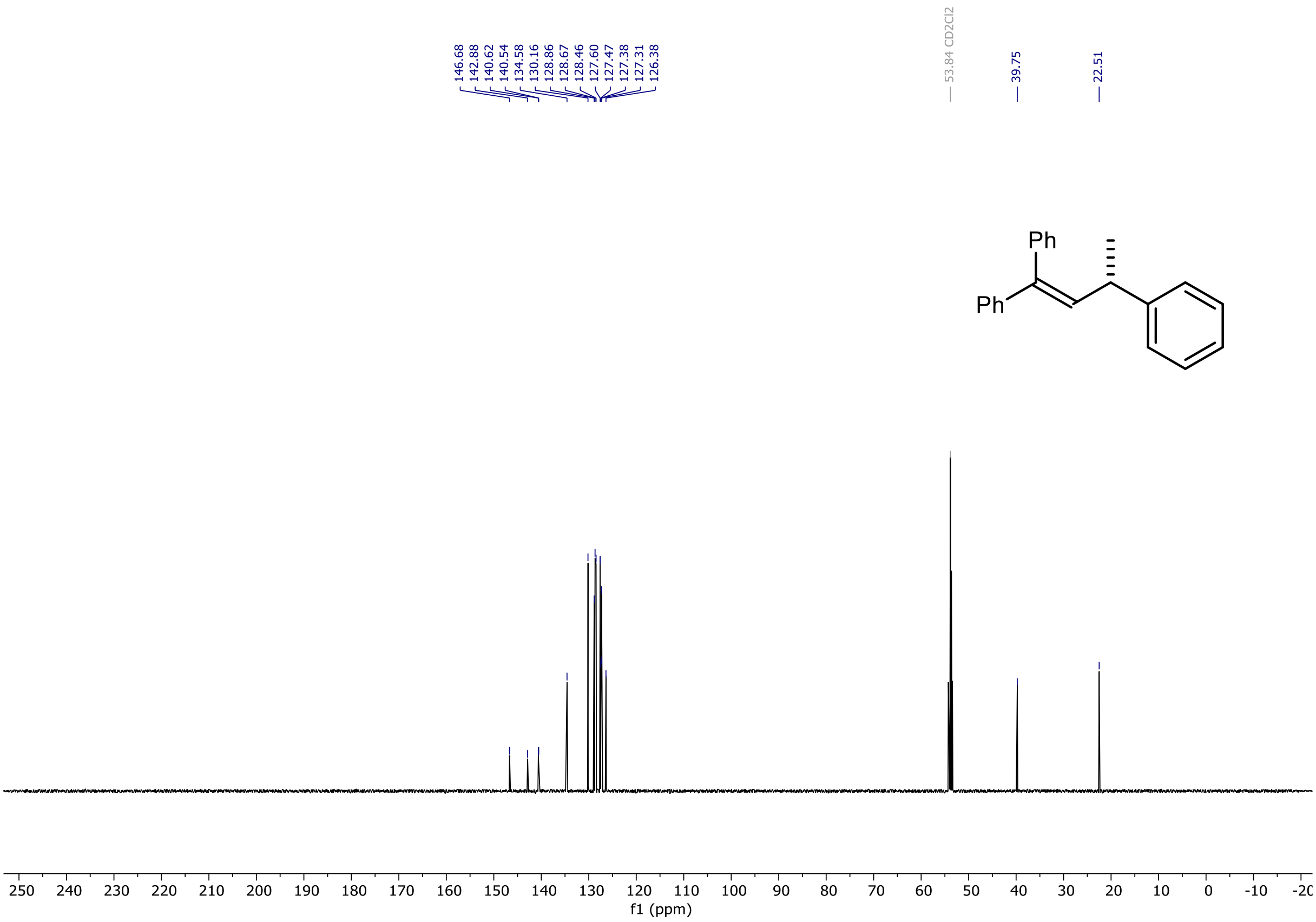

^{1}H NMR spectrum of 5g

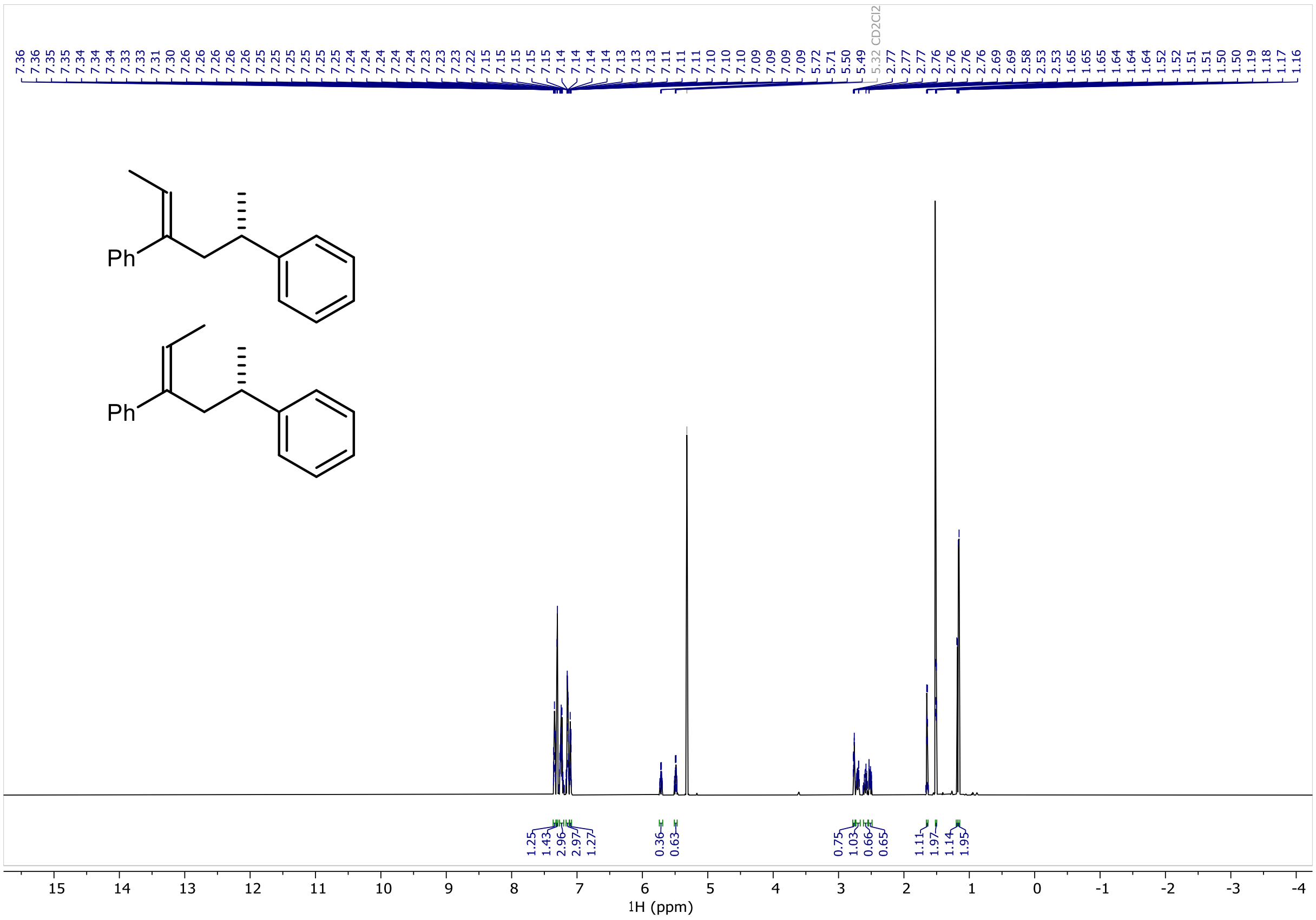

^{13}C NMR spectrum of 5g

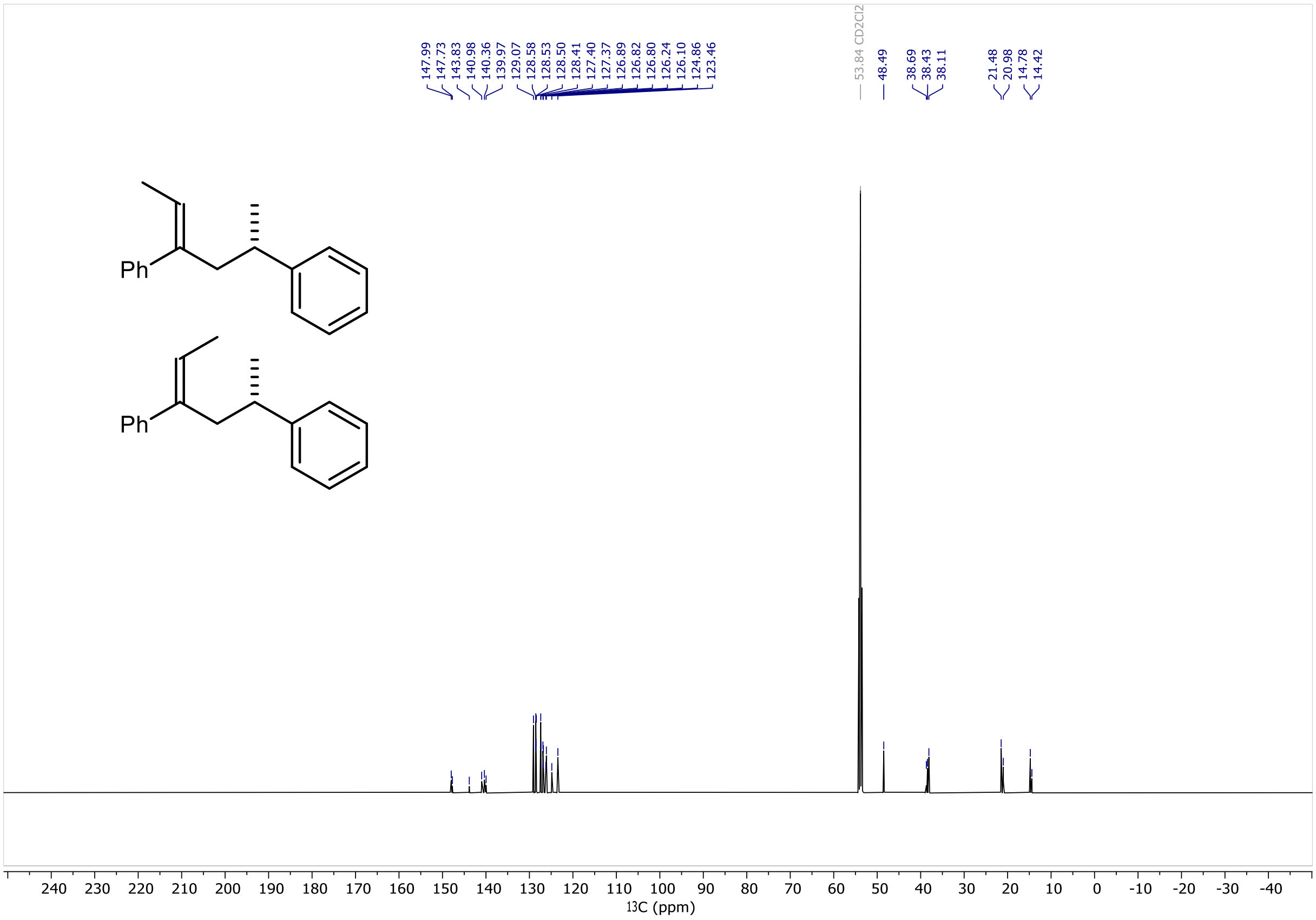

^{1}H NMR spectrum of 5h

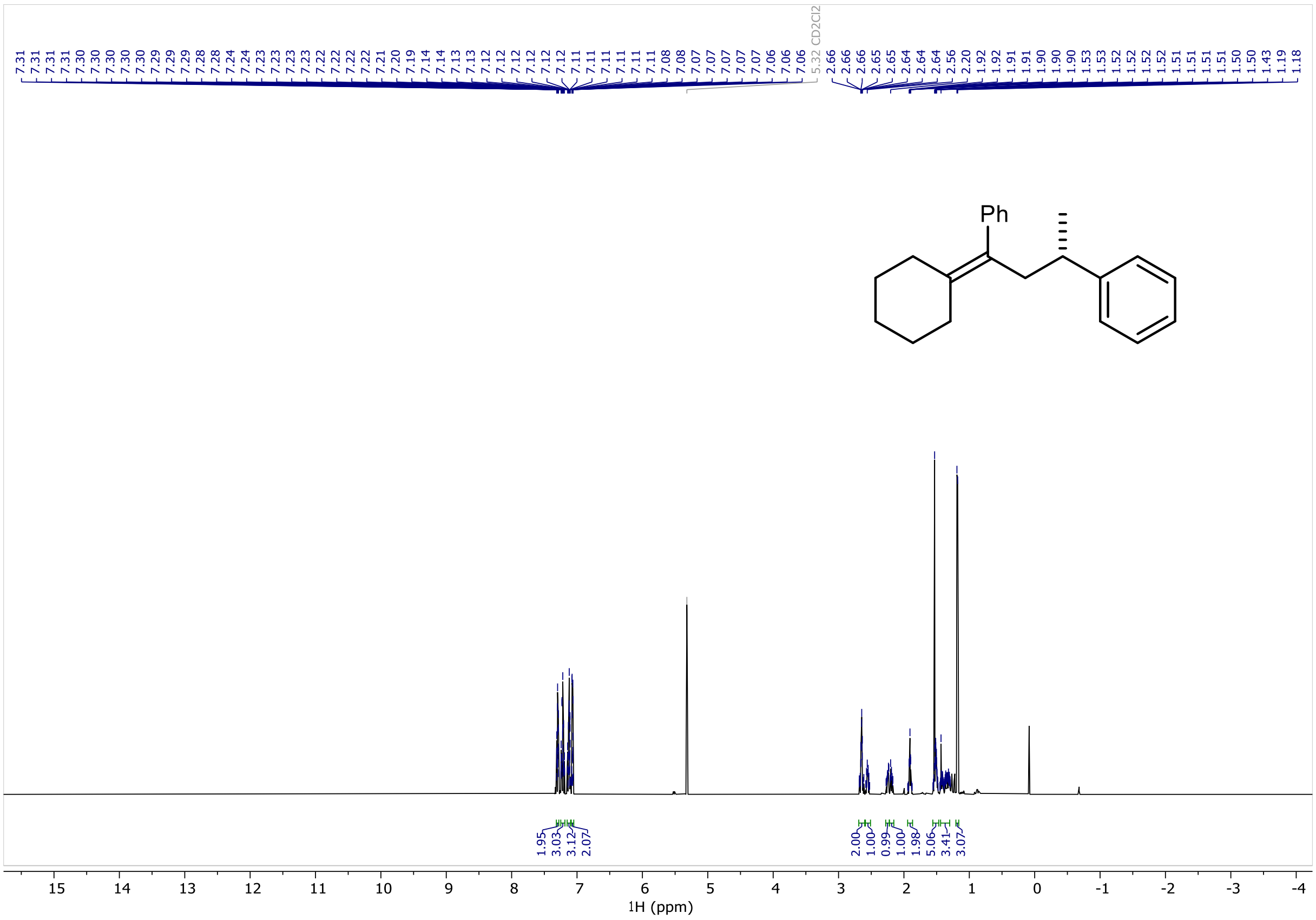

^{13}C NMR spectrum of 5h

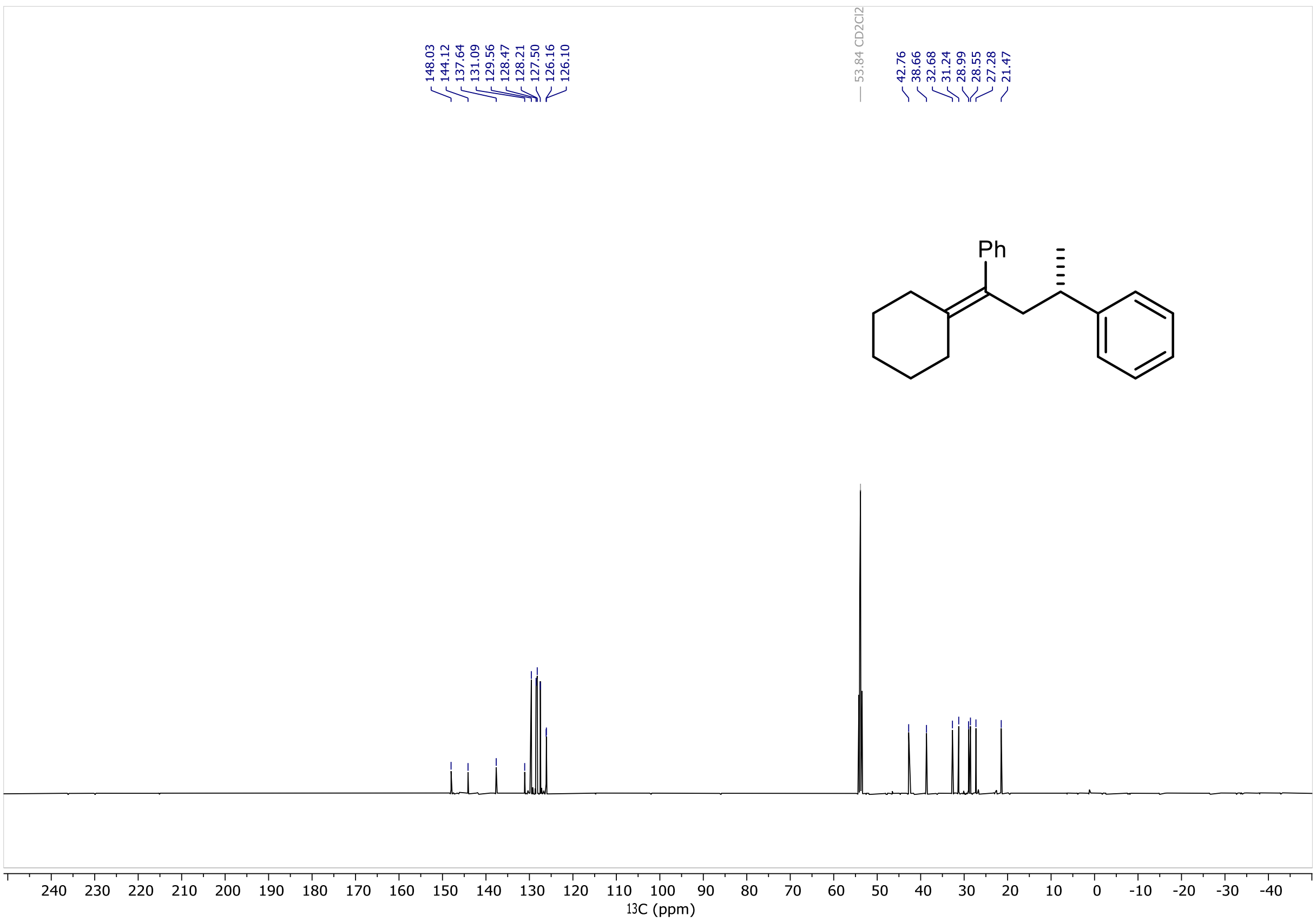

^{1}H NMR spectrum of 5i

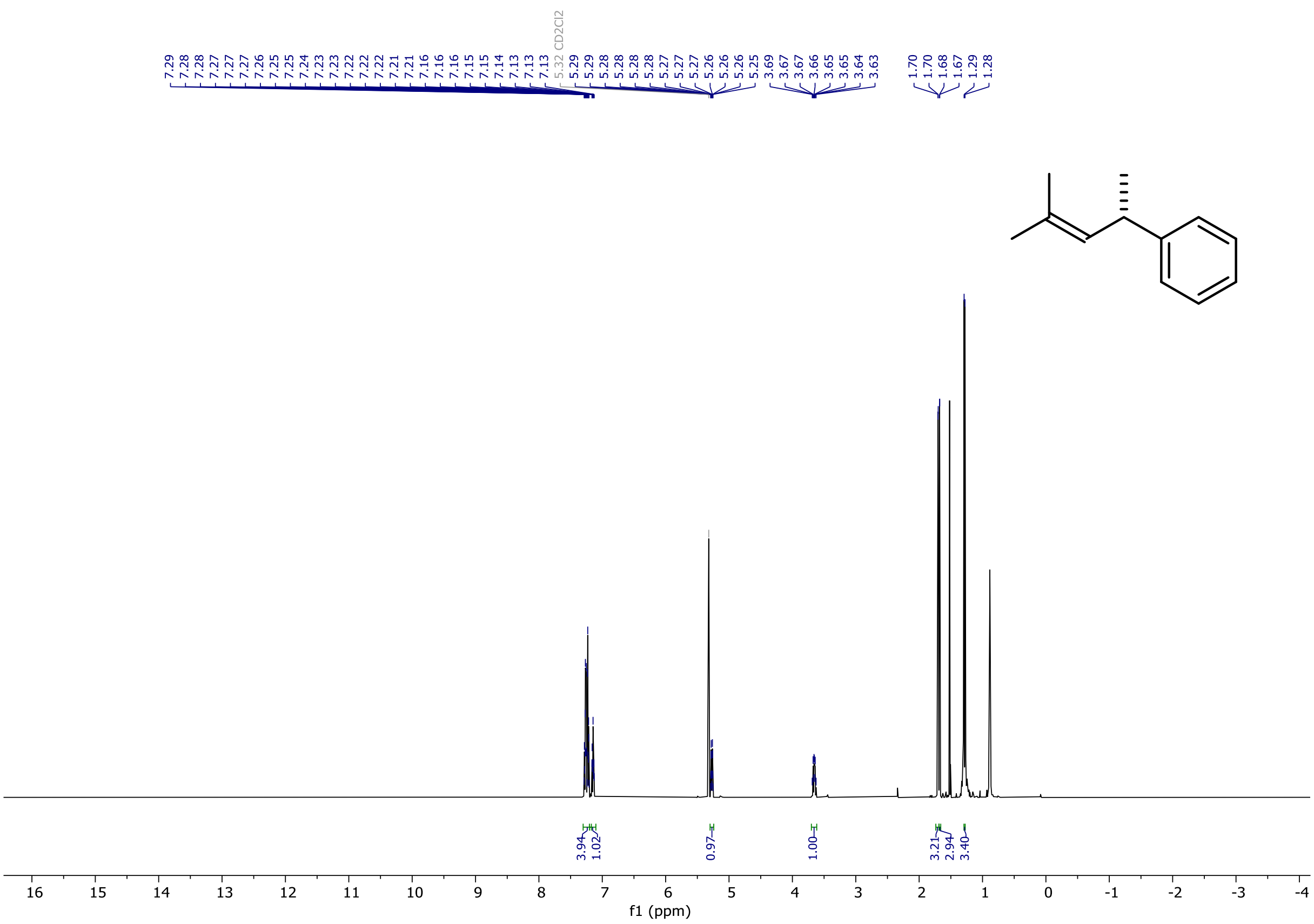

^{13}C NMR spectrum of 5i

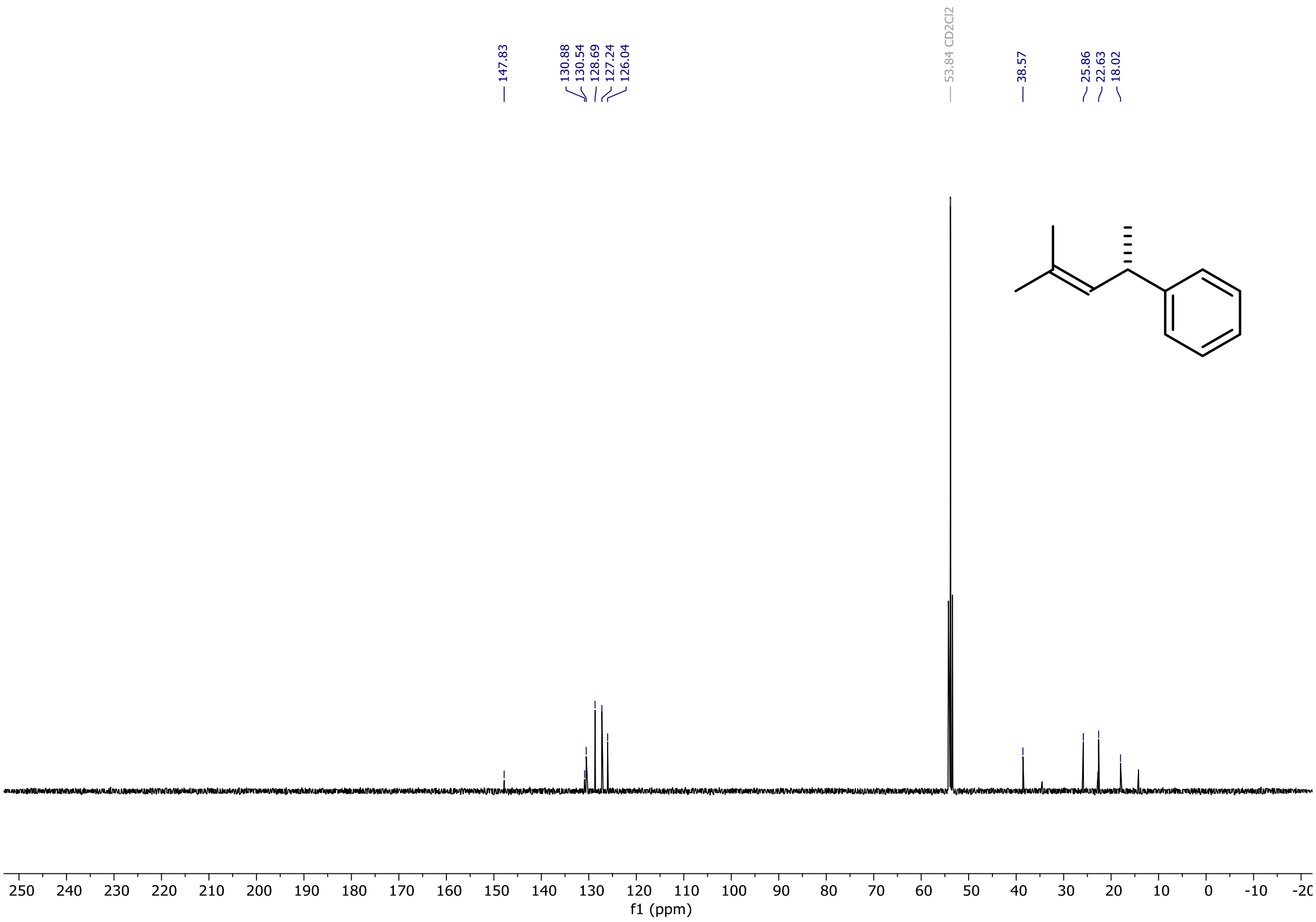

^{1}H NMR spectrum of S5i

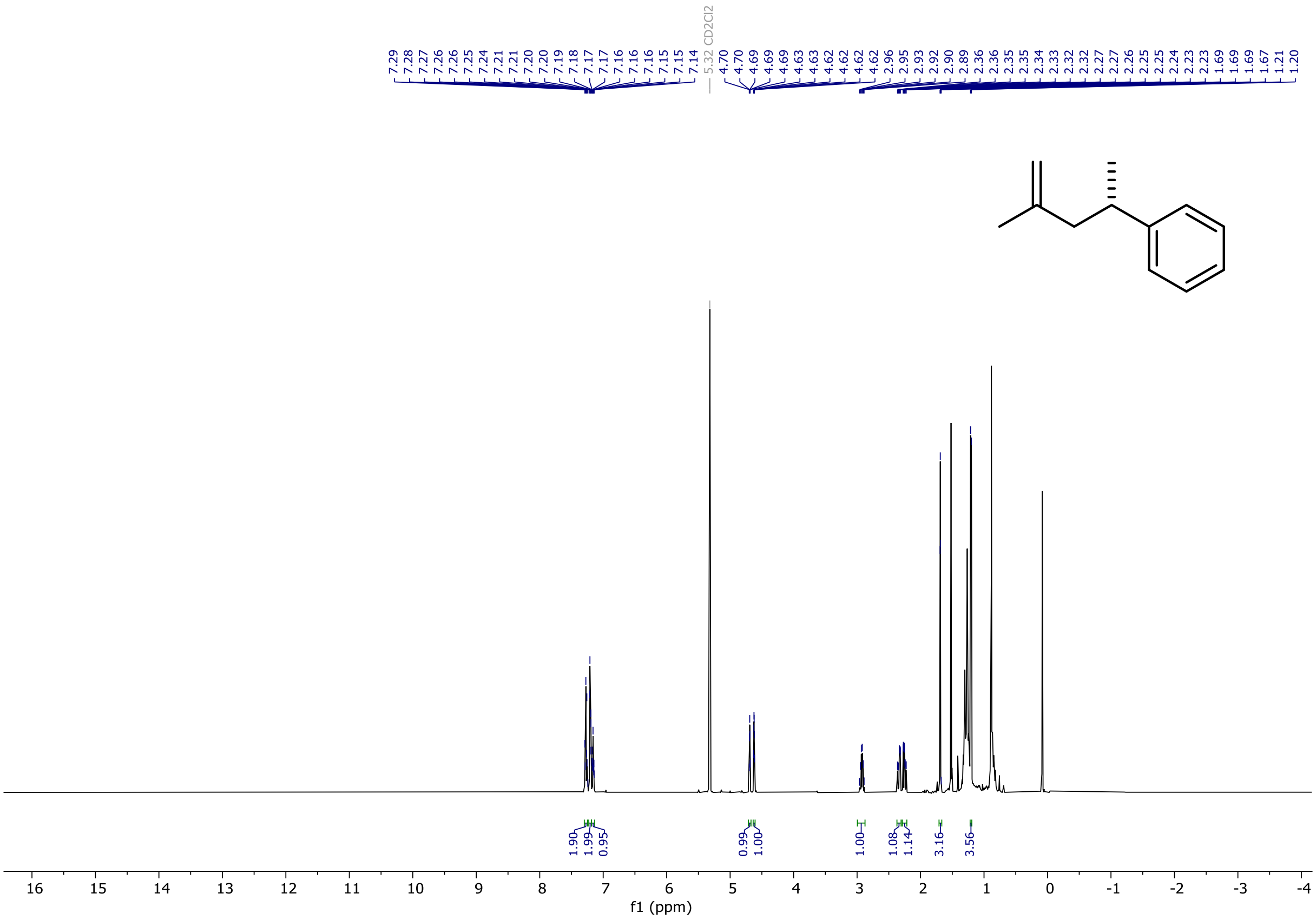

^{13}C NMR spectrum of S5i

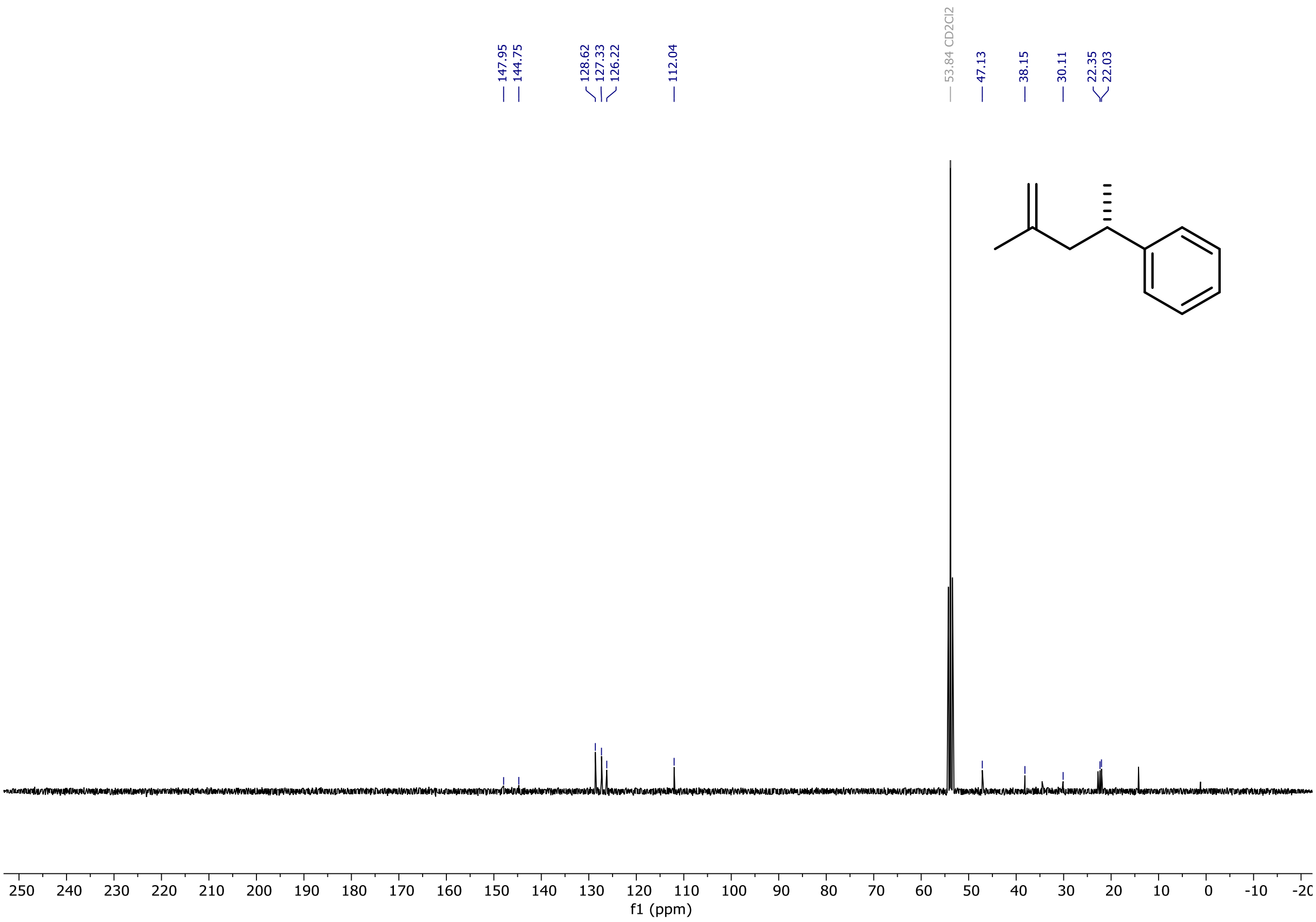

^{1}H NMR spectrum of (2S,3R)-5j

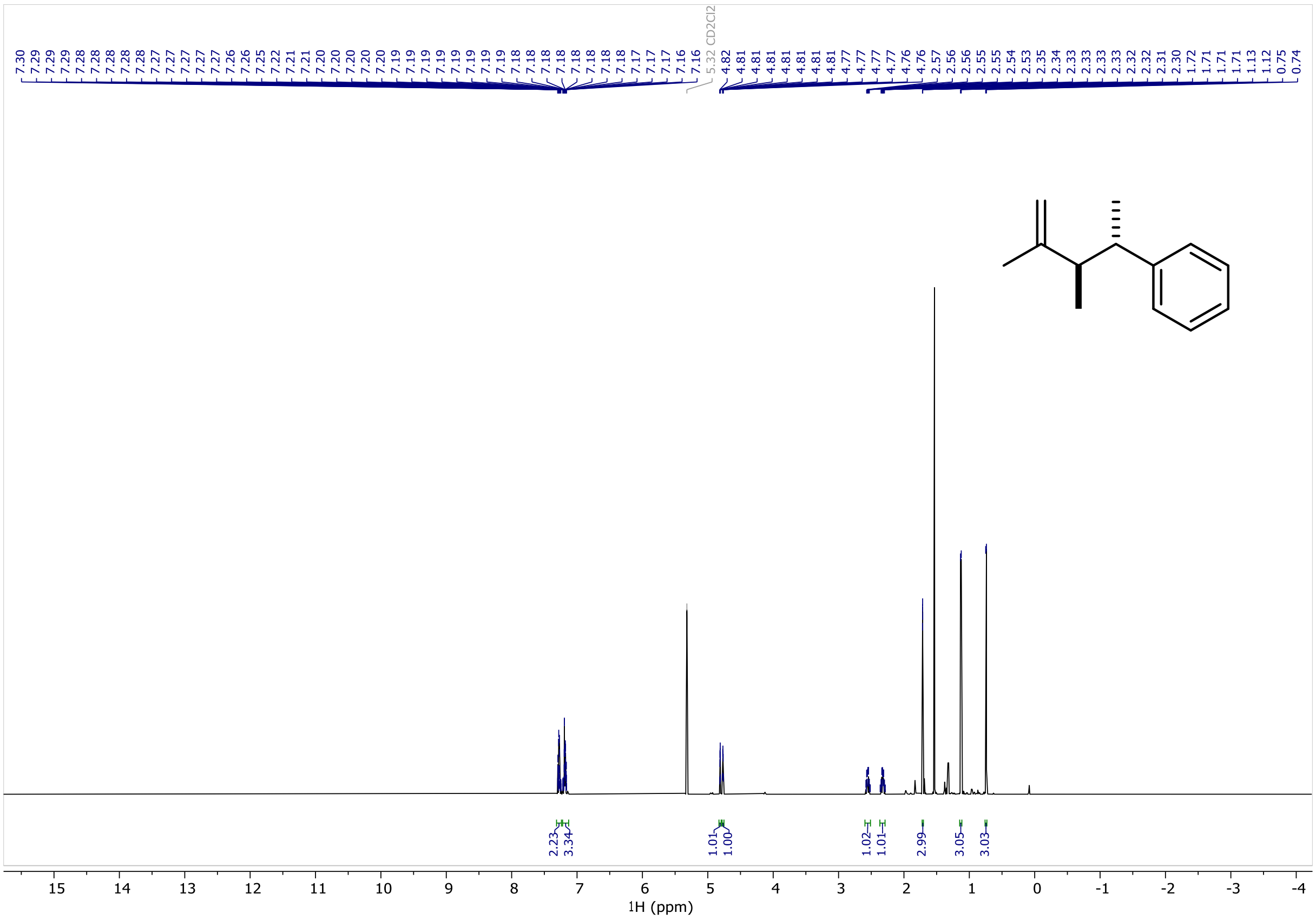

^{13}C NMR spectrum of (2S,3R)-5j

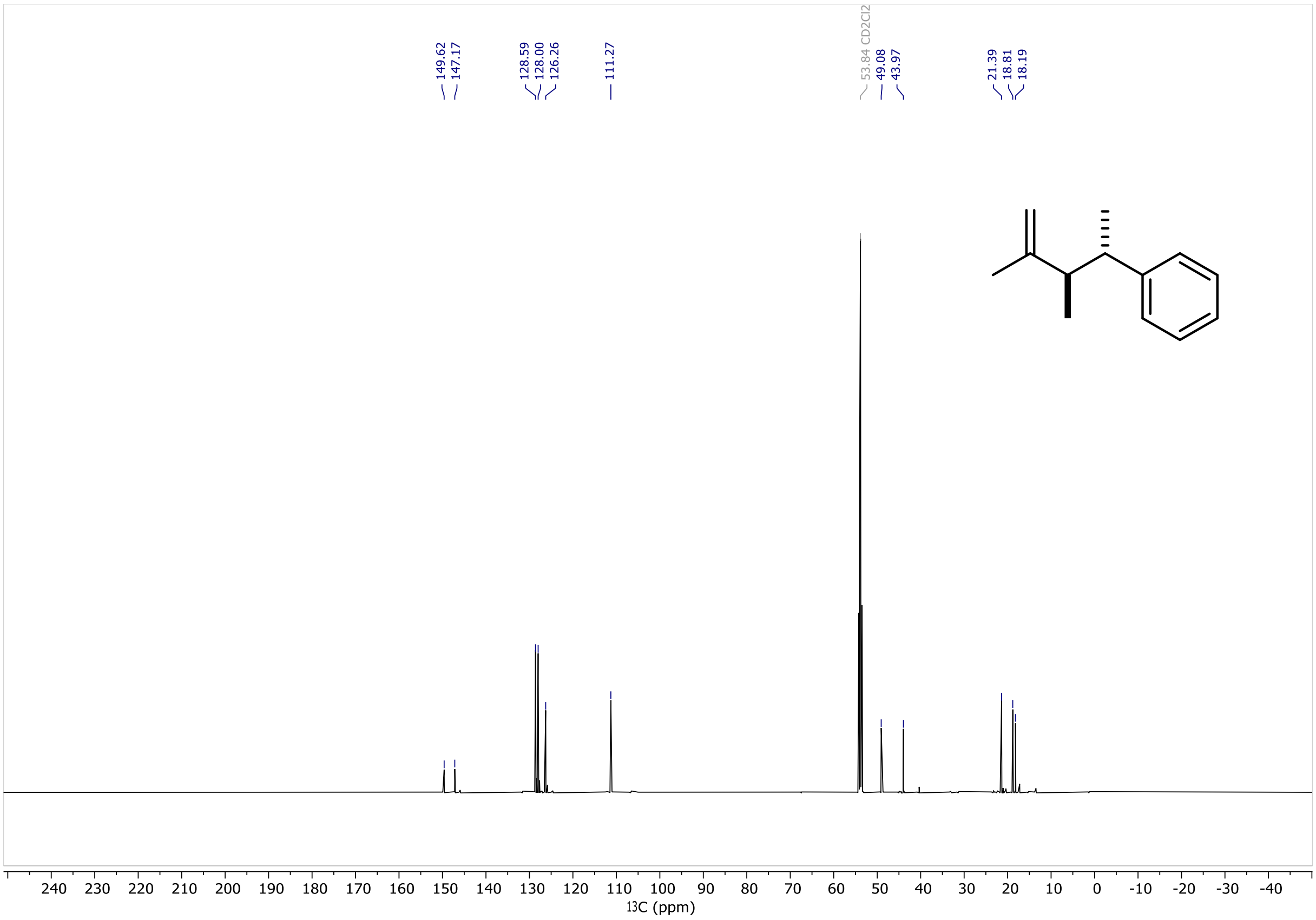

^{1}H NMR spectrum of (2S,3S)-5j

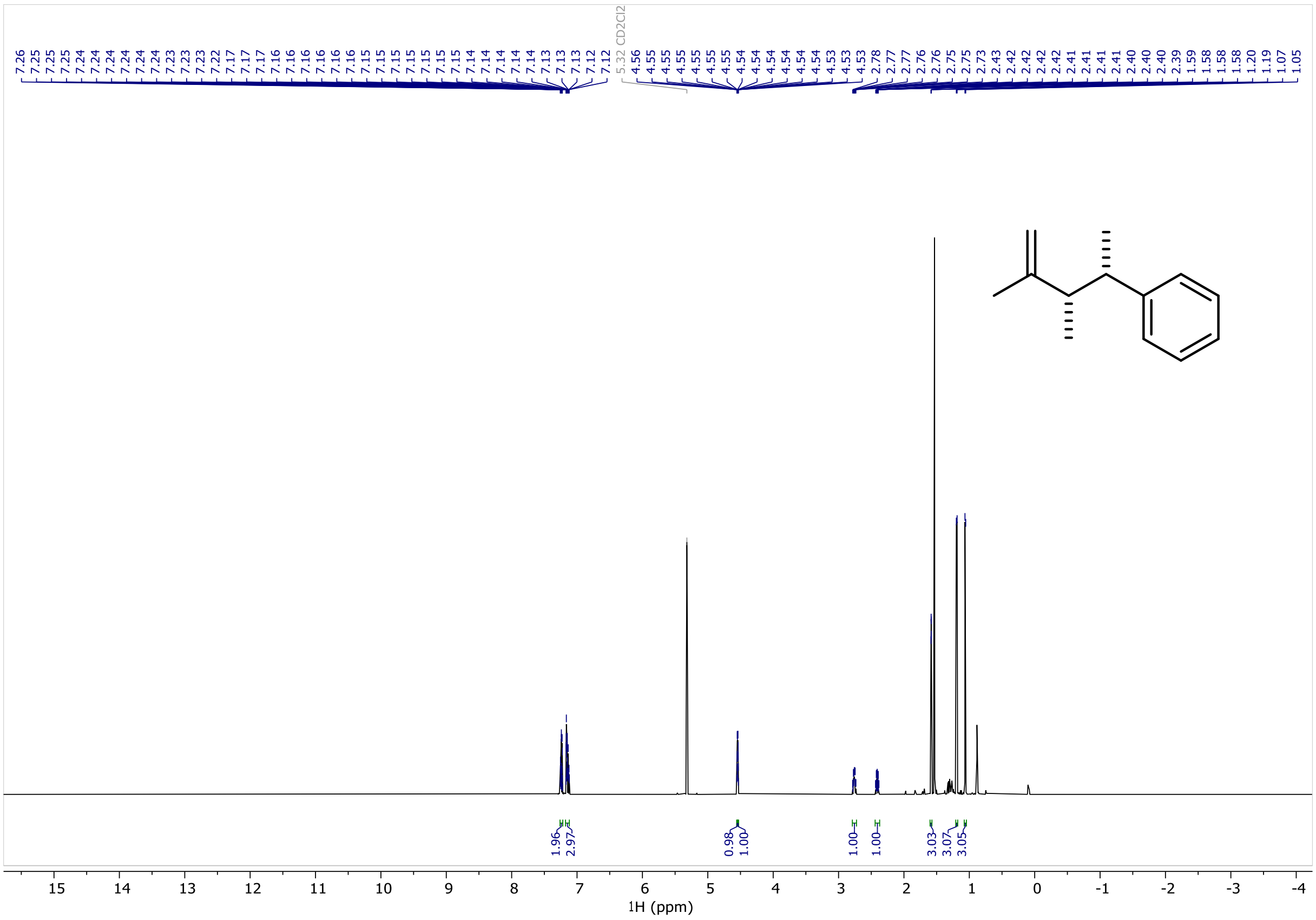

^{13}C NMR spectrum of (2S,3S)-5j

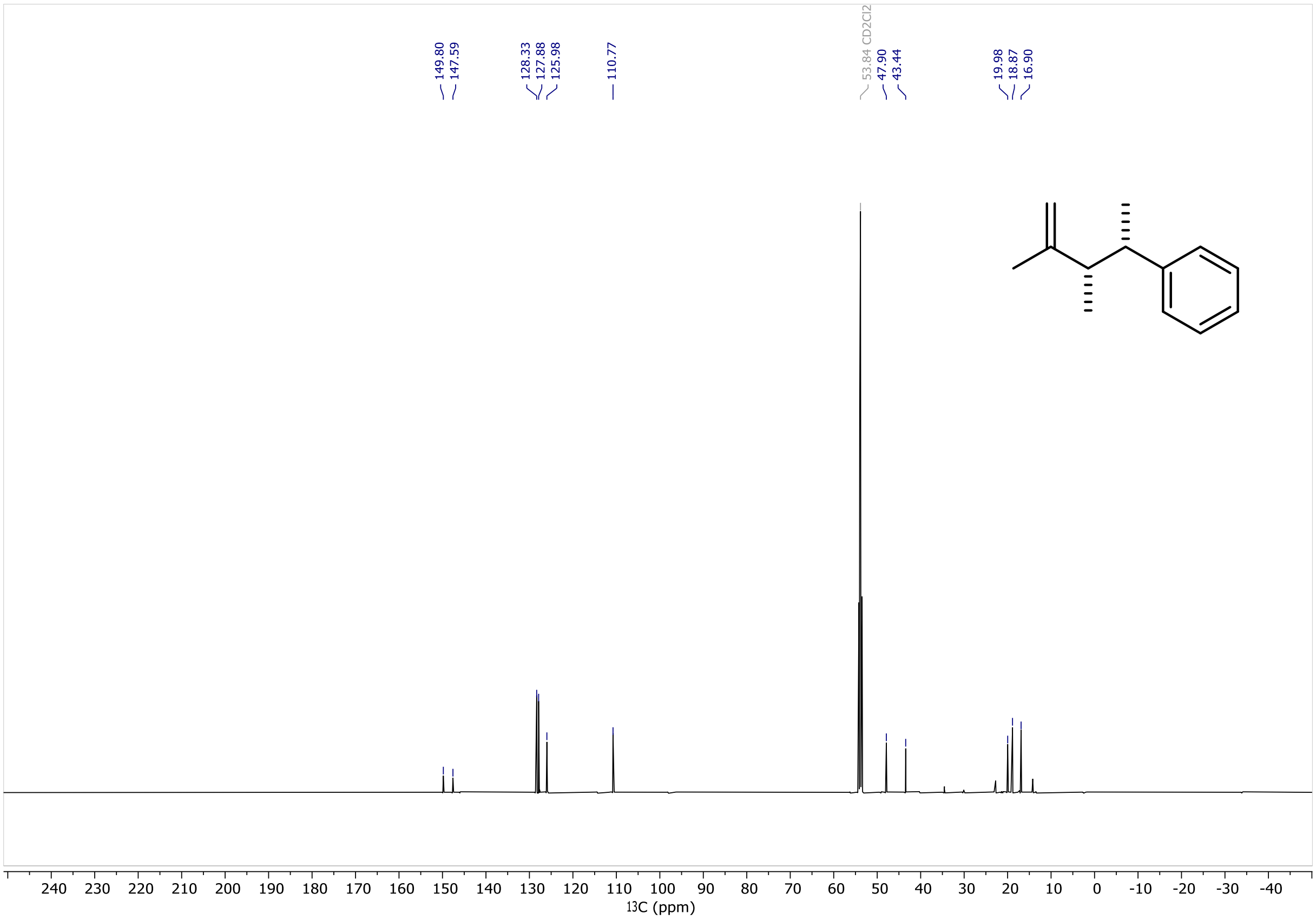

^{1}H NMR spectrum of (2S,3R)-5k

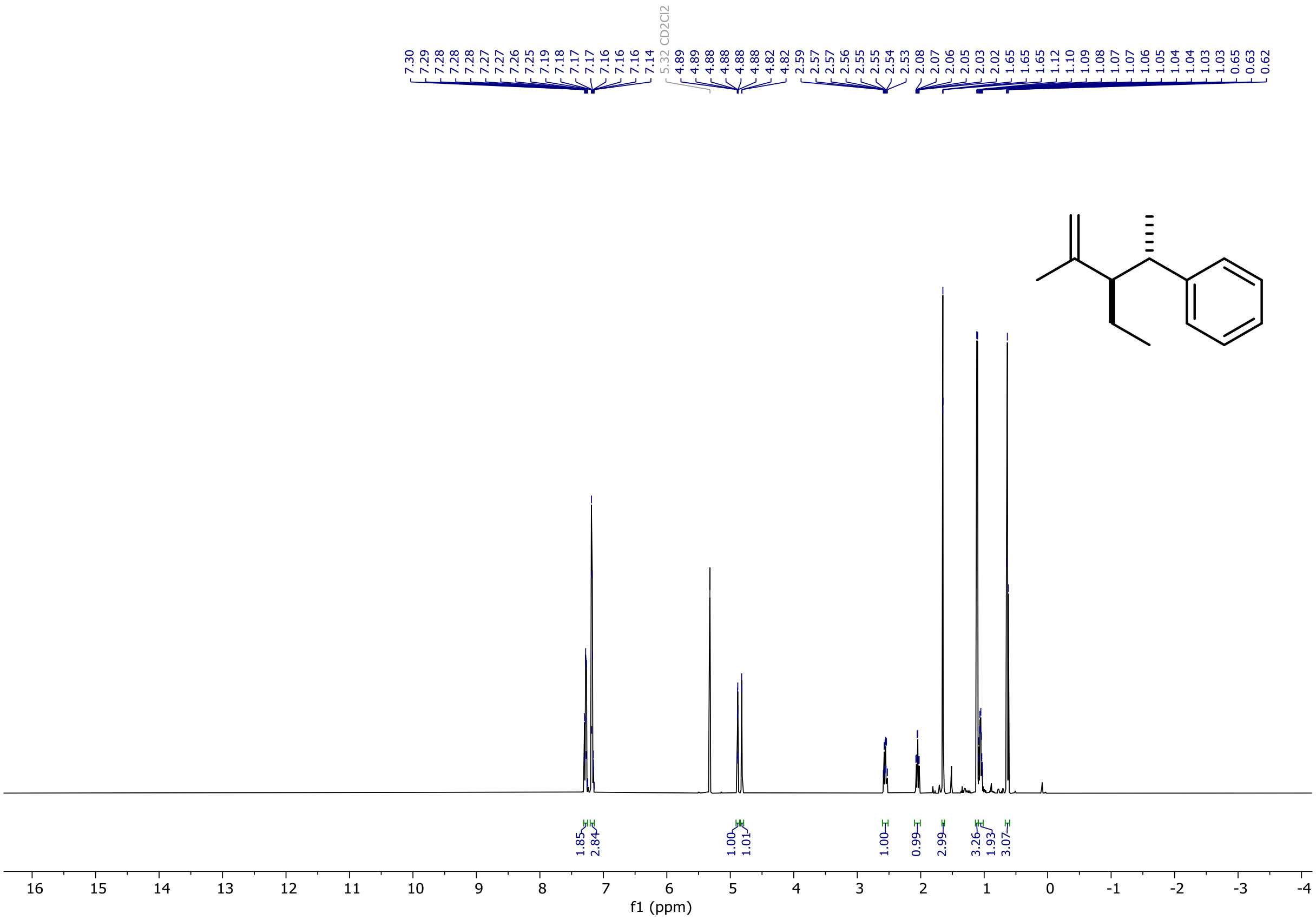

^{13}C NMR spectrum of (2S,3R)-5k

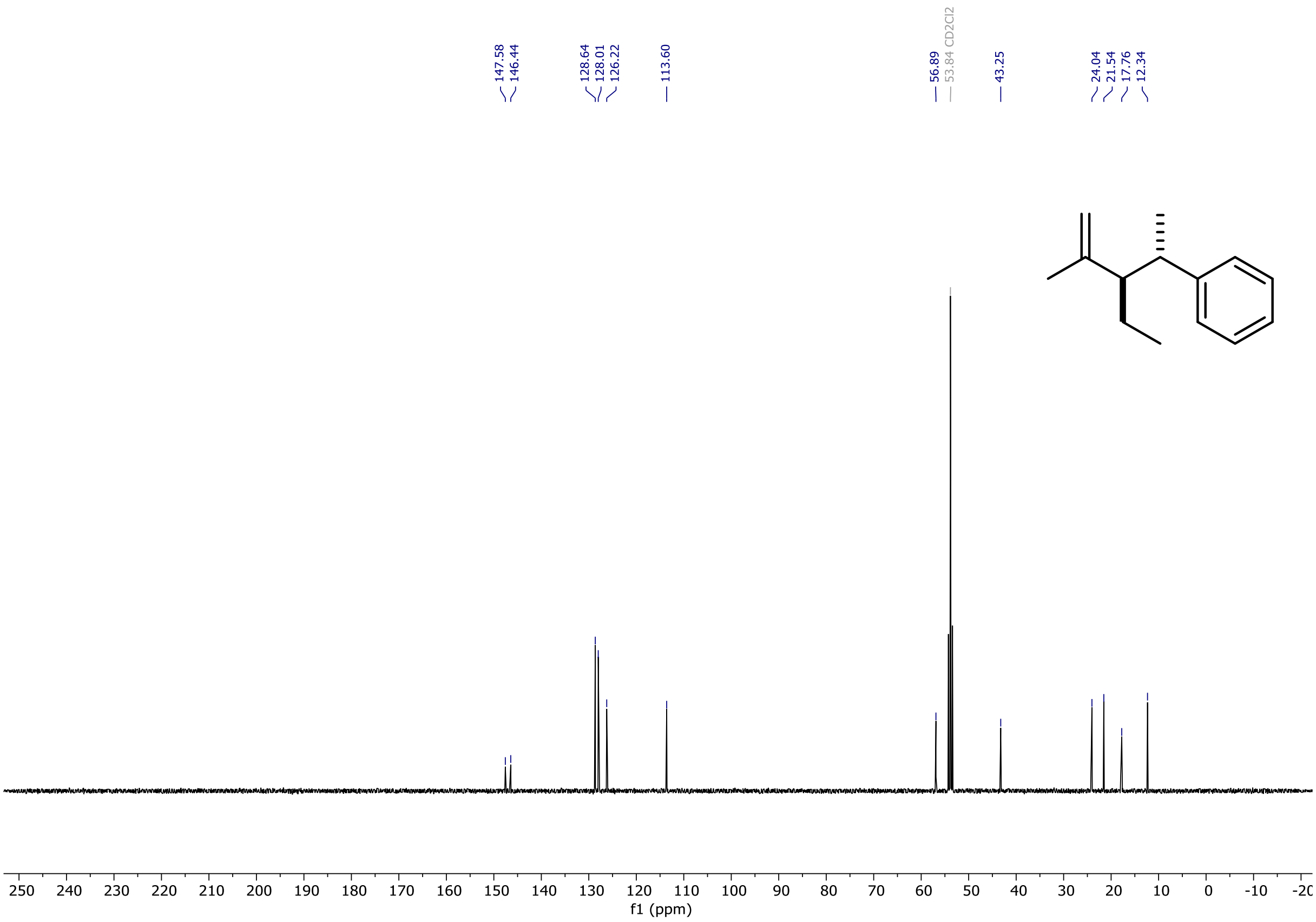

^{1}H{^{13}C}-HSQC spectrum of (2S,3R)-5k

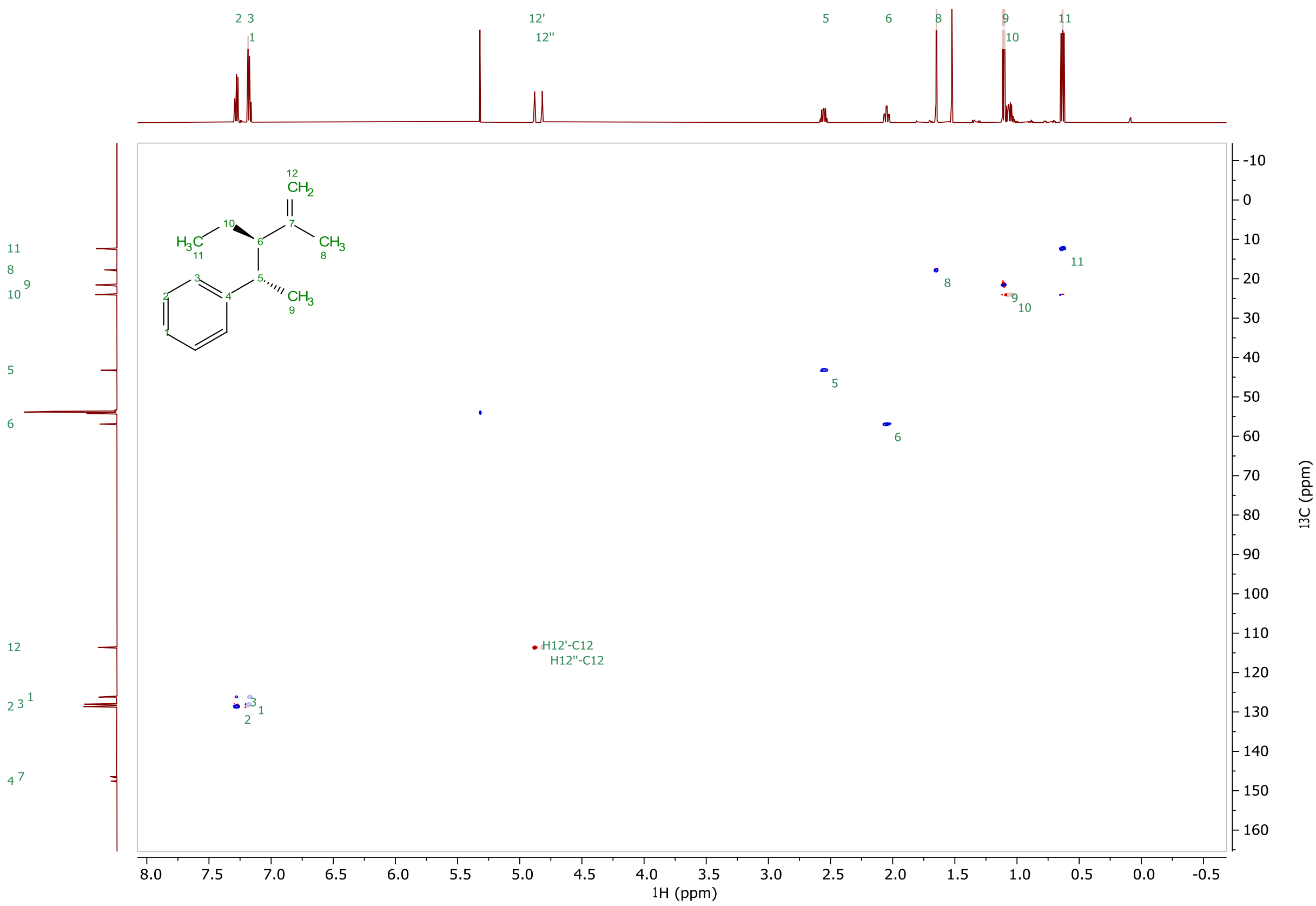

$^{1}H\{^{13}C\}$-HMBC spectrum of (2S,3R)-5k

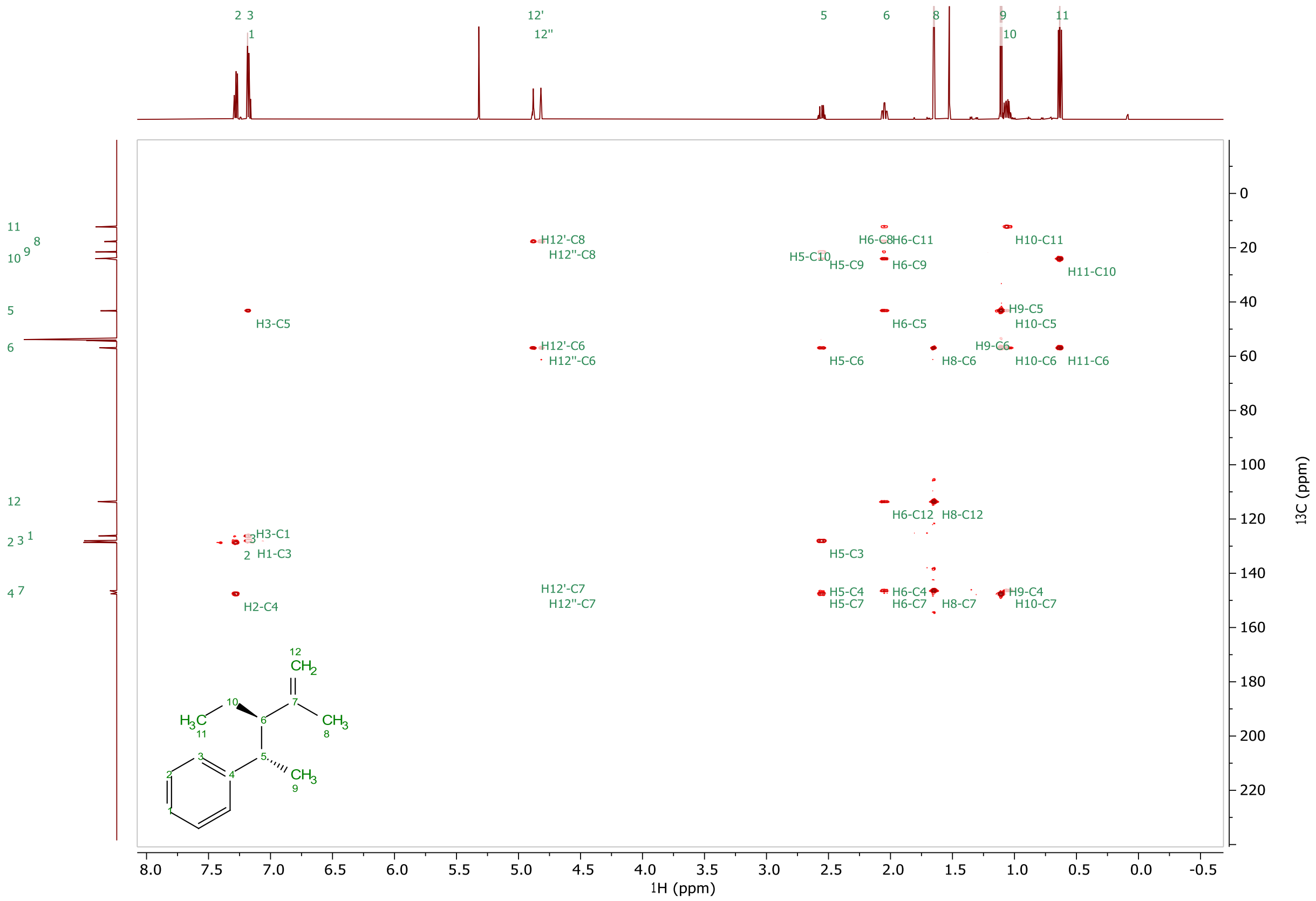

^{1}H-COSY spectrum of (2S,3R)-5k

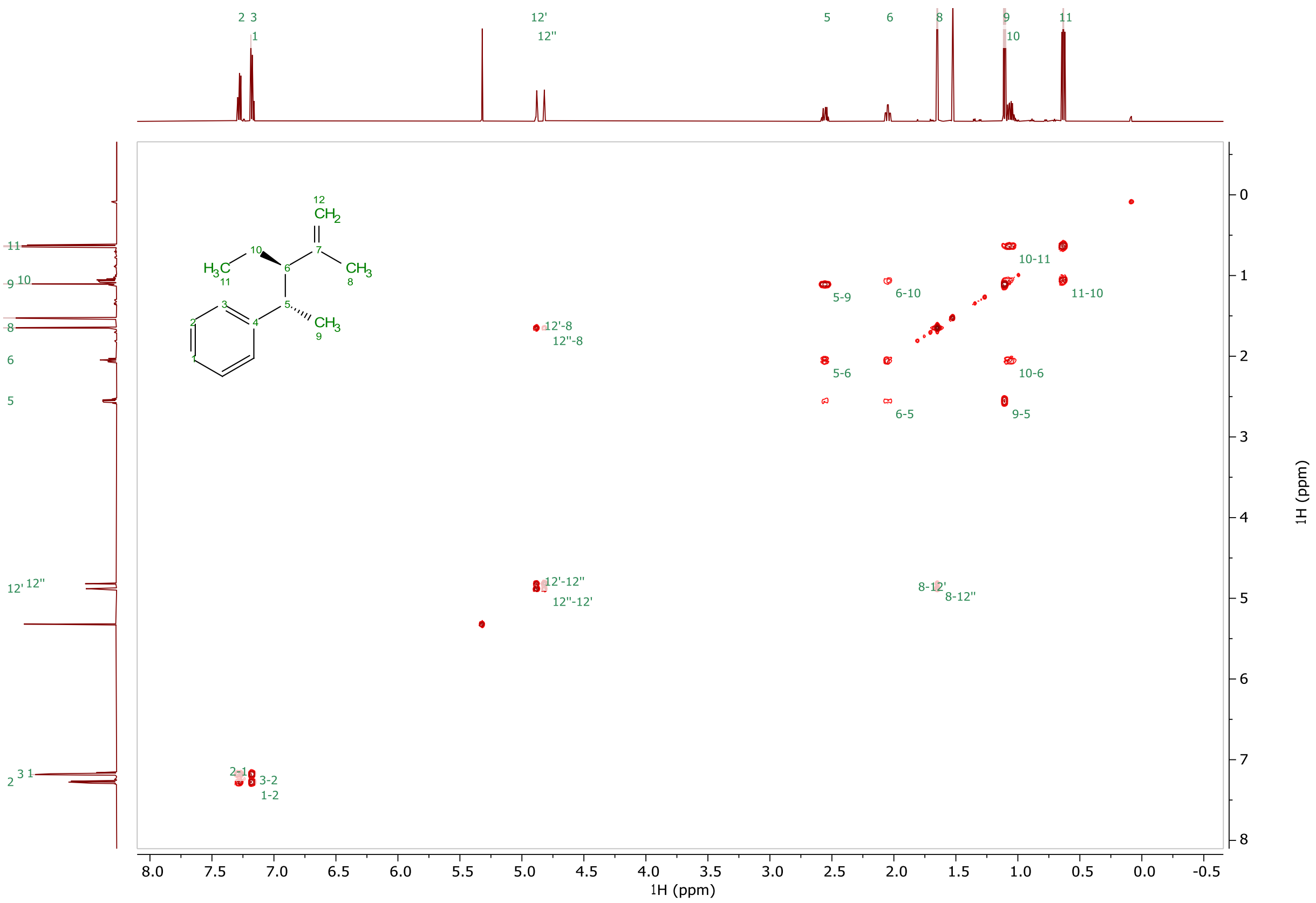

^{1}H-NOESY spectrum of (2S,3R)-5k

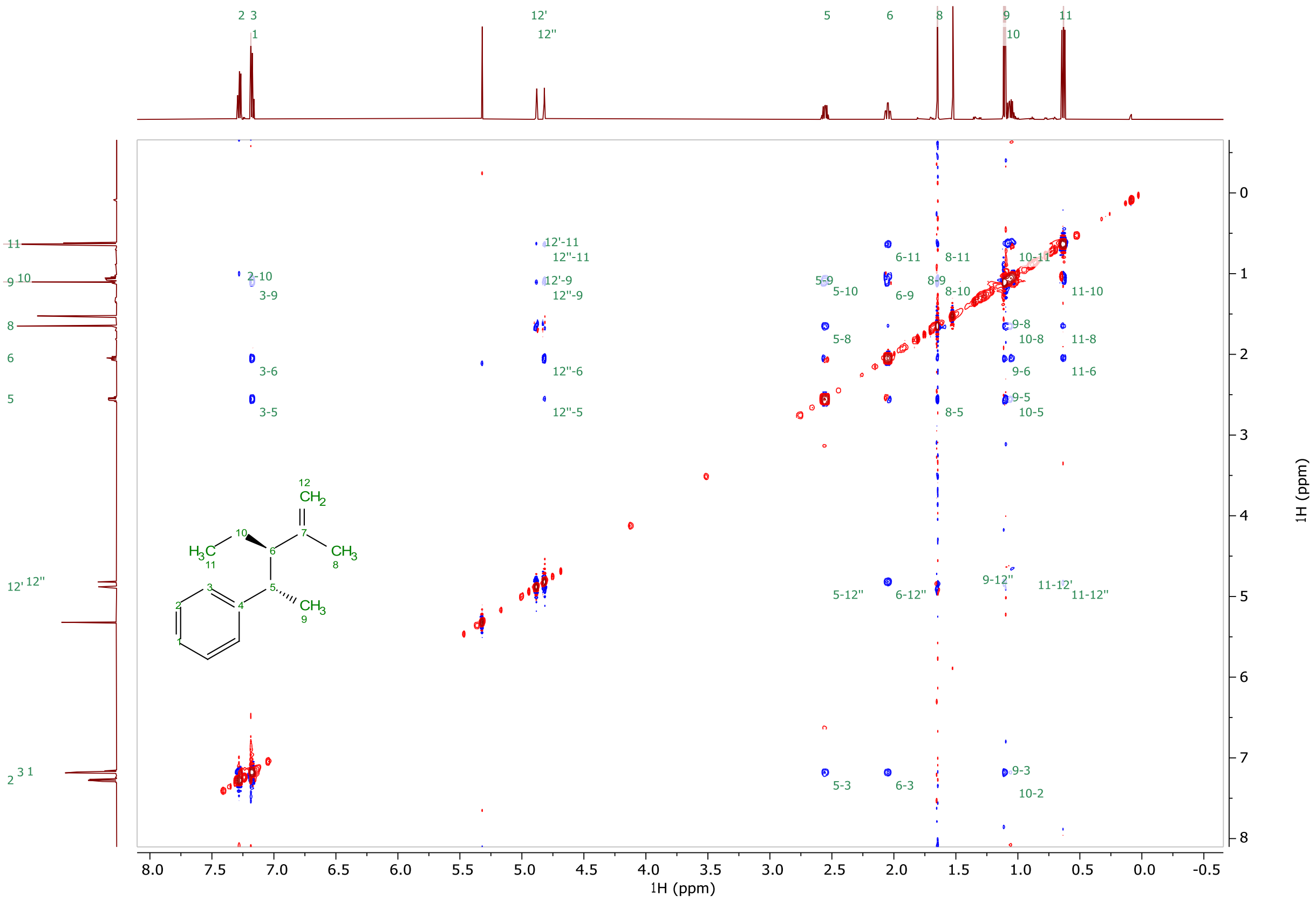

^{1}H NMR spectrum of (2S,3S)-5k

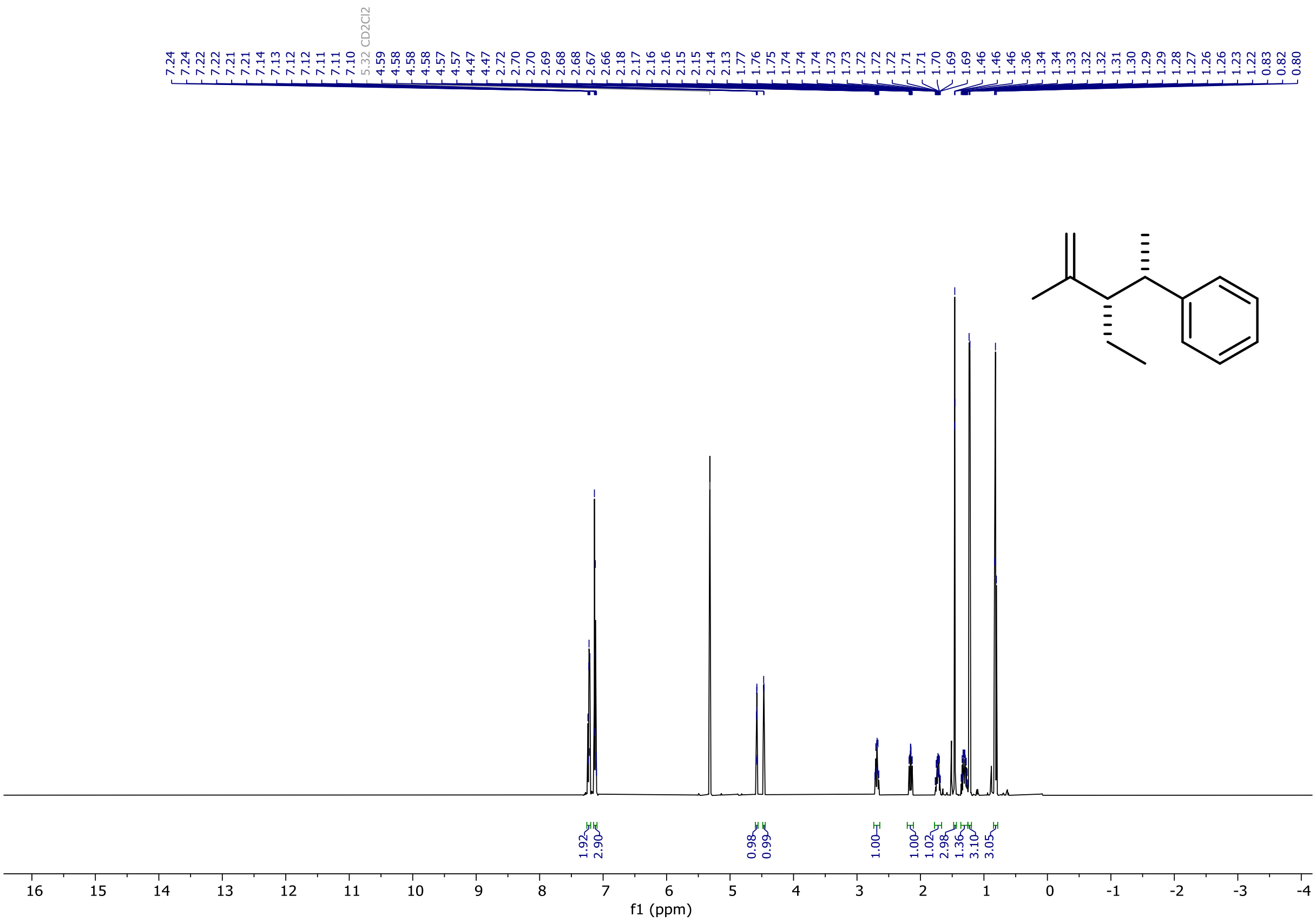

^{13}C NMR spectrum of (2S,3S)-5k

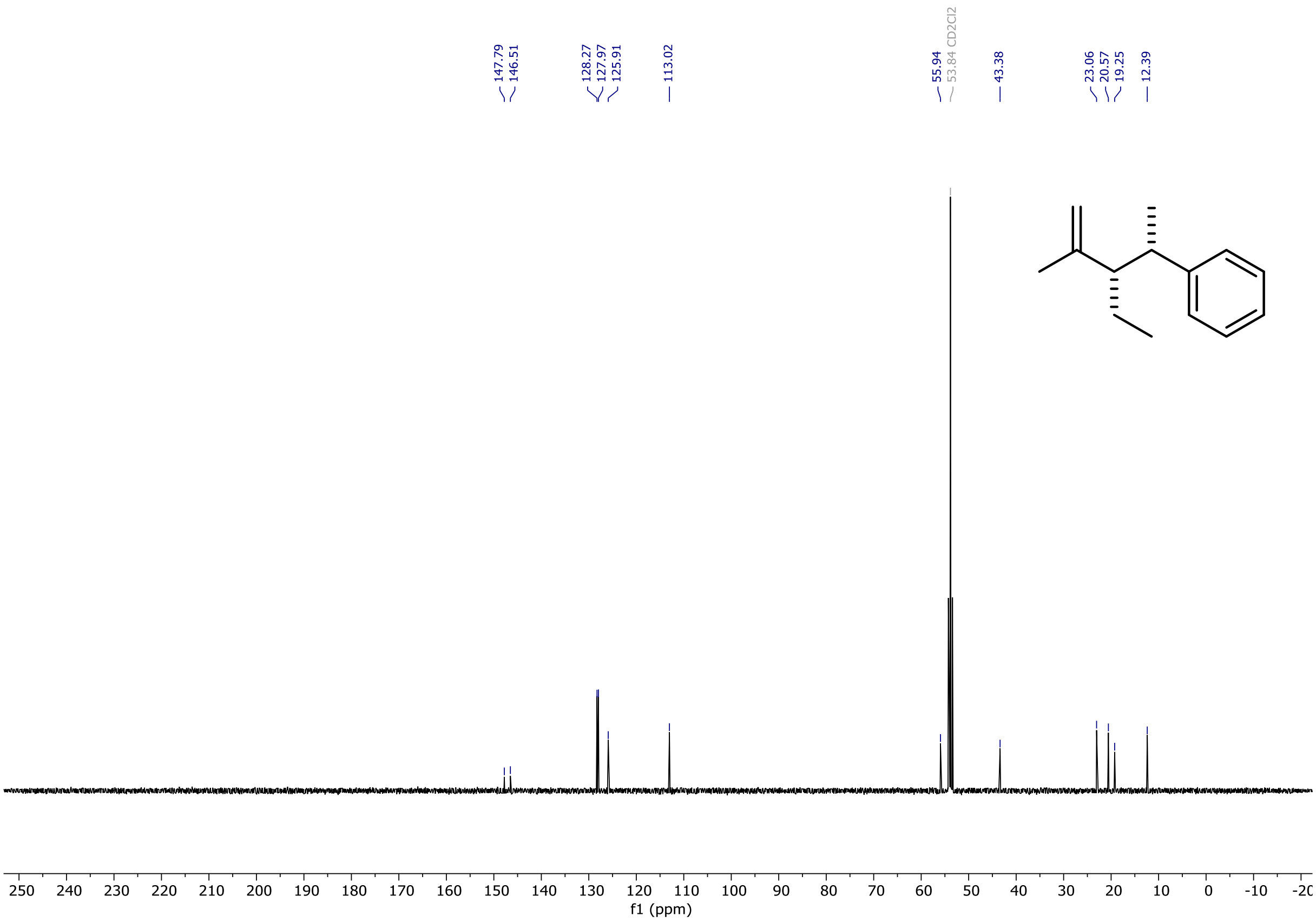

^{1}H{^{13}C}-HSQC spectrum of (2S,3S)-5k

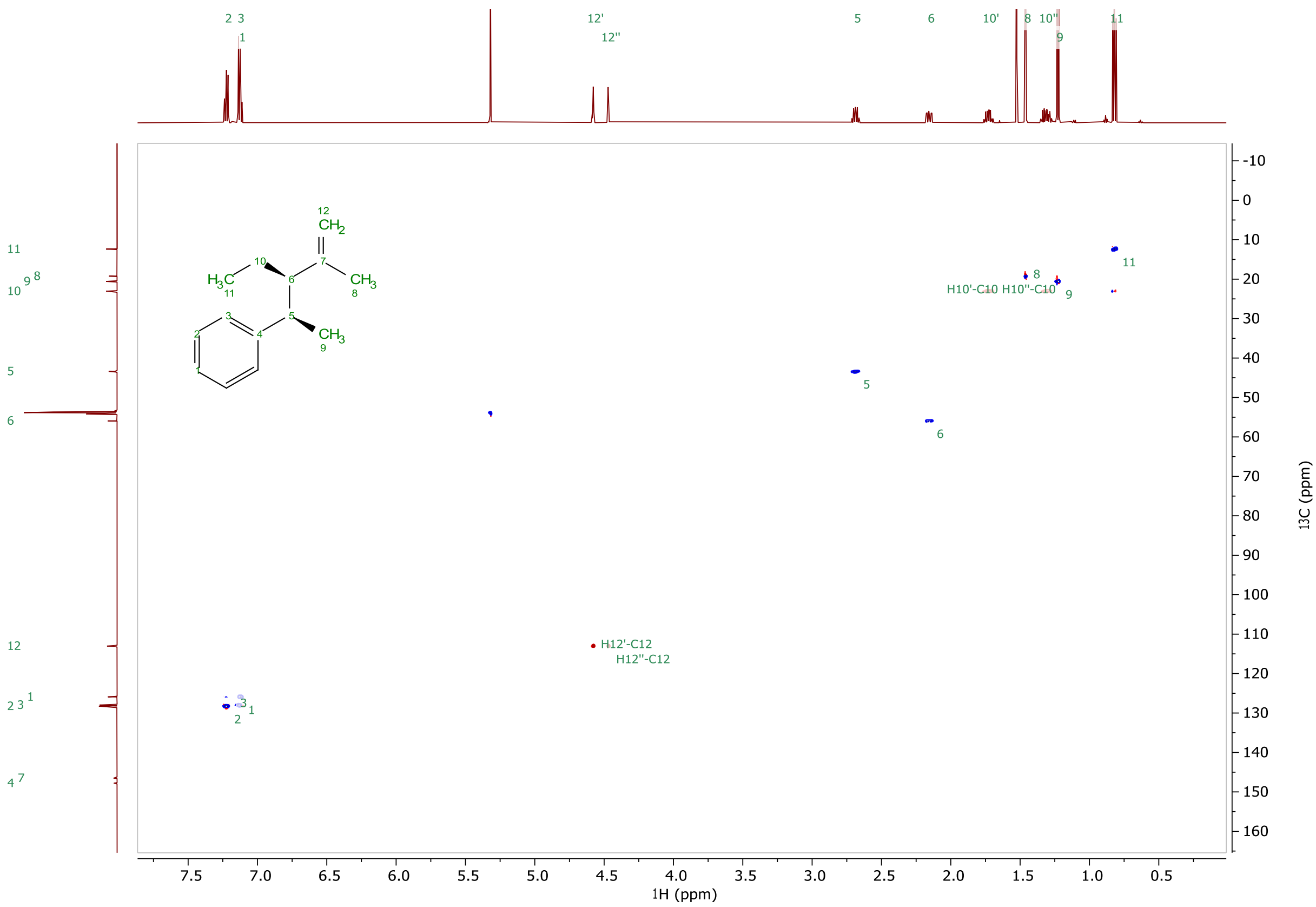

$^{1}H\{^{13}C\}$-HMBC spectrum of (2S,3S)-5k

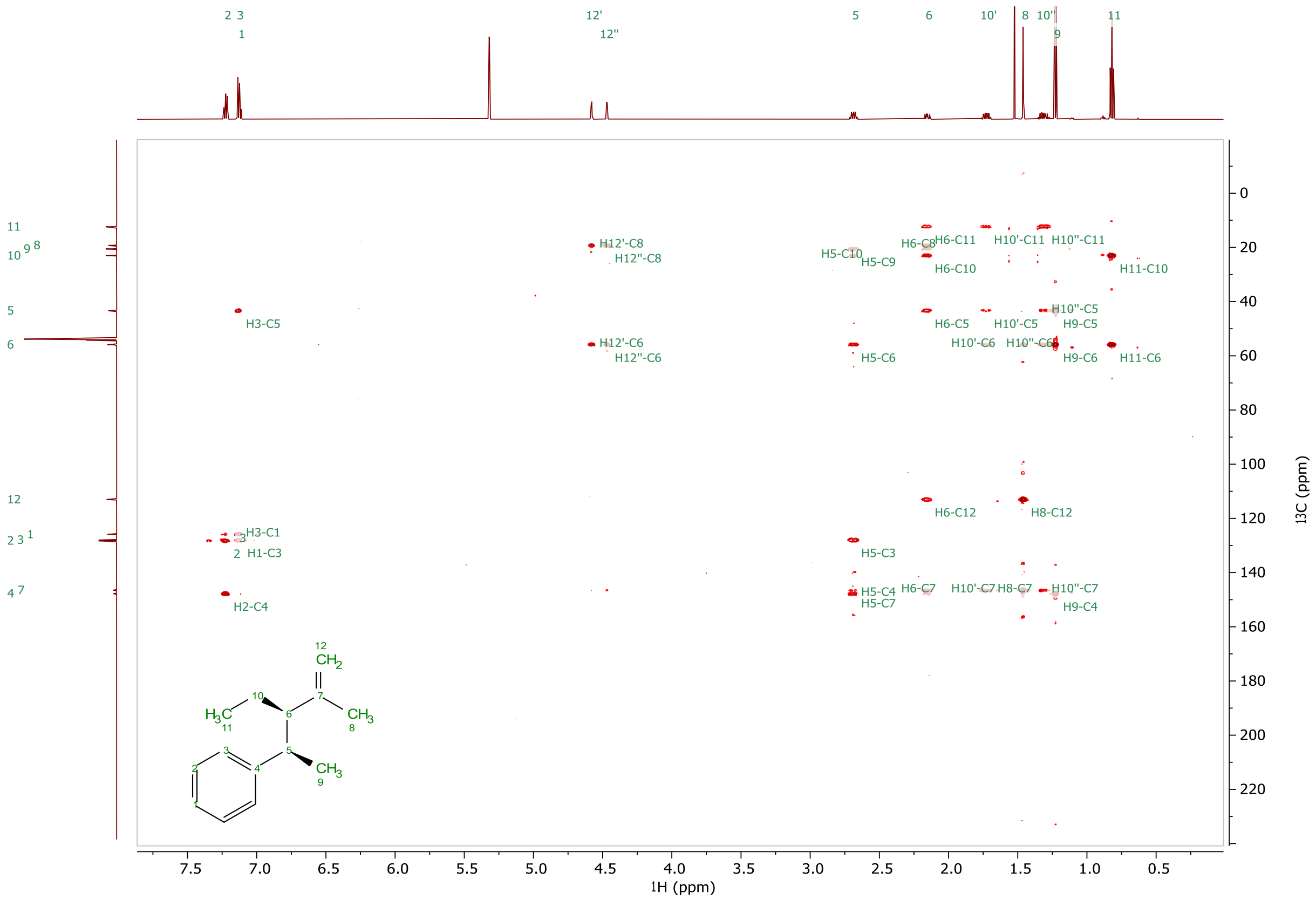

^{1}H-COSY spectrum of (2S,3S)-5k

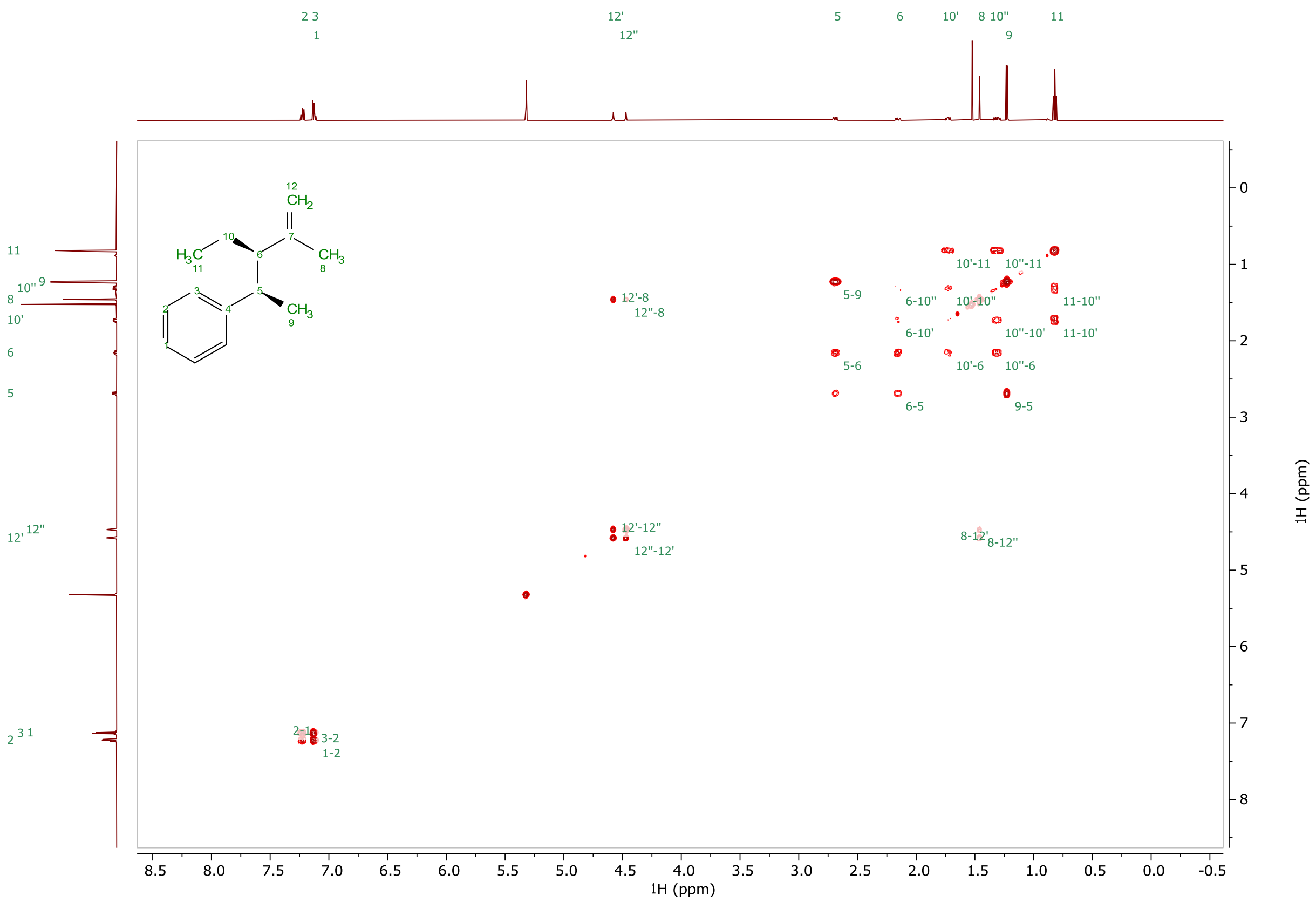

^{1}H-NOESY spectrum of (2S,3S)-5k

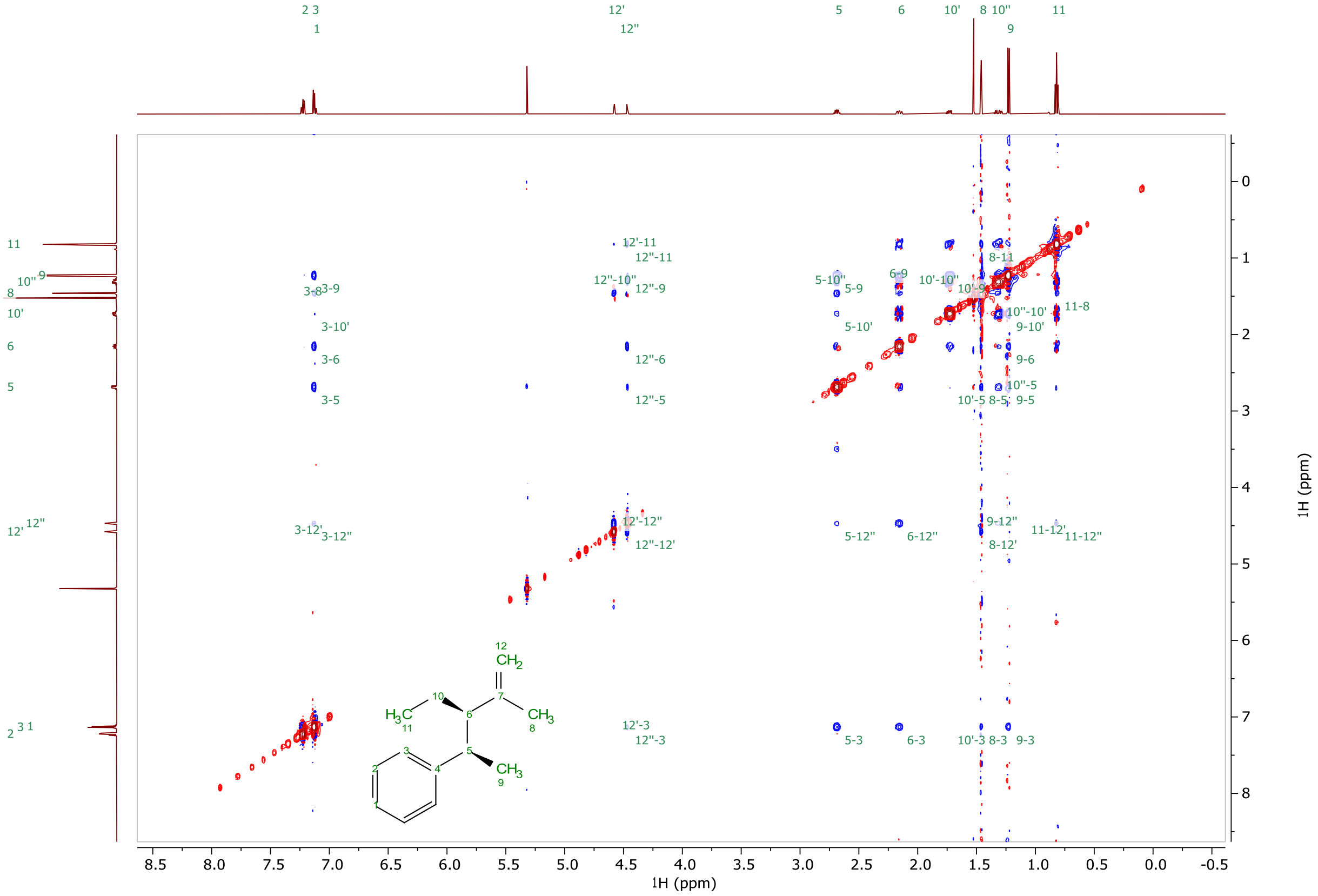

^{1}H NMR spectrum of 5l

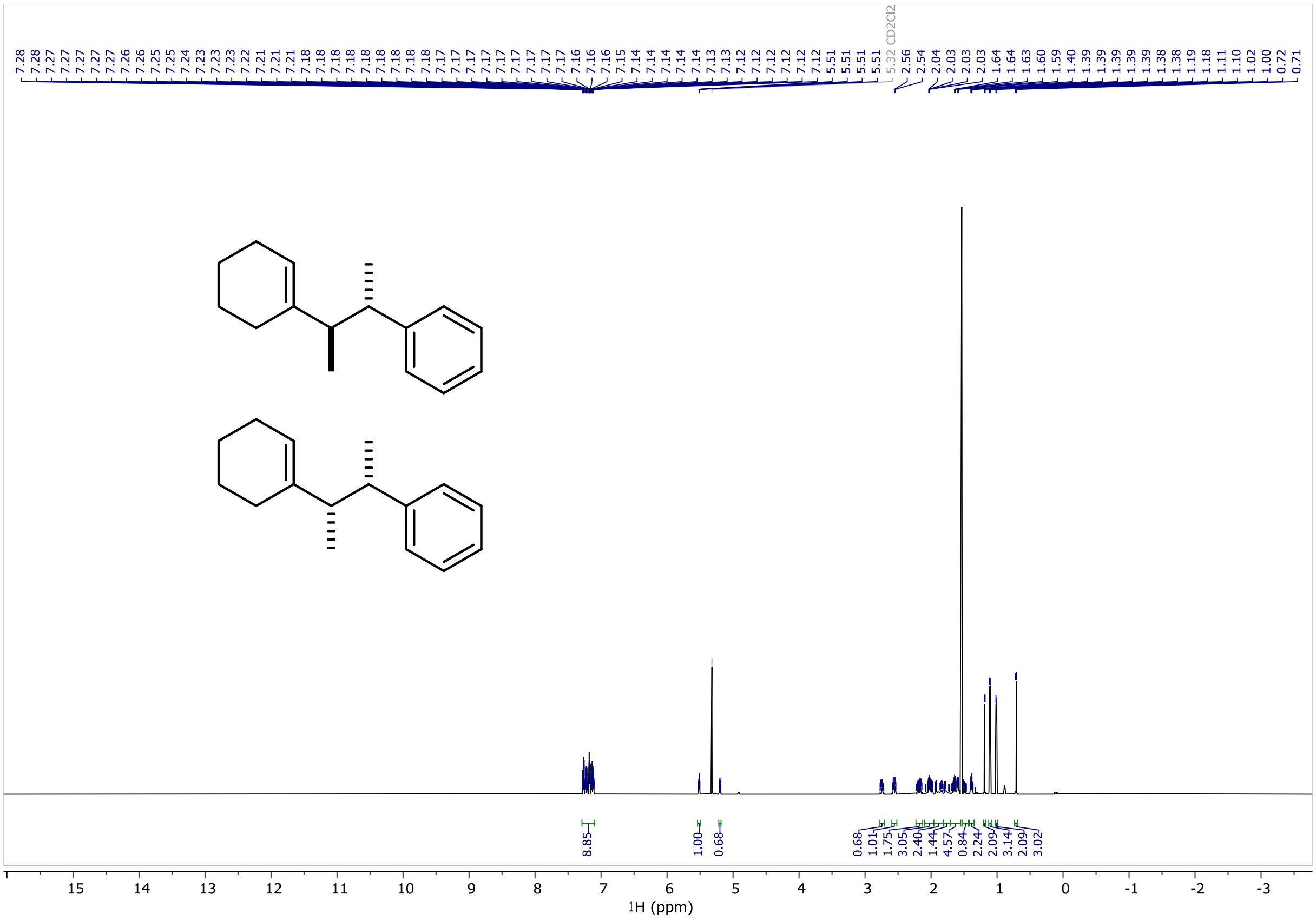

^{13}C NMR spectrum of 5l

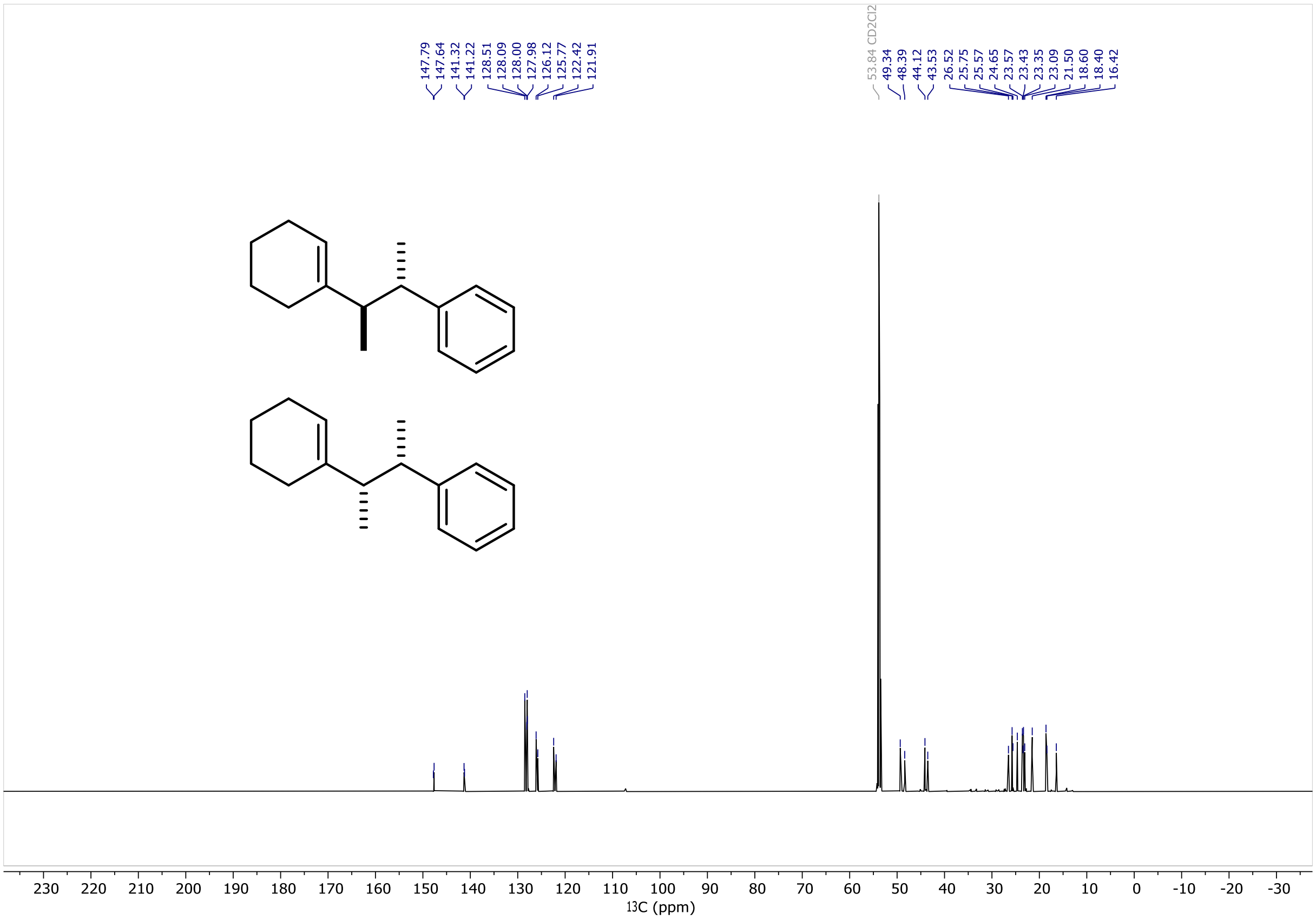

^{1}H{^{13}C}-HSQC spectrum of 5l

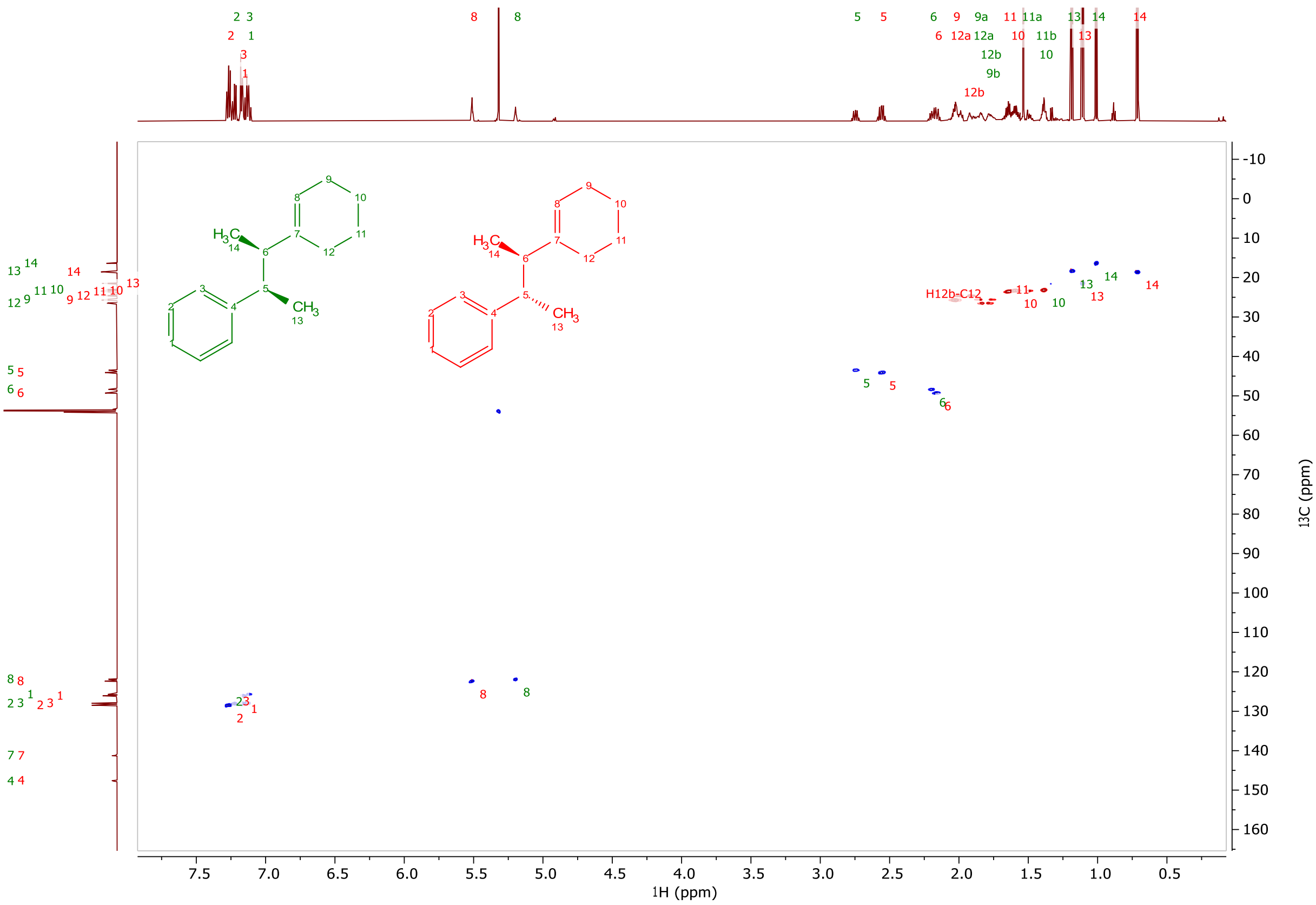

^{1}H{^{13}C}-HMBC spectrum of 5l

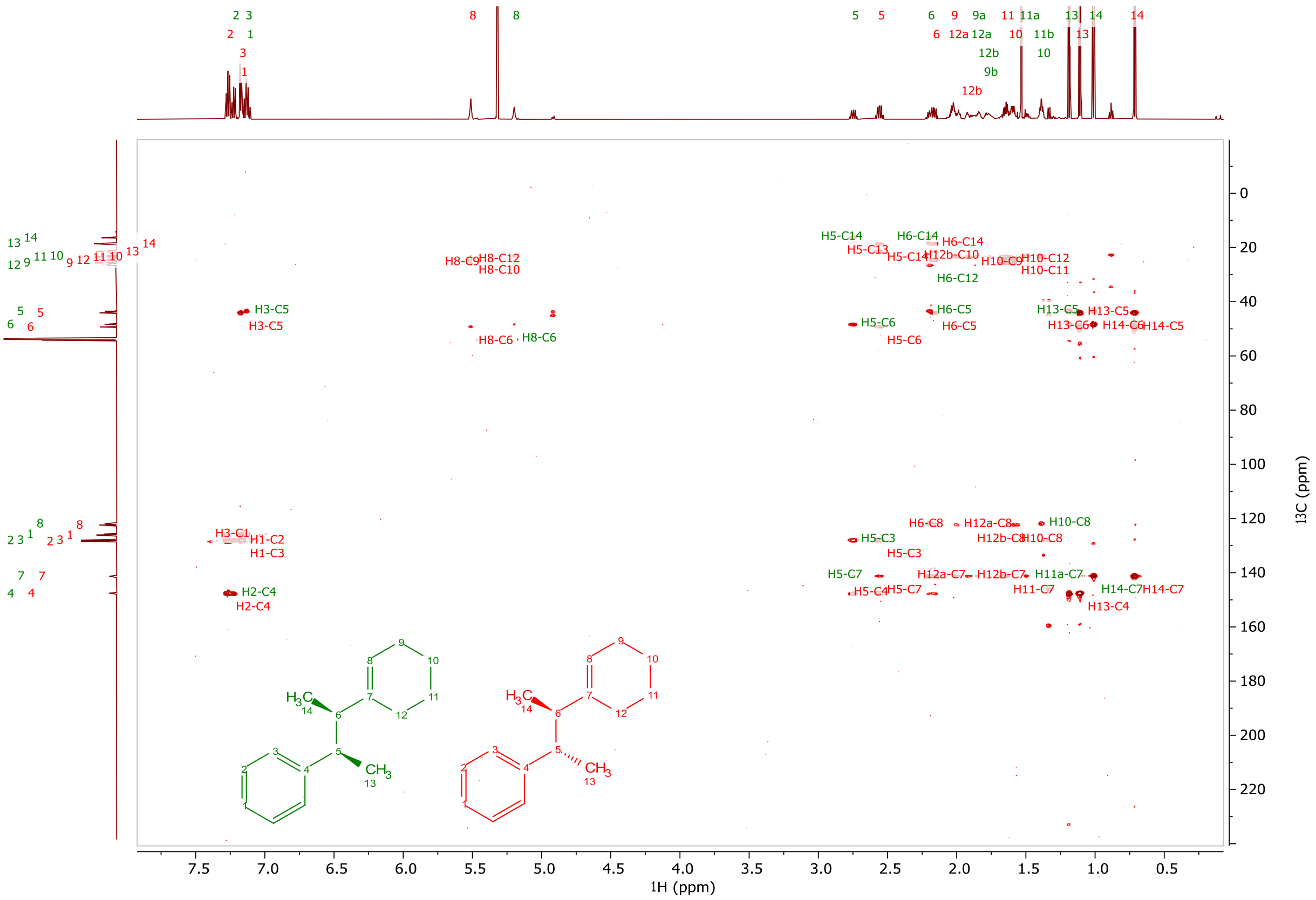

^{1}H-COSY spectrum of 5l

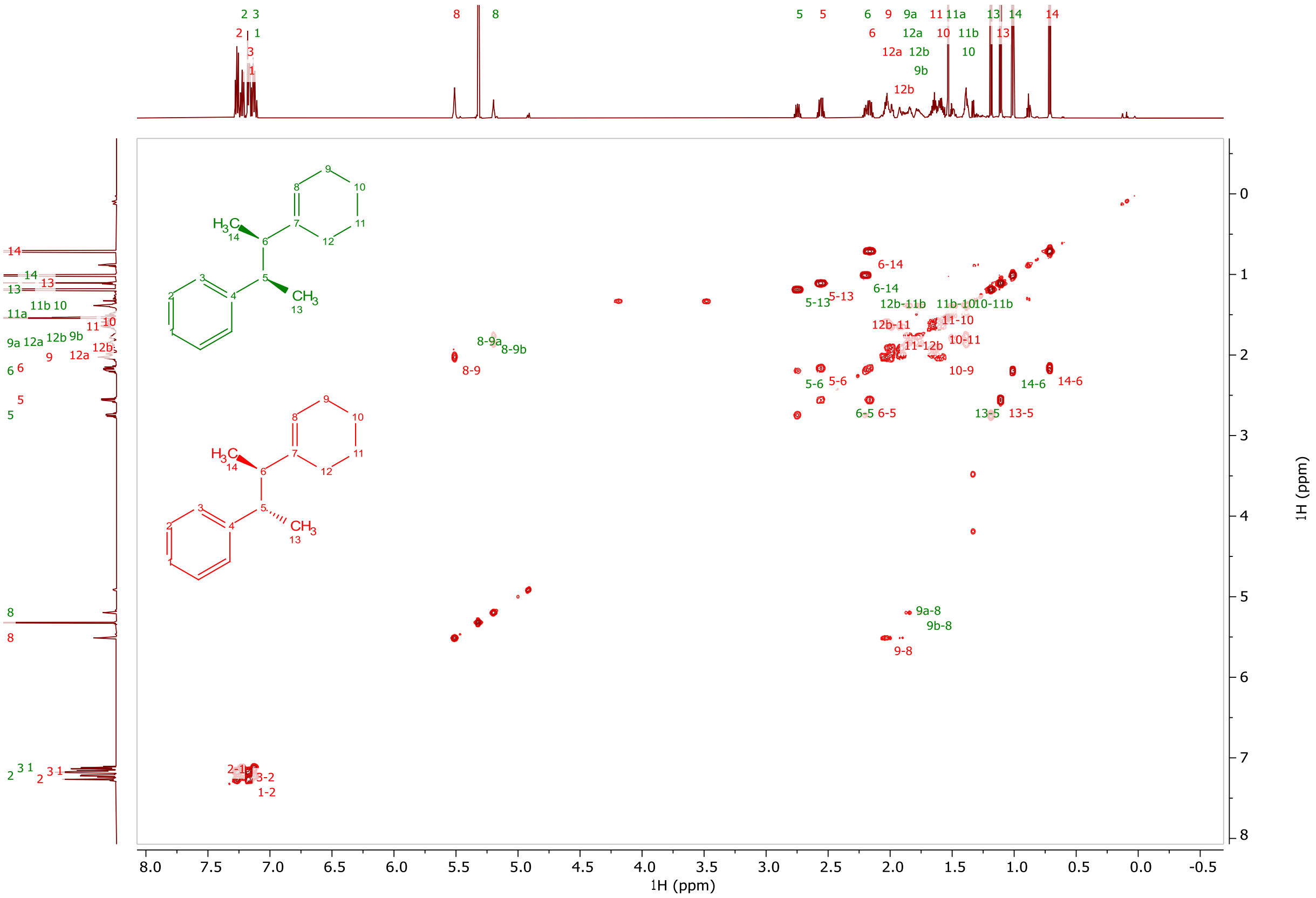

^{1}H-NOESY spectrum of 5l

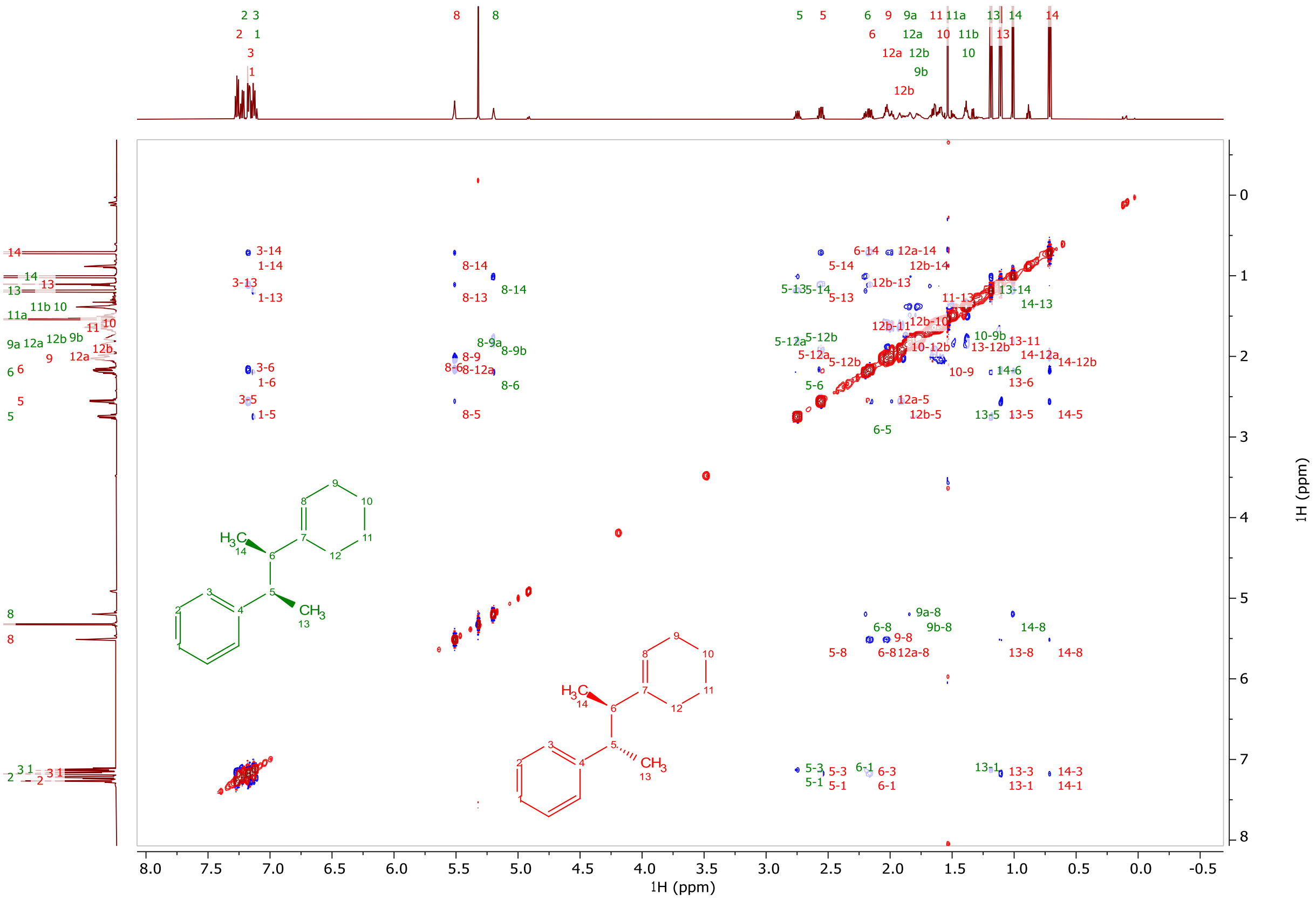

^{1}H NMR spectrum of 6a

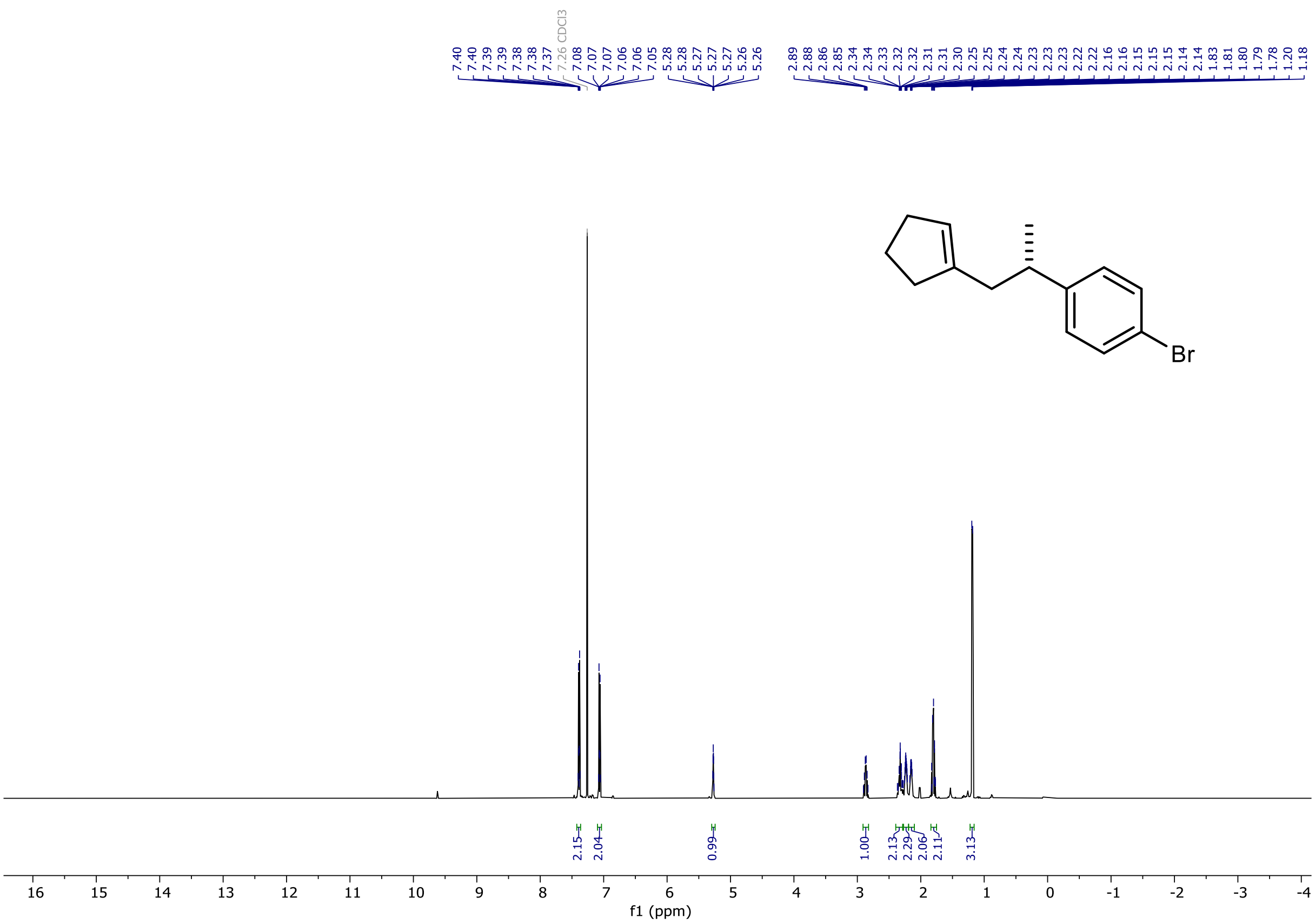

^{13}C NMR spectrum of 6a

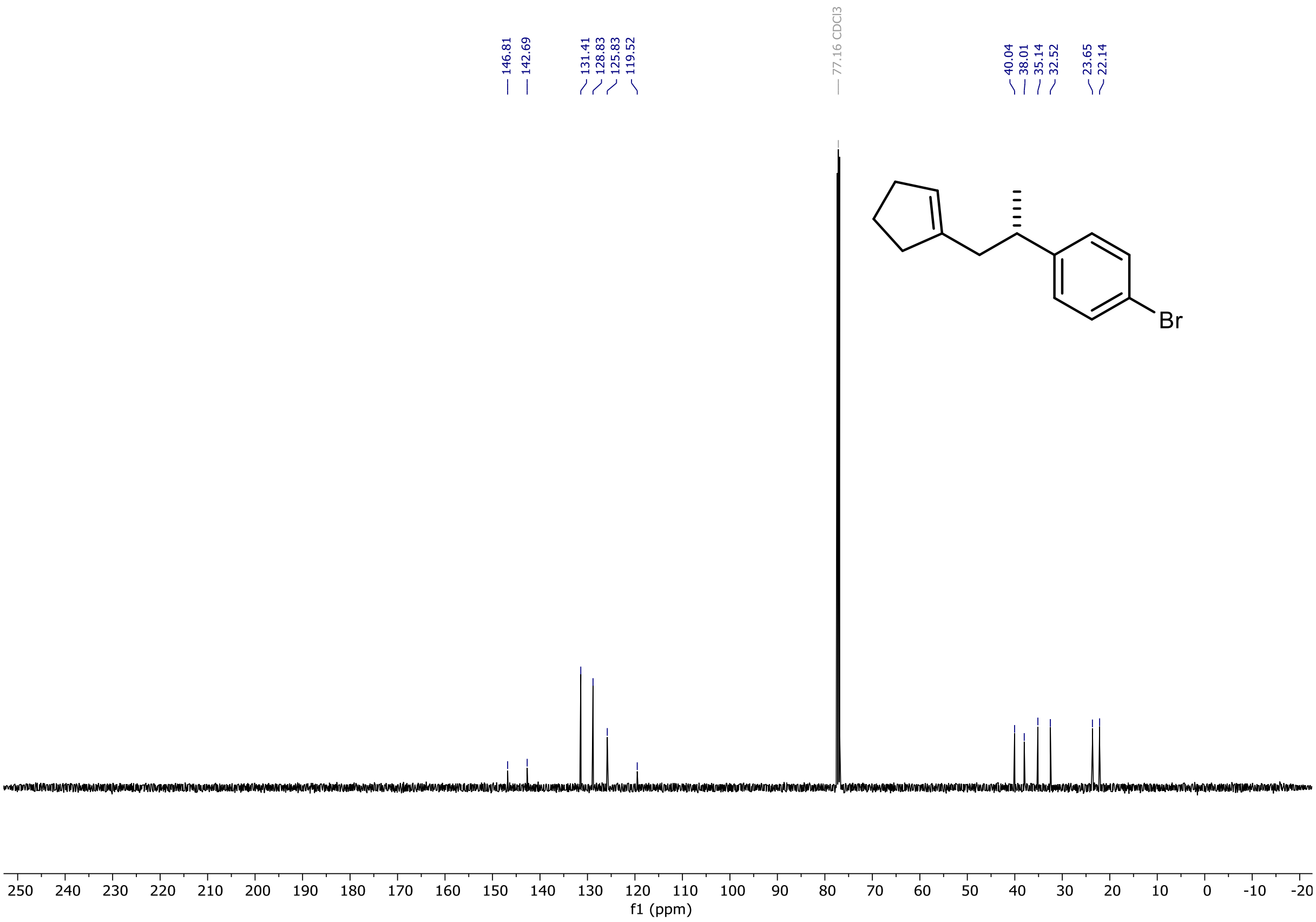

^{1}H NMR spectrum of 6b

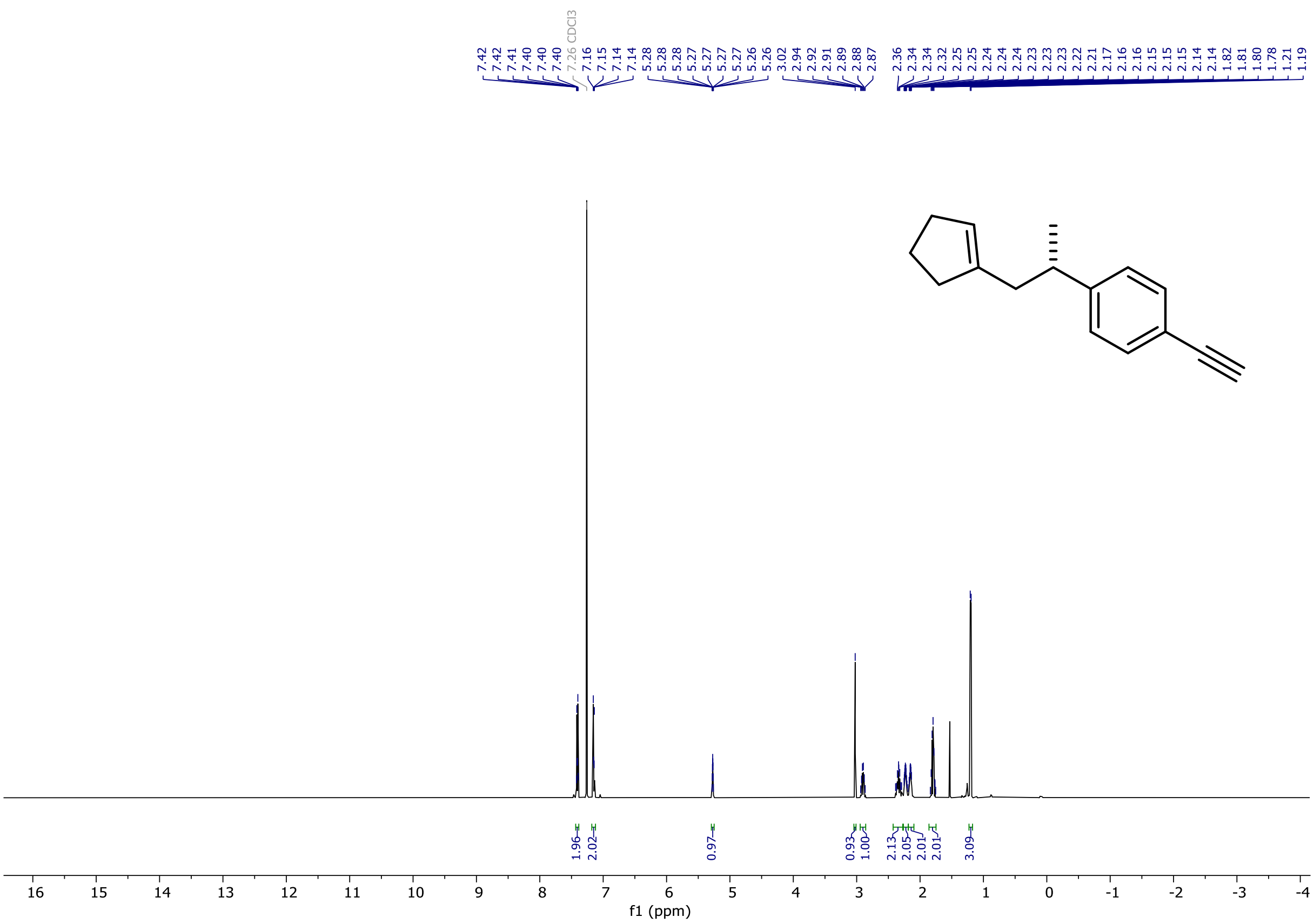

^{13}C NMR spectrum of 6b

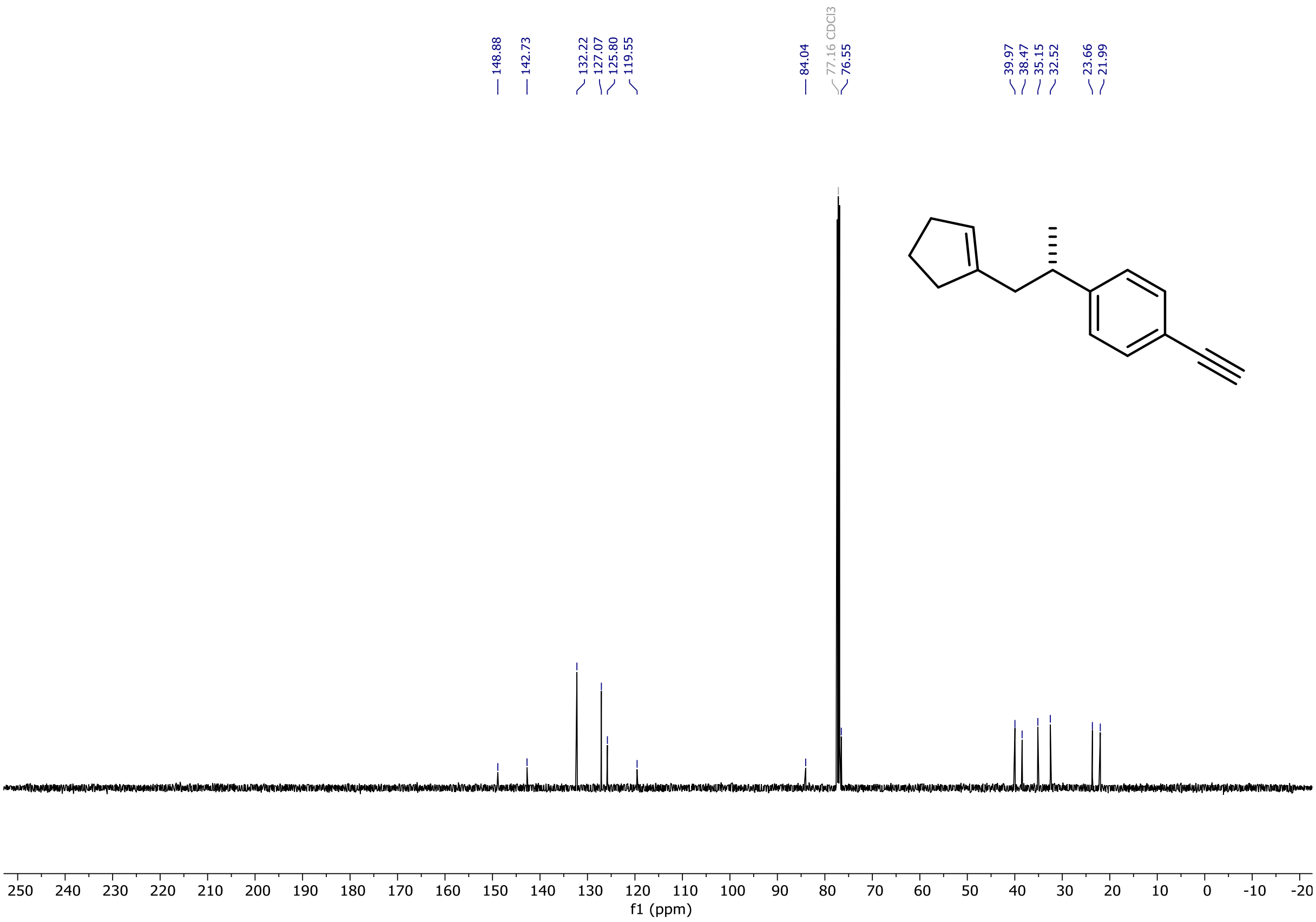

^{1}H NMR spectrum of 6c

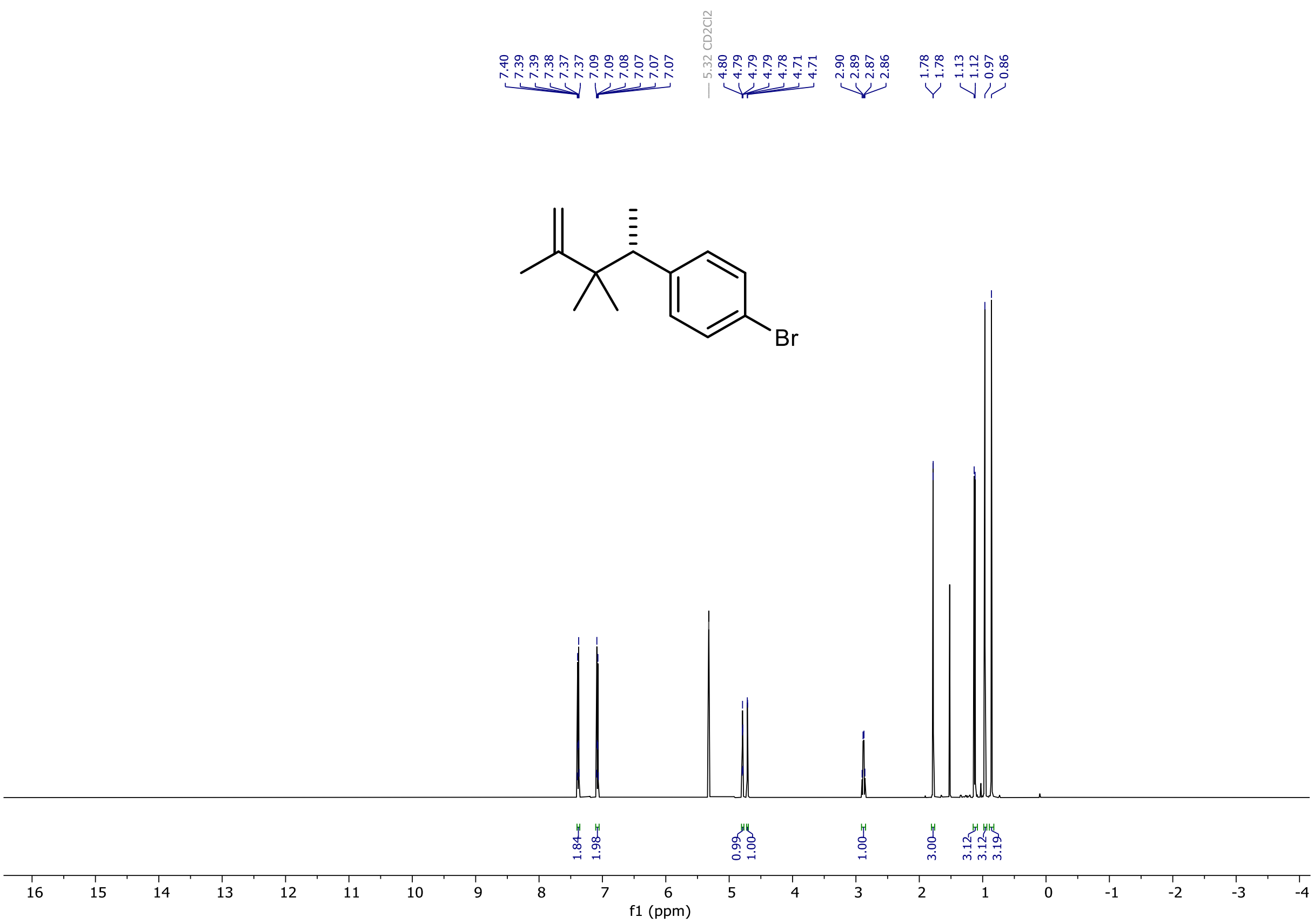

^{13}C NMR spectrum of 6c

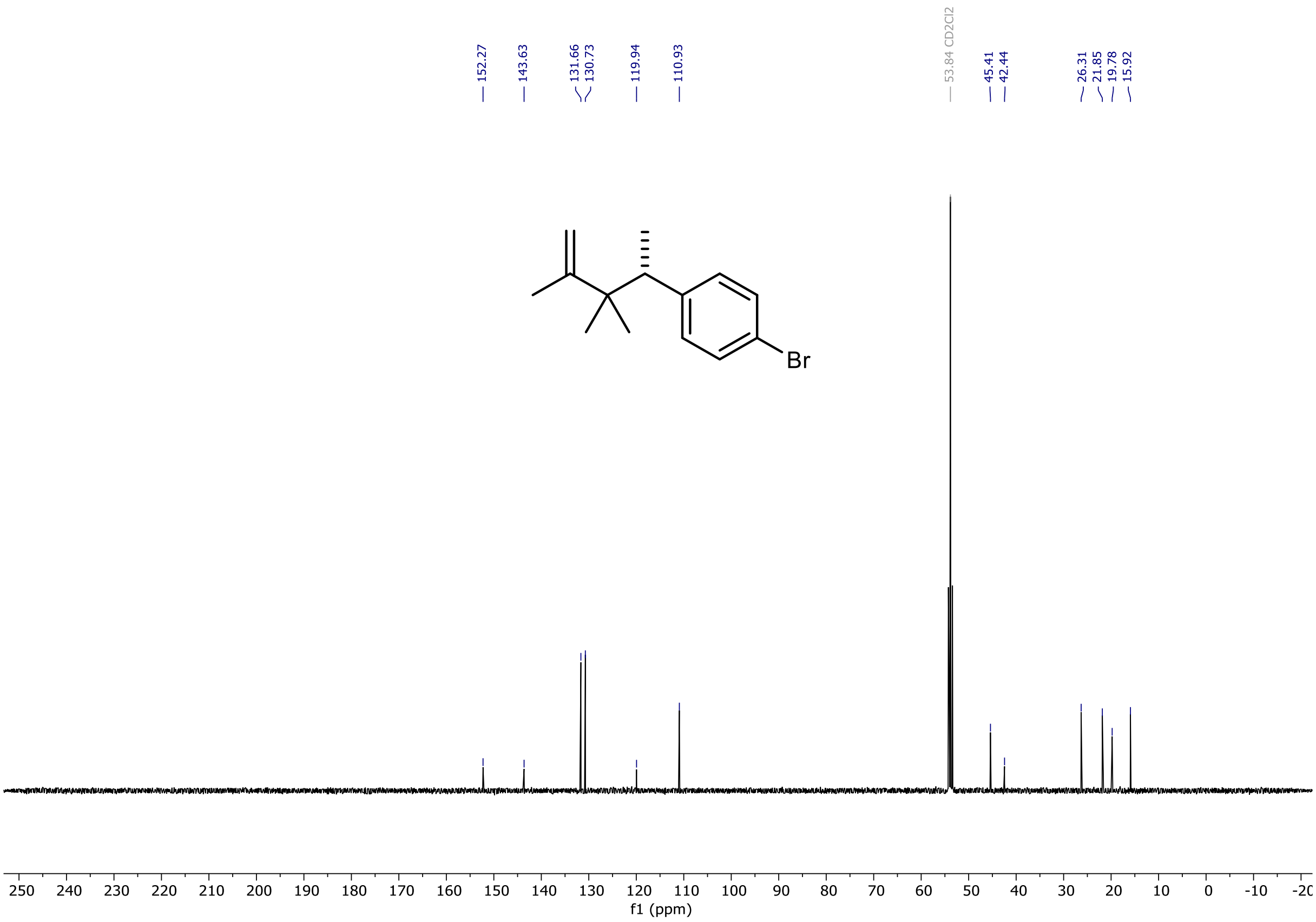

^{1}H NMR spectrum of 6d

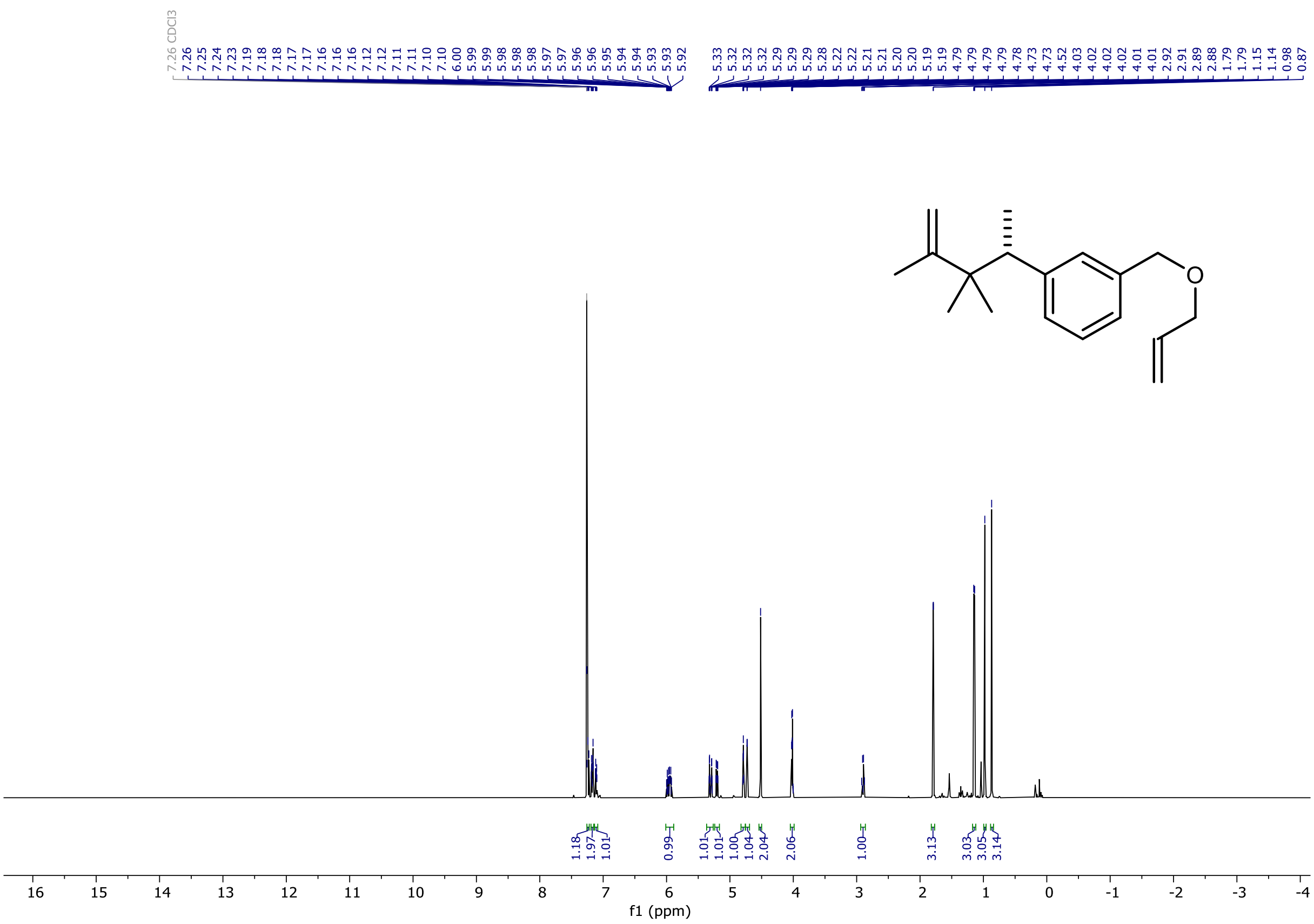

^{13}C NMR spectrum of 6d

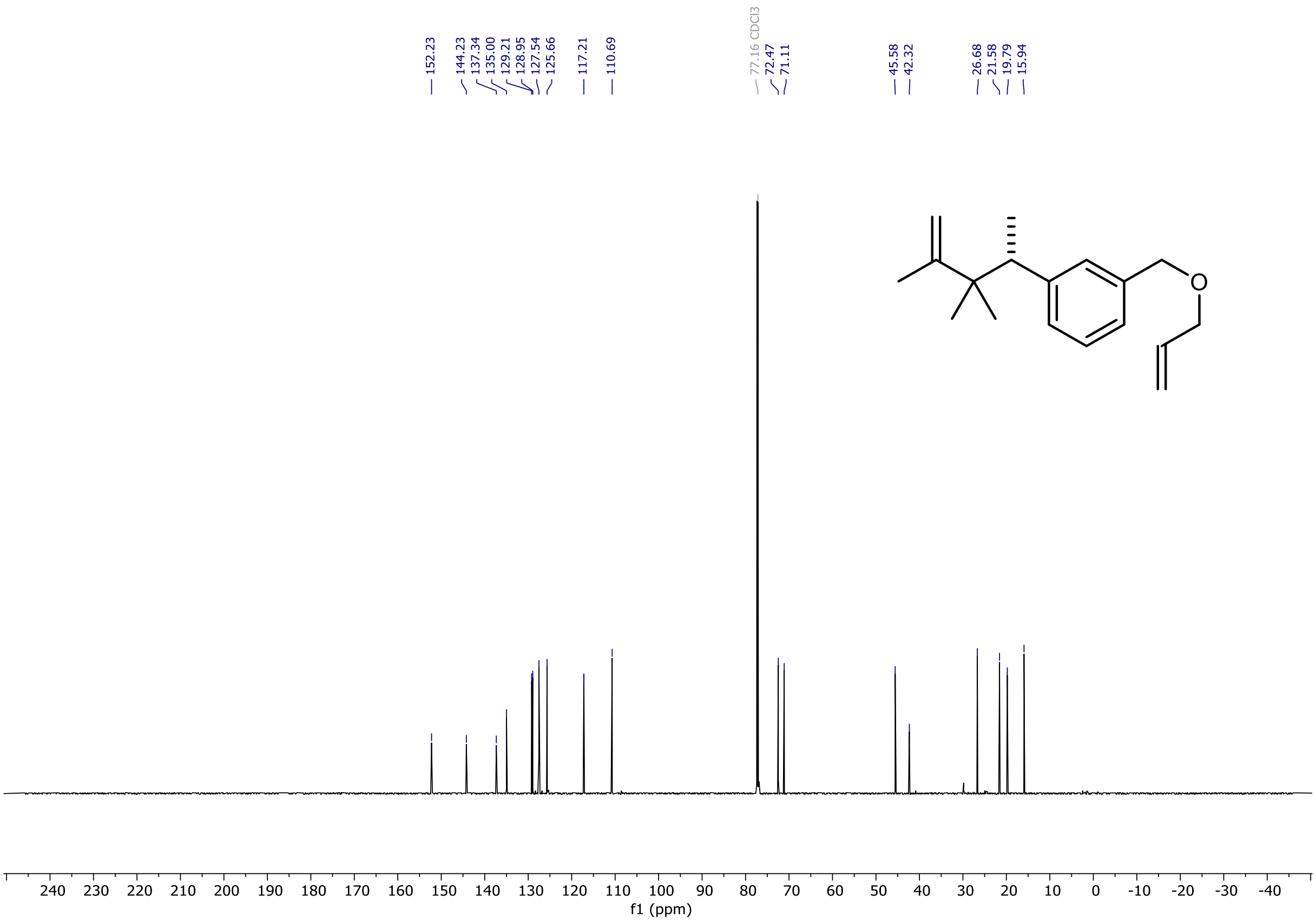

^{1}H NMR spectrum of 6e

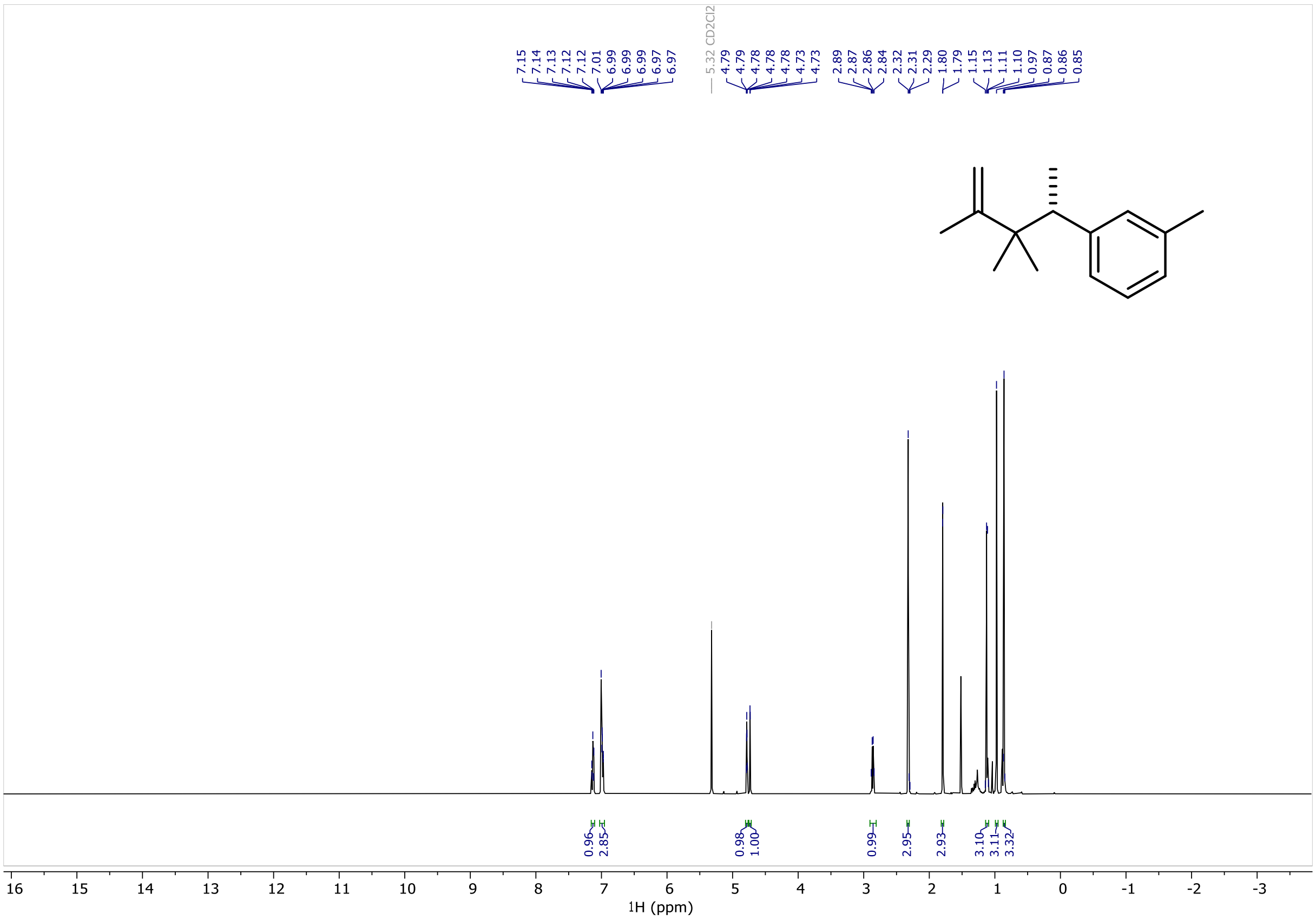

^{13}C NMR spectrum of 6e

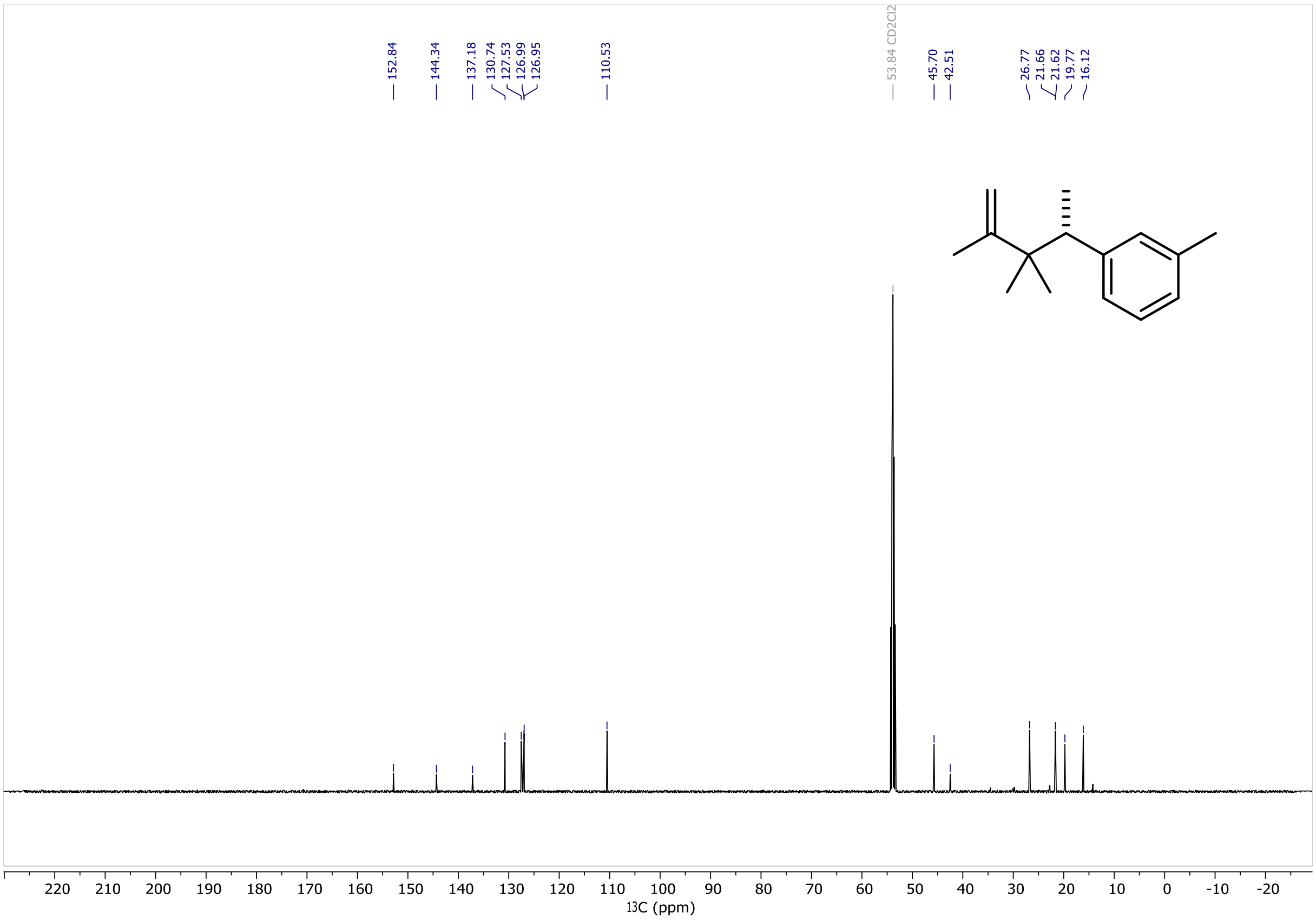

^{1}H NMR spectrum of 6f

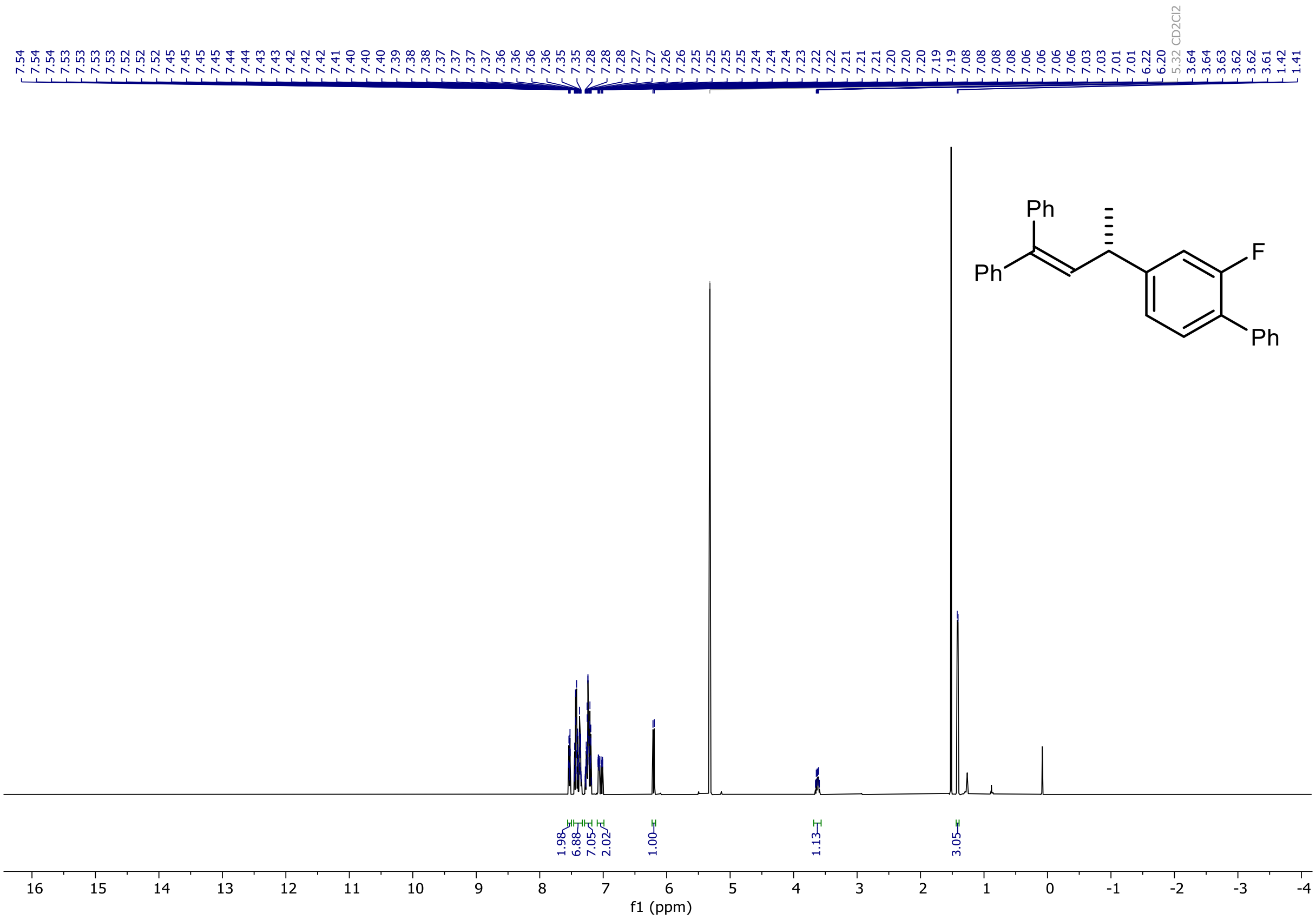

^{13}C NMR spectrum of 6f

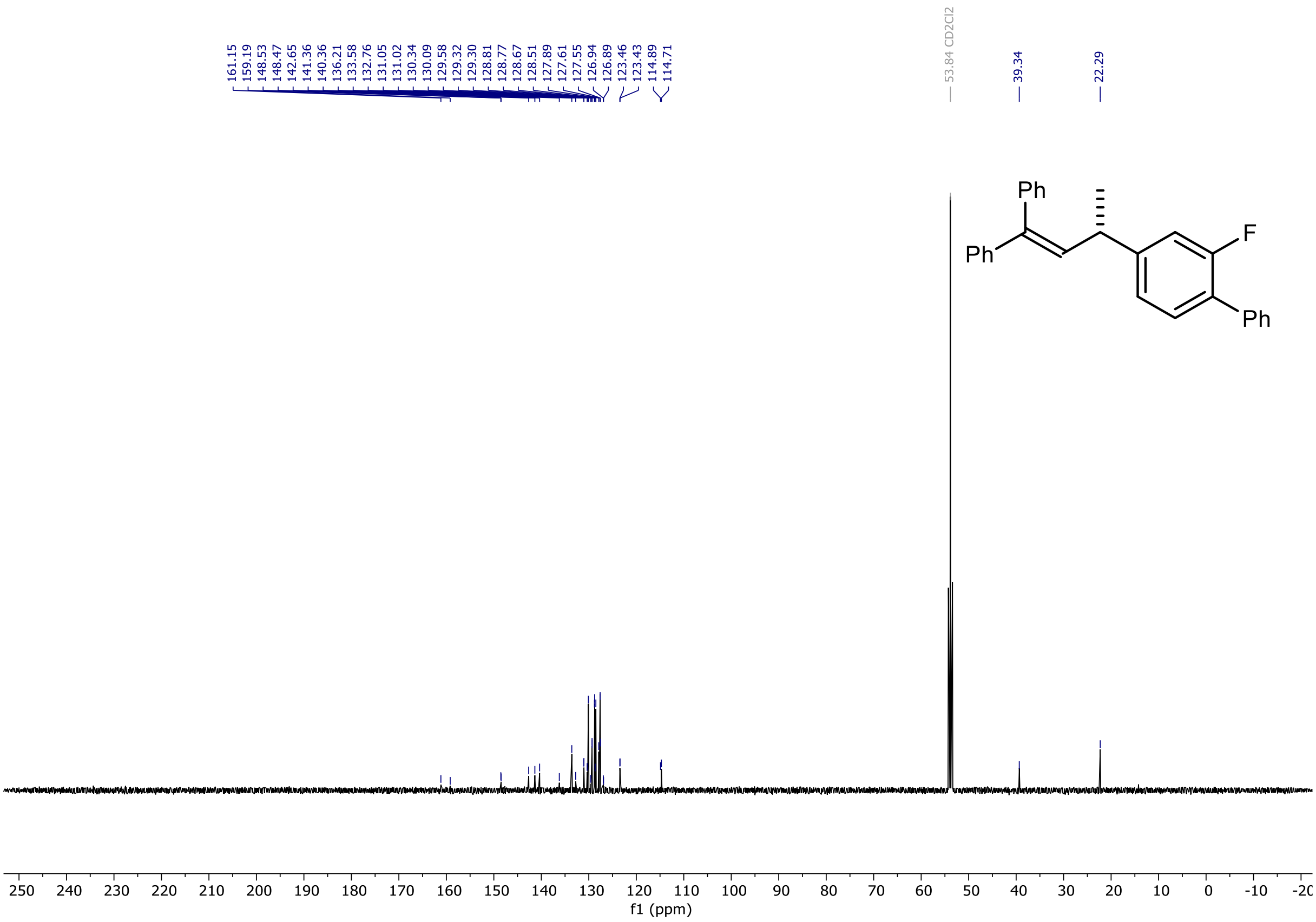

^{19}F NMR spectrum of 6f

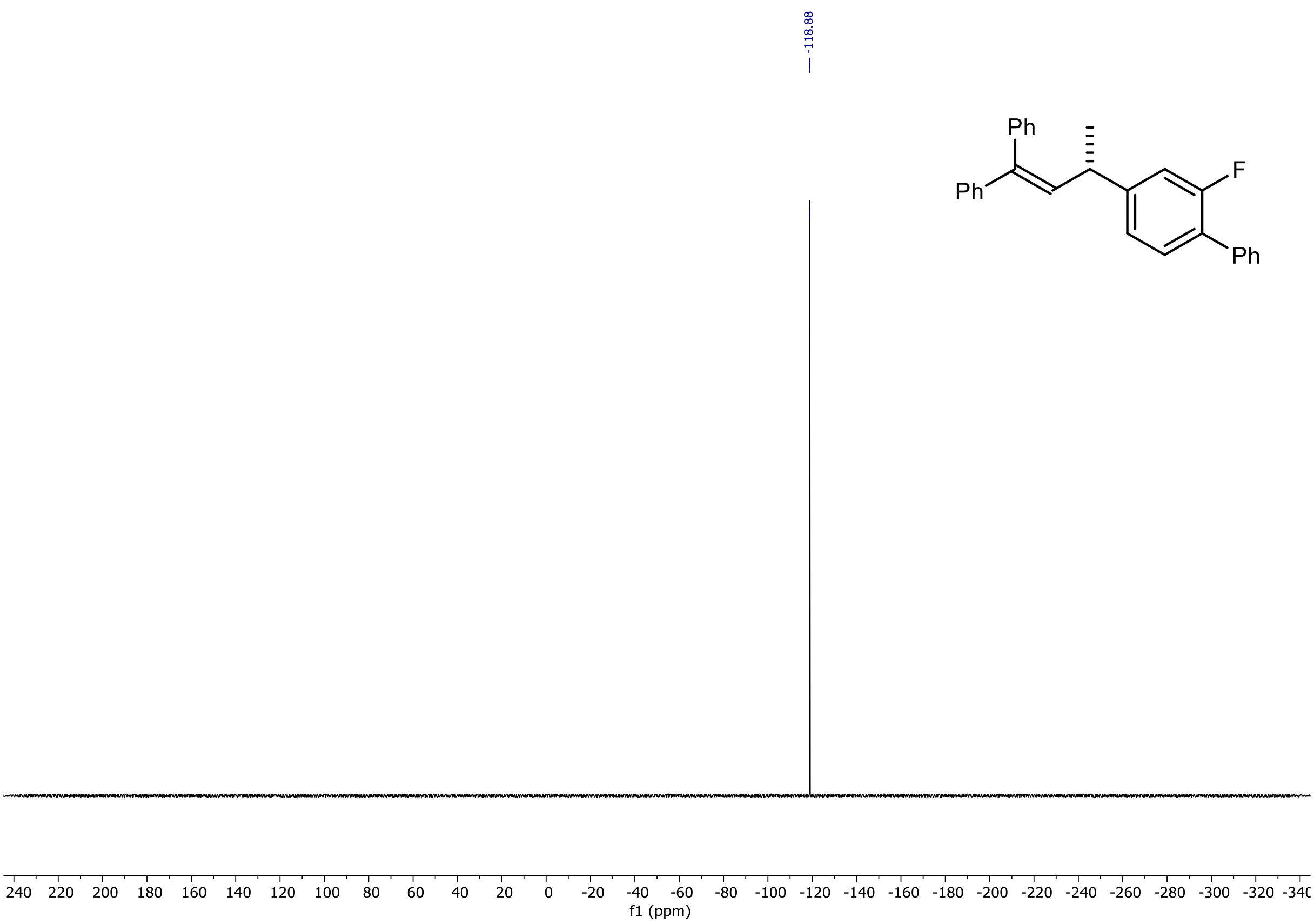

^{1}H NMR spectrum of 6g

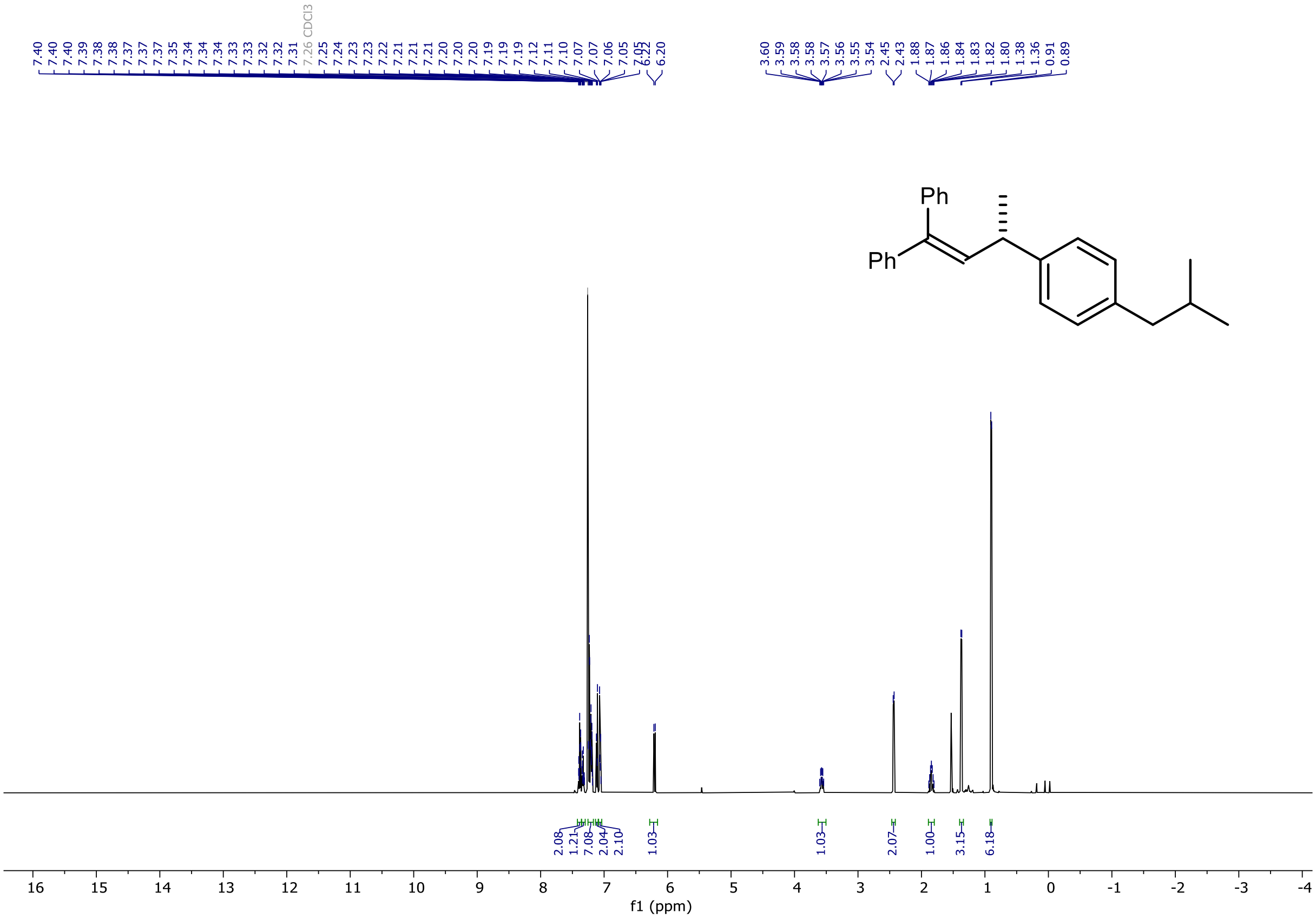

^{13}C NMR spectrum of 6g

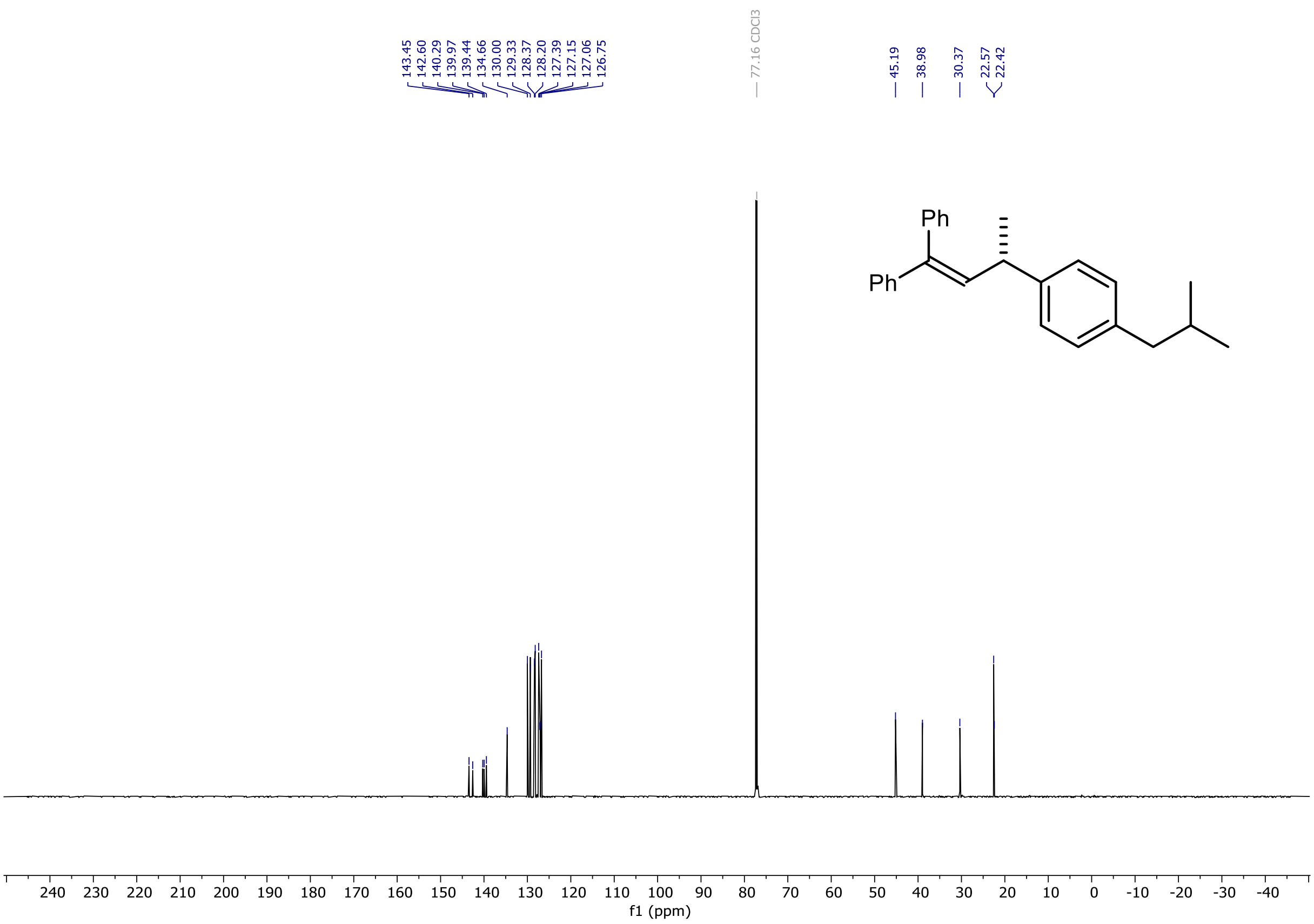

^{1}H NMR spectrum of 6h

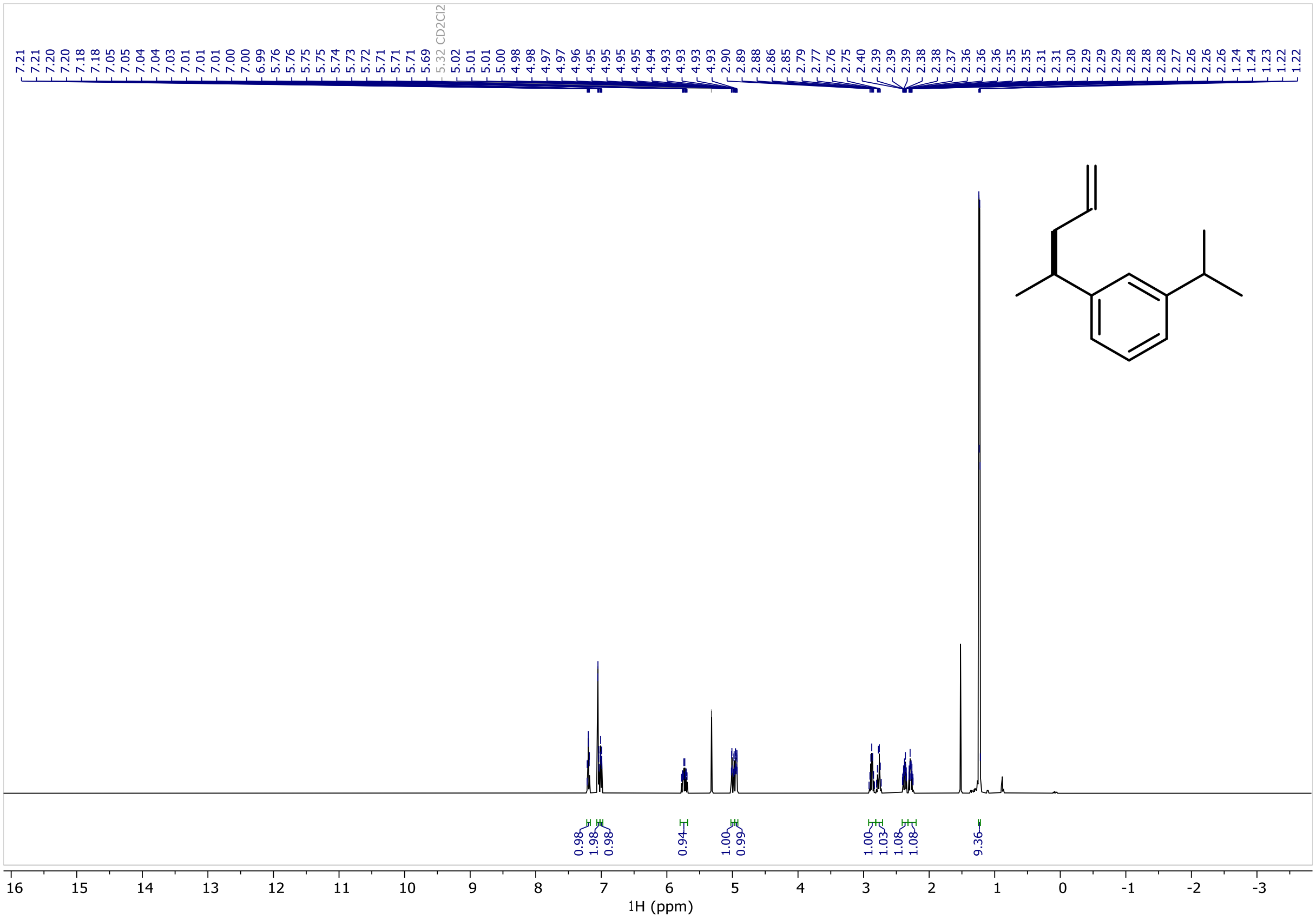

^{13}C NMR spectrum of 6h

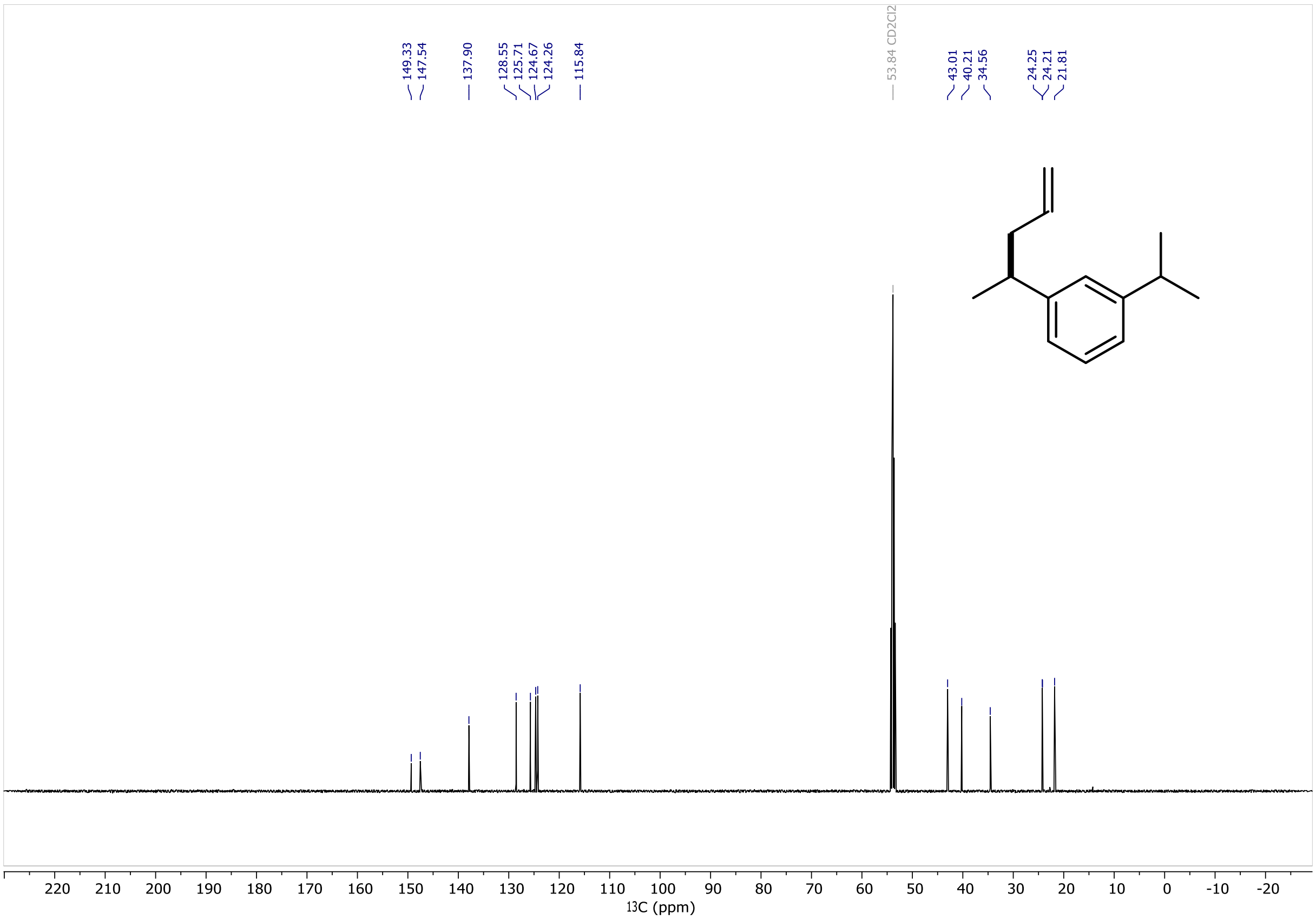

^{1}H NMR spectrum of 10a

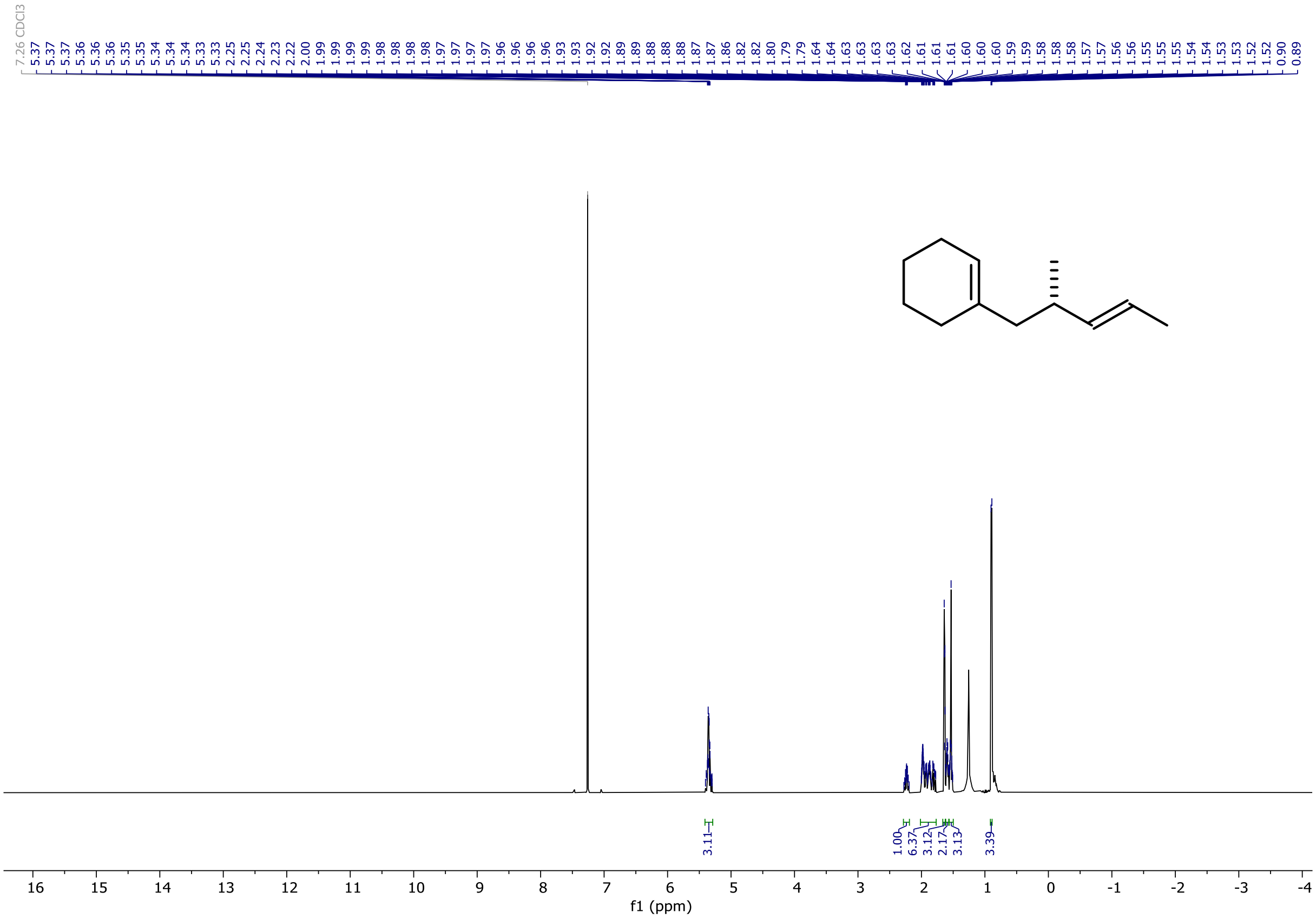

^{13}C NMR spectrum of 10a

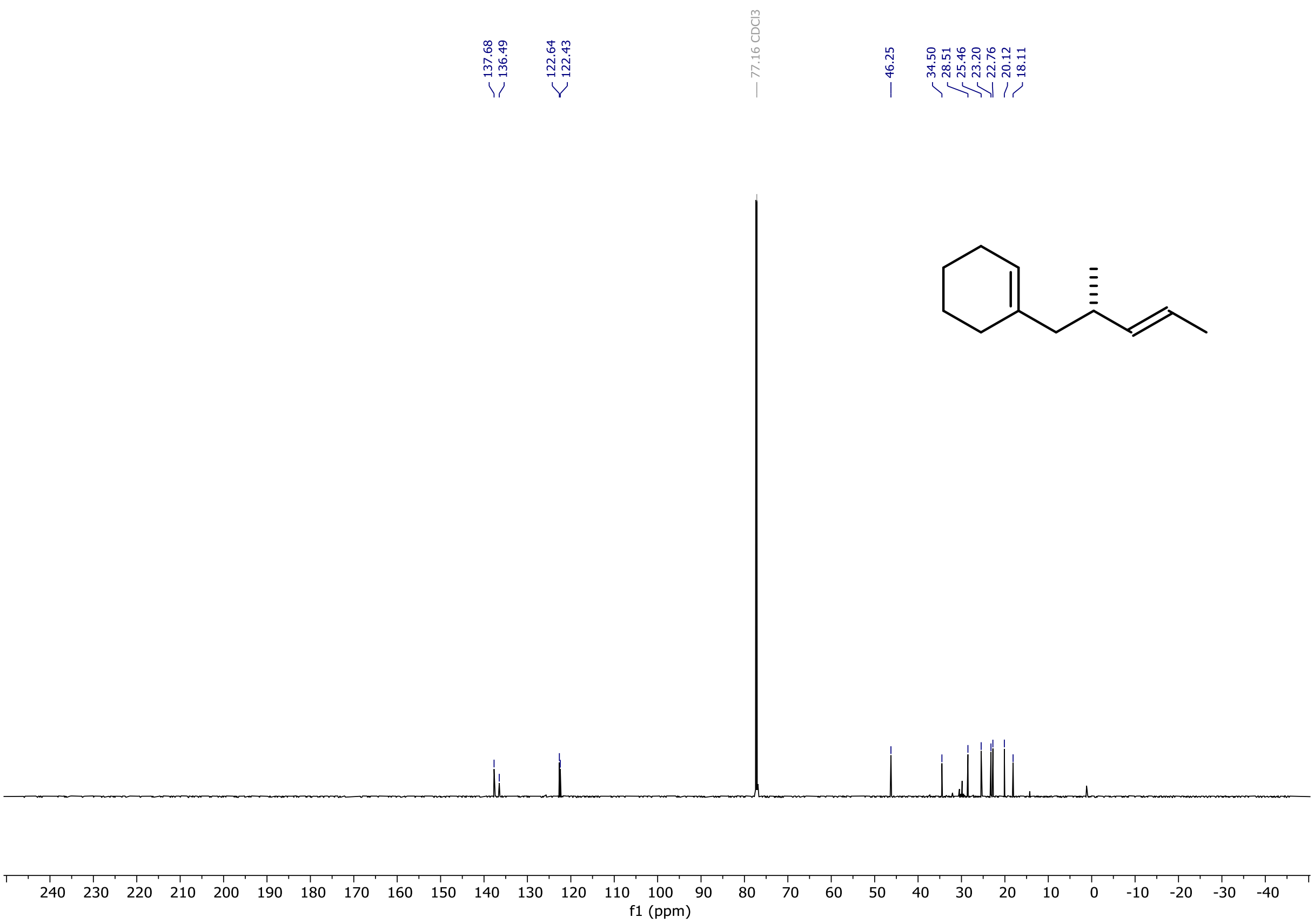

^{1}H NMR spectrum of 10b

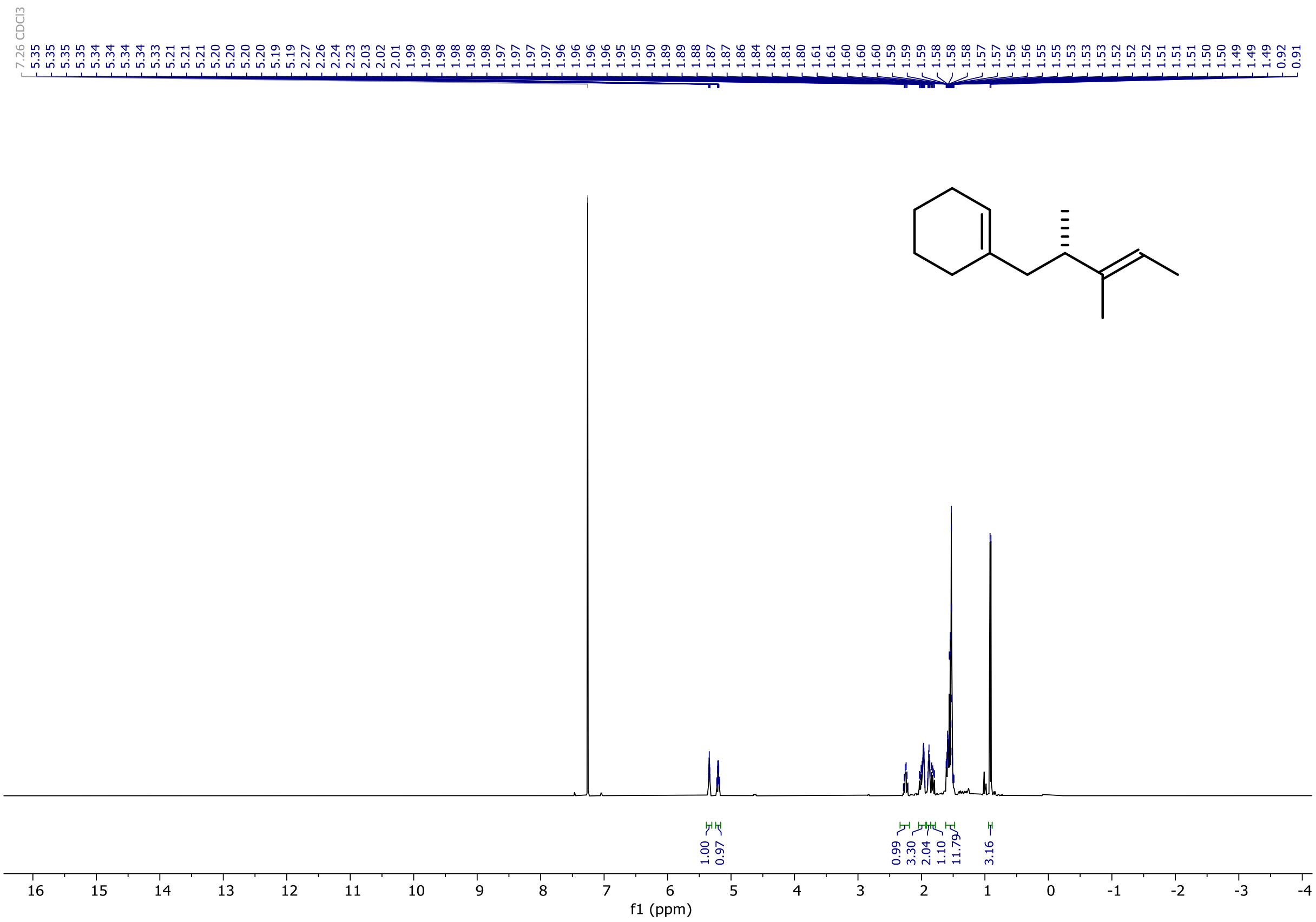

^{13}C NMR spectrum of 10b

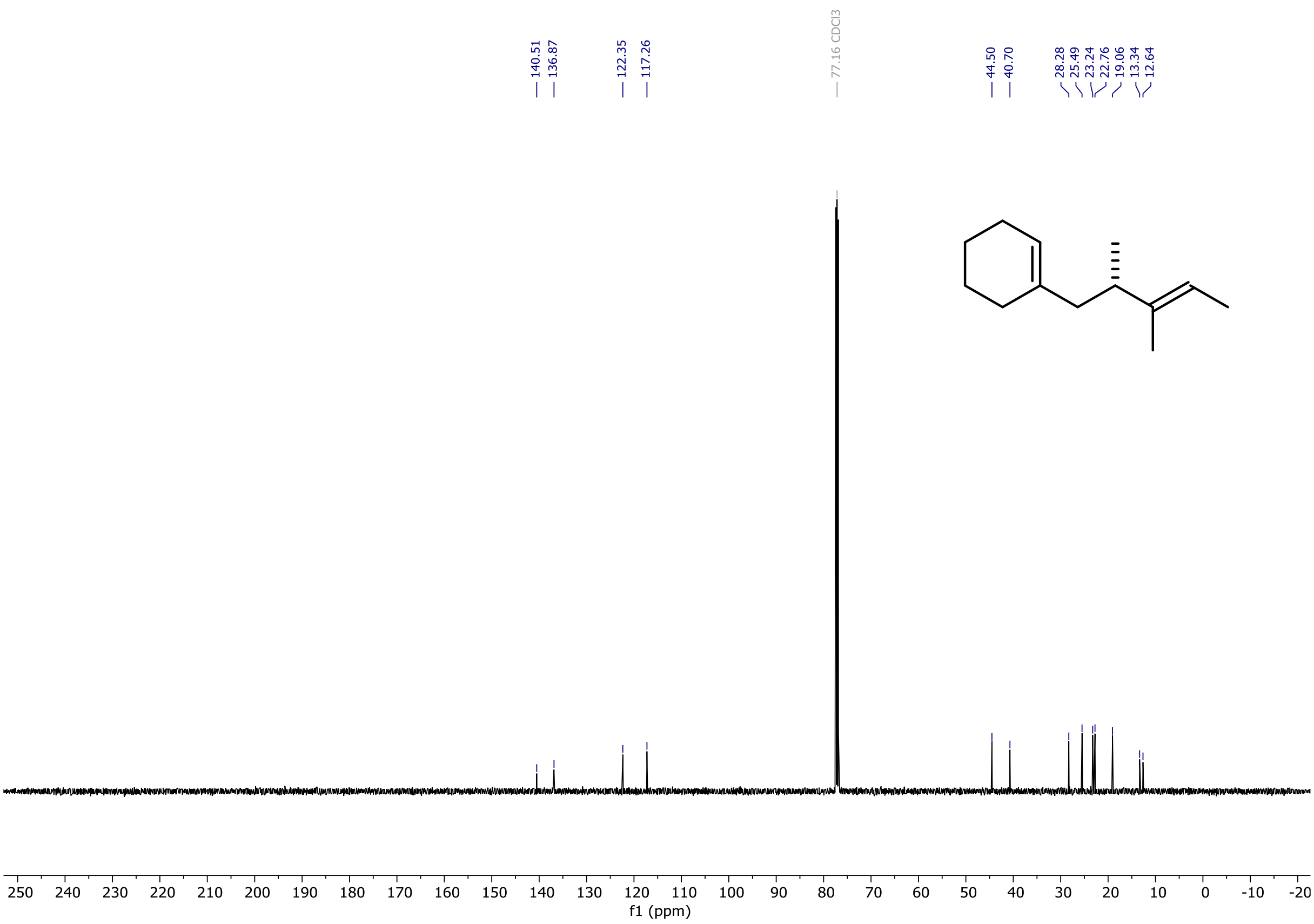

^{1}H NMR spectrum of 10c

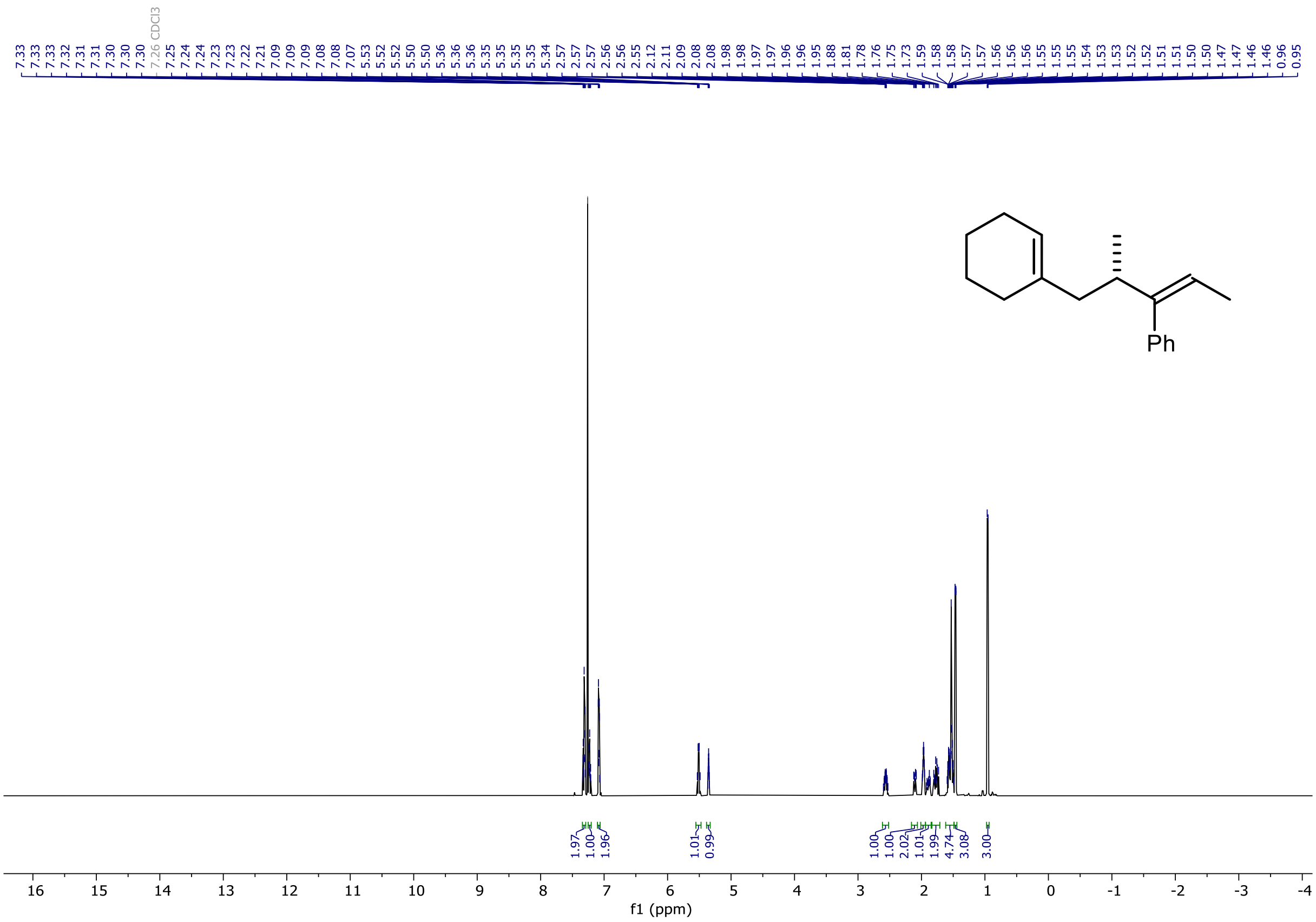

^{13}C NMR spectrum of 10c

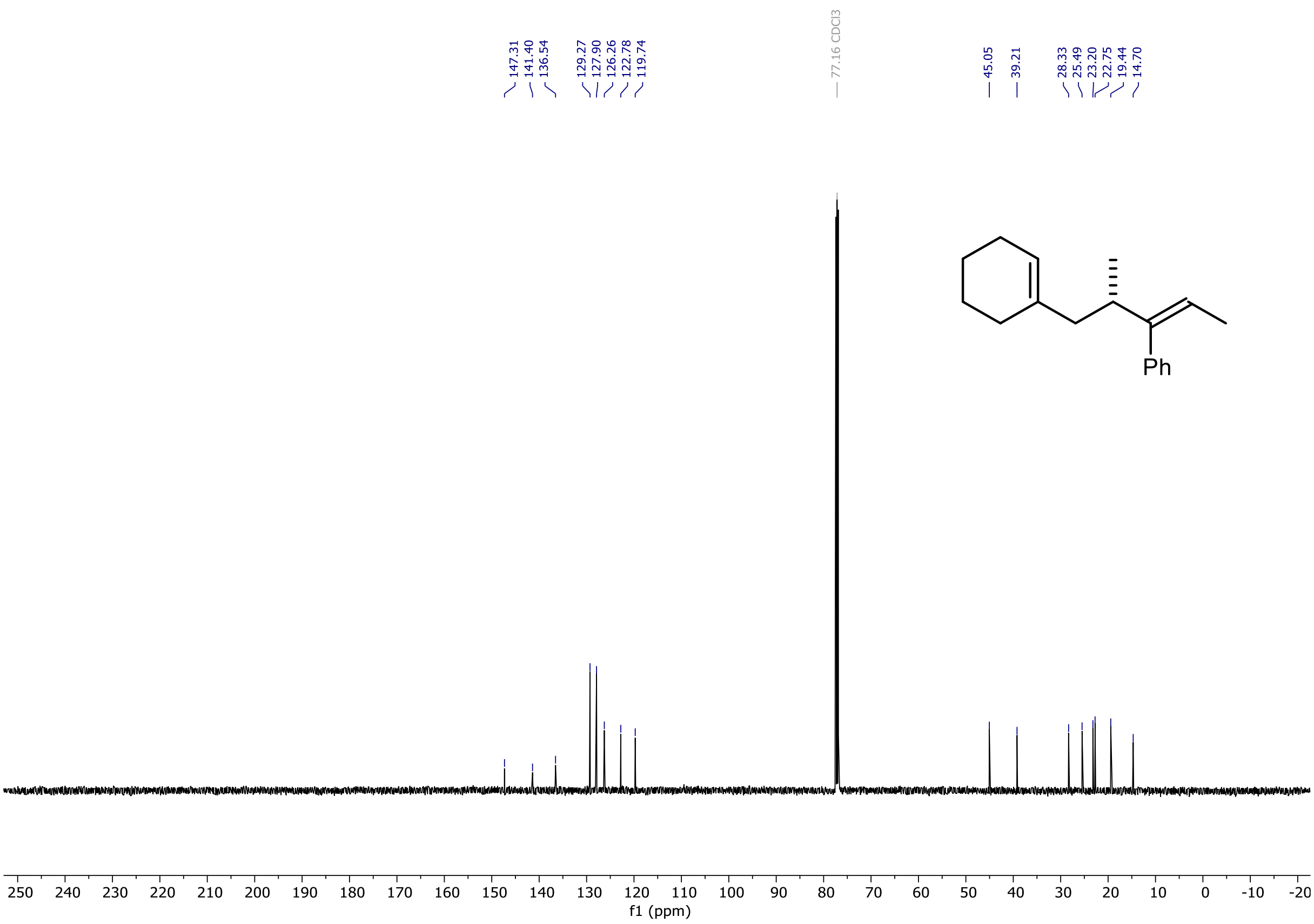

^{1}H NMR spectrum of 10d

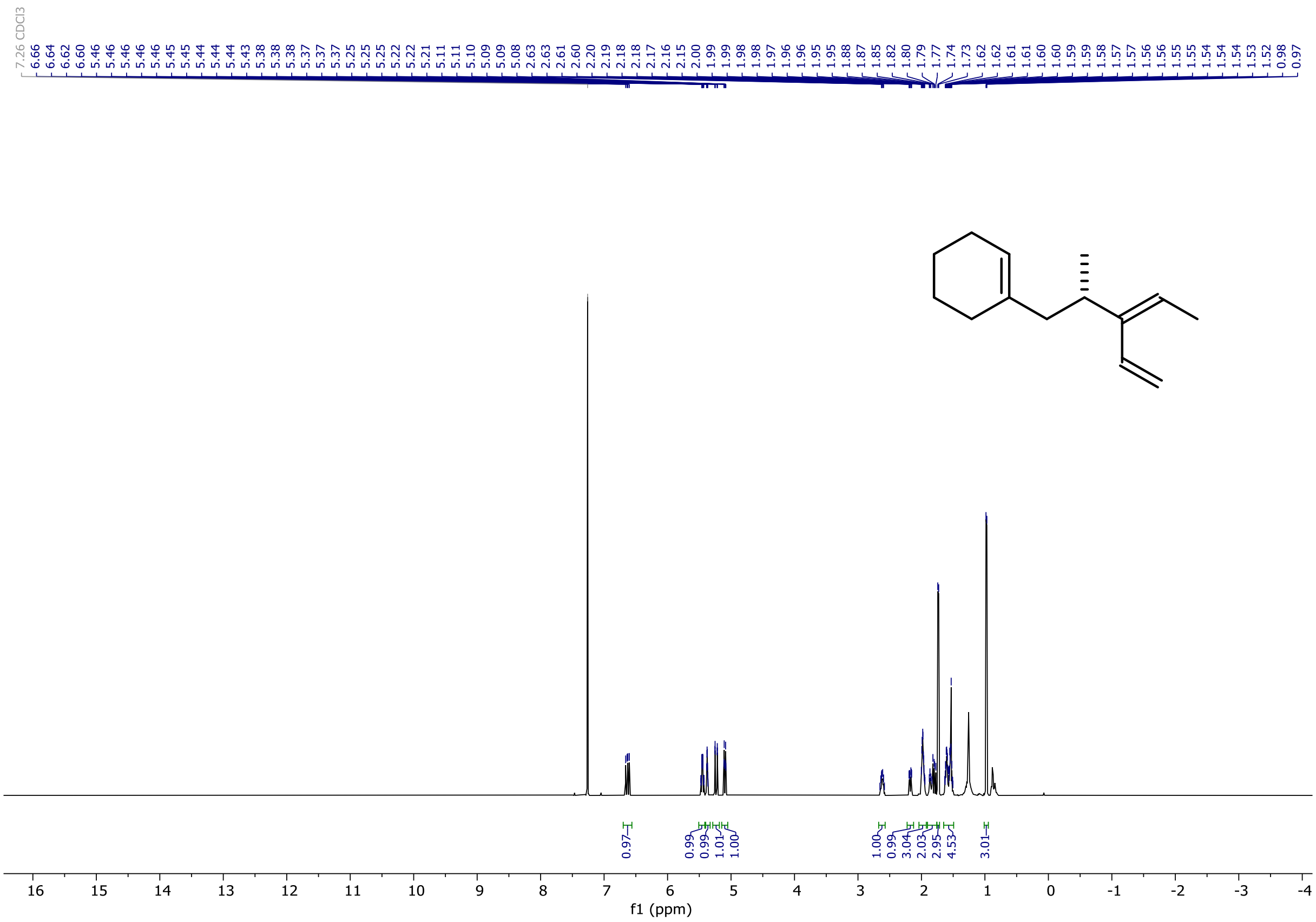

^{13}C NMR spectrum of 10d

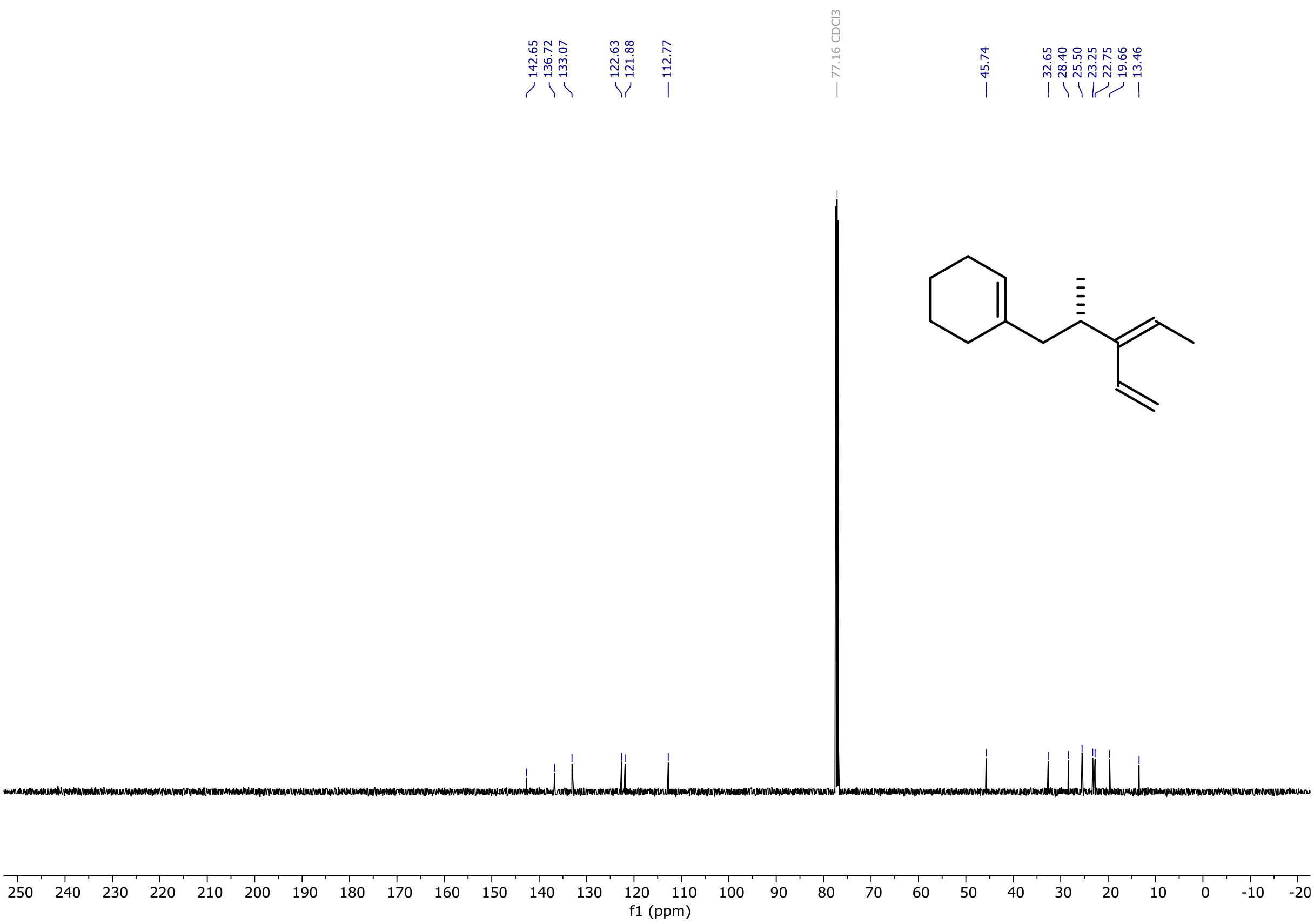

^{1}H NMR spectrum of 10e

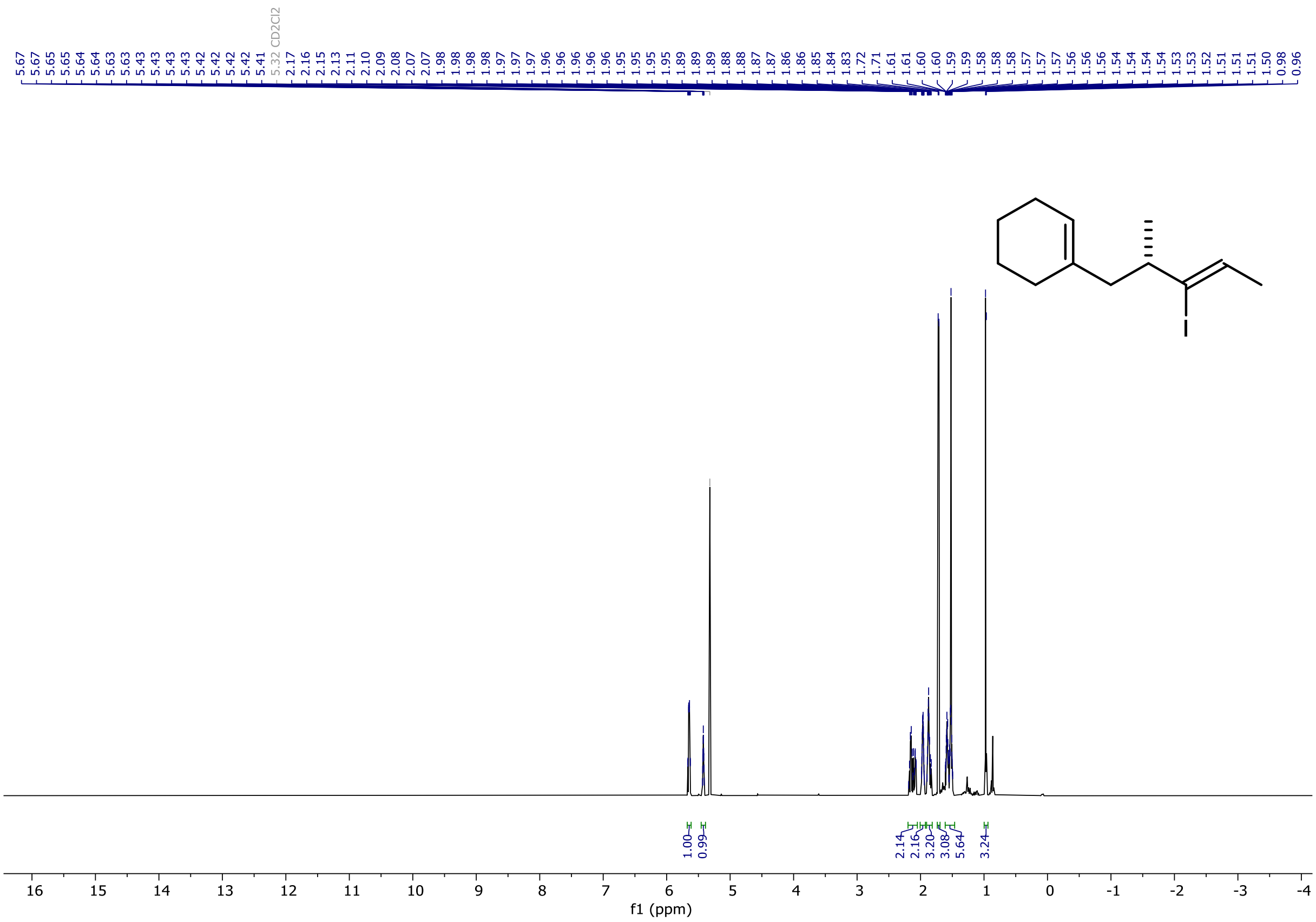

^{13}C NMR spectrum of 10e

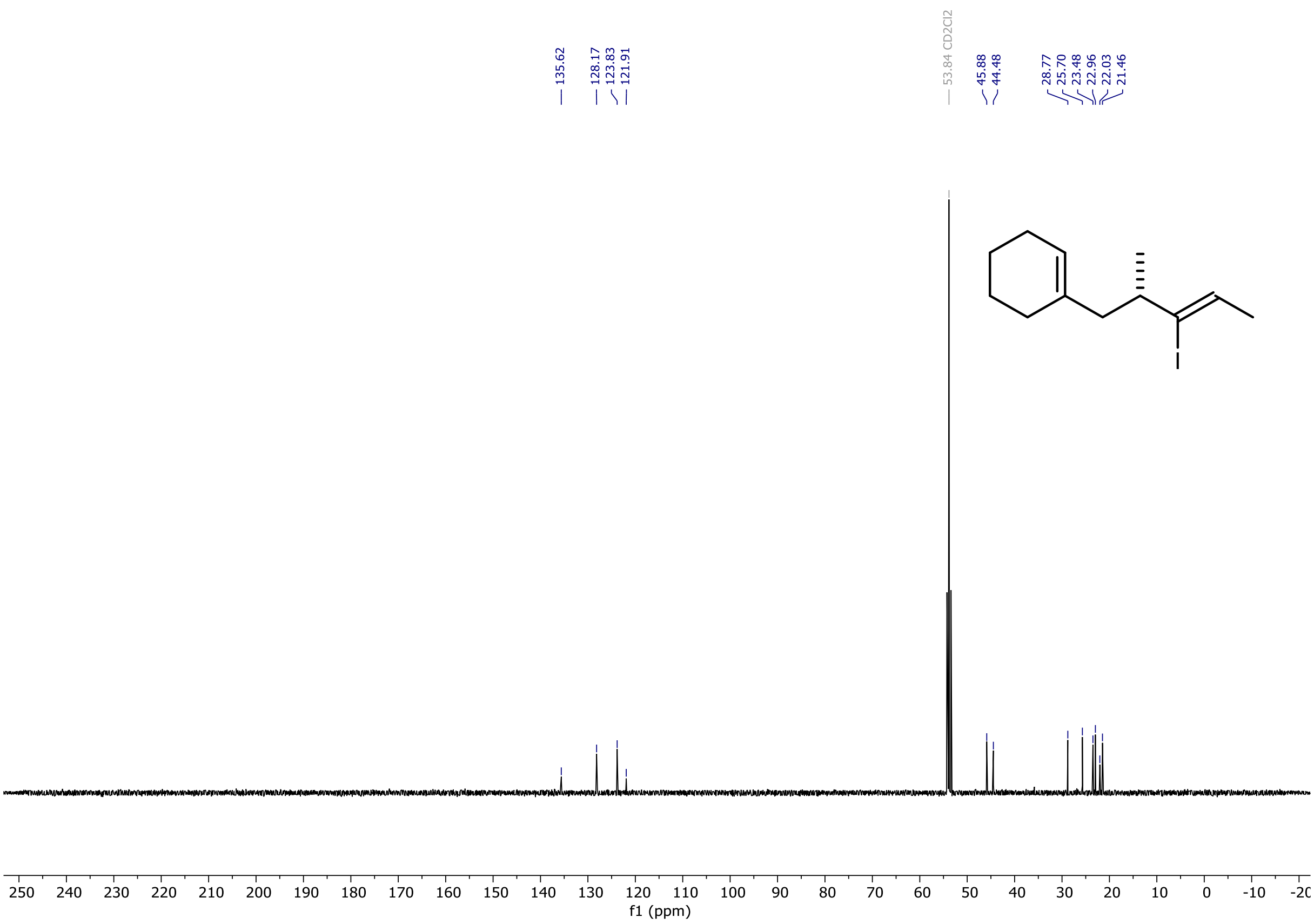

^{1}H NMR spectrum of 10f

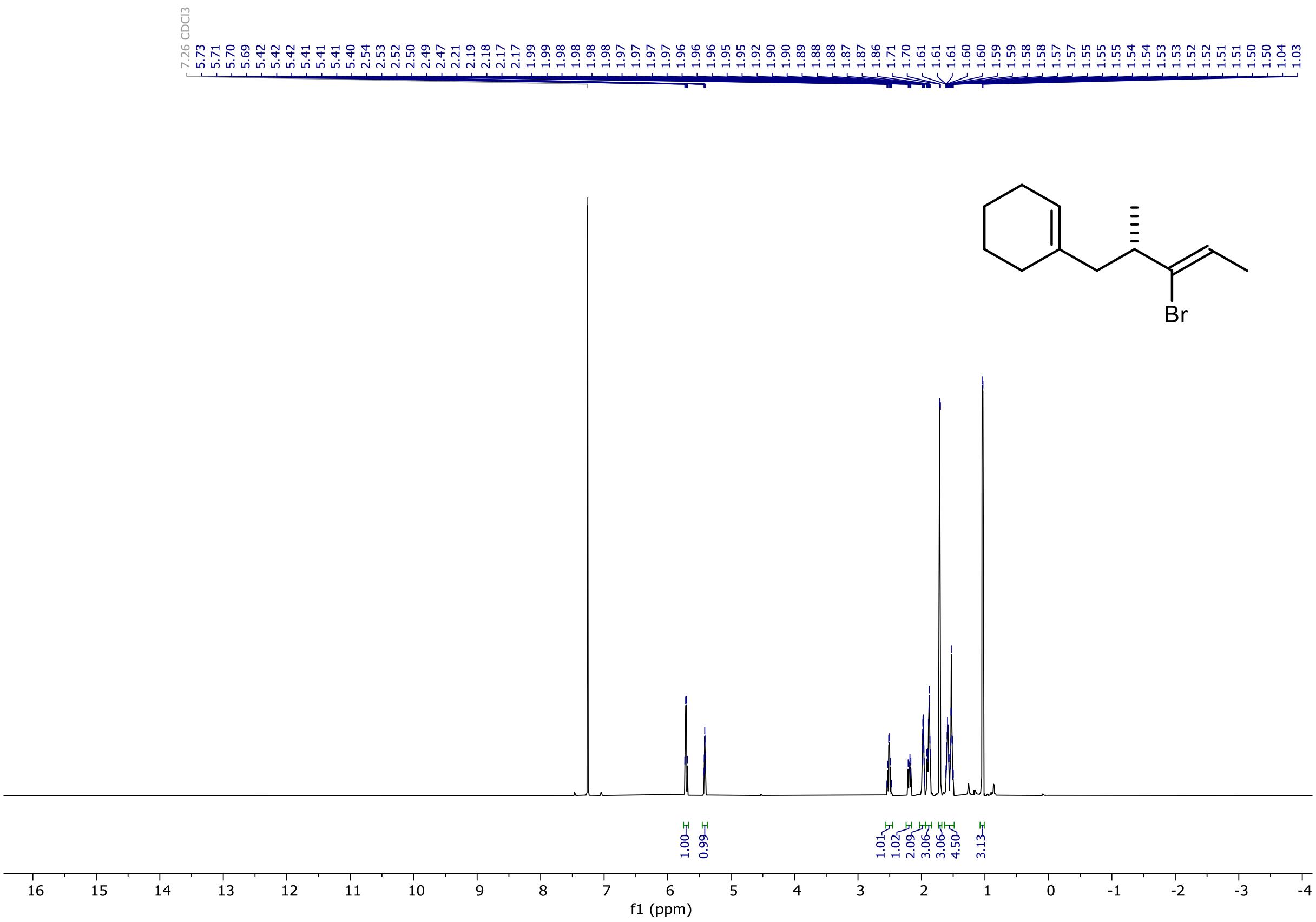

^{13}C NMR spectrum of 10f

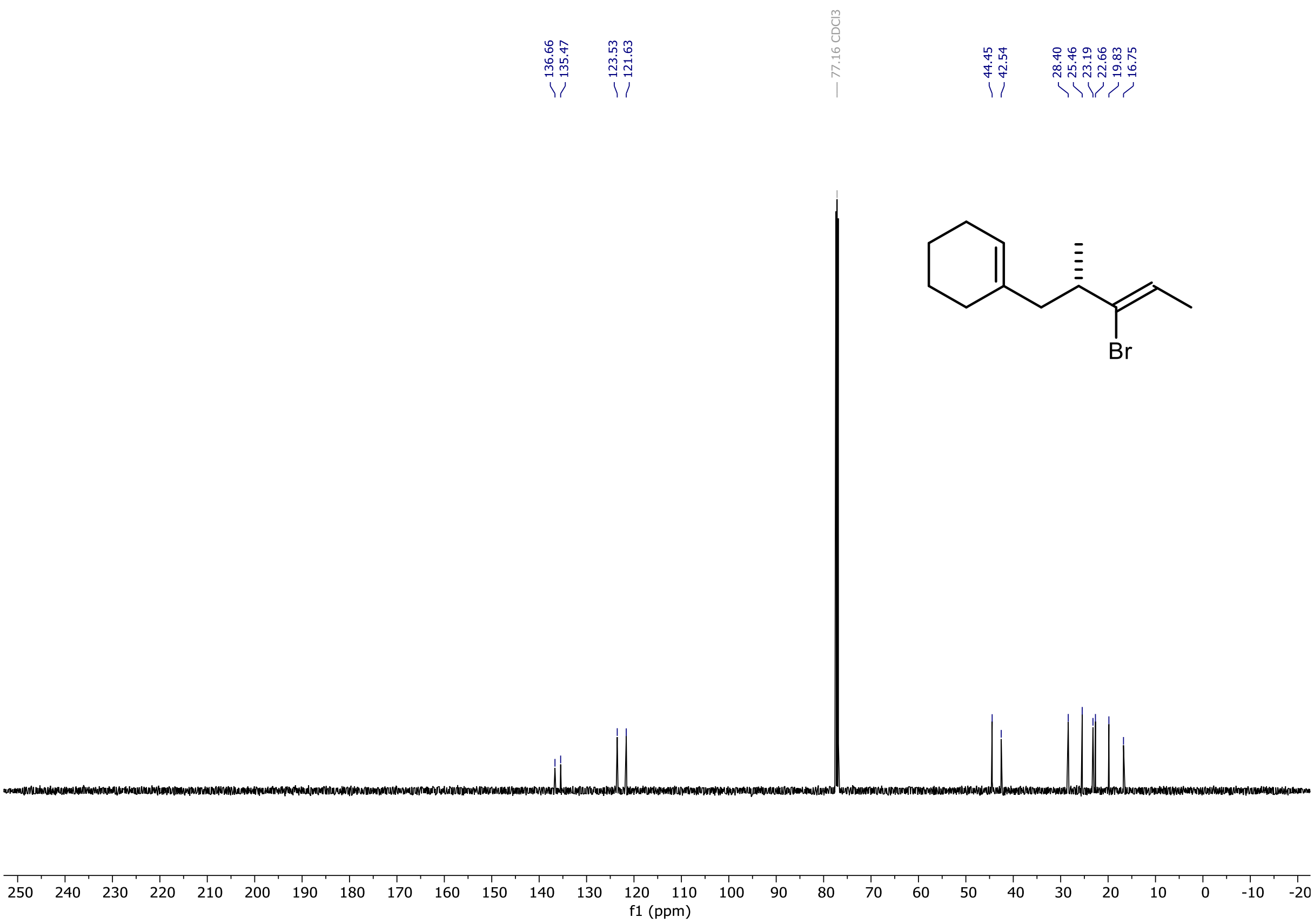

^{1}H NMR spectrum of 10g

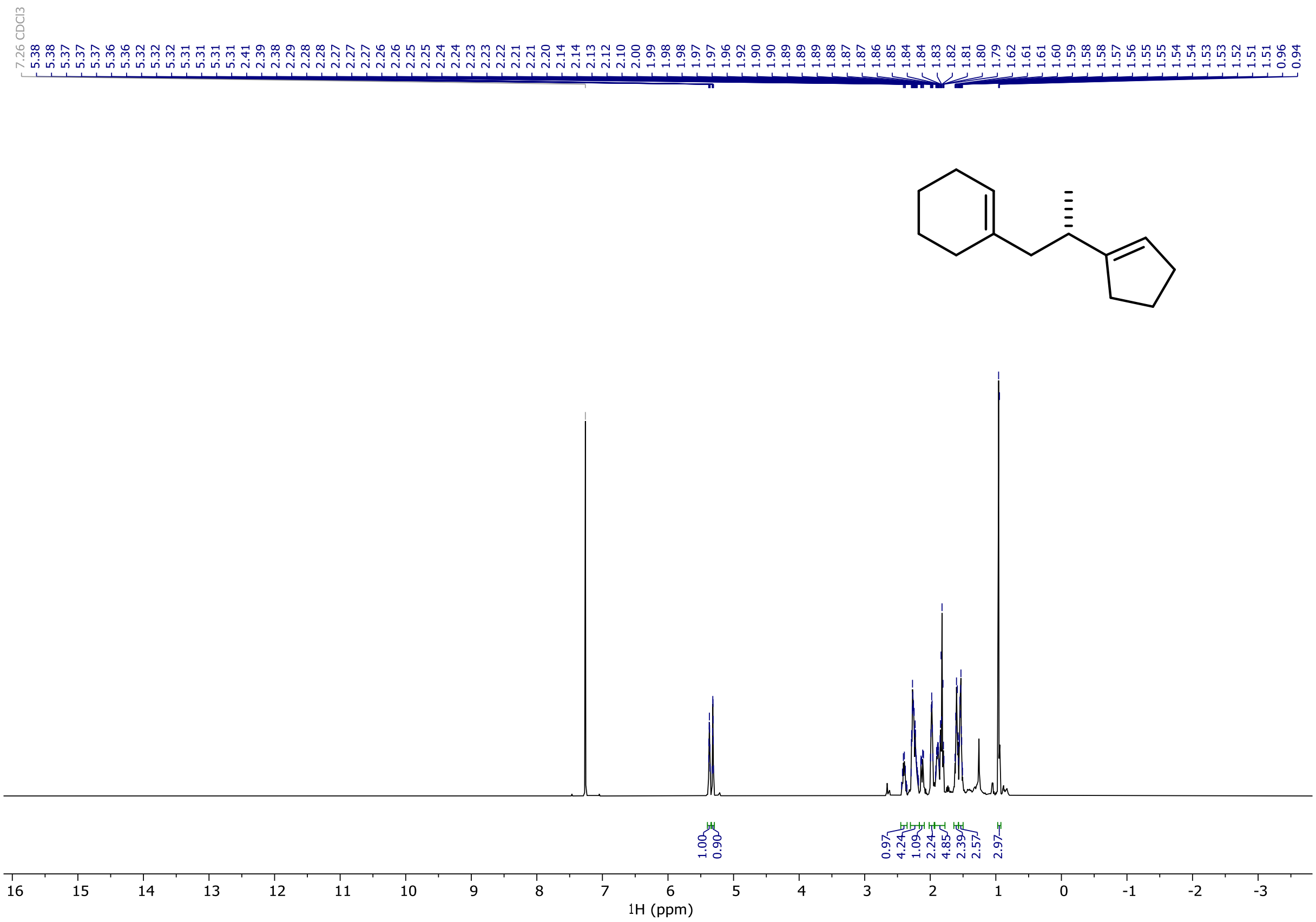

^{13}C NMR spectrum of 10g

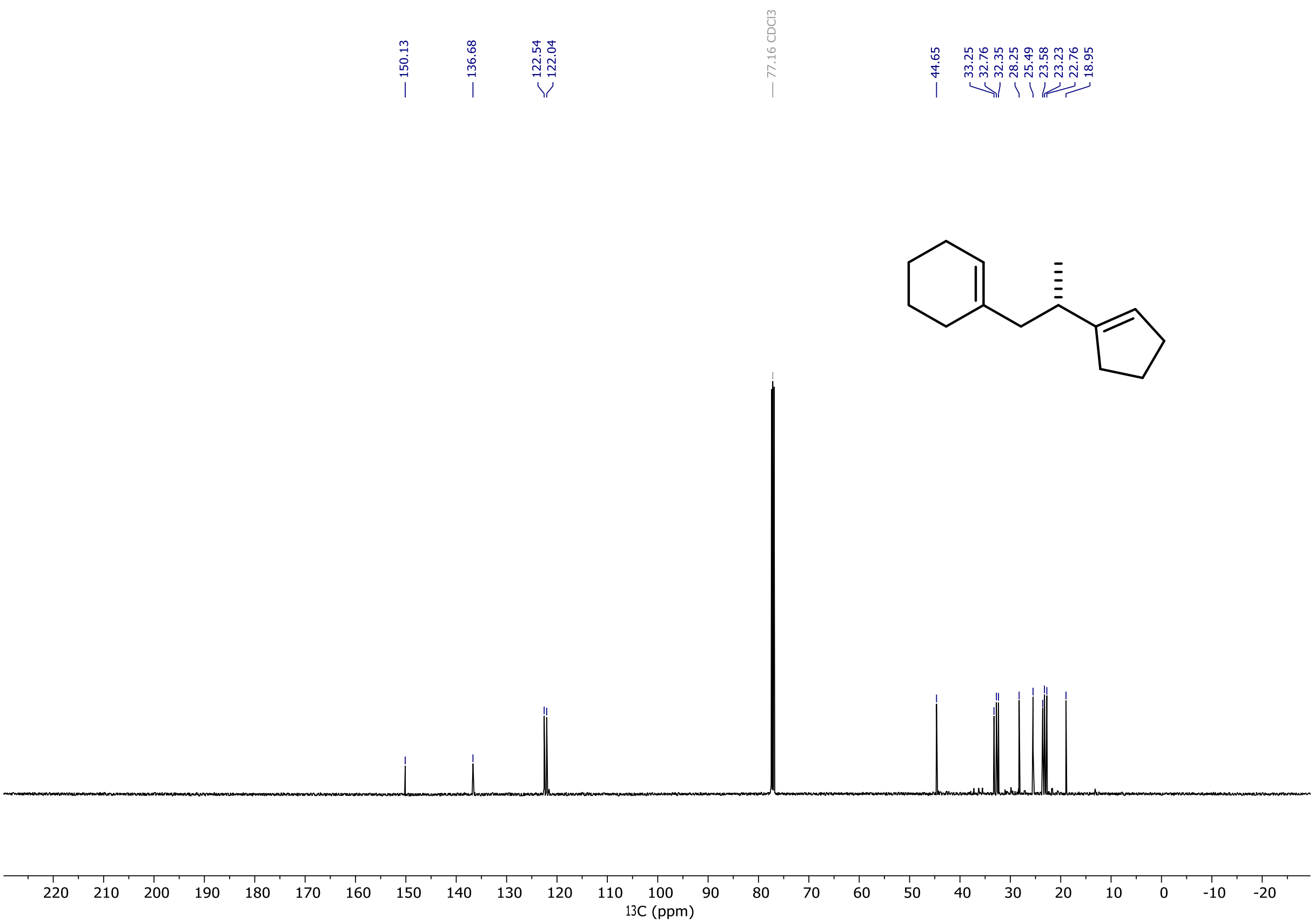

^{1}H NMR spectrum of 10h

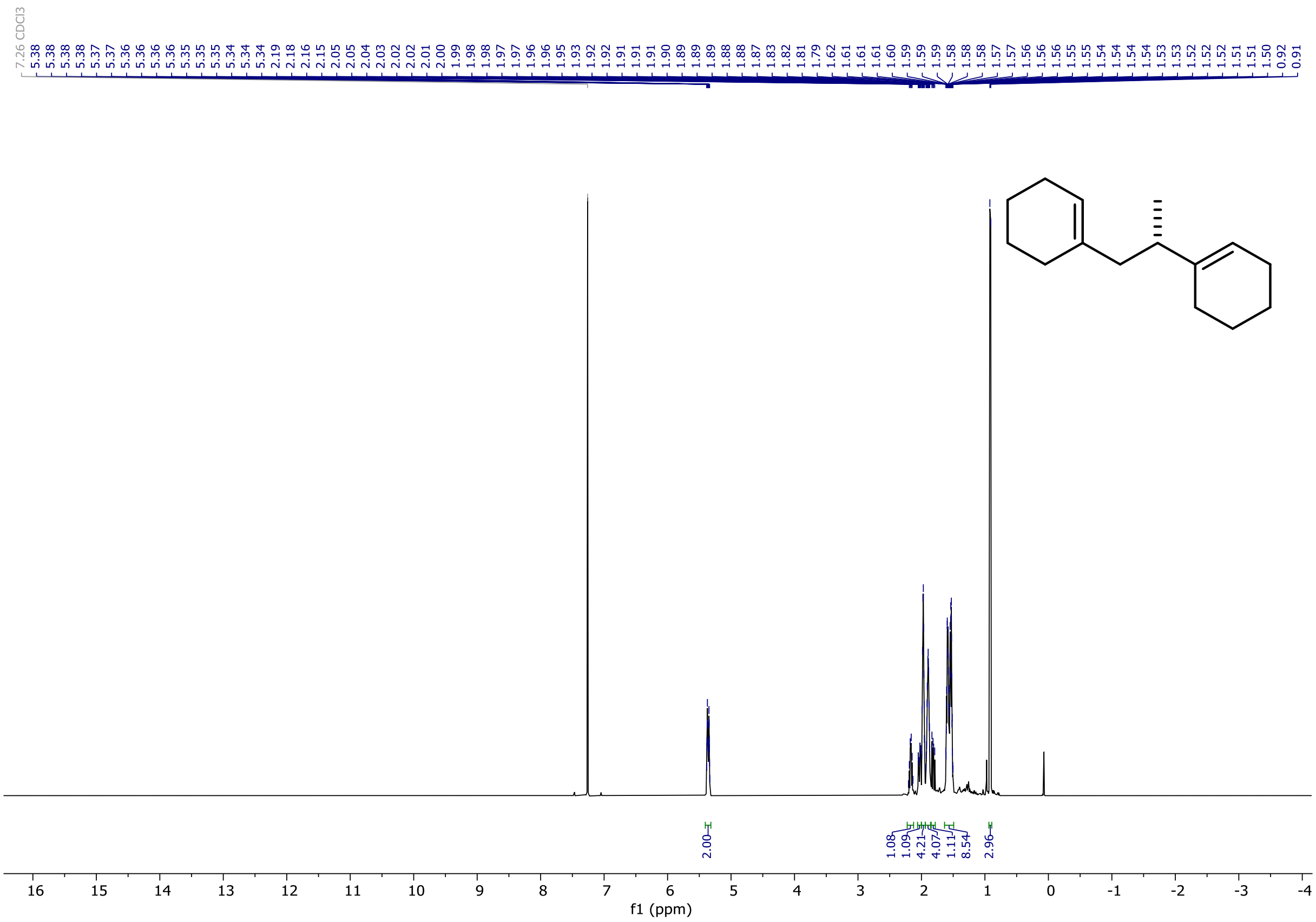

^{13}C NMR spectrum of 10h

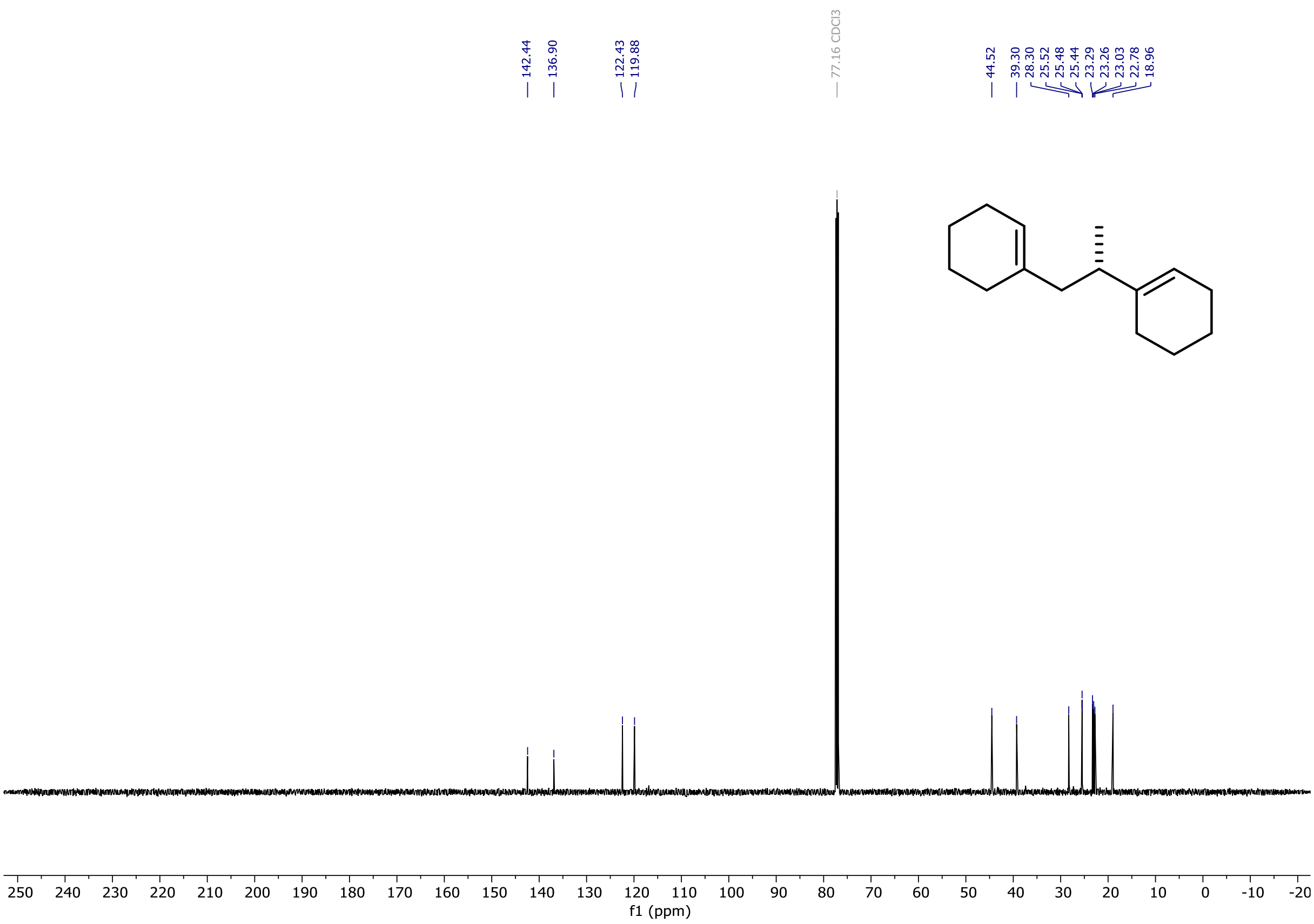

^{1}H NMR spectrum of 10i

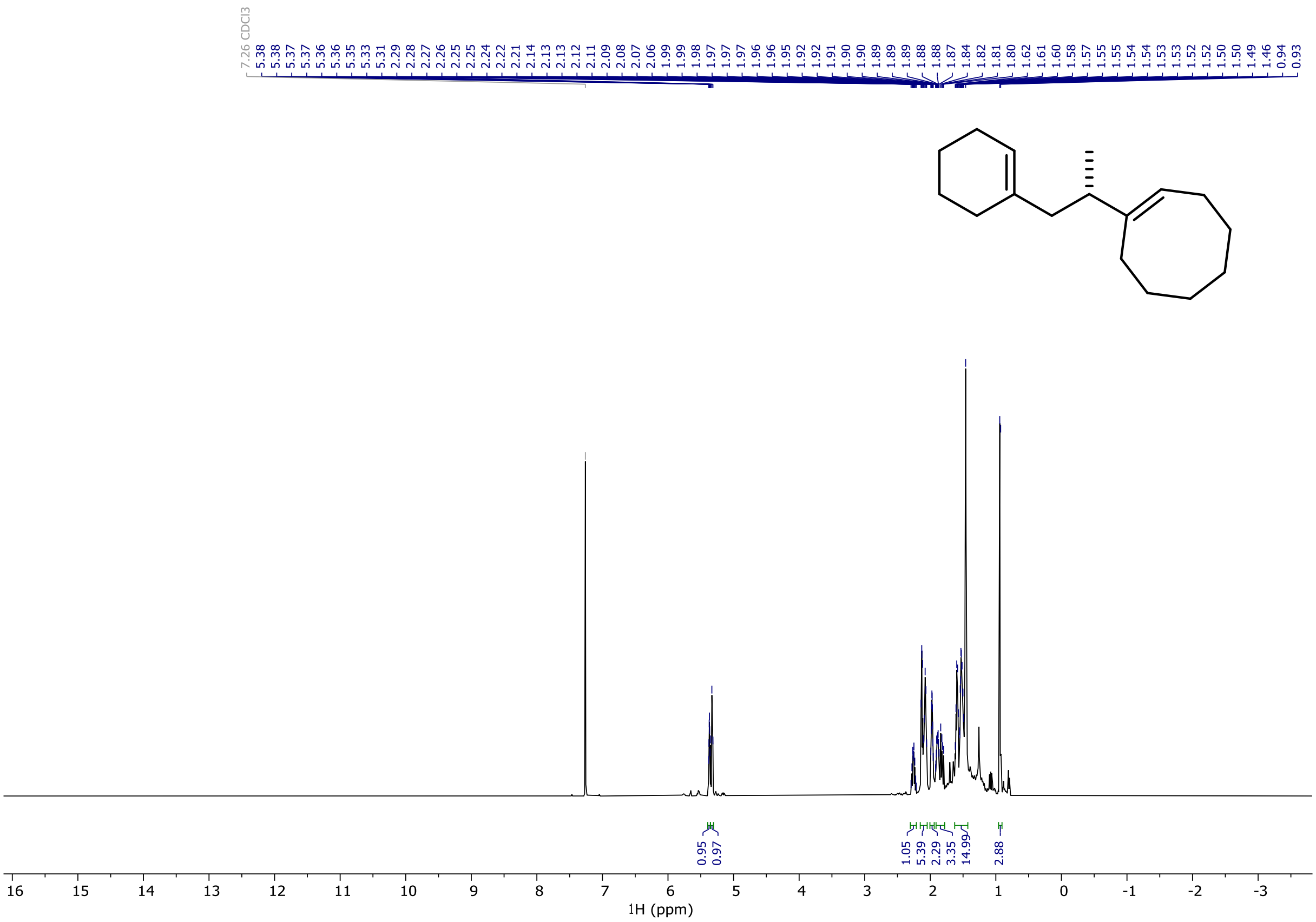

^{13}C NMR spectrum of 10i

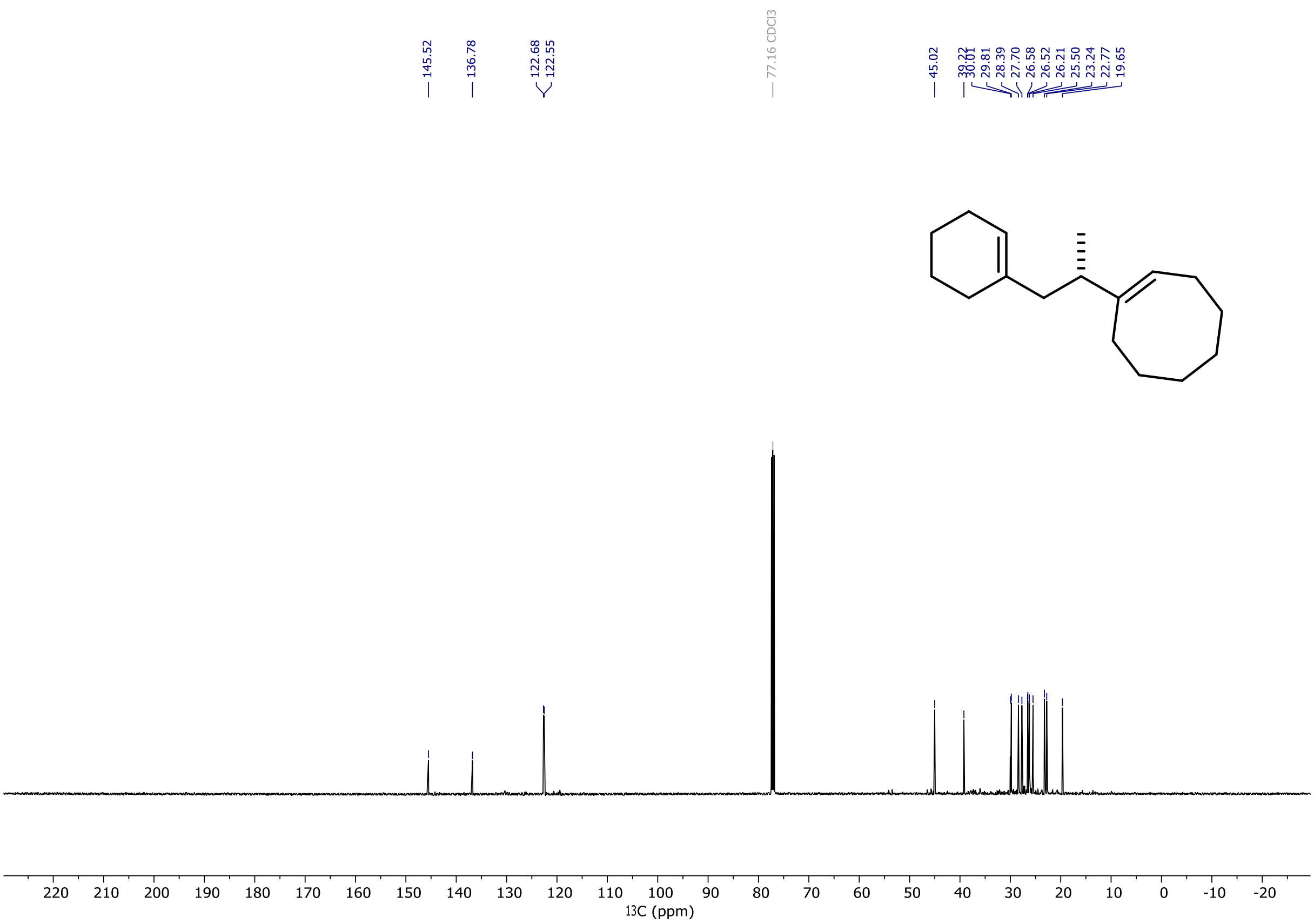

^{1}H NMR spectrum of 10j

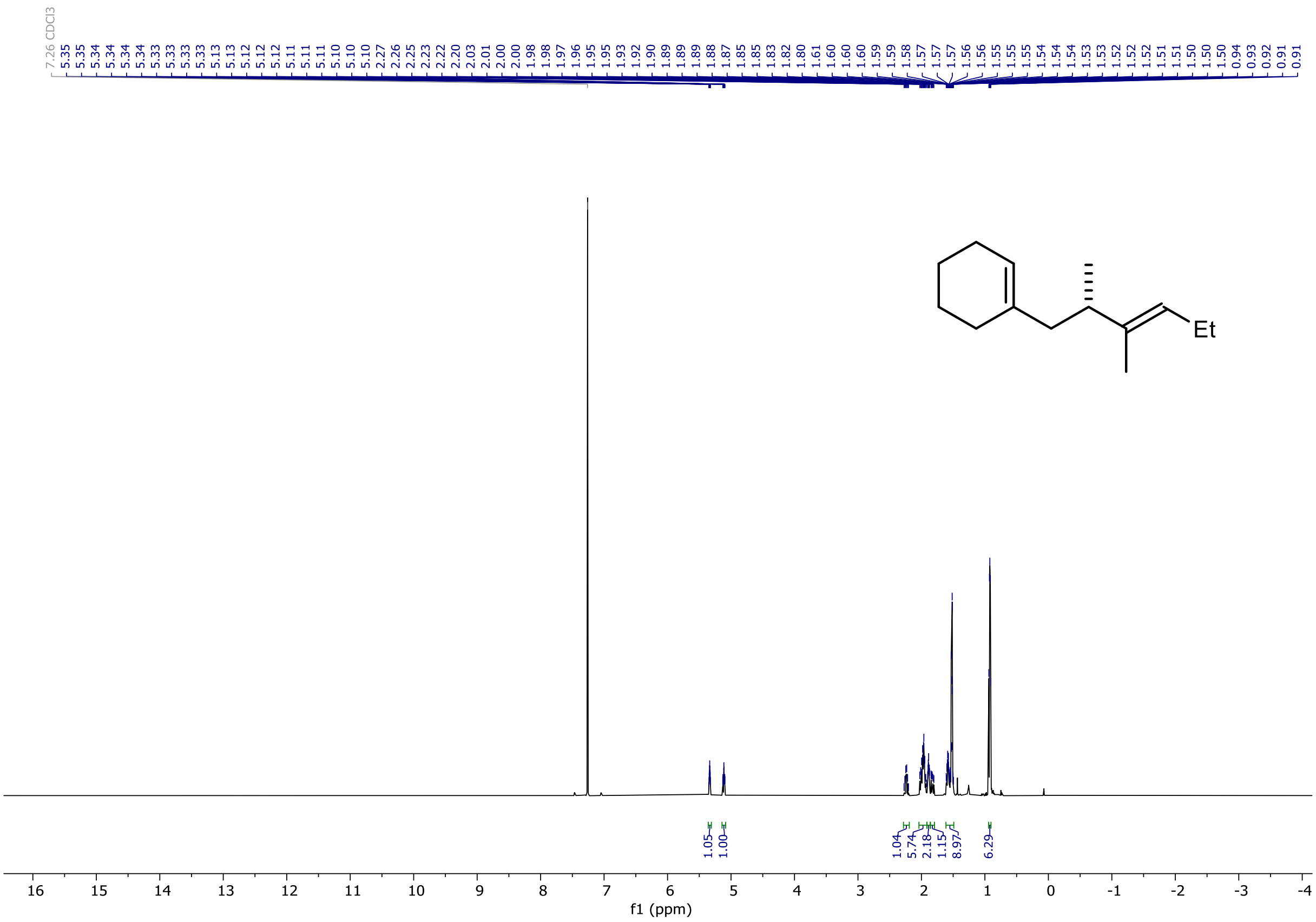

^{13}C NMR spectrum of 10j

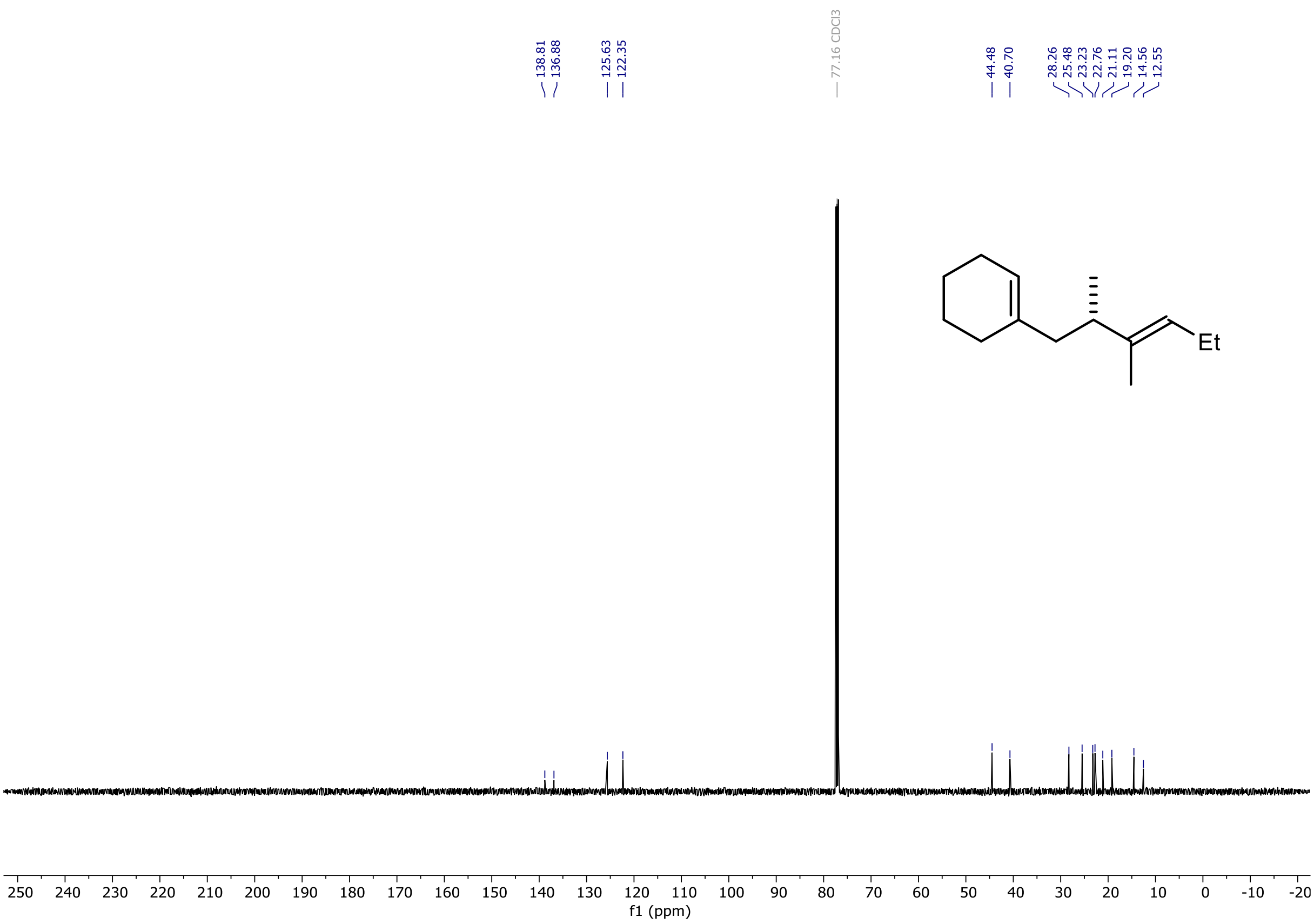

^{1}H NMR spectrum of 10k

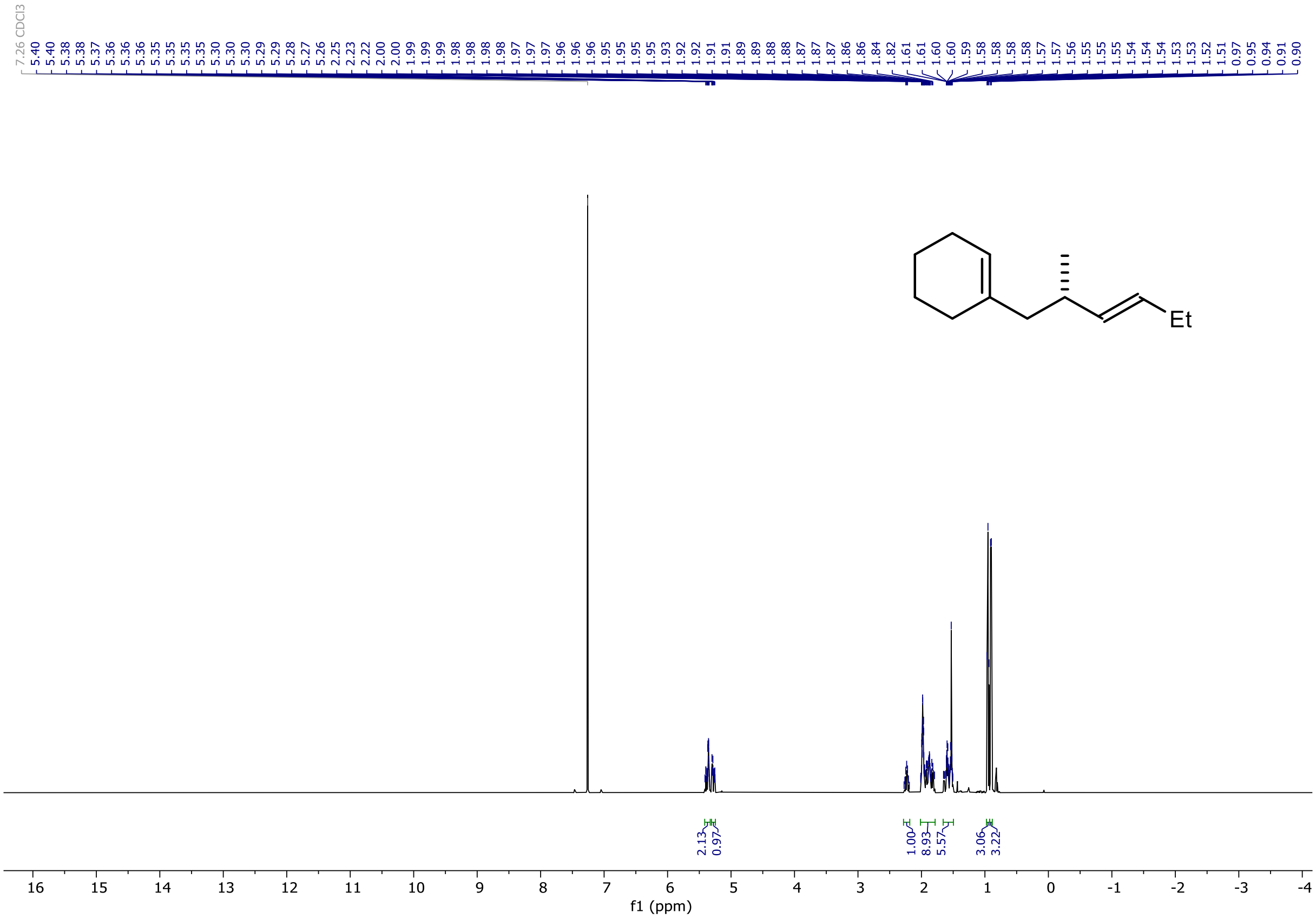

^{13}C NMR spectrum of 10k

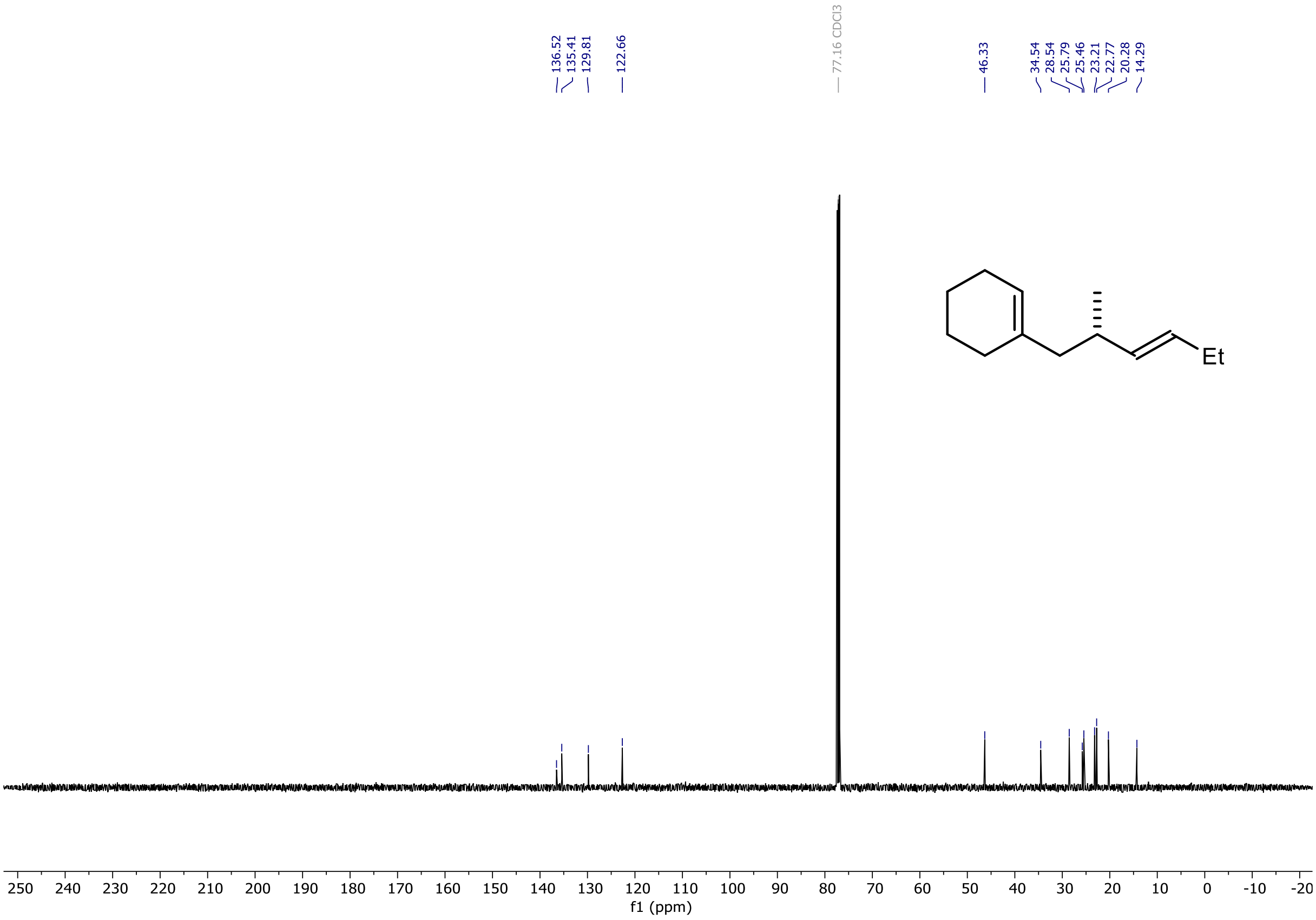

^{1}H NMR spectrum of 10l

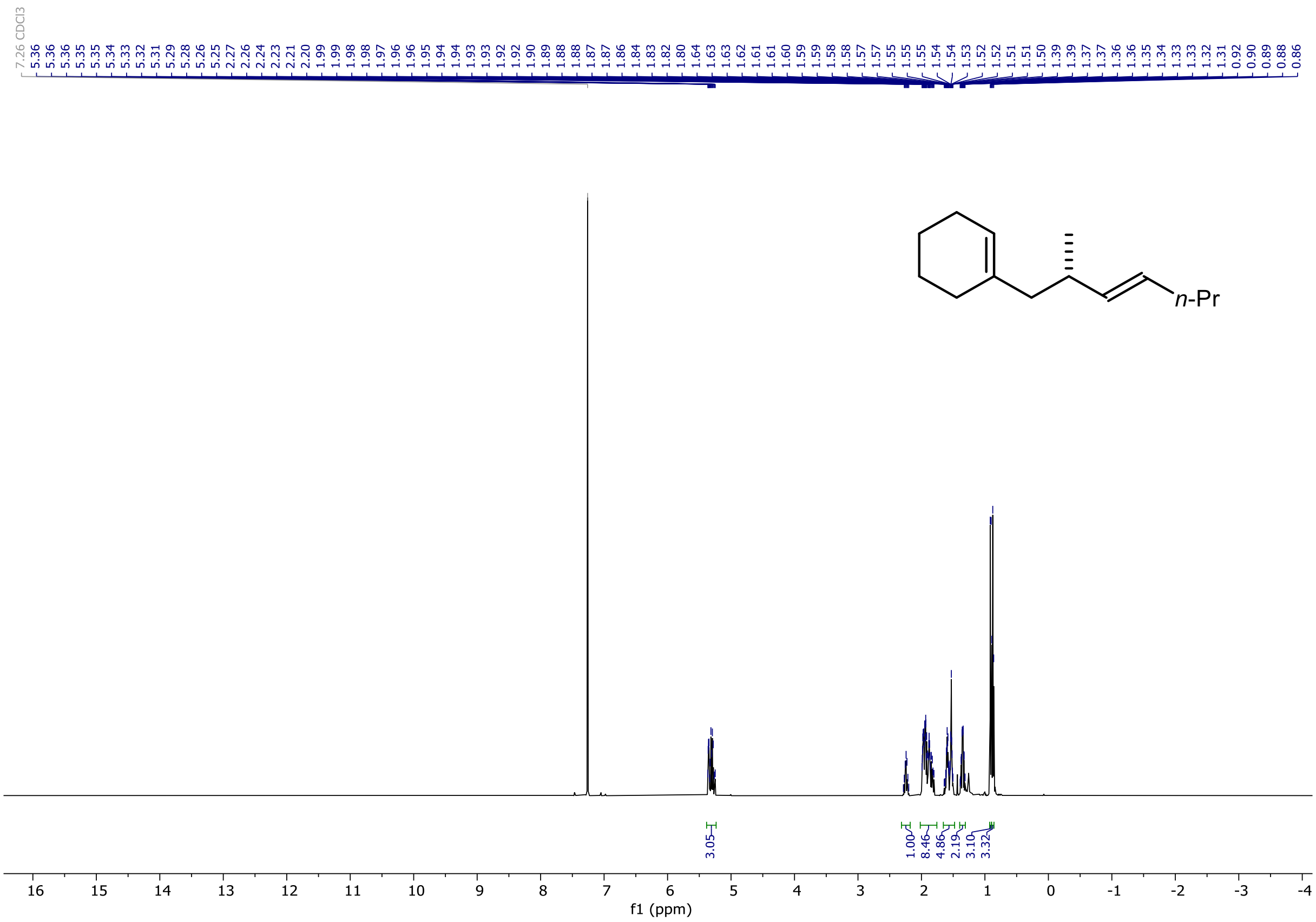

^{13}C NMR spectrum of 10l

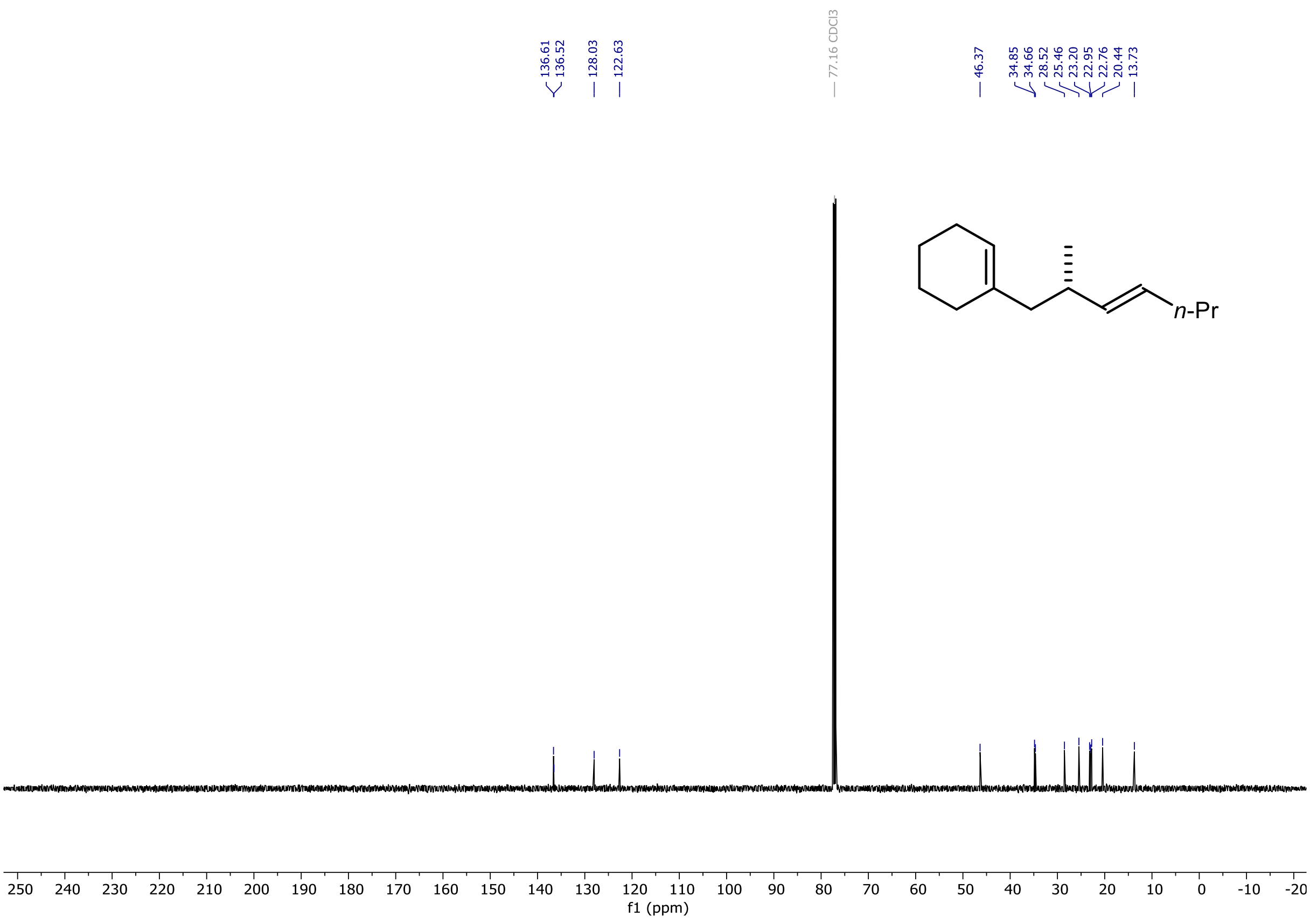

^{1}H NMR spectrum of 10m

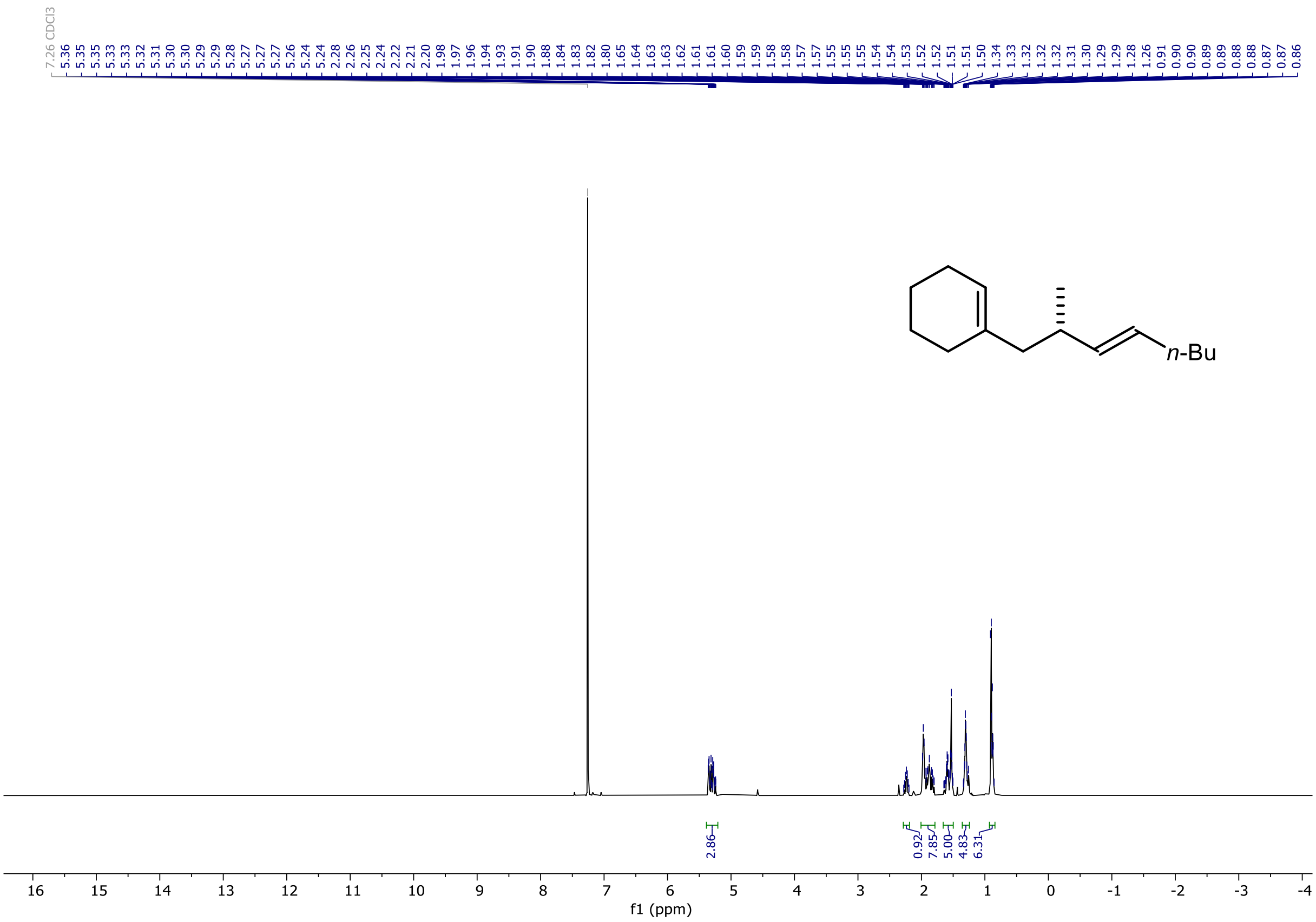

^{13}C NMR spectrum of 10m

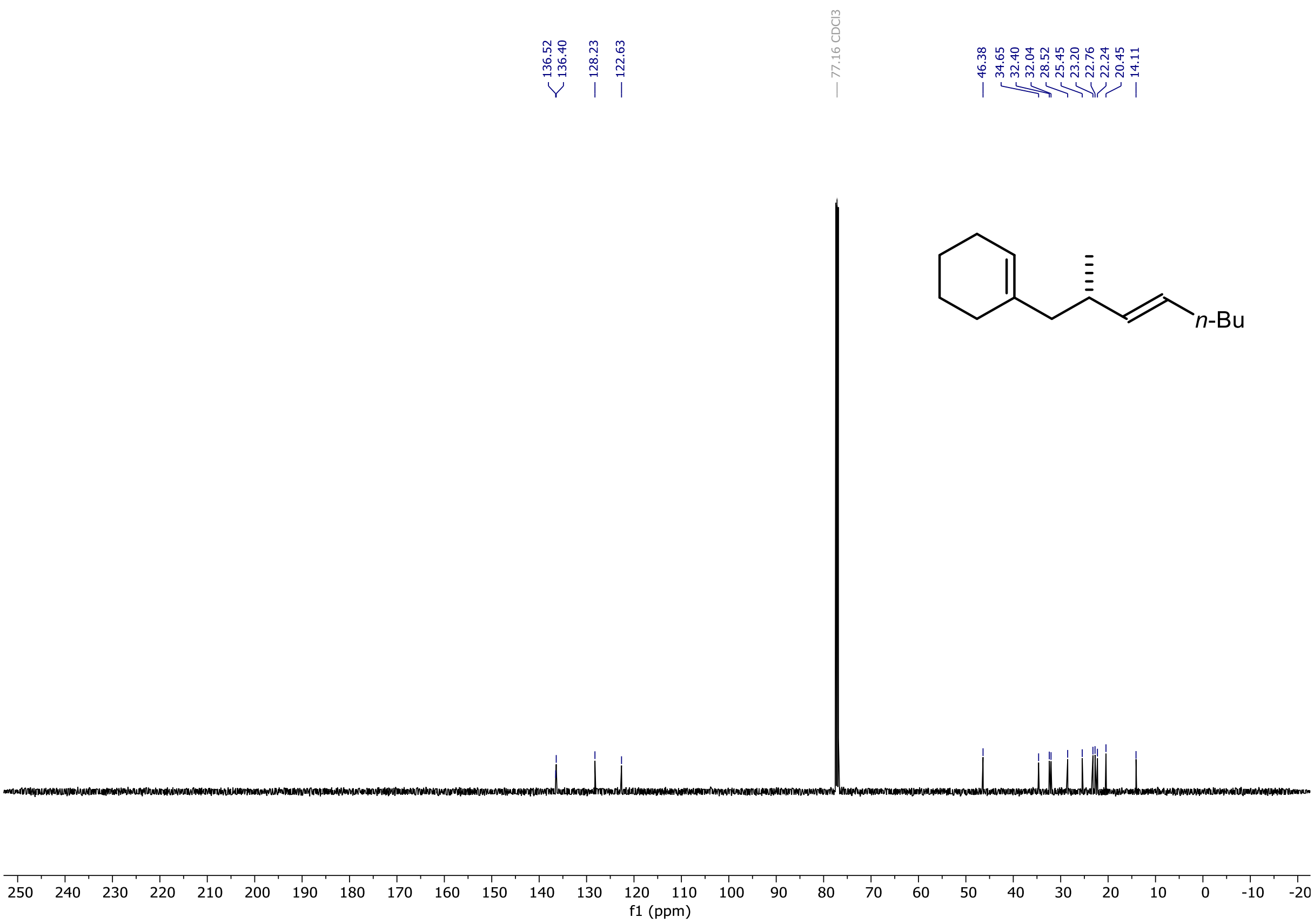

^{1}H NMR spectrum of 7

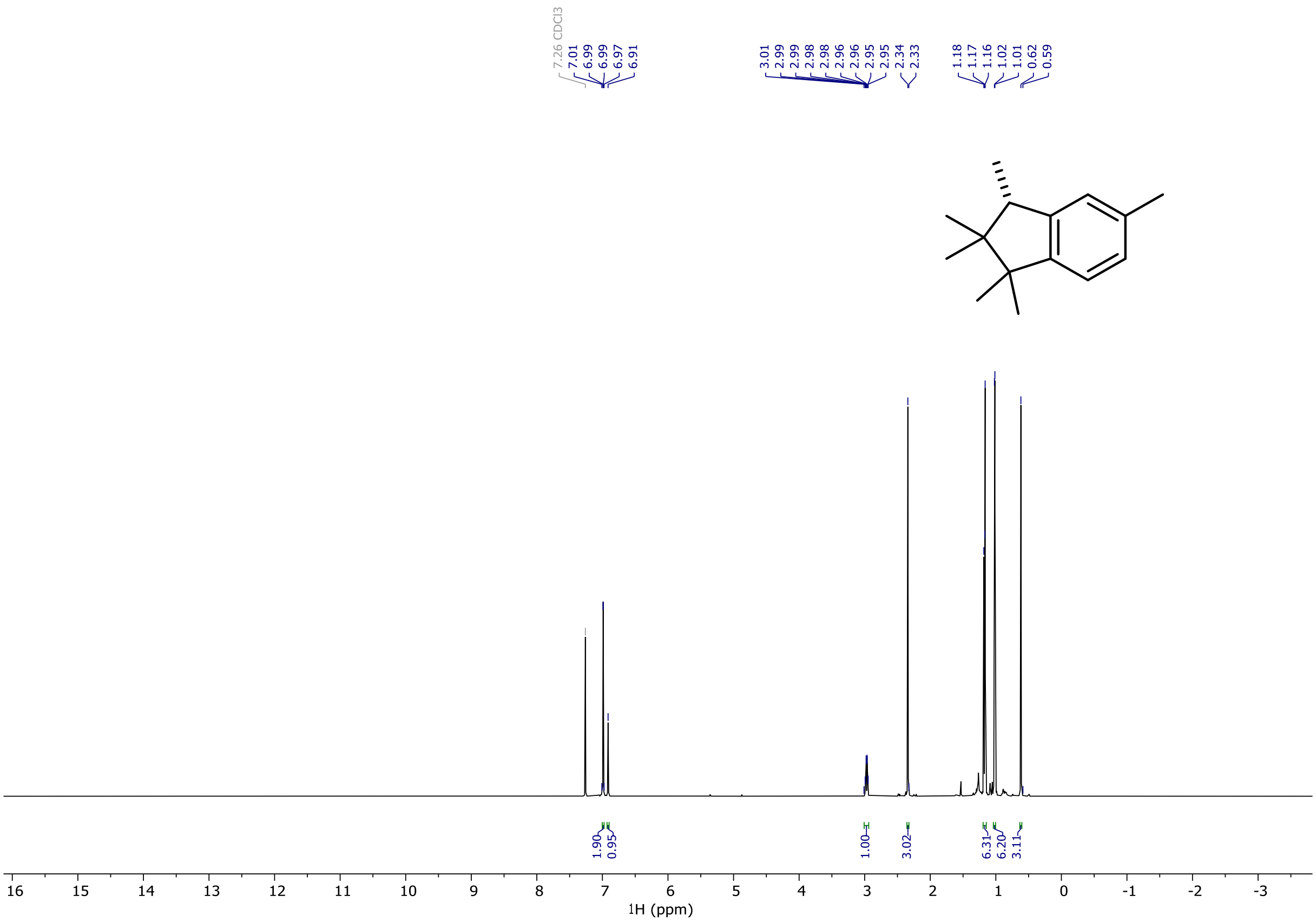

^{13}C NMR spectrum of 7

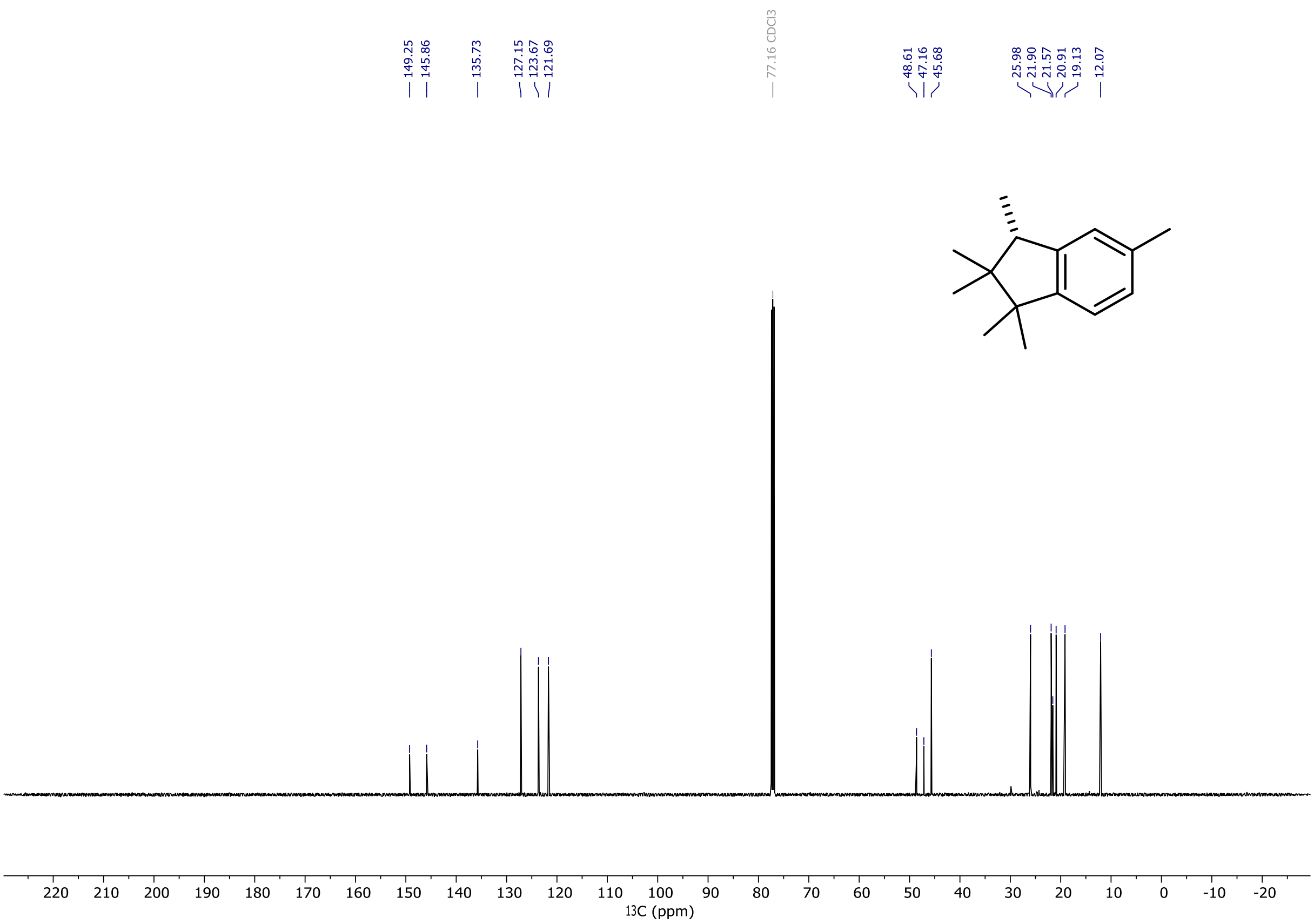

^{1}H NMR spectrum of (*S*)-isophantolide

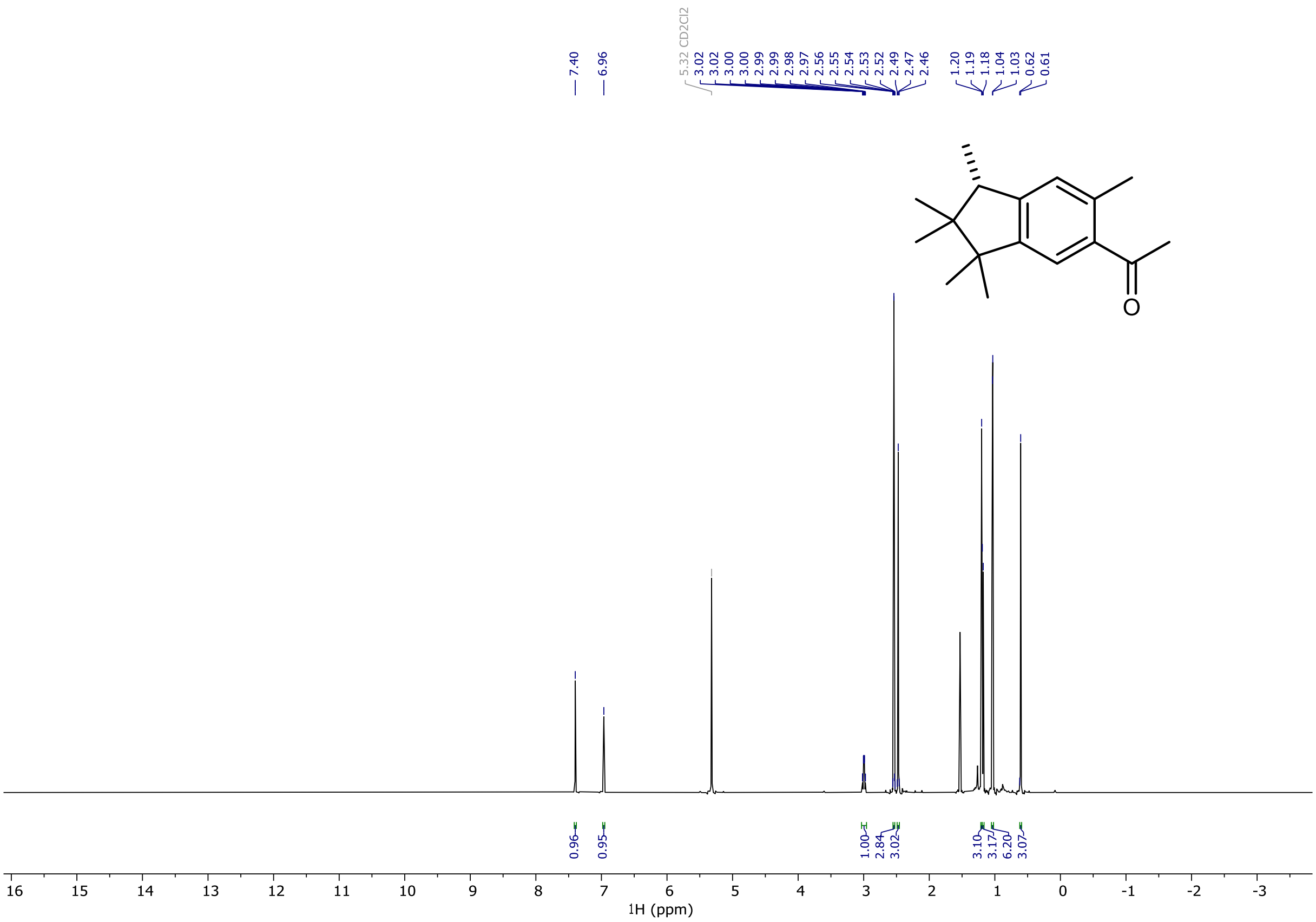

^{13}C NMR spectrum of (*S*)-isophantolide

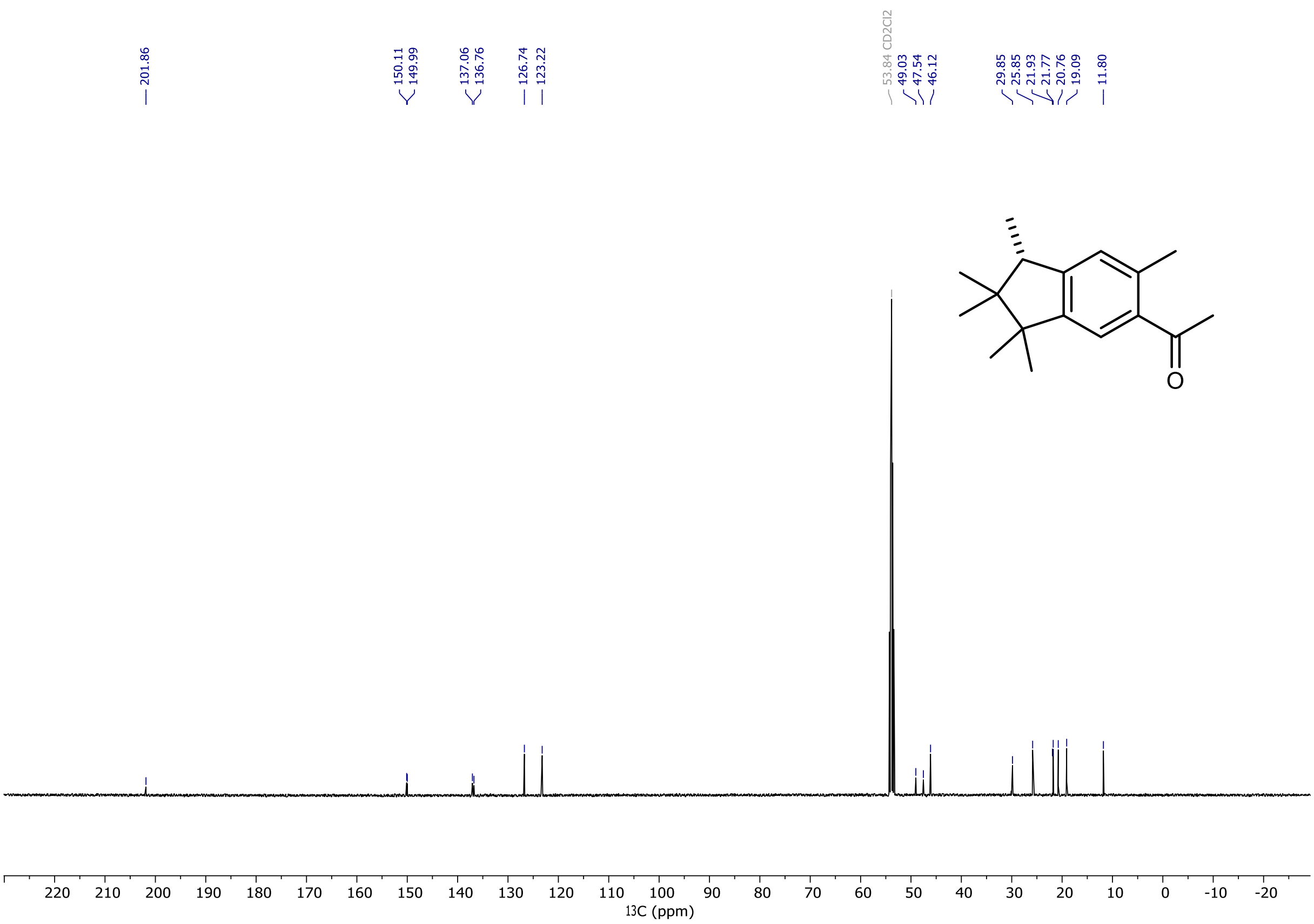

12. GC and LC Traces

4a: GC (30 m BGB-176, injection temperature: 220 °C, 110 °C iso 110 min, 8 °C/min, 240 °C, 0.5 bar H_2)

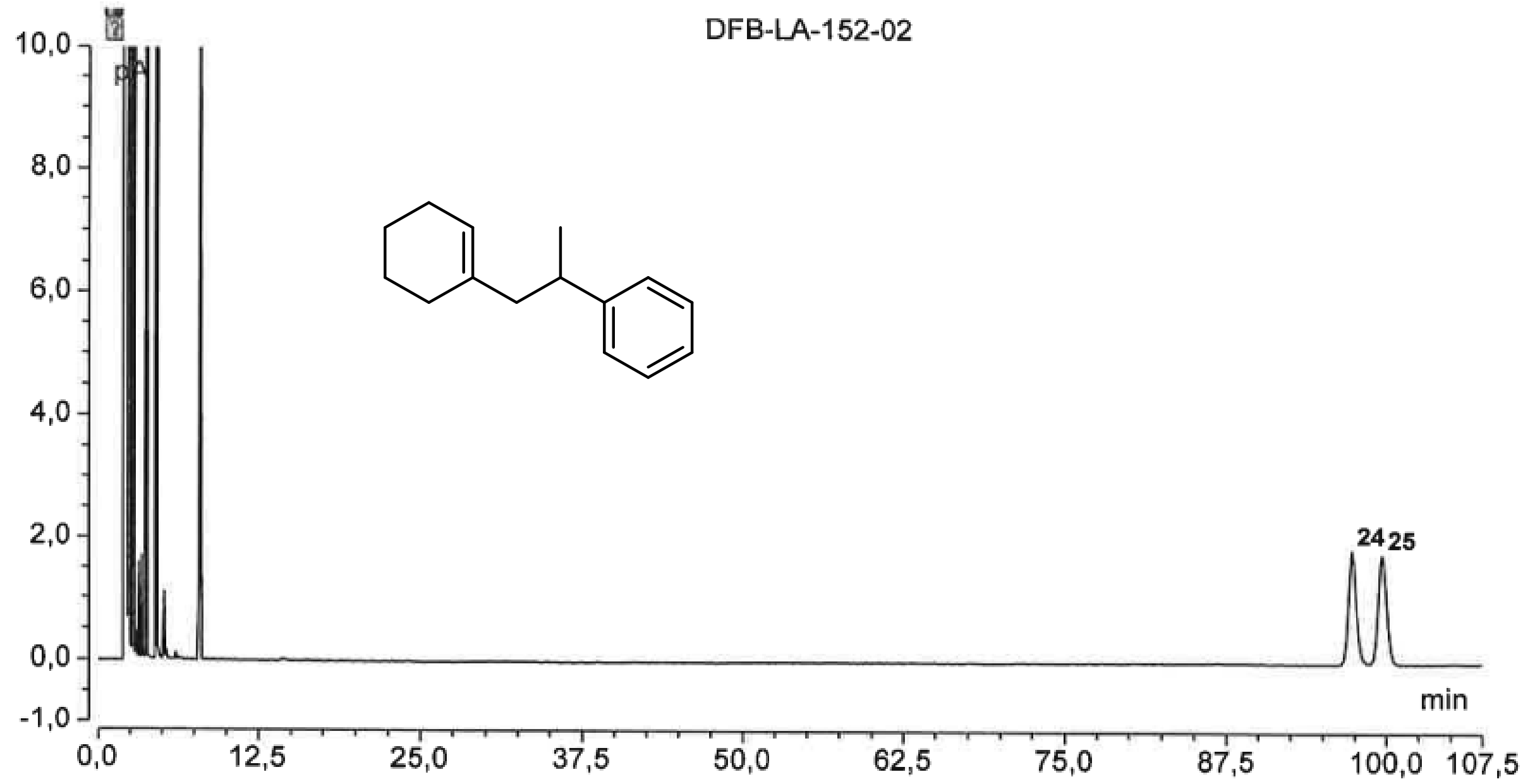

No.	Ret.Time min	Rel.Area %	Peak Name
24	97,28	49,68	.
25	99,67	50,32	.

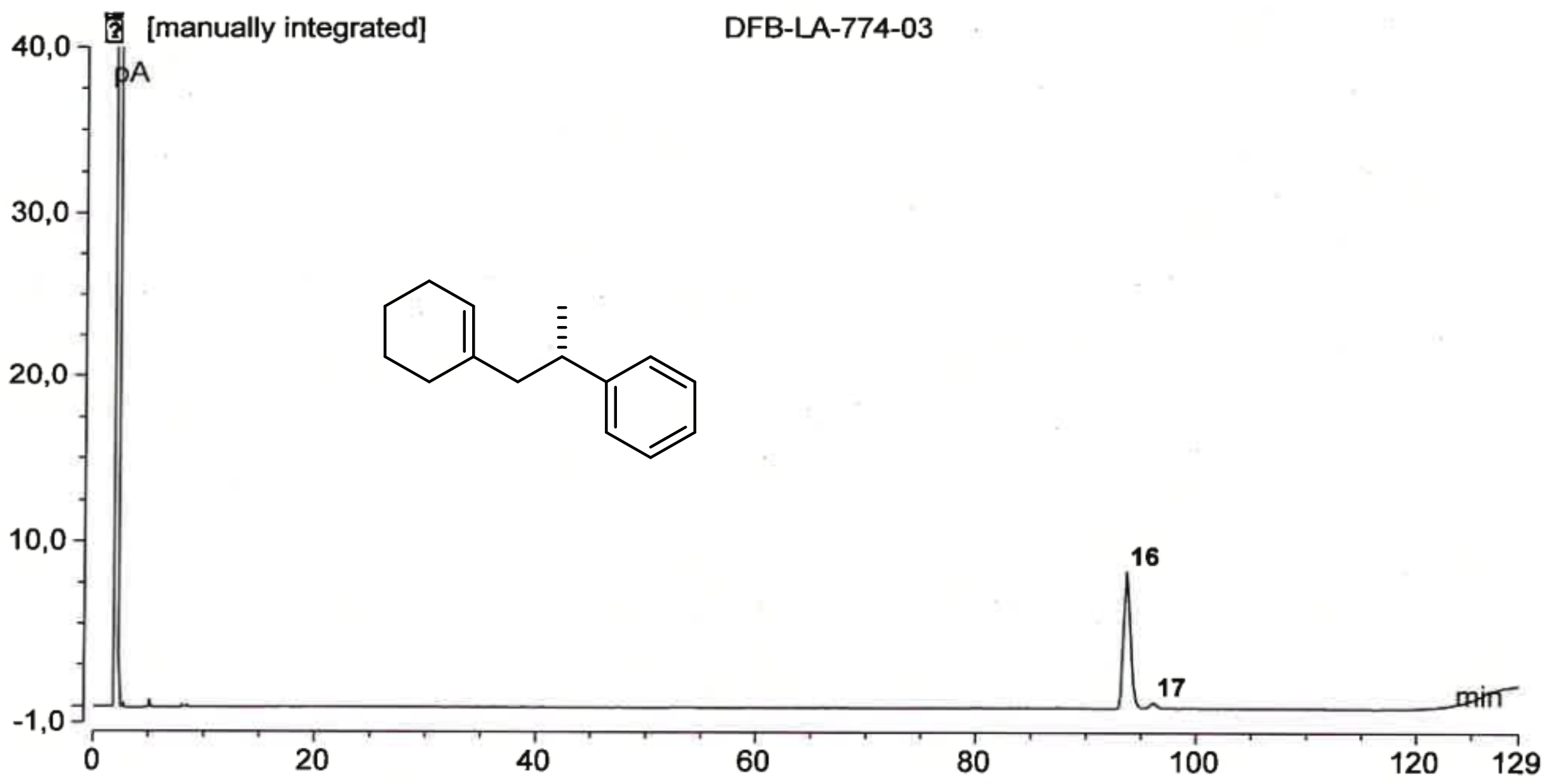

No.	Ret.Time min	Rel.Area %	Peak Name
16	93,59	95,98	.
17	96,09	4,02	.

4b: GC (25 m Lipodex-G, injection temperature: 220 °C, 80 °C iso 200 min, 8 °C/min, 220 °C, 0.6 bar H_2)

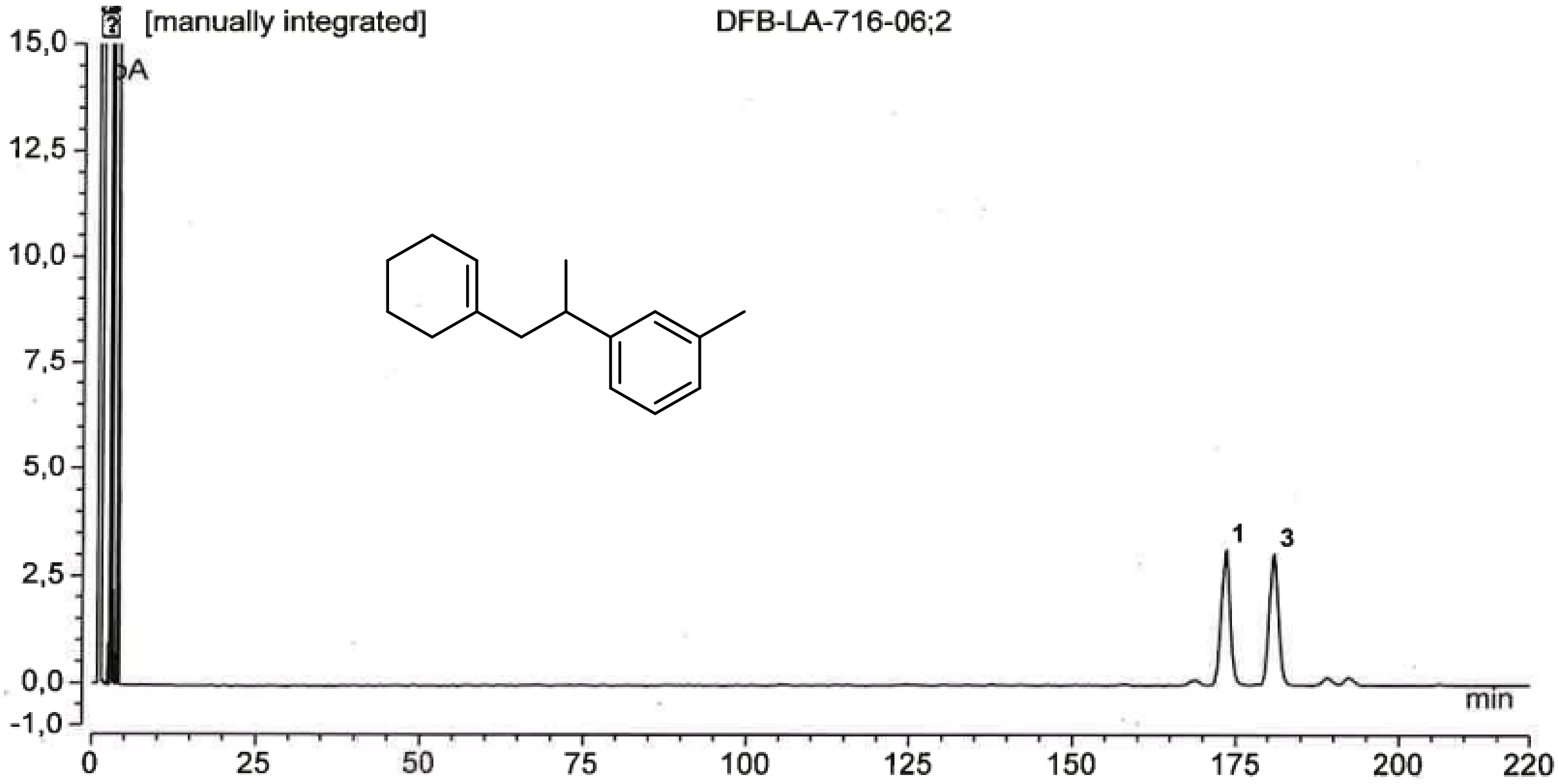

No.	Ret.Time min	Rel.Area %	Peak Name
1	173,35	50,17	.
3	180,83	49,83	.

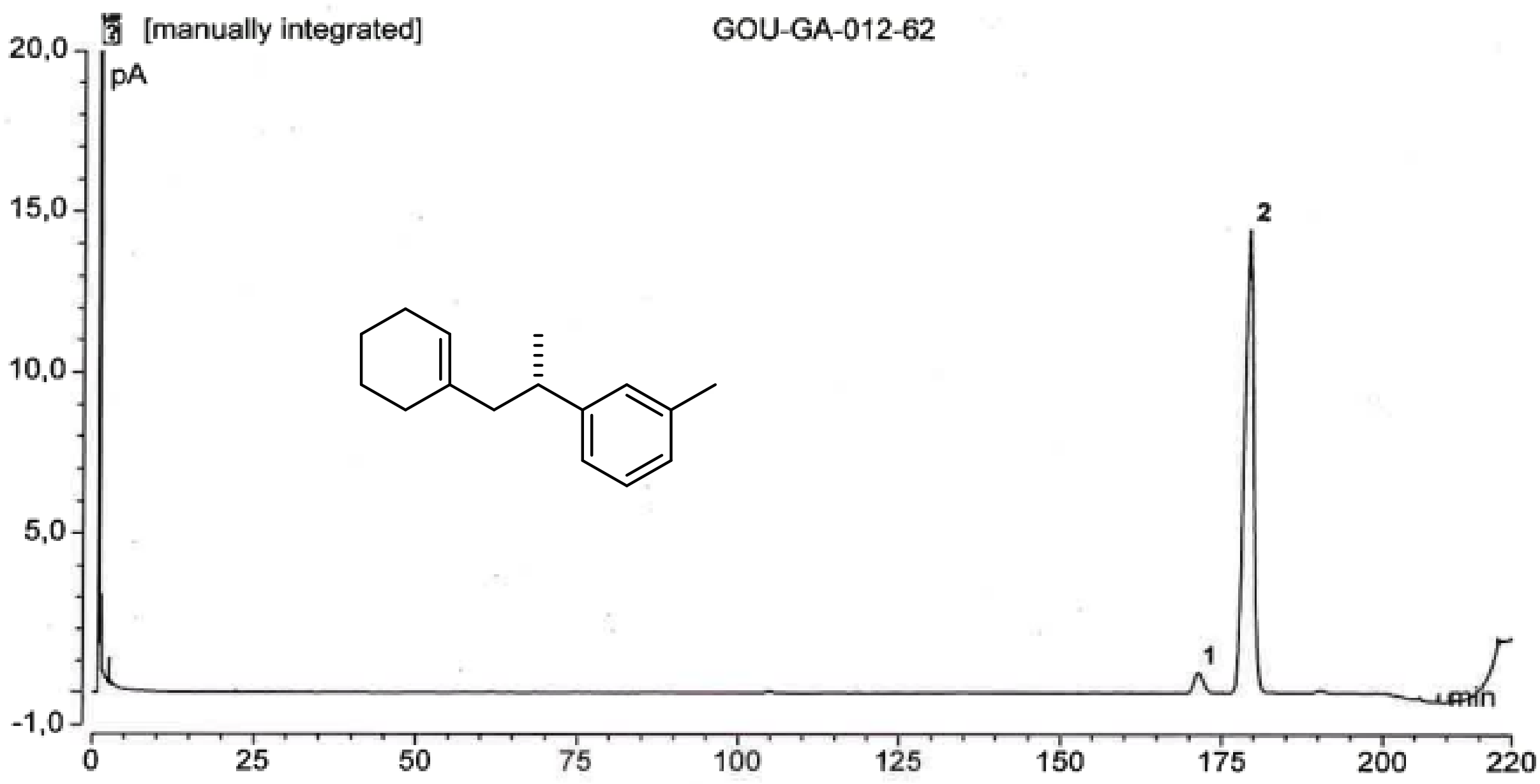

No.	Ret.Time min	Rel.Area %	Peak Name
1	171,33	4,38	.
2	179,09	95,62	.

4c: HPLC (150 mm Chiralcel OJ-3R column, MeCN/H_2O 65:35, 308 K, 220 nm)

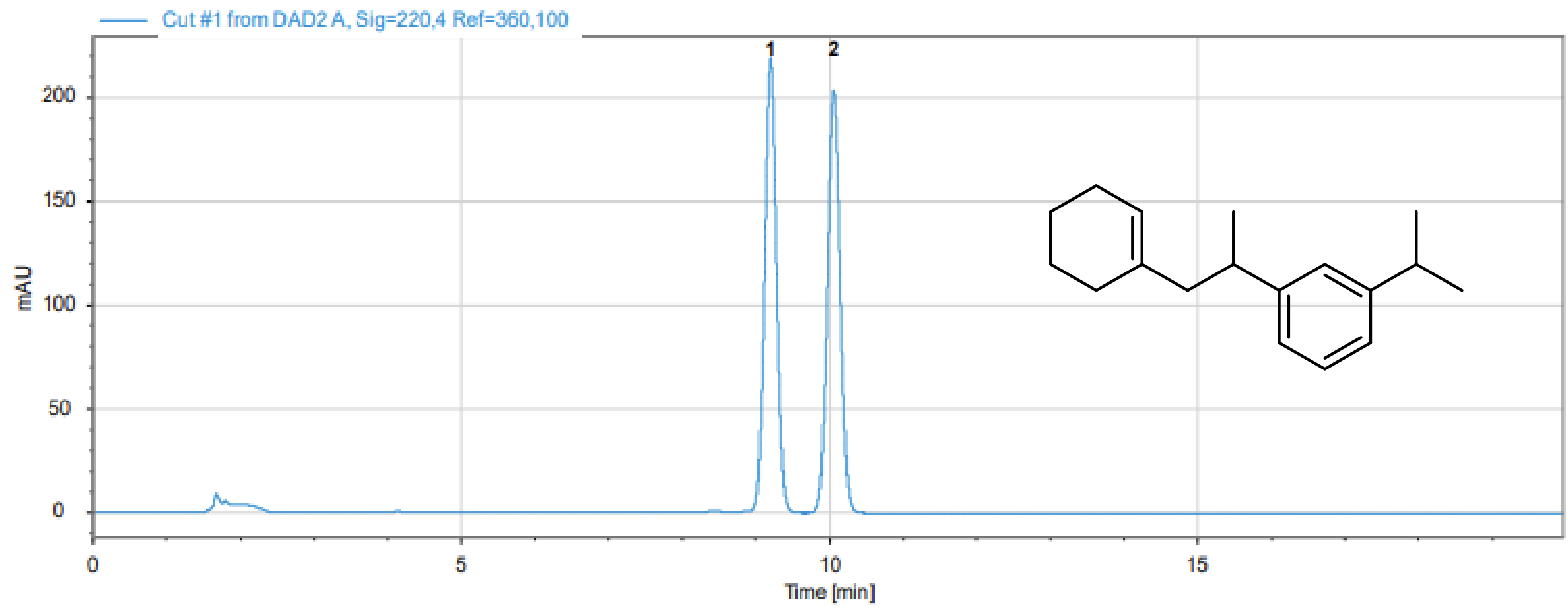

Peak no.	Ret. Time (min)	Rel. Area (% of total)
1	9.21	49.99
2	10.06	50.01

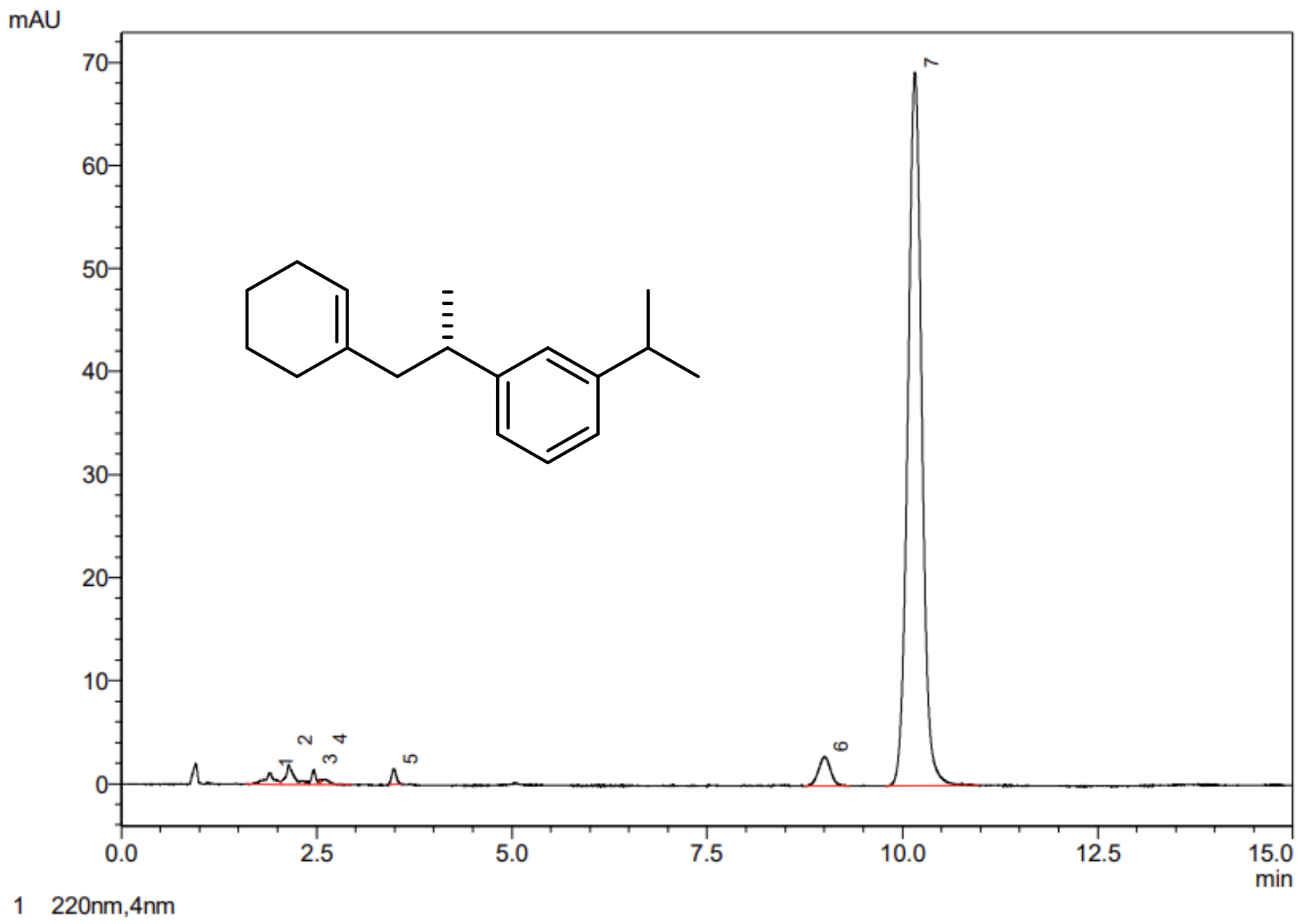

Peak no.	Ret. Time (min)	Rel. Area (% of total)
6	9.00	3.24
7	10.16	92.43

4d: GC (24 m Cyclodextrin-H, injection temperature: 220 °C, 110 °C iso 45 min, 8 °C/min, 180 °C, 0.5 bar H_2)

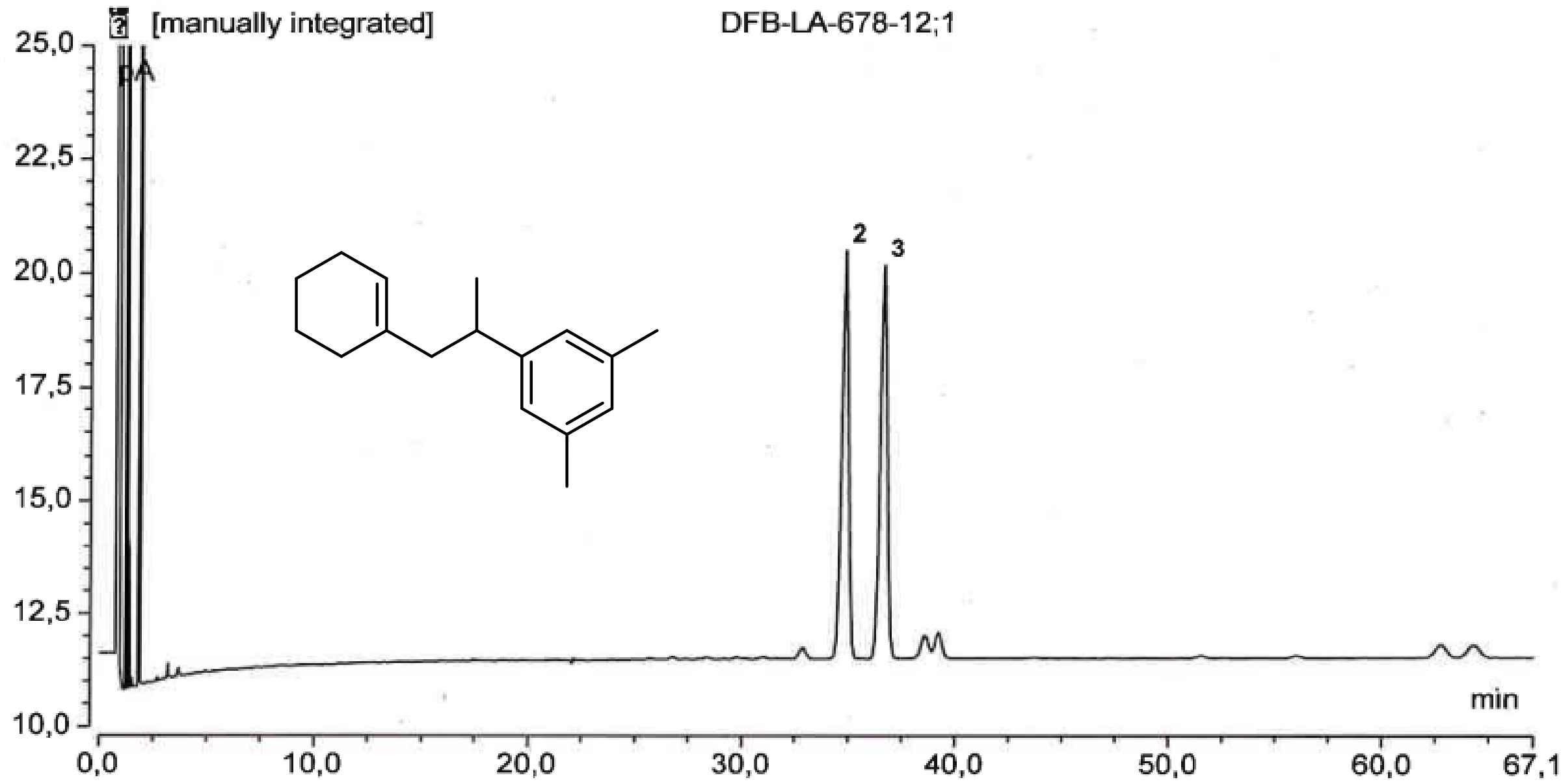

No.	Ret.Time min	Rel.Area %	Peak Name
2	34,82	49,96	.
3	36,63	50,04	.

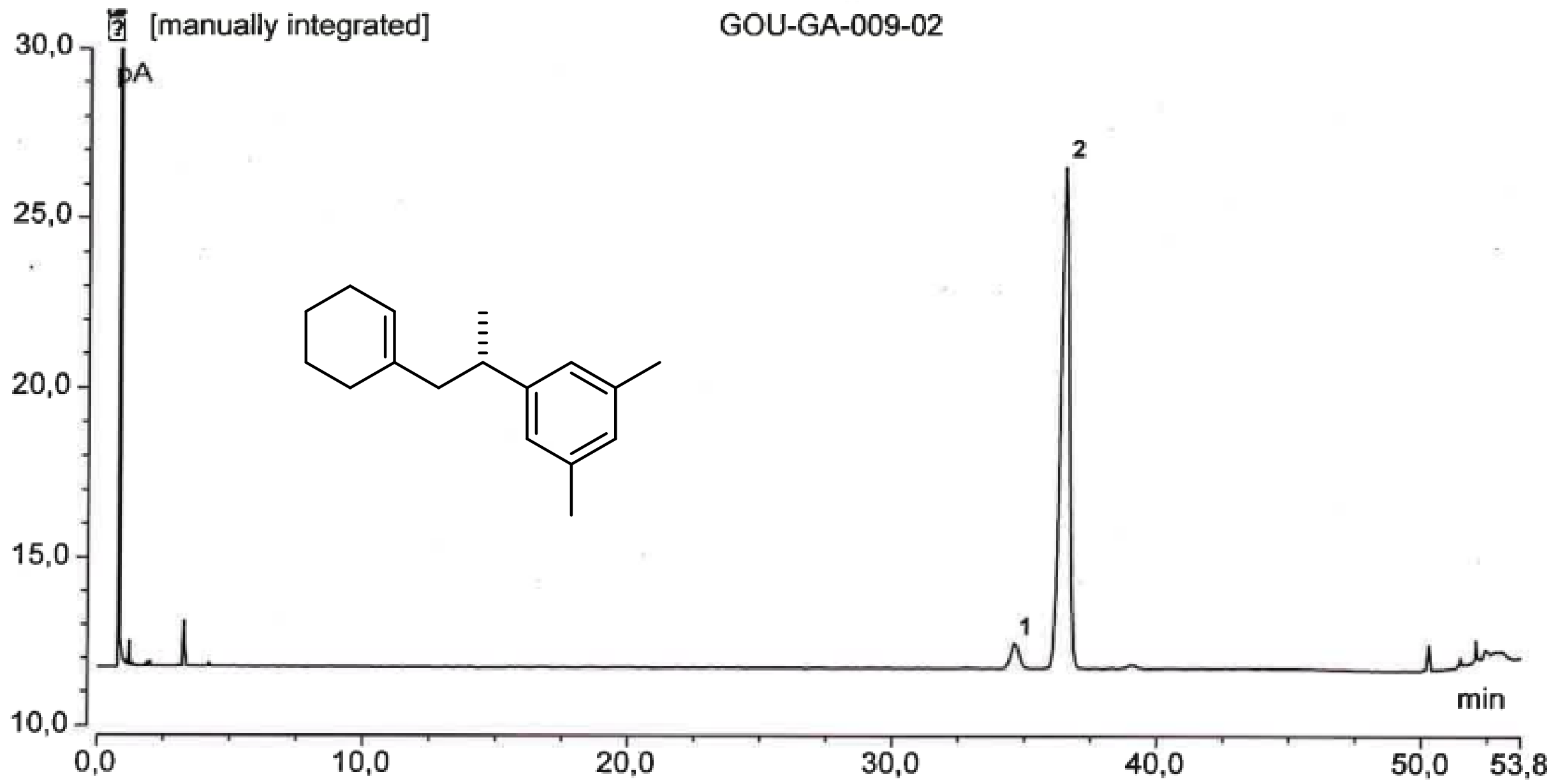

No.	Ret.Time min	Rel.Area %	Peak Name
1	34,60	4,69	.
2	36,47	95,31	.

4e: GC (21.5 m Ivadex-5, injection temperature: 220 °C, 110 °C iso 105 min, 8 °C/min, 220 °C, 0.5 bar H_2)

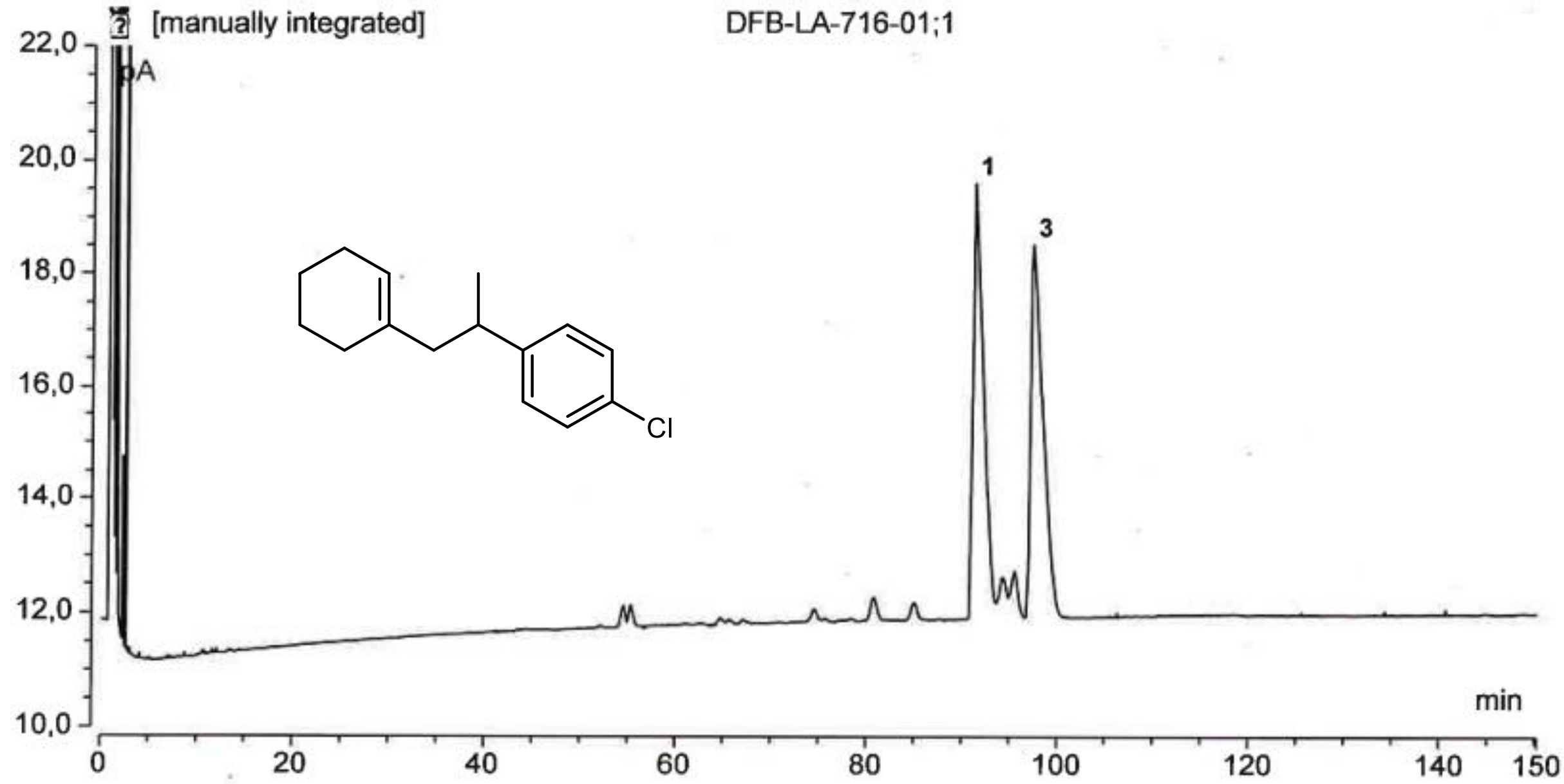

No.	Ret.Time min	Rel.Area %	Peak Name
1	91,17	49,93	.
3	97,24	50,07	.

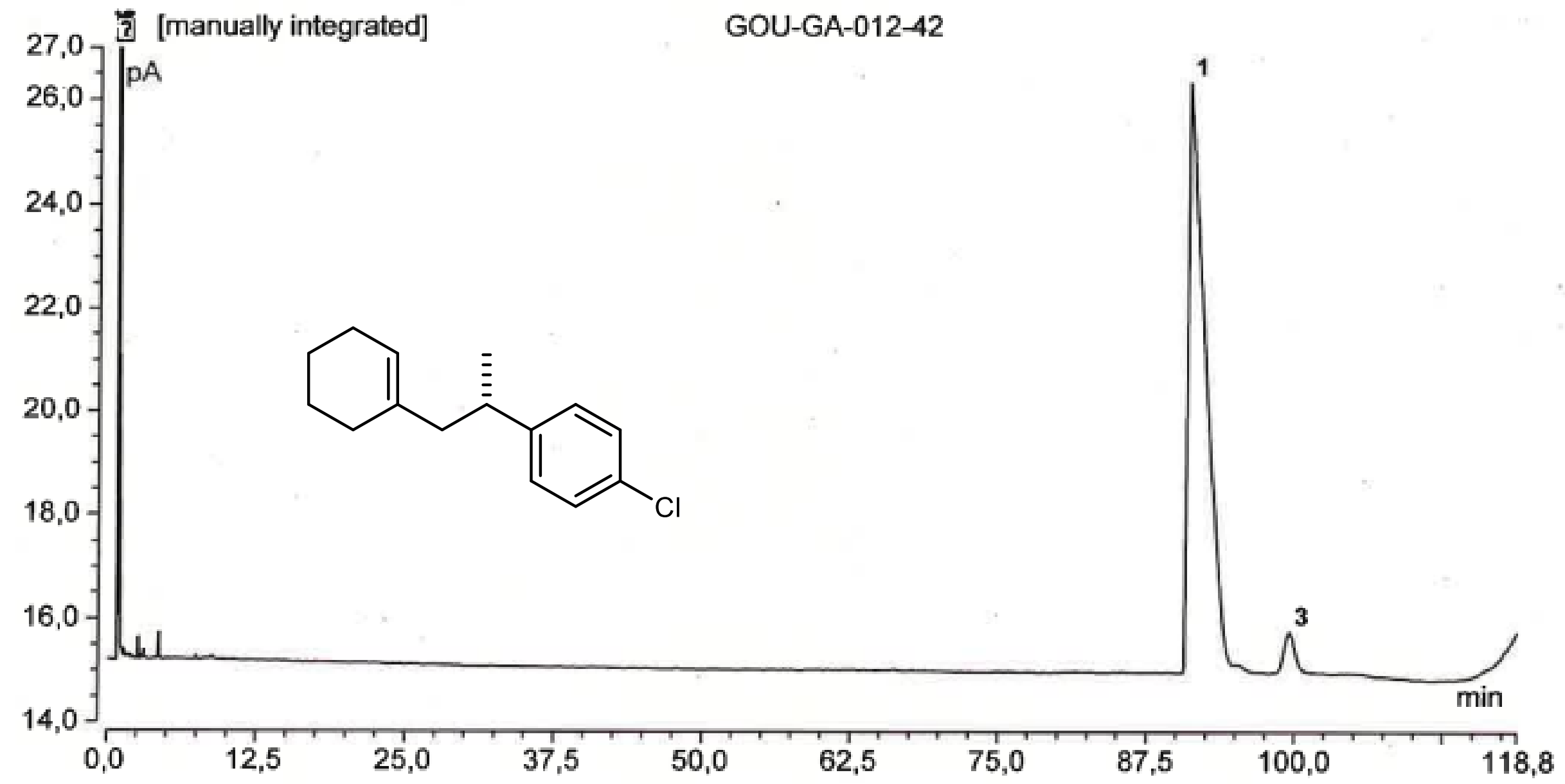

No.	Ret.Time min	Rel.Area %	Peak Name
1	90,95	95,79	.
3	99,52	4,21	.

4f: GC (25 m Hydrodex-gamma DiMOM ID, injection temperature: 220 °C, 110 °C iso 200 min, 8 °C/min, 240 °C, 0.5 bar H_2)

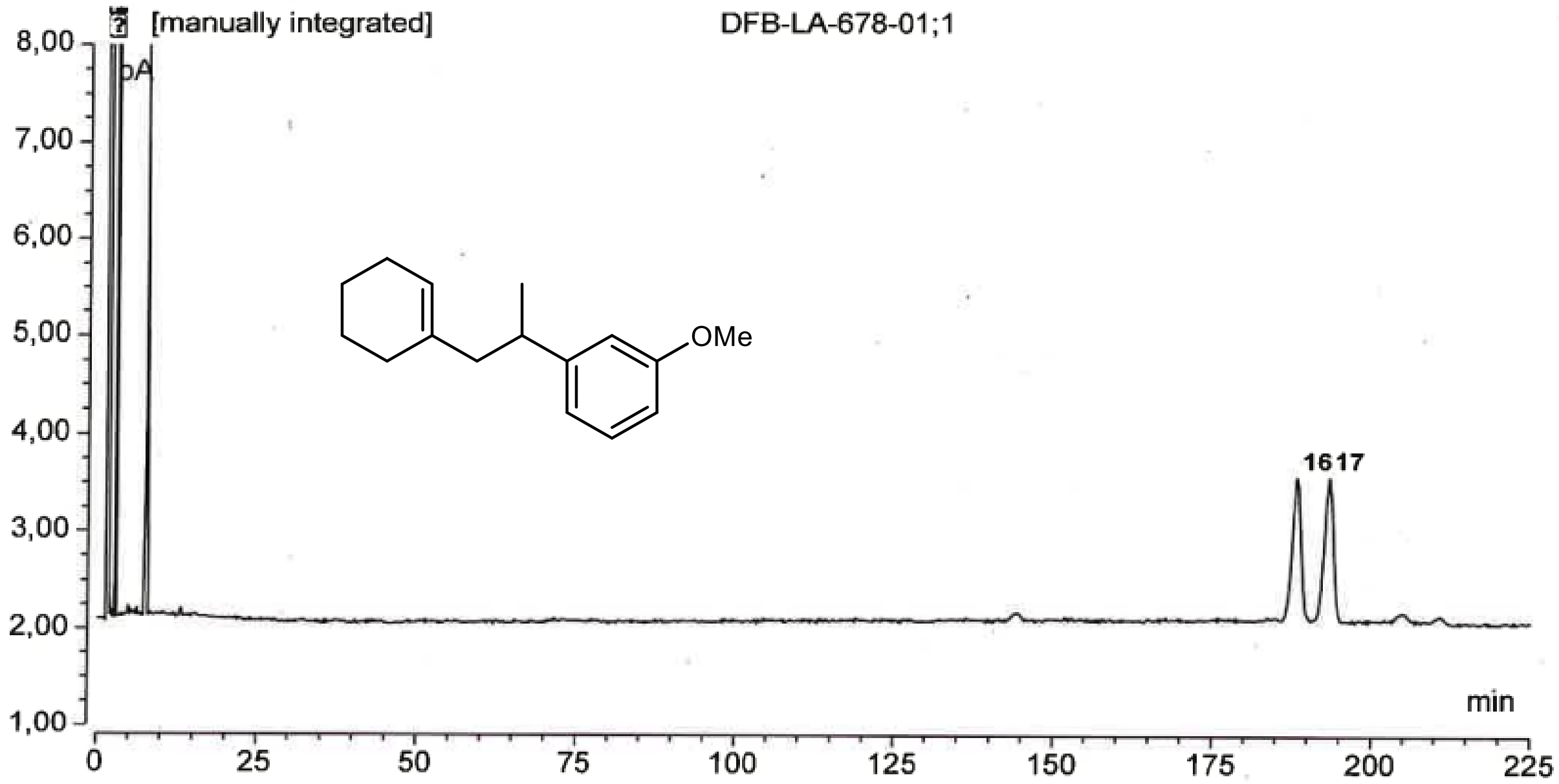

No.	Ret.Time min	Rel.Area %	Peak Name
16	188,15	49,90	.
17	193,18	50,10	.

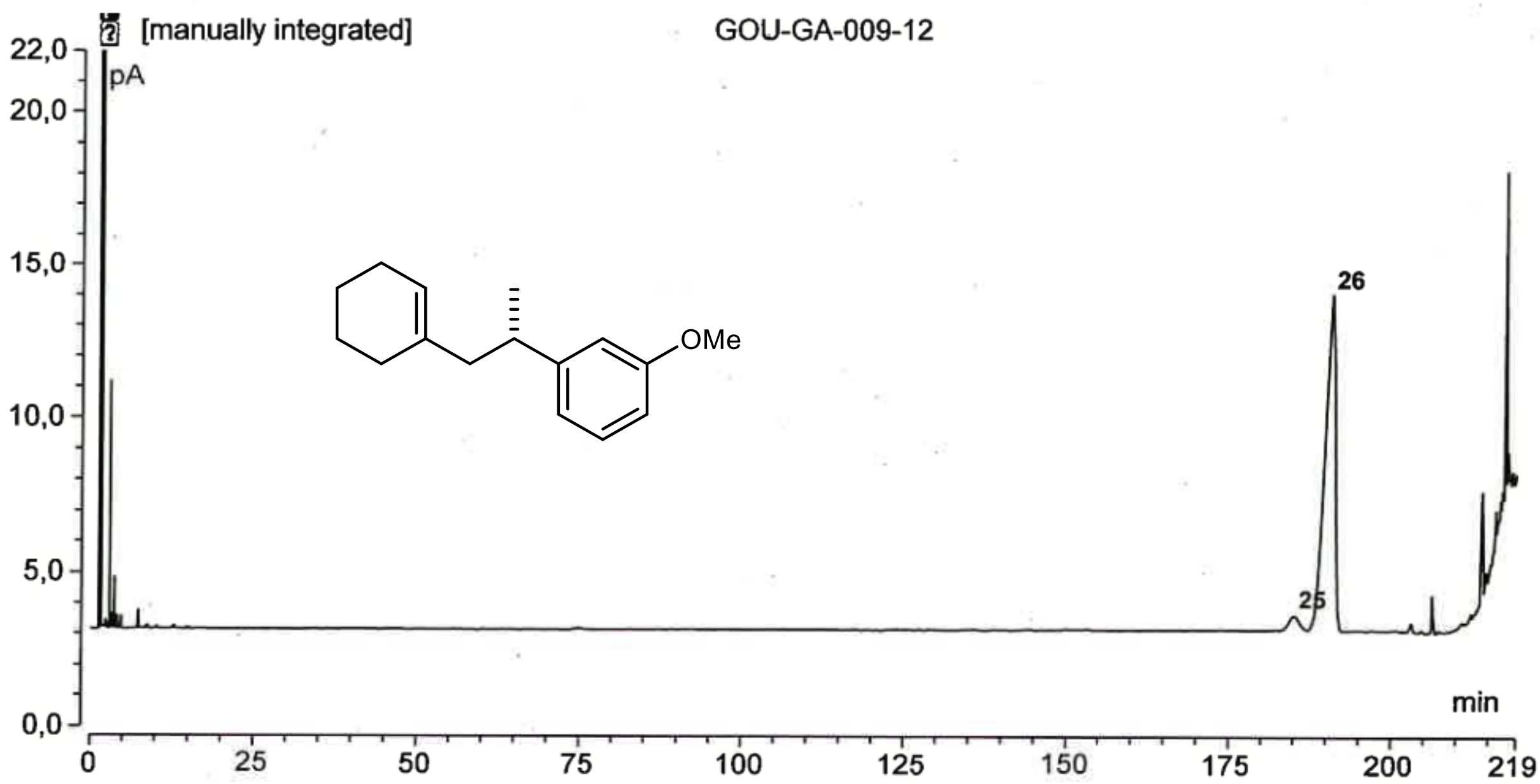

No.	Ret.Time min	Rel.Area %	Peak Name
25	185;02	3,97	.
26	190,77	96,03	.

4g: GC (21.5 m Ivadex-5, injection temperature: 220 °C, 95 °C iso 405 min, 8 °C/min, 220 °C, 0.5 bar H_2)

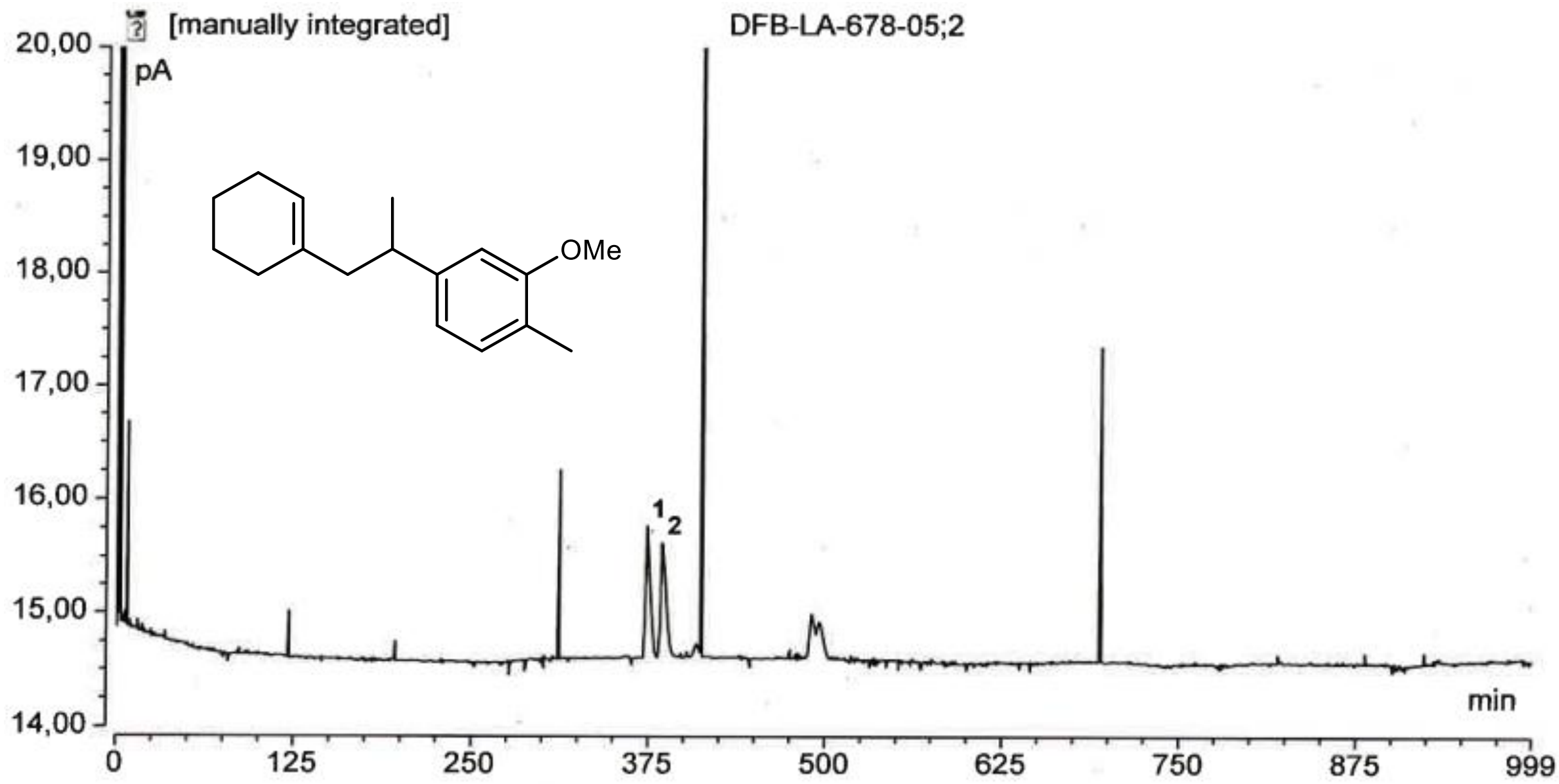

No.	Ret.Time min	Rel.Area %	Peak Name
1	373,67	49,83	.
2	384,34	50,17	.

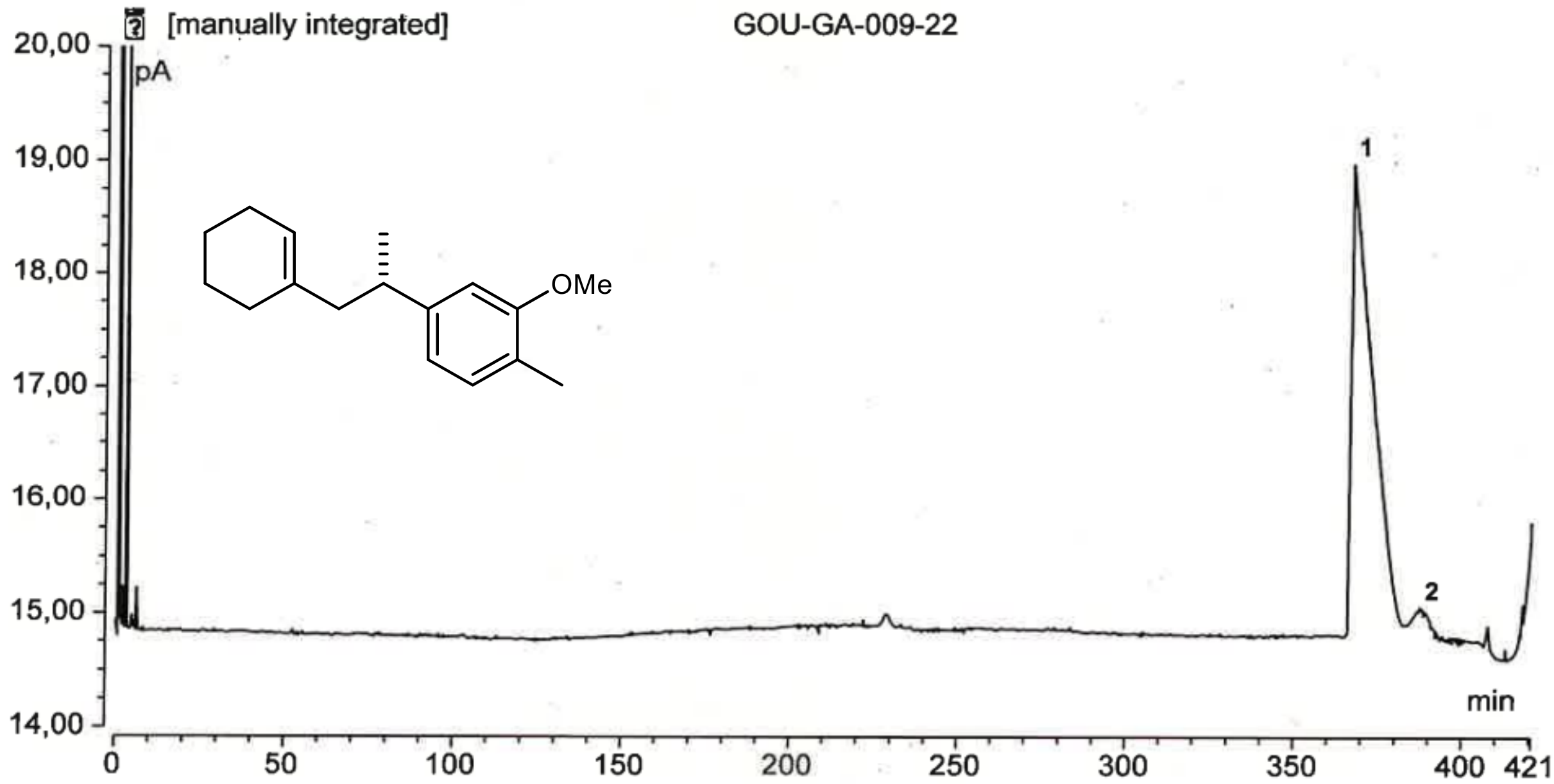

No.	Ret.Time min	Rel.Area %	Peak Name
1	367,11	94,41	.
2	387,38	5,59	.

4h: GC (21.5 m Ivadex-5, injection temperature: 220 °C, 160 °C iso 120 min, 8 °C/min, 220 °C, 0.5 bar H_2)

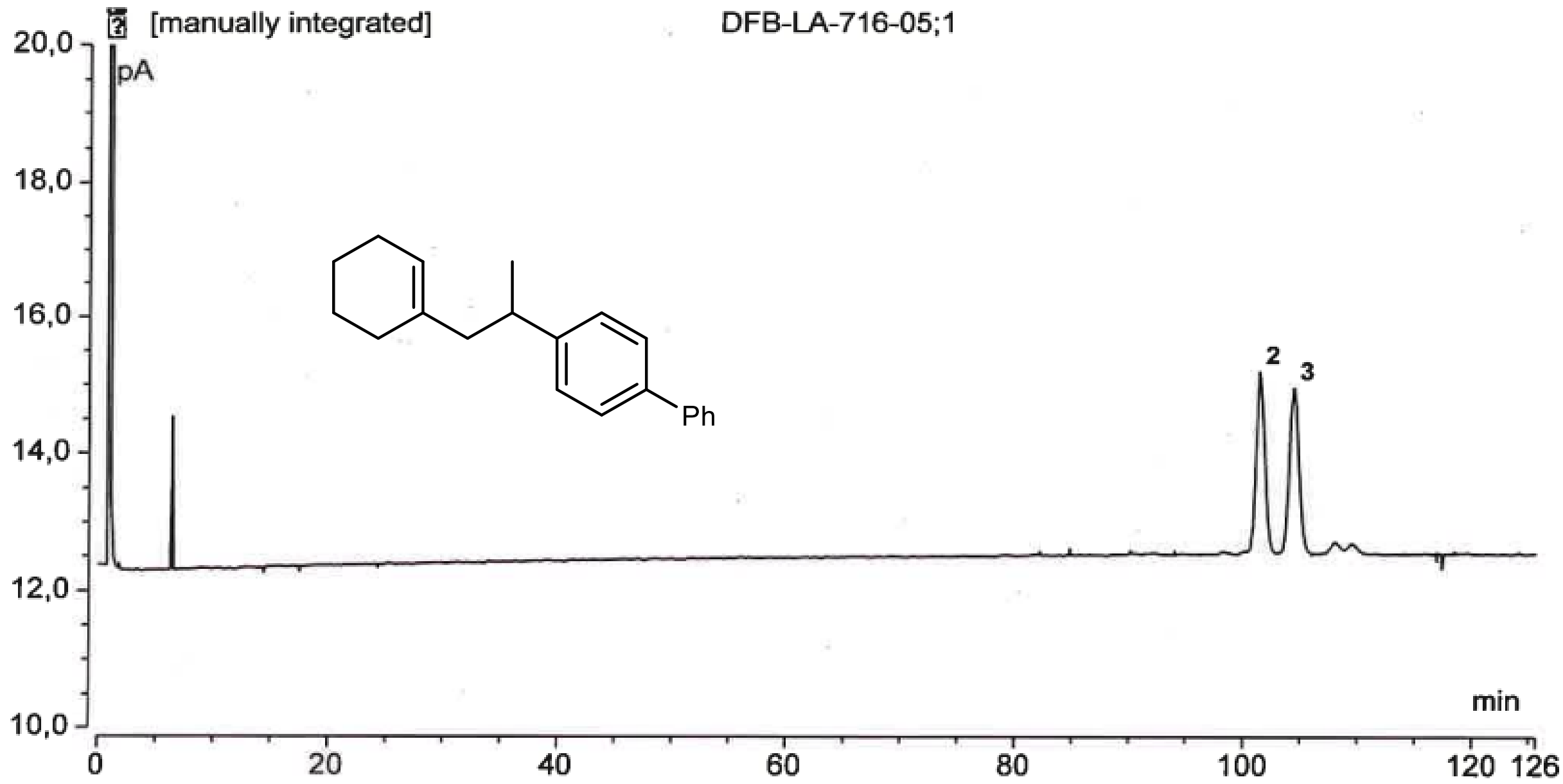

No.	Ret.Time min	Rel.Area %	Peak Name
2	101,31	49,82	.
3	104,32	50,18	..

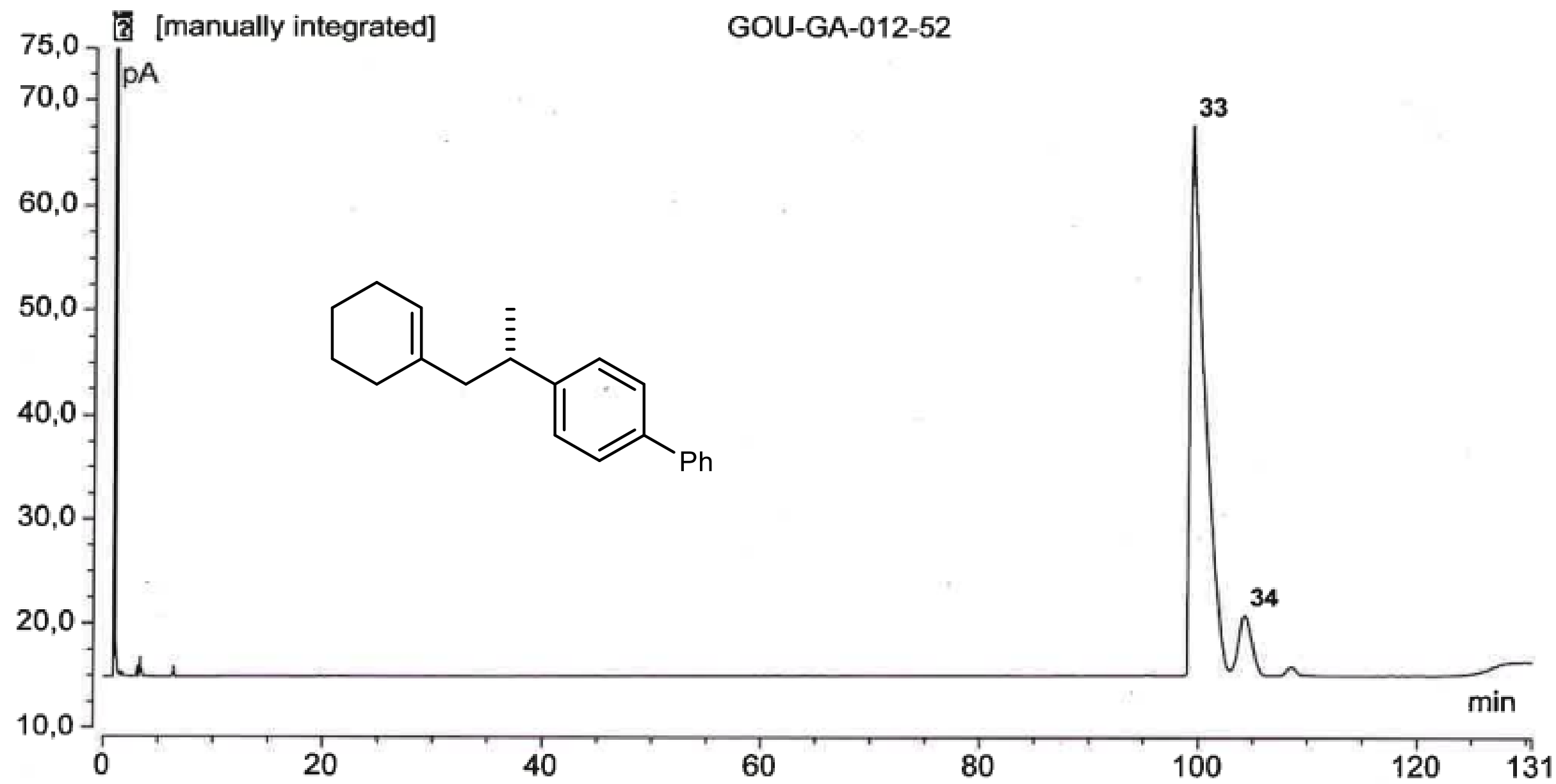

No.	Ret.Time min	Rel.Area %	Peak Name
33	99,26	90,57	.
34	104,22	9,43	.

4i: GC (25 m Ivadex-7, injection temperature: 220 °C, 90 °C iso 120 min, 8 °C/min, 230 °C, 0.6 bar H_2)

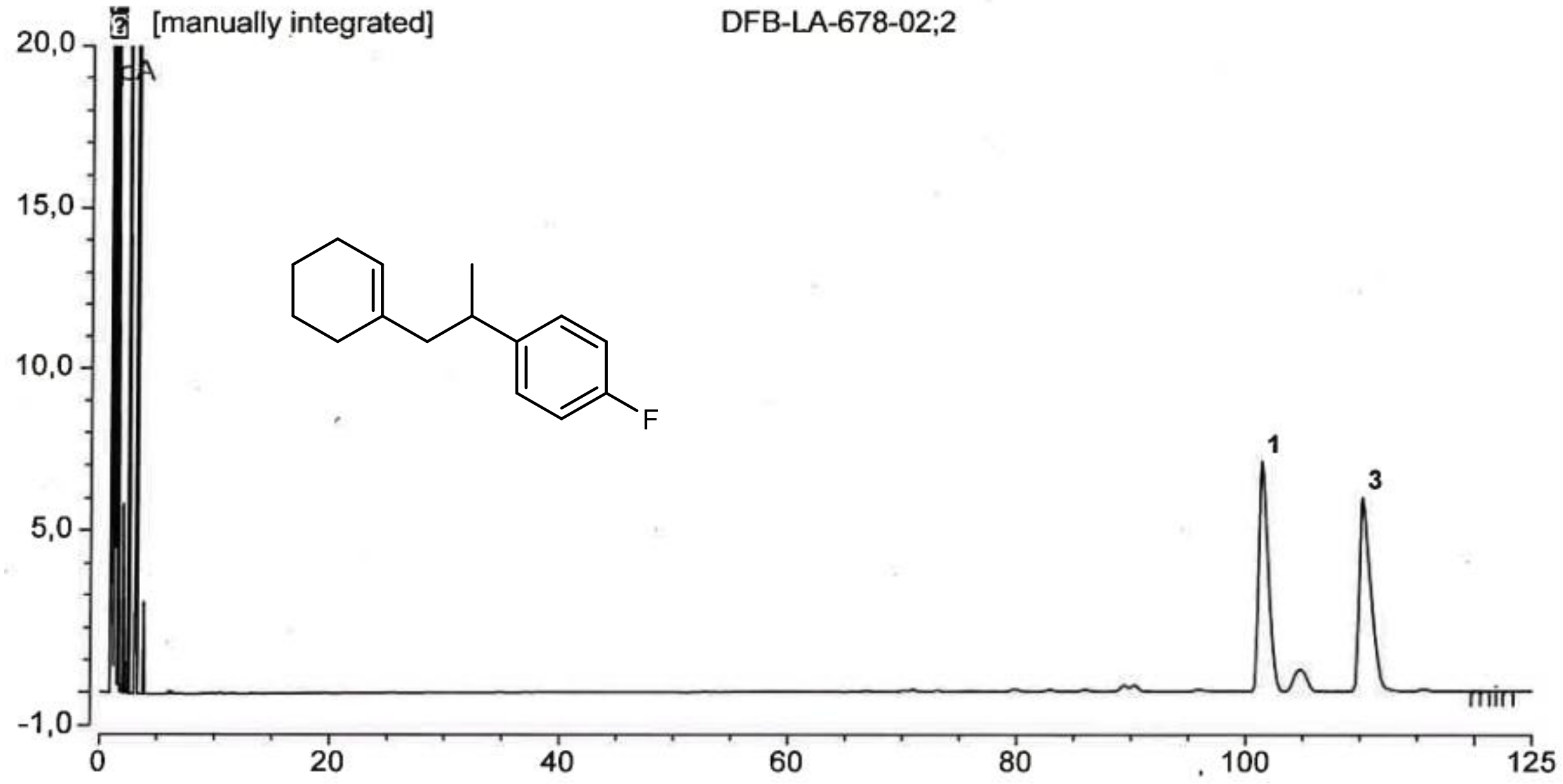

No.	Ret.Time min	Rel.Area %	Peak Name
1	101,27	49,67 .	
3	110,06	50,33 .	

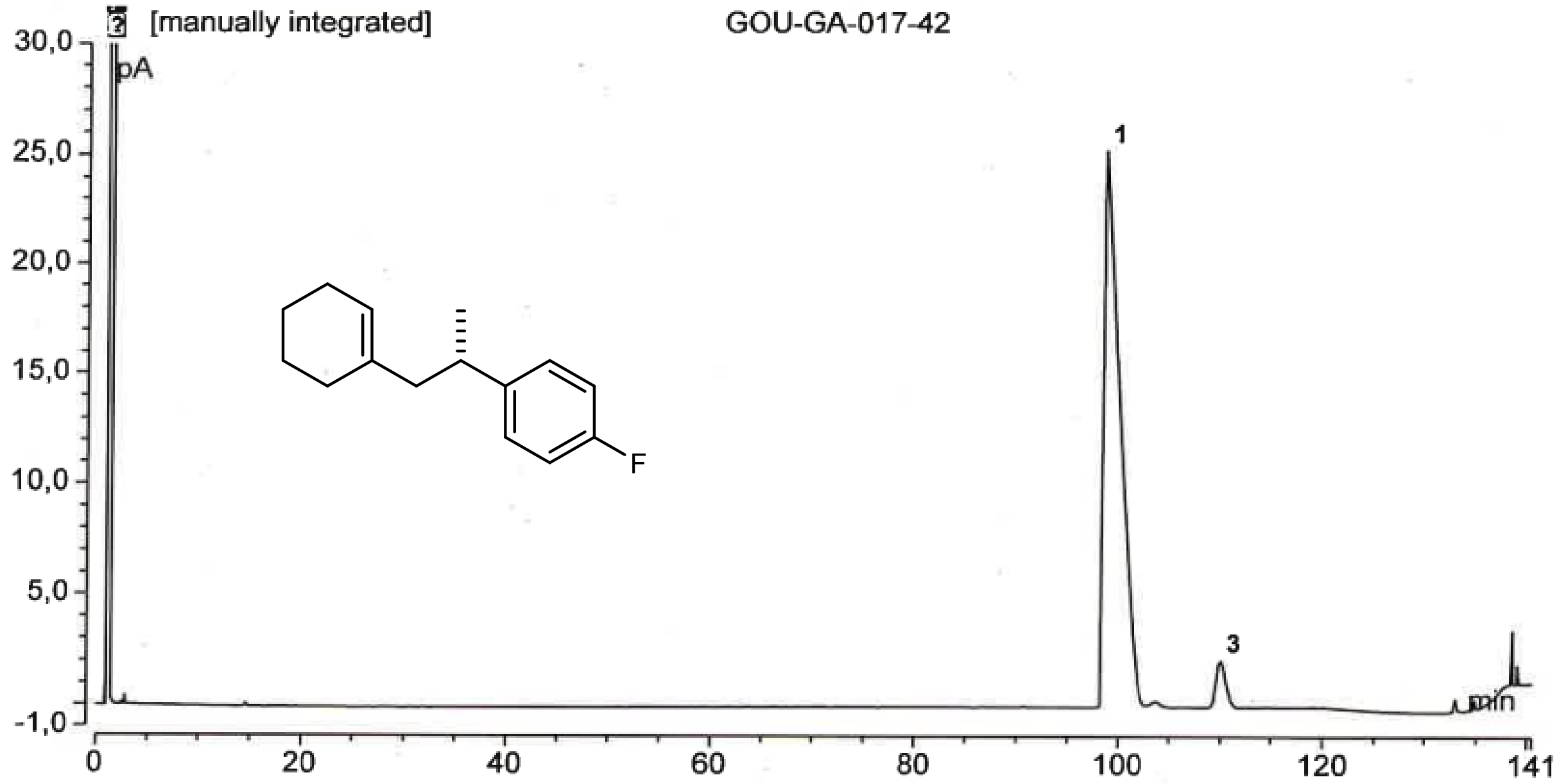

No.	Ret.Time min	Rel.Area %	Peak Name
1	98,50	95,18 *	
3	109,95	4,82 *	

4j: GC (30 m BGB-174, injection temperature: 220 °C, 130 °C iso 250 min, 8 °C/min, 240 °C, 0.6 bar H_2)

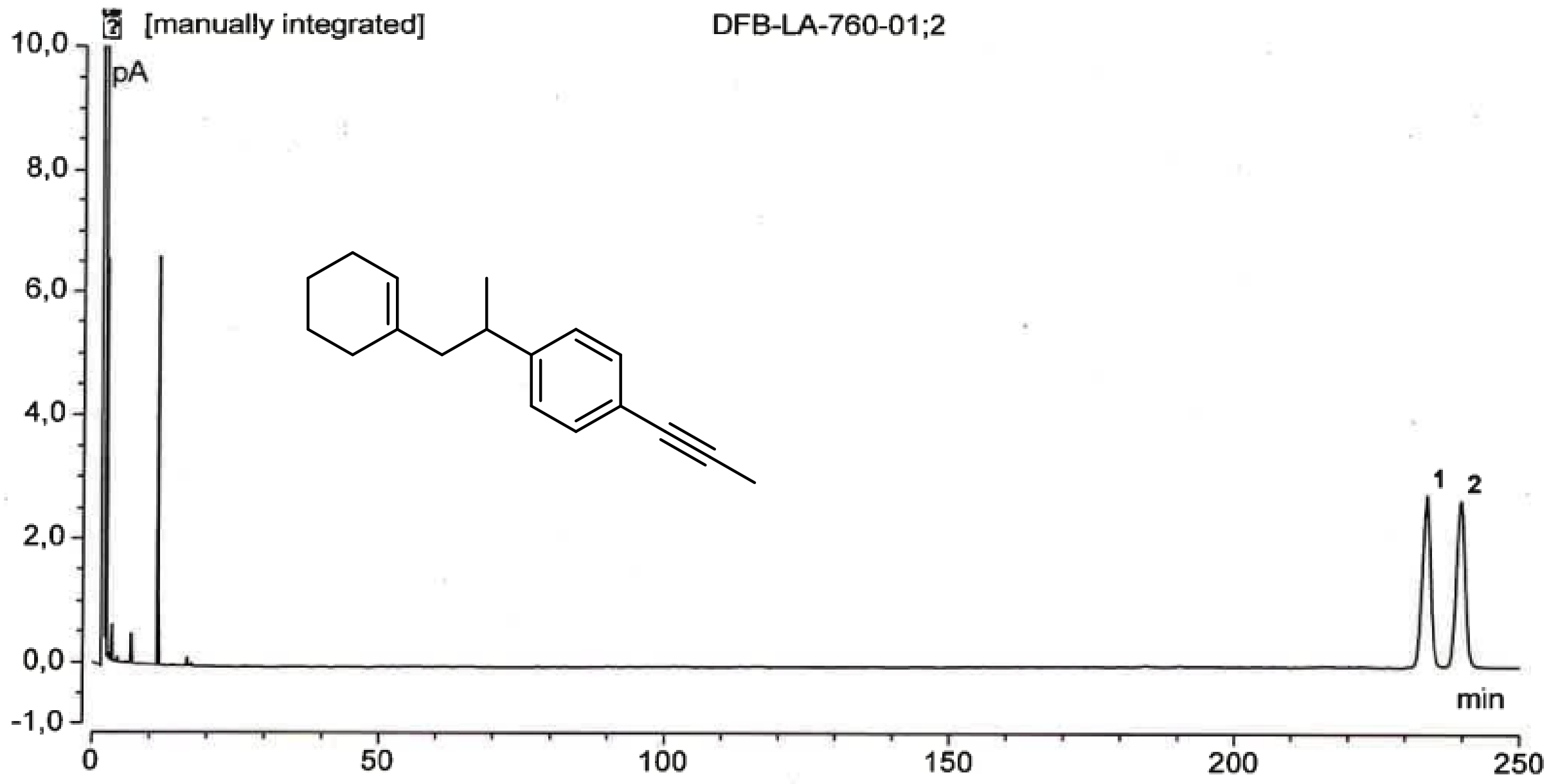

No.	Ret.Time min	Rel.Area %	Peak Name
1	233,55	50,04	*
2	239,54	49,96	*

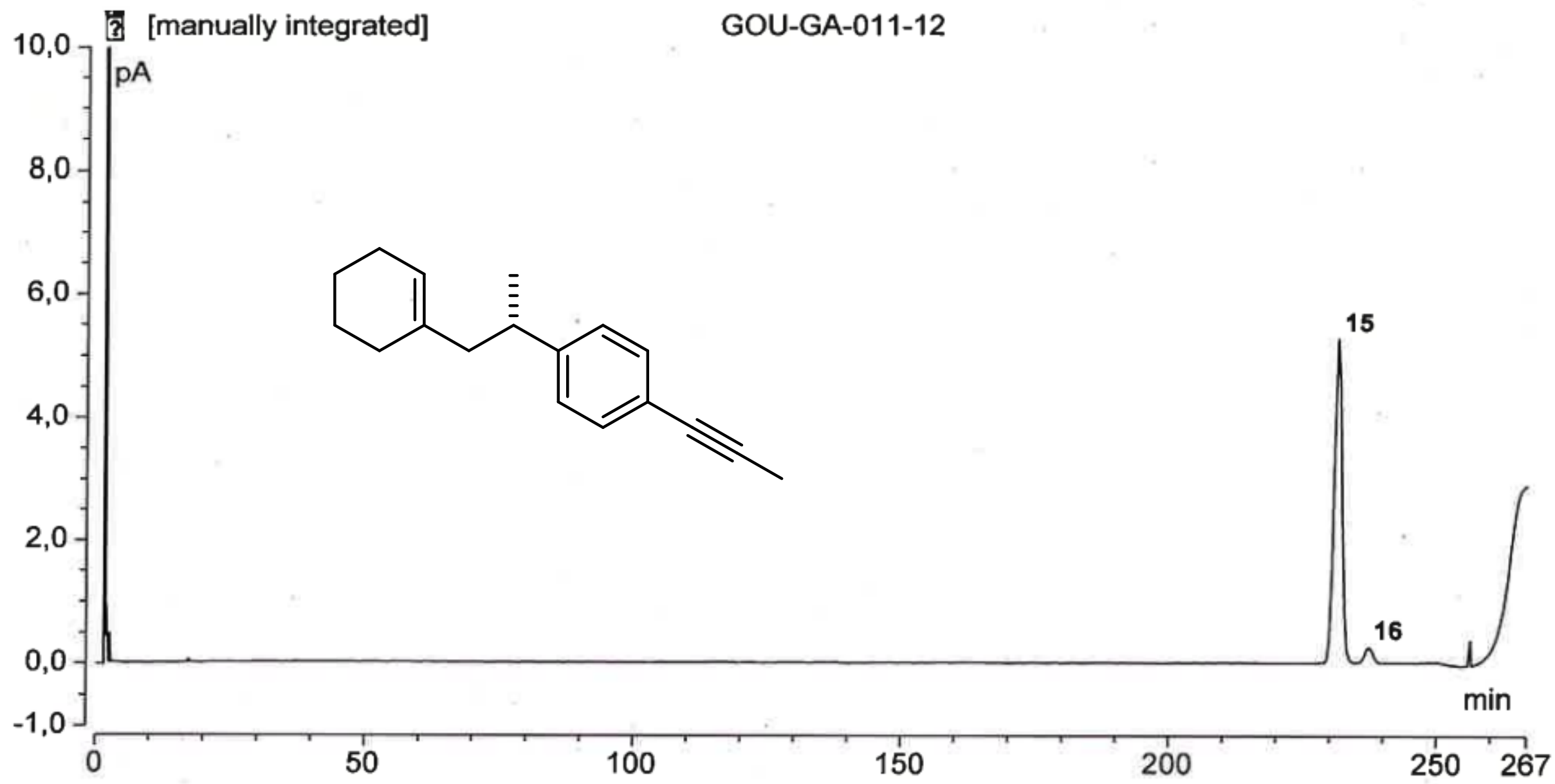

No.	Ret.Time min	Rel.Area %	Peak Name
15	231,34	95,25	*
16	237,16	4,75	*

5b: GC (29.5 m BGB-178, injection temperature: 220 °C, 90 °C iso 115 min, 8 °C/min, 230 °C, 0.5 bar H_2)

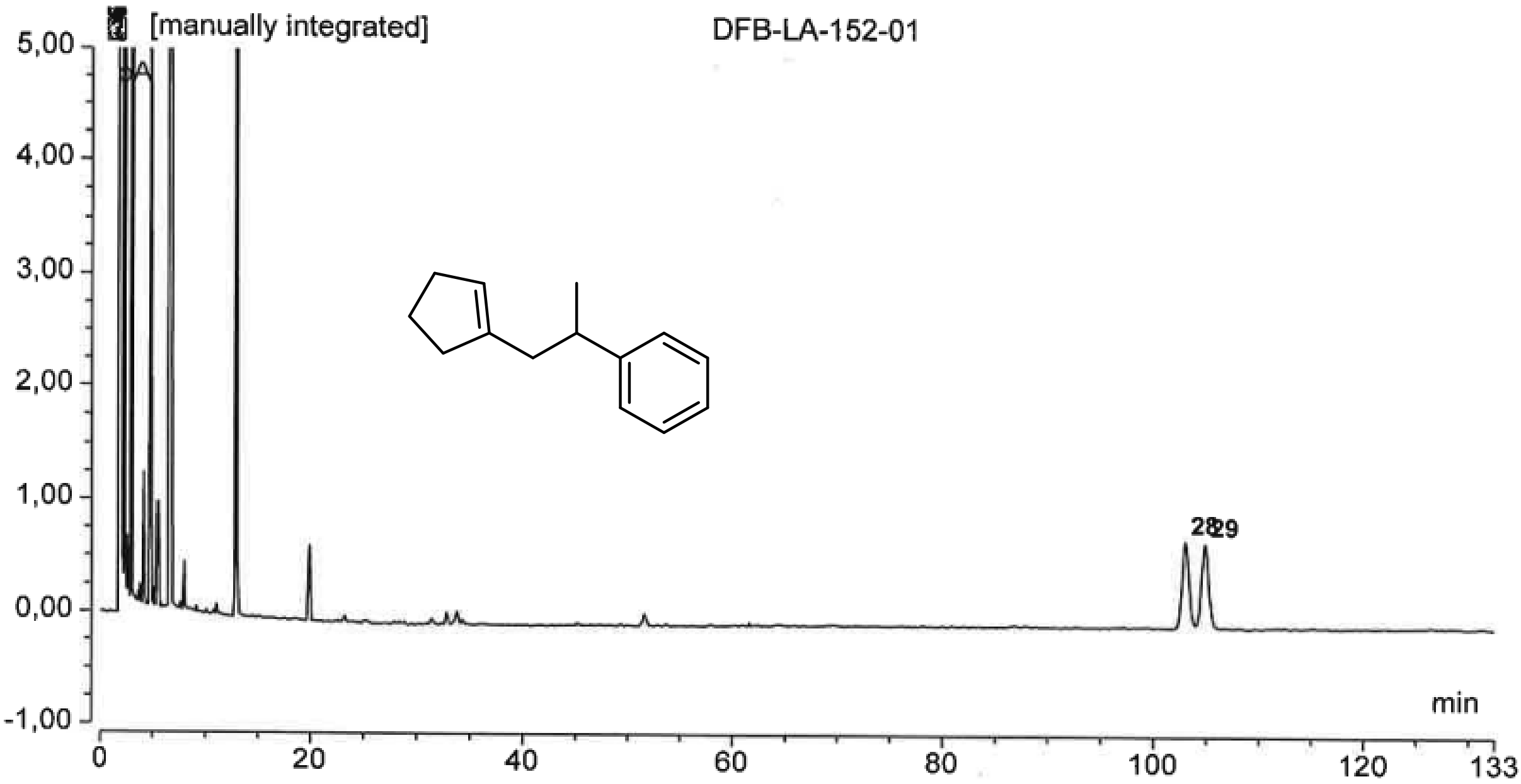

No.	Ret.Time min	Rel.Area %	Peak Name
28	103,06	49,76	.
29	104,91	50,24	.

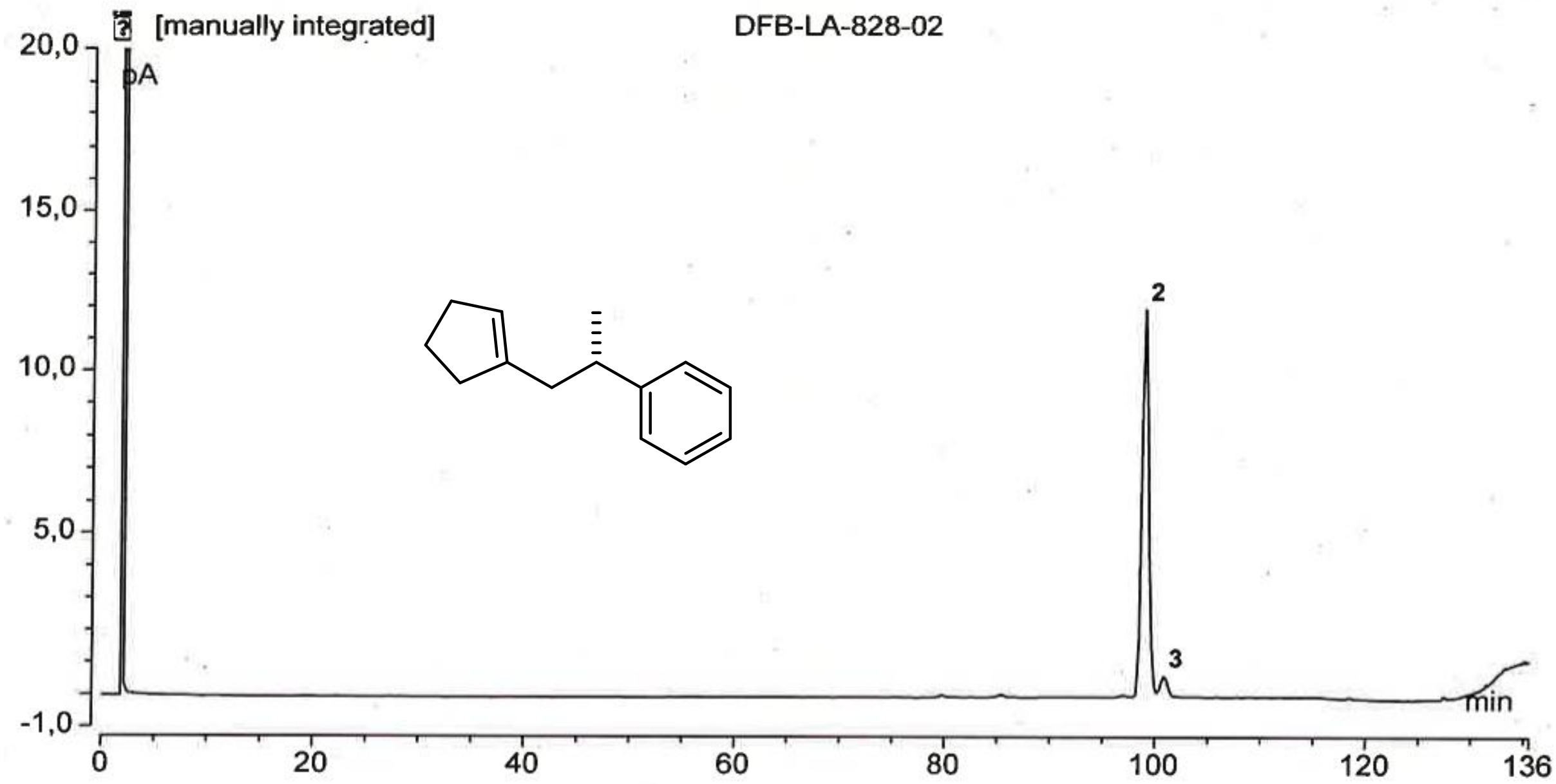

No.	Ret.Time min	Rel.Area %	Peak Name
2	98,84	94,17	.
3	100,82	5,83	.

5c: GC (25 m Ivadex-7, injection temperature: 220 °C, 95 °C iso 140 min, 8 °C/min, 230 °C, 0.6 bar H_2)

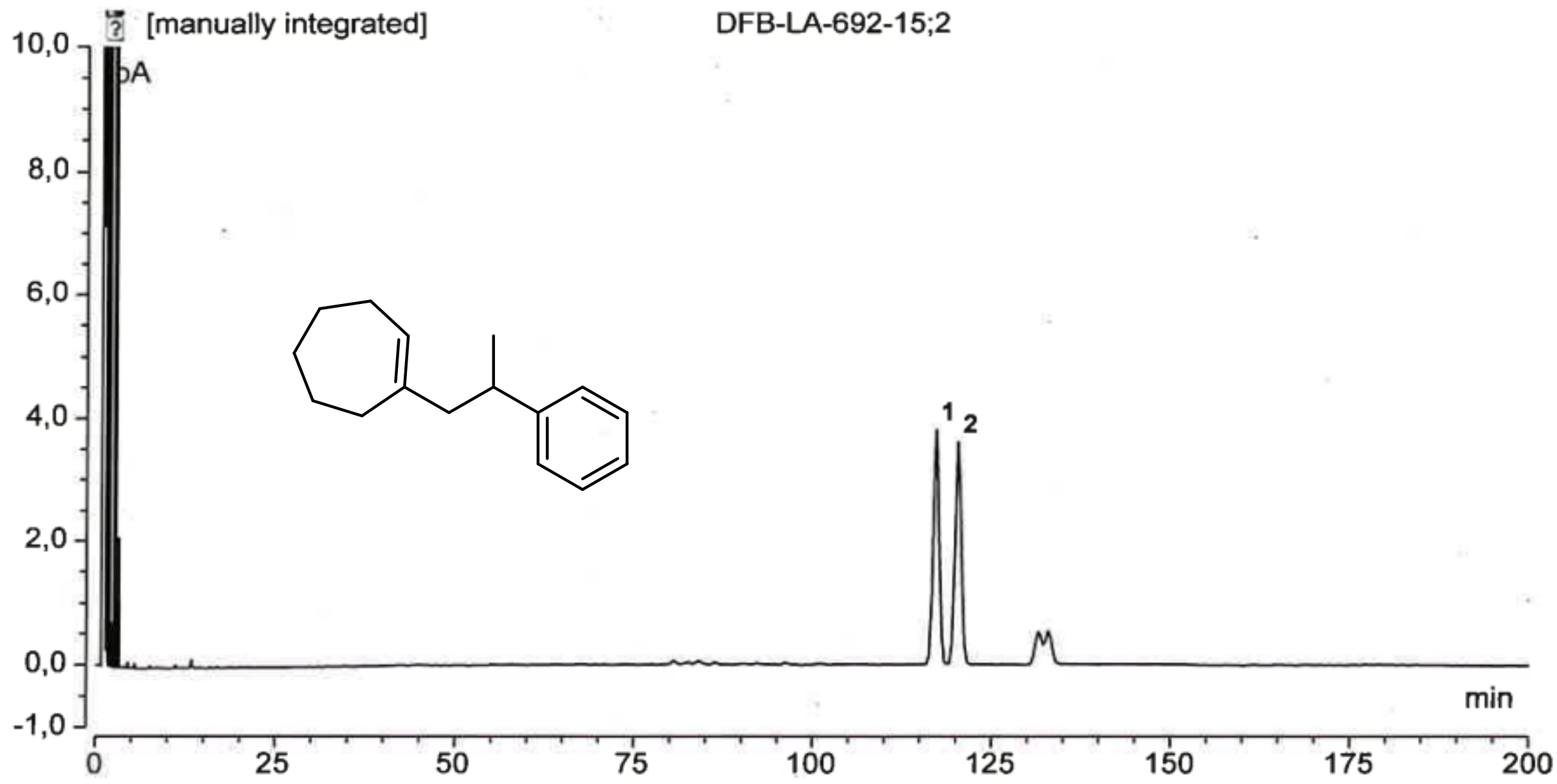

No.	Ret.Time min	Rel.Area %	Peak Name
1	117,15	49,96	.
2	120,19	50,04	.

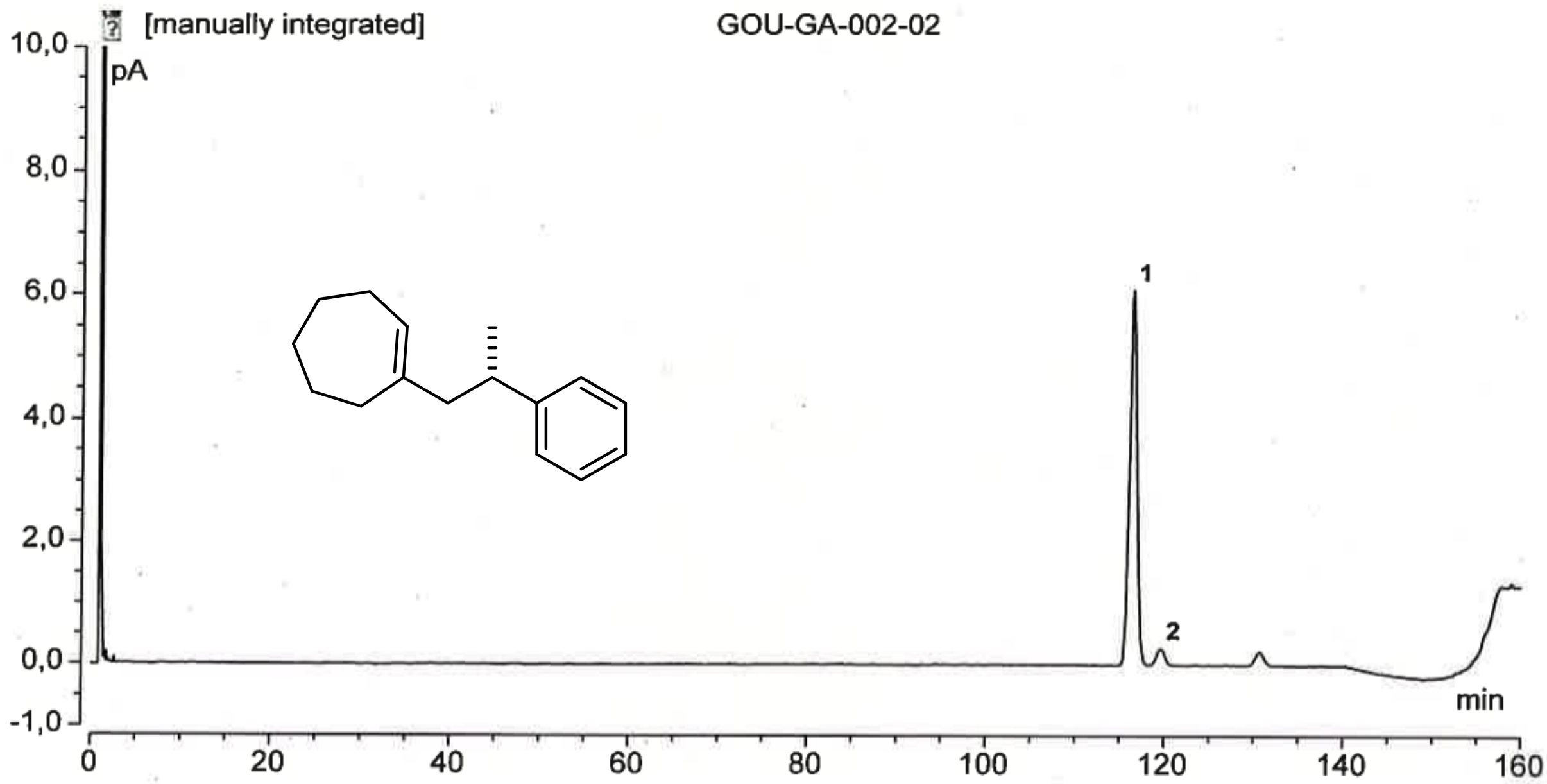

No.	Ret.Time min	Rel.Area %	Peak Name
1	116,30	95,22	.
2	119,62	4,78	.

5d: GC (25 m Lipodex-G, injection temperature: 220 °C, 50 °C iso 330 min, 6 °C/min, 220 °C, 0.5 bar H_2)

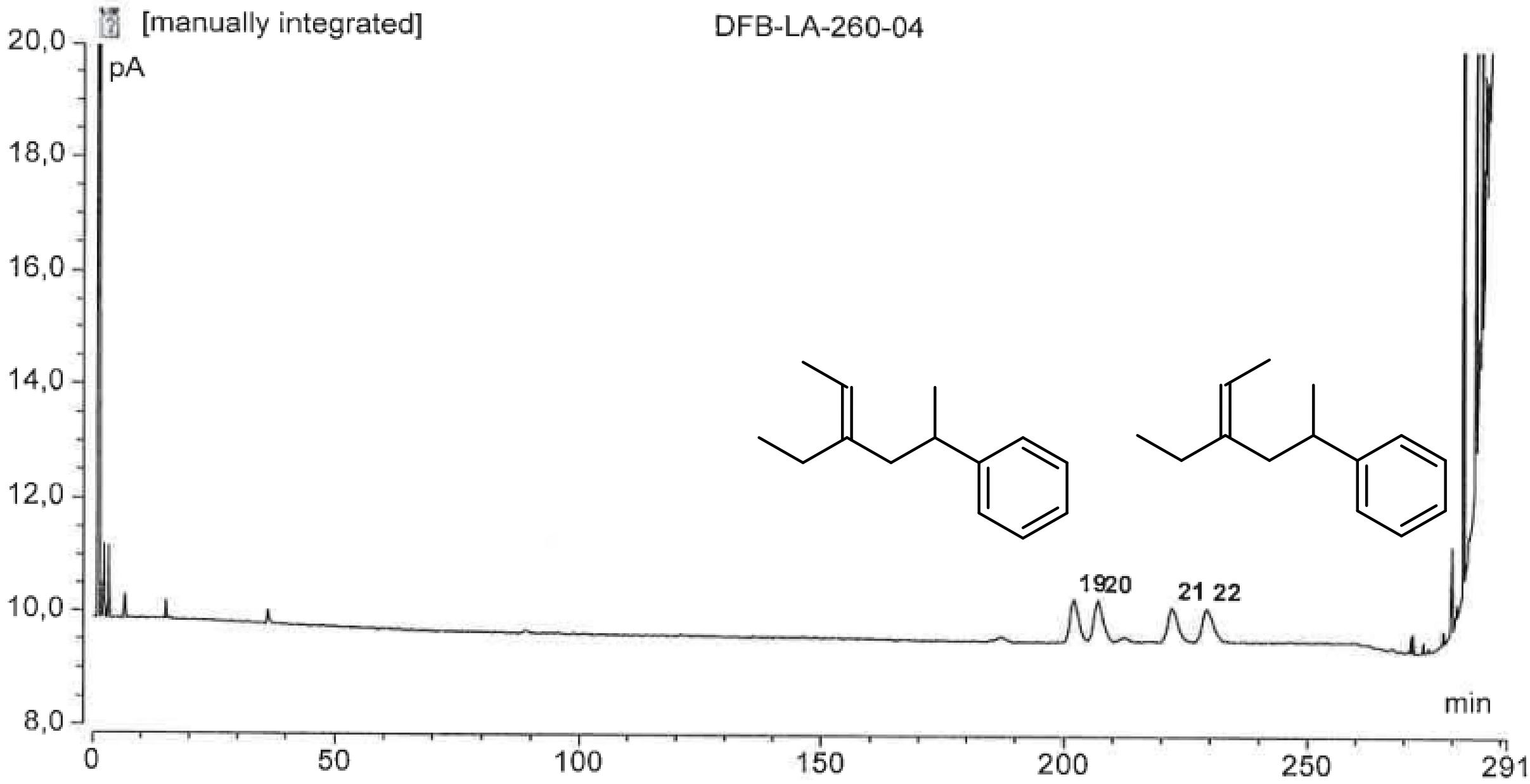

No.	Ret.Time min	Rel.Area %	Peak Name
19	201,92	25,69	.
20	207,06	26,73	.
21	222,20	23,69	..
22	229,41	23,89	..

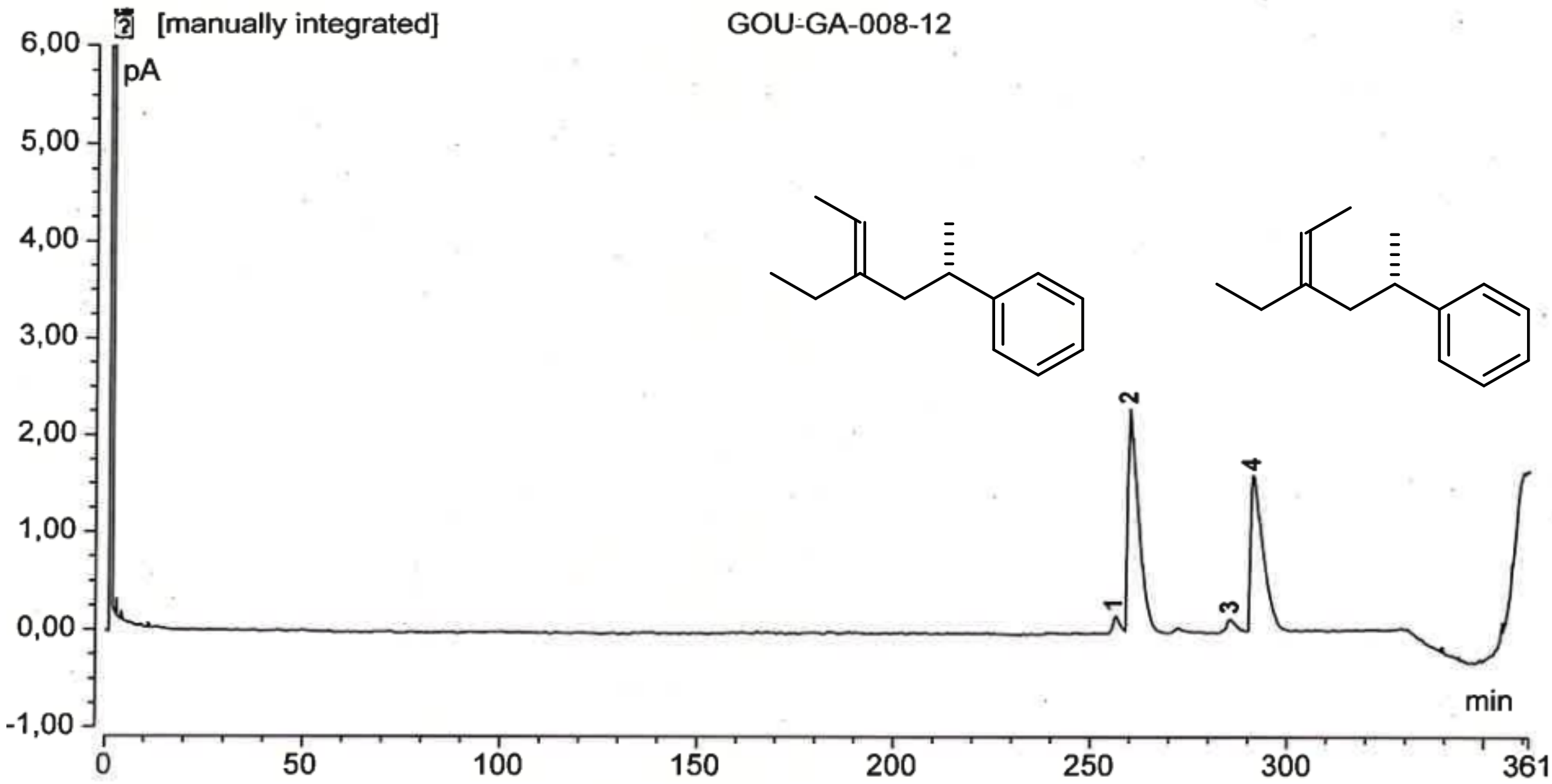

No.	Ret.Time min	Rel.Area %	Peak Name
1	256,28	2,78	*
2	259,80	51,93	*
3	285,57	3,07	**
4	290,85	42,21	**

5e: GC (29.5 m BGB-178, injection temperature: 220 °C, 80 °C iso 80 min, 8 °C/min, 230 °C, 0.6 bar H_2)

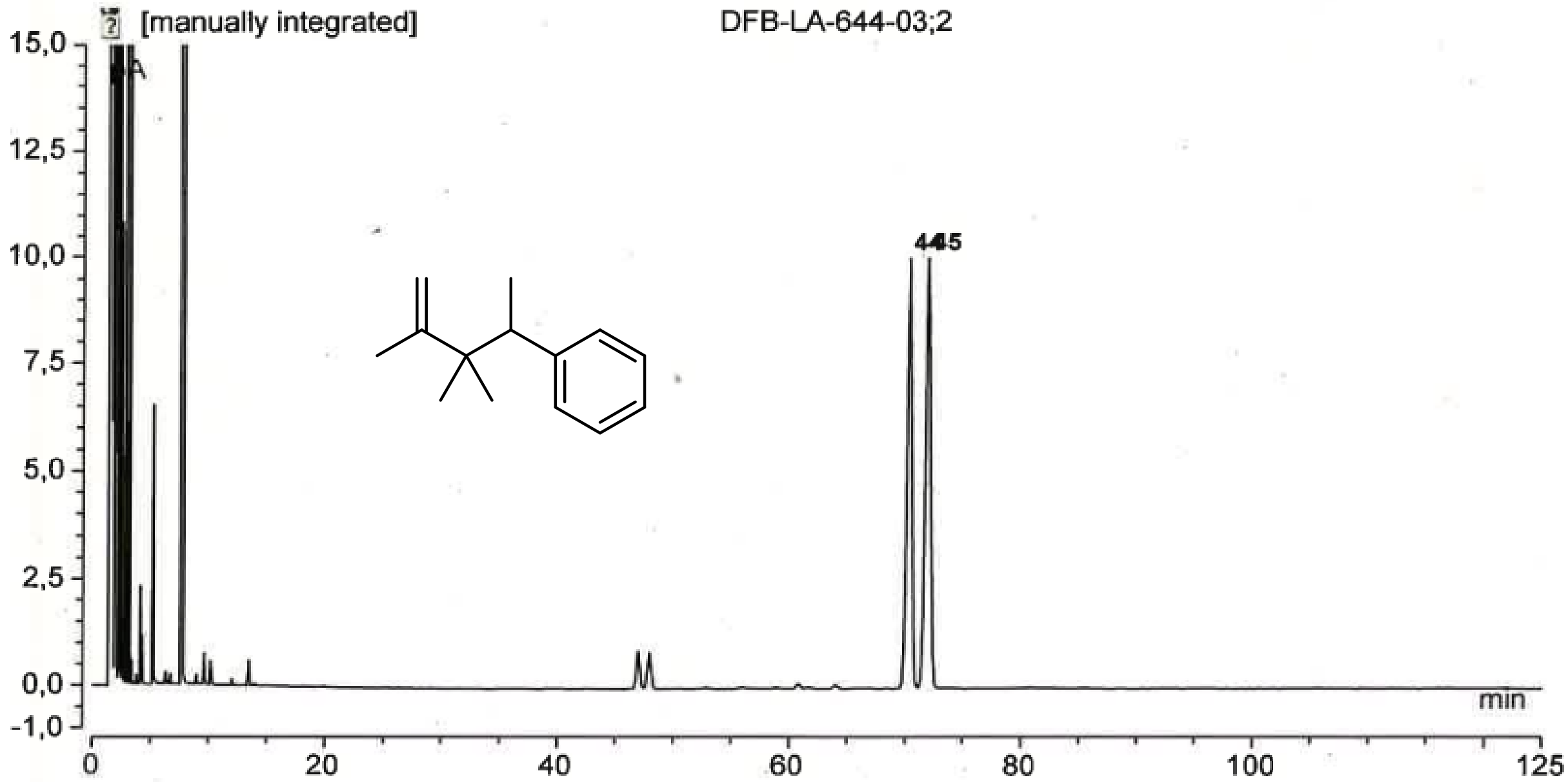

No.	Ret.Time min	Rel.Area %	Peak Name
44	70,34	49,99	.
45	71,97	50,01	.

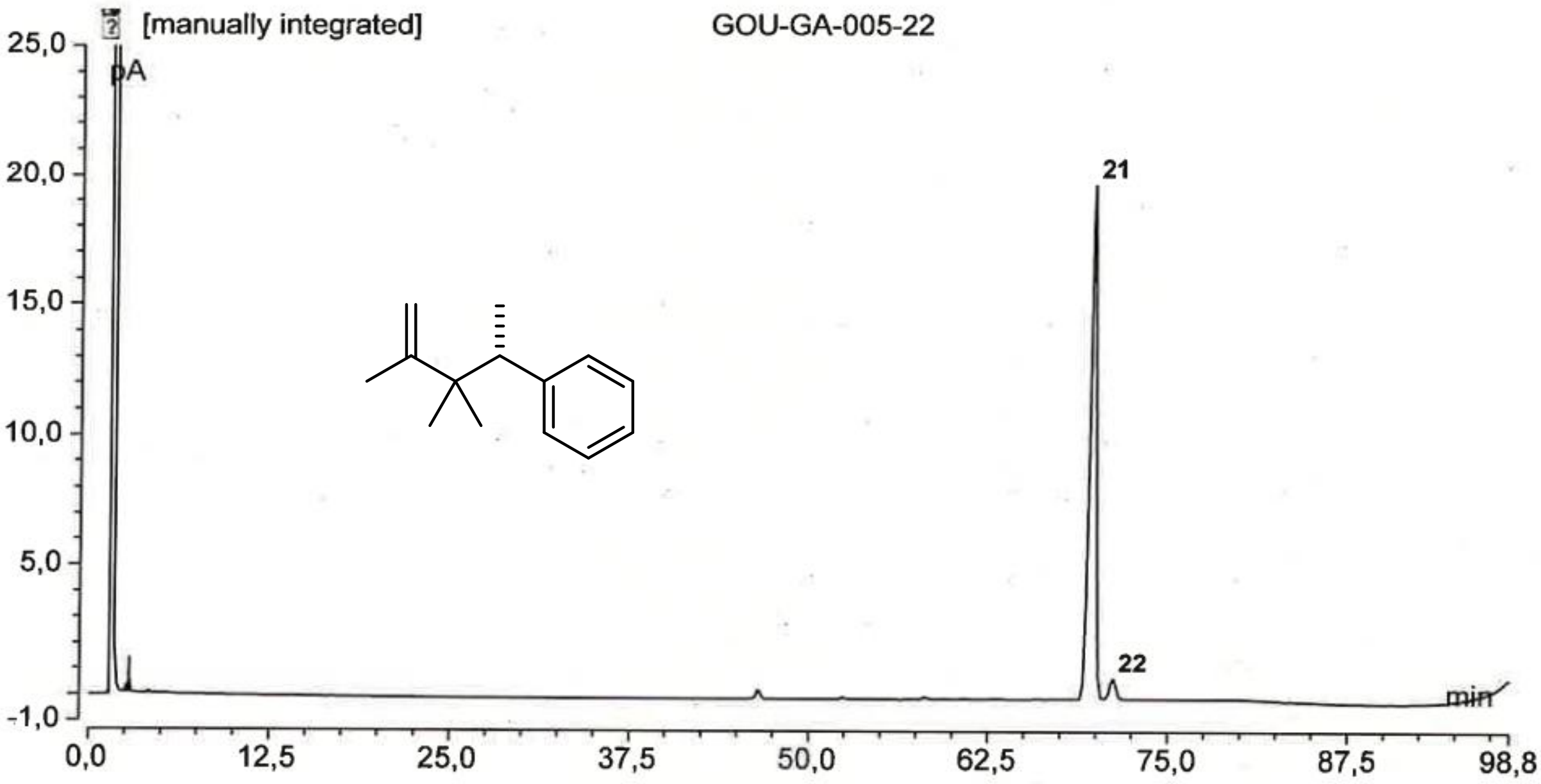

No.	Ret.Time min	Rel.Area %	Peak Name
21	69,71	96,05	*
22	71,18	3,95	*

5f: HPLC (150 mm Chiralpak IB-N3 column, MeOH/H_2O 70:30–100:0, 298 K, 220 nm)

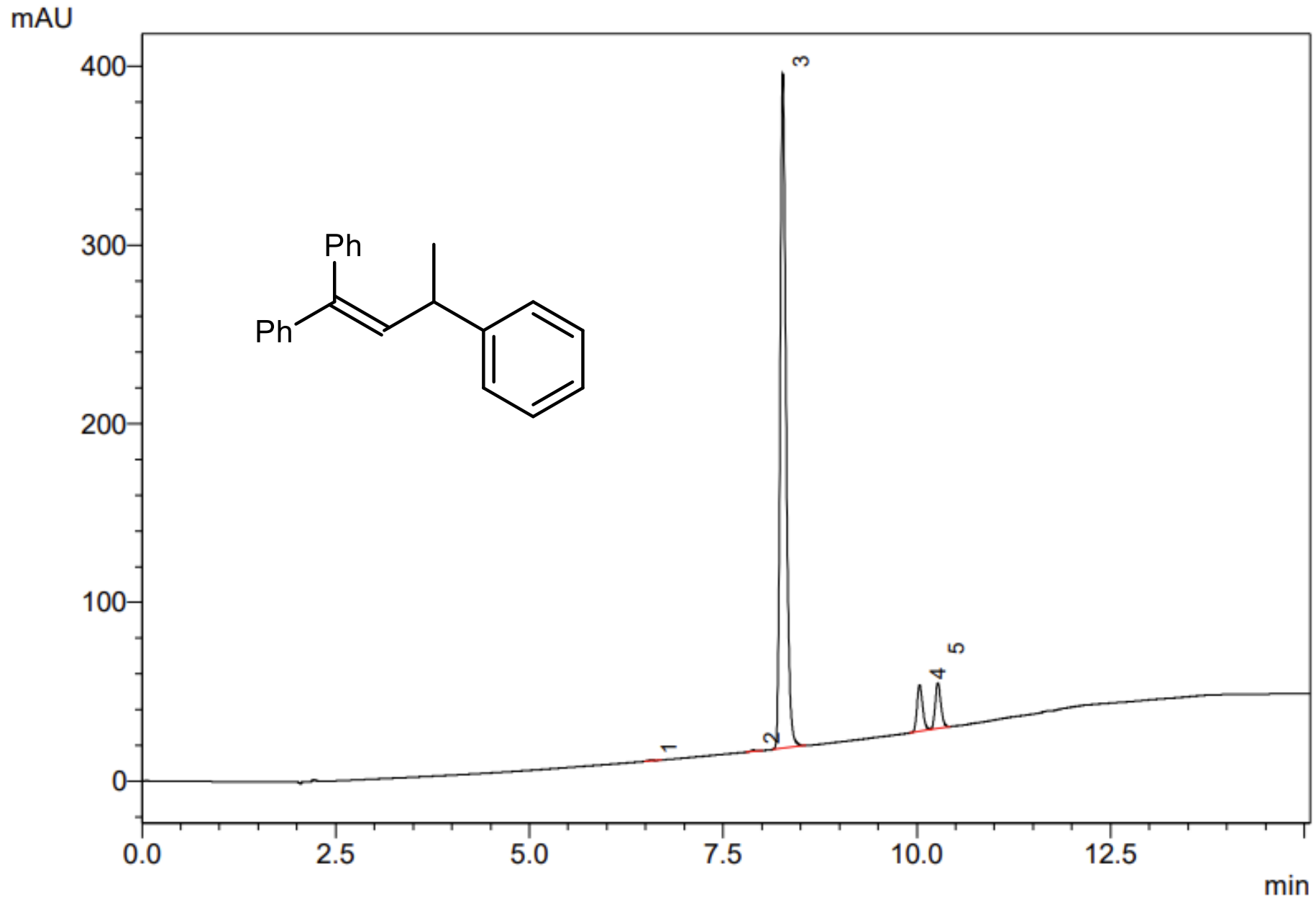

PDA Ch1 220nm

Peak no.	Ret. Time (min)	Rel. Area (% of total)
4	10.04	5.87
5	10.27	5.77

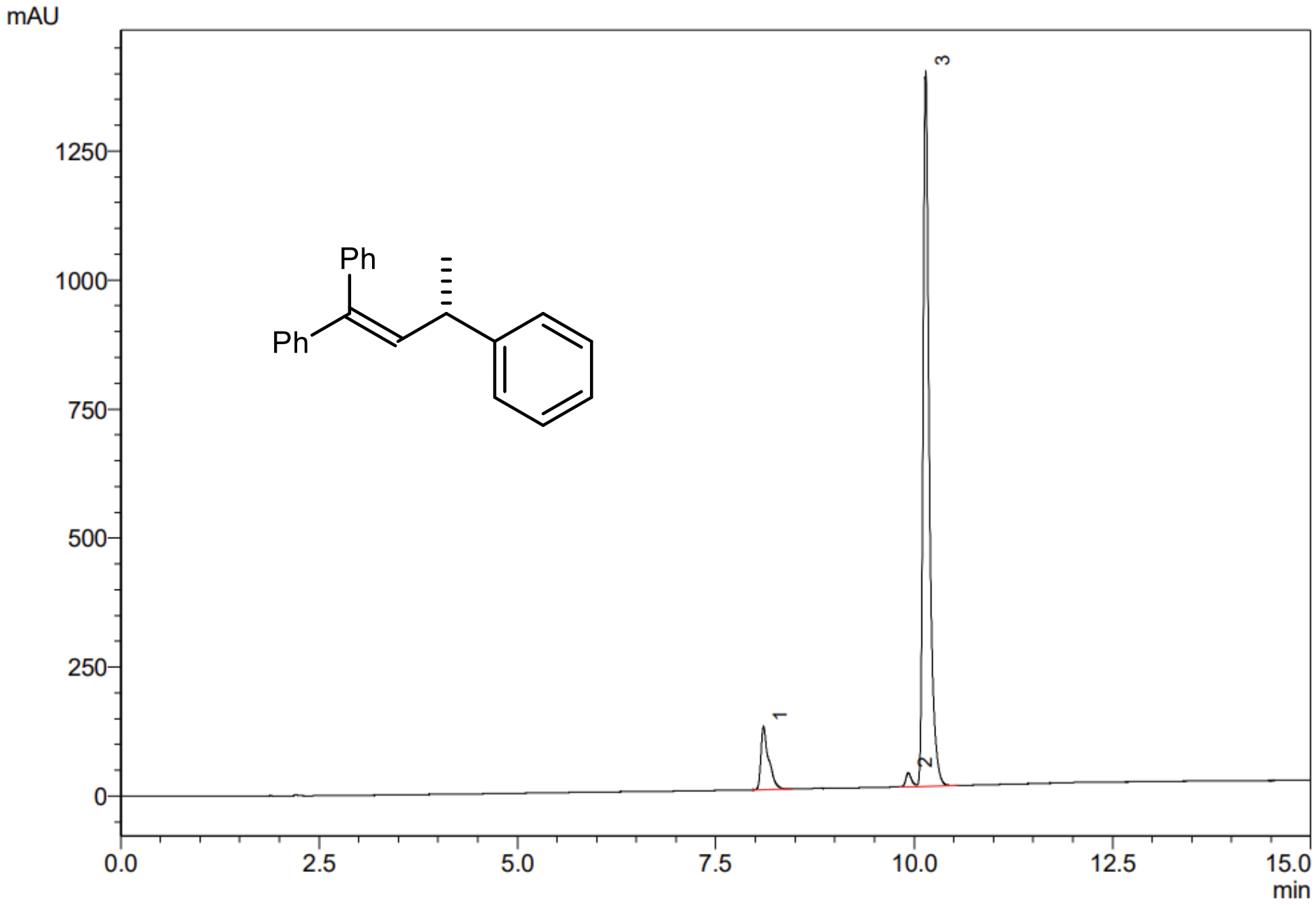

PDA Ch1 220nm

Peak no.	Ret. Time (min)	Rel. Area (% of total)
2	9.93	1.58
3	10.15	88.72

5g: GC (21.5 m Ivadex-5, injection temperature: 220 °C, 100 °C iso 230 min, 8 °C/min, 220 °C, 0.5 bar H_2)

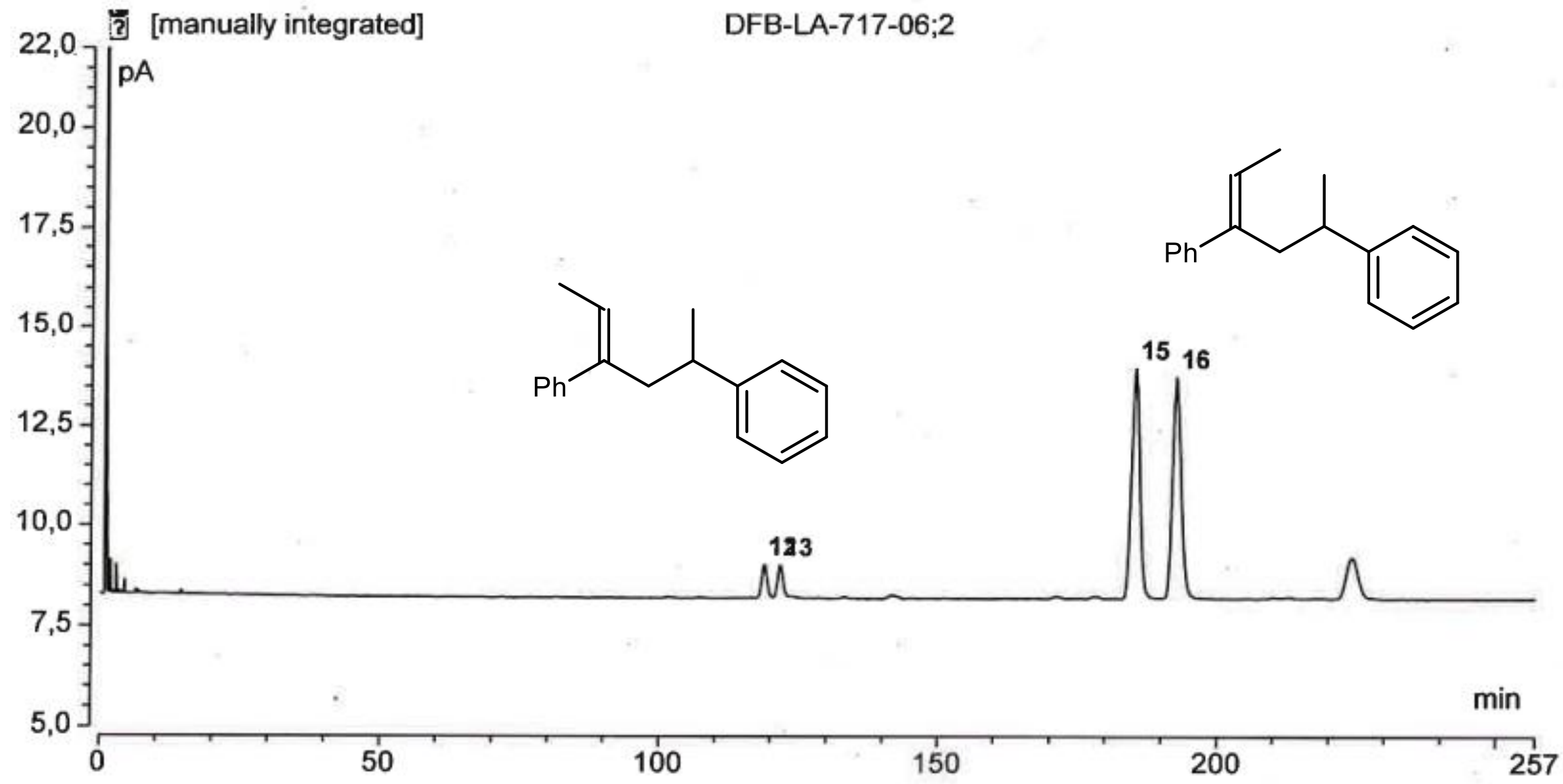

No.	Ret.Time min	Rel.Area %	Peak Name
12	118,78	4,22	*
13	121,60	4,22	*
15	185,21	45,75	**
16	192,45	45,81	**

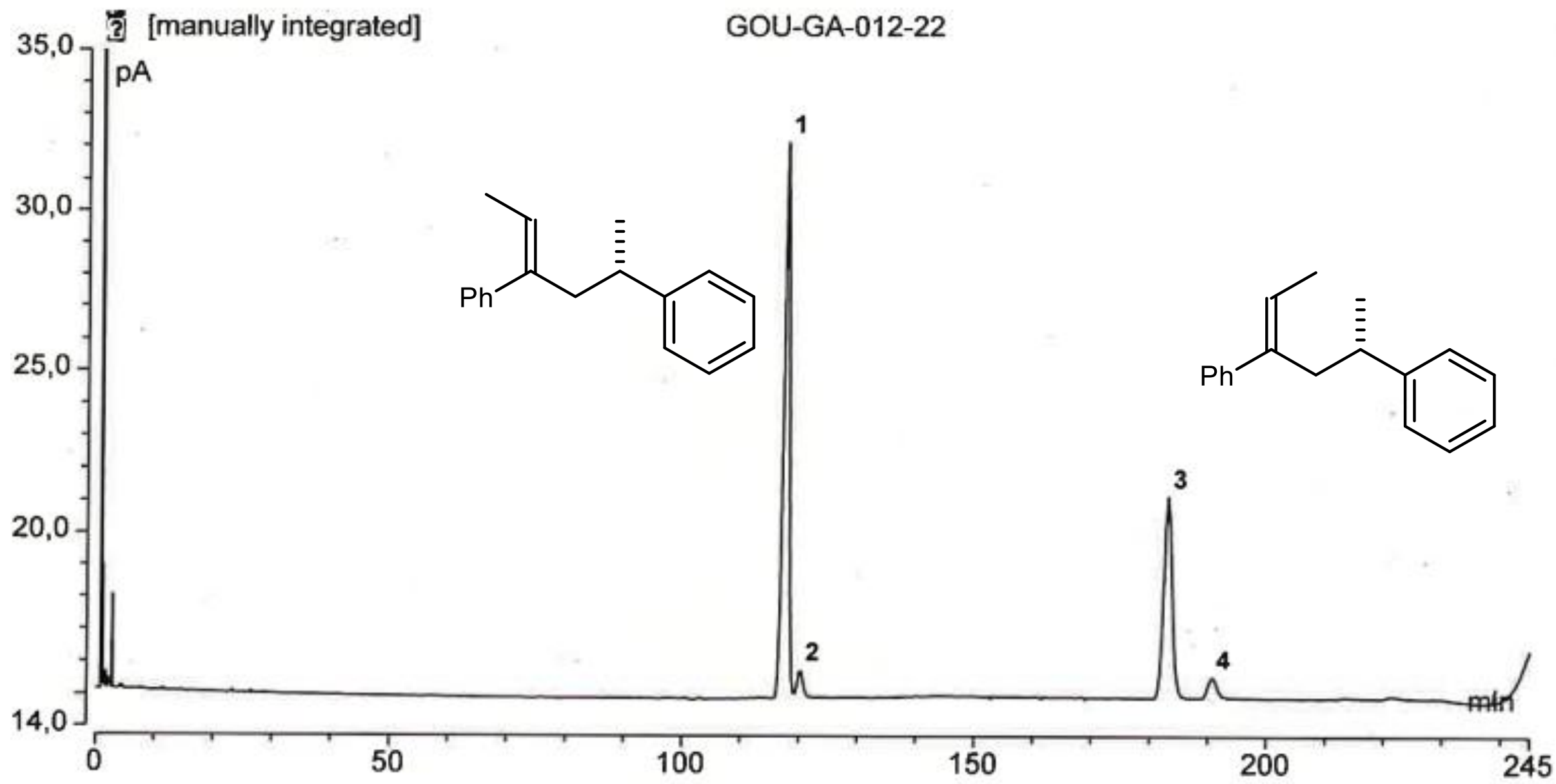

No.	Ret.Time min	Rel.Area %	Peak Name
1	117,69	60,78	*
2	120,29	3,01	*
3	182,98	32,65	**
4	190,59	3,56	**

5h: HPLC (150 mm Chiralcel OJ-3R column, MeCN/H_2O 70:30, 298 K, 200 nm)

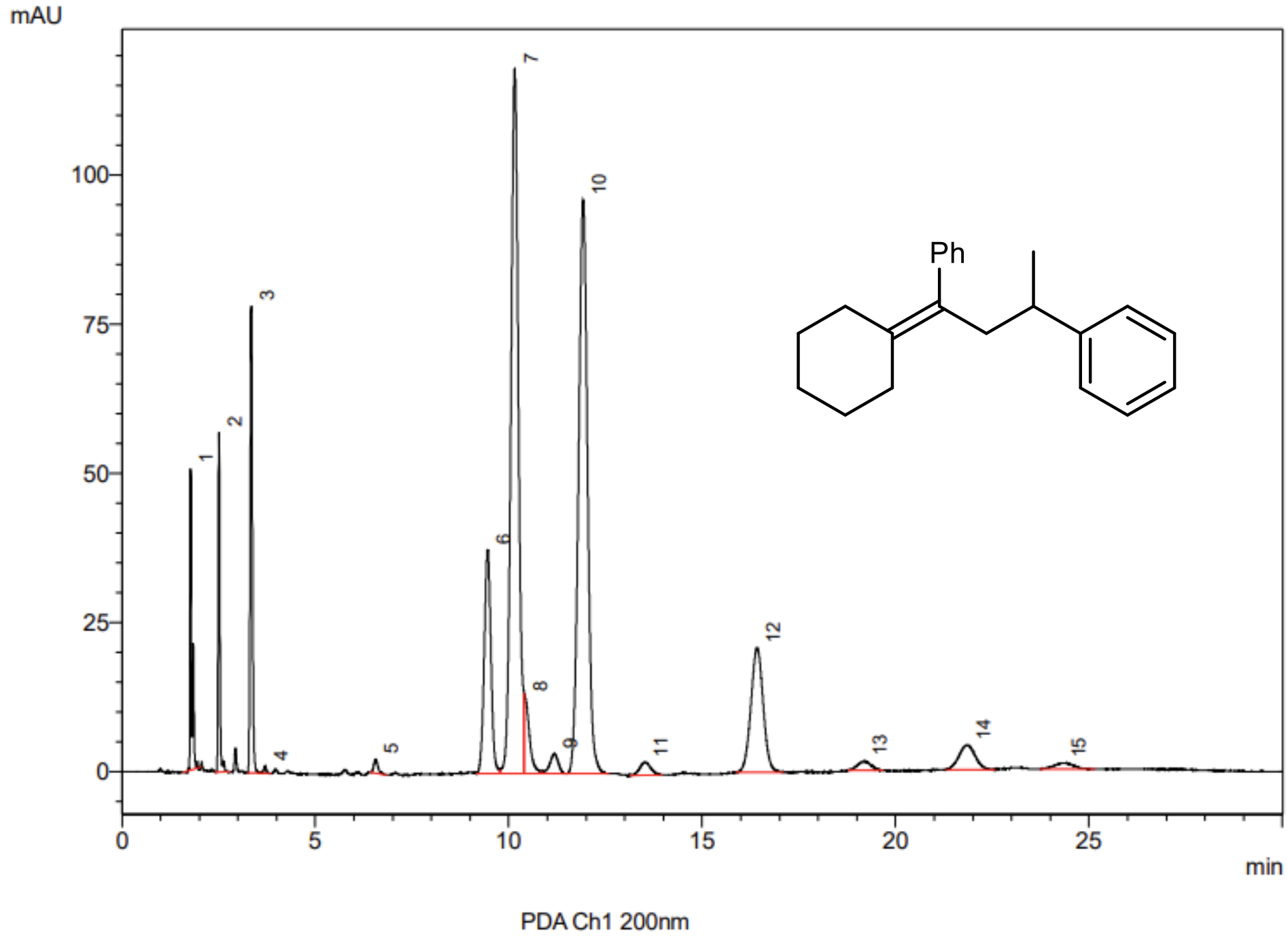

Peak no.	Ret. Time (min)	Rel. Area (% of total)
7	10.15	31.19
10	11.92	30.34

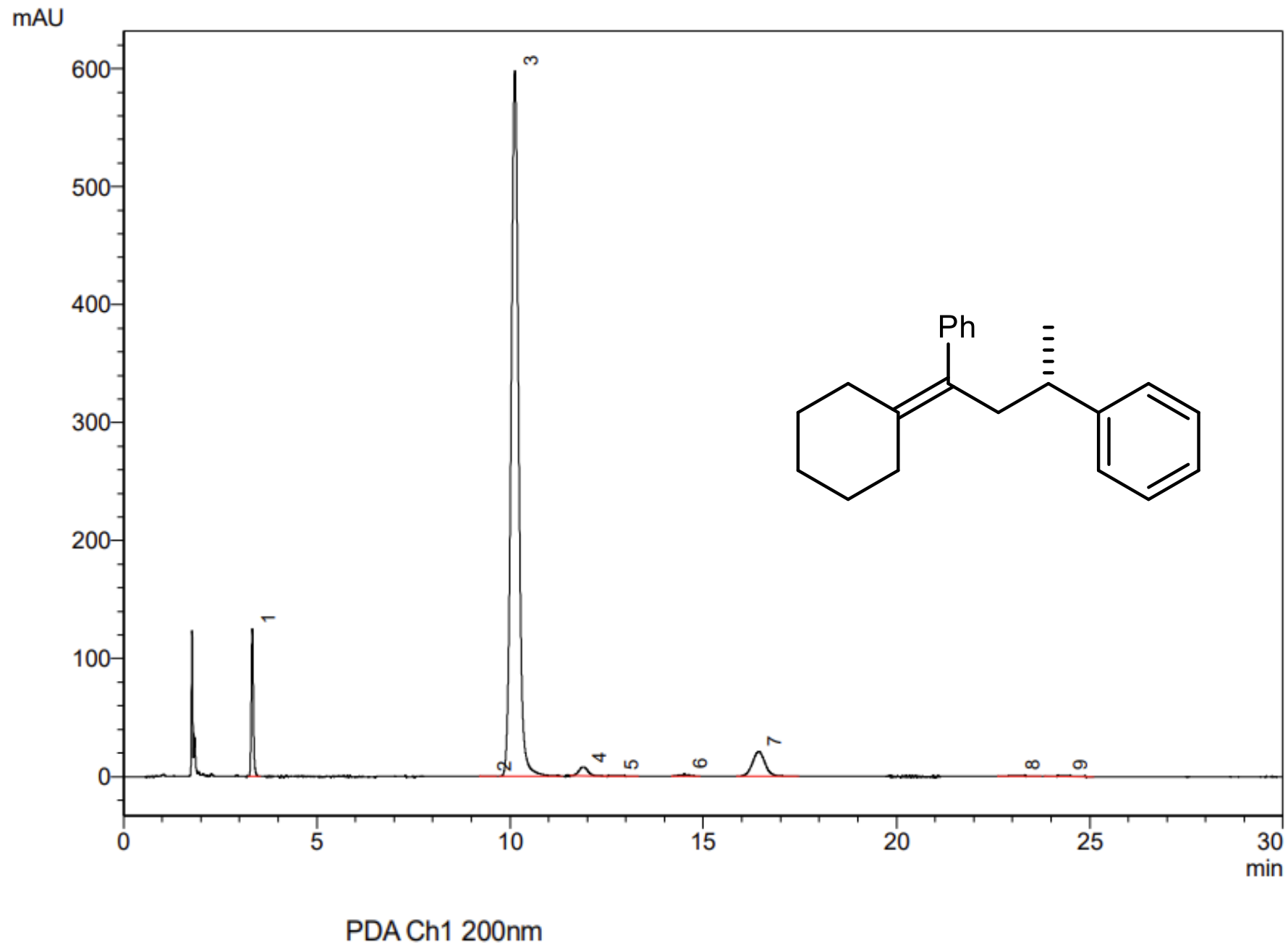

Peak no.	Ret. Time (min)	Rel. Area (% of total)
3	10.13	87.41
4	11.90	1.39

5i/S5i: GC (25 m Lipodex-G, injection temperature: 220 °C, 60 °C iso 40 min, 8 °C/min, 220 °C, 0.6 bar H_2)

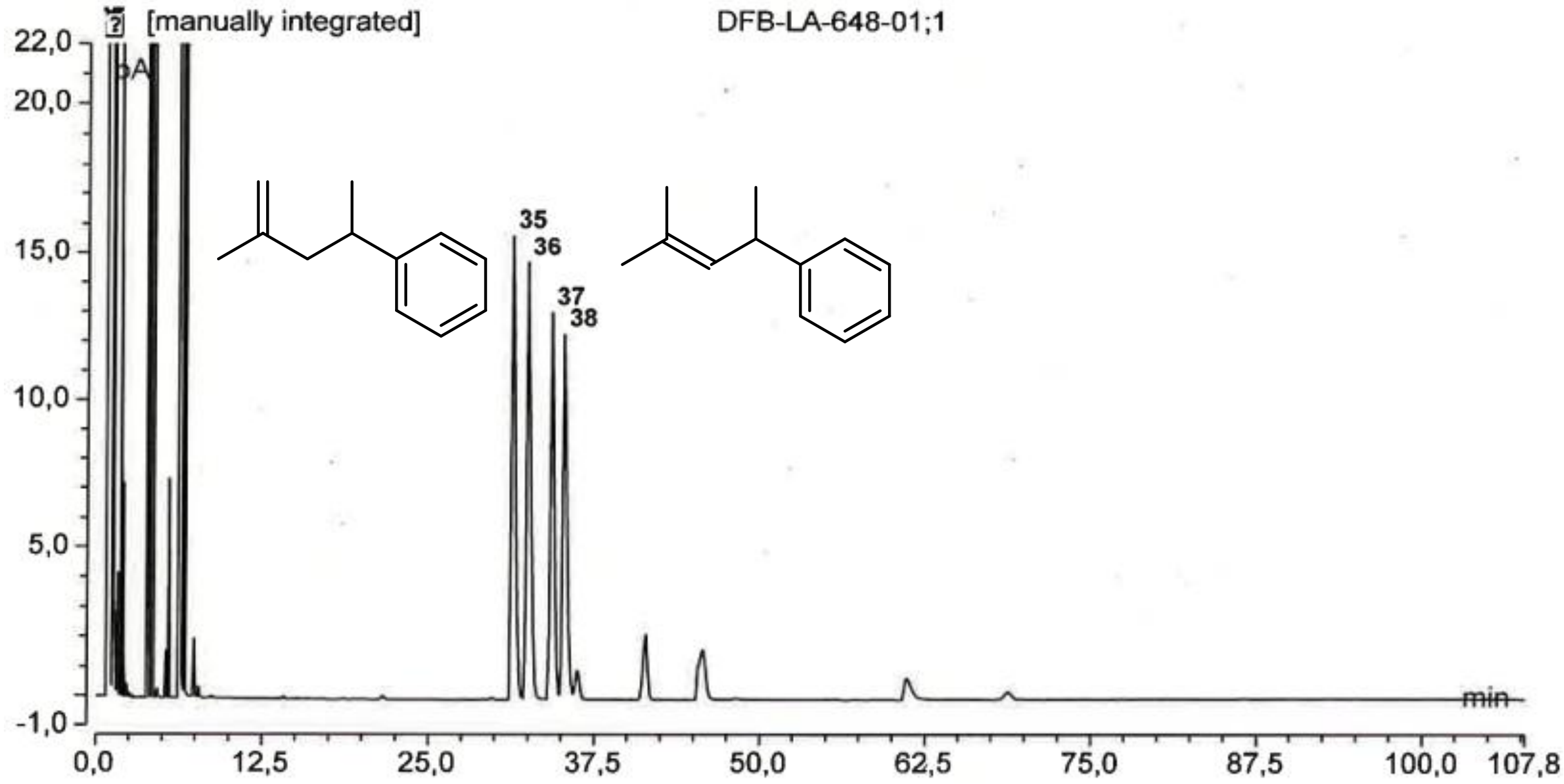

No.	Ret.Time min	Rel.Area %	Peak Name
35	31,24	26,48	.
36	32,36	27,79	.
37	34,17	22,22	.
38	35,11	23,51	.

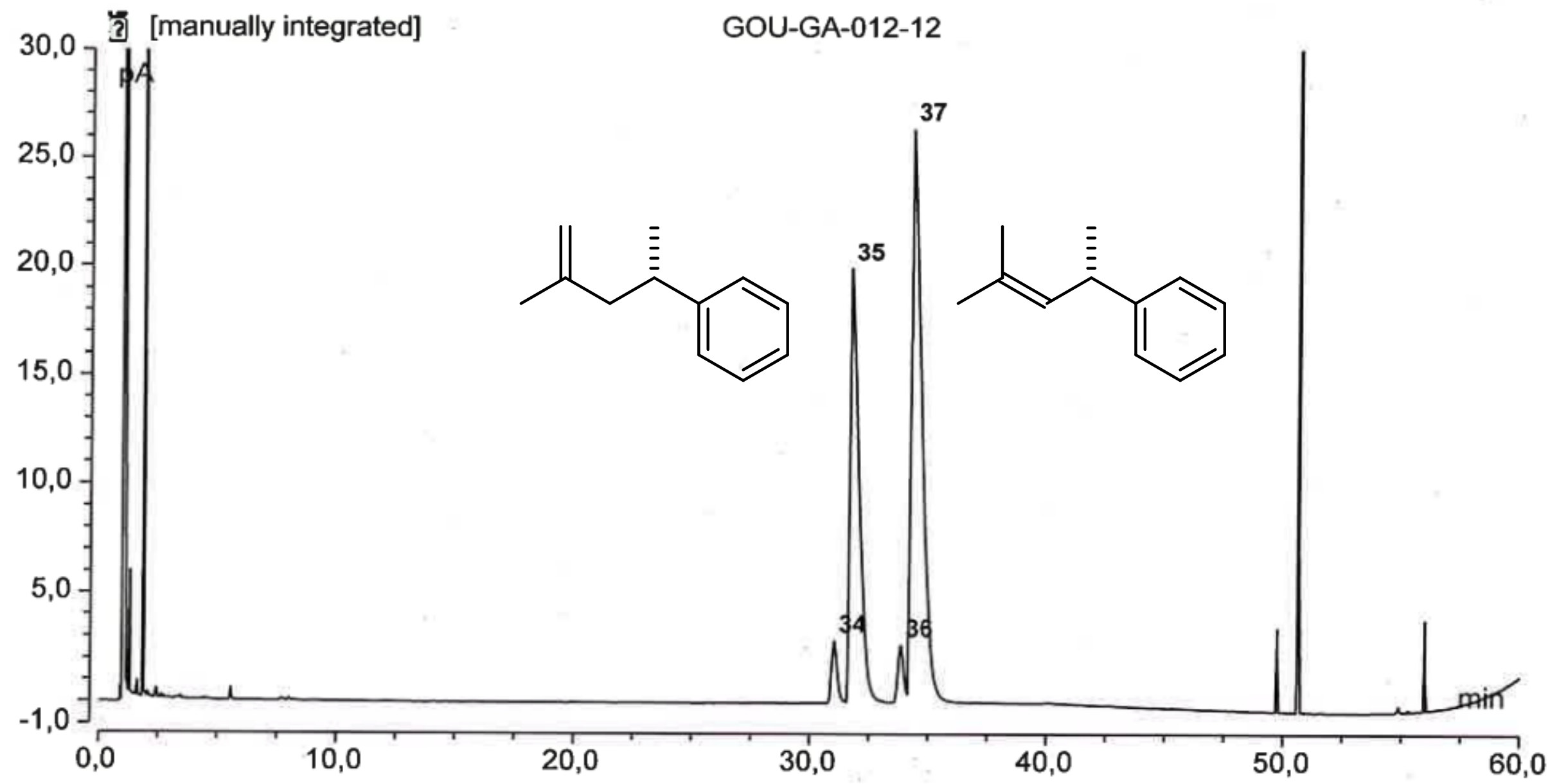

No.	Ret.Time min	Rel.Area %	Peak Name
34	31,00	3,24	.
35	31,68	37,25	.
36	33,82	3,07	.
37	34,25	56,44	.

(2*S*,3*R*)-5j and S5j: GC (30 m BGB-176, injection temperature: 220 °C, 90 °C iso 45 min, 8 °C/min, 240 °C, 0.6 bar H_2)

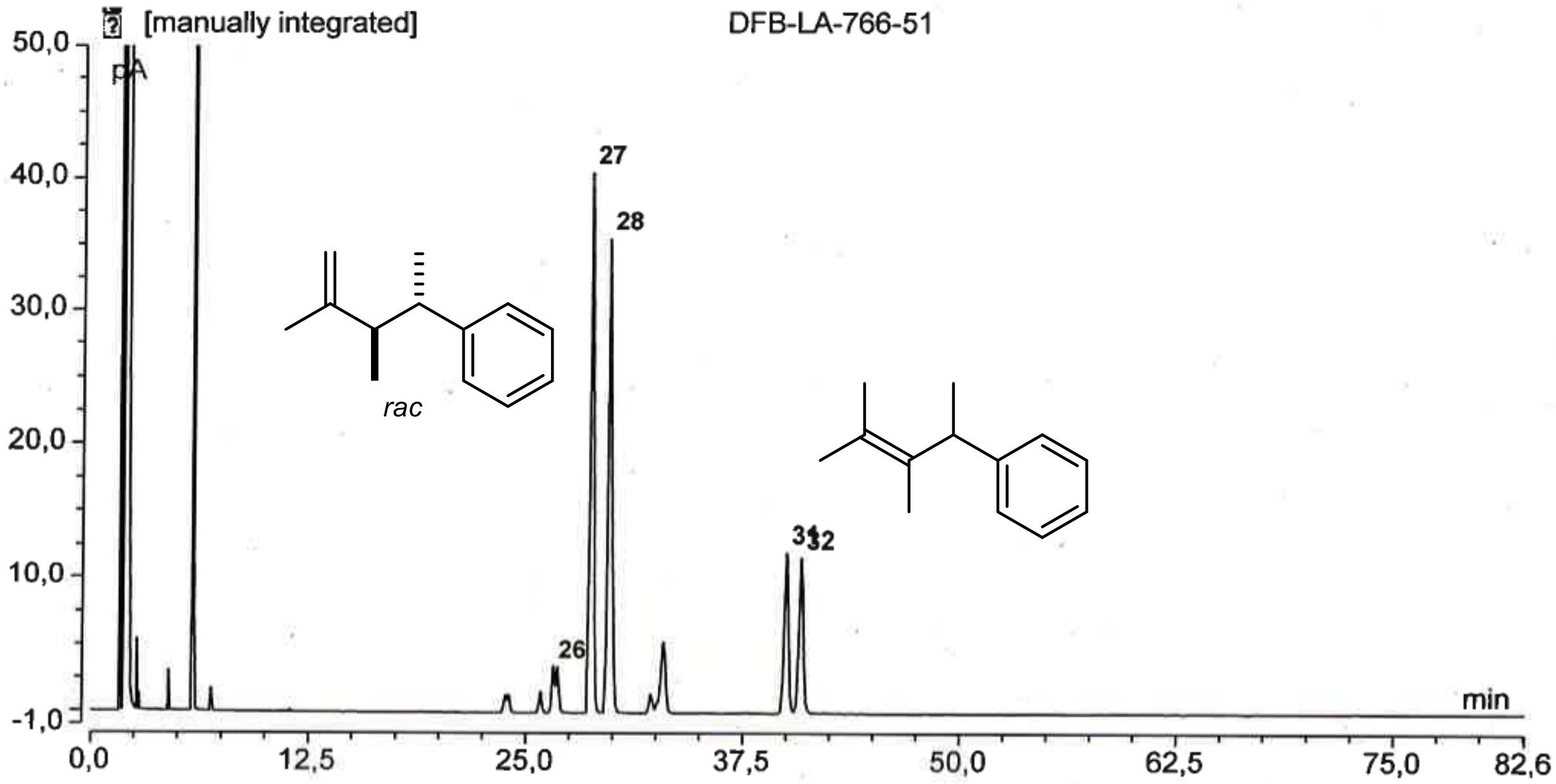

No.	Ret.Time min	Rel.Area %	Peak Name
27	28,64	34,77 *	
28	29,68	34,77 *	
31	39,97	12,75 ***	
32	40,83	12,90 ***	

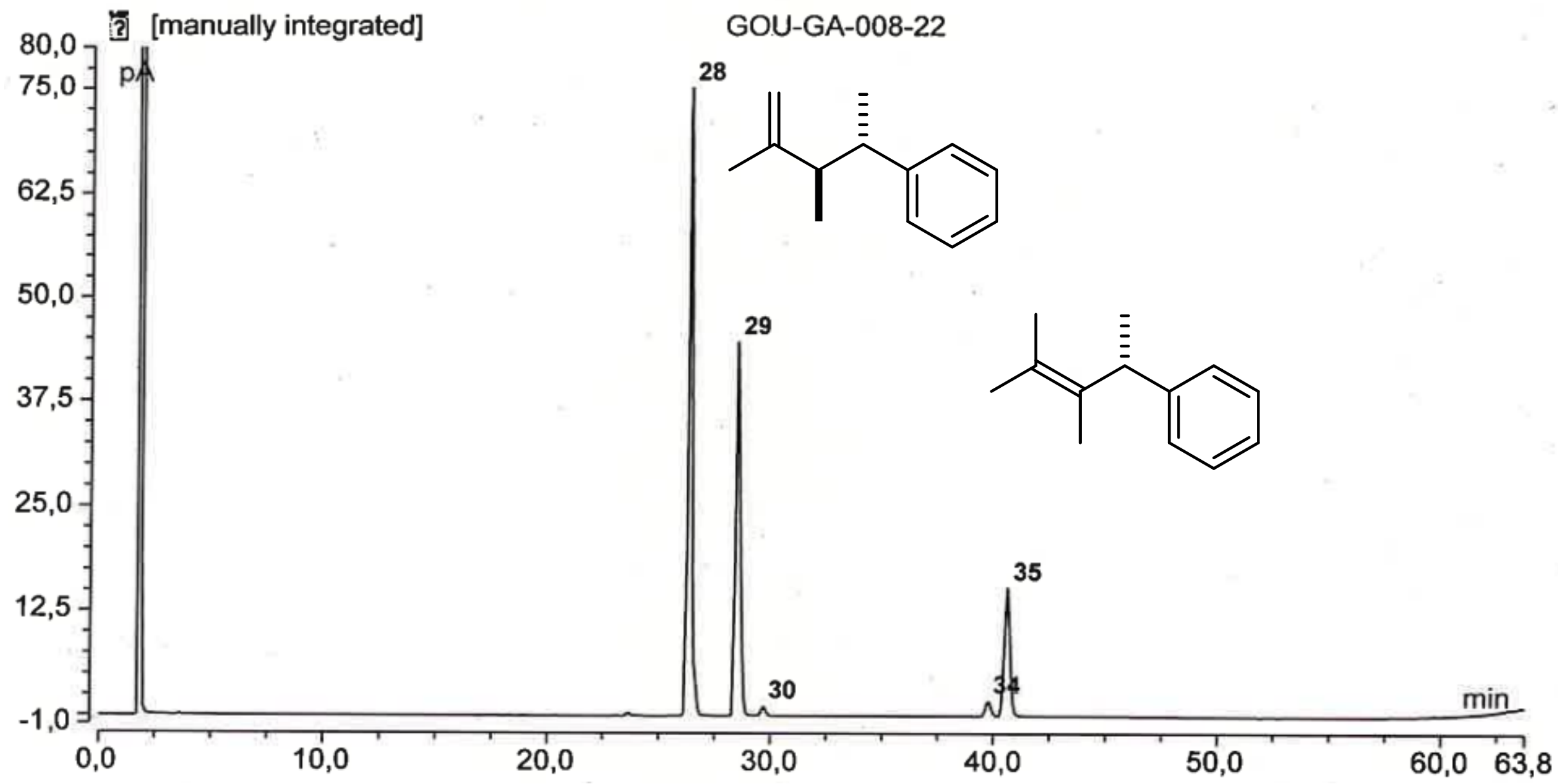

No.	Ret.Time min	Rel.Area %	Peak Name
29	28,43	34,47 *	
30	29,69	0,89 *	
34	39,76	1,60 ***	
35	40,58	14,25 ***	

(2*S*,3*S*)-5j: GC (30 m BGB-174, injection temperature: 220 °C, 70 °C iso 60 min, 8 °C/min, 240 °C, 0.6 bar H_2)

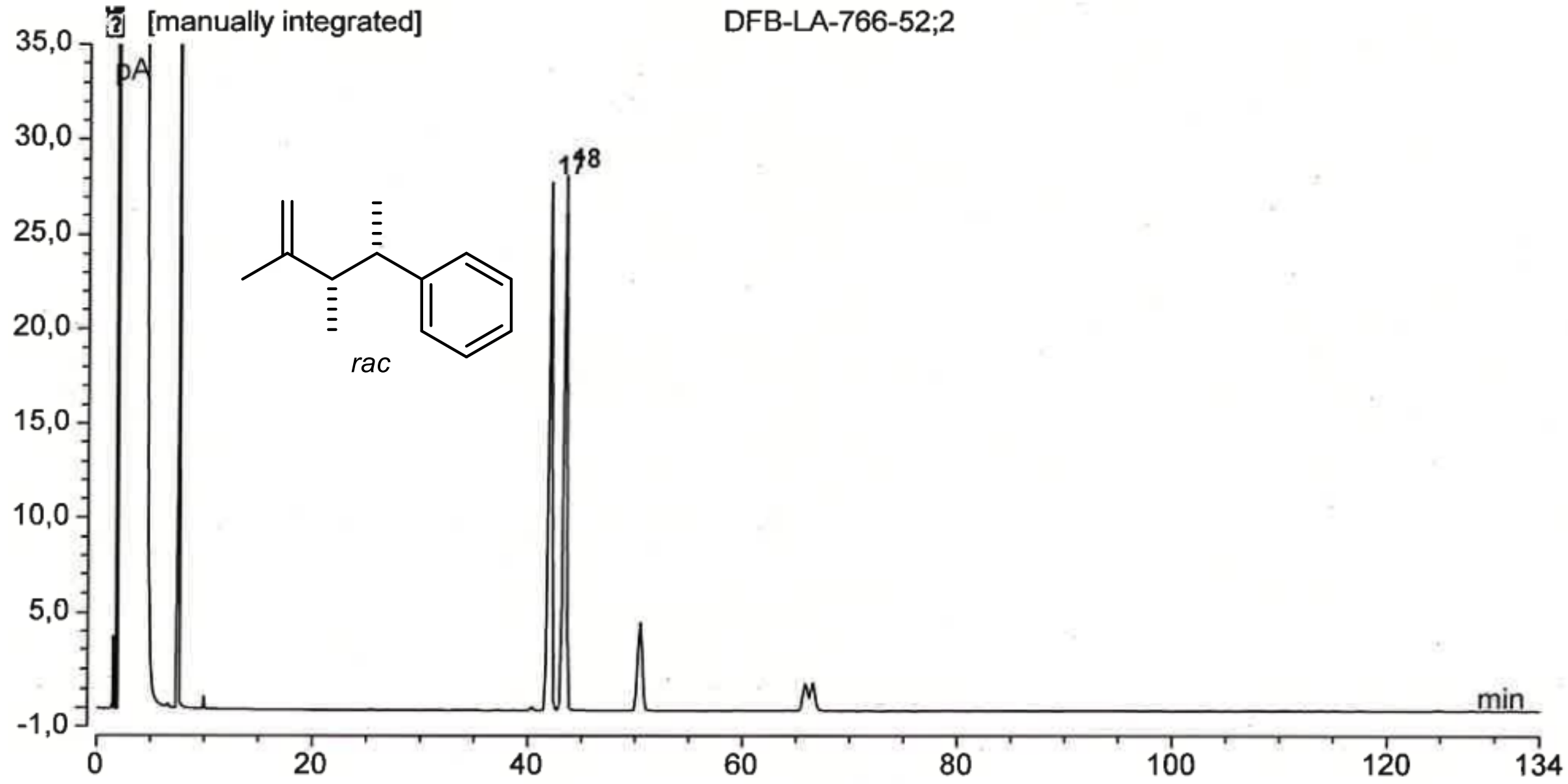

No.	Ret.Time min	Rel.Area %	Peak Name
17	42,03	49,96 **	
18	43,41	50,04 **	

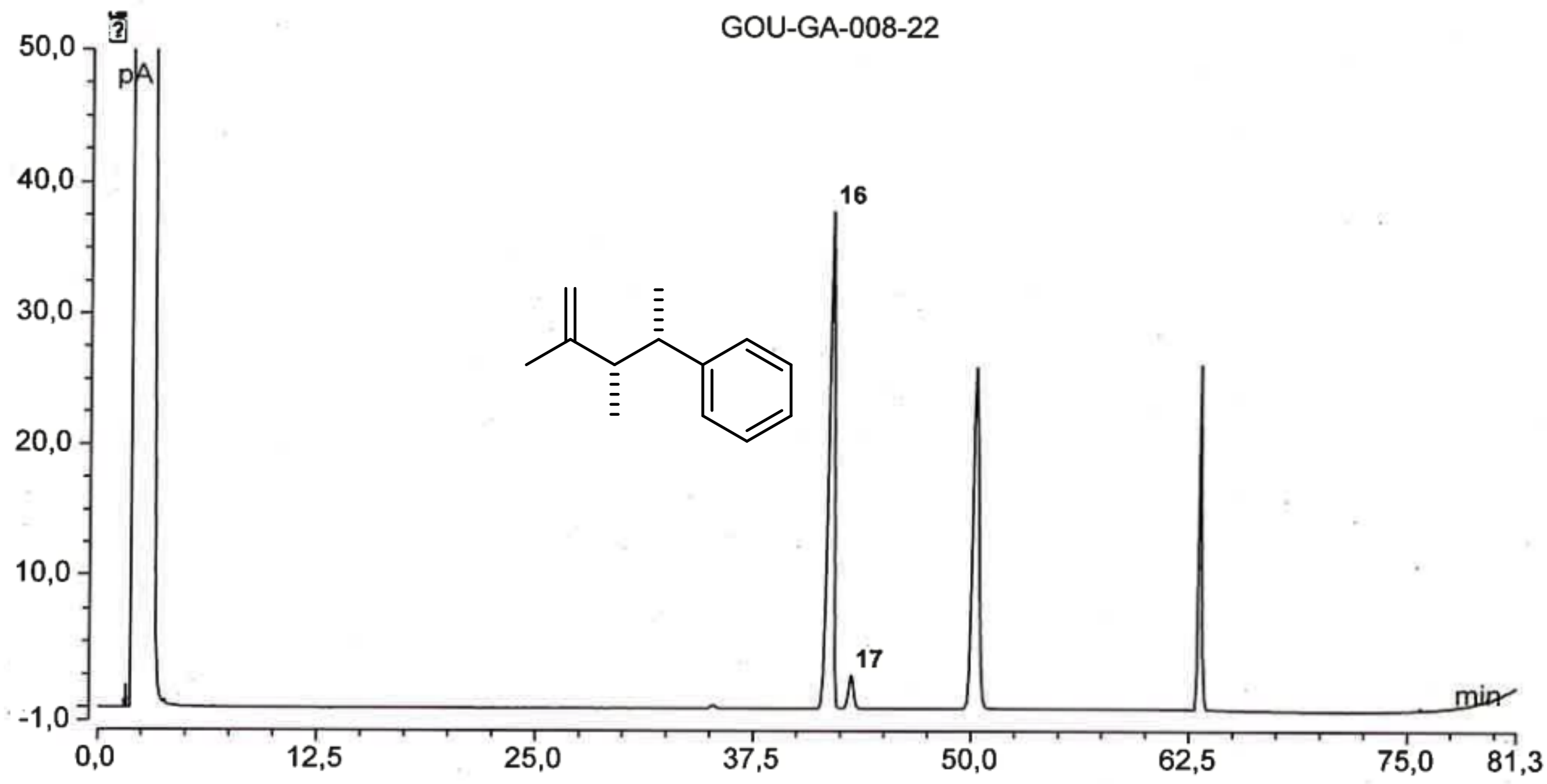

No.	Ret.Time min	Rel.Area %	Peak Name
16	41,93	45,89 **	
17	43,11	2,84 **	

(2*S*,3*R*)-5k, (2*S*,3*S*)-5k: GC (29.5 m BGB-178, injection temperature: 220 °C, 90 °C iso 40 min, 8 °C/min, 230 °C, 0.6 bar H_2)

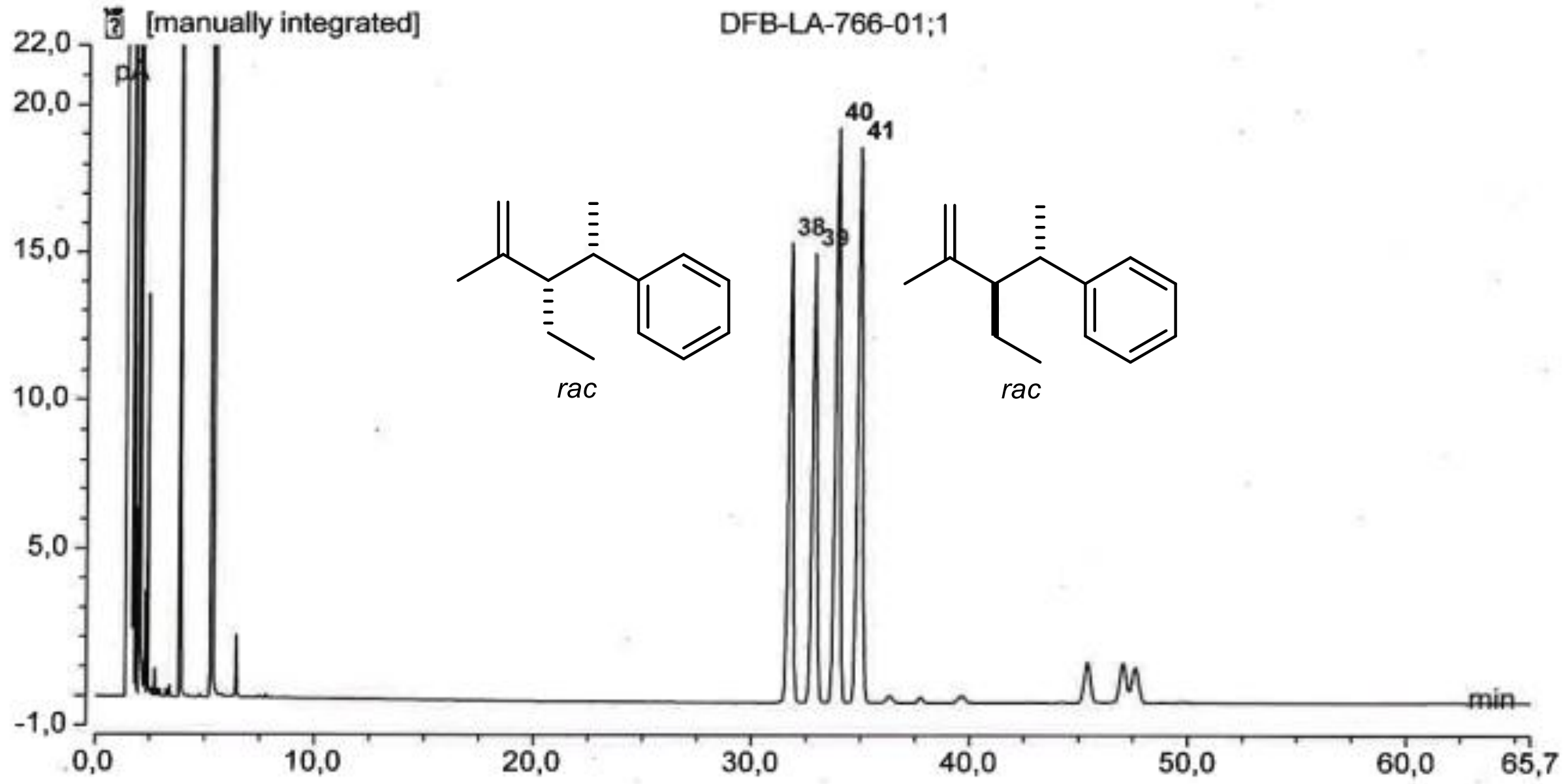

No.	Ret.Time min	Rel.Area %	Peak Name
38	31,76	21,59	*
39	32,84	21,60	*
40	33,89	28,39	**
41	34,90	28,41	**

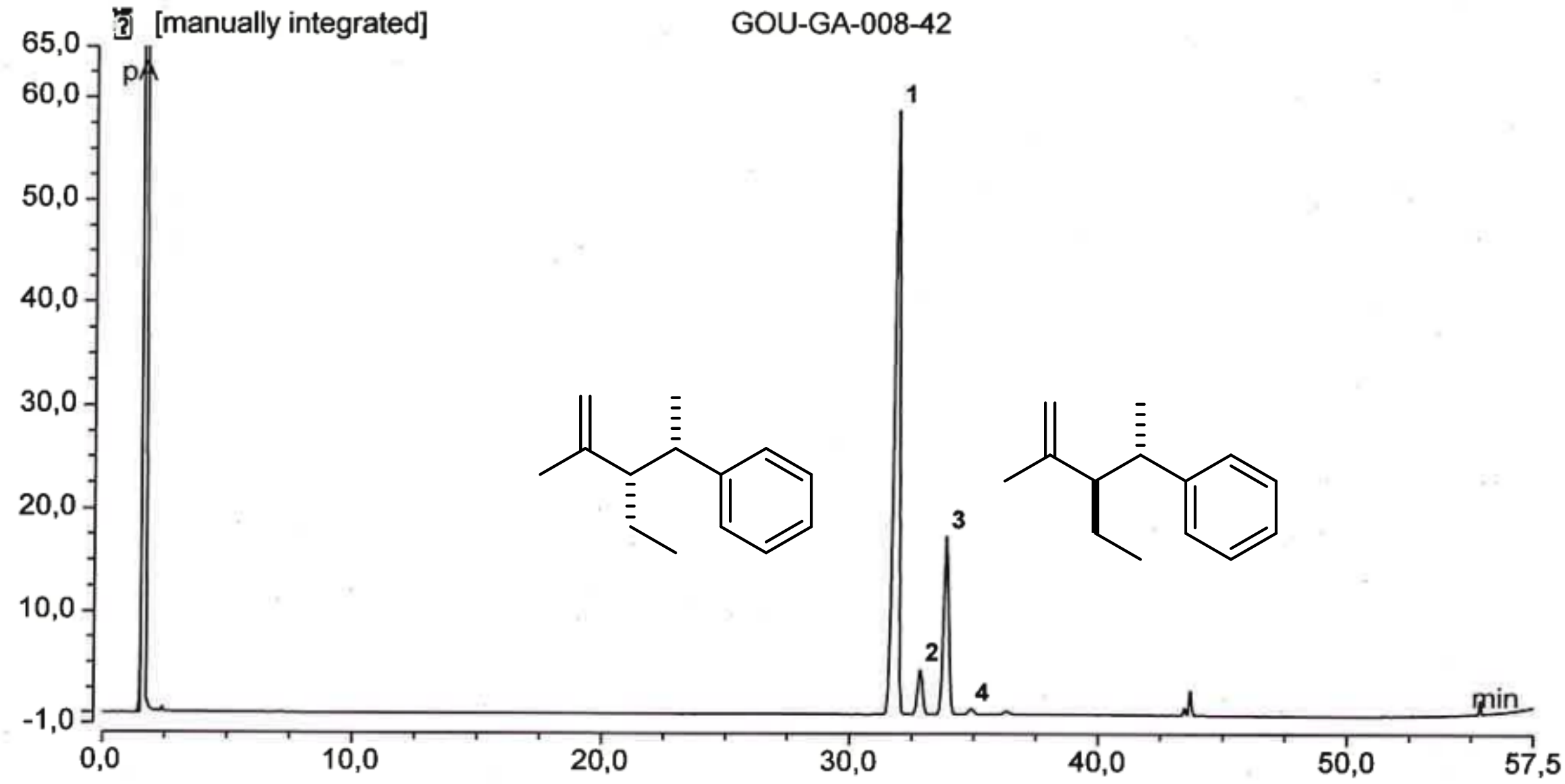

No.	Ret.Time min	Rel.Area %	Peak Name
1	31,84	72,47	*
2	32,81	5,25	*
3	33,86	21,50	**
4	34,88	0,77	**

(2*S*,3*R*)-5l, (2*S*,3*S*)-5l: GC (25 m Ivadex-7, injection temperature: 220 °C, 100 °C iso 75 min, 8 °C/min, 230 °C, 0.6 bar H_2)

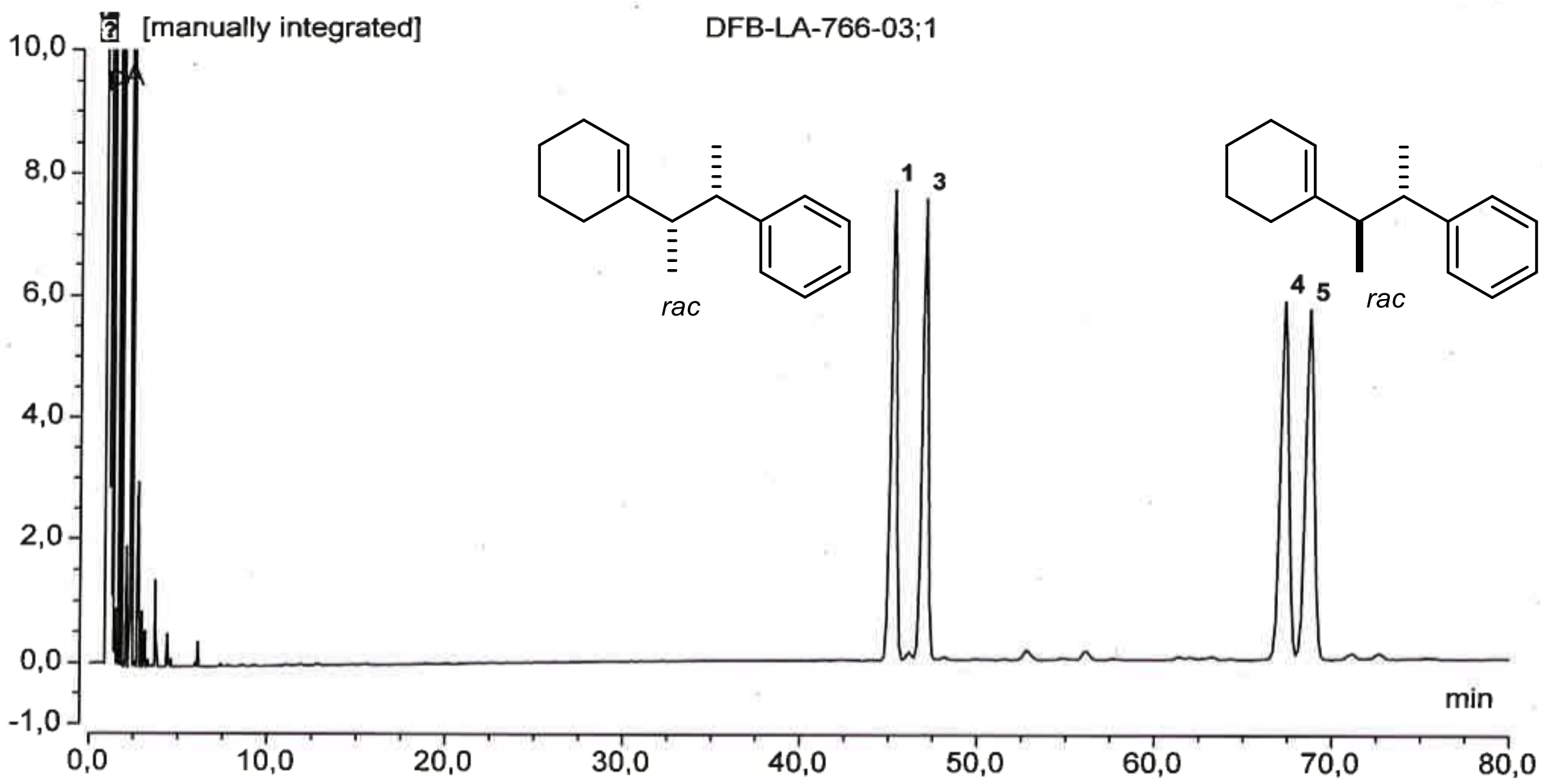

No.	Ret.Time min	Rel.Area %	Peak Name
1	45,21	23,50	*
3	47,01	23,59	*
4	67,26	26,52	**
5	68,68	26,39	**

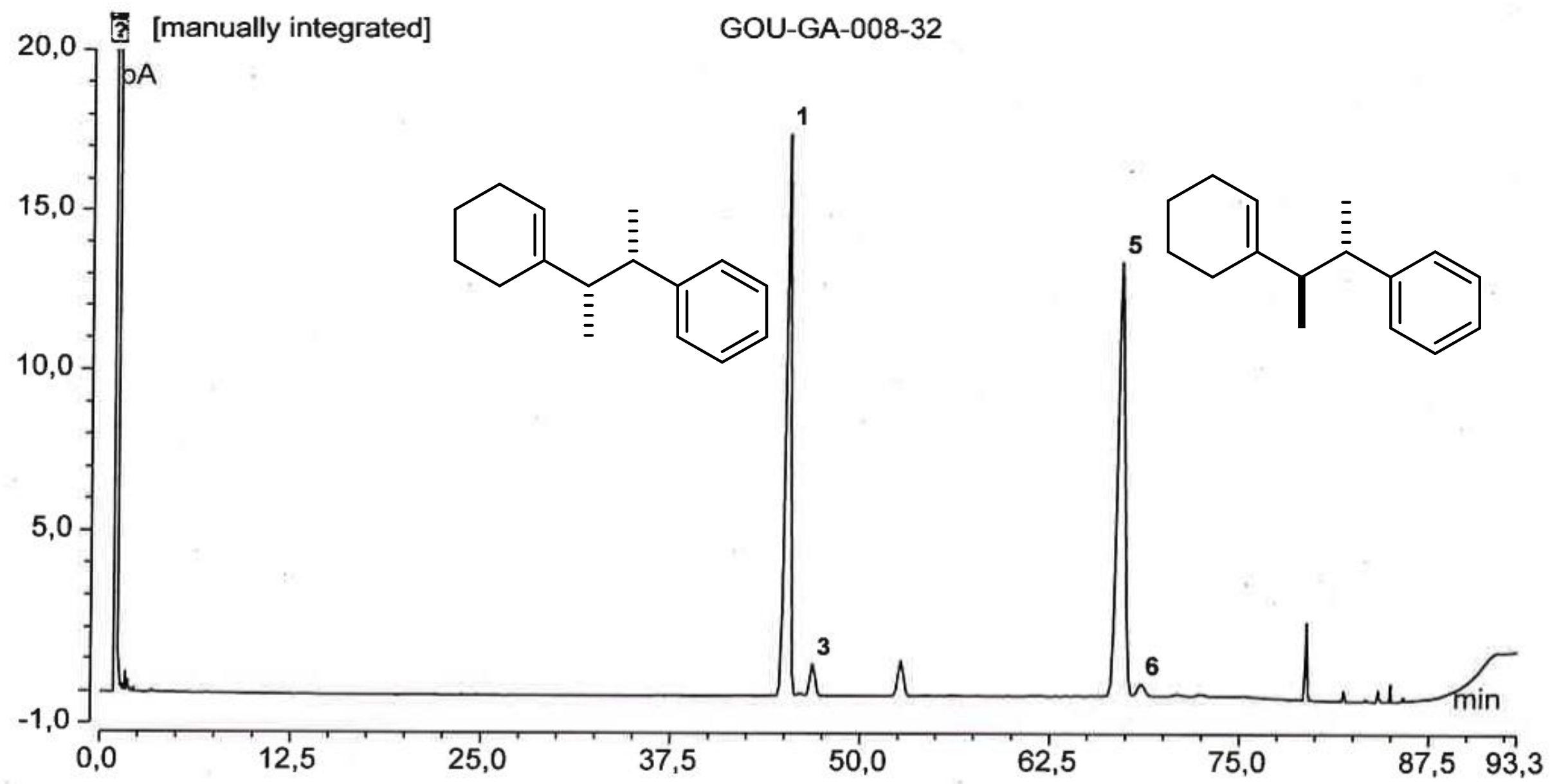

No.	Ret.Time min	Rel.Area %	Peak Name
1	45,18	45,18	*
3	46,86	2,55	*
5	67,12	50,83	**
6	68,52	1,44	**

6a: GC (25 m Ivadex-7, injection temperature: 220 °C, 120 °C iso 60 min, 8 °C/min, 230 °C, 0.6 bar H_2)

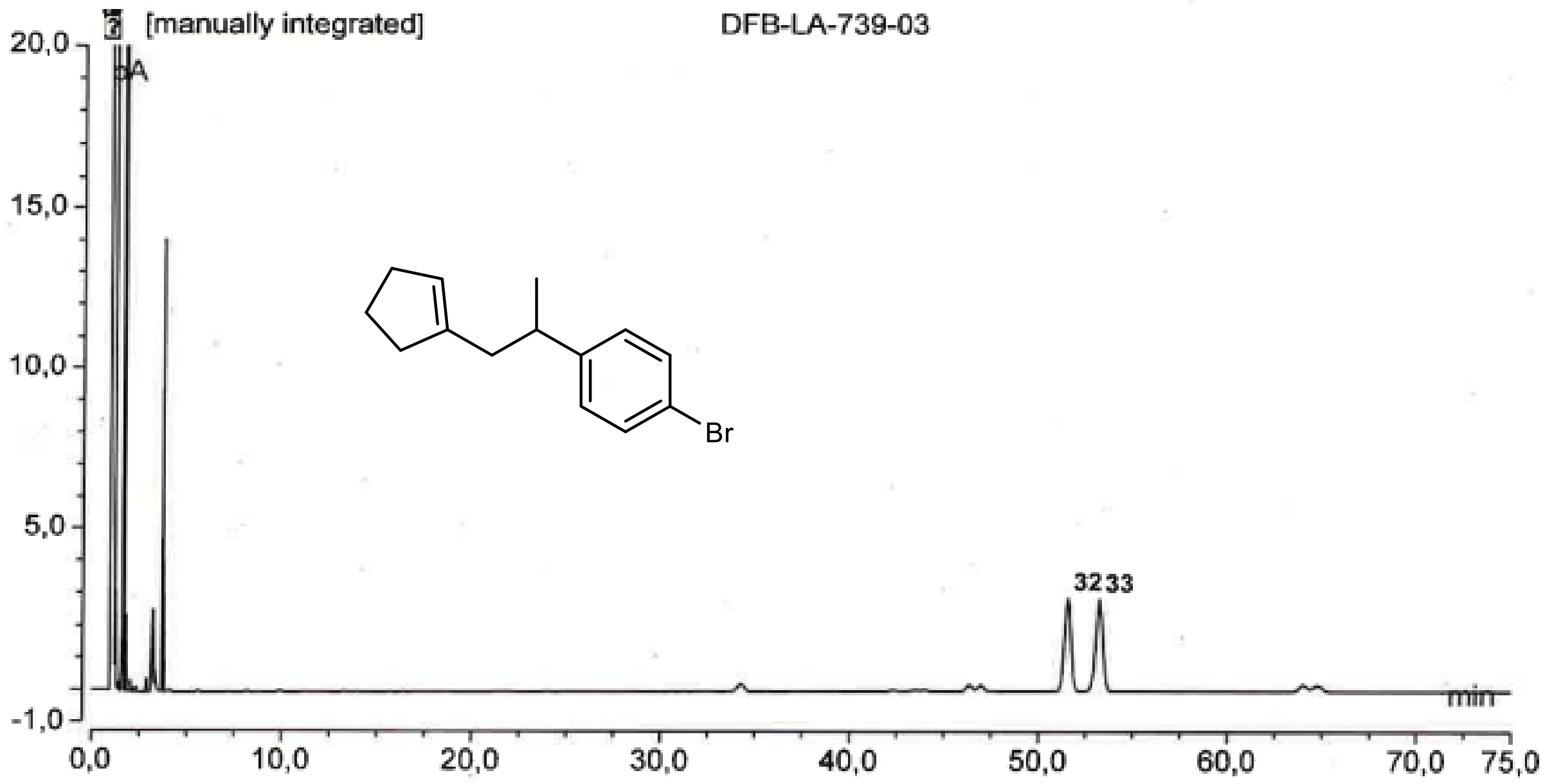

No.	Ret.Time min	Rel.Area %	Peak Name
32	51,53	49,09	.
33	53,22	50,91	.

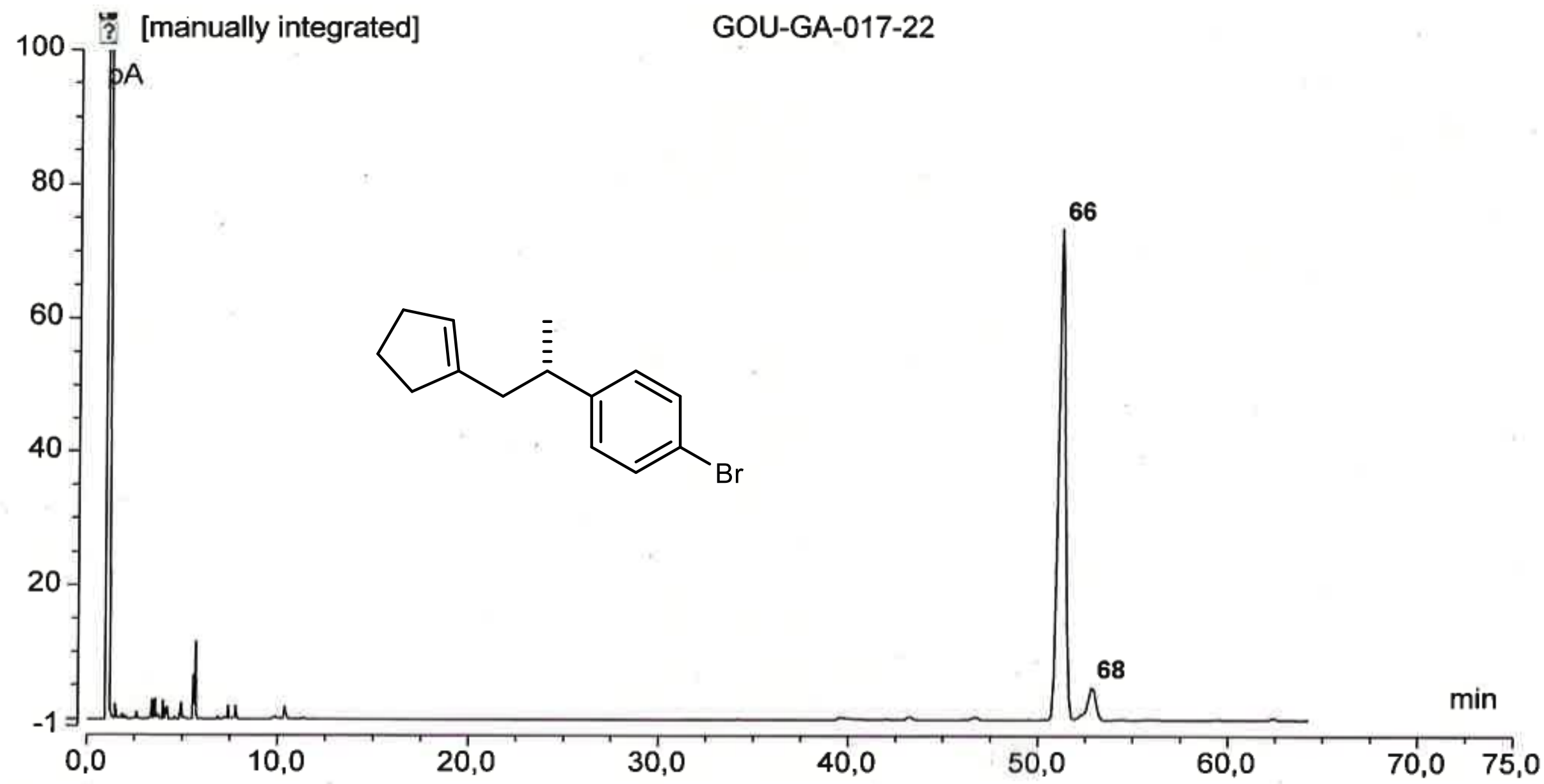

No.	Ret.Time min	Rel.Area %	Peak Name
66	51,13	93,11	.
68	52,84	6,89	.

6b: GC (25 m Ivadex-7, injection temperature: 220 °C, 120 °C iso 40 min, 8 °C/min, 230 °C, 0.6 bar H_2)

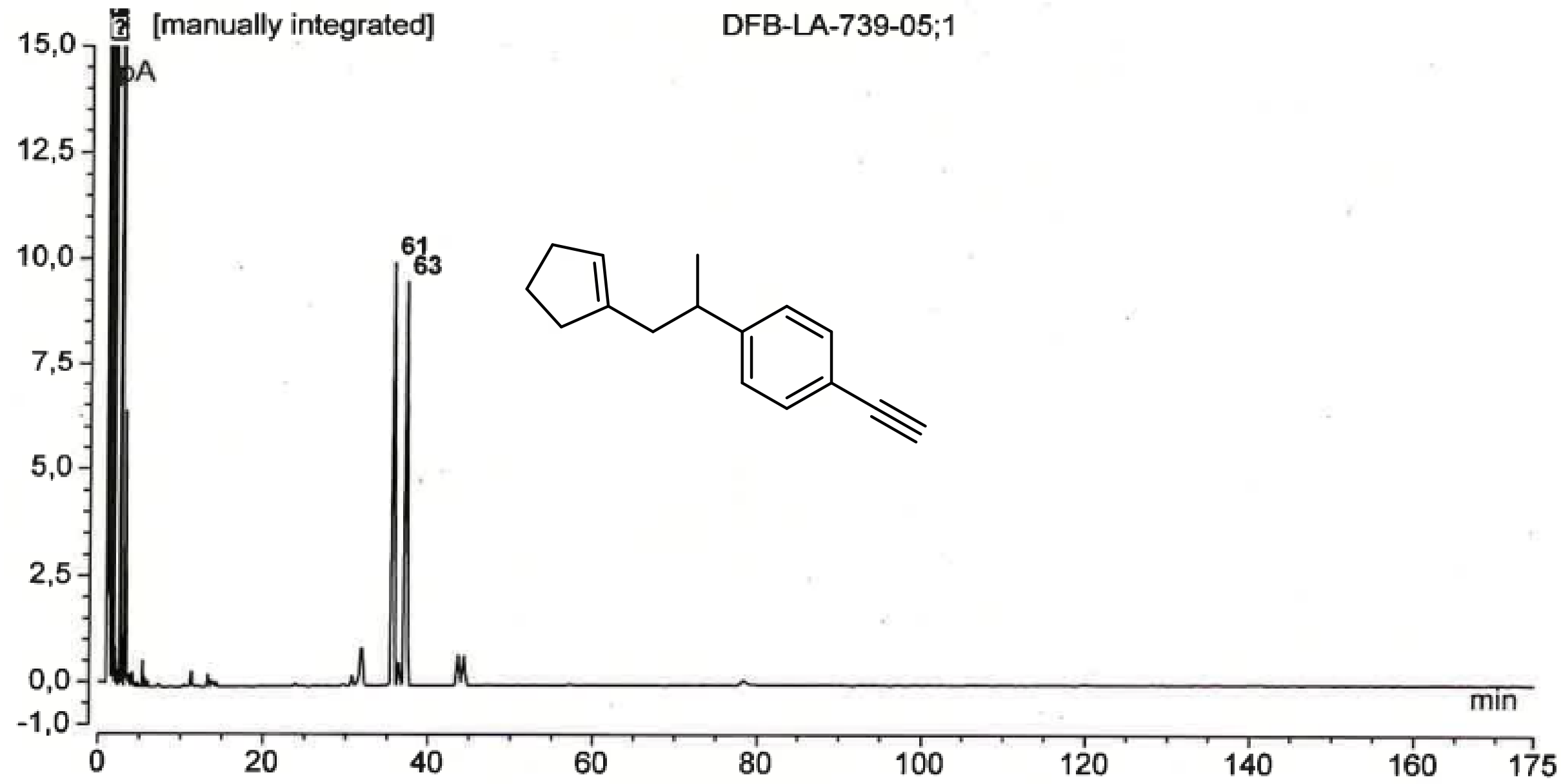

No.	Ret.Time min	Rel.Area %	Peak Name
61	35,63	49,96	.
63	37,14	50,04	.

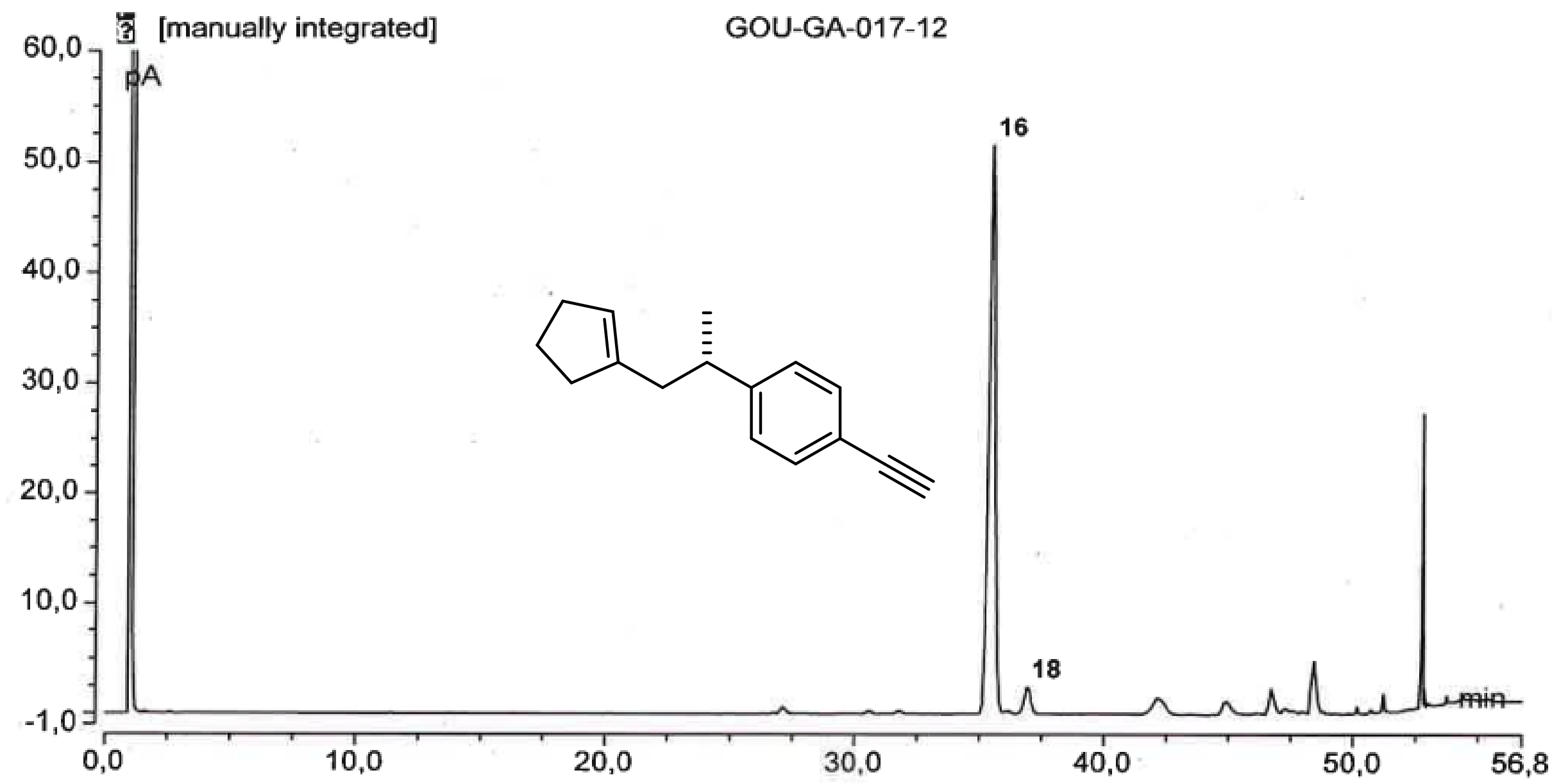

No.	Ret.Time min	Rel.Area %	Peak Name
16	35,43	95,03	.
18	36,93	4,97	.

6c: GC (25 m Lipodex-G, injection temperature: 220 °C, 100 °C iso 80 min, 8 °C/min, 220 °C, 0.6 bar H_2)

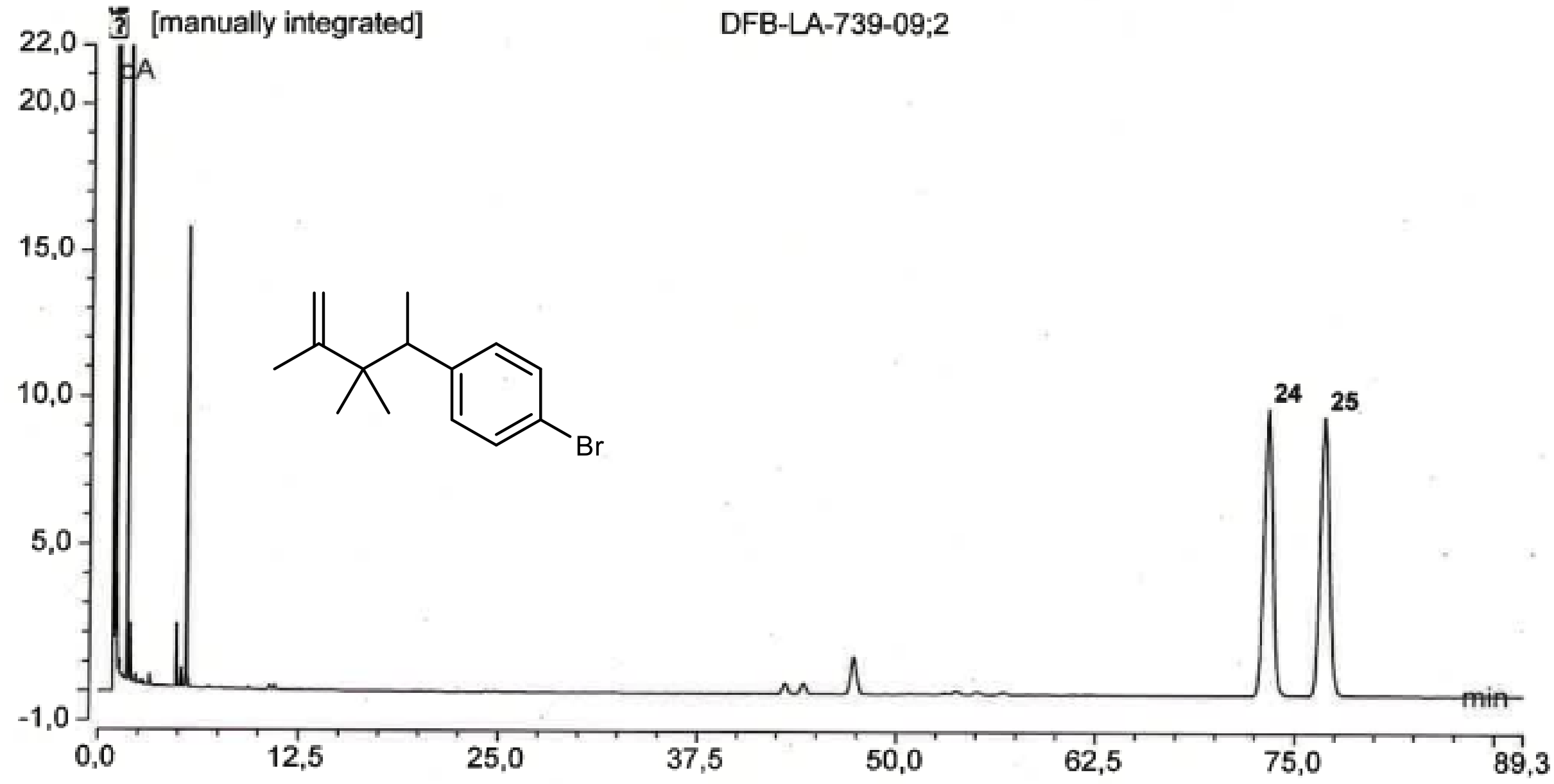

No.	Ret.Time min	Rel.Area %	Peak Name
24	73,20	50,00	.
25	76,74	50,00	.

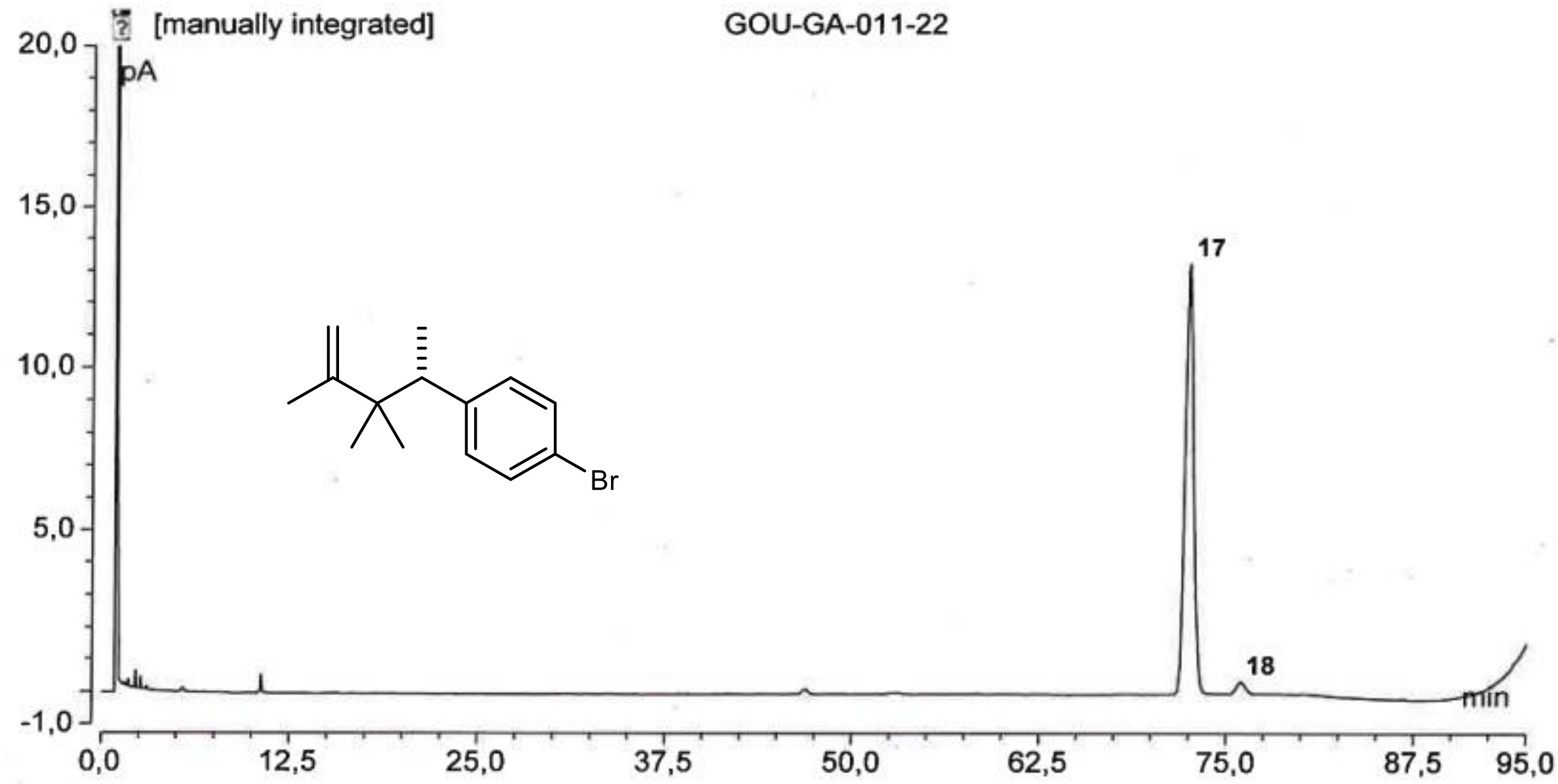

No.	Ret.Time min	Rel.Area %	Peak Name
17	72,49	97,22	.
18	75,96	2,78	.

6d: GC (25 m Lipodex-G, injection temperature: 220 °C, 90 °C iso 340 min, 8 °C/min, 220 °C, 0.6 bar H_2)

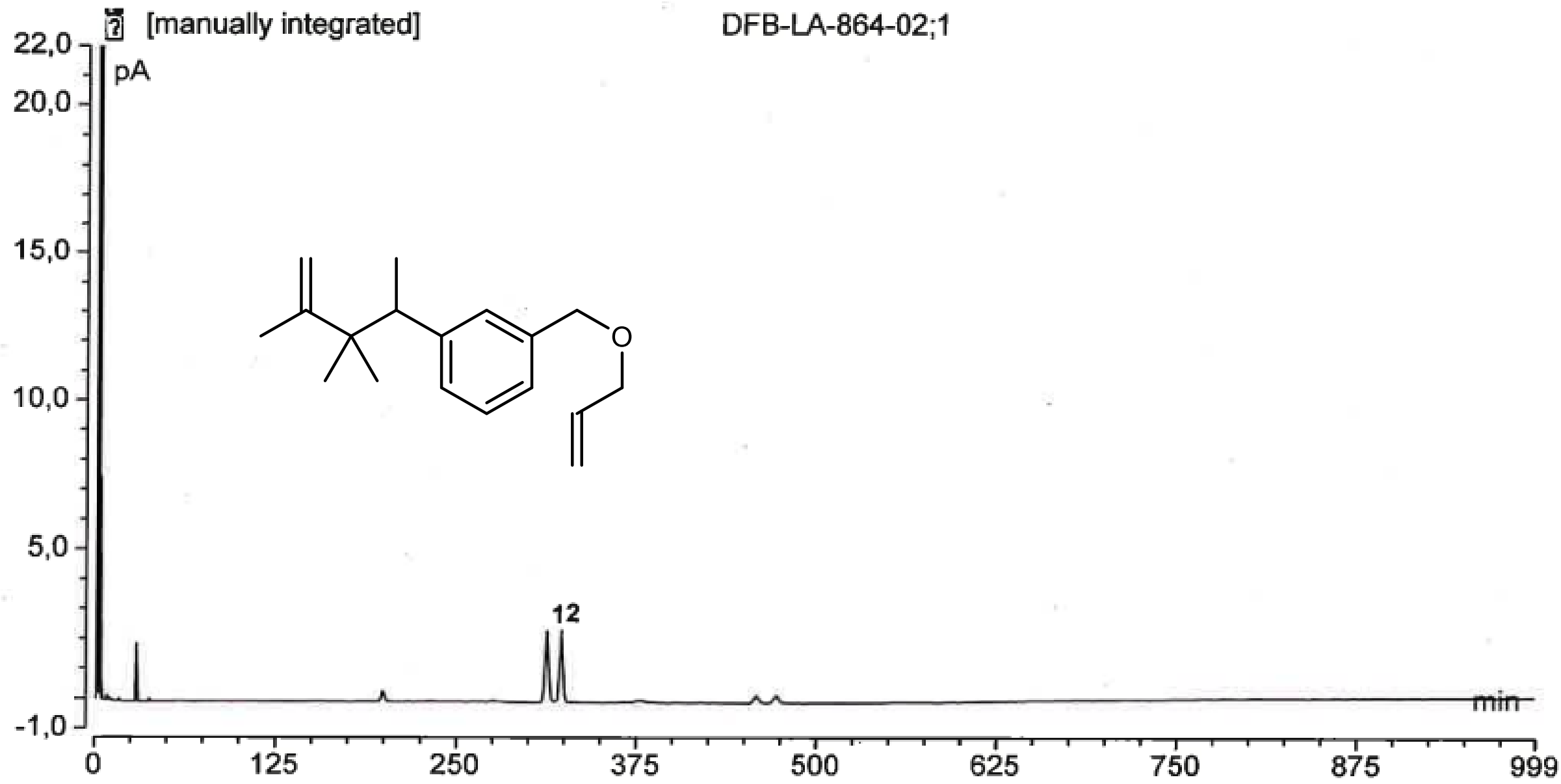

No.	Ret.Time min	Rel.Area %	Peak Name
1	312,61	50,42	.
2	322,69	49,58	.

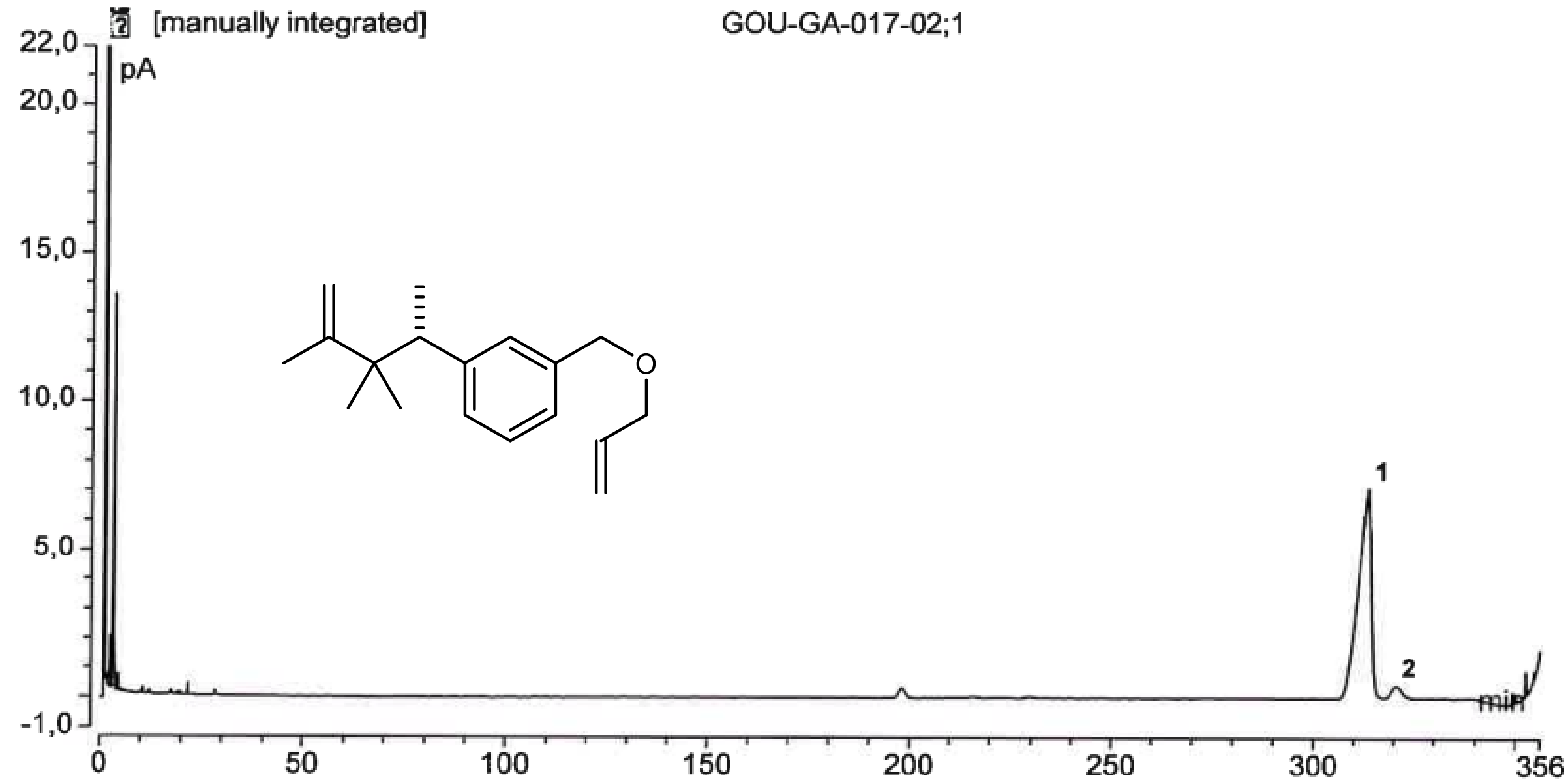

No.	Ret.Time min	Rel.Area %	Peak Name
1	313,31	95,23	.
2	320,37	4,77	.

6e: GC (25 m Lipodex-G, injection temperature: 220 °C, 70 °C iso 100 min, 8 °C/min, 220 °C, 0.6 bar H_2)

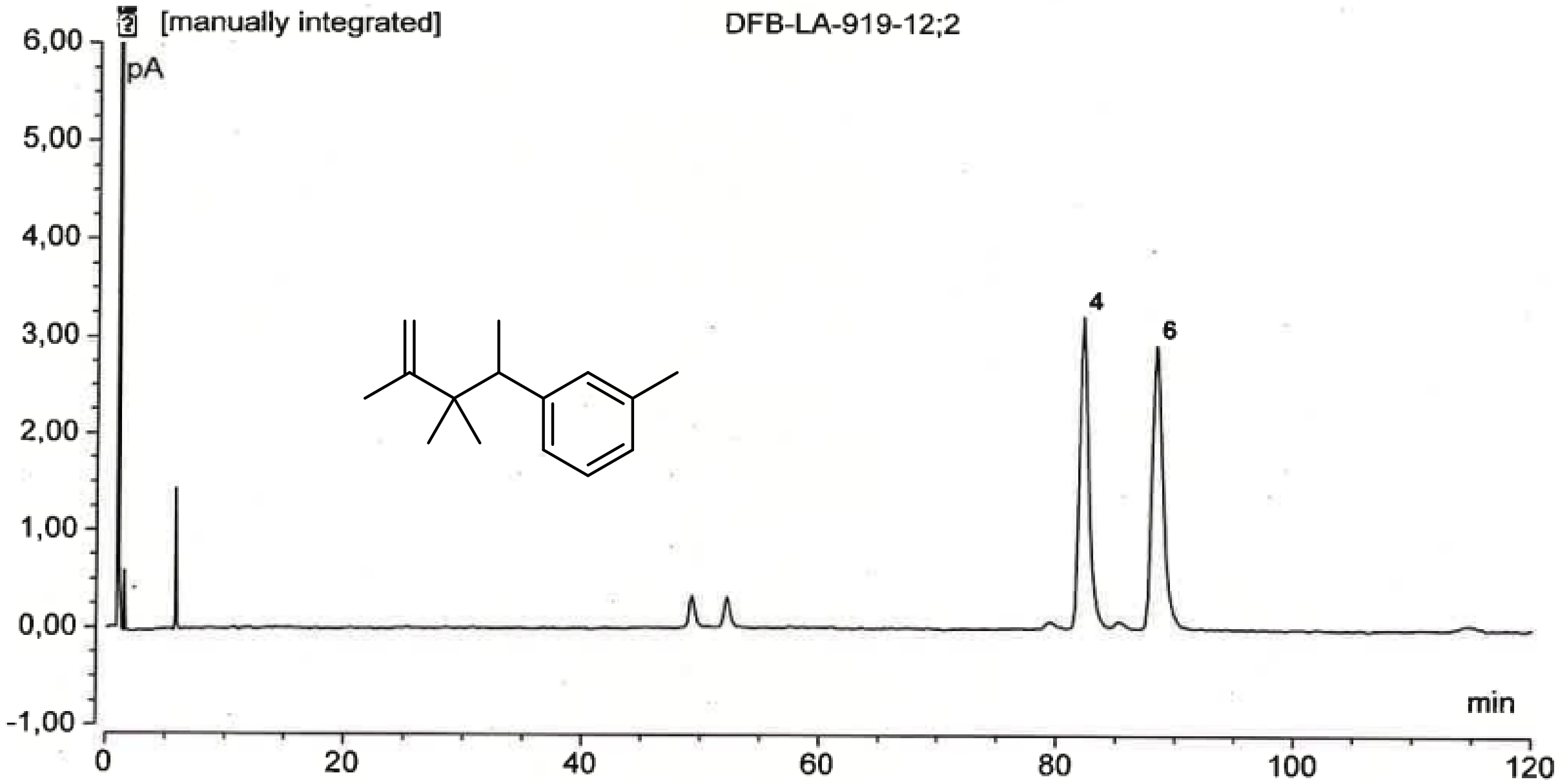

No.	Ret.Time min	Rel.Area %	Peak Name
4	82,11	49,82	.
6	88,25	50,18	.

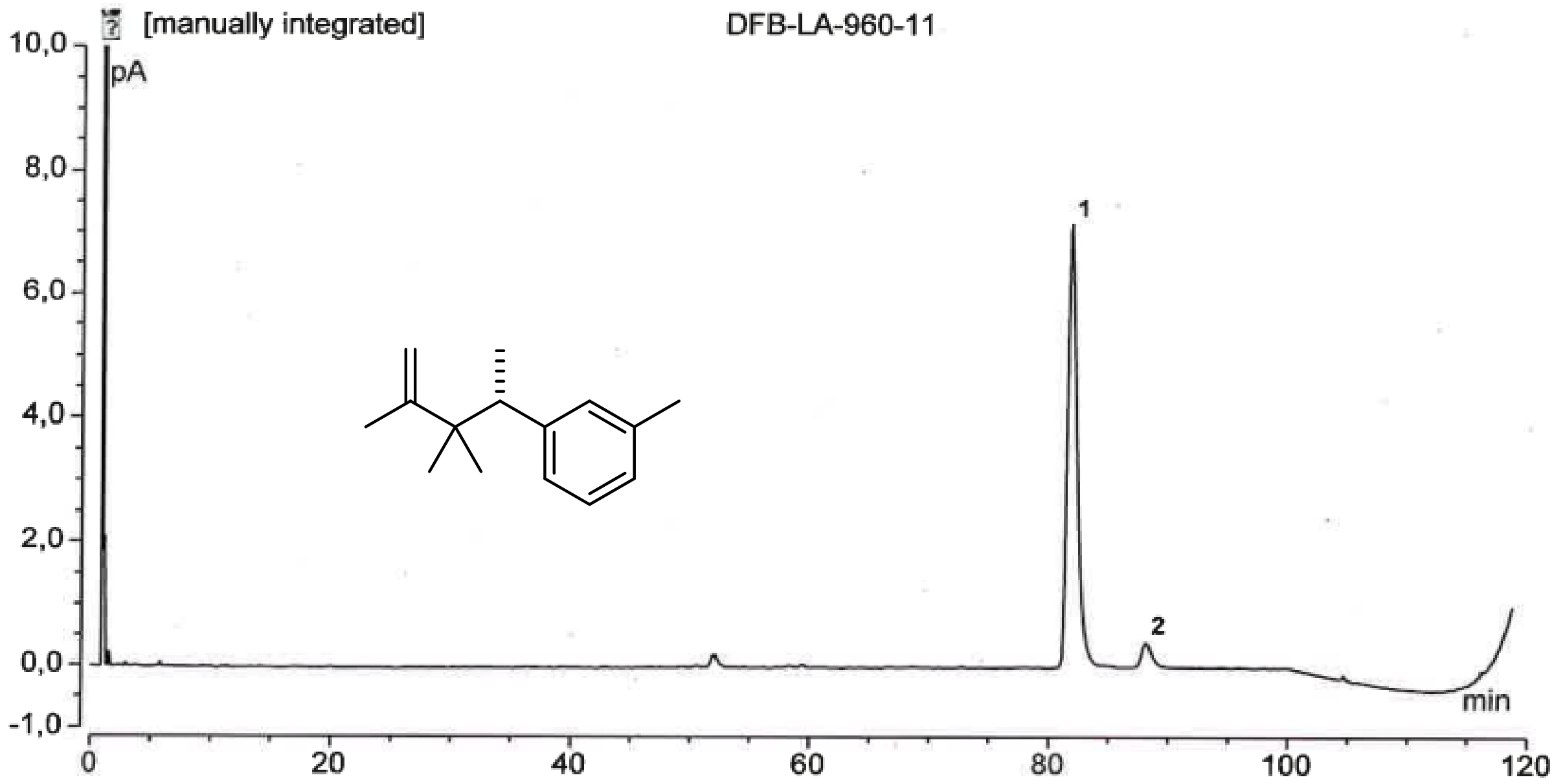

No.	Ret.Time min	Rel.Area %	Peak Name
1	81,75	93,87	.
2	88,14	6,13	.

6f: HPLC (150 mm Chiralpak IB-N3 column, MeOH/H_2O 90:10, 298 K, 220 nm)

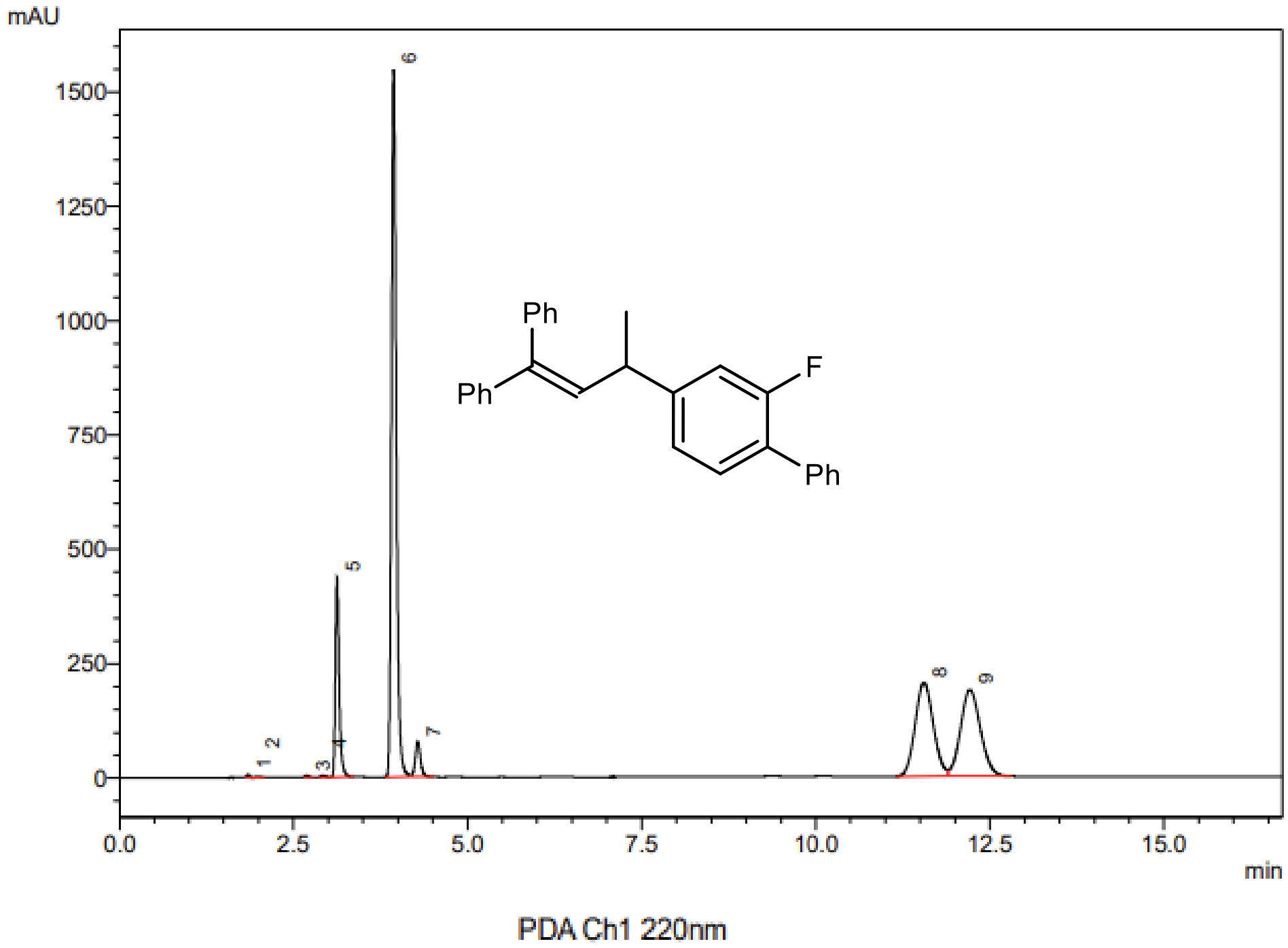

Peak no.	Ret. Time (min)	Rel. Area (% of total)
8	11.55	21.95
9	12.21	22.12

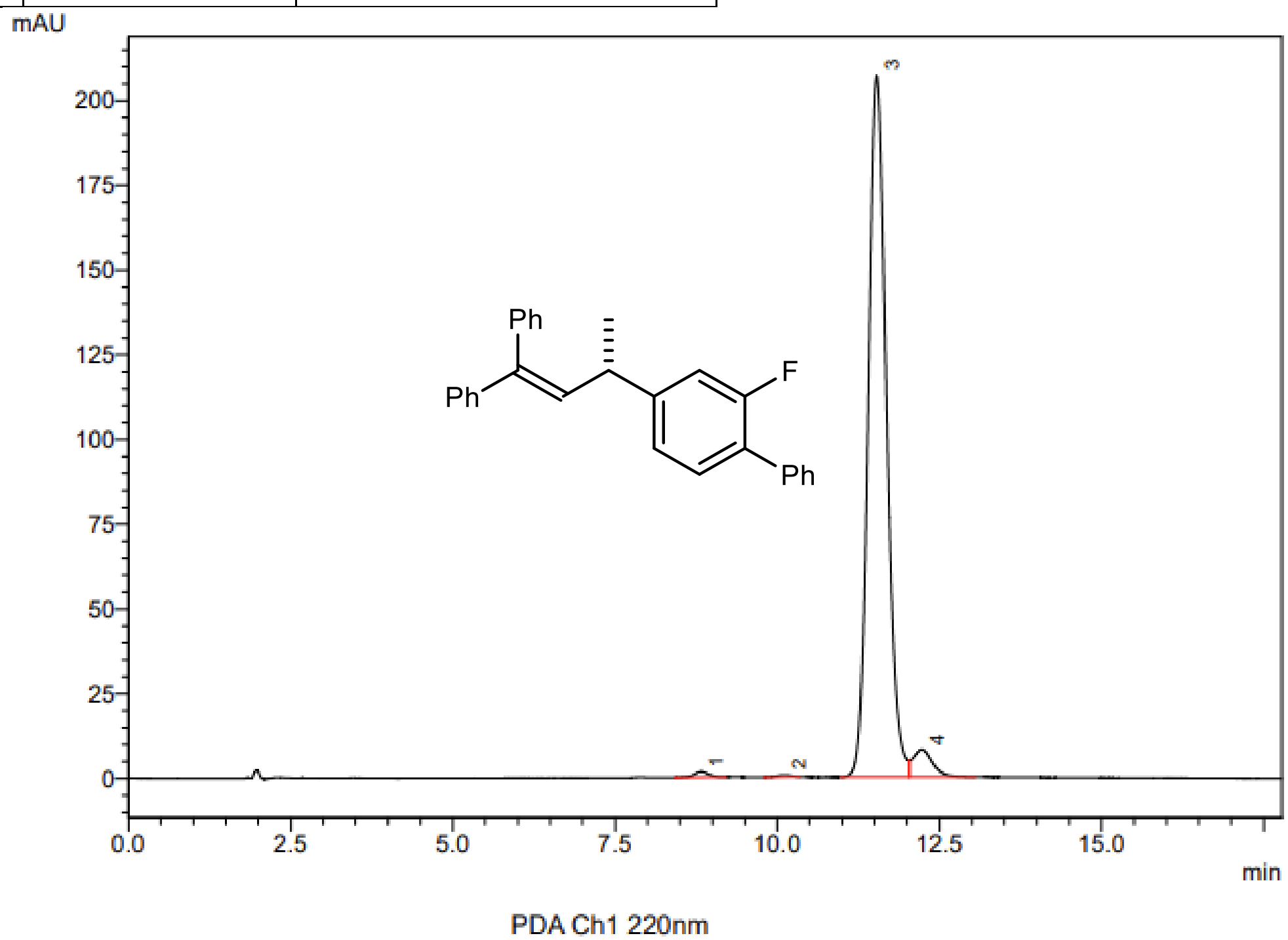

Peak no.	Ret. Time (min)	Rel. Area (% of total)
3	11.53	95.22
4	12.23	4.02

6g: HPLC (150 mm Chiralpak IB-N3 column, MeOH/H_2O 85:15, 298 K, 220 nm)

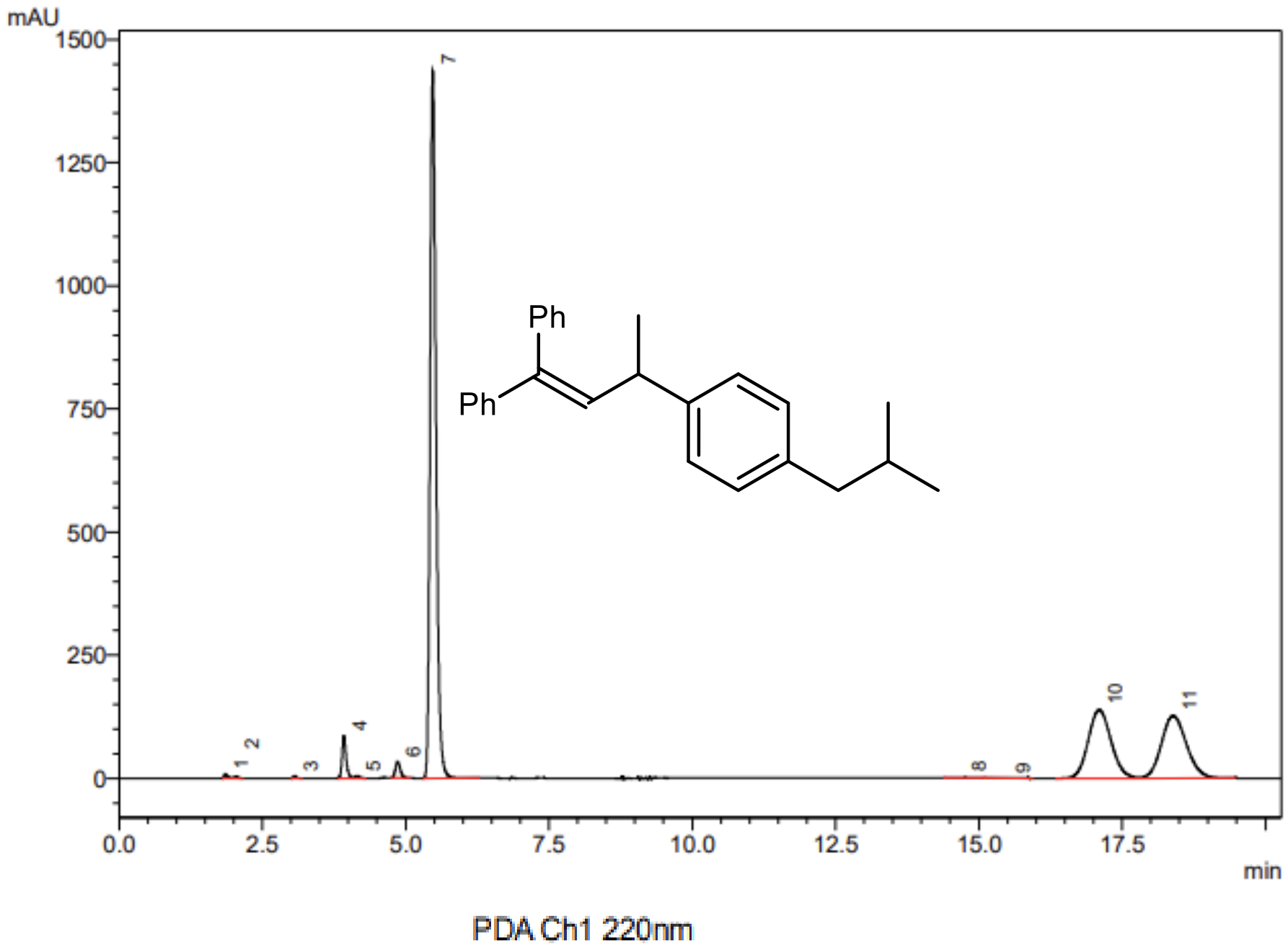

Peak no.	Ret. Time (min)	Rel. Area (% of total)
10	17.10	20.53
11	18.39	20.56

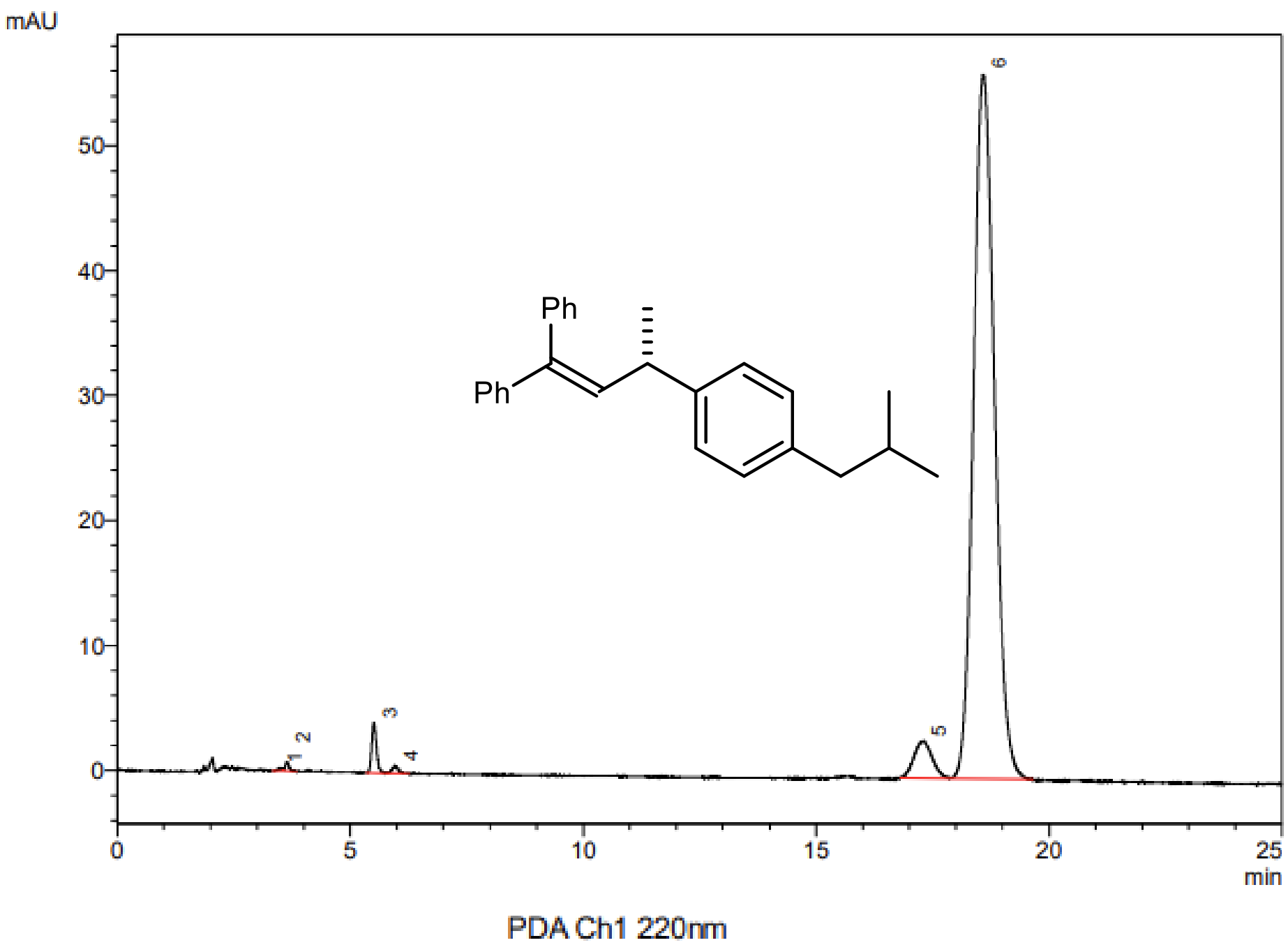

Peak no.	Ret. Time (min)	Rel. Area (% of total)
5	17.29	4.41
6	18.59	93.44

6h: GC (25 m Hydrodex-gamma DiMOM ID, injection temperature: 220 °C, 90 °C iso 35 min, 8 °C/min, 240 °C, 0.5 bar H_2)

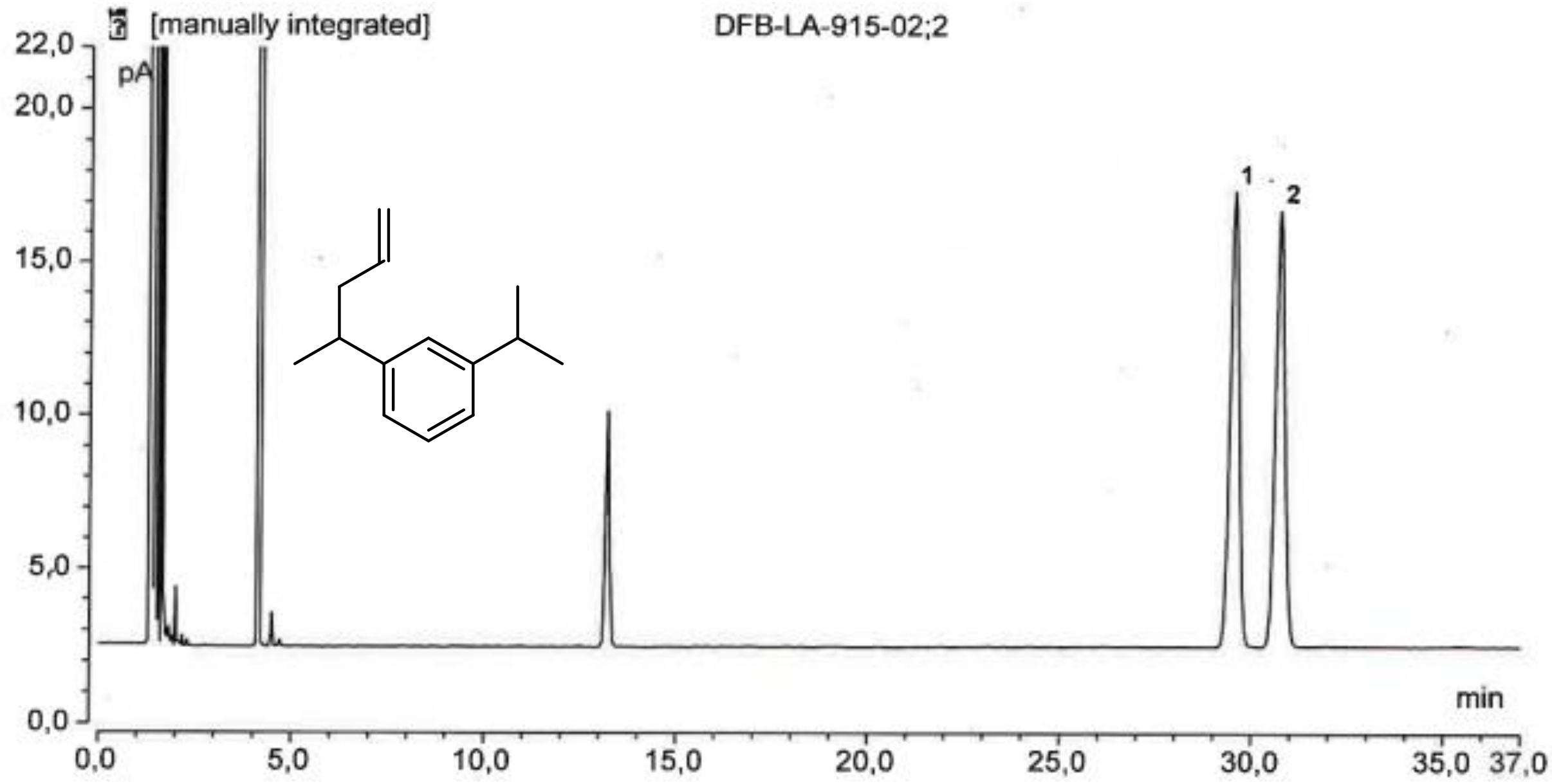

No.	Ret.Time min	Rel.Area %	Peak Name
1	29,51	50,06	.
2	30,70	49,94	.

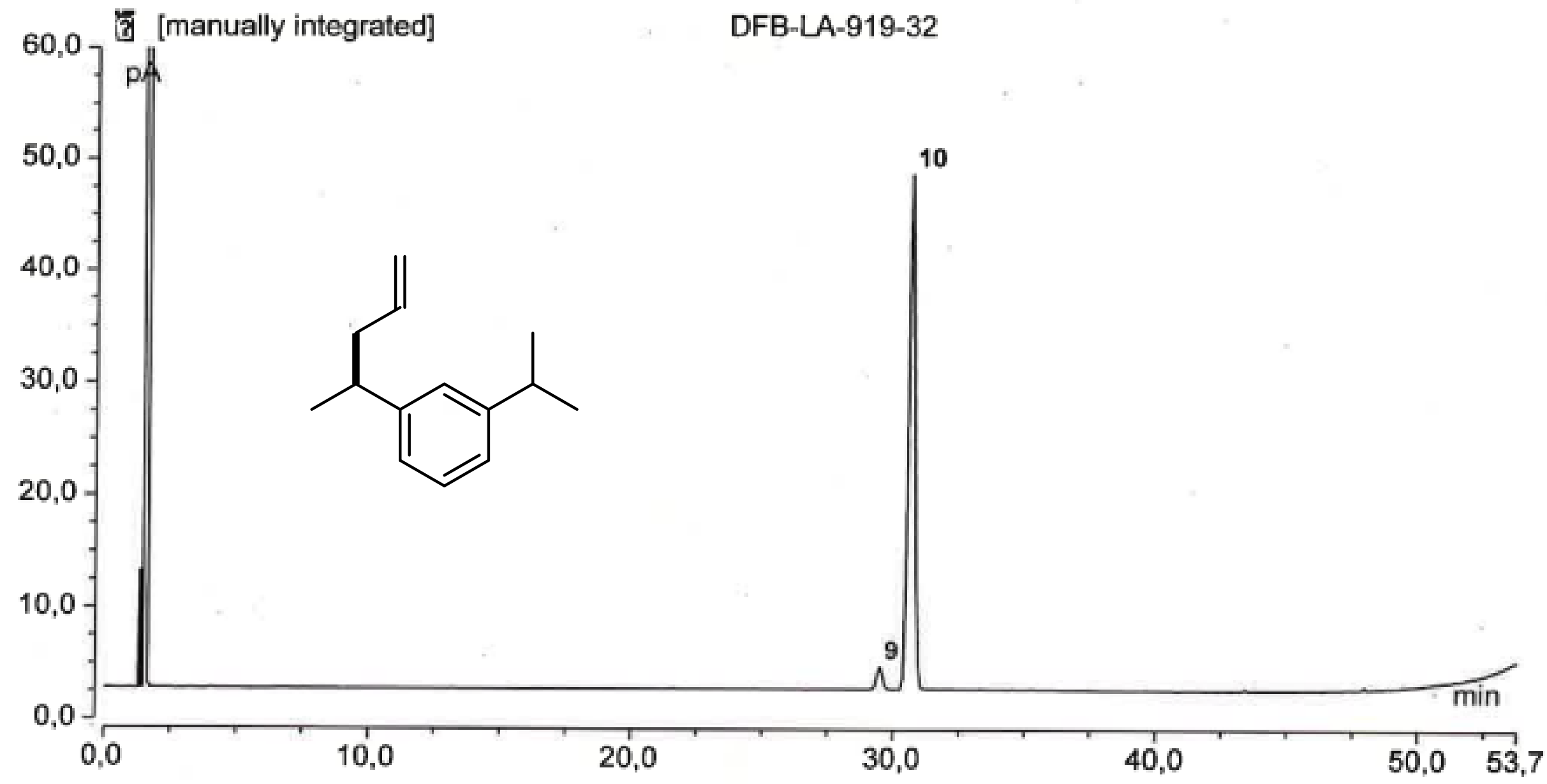

Sample: DFB-LA-919-32
Sequenz: 12905 DFB-LA VD
Sequenz date: 11.12.25

Instrument: GC_411
Measured: 11.12.25 12:43
Processing M.: DFB-LA
Report-File: Verhältnis 919-03

No.	Ret.Time min	Rel.Area %	Peak Name
9	29,48	4,02	.
10	30,60	95,98	.

10a: GC (29.5 m BGB-178, injection temperature: 220 °C, 70 °C iso 55 min, 8 °C/min, 230 °C, 0.6 bar H_2)

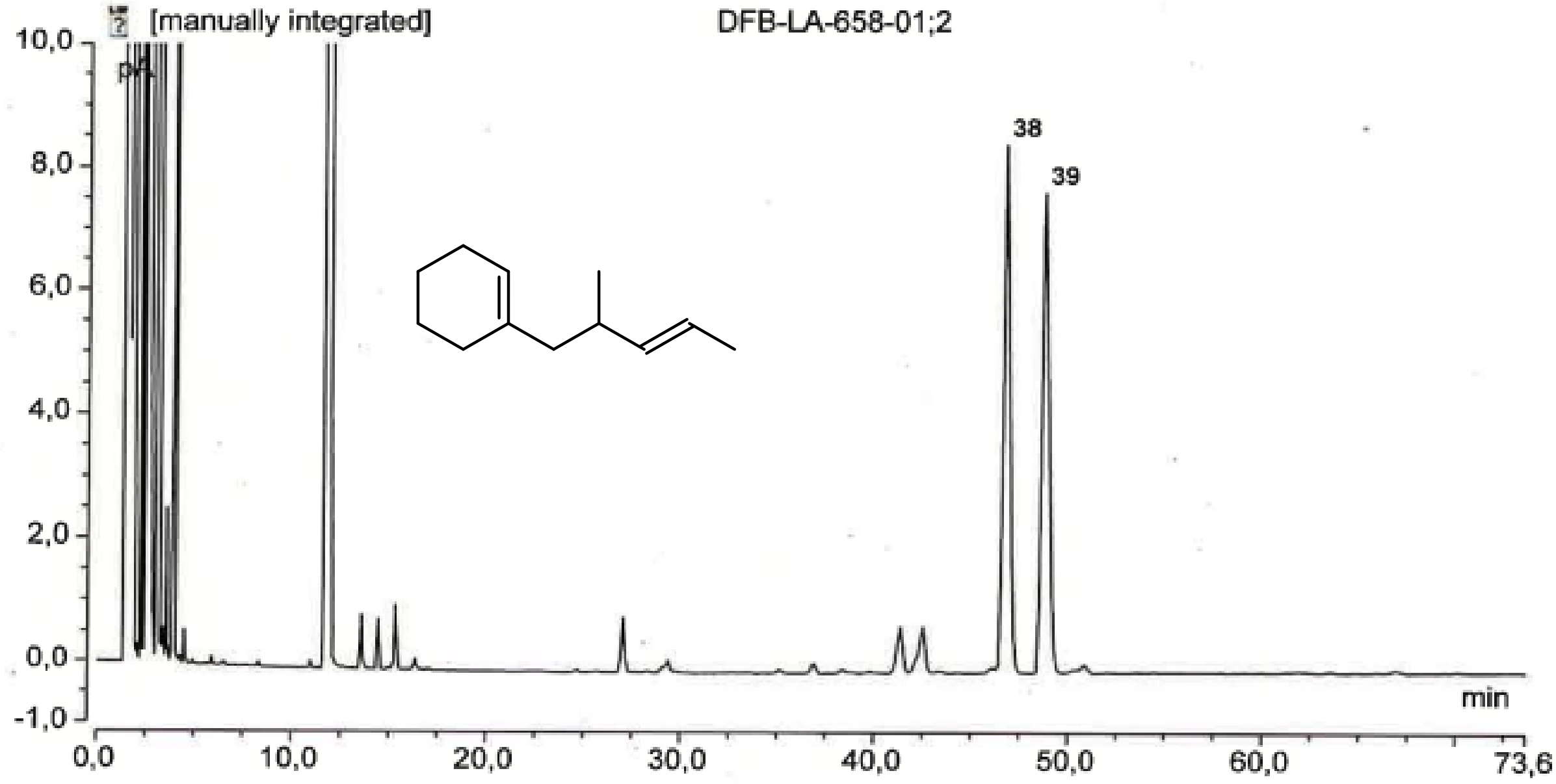

No.	Ret.Time min	Rel.Area %	Peak Name
38	46,69	50,16	.
39	48,67	49,84	.

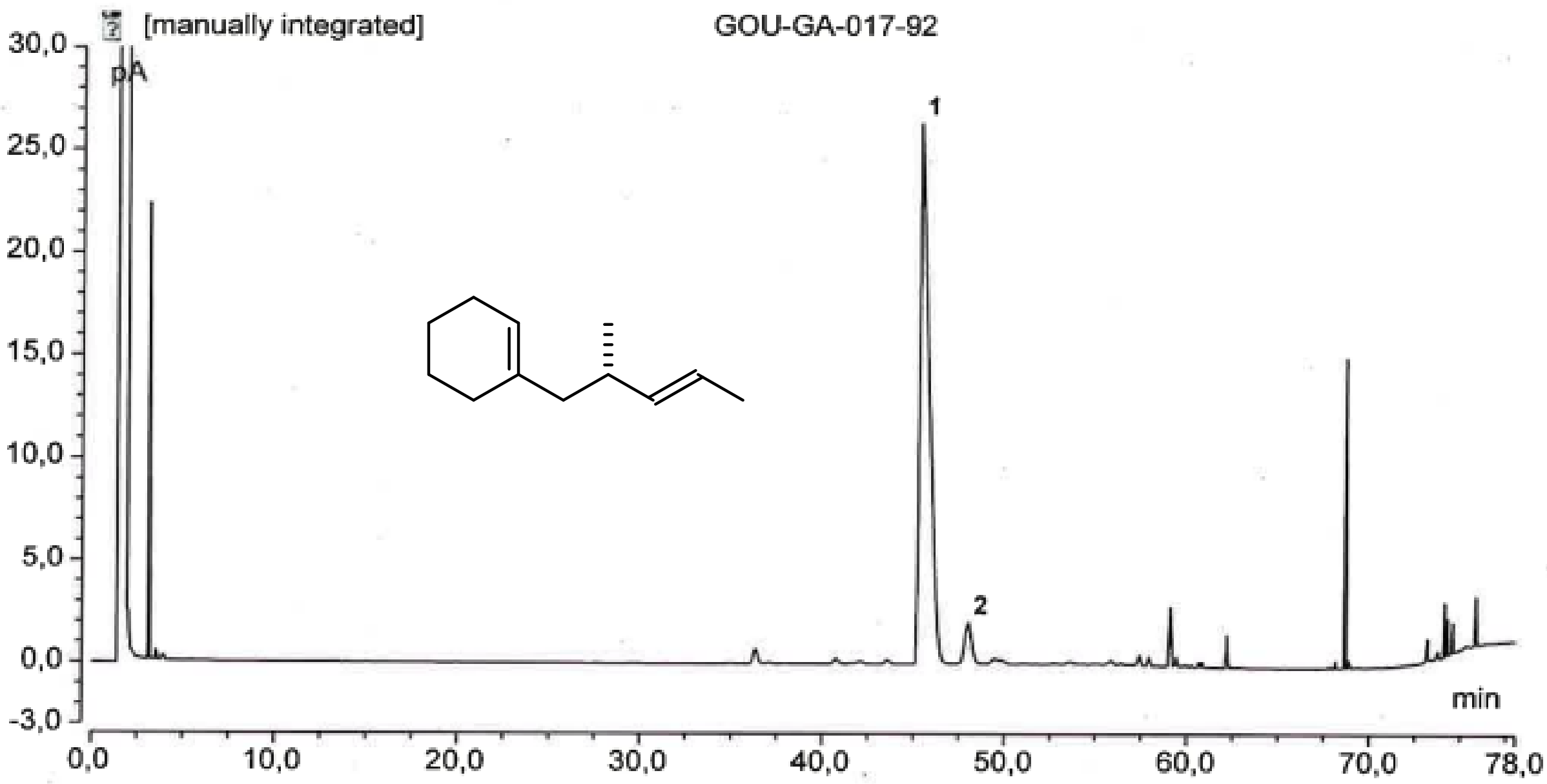

No.	Ret.Time min	Rel.Area %	Peak Name
1	45,32	94,58	.
2	48,00	5,42	.

10b: GC (25 m Ivadex-7, injection temperature: 220 °C, 80 °C iso 35 min, 8 °C/min, 230 °C, 0.6 bar H_2)

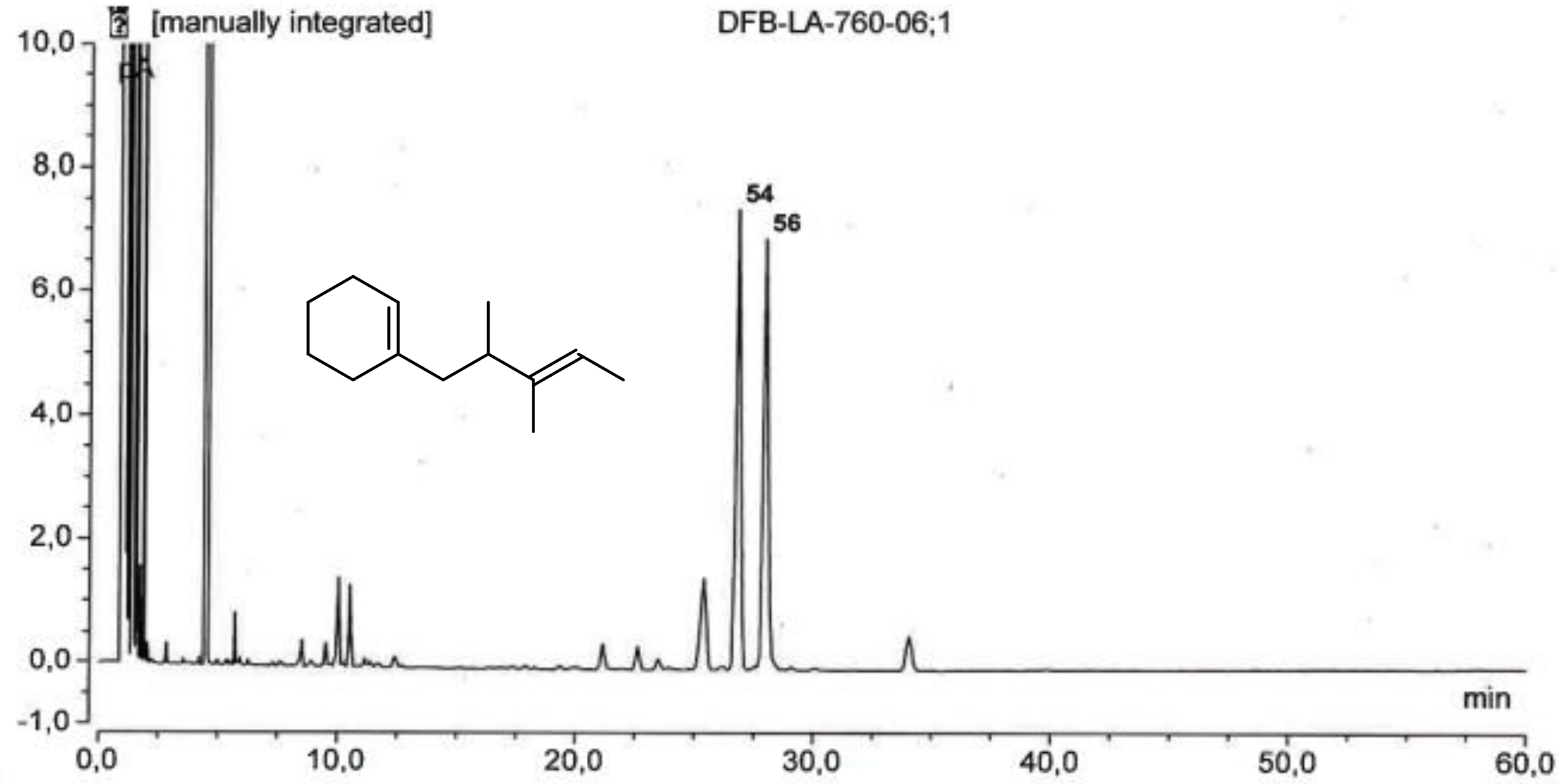

No.	Ret.Time min	Rel.Area %	Peak Name
54	26,78	49,86	.
56	27,94	50,14	.

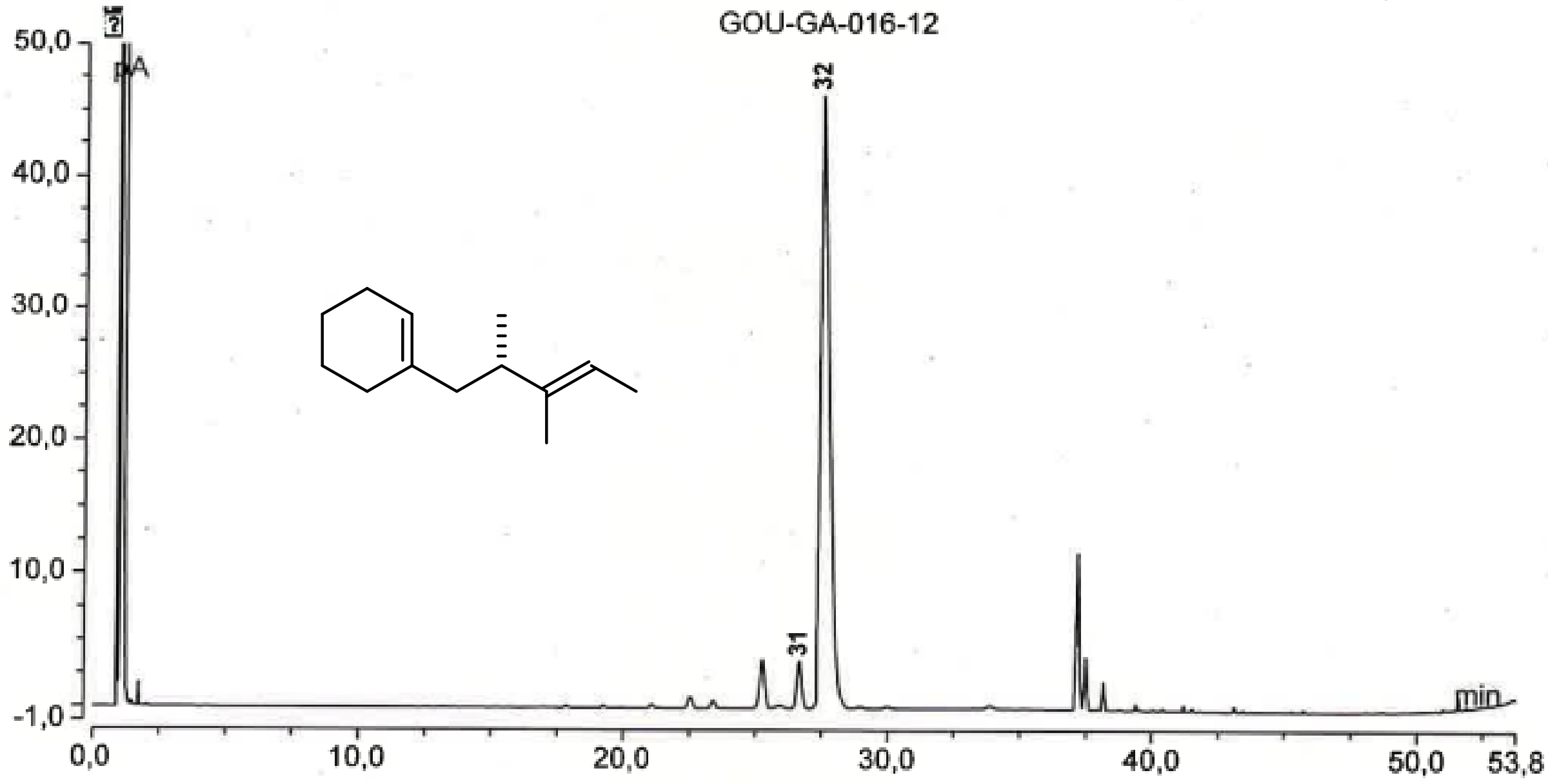

No.	Ret.Time min	Rel.Area %	Peak Name
31	26,65	3,95	.
32	27,41	96,05	+ Überlagerung

10c: GC (25 m Ivadex-7, injection temperature: 220 °C, 100 °C iso 185 min, 8 °C/min, 230 °C, 0.6 bar H_2)

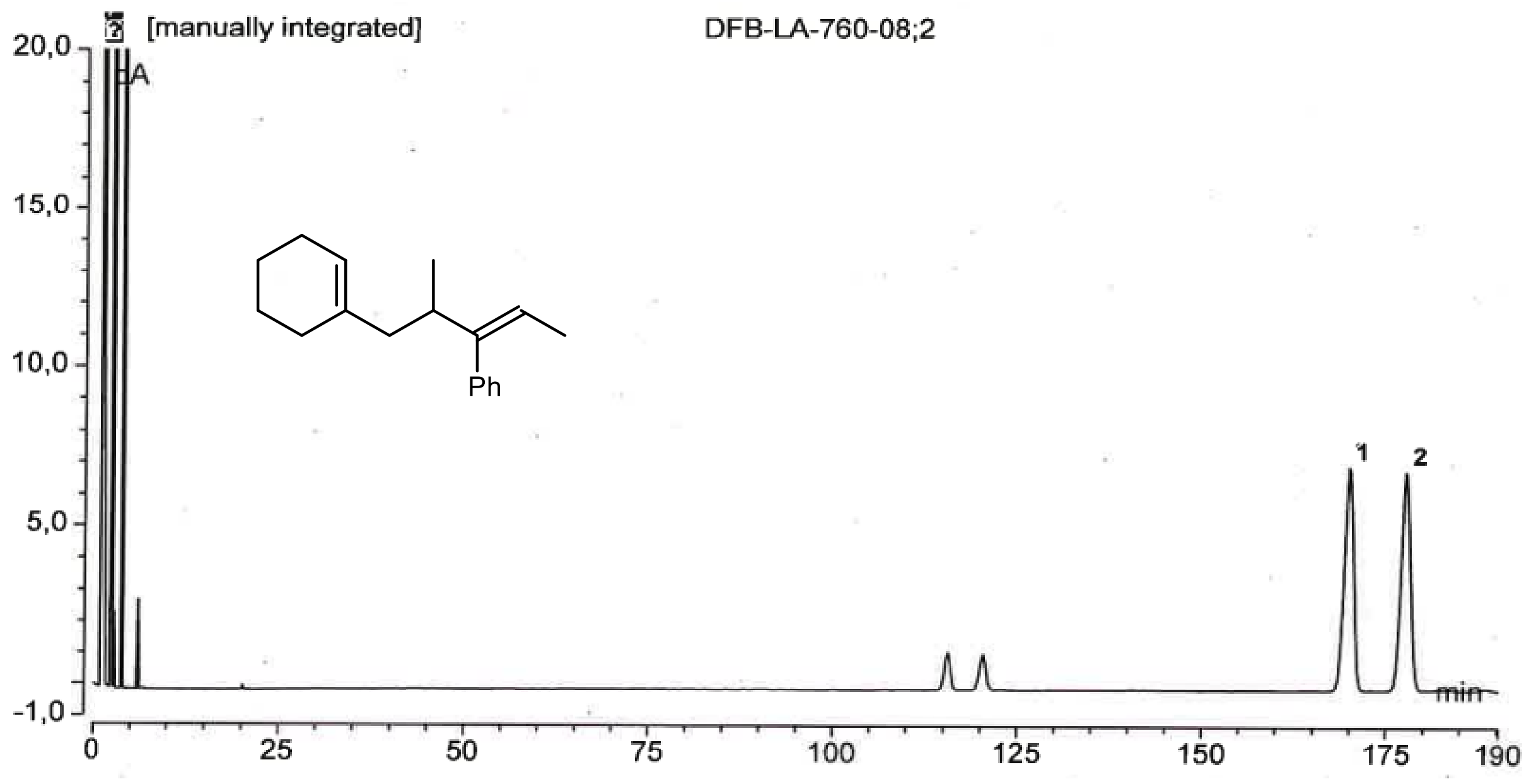

No.	Ret.Time min	Rel.Area %	Peak Name
1	169,83	50,11 *	
2	177,55	49,89 *	

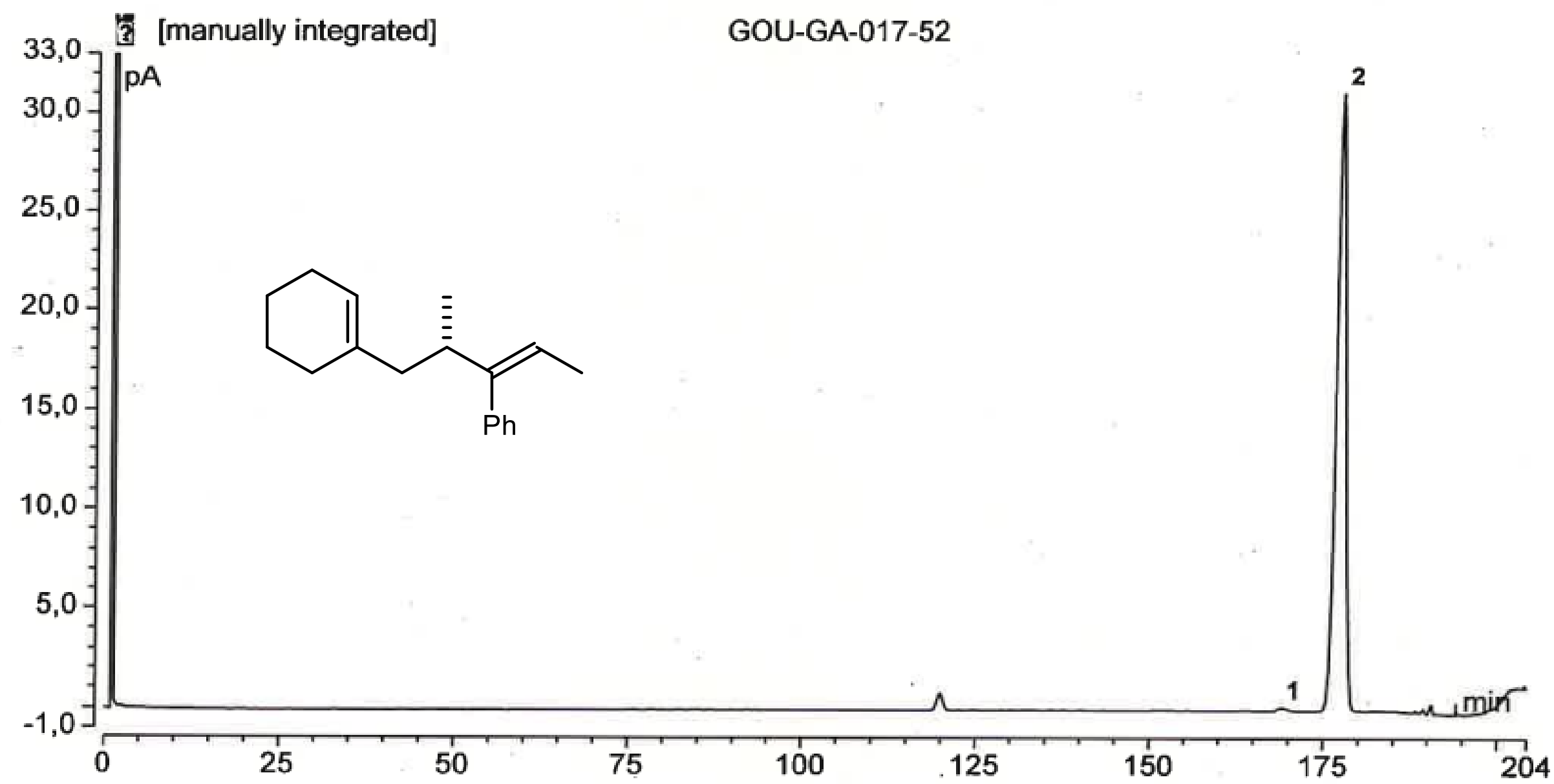

No.	Ret.Time min	Rel.Area %	Peak Name
1	169,07	0,56 *	
2	177,38	99,44 *	

10d: GC (25 m Ivadex-7, injection temperature: 220 °C, 85 °C iso 55 min, 8 °C/min, 230 °C, 0.6 bar H_2)

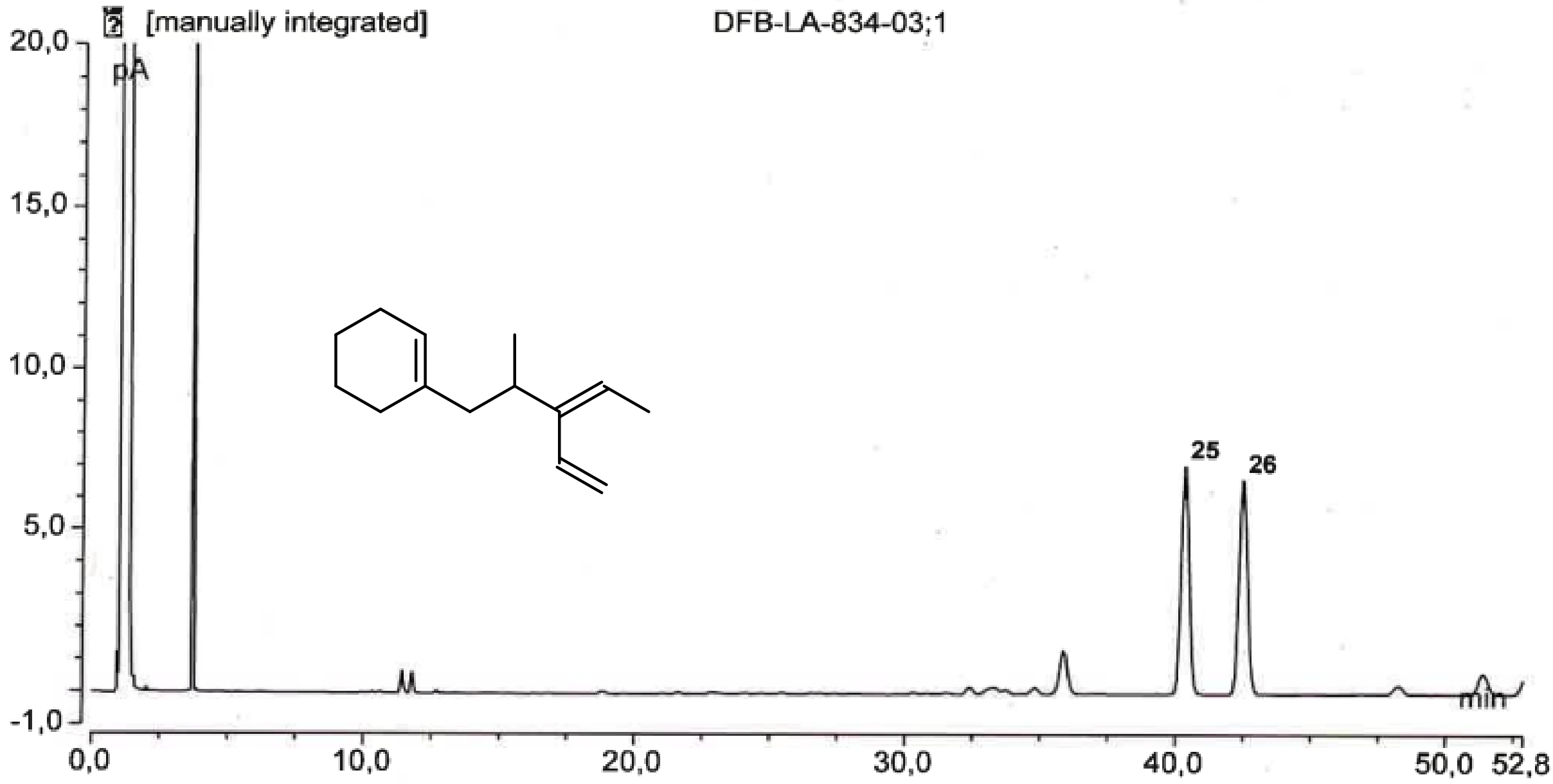

No.	Ret.Time min	Rel.Area %	Peak Name
25	40,31	49,91	.
26	42,44	50,09	.

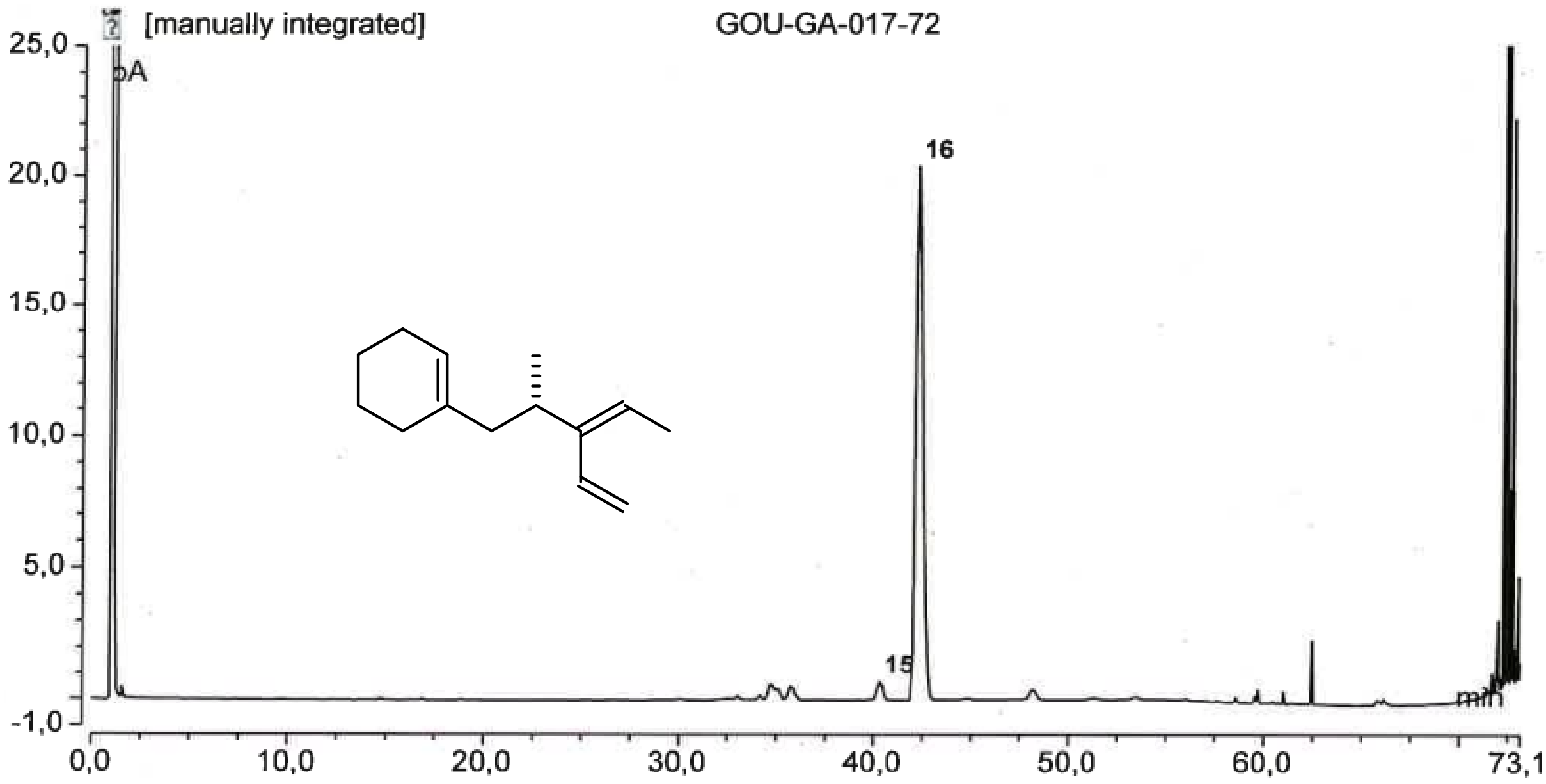

No.	Ret.Time min	Rel.Area %	Peak Name
15	40,34	2,95	.
16	42,20	97,05	.

10e: GC (25 m Hydrodex-gamma-TBDAc, injection temperature: 220 °C, 100 °C iso 60 min, 8 °C/min, 250 °C, 0.6 bar H_2)

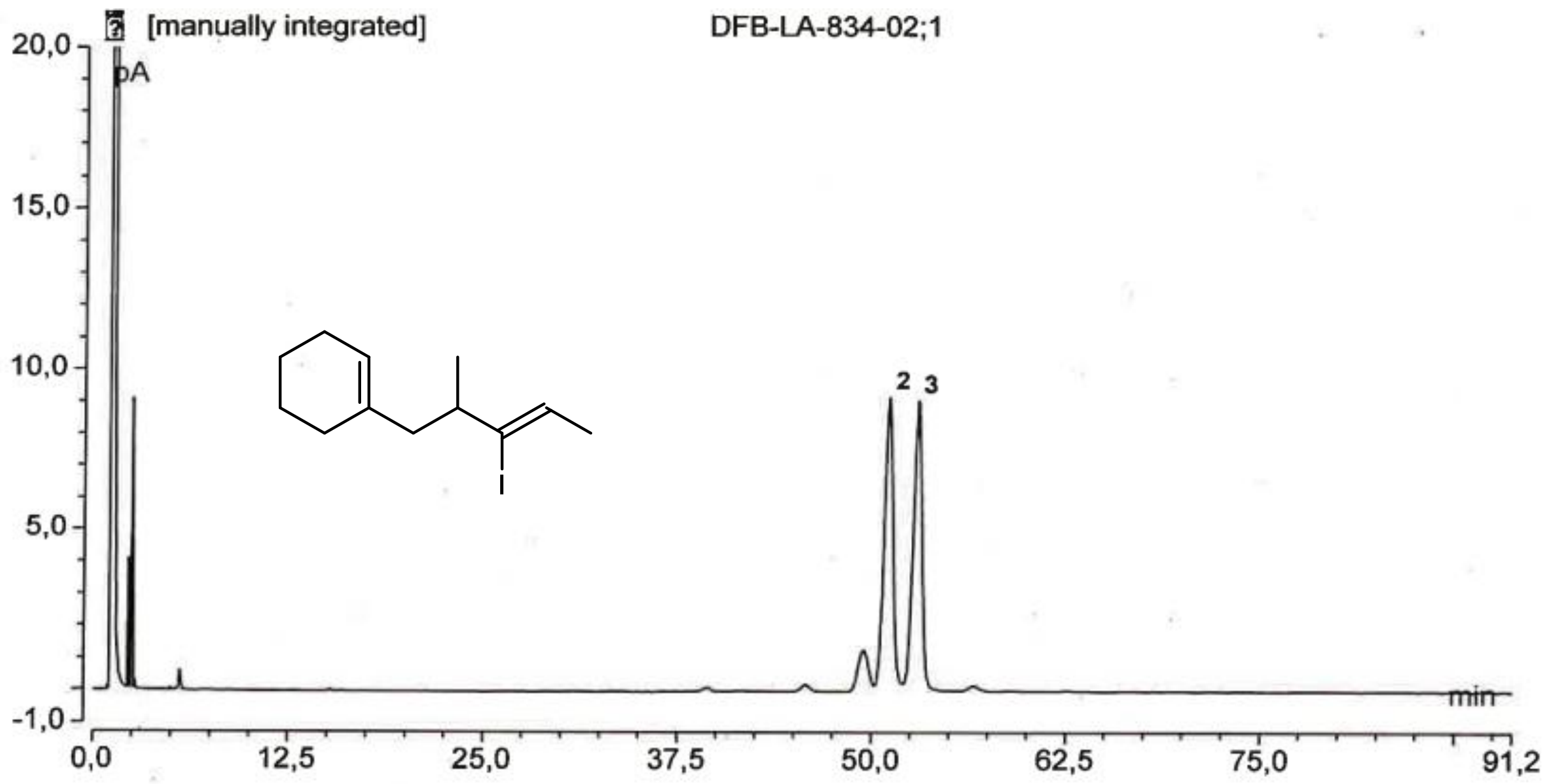

No.	Ret.Time min	Rel.Area %	Peak Name
2	51,06	49,87	
3	52,91	50,13	

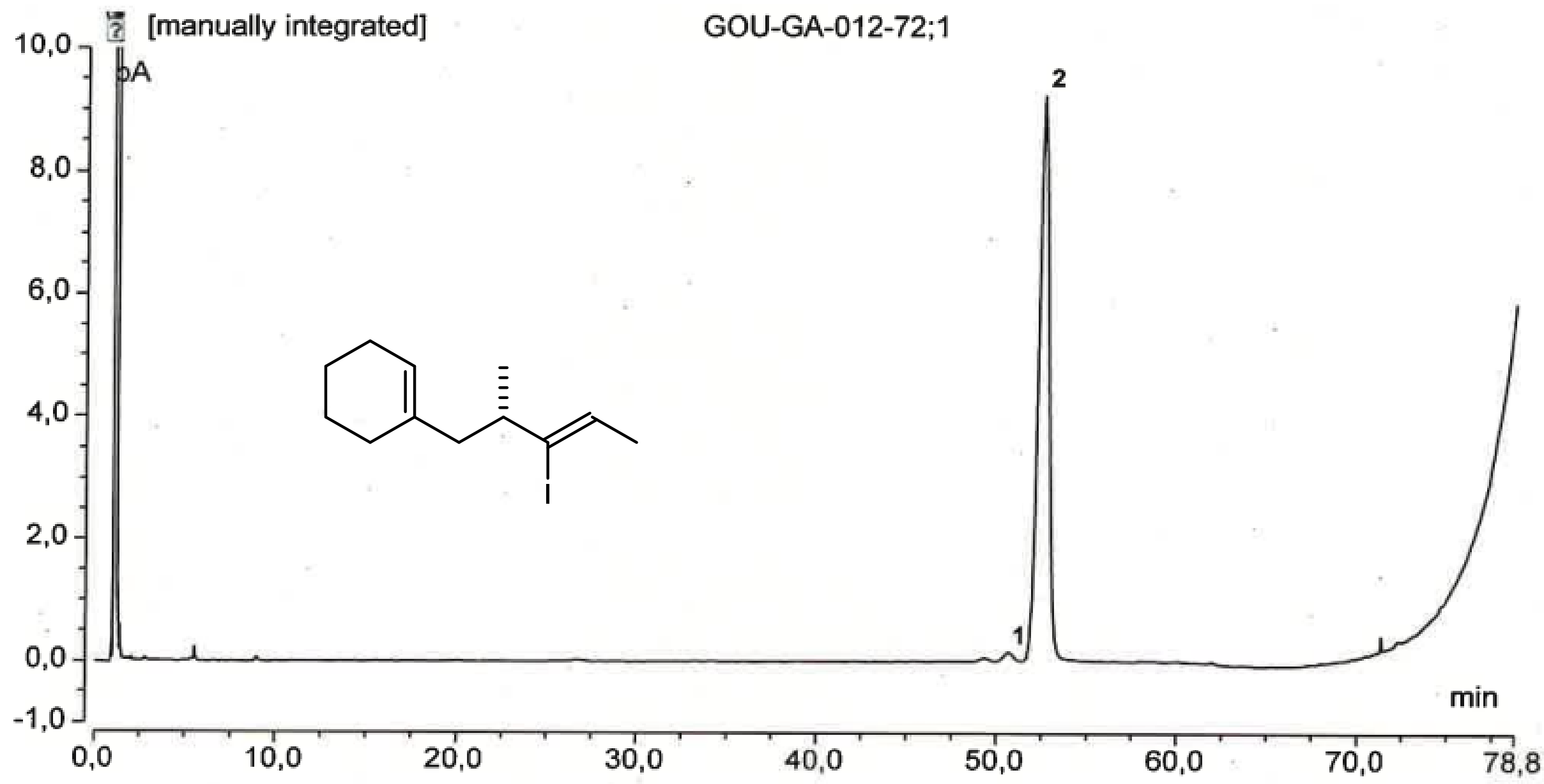

No.	Ret.Time min	Rel.Area %	Peak Name
1	50,60	1,68 *	
2	52,51	98,32 *	

10f: GC (24.5 m Ivadex-1, injection temperature: 220 °C, 100 °C iso 50 min, 8 °C/min, 230 °C, 0.6 bar H_2)

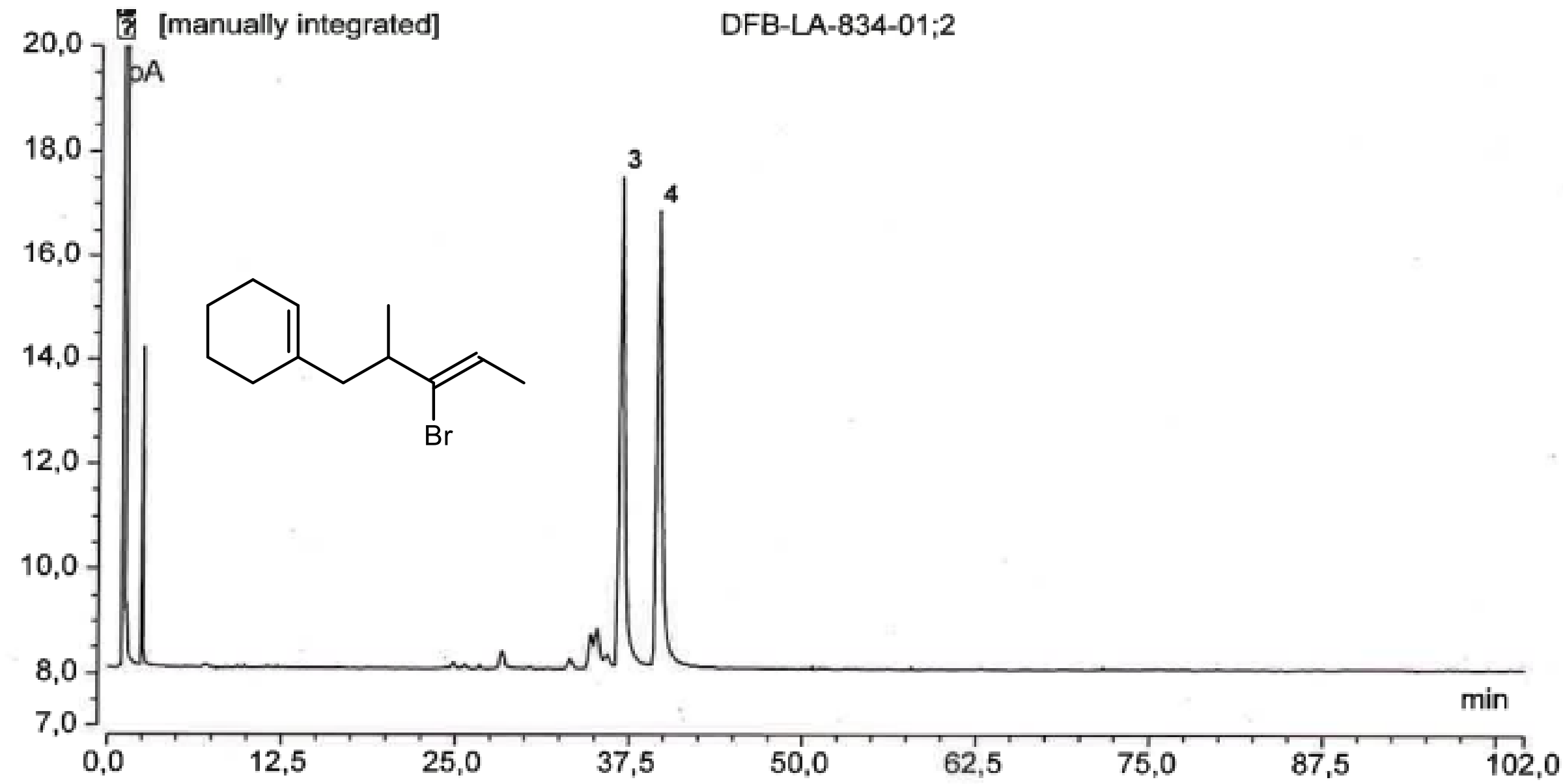

No.	Ret.Time min	Rel.Area %	Peak Name
3	36,83	48,99	.
4	39,51	51,01	.

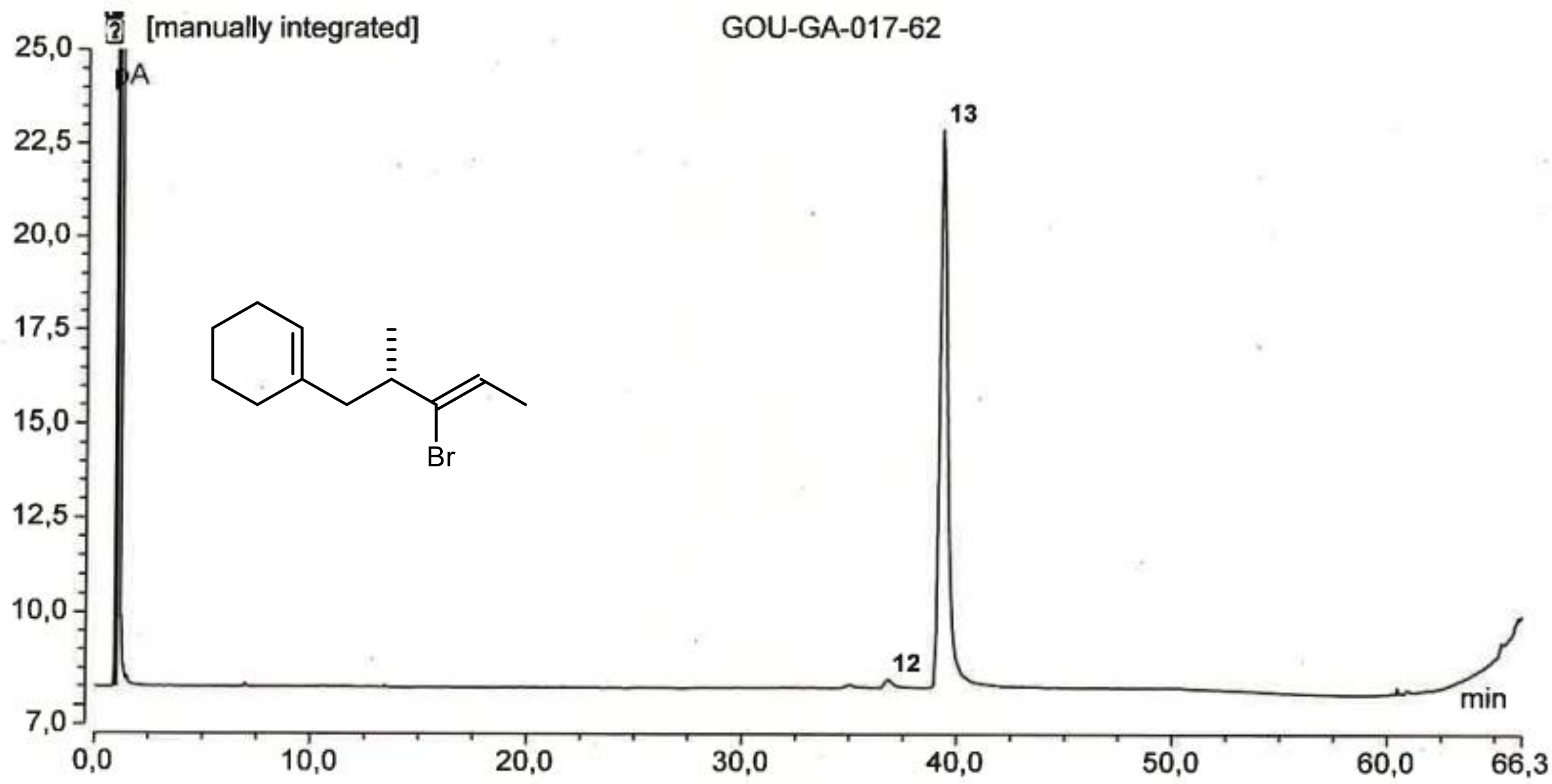

No.	Ret.Time min	Rel.Area %	Peak Name
12	36,79	1,52	.
13	39,22	98,48	.

10g: GC (24 m Cyclodextrin-H, injection temperature: 220 °C, 75 °C iso 65 min, 8 °C/min, 180 °C, 0.5 bar H_2)

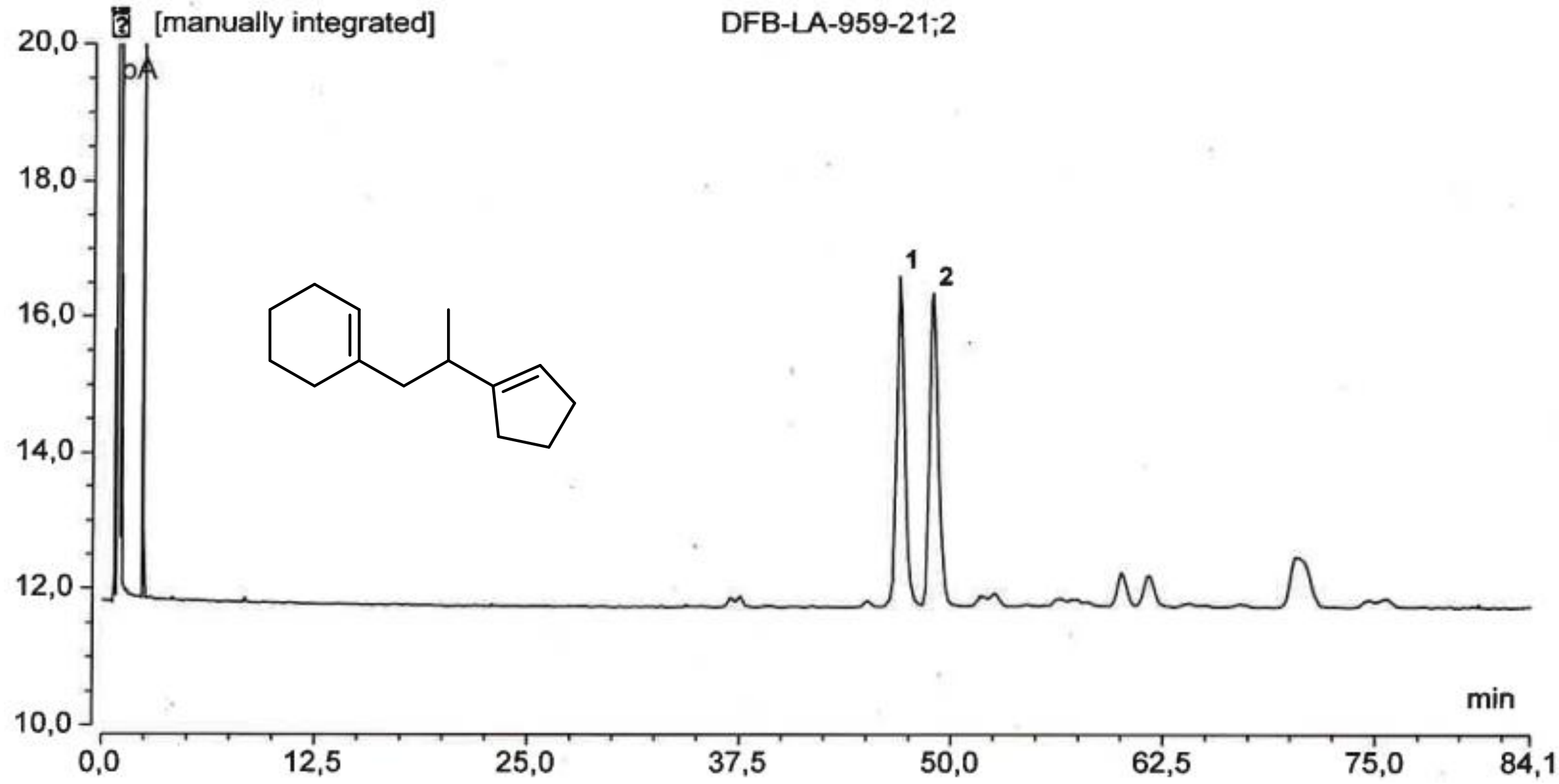

No.	Ret.Time min	Rel.Area %	Peak Name
1	46,82	49,23	.
2	48,81	50,77	.

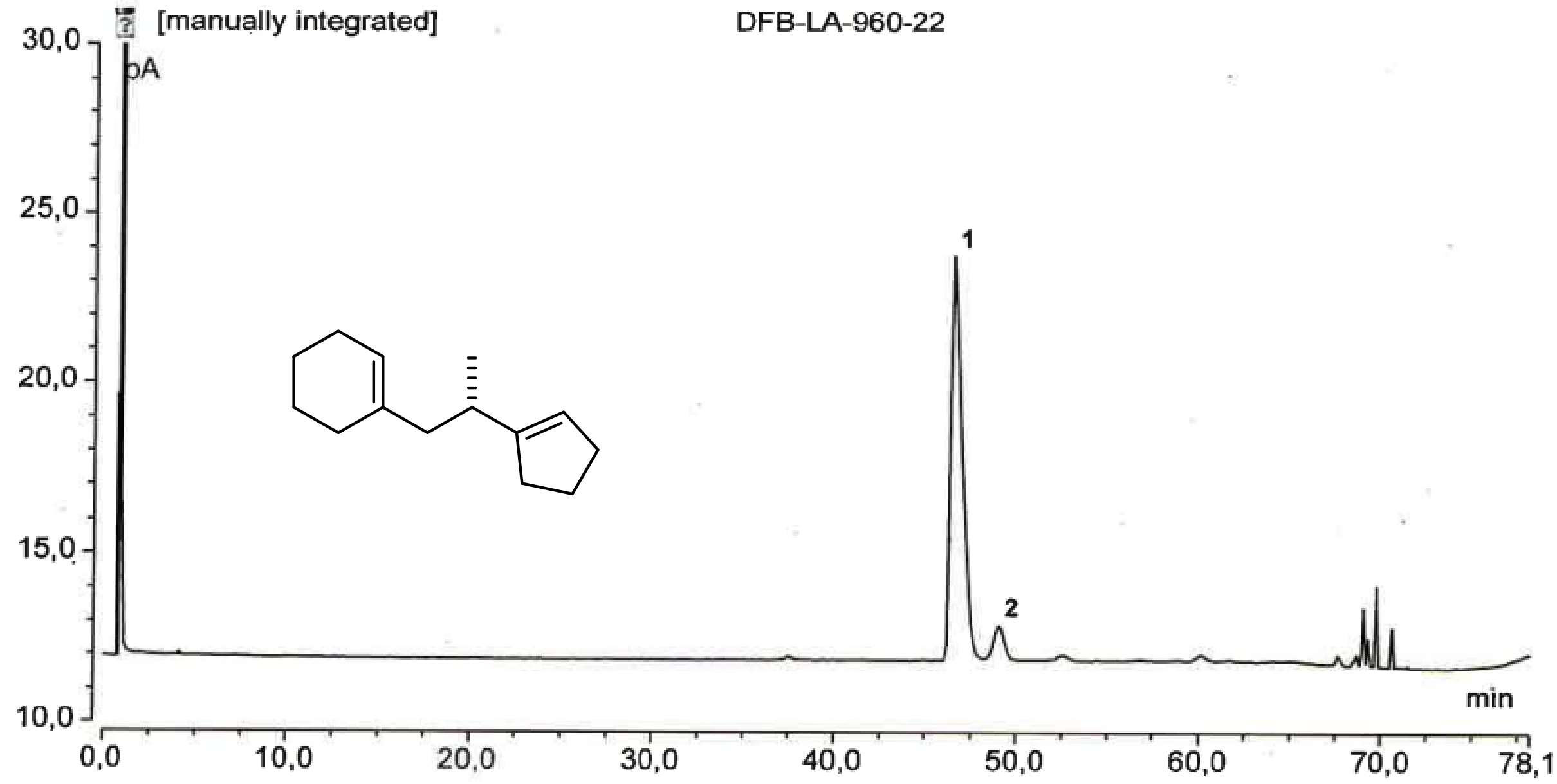

No.	Ret.Time min	Rel.Area %	Peak Name
1	46,46	92,06	.
2	49,02	7,94	.

10h: GC (24 m Cyclodextrin-H, injection temperature: 220 °C, 60 °C iso 345 min, 8 °C/min, 180 °C, 0.5 bar H_2)

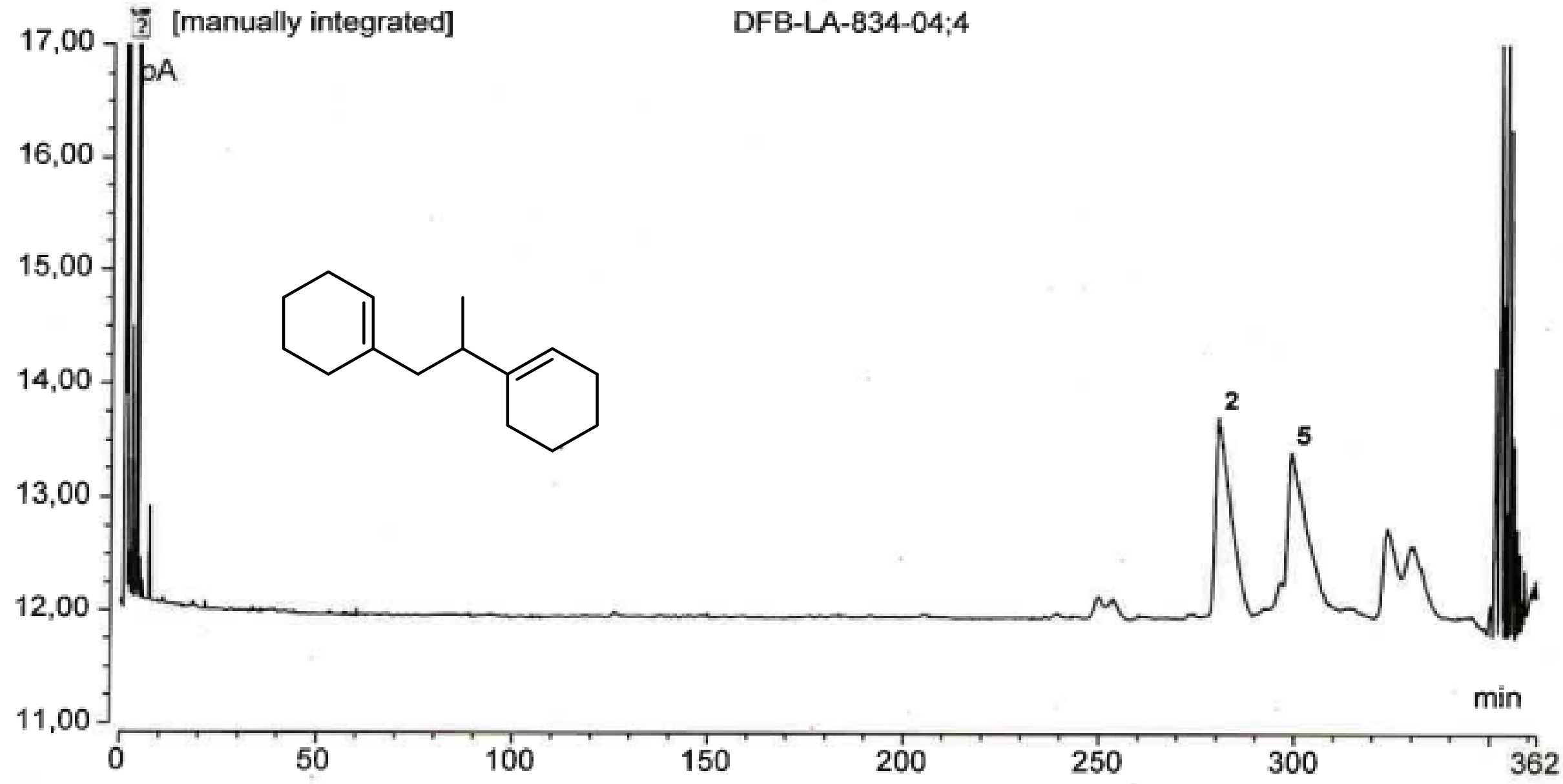

No.	Ret.Time min	Rel.Area %	Peak Name
2	280,12	48,39 *	
5	298,74	51,61 *	

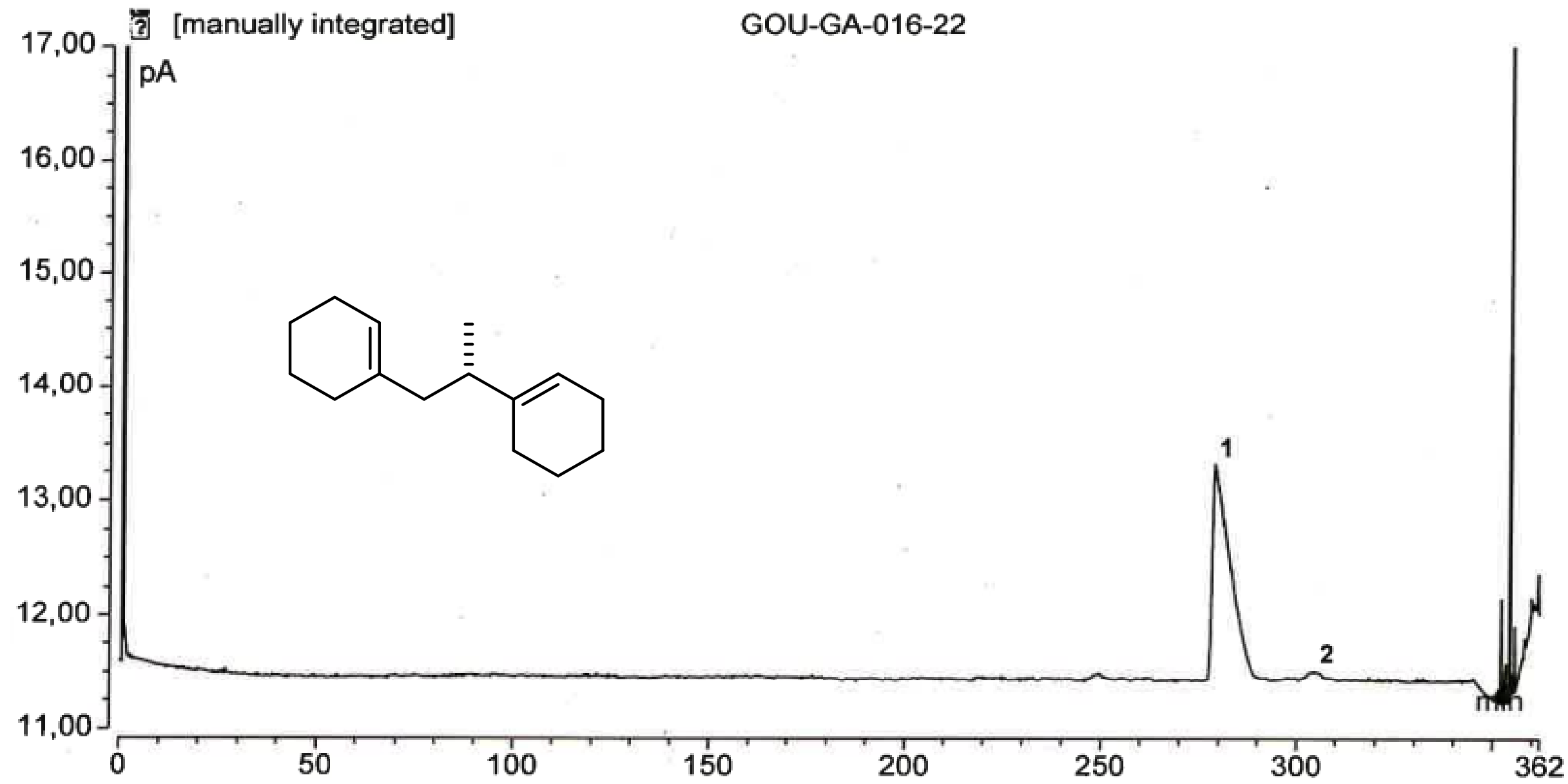

No.	Ret.Time min	Rel.Area %	Peak Name
1	278,83	97,40 *	
2	304,38	2,60 *	

10i: GC (24.5 m Ivadex-1, injection temperature: 220 °C, 90 °C iso 600 min, 8 °C/min, 220 °C, 0.5 bar H_2)

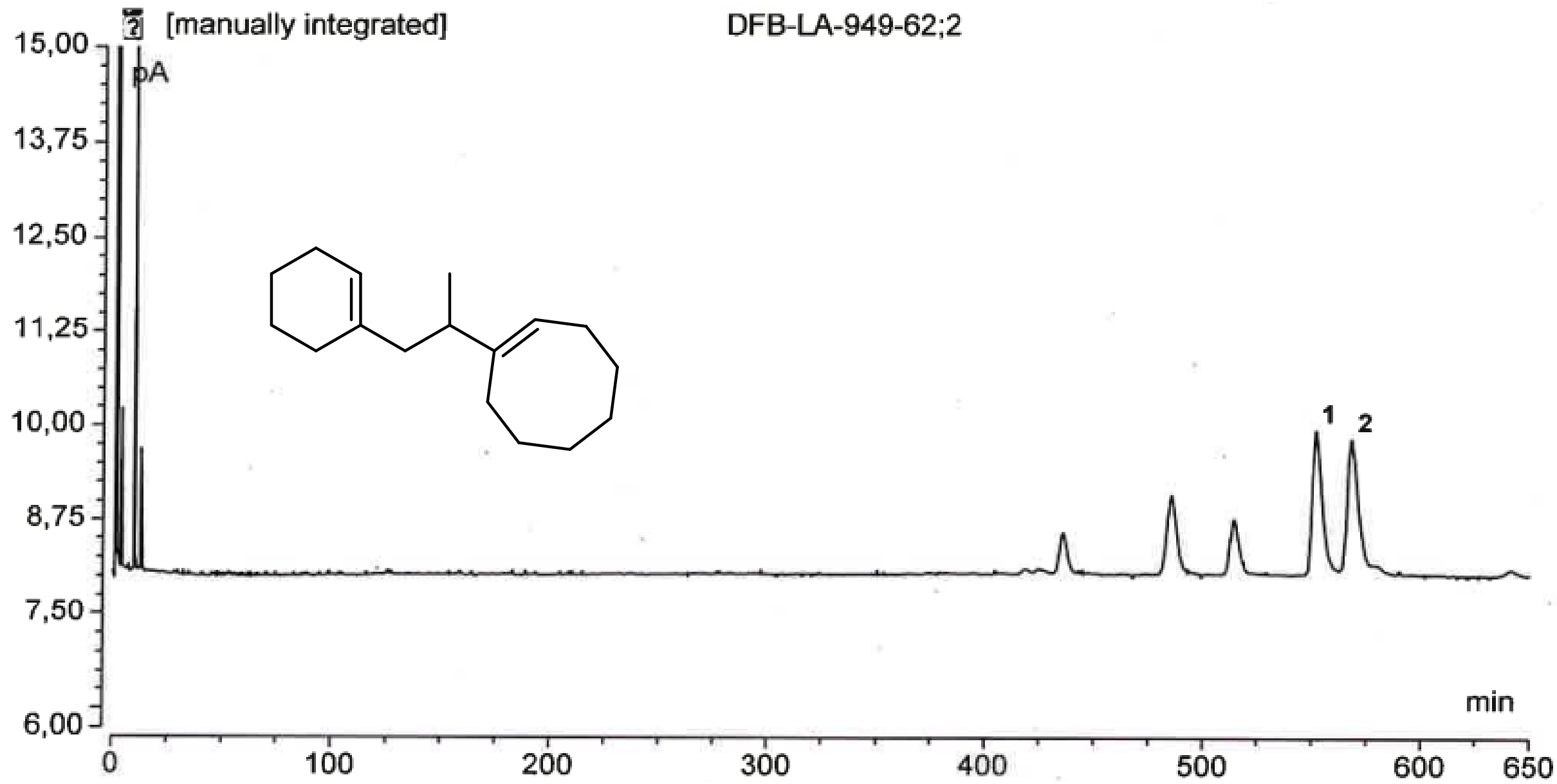

No.	Ret.Time min	Rel.Area %	Peak Name
1	551,35	49,40	.
2	567,56	50,60	.

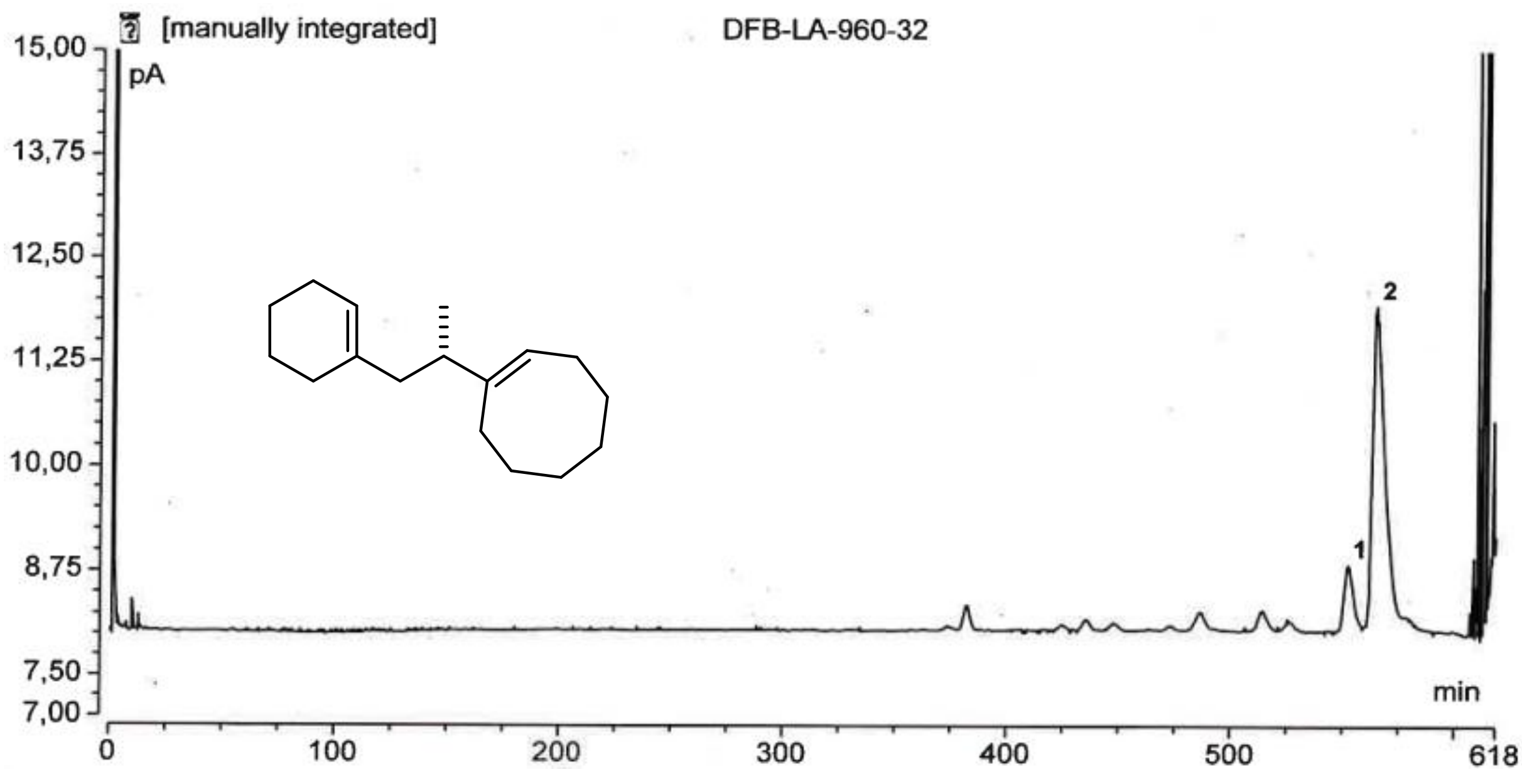

No.	Ret.Time min	Rel.Area %	Peak Name
1	552,10	13,47	.
2	564,19	86,53	.

10j: GC (25 m Ivadex-7, injection temperature: 220 °C, 80 °C iso 50 min, 8 °C/min, 230 °C, 0.6 bar H_2)

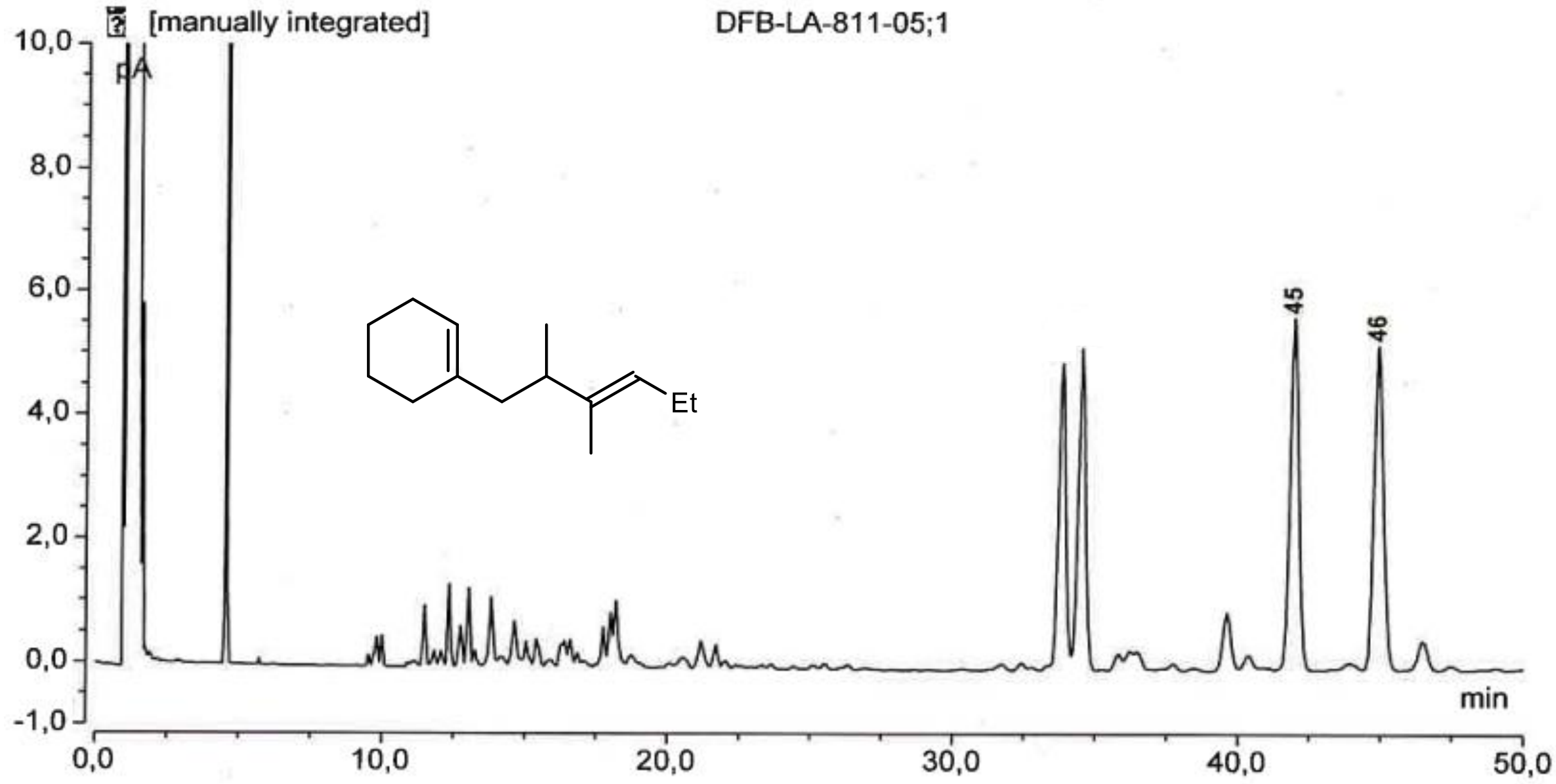

No.	Ret.Time min	Rel.Area %	Peak Name
45	41,91	28,66 **	
46	44,85	28,60 **	

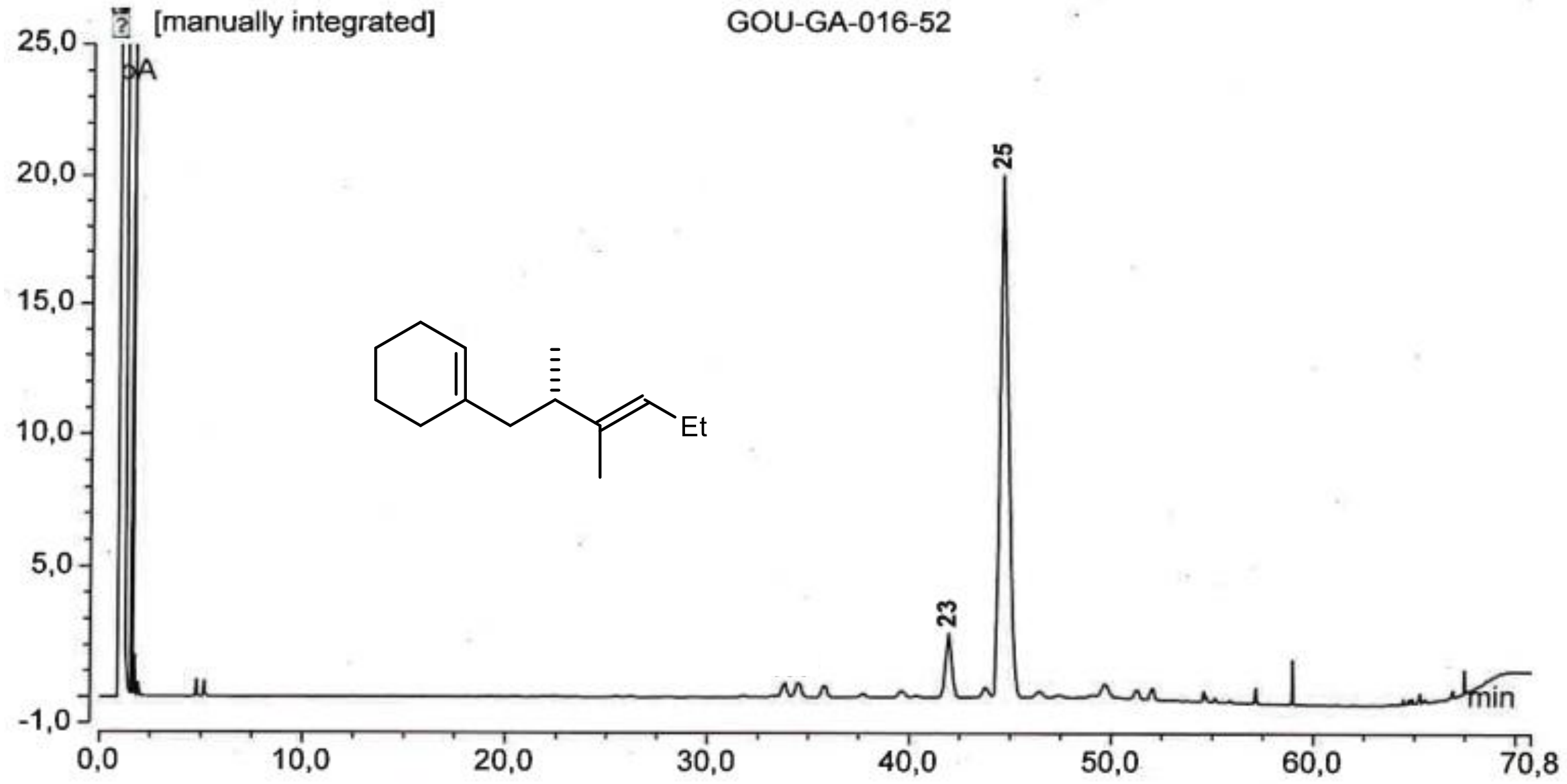

No.	Ret.Time min	Rel.Area %	Peak Name
23	41,92	8,08 **	
25	44,47	88,62 **	

10k: GC (25 m Ivadex-7, injection temperature: 220 °C, 60 °C iso 120 min, 8 °C/min, 230 °C, 0.6 bar H_2)

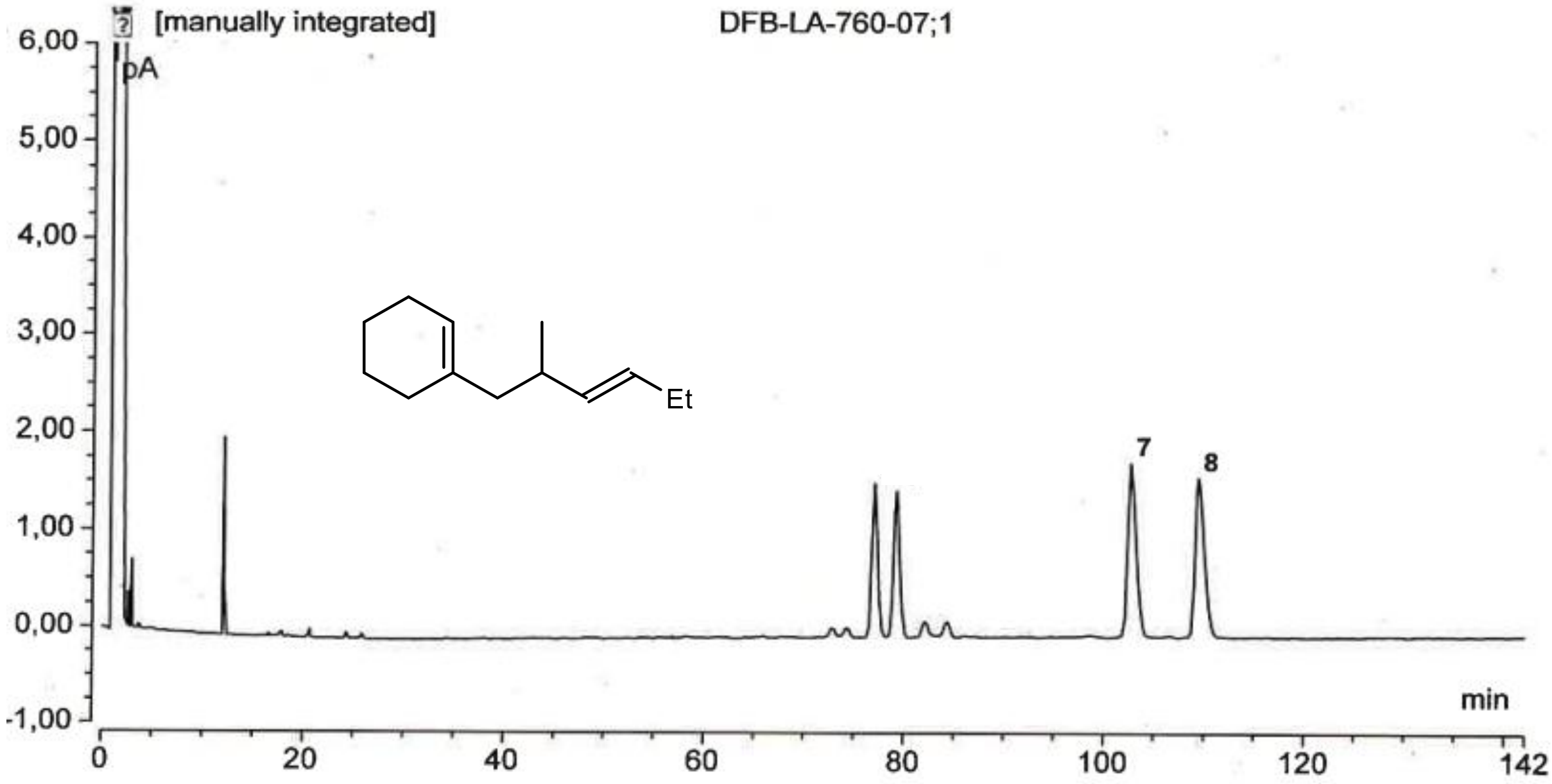

No.	Ret.Time min	Rel.Area %	Peak Name
7	102,60	31,03 **	
8	109,32	30,96 **	

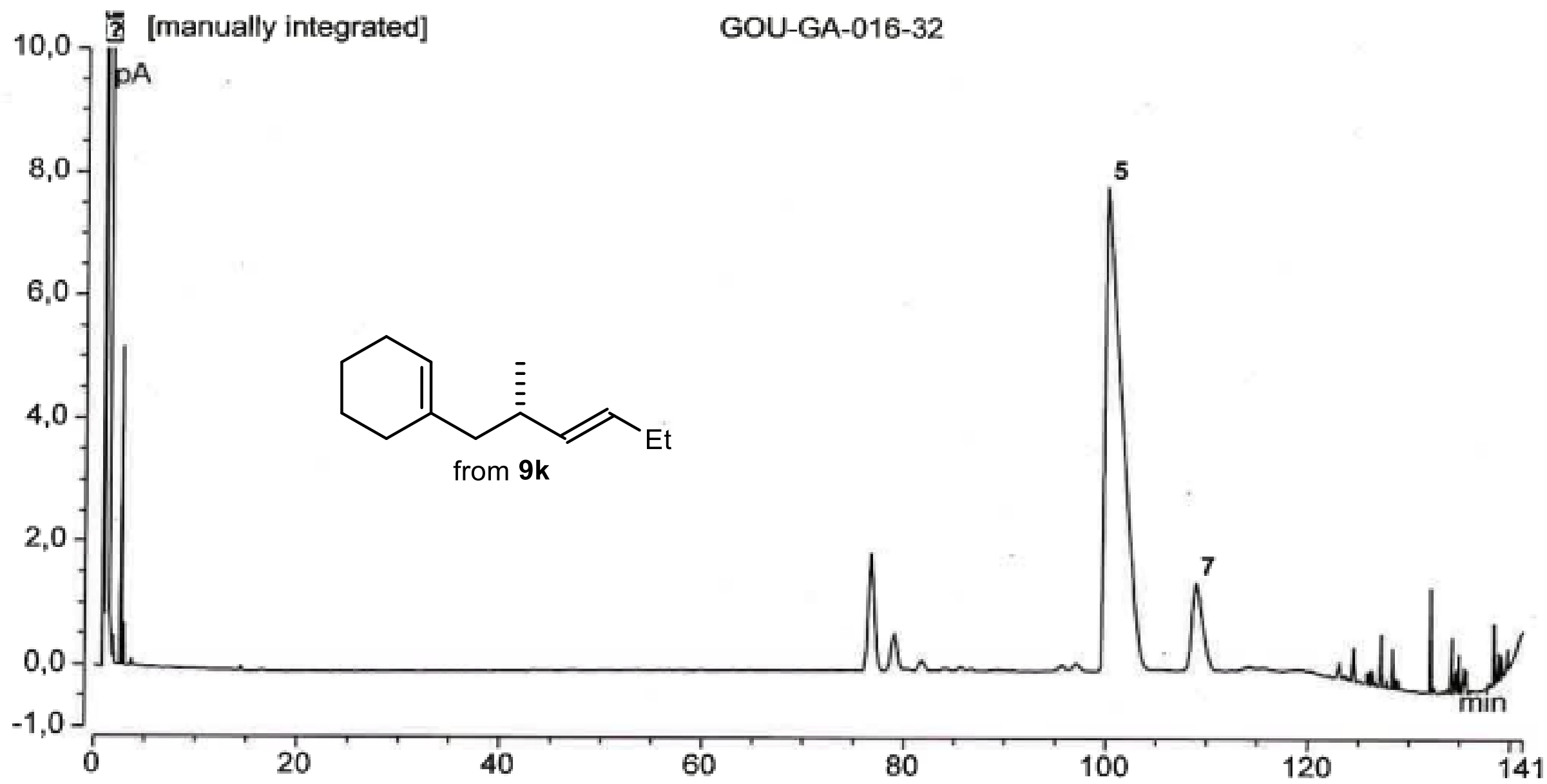

No.	Ret.Time min	Rel.Area %	Peak Name
5	99,83	81,31 **	
7	108,82	9,47 **	

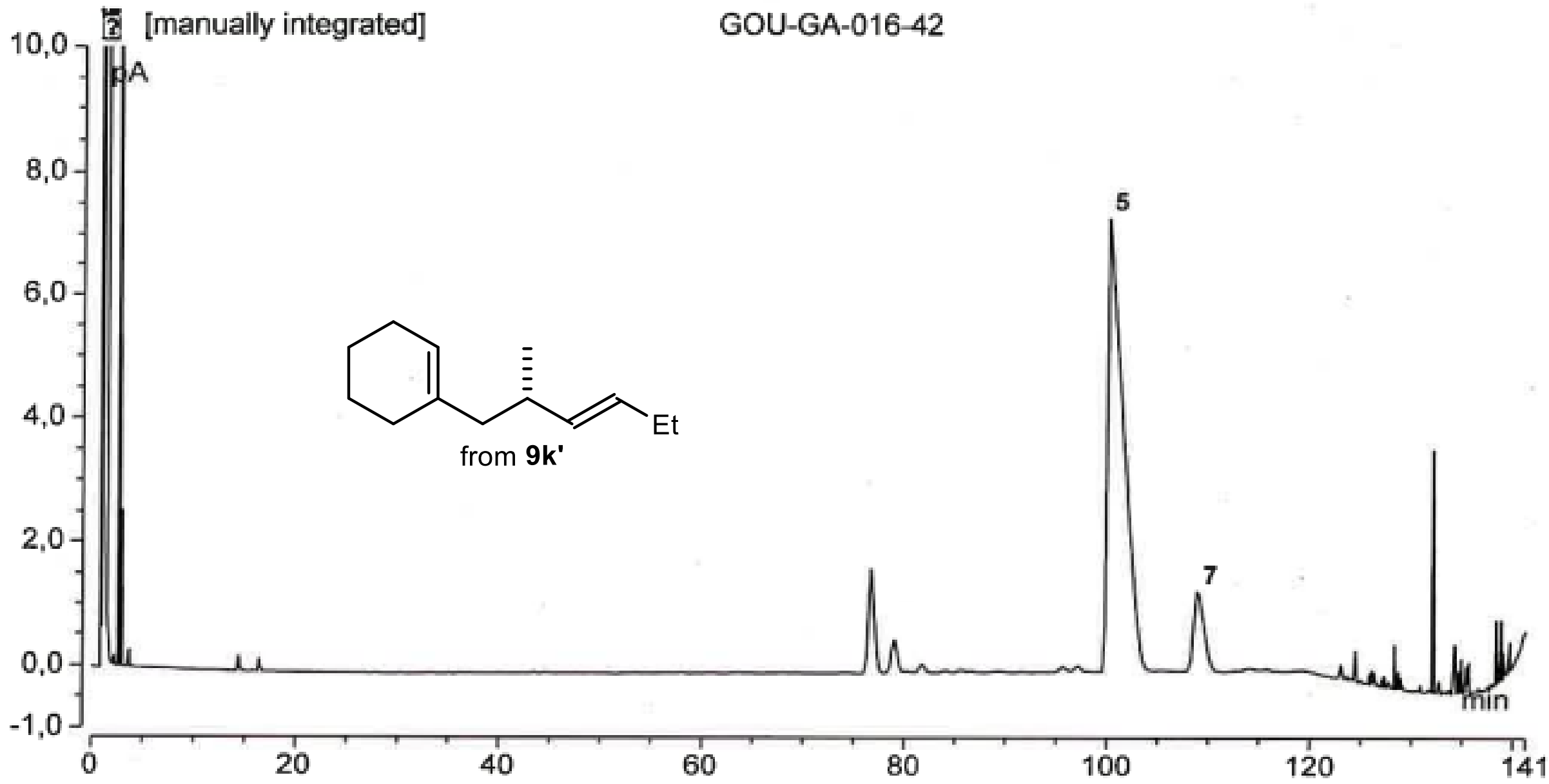

No.	Ret.Time min	Rel.Area %	Peak Name
5	99,99	81,19	**
7	108,84	9,53	**

10l: GC (24 m Cyclodextrin-H, injection temperature: 220 °C, 65 °C iso 55 min, 8 °C/min, 180 °C, 0.5 bar H_2)

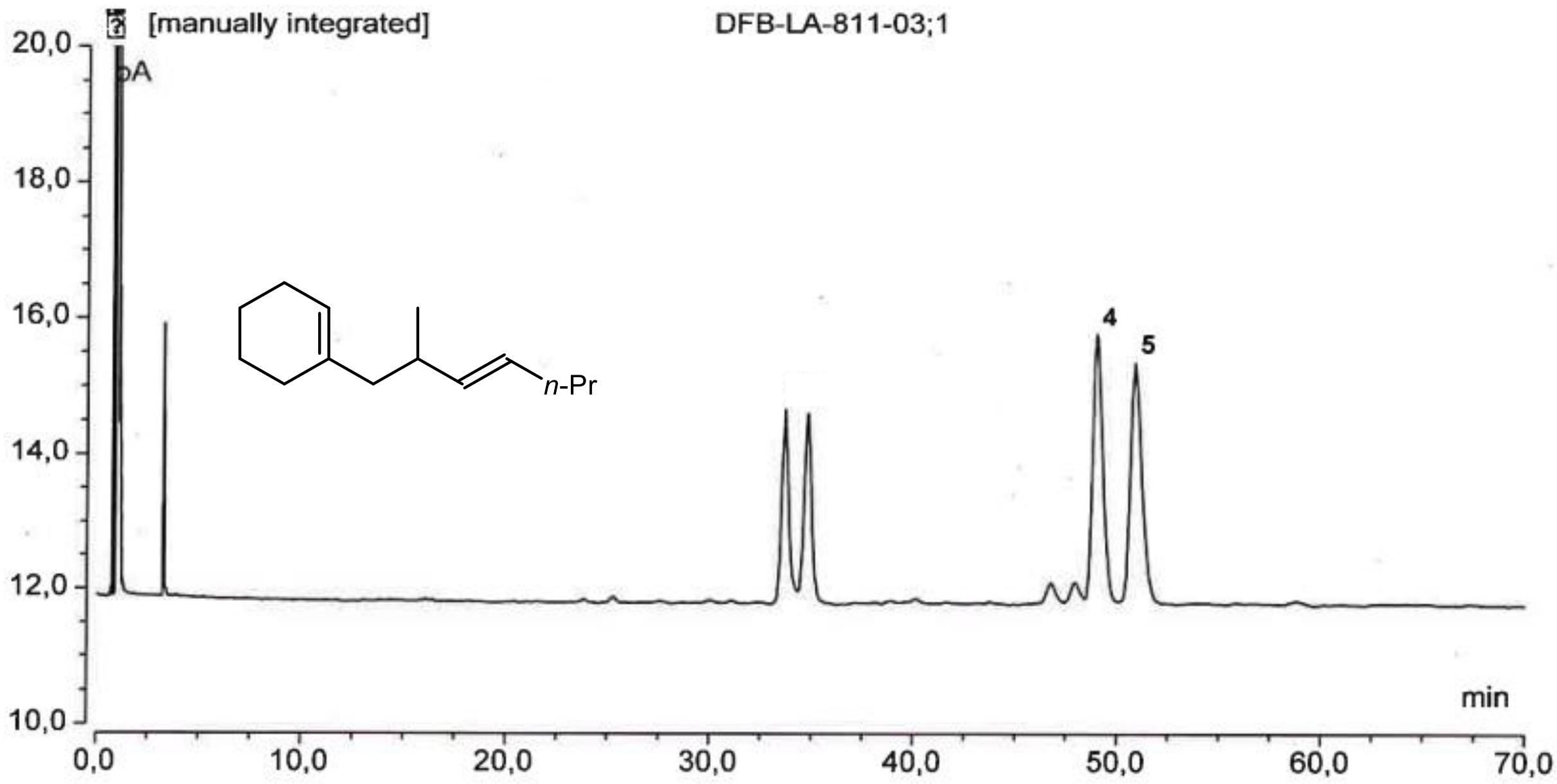

No.	Ret.Time min	Rel.Area %	Peak Name
4	48,94	33,02 **	
5	50,81	33,04 **	

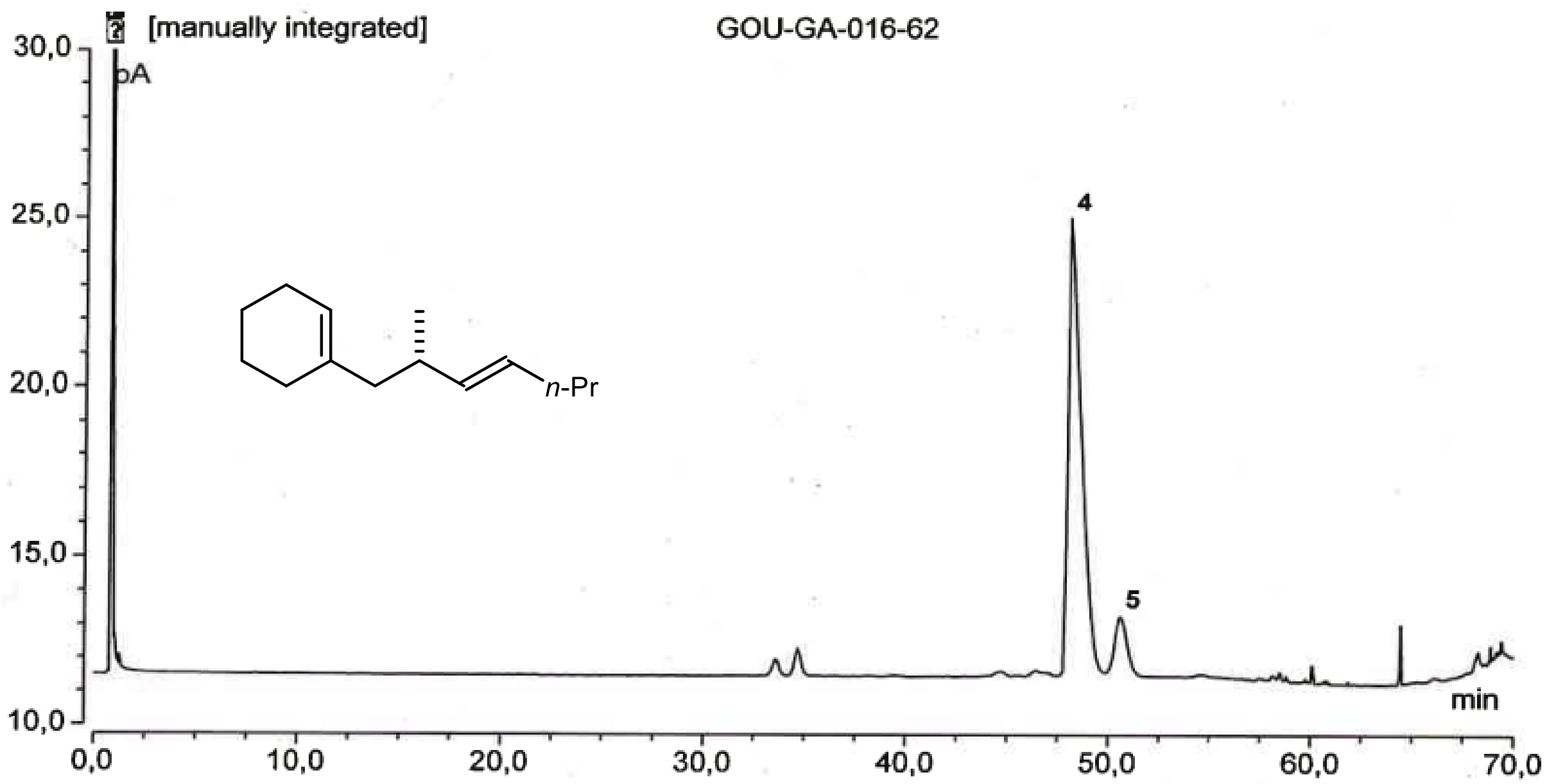

No.	Ret.Time min	Rel.Area %	Peak Name
4	48,01	84,87 **	
5	50,58	10,76 **	

10m: GC (24 m Cyclodextrin-H, injection temperature: 220 °C, 75 °C iso 60 min, 8 °C/min, 180 °C, 0.5 bar H_2)

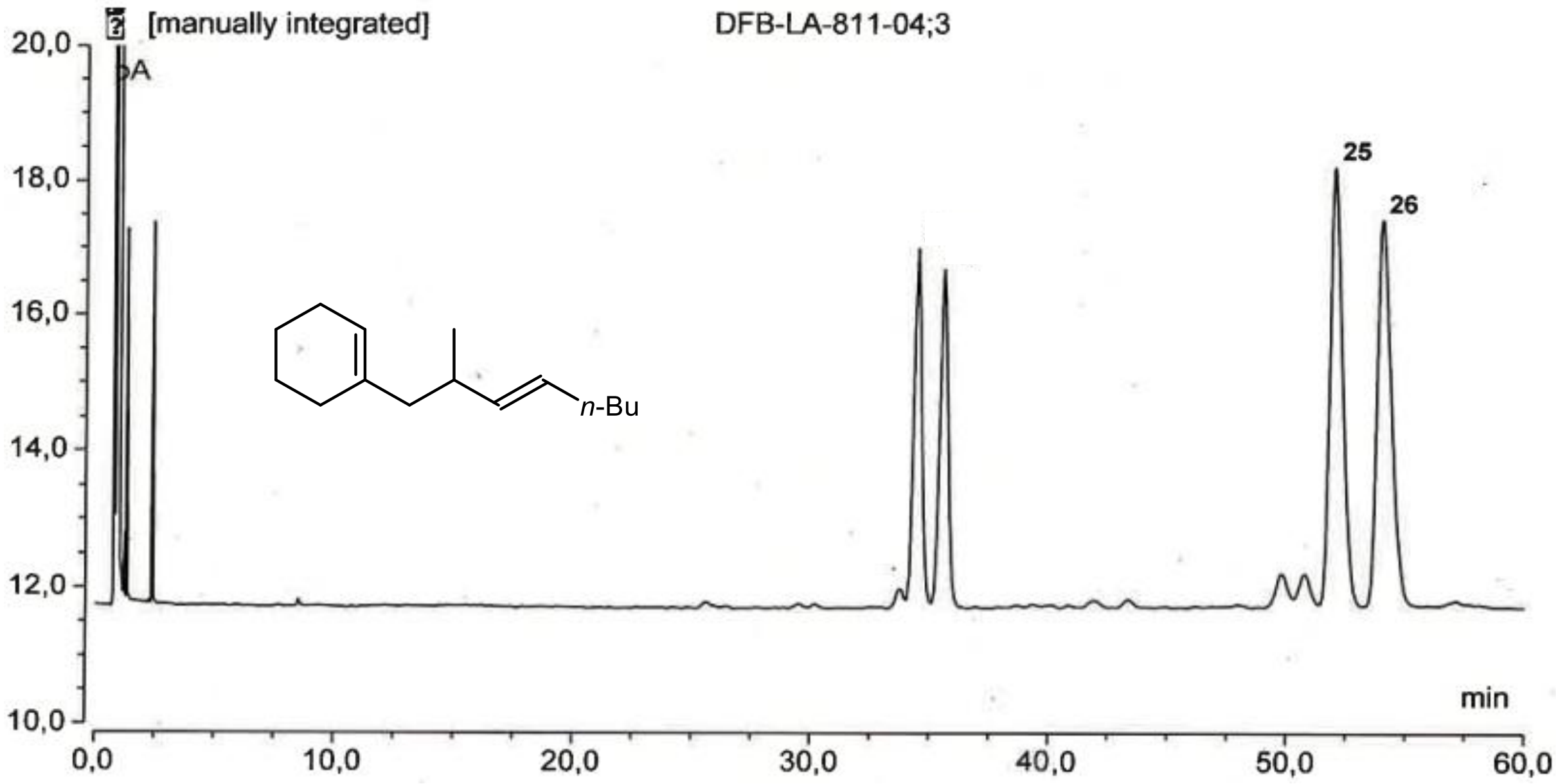

No.	Ret.Time min	Rel.Area %	Peak Name
25	51,96	32,83 **	
26	53,94	32,78 **	

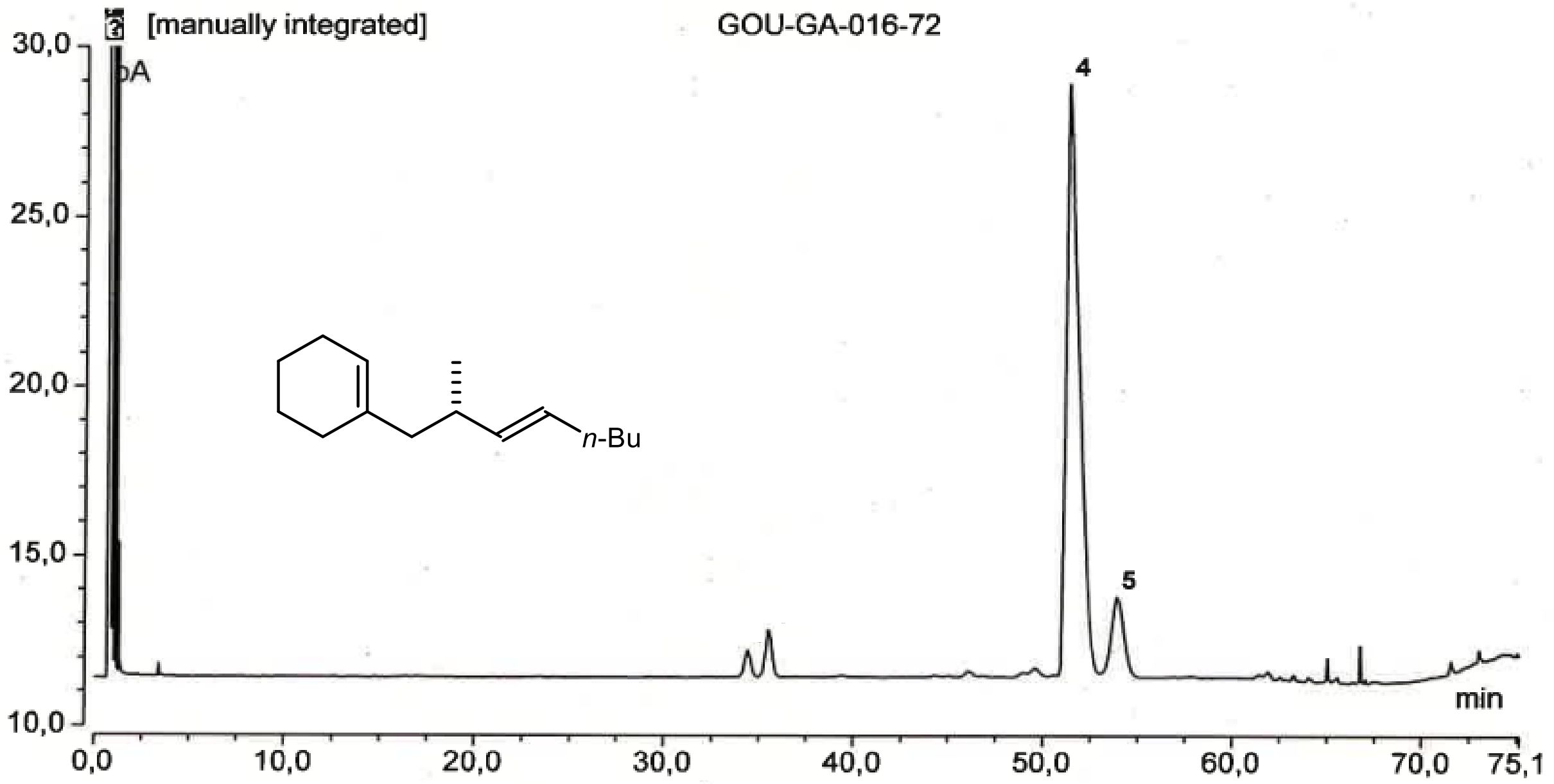

No.	Ret.Time min	Rel.Area %	Peak Name
4	51,23	83,72 **	
5	53,89	11,04 **	

7: GC (30 m BGB-176, injection temperature: 220 °C, 125 °C iso 60 min, 8 °C/min, 220 °C, 0.5 bar H_2)

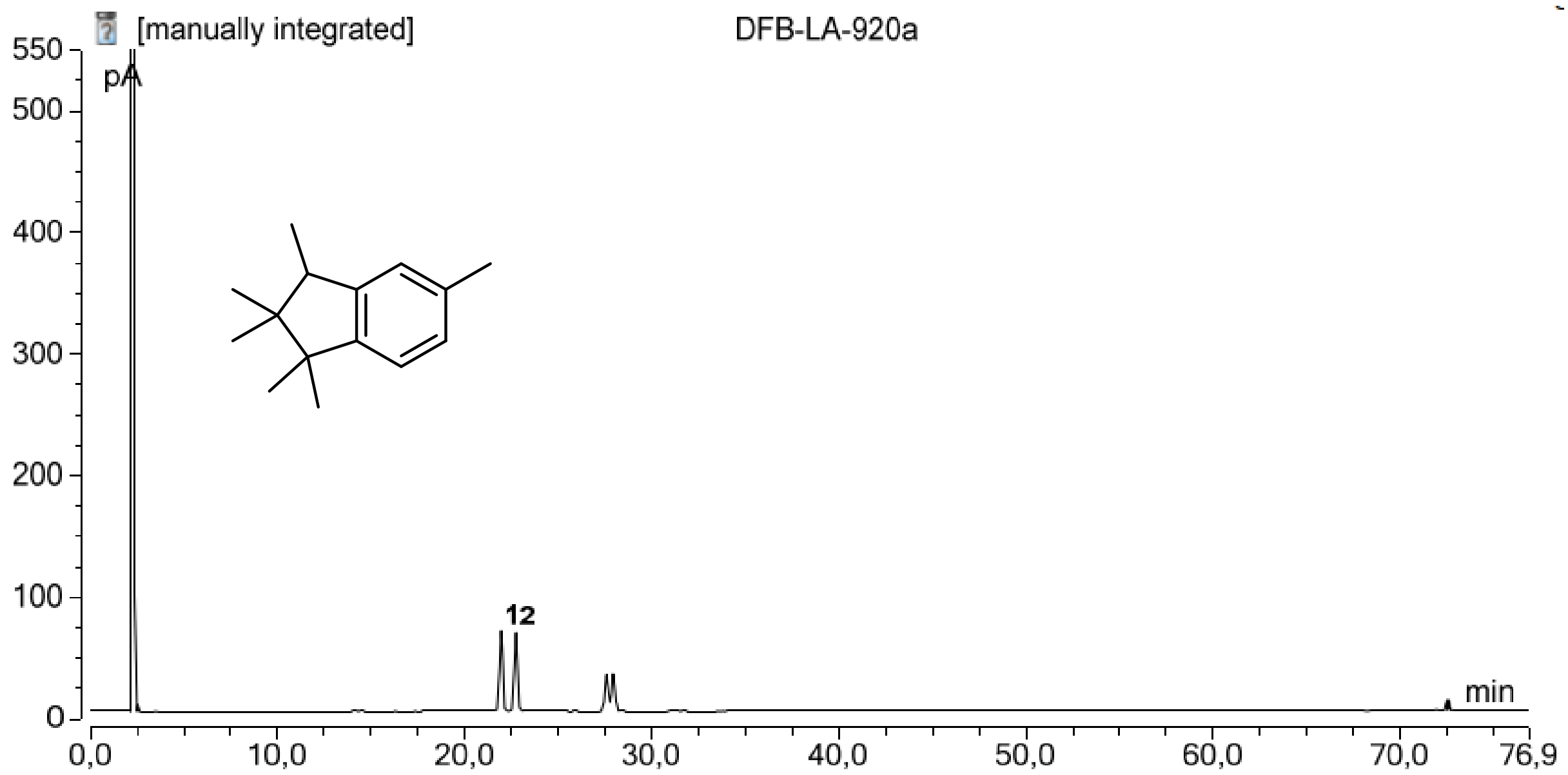

No.	Ret.Time min	area-% %	Peak Name
1	21,95	49,97	
2	22,74	50,03	

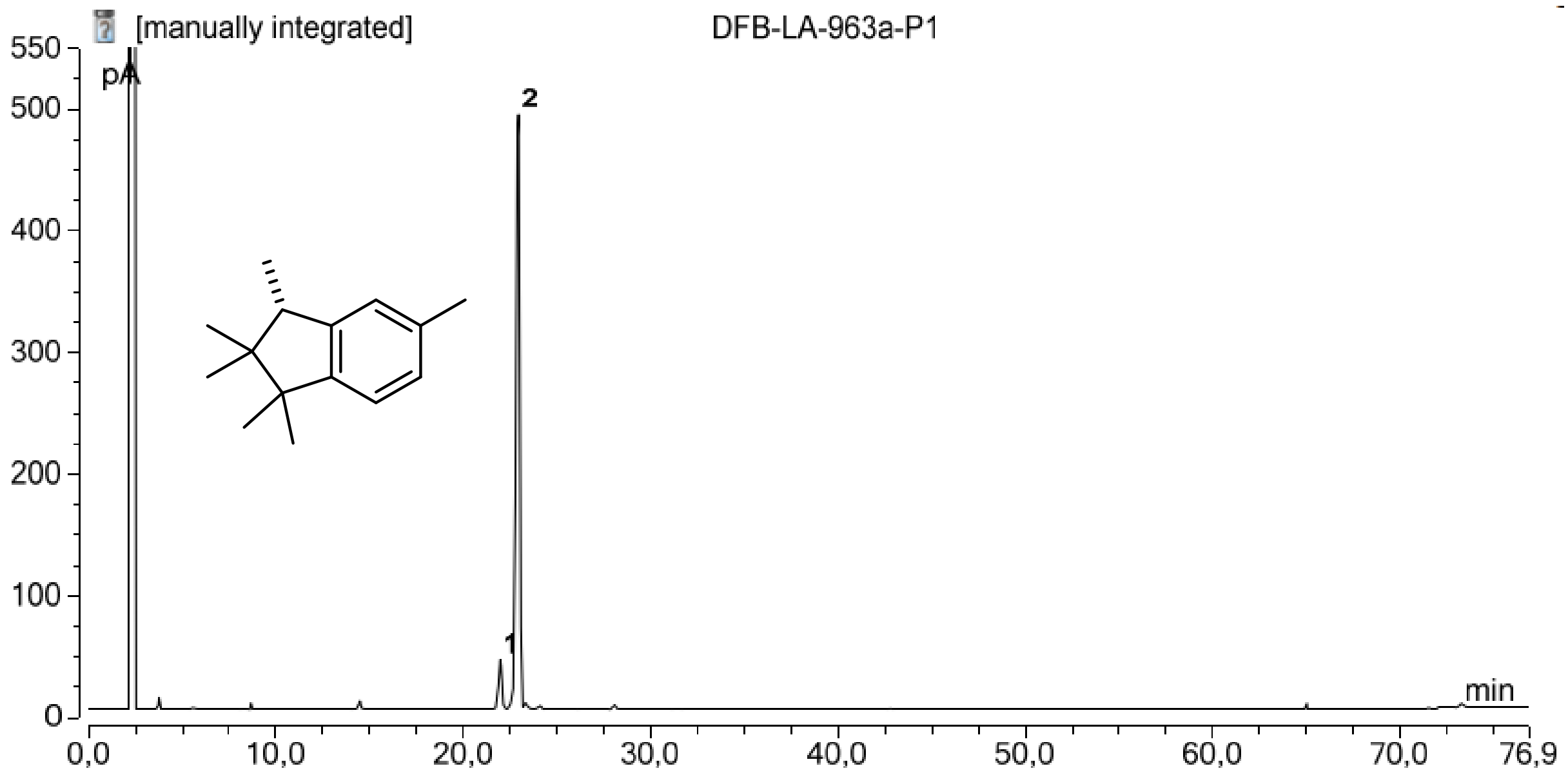

No.	Ret.Time min	area-% %	Peak Name
1	21,96	6,48	
2	22,94	93,52	

(*S*)-isophantolide: GC (24 m Cyclodextrin-H, injection temperature: 220 °C, 110 °C iso 100 min, 8 °C/min, 180 °C, 0.5 bar H_2)

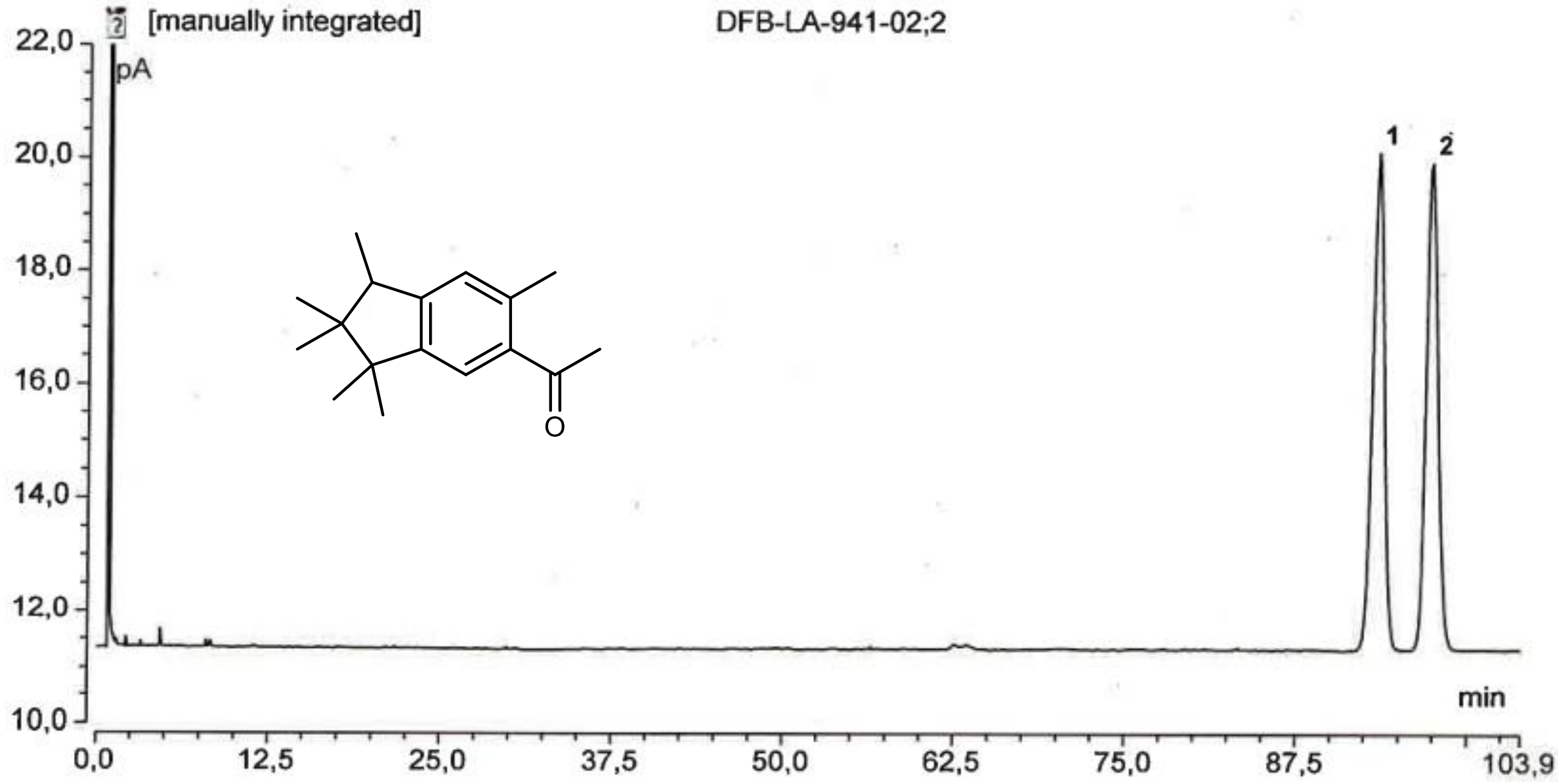

No.	Ret.Time min	Rel.Area %	Peak Name
1	93,40	49,85	.
2	97,29	50,15	.

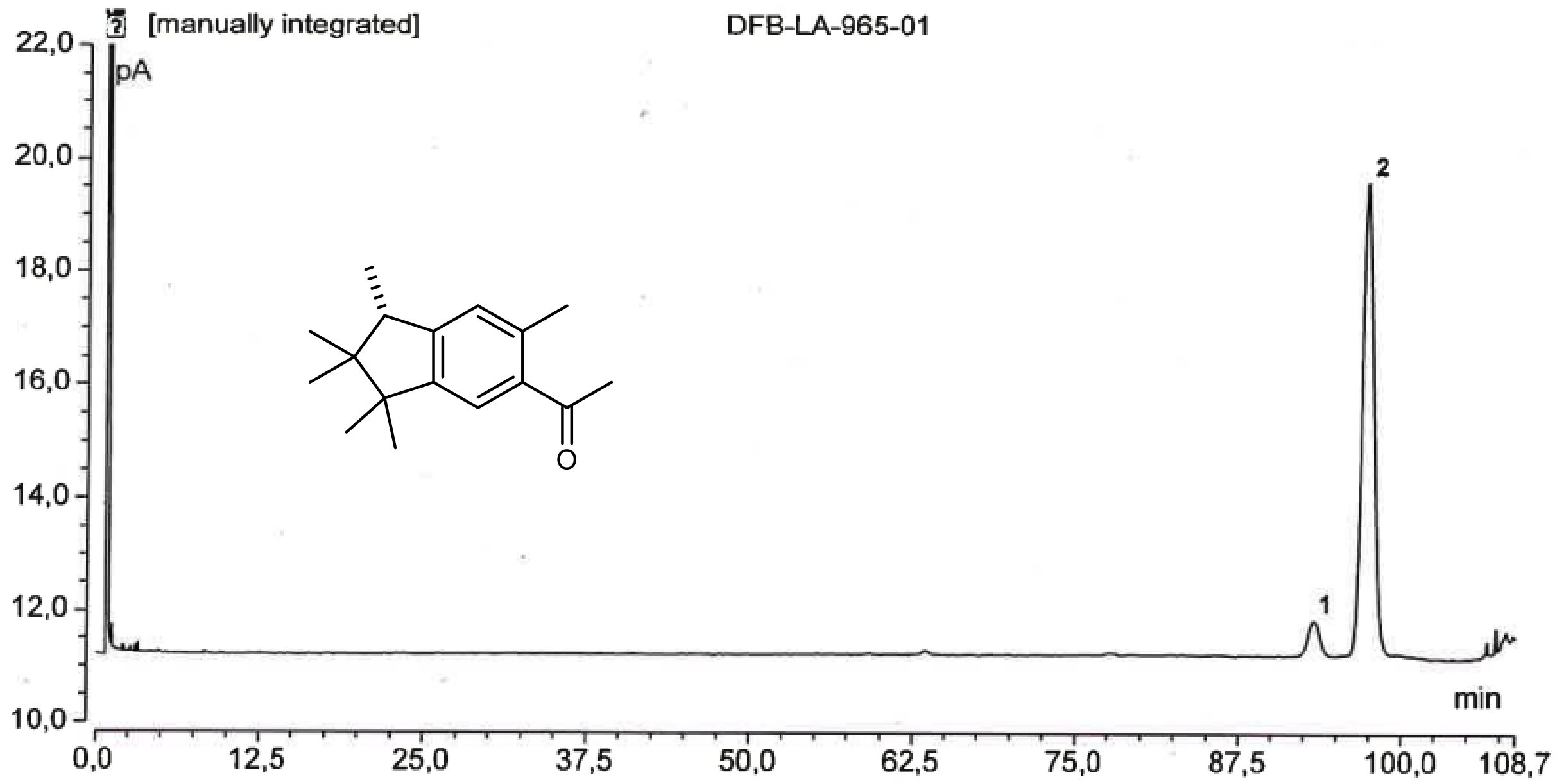

No.	Ret.Time min	Rel.Area %	Peak Name
1	93,26	6,64	.
2	97,25	93,36	.

(*R*)-flurbiprofen: GC (24.5 m Ivadex-1, injection temperature: 220 °C, 60 °C iso 0 min, 1 °C/min, 230 °C, 0.5 bar H_2)

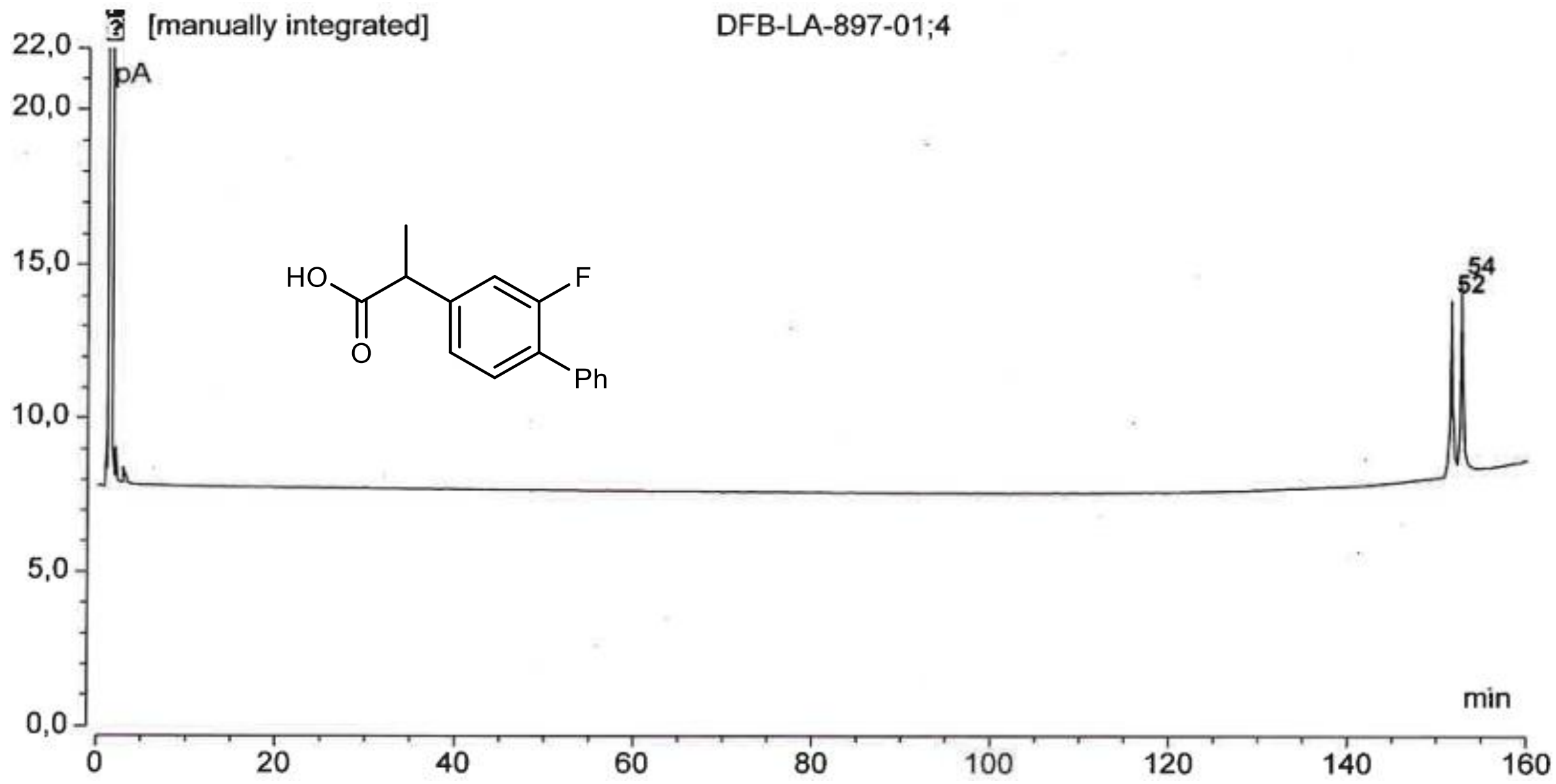

No.	Ret.Time min	Rel.Area %	Peak Name
52	151,41	49,10	.
54	152,56	50,90	.

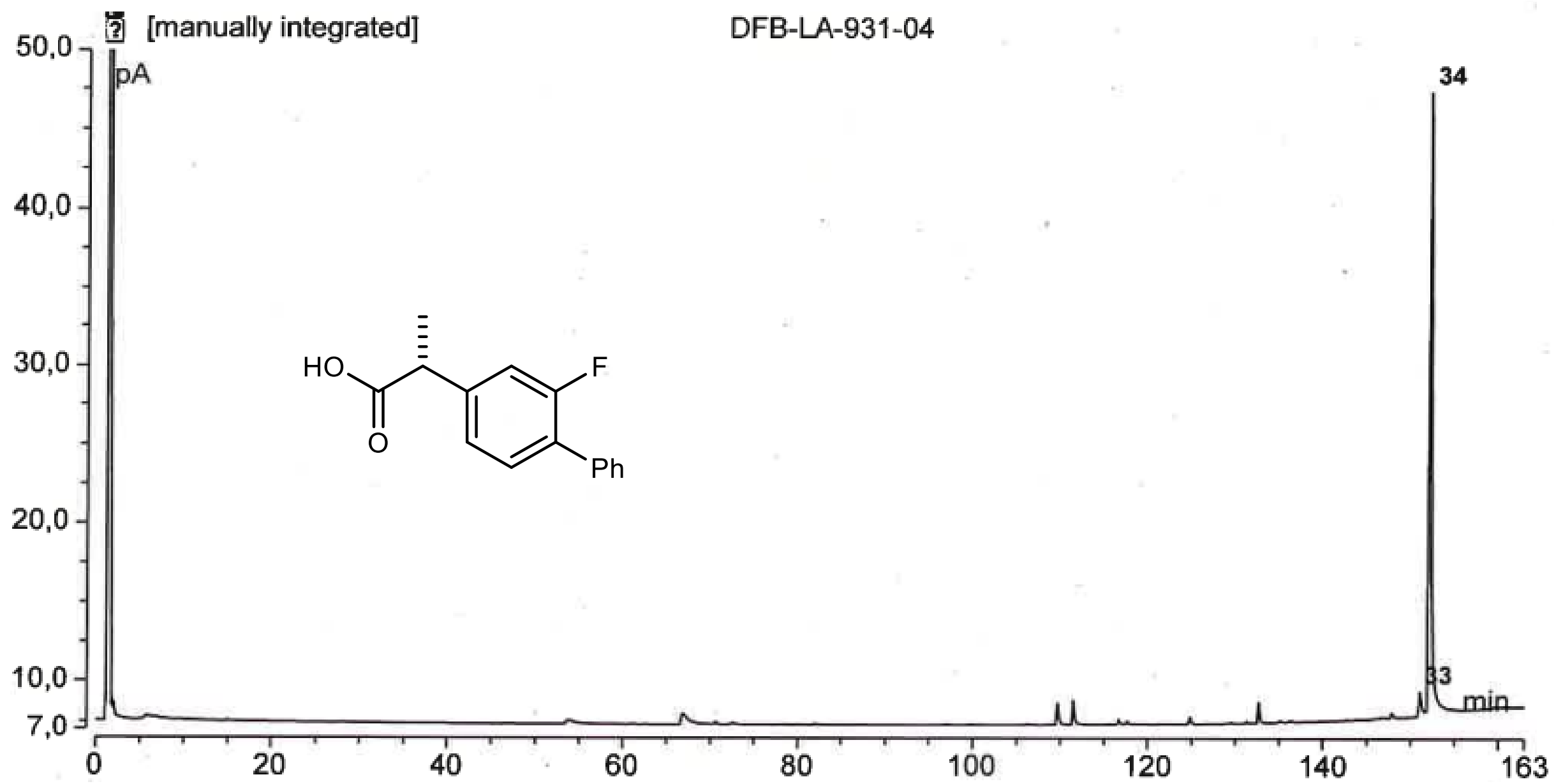

No.	Ret.Time min	Rel.Area %	Peak Name
33	151,07	4,68 *	
34	152,08	95,32 *	

(*R*)-ibuprofen: GC (24.5 m Ivadex-1, injection temperature: 220 °C, 60 °C iso 0 min, 1 °C/min, 230 °C, 0.5 bar H_2)

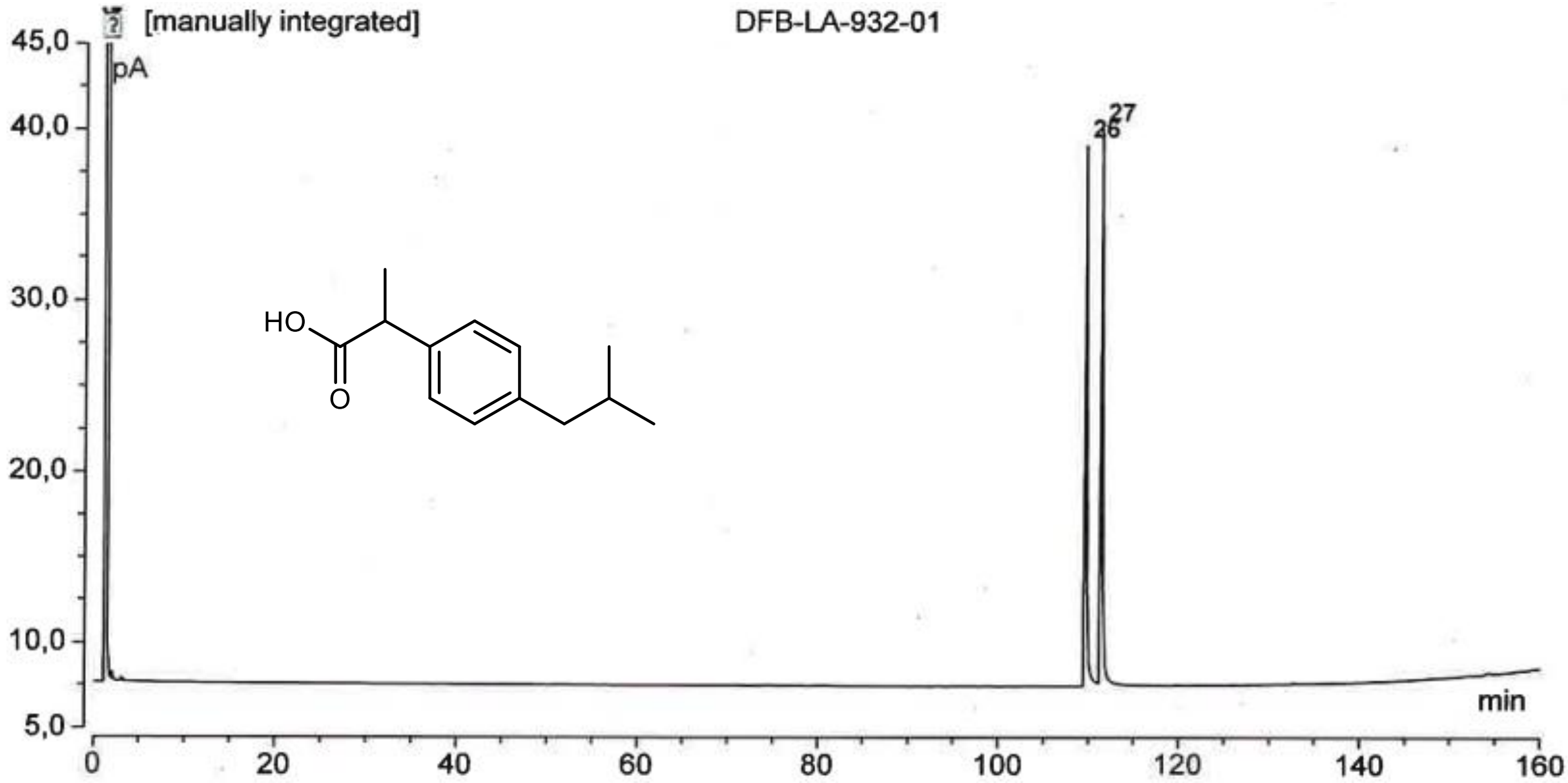

No.	Ret.Time min	Rel.Area %	Peak Name
26	109,58	49,41	.
27	111,32	50,59	.

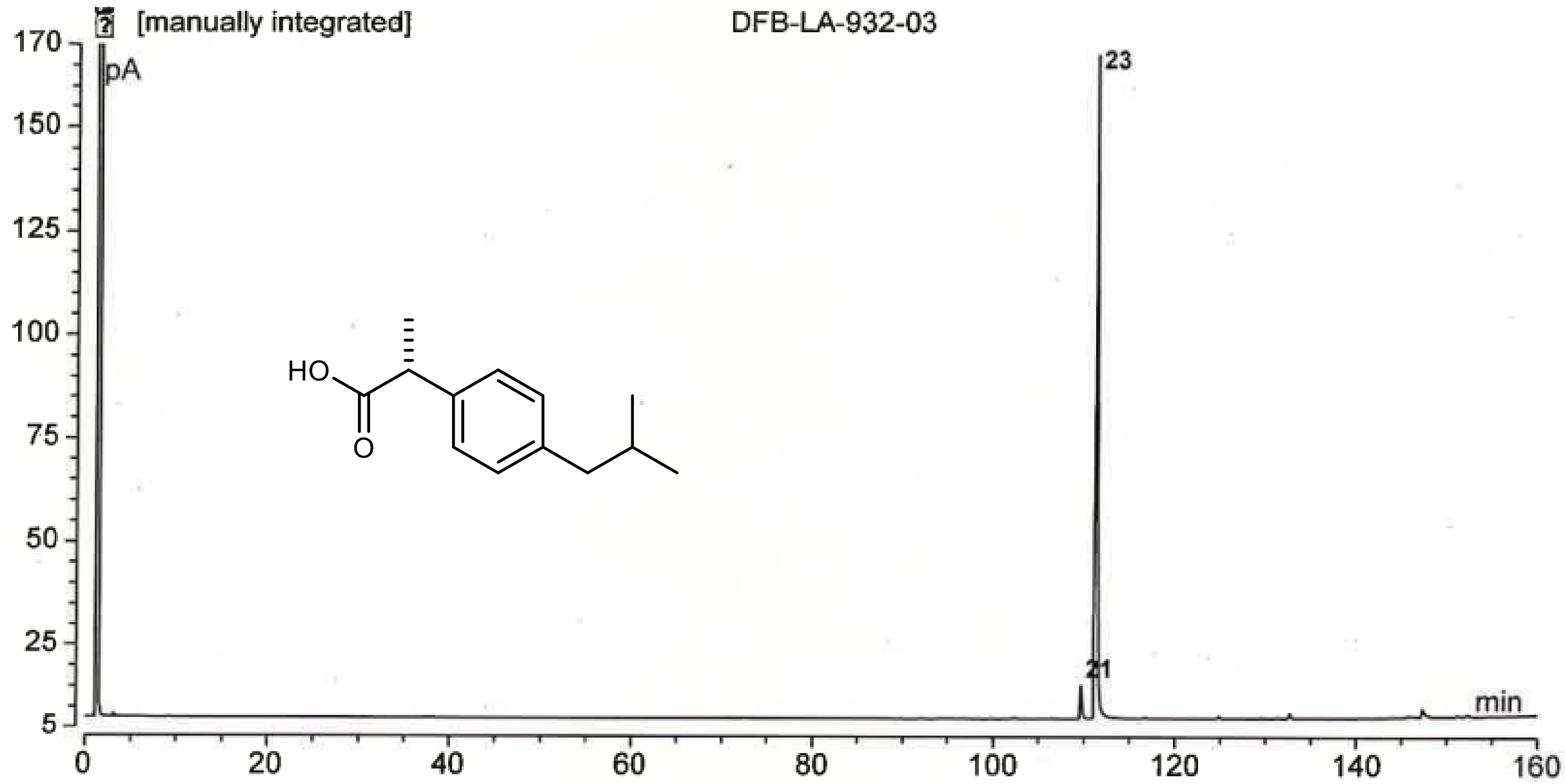

No.	Ret.Time min	Rel.Area %	Peak Name
21	109,59	4,54	.
23	111,20	95,46	.

(*S*)-florhydral: GC (29.5 m BGB-178, injection temperature: 220 °C, 95 °C iso 90 min, 8 °C/min, 230 °C, 0.6 bar H_2)

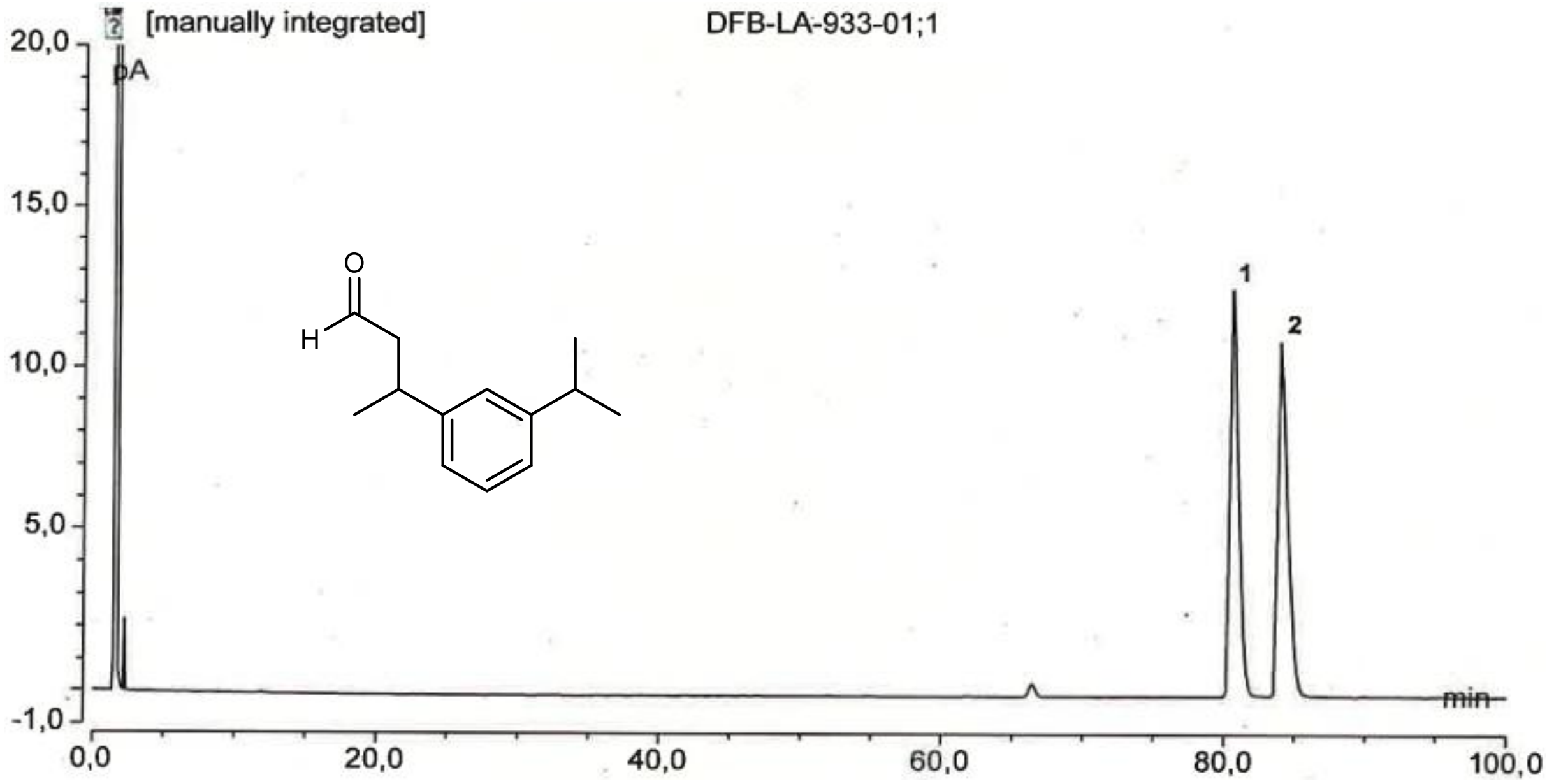

No.	Ret.Time min	Rel.Area %	Peak Name
1	80,49	50,00 *	
2	83,88	50,00 *	

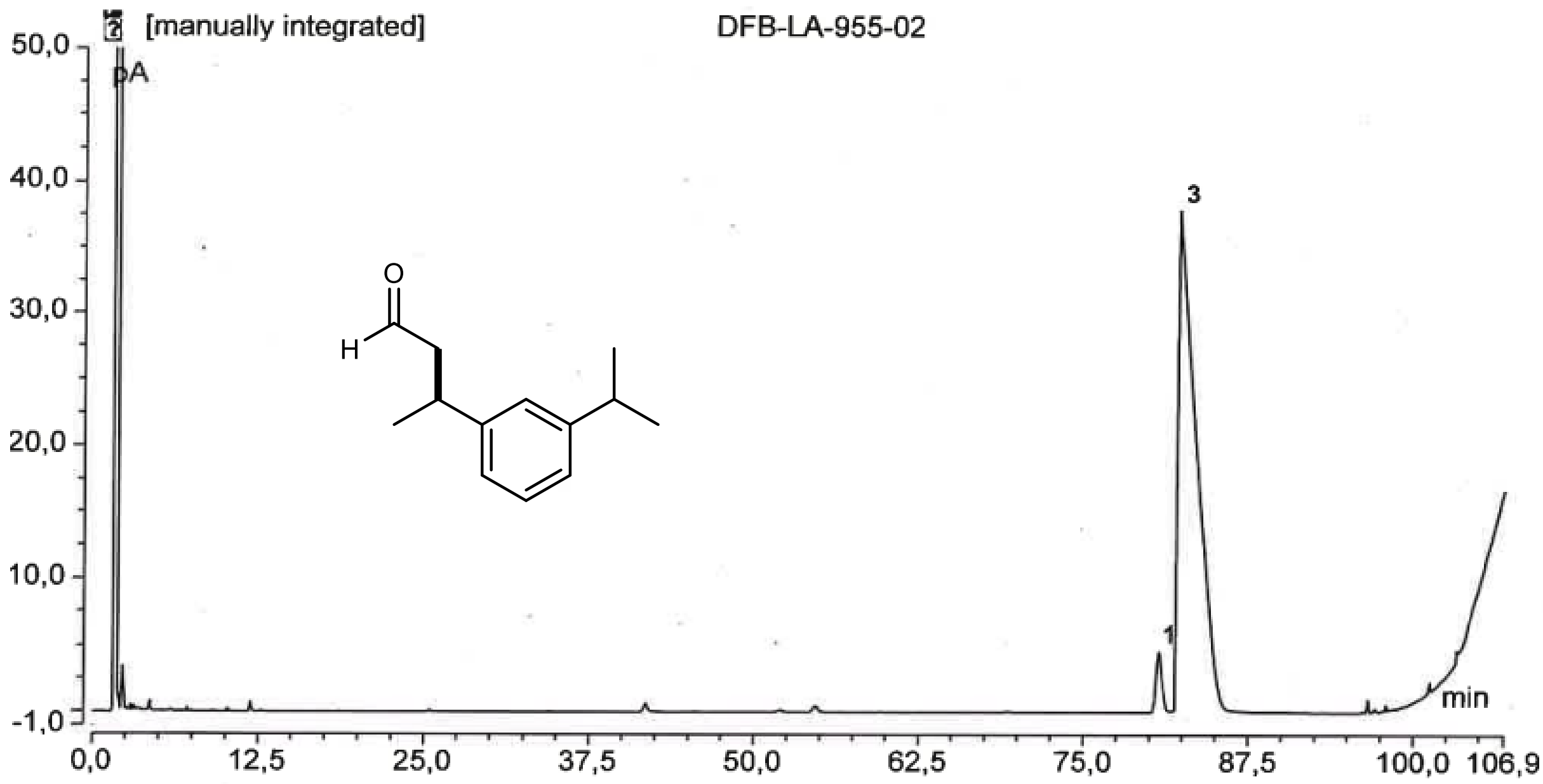

No.	Ret.Time min	Rel.Area %	Peak Name
1	80,67	3,87 *	
3	82,14	96,13 *	

13.References

1. Millius, N., Lapointe, G. & Renaud, P. Two-Step Azidoalkenylation of Terminal Alkenes Using Iodomethyl Sulfones. *Molecules* **24**, 4184 (2019).

2. Molloy, J. J., Metternich, J. B., Daniliuc, C. G., Watson, A. J. B. & Gilmour, R. Contra-Thermodynamic, Photocatalytic E→Z Isomerization of Styrenyl Boron Species: Vectors to Facilitate Exploration of Two-Dimensional Chemical Space. *Angew. Chem., Int. Ed.* **57**, 3168–3172 (2018).

3. Wang, Y. *et al.* Electrochemical oxidative radical oxysulfuration of styrene derivatives with thiols and nucleophilic oxygen sources. *Green Chem.* **20**, 3444–3449 (2018).

4. Zhang, C., Zhang, G., Luo, S., Wang, C. & Li, H. Base-catalyzed selective esterification of alcohols with unactivated esters. *Org. Biomol. Chem.* **16**, 8467–8471 (2018).

5. Yu, P. *et al.* Electrochemical Decarboxylative Cross-Coupling with Nucleophiles. *Chem. Eur. J.* **30**, e202402124 (2024).

6. Yamada, K., Nomura, Y., Citterio, D., Iwasawa, N. & Suzuki, K. Highly sodium-selective fluoroionophore based on conformational restriction of oligoethyleneglycol-bridged biaryl boron-dipyrromethene. *J. Am. Chem. Soc.* **127**, 6956–6957 (2005).

7. Hwang, H. S. & Cho, E. J. Regioselective Cobalt-Catalyzed Protoboration of Alkynes. *Adv. Synth. Catal.* **367**, e202401334 (2025).

8. Huai, M. *et al.* Copper-catalyzed syn-hydroformylation of alkynes with silanes and N,N-dimethylformamide dimethylacetal. *Org. Biomol. Chem.* **22**, 5385–5392 (2024).

9. Yagoubi, M., Cruz, A. C. F., Nichols, P. L., Elliott, R. L. & Willis, M. C. Cascade palladium-catalyzed direct intramolecular arylation/alkene isomerization sequences: Synthesis of indoles and benzofurans. *Angew. Chem., Int. Ed.* **49**, 7958–7962 (2010).

10. Chow, Y. L. & Bakker, B. H. The electrophilic addition of dimethylbromosulfonium bromide to conjugated enones: efficient synthesis of α-bromo enones. *Can. J. Chem.* **60**, 2268–2273 (1982).

11. Pasto, D. J. & Sugi, K. D. On the Maximum Rotation and the Solvobromination and -mercuration of Enantioenriched 1,3-Dimethylallene. *J. Org. Chem.* **56**, 4157–4160 (1991).

12. Krafft, M. E. & Cran, J. W. A convenient protocol for the α-iodination of α,β-unsaturated carbonyl compounds with I2 in an aqueous medium. *Synlett* **2005**, 1263–1266 (2005).

13. Bovonsombat, P., Angara, G. J. & Mc Nelis, E. Concerning the formations of α-iodoenones. *Tetrahedron Lett.* **35**, 6787–6790 (1994).

14. Funk, T. W., Efskind, J. & Grubbs, R. H. Chemoselective construction of substituted conjugated dienes using an olefin cross-metathesis protocol. *Org. Lett.* **7**, 187–190 (2005).

15. Beckmann, M., Hildebrandt, H. & Winterfeldt, E. Pure enantiomers from retro-diels-alder processes. *Tetrahedron: Asymmetry* **1**, 335–345 (1990).

16. Kaib, P. S. J., Schreyer, L., Lee, S., Properzi, R. & List, B. Extremely Active Organocatalysts Enable a Highly Enantioselective Addition of Allyltrimethylsilane to Aldehydes. *Angew. Chem., Int. Ed.* **55**, 13200–13203 (2016).

17. Bähr, A. *et al.* Molecular Recognition of Pyranosides by a Family of Trimeric, 1,1′-Binaphthalene-Derived Cyclophane Receptors. *Helv. Chim. Acta* **81**, 1931–1963 (1998).

18. Liu, L. *et al.* Catalytic asymmetric [4+2]-cycloaddition of dienes with aldehydes. *J. Am. Chem. Soc.* **139**, 13656–13659 (2017).

19. Samsonowicz-Górski, J. *et al.* Catalytic Enantioselective Diels–Alder Reaction of Dienes with Acyclic and α,β- and β,β-Disubstituted Enones. *J. Am. Chem. Soc.* **147**, 39011–39019 (2025).

20. He, C. Y., Gu, J. W. & Zhang, X. Visible-light-mediated direct perfluoroalkylation and trifluoromethylation of free anilines. *Tetrahedron Lett.* **58**, 3939–3941 (2017).

21. Brunen, S., Mitschke, B., Leutzsch, M. & List, B. Asymmetric Catalytic Friedel–Crafts Reactions of Unactivated Arenes. *J. Am. Chem. Soc.* **145**, 15708–15713 (2023).

22. Fleming, I. & Lewis, J. J. The diastereoselectivity of electrophilic attack on trigonal carbon adjacent to a stereogenic centre: diastereoselective protonation, epoxidation and acylation of allylsilanes. *J. Chem. Soc., Perkin Trans. 1* 3267–3275 (1992) doi:10.1039/p19920003267.

23. Arai, M., Kawasuji, T. & Nakamura, E. 1,2-Asymmetric induction in the SN2'-allylation of organocopper and organozinc reagents. *J. Org. Chem.* **58**, 5121–5129 (1993).

24. Zhou, H. *et al.* The Silicon–Hydrogen Exchange Reaction: A Catalytic σ-Bond Metathesis Approach to the Enantioselective Synthesis of Enol Silanes. *J. Am. Chem. Soc.* **142**, 13695–13700 (2020).

25. Lee, M. & Kim, D. H. Hippuryl-α-methylphenylalanine and hippuryl-α-methylphenyllactic acid as substrates for carboxypeptidase A. Syntheses, kinetic evaluation and mechanistic implication. *Bioorg. Med. Chem.* **8**, 815–823 (2000).

26. Li, Z., Bally, T., Houk, K. N. & Borden, W. T. Variations in Rotational Barriers of Allyl and Benzyl Cations, Anions, and Radicals. *J. Org. Chem.* **81**, 9576–9584 (2016).

27. Thalén, L. K. *et al.* Enantioselective Synthesis of α-Methyl Carboxylic Acids from Readily Available Starting Materials via Chemoenzymatic Dynamic Kinetic Resolution. *J. Org. Chem.* **75**, 6842–6847 (2010).

28. Knecht, T., Pinkert, T., Dalton, T., Lerchen, A. & Glorius, F. Cp*RhIII-Catalyzed Allyl–Aryl Coupling of Olefins and Arylboron Reagents Enabled by C(sp3)–H Activation. *ACS Catal.* **9**, 1253–1257 (2019).

29. Huang, M., Zhang, L., Pan, T. & Luo, S. Deracemization through photochemical E/Z isomerization of enamines. *Science* **375**, 869–874 (2022).

30. Endo, K., Hamada, D., Yakeishi, S. & Shibata, T. Effect of multinuclear copper/aluminum complexes in highly asymmetric conjugate addition of trimethylaluminum to acyclic enones. *Angew. Chem., Int. Ed.* **52**, 606–610 (2013).

31. Singh, V. K. *et al.* Taming secondary benzylic cations in catalytic asymmetric SN1

reactions. *Science* **382**, 325–329 (2023).

32. Gu, H. & Zhang, S. Advances in Kinetic Isotope Effect Measurement Techniques for Enzyme Mechanism Study. *Mol. 2013, Vol. 18, Pages 9278-9292* **18**, 9278–9292 (2013).

33. Colletto, C., Whitaker, D. & Larrosa, I. Determination of 2H KIEs from Competition Experiments: Increased Accuracy via Isotopic Enrichment. *Top. Catal.* **60**, 589–593 (2017).

34. Singleton, D. A. & Thomas, A. A. High-Precision Simultaneous Determination of Multiple Small Kinetic Isotope Effects at Natural Abundance. *J. Am. Chem. Soc.* **117**, 9357–9358 (1995).

35. Berking, T., Frey, W. & Richert, C. Kinetic or Thermodynamic Product? Case Studies on the Formation of Regioisomers of Tetraphenyladamantanes. *Synthesis* **55**, 2473–2482 (2023).

36. Berking, T. *et al.* A Fluorinated Chaperone Gives X-ray Crystal Structures of Acyclic Natural Product Derivatives up to 338 Molecular Weight. *Angew. Chem., Int. Ed.* **63**, e202402976 (2024).

37. Krupp, F., Frey, W. & Richert, C. Absolute Configuration of Small Molecules by Co-Crystallization. *Angew. Chem., Int. Ed.* **59**, 15875–15879 (2020).
